# Transcription and Signaling Regulators in Developing Neuronal Subtypes of Mouse and Human Enteric Nervous System

**DOI:** 10.1053/j.gastro.2017.10.005

**Published:** 2018-02

**Authors:** Fatima Memic, Viktoria Knoflach, Khomgrit Morarach, Rebecca Sadler, Catia Laranjeira, Jens Hjerling-Leffler, Erik Sundström, Vassilis Pachnis, Ulrika Marklund

**Affiliations:** 1 Division of Molecular Neurobiology, Department for Medical Biochemistry and Biophysics, Karolinska Institute, Stockholm, Sweden; 2 Division of Molecular Neurobiology, National Institute for Medical Research, Medical Research Council, London, United Kingdom; 3 Division of Neurodegeneration, Department of Neurobiology, Care Sciences and Society, Karolinska Institutet, Stockholm, Sweden; 4 Stockholms Sjukhem, Stockholm, Sweden; 5 Francis Crick Institute, London, United Kingdom

**Keywords:** HOX, Tyrosine Hydroxylase, Gastric Motility, Neural Crest, AHR, aryl hydrocarbon receptor, BMP, bone morphogenetic protein, CTGF, connective tissue growth factor, C11, transcriptome of non-ENS gut cells at E11.5, C15, transcriptome of non-ENS gut cells at E15.5, E, embryonic day, ENS, enteric nervous system, ENSC, enteric neural stem cell, FACS, fluorescent-activated cell sorting, FGF, fibroblast growth factor, GI, gastrointestinal, IHC, immunohistochemistry, ISH, *in situ* hybridization, MDK, midkine, NTN, netrin, PBS, phosphate-buffered saline, RT, room temperature, SEMA, semaphorin, S11, transcriptome of enteric progenitor cells at E11.5, S15, transcriptome of enteric progenitor cells at E15.5, TALE, 3 amino acid loop extension, TGF, transforming growth factor, UCL, University College of London, W, embryonic week, W11, transcriptome of ENS cells at E11.5, W15, transcriptome of ENS cells at E15.5, YFP, yellow fluorescence protein

## Abstract

**Background & Aims:**

The enteric nervous system (ENS) regulates gastrointestinal function via different subtypes of neurons, organized into fine-tuned neural circuits. It is not clear how cell diversity is created within the embryonic ENS; information required for development of cell-based therapies and models of enteric neuropathies. We aimed to identify proteins that regulate ENS differentiation and network formation.

**Methods:**

We generated and compared RNA expression profiles of the entire ENS, ENS progenitor cells, and non-ENS gut cells of mice, collected at embryonic days 11.5 and 15.5, when different subtypes of neurons are formed. Gastrointestinal tissues from *R26ReYFP* reporter mice crossed to *Sox10-CreER*^*T2*^ or *Wnt1-Cre* mice were dissected and the 6 populations of cells were isolated by flow cytometry. We used histochemistry to map differentially expressed proteins in mouse and human gut tissues at different stages of development, in different regions. We examined enteric neuronal diversity and gastric function in *Wnt1-Cre x Sox6*^*fl/fl*^ mice, which do not express the *Sox6* gene in the ENS.

**Results:**

We identified 147 transcription and signaling factors that varied in spatial and temporal expression during development of the mouse ENS. Of the factors also analyzed in human ENS, most were conserved. We uncovered 16 signaling pathways (such as fibroblast growth factor and Eph/ephrin pathways). Transcription factors were grouped according to their specific expression in enteric progenitor cells (such as MEF2C), enteric neurons (such as SOX4), or neuron subpopulations (such as SATB1 and SOX6). Lack of SOX6 in the ENS reduced the numbers of gastric dopamine neurons and delayed gastric emptying.

**Conclusions:**

Using transcriptome and histochemical analyses of the developing mouse and human ENS, we mapped expression patterns of transcription and signaling factors. Further studies of these candidate determinants might elucidate the mechanisms by which enteric stem cells differentiate into neuronal subtypes and form distinct connectivity patterns during ENS development. We found expression of SOX6 to be required for development of gastric dopamine neurons.

## Graphical abstract

**Figure:**
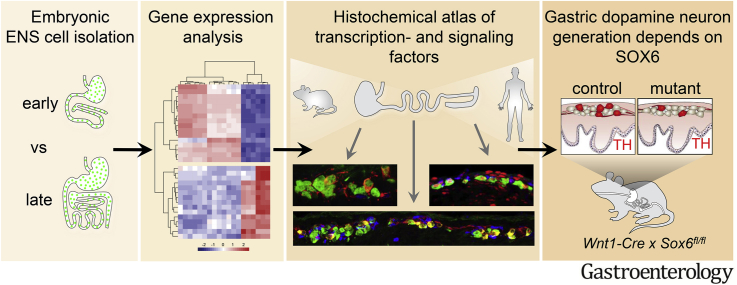

See Covering the Cover synopsis on page 458; see editorial on page 478.

**Editor's Notes**

**Background and Context**

The diverse types of neurons of the enteric nervous system (ENS) are required for normal gastrointestinal functions, yet how cellular diversity is created during ENS development remains unknown.

**New Findings**

RNA profiles and histochemical patterns identified regulatory genes in developing enteric neuron subtypes in mouse and human embryos. The transcription factor SOX6 was found essential for gastric dopamine neuron formation.

**Limitations**

Although many of the identified gene expressions were described in different regions and stages of the gastrointestinal tract by immunohistochemical stainings, the large majority need validating analyses.

**Impact**

This resource of gene expression patterns can be implemented in molecular research on ENS development and in studies aimed at differentiating specific types of enteric neurons from embryonic stem cells.

Vital gastrointestinal (GI) functions, including bowel motility, blood flow, and fluid exchange, are regulated by the enteric nervous system (ENS). To accomplish these complex tasks, the ENS is organized into full neural circuits composed of a multitude of different cell types, including intrinsic sensory neurons, motor neurons, interneurons, and enteric glia.^1^ In the congenital ENS disorder Hirschsprung disease, children are typically born with the distal bowel lacking motility due to local absence of enteric neurons.^2^ In other enteric neuropathies, including achalasia and gastroparesis, only subsets of neuronal subtypes are affected.^1^, ^2^ Hence, the diversity of enteric neuronal subtypes is critical for normal gut function and selective dysfunction or local neuronal loss leads to GI disorders. Recent progress in producing stem cell–derived ENS cells raises hope for development of novel cell-based treatments of ENS disorders,^2^, ^3^ but functional recovery rests on recreating enteric circuits with diverse cellular composites. To date, knowledge of the molecular mechanisms underlying the generation of different ENS cell types in correct numbers and proportions is incomplete,^4^ but would be instrumental in the engineering of functional ENS networks.

The ENS is primarily derived from neural crest cells, which emigrate from the vagal (and partly sacral) neural tube and colonize the foregut at embryonic day (E) 9.0 in mice and week (W) 4 in humans. Extensive proliferation and migration of these enteric neural stem cells (ENSCs) eventually results in an interconnected ganglionic network along the GI tract.^5^ Asynchronous differentiation into neurons starts at E10 and continues into postnatal stages,^6^ resulting in 16 mature neuronal subtypes as defined by axonal projection patterns and neurotransmitter expression.^4^ However, detailed investigation of enteric neuronal subtypes and their lineage development is challenged by the lack of a clear functional architecture in the ENS. In contrast, the patterned germinal zones generating groups of functionally related neurons in the central nervous system has allowed extensive research on its development. There, establishment of distinct neurons relies on the interplay between instructive signaling factors and intrinsic programs of transcription factor networks, sequentially regulating specification and differentiation.^7^ On account of this, the stage of maturity and identity of a neural cell can be inferred from its transcription factor signature. Many transcription factors have been detected during ENS development, but only a few have been described in detail and shown to influence neuronal subtype commitment.^8^, ^9^ The bowel mesenchyme secretes several growth factors essential for ENS development, but our understanding is mainly restricted to the roles of these factors in general differentiation and proliferation.^8^ Intricate cell-to-cell communication also is required for the subsequent establishment of neuron-specific connections to form functional circuits, but remains uncharacterized. Expression profiles of transcriptional and signaling regulators in ENS sublineages at different stages could thus be deployed to uncover the molecular mechanisms underlying neuronal diversification and network formation during ENS development.

Birth-dating studies demonstrate that the different enteric neuronal subclasses are born (undergo neurogenesis) in a temporally sequential manner.^10^, ^11^ Enteric serotonergic neurons (5-HT^+^) are born early (E10–E12.5), whereas for instance, dopaminergic neurons (TH^+^) appear slightly later (peaks at E13–E15.5). The timed order of neuronal subtype generation indicates a progressive change in the differentiation competence of ENSCs. To reveal intrinsic and extrinsic regulatory genes at discrete steps during differentiation of enteric neuronal subclasses, we performed RNA expression analysis of ENS subpopulations and surrounding gut tissue at developmentally distinct stages. Adding to the transcriptome analysis, we present a spatiotemporal histochemical expression pattern map of transcriptional and signaling factors in both mouse and human developing gut. Demonstrating the strength of our strategy to find determining genes, we show that one candidate transcription factor, Sox6, is essential for the selective development of gastric dopamine neurons. Implementation of this novel gene platform would significantly refine basic and translational studies of the successive processes in which ENSCs differentiate into various enteric neurons and assemble into discrete ENS circuitries to control bowel physiology.

## Material and Methods

### Mouse and Human Embryos

The generation of *Sox10CreER*^*T2*^,^6^ *Wnt1Cre*,^12^ and *R26ReYFP*^13^ mouse strains has been described. *Sox6*^*fl*^ mice were kindly provided by V. Lefebvre (Cleveland Clinic Lerner Research Institute, Cleveland, OH).^14^ Histochemical analysis was performed on C57 and MF1 mice. Animal experiments adhered to Animal Research: Reporting In Vivo Experiments standards, and were approved by the local National Institute for Medical Research ethical review panel or by the local ethics committee in Stockholm (N87/13). Remains of human embryos and fetuses (5.5–10.0 weeks after conception) were obtained after elective routine abortions with written consent given by the pregnant women. Collection of human tissue for research was approved by the Regional Human Ethics Committee, Stockholm (2013/564–32) and conducted by the Karolinska Institutet Stem Cell and Tissue Bank.

### Induction of Inducible Reporter

Time-mated *Sox10CreER*^*T2*^ × *R26ReYFP* mice received a single intraperitoneal injection of 4-hydroxytamoxifen (4-OHT; Sigma-Aldrich, Saint Louis, MO) dissolved in ethanol/corn oil (1:9) (0.1 mg/g body weight) at E10.5 or E14.5. After 20 hours, embryos were harvested.

### Preparation of Cell Populations

Dissected guts were digested in 1 mg/mL dispase/collagenase (Roche, Basel, Switzerland) for 3 to 5 minutes (E11.5) at room temperature (RT) or 1 hour (E15.5) at 37°C. Tissue was then dissociated in OPTIMEM (Life Technologies, Carlsbad, CA) supplemented with 10% fetal calf serum, collected by centrifugation and passed through a cell strainer cap (Falcon; 352235). Fluorescent-activated cell sorting (FACS) of cells was performed using a MoFlo Cell Sorter (BeckmanCoulter, Brea, CA) or a FACSAria II (BD Biosciences, San Jose, CA) (Supplementary Figure 1). Purity-checked cell samples were counted using a cytometer, spun at 200*g* for 12 minutes at 4°C and resuspended in lysis buffer from RNeasy Micro kit (Qiagen, Hilden, Germany). Samples were vortexed or run through a shredder (Qiagen), snap-frozen, and kept at −80°C.

### RNA Preparation

Samples from different FACS sortings were pooled to obtain appropriate final RNA concentrations. Total RNA was extracted from the samples using an RNeasy Micro kit (Qiagen). Purity of RNA was verified by Bioanalyzer Total RNA Nano (Agilent Technologies, Santa Clara, CA). Biotin-labeled complementary DNA was prepared using the Ovation Pico kit (Nugen, Manchester, UK) at the University College of London (UCL) Genomics (London, UK). For detailed information of cell samples and RNA analysis see Supplementary Figure 1.

### Microarray Analysis

The samples were hybridized to GeneChip Mouse Gene 1.0 ST arrays at UCL Genomics and processed using the rma algorithm using the Partek software (Partek Inc., St. Louis, MO). Principal component analysis and box-plots showed normal distribution.

### Bioinformatic Analysis

Partek software was used at UCL Genomics to perform pairwise comparisons. Significant genes were based on 5% false discovery rate using the Benjamini & Hochberg correction. Functional annotation clustering was performed in the DAVID resource 6.7.^15^ Although filtered away by the applied false discovery rate, expression of a few genes was found by *in situ* hybridization (ISH), immunohistochemistry (IHC), or other published study. These genes were introduced into resulting gene lists.

### Tissue Preparation

E11.5 to 12.5 mouse embryos were fixed in 4% paraformaldehyde in phosphate-buffered saline (PBS) at 4°C for 1.5 hours. Isolated E15.5 to 19 mouse guts and human guts were fixed for 2 hours. Tissue was incubated at 4°C overnight in 30% sucrose in PBS, embedded in optimum cutting temperature compound (Histolab, Leiden, The Netherlands) and stored at −80°C. Tissue was sectioned at 14 μm.

### Immunohistochemistry

IHC was performed as described.^9^ Briefly, mouse sections were preincubated for 2 hours at RT with unconjugated donkey anti-mouse IgG (Jackson ImmunoResearch, West Grove, PA). Mouse and human tissue were then blocked 1 hour (2% donkey or goat serum, 0.1% Triton x-100, PBS) and incubated overnight with primary antibodies and detected with secondary antibodies the following day (Supplementary Table 1). Before IHC, some antibodies required antigen retrieval: microwave heating of slides in antigen retrieval solution (Dako, Santa Clara, CA) followed by cooling to RT.

### Imaging

Images were taken using a Zeiss (Oberkochen, Germany) LSM700 confocal microscope and processed in Adobe Photoshop CS6 (Adobe Systems Inc., San Jose, CA) or Image J (National Institutes of Health, Bethesda, MD). Counting of fluorescent cells was performed on images or directly under a Zeiss fluorescent microscope. The abundance of protein coexpression was estimated by visual inspection. For all ISH analysis, we used Allen Developing Mouse Brain Atlas (http://developingmouse.brain-map.org) and GenePaint (http://www.GenePaint.org).

### Gastric Emptying

Liquid gastric emptying assay was essentially performed as described.^16^ Mice were fasted 6 hours and water withdrawn 1 hour before the experiment. Animals were killed 15 minutes after gavage (rhodamine B dextran: 100 μL; 10 mg/mL in 2% methylcellulose; Invitrogen, Carlsbad, CA), whereby stomach and small intestine (10 equal-length segments) were collected in 0.9% NaCl, homogenized, and centrifuged. Fluorescence was measured in 200-μL aliquots (triplets) of the supernatant (Fluostar Omega; BMG Labtech, Offenburg, Germany) and percentage of total fluorescence that emptied from the stomach was calculated.

Stomach size assessment was made in mice culled at 9 AM with prior free access to food and drink.

### Statistical Analysis

Mutant embryos were compared with littermate controls. Student paired *t* test was performed for cell countings, and Student *t* test for the functional assay. Bars indicate means ± standard deviation. Significance levels for the tests were assumed at *P < .05; **P < .01; ***P < .001.

### Data Availability

The microarray data have been submitted to the GEO database (http://www.ncbi.nlm.nih.gov/geo/) and assigned the identifier GSE100130.

## Results

### Transcriptome Analysis Identifies Differentially Expressed Genes in Enteric Progenitors, Neurons, and Non-ENS Gut Cells at Different Developmental Stages

Aiming to identify intrinsic and extrinsic genes with regulatory functions at successive steps during differentiation of phenotypically distinct neurons, we designed a transcriptome analysis comparing ENS progenitor cells, the entire ENS (including also immature neurons), and surrounding gut tissue at 2 developmentally distinct stages (E11.5 and E15.5).

**Figure 1:**
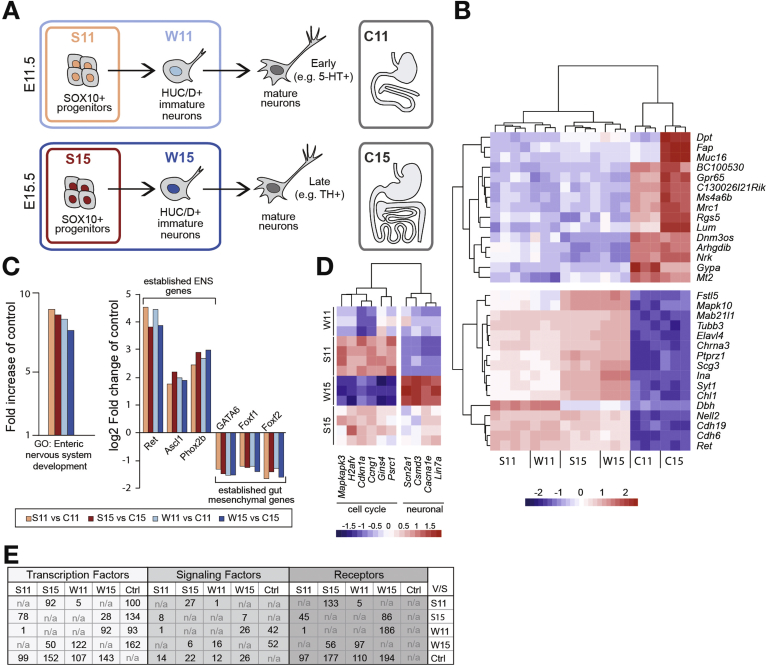
Transcriptome screen design, verification, and identification of enriched genes. (*A*) Schematic drawing depicting the transcriptome analysis. ENS populations included SOX10^+^ progenitors (red boxes) and the whole ENS (blue boxes), each at 2 distinct development stages: E11.5 (S11 and W11) and E15.5 (S15 and W15). W11 contained immature neurons differentiating into, for example, 5-HT^+^ neurons, whereas W15 included immature neurons differentiating into other subtypes (eg, TH^+^). Non-ENS control gut tissue (gray boxes) at E11.5 (C11) and E15.5 (C15) was also included. (*B*) Heat map summarizing differentially expressed genes, compiled from the union of genes with top-10 absolute fold change (and *P* < .05) in the comparisons of S11vsC11, S15vsC15, W11vsC11, and W15vsC15. Genes and conditions are clustered by their hierarchical similarity. Color intensity represents the mean-centred log_2_ expression values. (*C*) Graphs comparing gene ontology (GO) term enrichment, ENS, and gut marker genes in the 4 ENS populations to control populations. (*D*) Heat map depicting cell cycle or neuronal genes. Genes are clustered by their hierarchical similarity. Color intensity represents the mean-centred log_2_ expression values. (*E*) Tables showing the number of transcription factors, signaling factors, and receptors found in pairwise comparisons (absolute fold change >1.2; *P* < .05) of the transcriptomes. n/a, not analyzed.

As SOX10 specifically marks dividing ENS progenitor cells,^8^ we could retrieve this subpopulation by inducing reporter expression in *Sox10CreER*^*T2*^ × *R26ReYFP* embryos.^6^ The whole ENS could be collected from *Wnt1Cre* × *R26ReYFP* mouse embryos, owing to ENS-specific yellow fluorescence protein (YFP) reporter expression in the GI tract.^12^ YFP^+^ and YFP^−^ cells from dissociated E11.5 and E15.5 guts of transgenic embryos were separated using FACS, after which RNA expression was determined using gene arrays. In total, the transcriptomes of 4 different ENS populations were analyzed: *Sox10CreER*^*T2*^ × *R26ReYFP* at E11.5 and E15.5 (hereinafter denoted S11 and S15) and *Wnt1Cre* × *R26ReYFP* at E11.5 and E15.5 (hereinafter denoted W11 and W15) (Figure 1*A*). Transcriptomes of YFP^−^ cells, derived from *Wnt1Cre* × *R26ReYFP* gut at E11.5 and E15.5 representing non-ENS gut tissue also were included in the analysis (partly as a control and hereinafter denoted C11 and C15) (Figure 1*A*). Pairwise comparisons between the 6 populations can be found in Supplementary Table 2.

By distributing the top-10 enriched genes obtained from pairwise comparisons between ENS populations (S11, S15, W11, and W15) and non-ENS controls (C11 or C15) in a combined unsupervised chart, we confirmed clustering of each data set and an overall similarity between ENS populations (Figure 1*B*). Gene ontology analysis verified that enteric nervous system development (7.6–8.3) (Figure 1*C*) was highly enriched in the 4 ENS transcriptomes in comparison with non-ENS controls. Well-known ENS-expressed genes also displayed high expression levels in ENS populations, whereas gut regulatory genes were enriched in C11 and C15 (Figure 1*C*). Functional cluster analysis of the S15 versus W15 (S15vsW15) comparison confirmed a high enrichment score for the gene ontology term *cell cycle* (37.2) in S15 and *neuron projection* (21.6) in W15. The S11vsW11 comparison yielded too few genes for such analysis. However, several genes involved in cell cycle regulation were enriched in both S11 and S15, whereas genes associated with neurons were enriched in W11 and W15 (Figure 1*D*). In summary, this analysis verified that the 4 isolated ENS transcriptomes (S11, W11, S15, W15) represented ENS cells. S11 and S15 mostly contained dividing progenitors, whereas W11 and W15 also included differentiating neurons (Figure 1*A*).

As this study focused on revealing regulatory genes in cellular diversification of the developing ENS, we decided to mine the pairwise transcriptome comparisons for 3 sets of enriched genes: transcription factors, signaling factors, and receptors (summarized in Figure 1*E*; corresponding gene lists in Supplementary Tables 3–5).

### Identification of Novel Transcription Factors in the Developing ENS

**Figure 2:**
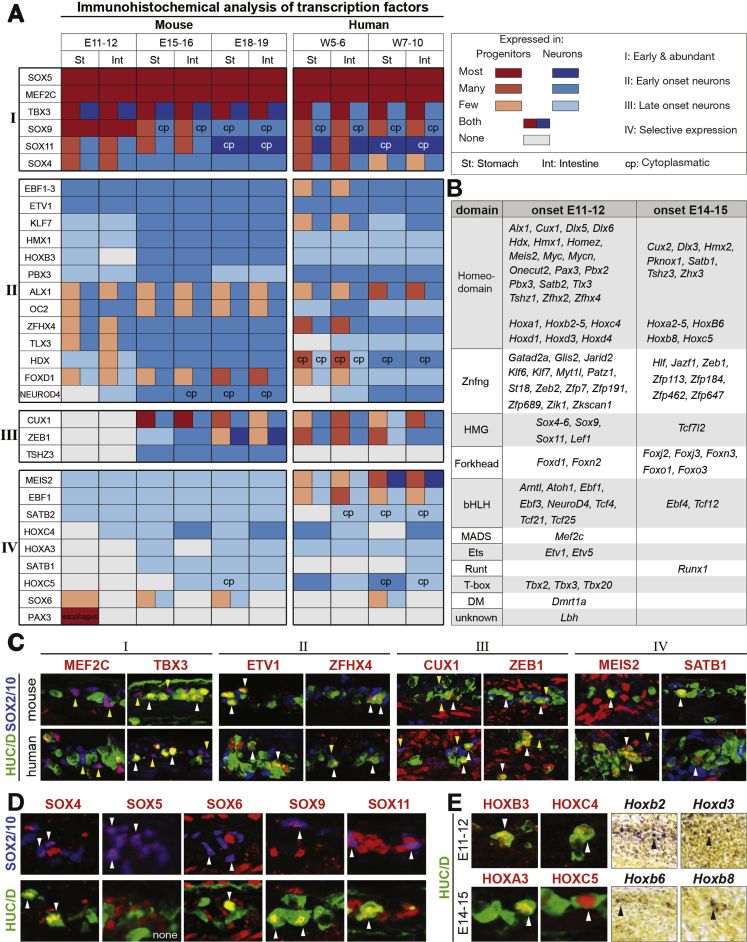
Expression dynamics of transcription factors in the developing ENS. (*A*) Table summarizing IHC expression analysis (Supplementary Figures 3 and 4) of transcription factors in relation to HUC/D^+^ neurons and SOX10^+^ progenitor cells in stomach and intestine of mouse and human embryos at different stages. Genes are grouped according to their expression dynamics. (*B*) Table showing transcription factors ordered according to their DNA binding domain and their onset of expression. Onset time was estimated based on IHC, ISH (Supplementary Figure 2), and/or the transcriptome analysis. (*C*) Examples of IHC from groups I to IV showing similar gene expression in mouse and human. Yellow arrowheads, expression in progenitors; white arrowheads, expression in neurons. (*D*) Expression of SOX proteins in the developing ENS together with SOX2/10^+^ progenitors or HUC/D^+^ neurons at E15.5 (SOX4 at E12.5). Arrowheads indicate double-positive cells. (*E*) Expression of Hox genes in the developing ENS. Note localization of *Hoxa3*, *Hoxc5*, *Hoxb3*, and *Hoxc4* in HUC/D^+^ neurons (arrowheads).

Out of the hundreds of enriched transcription factors (Figure 1*E*; Supplementary Table 3) we performed a confirmative in-depth analysis primarily including transcription factors already linked to developmental processes and omitting those associated with mitochondria or the general transcriptional machinery. The expression of these transcription factors was first examined using online ISH resources (Supplementary Table 6; Supplementary Figure 2). A total of 31 genes were then selected for an extensive IHC analysis. In this mapping, we determined the expression in relation to HUC/D^+^ neurons and SOX10^+^ progenitor cells in mouse stomach and intestine at E11-12, E15-16, and E18-19 (Figure 2*A*, Supplementary Figure 3). Taken together, we identified and confirmed the expression of 73 novel transcription factors in the developing ENS. The expression of additionally 39 previously found transcription factors (Supplementary Table 6) was also confirmed, and in some cases included in the IHC expression analysis.

To address evolutionary conservation and directly bridge our findings to translational implementations, IHC analysis was performed in human embryonic gut at W5-6 (equivalent to ∼E12–E14) and W7-10 (equivalent to ∼E14.5–E17.5) (Figure 2*A* and *C*; Supplementary Figure 4). With very few exceptions, the transcription factors found in mouse ENS were also detected in the developing human ENS and with very similar expression dynamics (Figure 2*C*, Supplementary Figure 4). To view a comprehensive list of transcription factors expressed in the developing ENS (novel and previously known), see Supplementary Table 6.

### Expression Dynamics of Transcription Factors of Key Developmental Gene Families

Based on the spatiotemporal expression patterns revealed by IHC, we subdivided the transcription factors into 4 groups characterized by (I) early and abundant; (II) early onset, mainly neuronal; (III) late onset, mainly neuronal; or (IV) highly selective expression dynamics (Figure 2*A* and *C*; Supplementary Figures 3 and 4). Expression pattern I likely represented genes controlling neurogenesis or generic aspects of ENS lineage differentiation. Expression patterns II and III suggested roles in neuronal differentiation, perhaps of specific sublineages. The selective expression of group IV transcription factors indicated roles in neuronal subtype differentiation.

Taking all transcription factors into account, a wide range of gene families was represented (Figure 2*B*). In particular, we uncovered many genes of the Sox (HMG-box) and Hox (homeo-box) families, which play key roles in diverse developmental processes.^17^, ^18^ SOX2, SOX8, and SOX10 already have defined functions in the ENS (Supplementary Table 6). The present screen identified 5 additional SOX proteins with distinct expression patterns at various stages (Figure 2*D*; Supplementary Figures 3 and 4). SOX6 belonged to group IV and is revisited later in this study. Expression of SOX5 largely coincided with progenitors akin to the previously described SOX genes (Figure 2*A* and *D*; Supplementary Figures 3 and 4), whereas SOX4, SOX9, and SOX11 also were detected in neurons. Our screen identified 9 Hox genes in addition to the 5 already reported Hox genes (Figure 2*A*, *B*, and *E*; Supplementary Figures 2–4; Supplementary Table 6). Notably, our analysis revealed that many of the Hox genes were exclusively expressed in differentiating neurons (Figure 2*E*).

In summary, our screen uncovered a large set of novel transcription factors, the gene family and expression dynamics indicating roles for generic or specific aspects within proliferating ENSCs and/or immature neurons.

### Unique Combinatorial Expression of Transcription Factors in Enteric Neuronal Subtypes

**Figure 3:**
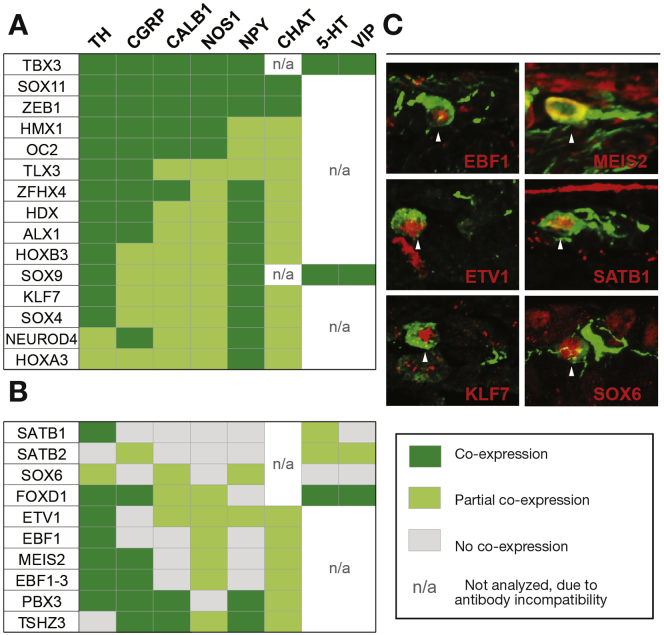
Coexpression analysis of transcription factors with enteric neurotransmitters/neuropeptides. (*A* and *B*) Tables summarizing IHC coexpression analysis of abundant (*A*) or selectively expressed (*B*) transcription factors together with ENS marker genes at E18.5. TH, NPY, 5-HT, and ChAT were analyzed in the stomach, and all other markers in the small intestine. Coexpression with SOX6 could be addressed only in the stomach. (*C*) Coexpression (arrowheads) between TH and 6 transcription factors in the stomach at E18.5. CALB1, calbindin; CGRP, calcitonin gene-related peptide; ChAT, choline acetyltransferase; 5-HT, 5-hydroxytryptamine; NOS1, nitric oxide synthase 1; NPY, neuropeptide Y; TH, tyrosine hydroxylase; VIP, vasoactive intestinal polypeptide.

To gain insights into the correlation between the newly identified transcription factors and neuronal subtypes, we next determined the colocalization pattern of 25 transcription factors with common neurotransmitter/peptide markers. Analysis at E18-19 revealed that 10 transcription factors showed selective expression with subsets of the marker proteins (Figure 3*B*), whereas others colocalized with all (Figure 3*A*). Taken together, the transcription factors constituted unique combinatorial codes for each neurotransmitter/peptide marker.

Most enteric neurotransmitters are used by several functionally distinct neuronal subclasses; however, dopamine is selectively produced in only subsets of enteric neurons.^4^ Dopamine neurons mature slowly, and at E18.5, only dopamine neurons of the stomach and not intestines have started to robustly express the indicative marker gene TH. The combinatorial expression code of the gastric TH^+^ neurons (Figure 3*A* and *B*) might therefore specifically define a single neuronal subtype, and included, for example, EBF1, MEIS2, ETV1, SATB1, KLF7, and SOX6 (Figure 3*C*).

### Loss of Sox6 Impairs Development of Gastric TH^+^ Neurons

**Figure 4:**
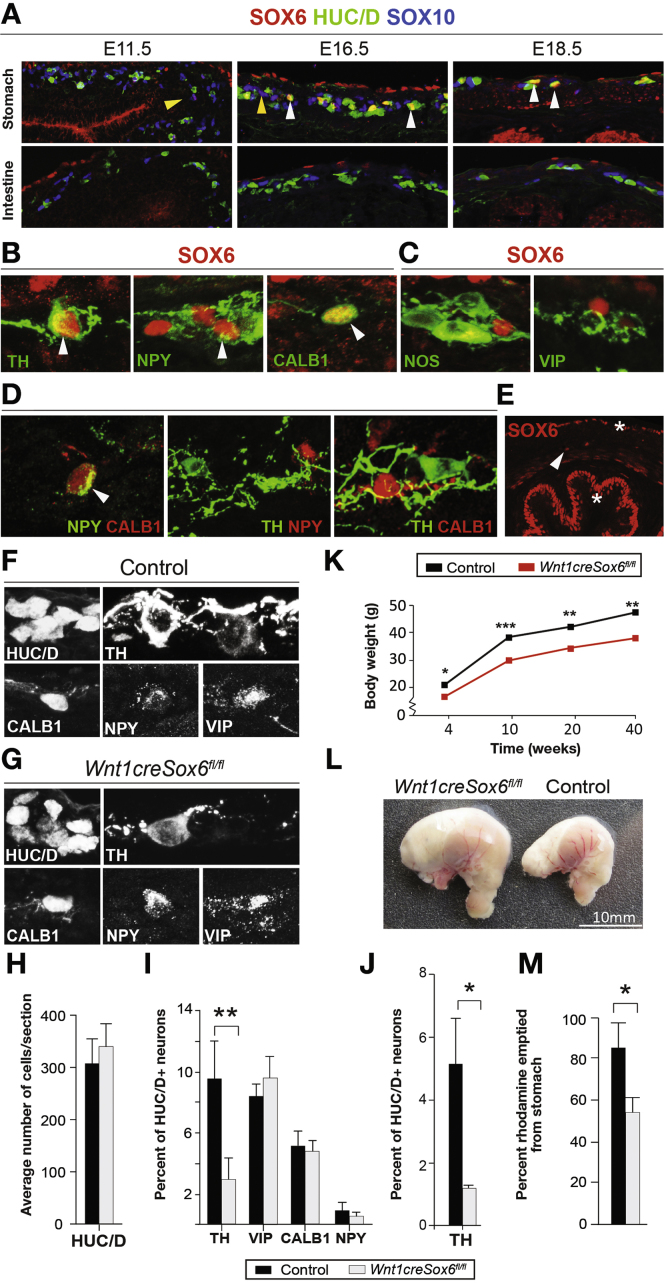
Loss of Sox6 results in selective reduction of gastric TH^+^ neurons and gastric motility. (*A*) IHC of SOX6 expression in progenitor cells (yellow arrowheads) or neurons (white arrowheads) in the developing stomach but not intestine. (*B* and *C*) IHC showing neurotransmitters that are coexpressed (arrowheads) (*B*), or not coexpressed (*C*) with SOX6 in enteric neurons at E18.5. (*D*) Pairwise IHC analysis showing expression of NPY and CALB1 with each other but not with TH in enteric neurons at E18.5. (*E*) IHC of SOX6 at E18.5 showing expression in ENS (arrowhead) and non-ENS tissue (stars). (*F* and *G*) Representative IHC images depicting expression of phenotypic marker proteins in the stomach of control embryos (*F*) and *Sox6* mutant embryos (*G*). (*H*–*J*) Average percentage of neurons expressing specific markers in the stomach of *Sox6* mutant and littermate control E18.5 embryos (*H* and *I*) or adults (*J*). n = 3–4. (*K*) Decreased weight of *Sox6* mutant males compared with littermate control mice. n = 3–5. (*L*) Enlarged stomach with more residual food in a *Sox6* mutant (left) in comparison with a control (right) mouse. n = 4. (*M*) Gastric emptying shown as percentage of administered rhodamine dextran that emptied from the stomach of *Sox6* mutant and littermate control male adult mice. n = 3 *P < .05, **P < .01, *** *P* < .001.

SOX6 colocalized with gastric TH^+^ neurons and only 2 other markers: CALB1 and NPY (Figure 3*B* and *C*; Figure 4*B* and *C*). SOX6 expression initiated at E11.5 in a small subset of SOX10^+^ progenitor cells, but gradually increased its to a higher proportion of cells (it was identified in S15vsS11) and coincided with HUC/D^+^ neurons at E15.5 (Figure 4*A*). Expression of SOX6 thus correlated well with the development of gastric dopamine neurons.

The neuronal subtypes of the stomach are poorly characterized. To gain insights to the combinatorial expression of gastric phenotypic markers, we performed pairwise IHC at E18.5. The analysis revealed that most NPY^+^ neurons displayed CALB1 expression (95%), whereas TH^+^ cells neither expressed NPY nor CALB1 (Figure 4*D*). Therefore, we concluded that SOX6 is expressed in 2 gastric subpopulations characterized by either TH or NPY/CALB1 expression.

To address the possible role of SOX6 in the generation of these neuronal subtypes, we analyzed *Wnt1Cre* × *Sox6*^*fl/fl*^ embryos at E18.5. A conditional knockout approach was crucial to attribute possible alterations in ENS development to cell autonomous effects, as SOX6 expression also localized to the serosa and mucosa (Figure 4*E*). Of the markers normally coexpressed with SOX6, TH expression was drastically reduced (by 70%), whereas CALB1^+^ and NPY^+^ neurons were found in similar numbers in mutant and littermate control stomachs (Figure 4*F*, *G*, and *I*). The total numbers of HUC/D^+^ neurons or the unrelated phenotypic marker, VIP, were unchanged in *Sox6* mutants in comparison with controls (Figure 4*F*–*I*). The reduction of TH^+^ neurons remained in the stomach of adult *Sox6* mutant mice (Figure 4*J*), which also weighed 20% less than littermate control mice (Figure 4*K*). To determine the functional impact of Sox6 deletion on gastric motility, we performed a liquid gastric transit test, which showed a reduction in the rate of gastric emptying in Sox6 mutants compared with littermate controls (Figure 4*M*). Moreover, in *Sox6* mutant mice with free access to food and drink, stomachs were enlarged with more contents than stomachs of littermate controls (Figure 4*L*). In conclusion, our analysis demonstrates that SOX6 expression is required for the specific generation of gastric TH^+^ neurons, with possible importance for normal gastric motility.

### Identification of Signaling Ligands and Receptors in the Developing Gut Wall

**Figure 5:**
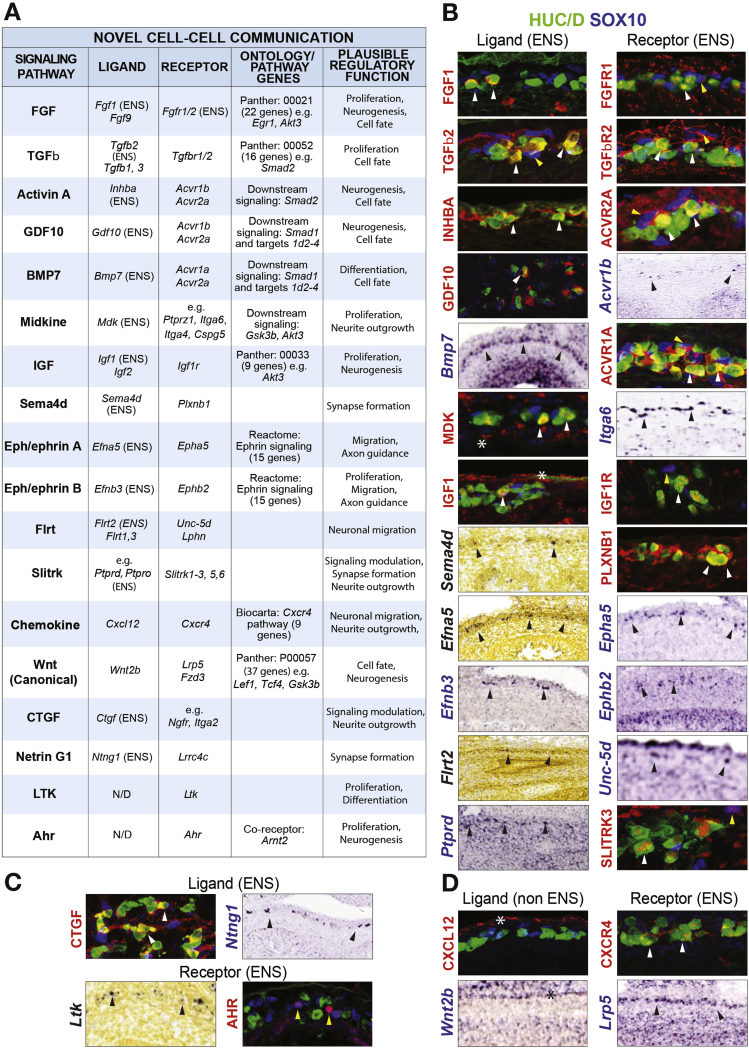
Novel cell-cell communication pathways during ENS development. (*A*) Table summarizing novel signaling pathways, including the identified ligands and receptors, signaling pathway associated genes (see Supplementary Table 8), and putative functions based on studies in other developing tissue. N/D, not determined; eg, indicates examples of binding partners found in the screen when ligand or receptor are incompletely studied or show promiscuous binding capacities. (*B*) Expression of ligand-receptor couples found within the ENS using IHC and analysis of ISH images. (*C*) IHC or ISH depicting ligands and receptors expressed in the ENS without the image of corresponding binding partners. (*D*) Expression of ligand-receptor couples using IHC and ISH, where ligands are expressed outside the ENS. Arrowheads indicate expression in HUC/D^+^ (yellow), SOX10^+^ (white), or ENS (black) cells. *Expression outside ENS. All images are shown in Supplementary Figures 5–7.

We next characterized the signaling factors and receptors found to be enriched in the transcriptome comparisons (Figure 1*E*, Supplementary Tables 4 and 5). From the combined identified genes, the expression of 157 receptors and signaling factors was first analyzed in ISH images (Supplementary Table 7, Supplementary Figure 5). Additionally, we determined the expression of 19 signaling genes in relation to HUC/D^+^ neurons and SOX10^+^ progenitors in the stomach and intestine of mouse (at E11–12, E15–16, and E18–19) and human (at W6–11) embryos (Figure 5*B* and *C*, Supplementary Figures 6 and 7, Supplementary Table 7). In total, this screen identified and confirmed the novel expression of 74 ligands and receptors. A total of 82 previously described receptors and ligands in or around the ENS were additionally found, and in a few cases included in the IHC expression analysis. With few exceptions, all signaling factors confirmed in mouse were also detected in the developing human ENS (Supplementary Figure 7). To view a comprehensive list of signaling factors/receptors expressed in the developing ENS (novel and previously known), see Supplementary Table 7.

### Novel Signaling Pathways During ENS Development

Nearly all signaling ligands and receptors previously shown to operate during ENS development were picked up by our screening strategy and belonged for example to the signaling pathways of bone morphogenetic protein (BMP), netrin (NTN), semaphorin (SEMA), and Wnt (Supplementary Table 7). In addition to previously reported components, we detected numerous novel molecules in these major signaling families. Special effort was made to reveal ligand-receptor pairs known to interact in other contexts and included BMP7-ACVR1a/ACVR2a, GDF10-ACVR1b/ACVR2a, NTNG1-LRRC4C, SEMA4d-PLXNB1, and WNT2b-LRP5/FZD3 (Figure 5*A* and *B*; Supplementary Figures 5–7). Furthermore, we found receptor-ligand expression indicative of 9 unreported signaling pathways: fibroblast growth factor (FGF), Activin A (Inhba dimers), transforming growth factor beta (TGFβ), chemokine (CXCL12), ephrin/Eph, insulin-like growth factor (IGF), fibronectin leucin rich transmembrane protein (FLRT), connective tissue growth factor (CTGF), and midkine (MDK) (Figure 5*A*–*C*; Supplementary Figures 5–7). Novel receptors with incomplete signaling characterization also were found and included in the analysis: leukocyte receptor tyrosine kinase (LTK) and aryl hydrocarbon receptor (AHR) (Figure 5*A* and *C*; Supplementary Figures 5–7). Taken together, we present signaling components indicative of 16 cell-cell communication pathways with unexplored roles in the developing ENS.

### Candidate Regulatory Factors in Auto/Paracrine Signaling During ENS Development

**Figure 6:**
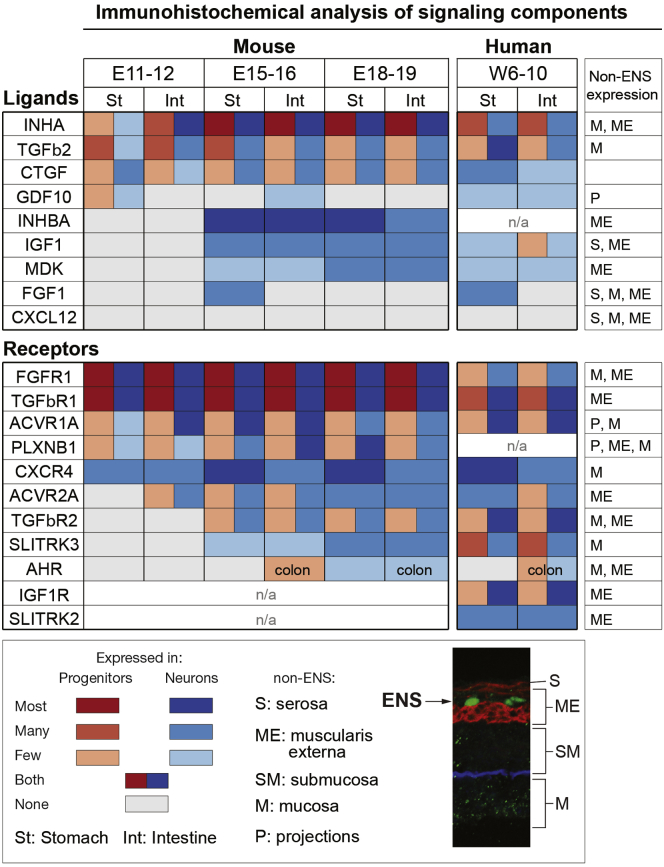
Summary of IHC analysis of cell-cell communication components in the developing gut wall. Table summarizing the IHC expression analysis of receptors and ligands in the stomach and intestine at different developmental stages in mouse and human. Column to the right indicates non-ENS expression. n/a, antibody staining inconclusive or incompatible with species.

The temporally ordered formation of specific neurons in correct proportions may be controlled by auto/paracrine signaling within the developing ENS.^16^ In light of this, it is noteworthy that 9 secreted ligands and many membrane-bound ligands were found to be produced by ENS cells (Figure 5*B* and *C*; more in Supplementary Table 7 and Supplementary Figure 5). The IHC analysis showed that most novel ligands were expressed throughout development (eg, CTGF), but several factors also displayed temporally patterned expressions: early to mid-stages (GDF10), mid-stages only (FGF1), or from mid- to late-stages (eg, MDK, Activin A) (Figure 6). In addition, some receptors showed regulated spatiotemporal expressions: AHR expression was limited to the colon between E15 and 18, whereas FGFR1 was expressed at all regions except for the colon, and SLITRK3 was found in neurons only at E15 to E18 (Figure 6).

## Discussion

**Figure 7:**
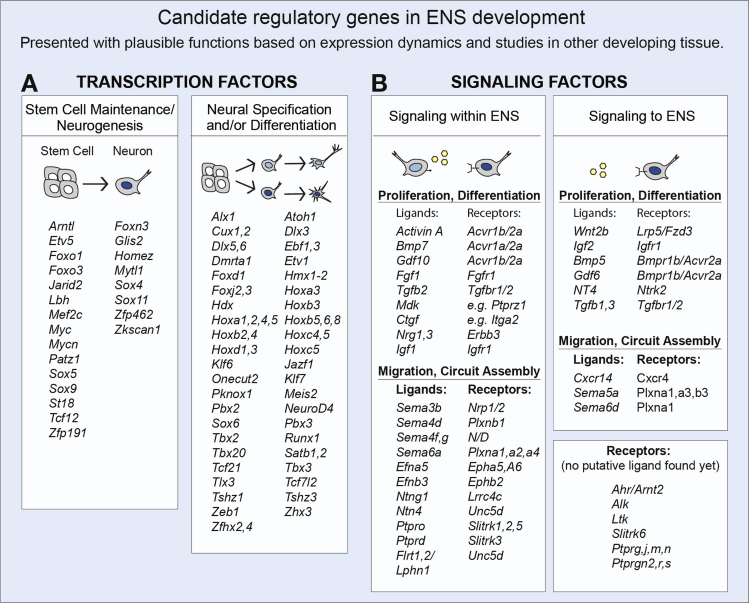
Summary of genes with putative regulatory functions in the developing ENS. (*A*) Transcription factors in the developing ENS. Left column includes genes with putative functions in stem cell maintenance versus neurogenesis. Right column includes genes with putative functions in specification and/or differentiation of enteric neurons. (*B*) Signaling factors in the developing ENS. Ligands expressed in the ENS (left column) or in proximal gut tissue (right column) presented with putative receptors. Ligand/receptor couples are likely involved in proliferation/differentiation or migration/network formation as indicated. See Supplementary Tables 6 and 7 for more information.

Recapitulation of developmental programs will be an important tool in future cell-engineering to treat enteric neuropathies. Through a comprehensive transcriptome and histochemical analysis in mouse and human developing ENS, we have here identified substantial numbers of transcription factors and signaling pathways with likely roles in stem cell maintenance/neurogenesis, neuronal specification/differentiation, and neural connectivity. A summary of key candidate genes with respect to plausible functions is depicted in Figure 7 and discussed as follows.

### Candidate Transcription Factors in ENS Stem Cell Maintenance and Differentiation

To ensure functional ganglia along the full extent of the gut, the relatively small number of ENSCs must balance extensive expansion with differentiation. The expression dynamics of SOX4, 5, 9, and 11 shown here suggest that they complement the currently known set of SOX genes (2, 8, and 10) in the sequential differentiation process of ENS stem cells to neurons, akin to their intricate functions in the developing spinal cord.^17^ Other novel genes with likely regulatory roles in proliferation/neurogenesis include, for instance, the Nzf-family (*Myt1l, Myt1,* and *St18*) (Figure 7*A*).

### Sox6, Dopamine Neurons, and Parkinson’s Disease

In the central nervous system, the set of transcription factors expressed in progenitor cells as they commence neurogenesis initiate a molecular program of subtype-specific differentiation.^19^ Several lines of evidence show that enteric neuron specification follows a similar mechanistic logic. First, the subtype fate of an enteric neuron depends on the time point when it was generated during embryogenesis.^10^ Second, clonal lineage-tracing indicates that progenitors undergoing their last cell cycle are fixed to a particular neuronal subfate.^20^ Corroborating these findings, our study uncovered that SOX6 expression correlates with dopamine neuron birth^11^ and is necessary for acquisition of the dopaminergic trait. The transcription factor ASCL1 also regulates generation of gastric dopamine neurons; however, several other neuronal subtypes as well.^9^ SOX6 is thus the first transcription factor linked to the generation of a single neuronal subtype in the developing ENS.

Within the midbrain, SOX6 is similarly important for the generation of substantia nigra dopamine neurons, and lower levels of SOX6 accompany the loss of these neurons in patients with Parkinson’s disease.^21^ Although Parkinson’s disease is classified as a movement disorder of the brain, most patients also suffer from GI dysfunction, including gastroparesis and constipation.^22^ Characteristic Parkinson pathology has been observed in TH^+^ (and vasoactive intestinal polypeptide–positive) enteric neurons, primarily in anterior gut regions.^22^ We note that the reduced numbers of TH^+^ gastric neurons and the gastric emptying phenotype in *Sox6* mutant mice correlate well with these symptoms. Sophisticated solid gastric emptying tests and investigation of the lower-GI tract on large cohorts of *Sox6* mutant mice could perhaps further delineate the role of TH^+^ neurons in gastrointestinal physiology. Mounting experimental evidence suggests that Parkinson pathology may initiate in the ENS and subsequently spread to the brain.^22^ TH^+^ gastric neurons may thus be relevant to understand early pathological events in Parkinson’s disease. We found a battery of genes, shared between gastric and brain dopamine neurons (Figure 3*C*),^23^, ^24^, ^25^, ^26^ indicating conservation of gene regulatory networks in different types of dopamine neurons, possibly forming a basis for further studies of dopamine neuron pathology.

### Candidate Transcription Factors in ENS Cell Diversification

We identified Hox genes with an unanticipated expression in enteric neurons. Reminiscent of this, sets of Hox proteins are differentially expressed in spinal motor neuron subclasses, where they function in neuronal subtype differentiation, morphogenesis, and synaptic specificity.^18^ To impose differential transcriptional read-out, Hox proteins depend on the interaction with members of the 3 amino acid loop extension (TALE) family. Four TALE genes were found in the developing ENS (*Pbx2*, *Pbx3*, *Meis2*, and *Pknox1*). A gene network analysis predicted a high level of interactions between the ENS-expressed TALE and Hox proteins (Supplementary Figure 8). We therefore propose that combinatorial expression of various HOX and TALE proteins may contribute to the acquisition of subtype-specific features in enteric neurons.

Our transcriptome screen also identified, for example, *Foxd1, Cux2,* and *Tbx20* as plausible regulators of enteric neuronal specification, and *Atoh1* and *Runx1* in postmitotic subtype differentiation (Figure 7*A*). Even promiscuously expressed transcription factors (eg, *Onecut2* and *Hmx1*) may contribute to differentiation programs in various enteric neuronal subtypes by forming distinct combinatorial expression patterns.

### Candidate Signaling Pathways Mediating Autonomous Development of the ENS

This study demonstrates several signaling pathways with plausible roles in the fine-tuned orchestration of proliferation, neurogenesis, neuronal diversification, and network formation, which are needed for establishment of functional enteric networks (Figure 7*B*).

IGF1, LTK, AHR, MDK, GDF10, Activin A, and NRG1 have been attributed neurotrophic, gliogenic, or neurogenic activities in developing nervous tissue.^27^, ^28^, ^29^, ^30^, ^31^, ^32^, ^33^ Thus, our identification of these signaling factors within the developing ENS indicates that immature enteric neurons could play an active role in controlling generation of the appropriate numbers and ratio of glia and neurons (Figure 7*B*).

The idea that phenotypically distinct neurons are generated in a temporally defined manner currently lacks a clear underlying molecular mechanism. One hypothesis states that ENS diversity is governed by self-regulatory mechanisms where early-born neurons would affect the genesis of later-born neurons.^16^ Among identified factors, FGF, TGFβ, and BMP7 have important instructive roles during the sequential generation of neuronal subtypes in other developing tissues,^34^, ^35^, ^36^ and are thus prime candidates as auto/paracrine regulators of ENS diversification (Figure 7*B*). Cell fate also can be influenced in a temporal manner by differential expression of modulatory proteins. CTGF is a modulatory factor that can adjust TGFβ and BMP7 signaling,^37^ whereas INHA forms inhibitory dimers with INHBA, thus preventing Activin A signaling.^38^ It is therefore possible that the differential levels of CTGF and INHA expression found in the ENS (Supplementary Figures 6 and 7) bestows ENS subpopulations a means to modify their response to signaling ligands.

Establishment of fine-tuned connectivity patterns of appropriate synapses is required for the functionality of enteric networks. Recent studies of brain development have implicated SEMA4d-PLXNB1, NTNG1-LRRC4C, SLITRK2-PTPRO, SLITRK3-PTPRD, and FLRT–LPHN/UNC5D in the formation of selective synapses.^39^, ^40^, ^41^, ^42^, ^43^ Our identification of these signaling molecules in the developing ENS thus provides novel candidate genes with likely roles in mediating precise synaptic connections during enteric circuit formation.

### Future Treatments of Enteric Neuropathies

Cell-based regenerative medicine is considered a promising future therapeutic approach for Hirschsprung disease, but may initially be more applicable for disorders in which small phenotypic changes underlie ENS dysfunction.^2^ Substantial progress has been made in deriving ENSCs that can survive, proliferate, and migrate on transplantation; however, the challenge of controlling neuronal differentiation remains. We have here aspired to provide an extensive expression pattern resource of mouse and human developing ENS for basic understanding of ENS diversification and implementation in translational studies. The important role of Sox6 for gastric dopamine neurons encourages further exploration of this and other identified genes (Figure 7) for their possible capacity to influence subtype-specific or other aspects of ENS cell development; abilities that could advance the engineering of clinically relevant ENS cells. Notably, the expression signature of in vitro–derived ENS stem cells^3^ contained many of the genes we describe (eg, *Tlx3*, *Hoxb2*, and *Tbx2*). Cross-comparisons to our histological gene expression maps could therefore provide valuable insights into the cellular composition and maturity stage of human ENS cell cultures, being essential for further development of this tool.

## Supplementary Material

Supplementary Table of Contents
