# Supplementary material for: Transcription and Signaling Regulators in Developing Neuronal Subtypes of Mouse and Human Enteric Nervous System
Source: Gastroenterology. 2018 Feb;154(3):624–36. doi: 10.1053/j.gastro.2017.10.005 (PMC6381388; doi:10.1053/j.gastro.2017.10.005)
Supplement: Supplementary Table of Contents [file mmc1.pdf]

SUPPLEMENTARY TABLE OF CONTENTS:

Supplementary Figure 1: Preparation of cells and RNA used in microarrays.

Supplementary Figure 2: In situ hybridisation images of transcription factors in the developing mouse ENS.

Supplementary Figure 3: Immunohistochemical analysis of transcription factors in the developing mouse ENS.

Supplementary Figure 4: Immunohistochemical analysis of transcription factors in the developing human ENS.

Supplementary Figure 5: In situ hybridisation images of signalling factors in the developing mouse ENS.

Supplementary Figure 6: Immunohistochemical analysis of cell-cell communication components in the developing mouse ENS.

Supplementary Figure 7: Immunohistochemical analysis of cell-cell communication components in the developing human ENS.

Supplementary Figure 8: STRING interaction scheme of Hox and TALE-genes.

Supplementary Text: Comments and Discussion of Material and Methods.

Supplementary Table 1: Antibodies

Supplementary Table 2: Enriched genes in pairwise comparisons between cell populations in the developing gut (xls).

Supplementary Table 3: Transcription Factors enriched (>1.2) in pairwise comparisons between cell populations in the developing gut. (xls).

Supplementary Table 4: Signalling ligands enriched (>1.2) in pairwise comparisons between cell populations in the developing gut. (xls).

Supplementary Table 5: Signalling receptors enriched (>1.2) in pairwise comparisons between cell populations in the developing gut. (xls).

Supplementary Table 6: Transcription factors in the developing ENS.

Supplementary Table 7: Signaling Pathways in the developing ENS.

Supplementary Table 8: ENS enriched genes associated with signalling pathways.
