# Supplementary material for: Transcription and Signaling Regulators in Developing Neuronal Subtypes of Mouse and Human Enteric Nervous System
Source: Gastroenterology. 2018 Feb;154(3):624–36. doi: 10.1053/j.gastro.2017.10.005 (PMC6381388; doi:10.1053/j.gastro.2017.10.005)
Supplement: Supplementary Text [file mmc2.pdf]

Supplementary Text – Comments and Discussion of Material and Methods

Tracing of SOX10$^+$ cells in *Sox10CreER^{T2} x R26ReYFP* embryos:
20 h duration between 4-OHT and harvesting of embryos resulted in efficient and specific labeling of SOX10$^+$ enteric cells as verified by IHC on sectioned tissue. 95±2.3% at E11.5 and 90±0.4% at E15.5, of Sox10$^+$ cells expressed YFP. However, a sub-population of YFP$^+$ cells expressed the neuronal marker HuC/D (9.3±3.9% at E11.5 and 13.8±0.9% at E15.5). This population likely contained dividing HuC/D$^+$/SOX10$^+$ cells[1] and newly formed non-dividing immature HuC/D$^+$/SOX10$^-$ neurons. Since the proportion of differentiating neurons in the ENS is overall low at E11.5, the S11 and W11 data-sets were rather alike and only few genes were differentially expressed in the W11vsS11 comparison (Supplementary Table 2).

Considerations for transcriptome pairwise comparison analysis:
YFP-expressing cells of the *Sox10CreER^{T2}* x *R26ReYFP* line showed variable strength (compare FACS plots from the *Wnt1Cre x R26ReYFP* and *Sox10CreER^{T2}* x *R26ReYFP* lines; Supplementary Figure 1). This resulted in a less clear separation of YFP$^+$ and YFP$^-$ cells in samples from the *Sox10CreER^{T2}* x *R26ReYFP* and therefore a certain degree of impurity in the S11 and S15 data-sets. Thus, a number of non-ENS genes were detected in for example the S11vsS15 comparison, including the hematopoetic genes *Hbb-y*, *Hbb-x*, *Hbb-bh1,* and *Notch3*; and mesenchymally expressed transcription factors: *Nkx6.1, Hand1, Isl1* and *Gata3*. S15vsS11 was enriched for pancreatic amylase genes (*Amy2a5, Amy2a1*) and hematopoetic gene *Hbb-b1*. These genes were removed in the analysis described in Figure 1B.

The pairwise comparisons between enteric populations and non-ENS gut control tissue were generally the most sensitive and potent in detecting novel ENS-expressed genes. However, the IHC analysis revealed many novel genes with selective expression both in ENS and non-ENS cells, which could only be identified in the pairwise ENS-vs-ENS comparisons (e.g. *Ebf1*, *Meis2, Runx1, Sox6* and *Zfhx4*). Hence, all eight pair-wise comparisons were relevant and complemented each other in the search for novel ENS-expressed genes.

In the very most cases, our IHC analysis verified the expression dynamics indicated by the microarray. However, a few deviations were observed which perhaps could be explained by a natural difference between RNA (microarray) and protein expression (IHC), the relative rather than absolute expression dynamics in pair-wise transcriptomic analyses and possible limitation in specificity/sensitivity of antibodies.

1. Memic F, Knoflach V, Sadler R, et al. Ascl1 Is Required for the Development of Specific Neuronal Subtypes in the Enteric Nervous System. J Neurosci 2016;36:4339-50.
