# Supplementary material for: Transcription and Signaling Regulators in Developing Neuronal Subtypes of Mouse and Human Enteric Nervous System
Source: Gastroenterology. 2018 Feb;154(3):624–36. doi: 10.1053/j.gastro.2017.10.005 (PMC6381388; doi:10.1053/j.gastro.2017.10.005)
Supplement: Supplementary Figure 1 [file mmc3.pdf]

SUPPLEMENTARY FIGURE 1

A

Sample	Stage	Cell Type	guts (no.)	cells (no.)	ng/ul	ul	RIN	BioAnalyzer
S11:1	E11.5	Sox10+	64	18966	4,84	13	9,6	OK
S11:2	E11.5	Sox10+	51	19666	4,77	13	9	OK
S11:3	E11.5	Sox10+	56	17606	4,49	13	9,2	OK
S11:4	E11.5	Sox10+	74	21088	7,41	12	8,5	OK
S15:1	E15.5	Sox10+	3	30300	5,46	11	8,7	OK
S15:2	E15.5	Sox10+	3	29000	6,76	11	9,2	OK
S15:3	E15.5	Sox10+	3	37000	2,95	11	9,3	OK
S15:4	E15.5	Sox10+	3	39488	7,87	11	8,2	OK
W11:1	E11.5	ENS	17	17721	7,5	11	9	OK
W11:2	E11.5	ENS	29	19831	2,81	13	9,2	OK
W11:3	E11.5	ENS	26	25887	11,39	12	7,6	OK
C11:1	E11.5	Ctrl (Wnt1-)	17	416800	33,68	33	7,4	OK
C11:2	E11.5	Ctrl (Wnt1-)	29	329743	28,33	37	8,7	OK
C11:3	E11.5	Ctrl (Wnt1-)	26	424000	65,5	24	N/D	OK
W15:1	E15.5	ENS	3	89700	16,32	11	8,6	OK
W15:2	E15.5	ENS	3	114700	27,98	11	8,5	OK
W15:3	E15.5	ENS	3	103777	14,73	11	7,3	OK
C15:1	E15.5	Ctrl (Wnt1-)	3	952000	48,43	33	9,6	OK
C15:2	E15.5	Ctrl (Wnt1-)	3	894999	53,93	37	7,8	OK
C15:3	E15.5	Ctrl (Wnt1-)	3	722000	51,29	37	7,3	OK

D Bioanalysis

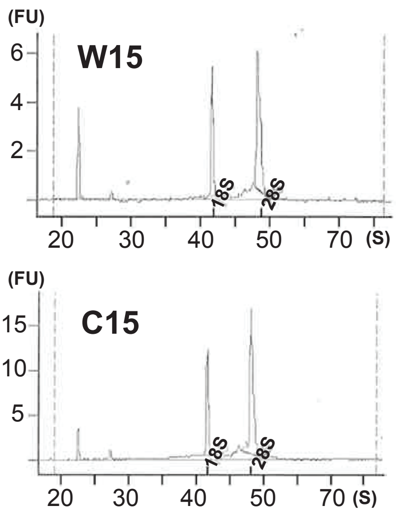

B *Wnt1Cre x R26ReYFP* at E15.5

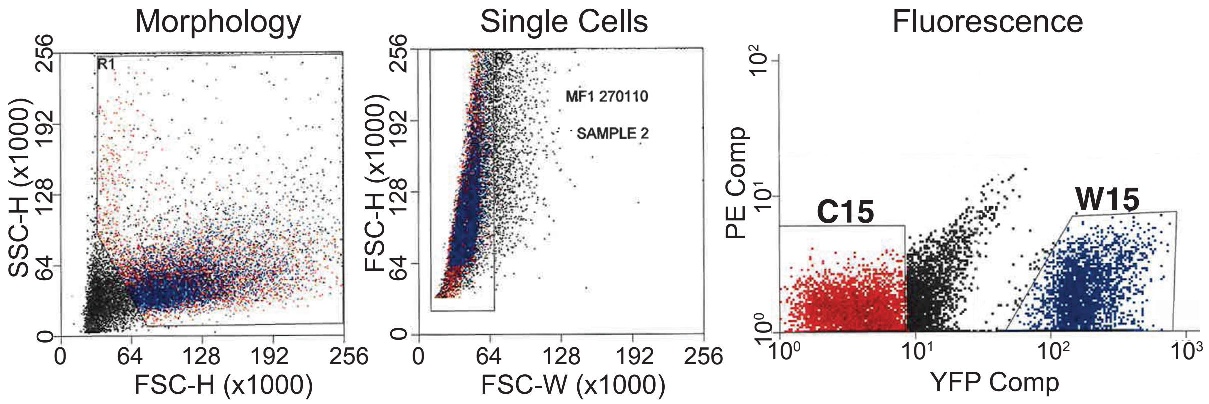

C *Sox10CreER^{T2} x R26ReYFP* at E15.5

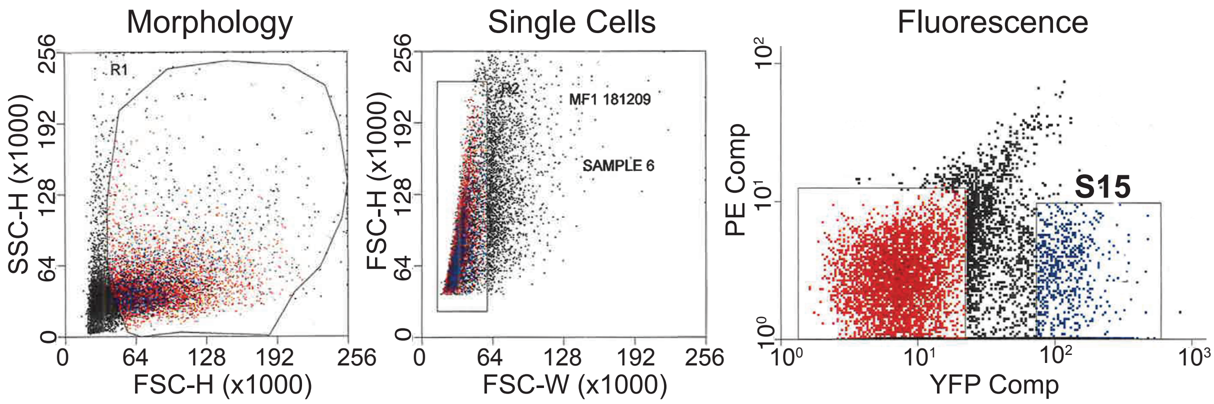

Supplementary Figure 1: Preparation of cells and RNA used in microarrays.
A) Summary of analyzed pooled samples, including starting material and amount /quality of retreived RNA. Note that the same absolute amount of RNA from each sample was used to prepare cDNA to be hybridised on microarrays.
B,C) Examples of FACS sortings of YFP^{+} or YFP^{-} cells of one gut from each of the *Wnt1Cre-R26ReYFP* (B) and *Sox10CreER^{T2}-R26ReYFP* (C) mouse lines.
D) Examples of RNA analysis using Bioanalyzer. RIN: RNA Integrity Number; FSC:forward-scattered light; SSC:side-scattered light;-H(height); -W(width); PE: phycoerythrin, used to detect autofluorescence; YFP:yellow fluorescent protein, reporter gene; Comp: compensated data; N/D: not determined;
FU: fluorescence absorption units; s:seconds.
