# Supplementary material for: Transcription and Signaling Regulators in Developing Neuronal Subtypes of Mouse and Human Enteric Nervous System
Source: Gastroenterology. 2018 Feb;154(3):624–36. doi: 10.1053/j.gastro.2017.10.005 (PMC6381388; doi:10.1053/j.gastro.2017.10.005)
Supplement: Supplementary Figure 2 [file mmc4.pdf]

SUPPLEMENTARY FIGURE 2

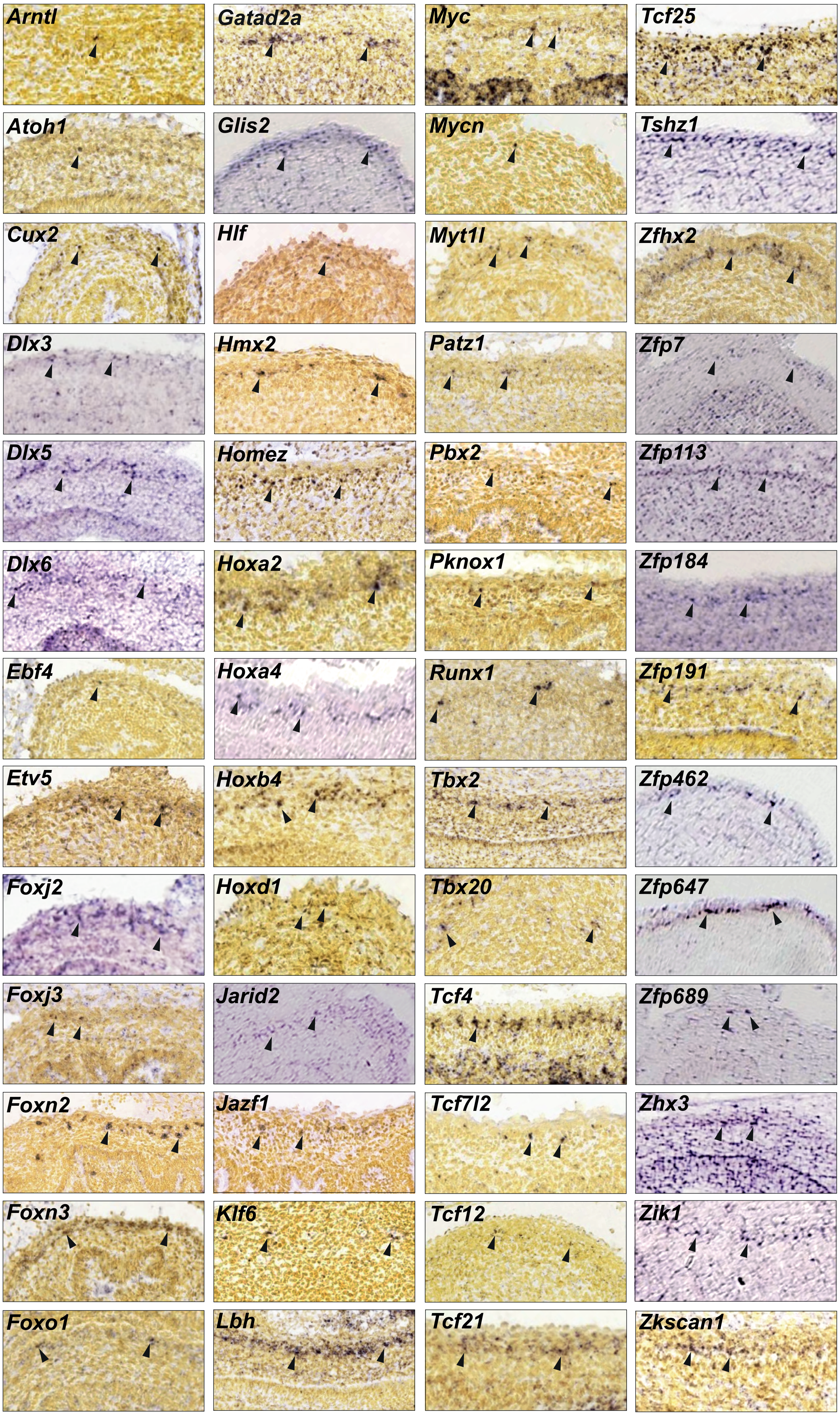

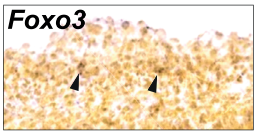

Supplementary Figure 2: RNA expression of novel transcription factors in the developing ENS.
ISH (Allen Brain Atlas or GenePaint) of transcription factors indicate expression in the ENS (arrowheads). Pictures show gut at E13-15, except *Klf6* and *Tbx20*, which are depicted at E11.5.
