# Supplementary material for: Transcription and Signaling Regulators in Developing Neuronal Subtypes of Mouse and Human Enteric Nervous System
Source: Gastroenterology. 2018 Feb;154(3):624–36. doi: 10.1053/j.gastro.2017.10.005 (PMC6381388; doi:10.1053/j.gastro.2017.10.005)
Supplement: Supplementary Figure 3 [file mmc5.pdf]

SUPPLEMENTARY FIGURE 3

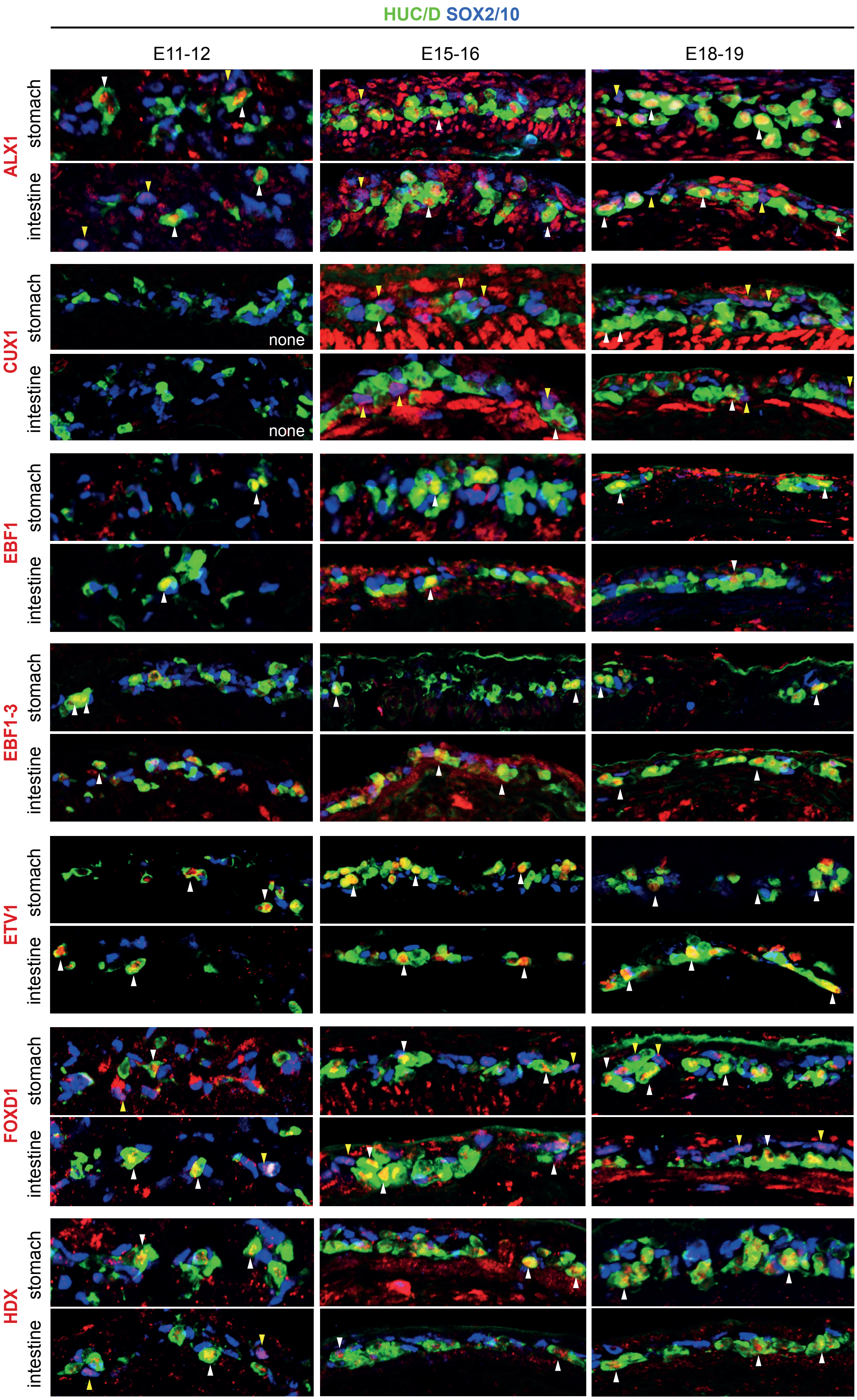

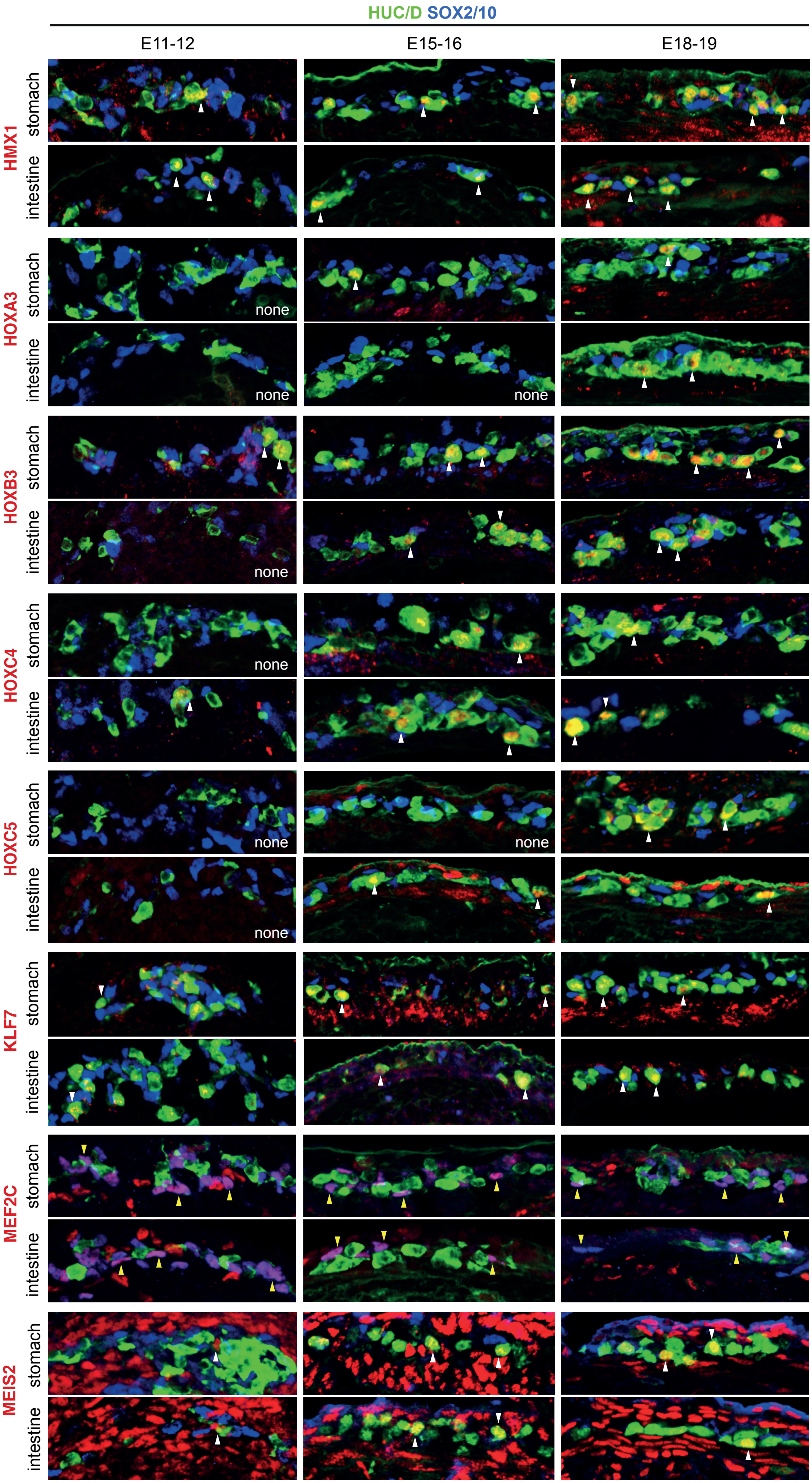

HUC/D SOX2/10
E11-12
E15-16
E18-19
HMX1
HOXA3
HOXB3
HOXC4
HOXC5
KLF7
MEF2C
MEIS2
stomach
intestine
none

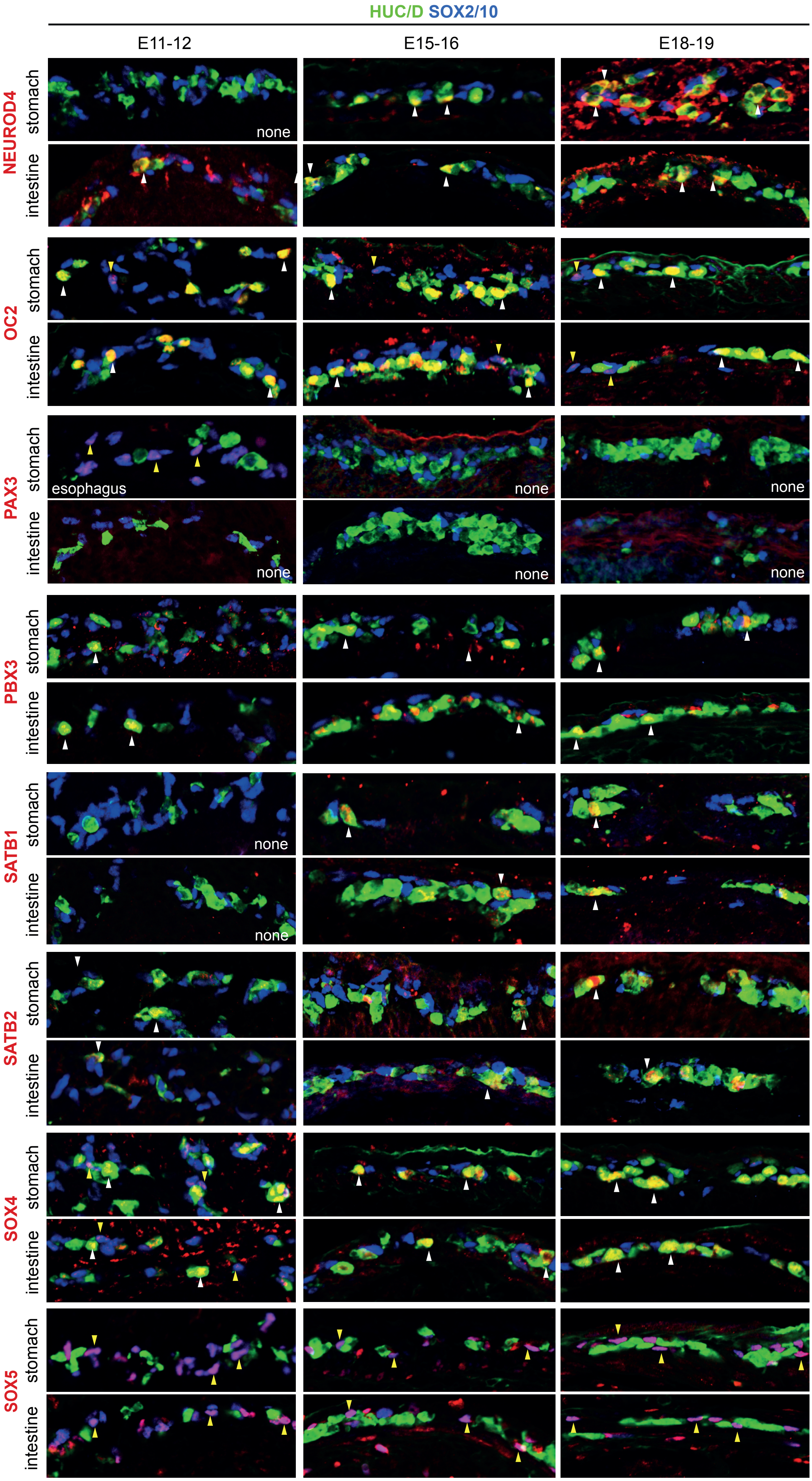

HUC/D SOX2/10
E11-12
E15-16
E18-19
NEUROD4
OC2
PAX3
PBX3
SATB1
SATB2
SOX4
SOX5
stomach
intestine
none
esophagus

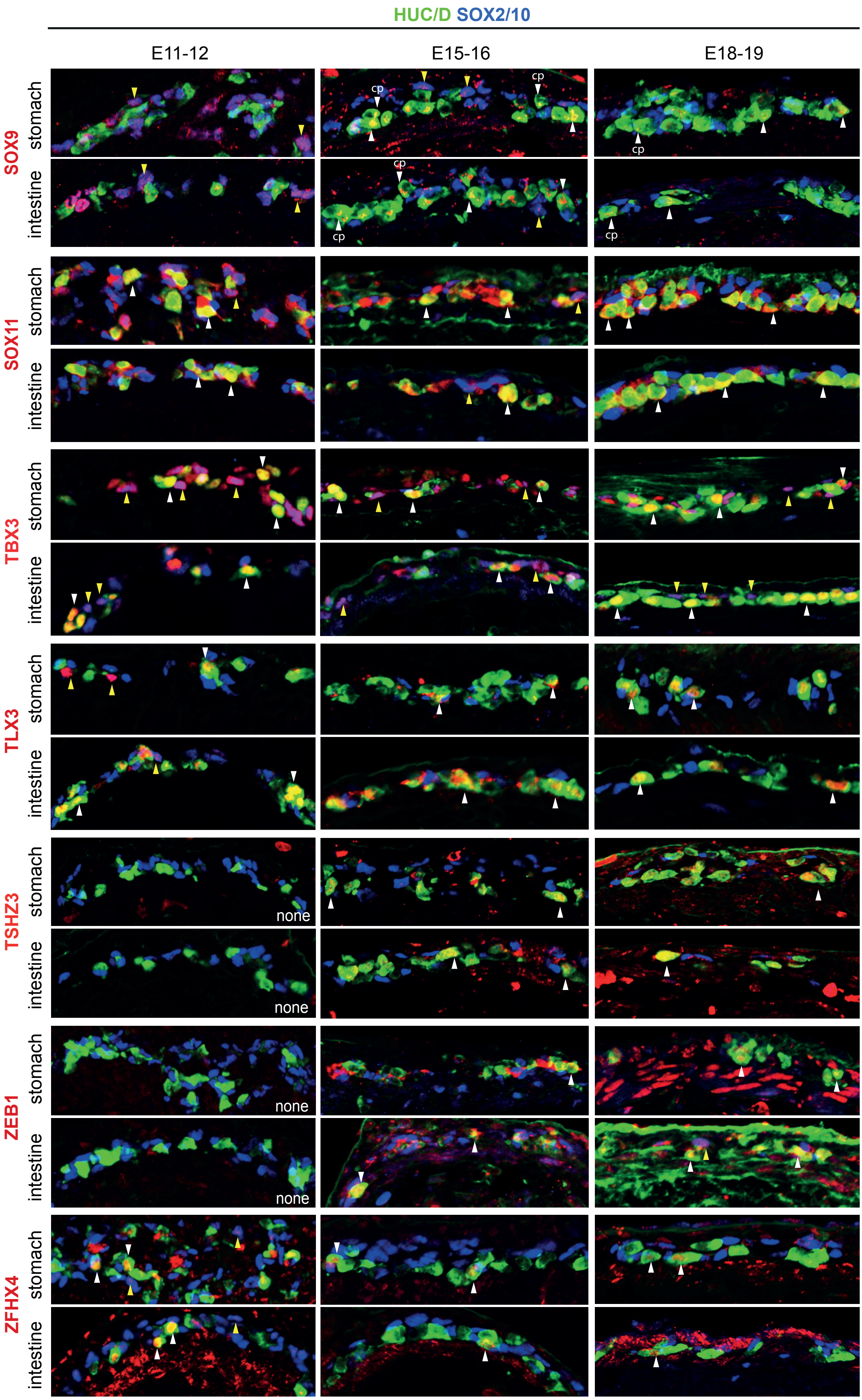

Supplementary Figure 3: IHC analysis of transcription factors in the developing mouse ENS. Co-expression analysis of transcription factors with the neuronal marker HUC/D (white arrowheads) and the progenitor marker SOX2/10 (yellow arrowheads) at E11-12, E15-16 and E18-19 in stomach and intestine of mouse embryos. “cp” above arrowhead indicates cytoplasmatic expression (SOX9).
