# Supplementary material for: Transcription and Signaling Regulators in Developing Neuronal Subtypes of Mouse and Human Enteric Nervous System
Source: Gastroenterology. 2018 Feb;154(3):624–36. doi: 10.1053/j.gastro.2017.10.005 (PMC6381388; doi:10.1053/j.gastro.2017.10.005)
Supplement: Supplementary Figure 4 [file mmc6.pdf]

SUPPLEMENTARY FIGURE 4

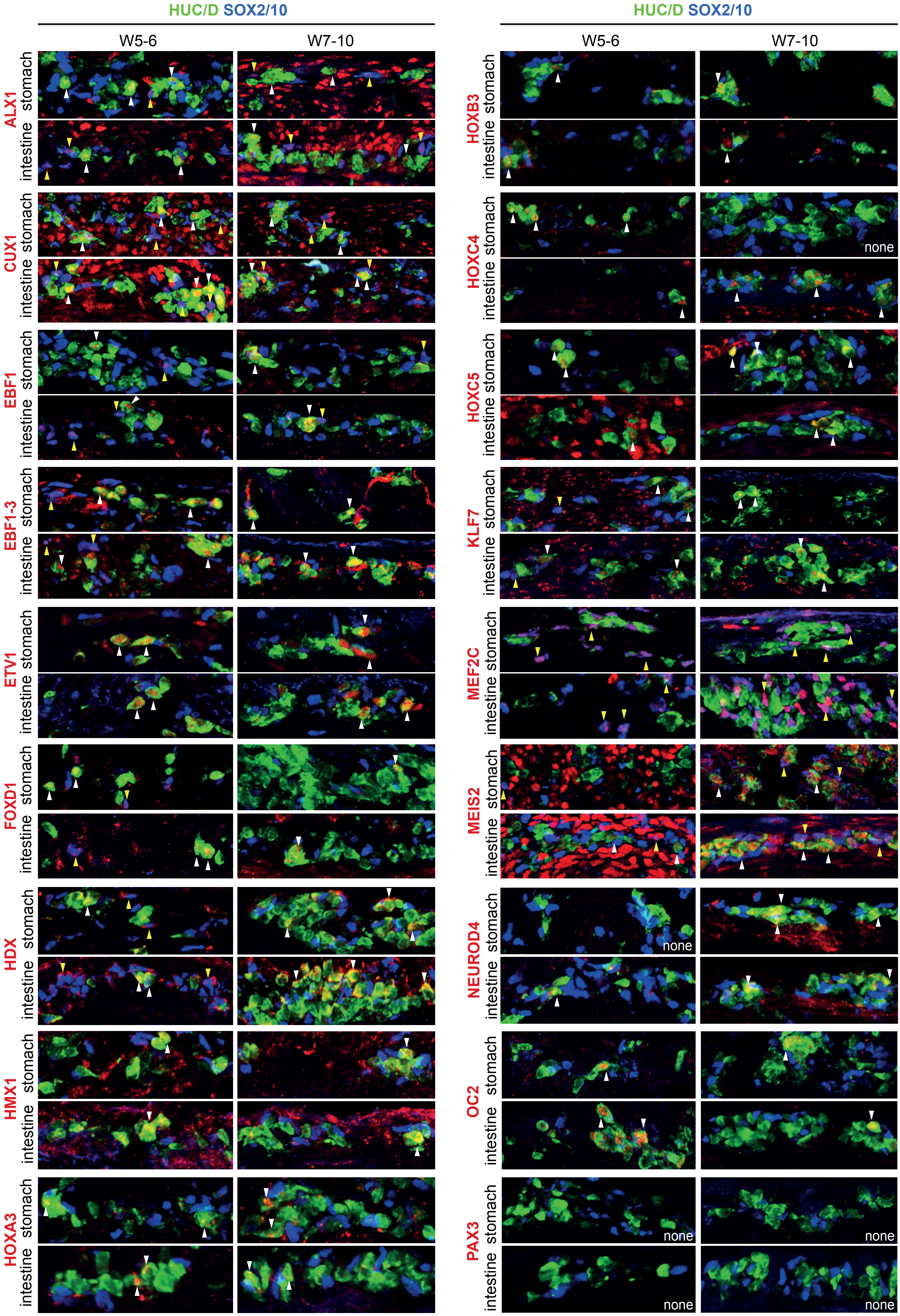

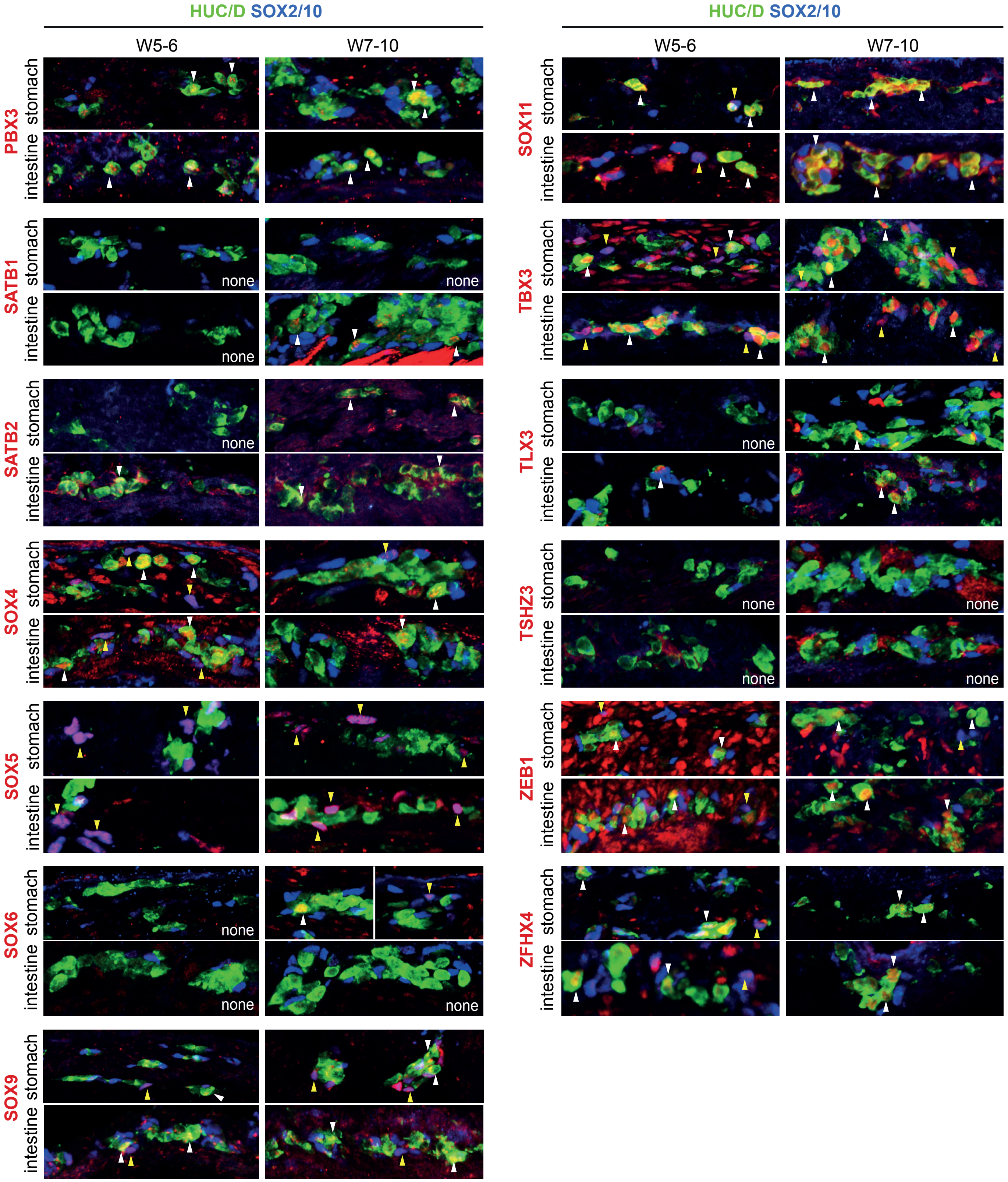

Supplementary Figure 4: IHC analysis of transcription factors in the developing human ENS. Co-expression analysis of transcription factors with the neuronal marker HUC/D (white arrowheads) and the progenitor marker SOX2/10 (yellow arrowheads) at W5-6 and W7-10 in stomach and intestine of human embryos.
