# Supplementary material for: Transcription and Signaling Regulators in Developing Neuronal Subtypes of Mouse and Human Enteric Nervous System
Source: Gastroenterology. 2018 Feb;154(3):624–36. doi: 10.1053/j.gastro.2017.10.005 (PMC6381388; doi:10.1053/j.gastro.2017.10.005)
Supplement: Supplementary Figure 5 [file mmc7.pdf]

SUPPLEMENTARY FIGURE 5

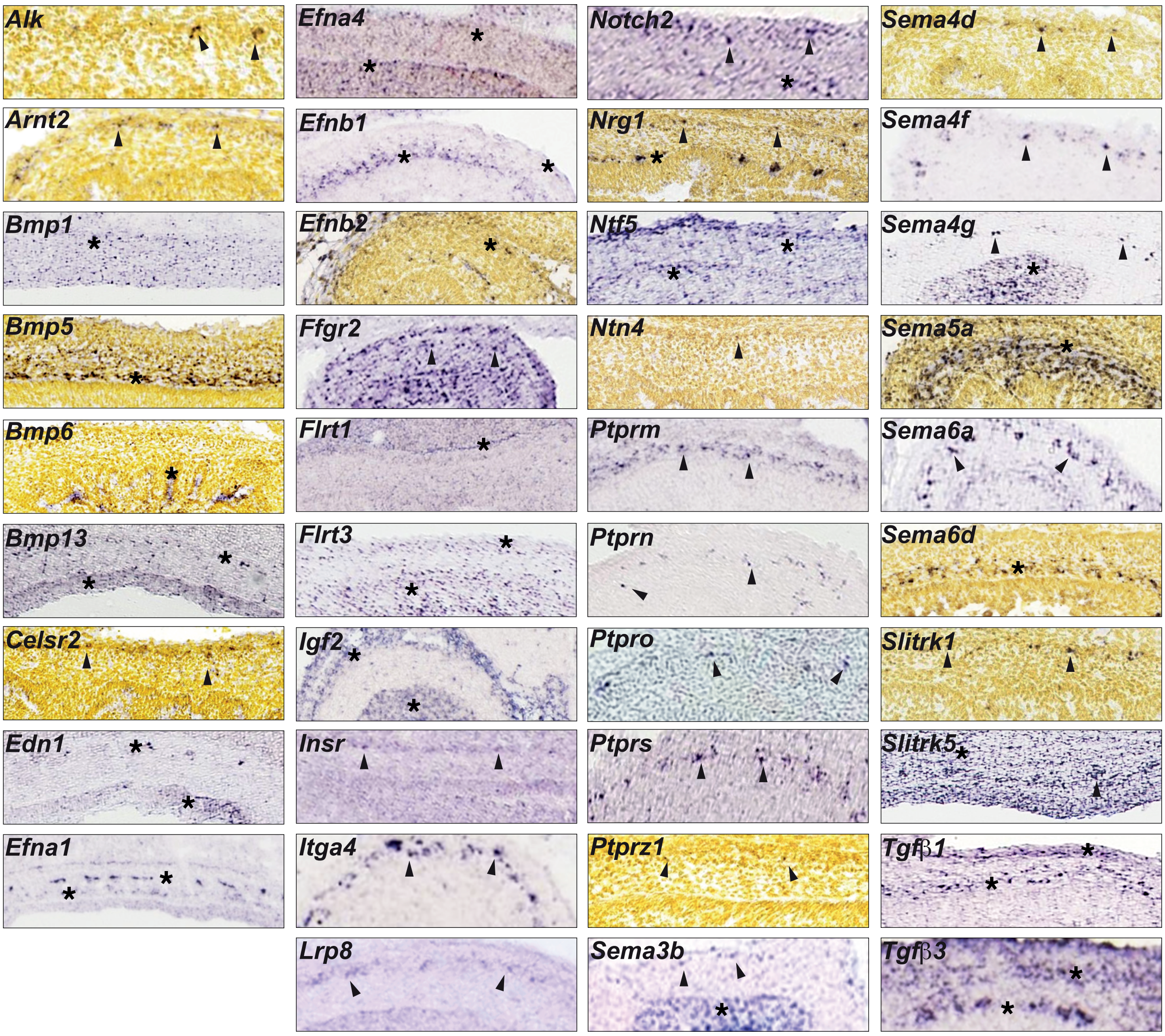

Supplementary Figure 5: RNA expression of novel signaling ligands and receptors in the developing bowel wall. ISH (Allen Brain Atlas or GenePaint) of signaling factors and their receptors at E14-15. Arrowheads indicate expression in the ENS, stars indicate expression in non-ENS bowel tissue. This summary also includes signaling components not highlighted in figure 5, but listed in supplementary table 7.
