# Supplementary material for: Transcription and Signaling Regulators in Developing Neuronal Subtypes of Mouse and Human Enteric Nervous System
Source: Gastroenterology. 2018 Feb;154(3):624–36. doi: 10.1053/j.gastro.2017.10.005 (PMC6381388; doi:10.1053/j.gastro.2017.10.005)
Supplement: Supplementary Figure 6 [file mmc8.pdf]

SUPPLEMENTARY FIGURE 6

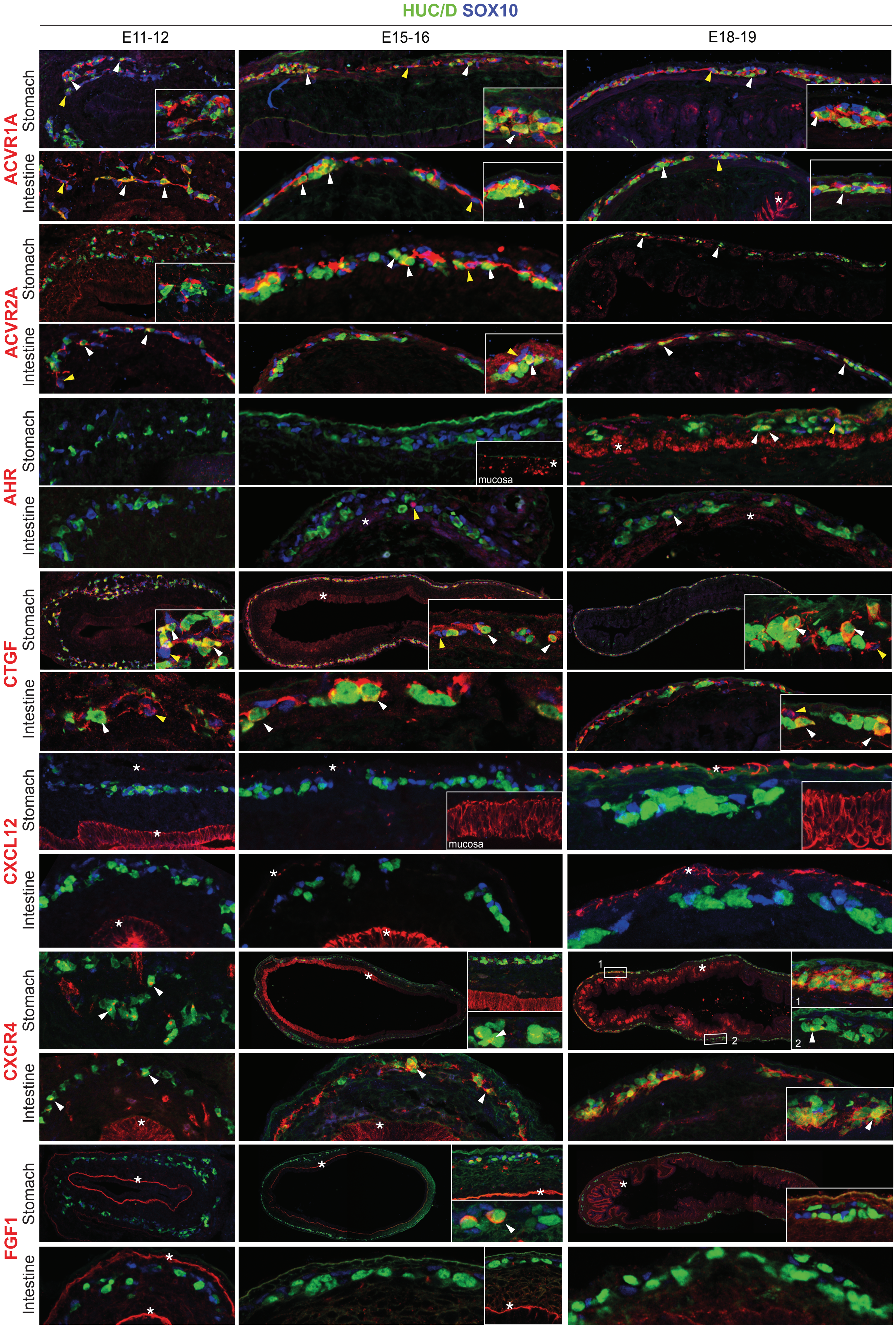

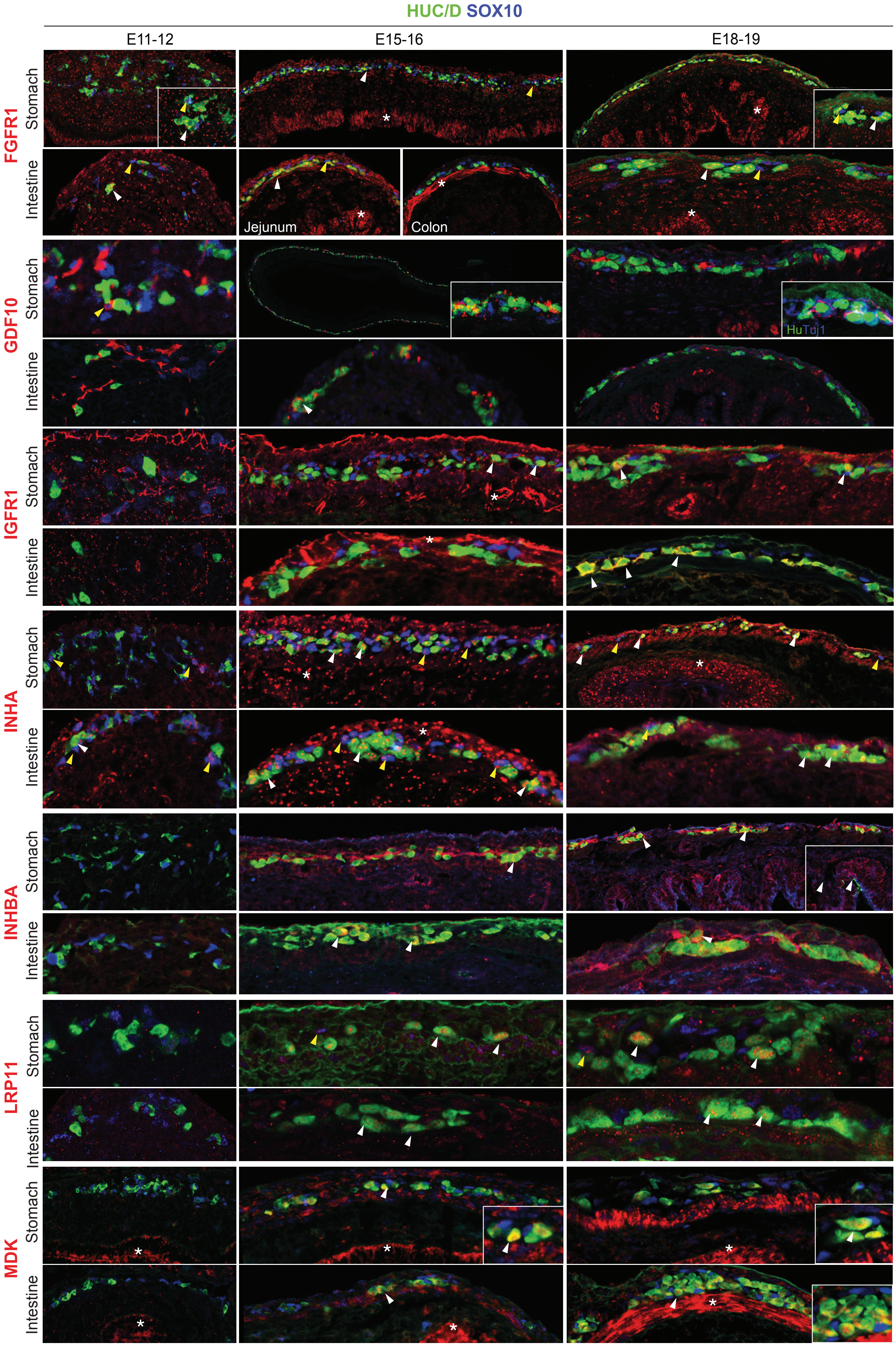
HUC/D SOX10
E11-12
E15-16
E18-19
FGFR1
Stomach
Intestine
Jejunum
Colon
GDF10
HuTuj1
IGFR1
INHA
INHBA
LRP11
MDK

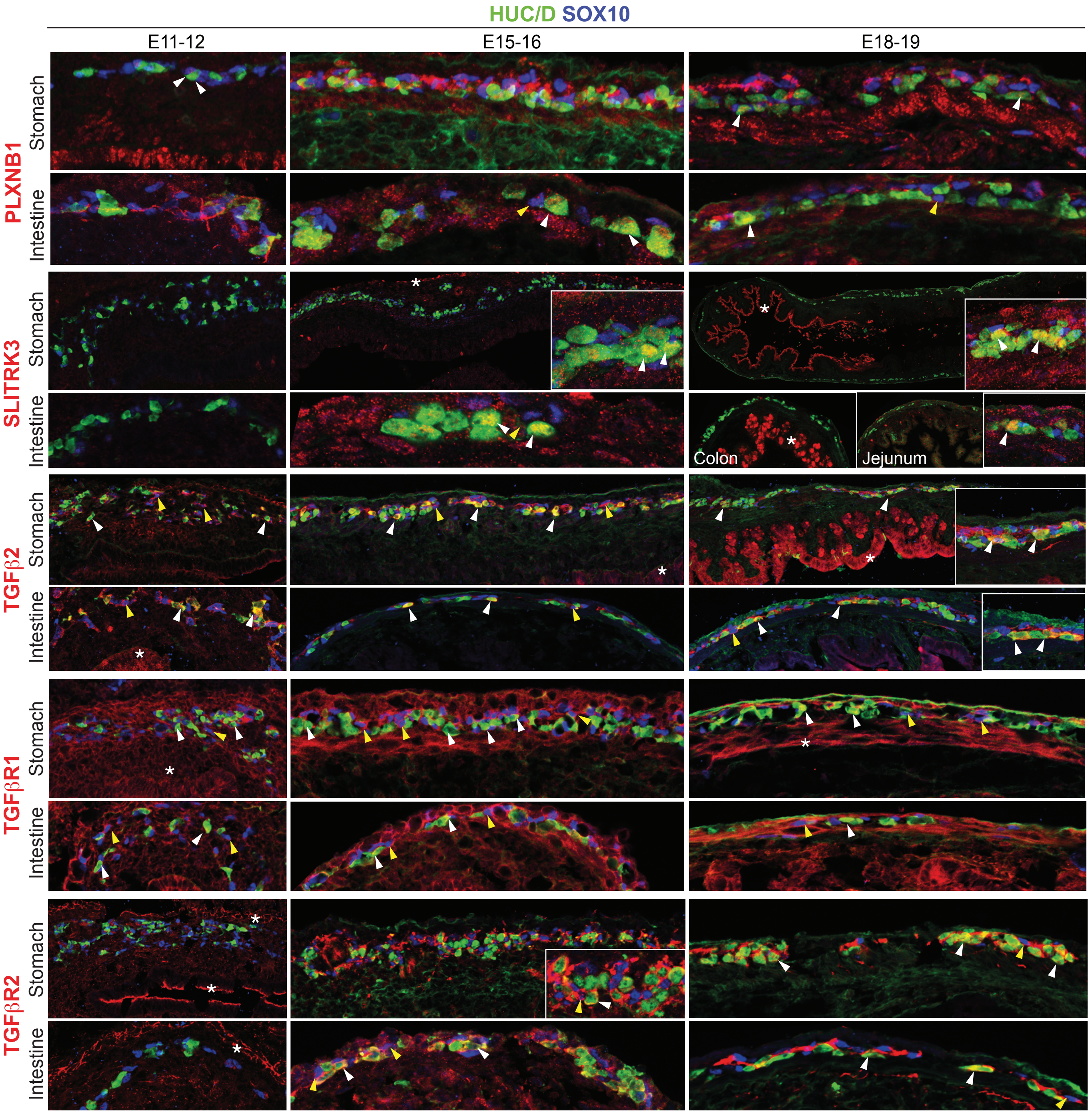

Supplementary Figure 6: IHC analysis of cell-cell communication components in the developing mouse bowel wall. Co-expression analysis of ligands and receptors with the neuronal marker HUC/D (white arrowheads) and the progenitor marker SOX10 (yellow arrowheads) at E11-12, E15-16 and E18-19 in stomach and intestine of mouse embryos. White boxes show higher magnification. *: expression outside the ENS (summarised also in Figure 6). CXCR4 show regional-specific subcellular distribution in the stomach at E18.5, which is indicated in box 1 (membrane) and 2 (vesicular).
