# Supplementary material for: Transcription and Signaling Regulators in Developing Neuronal Subtypes of Mouse and Human Enteric Nervous System
Source: Gastroenterology. 2018 Feb;154(3):624–36. doi: 10.1053/j.gastro.2017.10.005 (PMC6381388; doi:10.1053/j.gastro.2017.10.005)
Supplement: Supplementary Figure 7 [file mmc9.pdf]

SUPPLEMENTARY FIGURE 7

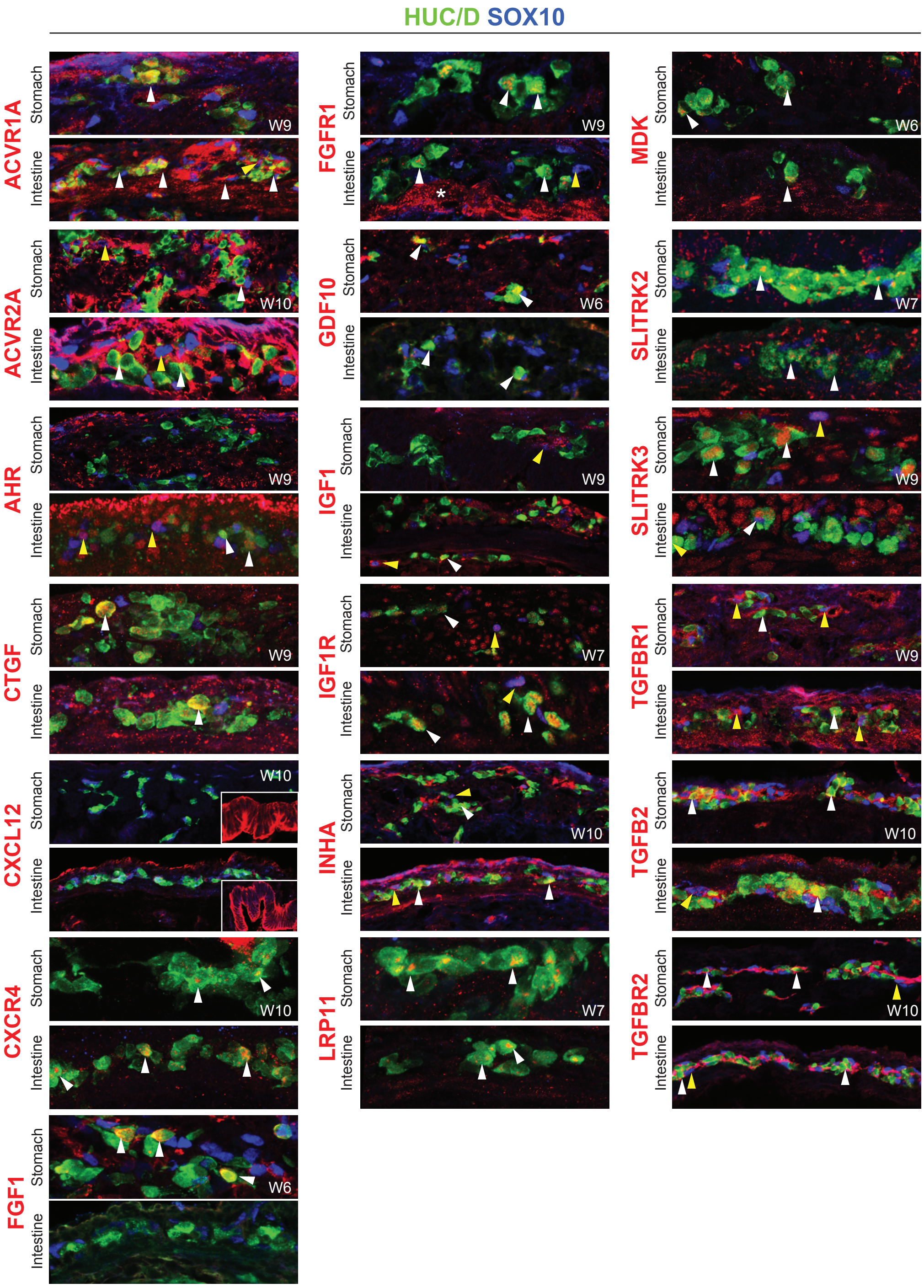

Supplementary Figure 7: IHC analysis of cell-cell communication components in the developing human ENS.
Co-expression analysis of ligands and receptors with the neuronal marker HUC/D (white arrowheads) and the progenitor marker SOX10 (yellow arrowheads) at W6-10 in stomach and intestine of human embryos. Insets show mucosal expression.
