# Supplementary material for: Transcription and Signaling Regulators in Developing Neuronal Subtypes of Mouse and Human Enteric Nervous System
Source: Gastroenterology. 2018 Feb;154(3):624–36. doi: 10.1053/j.gastro.2017.10.005 (PMC6381388; doi:10.1053/j.gastro.2017.10.005)
Supplement: Supplementary Figure 8 [file mmc10.pdf]

SUPPLEMENTARY FIGURE 8

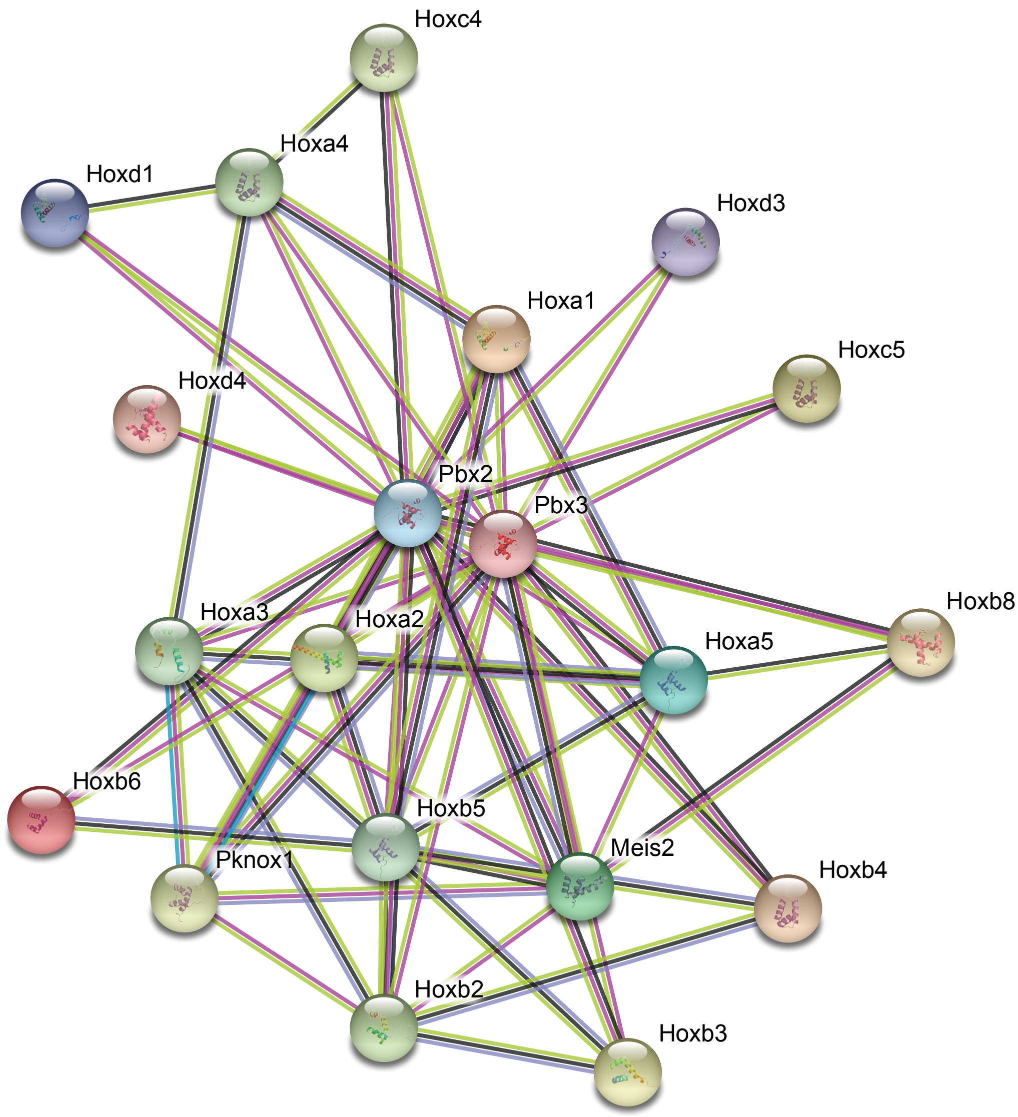

Supplementary Figure 8. String interaction scheme of HOX and TALE-proteins. Identified HOX and TALE proteins (20 genes) analysed for validated interactions using "Search tool for the retrieval of interacting genes/proteins" (STRING; string-db.org). Interaction code: blue:from curated databases; pink: experimentally deterined; black: co-expressed; light green: textmining; light blue: homology.
