# Supplementary material for: Transcription and Signaling Regulators in Developing Neuronal Subtypes of Mouse and Human Enteric Nervous System
Source: Gastroenterology. 2018 Feb;154(3):624–36. doi: 10.1053/j.gastro.2017.10.005 (PMC6381388; doi:10.1053/j.gastro.2017.10.005)
Supplement: Supplementary Table 1 [file mmc11.pdf]

Supplementary Table 1: Antibodies

Antigen	Host	Dilution	Source
**5-HT**	Rabbit	1:2000	Sigma S5545
**ActivinR1A**	Rabbit	1:500	Abcam ab60157
**ActivinR2A**	Rabbit	1:1000	Abcam ab96793
**AHR**	Rabbit	1:300	Abcam ab84833
**ALX1**	Rabbit	1:100; AR	Sigma HPA018905
**Calbindin**	Rabbit	1:1000	Chemicon AB1778
**Calbindin**	Goat	1:500	R&D AF3320
**CGRP**	Rabbit	1:5000	Sigma C8198
**CGRP**	Goat	1:1500	AdBsero 1720-9007
**ChAT**	Goat	1:300; AR	Millipore AB144P
**CTGF**	Rabbit	1:400	Abcam ab6992
**CUX1**	Rabbit	1:200; AR	Santa Cruz sc-13024
**CXCR4**	Rabbit	1:500	Abcam ab124824
**CXCL12**	Mouse	1:100	R&D 79018
**EBF1**	Rabbit	1:2000	Nordic Bio LSB6814
**EBF1-3**	Rabbit	1:500	Santa Cruz (H-300) sc-33552
**ETV1**	Rabbit	1:250	Kind gift from S. Arber[1]
**FGF1**	Rabbit	1:2000	Abcam ab9588
**FGFR1**	Rabbit	1:1000	Abcam ab10646
**FOXD1**	Goat	1:100	Nordic Bio LSB6453
**GDF10**	Rabbit	1:50	Atlas ab HPA015498
**HDX**	Rabbit	1:150; AR	Atlas ab HPA047189
**HMX1**	Rabbit	1:2000	Kind gift from E. Turner [2]
**HOXA3**	Rabbit	1:500	Kind gift from T.M. Jessell [3]
**HOXB3**	Rabbit	1:500	Santa Cruz (H50) sc-28606
**HOXC4**	Rabbit	1:500	Kind gift from T.M. Jessell [3]
**HOXC5**	Rabbit	1:1000	Kind gift from T.M. Jessell [4]
**HuC/D**	Mouse	1:300	Mol Probes A21271
**IGF1**	Goat	1:1000	Sigma SAB2501424
**IGF1R**	Rabbit	1:200	Abcam 131476; human
**INHA**	Mouse	1:1000	Thermo Fisher MA5-15703
**INHBA**	Rabbit	1:500	Atlas ab HPA020031
**ISL1/2**	Guinea Pig	1:10000	Kind gift from T.M. Jessell [5]
**KLF7**	Rabbit	1:250; AR	Novus Bio NBP1-80638
**LRP11**	Rabbit	1:1000	Abcam ab155310
**MEF2C**	Rabbit	1:400	Cell Signaling 5030XP
**MEIS2**	Rabbit	1:500; AR	Atlas ab HPA003256
**Midkine**	Rabbit	1:200	Abcam ab36038
**NEUROD4**	Rabbit	1:300; AR	Atlas ab HPA026998
**NKX6.1**	Mouse	1:4	DSHB by O.D Madsen
**nNOS**	Rabbit	1:500	Santa Cruz sc-648
**nNOS**	Goat	1:1000	Abcam ab1376
**NPY**	Rabbit	1:3000	DiaSorin
**NPY**	Sheep	1:2000	Abcam ab6173

**OC2**	Rabbit	1:250	Atlas ab HPA057058
**PAX3**	Mouse	1:100	DSHB by C.P. Ordahl
**PBX3**	Rabbit	1:500	Santa Cruz sc-891
**Plexin B1**	Rabbit	1:200	Abcam ab90087
**SATB1**	Goat	1:500, AR	Santa Cruz (E15) sc-5990
**SATB2**	Mouse	1:500	Santa Cruz sc-81376
**SLITRK2**	Rabbit	1:100	Abcam ab67305, human
**SLITRK3**	Rabbit	1:5000	Abcam ab67306
**SOX2**	Rabbit	1:5000	Seven Hills WRAB-1236
**SOX4**	Rabbit	1:150	Atlas ab HPA029901
**SOX5**	Rabbit	1:1000	Kind gift from J. Muhr
**SOX6**	Guinea Pig	1:1000	Kind gift from M. Wegner [6]
**SOX6**	Rabbit	1:300	Atlas ab HPA001923; human
**SOX9**	Goat	1:500, AR	R&D AF3075
**SOX10**	Goat	1:200	Santa Cruz (N20) sc-17342
**SOX10**	Guinea Pig	1:500	Kind gift from M. Wegner[7]
**SOX11**	Rabbit	1:1500	Abcam ab134107
**TBX3**	Goat	1:300	R&D AF4509
**TGFβ2**	Rabbit	1:500	Santa Cruz sc-90
**TGFβR1**	Rabbit	1:500	Santa Cruz sc398 (v22)
**TGFβR2**	Rabbit	1:500	Abcam ab186838
**TLX3**	Guinea pig	1:5000; AR	Kind gift from T. Müller and C. Birchmeier
**TLX3**	Rabbit	1:5000; AR	Kind gift from T. Müller and C. Birchmeier
**TH**	Rabbit	1:500	Pelfreeze P40101-0
**TH**	Sheep	1:300	Novus Bio NB300-110
**TH**	Chicken	1:500	Abcam ab76442
**TSHZ3**	Rabbit	1.200; AR	Sigma HPA008834
**VIP**	Rabbit	1:2500	Abcam ab43841
**ZEB1**	Rabbit	1:400; AR	Santa Cruz sc-25388
**ZFHX4**	Rabbit	1:100; AR	Sigma HPA023837
**Secondary Antibodies**	**Recognition of**	**Dilution**	**Source**
**Alexa 555**	Rabbit, Mouse, Guinea pig, Goat	1:1000	Molecular Probes
**Alexa 555**	Mouse IgG1& IgG2a	1:1000	Molecular Probes
**Alexa 488**	Mouse IgG1 & IgG2b, Goat, Rabbit, Chicken, Sheep	1:400	Molecular Probes
**Alexa 647**	Goat, Rabbit, Mouse	1:200	Molecular Probes

**Cy5**	Goat, Guinea pig	1:200	Jackson ImmunoResearch
**Cy3**	Guinea pig	1:1000	Jackson ImmunoResearch

AR: antigen retrieval

REFERENCES:

1. Arber S, Ladle DR, Lin JH, et al. ETS gene Er81 controls the formation of functional connections between group Ia sensory afferents and motor neurons. Cell 2000;101:485-98.
2. Quina LA, Tempest L, Hsu YW, et al. Hmx1 is required for the normal development of somatosensory neurons in the geniculate ganglion. Dev Biol 2012;365:152-63.
3. Dasen JS, Tice BC, Brenner-Morton S, et al. A Hox regulatory network establishes motor neuron pool identity and target-muscle connectivity. Cell 2005;123:477-91.
4. Liu JP, Laufer E, Jessell TM. Assigning the positional identity of spinal motor neurons: rostrocaudal patterning of Hox-c expression by FGFs, Gdf11, and retinoids. Neuron 2001;32:997-1012.
5. Tanabe Y, William C, Jessell TM. Specification of motor neuron identity by the MNR2 homeodomain protein. Cell 1998;95:67-80.
6. Stolt CC, Schlierf A, Lommes P, et al. SoxD proteins influence multiple stages of oligodendrocyte development and modulate SoxE protein function. Dev Cell 2006;11:697-709.
7. Maka M, Stolt CC, Wegner M. Identification of Sox8 as a modifier gene in a mouse model of Hirschsprung disease reveals underlying molecular defect. Dev Biol 2005;277:155-69.
