# Supplementary material for: Transcription and Signaling Regulators in Developing Neuronal Subtypes of Mouse and Human Enteric Nervous System
Source: Gastroenterology. 2018 Feb;154(3):624–36. doi: 10.1053/j.gastro.2017.10.005 (PMC6381388; doi:10.1053/j.gastro.2017.10.005)
Supplement: Supplementary Table 2 [file mmc12.pdf]

Supplementary Table 2: Enriched genes in pairwise comparisons between cell populations in the developing gut.
Significant genes based on p-value <0.05 and 5% FDR (false discovery rate).

W11 vs C11

Probeset ID	Gene Symbol	RefSeq	p-value	Fold-change	Comparison
10593756	Chrna3	NM_145129	5.55267e-017	27.9817	E11.5 * Wnt1 up vs E11.5 * Ctrl
10357103	Cdh19	NM_001081386	1.478e-012	26.3844	E11.5 * Wnt1 up vs E11.5 * Ctrl
10547227	Ret	NM_001080780	1.3369e-015	21.7731	E11.5 * Wnt1 up vs E11.5 * Ctrl
10515095	Elavl4	NM_010488	3.13616e-015	18.2435	E11.5 * Wnt1 up vs E11.5 * Ctrl
10576332	Tubb3	NM_023279	9.43309e-012	17.4451	E11.5 * Wnt1 up vs E11.5 * Ctrl
10536667	Ptprz1	NM_001081306	3.3449e-015	16.5099	E11.5 * Wnt1 up vs E11.5 * Ctrl
10492169	Mab21l1	NM_010750	1.04657e-014	14.7884	E11.5 * Wnt1 up vs E11.5 * Ctrl
10470412	Dbh	NM_138942	3.27595e-014	14.6623	E11.5 * Wnt1 up vs E11.5 * Ctrl
10427862	Cdh6	NM_007666	5.92824e-016	13.9498	E11.5 * Wnt1 up vs E11.5 * Ctrl
10595033	Scg3	NM_009130	2.43695e-015	13.4743	E11.5 * Wnt1 up vs E11.5 * Ctrl
10431812	Nell2	NM_016743	8.29229e-015	13.3545	E11.5 * Wnt1 up vs E11.5 * Ctrl
10373467	Erbb3	NM_010153	8.6074e-013	13.069	E11.5 * Wnt1 up vs E11.5 * Ctrl
10345016	Tcfap2b	NM_001025305	4.44058e-013	11.5813	E11.5 * Wnt1 up vs E11.5 * Ctrl
10417628	Cadps	NM_012061	7.86276e-013	11.2763	E11.5 * Wnt1 up vs E11.5 * Ctrl
10523231	Art3	NM_181728	1.541e-014	10.9224	E11.5 * Wnt1 up vs E11.5 * Ctrl
10463737	Ina	AK144917	3.60861e-011	10.5517	E11.5 * Wnt1 up vs E11.5 * Ctrl
10431659	Kif21a	NM_001109040	2.70076e-015	10.425	E11.5 * Wnt1 up vs E11.5 * Ctrl
10522895	Csn3	NM_007786	1.44314e-011	10.1087	E11.5 * Wnt1 up vs E11.5 * Ctrl
10464471	Gal	NM_010253	3.05005e-010	10.0649	E11.5 * Wnt1 up vs E11.5 * Ctrl
10605113	L1cam	NM_008478	4.68031e-014	10.0402	E11.5 * Wnt1 up vs E11.5 * Ctrl
10543802	Plxna4	NM_175750	2.31288e-014	9.95	E11.5 * Wnt1 up vs E11.5 * Ctrl
10432675	I730030J21Rik	ENSMUST00000(	1.07583e-008	9.37961	E11.5 * Wnt1 up vs E11.5 * Ctrl
10408798	Tcfap2a	NM_011547	3.40865e-013	9.34378	E11.5 * Wnt1 up vs E11.5 * Ctrl
10499431	Syt11	NM_018804	1.39618e-013	8.34575	E11.5 * Wnt1 up vs E11.5 * Ctrl
10428698	Sntb1	NM_016667	1.0835e-013	8.19512	E11.5 * Wnt1 up vs E11.5 * Ctrl
10490818	Stmn2	NM_025285	9.55378e-013	8.00188	E11.5 * Wnt1 up vs E11.5 * Ctrl
10593767	Chrnb4	NM_148944	4.29952e-011	7.47835	E11.5 * Wnt1 up vs E11.5 * Ctrl
10575693	Vat1l	NM_173016	2.06623e-012	7.46626	E11.5 * Wnt1 up vs E11.5 * Ctrl
10464370	Slc18a2	NM_172523	6.44462e-013	7.34596	E11.5 * Wnt1 up vs E11.5 * Ctrl
10601888	Plp1	NM_011123	6.00784e-011	7.19343	E11.5 * Wnt1 up vs E11.5 * Ctrl
10597817	Cck	NM_031161	2.68205e-009	7.1758	E11.5 * Wnt1 up vs E11.5 * Ctrl
10402020	Eml5	NM_001081191	2.01826e-014	7.17219	E11.5 * Wnt1 up vs E11.5 * Ctrl
10531869	Mapk10	NM_009158	1.31669e-012	7.17041	E11.5 * Wnt1 up vs E11.5 * Ctrl
10372324	Syt1	NM_009306	5.0579e-011	7.02094	E11.5 * Wnt1 up vs E11.5 * Ctrl
10463732	Ina	NM_146100	1.42873e-014	7.00993	E11.5 * Wnt1 up vs E11.5 * Ctrl
10363224	Fabp7	NM_021272	2.20508e-012	6.99935	E11.5 * Wnt1 up vs E11.5 * Ctrl
10423243	Cdh10	NM_009865	9.1247e-011	6.96576	E11.5 * Wnt1 up vs E11.5 * Ctrl
10367982	Gpr126	NM_001002268	9.1402e-013	6.83879	E11.5 * Wnt1 up vs E11.5 * Ctrl
10422164	Ednrb	NM_007904	5.20127e-015	6.72604	E11.5 * Wnt1 up vs E11.5 * Ctrl
10540298	Chl1	NM_007697	6.05236e-013	6.60729	E11.5 * Wnt1 up vs E11.5 * Ctrl
10491477	Sox2	NM_011443	2.23254e-009	6.53277	E11.5 * Wnt1 up vs E11.5 * Ctrl
10439514	Gap43	NM_008083	5.08332e-014	6.51269	E11.5 * Wnt1 up vs E11.5 * Ctrl
10586591	Car12	NM_178396	4.50845e-012	6.40402	E11.5 * Wnt1 up vs E11.5 * Ctrl
10530306	Phox2b	NM_008888	8.2495e-012	6.39947	E11.5 * Wnt1 up vs E11.5 * Ctrl
10585484	Chrna5	NM_176844	3.13304e-011	6.22389	E11.5 * Wnt1 up vs E11.5 * Ctrl
10368175	Pde7b	NM_013875	2.02444e-011	6.21949	E11.5 * Wnt1 up vs E11.5 * Ctrl
10353192	Eya1	NM_010164	2.24493e-011	6.16516	E11.5 * Wnt1 up vs E11.5 * Ctrl
10458663	Dpysl3	NM_009468	1.85662e-013	6.08176	E11.5 * Wnt1 up vs E11.5 * Ctrl
10423836	Cthrc1	NM_026778	4.31932e-014	6.06502	E11.5 * Wnt1 up vs E11.5 * Ctrl
10514510	Cyp2j6	NM_010008	4.05356e-012	6.01521	E11.5 * Wnt1 up vs E11.5 * Ctrl
10508052	Grik3	NM_001081097	2.20636e-010	5.89643	E11.5 * Wnt1 up vs E11.5 * Ctrl
10416340	Gfra2	NM_008115	2.23752e-012	5.72119	E11.5 * Wnt1 up vs E11.5 * Ctrl
10605437	Pls3	NM_145629	3.43879e-013	5.67028	E11.5 * Wnt1 up vs E11.5 * Ctrl
10395553	Nrcam	NM_176930	3.40568e-012	5.55752	E11.5 * Wnt1 up vs E11.5 * Ctrl
10408629	1300014I06Rik	NM_025831	1.89085e-011	5.55653	E11.5 * Wnt1 up vs E11.5 * Ctrl
10494043	Tdrkh	NM_028307	2.2442e-012	5.50841	E11.5 * Wnt1 up vs E11.5 * Ctrl
10362372	9330159F19Rik	BC138282	2.52885e-010	5.43481	E11.5 * Wnt1 up vs E11.5 * Ctrl
10584549	Scn3b	NM_178227	2.31646e-011	5.41251	E11.5 * Wnt1 up vs E11.5 * Ctrl
10368999	Grik2	NM_010349	9.8945e-012	5.39765	E11.5 * Wnt1 up vs E11.5 * Ctrl

10491136	Tnik	BC137799	1.40255e-015	5.38032	E11.5 * Wnt1 up vs E11.5 * Ctrl
10400926	Rtn1	NM_153457	5.51564e-011	5.18128	E11.5 * Wnt1 up vs E11.5 * Ctrl
10603151	Gpm6b	NM_023122	6.43349e-014	5.0995	E11.5 * Wnt1 up vs E11.5 * Ctrl
10472809	Dlx1	NM_010053	2.10539e-012	5.09389	E11.5 * Wnt1 up vs E11.5 * Ctrl
10569646	Ccnd1	NM_007631	3.88396e-013	5.08293	E11.5 * Wnt1 up vs E11.5 * Ctrl
10498885	Gria2	NM_013540	4.02994e-010	5.0327	E11.5 * Wnt1 up vs E11.5 * Ctrl
10522208	Uchl1	NM_011670	1.69016e-009	5.0191	E11.5 * Wnt1 up vs E11.5 * Ctrl
10402708	Ckb	NM_021273	7.8438e-011	4.99711	E11.5 * Wnt1 up vs E11.5 * Ctrl
10476512	Snap25	NM_011428	6.36015e-012	4.98664	E11.5 * Wnt1 up vs E11.5 * Ctrl
10346882	Adam23	NM_011780	3.76113e-011	4.96819	E11.5 * Wnt1 up vs E11.5 * Ctrl
10492136	Dclk1	NM_019978	3.61592e-013	4.94534	E11.5 * Wnt1 up vs E11.5 * Ctrl
10599880	Slitrk2	NM_198863	3.03626e-011	4.94126	E11.5 * Wnt1 up vs E11.5 * Ctrl
10603896	Klhl13	NM_026167	1.10247e-013	4.84176	E11.5 * Wnt1 up vs E11.5 * Ctrl
10571865	Scrg1	NM_009136	2.22772e-009	4.76097	E11.5 * Wnt1 up vs E11.5 * Ctrl
10599884		---	3.2008e-005	4.6645	E11.5 * Wnt1 up vs E11.5 * Ctrl
10554900	Dlg2	NM_011807	4.07211e-012	4.66011	E11.5 * Wnt1 up vs E11.5 * Ctrl
10543058	Dlx5	NM_010056	5.47266e-008	4.60176	E11.5 * Wnt1 up vs E11.5 * Ctrl
10485955	Scg5	NM_009162	3.17085e-012	4.56607	E11.5 * Wnt1 up vs E11.5 * Ctrl
10351400	Fam78b	NM_175461	2.80275e-011	4.51303	E11.5 * Wnt1 up vs E11.5 * Ctrl
10354529	1700019D03Rik	NM_144953	2.40686e-009	4.44768	E11.5 * Wnt1 up vs E11.5 * Ctrl
10522388	Slc10a4	NM_173403	3.87104e-010	4.33864	E11.5 * Wnt1 up vs E11.5 * Ctrl
10359113	Fam163a	NM_177838	7.0512e-009	4.32935	E11.5 * Wnt1 up vs E11.5 * Ctrl
10529903	Fam184b	NM_021416	1.26761e-009	4.29196	E11.5 * Wnt1 up vs E11.5 * Ctrl
10562223	Fxyd3	NM_008557	6.60743e-012	4.28844	E11.5 * Wnt1 up vs E11.5 * Ctrl
10483803	6720416L17Rik	ENSMUST00000	1.72183e-010	4.25208	E11.5 * Wnt1 up vs E11.5 * Ctrl
10514520	Cyp2j9	NM_028979	4.02258e-010	4.23413	E11.5 * Wnt1 up vs E11.5 * Ctrl
10533055	1500001A10Rik	NM_026886	6.51542e-013	4.21958	E11.5 * Wnt1 up vs E11.5 * Ctrl
10473125	Itga4	NM_010576	2.65603e-009	4.20432	E11.5 * Wnt1 up vs E11.5 * Ctrl
10468452	Sorcs1	NM_021377	1.85888e-012	4.10891	E11.5 * Wnt1 up vs E11.5 * Ctrl
10458052	Epb4.1l4a	NM_013512	4.94244e-012	4.08825	E11.5 * Wnt1 up vs E11.5 * Ctrl
10598626	Tspan7	NM_019634	9.70718e-012	4.08326	E11.5 * Wnt1 up vs E11.5 * Ctrl
10474096	Lrrc4c	NM_178725	1.21754e-011	4.03544	E11.5 * Wnt1 up vs E11.5 * Ctrl
10584259	Fez1	NM_183171	3.24499e-013	3.99424	E11.5 * Wnt1 up vs E11.5 * Ctrl
10505998	Fggy	NM_001113412	5.69399e-007	3.99178	E11.5 * Wnt1 up vs E11.5 * Ctrl
10492165		---	4.62182e-007	3.95833	E11.5 * Wnt1 up vs E11.5 * Ctrl
10505994	Fggy	NM_001113412	8.01976e-006	3.94861	E11.5 * Wnt1 up vs E11.5 * Ctrl
10371578	Ascl1	NM_008553	8.61029e-007	3.93953	E11.5 * Wnt1 up vs E11.5 * Ctrl
10471994	Kif5c	NM_008449	1.67602e-010	3.92982	E11.5 * Wnt1 up vs E11.5 * Ctrl
10465820	Gng3	NM_010316	3.73254e-009	3.92909	E11.5 * Wnt1 up vs E11.5 * Ctrl
10520950	Pdlim1	NM_016861	4.31168e-010	3.89099	E11.5 * Wnt1 up vs E11.5 * Ctrl
10467420	Pdlim1	NM_016861	7.73648e-010	3.85876	E11.5 * Wnt1 up vs E11.5 * Ctrl
10568107		---	8.20629e-006	3.80555	E11.5 * Wnt1 up vs E11.5 * Ctrl
10535956	Stard13	NM_146258	1.14136e-008	3.80342	E11.5 * Wnt1 up vs E11.5 * Ctrl
10440258	Epha3	NM_010140	6.16025e-014	3.7881	E11.5 * Wnt1 up vs E11.5 * Ctrl
10362003	F730021E23Rik	AK155606	3.81831e-006	3.69626	E11.5 * Wnt1 up vs E11.5 * Ctrl
10502510	Lmo4	NM_010723	1.23824e-010	3.69138	E11.5 * Wnt1 up vs E11.5 * Ctrl
10549222	Bcat1	NM_001024468	9.33499e-010	3.68496	E11.5 * Wnt1 up vs E11.5 * Ctrl
10366163	Slc6a15	NM_175328	1.49213e-008	3.68259	E11.5 * Wnt1 up vs E11.5 * Ctrl
10362363	6330407J23Rik	NM_026138	1.2479e-010	3.67398	E11.5 * Wnt1 up vs E11.5 * Ctrl
10503218	Chd7	NM_001081417	1.35275e-006	3.66735	E11.5 * Wnt1 up vs E11.5 * Ctrl
10564163	Snord116	NR_002895	1.05174e-005	3.65318	E11.5 * Wnt1 up vs E11.5 * Ctrl
10564167	Snord116	NR_002895	1.05174e-005	3.65318	E11.5 * Wnt1 up vs E11.5 * Ctrl
10564171	Snord116	NR_002895	1.05174e-005	3.65318	E11.5 * Wnt1 up vs E11.5 * Ctrl
10564173	Snord116	NR_002895	1.05174e-005	3.65318	E11.5 * Wnt1 up vs E11.5 * Ctrl
10564175	Snord116	NR_002895	1.05174e-005	3.65318	E11.5 * Wnt1 up vs E11.5 * Ctrl
10564179	Snord116	NR_002895	1.05174e-005	3.65318	E11.5 * Wnt1 up vs E11.5 * Ctrl
10564181	Snord116	NR_002895	1.05174e-005	3.65318	E11.5 * Wnt1 up vs E11.5 * Ctrl
10564185	Snord116	NR_002895	1.05174e-005	3.65318	E11.5 * Wnt1 up vs E11.5 * Ctrl
10564187	Snord116	NR_002895	1.05174e-005	3.65318	E11.5 * Wnt1 up vs E11.5 * Ctrl
10564189	Snord116	NR_002895	1.05174e-005	3.65318	E11.5 * Wnt1 up vs E11.5 * Ctrl
10564191	Snord116	NR_002895	1.05174e-005	3.65318	E11.5 * Wnt1 up vs E11.5 * Ctrl
10564193	Snord116	NR_002895	1.05174e-005	3.65318	E11.5 * Wnt1 up vs E11.5 * Ctrl
10564195	Snord116	NR_002895	1.05174e-005	3.65318	E11.5 * Wnt1 up vs E11.5 * Ctrl
10564197	Snord116	NR_002895	1.05174e-005	3.65318	E11.5 * Wnt1 up vs E11.5 * Ctrl
10564199	Snord116	NR_002895	1.05174e-005	3.65318	E11.5 * Wnt1 up vs E11.5 * Ctrl
10564201	Snord116	AF241256	1.05174e-005	3.65318	E11.5 * Wnt1 up vs E11.5 * Ctrl

10564205	Snord116	AF241256	1.05174e-005	3.65318	E11.5 * Wnt1 up vs E11.5 * Ctrl
10564207	Snord116	AF241256	1.05174e-005	3.65318	E11.5 * Wnt1 up vs E11.5 * Ctrl
10406461	C130071C03Rik	ENSMUST00000(	1.58062e-008	3.64034	E11.5 * Wnt1 up vs E11.5 * Ctrl
10428619	Enpp2	NM_015744	1.15831e-009	3.63756	E11.5 * Wnt1 up vs E11.5 * Ctrl
10501924	Ndst3	NM_031186	6.69149e-010	3.63028	E11.5 * Wnt1 up vs E11.5 * Ctrl
10457963	Gpr17	NM_001025381	1.39318e-008	3.60987	E11.5 * Wnt1 up vs E11.5 * Ctrl
10534960	Gje1	NM_080450	2.43184e-007	3.58489	E11.5 * Wnt1 up vs E11.5 * Ctrl
10503214	Chd7	NM_001081417	1.38007e-009	3.56639	E11.5 * Wnt1 up vs E11.5 * Ctrl
10507840	Heyl	NM_013905	6.47043e-008	3.54791	E11.5 * Wnt1 up vs E11.5 * Ctrl
10564161	Snord116	NR_002895	1.03894e-005	3.53897	E11.5 * Wnt1 up vs E11.5 * Ctrl
10381154	Cnp	NM_009923	1.65758e-010	3.5367	E11.5 * Wnt1 up vs E11.5 * Ctrl
10411527	Cartpt	NM_013732	4.16646e-006	3.51589	E11.5 * Wnt1 up vs E11.5 * Ctrl
10556509	Spon1	NM_145584	3.16837e-012	3.49017	E11.5 * Wnt1 up vs E11.5 * Ctrl
10447056	Qpct	NM_027455	6.63397e-010	3.48957	E11.5 * Wnt1 up vs E11.5 * Ctrl
10373407	Mbc2	NM_011843	6.10435e-009	3.44728	E11.5 * Wnt1 up vs E11.5 * Ctrl
10363541	Ass1	NM_007494	2.19804e-008	3.41927	E11.5 * Wnt1 up vs E11.5 * Ctrl
10492640	Fstl5	NM_178673	4.18971e-009	3.41381	E11.5 * Wnt1 up vs E11.5 * Ctrl
10553537	Luzp2	NM_178705	1.65784e-007	3.4112	E11.5 * Wnt1 up vs E11.5 * Ctrl
10564177	Snord116	AF241256	1.52834e-005	3.39238	E11.5 * Wnt1 up vs E11.5 * Ctrl
10503234	Rlbp1l1	NM_028940	5.67685e-012	3.38405	E11.5 * Wnt1 up vs E11.5 * Ctrl
10413419	Arhgef3	NM_027871	3.30963e-010	3.37175	E11.5 * Wnt1 up vs E11.5 * Ctrl
10492341	4631416L12Rik	NM_001081295	5.06174e-009	3.30314	E11.5 * Wnt1 up vs E11.5 * Ctrl
10479698	Myt1	NM_008665	7.29682e-009	3.30266	E11.5 * Wnt1 up vs E11.5 * Ctrl
10411519	Mtap1b	NM_008634	6.95782e-011	3.30258	E11.5 * Wnt1 up vs E11.5 * Ctrl
10468722	Gfra1	NM_010279	3.29396e-010	3.24895	E11.5 * Wnt1 up vs E11.5 * Ctrl
10471945	Zeb2	NM_015753	3.14418e-009	3.24799	E11.5 * Wnt1 up vs E11.5 * Ctrl
10350733	Rgs16	NM_011267	7.17857e-007	3.24569	E11.5 * Wnt1 up vs E11.5 * Ctrl
10416090	Stmn4	NM_019675	5.31879e-008	3.24355	E11.5 * Wnt1 up vs E11.5 * Ctrl
10604751	Fgf13	NM_010200	1.85158e-007	3.22698	E11.5 * Wnt1 up vs E11.5 * Ctrl
10514352	Elavl2	NM_207685	2.307e-009	3.21067	E11.5 * Wnt1 up vs E11.5 * Ctrl
10465943	Dagla	NM_198114	3.5692e-007	3.20943	E11.5 * Wnt1 up vs E11.5 * Ctrl
10548030	Cd9	NM_007657	1.21836e-008	3.19201	E11.5 * Wnt1 up vs E11.5 * Ctrl
10388430	Serpinf1	NM_011340	2.60328e-012	3.17943	E11.5 * Wnt1 up vs E11.5 * Ctrl
10362538	Lama4	NM_010681	1.74267e-013	3.17633	E11.5 * Wnt1 up vs E11.5 * Ctrl
10439651	Cd200	NM_010818	5.69644e-009	3.17305	E11.5 * Wnt1 up vs E11.5 * Ctrl
10357736	Nfasc	NM_182716	2.06462e-008	3.17218	E11.5 * Wnt1 up vs E11.5 * Ctrl
10418835	Slc18a3	NM_021712	8.33535e-007	3.13649	E11.5 * Wnt1 up vs E11.5 * Ctrl
10452766		---	0.00241752	3.1272	E11.5 * Wnt1 up vs E11.5 * Ctrl
10485198	Tspan18	NM_183180	5.08186e-010	3.11657	E11.5 * Wnt1 up vs E11.5 * Ctrl
10440534	Adamts5	NM_011782	9.20731e-008	3.11541	E11.5 * Wnt1 up vs E11.5 * Ctrl
10344679	St18	NM_173868	2.79625e-009	3.10127	E11.5 * Wnt1 up vs E11.5 * Ctrl
10471154	Ass1	NM_007494	1.09103e-007	3.10121	E11.5 * Wnt1 up vs E11.5 * Ctrl
10359908	Rgs4	NM_009062	7.30812e-008	3.07917	E11.5 * Wnt1 up vs E11.5 * Ctrl
10564183	Snord116	AF241256	2.54107e-005	3.0676	E11.5 * Wnt1 up vs E11.5 * Ctrl
10346150	Tmeff2	NM_019790	2.00188e-011	3.05115	E11.5 * Wnt1 up vs E11.5 * Ctrl
10487139	Shc4	NM_199022	2.44398e-011	3.05113	E11.5 * Wnt1 up vs E11.5 * Ctrl
10564159	Snord116	AF241256	3.06453e-005	3.04541	E11.5 * Wnt1 up vs E11.5 * Ctrl
10435733	Igsf11	NM_170599	2.27651e-009	3.03287	E11.5 * Wnt1 up vs E11.5 * Ctrl
10532753	Coro1c	NM_011779	1.33464e-009	3.03177	E11.5 * Wnt1 up vs E11.5 * Ctrl
10458534	Pcdh1	NM_029357	1.66877e-008	3.02755	E11.5 * Wnt1 up vs E11.5 * Ctrl
10505996	Fggy	NM_001113412	9.66708e-008	3.01445	E11.5 * Wnt1 up vs E11.5 * Ctrl
10362186	Moxd1	NM_021509	3.29139e-007	2.99873	E11.5 * Wnt1 up vs E11.5 * Ctrl
10482448	Zeb2	NM_015753	2.67738e-012	2.99606	E11.5 * Wnt1 up vs E11.5 * Ctrl
10518947	Ajap1	NM_001099299	1.9721e-009	2.993	E11.5 * Wnt1 up vs E11.5 * Ctrl
10503202	Chd7	NM_001081417	1.44607e-009	2.97831	E11.5 * Wnt1 up vs E11.5 * Ctrl
10525016	Tbx3	NM_011535	1.39399e-012	2.95064	E11.5 * Wnt1 up vs E11.5 * Ctrl
10344973	Gdap1	NM_010267	7.61697e-007	2.94362	E11.5 * Wnt1 up vs E11.5 * Ctrl
10485979	Gjd2	NM_010290	1.64245e-005	2.93681	E11.5 * Wnt1 up vs E11.5 * Ctrl
10404792	Phactr1	NM_198419	4.09375e-010	2.93334	E11.5 * Wnt1 up vs E11.5 * Ctrl
10455826	Megf10	NM_001001979	4.24431e-008	2.92897	E11.5 * Wnt1 up vs E11.5 * Ctrl
10503198	Chd7	NM_001081417	5.02305e-008	2.9056	E11.5 * Wnt1 up vs E11.5 * Ctrl
10395702	Akap6	NM_198111	3.02554e-011	2.89066	E11.5 * Wnt1 up vs E11.5 * Ctrl
10371332	Aldh1l2	NM_153543	1.11366e-009	2.88752	E11.5 * Wnt1 up vs E11.5 * Ctrl
10503194	Chd7	NM_001081417	6.13399e-007	2.8764	E11.5 * Wnt1 up vs E11.5 * Ctrl
10362596	Fyn	NM_001122893	7.62072e-017	2.85734	E11.5 * Wnt1 up vs E11.5 * Ctrl
10434845	Il1rap	NM_008364	1.06359e-010	2.85636	E11.5 * Wnt1 up vs E11.5 * Ctrl

10363146	Slc35f1	NM_178675	1.51847e-007	2.8559	E11.5 * Wnt1 up vs E11.5 * Ctrl
10350438	Kcnt2	NM_001081027	1.32626e-006	2.84774	E11.5 * Wnt1 up vs E11.5 * Ctrl
10564165	Snord116	AF241256	6.51493e-005	2.84336	E11.5 * Wnt1 up vs E11.5 * Ctrl
10498710	Bche	NM_009738	3.98374e-008	2.83902	E11.5 * Wnt1 up vs E11.5 * Ctrl
10594447	Map2k1	NM_008927	1.33453e-010	2.83821	E11.5 * Wnt1 up vs E11.5 * Ctrl
10422244	Slitrk6	NM_175499	1.37685e-008	2.82057	E11.5 * Wnt1 up vs E11.5 * Ctrl
10431697	Abcd2	NM_011994	7.06257e-007	2.8179	E11.5 * Wnt1 up vs E11.5 * Ctrl
10393379	Mxra7	NM_026280	1.17629e-009	2.8118	E11.5 * Wnt1 up vs E11.5 * Ctrl
10573979	Gnao1	NM_010308	1.17217e-010	2.80762	E11.5 * Wnt1 up vs E11.5 * Ctrl
10448967	Sox8	NM_011447	1.04303e-008	2.80232	E11.5 * Wnt1 up vs E11.5 * Ctrl
10588755	Camkv	NM_145621	1.22655e-007	2.79669	E11.5 * Wnt1 up vs E11.5 * Ctrl
10395414	Tmem195	NM_178767	2.37738e-006	2.7954	E11.5 * Wnt1 up vs E11.5 * Ctrl
10566723	Lmo1	NM_057173	7.94399e-011	2.78686	E11.5 * Wnt1 up vs E11.5 * Ctrl
10503200	Chd7	NM_001081417	5.05302e-008	2.77209	E11.5 * Wnt1 up vs E11.5 * Ctrl
10577190	Rasa3	NM_009025	2.20515e-009	2.75507	E11.5 * Wnt1 up vs E11.5 * Ctrl
10431017	Ttll1	NM_178869	7.65956e-011	2.75505	E11.5 * Wnt1 up vs E11.5 * Ctrl
10385391	Cyfip2	NM_133769	5.53023e-010	2.75199	E11.5 * Wnt1 up vs E11.5 * Ctrl
10417798	Kcnk5	NM_021542	1.59119e-007	2.75006	E11.5 * Wnt1 up vs E11.5 * Ctrl
10539310	Pcgf1	NM_197992	2.08977e-007	2.74869	E11.5 * Wnt1 up vs E11.5 * Ctrl
10594301	Coro2b	NM_175484	2.42447e-008	2.74706	E11.5 * Wnt1 up vs E11.5 * Ctrl
10503222	Chd7	NM_001081417	3.55165e-009	2.74097	E11.5 * Wnt1 up vs E11.5 * Ctrl
10366043	Dusp6	NM_026268	4.02725e-007	2.74047	E11.5 * Wnt1 up vs E11.5 * Ctrl
10585048	Cadm1	NM_207675	8.64761e-012	2.73501	E11.5 * Wnt1 up vs E11.5 * Ctrl
10439695	Tagln3	NM_019754	3.52756e-009	2.7348	E11.5 * Wnt1 up vs E11.5 * Ctrl
10499138	Dclk2	NM_027539	1.60456e-010	2.72725	E11.5 * Wnt1 up vs E11.5 * Ctrl
10358547	Hmcn1	NM_001024720	2.24024e-006	2.72232	E11.5 * Wnt1 up vs E11.5 * Ctrl
10494069	Tnrc4	NM_172434	1.7244e-005	2.71853	E11.5 * Wnt1 up vs E11.5 * Ctrl
10478374	Gdap1l1	NM_144891	3.79551e-009	2.71672	E11.5 * Wnt1 up vs E11.5 * Ctrl
10354506	Mfsd6	NM_133829	5.68404e-007	2.69921	E11.5 * Wnt1 up vs E11.5 * Ctrl
10568668	Adam12	NM_007400	1.42108e-008	2.67708	E11.5 * Wnt1 up vs E11.5 * Ctrl
10430458	Sox10	NM_011437	5.08671e-007	2.66898	E11.5 * Wnt1 up vs E11.5 * Ctrl
10416175	Nefl	NM_010910	2.1972e-008	2.66784	E11.5 * Wnt1 up vs E11.5 * Ctrl
10375313	Ccnjl	NM_001045530	8.93736e-009	2.66519	E11.5 * Wnt1 up vs E11.5 * Ctrl
10584674	Mcam	NM_023061	4.95701e-010	2.65847	E11.5 * Wnt1 up vs E11.5 * Ctrl
10503216	Chd7	NM_001081417	1.90056e-009	2.65665	E11.5 * Wnt1 up vs E11.5 * Ctrl
10462613	Ifit2	NM_008332	1.23001e-010	2.6486	E11.5 * Wnt1 up vs E11.5 * Ctrl
10538356	Chn2	NM_023543	8.2751e-009	2.63684	E11.5 * Wnt1 up vs E11.5 * Ctrl
10603289	Clcn5	NM_016691	5.13252e-012	2.63019	E11.5 * Wnt1 up vs E11.5 * Ctrl
10506433	Dab1	NM_177259	4.53409e-009	2.62571	E11.5 * Wnt1 up vs E11.5 * Ctrl
10424559	Khdrbs3	NM_010158	1.14497e-011	2.6241	E11.5 * Wnt1 up vs E11.5 * Ctrl
10472820	Itga6	NM_008397	4.95411e-009	2.61806	E11.5 * Wnt1 up vs E11.5 * Ctrl
10360418	Rgs7	NM_011880	7.71681e-007	2.61095	E11.5 * Wnt1 up vs E11.5 * Ctrl
10503212	Chd7	NM_001081417	1.65465e-006	2.60184	E11.5 * Wnt1 up vs E11.5 * Ctrl
10503166	Chd7	NM_001081417	2.88344e-006	2.5963	E11.5 * Wnt1 up vs E11.5 * Ctrl
10602805	Mtap7d2	NM_001081124	9.24453e-009	2.5829	E11.5 * Wnt1 up vs E11.5 * Ctrl
10521498	Crmp1	NM_007765	1.76652e-008	2.57038	E11.5 * Wnt1 up vs E11.5 * Ctrl
10396125	Atl1	NM_178628	2.7072e-007	2.56736	E11.5 * Wnt1 up vs E11.5 * Ctrl
10422537	Nalcn	NM_177393	4.98349e-009	2.56576	E11.5 * Wnt1 up vs E11.5 * Ctrl
10476628	Otor	NM_020595	1.53314e-007	2.55402	E11.5 * Wnt1 up vs E11.5 * Ctrl
10402061	Eml5	BC049855	1.29313e-006	2.55335	E11.5 * Wnt1 up vs E11.5 * Ctrl
10492021	Postn	NM_015784	2.83547e-009	2.55284	E11.5 * Wnt1 up vs E11.5 * Ctrl
10410547	Nkd2	NM_028186	2.29376e-007	2.54501	E11.5 * Wnt1 up vs E11.5 * Ctrl
10603051	Ap1s2	NM_026887	2.02144e-005	2.54308	E11.5 * Wnt1 up vs E11.5 * Ctrl
10408850	Nedd9	NM_001111324	1.28215e-010	2.53709	E11.5 * Wnt1 up vs E11.5 * Ctrl
10564169	Snord116	AF241256	0.000146323	2.53164	E11.5 * Wnt1 up vs E11.5 * Ctrl
10552075	Lgi4	NM_144556	3.12781e-008	2.52671	E11.5 * Wnt1 up vs E11.5 * Ctrl
10503220	Chd7	NM_001081417	6.45543e-007	2.52462	E11.5 * Wnt1 up vs E11.5 * Ctrl
10530499	EG545758	NM_001024147	4.45958e-008	2.52192	E11.5 * Wnt1 up vs E11.5 * Ctrl
10461057	Rcor2	NM_054048	7.6588e-007	2.51908	E11.5 * Wnt1 up vs E11.5 * Ctrl
10595298	Filip1	NM_001081243	3.65591e-006	2.51498	E11.5 * Wnt1 up vs E11.5 * Ctrl
10574350	Mmp15	NM_008609	6.21354e-008	2.51218	E11.5 * Wnt1 up vs E11.5 * Ctrl
10587299	Ick	NM_019987	9.0156e-011	2.51165	E11.5 * Wnt1 up vs E11.5 * Ctrl
10495781	Bcar3	NM_013867	1.17453e-006	2.50281	E11.5 * Wnt1 up vs E11.5 * Ctrl
10384378	Ddc	NM_016672	7.66233e-010	2.49306	E11.5 * Wnt1 up vs E11.5 * Ctrl
10503190	Chd7	NM_001081417	4.33284e-008	2.49109	E11.5 * Wnt1 up vs E11.5 * Ctrl
10562532	Zfp536	NM_172385	3.69085e-008	2.48071	E11.5 * Wnt1 up vs E11.5 * Ctrl

10479950	Cugbp2	NM_001110231	2.85035e-011	2.47889	E11.5 * Wnt1 up vs E11.5 * Ctrl
10538482	Adcyap1r1	NM_007407	1.6378e-007	2.47626	E11.5 * Wnt1 up vs E11.5 * Ctrl
10368997	C130030K03Rik	AK048022	0.00068519	2.47533	E11.5 * Wnt1 up vs E11.5 * Ctrl
10492628	Serpini1	NM_009250	2.91785e-007	2.47436	E11.5 * Wnt1 up vs E11.5 * Ctrl
10602068	Mid2	NM_011845	1.69533e-010	2.47297	E11.5 * Wnt1 up vs E11.5 * Ctrl
10503206	Chd7	NM_001081417	9.91686e-008	2.47146	E11.5 * Wnt1 up vs E11.5 * Ctrl
10548051	Kcna6	NM_013568	7.15162e-008	2.46754	E11.5 * Wnt1 up vs E11.5 * Ctrl
10399505	Greb1	NM_015764	9.22376e-008	2.46619	E11.5 * Wnt1 up vs E11.5 * Ctrl
10503208	Chd7	NM_001081417	1.94108e-007	2.46573	E11.5 * Wnt1 up vs E11.5 * Ctrl
10497663	Slc7a14	NM_172861	4.52374e-008	2.46518	E11.5 * Wnt1 up vs E11.5 * Ctrl
10365290	Chst11	NM_021439	6.45204e-008	2.46358	E11.5 * Wnt1 up vs E11.5 * Ctrl
10490559	Chrna4	NM_015730	7.20095e-007	2.46063	E11.5 * Wnt1 up vs E11.5 * Ctrl
10350136	Csrp1	NM_007791	1.23423e-008	2.45723	E11.5 * Wnt1 up vs E11.5 * Ctrl
10500114	Mllt11	NM_019914	8.90387e-011	2.45594	E11.5 * Wnt1 up vs E11.5 * Ctrl
10503196	Chd7	NM_001081417	5.09971e-008	2.45278	E11.5 * Wnt1 up vs E11.5 * Ctrl
10469138	4930412O13Rik	NR_024257	4.8491e-005	2.45127	E11.5 * Wnt1 up vs E11.5 * Ctrl
10468992	Frmd4a	NM_172475	7.6364e-010	2.44387	E11.5 * Wnt1 up vs E11.5 * Ctrl
10472984	Hoxd3	NM_010468	1.7162e-008	2.44088	E11.5 * Wnt1 up vs E11.5 * Ctrl
10374197	Ramp3	NM_019511	5.17216e-008	2.43374	E11.5 * Wnt1 up vs E11.5 * Ctrl
10555027	Gab2	NM_010248	1.77836e-010	2.43271	E11.5 * Wnt1 up vs E11.5 * Ctrl
10522976	Rufy3	NM_027530	8.48225e-012	2.42358	E11.5 * Wnt1 up vs E11.5 * Ctrl
10403604	Lyst	NM_010748	6.31302e-010	2.41962	E11.5 * Wnt1 up vs E11.5 * Ctrl
10358549	Hmcn1	NM_001024720	3.18773e-007	2.41892	E11.5 * Wnt1 up vs E11.5 * Ctrl
10457606	Kctd1	NM_134112	1.08418e-007	2.40604	E11.5 * Wnt1 up vs E11.5 * Ctrl
10499062	Fhdc1	NM_001033301	5.95876e-006	2.40576	E11.5 * Wnt1 up vs E11.5 * Ctrl
10358557	Hmcn1	NM_001024720	7.15159e-007	2.40045	E11.5 * Wnt1 up vs E11.5 * Ctrl
10399973	Hdac9	NM_024124	6.34885e-010	2.39261	E11.5 * Wnt1 up vs E11.5 * Ctrl
10493798	S100a16	NM_026416	3.28462e-007	2.37522	E11.5 * Wnt1 up vs E11.5 * Ctrl
10523758	Lrrc8b	NM_001033550	6.12129e-008	2.36819	E11.5 * Wnt1 up vs E11.5 * Ctrl
10415132	Cmtm5	NM_026066	1.94715e-008	2.36183	E11.5 * Wnt1 up vs E11.5 * Ctrl
10546685	Eif4e3	NM_025829	2.33966e-010	2.35969	E11.5 * Wnt1 up vs E11.5 * Ctrl
10397002	Sipa1l1	NM_172579	2.19136e-009	2.35117	E11.5 * Wnt1 up vs E11.5 * Ctrl
10355836	Resp18	NM_009049	4.38664e-007	2.34974	E11.5 * Wnt1 up vs E11.5 * Ctrl
10474064	Trp53i11	NM_001025246	7.6294e-006	2.34512	E11.5 * Wnt1 up vs E11.5 * Ctrl
10481711	Stxbp1	NM_001113569	3.63536e-008	2.34438	E11.5 * Wnt1 up vs E11.5 * Ctrl
10345241	Dst	NM_134448	4.71859e-012	2.34129	E11.5 * Wnt1 up vs E11.5 * Ctrl
10567546	Crym	NM_016669	8.09937e-009	2.33621	E11.5 * Wnt1 up vs E11.5 * Ctrl
10452734	Alk	NM_007439	8.82002e-009	2.31877	E11.5 * Wnt1 up vs E11.5 * Ctrl
10423855	Rims2	NM_053271	9.31221e-008	2.31829	E11.5 * Wnt1 up vs E11.5 * Ctrl
10352092	Zfp238	NM_001012330	5.26771e-009	2.31524	E11.5 * Wnt1 up vs E11.5 * Ctrl
10606259		---	0.0032347	2.30993	E11.5 * Wnt1 up vs E11.5 * Ctrl
10607877	Prps2	NM_026662	7.75743e-009	2.30984	E11.5 * Wnt1 up vs E11.5 * Ctrl
10503178	Chd7	NM_001081417	3.04541e-009	2.30884	E11.5 * Wnt1 up vs E11.5 * Ctrl
10490665	Stmn3	NM_009133	1.273e-007	2.30761	E11.5 * Wnt1 up vs E11.5 * Ctrl
10566668	Socs6	NM_018821	4.74976e-005	2.30751	E11.5 * Wnt1 up vs E11.5 * Ctrl
10601152	Nlgn3	NM_172932	3.89172e-009	2.30514	E11.5 * Wnt1 up vs E11.5 * Ctrl
10503176	Chd7	NM_001081417	2.36176e-008	2.29611	E11.5 * Wnt1 up vs E11.5 * Ctrl
10457644	Cdh2	NM_007664	3.70667e-012	2.29551	E11.5 * Wnt1 up vs E11.5 * Ctrl
10506360	Sgip1	NM_144906	1.95845e-010	2.29373	E11.5 * Wnt1 up vs E11.5 * Ctrl
10553917	Apba2	NM_007461	2.12512e-006	2.29018	E11.5 * Wnt1 up vs E11.5 * Ctrl
10607089	Acsl4	NM_207625	1.90872e-008	2.28995	E11.5 * Wnt1 up vs E11.5 * Ctrl
10503172	Chd7	NM_001081417	0.000395627	2.28763	E11.5 * Wnt1 up vs E11.5 * Ctrl
10421100	Nefm	NM_008691	9.79668e-011	2.28438	E11.5 * Wnt1 up vs E11.5 * Ctrl
10422227	Spry2	NM_011897	6.59935e-008	2.28122	E11.5 * Wnt1 up vs E11.5 * Ctrl
10406434	Mef2c	NM_025282	2.05351e-010	2.279	E11.5 * Wnt1 up vs E11.5 * Ctrl
10448925	Cacna1h	NM_021415	2.98572e-007	2.27413	E11.5 * Wnt1 up vs E11.5 * Ctrl
10503180	Chd7	NM_001081417	8.08753e-008	2.27344	E11.5 * Wnt1 up vs E11.5 * Ctrl
10486201	OTTMUSG00000	ENSMUST00000	9.1091e-006	2.27053	E11.5 * Wnt1 up vs E11.5 * Ctrl
10399360	Rhob	NM_007483	7.40416e-008	2.26986	E11.5 * Wnt1 up vs E11.5 * Ctrl
10355456	Mreg	NM_001005423	1.81887e-006	2.26632	E11.5 * Wnt1 up vs E11.5 * Ctrl
10570516	Kbtbd11	NM_029116	2.538e-010	2.26507	E11.5 * Wnt1 up vs E11.5 * Ctrl
10458645	Ppp2r2b	NM_028392	8.3615e-010	2.26408	E11.5 * Wnt1 up vs E11.5 * Ctrl
10521602	Cpeb2	NM_175937	3.67464e-006	2.26245	E11.5 * Wnt1 up vs E11.5 * Ctrl
10567289	Syt17	NM_138649	5.95788e-008	2.25735	E11.5 * Wnt1 up vs E11.5 * Ctrl
10381934	Tanc2	NM_181071	1.03248e-006	2.24173	E11.5 * Wnt1 up vs E11.5 * Ctrl
10591706	Elavl3	NM_010487	1.72701e-006	2.24087	E11.5 * Wnt1 up vs E11.5 * Ctrl

10427496	Egflam	NM_178748	1.02245e-009	2.24035	E11.5 * Wnt1 up vs E11.5 * Ctrl
10555197	Mtap6	NM_010837	4.98404e-009	2.2392	E11.5 * Wnt1 up vs E11.5 * Ctrl
10375265	Atp10b	NM_176999	7.64243e-008	2.23792	E11.5 * Wnt1 up vs E11.5 * Ctrl
10527158	Fscn1	NM_007984	0.000238584	2.2357	E11.5 * Wnt1 up vs E11.5 * Ctrl
10549200	Sox5	NM_011444	1.79491e-007	2.23562	E11.5 * Wnt1 up vs E11.5 * Ctrl
10572989	Slc10a7	NM_029736	6.31522e-010	2.22537	E11.5 * Wnt1 up vs E11.5 * Ctrl
10593497	Zc3h12c	ENSMUST00000(	3.77372e-007	2.2223	E11.5 * Wnt1 up vs E11.5 * Ctrl
10518484	Fbxo44	NM_173401	5.08054e-008	2.21409	E11.5 * Wnt1 up vs E11.5 * Ctrl
10449041	Metrn	NM_133719	1.0137e-006	2.20358	E11.5 * Wnt1 up vs E11.5 * Ctrl
10431051	Scube1	NM_022723	9.23489e-008	2.19753	E11.5 * Wnt1 up vs E11.5 * Ctrl
10514466	Jun	NM_010591	1.08579e-005	2.19102	E11.5 * Wnt1 up vs E11.5 * Ctrl
10598996	EG331392	NM_198633	4.37449e-007	2.18555	E11.5 * Wnt1 up vs E11.5 * Ctrl
10520527	Dpysl5	NM_023047	2.04646e-007	2.18392	E11.5 * Wnt1 up vs E11.5 * Ctrl
10564211	Snrpn	NM_013670	1.60003e-011	2.18346	E11.5 * Wnt1 up vs E11.5 * Ctrl
10500802	Atg4a	NM_174875	4.94281e-006	2.17951	E11.5 * Wnt1 up vs E11.5 * Ctrl
10503182	Chd7	NM_001081417	6.53783e-010	2.17417	E11.5 * Wnt1 up vs E11.5 * Ctrl
10471424	Fam102a	NM_153560	8.29945e-007	2.17374	E11.5 * Wnt1 up vs E11.5 * Ctrl
10519612	9330182L06Rik	NM_172706	4.67898e-007	2.17363	E11.5 * Wnt1 up vs E11.5 * Ctrl
10426244	Mapk8ip2	NM_021921	6.19373e-006	2.1629	E11.5 * Wnt1 up vs E11.5 * Ctrl
10548735	Dusp16	NM_130447	3.48337e-010	2.16094	E11.5 * Wnt1 up vs E11.5 * Ctrl
10503186	Chd7	NM_001081417	1.01529e-008	2.16073	E11.5 * Wnt1 up vs E11.5 * Ctrl
10347781	9430031J16Rik	BC082310	9.27969e-007	2.15919	E11.5 * Wnt1 up vs E11.5 * Ctrl
10432540	Lima1	NM_001113545	2.25439e-011	2.14962	E11.5 * Wnt1 up vs E11.5 * Ctrl
10394593	Fam49a	NM_029758	1.07287e-006	2.14235	E11.5 * Wnt1 up vs E11.5 * Ctrl
10581914	Chst5	NM_019950	7.39153e-008	2.14188	E11.5 * Wnt1 up vs E11.5 * Ctrl
10503184	Chd7	NM_001081417	1.25959e-009	2.1403	E11.5 * Wnt1 up vs E11.5 * Ctrl
10348493	Lrrfip1	NM_008515	1.97773e-010	2.13892	E11.5 * Wnt1 up vs E11.5 * Ctrl
10518751	Car6	NM_009802	0.000769734	2.13875	E11.5 * Wnt1 up vs E11.5 * Ctrl
10358635	Hmcn1	NM_001024720	9.91767e-006	2.13743	E11.5 * Wnt1 up vs E11.5 * Ctrl
10574166	Cpne2	NM_153507	2.53064e-009	2.13699	E11.5 * Wnt1 up vs E11.5 * Ctrl
10501971	Ank2	NM_178655	4.35115e-009	2.1359	E11.5 * Wnt1 up vs E11.5 * Ctrl
10599001	Agtr2	NM_007429	0.00346454	2.13429	E11.5 * Wnt1 up vs E11.5 * Ctrl
10605431	Rab39b	NM_175122	2.38787e-006	2.13291	E11.5 * Wnt1 up vs E11.5 * Ctrl
10358555	Hmcn1	NM_001024720	2.90833e-006	2.12918	E11.5 * Wnt1 up vs E11.5 * Ctrl
10389590	Gdpd1	NM_025638	8.03203e-008	2.12866	E11.5 * Wnt1 up vs E11.5 * Ctrl
10540227	Kbtbd8	NM_001008785	3.33958e-005	2.12533	E11.5 * Wnt1 up vs E11.5 * Ctrl
10356712	Kif1a	NM_008440	3.83236e-009	2.12501	E11.5 * Wnt1 up vs E11.5 * Ctrl
10518585	Kif1b	NM_207682	2.69475e-010	2.12492	E11.5 * Wnt1 up vs E11.5 * Ctrl
10545538	Ctnna2	NM_009819	1.21589e-006	2.11886	E11.5 * Wnt1 up vs E11.5 * Ctrl
10370766	Gamt	NM_010255	1.3945e-006	2.11592	E11.5 * Wnt1 up vs E11.5 * Ctrl
10503210	Chd7	NM_001081417	1.28427e-006	2.11409	E11.5 * Wnt1 up vs E11.5 * Ctrl
10481845	Fam125b	BC059907	7.33734e-008	2.11332	E11.5 * Wnt1 up vs E11.5 * Ctrl
10595013	Tmod2	NM_016711	2.53336e-009	2.11091	E11.5 * Wnt1 up vs E11.5 * Ctrl
10495285	Sort1	NM_019972	2.7874e-010	2.10954	E11.5 * Wnt1 up vs E11.5 * Ctrl
10355984	Serpine2	NM_009255	5.1519e-011	2.10446	E11.5 * Wnt1 up vs E11.5 * Ctrl
10365471	Fbxo7	NM_153195	9.03173e-010	2.10293	E11.5 * Wnt1 up vs E11.5 * Ctrl
10360920	Tgfb2	NM_009367	2.88006e-014	2.09873	E11.5 * Wnt1 up vs E11.5 * Ctrl
10543145	Thsd7a	ENSMUST00000	6.8467e-008	2.09758	E11.5 * Wnt1 up vs E11.5 * Ctrl
10418895	Zfp488	NM_001013777	5.69408e-008	2.09597	E11.5 * Wnt1 up vs E11.5 * Ctrl
10440522	Adamts1	NM_009621	3.14463e-008	2.09539	E11.5 * Wnt1 up vs E11.5 * Ctrl
10446553	Epb4.1l3	NM_013813	1.40321e-010	2.09519	E11.5 * Wnt1 up vs E11.5 * Ctrl
10452571	Ptprm	NM_008984	1.07352e-006	2.08651	E11.5 * Wnt1 up vs E11.5 * Ctrl
10347036	Mtap2	NM_001039934	4.0756e-010	2.08384	E11.5 * Wnt1 up vs E11.5 * Ctrl
10497920	Ankrd50	NM_001033198	1.25438e-009	2.08079	E11.5 * Wnt1 up vs E11.5 * Ctrl
10593492	Zc3h12c	AK220416	3.03632e-010	2.08035	E11.5 * Wnt1 up vs E11.5 * Ctrl
10556553	Insc	NM_173767	7.80718e-007	2.07916	E11.5 * Wnt1 up vs E11.5 * Ctrl
10497817	Anxa5	NM_009673	2.44529e-008	2.07834	E11.5 * Wnt1 up vs E11.5 * Ctrl
10607113	Rgs3	NM_134257	5.74402e-007	2.07777	E11.5 * Wnt1 up vs E11.5 * Ctrl
10454369	Fhod3	NM_175276	7.15383e-007	2.07463	E11.5 * Wnt1 up vs E11.5 * Ctrl
10535866	Ubl3	NM_011908	1.24502e-010	2.07307	E11.5 * Wnt1 up vs E11.5 * Ctrl
10355225	ENSMUSG0000C	ENSMUST00000(	5.57498e-006	2.0713	E11.5 * Wnt1 up vs E11.5 * Ctrl
10466410	Psat1	NM_177420	4.72825e-008	2.07129	E11.5 * Wnt1 up vs E11.5 * Ctrl
10531437	Scarb2	NM_007644	2.33819e-007	2.06366	E11.5 * Wnt1 up vs E11.5 * Ctrl
10499091	Dear1	NM_001040461	1.68388e-006	2.06269	E11.5 * Wnt1 up vs E11.5 * Ctrl
10557992	Bag3	NM_013863	1.77809e-008	2.0618	E11.5 * Wnt1 up vs E11.5 * Ctrl
10490129	Bmp7	NM_007557	4.3095e-007	2.05582	E11.5 * Wnt1 up vs E11.5 * Ctrl

10358633	Hmcn1	NM_001024720	9.05307e-005	2.05474	E11.5 * Wnt1 up vs E11.5 * Ctrl
10421810	1190002H23Rik	NM_025427	5.79594e-007	2.05387	E11.5 * Wnt1 up vs E11.5 * Ctrl
10607156	Dcx	NM_001110222	2.5309e-006	2.05351	E11.5 * Wnt1 up vs E11.5 * Ctrl
10542414	Ptpro	NM_011216	1.26797e-006	2.05193	E11.5 * Wnt1 up vs E11.5 * Ctrl
10599654	Cxx1c	NM_028375	2.11298e-006	2.05124	E11.5 * Wnt1 up vs E11.5 * Ctrl
10556463	Arntl	NM_007489	2.31013e-008	2.05058	E11.5 * Wnt1 up vs E11.5 * Ctrl
10485402	Fjx1	NM_010218	5.31068e-011	2.04417	E11.5 * Wnt1 up vs E11.5 * Ctrl
10470564	Ralgds	NM_001145835	2.79203e-006	2.04351	E11.5 * Wnt1 up vs E11.5 * Ctrl
10481056	Notch1	NM_008714	1.02572e-005	2.03533	E11.5 * Wnt1 up vs E11.5 * Ctrl
10468311	Sh3pxd2a	NM_008018	1.97792e-006	2.03481	E11.5 * Wnt1 up vs E11.5 * Ctrl
10503902	Cnr1	NM_007726	1.51776e-006	2.0318	E11.5 * Wnt1 up vs E11.5 * Ctrl
10459671	Dcc	NM_007831	7.93999e-009	2.03127	E11.5 * Wnt1 up vs E11.5 * Ctrl
10350742	Rnasel	NM_011882	0.000103174	2.02562	E11.5 * Wnt1 up vs E11.5 * Ctrl
10588509	Pcbp4	NM_021567	0.000176617	2.02374	E11.5 * Wnt1 up vs E11.5 * Ctrl
10345777	Il1rl2	NM_133193	6.25672e-005	2.02258	E11.5 * Wnt1 up vs E11.5 * Ctrl
10520304	Actr3b	NM_001004365	1.40443e-006	2.01992	E11.5 * Wnt1 up vs E11.5 * Ctrl
10358587	Hmcn1	NM_001024720	0.000330018	2.01921	E11.5 * Wnt1 up vs E11.5 * Ctrl
10449034	Fam173a	BC096050	1.30099e-007	2.01676	E11.5 * Wnt1 up vs E11.5 * Ctrl
10503188	Chd7	NM_001081417	8.6295e-006	2.01506	E11.5 * Wnt1 up vs E11.5 * Ctrl
10476759	Rin2	NM_028724	2.43307e-007	2.01455	E11.5 * Wnt1 up vs E11.5 * Ctrl
10595371	Hmgn3	NM_026122	2.76032e-006	2.01424	E11.5 * Wnt1 up vs E11.5 * Ctrl
10576639	Nrp1	NM_008737	7.17892e-008	2.00933	E11.5 * Wnt1 up vs E11.5 * Ctrl
10526191	Gatsl2	BC026208	1.51643e-006	2.00334	E11.5 * Wnt1 up vs E11.5 * Ctrl
10596812	6230427J02Rik	NM_026597	9.93708e-008	2.00238	E11.5 * Wnt1 up vs E11.5 * Ctrl
10358521	Hmcn1	NM_001024720	0.000732485	1.98233	E11.5 * Wnt1 up vs E11.5 * Ctrl
10503174	Chd7	NM_001081417	2.63018e-007	1.98214	E11.5 * Wnt1 up vs E11.5 * Ctrl
10503192	Chd7	NM_001081417	1.27436e-006	1.98204	E11.5 * Wnt1 up vs E11.5 * Ctrl
10358605	Hmcn1	NM_001024720	2.95829e-006	1.98162	E11.5 * Wnt1 up vs E11.5 * Ctrl
10462918	Tmem20	NM_175507	4.05347e-009	1.97826	E11.5 * Wnt1 up vs E11.5 * Ctrl
10495623	ENSMUSG00000	ENSMUST00000	0.000519806	1.977	E11.5 * Wnt1 up vs E11.5 * Ctrl
10392449	Wipi1	NM_145940	1.05703e-006	1.97564	E11.5 * Wnt1 up vs E11.5 * Ctrl
10577586	Ap3m2	NM_001122820	4.12852e-008	1.97351	E11.5 * Wnt1 up vs E11.5 * Ctrl
10503170	Chd7	NM_001081417	3.62788e-009	1.97257	E11.5 * Wnt1 up vs E11.5 * Ctrl
10363455	Pcbd1	NM_025273	4.11725e-006	1.96728	E11.5 * Wnt1 up vs E11.5 * Ctrl
10402283	Itpk1	NM_172584	0.000309564	1.96682	E11.5 * Wnt1 up vs E11.5 * Ctrl
10503168	Chd7	NM_001081417	2.46646e-007	1.96668	E11.5 * Wnt1 up vs E11.5 * Ctrl
10462683	Pcgf5	NM_029508	1.5324e-005	1.96652	E11.5 * Wnt1 up vs E11.5 * Ctrl
10494200	Cdc42se1	NM_172395	6.77887e-007	1.96582	E11.5 * Wnt1 up vs E11.5 * Ctrl
10366391	Kcnc2	NM_001025581	2.26471e-006	1.96559	E11.5 * Wnt1 up vs E11.5 * Ctrl
10602090	Atg4a	NM_174875	2.6211e-006	1.9647	E11.5 * Wnt1 up vs E11.5 * Ctrl
10464283	ENSMUSG00000	ENSMUST00000	2.84126e-007	1.9632	E11.5 * Wnt1 up vs E11.5 * Ctrl
10499093		---	8.96921e-006	1.96215	E11.5 * Wnt1 up vs E11.5 * Ctrl
10353632	Bai3	NM_175642	2.07322e-007	1.95898	E11.5 * Wnt1 up vs E11.5 * Ctrl
10575019	ENSMUSG00000	ENSMUST00000	0.00826294	1.95736	E11.5 * Wnt1 up vs E11.5 * Ctrl
10503161	Chd7	NM_001081417	7.95942e-009	1.95587	E11.5 * Wnt1 up vs E11.5 * Ctrl
10399421	Mycn	NM_008709	3.39508e-007	1.95565	E11.5 * Wnt1 up vs E11.5 * Ctrl
10435789	Zbtb20	ENSMUST00000	4.7787e-006	1.95457	E11.5 * Wnt1 up vs E11.5 * Ctrl
10544906	Ggct	NM_026637	1.23513e-006	1.95317	E11.5 * Wnt1 up vs E11.5 * Ctrl
10381939	Tanc2	NM_181071	5.81361e-008	1.95219	E11.5 * Wnt1 up vs E11.5 * Ctrl
10495854	Prss12	NM_008939	5.91397e-008	1.95217	E11.5 * Wnt1 up vs E11.5 * Ctrl
10358535	Hmcn1	NM_001024720	8.53424e-006	1.9521	E11.5 * Wnt1 up vs E11.5 * Ctrl
10358631	Hmcn1	NM_001024720	7.20002e-005	1.95167	E11.5 * Wnt1 up vs E11.5 * Ctrl
10492826	Fbxw7	NM_080428	2.5623e-010	1.95136	E11.5 * Wnt1 up vs E11.5 * Ctrl
10460118	Socs6	NM_018821	1.29913e-006	1.94779	E11.5 * Wnt1 up vs E11.5 * Ctrl
10391084	Hap1	NM_010404	9.09404e-007	1.94724	E11.5 * Wnt1 up vs E11.5 * Ctrl
10474141	Slc1a2	NM_001077514	2.56221e-006	1.94619	E11.5 * Wnt1 up vs E11.5 * Ctrl
10368981	Lin28b	NM_001031772	5.67129e-007	1.94132	E11.5 * Wnt1 up vs E11.5 * Ctrl
10508651	Sdc3	NM_011520	4.01019e-006	1.93934	E11.5 * Wnt1 up vs E11.5 * Ctrl
10358525	Hmcn1	NM_001024720	6.56722e-006	1.93837	E11.5 * Wnt1 up vs E11.5 * Ctrl
10480329	Dnajc1	NM_007869	9.88145e-007	1.93592	E11.5 * Wnt1 up vs E11.5 * Ctrl
10416887	Slain1	NM_198014	1.49073e-005	1.93506	E11.5 * Wnt1 up vs E11.5 * Ctrl
10414417	Peli2	NM_033602	9.24494e-008	1.93478	E11.5 * Wnt1 up vs E11.5 * Ctrl
10448081	Rgmb	NM_178615	8.0264e-011	1.9337	E11.5 * Wnt1 up vs E11.5 * Ctrl
10367746	Sash1	NM_175155	1.05817e-007	1.93264	E11.5 * Wnt1 up vs E11.5 * Ctrl
10458685	Jakmip2	ENSMUST00000	0.000134892	1.93179	E11.5 * Wnt1 up vs E11.5 * Ctrl
10441456	Synj2	NM_011523	1.26339e-007	1.93144	E11.5 * Wnt1 up vs E11.5 * Ctrl

10358575	Hmcn1	NM_001024720	1.17578e-005	1.93049	E11.5 * Wnt1 up vs E11.5 * Ctrl
10358551	Hmcn1	NM_001024720	2.81868e-005	1.92504	E11.5 * Wnt1 up vs E11.5 * Ctrl
10358637	Hmcn1	NM_001024720	6.27309e-006	1.92299	E11.5 * Wnt1 up vs E11.5 * Ctrl
10454286	Mapre2	NM_153058	5.41758e-010	1.92269	E11.5 * Wnt1 up vs E11.5 * Ctrl
10466976	Gldc	NM_138595	7.26557e-007	1.92182	E11.5 * Wnt1 up vs E11.5 * Ctrl
10358607	Hmcn1	NM_001024720	1.84983e-006	1.91665	E11.5 * Wnt1 up vs E11.5 * Ctrl
10549594	Ttyh1	NM_021324	3.33887e-008	1.91486	E11.5 * Wnt1 up vs E11.5 * Ctrl
10421046	Dock5	NM_177780	5.8747e-010	1.91174	E11.5 * Wnt1 up vs E11.5 * Ctrl
10503204	Chd7	NM_001081417	1.9054e-006	1.90869	E11.5 * Wnt1 up vs E11.5 * Ctrl
10383309		---	0.00376231	1.90735	E11.5 * Wnt1 up vs E11.5 * Ctrl
10571312	Dusp4	NM_176933	2.18465e-005	1.90412	E11.5 * Wnt1 up vs E11.5 * Ctrl
10598833	Chst7	NM_021715	0.000369737	1.90302	E11.5 * Wnt1 up vs E11.5 * Ctrl
10408359	Nrsn1	NM_009513	2.3105e-005	1.90071	E11.5 * Wnt1 up vs E11.5 * Ctrl
10358613	Hmcn1	NM_001024720	3.32233e-005	1.90056	E11.5 * Wnt1 up vs E11.5 * Ctrl
10513869	Megf9	NM_172694	2.81638e-010	1.90003	E11.5 * Wnt1 up vs E11.5 * Ctrl
10420988	Dpysl2	NM_009955	2.91972e-005	1.89979	E11.5 * Wnt1 up vs E11.5 * Ctrl
10568735	Ebf3	NM_001113415	2.00065e-007	1.89736	E11.5 * Wnt1 up vs E11.5 * Ctrl
10406852	Cnn3	NM_028044	8.87438e-005	1.89361	E11.5 * Wnt1 up vs E11.5 * Ctrl
10428857	Mtss1	NM_001146180	5.63845e-008	1.89337	E11.5 * Wnt1 up vs E11.5 * Ctrl
10392910	C630004H02Rik	BC024617	0.00048129	1.89239	E11.5 * Wnt1 up vs E11.5 * Ctrl
10358529	Hmcn1	NM_001024720	4.60516e-006	1.8872	E11.5 * Wnt1 up vs E11.5 * Ctrl
10501456	Fam102b	ENSMUST00000(	4.23996e-007	1.88637	E11.5 * Wnt1 up vs E11.5 * Ctrl
10358490	Hmcn1	NM_001024720	3.03668e-007	1.88037	E11.5 * Wnt1 up vs E11.5 * Ctrl
10358553	Hmcn1	NM_001024720	0.000626848	1.87938	E11.5 * Wnt1 up vs E11.5 * Ctrl
10599120	Dock11	NM_001009947	3.83483e-007	1.87659	E11.5 * Wnt1 up vs E11.5 * Ctrl
10399457	Akr1b3	NM_009658	7.17309e-008	1.87447	E11.5 * Wnt1 up vs E11.5 * Ctrl
10468668	Afap1l2	NM_146102	8.6816e-005	1.87335	E11.5 * Wnt1 up vs E11.5 * Ctrl
10582162	Cotl1	NM_028071	0.000694911	1.87203	E11.5 * Wnt1 up vs E11.5 * Ctrl
10362005	Ahi1	NM_026203	2.00188e-006	1.87159	E11.5 * Wnt1 up vs E11.5 * Ctrl
10498210	Nbea	NM_030595	2.9133e-010	1.87144	E11.5 * Wnt1 up vs E11.5 * Ctrl
10407481	Pfkp	NM_019703	1.88282e-006	1.87012	E11.5 * Wnt1 up vs E11.5 * Ctrl
10358595	Hmcn1	NM_001024720	4.87673e-005	1.87001	E11.5 * Wnt1 up vs E11.5 * Ctrl
10406817	Enc1	NM_007930	2.34097e-007	1.86944	E11.5 * Wnt1 up vs E11.5 * Ctrl
10358650	Hmcn1	NM_001024720	2.6967e-006	1.86904	E11.5 * Wnt1 up vs E11.5 * Ctrl
10408613	Tubb2b	NM_023716	8.59e-005	1.86809	E11.5 * Wnt1 up vs E11.5 * Ctrl
10595496	Snap91	NM_013669	2.95933e-005	1.86414	E11.5 * Wnt1 up vs E11.5 * Ctrl
10455942	A730017C20Rik	NM_173759	2.00857e-005	1.86269	E11.5 * Wnt1 up vs E11.5 * Ctrl
10426656	Prph	NM_013639	1.75033e-006	1.86126	E11.5 * Wnt1 up vs E11.5 * Ctrl
10375432	C030019I05Rik	NM_177075	0.000665995	1.8582	E11.5 * Wnt1 up vs E11.5 * Ctrl
10408450	Sox4	NM_009238	1.79313e-005	1.85742	E11.5 * Wnt1 up vs E11.5 * Ctrl
10571860	Hand2	NM_010402	1.75767e-007	1.8559	E11.5 * Wnt1 up vs E11.5 * Ctrl
10538338	Creb5	NM_172728	6.568e-009	1.85588	E11.5 * Wnt1 up vs E11.5 * Ctrl
10469151	Itih5	NM_172471	9.58317e-008	1.85582	E11.5 * Wnt1 up vs E11.5 * Ctrl
10425945	Fbln1	NM_010180	3.48093e-006	1.85411	E11.5 * Wnt1 up vs E11.5 * Ctrl
10406845	Foxd1	NM_008242	0.00686457	1.8541	E11.5 * Wnt1 up vs E11.5 * Ctrl
10451679	Daam2	NM_001008231	1.30227e-006	1.85289	E11.5 * Wnt1 up vs E11.5 * Ctrl
10354741	Rftn2	NM_028713	2.03203e-008	1.85249	E11.5 * Wnt1 up vs E11.5 * Ctrl
10588283	Rab6b	NM_173781	0.000208034	1.85129	E11.5 * Wnt1 up vs E11.5 * Ctrl
10595831	Zbtb38	NM_175537	1.54404e-006	1.8498	E11.5 * Wnt1 up vs E11.5 * Ctrl
10358627	Hmcn1	NM_001024720	9.3543e-006	1.84792	E11.5 * Wnt1 up vs E11.5 * Ctrl
10345172	ENSMUSG0000C	ENSMUST00000(	0.000283962	1.84561	E11.5 * Wnt1 up vs E11.5 * Ctrl
10452295	Tubb4	NM_009451	5.17576e-005	1.84364	E11.5 * Wnt1 up vs E11.5 * Ctrl
10374406	Cnrip1	NM_029861	2.64194e-007	1.8429	E11.5 * Wnt1 up vs E11.5 * Ctrl
10350896	Astn1	NM_007495	1.85284e-006	1.83515	E11.5 * Wnt1 up vs E11.5 * Ctrl
10564482	Synm	NM_201639	9.42364e-008	1.83387	E11.5 * Wnt1 up vs E11.5 * Ctrl
10566767	St5	NM_001001326	1.70172e-006	1.83264	E11.5 * Wnt1 up vs E11.5 * Ctrl
10445879	Kcnh8	NM_001031811	0.000185608	1.83141	E11.5 * Wnt1 up vs E11.5 * Ctrl
10358577	Hmcn1	NM_001024720	0.000159413	1.83064	E11.5 * Wnt1 up vs E11.5 * Ctrl
10376017	ENSMUSG0000C	ENSMUST00000(	0.000172525	1.83044	E11.5 * Wnt1 up vs E11.5 * Ctrl
10358623	Hmcn1	NM_001024720	8.47474e-006	1.8294	E11.5 * Wnt1 up vs E11.5 * Ctrl
10358648	Hmcn1	NM_001024720	3.49332e-005	1.82875	E11.5 * Wnt1 up vs E11.5 * Ctrl
10429160	St3gal1	NM_009177	5.75325e-006	1.82673	E11.5 * Wnt1 up vs E11.5 * Ctrl
10379936	Tbx2	NM_009324	4.08063e-005	1.82122	E11.5 * Wnt1 up vs E11.5 * Ctrl
10450603		---	0.00280764	1.82031	E11.5 * Wnt1 up vs E11.5 * Ctrl
10459835		---	2.94811e-005	1.81994	E11.5 * Wnt1 up vs E11.5 * Ctrl
10600688	Map3k7ip3	NM_025729	4.60996e-007	1.81856	E11.5 * Wnt1 up vs E11.5 * Ctrl

10404975	Id4	NM_031166	2.07931e-006	1.81801	E11.5 * Wnt1 up vs E11.5 * Ctrl
10358597	Hmcn1	NM_001024720	0.000234196	1.8153	E11.5 * Wnt1 up vs E11.5 * Ctrl
10406456	AY512938	AY512938	0.00106322	1.8144	E11.5 * Wnt1 up vs E11.5 * Ctrl
10358513	Hmcn1	NM_001024720	0.000724083	1.81429	E11.5 * Wnt1 up vs E11.5 * Ctrl
10550915	Cadm4	NM_153112	7.96049e-005	1.81429	E11.5 * Wnt1 up vs E11.5 * Ctrl
10492335	Rap2b	NM_028712	1.81856e-007	1.81211	E11.5 * Wnt1 up vs E11.5 * Ctrl
10359917	Hsd17b7	NM_010476	0.00019855	1.81089	E11.5 * Wnt1 up vs E11.5 * Ctrl
10350697	Nmnat2	NM_175460	2.12739e-005	1.81015	E11.5 * Wnt1 up vs E11.5 * Ctrl
10358527	Hmcn1	NM_001024720	3.68677e-005	1.80924	E11.5 * Wnt1 up vs E11.5 * Ctrl
10575777	4933407C03Rik	BC158118	1.92467e-006	1.80924	E11.5 * Wnt1 up vs E11.5 * Ctrl
10361023	Prox1	NM_008937	0.000105418	1.8081	E11.5 * Wnt1 up vs E11.5 * Ctrl
10528527	Fam126a	NM_053090	3.59696e-007	1.80762	E11.5 * Wnt1 up vs E11.5 * Ctrl
10439092	1700021K19Rik	BC060601	1.07651e-007	1.80624	E11.5 * Wnt1 up vs E11.5 * Ctrl
10592471	Gramd1b	NM_172768	3.85049e-006	1.80622	E11.5 * Wnt1 up vs E11.5 * Ctrl
10358599	Hmcn1	NM_001024720	8.90993e-005	1.80584	E11.5 * Wnt1 up vs E11.5 * Ctrl
10579925	Gab1	NM_021356	8.21928e-009	1.80338	E11.5 * Wnt1 up vs E11.5 * Ctrl
10372338	EG368203	NM_203660	0.00274463	1.79824	E11.5 * Wnt1 up vs E11.5 * Ctrl
10364502	Palm	NM_023128	1.34503e-005	1.79788	E11.5 * Wnt1 up vs E11.5 * Ctrl
10474373	Kcna4	NM_021275	0.000399638	1.79693	E11.5 * Wnt1 up vs E11.5 * Ctrl
10605820	Zc4h2	NM_001003916	2.14648e-007	1.79666	E11.5 * Wnt1 up vs E11.5 * Ctrl
10433445	Abat	NM_172961	0.000106897	1.79524	E11.5 * Wnt1 up vs E11.5 * Ctrl
10490159	Pmepa1	NM_022995	8.72566e-007	1.79453	E11.5 * Wnt1 up vs E11.5 * Ctrl
10454580	Bin1	NM_009668	7.58201e-005	1.79235	E11.5 * Wnt1 up vs E11.5 * Ctrl
10561369	BC089491	NM_175033	2.37957e-006	1.78756	E11.5 * Wnt1 up vs E11.5 * Ctrl
10407072	Elovl7	NM_029001	0.000820283	1.78629	E11.5 * Wnt1 up vs E11.5 * Ctrl
10553773	Gabrb3	NM_008071	5.20964e-007	1.78499	E11.5 * Wnt1 up vs E11.5 * Ctrl
10599562	1100001E04Rik	NM_001081123	1.71255e-005	1.78268	E11.5 * Wnt1 up vs E11.5 * Ctrl
10375263	ENSMUSG0000C	ENSMUST00000(	0.00090716	1.77984	E11.5 * Wnt1 up vs E11.5 * Ctrl
10430519	Csnk1e	NM_013767	1.8743e-006	1.77964	E11.5 * Wnt1 up vs E11.5 * Ctrl
10411274	Sv2c	NM_029210	2.98855e-006	1.77918	E11.5 * Wnt1 up vs E11.5 * Ctrl
10436456	Pros1	NM_011173	3.90498e-006	1.77522	E11.5 * Wnt1 up vs E11.5 * Ctrl
10375175	Slit3	NM_011412	3.31656e-007	1.77441	E11.5 * Wnt1 up vs E11.5 * Ctrl
10375322	4933415A04Rik	ENSMUST00000(	2.04999e-006	1.77132	E11.5 * Wnt1 up vs E11.5 * Ctrl
10509127	Fuca1	NM_024243	9.83929e-007	1.76633	E11.5 * Wnt1 up vs E11.5 * Ctrl
10492355	Mme	NM_008604	0.000793227	1.76626	E11.5 * Wnt1 up vs E11.5 * Ctrl
10575775	4933407C03Rik	BC158118	1.08528e-006	1.7647	E11.5 * Wnt1 up vs E11.5 * Ctrl
10590844	9030420J04Rik	BC137891	1.5813e-007	1.76453	E11.5 * Wnt1 up vs E11.5 * Ctrl
10397912	9030205A07Rik	AB257853	5.75275e-007	1.7644	E11.5 * Wnt1 up vs E11.5 * Ctrl
10590860	9030420J04Rik	BC137891	5.8132e-006	1.76145	E11.5 * Wnt1 up vs E11.5 * Ctrl
10358531	Hmcn1	NM_001024720	8.3707e-005	1.75916	E11.5 * Wnt1 up vs E11.5 * Ctrl
10472794	Metapl1	NM_025633	1.94357e-005	1.75696	E11.5 * Wnt1 up vs E11.5 * Ctrl
10385283	Gabrg2	NM_008073	0.000104586	1.75533	E11.5 * Wnt1 up vs E11.5 * Ctrl
10578493	Tlr3	NM_126166	0.000258787	1.755	E11.5 * Wnt1 up vs E11.5 * Ctrl
10457820	Nol4	NM_199024	4.17937e-005	1.75488	E11.5 * Wnt1 up vs E11.5 * Ctrl
10593196	ENSMUSG0000C	ENSMUST00000(	7.62967e-007	1.75392	E11.5 * Wnt1 up vs E11.5 * Ctrl
10438626	Etv5	NM_023794	5.54467e-005	1.75149	E11.5 * Wnt1 up vs E11.5 * Ctrl
10459552	Spire1	NM_194355	1.06619e-008	1.75135	E11.5 * Wnt1 up vs E11.5 * Ctrl
10575497	Mtss1l	NM_198625	5.85568e-005	1.75072	E11.5 * Wnt1 up vs E11.5 * Ctrl
10466712	Mamdc2	NM_174857	2.94093e-006	1.75053	E11.5 * Wnt1 up vs E11.5 * Ctrl
10372557	Gm239	NM_001033333	4.74337e-005	1.75053	E11.5 * Wnt1 up vs E11.5 * Ctrl
10407841	Hecw1	NM_001081348	0.000742573	1.74994	E11.5 * Wnt1 up vs E11.5 * Ctrl
10411171	Pde8b	NM_172263	9.37692e-008	1.74545	E11.5 * Wnt1 up vs E11.5 * Ctrl
10458555	Spry4	NM_011898	2.94876e-005	1.74533	E11.5 * Wnt1 up vs E11.5 * Ctrl
10363161	6330442E10Rik	BC079613	0.00073547	1.74468	E11.5 * Wnt1 up vs E11.5 * Ctrl
10434165	Arvcf	NM_033474	2.23356e-006	1.74379	E11.5 * Wnt1 up vs E11.5 * Ctrl
10503259	Trp53inp1	NM_021897	4.71398e-007	1.74365	E11.5 * Wnt1 up vs E11.5 * Ctrl
10381082	Rara	NM_009024	4.19262e-005	1.74356	E11.5 * Wnt1 up vs E11.5 * Ctrl
10540233	Fam19a1	NM_182808	2.62212e-005	1.74059	E11.5 * Wnt1 up vs E11.5 * Ctrl
10607302	Gnl3l	NM_198110	5.22677e-009	1.74003	E11.5 * Wnt1 up vs E11.5 * Ctrl
10476443	Plcb4	NM_013829	4.26689e-007	1.73954	E11.5 * Wnt1 up vs E11.5 * Ctrl
10460100		---	1.19316e-005	1.73909	E11.5 * Wnt1 up vs E11.5 * Ctrl
10590383	Deb1	NM_026794	9.99269e-008	1.73906	E11.5 * Wnt1 up vs E11.5 * Ctrl
10358537	Hmcn1	NM_001024720	6.61439e-007	1.7387	E11.5 * Wnt1 up vs E11.5 * Ctrl
10352503	Bpnt1	NM_011794	2.20563e-005	1.73829	E11.5 * Wnt1 up vs E11.5 * Ctrl
10358609	Hmcn1	NM_001024720	4.74718e-005	1.73649	E11.5 * Wnt1 up vs E11.5 * Ctrl
10462702	Hectd2	NM_172637	5.18272e-005	1.73641	E11.5 * Wnt1 up vs E11.5 * Ctrl

10426798	Smarcd1	NM_031842	7.2188e-006	1.7364	E11.5 * Wnt1 up vs E11.5 * Ctrl
10439009	Apod	NM_007470	4.90874e-005	1.73604	E11.5 * Wnt1 up vs E11.5 * Ctrl
10490794	Pkia	NM_008862	2.79609e-005	1.73402	E11.5 * Wnt1 up vs E11.5 * Ctrl
10392388	Prkca	NM_011101	4.57208e-007	1.73326	E11.5 * Wnt1 up vs E11.5 * Ctrl
10444932	2310014H01Rik	NM_001146711	5.52844e-006	1.73218	E11.5 * Wnt1 up vs E11.5 * Ctrl
10573172	Clgn	NM_009904	1.46878e-005	1.72986	E11.5 * Wnt1 up vs E11.5 * Ctrl
10359849	Uck2	ENSMUST00000(	0.00324412	1.72949	E11.5 * Wnt1 up vs E11.5 * Ctrl
10516371	Eif2c1	NM_153403	5.45734e-006	1.72936	E11.5 * Wnt1 up vs E11.5 * Ctrl
10569370	Th	NM_009377	1.97492e-006	1.72819	E11.5 * Wnt1 up vs E11.5 * Ctrl
10358625	Hmcn1	NM_001024720	0.000272653	1.72766	E11.5 * Wnt1 up vs E11.5 * Ctrl
10470314		---	0.00401972	1.72724	E11.5 * Wnt1 up vs E11.5 * Ctrl
10422249	Dct	NM_010024	0.000148983	1.72579	E11.5 * Wnt1 up vs E11.5 * Ctrl
10538732	Grid2	NM_008167	5.32745e-005	1.72563	E11.5 * Wnt1 up vs E11.5 * Ctrl
10408838	Elovl2	NM_019423	5.34904e-005	1.72509	E11.5 * Wnt1 up vs E11.5 * Ctrl
10542369	EG668137	NM_001142734	1.2841e-005	1.7249	E11.5 * Wnt1 up vs E11.5 * Ctrl
10589913	Dync1li1	NM_146229	5.6159e-007	1.72438	E11.5 * Wnt1 up vs E11.5 * Ctrl
10436978	Cbr3	NM_173047	0.00317074	1.72366	E11.5 * Wnt1 up vs E11.5 * Ctrl
10506150	Foxd3	NM_010425	0.000653477	1.72239	E11.5 * Wnt1 up vs E11.5 * Ctrl
10492448	Ptx3	NM_008987	0.00116376	1.72209	E11.5 * Wnt1 up vs E11.5 * Ctrl
10354494	Nab1	NM_008667	3.26389e-006	1.72197	E11.5 * Wnt1 up vs E11.5 * Ctrl
10462005	Tmem2	NM_031997	1.15232e-007	1.72144	E11.5 * Wnt1 up vs E11.5 * Ctrl
10496091	Lef1	NM_010703	6.29464e-006	1.71803	E11.5 * Wnt1 up vs E11.5 * Ctrl
10427796	Npr3	NM_008728	7.86526e-007	1.71493	E11.5 * Wnt1 up vs E11.5 * Ctrl
10438017	Fgd4	NM_139232	8.36966e-006	1.71024	E11.5 * Wnt1 up vs E11.5 * Ctrl
10415911	Kif13b	NM_001081177	2.37026e-005	1.70917	E11.5 * Wnt1 up vs E11.5 * Ctrl
10389581	Ypel2	NM_001005341	4.39135e-007	1.70915	E11.5 * Wnt1 up vs E11.5 * Ctrl
10578521	Snx25	NM_207213	9.01257e-007	1.70875	E11.5 * Wnt1 up vs E11.5 * Ctrl
10483604	Slc25a12	NM_172436	3.61036e-006	1.70816	E11.5 * Wnt1 up vs E11.5 * Ctrl
10518408	Plod1	NM_011122	6.06563e-005	1.70803	E11.5 * Wnt1 up vs E11.5 * Ctrl
10446777	Ehd3	NM_020578	2.62247e-005	1.70775	E11.5 * Wnt1 up vs E11.5 * Ctrl
10561376	Dll3	NM_007866	4.30234e-006	1.70595	E11.5 * Wnt1 up vs E11.5 * Ctrl
10404848	Jarid2	NM_021878	8.1397e-010	1.70489	E11.5 * Wnt1 up vs E11.5 * Ctrl
10560919	Atp1a3	NM_144921	0.000990063	1.70447	E11.5 * Wnt1 up vs E11.5 * Ctrl
10405343	Tspan17	NM_028841	7.11361e-006	1.70415	E11.5 * Wnt1 up vs E11.5 * Ctrl
10510125	9430007A20Rik	ENSMUST00000(	0.000783281	1.70176	E11.5 * Wnt1 up vs E11.5 * Ctrl
10493114	Nes	NM_016701	0.0013284	1.70037	E11.5 * Wnt1 up vs E11.5 * Ctrl
10358629	Hmcn1	NM_001024720	0.000205548	1.69979	E11.5 * Wnt1 up vs E11.5 * Ctrl
10355176	4921521F21Rik	BC051128	2.15781e-005	1.69928	E11.5 * Wnt1 up vs E11.5 * Ctrl
10506452	AY512949	AY512949	0.00136978	1.69886	E11.5 * Wnt1 up vs E11.5 * Ctrl
10488709	8430427H17Rik	NM_001134300	3.45742e-006	1.69823	E11.5 * Wnt1 up vs E11.5 * Ctrl
10441361	Tiam2	NM_001122998	4.90591e-006	1.69815	E11.5 * Wnt1 up vs E11.5 * Ctrl
10478364	Tox2	NM_001098799	2.58884e-006	1.69789	E11.5 * Wnt1 up vs E11.5 * Ctrl
10504234	Unc13b	NM_001081413	2.31396e-007	1.69711	E11.5 * Wnt1 up vs E11.5 * Ctrl
10358652	Hmcn1	NM_001024720	1.25762e-005	1.69675	E11.5 * Wnt1 up vs E11.5 * Ctrl
10402560	A130014H13Rik	AK079474	0.00240606	1.69661	E11.5 * Wnt1 up vs E11.5 * Ctrl
10536805	Fam71f1	NM_207258	1.94049e-005	1.69661	E11.5 * Wnt1 up vs E11.5 * Ctrl
10496727	Ddah1	NM_026993	3.51157e-007	1.69583	E11.5 * Wnt1 up vs E11.5 * Ctrl
10524684	Msi1	NM_008629	0.000414782	1.69567	E11.5 * Wnt1 up vs E11.5 * Ctrl
10514561	E130114P18Rik	ENSMUST00000(	6.34187e-005	1.69558	E11.5 * Wnt1 up vs E11.5 * Ctrl
10358601	Hmcn1	NM_001024720	0.000177918	1.69313	E11.5 * Wnt1 up vs E11.5 * Ctrl
10384138	Tmed4	NM_134020	0.000300591	1.69008	E11.5 * Wnt1 up vs E11.5 * Ctrl
10527965	Cldn12	NM_022890	0.00137222	1.6885	E11.5 * Wnt1 up vs E11.5 * Ctrl
10425158	Pdxp	NM_020271	4.81814e-005	1.68713	E11.5 * Wnt1 up vs E11.5 * Ctrl
10400357	Baz1a	NM_013815	1.53301e-005	1.68683	E11.5 * Wnt1 up vs E11.5 * Ctrl
10459827	Rnf165	ENSMUST00000(	1.82264e-007	1.68474	E11.5 * Wnt1 up vs E11.5 * Ctrl
10358660	Hmcn1	NM_001024720	0.000256124	1.68389	E11.5 * Wnt1 up vs E11.5 * Ctrl
10538247	Npy	NM_023456	0.000245892	1.68309	E11.5 * Wnt1 up vs E11.5 * Ctrl
10437580		---	0.000216654	1.68242	E11.5 * Wnt1 up vs E11.5 * Ctrl
10358656	Hmcn1	NM_001024720	1.46556e-006	1.68162	E11.5 * Wnt1 up vs E11.5 * Ctrl
10593671	Dmxl2	NM_172771	4.71579e-007	1.68161	E11.5 * Wnt1 up vs E11.5 * Ctrl
10370072	Prmt2	NM_133182	8.65449e-005	1.68134	E11.5 * Wnt1 up vs E11.5 * Ctrl
10392369	Cacng4	NM_019431	3.66992e-006	1.6811	E11.5 * Wnt1 up vs E11.5 * Ctrl
10523595	Ptpn13	NM_011204	7.66554e-009	1.68015	E11.5 * Wnt1 up vs E11.5 * Ctrl
10483626	Dlx2	NM_010054	3.99127e-006	1.67977	E11.5 * Wnt1 up vs E11.5 * Ctrl
10558971	Ap2a2	NM_007459	1.46588e-006	1.67943	E11.5 * Wnt1 up vs E11.5 * Ctrl
10374106	Ykt6	NM_019661	0.000194195	1.67831	E11.5 * Wnt1 up vs E11.5 * Ctrl

10463799	EG329070	ENSMUST00000	1.10686e-007	1.67471	E11.5 * Wnt1 up vs E11.5 * Ctrl
10391146	Acly	NM_134037	3.51403e-005	1.67436	E11.5 * Wnt1 up vs E11.5 * Ctrl
10460645	Map3k11	NM_022012	0.00195951	1.67408	E11.5 * Wnt1 up vs E11.5 * Ctrl
10377018	Myh3	NM_001099635	1.05455e-007	1.67283	E11.5 * Wnt1 up vs E11.5 * Ctrl
10399470	Trib2	NM_144551	6.86169e-007	1.6723	E11.5 * Wnt1 up vs E11.5 * Ctrl
10358515	Hmcn1	NM_001024720	0.00687926	1.67121	E11.5 * Wnt1 up vs E11.5 * Ctrl
10357220	Tmem177	NM_175106	0.000378264	1.67108	E11.5 * Wnt1 up vs E11.5 * Ctrl
10441565	Rps6ka2	NM_011299	4.85562e-006	1.66973	E11.5 * Wnt1 up vs E11.5 * Ctrl
10364293	Ube2g2	NM_019803	0.000170742	1.6691	E11.5 * Wnt1 up vs E11.5 * Ctrl
10558001	Inpp5f	NM_178641	4.24234e-006	1.66723	E11.5 * Wnt1 up vs E11.5 * Ctrl
10564203		---	0.00649516	1.66502	E11.5 * Wnt1 up vs E11.5 * Ctrl
10370242	Pcbp3	NM_021568	1.2075e-006	1.66367	E11.5 * Wnt1 up vs E11.5 * Ctrl
10354768	Akr1b3	NM_009658	1.73163e-008	1.66359	E11.5 * Wnt1 up vs E11.5 * Ctrl
10541260	Cecr2	NM_001128151	0.000134199	1.66295	E11.5 * Wnt1 up vs E11.5 * Ctrl
10478897	Ptpn1	NM_011201	1.52337e-007	1.66282	E11.5 * Wnt1 up vs E11.5 * Ctrl
10472923	Ak3l1	NM_009647	0.00691897	1.66281	E11.5 * Wnt1 up vs E11.5 * Ctrl
10455374	Stk32a	NM_178749	1.216e-005	1.66123	E11.5 * Wnt1 up vs E11.5 * Ctrl
10354777	Satb2	NM_139146	0.000410039	1.66077	E11.5 * Wnt1 up vs E11.5 * Ctrl
10412038	Zswim6	NM_145456	7.59461e-009	1.66	E11.5 * Wnt1 up vs E11.5 * Ctrl
10534120	Asl	NM_133768	3.37898e-006	1.65973	E11.5 * Wnt1 up vs E11.5 * Ctrl
10460057	Tshz1	NM_001081300	5.54133e-006	1.65958	E11.5 * Wnt1 up vs E11.5 * Ctrl
10435769	Zbtb20	NM_019778	1.22644e-006	1.65924	E11.5 * Wnt1 up vs E11.5 * Ctrl
10483719	Chn1	NM_001113246	3.73885e-007	1.65886	E11.5 * Wnt1 up vs E11.5 * Ctrl
10381250	Tubg1	NM_134024	0.000111552	1.65881	E11.5 * Wnt1 up vs E11.5 * Ctrl
10369835	Phyhipl	NM_178621	3.49396e-005	1.65861	E11.5 * Wnt1 up vs E11.5 * Ctrl
10537146	Akr1b8	NM_008012	0.00428729	1.65826	E11.5 * Wnt1 up vs E11.5 * Ctrl
10498284	Wwtr1	NM_133784	3.1721e-007	1.65731	E11.5 * Wnt1 up vs E11.5 * Ctrl
10420846	Fzd3	NM_021458	2.63812e-007	1.65714	E11.5 * Wnt1 up vs E11.5 * Ctrl
10476301	Smox	NM_145533	0.000155914	1.65675	E11.5 * Wnt1 up vs E11.5 * Ctrl
10358533	Hmcn1	NM_001024720	8.29209e-005	1.65537	E11.5 * Wnt1 up vs E11.5 * Ctrl
10436666	Jam2	NM_023844	8.11448e-006	1.6549	E11.5 * Wnt1 up vs E11.5 * Ctrl
10568651	Dhx32	NM_133941	2.21928e-006	1.65222	E11.5 * Wnt1 up vs E11.5 * Ctrl
10475643	Fgf7	NM_008008	0.00240836	1.65161	E11.5 * Wnt1 up vs E11.5 * Ctrl
10595392	Elovl4	NM_148941	0.00135161	1.64836	E11.5 * Wnt1 up vs E11.5 * Ctrl
10564417	Aldh1a3	NM_053080	9.49911e-005	1.64835	E11.5 * Wnt1 up vs E11.5 * Ctrl
10392943	Hn1	NM_008258	9.24334e-006	1.64722	E11.5 * Wnt1 up vs E11.5 * Ctrl
10474814	Disp2	NM_170593	0.000257159	1.64616	E11.5 * Wnt1 up vs E11.5 * Ctrl
10396476	Rhoj	NM_023275	4.25617e-005	1.64598	E11.5 * Wnt1 up vs E11.5 * Ctrl
10566846	Dennd5a	NM_021494	7.28373e-009	1.64592	E11.5 * Wnt1 up vs E11.5 * Ctrl
10378914	Myo18a	NM_011586	1.68034e-008	1.64576	E11.5 * Wnt1 up vs E11.5 * Ctrl
10554960	Fam181b	NM_021427	3.33133e-005	1.64528	E11.5 * Wnt1 up vs E11.5 * Ctrl
10464504	Lrp5	NM_008513	0.00117722	1.64468	E11.5 * Wnt1 up vs E11.5 * Ctrl
10431170	5031439G07Rik	NM_001033273	1.26613e-005	1.64445	E11.5 * Wnt1 up vs E11.5 * Ctrl
10527516	Wasf3	NM_145155	3.22487e-005	1.64398	E11.5 * Wnt1 up vs E11.5 * Ctrl
10470696	Med27	NM_026896	0.000216093	1.6435	E11.5 * Wnt1 up vs E11.5 * Ctrl
10378549	Rtn4rl1	NM_177708	1.61276e-005	1.64058	E11.5 * Wnt1 up vs E11.5 * Ctrl
10591853	Tbx20	NM_194263	0.000214703	1.64019	E11.5 * Wnt1 up vs E11.5 * Ctrl
10543859	Akr1b3	NM_009658	3.67459e-008	1.63705	E11.5 * Wnt1 up vs E11.5 * Ctrl
10527306	Lmtk2	NM_001081109	6.58538e-007	1.63629	E11.5 * Wnt1 up vs E11.5 * Ctrl
10481830	Zbtb43	NM_027947	0.000150964	1.63484	E11.5 * Wnt1 up vs E11.5 * Ctrl
10456237	St8sia3	NM_009182	0.00019936	1.63452	E11.5 * Wnt1 up vs E11.5 * Ctrl
10355974	Wdfy1	NM_001111279	3.69328e-006	1.63377	E11.5 * Wnt1 up vs E11.5 * Ctrl
10365833	Usp44	NM_183199	0.0001437	1.63371	E11.5 * Wnt1 up vs E11.5 * Ctrl
10491780	Hspa4l	NM_011020	0.000184804	1.63339	E11.5 * Wnt1 up vs E11.5 * Ctrl
10365482	Timp3	NM_011595	0.000451328	1.63316	E11.5 * Wnt1 up vs E11.5 * Ctrl
10604633	Cxx1b	NM_001018063	1.76652e-008	1.63293	E11.5 * Wnt1 up vs E11.5 * Ctrl
10358579	Hmcn1	NM_001024720	0.000107488	1.63268	E11.5 * Wnt1 up vs E11.5 * Ctrl
10505008	Slc44a1	NM_133891	3.0794e-008	1.63148	E11.5 * Wnt1 up vs E11.5 * Ctrl
10388465	Doc2b	NM_007873	1.49349e-005	1.63148	E11.5 * Wnt1 up vs E11.5 * Ctrl
10579335	Pgpep1	NM_023217	8.52778e-007	1.63065	E11.5 * Wnt1 up vs E11.5 * Ctrl
10478962	2010011I20Rik	NM_025912	0.000552071	1.63023	E11.5 * Wnt1 up vs E11.5 * Ctrl
10485309	E530001K10Rik	ENSMUST00000	0.00855979	1.62992	E11.5 * Wnt1 up vs E11.5 * Ctrl
10582275	Slc7a5	NM_011404	0.0017092	1.62965	E11.5 * Wnt1 up vs E11.5 * Ctrl
10600836	Msn	NM_010833	1.7234e-008	1.6295	E11.5 * Wnt1 up vs E11.5 * Ctrl
10362394	Hddc2	NM_027168	9.66331e-005	1.62901	E11.5 * Wnt1 up vs E11.5 * Ctrl
10375880	Nhp2	NM_026631	4.32007e-005	1.62895	E11.5 * Wnt1 up vs E11.5 * Ctrl

10569429	Cdkn1c	NM_009876	2.50243e-009	1.6288	E11.5 * Wnt1 up vs E11.5 * Ctrl
10503643	Ndufaf4	NM_026742	1.53099e-007	1.62852	E11.5 * Wnt1 up vs E11.5 * Ctrl
10392056	Cyb561	NM_007805	0.000268487	1.62826	E11.5 * Wnt1 up vs E11.5 * Ctrl
10358581	Hmcn1	NM_001024720	0.000281245	1.62779	E11.5 * Wnt1 up vs E11.5 * Ctrl
10358539	Hmcn1	NM_001024720	0.00135082	1.62557	E11.5 * Wnt1 up vs E11.5 * Ctrl
10358611	Hmcn1	NM_001024720	6.82954e-005	1.62317	E11.5 * Wnt1 up vs E11.5 * Ctrl
10594001	Arid3b	NM_019689	9.60543e-007	1.6223	E11.5 * Wnt1 up vs E11.5 * Ctrl
10577315	Angpt2	NM_007426	2.53794e-006	1.62201	E11.5 * Wnt1 up vs E11.5 * Ctrl
10536483	Tes	NM_207176	1.88169e-011	1.62199	E11.5 * Wnt1 up vs E11.5 * Ctrl
10494595	Notch2	NM_010928	1.59858e-005	1.62194	E11.5 * Wnt1 up vs E11.5 * Ctrl
10456522	Tcf4	NM_013685	4.67645e-009	1.62184	E11.5 * Wnt1 up vs E11.5 * Ctrl
10594048	Islr2	NM_177193	0.000122374	1.62084	E11.5 * Wnt1 up vs E11.5 * Ctrl
10380341	Spag9	NM_027569	3.48418e-008	1.62036	E11.5 * Wnt1 up vs E11.5 * Ctrl
10373680	Neurod4	NM_007501	0.00192516	1.61977	E11.5 * Wnt1 up vs E11.5 * Ctrl
10464583	Gstp1	NM_013541	2.93781e-005	1.61955	E11.5 * Wnt1 up vs E11.5 * Ctrl
10535449	E130309D02Rik	NM_172726	4.06749e-005	1.61759	E11.5 * Wnt1 up vs E11.5 * Ctrl
10435271	Heg1	NM_175256	3.268e-007	1.61746	E11.5 * Wnt1 up vs E11.5 * Ctrl
10404763	Tmem170b	XM_886379	7.83102e-006	1.61614	E11.5 * Wnt1 up vs E11.5 * Ctrl
10368101	D10Bwg1379e	NM_001033258	1.42412e-005	1.61534	E11.5 * Wnt1 up vs E11.5 * Ctrl
10379795	Ap1gbp1	NM_001115009	1.74169e-006	1.61497	E11.5 * Wnt1 up vs E11.5 * Ctrl
10415784	Trim13	NM_023233	0.00451344	1.61455	E11.5 * Wnt1 up vs E11.5 * Ctrl
10532857	Gltp	NM_019821	1.1199e-005	1.6129	E11.5 * Wnt1 up vs E11.5 * Ctrl
10424781	Grina	NM_023168	2.60815e-005	1.61247	E11.5 * Wnt1 up vs E11.5 * Ctrl
10558481	Dpysl4	NM_011993	5.04805e-005	1.61242	E11.5 * Wnt1 up vs E11.5 * Ctrl
10596148	Trf	NM_133977	1.09267e-005	1.61146	E11.5 * Wnt1 up vs E11.5 * Ctrl
10358603	Hmcn1	NM_001024720	0.000147309	1.61135	E11.5 * Wnt1 up vs E11.5 * Ctrl
10600210	Slc6a8	NM_133987	3.04947e-005	1.61099	E11.5 * Wnt1 up vs E11.5 * Ctrl
10462035	Ldhb	NM_008492	2.86656e-007	1.61038	E11.5 * Wnt1 up vs E11.5 * Ctrl
10358565	Hmcn1	NM_001024720	0.000667735	1.61012	E11.5 * Wnt1 up vs E11.5 * Ctrl
10494735	Gdap2	NM_010269	4.83142e-005	1.6086	E11.5 * Wnt1 up vs E11.5 * Ctrl
10483624	Dlx1as	NR_002854	0.000236524	1.60789	E11.5 * Wnt1 up vs E11.5 * Ctrl
10378870	Git1	NM_001004144	0.000285867	1.60728	E11.5 * Wnt1 up vs E11.5 * Ctrl
10358654	Hmcn1	NM_001024720	1.97281e-005	1.60681	E11.5 * Wnt1 up vs E11.5 * Ctrl
10453518	Nrxn1	NM_020252	0.000651777	1.60676	E11.5 * Wnt1 up vs E11.5 * Ctrl
10488387	Napb	NM_019632	1.00986e-005	1.60642	E11.5 * Wnt1 up vs E11.5 * Ctrl
10497996	Ikzf5	NM_175115	6.69004e-006	1.60641	E11.5 * Wnt1 up vs E11.5 * Ctrl
10587107	Myo5a	NM_010864	5.23933e-009	1.6062	E11.5 * Wnt1 up vs E11.5 * Ctrl
10467139	Lipa	NM_021460	1.7559e-006	1.60417	E11.5 * Wnt1 up vs E11.5 * Ctrl
10459329	2700046A07Rik	ENSMUST00000	0.000351215	1.60391	E11.5 * Wnt1 up vs E11.5 * Ctrl
10499168	Kirrel	NM_130867	0.000508303	1.60243	E11.5 * Wnt1 up vs E11.5 * Ctrl
10416541	Enox1	NM_172813	3.22681e-007	1.60217	E11.5 * Wnt1 up vs E11.5 * Ctrl
10451838	Slc5a7	NM_022025	0.000672206	1.60103	E11.5 * Wnt1 up vs E11.5 * Ctrl
10434758	St6gal1	NM_145933	4.85229e-007	1.60038	E11.5 * Wnt1 up vs E11.5 * Ctrl
10603316	2010204K13Rik	AF038507	1.49142e-005	1.60012	E11.5 * Wnt1 up vs E11.5 * Ctrl
10490551	Nkain4	NM_021426	1.83607e-005	1.59951	E11.5 * Wnt1 up vs E11.5 * Ctrl
10361807	Hivep2	NM_010437	0.00040939	1.59787	E11.5 * Wnt1 up vs E11.5 * Ctrl
10551065	D930028M14Rik	ENSMUST00000	2.7742e-005	1.5974	E11.5 * Wnt1 up vs E11.5 * Ctrl
10500304	Vps45	NM_013841	0.00666198	1.59675	E11.5 * Wnt1 up vs E11.5 * Ctrl
10575685	Nudt7	NM_024437	0.00182043	1.59523	E11.5 * Wnt1 up vs E11.5 * Ctrl
10549842	Zfp667	NM_001024928	6.88248e-005	1.59468	E11.5 * Wnt1 up vs E11.5 * Ctrl
10564262	Mkrn3	NM_011746	0.00142595	1.59329	E11.5 * Wnt1 up vs E11.5 * Ctrl
10358571	Hmcn1	NM_001024720	0.000199286	1.59318	E11.5 * Wnt1 up vs E11.5 * Ctrl
10358583	Hmcn1	NM_001024720	0.000128507	1.59264	E11.5 * Wnt1 up vs E11.5 * Ctrl
10358559	Hmcn1	NM_001024720	8.02128e-006	1.59086	E11.5 * Wnt1 up vs E11.5 * Ctrl
10538290	Snx10	NM_028035	1.18798e-005	1.58926	E11.5 * Wnt1 up vs E11.5 * Ctrl
10479948	Cugbp2	NM_001110228	0.002377	1.58851	E11.5 * Wnt1 up vs E11.5 * Ctrl
10403413	Idi1	NM_145360	0.00803013	1.58765	E11.5 * Wnt1 up vs E11.5 * Ctrl
10495343	Wdr47	NM_181400	0.000487007	1.5876	E11.5 * Wnt1 up vs E11.5 * Ctrl
10430389	Mfng	NM_008595	1.34843e-005	1.58643	E11.5 * Wnt1 up vs E11.5 * Ctrl
10359624	Prrx1	NM_175686	0.00471733	1.58504	E11.5 * Wnt1 up vs E11.5 * Ctrl
10378833	Ssh2	NM_177710	3.55997e-006	1.5844	E11.5 * Wnt1 up vs E11.5 * Ctrl
10581266	Tppp3	NM_026481	2.66719e-005	1.58368	E11.5 * Wnt1 up vs E11.5 * Ctrl
10391513	Dusp3	NM_028207	0.00121079	1.58352	E11.5 * Wnt1 up vs E11.5 * Ctrl
10569129	Sct	NM_011328	0.000127036	1.58342	E11.5 * Wnt1 up vs E11.5 * Ctrl
10512308	Sigmar1	NM_011014	0.000166383	1.58303	E11.5 * Wnt1 up vs E11.5 * Ctrl
10561335	Prkcz	NM_008860	0.00229943	1.58219	E11.5 * Wnt1 up vs E11.5 * Ctrl

10520650	Cad	NM_023525	4.82694e-005	1.58151	E11.5 * Wnt1 up vs E11.5 * Ctrl
10451167	Tmem63b	NM_198167	5.3187e-005	1.58144	E11.5 * Wnt1 up vs E11.5 * Ctrl
10472289	Tank	NM_011529	5.12393e-005	1.58082	E11.5 * Wnt1 up vs E11.5 * Ctrl
10535017	Gal3st4	NM_001033416	4.15112e-006	1.58014	E11.5 * Wnt1 up vs E11.5 * Ctrl
10358591	Hmcn1	NM_001024720	0.000144931	1.57996	E11.5 * Wnt1 up vs E11.5 * Ctrl
10358615	Hmcn1	NM_001024720	0.000190152	1.57891	E11.5 * Wnt1 up vs E11.5 * Ctrl
10465395	Ppp2r5b	NM_198168	0.0049272	1.57887	E11.5 * Wnt1 up vs E11.5 * Ctrl
10469322	Vim	NM_011701	3.2627e-009	1.57865	E11.5 * Wnt1 up vs E11.5 * Ctrl
10527148	Slc29a4	NM_146257	9.25388e-007	1.57836	E11.5 * Wnt1 up vs E11.5 * Ctrl
10455238	Ndfip1	NM_022996	2.88995e-008	1.5779	E11.5 * Wnt1 up vs E11.5 * Ctrl
10382341	Sstr2	NM_009217	0.00381862	1.5775	E11.5 * Wnt1 up vs E11.5 * Ctrl
10397450	Vash1	NM_177354	2.73778e-005	1.57706	E11.5 * Wnt1 up vs E11.5 * Ctrl
10470027	Npdc1	NM_008721	7.42e-005	1.57636	E11.5 * Wnt1 up vs E11.5 * Ctrl
10395394	4930579E17Rik	NM_178629	0.000381131	1.57571	E11.5 * Wnt1 up vs E11.5 * Ctrl
10501358	Sars	NM_011319	3.18165e-007	1.5751	E11.5 * Wnt1 up vs E11.5 * Ctrl
10398936	Pacs2	NM_001081170	8.23192e-005	1.57489	E11.5 * Wnt1 up vs E11.5 * Ctrl
10482762	Idi1	NM_145360	0.00332698	1.57451	E11.5 * Wnt1 up vs E11.5 * Ctrl
10507273	Pik3r3	NM_181585	1.42562e-008	1.57409	E11.5 * Wnt1 up vs E11.5 * Ctrl
10412066	Rab3c	NM_023852	0.000315747	1.57215	E11.5 * Wnt1 up vs E11.5 * Ctrl
10355916	Pax3	NM_008781	1.24237e-006	1.57191	E11.5 * Wnt1 up vs E11.5 * Ctrl
10345715	Map4k4	NM_008696	6.38438e-007	1.57172	E11.5 * Wnt1 up vs E11.5 * Ctrl
10435752	Lsamp	NM_175548	2.7809e-006	1.57157	E11.5 * Wnt1 up vs E11.5 * Ctrl
10389719	Scpep1	NM_029023	8.60865e-007	1.57097	E11.5 * Wnt1 up vs E11.5 * Ctrl
10595836	E030011O05Rik	ENSMUST00000	4.98073e-005	1.57096	E11.5 * Wnt1 up vs E11.5 * Ctrl
10368227	Ube2q2	NM_180600	1.01748e-005	1.57081	E11.5 * Wnt1 up vs E11.5 * Ctrl
10427877	ENSMUSG0000C	AK157781	0.00042565	1.57067	E11.5 * Wnt1 up vs E11.5 * Ctrl
10371092	Atcay	NM_178662	0.000872668	1.57064	E11.5 * Wnt1 up vs E11.5 * Ctrl
10385361	Ublcp1	NM_024475	0.00573268	1.56987	E11.5 * Wnt1 up vs E11.5 * Ctrl
10570180	EG434280	ENSMUST00000	2.28475e-005	1.56939	E11.5 * Wnt1 up vs E11.5 * Ctrl
10412076	Gemin8	NM_146238	5.99224e-005	1.5693	E11.5 * Wnt1 up vs E11.5 * Ctrl
10358523	Hmcn1	NM_001024720	9.74542e-005	1.56864	E11.5 * Wnt1 up vs E11.5 * Ctrl
10492590	Ppm1l	NM_178726	8.60953e-005	1.56839	E11.5 * Wnt1 up vs E11.5 * Ctrl
10493519	Shc1	NM_001113331	0.00426676	1.56754	E11.5 * Wnt1 up vs E11.5 * Ctrl
10467730	Morn4	NM_198108	0.00558337	1.56748	E11.5 * Wnt1 up vs E11.5 * Ctrl
10568529	Ikzf5	NM_175115	0.00115156	1.56737	E11.5 * Wnt1 up vs E11.5 * Ctrl
10396740	Gphn	NM_172952	1.33096e-005	1.56641	E11.5 * Wnt1 up vs E11.5 * Ctrl
10450212	Egfl8	NM_152922	3.8407e-007	1.5662	E11.5 * Wnt1 up vs E11.5 * Ctrl
10468200	Cuedc2	NM_024192	1.00967e-006	1.56535	E11.5 * Wnt1 up vs E11.5 * Ctrl
10425293	Cby1	NM_028634	5.81413e-007	1.565	E11.5 * Wnt1 up vs E11.5 * Ctrl
10487748	4930402H24Rik	BC052447	4.86376e-007	1.56314	E11.5 * Wnt1 up vs E11.5 * Ctrl
10464586	Gstp2	NM_181796	1.08894e-005	1.56314	E11.5 * Wnt1 up vs E11.5 * Ctrl
10358585	Hmcn1	NM_001024720	0.00018964	1.5613	E11.5 * Wnt1 up vs E11.5 * Ctrl
10585494	Ube2q2	NM_180600	4.34176e-006	1.56127	E11.5 * Wnt1 up vs E11.5 * Ctrl
10393970	Fasn	NM_007988	0.00221314	1.5612	E11.5 * Wnt1 up vs E11.5 * Ctrl
10448034	Dll1	NM_007865	4.54152e-005	1.55928	E11.5 * Wnt1 up vs E11.5 * Ctrl
10541862	Iffo1	NM_178787	0.000338973	1.55905	E11.5 * Wnt1 up vs E11.5 * Ctrl
10495625	Dpyd	NM_170778	0.000230758	1.55903	E11.5 * Wnt1 up vs E11.5 * Ctrl
10578222	Dlc1	NM_015802	3.04116e-005	1.55885	E11.5 * Wnt1 up vs E11.5 * Ctrl
10385236	Akr1b3	NM_009658	3.20206e-007	1.55839	E11.5 * Wnt1 up vs E11.5 * Ctrl
10451580	Bysl	NM_016859	1.38787e-005	1.55818	E11.5 * Wnt1 up vs E11.5 * Ctrl
10400006	Ahr	NM_013464	5.34878e-006	1.55795	E11.5 * Wnt1 up vs E11.5 * Ctrl
10359235	Rasal2	NM_177644	4.66893e-008	1.5578	E11.5 * Wnt1 up vs E11.5 * Ctrl
10388884	Nlk	NM_008702	3.28619e-007	1.55677	E11.5 * Wnt1 up vs E11.5 * Ctrl
10543466	Gpr37	NM_010338	0.00122698	1.55669	E11.5 * Wnt1 up vs E11.5 * Ctrl
10345350		---	1.2229e-005	1.55589	E11.5 * Wnt1 up vs E11.5 * Ctrl
10499108	Glt28d2	NM_177130	0.000168134	1.55583	E11.5 * Wnt1 up vs E11.5 * Ctrl
10467425	Sorbs1	NM_178362	1.93667e-006	1.55506	E11.5 * Wnt1 up vs E11.5 * Ctrl
10358573	Hmcn1	NM_001024720	0.000313059	1.55373	E11.5 * Wnt1 up vs E11.5 * Ctrl
10527229	2810453I06Rik	NM_026050	2.11859e-005	1.55359	E11.5 * Wnt1 up vs E11.5 * Ctrl
10384985	Rhbdf1	NM_010117	7.45793e-005	1.55286	E11.5 * Wnt1 up vs E11.5 * Ctrl
10456587	Mro	NM_027741	3.92406e-005	1.55243	E11.5 * Wnt1 up vs E11.5 * Ctrl
10523058	Eif5a	NM_181582	0.000184402	1.55215	E11.5 * Wnt1 up vs E11.5 * Ctrl
10559146	Brsk2	NM_029426	3.57696e-005	1.55139	E11.5 * Wnt1 up vs E11.5 * Ctrl
10593966	Csk	NM_007783	0.00170471	1.551	E11.5 * Wnt1 up vs E11.5 * Ctrl
10350951	Tnr	NM_022312	0.000195048	1.55052	E11.5 * Wnt1 up vs E11.5 * Ctrl
10565910	Plekhb1	NM_013746	0.000548765	1.55051	E11.5 * Wnt1 up vs E11.5 * Ctrl

10348653	Gpc1	NM_016696	5.81351e-005	1.55018	E11.5 * Wnt1 up vs E11.5 * Ctrl
10589130	Celsr3	NM_080437	5.67648e-005	1.54868	E11.5 * Wnt1 up vs E11.5 * Ctrl
10511725	Cyb5r4	NM_024195	6.51313e-006	1.54856	E11.5 * Wnt1 up vs E11.5 * Ctrl
10358664	Hmcn1	NM_001024720	1.54957e-005	1.54819	E11.5 * Wnt1 up vs E11.5 * Ctrl
10352815	Irf6	NM_016851	8.58164e-005	1.54663	E11.5 * Wnt1 up vs E11.5 * Ctrl
10358545	Hmcn1	NM_001024720	0.00202455	1.54595	E11.5 * Wnt1 up vs E11.5 * Ctrl
10373388	Obfc2b	NM_027257	0.000221621	1.54594	E11.5 * Wnt1 up vs E11.5 * Ctrl
10360479	Cep170	NM_001099637	6.64897e-007	1.54546	E11.5 * Wnt1 up vs E11.5 * Ctrl
10372891	Srgap1	NM_001081037	6.2045e-006	1.54495	E11.5 * Wnt1 up vs E11.5 * Ctrl
10361152	Gstp2	NM_181796	8.87335e-006	1.54364	E11.5 * Wnt1 up vs E11.5 * Ctrl
10423293	Myo10	NM_019472	5.7075e-005	1.54286	E11.5 * Wnt1 up vs E11.5 * Ctrl
10418198	D14Ertd449e	NM_025311	0.000111287	1.5412	E11.5 * Wnt1 up vs E11.5 * Ctrl
10358563	Hmcn1	NM_001024720	9.70318e-005	1.5411	E11.5 * Wnt1 up vs E11.5 * Ctrl
10588037	Rbp1	NM_011254	1.23934e-006	1.541	E11.5 * Wnt1 up vs E11.5 * Ctrl
10431154	Phf21b	NM_001081166	2.91042e-005	1.54054	E11.5 * Wnt1 up vs E11.5 * Ctrl
10472436	B3galt1	NM_020283	0.000138119	1.5398	E11.5 * Wnt1 up vs E11.5 * Ctrl
10509838	Padi2	NM_008812	0.00177784	1.53916	E11.5 * Wnt1 up vs E11.5 * Ctrl
10426835	Dip2b	NM_001159361	1.53006e-006	1.53913	E11.5 * Wnt1 up vs E11.5 * Ctrl
10497673	Zmat3	NM_009517	0.000214954	1.53864	E11.5 * Wnt1 up vs E11.5 * Ctrl
10418185	D14Ertd449e	NM_025311	0.000156131	1.53746	E11.5 * Wnt1 up vs E11.5 * Ctrl
10604961	Gabra3	NM_008067	7.87011e-005	1.53741	E11.5 * Wnt1 up vs E11.5 * Ctrl
10504692	Tmod1	NM_021883	0.00025692	1.53726	E11.5 * Wnt1 up vs E11.5 * Ctrl
10469936	Nrarp	NM_025980	4.30128e-006	1.53716	E11.5 * Wnt1 up vs E11.5 * Ctrl
10593225	Zbtb16	NM_001033324	0.000102263	1.53688	E11.5 * Wnt1 up vs E11.5 * Ctrl
10347970	Cab39	NM_133781	1.5701e-007	1.53687	E11.5 * Wnt1 up vs E11.5 * Ctrl
10495659	Cnn3	NM_028044	3.39004e-008	1.53639	E11.5 * Wnt1 up vs E11.5 * Ctrl
10373964	Thoc5	NM_172438	3.20655e-005	1.53571	E11.5 * Wnt1 up vs E11.5 * Ctrl
10423503	EG625796	XM_890363	0.00482374	1.53543	E11.5 * Wnt1 up vs E11.5 * Ctrl
10603000	Rai2	NM_198409	0.00406482	1.53536	E11.5 * Wnt1 up vs E11.5 * Ctrl
10371319	ENSMUSG00000	ENSMUST00000	0.00264278	1.53393	E11.5 * Wnt1 up vs E11.5 * Ctrl
10423471	Ctnnd2	NM_008729	5.14283e-005	1.53367	E11.5 * Wnt1 up vs E11.5 * Ctrl
10463138	Dppa2	NM_028615	0.000444769	1.53314	E11.5 * Wnt1 up vs E11.5 * Ctrl
10538082	Atp6v0e2	NM_133764	7.42122e-005	1.53235	E11.5 * Wnt1 up vs E11.5 * Ctrl
10499643	Chrnb2	NM_009602	0.000121634	1.53172	E11.5 * Wnt1 up vs E11.5 * Ctrl
10420787	Mtmr9	NM_177594	2.59132e-005	1.53109	E11.5 * Wnt1 up vs E11.5 * Ctrl
10398996	Crip2	NM_024223	0.00188176	1.5308	E11.5 * Wnt1 up vs E11.5 * Ctrl
10405211	Gadd45g	NM_011817	0.00156158	1.53075	E11.5 * Wnt1 up vs E11.5 * Ctrl
10528143	Ppp1r14b	NM_008889	6.11375e-008	1.53074	E11.5 * Wnt1 up vs E11.5 * Ctrl
10573128	Tbc1d9	NM_001111304	4.86463e-009	1.53026	E11.5 * Wnt1 up vs E11.5 * Ctrl
10534456	Hip1	NM_146001	8.38788e-005	1.52974	E11.5 * Wnt1 up vs E11.5 * Ctrl
10362829	Ostm1	NM_172416	4.83045e-005	1.52905	E11.5 * Wnt1 up vs E11.5 * Ctrl
10550400	Pnmal2	NM_001099636	0.00011896	1.52892	E11.5 * Wnt1 up vs E11.5 * Ctrl
10524941	Fbxo21	NM_145564	1.5993e-005	1.52854	E11.5 * Wnt1 up vs E11.5 * Ctrl
10468217	Actr1a	NM_016860	0.000104162	1.52702	E11.5 * Wnt1 up vs E11.5 * Ctrl
10358569	Hmcn1	NM_001024720	0.00042008	1.52665	E11.5 * Wnt1 up vs E11.5 * Ctrl
10465521	Plcb3	NM_008874	7.42495e-006	1.52608	E11.5 * Wnt1 up vs E11.5 * Ctrl
10595805	Rasa2	NM_053268	1.51995e-005	1.52529	E11.5 * Wnt1 up vs E11.5 * Ctrl
10506714	Lrp8	NM_053073	4.79959e-005	1.52515	E11.5 * Wnt1 up vs E11.5 * Ctrl
10353117	Slco5a1	NM_172841	4.81245e-005	1.5243	E11.5 * Wnt1 up vs E11.5 * Ctrl
10482059	Ggta1	NM_010283	0.000401517	1.52417	E11.5 * Wnt1 up vs E11.5 * Ctrl
10490838	Fabp5	NM_010634	1.75014e-006	1.52411	E11.5 * Wnt1 up vs E11.5 * Ctrl
10451665	Apobec2	NM_009694	0.000751664	1.5241	E11.5 * Wnt1 up vs E11.5 * Ctrl
10579872	Tpd52	NM_001025263	0.000234025	1.52405	E11.5 * Wnt1 up vs E11.5 * Ctrl
10605465	Prkx	NM_016979	0.00129611	1.5239	E11.5 * Wnt1 up vs E11.5 * Ctrl
10450622	2610110G12Rik	NM_001142744	0.00150366	1.52306	E11.5 * Wnt1 up vs E11.5 * Ctrl
10386636	Usp22	NM_001004143	2.86884e-007	1.52222	E11.5 * Wnt1 up vs E11.5 * Ctrl
10391454	Vat1	NM_012037	0.000525438	1.52205	E11.5 * Wnt1 up vs E11.5 * Ctrl
10604637	Cxx1b	NM_001018063	1.0906e-007	1.52201	E11.5 * Wnt1 up vs E11.5 * Ctrl
10489660	Elmo2	NM_207706	0.000218298	1.52174	E11.5 * Wnt1 up vs E11.5 * Ctrl
10593245	Htr3b	NM_020274	0.0011948	1.52125	E11.5 * Wnt1 up vs E11.5 * Ctrl
10472162	Gpd2	NM_010274	3.71355e-005	1.52099	E11.5 * Wnt1 up vs E11.5 * Ctrl
10359851	Uck2	NM_030724	2.80489e-005	1.52091	E11.5 * Wnt1 up vs E11.5 * Ctrl
10346250	Mstn	NM_010834	8.11868e-006	1.52044	E11.5 * Wnt1 up vs E11.5 * Ctrl
10444420	Agpat1	NM_018862	0.000114579	1.52003	E11.5 * Wnt1 up vs E11.5 * Ctrl
10354141	Lonrf2	NM_001029878	0.00360828	1.51978	E11.5 * Wnt1 up vs E11.5 * Ctrl
10550316	Tmem160	NM_026938	7.41702e-006	1.51944	E11.5 * Wnt1 up vs E11.5 * Ctrl

10389391	OTTMUSG00000	ENSMUST00000	0.00121051	1.51923	E11.5 * Wnt1 up vs E11.5 * Ctrl
10451054	Enpp4	NM_199016	0.000235533	1.51864	E11.5 * Wnt1 up vs E11.5 * Ctrl
10581547	Nob1	NM_026277	4.42084e-005	1.51804	E11.5 * Wnt1 up vs E11.5 * Ctrl
10457733	B4galt6	NM_019737	1.36405e-005	1.51801	E11.5 * Wnt1 up vs E11.5 * Ctrl
10418210	D14Ertd449e	NM_025311	0.000146933	1.51801	E11.5 * Wnt1 up vs E11.5 * Ctrl
10444098	Wdr46	NM_020603	5.82938e-005	1.51759	E11.5 * Wnt1 up vs E11.5 * Ctrl
10518428	Clcn6	NM_011929	0.00131903	1.51709	E11.5 * Wnt1 up vs E11.5 * Ctrl
10573115	Rnf150	NM_177378	2.7304e-005	1.51694	E11.5 * Wnt1 up vs E11.5 * Ctrl
10543249	EG232599	ENSMUST00000	0.00434201	1.51676	E11.5 * Wnt1 up vs E11.5 * Ctrl
10600390	Gdi1	NM_010273	1.35881e-005	1.51599	E11.5 * Wnt1 up vs E11.5 * Ctrl
10377215	Gas7	NM_008088	1.26237e-006	1.51542	E11.5 * Wnt1 up vs E11.5 * Ctrl
10587315	Gsta4	NM_010357	2.58416e-005	1.51534	E11.5 * Wnt1 up vs E11.5 * Ctrl
10581493	Pdxp	NM_020271	0.000314586	1.51533	E11.5 * Wnt1 up vs E11.5 * Ctrl
10538519	Gsbs	NM_011153	0.00100102	1.51432	E11.5 * Wnt1 up vs E11.5 * Ctrl
10388869	Tnfaip1	NM_009395	0.000128782	1.51426	E11.5 * Wnt1 up vs E11.5 * Ctrl
10526977	Eif3b	NM_133916	0.000114097	1.51324	E11.5 * Wnt1 up vs E11.5 * Ctrl
10570437	Fbxo25	NM_025785	4.74707e-005	1.51101	E11.5 * Wnt1 up vs E11.5 * Ctrl
10585905	Parp6	NM_029922	4.51962e-006	1.51061	E11.5 * Wnt1 up vs E11.5 * Ctrl
10442224	BC049807	BC076634	0.000534839	1.51061	E11.5 * Wnt1 up vs E11.5 * Ctrl
10570000	Gpi1	NM_008155	0.00145097	1.51049	E11.5 * Wnt1 up vs E11.5 * Ctrl
10508907	Lin28	NM_145833	0.00617022	1.51047	E11.5 * Wnt1 up vs E11.5 * Ctrl
10376021	08-Sep	NM_033144	2.48558e-005	1.50968	E11.5 * Wnt1 up vs E11.5 * Ctrl
10367122	Baz2a	NM_054078	3.16845e-008	1.50951	E11.5 * Wnt1 up vs E11.5 * Ctrl
10358662	Hmcn1	NM_001024720	7.40849e-005	1.50841	E11.5 * Wnt1 up vs E11.5 * Ctrl
10476355	Chgb	NM_007694	0.00302499	1.506	E11.5 * Wnt1 up vs E11.5 * Ctrl
10497321	Pgam1	NM_023418	6.6659e-006	1.50573	E11.5 * Wnt1 up vs E11.5 * Ctrl
10571907	Mfap3l	NM_027756	9.23468e-005	1.50493	E11.5 * Wnt1 up vs E11.5 * Ctrl
10470834	Spna2	NM_001076554	6.53307e-006	1.50436	E11.5 * Wnt1 up vs E11.5 * Ctrl
10401181	Rdh11	NM_021557	2.80063e-005	1.50391	E11.5 * Wnt1 up vs E11.5 * Ctrl
10400395	Ppp2r3c	NM_021529	0.00199843	1.50373	E11.5 * Wnt1 up vs E11.5 * Ctrl
10358561	Hmcn1	NM_001024720	0.00102197	1.50303	E11.5 * Wnt1 up vs E11.5 * Ctrl
10390635	1810046J19Rik	NM_025559	0.00011926	1.50271	E11.5 * Wnt1 up vs E11.5 * Ctrl
10425267	Pick1	NM_008837	9.22948e-005	1.50092	E11.5 * Wnt1 up vs E11.5 * Ctrl
10358543	Hmcn1	NM_001024720	0.00199736	1.50081	E11.5 * Wnt1 up vs E11.5 * Ctrl
10379543	Tmem132e	NM_023438	0.000488685	1.49972	E11.5 * Wnt1 up vs E11.5 * Ctrl
10534531	Ywhag	NM_018871	0.000482718	1.49966	E11.5 * Wnt1 up vs E11.5 * Ctrl
10464572	Ndufv1	NM_133666	0.000803123	1.49921	E11.5 * Wnt1 up vs E11.5 * Ctrl
10585214	Cryab	NM_009964	0.00285648	1.49892	E11.5 * Wnt1 up vs E11.5 * Ctrl
10511881	Manea	NM_172865	4.65049e-005	1.49851	E11.5 * Wnt1 up vs E11.5 * Ctrl
10509163	Id3	NM_008321	0.000467048	1.49794	E11.5 * Wnt1 up vs E11.5 * Ctrl
10528484	Srpk2	NM_009274	2.08493e-006	1.49785	E11.5 * Wnt1 up vs E11.5 * Ctrl
10447354	Txndc14	NM_025868	7.86096e-005	1.49775	E11.5 * Wnt1 up vs E11.5 * Ctrl
10461022	Ppp1r14b	NM_008889	1.5414e-008	1.49774	E11.5 * Wnt1 up vs E11.5 * Ctrl
10597883	Ano10	NM_133979	0.00384432	1.49761	E11.5 * Wnt1 up vs E11.5 * Ctrl
10434934	Bdh1	NM_175177	0.000326045	1.49755	E11.5 * Wnt1 up vs E11.5 * Ctrl
10596543	Rad54l2	NM_030730	0.00177059	1.49732	E11.5 * Wnt1 up vs E11.5 * Ctrl
10352554		---	0.000703214	1.49732	E11.5 * Wnt1 up vs E11.5 * Ctrl
10418766	Ankrd28	NM_001024604	6.17298e-005	1.49707	E11.5 * Wnt1 up vs E11.5 * Ctrl
10374068	Dbnl	NM_001146308	0.00253491	1.49637	E11.5 * Wnt1 up vs E11.5 * Ctrl
10407803	Gpr137b	NM_031999	0.00445749	1.49633	E11.5 * Wnt1 up vs E11.5 * Ctrl
10376747	Kcnj12	NM_010603	0.000320927	1.49563	E11.5 * Wnt1 up vs E11.5 * Ctrl
10571214	Rnf122	NM_175136	0.000502597	1.49542	E11.5 * Wnt1 up vs E11.5 * Ctrl
10533633	Diablo	NM_023232	8.40067e-007	1.49521	E11.5 * Wnt1 up vs E11.5 * Ctrl
10358658	Hmcn1	NM_001024720	9.54173e-005	1.49498	E11.5 * Wnt1 up vs E11.5 * Ctrl
10516064	Mfsd2	NM_029662	0.00188702	1.49485	E11.5 * Wnt1 up vs E11.5 * Ctrl
10412100	Map3k1	NM_011945	0.00022053	1.49482	E11.5 * Wnt1 up vs E11.5 * Ctrl
10354472	Gls	NM_001081081	0.00255265	1.49407	E11.5 * Wnt1 up vs E11.5 * Ctrl
10448803	Hn1l	NM_198937	1.8068e-006	1.494	E11.5 * Wnt1 up vs E11.5 * Ctrl
10434366	Dvl3	NM_007889	0.00813506	1.49294	E11.5 * Wnt1 up vs E11.5 * Ctrl
10445241	Tnfrsf21	NM_178589	5.07311e-005	1.49223	E11.5 * Wnt1 up vs E11.5 * Ctrl
10410124	Ctsl	NM_009984	1.55028e-007	1.49208	E11.5 * Wnt1 up vs E11.5 * Ctrl
10358567	Hmcn1	NM_001024720	0.000316394	1.49196	E11.5 * Wnt1 up vs E11.5 * Ctrl
10358593	Hmcn1	NM_001024720	0.000759636	1.49171	E11.5 * Wnt1 up vs E11.5 * Ctrl
10398727	Klc1	NM_001025360	0.000154562	1.49166	E11.5 * Wnt1 up vs E11.5 * Ctrl
10352119	Pppde1	NM_024282	4.06792e-006	1.49162	E11.5 * Wnt1 up vs E11.5 * Ctrl
10485151	Mapk8ip1	NM_011162	0.000222136	1.49127	E11.5 * Wnt1 up vs E11.5 * Ctrl

10481368 Trub2	NM_145520	7.09448e-005	1.49107	E11.5 * Wnt1 up vs E11.5 * Ctrl
10558948 Cd151	NM_009842	0.000991745	1.49009	E11.5 * Wnt1 up vs E11.5 * Ctrl
10445338 Enpp5	NM_032003	0.000370081	1.48948	E11.5 * Wnt1 up vs E11.5 * Ctrl
10555205 Gdpd5	NM_201352	0.000469711	1.48917	E11.5 * Wnt1 up vs E11.5 * Ctrl
10509777 Iffo2	NM_183148	0.000102	1.48898	E11.5 * Wnt1 up vs E11.5 * Ctrl
10598359 Syp	NM_009305	8.37909e-005	1.48849	E11.5 * Wnt1 up vs E11.5 * Ctrl
10479230 Phactr3	NM_028806	0.000928587	1.48738	E11.5 * Wnt1 up vs E11.5 * Ctrl
10606174 Nap1l2	NM_008671	0.00676623	1.48643	E11.5 * Wnt1 up vs E11.5 * Ctrl
10484431 Txndc14	NM_025868	6.8752e-005	1.48641	E11.5 * Wnt1 up vs E11.5 * Ctrl
10367734 Ust	NM_177387	0.000216786	1.48526	E11.5 * Wnt1 up vs E11.5 * Ctrl
10530201 Ugdh	NM_009466	7.341e-005	1.48453	E11.5 * Wnt1 up vs E11.5 * Ctrl
10443383 Tmhs	NM_026571	0.00308303	1.48441	E11.5 * Wnt1 up vs E11.5 * Ctrl
10488589 Fam110a	NM_028666	9.30693e-005	1.48371	E11.5 * Wnt1 up vs E11.5 * Ctrl
10594631 Aph1b	NM_177583	2.15322e-005	1.48362	E11.5 * Wnt1 up vs E11.5 * Ctrl
10348580 Klhl30	NM_027551	0.000248301	1.48262	E11.5 * Wnt1 up vs E11.5 * Ctrl
10394625	---	0.00242491	1.48195	E11.5 * Wnt1 up vs E11.5 * Ctrl
10484894 Ptprj	NM_008982	0.00047822	1.48189	E11.5 * Wnt1 up vs E11.5 * Ctrl
10460517 Brms1	NM_134155	0.000740463	1.48176	E11.5 * Wnt1 up vs E11.5 * Ctrl
10363905 Zwint	NM_025635	1.92437e-007	1.4817	E11.5 * Wnt1 up vs E11.5 * Ctrl
10437590 Carhsp1	NM_025821	8.34461e-007	1.48149	E11.5 * Wnt1 up vs E11.5 * Ctrl
10480492 Cacna1b	NM_001042528	1.30018e-006	1.48148	E11.5 * Wnt1 up vs E11.5 * Ctrl
10434348 Eif2b5	NM_172265	2.72159e-005	1.48126	E11.5 * Wnt1 up vs E11.5 * Ctrl
10581289 Atp6v0d1	NM_013477	0.000576634	1.48091	E11.5 * Wnt1 up vs E11.5 * Ctrl
10452613 Arhgap28	NM_172964	1.89842e-006	1.48069	E11.5 * Wnt1 up vs E11.5 * Ctrl
10402585 Wars	NM_011710	2.1516e-005	1.48037	E11.5 * Wnt1 up vs E11.5 * Ctrl
10409767 Golm1	NM_027307	0.000226539	1.47953	E11.5 * Wnt1 up vs E11.5 * Ctrl
10521709 Lap3	NM_024434	1.13401e-005	1.47951	E11.5 * Wnt1 up vs E11.5 * Ctrl
10388898 1810009O10Rik	BC027022	0.00650419	1.47943	E11.5 * Wnt1 up vs E11.5 * Ctrl
10571567 Sorbs2	NM_172752	3.02294e-006	1.47907	E11.5 * Wnt1 up vs E11.5 * Ctrl
10524703 Pxn	NM_011223	6.12144e-005	1.4787	E11.5 * Wnt1 up vs E11.5 * Ctrl
10449971 Zfp763	NM_028543	0.00425758	1.47786	E11.5 * Wnt1 up vs E11.5 * Ctrl
10390283 Cdk5rap3	NM_030248	0.000468294	1.47773	E11.5 * Wnt1 up vs E11.5 * Ctrl
10535273 Gna12	NM_010302	0.00045132	1.4777	E11.5 * Wnt1 up vs E11.5 * Ctrl
10506274 Dnajc6	NM_198412	1.11248e-005	1.47764	E11.5 * Wnt1 up vs E11.5 * Ctrl
10566730 Stk33	ENSMUST00000	0.000307675	1.47748	E11.5 * Wnt1 up vs E11.5 * Ctrl
10491300 Skil	NM_011386	1.89201e-005	1.47696	E11.5 * Wnt1 up vs E11.5 * Ctrl
10551852 Clip3	NM_001081114	0.00139888	1.47666	E11.5 * Wnt1 up vs E11.5 * Ctrl
10525439 P2rx4	NM_011026	0.00304149	1.47594	E11.5 * Wnt1 up vs E11.5 * Ctrl
10348087 Cops7b	NM_172974	0.00219259	1.47586	E11.5 * Wnt1 up vs E11.5 * Ctrl
10474112 Traf6	NM_009424	3.10289e-005	1.47496	E11.5 * Wnt1 up vs E11.5 * Ctrl
10519770 Pclo	NM_011995	0.000531173	1.47405	E11.5 * Wnt1 up vs E11.5 * Ctrl
10456836 St8sia5	NM_153124	0.000119494	1.47395	E11.5 * Wnt1 up vs E11.5 * Ctrl
10594590 Snx1	NM_019727	4.63084e-006	1.47355	E11.5 * Wnt1 up vs E11.5 * Ctrl
10456501 Rnmt	NM_026440	2.67604e-005	1.47302	E11.5 * Wnt1 up vs E11.5 * Ctrl
10447502 Adcyap1	NM_009625	0.00296526	1.47294	E11.5 * Wnt1 up vs E11.5 * Ctrl
10517425 Lypla2	NM_011942	0.00641824	1.47288	E11.5 * Wnt1 up vs E11.5 * Ctrl
10505911 Dmrta1	NM_175647	0.00154786	1.47254	E11.5 * Wnt1 up vs E11.5 * Ctrl
10518812 Camta1	NM_001081557	0.00021661	1.47244	E11.5 * Wnt1 up vs E11.5 * Ctrl
10430113 D15Wsu169e	BC060637	0.000614502	1.47199	E11.5 * Wnt1 up vs E11.5 * Ctrl
10438585 Tmem41a	NM_025693	0.00216659	1.47132	E11.5 * Wnt1 up vs E11.5 * Ctrl
10394158 Kif3c	NM_008445	0.000143349	1.47099	E11.5 * Wnt1 up vs E11.5 * Ctrl
10491962 Foxo1	NM_019739	8.46949e-006	1.4709	E11.5 * Wnt1 up vs E11.5 * Ctrl
10358589 Hmcn1	NM_001024720	0.00065084	1.4709	E11.5 * Wnt1 up vs E11.5 * Ctrl
10489694 Zfp334	NM_178411	0.000231466	1.4705	E11.5 * Wnt1 up vs E11.5 * Ctrl
10356020 Dock10	NM_175291	0.000865323	1.47039	E11.5 * Wnt1 up vs E11.5 * Ctrl
10429295 Kcnk9	NM_001033876	0.000123557	1.47024	E11.5 * Wnt1 up vs E11.5 * Ctrl
10373814 Pes1	NM_022889	0.000343142	1.46982	E11.5 * Wnt1 up vs E11.5 * Ctrl
10541845 Nop2	NM_138747	0.00441441	1.46951	E11.5 * Wnt1 up vs E11.5 * Ctrl
10400141 Zbed4	NM_181412	0.00195028	1.46875	E11.5 * Wnt1 up vs E11.5 * Ctrl
10368577 Rnf217	NM_001146349	0.000310234	1.46851	E11.5 * Wnt1 up vs E11.5 * Ctrl
10367641 Mthfd1l	NM_172308	2.49033e-007	1.4675	E11.5 * Wnt1 up vs E11.5 * Ctrl
10515613 Ptprf	NM_011213	0.00365965	1.46641	E11.5 * Wnt1 up vs E11.5 * Ctrl
10555118 Pak1	NM_011035	1.32471e-005	1.46624	E11.5 * Wnt1 up vs E11.5 * Ctrl
10361642 Lrp11	NM_172784	0.00376053	1.46554	E11.5 * Wnt1 up vs E11.5 * Ctrl
10565018 Iqgap1	NM_016721	4.99615e-006	1.46446	E11.5 * Wnt1 up vs E11.5 * Ctrl
10548163 Tulp3	NM_011657	7.85588e-005	1.46372	E11.5 * Wnt1 up vs E11.5 * Ctrl

10587085	BC031353	NM_001113283	2.7673e-005	1.46362	E11.5 * Wnt1 up vs E11.5 * Ctrl
10529549	Tbc1d14	NM_001113362	1.41551e-005	1.46247	E11.5 * Wnt1 up vs E11.5 * Ctrl
10536353	Dlx6	NM_010057	0.00181223	1.46232	E11.5 * Wnt1 up vs E11.5 * Ctrl
10409345	Cltb	NM_028870	0.000716371	1.46176	E11.5 * Wnt1 up vs E11.5 * Ctrl
10467979	Scd1	NM_009127	0.00310239	1.46152	E11.5 * Wnt1 up vs E11.5 * Ctrl
10401997	Ptpn21	NM_011877	1.80298e-005	1.46142	E11.5 * Wnt1 up vs E11.5 * Ctrl
10557156	Plk1	NM_011121	0.000212266	1.46132	E11.5 * Wnt1 up vs E11.5 * Ctrl
10594652	Lactb	NM_030717	0.00317728	1.46063	E11.5 * Wnt1 up vs E11.5 * Ctrl
10570144	Arhgef7	NM_001113518	1.03775e-006	1.4605	E11.5 * Wnt1 up vs E11.5 * Ctrl
10583145	Tmem123	NM_133739	3.47493e-006	1.46025	E11.5 * Wnt1 up vs E11.5 * Ctrl
10567355	Gprc5b	NM_022420	4.31877e-005	1.46008	E11.5 * Wnt1 up vs E11.5 * Ctrl
10585699	Fabp5	NM_010634	5.72884e-006	1.45987	E11.5 * Wnt1 up vs E11.5 * Ctrl
10510391	Srm	NM_009272	0.000118638	1.45969	E11.5 * Wnt1 up vs E11.5 * Ctrl
10497214	Tpd52	NM_001025261	0.00409595	1.45948	E11.5 * Wnt1 up vs E11.5 * Ctrl
10549097	Ldhb	NM_008492	2.01773e-007	1.45946	E11.5 * Wnt1 up vs E11.5 * Ctrl
10605797	Arhgef9	NM_001033329	4.87714e-006	1.45904	E11.5 * Wnt1 up vs E11.5 * Ctrl
10501909	Mettl14	NM_201638	3.12745e-007	1.45859	E11.5 * Wnt1 up vs E11.5 * Ctrl
10517373	Rcan3	NM_022980	1.7873e-007	1.45835	E11.5 * Wnt1 up vs E11.5 * Ctrl
10583384		---	0.00228106	1.4583	E11.5 * Wnt1 up vs E11.5 * Ctrl
10467749	Crtac1	NM_145123	3.39656e-006	1.45825	E11.5 * Wnt1 up vs E11.5 * Ctrl
10607587	Pdha1	NM_008810	4.269e-005	1.45825	E11.5 * Wnt1 up vs E11.5 * Ctrl
10540333	Cntn6	NM_017383	0.00257454	1.45792	E11.5 * Wnt1 up vs E11.5 * Ctrl
10607004	Psmd10	NM_016883	0.00015909	1.45768	E11.5 * Wnt1 up vs E11.5 * Ctrl
10446763	Lbh	NM_029999	9.02323e-007	1.45765	E11.5 * Wnt1 up vs E11.5 * Ctrl
10536216	Gng11	NM_025331	0.000878501	1.45733	E11.5 * Wnt1 up vs E11.5 * Ctrl
10378088	Mybbp1a	NM_016776	0.00133271	1.4573	E11.5 * Wnt1 up vs E11.5 * Ctrl
10529957	Gpr125	ENSMUST00000(	1.4062e-005	1.45688	E11.5 * Wnt1 up vs E11.5 * Ctrl
10481291	Gtf3c5	NM_148928	0.00188926	1.45646	E11.5 * Wnt1 up vs E11.5 * Ctrl
10442914	0610011F06Rik	NM_026686	2.64574e-005	1.45598	E11.5 * Wnt1 up vs E11.5 * Ctrl
10439955	Fam55c	NM_001134457	0.00114725	1.45586	E11.5 * Wnt1 up vs E11.5 * Ctrl
10524588	Ankrd13a	NM_026718	0.000186345	1.45573	E11.5 * Wnt1 up vs E11.5 * Ctrl
10375485		---	9.6379e-006	1.45529	E11.5 * Wnt1 up vs E11.5 * Ctrl
10452918	Strn	NM_011500	8.72141e-006	1.45523	E11.5 * Wnt1 up vs E11.5 * Ctrl
10490061	Bcas1	NM_029815	1.5944e-005	1.45519	E11.5 * Wnt1 up vs E11.5 * Ctrl
10549653	Atp6v0c	NM_009729	0.00500411	1.45453	E11.5 * Wnt1 up vs E11.5 * Ctrl
10358666	Hmcn1	NM_001024720	3.80569e-005	1.45421	E11.5 * Wnt1 up vs E11.5 * Ctrl
10576844	BC068157	NM_207203	0.00021201	1.45397	E11.5 * Wnt1 up vs E11.5 * Ctrl
10376854	Pigl	NM_001039536	4.81379e-005	1.45393	E11.5 * Wnt1 up vs E11.5 * Ctrl
10501121	Fam40a	NM_153563	0.000109923	1.4537	E11.5 * Wnt1 up vs E11.5 * Ctrl
10478997	Tcfap2c	NM_009335	1.17351e-006	1.45324	E11.5 * Wnt1 up vs E11.5 * Ctrl
10473244	Zfp804a	NM_175513	0.000525774	1.45274	E11.5 * Wnt1 up vs E11.5 * Ctrl
10525542	Bcl7a	NM_029850	0.000247471	1.45254	E11.5 * Wnt1 up vs E11.5 * Ctrl
10399419	Tubb2b	NM_023716	0.000474672	1.45212	E11.5 * Wnt1 up vs E11.5 * Ctrl
10510687	Acot7	NM_133348	0.000134357	1.45191	E11.5 * Wnt1 up vs E11.5 * Ctrl
10504218	Dnajb5	NM_019874	0.000429281	1.45185	E11.5 * Wnt1 up vs E11.5 * Ctrl
10478924	ENSMUSG0000C	ENSMUST00000(	0.00625851	1.45176	E11.5 * Wnt1 up vs E11.5 * Ctrl
10503966	Aco1	NM_007386	2.65482e-005	1.45165	E11.5 * Wnt1 up vs E11.5 * Ctrl
10453717	Fabp5l2	ENSMUST00000(	0.0022163	1.45154	E11.5 * Wnt1 up vs E11.5 * Ctrl
10436487	Vgll3	AK007165	0.00343097	1.45123	E11.5 * Wnt1 up vs E11.5 * Ctrl
10499438	Msto1	NM_144898	0.0036747	1.45092	E11.5 * Wnt1 up vs E11.5 * Ctrl
10411882	Nln	NM_029447	5.41322e-005	1.45064	E11.5 * Wnt1 up vs E11.5 * Ctrl
10384398	Grb10	NM_010345	3.08639e-005	1.45041	E11.5 * Wnt1 up vs E11.5 * Ctrl
10476189	Mrps26	NM_207207	0.00167789	1.45028	E11.5 * Wnt1 up vs E11.5 * Ctrl
10356833	Stk25	NM_021537	0.00160673	1.44976	E11.5 * Wnt1 up vs E11.5 * Ctrl
10384452	Ublcp1	NM_024475	0.00096641	1.44937	E11.5 * Wnt1 up vs E11.5 * Ctrl
10440840	1110004E09Rik	BC019533	7.74108e-006	1.44928	E11.5 * Wnt1 up vs E11.5 * Ctrl
10566714	Ric3	NM_001038624	0.00025333	1.4482	E11.5 * Wnt1 up vs E11.5 * Ctrl
10529656	Nsg1	NM_010942	2.10542e-005	1.44789	E11.5 * Wnt1 up vs E11.5 * Ctrl
10351825	Tagln2	NM_178598	0.000427889	1.44778	E11.5 * Wnt1 up vs E11.5 * Ctrl
10554094	Igf1r	NM_010513	0.00056012	1.44721	E11.5 * Wnt1 up vs E11.5 * Ctrl
10366951	Ndufa4l2	NM_001098789	0.00841381	1.44622	E11.5 * Wnt1 up vs E11.5 * Ctrl
10457536	Ankrd29	ENSMUST00000	0.00040058	1.44579	E11.5 * Wnt1 up vs E11.5 * Ctrl
10434577	Vps8	NM_001081366	9.19153e-009	1.44558	E11.5 * Wnt1 up vs E11.5 * Ctrl
10603706	Med4	NM_026119	0.00385632	1.44495	E11.5 * Wnt1 up vs E11.5 * Ctrl
10448865	Gnptg	NM_172529	1.22139e-006	1.44495	E11.5 * Wnt1 up vs E11.5 * Ctrl
10457942	Syt4	NM_009308	0.00231261	1.44485	E11.5 * Wnt1 up vs E11.5 * Ctrl

10422179	Pou4f1	NM_011143	0.000314658	1.44483	E11.5 * Wnt1 up vs E11.5 * Ctrl
10482772	Nr4a2	NM_001139509	0.00162725	1.44471	E11.5 * Wnt1 up vs E11.5 * Ctrl
10494060	Mrpl9	NM_030116	1.79937e-005	1.44419	E11.5 * Wnt1 up vs E11.5 * Ctrl
10419170	Txndc16	NM_172597	0.00146216	1.44396	E11.5 * Wnt1 up vs E11.5 * Ctrl
10357345	E030049G20Rik	NM_172484	0.00303803	1.4439	E11.5 * Wnt1 up vs E11.5 * Ctrl
10507105	OTTMUSG00000	ENSMUST00000(	0.00534321	1.44381	E11.5 * Wnt1 up vs E11.5 * Ctrl
10348432	Agap1	NM_178119	0.000827868	1.44332	E11.5 * Wnt1 up vs E11.5 * Ctrl
10386844	Zswim7	BC109343	0.00459535	1.44311	E11.5 * Wnt1 up vs E11.5 * Ctrl
10493309	Rit1	NM_009069	0.00479185	1.44283	E11.5 * Wnt1 up vs E11.5 * Ctrl
10379654	Ap2b1	NM_001035854	3.05236e-007	1.44268	E11.5 * Wnt1 up vs E11.5 * Ctrl
10410959	Atg10	NM_025770	0.00375203	1.44261	E11.5 * Wnt1 up vs E11.5 * Ctrl
10521243	Grk4	NM_019497	0.00520576	1.44192	E11.5 * Wnt1 up vs E11.5 * Ctrl
10476005	A730036I17Rik	ENSMUST00000(	0.00117815	1.44185	E11.5 * Wnt1 up vs E11.5 * Ctrl
10453887	Cables1	NM_001146287	2.84694e-005	1.44161	E11.5 * Wnt1 up vs E11.5 * Ctrl
10540248	Mitf	NM_001113198	0.00259605	1.44141	E11.5 * Wnt1 up vs E11.5 * Ctrl
10555570	Phox2a	NM_008887	0.00220145	1.44124	E11.5 * Wnt1 up vs E11.5 * Ctrl
10421526	Rb1	NM_009029	0.000293052	1.44104	E11.5 * Wnt1 up vs E11.5 * Ctrl
10557420	Tufm	NM_172745	0.00449779	1.44104	E11.5 * Wnt1 up vs E11.5 * Ctrl
10458183	Gfra3	NM_010280	0.000536529	1.44045	E11.5 * Wnt1 up vs E11.5 * Ctrl
10369630	Ddx21	NM_019553	5.37031e-005	1.43982	E11.5 * Wnt1 up vs E11.5 * Ctrl
10589368	Plxnb1	NM_172775	0.00481016	1.43972	E11.5 * Wnt1 up vs E11.5 * Ctrl
10442396	Abca3	NM_013855	1.07995e-005	1.4396	E11.5 * Wnt1 up vs E11.5 * Ctrl
10566457	Apbb1	NM_009685	0.000331286	1.43869	E11.5 * Wnt1 up vs E11.5 * Ctrl
10450226	Ppt2	NM_019441	0.00729624	1.43849	E11.5 * Wnt1 up vs E11.5 * Ctrl
10593668	Dmxl2	NM_172771	0.00226384	1.4383	E11.5 * Wnt1 up vs E11.5 * Ctrl
10419073	Tspan14	NM_145928	0.000152712	1.4383	E11.5 * Wnt1 up vs E11.5 * Ctrl
10521440	Afap1	NM_027373	3.82336e-005	1.43818	E11.5 * Wnt1 up vs E11.5 * Ctrl
10363575	Dna2	NM_177372	6.27922e-007	1.43772	E11.5 * Wnt1 up vs E11.5 * Ctrl
10457118	Rttn	NM_175542	0.000406568	1.4375	E11.5 * Wnt1 up vs E11.5 * Ctrl
10513061	Ctnnal1	NM_018761	1.96096e-005	1.43699	E11.5 * Wnt1 up vs E11.5 * Ctrl
10542896	Bicd1	NM_009753	0.000411561	1.43685	E11.5 * Wnt1 up vs E11.5 * Ctrl
10500582	Wdr3	NM_175552	0.000109024	1.43665	E11.5 * Wnt1 up vs E11.5 * Ctrl
10528648	Abcf2	NM_013853	2.44528e-005	1.43584	E11.5 * Wnt1 up vs E11.5 * Ctrl
10504518	Polr1e	NM_022811	8.4399e-006	1.43554	E11.5 * Wnt1 up vs E11.5 * Ctrl
10553897	Mtmr10	NM_172742	0.00420393	1.43541	E11.5 * Wnt1 up vs E11.5 * Ctrl
10534575	Prkrip1	NM_025774	1.49956e-005	1.4352	E11.5 * Wnt1 up vs E11.5 * Ctrl
10581577	Dhx38	NM_178380	0.00125446	1.43508	E11.5 * Wnt1 up vs E11.5 * Ctrl
10429327	Eif2c2	NM_153178	0.000557563	1.4347	E11.5 * Wnt1 up vs E11.5 * Ctrl
10400023	Tspan13	NM_025359	4.59571e-005	1.43429	E11.5 * Wnt1 up vs E11.5 * Ctrl
10412909	Fdft1	NM_010191	0.00131345	1.43313	E11.5 * Wnt1 up vs E11.5 * Ctrl
10449920	Zfp811	NM_183177	0.00452178	1.43306	E11.5 * Wnt1 up vs E11.5 * Ctrl
10465638	Nat11	BC020020	0.00593877	1.43302	E11.5 * Wnt1 up vs E11.5 * Ctrl
10494804	Casq2	NM_009814	0.000728783	1.43296	E11.5 * Wnt1 up vs E11.5 * Ctrl
10391103	Jup	NM_010593	0.00322432	1.43255	E11.5 * Wnt1 up vs E11.5 * Ctrl
10545379	Usp39	NM_138592	4.25787e-006	1.43226	E11.5 * Wnt1 up vs E11.5 * Ctrl
10425116	Cdc42ep1	NM_027219	0.00768275	1.43215	E11.5 * Wnt1 up vs E11.5 * Ctrl
10526181	Gatsl2	NM_030719	0.000124849	1.43178	E11.5 * Wnt1 up vs E11.5 * Ctrl
10510482	Clstn1	NM_023051	0.00187247	1.43166	E11.5 * Wnt1 up vs E11.5 * Ctrl
10521467	Ccdc96	NM_025725	0.000781375	1.43146	E11.5 * Wnt1 up vs E11.5 * Ctrl
10518561	Apitd1	NM_027263	0.000975899	1.43119	E11.5 * Wnt1 up vs E11.5 * Ctrl
10593270	Ttc12	NM_172770	1.74229e-005	1.43084	E11.5 * Wnt1 up vs E11.5 * Ctrl
10363901	Etv5	NM_023794	0.000129198	1.43083	E11.5 * Wnt1 up vs E11.5 * Ctrl
10394770	Odc1	NM_013614	2.56136e-006	1.43072	E11.5 * Wnt1 up vs E11.5 * Ctrl
10350113	Arl8a	NM_026823	0.000841416	1.43061	E11.5 * Wnt1 up vs E11.5 * Ctrl
10352242	Parp1	NM_007415	0.00012399	1.43041	E11.5 * Wnt1 up vs E11.5 * Ctrl
10358617	Hmcn1	NM_001024720	0.00354611	1.42986	E11.5 * Wnt1 up vs E11.5 * Ctrl
10535852	Slc7a1	NM_007513	0.000123743	1.42986	E11.5 * Wnt1 up vs E11.5 * Ctrl
10414025	Gdf10	NM_145741	1.31802e-006	1.42961	E11.5 * Wnt1 up vs E11.5 * Ctrl
10430020	Vps28	NM_025842	0.000823252	1.42929	E11.5 * Wnt1 up vs E11.5 * Ctrl
10353915	Fam178b	NM_027957	0.00488665	1.42919	E11.5 * Wnt1 up vs E11.5 * Ctrl
10485582	Tcp11l1	NM_177190	0.000575063	1.42918	E11.5 * Wnt1 up vs E11.5 * Ctrl
10347232	Xrcc5	NM_009533	5.78792e-006	1.42915	E11.5 * Wnt1 up vs E11.5 * Ctrl
10544837	1200009O22Rik	BC043099	2.96328e-005	1.42911	E11.5 * Wnt1 up vs E11.5 * Ctrl
10441497	Tulp4	NM_054040	0.00471849	1.42879	E11.5 * Wnt1 up vs E11.5 * Ctrl
10433264	Glis2	NM_031184	7.20773e-005	1.42852	E11.5 * Wnt1 up vs E11.5 * Ctrl
10458767	Trim36	NM_178872	0.00102772	1.42839	E11.5 * Wnt1 up vs E11.5 * Ctrl

10404380	Dusp22	NM_001037955	2.07996e-005	1.42833	E11.5 * Wnt1 up vs E11.5 * Ctrl
10576305	Tcf25	NM_001037878	2.79566e-005	1.42738	E11.5 * Wnt1 up vs E11.5 * Ctrl
10538354	1200009O22Rik	BC043099	1.43683e-006	1.42697	E11.5 * Wnt1 up vs E11.5 * Ctrl
10492890	Lrba	NM_030695	2.26725e-005	1.42696	E11.5 * Wnt1 up vs E11.5 * Ctrl
10421648	Slc25a30	NM_026232	4.33909e-005	1.42674	E11.5 * Wnt1 up vs E11.5 * Ctrl
10435057	Pcyt1a	NM_009981	0.000241738	1.42638	E11.5 * Wnt1 up vs E11.5 * Ctrl
10458461	Hdac3	NM_010411	1.41568e-005	1.42633	E11.5 * Wnt1 up vs E11.5 * Ctrl
10358670	Hmcn1	NM_001024720	0.000222377	1.42628	E11.5 * Wnt1 up vs E11.5 * Ctrl
10409660	Gkap1	NM_019832	0.00436397	1.42544	E11.5 * Wnt1 up vs E11.5 * Ctrl
10595466	Pgm3	NM_028352	4.63633e-005	1.42541	E11.5 * Wnt1 up vs E11.5 * Ctrl
10347734	Sgpp2	NM_001004173	6.61685e-005	1.42522	E11.5 * Wnt1 up vs E11.5 * Ctrl
10535080	Cox19	NM_197980	0.000404339	1.42501	E11.5 * Wnt1 up vs E11.5 * Ctrl
10540085	Fbln2	NM_007992	0.00131795	1.42383	E11.5 * Wnt1 up vs E11.5 * Ctrl
10555953	Fxc1	NM_019502	0.000112024	1.42339	E11.5 * Wnt1 up vs E11.5 * Ctrl
10373728	Patz1	NM_019574	0.00253785	1.4233	E11.5 * Wnt1 up vs E11.5 * Ctrl
10595680	Tbc1d2b	NM_194334	0.000209052	1.42322	E11.5 * Wnt1 up vs E11.5 * Ctrl
10409021	Tpmt	NM_016785	0.000264184	1.42305	E11.5 * Wnt1 up vs E11.5 * Ctrl
10430748	Rangap1	NM_011241	0.00136237	1.42277	E11.5 * Wnt1 up vs E11.5 * Ctrl
10373113	Kif5a	NM_008447	0.00407199	1.42248	E11.5 * Wnt1 up vs E11.5 * Ctrl
10463535	Nolc1	NM_053086	8.94757e-006	1.4223	E11.5 * Wnt1 up vs E11.5 * Ctrl
10479514	Col20a1	BC016112	0.000760168	1.42218	E11.5 * Wnt1 up vs E11.5 * Ctrl
10511580	Ppm2c	NM_001098230	2.38987e-005	1.42206	E11.5 * Wnt1 up vs E11.5 * Ctrl
10592891	Phldb1	NM_153537	0.00013569	1.42175	E11.5 * Wnt1 up vs E11.5 * Ctrl
10576274	Zfp276	NM_020497	0.000514215	1.42132	E11.5 * Wnt1 up vs E11.5 * Ctrl
10404702	Gcnt2	NM_023887	0.00010111	1.42103	E11.5 * Wnt1 up vs E11.5 * Ctrl
10447341	Rhoq	NM_145491	0.000324178	1.42078	E11.5 * Wnt1 up vs E11.5 * Ctrl
10474977	1500003O03Rik	NM_019769	1.21587e-005	1.42074	E11.5 * Wnt1 up vs E11.5 * Ctrl
10363231	Smpdl3a	NM_020561	0.00169463	1.42012	E11.5 * Wnt1 up vs E11.5 * Ctrl
10381226	Coasy	NM_027896	0.000693212	1.42009	E11.5 * Wnt1 up vs E11.5 * Ctrl
10583788	Prkcsh	NM_008925	0.00171533	1.41901	E11.5 * Wnt1 up vs E11.5 * Ctrl
10590909	Endod1	NM_028013	8.88375e-005	1.41887	E11.5 * Wnt1 up vs E11.5 * Ctrl
10437174	Wrb	NM_207301	0.00161768	1.4183	E11.5 * Wnt1 up vs E11.5 * Ctrl
10526838	Got2	NM_010325	2.27294e-005	1.41793	E11.5 * Wnt1 up vs E11.5 * Ctrl
10419578	Ndrg2	NM_013864	0.000547754	1.41791	E11.5 * Wnt1 up vs E11.5 * Ctrl
10546476	Magi1	NM_001029850	1.63412e-005	1.41789	E11.5 * Wnt1 up vs E11.5 * Ctrl
10559415	Leng1	NM_027203	0.000661969	1.41716	E11.5 * Wnt1 up vs E11.5 * Ctrl
10491083	Aadacl1	NM_178772	0.0009033	1.41691	E11.5 * Wnt1 up vs E11.5 * Ctrl
10568109	Asphd1	NM_001039645	0.00148379	1.4167	E11.5 * Wnt1 up vs E11.5 * Ctrl
10439218	Pdia5	NM_028295	4.88268e-006	1.41658	E11.5 * Wnt1 up vs E11.5 * Ctrl
10542791	Ppfibp1	NM_026221	0.000107145	1.41622	E11.5 * Wnt1 up vs E11.5 * Ctrl
10580537	Aktip	NM_010241	0.00077883	1.41575	E11.5 * Wnt1 up vs E11.5 * Ctrl
10592126	Fam118b	NM_194257	1.85226e-005	1.41523	E11.5 * Wnt1 up vs E11.5 * Ctrl
10526853	Fam20c	NM_030565	0.00372974	1.41388	E11.5 * Wnt1 up vs E11.5 * Ctrl
10578984	Tufm	NM_172745	0.00456527	1.41336	E11.5 * Wnt1 up vs E11.5 * Ctrl
10596545	Rad54l2	NM_030730	2.06244e-006	1.4131	E11.5 * Wnt1 up vs E11.5 * Ctrl
10572613	Mrpl34	NM_053162	0.00644638	1.41295	E11.5 * Wnt1 up vs E11.5 * Ctrl
10346235	Hibch	NM_146108	0.00180791	1.41278	E11.5 * Wnt1 up vs E11.5 * Ctrl
10451763	Satb1	NM_009122	3.96486e-007	1.41208	E11.5 * Wnt1 up vs E11.5 * Ctrl
10397332	Eif2b2	NM_145445	0.00663571	1.41194	E11.5 * Wnt1 up vs E11.5 * Ctrl
10362490	Tspyl4	NM_030203	0.000253091	1.41184	E11.5 * Wnt1 up vs E11.5 * Ctrl
10375730		---	0.0026126	1.41145	E11.5 * Wnt1 up vs E11.5 * Ctrl
10383575	Tbcd	NM_029878	1.19626e-006	1.41135	E11.5 * Wnt1 up vs E11.5 * Ctrl
10415725	Spata13	BC138455	0.000705536	1.4112	E11.5 * Wnt1 up vs E11.5 * Ctrl
10358668	Hmcn1	NM_001024720	8.82127e-005	1.41099	E11.5 * Wnt1 up vs E11.5 * Ctrl
10501629	Cdc14a	NM_001080818	6.61117e-006	1.41049	E11.5 * Wnt1 up vs E11.5 * Ctrl
10606554	Nap1l3	NM_138742	0.00153239	1.41017	E11.5 * Wnt1 up vs E11.5 * Ctrl
10518147	Pdpn	NM_010329	0.00877869	1.41012	E11.5 * Wnt1 up vs E11.5 * Ctrl
10492325	Ube2v1	NM_023230	0.000229056	1.4096	E11.5 * Wnt1 up vs E11.5 * Ctrl
10550274	Meis3	NM_008627	2.68578e-005	1.40916	E11.5 * Wnt1 up vs E11.5 * Ctrl
10409464	Dbn1	NM_019813	0.00103498	1.40896	E11.5 * Wnt1 up vs E11.5 * Ctrl
10355141	Klf7	NM_033563	4.66538e-006	1.40817	E11.5 * Wnt1 up vs E11.5 * Ctrl
10529041	Preb	NM_016703	0.00351691	1.40772	E11.5 * Wnt1 up vs E11.5 * Ctrl
10388018	Dhx33	NM_178367	0.000315102	1.40768	E11.5 * Wnt1 up vs E11.5 * Ctrl
10562211	Fxyd1	NM_052992	0.00370171	1.40764	E11.5 * Wnt1 up vs E11.5 * Ctrl
10515943	Ctps	NM_016748	2.19575e-005	1.40739	E11.5 * Wnt1 up vs E11.5 * Ctrl
10517488	Ephb2	NM_010142	0.000346285	1.40727	E11.5 * Wnt1 up vs E11.5 * Ctrl

10524882	Wsb2	NM_021539	0.000503754	1.40701	E11.5 * Wnt1 up vs E11.5 * Ctrl
10501319	Celsr2	NM_017392	0.000919283	1.407	E11.5 * Wnt1 up vs E11.5 * Ctrl
10533858	Eif2b1	NM_145371	1.74131e-006	1.40686	E11.5 * Wnt1 up vs E11.5 * Ctrl
10561679	Psmd8	NM_026545	6.89193e-005	1.40671	E11.5 * Wnt1 up vs E11.5 * Ctrl
10419966	Zfhx2	NM_001039198	0.00209478	1.40533	E11.5 * Wnt1 up vs E11.5 * Ctrl
10358733	Rgl1	NM_016846	0.0006032	1.4052	E11.5 * Wnt1 up vs E11.5 * Ctrl
10477994	Ctnnbl1	NM_025680	3.45325e-005	1.4047	E11.5 * Wnt1 up vs E11.5 * Ctrl
10583929	B3gat1	NM_029792	0.000182413	1.40457	E11.5 * Wnt1 up vs E11.5 * Ctrl
10480950	D2Bwg1335e	NM_026828	0.000406098	1.4045	E11.5 * Wnt1 up vs E11.5 * Ctrl
10354031	Tsga10	NM_207228	0.00592661	1.40366	E11.5 * Wnt1 up vs E11.5 * Ctrl
10494007	Them4	NM_029431	0.00330071	1.40363	E11.5 * Wnt1 up vs E11.5 * Ctrl
10571384	Efha2	ENSMUST00000	0.000825254	1.40328	E11.5 * Wnt1 up vs E11.5 * Ctrl
10531146	Mkrn1-ps1	AF494488	0.00131828	1.40314	E11.5 * Wnt1 up vs E11.5 * Ctrl
10427772	Tars	NM_033074	0.000398595	1.4016	E11.5 * Wnt1 up vs E11.5 * Ctrl
10578916	Sc4mol	NM_025436	0.00242051	1.40152	E11.5 * Wnt1 up vs E11.5 * Ctrl
10388227	Cyb5d2	NM_001024926	0.00491853	1.40122	E11.5 * Wnt1 up vs E11.5 * Ctrl
10516894	Rcc1	NM_133878	0.000712153	1.40107	E11.5 * Wnt1 up vs E11.5 * Ctrl
10523479	Slc25a5	NM_007451	1.7845e-005	1.40105	E11.5 * Wnt1 up vs E11.5 * Ctrl
10412227	Snx18	NM_130796	2.73929e-005	1.40077	E11.5 * Wnt1 up vs E11.5 * Ctrl
10402318	Prima1	NM_133364	0.00753324	1.40056	E11.5 * Wnt1 up vs E11.5 * Ctrl
10381744	Arf2	NM_007477	0.000173918	1.40054	E11.5 * Wnt1 up vs E11.5 * Ctrl
10528227	Gnai1	NM_010305	0.000184582	1.40043	E11.5 * Wnt1 up vs E11.5 * Ctrl
10486875	Frmd5	NM_172673	0.000473592	1.4003	E11.5 * Wnt1 up vs E11.5 * Ctrl
10390211	Igf2bp1	NM_009951	0.00223197	1.39975	E11.5 * Wnt1 up vs E11.5 * Ctrl
10401753	AI413782	NM_001142580	0.000357728	1.39965	E11.5 * Wnt1 up vs E11.5 * Ctrl
10533729	Vps37b	NM_177876	0.000755855	1.39959	E11.5 * Wnt1 up vs E11.5 * Ctrl
10432190	Adcy6	NM_007405	0.00717413	1.39937	E11.5 * Wnt1 up vs E11.5 * Ctrl
10382912	09-Sep	NM_001113486	5.51119e-005	1.39929	E11.5 * Wnt1 up vs E11.5 * Ctrl
10594501	Ptplad1	NM_021345	0.0007452	1.39878	E11.5 * Wnt1 up vs E11.5 * Ctrl
10366866	Dctn2	NM_027151	0.00242071	1.39823	E11.5 * Wnt1 up vs E11.5 * Ctrl
10574204	Arl2bp	NM_024191	2.83168e-006	1.39756	E11.5 * Wnt1 up vs E11.5 * Ctrl
10417713	Rarb	NM_011243	0.00025335	1.39755	E11.5 * Wnt1 up vs E11.5 * Ctrl
10591726	Ecsit	NM_012029	0.000272092	1.39745	E11.5 * Wnt1 up vs E11.5 * Ctrl
10475708	Blvra	NM_026678	0.000125738	1.39739	E11.5 * Wnt1 up vs E11.5 * Ctrl
10387757	Rai12	NM_018740	0.00165863	1.39735	E11.5 * Wnt1 up vs E11.5 * Ctrl
10571344	D8Ertd82e	NM_172911	0.000799156	1.39681	E11.5 * Wnt1 up vs E11.5 * Ctrl
10520842	Bre	NM_181279	6.23165e-005	1.39664	E11.5 * Wnt1 up vs E11.5 * Ctrl
10414325	Cgrrf1	NM_026832	0.00515156	1.39612	E11.5 * Wnt1 up vs E11.5 * Ctrl
10429908	Bop1	NM_013481	0.000958352	1.39609	E11.5 * Wnt1 up vs E11.5 * Ctrl
10362767	Mical1	NM_138315	0.000470568	1.39589	E11.5 * Wnt1 up vs E11.5 * Ctrl
10349239	Mki67ip	NM_026472	6.91645e-005	1.39584	E11.5 * Wnt1 up vs E11.5 * Ctrl
10419288	Gch1	NM_008102	0.00119264	1.39576	E11.5 * Wnt1 up vs E11.5 * Ctrl
10498204	ENSMUSG00000	ENSMUST00000	0.00501889	1.39463	E11.5 * Wnt1 up vs E11.5 * Ctrl
10406856	Gm807	ENSMUST00000	0.00348183	1.39457	E11.5 * Wnt1 up vs E11.5 * Ctrl
10427035	Nr4a1	NM_010444	0.000437396	1.39444	E11.5 * Wnt1 up vs E11.5 * Ctrl
10430804	Pppde2	NM_134095	0.000426363	1.39444	E11.5 * Wnt1 up vs E11.5 * Ctrl
10400866	Trim9	NM_053167	0.0012536	1.39343	E11.5 * Wnt1 up vs E11.5 * Ctrl
10404941	Aof1	NM_172262	0.000421568	1.39319	E11.5 * Wnt1 up vs E11.5 * Ctrl
10534102	Gusb	NM_010368	0.00121129	1.3931	E11.5 * Wnt1 up vs E11.5 * Ctrl
10353934	Actr1b	NM_146107	0.00663602	1.39289	E11.5 * Wnt1 up vs E11.5 * Ctrl
10430956	Cyb5r3	NM_029787	0.00419671	1.39283	E11.5 * Wnt1 up vs E11.5 * Ctrl
10381416	Rnd2	NM_009708	0.0023031	1.39276	E11.5 * Wnt1 up vs E11.5 * Ctrl
10409338	Nop16	NM_178605	0.00174423	1.39249	E11.5 * Wnt1 up vs E11.5 * Ctrl
10584142	Ets1	NM_011808	0.000146584	1.39232	E11.5 * Wnt1 up vs E11.5 * Ctrl
10363563	Slc25a16	NM_175194	0.00294346	1.39227	E11.5 * Wnt1 up vs E11.5 * Ctrl
10542200	Gabarapl1	NM_020590	9.90827e-005	1.39226	E11.5 * Wnt1 up vs E11.5 * Ctrl
10370824	Mbd3	NM_013595	0.00420163	1.39218	E11.5 * Wnt1 up vs E11.5 * Ctrl
10566281	Dnajc19	NM_026332	0.00610242	1.39206	E11.5 * Wnt1 up vs E11.5 * Ctrl
10510286	Mad2l2	NM_027985	0.000203472	1.39206	E11.5 * Wnt1 up vs E11.5 * Ctrl
10563390	Cyth2	NM_011181	0.0034773	1.39142	E11.5 * Wnt1 up vs E11.5 * Ctrl
10501244	Ampd2	NM_028779	0.000610567	1.39096	E11.5 * Wnt1 up vs E11.5 * Ctrl
10381574	Rundc3a	NM_016759	0.00062423	1.39092	E11.5 * Wnt1 up vs E11.5 * Ctrl
10584422	Olfr908	NM_146872	0.00020545	1.3908	E11.5 * Wnt1 up vs E11.5 * Ctrl
10436369	Filip1l	NM_030163	2.17876e-006	1.39053	E11.5 * Wnt1 up vs E11.5 * Ctrl
10507040	Spata6	NM_026470	0.00119851	1.3905	E11.5 * Wnt1 up vs E11.5 * Ctrl
10511226	Acap3	NM_207223	0.00015392	1.39033	E11.5 * Wnt1 up vs E11.5 * Ctrl

10388749	Traf4	NM_009423	0.000506842	1.3903	E11.5 * Wnt1 up vs E11.5 * Ctrl
10557450	Bola2	NM_175103	2.15638e-005	1.39003	E11.5 * Wnt1 up vs E11.5 * Ctrl
10413255	Duxbl	NM_183389	0.00245655	1.38953	E11.5 * Wnt1 up vs E11.5 * Ctrl
10413265	Duxbl	NM_183389	0.00245655	1.38953	E11.5 * Wnt1 up vs E11.5 * Ctrl
10374529	AV249152	NM_145425	0.000563737	1.38894	E11.5 * Wnt1 up vs E11.5 * Ctrl
10371321	Slc41a2	NM_177388	0.00542056	1.38882	E11.5 * Wnt1 up vs E11.5 * Ctrl
10380648	Hoxb4	NM_010459	0.000141775	1.38882	E11.5 * Wnt1 up vs E11.5 * Ctrl
10547553	Mical3	NM_153396	0.000169386	1.38867	E11.5 * Wnt1 up vs E11.5 * Ctrl
10408975	Kif13a	NM_010617	0.000370178	1.38864	E11.5 * Wnt1 up vs E11.5 * Ctrl
10376813	Cytsb	NM_001029936	9.7813e-005	1.38759	E11.5 * Wnt1 up vs E11.5 * Ctrl
10447490	Pja2	NM_001025309	1.28523e-006	1.38698	E11.5 * Wnt1 up vs E11.5 * Ctrl
10496110	Papss1	NM_011863	0.00061408	1.38677	E11.5 * Wnt1 up vs E11.5 * Ctrl
10519951	Pion	NM_175437	0.00351428	1.38653	E11.5 * Wnt1 up vs E11.5 * Ctrl
10597323	Arpp21	NM_033264	2.49231e-005	1.38623	E11.5 * Wnt1 up vs E11.5 * Ctrl
10405757	2010111I01Rik	NM_028079	0.000241217	1.38617	E11.5 * Wnt1 up vs E11.5 * Ctrl
10585601	Snupn	NM_178374	0.000533258	1.38606	E11.5 * Wnt1 up vs E11.5 * Ctrl
10578623	Wwc2	NM_133791	5.64672e-006	1.38559	E11.5 * Wnt1 up vs E11.5 * Ctrl
10388648	Ankrd13b	NM_172945	0.000926243	1.38537	E11.5 * Wnt1 up vs E11.5 * Ctrl
10440929	Gart	NM_010256	2.85002e-005	1.38508	E11.5 * Wnt1 up vs E11.5 * Ctrl
10393836	Arhgdia	NM_133796	0.00728596	1.38461	E11.5 * Wnt1 up vs E11.5 * Ctrl
10507804	Trit1	NM_025873	0.00232407	1.38456	E11.5 * Wnt1 up vs E11.5 * Ctrl
10570513	2900016B01Rik	ENSMUST00000	0.00298011	1.38455	E11.5 * Wnt1 up vs E11.5 * Ctrl
10454735	Fam53c	BC057111	0.00390731	1.38446	E11.5 * Wnt1 up vs E11.5 * Ctrl
10430489	Pla2g6	NM_016915	0.000332392	1.38438	E11.5 * Wnt1 up vs E11.5 * Ctrl
10481128	Med22	NM_011513	0.00290101	1.38434	E11.5 * Wnt1 up vs E11.5 * Ctrl
10589800	Clasp2	NM_001114347	2.84921e-007	1.38421	E11.5 * Wnt1 up vs E11.5 * Ctrl
10350247	Kif21b	NM_001039472	0.00013826	1.38407	E11.5 * Wnt1 up vs E11.5 * Ctrl
10590663	Gria4	NM_019691	0.000237643	1.38373	E11.5 * Wnt1 up vs E11.5 * Ctrl
10365260	Txnrd1	NM_001042523	4.40481e-007	1.38355	E11.5 * Wnt1 up vs E11.5 * Ctrl
10433620	Ercc4	NM_015769	0.00350976	1.38345	E11.5 * Wnt1 up vs E11.5 * Ctrl
10454077	Taf4b	NM_001100449	0.000253922	1.38241	E11.5 * Wnt1 up vs E11.5 * Ctrl
10599232	Nkap	NM_025937	0.00442251	1.38169	E11.5 * Wnt1 up vs E11.5 * Ctrl
10525111	Ddx54	NM_028041	0.000814118	1.38162	E11.5 * Wnt1 up vs E11.5 * Ctrl
10355958	Ube2v1	NM_023230	0.000779371	1.38156	E11.5 * Wnt1 up vs E11.5 * Ctrl
10440738	Tiam1	NM_009384	0.000320044	1.3815	E11.5 * Wnt1 up vs E11.5 * Ctrl
10465011	Sart1	NM_016882	5.56166e-005	1.38121	E11.5 * Wnt1 up vs E11.5 * Ctrl
10456346	Sec11c	NM_025468	0.000631222	1.38119	E11.5 * Wnt1 up vs E11.5 * Ctrl
10382508	Ict1	NM_026729	0.00362979	1.38108	E11.5 * Wnt1 up vs E11.5 * Ctrl
10552311	OTTMUSG00000	XR_030737	0.000176161	1.38097	E11.5 * Wnt1 up vs E11.5 * Ctrl
10374119	Ogdh	NM_010956	0.00132214	1.38029	E11.5 * Wnt1 up vs E11.5 * Ctrl
10516305	Mtap7d1	NM_144941	2.54234e-006	1.38014	E11.5 * Wnt1 up vs E11.5 * Ctrl
10361007	Smyd2	NM_026796	5.17024e-006	1.38008	E11.5 * Wnt1 up vs E11.5 * Ctrl
10383970	Kremen1	NM_032396	0.000479315	1.37987	E11.5 * Wnt1 up vs E11.5 * Ctrl
10355214	Idh1	NM_010497	1.07366e-005	1.37986	E11.5 * Wnt1 up vs E11.5 * Ctrl
10498775	Golim4	NM_175193	4.84611e-005	1.37969	E11.5 * Wnt1 up vs E11.5 * Ctrl
10383799	Tcn2	NM_015749	8.88861e-005	1.37967	E11.5 * Wnt1 up vs E11.5 * Ctrl
10398483	Dync1h1	NM_030238	2.83763e-005	1.37957	E11.5 * Wnt1 up vs E11.5 * Ctrl
10456378	Chmp1b	NM_024190	0.00691463	1.37911	E11.5 * Wnt1 up vs E11.5 * Ctrl
10409352	Rnf44	NR_027395	0.000403254	1.37906	E11.5 * Wnt1 up vs E11.5 * Ctrl
10511207	Cpsf3l	NM_028020	8.52342e-005	1.37886	E11.5 * Wnt1 up vs E11.5 * Ctrl
10468533	Gpam	NM_008149	7.49001e-005	1.37872	E11.5 * Wnt1 up vs E11.5 * Ctrl
10547540	Mical3	BC043122	0.00322093	1.37871	E11.5 * Wnt1 up vs E11.5 * Ctrl
10423629	Pop1	NM_152894	0.000415882	1.37804	E11.5 * Wnt1 up vs E11.5 * Ctrl
10363173	Gja1	NM_010288	1.22271e-005	1.37795	E11.5 * Wnt1 up vs E11.5 * Ctrl
10390175	Ngfr	NM_033217	0.000548954	1.37778	E11.5 * Wnt1 up vs E11.5 * Ctrl
10582981	Tfdp1	NM_009361	4.34318e-005	1.37723	E11.5 * Wnt1 up vs E11.5 * Ctrl
10424126	Depdc6	NM_001037937	0.000147334	1.37631	E11.5 * Wnt1 up vs E11.5 * Ctrl
10587627	Cyb5r4	NM_024195	0.000111379	1.37609	E11.5 * Wnt1 up vs E11.5 * Ctrl
10425578	Zc3h7b	NM_001081016	0.00868421	1.37542	E11.5 * Wnt1 up vs E11.5 * Ctrl
10540544	Thumpd3	NM_008188	3.853e-005	1.37476	E11.5 * Wnt1 up vs E11.5 * Ctrl
10355996	Slc25a5	NM_007451	3.38503e-005	1.37463	E11.5 * Wnt1 up vs E11.5 * Ctrl
10452000	Dpp9	NM_172624	0.00064183	1.37449	E11.5 * Wnt1 up vs E11.5 * Ctrl
10455901	Slc27a6	NM_001081072	0.00567942	1.37386	E11.5 * Wnt1 up vs E11.5 * Ctrl
10373571	1110012D08Rik	BC037624	0.00202063	1.3738	E11.5 * Wnt1 up vs E11.5 * Ctrl
10511810	Pou3f2	ENSMUST00000	0.00184536	1.37359	E11.5 * Wnt1 up vs E11.5 * Ctrl
10477649	Acss2	NM_019811	0.000284416	1.37357	E11.5 * Wnt1 up vs E11.5 * Ctrl

10392983	Slc25a19	NM_026071	0.000691638	1.37342	E11.5 * Wnt1 up vs E11.5 * Ctrl
10596583	Dock3	NM_153413	0.000646435	1.37322	E11.5 * Wnt1 up vs E11.5 * Ctrl
10344624	Lypla1	NM_008866	0.00114194	1.37305	E11.5 * Wnt1 up vs E11.5 * Ctrl
10489985	Atp9a	NM_015731	0.00505408	1.37304	E11.5 * Wnt1 up vs E11.5 * Ctrl
10566512		---	0.00105528	1.37268	E11.5 * Wnt1 up vs E11.5 * Ctrl
10504008	Chmp5	NM_029814	3.80947e-005	1.37262	E11.5 * Wnt1 up vs E11.5 * Ctrl
10514896	2210012G02Rik	NM_025617	0.00116723	1.37227	E11.5 * Wnt1 up vs E11.5 * Ctrl
10550638	Rtn2	NM_013648	1.55912e-005	1.372	E11.5 * Wnt1 up vs E11.5 * Ctrl
10593927	Scamp5	NM_020270	0.00325742	1.37196	E11.5 * Wnt1 up vs E11.5 * Ctrl
10575249	Txnl4b	NM_175646	0.00040437	1.37154	E11.5 * Wnt1 up vs E11.5 * Ctrl
10380986	Psmd3	NM_009439	0.000432377	1.3715	E11.5 * Wnt1 up vs E11.5 * Ctrl
10578027	Mak16	NM_026453	0.00247141	1.37117	E11.5 * Wnt1 up vs E11.5 * Ctrl
10567022	Btbd10	NM_133700	0.0025995	1.37117	E11.5 * Wnt1 up vs E11.5 * Ctrl
10535025	Got2	NM_010325	0.00182641	1.37082	E11.5 * Wnt1 up vs E11.5 * Ctrl
10476817	Atpaf1	NM_181040	0.00206203	1.37062	E11.5 * Wnt1 up vs E11.5 * Ctrl
10546163	Mcm2	NM_008564	0.00178943	1.37044	E11.5 * Wnt1 up vs E11.5 * Ctrl
10566804	Tmem9b	NM_020050	0.000799243	1.37035	E11.5 * Wnt1 up vs E11.5 * Ctrl
10558248	Bub3	NM_009774	2.18191e-006	1.37011	E11.5 * Wnt1 up vs E11.5 * Ctrl
10382376	Ttyh2	NM_053273	0.00204665	1.36956	E11.5 * Wnt1 up vs E11.5 * Ctrl
10413243	Duxbl	NM_183389	0.00345069	1.36953	E11.5 * Wnt1 up vs E11.5 * Ctrl
10525726	2810006K23Rik	NM_028310	0.00144745	1.36918	E11.5 * Wnt1 up vs E11.5 * Ctrl
10432404	Tuba1a	NM_011653	2.53276e-005	1.36835	E11.5 * Wnt1 up vs E11.5 * Ctrl
10427590	Slc1a3	NM_148938	0.00554116	1.36822	E11.5 * Wnt1 up vs E11.5 * Ctrl
10527133	Wipi2	NM_178398	0.000956918	1.36811	E11.5 * Wnt1 up vs E11.5 * Ctrl
10468309	2310014D11Rik	AK009333	0.000376917	1.36802	E11.5 * Wnt1 up vs E11.5 * Ctrl
10373740	Pik3ip1	NM_178149	0.0008263	1.36778	E11.5 * Wnt1 up vs E11.5 * Ctrl
10365729	Pctk2	NM_146239	7.34456e-006	1.36744	E11.5 * Wnt1 up vs E11.5 * Ctrl
10565315	Fah	NM_010176	0.00643238	1.36739	E11.5 * Wnt1 up vs E11.5 * Ctrl
10412921	Nid2	NM_008695	4.38529e-005	1.36737	E11.5 * Wnt1 up vs E11.5 * Ctrl
10430997	Pacsin2	NM_011862	0.000327482	1.36701	E11.5 * Wnt1 up vs E11.5 * Ctrl
10460879	Men1	NM_008583	0.00174367	1.36667	E11.5 * Wnt1 up vs E11.5 * Ctrl
10407222	Dhx29	NM_172594	0.000261181	1.36663	E11.5 * Wnt1 up vs E11.5 * Ctrl
10393387	Jmjd6	NM_033398	0.000130677	1.36609	E11.5 * Wnt1 up vs E11.5 * Ctrl
10430370	EG626952	XM_891575	0.00287482	1.36565	E11.5 * Wnt1 up vs E11.5 * Ctrl
10362922	Atg5	NM_053069	0.000223955	1.36539	E11.5 * Wnt1 up vs E11.5 * Ctrl
10538420	Gars	NM_180678	1.09844e-006	1.36532	E11.5 * Wnt1 up vs E11.5 * Ctrl
10410929	Agk	NM_023538	0.00249844	1.36528	E11.5 * Wnt1 up vs E11.5 * Ctrl
10376557	Nt5m	NM_134029	0.000849968	1.36524	E11.5 * Wnt1 up vs E11.5 * Ctrl
10503508	Ggh	NM_010281	0.000971046	1.36498	E11.5 * Wnt1 up vs E11.5 * Ctrl
10503523	Ggh	NM_010281	0.000971046	1.36498	E11.5 * Wnt1 up vs E11.5 * Ctrl
10372145	Alx1	NM_172553	0.00861982	1.36487	E11.5 * Wnt1 up vs E11.5 * Ctrl
10407955	Epdr1	NM_134065	0.00235543	1.36433	E11.5 * Wnt1 up vs E11.5 * Ctrl
10557705	Phkg2	NM_026888	0.000155285	1.36425	E11.5 * Wnt1 up vs E11.5 * Ctrl
10482824	Acvr1	NM_001110204	1.95781e-006	1.36412	E11.5 * Wnt1 up vs E11.5 * Ctrl
10364890	Sf3a2	NM_013651	0.0081757	1.36399	E11.5 * Wnt1 up vs E11.5 * Ctrl
10457853	Ino80c	NM_172625	0.00360158	1.36377	E11.5 * Wnt1 up vs E11.5 * Ctrl
10575550	Aars	NM_146217	6.78779e-005	1.36368	E11.5 * Wnt1 up vs E11.5 * Ctrl
10599215	Slc25a5	NM_007451	2.51365e-005	1.36358	E11.5 * Wnt1 up vs E11.5 * Ctrl
10394611	Nbas	ENSMUST00000	0.000137548	1.36239	E11.5 * Wnt1 up vs E11.5 * Ctrl
10458293	Dnajc18	NM_029669	0.000283066	1.36219	E11.5 * Wnt1 up vs E11.5 * Ctrl
10534974	Mcm7	NM_008568	0.00224244	1.36194	E11.5 * Wnt1 up vs E11.5 * Ctrl
10362941	Prep	NM_011156	0.000192149	1.36145	E11.5 * Wnt1 up vs E11.5 * Ctrl
10405729	Selk	NM_019979	0.0027417	1.3613	E11.5 * Wnt1 up vs E11.5 * Ctrl
10590983	Panx1	NM_019482	0.000454975	1.3611	E11.5 * Wnt1 up vs E11.5 * Ctrl
10413220	ENSMUSG0000C	ENSMUST00000	0.00225011	1.36099	E11.5 * Wnt1 up vs E11.5 * Ctrl
10398972	Mta1	NM_054081	0.00279745	1.36043	E11.5 * Wnt1 up vs E11.5 * Ctrl
10566516	Rrp8	NM_025897	0.00147793	1.35945	E11.5 * Wnt1 up vs E11.5 * Ctrl
10508420	Yars	NM_134151	0.000844864	1.3592	E11.5 * Wnt1 up vs E11.5 * Ctrl
10392207	Tex2	NM_198292	0.002766	1.35882	E11.5 * Wnt1 up vs E11.5 * Ctrl
10507379	Zswim5	NM_001029912	0.000682913	1.35881	E11.5 * Wnt1 up vs E11.5 * Ctrl
10403455	Dip2c	NM_001081426	0.000328348	1.35852	E11.5 * Wnt1 up vs E11.5 * Ctrl
10566067	Rnf121	NM_029211	0.00461269	1.35838	E11.5 * Wnt1 up vs E11.5 * Ctrl
10438262	Slc25a1	NM_153150	0.0024521	1.35837	E11.5 * Wnt1 up vs E11.5 * Ctrl
10426093	Zbed4	NM_181412	4.75127e-005	1.35834	E11.5 * Wnt1 up vs E11.5 * Ctrl
10428222	Ncald	NM_134094	0.000184952	1.35811	E11.5 * Wnt1 up vs E11.5 * Ctrl
10431410	Mapk11	NM_011161	0.00183073	1.35805	E11.5 * Wnt1 up vs E11.5 * Ctrl

10404840	Cd83	NM_009856	0.000310395	1.35774	E11.5 * Wnt1 up vs E11.5 * Ctrl
10536541	St7	NM_022332	0.00051759	1.35771	E11.5 * Wnt1 up vs E11.5 * Ctrl
10463211	Pi4k2a	NM_145501	0.0047606	1.35766	E11.5 * Wnt1 up vs E11.5 * Ctrl
10561527	Actn4	NM_021895	0.00293243	1.35749	E11.5 * Wnt1 up vs E11.5 * Ctrl
10449452	Fkbp5	NM_010220	0.00106333	1.3573	E11.5 * Wnt1 up vs E11.5 * Ctrl
10379564	Lig3	NM_010716	6.22034e-005	1.357	E11.5 * Wnt1 up vs E11.5 * Ctrl
10544462	Fam115a	NM_029930	0.000513696	1.3567	E11.5 * Wnt1 up vs E11.5 * Ctrl
10561004	Erf	NM_010155	0.000531904	1.35663	E11.5 * Wnt1 up vs E11.5 * Ctrl
10469613	Thnsl1	NM_177588	0.000775342	1.35651	E11.5 * Wnt1 up vs E11.5 * Ctrl
10563037	Scaf1	NM_001008422	0.00273568	1.35587	E11.5 * Wnt1 up vs E11.5 * Ctrl
10368011	Vta1	NM_025418	5.82879e-005	1.35572	E11.5 * Wnt1 up vs E11.5 * Ctrl
10411332	Hmgcr	NM_008255	0.00297317	1.35566	E11.5 * Wnt1 up vs E11.5 * Ctrl
10465282	Pola2	NM_008893	0.00367627	1.35548	E11.5 * Wnt1 up vs E11.5 * Ctrl
10382022	Ccdc44	NM_027346	0.00533901	1.35508	E11.5 * Wnt1 up vs E11.5 * Ctrl
10532538	Asphd2	NM_028386	0.00165655	1.35505	E11.5 * Wnt1 up vs E11.5 * Ctrl
10367843	Utrn	NM_011682	7.05258e-005	1.35485	E11.5 * Wnt1 up vs E11.5 * Ctrl
10387111	2310004I24Rik	NM_025510	0.00115733	1.35474	E11.5 * Wnt1 up vs E11.5 * Ctrl
10371230	Gna11	NM_010301	0.0013739	1.35449	E11.5 * Wnt1 up vs E11.5 * Ctrl
10579437	Ccdc124	NM_026964	0.00437373	1.35439	E11.5 * Wnt1 up vs E11.5 * Ctrl
10382701	Sap30bp	NM_020483	0.00233552	1.35438	E11.5 * Wnt1 up vs E11.5 * Ctrl
10464877	Dpp3	NM_133803	0.00106611	1.35433	E11.5 * Wnt1 up vs E11.5 * Ctrl
10589685	Lrrc2	NM_028838	0.00674175	1.35402	E11.5 * Wnt1 up vs E11.5 * Ctrl
10403466	Dip2c	NM_001081426	0.000456818	1.35374	E11.5 * Wnt1 up vs E11.5 * Ctrl
10585509	Fbxo22	NM_028049	0.00221322	1.35345	E11.5 * Wnt1 up vs E11.5 * Ctrl
10584941	Bace1	NM_011792	0.00155592	1.35332	E11.5 * Wnt1 up vs E11.5 * Ctrl
10477012	Fkbp1a	NM_008019	0.000125029	1.35326	E11.5 * Wnt1 up vs E11.5 * Ctrl
10365518	Nt5dc3	NM_175331	0.00610044	1.35307	E11.5 * Wnt1 up vs E11.5 * Ctrl
10516666	Txlna	NM_001005506	0.00036158	1.35305	E11.5 * Wnt1 up vs E11.5 * Ctrl
10600114	Pnma3	NM_153169	0.00449485	1.35303	E11.5 * Wnt1 up vs E11.5 * Ctrl
10556005	Ilk	NM_010562	3.3205e-005	1.35286	E11.5 * Wnt1 up vs E11.5 * Ctrl
10369877	Ube2d1	NM_145420	4.68162e-005	1.35273	E11.5 * Wnt1 up vs E11.5 * Ctrl
10367413	Dnajc14	NM_028873	0.000287872	1.35228	E11.5 * Wnt1 up vs E11.5 * Ctrl
10417183	Pcca	NM_144844	0.000410868	1.35218	E11.5 * Wnt1 up vs E11.5 * Ctrl
10529344	Poln	NM_181857	8.00559e-005	1.35207	E11.5 * Wnt1 up vs E11.5 * Ctrl
10386789	Ulk2	NM_013881	1.22857e-006	1.3519	E11.5 * Wnt1 up vs E11.5 * Ctrl
10544858	Chn2	NM_023543	0.00706296	1.35189	E11.5 * Wnt1 up vs E11.5 * Ctrl
10380415	Cdc34	BC094502	0.00469687	1.35175	E11.5 * Wnt1 up vs E11.5 * Ctrl
10381697	Hexim1	NM_138753	0.000165328	1.35141	E11.5 * Wnt1 up vs E11.5 * Ctrl
10510604	Dnajc11	NM_172704	0.00110251	1.35131	E11.5 * Wnt1 up vs E11.5 * Ctrl
10457205	Crem	NM_001110856	0.00318741	1.35122	E11.5 * Wnt1 up vs E11.5 * Ctrl
10547521	Atp6v1e1	NM_007510	0.00285241	1.35107	E11.5 * Wnt1 up vs E11.5 * Ctrl
10430201	Myh9	NM_022410	0.000488148	1.35102	E11.5 * Wnt1 up vs E11.5 * Ctrl
10575630	Cntnap4	NM_130457	0.00317229	1.35098	E11.5 * Wnt1 up vs E11.5 * Ctrl
10548745	Gpr19	NM_008157	0.0015665	1.35071	E11.5 * Wnt1 up vs E11.5 * Ctrl
10580986	Rbmxrt	NM_009033	9.62843e-005	1.35064	E11.5 * Wnt1 up vs E11.5 * Ctrl
10472136	Galnt13	NM_173030	0.00535693	1.35048	E11.5 * Wnt1 up vs E11.5 * Ctrl
10427026	Grasp	NM_019518	0.00319741	1.3503	E11.5 * Wnt1 up vs E11.5 * Ctrl
10425461	Adsl	NM_009634	3.31591e-005	1.35004	E11.5 * Wnt1 up vs E11.5 * Ctrl
10600082	Nsdhl	NM_010941	0.00324006	1.34918	E11.5 * Wnt1 up vs E11.5 * Ctrl
10558454	Glrx3	NM_023140	0.00212649	1.34867	E11.5 * Wnt1 up vs E11.5 * Ctrl
10575129	Vps4a	NM_126165	8.34407e-006	1.34777	E11.5 * Wnt1 up vs E11.5 * Ctrl
10405619	5133401N09Rik	NM_198004	0.00011999	1.34774	E11.5 * Wnt1 up vs E11.5 * Ctrl
10368918	Sobp	NM_175407	0.000208555	1.34752	E11.5 * Wnt1 up vs E11.5 * Ctrl
10345357	Imp4	NM_178601	0.000587826	1.34713	E11.5 * Wnt1 up vs E11.5 * Ctrl
10511803	2610029I01Rik	ENSMUST00000	0.00301391	1.34685	E11.5 * Wnt1 up vs E11.5 * Ctrl
10590343	Trak1	NM_175114	0.00195993	1.34664	E11.5 * Wnt1 up vs E11.5 * Ctrl
10433735	Abcc1	NM_008576	0.000860165	1.34573	E11.5 * Wnt1 up vs E11.5 * Ctrl
10375083	Stk10	NM_009288	0.00016247	1.34559	E11.5 * Wnt1 up vs E11.5 * Ctrl
10380761	Socs7	NM_138657	0.000456338	1.34558	E11.5 * Wnt1 up vs E11.5 * Ctrl
10364824	Csnk1g2	NM_134002	0.00812119	1.34554	E11.5 * Wnt1 up vs E11.5 * Ctrl
10512391	Vcp	NM_009503	2.89417e-006	1.34554	E11.5 * Wnt1 up vs E11.5 * Ctrl
10519203	A230069A22Rik	NM_001033394	0.00280513	1.34518	E11.5 * Wnt1 up vs E11.5 * Ctrl
10514576	Kank4	NM_172872	0.00123561	1.34491	E11.5 * Wnt1 up vs E11.5 * Ctrl
10363669	Dnajc12	NM_013888	0.00140451	1.3449	E11.5 * Wnt1 up vs E11.5 * Ctrl
10464836	Actn3	NM_013456	5.27909e-005	1.34489	E11.5 * Wnt1 up vs E11.5 * Ctrl
10431113	Sult4a1	NM_013873	0.00252627	1.34476	E11.5 * Wnt1 up vs E11.5 * Ctrl

10378649	Slc43a2	NM_173388	0.00128359	1.34469	E11.5 * Wnt1 up vs E11.5 * Ctrl
10401891	Ston2	NM_175367	0.000938364	1.34468	E11.5 * Wnt1 up vs E11.5 * Ctrl
10384936	Acyp2	NM_029344	0.000224825	1.34452	E11.5 * Wnt1 up vs E11.5 * Ctrl
10524621	Oasl2	NM_011854	0.000268949	1.34431	E11.5 * Wnt1 up vs E11.5 * Ctrl
10525751	Ddx55	NM_026409	0.000654386	1.34425	E11.5 * Wnt1 up vs E11.5 * Ctrl
10579830	Rbmxrt	NM_009033	5.42091e-006	1.34416	E11.5 * Wnt1 up vs E11.5 * Ctrl
10535184	Psmg3	NM_025604	0.00246332	1.34395	E11.5 * Wnt1 up vs E11.5 * Ctrl
10376094		---	3.72835e-005	1.34369	E11.5 * Wnt1 up vs E11.5 * Ctrl
10507203	Atpaf1	NM_181040	0.00163059	1.34364	E11.5 * Wnt1 up vs E11.5 * Ctrl
10592099	Dcps	NM_027030	0.00753012	1.34358	E11.5 * Wnt1 up vs E11.5 * Ctrl
10375038	Mpg	NM_010822	0.0072851	1.34357	E11.5 * Wnt1 up vs E11.5 * Ctrl
10607317	Tsr2	NM_175146	0.00314853	1.34341	E11.5 * Wnt1 up vs E11.5 * Ctrl
10364194	Lss	NM_146006	0.000922339	1.34338	E11.5 * Wnt1 up vs E11.5 * Ctrl
10499045	Trim2	NM_030706	5.43574e-005	1.34323	E11.5 * Wnt1 up vs E11.5 * Ctrl
10541131	Dcp1b	NM_001033379	0.00427585	1.34315	E11.5 * Wnt1 up vs E11.5 * Ctrl
10394627	Nbas	BC057020	6.52055e-006	1.34309	E11.5 * Wnt1 up vs E11.5 * Ctrl
10544199	Dennd2a	NM_172477	0.0024988	1.34295	E11.5 * Wnt1 up vs E11.5 * Ctrl
10390653	Med24	NM_011869	1.40521e-005	1.34287	E11.5 * Wnt1 up vs E11.5 * Ctrl
10400805	Nin	NM_008697	6.43754e-006	1.34272	E11.5 * Wnt1 up vs E11.5 * Ctrl
10386604	Shmt1	NM_009171	0.00382961	1.34248	E11.5 * Wnt1 up vs E11.5 * Ctrl
10468049	Poll	NM_020032	0.00109939	1.34165	E11.5 * Wnt1 up vs E11.5 * Ctrl
10448707	Tbl3	NM_145396	0.000658326	1.34138	E11.5 * Wnt1 up vs E11.5 * Ctrl
10406934	Etv1	NM_007960	0.000621696	1.34117	E11.5 * Wnt1 up vs E11.5 * Ctrl
10377889	Mink1	NM_001045959	0.00144242	1.34074	E11.5 * Wnt1 up vs E11.5 * Ctrl
10449018	Haghl	NM_026897	0.00423646	1.34063	E11.5 * Wnt1 up vs E11.5 * Ctrl
10560315	Ppp5c	NM_011155	0.00178365	1.34051	E11.5 * Wnt1 up vs E11.5 * Ctrl
10512226	Wdr40a	NM_026893	0.00115132	1.33979	E11.5 * Wnt1 up vs E11.5 * Ctrl
10516544	Hpca	NM_010471	0.00414767	1.33904	E11.5 * Wnt1 up vs E11.5 * Ctrl
10417065	Rap2a	NM_029519	5.56392e-005	1.33864	E11.5 * Wnt1 up vs E11.5 * Ctrl
10545720	Stambp	NM_024239	0.00083791	1.33812	E11.5 * Wnt1 up vs E11.5 * Ctrl
10358928	Cacna1e	NM_009782	0.00158694	1.33778	E11.5 * Wnt1 up vs E11.5 * Ctrl
10569569	Cttn	NM_007803	0.00704198	1.33778	E11.5 * Wnt1 up vs E11.5 * Ctrl
10533386	Sh2b3	NM_008507	0.000238924	1.33725	E11.5 * Wnt1 up vs E11.5 * Ctrl
10544891	Nod1	NM_172729	0.000597386	1.33711	E11.5 * Wnt1 up vs E11.5 * Ctrl
10484261	Cerkl	NM_001048176	0.00305244	1.33702	E11.5 * Wnt1 up vs E11.5 * Ctrl
10484888	Ptprj	NM_008982	0.0069577	1.33696	E11.5 * Wnt1 up vs E11.5 * Ctrl
10456400	Tubb6	NM_026473	1.73559e-005	1.33695	E11.5 * Wnt1 up vs E11.5 * Ctrl
10559312	Dhcr7	NM_007856	0.00290375	1.33643	E11.5 * Wnt1 up vs E11.5 * Ctrl
10507238	Lrrc41	NM_153521	0.00222346	1.33639	E11.5 * Wnt1 up vs E11.5 * Ctrl
10430593	Josd1	NM_028792	0.000382991	1.33622	E11.5 * Wnt1 up vs E11.5 * Ctrl
10358672	Hmcn1	NM_001024720	0.00330153	1.33601	E11.5 * Wnt1 up vs E11.5 * Ctrl
10477644	Trp53inp2	NM_178111	0.000950403	1.33601	E11.5 * Wnt1 up vs E11.5 * Ctrl
10542522	Plekha5	NM_144920	0.000198672	1.3354	E11.5 * Wnt1 up vs E11.5 * Ctrl
10544243	Mrps33	NM_010270	0.000148057	1.33535	E11.5 * Wnt1 up vs E11.5 * Ctrl
10477897	Dlgap4	NM_146128	2.06724e-008	1.33534	E11.5 * Wnt1 up vs E11.5 * Ctrl
10488303	Crnkl1	NM_025820	0.000517292	1.33453	E11.5 * Wnt1 up vs E11.5 * Ctrl
10407742	Actn2	NM_033268	5.89629e-005	1.33331	E11.5 * Wnt1 up vs E11.5 * Ctrl
10516974	Ppp1r8	NM_146154	0.00128876	1.33301	E11.5 * Wnt1 up vs E11.5 * Ctrl
10455656	Hsd17b4	NM_008292	3.65408e-006	1.33299	E11.5 * Wnt1 up vs E11.5 * Ctrl
10544186	Mkrn1	NM_018810	8.65072e-005	1.33267	E11.5 * Wnt1 up vs E11.5 * Ctrl
10439936	Nfkbiz	NM_030612	0.000188724	1.33256	E11.5 * Wnt1 up vs E11.5 * Ctrl
10525766	Gtf2h3	NM_181410	0.0023215	1.332	E11.5 * Wnt1 up vs E11.5 * Ctrl
10367673	Plekhg1	NM_001033253	0.00700658	1.33197	E11.5 * Wnt1 up vs E11.5 * Ctrl
10433953	Ypel1	NM_023249	0.000542528	1.33195	E11.5 * Wnt1 up vs E11.5 * Ctrl
10483786	Mrpl23	NM_011288	0.00571791	1.3318	E11.5 * Wnt1 up vs E11.5 * Ctrl
10597627	Oxsr1	NM_133985	0.000142326	1.33106	E11.5 * Wnt1 up vs E11.5 * Ctrl
10594551	Zfp609	NM_172536	0.000340252	1.33079	E11.5 * Wnt1 up vs E11.5 * Ctrl
10455372	C330007P06Rik	NM_029951	0.00225402	1.33074	E11.5 * Wnt1 up vs E11.5 * Ctrl
10415875	2610028A01Rik	NM_028228	0.00819546	1.33071	E11.5 * Wnt1 up vs E11.5 * Ctrl
10543551	Rbm28	NM_133925	7.82512e-005	1.33048	E11.5 * Wnt1 up vs E11.5 * Ctrl
10580969	Got2	NM_010325	0.00122207	1.33034	E11.5 * Wnt1 up vs E11.5 * Ctrl
10468762	4930506M07Rik	NM_001114312	0.00615352	1.33012	E11.5 * Wnt1 up vs E11.5 * Ctrl
10540168	Fgd5	NM_172731	0.000111827	1.32992	E11.5 * Wnt1 up vs E11.5 * Ctrl
10493193	Cct3	NM_009836	0.000226723	1.32979	E11.5 * Wnt1 up vs E11.5 * Ctrl
10533875	Ncor2	NM_011424	0.00117614	1.32966	E11.5 * Wnt1 up vs E11.5 * Ctrl
10596951	Arih2	NM_011790	0.0010099	1.32962	E11.5 * Wnt1 up vs E11.5 * Ctrl

10575184	Wwp2	NM_025830	0.00187393	1.3293	E11.5 * Wnt1 up vs E11.5 * Ctrl
10471247	Aif1l	NM_145144	0.00744673	1.329	E11.5 * Wnt1 up vs E11.5 * Ctrl
10605338	G6pdx	NM_008062	0.00562759	1.32891	E11.5 * Wnt1 up vs E11.5 * Ctrl
10540880	Syn2	NM_001111015	0.00124604	1.32845	E11.5 * Wnt1 up vs E11.5 * Ctrl
10524723	Gcn1l1	NM_172719	0.00471965	1.32804	E11.5 * Wnt1 up vs E11.5 * Ctrl
10480999	Sdccag3	NM_026563	0.000466334	1.32735	E11.5 * Wnt1 up vs E11.5 * Ctrl
10399178	Cdca7l	NM_146040	0.00428813	1.32733	E11.5 * Wnt1 up vs E11.5 * Ctrl
10389010	5730455P16Rik	NM_027472	0.00575759	1.32729	E11.5 * Wnt1 up vs E11.5 * Ctrl
10465625	Otub1	NM_134150	0.00149892	1.32714	E11.5 * Wnt1 up vs E11.5 * Ctrl
10433672	Rrn3	NM_001039521	7.06876e-005	1.32711	E11.5 * Wnt1 up vs E11.5 * Ctrl
10554370	Zfp710	NM_175433	0.00639078	1.32698	E11.5 * Wnt1 up vs E11.5 * Ctrl
10536611	Kcnd2	NM_019697	0.00807463	1.32588	E11.5 * Wnt1 up vs E11.5 * Ctrl
10559233	Mrpl23	NM_011288	0.00488707	1.32588	E11.5 * Wnt1 up vs E11.5 * Ctrl
10369295	D10Ertd641e	NM_025514	0.000371196	1.32587	E11.5 * Wnt1 up vs E11.5 * Ctrl
10474073	C230071H18Rik	BC147668	0.00189116	1.32556	E11.5 * Wnt1 up vs E11.5 * Ctrl
10468517	Mxi1	NM_010847	0.00491943	1.3255	E11.5 * Wnt1 up vs E11.5 * Ctrl
10369086	Gopc	NM_053187	1.90918e-006	1.32529	E11.5 * Wnt1 up vs E11.5 * Ctrl
10528507	Pus7	NM_178403	0.00036366	1.32486	E11.5 * Wnt1 up vs E11.5 * Ctrl
10350823	Abl2	NM_001136104	0.000392653	1.32477	E11.5 * Wnt1 up vs E11.5 * Ctrl
10391768	Eftud2	NM_011431	0.000991914	1.32454	E11.5 * Wnt1 up vs E11.5 * Ctrl
10487392	Kcnip3	NM_019789	0.00408644	1.32441	E11.5 * Wnt1 up vs E11.5 * Ctrl
10452257	Slc25a23	NM_025877	0.0059641	1.3243	E11.5 * Wnt1 up vs E11.5 * Ctrl
10561854	Tbcb	NM_025548	0.000590805	1.32422	E11.5 * Wnt1 up vs E11.5 * Ctrl
10485361	B230118H07Rik	NM_026592	0.000882296	1.32412	E11.5 * Wnt1 up vs E11.5 * Ctrl
10358754	EG639787	XR_002131	0.00559718	1.32401	E11.5 * Wnt1 up vs E11.5 * Ctrl
10471675	Glo1	NM_025374	0.00488116	1.32388	E11.5 * Wnt1 up vs E11.5 * Ctrl
10389680	Msi2	NM_054043	9.70153e-005	1.32369	E11.5 * Wnt1 up vs E11.5 * Ctrl
10511149	Mrpl20	NM_025570	0.00353918	1.32338	E11.5 * Wnt1 up vs E11.5 * Ctrl
10546855	Srgap3	NM_080448	2.2853e-005	1.32331	E11.5 * Wnt1 up vs E11.5 * Ctrl
10584954	Pcsk7	NM_008794	0.000261804	1.3233	E11.5 * Wnt1 up vs E11.5 * Ctrl
10545910	Pcyox1	NM_025823	0.000341282	1.32306	E11.5 * Wnt1 up vs E11.5 * Ctrl
10402665	Cdc42bpb	NM_183016	0.00255098	1.32296	E11.5 * Wnt1 up vs E11.5 * Ctrl
10383289	Baiap2	NM_130862	0.00183077	1.32287	E11.5 * Wnt1 up vs E11.5 * Ctrl
10578679	ENSMUSG0000C	ENSMUST00000	0.000695949	1.32277	E11.5 * Wnt1 up vs E11.5 * Ctrl
10446470	ENSMUSG0000C	ENSMUST00000	0.00429795	1.32275	E11.5 * Wnt1 up vs E11.5 * Ctrl
10522075	Klhl5	NM_175174	7.65876e-005	1.32201	E11.5 * Wnt1 up vs E11.5 * Ctrl
10512291	Dctn3	NM_016890	0.000142565	1.32198	E11.5 * Wnt1 up vs E11.5 * Ctrl
10523012	Dck	NM_007832	0.00451713	1.32187	E11.5 * Wnt1 up vs E11.5 * Ctrl
10587604	Rwdd2a	NM_027100	0.00449208	1.32173	E11.5 * Wnt1 up vs E11.5 * Ctrl
10386230	Rnf187	AB030190	0.0019351	1.32162	E11.5 * Wnt1 up vs E11.5 * Ctrl
10414256	Rbmxrt	NM_009033	1.16271e-005	1.32121	E11.5 * Wnt1 up vs E11.5 * Ctrl
10460573	Eif1ad	NM_027236	0.000337937	1.3212	E11.5 * Wnt1 up vs E11.5 * Ctrl
10421214	Rhobtb2	NM_153514	0.00205144	1.32108	E11.5 * Wnt1 up vs E11.5 * Ctrl
10352396	Trp53bp2	NM_173378	3.06145e-005	1.32085	E11.5 * Wnt1 up vs E11.5 * Ctrl
10363498	Ppa1	NM_026438	1.56535e-005	1.32062	E11.5 * Wnt1 up vs E11.5 * Ctrl
10471505	Sh2d3c	NM_013781	0.00161621	1.32052	E11.5 * Wnt1 up vs E11.5 * Ctrl
10351525	Mpz	NM_008623	0.00171696	1.32019	E11.5 * Wnt1 up vs E11.5 * Ctrl
10593130	Sidt2	NM_172257	0.000521546	1.32007	E11.5 * Wnt1 up vs E11.5 * Ctrl
10473880	Lrp4	NM_172668	0.00126578	1.31971	E11.5 * Wnt1 up vs E11.5 * Ctrl
10564220	Gkap1	NM_019832	0.00727726	1.31949	E11.5 * Wnt1 up vs E11.5 * Ctrl
10399636	Mrto4	NM_023536	0.000469138	1.31943	E11.5 * Wnt1 up vs E11.5 * Ctrl
10435162	Lrch3	NM_001081255	9.52305e-005	1.31937	E11.5 * Wnt1 up vs E11.5 * Ctrl
10375065	Sh3pxd2b	NM_177364	0.000143558	1.3191	E11.5 * Wnt1 up vs E11.5 * Ctrl
10559406	Tfpt	NM_023524	0.00844021	1.3191	E11.5 * Wnt1 up vs E11.5 * Ctrl
10506643	Tmem59	NM_029565	0.000149507	1.31904	E11.5 * Wnt1 up vs E11.5 * Ctrl
10602401	Fgd1	NM_008001	0.00134555	1.31901	E11.5 * Wnt1 up vs E11.5 * Ctrl
10592790	Hinfp	NM_172162	0.00205131	1.31898	E11.5 * Wnt1 up vs E11.5 * Ctrl
10522250	Tmem33	NM_028975	0.00034208	1.31874	E11.5 * Wnt1 up vs E11.5 * Ctrl
10354233	Tgfbrap1	NM_001013025	0.00296313	1.31864	E11.5 * Wnt1 up vs E11.5 * Ctrl
10462113	Apba1	NM_177034	0.00532018	1.31831	E11.5 * Wnt1 up vs E11.5 * Ctrl
10563303	Bax	NM_007527	0.00248124	1.31822	E11.5 * Wnt1 up vs E11.5 * Ctrl
10402715	Bag5	NM_027404	0.000254373	1.31811	E11.5 * Wnt1 up vs E11.5 * Ctrl
10537712	Gstk1	NM_029555	0.0043518	1.31809	E11.5 * Wnt1 up vs E11.5 * Ctrl
10353899	Sema4c	NM_001126047	0.00346942	1.31809	E11.5 * Wnt1 up vs E11.5 * Ctrl
10402650	2810452K22Rik	NM_026048	0.000842191	1.31792	E11.5 * Wnt1 up vs E11.5 * Ctrl
10505894	Mtap	NM_024433	0.000238859	1.31762	E11.5 * Wnt1 up vs E11.5 * Ctrl

10553993	Snrpa1	NM_021336	1.14049e-005	1.31747	E11.5 * Wnt1 up vs E11.5 * Ctrl
10399430	Ddx1	NM_134040	2.72074e-006	1.3173	E11.5 * Wnt1 up vs E11.5 * Ctrl
10510464	Lzic	NM_026963	0.000447788	1.31693	E11.5 * Wnt1 up vs E11.5 * Ctrl
10510270	Mthfr	NM_010840	1.65914e-005	1.31662	E11.5 * Wnt1 up vs E11.5 * Ctrl
10450579	Ddr1	NM_007584	0.00754415	1.31629	E11.5 * Wnt1 up vs E11.5 * Ctrl
10474411	Lin7c	NM_011699	1.07223e-007	1.31628	E11.5 * Wnt1 up vs E11.5 * Ctrl
10461844	Gnaq	NM_008139	0.000615467	1.31608	E11.5 * Wnt1 up vs E11.5 * Ctrl
10590445	Snrk	NM_133741	0.000889545	1.31602	E11.5 * Wnt1 up vs E11.5 * Ctrl
10365601	Gnptab	NM_001004164	0.000408071	1.31599	E11.5 * Wnt1 up vs E11.5 * Ctrl
10457844	Zfp191	NM_021559	9.85048e-006	1.31578	E11.5 * Wnt1 up vs E11.5 * Ctrl
10454851	Cxxc5	NM_133687	0.00164821	1.3157	E11.5 * Wnt1 up vs E11.5 * Ctrl
10557432	G730046D07Rik	AK144875	0.000434789	1.31567	E11.5 * Wnt1 up vs E11.5 * Ctrl
10531899	Klhl8	NM_178741	0.00121494	1.31562	E11.5 * Wnt1 up vs E11.5 * Ctrl
10578149	Leprotl1	NM_026609	5.88828e-006	1.31538	E11.5 * Wnt1 up vs E11.5 * Ctrl
10442816	Lmf1	NM_029624	0.000945811	1.3153	E11.5 * Wnt1 up vs E11.5 * Ctrl
10357579	Mapkapk2	NM_008551	0.00778428	1.31501	E11.5 * Wnt1 up vs E11.5 * Ctrl
10376959	Elac2	NM_023479	0.000876547	1.3147	E11.5 * Wnt1 up vs E11.5 * Ctrl
10454564	Ercc3	NM_133658	0.000134182	1.31455	E11.5 * Wnt1 up vs E11.5 * Ctrl
10551760	Zfp84	NM_023750	0.000384808	1.31447	E11.5 * Wnt1 up vs E11.5 * Ctrl
10563829	Mrps33	NM_010270	0.000161401	1.31445	E11.5 * Wnt1 up vs E11.5 * Ctrl
10530806	Ppat	BC023841	8.86828e-006	1.31442	E11.5 * Wnt1 up vs E11.5 * Ctrl
10598638	Mid1ip1	NM_026524	6.20598e-005	1.31406	E11.5 * Wnt1 up vs E11.5 * Ctrl
10466304	Dtx4	NM_172442	0.000358445	1.31394	E11.5 * Wnt1 up vs E11.5 * Ctrl
10589041	Impdh2	NM_011830	0.000347157	1.3138	E11.5 * Wnt1 up vs E11.5 * Ctrl
10510809	A430005L14Rik	BC052148	0.00469419	1.31332	E11.5 * Wnt1 up vs E11.5 * Ctrl
10500412	Gpr89	NM_026229	0.000104167	1.31317	E11.5 * Wnt1 up vs E11.5 * Ctrl
10476314	Prnp	NM_011170	0.000300037	1.31239	E11.5 * Wnt1 up vs E11.5 * Ctrl
10465342	Tm7sf2	NM_028454	0.00203248	1.31215	E11.5 * Wnt1 up vs E11.5 * Ctrl
10522661	C530008M17Rik	AK122469	0.00296571	1.31196	E11.5 * Wnt1 up vs E11.5 * Ctrl
10431874	Slc38a1	NM_134086	0.000146143	1.3117	E11.5 * Wnt1 up vs E11.5 * Ctrl
10364559	Arid3a	NM_007880	0.0038646	1.31142	E11.5 * Wnt1 up vs E11.5 * Ctrl
10373036	Os9	NM_177614	0.00420082	1.31138	E11.5 * Wnt1 up vs E11.5 * Ctrl
10598493	Pcsk1n	NM_013892	0.000915362	1.31114	E11.5 * Wnt1 up vs E11.5 * Ctrl
10463448	Peo1	NM_153796	0.00635841	1.31114	E11.5 * Wnt1 up vs E11.5 * Ctrl
10481577	Uck1	NM_011675	0.00177679	1.31107	E11.5 * Wnt1 up vs E11.5 * Ctrl
10432256	Arf3	NM_007478	0.000877544	1.31089	E11.5 * Wnt1 up vs E11.5 * Ctrl
10434467	Psmd2	NR_027485	0.000905541	1.3108	E11.5 * Wnt1 up vs E11.5 * Ctrl
10579860	Smad1	NM_008539	5.28424e-005	1.31058	E11.5 * Wnt1 up vs E11.5 * Ctrl
10581940	Kars	NM_001130868	0.0003297	1.31022	E11.5 * Wnt1 up vs E11.5 * Ctrl
10360349	Cadm3	NM_053199	0.00441589	1.30973	E11.5 * Wnt1 up vs E11.5 * Ctrl
10585417	Idh3a	NM_029573	0.00505809	1.30946	E11.5 * Wnt1 up vs E11.5 * Ctrl
10586347	Parp16	NM_177460	0.00209993	1.30946	E11.5 * Wnt1 up vs E11.5 * Ctrl
10540391	Trnt1	NM_027296	0.00344667	1.30944	E11.5 * Wnt1 up vs E11.5 * Ctrl
10417787	Gng2	NM_010315	0.000145266	1.30922	E11.5 * Wnt1 up vs E11.5 * Ctrl
10473737	Mtch2	NM_019758	0.000255047	1.30914	E11.5 * Wnt1 up vs E11.5 * Ctrl
10382802	Sphk1	NM_011451	0.00129122	1.30872	E11.5 * Wnt1 up vs E11.5 * Ctrl
10500685	Atp1a1	NM_144900	0.000271005	1.30849	E11.5 * Wnt1 up vs E11.5 * Ctrl
10606835	Bex2	NM_009749	0.00607926	1.30842	E11.5 * Wnt1 up vs E11.5 * Ctrl
10471062	Mettl11a	NM_170592	0.00373548	1.30789	E11.5 * Wnt1 up vs E11.5 * Ctrl
10501402	Gpsm2	NM_029522	0.00279107	1.30776	E11.5 * Wnt1 up vs E11.5 * Ctrl
10478424	Ywhab	NM_018753	9.46588e-007	1.30757	E11.5 * Wnt1 up vs E11.5 * Ctrl
10505109	BC026590	BC026590	0.00167948	1.3074	E11.5 * Wnt1 up vs E11.5 * Ctrl
10374364	Akt2	NM_001110208	0.00352581	1.3074	E11.5 * Wnt1 up vs E11.5 * Ctrl
10392098	Ftsj3	NM_025310	0.000262127	1.30696	E11.5 * Wnt1 up vs E11.5 * Ctrl
10381345	Psme3	NM_011192	0.00054032	1.3069	E11.5 * Wnt1 up vs E11.5 * Ctrl
10481621	1110008P14Rik	NM_198001	0.00089615	1.30671	E11.5 * Wnt1 up vs E11.5 * Ctrl
10516652	Iqcc	NM_198026	0.0069566	1.30656	E11.5 * Wnt1 up vs E11.5 * Ctrl
10511139	Ssu72	NM_026899	0.00061262	1.30622	E11.5 * Wnt1 up vs E11.5 * Ctrl
10345423	Plekhb2	NM_145516	0.00134519	1.30596	E11.5 * Wnt1 up vs E11.5 * Ctrl
10530319	Atp8a1	NM_001038999	1.14652e-005	1.30573	E11.5 * Wnt1 up vs E11.5 * Ctrl
10384192	Tbrg4	NM_134011	2.79475e-005	1.3056	E11.5 * Wnt1 up vs E11.5 * Ctrl
10578922	Klhl2	NM_178633	0.00315602	1.30544	E11.5 * Wnt1 up vs E11.5 * Ctrl
10543080	Rnps1	NM_009070	0.00204496	1.30496	E11.5 * Wnt1 up vs E11.5 * Ctrl
10350848	2810025M15Rik	BC055845	0.000468595	1.30488	E11.5 * Wnt1 up vs E11.5 * Ctrl
10345025	Iars	NM_172015	0.00421237	1.30488	E11.5 * Wnt1 up vs E11.5 * Ctrl
10358379	Trove2	NM_013835	0.00354318	1.30482	E11.5 * Wnt1 up vs E11.5 * Ctrl

10434252	Hira	NM_010435	0.000645717	1.30452	E11.5 * Wnt1 up vs E11.5 * Ctrl
10442435	Rnps1	NM_009070	0.00140789	1.30425	E11.5 * Wnt1 up vs E11.5 * Ctrl
10437040	Chaf1b	NM_028083	0.00193019	1.30412	E11.5 * Wnt1 up vs E11.5 * Ctrl
10496417	Rg9mtd2	NM_175389	0.00391923	1.30385	E11.5 * Wnt1 up vs E11.5 * Ctrl
10500295	Plekho1	NM_023320	0.000428316	1.3036	E11.5 * Wnt1 up vs E11.5 * Ctrl
10362904	Rtn4ip1	NM_130892	0.00332613	1.3035	E11.5 * Wnt1 up vs E11.5 * Ctrl
10460626	Rnaseh2c	NM_026616	0.00300855	1.30307	E11.5 * Wnt1 up vs E11.5 * Ctrl
10482109	Rbm18	NR_027515	0.000328383	1.30278	E11.5 * Wnt1 up vs E11.5 * Ctrl
10520096	Klhl7	NM_026448	0.00252666	1.3027	E11.5 * Wnt1 up vs E11.5 * Ctrl
10438098	Sdf2l1	NM_022324	0.00153566	1.30253	E11.5 * Wnt1 up vs E11.5 * Ctrl
10400157	Nova1	ENSMUST00000	0.00210575	1.30237	E11.5 * Wnt1 up vs E11.5 * Ctrl
10373577	Ormdl2	NM_024180	0.00356115	1.30208	E11.5 * Wnt1 up vs E11.5 * Ctrl
10435802	2610015P09Rik	ENSMUST00000	0.00746921	1.30205	E11.5 * Wnt1 up vs E11.5 * Ctrl
10589438	Mtap4	NM_008633	0.000138326	1.3018	E11.5 * Wnt1 up vs E11.5 * Ctrl
10562578	Pop4	NM_025390	0.00281091	1.30177	E11.5 * Wnt1 up vs E11.5 * Ctrl
10418300	Cacna2d3	NM_009785	0.00514107	1.30173	E11.5 * Wnt1 up vs E11.5 * Ctrl
10404578	Cdyl	NM_009881	0.000221604	1.30172	E11.5 * Wnt1 up vs E11.5 * Ctrl
10569203	Chid1	NM_001142681	0.00140483	1.30165	E11.5 * Wnt1 up vs E11.5 * Ctrl
10434029	Lztr1	NM_025808	0.000783345	1.30161	E11.5 * Wnt1 up vs E11.5 * Ctrl
10376482	2310033P09Rik	BC002181	7.80796e-005	1.3014	E11.5 * Wnt1 up vs E11.5 * Ctrl
10417813	Ecd	NM_027475	2.5182e-005	1.30103	E11.5 * Wnt1 up vs E11.5 * Ctrl
10391918	Plekhm1	NM_183034	0.0069907	1.30096	E11.5 * Wnt1 up vs E11.5 * Ctrl
10391577	Hdac5	NM_001077696	0.00210674	1.30068	E11.5 * Wnt1 up vs E11.5 * Ctrl
10346810	Pard3b	NM_001081050	0.00218068	1.30053	E11.5 * Wnt1 up vs E11.5 * Ctrl
10515007	Gpx7	NM_024198	0.00374567	1.30045	E11.5 * Wnt1 up vs E11.5 * Ctrl
10402096	Ttc7b	NM_001033213	0.000309333	1.30038	E11.5 * Wnt1 up vs E11.5 * Ctrl
10457585		---	0.00861153	1.30007	E11.5 * Wnt1 up vs E11.5 * Ctrl
10475630	Galk2	NM_175154	0.00302796	1.29982	E11.5 * Wnt1 up vs E11.5 * Ctrl
10574033	Nup93	NM_172410	1.67448e-007	1.29956	E11.5 * Wnt1 up vs E11.5 * Ctrl
10426479	Ano6	NM_175344	0.000726668	1.2994	E11.5 * Wnt1 up vs E11.5 * Ctrl
10586130	2310007F21Rik	BC027311	0.00339001	1.29937	E11.5 * Wnt1 up vs E11.5 * Ctrl
10416256	Bin3	NM_021328	0.00864002	1.29934	E11.5 * Wnt1 up vs E11.5 * Ctrl
10558921	Pnpla2	NM_025802	0.00206169	1.29918	E11.5 * Wnt1 up vs E11.5 * Ctrl
10578810	Clcn3	NM_173874	0.000265919	1.29882	E11.5 * Wnt1 up vs E11.5 * Ctrl
10529273	Ctbp1	NM_013502	0.00365171	1.29846	E11.5 * Wnt1 up vs E11.5 * Ctrl
10515295	Mast2	NM_001042743	4.62853e-005	1.29831	E11.5 * Wnt1 up vs E11.5 * Ctrl
10568099	Tmem219	NM_026827	0.00139017	1.29821	E11.5 * Wnt1 up vs E11.5 * Ctrl
10378768	Rnmtl1	NM_183263	0.00153263	1.29809	E11.5 * Wnt1 up vs E11.5 * Ctrl
10459241	Afap1l1	NM_178928	0.00188434	1.29808	E11.5 * Wnt1 up vs E11.5 * Ctrl
10548585	Csda	NM_139117	0.00203315	1.29779	E11.5 * Wnt1 up vs E11.5 * Ctrl
10469581	Etl4	NM_001081006	9.90031e-005	1.29766	E11.5 * Wnt1 up vs E11.5 * Ctrl
10465246	Frmd8	NM_026169	0.0061976	1.29764	E11.5 * Wnt1 up vs E11.5 * Ctrl
10576010	Gse1	NM_198671	0.00829653	1.29763	E11.5 * Wnt1 up vs E11.5 * Ctrl
10581625	2400003C14Rik	BC039052	0.00226958	1.29706	E11.5 * Wnt1 up vs E11.5 * Ctrl
10435676	Gsk3b	NM_019827	2.5672e-005	1.29698	E11.5 * Wnt1 up vs E11.5 * Ctrl
10493108	Crabp2	NM_007759	0.0015566	1.29675	E11.5 * Wnt1 up vs E11.5 * Ctrl
10508412	Fndc5	NM_027402	0.00413644	1.29654	E11.5 * Wnt1 up vs E11.5 * Ctrl
10477725	Mmp24	NM_010808	0.000748136	1.29627	E11.5 * Wnt1 up vs E11.5 * Ctrl
10526726	Zkscan1	NM_133906	9.98534e-005	1.29624	E11.5 * Wnt1 up vs E11.5 * Ctrl
10504703	AU014645	NM_001033201	0.000148849	1.29618	E11.5 * Wnt1 up vs E11.5 * Ctrl
10366026	Wdr51b	NM_027740	0.00861883	1.29612	E11.5 * Wnt1 up vs E11.5 * Ctrl
10498064	Setd7	NM_080793	0.000588121	1.2961	E11.5 * Wnt1 up vs E11.5 * Ctrl
10373313	Nab2	NM_008668	0.00826555	1.29588	E11.5 * Wnt1 up vs E11.5 * Ctrl
10446074	Uhrf1	NM_010931	0.00344687	1.29585	E11.5 * Wnt1 up vs E11.5 * Ctrl
10391431	Aarsd1	NM_144829	0.00351759	1.29578	E11.5 * Wnt1 up vs E11.5 * Ctrl
10507529	Med8	NM_020000	0.00165519	1.29568	E11.5 * Wnt1 up vs E11.5 * Ctrl
10510861	Megf6	ENSMUST00000	0.00576814	1.29557	E11.5 * Wnt1 up vs E11.5 * Ctrl
10384691	0610010F05Rik	NM_027860	0.000814761	1.29551	E11.5 * Wnt1 up vs E11.5 * Ctrl
10604057	06-Sep	NM_019942	0.00568504	1.29542	E11.5 * Wnt1 up vs E11.5 * Ctrl
10405001	Bicd2	NM_001039179	0.00250527	1.2954	E11.5 * Wnt1 up vs E11.5 * Ctrl
10583242	Sesn3	NM_030261	0.00200224	1.29526	E11.5 * Wnt1 up vs E11.5 * Ctrl
10517706	Mrto4	NM_023536	0.000196861	1.29521	E11.5 * Wnt1 up vs E11.5 * Ctrl
10509463	Eif4g3	NM_172703	2.55791e-005	1.29475	E11.5 * Wnt1 up vs E11.5 * Ctrl
10527233	Cyth3	NM_011182	0.000160147	1.2945	E11.5 * Wnt1 up vs E11.5 * Ctrl
10370471	Agpat3	NM_053014	0.000123633	1.2942	E11.5 * Wnt1 up vs E11.5 * Ctrl
10592593	Tecta	NM_009347	0.00721012	1.2941	E11.5 * Wnt1 up vs E11.5 * Ctrl

10573401	Trmt1	NM_198020	0.000578863	1.29364	E11.5 * Wnt1 up vs E11.5 * Ctrl
10364518	Ptbp1	NM_001077363	0.00403914	1.29345	E11.5 * Wnt1 up vs E11.5 * Ctrl
10465844	Asrgl1	NM_025610	0.000112965	1.29329	E11.5 * Wnt1 up vs E11.5 * Ctrl
10575763	Gan	NM_001081151	0.000444503	1.29315	E11.5 * Wnt1 up vs E11.5 * Ctrl
10592084	St3gal4	NM_009178	0.00300985	1.293	E11.5 * Wnt1 up vs E11.5 * Ctrl
10496789	Lpar3	NM_022983	0.000848141	1.2928	E11.5 * Wnt1 up vs E11.5 * Ctrl
10396919	4933426M11Rik	BC040401	0.000117185	1.29279	E11.5 * Wnt1 up vs E11.5 * Ctrl
10456723	BC031181	BC016084	0.00342094	1.2927	E11.5 * Wnt1 up vs E11.5 * Ctrl
10591967	Jam3	NM_023277	0.00119423	1.29256	E11.5 * Wnt1 up vs E11.5 * Ctrl
10553280	Gtf2h1	NM_008186	0.000695246	1.29227	E11.5 * Wnt1 up vs E11.5 * Ctrl
10561356	Psmc4	NM_011874	0.00055647	1.29219	E11.5 * Wnt1 up vs E11.5 * Ctrl
10526193		---	0.00186647	1.29201	E11.5 * Wnt1 up vs E11.5 * Ctrl
10357242	Dbi	NM_001037999	0.00280472	1.29183	E11.5 * Wnt1 up vs E11.5 * Ctrl
10445909	Kat2b	NM_020005	0.00101084	1.29171	E11.5 * Wnt1 up vs E11.5 * Ctrl
10395142	Sh3yl1	NM_013709	0.000864428	1.29094	E11.5 * Wnt1 up vs E11.5 * Ctrl
10393408	Tmc6	NM_145439	0.000493346	1.29082	E11.5 * Wnt1 up vs E11.5 * Ctrl
10548176	5930416I19Rik	NR_027360	0.00156045	1.29079	E11.5 * Wnt1 up vs E11.5 * Ctrl
10476582	Macrod2	NM_028387	3.79844e-005	1.29066	E11.5 * Wnt1 up vs E11.5 * Ctrl
10424624	Bai1	NM_174991	0.00130722	1.29033	E11.5 * Wnt1 up vs E11.5 * Ctrl
10370914	Fam108a	BC005632	0.0053119	1.2902	E11.5 * Wnt1 up vs E11.5 * Ctrl
10383395	Slc25a10	NM_013770	0.00159257	1.29009	E11.5 * Wnt1 up vs E11.5 * Ctrl
10391301	Stat3	NM_213659	0.0022437	1.29007	E11.5 * Wnt1 up vs E11.5 * Ctrl
10575961	Usp10	NM_009462	0.00278794	1.28985	E11.5 * Wnt1 up vs E11.5 * Ctrl
10375973	Taf13	NM_025444	0.00231602	1.28975	E11.5 * Wnt1 up vs E11.5 * Ctrl
10597309	Stac	NM_016853	0.00842328	1.28907	E11.5 * Wnt1 up vs E11.5 * Ctrl
10579554	Unc13a	NM_001029873	0.00314948	1.2889	E11.5 * Wnt1 up vs E11.5 * Ctrl
10442651	Ift140	NM_134126	0.000572013	1.28887	E11.5 * Wnt1 up vs E11.5 * Ctrl
10420320	Cenpj	NM_001014996	0.00319789	1.28883	E11.5 * Wnt1 up vs E11.5 * Ctrl
10475264	Ccndbp1	NM_010761	0.00551644	1.28866	E11.5 * Wnt1 up vs E11.5 * Ctrl
10504424	Reck	NM_016678	1.65413e-005	1.28864	E11.5 * Wnt1 up vs E11.5 * Ctrl
10539882	Ruvbl1	NM_019685	0.000167317	1.28854	E11.5 * Wnt1 up vs E11.5 * Ctrl
10474870	Rpusd2	NM_173450	0.000275314	1.28838	E11.5 * Wnt1 up vs E11.5 * Ctrl
10534021	Rimbp2	NM_001081388	1.40615e-005	1.28827	E11.5 * Wnt1 up vs E11.5 * Ctrl
10504884	5730528L13Rik	NM_001145924	0.00297706	1.28787	E11.5 * Wnt1 up vs E11.5 * Ctrl
10497379	ENSMUSG0000C	ENSMUST00000(	0.00237831	1.28778	E11.5 * Wnt1 up vs E11.5 * Ctrl
10524105	Chfr	NM_172717	0.000334949	1.28774	E11.5 * Wnt1 up vs E11.5 * Ctrl
10363392	Dnajb12	NM_019965	0.00285855	1.28755	E11.5 * Wnt1 up vs E11.5 * Ctrl
10456140	Sh3tc2	NM_172628	0.00780743	1.28748	E11.5 * Wnt1 up vs E11.5 * Ctrl
10546349	Xpc	NM_009531	4.94917e-006	1.28738	E11.5 * Wnt1 up vs E11.5 * Ctrl
10535739	Usp12	NM_011669	0.00508533	1.28704	E11.5 * Wnt1 up vs E11.5 * Ctrl
10402142	Ccdc88c	NM_026681	0.00377805	1.28676	E11.5 * Wnt1 up vs E11.5 * Ctrl
10582712	Egln1	NM_053207	2.85802e-005	1.28674	E11.5 * Wnt1 up vs E11.5 * Ctrl
10500054	Psmd4	NM_008951	0.0035607	1.28665	E11.5 * Wnt1 up vs E11.5 * Ctrl
10391221	Kat2a	NM_001038010	0.00879654	1.2866	E11.5 * Wnt1 up vs E11.5 * Ctrl
10460631	Rela	NM_009045	0.00297994	1.28627	E11.5 * Wnt1 up vs E11.5 * Ctrl
10409575	Neurog1	NM_010896	0.000436372	1.2859	E11.5 * Wnt1 up vs E11.5 * Ctrl
10373950	Nipsnap1	NM_008698	0.000490297	1.28579	E11.5 * Wnt1 up vs E11.5 * Ctrl
10417053	Mbnl2	NM_175341	0.000126301	1.28569	E11.5 * Wnt1 up vs E11.5 * Ctrl
10436446	Gps1	NM_145370	0.00382208	1.28551	E11.5 * Wnt1 up vs E11.5 * Ctrl
10390574	Fbxl20	NM_028149	0.00152686	1.28549	E11.5 * Wnt1 up vs E11.5 * Ctrl
10446594		---	0.00784569	1.28539	E11.5 * Wnt1 up vs E11.5 * Ctrl
10501649	Rtcd1	NM_025517	3.33721e-006	1.28462	E11.5 * Wnt1 up vs E11.5 * Ctrl
10575102	Cirh1a	NM_011574	0.00171774	1.2845	E11.5 * Wnt1 up vs E11.5 * Ctrl
10381419	Nbr1	NM_008676	0.00358284	1.28441	E11.5 * Wnt1 up vs E11.5 * Ctrl
10598422	Gripap1	NM_207670	0.00087107	1.28435	E11.5 * Wnt1 up vs E11.5 * Ctrl
10597531	Rbms3	NM_178660	4.42669e-005	1.28434	E11.5 * Wnt1 up vs E11.5 * Ctrl
10425226	Eif3l	NM_145139	1.65732e-005	1.2843	E11.5 * Wnt1 up vs E11.5 * Ctrl
10373873	Sf3a1	NM_026175	0.00385965	1.28424	E11.5 * Wnt1 up vs E11.5 * Ctrl
10434925	Hes1	NM_008235	0.000314828	1.28413	E11.5 * Wnt1 up vs E11.5 * Ctrl
10446334	Glcci1	NM_133236	0.00864427	1.28402	E11.5 * Wnt1 up vs E11.5 * Ctrl
10571048	Lsm1	NM_026032	0.00585535	1.28377	E11.5 * Wnt1 up vs E11.5 * Ctrl
10380660	Hoxb2	NM_134032	0.00337545	1.28364	E11.5 * Wnt1 up vs E11.5 * Ctrl
10575578	4930402E16Rik	NM_198308	0.00379679	1.28363	E11.5 * Wnt1 up vs E11.5 * Ctrl
10447431	Foxn2	NM_180974	2.6016e-005	1.28357	E11.5 * Wnt1 up vs E11.5 * Ctrl
10558707	Ric8	NM_053194	0.00420112	1.28328	E11.5 * Wnt1 up vs E11.5 * Ctrl
10406898	Taf9	NM_027592	0.00080861	1.2832	E11.5 * Wnt1 up vs E11.5 * Ctrl

10495539	Extl2	NM_021388	0.00714242	1.28307	E11.5 * Wnt1 up vs E11.5 * Ctrl
10364455	Cdc34	NM_177613	0.00785763	1.28306	E11.5 * Wnt1 up vs E11.5 * Ctrl
10420730	Fdft1	NM_010191	0.0011425	1.28297	E11.5 * Wnt1 up vs E11.5 * Ctrl
10579703	Cherp	NM_138585	0.0077524	1.28283	E11.5 * Wnt1 up vs E11.5 * Ctrl
10415844	Ctsb	NM_007798	0.00414205	1.28256	E11.5 * Wnt1 up vs E11.5 * Ctrl
10403076		---	0.00188486	1.28245	E11.5 * Wnt1 up vs E11.5 * Ctrl
10388492	Vps53	NM_026664	2.77138e-005	1.28233	E11.5 * Wnt1 up vs E11.5 * Ctrl
10420035	Ipo4	NM_024267	0.00196698	1.28232	E11.5 * Wnt1 up vs E11.5 * Ctrl
10420385	N6amt2	NM_026526	0.00348782	1.28223	E11.5 * Wnt1 up vs E11.5 * Ctrl
10437499	3930401K13Rik	NM_001079814	0.00265404	1.28216	E11.5 * Wnt1 up vs E11.5 * Ctrl
10539342	Rtkn	NM_133641	0.00198184	1.28207	E11.5 * Wnt1 up vs E11.5 * Ctrl
10579356	Pik3r2	NM_008841	0.00851935	1.28172	E11.5 * Wnt1 up vs E11.5 * Ctrl
10509858	Sdhb	NM_023374	0.00590317	1.2817	E11.5 * Wnt1 up vs E11.5 * Ctrl
10561351		---	0.00457091	1.28168	E11.5 * Wnt1 up vs E11.5 * Ctrl
10459730	Me2	NM_145494	0.00105154	1.28146	E11.5 * Wnt1 up vs E11.5 * Ctrl
10498802	Rapgef2	NM_001099624	0.000162159	1.28123	E11.5 * Wnt1 up vs E11.5 * Ctrl
10569265	Tollip	NM_023764	0.000832907	1.28123	E11.5 * Wnt1 up vs E11.5 * Ctrl
10388238	Tmem93	NM_025318	0.00849934	1.28114	E11.5 * Wnt1 up vs E11.5 * Ctrl
10370483	Rrp1	NM_010925	0.00329596	1.28107	E11.5 * Wnt1 up vs E11.5 * Ctrl
10433462	Pmm2	NM_016881	0.00683332	1.28102	E11.5 * Wnt1 up vs E11.5 * Ctrl
10411899	2410002O22Rik	NM_025879	0.00285335	1.28101	E11.5 * Wnt1 up vs E11.5 * Ctrl
10585860	Adpgk	NM_028121	0.00262025	1.2808	E11.5 * Wnt1 up vs E11.5 * Ctrl
10415980	Fbxo16	NM_015795	0.00837749	1.2808	E11.5 * Wnt1 up vs E11.5 * Ctrl
10516246	Cdca8	NM_026560	0.000181446	1.28075	E11.5 * Wnt1 up vs E11.5 * Ctrl
10478778	Arfgef2	NM_001085495	0.00471621	1.28033	E11.5 * Wnt1 up vs E11.5 * Ctrl
10396800	Plekhh1	ENSMUST00000	5.52094e-005	1.28025	E11.5 * Wnt1 up vs E11.5 * Ctrl
10456764	Smad2	NM_010754	5.82214e-005	1.27996	E11.5 * Wnt1 up vs E11.5 * Ctrl
10575512	Cog4	NM_133973	0.00170093	1.27994	E11.5 * Wnt1 up vs E11.5 * Ctrl
10582669	Ttc13	NM_145607	0.000257646	1.27993	E11.5 * Wnt1 up vs E11.5 * Ctrl
10483381	Stk39	NM_016866	8.00797e-005	1.27979	E11.5 * Wnt1 up vs E11.5 * Ctrl
10605659	OTTMUSG00000	ENSMUST00000	0.00771431	1.27963	E11.5 * Wnt1 up vs E11.5 * Ctrl
10576524	Gm505	NM_001111141	0.00236002	1.27922	E11.5 * Wnt1 up vs E11.5 * Ctrl
10461439	Fads1	NM_146094	0.00855255	1.27867	E11.5 * Wnt1 up vs E11.5 * Ctrl
10527430	Arpc1a	NM_019767	4.64859e-006	1.27853	E11.5 * Wnt1 up vs E11.5 * Ctrl
10461237	Bscl2	NM_001136064	0.000967283	1.2785	E11.5 * Wnt1 up vs E11.5 * Ctrl
10446739	Clip4	NM_030179	0.00868422	1.27825	E11.5 * Wnt1 up vs E11.5 * Ctrl
10583992	Igsf9b	NM_001129787	0.000469593	1.27807	E11.5 * Wnt1 up vs E11.5 * Ctrl
10438478	Abcc5	NM_013790	0.000120379	1.27767	E11.5 * Wnt1 up vs E11.5 * Ctrl
10471036	Dolpp1	NM_020329	0.00455461	1.27752	E11.5 * Wnt1 up vs E11.5 * Ctrl
10496397	Mapksp1	NM_019920	0.000547772	1.27716	E11.5 * Wnt1 up vs E11.5 * Ctrl
10547140	Tmcc1	ENSMUST00000	9.28046e-005	1.2771	E11.5 * Wnt1 up vs E11.5 * Ctrl
10576373	Gas8	NM_018855	5.02521e-005	1.277	E11.5 * Wnt1 up vs E11.5 * Ctrl
10589494	Cspg5	NM_013884	0.007786	1.27696	E11.5 * Wnt1 up vs E11.5 * Ctrl
10367634	Akap12	NM_031185	0.000159519	1.27683	E11.5 * Wnt1 up vs E11.5 * Ctrl
10500630	Ttf2	NM_001013026	0.00569717	1.27635	E11.5 * Wnt1 up vs E11.5 * Ctrl
10581560	Psmd7	NM_010817	0.000253158	1.27632	E11.5 * Wnt1 up vs E11.5 * Ctrl
10507851	Pabpc4	NM_130881	0.00169935	1.27623	E11.5 * Wnt1 up vs E11.5 * Ctrl
10470119	Edf1	NM_021519	0.000146546	1.27605	E11.5 * Wnt1 up vs E11.5 * Ctrl
10478447	Stk4	NM_021420	1.91256e-005	1.27599	E11.5 * Wnt1 up vs E11.5 * Ctrl
10558090	Tacc2	NM_001004468	0.000390215	1.27594	E11.5 * Wnt1 up vs E11.5 * Ctrl
10533644	Vps33a	NM_029929	0.000156549	1.27594	E11.5 * Wnt1 up vs E11.5 * Ctrl
10499777	Ints3	NM_145540	0.00280891	1.27591	E11.5 * Wnt1 up vs E11.5 * Ctrl
10547177	Rassf4	NM_178045	0.00689433	1.27591	E11.5 * Wnt1 up vs E11.5 * Ctrl
10480921	Qsox2	NM_153559	0.0010462	1.27582	E11.5 * Wnt1 up vs E11.5 * Ctrl
10457924	Bloc1s1	NM_015740	0.0041247	1.27567	E11.5 * Wnt1 up vs E11.5 * Ctrl
10423599	Matn2	NM_016762	0.00847201	1.27551	E11.5 * Wnt1 up vs E11.5 * Ctrl
10526381	Mdh2	NM_008617	0.000710807	1.27542	E11.5 * Wnt1 up vs E11.5 * Ctrl
10397428	1700020O03Rik	BC048169	0.00629525	1.27506	E11.5 * Wnt1 up vs E11.5 * Ctrl
10393177	Acox1	NM_015729	0.00567	1.27477	E11.5 * Wnt1 up vs E11.5 * Ctrl
10428157	Rnf19a	NM_013923	0.00116685	1.27462	E11.5 * Wnt1 up vs E11.5 * Ctrl
10395005	Kidins220	NM_001081378	5.05761e-005	1.27456	E11.5 * Wnt1 up vs E11.5 * Ctrl
10465336	Mrpl49	NM_026246	0.00214626	1.27439	E11.5 * Wnt1 up vs E11.5 * Ctrl
10361200	AA408296	NM_145415	4.16323e-005	1.27424	E11.5 * Wnt1 up vs E11.5 * Ctrl
10580274	Rnaseh2a	NM_027187	0.00649843	1.27418	E11.5 * Wnt1 up vs E11.5 * Ctrl
10479411	Ogfr	NM_031373	0.000159096	1.27418	E11.5 * Wnt1 up vs E11.5 * Ctrl
10430871	Tdg	NM_011561	0.000124923	1.27418	E11.5 * Wnt1 up vs E11.5 * Ctrl

10418991	Gcap14	NM_027045	0.000354472	1.27399	E11.5 * Wnt1 up vs E11.5 * Ctrl
10427277	Hoxc11	NM_001024842	0.000375663	1.27354	E11.5 * Wnt1 up vs E11.5 * Ctrl
10440491	App	NM_007471	0.00511864	1.27348	E11.5 * Wnt1 up vs E11.5 * Ctrl
10562360	Gpi1	NM_008155	0.00408145	1.27348	E11.5 * Wnt1 up vs E11.5 * Ctrl
10421917	Pabpc4	NM_148917	0.00204751	1.27345	E11.5 * Wnt1 up vs E11.5 * Ctrl
10486469	Vps39	NM_147153	0.00174431	1.27336	E11.5 * Wnt1 up vs E11.5 * Ctrl
10360794	Fbxo28	NM_175127	1.29262e-006	1.27322	E11.5 * Wnt1 up vs E11.5 * Ctrl
10485429	Pdhx	NM_175094	0.00197171	1.27286	E11.5 * Wnt1 up vs E11.5 * Ctrl
10502881	St6galnac5	NM_012028	0.00361459	1.27271	E11.5 * Wnt1 up vs E11.5 * Ctrl
10396306	1200003C05Rik	NM_024205	0.001826	1.27245	E11.5 * Wnt1 up vs E11.5 * Ctrl
10536425		---	0.00828817	1.27233	E11.5 * Wnt1 up vs E11.5 * Ctrl
10589004	Qars	NM_133794	0.000855629	1.27231	E11.5 * Wnt1 up vs E11.5 * Ctrl
10376033	Kif3a	NM_008443	3.38789e-005	1.27227	E11.5 * Wnt1 up vs E11.5 * Ctrl
10442458	Gbl	NM_019988	0.000389211	1.27222	E11.5 * Wnt1 up vs E11.5 * Ctrl
10529375	Mxd4	NM_010753	0.00241818	1.27185	E11.5 * Wnt1 up vs E11.5 * Ctrl
10409222	Shc3	NM_009167	0.00496789	1.27157	E11.5 * Wnt1 up vs E11.5 * Ctrl
10596680	Sema3b	NM_001042779	0.00813599	1.27127	E11.5 * Wnt1 up vs E11.5 * Ctrl
10598882	Uba1	NM_009457	2.89128e-006	1.27099	E11.5 * Wnt1 up vs E11.5 * Ctrl
10583697	Smarca4	NM_011417	0.000237293	1.27026	E11.5 * Wnt1 up vs E11.5 * Ctrl
10437712	Zc3h7a	NM_145931	0.00427786	1.27014	E11.5 * Wnt1 up vs E11.5 * Ctrl
10556178	Tub	NM_021885	0.00478953	1.2699	E11.5 * Wnt1 up vs E11.5 * Ctrl
10458398	Hars	NM_008214	0.000342203	1.26946	E11.5 * Wnt1 up vs E11.5 * Ctrl
10421661	Gtf2f2	NM_026816	0.000188158	1.26917	E11.5 * Wnt1 up vs E11.5 * Ctrl
10415766	Sacs	NM_172809	0.000859869	1.26903	E11.5 * Wnt1 up vs E11.5 * Ctrl
10517263	Fam54b	BC026808	0.000838126	1.26895	E11.5 * Wnt1 up vs E11.5 * Ctrl
10406111	Slc12a7	NM_011390	0.00878111	1.26872	E11.5 * Wnt1 up vs E11.5 * Ctrl
10460490	Dpp3	NM_133803	0.00310476	1.26863	E11.5 * Wnt1 up vs E11.5 * Ctrl
10525804	Atp6v0a2	NM_011596	0.00809574	1.26839	E11.5 * Wnt1 up vs E11.5 * Ctrl
10344637	Atp6v1h	NM_133826	0.000193819	1.26834	E11.5 * Wnt1 up vs E11.5 * Ctrl
10574632	Cbfb	NM_022309	7.77506e-005	1.26823	E11.5 * Wnt1 up vs E11.5 * Ctrl
10601915	BC031748	BC031748	0.000779578	1.2681	E11.5 * Wnt1 up vs E11.5 * Ctrl
10368883	Tdg	NM_011561	0.000245434	1.26794	E11.5 * Wnt1 up vs E11.5 * Ctrl
10556576	1110004F10Rik	NM_019772	0.00121685	1.26783	E11.5 * Wnt1 up vs E11.5 * Ctrl
10575662	Mon1b	NM_173015	0.000990474	1.26764	E11.5 * Wnt1 up vs E11.5 * Ctrl
10570236	Mcf2l	NM_178076	0.000808896	1.26753	E11.5 * Wnt1 up vs E11.5 * Ctrl
10395129	Tmem18	NM_172049	0.000451524	1.26733	E11.5 * Wnt1 up vs E11.5 * Ctrl
10388532	Nxn	NM_008750	0.00631907	1.26727	E11.5 * Wnt1 up vs E11.5 * Ctrl
10472994	Mtx2	NM_016804	5.77943e-005	1.26716	E11.5 * Wnt1 up vs E11.5 * Ctrl
10425559	L3mbtl2	NM_145993	0.000414188	1.26711	E11.5 * Wnt1 up vs E11.5 * Ctrl
10599498	Utp14a	NM_028276	5.59469e-005	1.26692	E11.5 * Wnt1 up vs E11.5 * Ctrl
10347774	Mrpl44	NM_001081210	0.00230029	1.26683	E11.5 * Wnt1 up vs E11.5 * Ctrl
10423333	Fam134b	NM_001034851	0.0040003	1.2667	E11.5 * Wnt1 up vs E11.5 * Ctrl
10500808	Olfml3	NM_133859	0.00154693	1.26631	E11.5 * Wnt1 up vs E11.5 * Ctrl
10394749	Nol10	NM_001008421	0.00337157	1.26619	E11.5 * Wnt1 up vs E11.5 * Ctrl
10477353	Mapre1	NM_007896	2.75084e-005	1.26596	E11.5 * Wnt1 up vs E11.5 * Ctrl
10438603	Igf2bp2	NM_183029	0.00363881	1.26594	E11.5 * Wnt1 up vs E11.5 * Ctrl
10437311	Trap1	NM_026508	0.000305735	1.2656	E11.5 * Wnt1 up vs E11.5 * Ctrl
10455227	Rnf14	NM_020012	2.15148e-005	1.26559	E11.5 * Wnt1 up vs E11.5 * Ctrl
10601473	Apool	NM_026565	0.00799289	1.26538	E11.5 * Wnt1 up vs E11.5 * Ctrl
10553301	Ldha	NM_010699	0.0037853	1.26476	E11.5 * Wnt1 up vs E11.5 * Ctrl
10376314	Cnot8	NM_026949	0.00397961	1.26414	E11.5 * Wnt1 up vs E11.5 * Ctrl
10386551	Flii	NM_022009	0.000838055	1.26414	E11.5 * Wnt1 up vs E11.5 * Ctrl
10483648	Ola1	NM_025942	0.00566835	1.26398	E11.5 * Wnt1 up vs E11.5 * Ctrl
10561673	Spred3	NM_182927	0.0020395	1.26397	E11.5 * Wnt1 up vs E11.5 * Ctrl
10393320	Ube2o	NM_173755	0.000922875	1.26373	E11.5 * Wnt1 up vs E11.5 * Ctrl
10585932	Pkm2	NM_011099	0.00719002	1.26354	E11.5 * Wnt1 up vs E11.5 * Ctrl
10463704	As3mt	NM_020577	0.000586034	1.2633	E11.5 * Wnt1 up vs E11.5 * Ctrl
10405074	Nol8	NM_001081350	0.00250453	1.26318	E11.5 * Wnt1 up vs E11.5 * Ctrl
10374485	Peli1	NM_023324	0.00460713	1.26317	E11.5 * Wnt1 up vs E11.5 * Ctrl
10345230	Rab23	NM_008999	0.00138867	1.26256	E11.5 * Wnt1 up vs E11.5 * Ctrl
10483584	Mettl8	NM_145524	0.00229635	1.2623	E11.5 * Wnt1 up vs E11.5 * Ctrl
10457546	Osbpl1a	NM_207530	0.000837796	1.26213	E11.5 * Wnt1 up vs E11.5 * Ctrl
10384885	Spnb2	NM_175836	7.67866e-005	1.26207	E11.5 * Wnt1 up vs E11.5 * Ctrl
10558134	Plekha1	NM_133942	0.00168124	1.26206	E11.5 * Wnt1 up vs E11.5 * Ctrl
10465263	Dpf2	NM_011262	0.000226781	1.26205	E11.5 * Wnt1 up vs E11.5 * Ctrl
10573434	Farsa	NM_025648	0.0067816	1.26187	E11.5 * Wnt1 up vs E11.5 * Ctrl

10468489	Xpnpep1	NM_133216	0.000327881	1.26175	E11.5 * Wnt1 up vs E11.5 * Ctrl
10370025	Smarcb1	NM_011418	0.000603248	1.26163	E11.5 * Wnt1 up vs E11.5 * Ctrl
10403041		---	0.000672495	1.26115	E11.5 * Wnt1 up vs E11.5 * Ctrl
10383485	Gps1	NM_145370	0.00277086	1.26098	E11.5 * Wnt1 up vs E11.5 * Ctrl
10425822	Pnpla3	NM_054088	0.00501531	1.26097	E11.5 * Wnt1 up vs E11.5 * Ctrl
10485828	Slc12a6	NM_133648	0.00295013	1.26091	E11.5 * Wnt1 up vs E11.5 * Ctrl
10385616	Rufy1	NM_172557	0.00248954	1.26089	E11.5 * Wnt1 up vs E11.5 * Ctrl
10432215	Ddx23	NM_001080981	0.00493159	1.26085	E11.5 * Wnt1 up vs E11.5 * Ctrl
10424400	Myc	NM_010849	0.00733068	1.26073	E11.5 * Wnt1 up vs E11.5 * Ctrl
10564266	Peg12	NM_013788	0.00205423	1.26024	E11.5 * Wnt1 up vs E11.5 * Ctrl
10521163	Gm1673	BC147444	0.00417006	1.26006	E11.5 * Wnt1 up vs E11.5 * Ctrl
10546567	A130022J15Rik	NM_175313	0.00460954	1.25994	E11.5 * Wnt1 up vs E11.5 * Ctrl
10419759	Prmt5	NM_013768	0.00547715	1.25981	E11.5 * Wnt1 up vs E11.5 * Ctrl
10502131	Lrit3	XM_143529	0.00862429	1.25961	E11.5 * Wnt1 up vs E11.5 * Ctrl
10399379	Pgk1	NM_008828	6.95896e-007	1.2596	E11.5 * Wnt1 up vs E11.5 * Ctrl
10498313	Pgk1	NM_008828	6.95896e-007	1.2596	E11.5 * Wnt1 up vs E11.5 * Ctrl
10528546	Gabarapl2	NM_026693	0.00303687	1.25952	E11.5 * Wnt1 up vs E11.5 * Ctrl
10365230	Tdg	NM_172552	0.000276225	1.25946	E11.5 * Wnt1 up vs E11.5 * Ctrl
10383671	Drg1	NM_007879	5.7267e-005	1.25898	E11.5 * Wnt1 up vs E11.5 * Ctrl
10517328	Tmem50a	NM_027935	4.45188e-005	1.25885	E11.5 * Wnt1 up vs E11.5 * Ctrl
10370259	Col18a1	NM_009929	0.00129171	1.25855	E11.5 * Wnt1 up vs E11.5 * Ctrl
10391755	Ccdc43	NM_025918	0.000641185	1.25853	E11.5 * Wnt1 up vs E11.5 * Ctrl
10573217	Ddx39	NM_197982	0.000318941	1.2585	E11.5 * Wnt1 up vs E11.5 * Ctrl
10450648	Abcf1	NM_013854	0.00491038	1.25808	E11.5 * Wnt1 up vs E11.5 * Ctrl
10575476	Vac14	NM_146216	0.0029963	1.25708	E11.5 * Wnt1 up vs E11.5 * Ctrl
10433721	Nde1	NM_023317	0.000710001	1.25703	E11.5 * Wnt1 up vs E11.5 * Ctrl
10568050	Aldoa	NM_007438	0.00496778	1.25694	E11.5 * Wnt1 up vs E11.5 * Ctrl
10580510	Sall1	NM_021390	0.00465346	1.25679	E11.5 * Wnt1 up vs E11.5 * Ctrl
10452110	2410015M20Rik	NM_153152	0.0080124	1.25677	E11.5 * Wnt1 up vs E11.5 * Ctrl
10591139	Naalad2	NM_028279	0.00174301	1.25661	E11.5 * Wnt1 up vs E11.5 * Ctrl
10557628	Zfp689	NM_175163	0.00389221	1.25623	E11.5 * Wnt1 up vs E11.5 * Ctrl
10349401	Gpr39	NM_027677	0.000593597	1.25601	E11.5 * Wnt1 up vs E11.5 * Ctrl
10541049	March8	NM_027920	0.00506596	1.25594	E11.5 * Wnt1 up vs E11.5 * Ctrl
10463751	Pdcd11	NM_011053	0.00116709	1.25567	E11.5 * Wnt1 up vs E11.5 * Ctrl
10607332		---	0.00665622	1.25563	E11.5 * Wnt1 up vs E11.5 * Ctrl
10559270	Tssc4	NM_020285	0.000503589	1.25557	E11.5 * Wnt1 up vs E11.5 * Ctrl
10561401	Supt5h	NM_013676	0.00364119	1.25538	E11.5 * Wnt1 up vs E11.5 * Ctrl
10519183	Atad3a	NM_179203	0.00390058	1.25536	E11.5 * Wnt1 up vs E11.5 * Ctrl
10476969	Pygb	NM_153781	0.00260924	1.25516	E11.5 * Wnt1 up vs E11.5 * Ctrl
10358259	Nek7	NM_021605	0.00375548	1.25515	E11.5 * Wnt1 up vs E11.5 * Ctrl
10397719	Tdp1	NM_028354	0.000905564	1.25513	E11.5 * Wnt1 up vs E11.5 * Ctrl
10463254	D19Ertd386e	NM_177464	0.00182508	1.25509	E11.5 * Wnt1 up vs E11.5 * Ctrl
10448064	Prdm9	NM_144809	0.0032601	1.25509	E11.5 * Wnt1 up vs E11.5 * Ctrl
10470751	Slc27a4	NM_011989	0.000751172	1.25486	E11.5 * Wnt1 up vs E11.5 * Ctrl
10561306	Pld3	NM_011116	0.00383766	1.25475	E11.5 * Wnt1 up vs E11.5 * Ctrl
10471535	Fam129b	NM_146119	0.00619949	1.25463	E11.5 * Wnt1 up vs E11.5 * Ctrl
10545812	Sfxn5	NM_178639	0.00699894	1.25456	E11.5 * Wnt1 up vs E11.5 * Ctrl
10464704	Adrbk1	NM_130863	0.00446338	1.25452	E11.5 * Wnt1 up vs E11.5 * Ctrl
10538087	Lrrc61	NM_177736	0.00479418	1.25421	E11.5 * Wnt1 up vs E11.5 * Ctrl
10392347	Pitpnc1	NM_145823	0.00023934	1.25412	E11.5 * Wnt1 up vs E11.5 * Ctrl
10378334	Tax1bp3	NM_029564	0.000333871	1.25407	E11.5 * Wnt1 up vs E11.5 * Ctrl
10477297	Kif3b	NM_008444	0.00811119	1.25394	E11.5 * Wnt1 up vs E11.5 * Ctrl
10469020	Bend7	NM_178663	0.00053314	1.25384	E11.5 * Wnt1 up vs E11.5 * Ctrl
10465604	Stip1	NM_016737	0.000150737	1.25383	E11.5 * Wnt1 up vs E11.5 * Ctrl
10410687		---	0.00595402	1.25381	E11.5 * Wnt1 up vs E11.5 * Ctrl
10557631	Prr14	NM_145589	2.07668e-005	1.25378	E11.5 * Wnt1 up vs E11.5 * Ctrl
10565288	Fam108c	NM_133722	0.000344282	1.25373	E11.5 * Wnt1 up vs E11.5 * Ctrl
10524124	Golga3	NM_008146	7.58996e-005	1.25325	E11.5 * Wnt1 up vs E11.5 * Ctrl
10385335		---	0.00582427	1.2527	E11.5 * Wnt1 up vs E11.5 * Ctrl
10477187	Tpx2	NM_001141977	0.00247875	1.25257	E11.5 * Wnt1 up vs E11.5 * Ctrl
10593842	Tspan3	NM_019793	8.8858e-006	1.25245	E11.5 * Wnt1 up vs E11.5 * Ctrl
10561907	0610010E21Rik	NM_001033140	0.000596058	1.25221	E11.5 * Wnt1 up vs E11.5 * Ctrl
10467470	Aldh18a1	NM_019698	0.00135572	1.252	E11.5 * Wnt1 up vs E11.5 * Ctrl
10601850	Bex4	NM_212457	0.00312886	1.25151	E11.5 * Wnt1 up vs E11.5 * Ctrl
10347873	Agfg1	NM_010472	0.000338383	1.25136	E11.5 * Wnt1 up vs E11.5 * Ctrl
10357164	Epb4.1l5	NM_145506	7.57738e-005	1.25117	E11.5 * Wnt1 up vs E11.5 * Ctrl

10431872	Slc38a1	ENSMUST00000	0.00159313	1.25115	E11.5 * Wnt1 up vs E11.5 * Ctrl
10574718	Tmem208	NM_025486	0.00728613	1.25111	E11.5 * Wnt1 up vs E11.5 * Ctrl
10564313	Mphosph10	NM_026483	0.00536787	1.25099	E11.5 * Wnt1 up vs E11.5 * Ctrl
10409365	Gprin1	NM_012014	0.00253164	1.25095	E11.5 * Wnt1 up vs E11.5 * Ctrl
10373454	Pa2g4	NM_011119	0.000410173	1.25079	E11.5 * Wnt1 up vs E11.5 * Ctrl
10537463	Agk	NM_023538	1.46058e-005	1.25069	E11.5 * Wnt1 up vs E11.5 * Ctrl
10578300	Mtmr7	NM_001040699	0.00693946	1.25058	E11.5 * Wnt1 up vs E11.5 * Ctrl
10480813	B230208H17Rik	BC085507	0.00428127	1.25052	E11.5 * Wnt1 up vs E11.5 * Ctrl
10498296	Commd2	NM_175095	5.22313e-005	1.25039	E11.5 * Wnt1 up vs E11.5 * Ctrl
10426550	Tmem106c	NM_201359	0.00357045	1.25028	E11.5 * Wnt1 up vs E11.5 * Ctrl
10379075	2610507B11Rik	NM_001002004	4.14396e-005	1.25009	E11.5 * Wnt1 up vs E11.5 * Ctrl
10355813	Ptprn	NM_008985	0.00545451	1.24982	E11.5 * Wnt1 up vs E11.5 * Ctrl
10488912	Edem2	NM_145537	0.00746467	1.24982	E11.5 * Wnt1 up vs E11.5 * Ctrl
10572146	Atp6v1b2	NM_007509	0.00850334	1.24971	E11.5 * Wnt1 up vs E11.5 * Ctrl
10409616	Spock1	NM_009262	0.00391012	1.2497	E11.5 * Wnt1 up vs E11.5 * Ctrl
10539606	Cct7	NM_007638	0.000333448	1.2497	E11.5 * Wnt1 up vs E11.5 * Ctrl
10521168	Nat8l	NM_001001985	0.000765996	1.24966	E11.5 * Wnt1 up vs E11.5 * Ctrl
10508376	Trim62	NM_178110	0.000175304	1.24957	E11.5 * Wnt1 up vs E11.5 * Ctrl
10447708	Qk	NM_021881	3.23041e-005	1.24939	E11.5 * Wnt1 up vs E11.5 * Ctrl
10458213	Etf1	NM_144866	8.67666e-005	1.24927	E11.5 * Wnt1 up vs E11.5 * Ctrl
10518385	Mfn2	NM_133201	0.00671055	1.24926	E11.5 * Wnt1 up vs E11.5 * Ctrl
10564663	Tdg	NM_172552	0.00170896	1.24872	E11.5 * Wnt1 up vs E11.5 * Ctrl
10469070	Nudt5	NM_016918	0.00595268	1.24857	E11.5 * Wnt1 up vs E11.5 * Ctrl
10563615	Hps5	NM_001005247	0.0023128	1.24816	E11.5 * Wnt1 up vs E11.5 * Ctrl
10459837	8030462N17Rik	NM_178670	0.00735452	1.24814	E11.5 * Wnt1 up vs E11.5 * Ctrl
10573519	Tnpo2	NM_145390	0.00444107	1.24812	E11.5 * Wnt1 up vs E11.5 * Ctrl
10560304	Calm3	NM_007590	0.00421664	1.24804	E11.5 * Wnt1 up vs E11.5 * Ctrl
10460732	Znhit2	NM_013859	0.00251979	1.24783	E11.5 * Wnt1 up vs E11.5 * Ctrl
10415377	2610027L16Rik	NM_026403	0.00191879	1.24733	E11.5 * Wnt1 up vs E11.5 * Ctrl
10357698	Tmcc2	NM_178874	0.00636717	1.24712	E11.5 * Wnt1 up vs E11.5 * Ctrl
10357133		---	6.3668e-005	1.24694	E11.5 * Wnt1 up vs E11.5 * Ctrl
10587211	Leo1	NM_001039522	8.93995e-005	1.24665	E11.5 * Wnt1 up vs E11.5 * Ctrl
10348866	Atg4b	NM_174874	0.00175254	1.24663	E11.5 * Wnt1 up vs E11.5 * Ctrl
10364675	Gpx4	NM_008162	0.00247665	1.24648	E11.5 * Wnt1 up vs E11.5 * Ctrl
10422518	Tmtc4	NM_028651	0.00152555	1.24646	E11.5 * Wnt1 up vs E11.5 * Ctrl
10367337	Rnf41	NM_026259	0.000327419	1.24634	E11.5 * Wnt1 up vs E11.5 * Ctrl
10575619	Terf2ip	NM_020584	0.00539326	1.24632	E11.5 * Wnt1 up vs E11.5 * Ctrl
10356628	Hdac4	NM_207225	0.00631601	1.24627	E11.5 * Wnt1 up vs E11.5 * Ctrl
10549552	Prpf31	NM_027328	0.00632172	1.24621	E11.5 * Wnt1 up vs E11.5 * Ctrl
10381187	Atp6v0a1	NM_016920	0.00126846	1.24619	E11.5 * Wnt1 up vs E11.5 * Ctrl
10382980	Syngr2	NM_009304	0.00764273	1.24611	E11.5 * Wnt1 up vs E11.5 * Ctrl
10435266	Heg1	NM_175256	0.0082397	1.24607	E11.5 * Wnt1 up vs E11.5 * Ctrl
10458607	Lars	NM_134137	0.000342727	1.246	E11.5 * Wnt1 up vs E11.5 * Ctrl
10523772	Lrrc8d	NM_178701	0.00530134	1.24586	E11.5 * Wnt1 up vs E11.5 * Ctrl
10517421	Pnrc2	NM_026383	0.000255617	1.24582	E11.5 * Wnt1 up vs E11.5 * Ctrl
10446235	Trip10	NM_134125	0.00412307	1.24554	E11.5 * Wnt1 up vs E11.5 * Ctrl
10385175	Wwc1	NM_170779	0.00374894	1.24538	E11.5 * Wnt1 up vs E11.5 * Ctrl
10468275	Pcgf6	NM_027654	0.000461557	1.24532	E11.5 * Wnt1 up vs E11.5 * Ctrl
10585610	Ptpn9	NM_019651	0.000266371	1.2453	E11.5 * Wnt1 up vs E11.5 * Ctrl
10375941	Vdac1	NM_011694	0.00408945	1.24516	E11.5 * Wnt1 up vs E11.5 * Ctrl
10360764	Enah	NM_010135	0.000194393	1.24481	E11.5 * Wnt1 up vs E11.5 * Ctrl
10510176	OTTMUSG00000	NM_001039233	0.00596885	1.24472	E11.5 * Wnt1 up vs E11.5 * Ctrl
10479560	Rtel1	NM_001001882	0.00115681	1.24458	E11.5 * Wnt1 up vs E11.5 * Ctrl
10591357	Eif3g	NM_016876	0.00392311	1.24444	E11.5 * Wnt1 up vs E11.5 * Ctrl
10475965	Polr1b	NM_009086	0.001143	1.24442	E11.5 * Wnt1 up vs E11.5 * Ctrl
10465804	Polr2g	NM_026329	0.00111459	1.24414	E11.5 * Wnt1 up vs E11.5 * Ctrl
10420532	Atp8a2	NM_015803	0.00179494	1.24376	E11.5 * Wnt1 up vs E11.5 * Ctrl
10541446	Cpamd8	NM_008646	0.00436445	1.24365	E11.5 * Wnt1 up vs E11.5 * Ctrl
10532892	1500011B03Rik	ENSMUST00000	0.000168162	1.24341	E11.5 * Wnt1 up vs E11.5 * Ctrl
10491977	2810046L04Rik	NM_173382	0.00858576	1.24339	E11.5 * Wnt1 up vs E11.5 * Ctrl
10496621	Gtf2b	NM_145546	0.0039599	1.24325	E11.5 * Wnt1 up vs E11.5 * Ctrl
10576696	Insr	NM_010568	0.000194375	1.24319	E11.5 * Wnt1 up vs E11.5 * Ctrl
10591192	Olfr847	NM_146525	0.0048441	1.24303	E11.5 * Wnt1 up vs E11.5 * Ctrl
10502165	Sec24b	NM_207209	1.5671e-006	1.24302	E11.5 * Wnt1 up vs E11.5 * Ctrl
10369761	Reep3	NM_178606	0.000616059	1.24296	E11.5 * Wnt1 up vs E11.5 * Ctrl
10413502	Actr8	NM_027493	0.00383765	1.24275	E11.5 * Wnt1 up vs E11.5 * Ctrl

10397741	Psmc1	NM_008947	0.000636707	1.2426	E11.5 * Wnt1 up vs E11.5 * Ctrl
10589420	Cdc25a	NM_007658	0.000983453	1.24254	E11.5 * Wnt1 up vs E11.5 * Ctrl
10519488	Tubb2c	NM_146116	0.00730997	1.24242	E11.5 * Wnt1 up vs E11.5 * Ctrl
10577560	Ikbkb	NM_010546	0.000362519	1.24235	E11.5 * Wnt1 up vs E11.5 * Ctrl
10562399	Kctd15	NM_146188	0.00408389	1.2422	E11.5 * Wnt1 up vs E11.5 * Ctrl
10360076	Ndufs2	NM_153064	0.00160096	1.2421	E11.5 * Wnt1 up vs E11.5 * Ctrl
10573566	Dhps	NM_001039514	0.000790457	1.24199	E11.5 * Wnt1 up vs E11.5 * Ctrl
10402835	Nudt14	NM_025399	0.00366677	1.24192	E11.5 * Wnt1 up vs E11.5 * Ctrl
10349249	Clasp1	NM_001081276	0.00187422	1.2418	E11.5 * Wnt1 up vs E11.5 * Ctrl
10443021	Atp6v0e	NM_025272	0.000984905	1.24179	E11.5 * Wnt1 up vs E11.5 * Ctrl
10473097	Plekha3	NM_031256	0.00496063	1.24157	E11.5 * Wnt1 up vs E11.5 * Ctrl
10485183	Slc35c1	NM_211358	0.00145438	1.24151	E11.5 * Wnt1 up vs E11.5 * Ctrl
10463168	Pgam1	NM_023418	0.0020829	1.2414	E11.5 * Wnt1 up vs E11.5 * Ctrl
10561302	Pgam1	NM_023418	0.0020829	1.2414	E11.5 * Wnt1 up vs E11.5 * Ctrl
10594988	Mapk6	NM_015806	2.85214e-005	1.24135	E11.5 * Wnt1 up vs E11.5 * Ctrl
10419223	Fermt2	NM_146054	0.000315705	1.24126	E11.5 * Wnt1 up vs E11.5 * Ctrl
10440406	Nrip1	NM_173440	0.000459871	1.24114	E11.5 * Wnt1 up vs E11.5 * Ctrl
10397818	Cpsf2	NM_016856	6.25352e-006	1.24101	E11.5 * Wnt1 up vs E11.5 * Ctrl
10363845	Ccdc6	NM_001111121	0.00217754	1.24098	E11.5 * Wnt1 up vs E11.5 * Ctrl
10567303	Coq7	NM_009940	0.00218273	1.24075	E11.5 * Wnt1 up vs E11.5 * Ctrl
10504349	Creb3	NM_013497	0.00672677	1.24037	E11.5 * Wnt1 up vs E11.5 * Ctrl
10395466	Dock4	NM_172803	0.00211027	1.24033	E11.5 * Wnt1 up vs E11.5 * Ctrl
10555414	Rab6	NM_024287	0.00272082	1.24016	E11.5 * Wnt1 up vs E11.5 * Ctrl
10452213	Gtf2f1	NM_133801	0.00172681	1.24015	E11.5 * Wnt1 up vs E11.5 * Ctrl
10524004	Pcgf3	NM_172716	0.00017964	1.24013	E11.5 * Wnt1 up vs E11.5 * Ctrl
10346668	Fam117b	NM_001037725	0.00211983	1.24009	E11.5 * Wnt1 up vs E11.5 * Ctrl
10449644	Glo1	NM_025374	0.00358531	1.24005	E11.5 * Wnt1 up vs E11.5 * Ctrl
10407782	Edaradd	NM_133643	0.00606134	1.24003	E11.5 * Wnt1 up vs E11.5 * Ctrl
10405994	Med10	NM_138596	0.00191054	1.23991	E11.5 * Wnt1 up vs E11.5 * Ctrl
10469951	Rnf208	NM_176834	0.00447596	1.2399	E11.5 * Wnt1 up vs E11.5 * Ctrl
10567941	Eif3c	NM_146200	3.35106e-006	1.23968	E11.5 * Wnt1 up vs E11.5 * Ctrl
10589784	Dclk3	NM_172928	0.00684367	1.23968	E11.5 * Wnt1 up vs E11.5 * Ctrl
10566405	Mapksp1	NM_019920	0.00244714	1.23933	E11.5 * Wnt1 up vs E11.5 * Ctrl
10395538	Pnpla8	NM_026164	0.00110781	1.23906	E11.5 * Wnt1 up vs E11.5 * Ctrl
10533849	Rilpl1	NM_021430	0.001989	1.23901	E11.5 * Wnt1 up vs E11.5 * Ctrl
10473045	Rbm45	NM_153405	0.00136325	1.23892	E11.5 * Wnt1 up vs E11.5 * Ctrl
10565996	Inppl1	NM_010567	0.00795495	1.23865	E11.5 * Wnt1 up vs E11.5 * Ctrl
10409557	H2afy	NM_012015	2.90291e-005	1.23853	E11.5 * Wnt1 up vs E11.5 * Ctrl
10590325	Ctnnb1	NM_007614	0.00305276	1.23837	E11.5 * Wnt1 up vs E11.5 * Ctrl
10520800	Gpn1	NM_133756	0.00027112	1.23825	E11.5 * Wnt1 up vs E11.5 * Ctrl
10364338	1810043G02Rik	NM_026431	0.00157217	1.23783	E11.5 * Wnt1 up vs E11.5 * Ctrl
10372116	Csl	NM_027945	0.000499501	1.23781	E11.5 * Wnt1 up vs E11.5 * Ctrl
10452496	Ralbp1	NM_009067	0.000676977	1.23741	E11.5 * Wnt1 up vs E11.5 * Ctrl
10460833	Ehd1	NM_010119	0.000186169	1.2374	E11.5 * Wnt1 up vs E11.5 * Ctrl
10498568	Shox2	NM_013665	0.00132826	1.23733	E11.5 * Wnt1 up vs E11.5 * Ctrl
10416082	Trim35	NM_029979	0.000752727	1.23707	E11.5 * Wnt1 up vs E11.5 * Ctrl
10504551	Rg9mtd3	NM_027266	0.00338919	1.23682	E11.5 * Wnt1 up vs E11.5 * Ctrl
10393222	Srp68	NM_146032	0.00290017	1.23671	E11.5 * Wnt1 up vs E11.5 * Ctrl
10356379	Ecel1	NM_021306	0.00876867	1.23656	E11.5 * Wnt1 up vs E11.5 * Ctrl
10472440	Tax1bp3	NM_029564	0.000430184	1.23634	E11.5 * Wnt1 up vs E11.5 * Ctrl
10404827	Nol7	NM_023554	0.00491713	1.23626	E11.5 * Wnt1 up vs E11.5 * Ctrl
10438112	Pi4ka	NM_001001983	5.87653e-005	1.23625	E11.5 * Wnt1 up vs E11.5 * Ctrl
10543369	Cadps2	NM_153163	0.00150511	1.23624	E11.5 * Wnt1 up vs E11.5 * Ctrl
10415144	Ngdn	NM_026890	0.0084038	1.23605	E11.5 * Wnt1 up vs E11.5 * Ctrl
10480381	Arhgap21	NM_001128084	8.63661e-006	1.23602	E11.5 * Wnt1 up vs E11.5 * Ctrl
10439878	Psmc1	NM_008947	0.00248313	1.23597	E11.5 * Wnt1 up vs E11.5 * Ctrl
10534585	Sh2b2	NM_018825	0.00396173	1.23582	E11.5 * Wnt1 up vs E11.5 * Ctrl
10363000	Gpx4	NM_001037741	0.00291641	1.23574	E11.5 * Wnt1 up vs E11.5 * Ctrl
10429081	Snrpc	NM_011432	0.00233833	1.23568	E11.5 * Wnt1 up vs E11.5 * Ctrl
10419810	Psmb5	NM_011186	0.00146693	1.23521	E11.5 * Wnt1 up vs E11.5 * Ctrl
10405094	Iars	NM_172015	9.62728e-005	1.23507	E11.5 * Wnt1 up vs E11.5 * Ctrl
10472764	Dync1i2	NM_010064	0.000268209	1.23507	E11.5 * Wnt1 up vs E11.5 * Ctrl
10458906	Ppic	NM_008908	5.67191e-005	1.23494	E11.5 * Wnt1 up vs E11.5 * Ctrl
10448765	Mapk8ip3	NM_013931	0.000635078	1.23489	E11.5 * Wnt1 up vs E11.5 * Ctrl
10513397	1110054O05Rik	NM_001013577	0.000338346	1.23476	E11.5 * Wnt1 up vs E11.5 * Ctrl
10470322	Surf2	NM_013678	0.000983778	1.23473	E11.5 * Wnt1 up vs E11.5 * Ctrl

10530851	Mtch2	NM_019758	0.00588327	1.23457	E11.5 * Wnt1 up vs E11.5 * Ctrl
10595000	Tmod3	NM_016963	0.00840434	1.23416	E11.5 * Wnt1 up vs E11.5 * Ctrl
10457888	5730494M16Rik	NM_001004361	1.98655e-005	1.23397	E11.5 * Wnt1 up vs E11.5 * Ctrl
10558345	Dock1	NM_001033420	0.000564796	1.2332	E11.5 * Wnt1 up vs E11.5 * Ctrl
10549854	Zfp28	NM_175247	0.00786935	1.23315	E11.5 * Wnt1 up vs E11.5 * Ctrl
10346323	02-Mar	NM_175439	0.00385958	1.23311	E11.5 * Wnt1 up vs E11.5 * Ctrl
10557399	Sbk1	NM_145587	0.00308834	1.23303	E11.5 * Wnt1 up vs E11.5 * Ctrl
10458498	Arap3	NM_139206	0.00498245	1.23285	E11.5 * Wnt1 up vs E11.5 * Ctrl
10602840	Sh3kbp1	NM_021389	1.165e-005	1.2326	E11.5 * Wnt1 up vs E11.5 * Ctrl
10392070	Strada	NM_028126	0.00888977	1.23257	E11.5 * Wnt1 up vs E11.5 * Ctrl
10388747	BC017647	ENSMUST00000	0.00430773	1.23252	E11.5 * Wnt1 up vs E11.5 * Ctrl
10594289	Glce	NM_033320	0.00252186	1.23183	E11.5 * Wnt1 up vs E11.5 * Ctrl
10368484	ENSMUSG0000C	ENSMUST00000	0.00610006	1.23182	E11.5 * Wnt1 up vs E11.5 * Ctrl
10367697	Ppp1r14c	NM_133485	0.00222494	1.23121	E11.5 * Wnt1 up vs E11.5 * Ctrl
10582501	Fanca	NM_016925	0.00160405	1.23106	E11.5 * Wnt1 up vs E11.5 * Ctrl
10579825	Pou4f2	NM_138944	0.000407879	1.23105	E11.5 * Wnt1 up vs E11.5 * Ctrl
10358191	Camsap1l1	NM_001081360	4.22974e-005	1.23091	E11.5 * Wnt1 up vs E11.5 * Ctrl
10581996	Cdyl2	NM_029441	0.00395813	1.23075	E11.5 * Wnt1 up vs E11.5 * Ctrl
10495111	Wdr77	NM_027432	0.00122949	1.23066	E11.5 * Wnt1 up vs E11.5 * Ctrl
10557035	Polr3e	NM_025298	0.0049477	1.23059	E11.5 * Wnt1 up vs E11.5 * Ctrl
10589511	Scap	NM_001001144	0.00584338	1.23057	E11.5 * Wnt1 up vs E11.5 * Ctrl
10474545	Slc12a6	NM_133649	0.0017135	1.23039	E11.5 * Wnt1 up vs E11.5 * Ctrl
10374348	OTTMUSG00000	XR_032931	0.00162214	1.23029	E11.5 * Wnt1 up vs E11.5 * Ctrl
10384811	Ccdc104	NM_025740	0.00852259	1.23025	E11.5 * Wnt1 up vs E11.5 * Ctrl
10476668	Csrp2bp	NM_181417	0.00125074	1.2302	E11.5 * Wnt1 up vs E11.5 * Ctrl
10505705	Sh3gl2	NM_019535	0.00797411	1.23012	E11.5 * Wnt1 up vs E11.5 * Ctrl
10601760	Hnrnph2	NM_019868	0.00161537	1.22989	E11.5 * Wnt1 up vs E11.5 * Ctrl
10404928	C78339	NM_001033192	0.000498659	1.22983	E11.5 * Wnt1 up vs E11.5 * Ctrl
10518726	Slc25a33	NM_027460	0.00238571	1.22978	E11.5 * Wnt1 up vs E11.5 * Ctrl
10525296	Brap	NM_028227	0.000590022	1.22964	E11.5 * Wnt1 up vs E11.5 * Ctrl
10605929	Awat2	NM_177746	0.00650306	1.2295	E11.5 * Wnt1 up vs E11.5 * Ctrl
10461497	Ddb1	NM_015735	0.00620711	1.22935	E11.5 * Wnt1 up vs E11.5 * Ctrl
10546853	Srgap3	NM_080448	0.00498905	1.22921	E11.5 * Wnt1 up vs E11.5 * Ctrl
10358726	Tsen15	NM_025677	0.00527269	1.22918	E11.5 * Wnt1 up vs E11.5 * Ctrl
10403765	Vps41	NM_172120	0.00131044	1.22913	E11.5 * Wnt1 up vs E11.5 * Ctrl
10489266	Chd6	NM_173368	0.000493989	1.22904	E11.5 * Wnt1 up vs E11.5 * Ctrl
10550925	Zfp428	NM_146183	0.000208201	1.2287	E11.5 * Wnt1 up vs E11.5 * Ctrl
10382010	Wdr68	NM_027946	0.000431277	1.22857	E11.5 * Wnt1 up vs E11.5 * Ctrl
10510305	Frap1	NM_020009	0.00494803	1.2284	E11.5 * Wnt1 up vs E11.5 * Ctrl
10578619	Cdkn2aip	NM_172407	0.00306564	1.2282	E11.5 * Wnt1 up vs E11.5 * Ctrl
10453792	Thoc1	BC024951	0.00866956	1.2281	E11.5 * Wnt1 up vs E11.5 * Ctrl
10589087	Prkar2a	NM_008924	0.00646856	1.22797	E11.5 * Wnt1 up vs E11.5 * Ctrl
10468929	Nmt2	NM_008708	0.00390351	1.22773	E11.5 * Wnt1 up vs E11.5 * Ctrl
10431726	Yaf2	NM_024189	0.000795096	1.22762	E11.5 * Wnt1 up vs E11.5 * Ctrl
10426016	Gtse1	NM_013882	0.00260188	1.22743	E11.5 * Wnt1 up vs E11.5 * Ctrl
10438517	Alg3	NM_145939	0.00461496	1.22729	E11.5 * Wnt1 up vs E11.5 * Ctrl
10603373	Pqbp1	NM_019478	0.00198887	1.22721	E11.5 * Wnt1 up vs E11.5 * Ctrl
10489065	Ndrg3	NM_013865	0.00255716	1.22719	E11.5 * Wnt1 up vs E11.5 * Ctrl
10364728	Mum1	NM_023431	0.00302476	1.22698	E11.5 * Wnt1 up vs E11.5 * Ctrl
10364955	Thop1	NM_022653	0.0080032	1.22679	E11.5 * Wnt1 up vs E11.5 * Ctrl
10448593	Traf7	NM_153792	0.00242642	1.22651	E11.5 * Wnt1 up vs E11.5 * Ctrl
10448292	Thoc6	NM_001008425	0.00432532	1.22561	E11.5 * Wnt1 up vs E11.5 * Ctrl
10586477	Ppib	NM_011149	0.000537239	1.22556	E11.5 * Wnt1 up vs E11.5 * Ctrl
10457780	Fam59a	NM_001033445	0.00728391	1.22534	E11.5 * Wnt1 up vs E11.5 * Ctrl
10418171	Zcchc24	NM_001101433	0.00779462	1.22518	E11.5 * Wnt1 up vs E11.5 * Ctrl
10558903	Taldo1	NM_011528	0.00504626	1.22515	E11.5 * Wnt1 up vs E11.5 * Ctrl
10418578	Nisch	NM_022656	0.000782354	1.22489	E11.5 * Wnt1 up vs E11.5 * Ctrl
10591497	Kri1	NM_145416	0.00803663	1.22482	E11.5 * Wnt1 up vs E11.5 * Ctrl
10345666	Pdcl3	NM_026850	0.00217445	1.22416	E11.5 * Wnt1 up vs E11.5 * Ctrl
10601390	Pgk1	NM_008828	4.299e-006	1.22396	E11.5 * Wnt1 up vs E11.5 * Ctrl
10418796	Dph3	NM_172254	0.000627497	1.22352	E11.5 * Wnt1 up vs E11.5 * Ctrl
10489784	Prex1	NM_177782	0.000582772	1.22334	E11.5 * Wnt1 up vs E11.5 * Ctrl
10520025	Psmc2	NM_011188	1.3476e-005	1.22325	E11.5 * Wnt1 up vs E11.5 * Ctrl
10375079	Ubtd2	NM_173784	0.000924758	1.22324	E11.5 * Wnt1 up vs E11.5 * Ctrl
10557139	Ubfd1	NM_138589	0.00828181	1.22305	E11.5 * Wnt1 up vs E11.5 * Ctrl
10419825	Acin1	NM_023190	0.00217435	1.22303	E11.5 * Wnt1 up vs E11.5 * Ctrl

10457054	Zadh2	NM_146090	0.00869444	1.22268	E11.5 * Wnt1 up vs E11.5 * Ctrl
10455989	Rbm22	NM_025776	0.000390741	1.22204	E11.5 * Wnt1 up vs E11.5 * Ctrl
10605919	Pja1	NM_001083110	4.79763e-005	1.22201	E11.5 * Wnt1 up vs E11.5 * Ctrl
10369290	Ddit4	NM_029083	0.000705593	1.22197	E11.5 * Wnt1 up vs E11.5 * Ctrl
10458804	EG225468	XM_911153	0.00319996	1.2218	E11.5 * Wnt1 up vs E11.5 * Ctrl
10467941	Cwf19l1	NM_001081077	0.00564041	1.22172	E11.5 * Wnt1 up vs E11.5 * Ctrl
10470936	Tbc1d13	NM_146252	0.00103026	1.22147	E11.5 * Wnt1 up vs E11.5 * Ctrl
10493565	Adar	NM_001038587	0.000163245	1.22142	E11.5 * Wnt1 up vs E11.5 * Ctrl
10502522	Hs2st1	NM_011828	5.15325e-006	1.22137	E11.5 * Wnt1 up vs E11.5 * Ctrl
10502058	BC002199	NM_145964	0.000111179	1.22084	E11.5 * Wnt1 up vs E11.5 * Ctrl
10449386	D17Wsu92e	NM_001033279	0.000658964	1.22077	E11.5 * Wnt1 up vs E11.5 * Ctrl
10381668	Nmt1	NM_008707	0.000578992	1.22075	E11.5 * Wnt1 up vs E11.5 * Ctrl
10546113	Sec61a1	NM_016906	0.000263827	1.22066	E11.5 * Wnt1 up vs E11.5 * Ctrl
10471438	Dpm2	NM_010073	0.00415537	1.2205	E11.5 * Wnt1 up vs E11.5 * Ctrl
10533007	Ccdc64	NM_001080808	0.00134878	1.22049	E11.5 * Wnt1 up vs E11.5 * Ctrl
10414623	E130112L23Rik	NM_198249	0.00269978	1.22015	E11.5 * Wnt1 up vs E11.5 * Ctrl
10541002	D6Wsu116e	BC056942	0.00227313	1.21974	E11.5 * Wnt1 up vs E11.5 * Ctrl
10375667	Rnf130	NM_021540	0.00861566	1.21967	E11.5 * Wnt1 up vs E11.5 * Ctrl
10481634	Slc25a25	NM_146118	0.00323007	1.21964	E11.5 * Wnt1 up vs E11.5 * Ctrl
10587012	Ccpg1	NM_001114328	0.0056041	1.21957	E11.5 * Wnt1 up vs E11.5 * Ctrl
10379901	Bcas3	NM_138681	0.00454436	1.21951	E11.5 * Wnt1 up vs E11.5 * Ctrl
10384672	Ahsa2	NM_172391	0.000718063	1.21944	E11.5 * Wnt1 up vs E11.5 * Ctrl
10605512	LOC636082	ENSMUST00000(	0.000859334	1.21858	E11.5 * Wnt1 up vs E11.5 * Ctrl
10431229	Celsr1	NM_009886	0.00310412	1.21851	E11.5 * Wnt1 up vs E11.5 * Ctrl
10427910	Fam105b	NM_001013792	0.00834665	1.21839	E11.5 * Wnt1 up vs E11.5 * Ctrl
10602062	Prps1	NM_021463	0.00770971	1.21835	E11.5 * Wnt1 up vs E11.5 * Ctrl
10407513	Wdr37	NM_172445	0.00602605	1.21746	E11.5 * Wnt1 up vs E11.5 * Ctrl
10563583	Saal1	NM_030233	0.00372334	1.21723	E11.5 * Wnt1 up vs E11.5 * Ctrl
10488482	Acss1	NM_080575	0.00262132	1.2171	E11.5 * Wnt1 up vs E11.5 * Ctrl
10510814	BC046331	NM_177673	0.00618281	1.21651	E11.5 * Wnt1 up vs E11.5 * Ctrl
10360884	Iars2	NM_198653	0.00255177	1.21639	E11.5 * Wnt1 up vs E11.5 * Ctrl
10550022	Psip1	NM_133948	0.000242005	1.21637	E11.5 * Wnt1 up vs E11.5 * Ctrl
10425354	Mgat3	NM_010795	0.000871268	1.21633	E11.5 * Wnt1 up vs E11.5 * Ctrl
10449394	Taf11	NM_026836	0.000292244	1.21618	E11.5 * Wnt1 up vs E11.5 * Ctrl
10479649	Prpf6	NM_133701	0.000395621	1.21616	E11.5 * Wnt1 up vs E11.5 * Ctrl
10486552	Lrrc57	NM_001159612	0.00187009	1.2161	E11.5 * Wnt1 up vs E11.5 * Ctrl
10569771	Mcoln1	NM_053177	0.00308219	1.21568	E11.5 * Wnt1 up vs E11.5 * Ctrl
10389421	Ints2	NM_027421	0.00167465	1.21567	E11.5 * Wnt1 up vs E11.5 * Ctrl
10494662	Ywhah	NM_011738	0.00027219	1.21565	E11.5 * Wnt1 up vs E11.5 * Ctrl
10541711	Phb2	NM_007531	0.00246885	1.21543	E11.5 * Wnt1 up vs E11.5 * Ctrl
10349512	Ubxn4	NM_026390	5.63295e-005	1.21543	E11.5 * Wnt1 up vs E11.5 * Ctrl
10582295	Odc1	NM_013614	0.00339249	1.2152	E11.5 * Wnt1 up vs E11.5 * Ctrl
10492078	Alg5	NM_025442	0.00370554	1.21445	E11.5 * Wnt1 up vs E11.5 * Ctrl
10388625	Efcab5	NM_176965	0.00690477	1.2143	E11.5 * Wnt1 up vs E11.5 * Ctrl
10529313	Letm1	NM_019694	0.00305345	1.21406	E11.5 * Wnt1 up vs E11.5 * Ctrl
10512098	Aptx	NM_025545	0.00880799	1.21405	E11.5 * Wnt1 up vs E11.5 * Ctrl
10582699	2810004N23Rik	NM_025615	0.00370025	1.21369	E11.5 * Wnt1 up vs E11.5 * Ctrl
10427162	Mfsd5	NM_134100	0.00145034	1.21363	E11.5 * Wnt1 up vs E11.5 * Ctrl
10363599	Rufy2	NM_027425	0.00632092	1.2136	E11.5 * Wnt1 up vs E11.5 * Ctrl
10451918	Ubxn6	NM_024432	0.000635007	1.21333	E11.5 * Wnt1 up vs E11.5 * Ctrl
10550345	Strn4	NM_133789	0.00313806	1.21309	E11.5 * Wnt1 up vs E11.5 * Ctrl
10604922	BC023829	NM_001033328	0.00344325	1.21292	E11.5 * Wnt1 up vs E11.5 * Ctrl
10401713	Pomt2	NM_153415	0.00234724	1.21282	E11.5 * Wnt1 up vs E11.5 * Ctrl
10497090	Ankrd13c	NM_001013806	0.00231536	1.21275	E11.5 * Wnt1 up vs E11.5 * Ctrl
10407543	Gtpbp4	NM_027000	0.000447661	1.21271	E11.5 * Wnt1 up vs E11.5 * Ctrl
10423185	Rnasen	NM_001130149	0.00451688	1.21262	E11.5 * Wnt1 up vs E11.5 * Ctrl
10485225	Ext2	NM_010163	0.00654061	1.21262	E11.5 * Wnt1 up vs E11.5 * Ctrl
10363706	Jmjd1c	NM_207221	0.00361565	1.21261	E11.5 * Wnt1 up vs E11.5 * Ctrl
10520234	2010209O12Rik	NM_133913	0.000291357	1.21238	E11.5 * Wnt1 up vs E11.5 * Ctrl
10539669	Add2	NM_013458	0.0010789	1.21232	E11.5 * Wnt1 up vs E11.5 * Ctrl
10459930	Ctdp1	NM_026295	0.00305376	1.21194	E11.5 * Wnt1 up vs E11.5 * Ctrl
10400326	Eapp	NM_025456	0.00700572	1.21176	E11.5 * Wnt1 up vs E11.5 * Ctrl
10372583	Rab3ip	NM_001003950	0.000926295	1.21152	E11.5 * Wnt1 up vs E11.5 * Ctrl
10537078	Mkln1	NM_013791	0.000556805	1.21146	E11.5 * Wnt1 up vs E11.5 * Ctrl
10526250	Abhd11	NM_145215	0.00309211	1.2109	E11.5 * Wnt1 up vs E11.5 * Ctrl
10482330	Atp6v1g1	NM_024173	0.00183624	1.21076	E11.5 * Wnt1 up vs E11.5 * Ctrl

10360270	Atp1a2	NM_178405	0.000818349	1.21066	E11.5 * Wnt1 up vs E11.5 * Ctrl
10573348	Cacna1a	NM_007578	0.00850918	1.2106	E11.5 * Wnt1 up vs E11.5 * Ctrl
10545780	Exoc6b	NM_177077	0.00434284	1.21054	E11.5 * Wnt1 up vs E11.5 * Ctrl
10356800	Hdlbp	NM_133808	4.43172e-006	1.21043	E11.5 * Wnt1 up vs E11.5 * Ctrl
10401805	Snw1	NM_025507	0.000150911	1.21008	E11.5 * Wnt1 up vs E11.5 * Ctrl
10582599	Nup133	NM_172288	0.0044779	1.20978	E11.5 * Wnt1 up vs E11.5 * Ctrl
10555695	Rrm1	NM_009103	0.000141076	1.20947	E11.5 * Wnt1 up vs E11.5 * Ctrl
10428238	Ubr5	NM_001081359	0.000930135	1.20945	E11.5 * Wnt1 up vs E11.5 * Ctrl
10460085	Cndp2	NM_023149	0.00113655	1.20933	E11.5 * Wnt1 up vs E11.5 * Ctrl
10546586	Tmf1	NM_001081111	0.00695663	1.20933	E11.5 * Wnt1 up vs E11.5 * Ctrl
10521031	Ywhah	NM_011738	0.00233788	1.20932	E11.5 * Wnt1 up vs E11.5 * Ctrl
10527732	Fry	ENSMUST00000(	0.00505056	1.20893	E11.5 * Wnt1 up vs E11.5 * Ctrl
10442870	Ccdc78	ENSMUST00000(	0.00560039	1.20882	E11.5 * Wnt1 up vs E11.5 * Ctrl
10479765	Suv39h2	NM_022724	0.000835163	1.20866	E11.5 * Wnt1 up vs E11.5 * Ctrl
10413795	Eaf1	NM_028932	0.000135631	1.20858	E11.5 * Wnt1 up vs E11.5 * Ctrl
10493585	Ube2q1	NM_027315	0.00157769	1.20853	E11.5 * Wnt1 up vs E11.5 * Ctrl
10535629	Pdap1	NM_001033313	0.00136779	1.20824	E11.5 * Wnt1 up vs E11.5 * Ctrl
10490773	Hnrnph2	NM_019868	0.00465139	1.2082	E11.5 * Wnt1 up vs E11.5 * Ctrl
10392936	Nt5c	NM_015807	0.00249102	1.20818	E11.5 * Wnt1 up vs E11.5 * Ctrl
10431266	Cerk	NM_145475	0.00376839	1.20799	E11.5 * Wnt1 up vs E11.5 * Ctrl
10375234	Nudcd2	NM_026023	0.000127488	1.20773	E11.5 * Wnt1 up vs E11.5 * Ctrl
10358717	1700025G04Rik	BC034723	0.000587718	1.20766	E11.5 * Wnt1 up vs E11.5 * Ctrl
10591270	Olfm2	NM_173777	0.00160211	1.20754	E11.5 * Wnt1 up vs E11.5 * Ctrl
10532187	Gak	NM_153569	0.000650122	1.20754	E11.5 * Wnt1 up vs E11.5 * Ctrl
10564849	2610034B18Rik	NM_027420	0.00205003	1.20741	E11.5 * Wnt1 up vs E11.5 * Ctrl
10380524	Slc35b1	NM_016752	0.000427791	1.20711	E11.5 * Wnt1 up vs E11.5 * Ctrl
10455533	Eif1a	NM_010120	0.00835789	1.20696	E11.5 * Wnt1 up vs E11.5 * Ctrl
10493247	Ubqln4	NM_033526	0.00371823	1.20651	E11.5 * Wnt1 up vs E11.5 * Ctrl
10359097	Tor1aip1	NM_144791	0.00300312	1.20621	E11.5 * Wnt1 up vs E11.5 * Ctrl
10456995	Txnl4a	NM_178604	0.00212594	1.20619	E11.5 * Wnt1 up vs E11.5 * Ctrl
10535282	Card11	NM_175362	0.00759838	1.20595	E11.5 * Wnt1 up vs E11.5 * Ctrl
10361828	Cited2	NM_010828	0.00862944	1.20588	E11.5 * Wnt1 up vs E11.5 * Ctrl
10511113	Cdc2l1	NM_007661	6.74105e-005	1.20581	E11.5 * Wnt1 up vs E11.5 * Ctrl
10401244	Actn1	NM_134156	0.00356881	1.20542	E11.5 * Wnt1 up vs E11.5 * Ctrl
10418218	Slmap	NM_032008	0.00136845	1.20517	E11.5 * Wnt1 up vs E11.5 * Ctrl
10426098	Creld2	NM_029720	0.000472308	1.20502	E11.5 * Wnt1 up vs E11.5 * Ctrl
10428232	Rrm2b	NM_199476	0.00768056	1.20488	E11.5 * Wnt1 up vs E11.5 * Ctrl
10573637	Phkb	NM_199446	0.00211033	1.20477	E11.5 * Wnt1 up vs E11.5 * Ctrl
10458913	Cep120	NM_178686	0.00618566	1.2045	E11.5 * Wnt1 up vs E11.5 * Ctrl
10394040	Csnk1d	NM_027874	0.00465684	1.2045	E11.5 * Wnt1 up vs E11.5 * Ctrl
10550734	Tomm40	NM_001109748	0.000418817	1.20425	E11.5 * Wnt1 up vs E11.5 * Ctrl
10369301	Chst3	NM_016803	0.00640252	1.20361	E11.5 * Wnt1 up vs E11.5 * Ctrl
10426292	Alg10b	NM_001033441	0.00584204	1.20342	E11.5 * Wnt1 up vs E11.5 * Ctrl
10424370	Trib1	NM_144549	0.00665085	1.20341	E11.5 * Wnt1 up vs E11.5 * Ctrl
10447589	Fbl	NM_007991	0.00144299	1.20335	E11.5 * Wnt1 up vs E11.5 * Ctrl
10376690	Llgl1	NM_008502	0.00886232	1.20286	E11.5 * Wnt1 up vs E11.5 * Ctrl
10461334	Mta2	NM_011842	0.0014844	1.20269	E11.5 * Wnt1 up vs E11.5 * Ctrl
10478772	Arfgef2	NM_001085495	0.00625569	1.20217	E11.5 * Wnt1 up vs E11.5 * Ctrl
10517312	Tmem57	NM_025382	0.00522049	1.20215	E11.5 * Wnt1 up vs E11.5 * Ctrl
10585037	Bud13	NM_146000	0.0059598	1.20208	E11.5 * Wnt1 up vs E11.5 * Ctrl
10473281	Itgav	NM_008402	0.00587249	1.2019	E11.5 * Wnt1 up vs E11.5 * Ctrl
10591369	Dnmt1	NM_010066	0.000644718	1.20138	E11.5 * Wnt1 up vs E11.5 * Ctrl
10426477	Snrpc	NM_011432	0.00359765	1.20134	E11.5 * Wnt1 up vs E11.5 * Ctrl
10429972	Cpsf1	NM_053193	0.000510051	1.20128	E11.5 * Wnt1 up vs E11.5 * Ctrl
10571288	Gtf2e2	NM_026584	0.0010607	1.20112	E11.5 * Wnt1 up vs E11.5 * Ctrl
10559513	Cab39	NM_133781	0.00259009	1.20093	E11.5 * Wnt1 up vs E11.5 * Ctrl
10480035	Pfkfb3	NM_133232	0.00445789	1.20082	E11.5 * Wnt1 up vs E11.5 * Ctrl
10471608	Cep110	NM_012018	0.00332914	1.20077	E11.5 * Wnt1 up vs E11.5 * Ctrl
10390271	Nfe2l1	NM_008686	0.00815154	1.20064	E11.5 * Wnt1 up vs E11.5 * Ctrl
10347460	Ttll4	NM_001014974	0.000243026	1.20035	E11.5 * Wnt1 up vs E11.5 * Ctrl
10388786	Supt6h	NM_009297	0.000692748	1.20019	E11.5 * Wnt1 up vs E11.5 * Ctrl
10415574	Ccni	NM_017367	0.00110269	1.2001	E11.5 * Wnt1 up vs E11.5 * Ctrl
10352348	Cnih4	NM_030131	0.00228815	1.20009	E11.5 * Wnt1 up vs E11.5 * Ctrl
10360934	Rrp15	NM_026041	0.00265899	1.20001	E11.5 * Wnt1 up vs E11.5 * Ctrl
10507933	Inpp5b	NM_008385	0.00270109	1.19992	E11.5 * Wnt1 up vs E11.5 * Ctrl
10510624	Klhl21	NM_001033352	0.00207933	1.19981	E11.5 * Wnt1 up vs E11.5 * Ctrl

10607724	Zrsr2	NM_178794	0.00220326	1.19844	E11.5 * Wnt1 up vs E11.5 * Ctrl
10348817	02-Sep	NM_001159719	0.00315001	1.19844	E11.5 * Wnt1 up vs E11.5 * Ctrl
10373924	Ascc2	NM_029291	0.0011384	1.1984	E11.5 * Wnt1 up vs E11.5 * Ctrl
10572637	Fam125a	NM_028617	0.00846534	1.19822	E11.5 * Wnt1 up vs E11.5 * Ctrl
10347163	Spag16	NM_029160	0.000183437	1.19816	E11.5 * Wnt1 up vs E11.5 * Ctrl
10503448	Mmp16	NM_019724	0.00206564	1.19812	E11.5 * Wnt1 up vs E11.5 * Ctrl
10385343	Ttc1	NM_133795	0.00161758	1.19792	E11.5 * Wnt1 up vs E11.5 * Ctrl
10569898	Evi5l	NM_001039578	0.00099904	1.19754	E11.5 * Wnt1 up vs E11.5 * Ctrl
10533327	Mapkapk5	NM_010765	0.00246274	1.19703	E11.5 * Wnt1 up vs E11.5 * Ctrl
10521136	Whsc1	NM_001081102	0.000880552	1.19696	E11.5 * Wnt1 up vs E11.5 * Ctrl
10345913	Nck2	NM_010879	0.00845988	1.19671	E11.5 * Wnt1 up vs E11.5 * Ctrl
10555550	Clpb	NM_009191	0.00252772	1.19615	E11.5 * Wnt1 up vs E11.5 * Ctrl
10579219	Ddx49	NM_001024922	0.00697052	1.19613	E11.5 * Wnt1 up vs E11.5 * Ctrl
10560842	Zfp94	NM_009568	0.00283754	1.19599	E11.5 * Wnt1 up vs E11.5 * Ctrl
10568956	Olfr60	NM_146955	0.00613699	1.19555	E11.5 * Wnt1 up vs E11.5 * Ctrl
10583942	Thyn1	NM_144543	0.00191089	1.19554	E11.5 * Wnt1 up vs E11.5 * Ctrl
10543835	Chchd3	NM_025336	0.00626632	1.19534	E11.5 * Wnt1 up vs E11.5 * Ctrl
10446044	Hdgfrp2	NM_008233	0.00260603	1.19493	E11.5 * Wnt1 up vs E11.5 * Ctrl
10457489	6030446N20Rik	BC094382	0.00379219	1.19491	E11.5 * Wnt1 up vs E11.5 * Ctrl
10383545	Foxk2	NM_001080932	0.00164082	1.19476	E11.5 * Wnt1 up vs E11.5 * Ctrl
10530215	1110003E01Rik	NM_133697	0.00672948	1.1946	E11.5 * Wnt1 up vs E11.5 * Ctrl
10460706	Syvn1	NM_028769	0.00486058	1.19445	E11.5 * Wnt1 up vs E11.5 * Ctrl
10399121	Ptprn2	NM_011215	0.00743132	1.19442	E11.5 * Wnt1 up vs E11.5 * Ctrl
10452087	Safb2	NM_001029979	0.00808021	1.19421	E11.5 * Wnt1 up vs E11.5 * Ctrl
10503845	Ube2j1	NM_019586	0.00789836	1.19417	E11.5 * Wnt1 up vs E11.5 * Ctrl
10447695		---	0.00108571	1.19386	E11.5 * Wnt1 up vs E11.5 * Ctrl
10498837	Etfdh	NM_025794	0.000124377	1.19365	E11.5 * Wnt1 up vs E11.5 * Ctrl
10494857	Nras	NM_010937	0.000528795	1.19364	E11.5 * Wnt1 up vs E11.5 * Ctrl
10380641	Hoxb5	NM_008268	0.00169148	1.19344	E11.5 * Wnt1 up vs E11.5 * Ctrl
10507557	Ebna1bp2	NM_026932	0.00239744	1.19268	E11.5 * Wnt1 up vs E11.5 * Ctrl
10373439	Zc3h10	NM_134003	0.00550173	1.19266	E11.5 * Wnt1 up vs E11.5 * Ctrl
10389865	Nme1	NM_008704	0.000102739	1.19246	E11.5 * Wnt1 up vs E11.5 * Ctrl
10460112		---	0.00688219	1.19239	E11.5 * Wnt1 up vs E11.5 * Ctrl
10367292	Cs	NM_026444	0.0030281	1.19234	E11.5 * Wnt1 up vs E11.5 * Ctrl
10488862	Ggt7	NM_144786	0.00329089	1.19225	E11.5 * Wnt1 up vs E11.5 * Ctrl
10348180	Eif4e2	NM_001039169	0.00774638	1.19215	E11.5 * Wnt1 up vs E11.5 * Ctrl
10555108	Clns1a	NM_023671	0.000358648	1.19213	E11.5 * Wnt1 up vs E11.5 * Ctrl
10365297	D10Wsu102e	BC094241	0.00491803	1.1918	E11.5 * Wnt1 up vs E11.5 * Ctrl
10476207	Atrn	NM_009730	0.00464889	1.19164	E11.5 * Wnt1 up vs E11.5 * Ctrl
10511084	Nadk	NM_138671	0.00865828	1.19157	E11.5 * Wnt1 up vs E11.5 * Ctrl
10379891	Ppm1d	NM_016910	0.00455529	1.19141	E11.5 * Wnt1 up vs E11.5 * Ctrl
10495243	Gstm5	NM_010360	0.00270155	1.19091	E11.5 * Wnt1 up vs E11.5 * Ctrl
10605740	Eif2s3x	NM_012010	0.00551909	1.19087	E11.5 * Wnt1 up vs E11.5 * Ctrl
10463557	Gbf1	NM_178930	0.000442168	1.19077	E11.5 * Wnt1 up vs E11.5 * Ctrl
10377550	Trp53	NM_001127233	0.00237326	1.19065	E11.5 * Wnt1 up vs E11.5 * Ctrl
10386495	Tom1l2	NM_153080	0.00248932	1.19035	E11.5 * Wnt1 up vs E11.5 * Ctrl
10522606	Exoc1	NM_027270	0.0065261	1.19032	E11.5 * Wnt1 up vs E11.5 * Ctrl
10387625	Chrnb1	NM_009601	0.00143959	1.19014	E11.5 * Wnt1 up vs E11.5 * Ctrl
10503401	Tmem55a	NM_028264	0.000969193	1.18972	E11.5 * Wnt1 up vs E11.5 * Ctrl
10425161	Lgals1	NM_008495	0.00160343	1.18939	E11.5 * Wnt1 up vs E11.5 * Ctrl
10525555	Mlxip	NM_133917	0.00157446	1.18912	E11.5 * Wnt1 up vs E11.5 * Ctrl
10454298	Zfp397	NM_027007	0.000516167	1.18877	E11.5 * Wnt1 up vs E11.5 * Ctrl
10470268	Pmpca	NM_173180	0.0032124	1.18876	E11.5 * Wnt1 up vs E11.5 * Ctrl
10593103	Rnf214	NM_178709	0.00508128	1.18845	E11.5 * Wnt1 up vs E11.5 * Ctrl
10453512	Kpna2	NM_010655	0.00855861	1.18817	E11.5 * Wnt1 up vs E11.5 * Ctrl
10456194	Napg	NM_028017	0.00265136	1.18814	E11.5 * Wnt1 up vs E11.5 * Ctrl
10546066	Isy1	NM_133934	0.00571142	1.18798	E11.5 * Wnt1 up vs E11.5 * Ctrl
10580309	1500041N16Rik	NM_026399	0.00740304	1.18796	E11.5 * Wnt1 up vs E11.5 * Ctrl
10457961	Sft2d3	ENSMUST00000(	0.00607214	1.18778	E11.5 * Wnt1 up vs E11.5 * Ctrl
10498038	Elf2	NM_023502	0.00354963	1.18777	E11.5 * Wnt1 up vs E11.5 * Ctrl
10529287	Fam53a	NM_178390	0.0049738	1.18768	E11.5 * Wnt1 up vs E11.5 * Ctrl
10382139	Psmd12	NM_025894	6.9069e-006	1.18762	E11.5 * Wnt1 up vs E11.5 * Ctrl
10541803	Chd4	NM_145979	0.00102872	1.18749	E11.5 * Wnt1 up vs E11.5 * Ctrl
10502537	Sh3glb1	NM_019464	0.00013953	1.18748	E11.5 * Wnt1 up vs E11.5 * Ctrl
10358057	Shisa4	NM_175259	0.000964513	1.18745	E11.5 * Wnt1 up vs E11.5 * Ctrl
10542310	Cdkn1b	NM_009875	0.00611129	1.18736	E11.5 * Wnt1 up vs E11.5 * Ctrl

10516658	Ccdc28b	NM_025455	0.00239488	1.18726	E11.5 * Wnt1 up vs E11.5 * Ctrl
10574184	Rspry1	NM_026274	0.00710045	1.18716	E11.5 * Wnt1 up vs E11.5 * Ctrl
10348558	Scly	NM_016717	0.0059221	1.1869	E11.5 * Wnt1 up vs E11.5 * Ctrl
10409645	Ubqln1	NM_152234	0.00032306	1.18676	E11.5 * Wnt1 up vs E11.5 * Ctrl
10384183	AB182283	NM_001081652	0.00287591	1.18671	E11.5 * Wnt1 up vs E11.5 * Ctrl
10401172	Vti1b	NM_016800	0.000793097	1.18667	E11.5 * Wnt1 up vs E11.5 * Ctrl
10499705	Hax1	NM_011826	0.00346279	1.18656	E11.5 * Wnt1 up vs E11.5 * Ctrl
10571653	Actg1	NM_009609	0.000404192	1.18647	E11.5 * Wnt1 up vs E11.5 * Ctrl
10592701	Tmem136	NM_001034863	0.00773861	1.18645	E11.5 * Wnt1 up vs E11.5 * Ctrl
10487154	Secisbp2l	NM_177608	0.00281744	1.18632	E11.5 * Wnt1 up vs E11.5 * Ctrl
10452937	Heatr5b	NM_001081179	0.00387632	1.18626	E11.5 * Wnt1 up vs E11.5 * Ctrl
10405971	Nsun2	NM_145354	0.000336895	1.18609	E11.5 * Wnt1 up vs E11.5 * Ctrl
10389269	Aatf	NM_019816	0.00378677	1.18602	E11.5 * Wnt1 up vs E11.5 * Ctrl
10560630	Tomm40	NM_001109748	0.00198305	1.18602	E11.5 * Wnt1 up vs E11.5 * Ctrl
10551469	Dyrk1b	NM_001037957	0.0077658	1.18593	E11.5 * Wnt1 up vs E11.5 * Ctrl
10402841	Brf1	NM_028193	0.00822864	1.18593	E11.5 * Wnt1 up vs E11.5 * Ctrl
10406881	Smn1	NM_011420	0.00791893	1.18582	E11.5 * Wnt1 up vs E11.5 * Ctrl
10388971	Utp6	NM_144826	0.00183077	1.18561	E11.5 * Wnt1 up vs E11.5 * Ctrl
10365830		---	0.00536238	1.18539	E11.5 * Wnt1 up vs E11.5 * Ctrl
10422608	Oxct1	NM_024188	2.17976e-005	1.18535	E11.5 * Wnt1 up vs E11.5 * Ctrl
10600765	Pcyt1b	NM_211138	0.0087403	1.18529	E11.5 * Wnt1 up vs E11.5 * Ctrl
10496742	2410004B18Rik	NM_025555	0.00648202	1.1852	E11.5 * Wnt1 up vs E11.5 * Ctrl
10546805	Ddx18	NM_025860	0.00574399	1.18501	E11.5 * Wnt1 up vs E11.5 * Ctrl
10352066	Sdccag8	NM_029756	0.00675286	1.18499	E11.5 * Wnt1 up vs E11.5 * Ctrl
10414433	6720456H20Rik	NM_172600	0.00809741	1.18475	E11.5 * Wnt1 up vs E11.5 * Ctrl
10395074	Myt1l	NM_001093775	0.00398448	1.18474	E11.5 * Wnt1 up vs E11.5 * Ctrl
10603431	Suv39h1	NM_011514	0.00587446	1.18462	E11.5 * Wnt1 up vs E11.5 * Ctrl
10479811	Mcm10	NM_027290	0.00735932	1.18379	E11.5 * Wnt1 up vs E11.5 * Ctrl
10500736	Vangl1	NM_177545	0.000862224	1.18368	E11.5 * Wnt1 up vs E11.5 * Ctrl
10558458	Ppp2r2d	NM_026391	0.00866092	1.18366	E11.5 * Wnt1 up vs E11.5 * Ctrl
10477630	Dynlrb1	NM_025947	4.93161e-008	1.18358	E11.5 * Wnt1 up vs E11.5 * Ctrl
10563991		---	0.00813363	1.1835	E11.5 * Wnt1 up vs E11.5 * Ctrl
10368930	1700021F05Rik	NM_026411	0.00376873	1.18317	E11.5 * Wnt1 up vs E11.5 * Ctrl
10568260	Zfp629	NM_177226	0.00749919	1.18283	E11.5 * Wnt1 up vs E11.5 * Ctrl
10582201	Cox4nb	NM_010926	0.00176093	1.18278	E11.5 * Wnt1 up vs E11.5 * Ctrl
10561868	Wdr62	BC054747	0.00850993	1.18267	E11.5 * Wnt1 up vs E11.5 * Ctrl
10407570	Zmynd11	NM_144516	0.00165276	1.18261	E11.5 * Wnt1 up vs E11.5 * Ctrl
10434869	Ccdc50	NM_026202	0.00111289	1.18252	E11.5 * Wnt1 up vs E11.5 * Ctrl
10455801	Phax	NM_019996	0.00135787	1.18251	E11.5 * Wnt1 up vs E11.5 * Ctrl
10406141	Brd9	NM_001024508	0.00328928	1.18168	E11.5 * Wnt1 up vs E11.5 * Ctrl
10470529	Olfm1	NM_019498	0.00703495	1.18151	E11.5 * Wnt1 up vs E11.5 * Ctrl
10538878	ENSMUSG0000C	ENSMUST00000	0.00815252	1.18126	E11.5 * Wnt1 up vs E11.5 * Ctrl
10592734	Cbl	NM_007619	0.00694142	1.18125	E11.5 * Wnt1 up vs E11.5 * Ctrl
10490225	Slmo2	NM_025531	0.00782135	1.18094	E11.5 * Wnt1 up vs E11.5 * Ctrl
10462346	Rcl1	NM_021525	0.0031581	1.18087	E11.5 * Wnt1 up vs E11.5 * Ctrl
10416588	1300010F03Rik	NM_027906	0.0015828	1.18061	E11.5 * Wnt1 up vs E11.5 * Ctrl
10579969	Zfp330	NM_145600	0.000903496	1.18031	E11.5 * Wnt1 up vs E11.5 * Ctrl
10411491	Tnpo1	NM_178716	0.000517437	1.17987	E11.5 * Wnt1 up vs E11.5 * Ctrl
10471519	Tor2a	NM_152800	0.00276128	1.17973	E11.5 * Wnt1 up vs E11.5 * Ctrl
10576101	Zc3h18	NM_001029993	0.00882572	1.17951	E11.5 * Wnt1 up vs E11.5 * Ctrl
10586274	2010321M09Rik	NM_175153	0.00663588	1.17941	E11.5 * Wnt1 up vs E11.5 * Ctrl
10505182	Gng10	NM_025277	0.000272051	1.17941	E11.5 * Wnt1 up vs E11.5 * Ctrl
10597575	Plcd1	NM_019676	0.00741006	1.17912	E11.5 * Wnt1 up vs E11.5 * Ctrl
10576056	Map1lc3b	NM_026160	0.00857818	1.17904	E11.5 * Wnt1 up vs E11.5 * Ctrl
10570321	Cul4a	NM_146207	0.00721158	1.17863	E11.5 * Wnt1 up vs E11.5 * Ctrl
10414522	Apex1	NM_009687	0.00134425	1.17852	E11.5 * Wnt1 up vs E11.5 * Ctrl
10484941	Madd	NM_145527	0.00201341	1.17819	E11.5 * Wnt1 up vs E11.5 * Ctrl
10397752	Calm1	NM_009790	1.30055e-005	1.17812	E11.5 * Wnt1 up vs E11.5 * Ctrl
10594645	Rab8b	NM_173413	0.00884204	1.17799	E11.5 * Wnt1 up vs E11.5 * Ctrl
10476702	Sec23b	NM_019787	0.00253579	1.17795	E11.5 * Wnt1 up vs E11.5 * Ctrl
10539822	Copg	NM_017477	0.0025913	1.17729	E11.5 * Wnt1 up vs E11.5 * Ctrl
10465686	Rtn3	NM_001003934	0.00106059	1.17726	E11.5 * Wnt1 up vs E11.5 * Ctrl
10462724	Tnks2	ENSMUST00000	0.00227061	1.17713	E11.5 * Wnt1 up vs E11.5 * Ctrl
10492480	Gfm1	NM_138591	0.00803261	1.17696	E11.5 * Wnt1 up vs E11.5 * Ctrl
10542445	Strap	NM_011499	0.000196722	1.17642	E11.5 * Wnt1 up vs E11.5 * Ctrl
10389674	Dynll2	NM_026556	0.000672943	1.17629	E11.5 * Wnt1 up vs E11.5 * Ctrl

10596570	Rbm15b	BC052180	0.00352395	1.17529	E11.5 * Wnt1 up vs E11.5 * Ctrl
10391242	Rab5c	NM_024456	0.00650401	1.17515	E11.5 * Wnt1 up vs E11.5 * Ctrl
10352947	Mrpl15	NM_025300	0.000903042	1.17499	E11.5 * Wnt1 up vs E11.5 * Ctrl
10552681	Josd2	NM_025368	0.00269055	1.17492	E11.5 * Wnt1 up vs E11.5 * Ctrl
10393021	Recql5	NM_130454	0.00800011	1.17459	E11.5 * Wnt1 up vs E11.5 * Ctrl
10588903	Usp4	NM_011678	0.00105522	1.17449	E11.5 * Wnt1 up vs E11.5 * Ctrl
10593367	Dlat	NM_145614	0.000947832	1.17443	E11.5 * Wnt1 up vs E11.5 * Ctrl
10424411	Tsg101	NM_021884	0.0063333	1.17345	E11.5 * Wnt1 up vs E11.5 * Ctrl
10583610	Ilf3	NM_010561	0.00196747	1.17334	E11.5 * Wnt1 up vs E11.5 * Ctrl
10389114	Nle1	NM_145431	0.00760754	1.17304	E11.5 * Wnt1 up vs E11.5 * Ctrl
10378508	Tsr1	NM_177325	0.00568243	1.17297	E11.5 * Wnt1 up vs E11.5 * Ctrl
10567576	Mettl9	NM_021554	0.00550874	1.17277	E11.5 * Wnt1 up vs E11.5 * Ctrl
10478938	Hax1	NM_011826	0.00176994	1.17245	E11.5 * Wnt1 up vs E11.5 * Ctrl
10605090	Idh3g	NM_008323	0.00203907	1.17214	E11.5 * Wnt1 up vs E11.5 * Ctrl
10580370	Dnaja2	NM_019794	3.08096e-005	1.1719	E11.5 * Wnt1 up vs E11.5 * Ctrl
10473793	Psmc3	NM_008948	0.00026258	1.17177	E11.5 * Wnt1 up vs E11.5 * Ctrl
10389601	1200011M11Rik	BC020005	0.00539078	1.17148	E11.5 * Wnt1 up vs E11.5 * Ctrl
10403361	Pitrm1	NM_145131	0.00552879	1.1714	E11.5 * Wnt1 up vs E11.5 * Ctrl
10511892		---	0.00804527	1.17128	E11.5 * Wnt1 up vs E11.5 * Ctrl
10438308	Ranbp1	NM_011239	0.000664343	1.17118	E11.5 * Wnt1 up vs E11.5 * Ctrl
10405662	Mak10	NM_030153	0.000602129	1.17118	E11.5 * Wnt1 up vs E11.5 * Ctrl
10476648	Dstn	NM_019771	0.00613003	1.17109	E11.5 * Wnt1 up vs E11.5 * Ctrl
10545450	Tgoln1	NM_009443	0.00764732	1.17085	E11.5 * Wnt1 up vs E11.5 * Ctrl
10388132	4933427D14Rik	BC043106	0.00502354	1.17084	E11.5 * Wnt1 up vs E11.5 * Ctrl
10576175	Cdh15	NM_007662	0.00286569	1.17069	E11.5 * Wnt1 up vs E11.5 * Ctrl
10503134	Sdcbp	NM_001098227	7.88883e-006	1.17019	E11.5 * Wnt1 up vs E11.5 * Ctrl
10578763	Sap30	NM_021788	0.0016422	1.16974	E11.5 * Wnt1 up vs E11.5 * Ctrl
10577070	Tubgcp3	NM_198031	0.00349105	1.16963	E11.5 * Wnt1 up vs E11.5 * Ctrl
10435784	BC002163	NR_002445	0.00336325	1.16954	E11.5 * Wnt1 up vs E11.5 * Ctrl
10466402	Eif4a1	NM_144958	0.000908969	1.16936	E11.5 * Wnt1 up vs E11.5 * Ctrl
10576191	Spg7	NM_153176	0.00100244	1.16924	E11.5 * Wnt1 up vs E11.5 * Ctrl
10443764	Slc37a1	NM_153062	0.000993214	1.16912	E11.5 * Wnt1 up vs E11.5 * Ctrl
10468816	2700078E11Rik	BC026363	0.00386586	1.16881	E11.5 * Wnt1 up vs E11.5 * Ctrl
10514590	Dock7	NM_026082	0.000258599	1.16876	E11.5 * Wnt1 up vs E11.5 * Ctrl
10572253	Sf4	NM_027481	0.00331405	1.16833	E11.5 * Wnt1 up vs E11.5 * Ctrl
10439991	Tfg	NM_019678	0.000226554	1.16815	E11.5 * Wnt1 up vs E11.5 * Ctrl
10406795	Gfm2	NM_177266	0.00342251	1.1677	E11.5 * Wnt1 up vs E11.5 * Ctrl
10603541		---	0.00270212	1.16756	E11.5 * Wnt1 up vs E11.5 * Ctrl
10468022	Mrpl43	NM_053164	0.0026998	1.16747	E11.5 * Wnt1 up vs E11.5 * Ctrl
10354168	Tbc1d8	NM_018775	0.00672998	1.16705	E11.5 * Wnt1 up vs E11.5 * Ctrl
10524314	Pitpnb	NM_019640	0.00197568	1.16698	E11.5 * Wnt1 up vs E11.5 * Ctrl
10391410	Becn1	NM_019584	0.00562417	1.16688	E11.5 * Wnt1 up vs E11.5 * Ctrl
10398195	Ccnk	NM_009832	0.00195167	1.1667	E11.5 * Wnt1 up vs E11.5 * Ctrl
10374908	Rtn4	NM_194054	0.00112438	1.16645	E11.5 * Wnt1 up vs E11.5 * Ctrl
10488929	Eif6	NM_010579	0.000942682	1.16641	E11.5 * Wnt1 up vs E11.5 * Ctrl
10386543	Pabpc4	NM_130881	0.00817277	1.16628	E11.5 * Wnt1 up vs E11.5 * Ctrl
10389701	Akap1	NM_009648	0.00880091	1.16571	E11.5 * Wnt1 up vs E11.5 * Ctrl
10561323	Map3k10	NM_001081292	0.00414376	1.16471	E11.5 * Wnt1 up vs E11.5 * Ctrl
10489413	Tomm34	NM_025996	0.0054302	1.16461	E11.5 * Wnt1 up vs E11.5 * Ctrl
10453916		---	0.00184642	1.16423	E11.5 * Wnt1 up vs E11.5 * Ctrl
10430945	Poldip3	NM_178627	0.00801743	1.16377	E11.5 * Wnt1 up vs E11.5 * Ctrl
10438358	05-Sep	NM_213614	0.00531979	1.16332	E11.5 * Wnt1 up vs E11.5 * Ctrl
10605831	Las1l	NM_152822	0.00739225	1.16299	E11.5 * Wnt1 up vs E11.5 * Ctrl
10347772	ENSMUSG0000C	ENSMUST00000(	0.00780602	1.16298	E11.5 * Wnt1 up vs E11.5 * Ctrl
10360863	Mark1	NM_145515	0.0025223	1.16288	E11.5 * Wnt1 up vs E11.5 * Ctrl
10477887	0610011L14Rik	NM_026661	0.00566272	1.16242	E11.5 * Wnt1 up vs E11.5 * Ctrl
10454709	Kif20a	NM_009004	0.000726924	1.16174	E11.5 * Wnt1 up vs E11.5 * Ctrl
10578539	Slc25a4	NM_007450	0.000298667	1.16171	E11.5 * Wnt1 up vs E11.5 * Ctrl
10451495	Ubr2	NM_146078	0.00059378	1.16073	E11.5 * Wnt1 up vs E11.5 * Ctrl
10578019	Nudc	NM_010948	0.00889762	1.16057	E11.5 * Wnt1 up vs E11.5 * Ctrl
10480057	Rbm17	NM_152824	0.00268277	1.16055	E11.5 * Wnt1 up vs E11.5 * Ctrl
10513824	Cdk5rap2	NM_145990	0.00777697	1.16015	E11.5 * Wnt1 up vs E11.5 * Ctrl
10441987	Chd1	NM_007690	0.000606863	1.1596	E11.5 * Wnt1 up vs E11.5 * Ctrl
10428052	Cct5	NM_007637	2.65102e-006	1.15882	E11.5 * Wnt1 up vs E11.5 * Ctrl
10504926	Rnf20	NM_182999	0.000817556	1.15873	E11.5 * Wnt1 up vs E11.5 * Ctrl
10539861	Rpn1	NM_133933	0.00577674	1.15871	E11.5 * Wnt1 up vs E11.5 * Ctrl

10604844	Sms	NM_009214	0.00744892	1.15856	E11.5 * Wnt1 up vs E11.5 * Ctrl
10597920	Zdhhc3	NM_026917	0.00271749	1.15855	E11.5 * Wnt1 up vs E11.5 * Ctrl
10426439	Pphln1	NM_146062	0.00221344	1.15848	E11.5 * Wnt1 up vs E11.5 * Ctrl
10385159	Rars	NM_025936	6.74611e-006	1.15836	E11.5 * Wnt1 up vs E11.5 * Ctrl
10486322		---	0.00542285	1.1582	E11.5 * Wnt1 up vs E11.5 * Ctrl
10493891	Ywhaz	NM_011740	1.1767e-005	1.15818	E11.5 * Wnt1 up vs E11.5 * Ctrl
10515590	Kdm4a	NM_172382	0.00799299	1.1581	E11.5 * Wnt1 up vs E11.5 * Ctrl
10479026	Rae1	NM_175112	0.000999509	1.15789	E11.5 * Wnt1 up vs E11.5 * Ctrl
10468037		---	0.00737918	1.15747	E11.5 * Wnt1 up vs E11.5 * Ctrl
10533285	Ptpn11	NM_011202	0.00826647	1.15742	E11.5 * Wnt1 up vs E11.5 * Ctrl
10530434	Commd8	NM_178599	0.00608606	1.15713	E11.5 * Wnt1 up vs E11.5 * Ctrl
10529425	Nop14	NM_029278	0.00362477	1.15617	E11.5 * Wnt1 up vs E11.5 * Ctrl
10360225	Timm23	NM_016897	0.0013913	1.15582	E11.5 * Wnt1 up vs E11.5 * Ctrl
10416069	Timm23	NM_016897	0.0013913	1.15582	E11.5 * Wnt1 up vs E11.5 * Ctrl
10569134	Deaf1	NM_016874	0.00740436	1.15581	E11.5 * Wnt1 up vs E11.5 * Ctrl
10413697	Pbrm1	NM_001081251	0.00680324	1.15573	E11.5 * Wnt1 up vs E11.5 * Ctrl
10570373	Tfdp1	NM_009361	0.00460546	1.15562	E11.5 * Wnt1 up vs E11.5 * Ctrl
10586390	Rasl12	NM_001033158	0.00596494	1.15541	E11.5 * Wnt1 up vs E11.5 * Ctrl
10442829	Gng13	NM_022422	0.00579589	1.15531	E11.5 * Wnt1 up vs E11.5 * Ctrl
10406417	Actg1	NM_009609	0.000937385	1.1551	E11.5 * Wnt1 up vs E11.5 * Ctrl
10545940	Gmcl1	NM_011818	0.000205138	1.15501	E11.5 * Wnt1 up vs E11.5 * Ctrl
10380189	Mks1	NM_001039684	0.00479013	1.15469	E11.5 * Wnt1 up vs E11.5 * Ctrl
10400030	Bzw2	NM_025840	0.00881092	1.15383	E11.5 * Wnt1 up vs E11.5 * Ctrl
10446785	Spast	NM_016962	5.43465e-005	1.15327	E11.5 * Wnt1 up vs E11.5 * Ctrl
10528268	Ptpn12	NM_011203	0.0044328	1.15323	E11.5 * Wnt1 up vs E11.5 * Ctrl
10496475	Adh5	NM_007410	6.16761e-005	1.15302	E11.5 * Wnt1 up vs E11.5 * Ctrl
10356577	Ilkap	NM_023343	0.00850151	1.15267	E11.5 * Wnt1 up vs E11.5 * Ctrl
10577866	Ash2l	NM_011791	2.05121e-005	1.15259	E11.5 * Wnt1 up vs E11.5 * Ctrl
10350489	Uchl5	NM_019562	0.00219276	1.15238	E11.5 * Wnt1 up vs E11.5 * Ctrl
10460196	1810055G02Rik	NM_028077	0.00548884	1.15161	E11.5 * Wnt1 up vs E11.5 * Ctrl
10543031	Slc25a13	NM_015829	0.00442601	1.15157	E11.5 * Wnt1 up vs E11.5 * Ctrl
10457508	Npc1	NM_008720	0.0063516	1.15086	E11.5 * Wnt1 up vs E11.5 * Ctrl
10499839	Snapin	NM_133854	0.000972369	1.15005	E11.5 * Wnt1 up vs E11.5 * Ctrl
10505461	Atp6v1g1	NM_024173	0.00557858	1.14934	E11.5 * Wnt1 up vs E11.5 * Ctrl
10384529	Cep68	NM_172260	0.00774745	1.14911	E11.5 * Wnt1 up vs E11.5 * Ctrl
10475027	Tyro3	NM_019392	0.00694407	1.14786	E11.5 * Wnt1 up vs E11.5 * Ctrl
10601466	Tex16	NM_031382	0.00795098	1.14754	E11.5 * Wnt1 up vs E11.5 * Ctrl
10571441	Adam39	NM_001025380	0.0073065	1.1475	E11.5 * Wnt1 up vs E11.5 * Ctrl
10460926	Sf1	NM_001110791	0.00125835	1.1474	E11.5 * Wnt1 up vs E11.5 * Ctrl
10375980	Aff4	NM_033565	0.00573709	1.14699	E11.5 * Wnt1 up vs E11.5 * Ctrl
10353420	Mcm3	NM_008563	0.00665138	1.14674	E11.5 * Wnt1 up vs E11.5 * Ctrl
10545658	Wdr54	NM_023790	0.00423422	1.14665	E11.5 * Wnt1 up vs E11.5 * Ctrl
10458940	Zfp608	NM_175751	0.00681347	1.14631	E11.5 * Wnt1 up vs E11.5 * Ctrl
10531488	Ccni	NM_017367	0.00839691	1.14594	E11.5 * Wnt1 up vs E11.5 * Ctrl
10601164	Nono	NM_023144	0.00688589	1.14576	E11.5 * Wnt1 up vs E11.5 * Ctrl
10607524	Sms	NM_009214	0.0072536	1.14549	E11.5 * Wnt1 up vs E11.5 * Ctrl
10348004	Psmd1	NM_027357	0.00021083	1.1451	E11.5 * Wnt1 up vs E11.5 * Ctrl
10347254	Smarcal1	NM_018817	0.00553174	1.14464	E11.5 * Wnt1 up vs E11.5 * Ctrl
10580771	Ciapin1	NM_134141	0.00545536	1.144	E11.5 * Wnt1 up vs E11.5 * Ctrl
10604100	Ndufa1	NM_019443	0.00721451	1.14388	E11.5 * Wnt1 up vs E11.5 * Ctrl
10477004	LOC100044416	XR_030619	0.00258559	1.1433	E11.5 * Wnt1 up vs E11.5 * Ctrl
10532901	OTTMUSG00000	XR_033041	0.00527426	1.14329	E11.5 * Wnt1 up vs E11.5 * Ctrl
10593591	Acat1	NM_144784	0.00329679	1.14236	E11.5 * Wnt1 up vs E11.5 * Ctrl
10446109	Safb	ENSMUST00000	0.00102986	1.1412	E11.5 * Wnt1 up vs E11.5 * Ctrl
10515878	Ybx1	NM_011732	0.00126203	1.14101	E11.5 * Wnt1 up vs E11.5 * Ctrl
10418804	Timm23	NM_016897	0.00288286	1.141	E11.5 * Wnt1 up vs E11.5 * Ctrl
10531133	Grsf1	NM_178700	0.000414537	1.14029	E11.5 * Wnt1 up vs E11.5 * Ctrl
10591905	Glb1l3	NM_001113323	0.00818837	1.14006	E11.5 * Wnt1 up vs E11.5 * Ctrl
10437687	Litaf	NM_019980	0.00883164	1.13986	E11.5 * Wnt1 up vs E11.5 * Ctrl
10490946	Hsp90aa1	NM_010480	0.00796136	1.13956	E11.5 * Wnt1 up vs E11.5 * Ctrl
10363368	Timm23	NM_016897	0.00296987	1.1393	E11.5 * Wnt1 up vs E11.5 * Ctrl
10460221	Chka	NM_013490	0.00723587	1.13821	E11.5 * Wnt1 up vs E11.5 * Ctrl
10382069	Psmc5	NM_008950	0.000686009	1.13815	E11.5 * Wnt1 up vs E11.5 * Ctrl
10377987	Rabep1	NM_019400	0.00186424	1.13687	E11.5 * Wnt1 up vs E11.5 * Ctrl
10549497	3010003L21Rik	BC106181	0.00528522	1.13658	E11.5 * Wnt1 up vs E11.5 * Ctrl
10434233	Ufd1l	NM_011672	0.00283921	1.13634	E11.5 * Wnt1 up vs E11.5 * Ctrl

10356657	Ndufa10	NM_024197	0.00751325	1.13555	E11.5 * Wnt1 up vs E11.5 * Ctrl
10518642	Ube4b	NM_022022	0.00391367	1.1348	E11.5 * Wnt1 up vs E11.5 * Ctrl
10372750	Cand1	NM_027994	0.000922015	1.13392	E11.5 * Wnt1 up vs E11.5 * Ctrl
10478409	Pkig	NM_001039390	0.00532359	1.13391	E11.5 * Wnt1 up vs E11.5 * Ctrl
10569927	Map2k7	NM_001042557	0.00685645	1.13333	E11.5 * Wnt1 up vs E11.5 * Ctrl
10490544	Ythdf1	NM_173761	0.00119458	1.13252	E11.5 * Wnt1 up vs E11.5 * Ctrl
10390352	Kpnb1	NM_008379	0.00135822	1.13154	E11.5 * Wnt1 up vs E11.5 * Ctrl
10380732	Mrpl10	NM_026154	0.000680765	1.13076	E11.5 * Wnt1 up vs E11.5 * Ctrl
10501143	Ahcyl1	NM_145542	0.00335475	1.13075	E11.5 * Wnt1 up vs E11.5 * Ctrl
10580704	Amfr	NM_011787	0.000245883	1.13015	E11.5 * Wnt1 up vs E11.5 * Ctrl
10466848	D19Bwg1357e	NM_177474	0.00375361	1.12969	E11.5 * Wnt1 up vs E11.5 * Ctrl
10435149	Fyttd1	NM_027226	0.000721894	1.12888	E11.5 * Wnt1 up vs E11.5 * Ctrl
10601091	Foxo4	NM_018789	0.00555035	1.12851	E11.5 * Wnt1 up vs E11.5 * Ctrl
10523206	Uso1	NM_019490	0.0085735	1.12732	E11.5 * Wnt1 up vs E11.5 * Ctrl
10437748	Gspt1	NM_146066	0.00254906	1.12612	E11.5 * Wnt1 up vs E11.5 * Ctrl
10543650	Tnpo3	NM_177296	0.00133237	1.12599	E11.5 * Wnt1 up vs E11.5 * Ctrl
10542397	H2afj	NM_177688	0.00793151	1.12559	E11.5 * Wnt1 up vs E11.5 * Ctrl
10375926	Ppp2ca	NM_019411	0.00266738	1.12341	E11.5 * Wnt1 up vs E11.5 * Ctrl
10358359	Cdc73	NM_145991	0.00810467	1.12142	E11.5 * Wnt1 up vs E11.5 * Ctrl
10426650	Tuba1c	NM_009448	0.00628306	1.12108	E11.5 * Wnt1 up vs E11.5 * Ctrl
10607646	Nhs	NM_001081052	0.00846705	1.11924	E11.5 * Wnt1 up vs E11.5 * Ctrl
10539177	Eif4a3	NM_138669	0.00643872	1.11907	E11.5 * Wnt1 up vs E11.5 * Ctrl
10391178	Dnajc7	NM_019795	0.00776964	1.11773	E11.5 * Wnt1 up vs E11.5 * Ctrl
10393754	Actg1	NM_009609	0.0025074	1.11773	E11.5 * Wnt1 up vs E11.5 * Ctrl
10448269	Zfp13	NM_011747	0.0078256	1.11761	E11.5 * Wnt1 up vs E11.5 * Ctrl
10385790	Hspa4	NM_008300	0.0045299	1.11675	E11.5 * Wnt1 up vs E11.5 * Ctrl
10533659	Clip1	NM_019765	0.00667715	1.11662	E11.5 * Wnt1 up vs E11.5 * Ctrl
10459405	Nars	NM_001142950	0.00591002	1.11614	E11.5 * Wnt1 up vs E11.5 * Ctrl
10416940	Tpm3	ENSMUST00000	0.000776792	1.11569	E11.5 * Wnt1 up vs E11.5 * Ctrl
10517465	Kdm1	NM_133872	0.00327961	1.11565	E11.5 * Wnt1 up vs E11.5 * Ctrl
10432162	2310037I24Rik	BC003301	0.00234109	1.11448	E11.5 * Wnt1 up vs E11.5 * Ctrl
10541067	Eif4a3	NM_138669	0.00510959	1.11387	E11.5 * Wnt1 up vs E11.5 * Ctrl
10506424	Actg1	NM_009609	0.00372025	1.11269	E11.5 * Wnt1 up vs E11.5 * Ctrl
10501265	Gnai3	NM_010306	0.00775723	1.11184	E11.5 * Wnt1 up vs E11.5 * Ctrl
10490370	Psma7	NM_011969	0.000198223	1.11079	E11.5 * Wnt1 up vs E11.5 * Ctrl
10478463	Svs6	NM_013679	0.00505935	1.11034	E11.5 * Wnt1 up vs E11.5 * Ctrl
10422852	Rpl19	NM_009078	0.0014317	1.11028	E11.5 * Wnt1 up vs E11.5 * Ctrl
10454469	Ammecr1l	NM_153515	0.00651845	1.10959	E11.5 * Wnt1 up vs E11.5 * Ctrl
10548785	Dynlt1	NM_009342	0.00067515	1.10909	E11.5 * Wnt1 up vs E11.5 * Ctrl
10428192	Pabpc1	NM_008774	0.00430162	1.10711	E11.5 * Wnt1 up vs E11.5 * Ctrl
10529873	Rab2a	NM_021518	0.00703599	1.10692	E11.5 * Wnt1 up vs E11.5 * Ctrl
10447594	Dynlt1	NM_009342	0.000723253	1.1053	E11.5 * Wnt1 up vs E11.5 * Ctrl
10503150	Rab2a	NM_021518	0.000226815	1.10511	E11.5 * Wnt1 up vs E11.5 * Ctrl
10521587	Dnaja1	NM_008298	0.00566373	1.10473	E11.5 * Wnt1 up vs E11.5 * Ctrl
10352514	Eprs	NM_029735	0.00235006	1.10378	E11.5 * Wnt1 up vs E11.5 * Ctrl
10378802	Blmh	NM_178645	0.00798982	1.10291	E11.5 * Wnt1 up vs E11.5 * Ctrl
10344633	Tcea1	NM_011541	0.00877566	1.10287	E11.5 * Wnt1 up vs E11.5 * Ctrl
10385686	Hnrnpab	NM_001048061	0.00276406	1.10117	E11.5 * Wnt1 up vs E11.5 * Ctrl
10434878	ENSMUSG0000C	ENSMUST00000	0.00613638	1.09976	E11.5 * Wnt1 up vs E11.5 * Ctrl
10544015	Ybx1	NM_011732	0.000828922	1.09867	E11.5 * Wnt1 up vs E11.5 * Ctrl
10581910	Tmem170	NM_025781	0.00771619	1.09834	E11.5 * Wnt1 up vs E11.5 * Ctrl
10395831	Brms1l	NM_001037756	0.00686743	1.09825	E11.5 * Wnt1 up vs E11.5 * Ctrl
10407811	Hnrnpf	NM_133834	0.00215795	1.09328	E11.5 * Wnt1 up vs E11.5 * Ctrl
10506893	Nrd1	NM_146150	0.00144153	1.09281	E11.5 * Wnt1 up vs E11.5 * Ctrl
10570625	Rpl19	NM_009078	0.00520599	1.09172	E11.5 * Wnt1 up vs E11.5 * Ctrl
10485514	Caprin1	NM_016739	0.00250154	1.08976	E11.5 * Wnt1 up vs E11.5 * Ctrl
10380833	Rpl19	NM_009078	0.00704372	1.08878	E11.5 * Wnt1 up vs E11.5 * Ctrl
10535471	Rac1	NM_009007	0.00608749	1.07925	E11.5 * Wnt1 up vs E11.5 * Ctrl
10371981	Ccdc38	NM_175488	0.00220454	1.07501	E11.5 * Wnt1 up vs E11.5 * Ctrl
10541104	Hnrnpf	NM_133834	0.00470149	1.0699	E11.5 * Wnt1 up vs E11.5 * Ctrl
10348929	Hnrnpf	NM_133834	0.00783075	1.06706	E11.5 * Wnt1 up vs E11.5 * Ctrl
10598721	Rpl3	NM_013762	0.00233956	1.0596	E11.5 * Wnt1 up vs E11.5 * Ctrl
10400470	Cox6c	NM_053071	0.00455355	1.05841	E11.5 * Wnt1 up vs E11.5 * Ctrl
10404262	Rps8	NM_009098	0.00585484	1.05375	E11.5 * Wnt1 up vs E11.5 * Ctrl
10430669	Rpl3	NM_013762	0.00626344	1.05285	E11.5 * Wnt1 up vs E11.5 * Ctrl
10607324	Rpl21	NM_019647	0.00701948	-1.03697	E11.5 * Wnt1 down vs E11.5 * Ctrl

10443492	Rpl21	NM_019647	0.00744904	-1.03739	E11.5 * Wnt1 down vs E11.5 * Ctrl
10392996	EG668319	NM_001111313	0.00740912	-1.03905	E11.5 * Wnt1 down vs E11.5 * Ctrl
10579839	EG668319	NM_001111313	0.00389698	-1.0401	E11.5 * Wnt1 down vs E11.5 * Ctrl
10567823	EG668319	NM_001111313	0.00870792	-1.04104	E11.5 * Wnt1 down vs E11.5 * Ctrl
10587651	Rpl21	NM_019647	0.00565319	-1.04106	E11.5 * Wnt1 down vs E11.5 * Ctrl
10599433	EG668319	NM_001111313	0.00614722	-1.0411	E11.5 * Wnt1 down vs E11.5 * Ctrl
10497918	EG668319	NM_001111313	0.00654031	-1.04175	E11.5 * Wnt1 down vs E11.5 * Ctrl
10469904	Rpl21	NM_019647	0.00482758	-1.04255	E11.5 * Wnt1 down vs E11.5 * Ctrl
10401695	Rpl21	BC106116	0.00443245	-1.04586	E11.5 * Wnt1 down vs E11.5 * Ctrl
10598103	Rpl23a	NM_207523	0.00734855	-1.04699	E11.5 * Wnt1 down vs E11.5 * Ctrl
10388776	Rpl23a	NM_207523	0.00536864	-1.04927	E11.5 * Wnt1 down vs E11.5 * Ctrl
10562663	EG668668	NM_001081036	0.00804005	-1.05215	E11.5 * Wnt1 down vs E11.5 * Ctrl
10543317	Rpl21	NM_019647	0.00414135	-1.0538	E11.5 * Wnt1 down vs E11.5 * Ctrl
10521593	Rpl7	NM_011291	0.00852734	-1.057	E11.5 * Wnt1 down vs E11.5 * Ctrl
10593320	Rpl10	NM_052835	0.00819475	-1.07116	E11.5 * Wnt1 down vs E11.5 * Ctrl
10601857	Ngfrap1	NM_009750	0.00627041	-1.0742	E11.5 * Wnt1 down vs E11.5 * Ctrl
10348078	Ptma	NM_008972	0.00246187	-1.08441	E11.5 * Wnt1 down vs E11.5 * Ctrl
10600349	Rpl10	NM_052835	0.0046997	-1.08633	E11.5 * Wnt1 down vs E11.5 * Ctrl
10467256	Rpl10	NM_052835	0.00438297	-1.08749	E11.5 * Wnt1 down vs E11.5 * Ctrl
10409424	Mxd3	NM_016662	0.00847322	-1.08766	E11.5 * Wnt1 down vs E11.5 * Ctrl
10528478	Rpl17	NM_001002239	0.00602039	-1.08971	E11.5 * Wnt1 down vs E11.5 * Ctrl
10417601	Ptma	NM_008972	0.00407545	-1.09031	E11.5 * Wnt1 down vs E11.5 * Ctrl
10404020	V1rh2	NM_145844	0.00384804	-1.09059	E11.5 * Wnt1 down vs E11.5 * Ctrl
10513195	Txn1	NM_011660	0.000867752	-1.09067	E11.5 * Wnt1 down vs E11.5 * Ctrl
10396059	Rpl17	NM_001002239	0.00382653	-1.0974	E11.5 * Wnt1 down vs E11.5 * Ctrl
10564563	Rpl17	NM_001002239	0.00214601	-1.10007	E11.5 * Wnt1 down vs E11.5 * Ctrl
10549375	ENSMUSG0000C	NM_001111300	0.00734496	-1.10145	E11.5 * Wnt1 down vs E11.5 * Ctrl
10520952	Ppp1cb	NM_172707	0.00185167	-1.10367	E11.5 * Wnt1 down vs E11.5 * Ctrl
10357965	Lgr6	NM_001033409	0.00757946	-1.10369	E11.5 * Wnt1 down vs E11.5 * Ctrl
10600884	Rpl17	NM_001002239	0.00427423	-1.10528	E11.5 * Wnt1 down vs E11.5 * Ctrl
10594161	Arih1	NM_019927	0.00278275	-1.10536	E11.5 * Wnt1 down vs E11.5 * Ctrl
10552242	Rpl17	NM_001002239	0.00321428	-1.10656	E11.5 * Wnt1 down vs E11.5 * Ctrl
10591773	Hmgn2	NM_016957	0.00134343	-1.10838	E11.5 * Wnt1 down vs E11.5 * Ctrl
10537397	Luc7l2	NM_138680	0.00507889	-1.10858	E11.5 * Wnt1 down vs E11.5 * Ctrl
10398461	Ppp2r5c	NM_012023	0.00863526	-1.10977	E11.5 * Wnt1 down vs E11.5 * Ctrl
10533812	Sbno1	NM_001081203	0.00435141	-1.11078	E11.5 * Wnt1 down vs E11.5 * Ctrl
10483563	Tlk1	NM_172664	0.00695127	-1.11084	E11.5 * Wnt1 down vs E11.5 * Ctrl
10516823	Epb4.1	NM_183428	0.0018318	-1.11146	E11.5 * Wnt1 down vs E11.5 * Ctrl
10517141	Hmgn2	NM_016957	0.000849893	-1.11236	E11.5 * Wnt1 down vs E11.5 * Ctrl
10410244	Zfp640	ENSMUST00000(	0.00302468	-1.11241	E11.5 * Wnt1 down vs E11.5 * Ctrl
10451736	Tbc1d5	NM_028162	0.00856558	-1.11242	E11.5 * Wnt1 down vs E11.5 * Ctrl
10506500	Usp24	ENSMUST00000(	0.00611369	-1.11243	E11.5 * Wnt1 down vs E11.5 * Ctrl
10399897	Hbp1	NM_153198	0.00822856	-1.11377	E11.5 * Wnt1 down vs E11.5 * Ctrl
10405626	Rpl17	NM_001002239	0.00864941	-1.11402	E11.5 * Wnt1 down vs E11.5 * Ctrl
10579659	Hmgn2	NM_016957	0.00119106	-1.11468	E11.5 * Wnt1 down vs E11.5 * Ctrl
10422321	Dzip1	NM_025943	0.00306377	-1.11522	E11.5 * Wnt1 down vs E11.5 * Ctrl
10594986	Rpl17	NM_001002239	0.00279279	-1.11771	E11.5 * Wnt1 down vs E11.5 * Ctrl
10530666	Lnx1	NM_010727	0.00557909	-1.11933	E11.5 * Wnt1 down vs E11.5 * Ctrl
10422912	2410089E03Rik	BC058107	0.000727112	-1.11979	E11.5 * Wnt1 down vs E11.5 * Ctrl
10388591	Cpd	NM_007754	0.005821	-1.11988	E11.5 * Wnt1 down vs E11.5 * Ctrl
10360589	Ahctf1	NM_026375	0.00337399	-1.11988	E11.5 * Wnt1 down vs E11.5 * Ctrl
10522472	Fip1l1	NM_001159573	0.00483645	-1.12169	E11.5 * Wnt1 down vs E11.5 * Ctrl
10405163	Spin1	NM_146043	0.00256218	-1.1227	E11.5 * Wnt1 down vs E11.5 * Ctrl
10516103	Macf1	NM_009600	0.00613145	-1.1234	E11.5 * Wnt1 down vs E11.5 * Ctrl
10448004	Phf10	NM_024250	0.00328782	-1.12398	E11.5 * Wnt1 down vs E11.5 * Ctrl
10353296	Tceb1	NM_026456	0.00707287	-1.12468	E11.5 * Wnt1 down vs E11.5 * Ctrl
10353167	Tram1	NM_028173	0.00174482	-1.12489	E11.5 * Wnt1 down vs E11.5 * Ctrl
10428912		---	0.00206021	-1.12496	E11.5 * Wnt1 down vs E11.5 * Ctrl
10597933		---	0.0076019	-1.12607	E11.5 * Wnt1 down vs E11.5 * Ctrl
10346808	Rpl17	NM_001002239	0.0015358	-1.12743	E11.5 * Wnt1 down vs E11.5 * Ctrl
10458138	Brd8	NM_030147	0.00607139	-1.12798	E11.5 * Wnt1 down vs E11.5 * Ctrl
10523279		---	0.00379501	-1.12854	E11.5 * Wnt1 down vs E11.5 * Ctrl
10492682	1110032E23Rik	NM_133187	0.00606877	-1.12978	E11.5 * Wnt1 down vs E11.5 * Ctrl
10417013	Dnajc3	NM_008929	0.00630398	-1.13092	E11.5 * Wnt1 down vs E11.5 * Ctrl
10417034	Dnajc3	NM_008929	0.00630398	-1.13092	E11.5 * Wnt1 down vs E11.5 * Ctrl
10374020	Rhbdd3	NM_177370	0.00433635	-1.13141	E11.5 * Wnt1 down vs E11.5 * Ctrl

10566698	Olfr514	NM_146726	0.00417928	-1.13218	E11.5 * Wnt1 down vs E11.5 * Ctrl
10489936	Adnp	NM_009628	0.00727011	-1.13281	E11.5 * Wnt1 down vs E11.5 * Ctrl
10418702	Sh3bp5	NM_011894	0.0084444	-1.13356	E11.5 * Wnt1 down vs E11.5 * Ctrl
10400321	1110002B05Rik	NM_134054	0.00481796	-1.1354	E11.5 * Wnt1 down vs E11.5 * Ctrl
10394477	Laptm4a	NM_008640	0.00148921	-1.13588	E11.5 * Wnt1 down vs E11.5 * Ctrl
10558533		---	0.00599219	-1.13656	E11.5 * Wnt1 down vs E11.5 * Ctrl
10368670	Amd1	NM_009665	0.00196626	-1.13729	E11.5 * Wnt1 down vs E11.5 * Ctrl
10518781	Per3	NM_011067	0.00603313	-1.13749	E11.5 * Wnt1 down vs E11.5 * Ctrl
10366238	Ppp1r12a	NM_027892	0.000820329	-1.13762	E11.5 * Wnt1 down vs E11.5 * Ctrl
10345037	Paqr8	NM_028829	0.00111991	-1.13786	E11.5 * Wnt1 down vs E11.5 * Ctrl
10373192	Inhbe	NM_008382	0.0045341	-1.13816	E11.5 * Wnt1 down vs E11.5 * Ctrl
10374500	Vps54	NM_139061	0.000809019	-1.13854	E11.5 * Wnt1 down vs E11.5 * Ctrl
10391378	Ezh1	NM_007970	0.00231703	-1.13997	E11.5 * Wnt1 down vs E11.5 * Ctrl
10492442		---	0.00523431	-1.14076	E11.5 * Wnt1 down vs E11.5 * Ctrl
10575021	Zfp90	NM_011764	0.000438809	-1.14089	E11.5 * Wnt1 down vs E11.5 * Ctrl
10552488	Klk10	NM_133712	0.00856145	-1.14096	E11.5 * Wnt1 down vs E11.5 * Ctrl
10368380	L3mbtl3	NM_172787	0.00758429	-1.14107	E11.5 * Wnt1 down vs E11.5 * Ctrl
10380321	Mbtd1	NM_134012	0.00245745	-1.14482	E11.5 * Wnt1 down vs E11.5 * Ctrl
10441610	Rshl2a	NM_025789	0.00516168	-1.14524	E11.5 * Wnt1 down vs E11.5 * Ctrl
10379496	Spaca3	NM_029367	0.00546004	-1.14597	E11.5 * Wnt1 down vs E11.5 * Ctrl
10435714	Tmem39a	NM_026407	0.00233093	-1.14628	E11.5 * Wnt1 down vs E11.5 * Ctrl
10394258	Adcy3	NM_001159537	0.00737983	-1.14633	E11.5 * Wnt1 down vs E11.5 * Ctrl
10399189	Sp8	NM_177082	0.00498454	-1.14634	E11.5 * Wnt1 down vs E11.5 * Ctrl
10593789	Etfa	NM_145615	0.000516082	-1.14773	E11.5 * Wnt1 down vs E11.5 * Ctrl
10390707	Top2a	NM_011623	0.00294253	-1.15108	E11.5 * Wnt1 down vs E11.5 * Ctrl
10391914	Sh3d20	NM_183288	0.00850833	-1.15139	E11.5 * Wnt1 down vs E11.5 * Ctrl
10527832	Pds5b	NM_175310	0.000869401	-1.15308	E11.5 * Wnt1 down vs E11.5 * Ctrl
10601235	Ogt	NM_139144	0.001554	-1.15316	E11.5 * Wnt1 down vs E11.5 * Ctrl
10598034	ND2	ENSMUST00000(	0.00312732	-1.15324	E11.5 * Wnt1 down vs E11.5 * Ctrl
10437852	4921513D23Rik	NM_001081154	0.00195631	-1.15367	E11.5 * Wnt1 down vs E11.5 * Ctrl
10554166	Akap13	NM_029332	0.00741041	-1.15381	E11.5 * Wnt1 down vs E11.5 * Ctrl
10474399	Bdnf	NM_001048139	0.00369553	-1.15382	E11.5 * Wnt1 down vs E11.5 * Ctrl
10437664	Dexi	NM_021428	0.00867187	-1.15412	E11.5 * Wnt1 down vs E11.5 * Ctrl
10408144	V1rh9	NM_134218	0.00837218	-1.15448	E11.5 * Wnt1 down vs E11.5 * Ctrl
10365134		---	0.00525291	-1.1558	E11.5 * Wnt1 down vs E11.5 * Ctrl
10561748	1700067C01Rik	NM_029714	0.003339	-1.15677	E11.5 * Wnt1 down vs E11.5 * Ctrl
10410221	C330022B21Rik	ENSMUST00000(	0.0050448	-1.15741	E11.5 * Wnt1 down vs E11.5 * Ctrl
10530278		---	0.00442577	-1.15758	E11.5 * Wnt1 down vs E11.5 * Ctrl
10528183	Speer4e	NM_001122661	0.000777781	-1.15786	E11.5 * Wnt1 down vs E11.5 * Ctrl
10349100	EG626075	XR_032823	0.00114848	-1.15859	E11.5 * Wnt1 down vs E11.5 * Ctrl
10544252	E330009J07Rik	NM_175528	0.00676757	-1.15895	E11.5 * Wnt1 down vs E11.5 * Ctrl
10582429	Cbfa2t3	NM_009824	0.00350005	-1.15938	E11.5 * Wnt1 down vs E11.5 * Ctrl
10447141	Eml4	NM_001114361	0.00210334	-1.15945	E11.5 * Wnt1 down vs E11.5 * Ctrl
10405633	Ntrk2	NM_001025074	0.00366249	-1.16066	E11.5 * Wnt1 down vs E11.5 * Ctrl
10516027	Rlf	NM_001081013	0.00857039	-1.16119	E11.5 * Wnt1 down vs E11.5 * Ctrl
10349562	AA986860	NM_177604	0.00456143	-1.16211	E11.5 * Wnt1 down vs E11.5 * Ctrl
10380297	Cox11	NM_199008	0.00015253	-1.16212	E11.5 * Wnt1 down vs E11.5 * Ctrl
10354816	Clk1	NM_001042634	0.00555833	-1.16252	E11.5 * Wnt1 down vs E11.5 * Ctrl
10551293	Cyp2f2	NM_007817	0.00431849	-1.16253	E11.5 * Wnt1 down vs E11.5 * Ctrl
10570703	Defa25	NM_007849	0.00532781	-1.1632	E11.5 * Wnt1 down vs E11.5 * Ctrl
10551365	Prx	NM_019412	0.00880038	-1.16325	E11.5 * Wnt1 down vs E11.5 * Ctrl
10404008	V1rh6	NM_134215	0.00641043	-1.16333	E11.5 * Wnt1 down vs E11.5 * Ctrl
10382228	Axin2	NM_015732	0.00666203	-1.16335	E11.5 * Wnt1 down vs E11.5 * Ctrl
10542632	Iapp	NM_010491	0.00606953	-1.1634	E11.5 * Wnt1 down vs E11.5 * Ctrl
10522024	Tbc1d1	NM_019636	0.00116803	-1.16369	E11.5 * Wnt1 down vs E11.5 * Ctrl
10408175	Btn1a1	NM_013483	0.00287887	-1.1641	E11.5 * Wnt1 down vs E11.5 * Ctrl
10532241	Slc26a1	NM_174870	0.00171085	-1.16418	E11.5 * Wnt1 down vs E11.5 * Ctrl
10434094	Klhl22	NM_145479	0.00167855	-1.16421	E11.5 * Wnt1 down vs E11.5 * Ctrl
10599463	Xpnpep2	NM_133213	0.00855991	-1.16461	E11.5 * Wnt1 down vs E11.5 * Ctrl
10527575	Pan3	NM_028291	0.00384708	-1.16484	E11.5 * Wnt1 down vs E11.5 * Ctrl
10479852	Camk1d	NM_177343	0.00849809	-1.16518	E11.5 * Wnt1 down vs E11.5 * Ctrl
10413333	Dnahc12	Z83811	0.00157686	-1.16541	E11.5 * Wnt1 down vs E11.5 * Ctrl
10397359	Batf	NM_016767	0.00480176	-1.16583	E11.5 * Wnt1 down vs E11.5 * Ctrl
10417130	Ubac2	NM_026861	0.0066678	-1.16655	E11.5 * Wnt1 down vs E11.5 * Ctrl
10396205	Arid4a	NM_001081195	0.006197	-1.16663	E11.5 * Wnt1 down vs E11.5 * Ctrl
10411229	F2r	NM_010169	0.00496054	-1.16663	E11.5 * Wnt1 down vs E11.5 * Ctrl

10489484	Sdc4	NM_011521	0.00813292	-1.16691	E11.5 * Wnt1 down vs E11.5 * Ctrl
10369647	Ddx50	NM_053183	0.0045634	-1.16697	E11.5 * Wnt1 down vs E11.5 * Ctrl
10582390	Aprt	NM_009698	0.00602541	-1.16738	E11.5 * Wnt1 down vs E11.5 * Ctrl
10441680	Pde10a	NM_011866	0.00459511	-1.16748	E11.5 * Wnt1 down vs E11.5 * Ctrl
10497399	Pde7a	NM_001122759	0.00835405	-1.16756	E11.5 * Wnt1 down vs E11.5 * Ctrl
10428763	Atad2	NM_027435	0.00597493	-1.16764	E11.5 * Wnt1 down vs E11.5 * Ctrl
10548000	Ltbr	NM_010736	0.00504417	-1.1681	E11.5 * Wnt1 down vs E11.5 * Ctrl
10361075	Mfsd7b	NM_001081259	0.00605462	-1.16811	E11.5 * Wnt1 down vs E11.5 * Ctrl
10589350	Shisa5	NM_025858	0.00232604	-1.16929	E11.5 * Wnt1 down vs E11.5 * Ctrl
10450325	Cfb	NM_008198	0.00444935	-1.16939	E11.5 * Wnt1 down vs E11.5 * Ctrl
10473708	Agbl2	NM_178755	0.00611466	-1.16955	E11.5 * Wnt1 down vs E11.5 * Ctrl
10492558	Smc4	NM_133786	0.0021405	-1.17001	E11.5 * Wnt1 down vs E11.5 * Ctrl
10462480	Cstf2t	NM_031249	0.00844626	-1.17056	E11.5 * Wnt1 down vs E11.5 * Ctrl
10450025	March2	NM_145486	0.00451074	-1.17086	E11.5 * Wnt1 down vs E11.5 * Ctrl
10452508	Twsg1	NM_023053	0.00126809	-1.17096	E11.5 * Wnt1 down vs E11.5 * Ctrl
10435019	2310010M20Rik	BC125555	0.00835336	-1.17152	E11.5 * Wnt1 down vs E11.5 * Ctrl
10567229	Smg1	NM_001031814	0.00210907	-1.17176	E11.5 * Wnt1 down vs E11.5 * Ctrl
10597493	Stt3b	NM_024222	0.000304449	-1.17238	E11.5 * Wnt1 down vs E11.5 * Ctrl
10504466	OTTMUSG00000	AK038173	0.00438092	-1.1733	E11.5 * Wnt1 down vs E11.5 * Ctrl
10545127	4930597O21Rik	ENSMUST00000	0.00412029	-1.17331	E11.5 * Wnt1 down vs E11.5 * Ctrl
10360003	Dusp12	NM_023173	0.00692524	-1.17339	E11.5 * Wnt1 down vs E11.5 * Ctrl
10484648	Olfr1104	NM_146767	0.00174467	-1.17347	E11.5 * Wnt1 down vs E11.5 * Ctrl
10365658	Uhrf1bp1l	NM_029166	0.00462533	-1.17401	E11.5 * Wnt1 down vs E11.5 * Ctrl
10534889	Agfg2	NM_178162	0.00157751	-1.17415	E11.5 * Wnt1 down vs E11.5 * Ctrl
10537957	Krba1	NM_133922	0.00343642	-1.17428	E11.5 * Wnt1 down vs E11.5 * Ctrl
10457038	ENSMUSG00000	ENSMUST00000	0.00458647	-1.17453	E11.5 * Wnt1 down vs E11.5 * Ctrl
10461867	Vps13a	AK015803	0.00765973	-1.17472	E11.5 * Wnt1 down vs E11.5 * Ctrl
10381172	Stat5a	NM_011488	0.00256408	-1.17522	E11.5 * Wnt1 down vs E11.5 * Ctrl
10516784		---	0.00756152	-1.17559	E11.5 * Wnt1 down vs E11.5 * Ctrl
10455954	EG240327	NM_001033767	0.00569412	-1.17628	E11.5 * Wnt1 down vs E11.5 * Ctrl
10585625	Sin3a	NM_011378	0.00173289	-1.17641	E11.5 * Wnt1 down vs E11.5 * Ctrl
10384448	Sec61g	NM_011343	9.78316e-005	-1.17668	E11.5 * Wnt1 down vs E11.5 * Ctrl
10568174	Spn	NM_009259	0.00422958	-1.1772	E11.5 * Wnt1 down vs E11.5 * Ctrl
10519886	Sema3c	NM_013657	0.00699181	-1.17722	E11.5 * Wnt1 down vs E11.5 * Ctrl
10431845	A130051J06Rik	AK037810	0.00117973	-1.17759	E11.5 * Wnt1 down vs E11.5 * Ctrl
10417004	Dzip1	NM_025943	3.41362e-005	-1.17782	E11.5 * Wnt1 down vs E11.5 * Ctrl
10424381		---	0.00596756	-1.17786	E11.5 * Wnt1 down vs E11.5 * Ctrl
10587075		---	0.00173037	-1.17846	E11.5 * Wnt1 down vs E11.5 * Ctrl
10564938	Fes	NM_010194	0.000764153	-1.17882	E11.5 * Wnt1 down vs E11.5 * Ctrl
10599781	Htatsf1	NM_028242	0.0029421	-1.1791	E11.5 * Wnt1 down vs E11.5 * Ctrl
10372716	Rap1b	NM_024457	0.000935903	-1.17972	E11.5 * Wnt1 down vs E11.5 * Ctrl
10350046	Kdm5b	NM_152895	0.000341407	-1.18109	E11.5 * Wnt1 down vs E11.5 * Ctrl
10536898	Irf5	NM_012057	0.00695176	-1.1812	E11.5 * Wnt1 down vs E11.5 * Ctrl
10436087	Retnlb	NM_023881	0.00530422	-1.18155	E11.5 * Wnt1 down vs E11.5 * Ctrl
10570723	Defa25	NM_007849	0.00147924	-1.18161	E11.5 * Wnt1 down vs E11.5 * Ctrl
10527213	Daglb	NM_144915	0.00709893	-1.18162	E11.5 * Wnt1 down vs E11.5 * Ctrl
10348150	Chrnd	NM_021600	0.00518442	-1.18187	E11.5 * Wnt1 down vs E11.5 * Ctrl
10349184	Cdh7	NM_172853	0.00388641	-1.18195	E11.5 * Wnt1 down vs E11.5 * Ctrl
10554061	Adamts17	NM_001033877	0.00636695	-1.1823	E11.5 * Wnt1 down vs E11.5 * Ctrl
10378443	Olfr412	NM_001011851	0.0081871	-1.18281	E11.5 * Wnt1 down vs E11.5 * Ctrl
10396511	Syne2	NM_001005510	0.00796592	-1.18346	E11.5 * Wnt1 down vs E11.5 * Ctrl
10605542	2410003J06Rik	NM_001113734	0.00320245	-1.18356	E11.5 * Wnt1 down vs E11.5 * Ctrl
10604234	EG636756	XM_912249	0.00840904	-1.18356	E11.5 * Wnt1 down vs E11.5 * Ctrl
10584276	Sec61g	NM_011343	0.000496212	-1.18371	E11.5 * Wnt1 down vs E11.5 * Ctrl
10344939	Terf1	NM_009352	0.0018254	-1.18378	E11.5 * Wnt1 down vs E11.5 * Ctrl
10364784	Reep6	NM_139292	0.00165323	-1.18476	E11.5 * Wnt1 down vs E11.5 * Ctrl
10426261	Shank3	NM_021423	0.00488493	-1.1854	E11.5 * Wnt1 down vs E11.5 * Ctrl
10592217	Stt3a	NM_008408	0.00885688	-1.18571	E11.5 * Wnt1 down vs E11.5 * Ctrl
10512011	Zfp292	NM_013889	0.0016769	-1.18588	E11.5 * Wnt1 down vs E11.5 * Ctrl
10440463	LOC436427	XR_034439	0.00454319	-1.18591	E11.5 * Wnt1 down vs E11.5 * Ctrl
10527423	Trrap	NM_001081362	0.00644425	-1.18603	E11.5 * Wnt1 down vs E11.5 * Ctrl
10398578	Tecpr2	NM_001081057	0.00435704	-1.18672	E11.5 * Wnt1 down vs E11.5 * Ctrl
10580782	Dok4	NM_053246	0.00309903	-1.18705	E11.5 * Wnt1 down vs E11.5 * Ctrl
10366725	Fam19a2	NM_182807	0.00570479	-1.18721	E11.5 * Wnt1 down vs E11.5 * Ctrl
10600718	Sec61g	NM_011343	0.00173759	-1.18733	E11.5 * Wnt1 down vs E11.5 * Ctrl
10494583	Sec22b	NM_011342	0.000272607	-1.18739	E11.5 * Wnt1 down vs E11.5 * Ctrl

10421624	Cog3	NM_177381	0.00439913	-1.18778	E11.5 * Wnt1 down vs E11.5 * Ctrl
10569100		---	0.00654177	-1.18825	E11.5 * Wnt1 down vs E11.5 * Ctrl
10408064	Olfr11	NM_146542	0.00136658	-1.18866	E11.5 * Wnt1 down vs E11.5 * Ctrl
10455342		---	0.00048572	-1.18926	E11.5 * Wnt1 down vs E11.5 * Ctrl
10435075	Tfrc	NM_011638	0.00234269	-1.18931	E11.5 * Wnt1 down vs E11.5 * Ctrl
10384044	Myl7	NM_022879	0.00437685	-1.1896	E11.5 * Wnt1 down vs E11.5 * Ctrl
10470584	Tsc1	NM_022887	0.00319011	-1.1896	E11.5 * Wnt1 down vs E11.5 * Ctrl
10388086	Nlrp1c	NM_001039234	0.00485335	-1.18965	E11.5 * Wnt1 down vs E11.5 * Ctrl
10568951	Olfr522	NM_146952	0.00665502	-1.18969	E11.5 * Wnt1 down vs E11.5 * Ctrl
10415903	1700049K14Rik	BC049714	0.00571095	-1.19011	E11.5 * Wnt1 down vs E11.5 * Ctrl
10357391	Zranb3	NM_027678	0.0047269	-1.19039	E11.5 * Wnt1 down vs E11.5 * Ctrl
10577973	Adrb3	NM_013462	0.00321194	-1.19116	E11.5 * Wnt1 down vs E11.5 * Ctrl
10398332		---	0.00340073	-1.19184	E11.5 * Wnt1 down vs E11.5 * Ctrl
10584067	Zbtb44	NM_001115130	0.00528732	-1.19189	E11.5 * Wnt1 down vs E11.5 * Ctrl
10365714	Sec61g	NM_011343	0.000642401	-1.19228	E11.5 * Wnt1 down vs E11.5 * Ctrl
10416522	Tsc22d1	NM_207652	0.000286686	-1.19239	E11.5 * Wnt1 down vs E11.5 * Ctrl
10400762	Map4k5	NM_201519	0.00427122	-1.19253	E11.5 * Wnt1 down vs E11.5 * Ctrl
10383758	Tug1	NR_002321	0.00624516	-1.19297	E11.5 * Wnt1 down vs E11.5 * Ctrl
10447510	Amd1	NM_009665	0.00813711	-1.19307	E11.5 * Wnt1 down vs E11.5 * Ctrl
10453678	Zeb1	NM_011546	0.000716078	-1.1933	E11.5 * Wnt1 down vs E11.5 * Ctrl
10410016	Fancc	NM_007985	0.00161121	-1.19336	E11.5 * Wnt1 down vs E11.5 * Ctrl
10438530	Clcn2	NM_009900	7.76016e-005	-1.19359	E11.5 * Wnt1 down vs E11.5 * Ctrl
10568765	Tcerg1l	NM_183289	0.00459601	-1.19412	E11.5 * Wnt1 down vs E11.5 * Ctrl
10531342	U90926	U90926	0.00137599	-1.19413	E11.5 * Wnt1 down vs E11.5 * Ctrl
10477311	Asxl1	NM_001039939	0.00434032	-1.19435	E11.5 * Wnt1 down vs E11.5 * Ctrl
10595871	Clstn2	NM_022319	0.000792067	-1.19442	E11.5 * Wnt1 down vs E11.5 * Ctrl
10588326	Nphp3	NM_028721	0.00226426	-1.19446	E11.5 * Wnt1 down vs E11.5 * Ctrl
10373502	Ikzf4	NM_011772	0.00809549	-1.19463	E11.5 * Wnt1 down vs E11.5 * Ctrl
10498477	E130311K13Rik	NM_177856	0.00813944	-1.19488	E11.5 * Wnt1 down vs E11.5 * Ctrl
10499552	Efna4	NM_007910	0.0077622	-1.19578	E11.5 * Wnt1 down vs E11.5 * Ctrl
10517401	Grhl3	NM_001013756	0.00618225	-1.19601	E11.5 * Wnt1 down vs E11.5 * Ctrl
10395163	Lamb1-1	NM_008482	0.00029816	-1.19607	E11.5 * Wnt1 down vs E11.5 * Ctrl
10586110	Cln6	NM_001033175	0.00442272	-1.19609	E11.5 * Wnt1 down vs E11.5 * Ctrl
10391685	Fam171a2	NM_199200	0.00505354	-1.19614	E11.5 * Wnt1 down vs E11.5 * Ctrl
10608089	Ube1y1	NM_011667	0.00644154	-1.1968	E11.5 * Wnt1 down vs E11.5 * Ctrl
10398350		---	0.007653	-1.19792	E11.5 * Wnt1 down vs E11.5 * Ctrl
10447417	Msh6	NM_010830	0.00135236	-1.19814	E11.5 * Wnt1 down vs E11.5 * Ctrl
10393559	Timp2	NM_011594	0.000282962	-1.1982	E11.5 * Wnt1 down vs E11.5 * Ctrl
10432907	Krt78	NM_212487	0.00600707	-1.19841	E11.5 * Wnt1 down vs E11.5 * Ctrl
10546272	Iqsec1	NM_001134384	0.00496337	-1.19906	E11.5 * Wnt1 down vs E11.5 * Ctrl
10528177	ENSMUSG0000C	NM_198666	0.000634043	-1.19908	E11.5 * Wnt1 down vs E11.5 * Ctrl
10445781	Trem2	NM_031254	0.00301078	-1.19918	E11.5 * Wnt1 down vs E11.5 * Ctrl
10424335	Rnf139	NM_175226	0.00151012	-1.19919	E11.5 * Wnt1 down vs E11.5 * Ctrl
10608085		---	0.00639837	-1.19977	E11.5 * Wnt1 down vs E11.5 * Ctrl
10480321	A930004D18Rik	ENSMUST00000(	0.00642684	-1.19999	E11.5 * Wnt1 down vs E11.5 * Ctrl
10453939	Lama3	NM_010680	0.00806575	-1.2001	E11.5 * Wnt1 down vs E11.5 * Ctrl
10420694	Ints6	NM_008715	0.000187779	-1.20015	E11.5 * Wnt1 down vs E11.5 * Ctrl
10457223	Cdh11	NM_009866	0.000117229	-1.20016	E11.5 * Wnt1 down vs E11.5 * Ctrl
10553477	Ano5	NM_177694	0.00503728	-1.20041	E11.5 * Wnt1 down vs E11.5 * Ctrl
10390974	Krt34	NM_027563	0.00778606	-1.20068	E11.5 * Wnt1 down vs E11.5 * Ctrl
10437552	AU021092	NM_001033220	0.00143324	-1.20094	E11.5 * Wnt1 down vs E11.5 * Ctrl
10365242	Hcfc2	NM_001081218	0.00822192	-1.20102	E11.5 * Wnt1 down vs E11.5 * Ctrl
10358978	Ier5	NM_010500	0.00306044	-1.20109	E11.5 * Wnt1 down vs E11.5 * Ctrl
10546417	Trh	NM_009426	0.00664242	-1.20115	E11.5 * Wnt1 down vs E11.5 * Ctrl
10356475	Arl4c	NM_177305	0.00326105	-1.20117	E11.5 * Wnt1 down vs E11.5 * Ctrl
10480238	St8sia6	NM_145838	0.00632814	-1.20135	E11.5 * Wnt1 down vs E11.5 * Ctrl
10595848	Spsb4	NM_145134	0.00594449	-1.20163	E11.5 * Wnt1 down vs E11.5 * Ctrl
10512628	Olfr159	NM_019476	0.00177637	-1.20172	E11.5 * Wnt1 down vs E11.5 * Ctrl
10421877	Diap3	NM_019670	0.00276156	-1.20172	E11.5 * Wnt1 down vs E11.5 * Ctrl
10420889	1110020C17Rik	ENSMUST00000(	0.00633847	-1.20254	E11.5 * Wnt1 down vs E11.5 * Ctrl
10538438	C330043M08Rik	NM_001142781	0.00219176	-1.20257	E11.5 * Wnt1 down vs E11.5 * Ctrl
10507870		---	0.00613436	-1.2026	E11.5 * Wnt1 down vs E11.5 * Ctrl
10454254	Dtna	NM_207650	0.00719294	-1.20317	E11.5 * Wnt1 down vs E11.5 * Ctrl
10371356	Appl2	NM_145220	0.00599523	-1.20327	E11.5 * Wnt1 down vs E11.5 * Ctrl
10488608	Trib3	NM_175093	0.00282678	-1.20331	E11.5 * Wnt1 down vs E11.5 * Ctrl
10376434	Butr1	NM_138678	0.00447846	-1.20347	E11.5 * Wnt1 down vs E11.5 * Ctrl

10464171	Tdrd1	NM_001002238	0.0013733	-1.20357	E11.5 * Wnt1 down vs E11.5 * Ctrl
10379060	Proca1	NM_001045516	0.00837848	-1.20373	E11.5 * Wnt1 down vs E11.5 * Ctrl
10368473		---	0.00110724	-1.20443	E11.5 * Wnt1 down vs E11.5 * Ctrl
10513381	Rod1	NM_144904	6.23335e-005	-1.2045	E11.5 * Wnt1 down vs E11.5 * Ctrl
10570291	F10	NM_007972	0.00662933	-1.20453	E11.5 * Wnt1 down vs E11.5 * Ctrl
10602009	Rnf128	NM_023270	0.00824187	-1.20457	E11.5 * Wnt1 down vs E11.5 * Ctrl
10422781	4921505C17Rik	NM_030168	0.00347512	-1.20496	E11.5 * Wnt1 down vs E11.5 * Ctrl
10529935		---	0.00432085	-1.20506	E11.5 * Wnt1 down vs E11.5 * Ctrl
10595856	Slc25a36	NM_138756	0.0052557	-1.20526	E11.5 * Wnt1 down vs E11.5 * Ctrl
10513320	Ptgr1	NM_025968	0.00148568	-1.20549	E11.5 * Wnt1 down vs E11.5 * Ctrl
10562729		---	0.0027041	-1.20666	E11.5 * Wnt1 down vs E11.5 * Ctrl
10489241	Gm826	NM_001033411	0.00789388	-1.20755	E11.5 * Wnt1 down vs E11.5 * Ctrl
10454653		---	0.00300005	-1.20761	E11.5 * Wnt1 down vs E11.5 * Ctrl
10377953	Kif1c	NM_153103	0.0015366	-1.20862	E11.5 * Wnt1 down vs E11.5 * Ctrl
10443946	1700029I08Rik	NM_183282	0.00727437	-1.2087	E11.5 * Wnt1 down vs E11.5 * Ctrl
10344772	6030422M02Rik	NM_177722	0.00408959	-1.20925	E11.5 * Wnt1 down vs E11.5 * Ctrl
10597162	Klhl18	NM_177771	0.00144045	-1.20936	E11.5 * Wnt1 down vs E11.5 * Ctrl
10399383	Kcns3	NM_173417	0.00550894	-1.20937	E11.5 * Wnt1 down vs E11.5 * Ctrl
10509280	Hspg2	NM_008305	0.00303805	-1.20941	E11.5 * Wnt1 down vs E11.5 * Ctrl
10497613	ENSMUSG0000C	ENSMUST00000	0.00256152	-1.20957	E11.5 * Wnt1 down vs E11.5 * Ctrl
10436804	Mrap	NM_029844	0.00048874	-1.20981	E11.5 * Wnt1 down vs E11.5 * Ctrl
10538150	Tmem176a	NM_025326	0.00414552	-1.21008	E11.5 * Wnt1 down vs E11.5 * Ctrl
10561247	Shkbp1	NM_138676	0.00568111	-1.21027	E11.5 * Wnt1 down vs E11.5 * Ctrl
10409905	Ctsr	NM_020284	0.00391719	-1.21037	E11.5 * Wnt1 down vs E11.5 * Ctrl
10588154	Stag1	NM_009282	0.00574313	-1.21146	E11.5 * Wnt1 down vs E11.5 * Ctrl
10561525	ENSMUSG0000C	ENSMUST00000	0.00464773	-1.21154	E11.5 * Wnt1 down vs E11.5 * Ctrl
10587383	Cd109	NM_153098	0.00175281	-1.21157	E11.5 * Wnt1 down vs E11.5 * Ctrl
10552632	Shank1	ENSMUST00000	0.000647525	-1.21163	E11.5 * Wnt1 down vs E11.5 * Ctrl
10428103	Stk3	NM_019635	0.00345347	-1.21194	E11.5 * Wnt1 down vs E11.5 * Ctrl
10571371	Tusc3	NM_030254	0.000269553	-1.21254	E11.5 * Wnt1 down vs E11.5 * Ctrl
10356423	Usp40	NM_001033291	0.00669629	-1.21277	E11.5 * Wnt1 down vs E11.5 * Ctrl
10412394	Nnt	NR_003544	0.00627595	-1.21342	E11.5 * Wnt1 down vs E11.5 * Ctrl
10348424	Sh3bp4	NM_133816	0.00362267	-1.21344	E11.5 * Wnt1 down vs E11.5 * Ctrl
10484575	Olfr1048	NM_147014	0.0010152	-1.21365	E11.5 * Wnt1 down vs E11.5 * Ctrl
10582337	Fam38a	ENSMUST00000	0.00451143	-1.21374	E11.5 * Wnt1 down vs E11.5 * Ctrl
10542857	Far2	NM_178797	0.00434838	-1.21395	E11.5 * Wnt1 down vs E11.5 * Ctrl
10357043	Bcl2	NM_009741	0.00112169	-1.21461	E11.5 * Wnt1 down vs E11.5 * Ctrl
10394846	LOC100043371	ENSMUST00000	0.00608199	-1.21471	E11.5 * Wnt1 down vs E11.5 * Ctrl
10529052	Slc5a6	NM_177870	0.00215902	-1.21501	E11.5 * Wnt1 down vs E11.5 * Ctrl
10442139	EG665525	XM_977557	0.0028918	-1.21519	E11.5 * Wnt1 down vs E11.5 * Ctrl
10519060	Tnfrsf14	NM_178931	0.00409315	-1.21571	E11.5 * Wnt1 down vs E11.5 * Ctrl
10491056	Tbl1xr1	NM_030732	0.00548738	-1.21623	E11.5 * Wnt1 down vs E11.5 * Ctrl
10437970	2310008H04Rik	NM_146068	0.000326764	-1.21696	E11.5 * Wnt1 down vs E11.5 * Ctrl
10495562	Lrrc39	NM_175413	0.00192158	-1.21701	E11.5 * Wnt1 down vs E11.5 * Ctrl
10570786	Thsd1	NM_019576	0.00351819	-1.2175	E11.5 * Wnt1 down vs E11.5 * Ctrl
10500847	Magi3	NM_133853	0.00648502	-1.2178	E11.5 * Wnt1 down vs E11.5 * Ctrl
10451061	Runx2	NM_001146038	0.00265169	-1.21801	E11.5 * Wnt1 down vs E11.5 * Ctrl
10553042	Rasip1	NM_028544	0.00674666	-1.21843	E11.5 * Wnt1 down vs E11.5 * Ctrl
10389207	Ccl5	NM_013653	0.00170939	-1.2186	E11.5 * Wnt1 down vs E11.5 * Ctrl
10508074	Csf3r	NM_007782	0.00388058	-1.21868	E11.5 * Wnt1 down vs E11.5 * Ctrl
10510201	Rex2	NM_009051	0.0060737	-1.21884	E11.5 * Wnt1 down vs E11.5 * Ctrl
10453178	Map4k3	NM_001081357	0.00139033	-1.21895	E11.5 * Wnt1 down vs E11.5 * Ctrl
10392863	Grin2c	NM_010350	0.00418428	-1.21929	E11.5 * Wnt1 down vs E11.5 * Ctrl
10409376	Hk3	NM_001033245	0.00757456	-1.21943	E11.5 * Wnt1 down vs E11.5 * Ctrl
10565218	Il16	NM_010551	0.00666661	-1.21954	E11.5 * Wnt1 down vs E11.5 * Ctrl
10345877	ENSMUSG0000C	ENSMUST00000	0.00600573	-1.22	E11.5 * Wnt1 down vs E11.5 * Ctrl
10482868	Wdsub1	NM_028118	0.00343555	-1.22007	E11.5 * Wnt1 down vs E11.5 * Ctrl
10372385	Zdhhc17	NM_172554	0.00544595	-1.22014	E11.5 * Wnt1 down vs E11.5 * Ctrl
10459944	Nfatc1	NM_198429	0.00512814	-1.22072	E11.5 * Wnt1 down vs E11.5 * Ctrl
10450384	Msh5	NM_013600	0.00548451	-1.22075	E11.5 * Wnt1 down vs E11.5 * Ctrl
10536827	Ccdc136	BC006583	0.00123358	-1.22154	E11.5 * Wnt1 down vs E11.5 * Ctrl
10489372	0610008F07Rik	BC025862	0.00431743	-1.22187	E11.5 * Wnt1 down vs E11.5 * Ctrl
10555729	Olfr555	NM_147103	0.000101524	-1.22226	E11.5 * Wnt1 down vs E11.5 * Ctrl
10595664	Tmed3	NM_025360	0.00359509	-1.22273	E11.5 * Wnt1 down vs E11.5 * Ctrl
10532040	Zfp644	NM_026856	0.000423381	-1.22276	E11.5 * Wnt1 down vs E11.5 * Ctrl
10423080	C1qtnf3	NM_030888	0.00209937	-1.22315	E11.5 * Wnt1 down vs E11.5 * Ctrl

10394819	LOC677565	XR_034928	0.00721774	-1.22325	E11.5 * Wnt1 down vs E11.5 * Ctrl
10395103	Pxdn	NM_181395	0.00741927	-1.22334	E11.5 * Wnt1 down vs E11.5 * Ctrl
10446376	Man2a1	NM_008549	0.00320082	-1.22388	E11.5 * Wnt1 down vs E11.5 * Ctrl
10365716	1200009F10Rik	NM_026166	0.00477662	-1.22396	E11.5 * Wnt1 down vs E11.5 * Ctrl
10570614	Defb6	NM_054074	0.00141242	-1.22409	E11.5 * Wnt1 down vs E11.5 * Ctrl
10551531	Sycn	NM_026716	0.00883251	-1.22421	E11.5 * Wnt1 down vs E11.5 * Ctrl
10599435	Ocrl	NM_177215	0.00124578	-1.22453	E11.5 * Wnt1 down vs E11.5 * Ctrl
10599296	Rhox6	NM_008955	0.00696453	-1.2257	E11.5 * Wnt1 down vs E11.5 * Ctrl
10510241	OTTMUSG00000	NM_001014397	0.00315224	-1.22577	E11.5 * Wnt1 down vs E11.5 * Ctrl
10346843	Nrp2	NM_001077403	0.00405217	-1.22585	E11.5 * Wnt1 down vs E11.5 * Ctrl
10512499	Tpm2	NM_009416	0.00344729	-1.22621	E11.5 * Wnt1 down vs E11.5 * Ctrl
10452998	Sult6b1	ENSMUST00000	0.0075723	-1.22679	E11.5 * Wnt1 down vs E11.5 * Ctrl
10359086	Cep350	BC089561	0.00283462	-1.22771	E11.5 * Wnt1 down vs E11.5 * Ctrl
10537676	1700034O15Rik	BC048592	0.00435025	-1.22778	E11.5 * Wnt1 down vs E11.5 * Ctrl
10511984	1700003M02Rik	NM_027041	0.00276017	-1.22793	E11.5 * Wnt1 down vs E11.5 * Ctrl
10501676	Hiat1	NM_008246	0.00862704	-1.22827	E11.5 * Wnt1 down vs E11.5 * Ctrl
10576027	ENSMUSG00000	ENSMUST00000	0.000455243	-1.22834	E11.5 * Wnt1 down vs E11.5 * Ctrl
10435237	Zfp148	NM_011749	0.000533328	-1.22838	E11.5 * Wnt1 down vs E11.5 * Ctrl
10450374	D17H6S56E-5	L78788	0.00763964	-1.22881	E11.5 * Wnt1 down vs E11.5 * Ctrl
10410279		---	0.00286305	-1.22907	E11.5 * Wnt1 down vs E11.5 * Ctrl
10361834	Txlnb	NM_138628	0.00628297	-1.2293	E11.5 * Wnt1 down vs E11.5 * Ctrl
10372342	Nav3	NM_001081035	0.00603261	-1.22932	E11.5 * Wnt1 down vs E11.5 * Ctrl
10465783	Tmem179b	NM_026325	0.000981906	-1.22955	E11.5 * Wnt1 down vs E11.5 * Ctrl
10585318	Rdx	NM_001104617	0.00131057	-1.2296	E11.5 * Wnt1 down vs E11.5 * Ctrl
10439854	Bbx	NM_027444	0.00678076	-1.22963	E11.5 * Wnt1 down vs E11.5 * Ctrl
10507655		---	0.00173256	-1.22964	E11.5 * Wnt1 down vs E11.5 * Ctrl
10407173	Il6st	NM_010560	0.000721471	-1.2301	E11.5 * Wnt1 down vs E11.5 * Ctrl
10565591	OTTMUSG00000	ENSMUST00000	0.000416675	-1.2303	E11.5 * Wnt1 down vs E11.5 * Ctrl
10605421	Mtcp1	NM_001039373	0.000399497	-1.23042	E11.5 * Wnt1 down vs E11.5 * Ctrl
10594825	Aqp9	NM_022026	0.000344338	-1.23048	E11.5 * Wnt1 down vs E11.5 * Ctrl
10579744	Large	NM_010687	0.00205077	-1.23057	E11.5 * Wnt1 down vs E11.5 * Ctrl
10492006	Trpc4	NM_016984	0.00487818	-1.23066	E11.5 * Wnt1 down vs E11.5 * Ctrl
10472501	Lass6	NM_172856	0.000269544	-1.23088	E11.5 * Wnt1 down vs E11.5 * Ctrl
10349431	Acmsd	NM_001033041	0.00340265	-1.2311	E11.5 * Wnt1 down vs E11.5 * Ctrl
10563355	Sec1	NM_019934	0.00231148	-1.23126	E11.5 * Wnt1 down vs E11.5 * Ctrl
10577412	6820431F20Rik	BC025151	0.0001647	-1.23188	E11.5 * Wnt1 down vs E11.5 * Ctrl
10594251	Kif23	NM_024245	0.0020151	-1.23234	E11.5 * Wnt1 down vs E11.5 * Ctrl
10385870	Irf1	NM_008390	0.000657142	-1.23237	E11.5 * Wnt1 down vs E11.5 * Ctrl
10451372	Ptk7	NM_175168	0.000286434	-1.23254	E11.5 * Wnt1 down vs E11.5 * Ctrl
10345752	Il1r2	NM_010555	0.00717014	-1.23281	E11.5 * Wnt1 down vs E11.5 * Ctrl
10602925	Phka2	NM_172783	0.00721455	-1.23288	E11.5 * Wnt1 down vs E11.5 * Ctrl
10568638	Uros	NM_009479	0.000551966	-1.23325	E11.5 * Wnt1 down vs E11.5 * Ctrl
10513186	OTTMUSG00000	XM_884359	0.00804817	-1.23327	E11.5 * Wnt1 down vs E11.5 * Ctrl
10435832	Gm608	NM_001029889	0.000237731	-1.23364	E11.5 * Wnt1 down vs E11.5 * Ctrl
10530151	Tlr6	NM_011604	0.000978423	-1.23364	E11.5 * Wnt1 down vs E11.5 * Ctrl
10606530		---	0.00144658	-1.23364	E11.5 * Wnt1 down vs E11.5 * Ctrl
10593430	Sik2	NM_178710	0.00236348	-1.23386	E11.5 * Wnt1 down vs E11.5 * Ctrl
10514666		---	0.00755054	-1.23415	E11.5 * Wnt1 down vs E11.5 * Ctrl
10555873	Olfr652	NM_147048	0.00481661	-1.23425	E11.5 * Wnt1 down vs E11.5 * Ctrl
10493292	2810403A07Rik	AK173057	0.000261646	-1.23438	E11.5 * Wnt1 down vs E11.5 * Ctrl
10433101	Gpr84	NM_030720	0.00454207	-1.23463	E11.5 * Wnt1 down vs E11.5 * Ctrl
10591112	Fat3	NM_001080814	0.00166147	-1.23467	E11.5 * Wnt1 down vs E11.5 * Ctrl
10578681		---	0.00450462	-1.23502	E11.5 * Wnt1 down vs E11.5 * Ctrl
10565840	Neu3	NM_016720	0.00226737	-1.23516	E11.5 * Wnt1 down vs E11.5 * Ctrl
10375232		---	0.00337395	-1.2353	E11.5 * Wnt1 down vs E11.5 * Ctrl
10381408	Ifi35	NM_027320	0.00467257	-1.23541	E11.5 * Wnt1 down vs E11.5 * Ctrl
10488465	Zfp345	NM_001034900	0.00303574	-1.23611	E11.5 * Wnt1 down vs E11.5 * Ctrl
10357480	Daf2	NM_007827	0.00487936	-1.23678	E11.5 * Wnt1 down vs E11.5 * Ctrl
10513162	Ptpn3	NM_011207	0.00813044	-1.23703	E11.5 * Wnt1 down vs E11.5 * Ctrl
10592629	Grik4	NM_175481	0.0029394	-1.23751	E11.5 * Wnt1 down vs E11.5 * Ctrl
10548146	Tead4	NM_011567	0.00157949	-1.23797	E11.5 * Wnt1 down vs E11.5 * Ctrl
10544779	Hoxa7	NM_010455	0.00132073	-1.23803	E11.5 * Wnt1 down vs E11.5 * Ctrl
10528191	Speer4d	NM_025759	0.00062083	-1.2383	E11.5 * Wnt1 down vs E11.5 * Ctrl
10456383	Impa2	NM_053261	0.000989053	-1.23836	E11.5 * Wnt1 down vs E11.5 * Ctrl
10473356	Ube2l6	NM_019949	0.00384197	-1.2388	E11.5 * Wnt1 down vs E11.5 * Ctrl
10542104	ENSMUSG00000	ENSMUST00000	0.00420019	-1.23891	E11.5 * Wnt1 down vs E11.5 * Ctrl

10532711	Cmklr1	NM_008153	0.00886315	-1.23927	E11.5 * Wnt1 down vs E11.5 * Ctrl
10356082	Plscr1	NM_011636	0.00578465	-1.23945	E11.5 * Wnt1 down vs E11.5 * Ctrl
10500434	Bcl9	NM_029933	0.00173969	-1.23945	E11.5 * Wnt1 down vs E11.5 * Ctrl
10383511	Tex19.1	AF285590	0.00172099	-1.23973	E11.5 * Wnt1 down vs E11.5 * Ctrl
10573419	Lyl1	NM_008535	0.00153075	-1.24007	E11.5 * Wnt1 down vs E11.5 * Ctrl
10593913	1700017B05Rik	NM_028820	0.00579811	-1.24036	E11.5 * Wnt1 down vs E11.5 * Ctrl
10545096	2410003J06Rik	NM_028025	0.00236199	-1.24036	E11.5 * Wnt1 down vs E11.5 * Ctrl
10491522	A330050B17Rik	AK138522	0.00322854	-1.24041	E11.5 * Wnt1 down vs E11.5 * Ctrl
10600786	Klhl15	NM_001039060	0.00276088	-1.24082	E11.5 * Wnt1 down vs E11.5 * Ctrl
10501051	Cept1	NM_133869	0.00466618	-1.24126	E11.5 * Wnt1 down vs E11.5 * Ctrl
10463224	Marveld1	BC054384	0.00297929	-1.24145	E11.5 * Wnt1 down vs E11.5 * Ctrl
10412267	Itga2	NM_008396	0.00871117	-1.24152	E11.5 * Wnt1 down vs E11.5 * Ctrl
10510399	Masp2	NM_001003893	0.00762569	-1.24178	E11.5 * Wnt1 down vs E11.5 * Ctrl
10377537	Chd3	NM_146019	8.26696e-006	-1.24184	E11.5 * Wnt1 down vs E11.5 * Ctrl
10380398	Chad	NM_007689	0.000675103	-1.24191	E11.5 * Wnt1 down vs E11.5 * Ctrl
10474045	Chst1	NM_023850	0.00729525	-1.24243	E11.5 * Wnt1 down vs E11.5 * Ctrl
10601412	Lpar4	NM_175271	0.000749545	-1.2426	E11.5 * Wnt1 down vs E11.5 * Ctrl
10607143	Capn6	NM_007603	0.0015598	-1.24267	E11.5 * Wnt1 down vs E11.5 * Ctrl
10569313	6330512M04Rik	NM_177265	0.00536628	-1.24304	E11.5 * Wnt1 down vs E11.5 * Ctrl
10592719	Oaf	NM_178644	0.000285437	-1.2432	E11.5 * Wnt1 down vs E11.5 * Ctrl
10367036	Rdh1	NM_080436	0.00451187	-1.24331	E11.5 * Wnt1 down vs E11.5 * Ctrl
10389783	ENSMUSG00000	ENSMUST00000	0.00711794	-1.24337	E11.5 * Wnt1 down vs E11.5 * Ctrl
10516221	D130007C19Rik	AK051152	0.00423	-1.2439	E11.5 * Wnt1 down vs E11.5 * Ctrl
10397606	Tshr	NM_011648	0.00570134	-1.2444	E11.5 * Wnt1 down vs E11.5 * Ctrl
10359648	Scyl3	NM_028776	5.72803e-005	-1.24471	E11.5 * Wnt1 down vs E11.5 * Ctrl
10403229	Itgb8	NM_177290	0.00586944	-1.24477	E11.5 * Wnt1 down vs E11.5 * Ctrl
10473107		---	0.00543073	-1.24504	E11.5 * Wnt1 down vs E11.5 * Ctrl
10539873	Gata2	NM_008090	0.00216498	-1.24525	E11.5 * Wnt1 down vs E11.5 * Ctrl
10411595	Naip2	NM_010872	0.00114979	-1.24535	E11.5 * Wnt1 down vs E11.5 * Ctrl
10559177	B130016D09Rik	AK044960	0.00696544	-1.24552	E11.5 * Wnt1 down vs E11.5 * Ctrl
10576034	Irf8	NM_008320	0.000464076	-1.24602	E11.5 * Wnt1 down vs E11.5 * Ctrl
10604735	Rbmx	NM_011252	0.00666176	-1.24676	E11.5 * Wnt1 down vs E11.5 * Ctrl
10375886	D930048N14Rik	BC095931	0.00079704	-1.24717	E11.5 * Wnt1 down vs E11.5 * Ctrl
10518967	1190007F08Rik	ENSMUST00000	0.000286047	-1.24734	E11.5 * Wnt1 down vs E11.5 * Ctrl
10366739		---	0.00345815	-1.24749	E11.5 * Wnt1 down vs E11.5 * Ctrl
10600777	AU015836	BC141215	0.00290247	-1.2478	E11.5 * Wnt1 down vs E11.5 * Ctrl
10448878	Baiap3	BC158116	0.000412087	-1.24814	E11.5 * Wnt1 down vs E11.5 * Ctrl
10428753		---	0.00413844	-1.24822	E11.5 * Wnt1 down vs E11.5 * Ctrl
10515385	Urod	NM_009478	0.00107834	-1.24835	E11.5 * Wnt1 down vs E11.5 * Ctrl
10465990	Tmem216	NM_026798	0.00755672	-1.24846	E11.5 * Wnt1 down vs E11.5 * Ctrl
10415013		---	0.00316072	-1.24848	E11.5 * Wnt1 down vs E11.5 * Ctrl
10585992	Myo9a	BC096035	0.00583914	-1.2486	E11.5 * Wnt1 down vs E11.5 * Ctrl
10446986	Crim1	NM_015800	0.00601887	-1.24865	E11.5 * Wnt1 down vs E11.5 * Ctrl
10454103		---	0.000635859	-1.24895	E11.5 * Wnt1 down vs E11.5 * Ctrl
10605351		---	0.000655148	-1.24932	E11.5 * Wnt1 down vs E11.5 * Ctrl
10437928	0610037P05Rik	BC011300	0.000449933	-1.24974	E11.5 * Wnt1 down vs E11.5 * Ctrl
10399290	4930417G10Rik	NM_029107	0.00364087	-1.25022	E11.5 * Wnt1 down vs E11.5 * Ctrl
10382698	1110017F19Rik	ENSMUST00000	0.000997744	-1.25055	E11.5 * Wnt1 down vs E11.5 * Ctrl
10504132	Ccl19	NM_011888	0.00482285	-1.25059	E11.5 * Wnt1 down vs E11.5 * Ctrl
10349769		---	0.00389182	-1.25119	E11.5 * Wnt1 down vs E11.5 * Ctrl
10491629	4932438A13Rik	NM_172679	0.00386749	-1.25149	E11.5 * Wnt1 down vs E11.5 * Ctrl
10507773		---	0.00861179	-1.25158	E11.5 * Wnt1 down vs E11.5 * Ctrl
10346799	Icos	NM_017480	0.00220171	-1.25175	E11.5 * Wnt1 down vs E11.5 * Ctrl
10399202	Macc1	ENSMUST00000	0.0065928	-1.25191	E11.5 * Wnt1 down vs E11.5 * Ctrl
10394829		---	0.00204482	-1.25196	E11.5 * Wnt1 down vs E11.5 * Ctrl
10516765	Serinc2	NM_172702	0.00309528	-1.25234	E11.5 * Wnt1 down vs E11.5 * Ctrl
10481312	1700026L06Rik	NM_027283	0.00549974	-1.25277	E11.5 * Wnt1 down vs E11.5 * Ctrl
10355329	Bard1	NM_007525	0.0028855	-1.25298	E11.5 * Wnt1 down vs E11.5 * Ctrl
10576973	Col4a1	NM_009931	7.99377e-005	-1.25305	E11.5 * Wnt1 down vs E11.5 * Ctrl
10499612	Zbtb7b	NM_009565	0.00837649	-1.25308	E11.5 * Wnt1 down vs E11.5 * Ctrl
10606362		---	0.00434302	-1.25311	E11.5 * Wnt1 down vs E11.5 * Ctrl
10370210	Col6a1	NM_009933	0.00277863	-1.25313	E11.5 * Wnt1 down vs E11.5 * Ctrl
10591988	Adamts15	NM_001024139	0.00878847	-1.25343	E11.5 * Wnt1 down vs E11.5 * Ctrl
10372082	Nudt4	NM_027722	0.00079919	-1.25349	E11.5 * Wnt1 down vs E11.5 * Ctrl
10374621	Ahsa2	NM_172391	4.98623e-005	-1.2543	E11.5 * Wnt1 down vs E11.5 * Ctrl
10363868	Fam13c	NM_024244	0.00104083	-1.25461	E11.5 * Wnt1 down vs E11.5 * Ctrl

10404731	Tmem14c	NM_025387	0.00113894	-1.25503	E11.5 * Wnt1 down vs E11.5 * Ctrl
10378523	Smg6	NM_001002764	0.00432695	-1.25557	E11.5 * Wnt1 down vs E11.5 * Ctrl
10417704	Lrrc3b	NM_146052	0.00794505	-1.25576	E11.5 * Wnt1 down vs E11.5 * Ctrl
10533446	Tect1	NM_001039153	0.00333994	-1.25584	E11.5 * Wnt1 down vs E11.5 * Ctrl
10551696	Rasgrp4	NM_145149	0.000377545	-1.25679	E11.5 * Wnt1 down vs E11.5 * Ctrl
10548375	Clec7a	NM_020008	0.00349033	-1.25759	E11.5 * Wnt1 down vs E11.5 * Ctrl
10560139	B430211C08Rik	NM_001039146	0.000586583	-1.25788	E11.5 * Wnt1 down vs E11.5 * Ctrl
10554586	4833418N17Rik	AK080712	0.00734127	-1.258	E11.5 * Wnt1 down vs E11.5 * Ctrl
10426999	Acvrl1	NM_009612	0.00868421	-1.25818	E11.5 * Wnt1 down vs E11.5 * Ctrl
10555186	Wnt11	NM_009519	0.00117396	-1.25818	E11.5 * Wnt1 down vs E11.5 * Ctrl
10548701	Lrp6	NM_008514	0.00175742	-1.25838	E11.5 * Wnt1 down vs E11.5 * Ctrl
10375815		---	0.00230842	-1.25841	E11.5 * Wnt1 down vs E11.5 * Ctrl
10500103	Gabpb2	NM_029885	2.88041e-005	-1.25894	E11.5 * Wnt1 down vs E11.5 * Ctrl
10575534	St3gal2	NM_009179	0.000219491	-1.25926	E11.5 * Wnt1 down vs E11.5 * Ctrl
10407929	Pou6f2	NM_175006	0.00204465	-1.25953	E11.5 * Wnt1 down vs E11.5 * Ctrl
10543600	Kcp	NM_001029985	0.000598979	-1.25954	E11.5 * Wnt1 down vs E11.5 * Ctrl
10575160	Nfat5	NM_133957	0.000153388	-1.25979	E11.5 * Wnt1 down vs E11.5 * Ctrl
10551102	Prr19	NM_001081294	0.00860285	-1.25987	E11.5 * Wnt1 down vs E11.5 * Ctrl
10578504	1700029J07Rik	NM_001033148	0.00675638	-1.25998	E11.5 * Wnt1 down vs E11.5 * Ctrl
10399314	Gm1964	NM_001033488	0.00174093	-1.26	E11.5 * Wnt1 down vs E11.5 * Ctrl
10394812		---	0.000750872	-1.26039	E11.5 * Wnt1 down vs E11.5 * Ctrl
10494565	Fmo5	NM_010232	0.00345701	-1.26041	E11.5 * Wnt1 down vs E11.5 * Ctrl
10447065	Fam82a1	NM_201361	0.00091904	-1.26051	E11.5 * Wnt1 down vs E11.5 * Ctrl
10396485	Syne2	NM_001005510	6.32165e-005	-1.26062	E11.5 * Wnt1 down vs E11.5 * Ctrl
10347593	Speg	NM_007463	0.000967004	-1.26083	E11.5 * Wnt1 down vs E11.5 * Ctrl
10598178	Disp1	NM_026866	0.00135403	-1.26104	E11.5 * Wnt1 down vs E11.5 * Ctrl
10514193		---	0.00658533	-1.26124	E11.5 * Wnt1 down vs E11.5 * Ctrl
10432294	C430014K11Rik	ENSMUST00000	0.00141238	-1.26152	E11.5 * Wnt1 down vs E11.5 * Ctrl
10425138	Sh3bp1	NM_009164	0.00010861	-1.26168	E11.5 * Wnt1 down vs E11.5 * Ctrl
10491621	4932438A13Rik	NM_172679	0.00715599	-1.26205	E11.5 * Wnt1 down vs E11.5 * Ctrl
10603026	Ctps2	NM_018737	0.000164429	-1.2621	E11.5 * Wnt1 down vs E11.5 * Ctrl
10524844	Taok3	NM_001081308	0.00117874	-1.26221	E11.5 * Wnt1 down vs E11.5 * Ctrl
10578045	Nrg1	NM_178591	0.000583013	-1.26222	E11.5 * Wnt1 down vs E11.5 * Ctrl
10358299	EG214403	NM_001029977	0.00108305	-1.26256	E11.5 * Wnt1 down vs E11.5 * Ctrl
10571321	Ppp1r3b	NM_177741	0.00173348	-1.26257	E11.5 * Wnt1 down vs E11.5 * Ctrl
10565193	Hdgfrp3	NM_013886	0.000779131	-1.26284	E11.5 * Wnt1 down vs E11.5 * Ctrl
10434942	Dlg1	NM_007862	0.000497802	-1.26303	E11.5 * Wnt1 down vs E11.5 * Ctrl
10555425	Fam168a	BC079886	2.34786e-005	-1.2633	E11.5 * Wnt1 down vs E11.5 * Ctrl
10525158	Oas1b	NR_003507	0.00207263	-1.26334	E11.5 * Wnt1 down vs E11.5 * Ctrl
10354111	Aff3	NM_010678	0.00301045	-1.26358	E11.5 * Wnt1 down vs E11.5 * Ctrl
10536908	Tspan33	NM_146173	0.000612783	-1.26364	E11.5 * Wnt1 down vs E11.5 * Ctrl
10383210		---	0.000516875	-1.26423	E11.5 * Wnt1 down vs E11.5 * Ctrl
10382425	Gprc5c	NM_001110337	0.000822423	-1.26436	E11.5 * Wnt1 down vs E11.5 * Ctrl
10515819		---	0.000781306	-1.26494	E11.5 * Wnt1 down vs E11.5 * Ctrl
10360858	C130074G19Rik	NM_178692	0.004546	-1.26541	E11.5 * Wnt1 down vs E11.5 * Ctrl
10385234		---	0.00502776	-1.26541	E11.5 * Wnt1 down vs E11.5 * Ctrl
10511975	Slc35a1	NM_011895	0.00752416	-1.26562	E11.5 * Wnt1 down vs E11.5 * Ctrl
10484630	Olfr1090	NM_146847	0.00355319	-1.2658	E11.5 * Wnt1 down vs E11.5 * Ctrl
10421029	Cdca2	NM_175384	0.000160619	-1.26583	E11.5 * Wnt1 down vs E11.5 * Ctrl
10447224	Dync2li1	NM_172256	0.0042635	-1.26589	E11.5 * Wnt1 down vs E11.5 * Ctrl
10560655	Bcam	NM_020486	0.00404143	-1.26606	E11.5 * Wnt1 down vs E11.5 * Ctrl
10353947	Tmem131	NM_018872	2.99333e-006	-1.26638	E11.5 * Wnt1 down vs E11.5 * Ctrl
10537410	Tbxas1	NM_011539	0.00138365	-1.2665	E11.5 * Wnt1 down vs E11.5 * Ctrl
10431894	Slc38a2	NM_175121	0.000424634	-1.26664	E11.5 * Wnt1 down vs E11.5 * Ctrl
10605651	4930415L06Rik	ENSMUST00000	0.000627447	-1.26669	E11.5 * Wnt1 down vs E11.5 * Ctrl
10378240	P2rx1	NM_008771	0.000376764	-1.26674	E11.5 * Wnt1 down vs E11.5 * Ctrl
10493789	S100a13	NM_009113	0.0002964	-1.26677	E11.5 * Wnt1 down vs E11.5 * Ctrl
10400708		---	0.00489251	-1.26733	E11.5 * Wnt1 down vs E11.5 * Ctrl
10596912	Klhdc8b	NM_030075	0.00498065	-1.26735	E11.5 * Wnt1 down vs E11.5 * Ctrl
10590801	Birc3	NM_007464	0.00236778	-1.26739	E11.5 * Wnt1 down vs E11.5 * Ctrl
10487321	2010106G01Rik	NM_023220	0.000842167	-1.26789	E11.5 * Wnt1 down vs E11.5 * Ctrl
10546024	Prokr1	NM_021381	0.00326241	-1.26796	E11.5 * Wnt1 down vs E11.5 * Ctrl
10414548	Rnase6	NM_030098	0.00372173	-1.26806	E11.5 * Wnt1 down vs E11.5 * Ctrl
10390072	OTTMUSG00000	NM_001007584	0.00800979	-1.2682	E11.5 * Wnt1 down vs E11.5 * Ctrl
10362245	Epb4.1l2	NM_013511	8.4433e-006	-1.26829	E11.5 * Wnt1 down vs E11.5 * Ctrl
10478678	Cd40	NM_170701	0.000727172	-1.26838	E11.5 * Wnt1 down vs E11.5 * Ctrl

10511755	Wwp1	NM_177327	0.00415301	-1.26838	E11.5 * Wnt1 down vs E11.5 * Ctrl
10606436	Nsbp1	NM_016710	0.00869119	-1.26843	E11.5 * Wnt1 down vs E11.5 * Ctrl
10409986	4933434E20Rik	NM_025762	0.00131625	-1.2686	E11.5 * Wnt1 down vs E11.5 * Ctrl
10474048	Syt13	NM_030725	4.78772e-005	-1.26893	E11.5 * Wnt1 down vs E11.5 * Ctrl
10575213	Zfhx3	NM_007496	0.00549997	-1.26899	E11.5 * Wnt1 down vs E11.5 * Ctrl
10349051	Tnfrsf11a	NM_009399	0.00632974	-1.26922	E11.5 * Wnt1 down vs E11.5 * Ctrl
10405216	Syk	NM_011518	0.0025264	-1.26928	E11.5 * Wnt1 down vs E11.5 * Ctrl
10438425	Olfr167	NM_146935	0.000156401	-1.26931	E11.5 * Wnt1 down vs E11.5 * Ctrl
10532248	Rnf212	ENSMUST00000(	0.00071587	-1.26933	E11.5 * Wnt1 down vs E11.5 * Ctrl
10588007	Tfdp2	NM_178667	3.09016e-006	-1.26945	E11.5 * Wnt1 down vs E11.5 * Ctrl
10357932	Ppp1r12b	NM_001081307	3.68073e-005	-1.26951	E11.5 * Wnt1 down vs E11.5 * Ctrl
10352777	Slc30a1	NM_009579	0.0020104	-1.27031	E11.5 * Wnt1 down vs E11.5 * Ctrl
10457225	Map3k8	NM_007746	0.00668934	-1.27052	E11.5 * Wnt1 down vs E11.5 * Ctrl
10556426	Parva	NM_020606	0.00676216	-1.2706	E11.5 * Wnt1 down vs E11.5 * Ctrl
10481857	Pbx3	NM_016768	0.000503388	-1.27111	E11.5 * Wnt1 down vs E11.5 * Ctrl
10492091	Smad9	NM_019483	0.00494317	-1.2718	E11.5 * Wnt1 down vs E11.5 * Ctrl
10557229	LOC100134980	BC016578	0.00206731	-1.27185	E11.5 * Wnt1 down vs E11.5 * Ctrl
10428453	Csmd3	NM_001081391	0.000588505	-1.27196	E11.5 * Wnt1 down vs E11.5 * Ctrl
10462130	Fam122a	NM_026520	0.00548514	-1.27201	E11.5 * Wnt1 down vs E11.5 * Ctrl
10415806	Fam124a	ENSMUST00000(	0.00376219	-1.27203	E11.5 * Wnt1 down vs E11.5 * Ctrl
10344707	Pcmtd1	NM_183028	0.000447924	-1.2724	E11.5 * Wnt1 down vs E11.5 * Ctrl
10467175	A830019P07Rik	ENSMUST00000(	0.00587831	-1.27244	E11.5 * Wnt1 down vs E11.5 * Ctrl
10493816	S100a5	NM_011312	0.000409128	-1.27286	E11.5 * Wnt1 down vs E11.5 * Ctrl
10373073	D10Ertd610e	NM_028027	0.000968655	-1.27289	E11.5 * Wnt1 down vs E11.5 * Ctrl
10443007	EG240055	NM_001081656	0.00279524	-1.27318	E11.5 * Wnt1 down vs E11.5 * Ctrl
10502469	Pdlim5	NM_019808	0.00177567	-1.27321	E11.5 * Wnt1 down vs E11.5 * Ctrl
10402063	Foxn3	BC029185	0.000197353	-1.27355	E11.5 * Wnt1 down vs E11.5 * Ctrl
10603440	Was	NM_009515	0.00199827	-1.27363	E11.5 * Wnt1 down vs E11.5 * Ctrl
10435043	Tm4sf19	BC147727	0.00710366	-1.27379	E11.5 * Wnt1 down vs E11.5 * Ctrl
10445071	Zfp57	NM_001013745	0.000418394	-1.27408	E11.5 * Wnt1 down vs E11.5 * Ctrl
10367436	Cd63	NM_001042580	0.00133217	-1.27439	E11.5 * Wnt1 down vs E11.5 * Ctrl
10421581	Lrch1	NM_001033439	0.00019307	-1.27478	E11.5 * Wnt1 down vs E11.5 * Ctrl
10350102	Ptpn7	NM_177081	0.00227416	-1.27511	E11.5 * Wnt1 down vs E11.5 * Ctrl
10392683	ENSMUSG0000C	ENSMUST00000	0.00405625	-1.27528	E11.5 * Wnt1 down vs E11.5 * Ctrl
10480891	Ubac1	NM_133835	0.00226036	-1.27541	E11.5 * Wnt1 down vs E11.5 * Ctrl
10506397	Mier1	NM_027696	0.0035441	-1.27548	E11.5 * Wnt1 down vs E11.5 * Ctrl
10425808	Tspo	NM_009775	0.00427977	-1.27679	E11.5 * Wnt1 down vs E11.5 * Ctrl
10411609		---	0.00533854	-1.27691	E11.5 * Wnt1 down vs E11.5 * Ctrl
10528548	Kcnh2	NM_013569	0.000751654	-1.27702	E11.5 * Wnt1 down vs E11.5 * Ctrl
10454235	Asxl3	ENSMUST00000	0.00122616	-1.27706	E11.5 * Wnt1 down vs E11.5 * Ctrl
10379721	Ccl4	NM_013652	0.000157441	-1.27769	E11.5 * Wnt1 down vs E11.5 * Ctrl
10577395	6820431F20Rik	BC058969	0.000531531	-1.27846	E11.5 * Wnt1 down vs E11.5 * Ctrl
10531370	Naaa	NM_025972	0.00286972	-1.27891	E11.5 * Wnt1 down vs E11.5 * Ctrl
10394833	2410018L13Rik	BC063063	0.00693156	-1.27907	E11.5 * Wnt1 down vs E11.5 * Ctrl
10502071	5730508B09Rik	NM_027482	0.00584197	-1.27952	E11.5 * Wnt1 down vs E11.5 * Ctrl
10606009	Gm614	BC147523	0.00815799	-1.27982	E11.5 * Wnt1 down vs E11.5 * Ctrl
10485070	Mdk	NM_010784	0.00422095	-1.27991	E11.5 * Wnt1 down vs E11.5 * Ctrl
10472042	OTTMUSG00000	AF390105	0.00371754	-1.27999	E11.5 * Wnt1 down vs E11.5 * Ctrl
10544436	Olfr458	NM_146444	0.00624987	-1.28004	E11.5 * Wnt1 down vs E11.5 * Ctrl
10469828	Psd4	NM_177611	0.00620894	-1.28008	E11.5 * Wnt1 down vs E11.5 * Ctrl
10531348	Ppef2	NM_011148	1.24103e-005	-1.28014	E11.5 * Wnt1 down vs E11.5 * Ctrl
10558645	Olfr533	NM_001011815	0.00278469	-1.2802	E11.5 * Wnt1 down vs E11.5 * Ctrl
10376144	Fnip1	NM_173753	0.00172515	-1.28087	E11.5 * Wnt1 down vs E11.5 * Ctrl
10405731	4933434E20Rik	NM_025762	0.000533324	-1.28099	E11.5 * Wnt1 down vs E11.5 * Ctrl
10419167	Ptgdr	NM_008962	0.00754992	-1.28114	E11.5 * Wnt1 down vs E11.5 * Ctrl
10363379	Cbara1	NM_144822	0.00193027	-1.28124	E11.5 * Wnt1 down vs E11.5 * Ctrl
10603247		---	0.00629053	-1.28141	E11.5 * Wnt1 down vs E11.5 * Ctrl
10433480	Rpl39l	NM_026594	0.000282586	-1.28143	E11.5 * Wnt1 down vs E11.5 * Ctrl
10363007	Ascc3	NM_001146089	2.26627e-006	-1.2821	E11.5 * Wnt1 down vs E11.5 * Ctrl
10503617	F730047E07Rik	NM_199467	0.00251236	-1.28212	E11.5 * Wnt1 down vs E11.5 * Ctrl
10510230	OTTMUSG00000	NM_001039209	0.00231006	-1.28221	E11.5 * Wnt1 down vs E11.5 * Ctrl
10586306	Igdcc4	NM_020043	0.00201629	-1.28255	E11.5 * Wnt1 down vs E11.5 * Ctrl
10475866	Bcl2l11	NM_207680	0.00167019	-1.28271	E11.5 * Wnt1 down vs E11.5 * Ctrl
10529801	Fbxl5	NM_178729	0.00252193	-1.28274	E11.5 * Wnt1 down vs E11.5 * Ctrl
10456492	D18Ertd653e	NM_172631	0.00851472	-1.28321	E11.5 * Wnt1 down vs E11.5 * Ctrl
10378216	Atp2a3	NM_016745	0.000453388	-1.28336	E11.5 * Wnt1 down vs E11.5 * Ctrl

10361234	Hsd11b1	NM_008288	0.00185398	-1.28341	E11.5 * Wnt1 down vs E11.5 * Ctrl
10440647	Cldn8	NM_018778	0.00529012	-1.2839	E11.5 * Wnt1 down vs E11.5 * Ctrl
10540408	Itpr1	NM_010585	0.000752138	-1.28408	E11.5 * Wnt1 down vs E11.5 * Ctrl
10432439	Fmnl3	NM_011711	0.00441804	-1.28411	E11.5 * Wnt1 down vs E11.5 * Ctrl
10408490	Exoc2	NM_025588	9.24722e-006	-1.28429	E11.5 * Wnt1 down vs E11.5 * Ctrl
10597945	Tmem158	NM_001002267	0.00201415	-1.2845	E11.5 * Wnt1 down vs E11.5 * Ctrl
10559238	Igf2as	NR_002855	0.00115634	-1.28482	E11.5 * Wnt1 down vs E11.5 * Ctrl
10346116		---	0.00719801	-1.28494	E11.5 * Wnt1 down vs E11.5 * Ctrl
10461408	Rab3il1	NM_144538	0.0011063	-1.28495	E11.5 * Wnt1 down vs E11.5 * Ctrl
10496656	Col24a1	NM_027770	0.00192489	-1.28513	E11.5 * Wnt1 down vs E11.5 * Ctrl
10548367	Clec1a	NM_175526	0.00181492	-1.28523	E11.5 * Wnt1 down vs E11.5 * Ctrl
10385466	Sgcd	NM_011891	0.00737797	-1.28523	E11.5 * Wnt1 down vs E11.5 * Ctrl
10552208	Nudt19	NM_033080	0.00267968	-1.28527	E11.5 * Wnt1 down vs E11.5 * Ctrl
10548905	Eps8	NM_007945	0.000687709	-1.28541	E11.5 * Wnt1 down vs E11.5 * Ctrl
10471844	Nek6	NM_021606	0.00743942	-1.28579	E11.5 * Wnt1 down vs E11.5 * Ctrl
10512384	BC049635	NM_177785	0.000608026	-1.2859	E11.5 * Wnt1 down vs E11.5 * Ctrl
10552351		---	0.00820556	-1.28615	E11.5 * Wnt1 down vs E11.5 * Ctrl
10511789	Nkain3	NM_172987	0.00403848	-1.28689	E11.5 * Wnt1 down vs E11.5 * Ctrl
10395287	Atxn7l1	NM_028139	8.90833e-005	-1.28705	E11.5 * Wnt1 down vs E11.5 * Ctrl
10590073	Vill	NM_011700	0.00286025	-1.28707	E11.5 * Wnt1 down vs E11.5 * Ctrl
10518335	OTTMUSG00000	NM_001083918	0.00152806	-1.28735	E11.5 * Wnt1 down vs E11.5 * Ctrl
10490491	Gata5	NM_008093	0.000219286	-1.28754	E11.5 * Wnt1 down vs E11.5 * Ctrl
10398224	Cyp46a1	NM_010010	0.000774202	-1.28757	E11.5 * Wnt1 down vs E11.5 * Ctrl
10459225		---	0.00668633	-1.28779	E11.5 * Wnt1 down vs E11.5 * Ctrl
10568714	Mki67	NM_001081117	0.000124744	-1.2879	E11.5 * Wnt1 down vs E11.5 * Ctrl
10433639	Mkl2	NM_153588	2.49292e-005	-1.28794	E11.5 * Wnt1 down vs E11.5 * Ctrl
10580191	Nfix	NM_001081981	0.0017201	-1.28851	E11.5 * Wnt1 down vs E11.5 * Ctrl
10414711		---	0.00608801	-1.29012	E11.5 * Wnt1 down vs E11.5 * Ctrl
10554945	Prcp	NM_028243	0.0048358	-1.29015	E11.5 * Wnt1 down vs E11.5 * Ctrl
10433797	Prkdc	NM_011159	0.00044219	-1.29031	E11.5 * Wnt1 down vs E11.5 * Ctrl
10483246		---	0.0063469	-1.29032	E11.5 * Wnt1 down vs E11.5 * Ctrl
10530827	Spink2	NM_183284	0.00241622	-1.29039	E11.5 * Wnt1 down vs E11.5 * Ctrl
10385774	Olfr1371	NM_207253	0.0065562	-1.29076	E11.5 * Wnt1 down vs E11.5 * Ctrl
10578025		---	0.00285665	-1.2911	E11.5 * Wnt1 down vs E11.5 * Ctrl
10472449	4933409G03Rik	NM_177651	0.000693735	-1.29168	E11.5 * Wnt1 down vs E11.5 * Ctrl
10367024	Tac2	NM_009312	0.00281326	-1.2918	E11.5 * Wnt1 down vs E11.5 * Ctrl
10461160		---	0.00137511	-1.2928	E11.5 * Wnt1 down vs E11.5 * Ctrl
10344725	Adhfe1	NM_175236	0.000303008	-1.29292	E11.5 * Wnt1 down vs E11.5 * Ctrl
10412830	Ngly1	NM_021504	0.0014878	-1.29308	E11.5 * Wnt1 down vs E11.5 * Ctrl
10427095	Tenc1	NM_153533	0.00163156	-1.29398	E11.5 * Wnt1 down vs E11.5 * Ctrl
10550509	Pglyrp1	NM_009402	0.00370851	-1.29424	E11.5 * Wnt1 down vs E11.5 * Ctrl
10403748		---	0.000435611	-1.29444	E11.5 * Wnt1 down vs E11.5 * Ctrl
10355514	Tns1	NM_027884	0.00316253	-1.29448	E11.5 * Wnt1 down vs E11.5 * Ctrl
10453629	9430020K01Rik	NM_001081963	0.00247161	-1.29457	E11.5 * Wnt1 down vs E11.5 * Ctrl
10468253	Nt5c2	NM_029810	0.00549884	-1.29464	E11.5 * Wnt1 down vs E11.5 * Ctrl
10455098	Pcdhb14	NM_053139	0.00114337	-1.29468	E11.5 * Wnt1 down vs E11.5 * Ctrl
10574259	Gpr56	NM_018882	0.000818831	-1.29471	E11.5 * Wnt1 down vs E11.5 * Ctrl
10552140	Cebpa	NM_007678	0.000905628	-1.29478	E11.5 * Wnt1 down vs E11.5 * Ctrl
10385893	Slc22a4	NM_019687	0.00172089	-1.29486	E11.5 * Wnt1 down vs E11.5 * Ctrl
10602592	Hsd17b10	NM_016763	0.000310167	-1.29556	E11.5 * Wnt1 down vs E11.5 * Ctrl
10384622	Ehbp1	NM_153078	0.00213728	-1.29568	E11.5 * Wnt1 down vs E11.5 * Ctrl
10394555	Rdh14	NM_023697	0.000312955	-1.29578	E11.5 * Wnt1 down vs E11.5 * Ctrl
10352439	Susd4	NM_144796	0.00205815	-1.29616	E11.5 * Wnt1 down vs E11.5 * Ctrl
10532305	4930522L14Rik	BC065392	0.00504707	-1.2964	E11.5 * Wnt1 down vs E11.5 * Ctrl
10367475		---	0.000682698	-1.29642	E11.5 * Wnt1 down vs E11.5 * Ctrl
10412123	Ncf2	NM_010877	0.00026577	-1.29646	E11.5 * Wnt1 down vs E11.5 * Ctrl
10451093	Spats1	NM_027649	0.00444222	-1.29698	E11.5 * Wnt1 down vs E11.5 * Ctrl
10556413	Micalcl	AB359922	0.00238311	-1.29711	E11.5 * Wnt1 down vs E11.5 * Ctrl
10405785	0610007P08Rik	NM_023507	0.00474654	-1.29718	E11.5 * Wnt1 down vs E11.5 * Ctrl
10595840	Acpl2	NM_153420	0.0061015	-1.29753	E11.5 * Wnt1 down vs E11.5 * Ctrl
10585535	Isl2	NM_027397	0.00150666	-1.29771	E11.5 * Wnt1 down vs E11.5 * Ctrl
10417579	4930452B06Rik	BC064468	0.00817459	-1.29804	E11.5 * Wnt1 down vs E11.5 * Ctrl
10416709	Tdrd3	NM_172605	8.06423e-005	-1.29824	E11.5 * Wnt1 down vs E11.5 * Ctrl
10476237	Hspa12b	NM_028306	0.00390331	-1.29885	E11.5 * Wnt1 down vs E11.5 * Ctrl
10519504	Sri	NM_025618	0.00045181	-1.2991	E11.5 * Wnt1 down vs E11.5 * Ctrl
10504127	Ccl21a	NM_011124	0.00543837	-1.29913	E11.5 * Wnt1 down vs E11.5 * Ctrl

10504154	Ccl21a	NM_011124	0.00543837	-1.29913	E11.5 * Wnt1 down vs E11.5 * Ctrl
10504183	Ccl21a	NM_011124	0.00543837	-1.29913	E11.5 * Wnt1 down vs E11.5 * Ctrl
10512377	Ccl21a	NM_011124	0.00543837	-1.29913	E11.5 * Wnt1 down vs E11.5 * Ctrl
10356170		---	0.00720251	-1.29942	E11.5 * Wnt1 down vs E11.5 * Ctrl
10512334	100038993	NM_001100596	0.000136882	-1.29952	E11.5 * Wnt1 down vs E11.5 * Ctrl
10512356	100038993	NM_001100596	0.000136882	-1.29952	E11.5 * Wnt1 down vs E11.5 * Ctrl
10601854	Wbp5	NM_011712	2.37809e-005	-1.29976	E11.5 * Wnt1 down vs E11.5 * Ctrl
10432640	Bin2	ENSMUST00000	0.00328451	-1.2999	E11.5 * Wnt1 down vs E11.5 * Ctrl
10483439	Lrp2	NM_001081088	0.000178219	-1.29996	E11.5 * Wnt1 down vs E11.5 * Ctrl
10421924	Pcdh9	NM_001081377	0.00727971	-1.30008	E11.5 * Wnt1 down vs E11.5 * Ctrl
10487645	Cpxm1	NM_019696	0.00470955	-1.30064	E11.5 * Wnt1 down vs E11.5 * Ctrl
10394938		---	0.00561484	-1.30079	E11.5 * Wnt1 down vs E11.5 * Ctrl
10399657		---	0.00561484	-1.30079	E11.5 * Wnt1 down vs E11.5 * Ctrl
10500204	Ecm1	NM_007899	0.00310224	-1.30121	E11.5 * Wnt1 down vs E11.5 * Ctrl
10415248	Pck2	NM_028994	0.000137083	-1.30132	E11.5 * Wnt1 down vs E11.5 * Ctrl
10384782	Vrk2	NM_027260	0.00102755	-1.30141	E11.5 * Wnt1 down vs E11.5 * Ctrl
10485117	Creb3l1	NM_011957	0.00130933	-1.30156	E11.5 * Wnt1 down vs E11.5 * Ctrl
10459510		---	0.00152122	-1.30199	E11.5 * Wnt1 down vs E11.5 * Ctrl
10362803	Cd164	NM_016898	0.00010007	-1.30247	E11.5 * Wnt1 down vs E11.5 * Ctrl
10567995	Nupr1	NM_019738	0.00138658	-1.30288	E11.5 * Wnt1 down vs E11.5 * Ctrl
10362432	Trdn	NM_029726	0.00528483	-1.30313	E11.5 * Wnt1 down vs E11.5 * Ctrl
10564343	Tjp1	NM_009386	5.79567e-007	-1.30314	E11.5 * Wnt1 down vs E11.5 * Ctrl
10477250	Hck	NM_010407	0.000252275	-1.30357	E11.5 * Wnt1 down vs E11.5 * Ctrl
10413839	Ncoa4	NM_001033988	2.33924e-005	-1.30369	E11.5 * Wnt1 down vs E11.5 * Ctrl
10598603			0.00107628	-1.30401	E11.5 * Wnt1 down vs E11.5 * Ctrl
10394852	EG668698	ENSMUST00000	0.00183861	-1.30426	E11.5 * Wnt1 down vs E11.5 * Ctrl
10344807	Cspp1	NM_026493	0.00145192	-1.30457	E11.5 * Wnt1 down vs E11.5 * Ctrl
10405605	Smad5	NM_008541	3.90555e-005	-1.30488	E11.5 * Wnt1 down vs E11.5 * Ctrl
10468746	Hspa12a	NM_175199	0.00279993	-1.30535	E11.5 * Wnt1 down vs E11.5 * Ctrl
10504169	4933409K07Rik	BC072647	0.0013073	-1.30558	E11.5 * Wnt1 down vs E11.5 * Ctrl
10595560	Tbx18	NM_023814	0.000140706	-1.30578	E11.5 * Wnt1 down vs E11.5 * Ctrl
10579347	Ifi30	NM_023065	0.00457455	-1.30605	E11.5 * Wnt1 down vs E11.5 * Ctrl
10504905	E130309F12Rik	NM_178756	0.00512052	-1.30622	E11.5 * Wnt1 down vs E11.5 * Ctrl
10593449	Layn	NM_001033534	0.00715789	-1.30632	E11.5 * Wnt1 down vs E11.5 * Ctrl
10359377	Zbtb37	NM_173424	9.19255e-005	-1.30635	E11.5 * Wnt1 down vs E11.5 * Ctrl
10585803	Stra6	NM_009291	0.00861366	-1.30639	E11.5 * Wnt1 down vs E11.5 * Ctrl
10487238	Hdc	NM_008230	0.000612348	-1.30697	E11.5 * Wnt1 down vs E11.5 * Ctrl
10605392	F8	NM_007977	0.00784364	-1.30713	E11.5 * Wnt1 down vs E11.5 * Ctrl
10588942	Lamb2	NM_008483	0.00182962	-1.30722	E11.5 * Wnt1 down vs E11.5 * Ctrl
10366983	Tmem194	NM_001113211	0.00403322	-1.30739	E11.5 * Wnt1 down vs E11.5 * Ctrl
10596166	1300017J02Rik	BC021390	9.68465e-005	-1.30796	E11.5 * Wnt1 down vs E11.5 * Ctrl
10500011	Tuft1	NM_011656	0.00339408	-1.30837	E11.5 * Wnt1 down vs E11.5 * Ctrl
10524331	C130026L21Rik	ENSMUST00000	0.00377298	-1.3091	E11.5 * Wnt1 down vs E11.5 * Ctrl
10607183	Lhfpl1	NM_178358	0.00431677	-1.3093	E11.5 * Wnt1 down vs E11.5 * Ctrl
10413943	Lrrc18	NM_026253	9.54018e-005	-1.30932	E11.5 * Wnt1 down vs E11.5 * Ctrl
10447513	ENSMUSG0000C	ENSMUST00000	0.00286607	-1.30971	E11.5 * Wnt1 down vs E11.5 * Ctrl
10395293	Atxn7l1	NM_001033436	0.00100094	-1.30994	E11.5 * Wnt1 down vs E11.5 * Ctrl
10551872	AI428936	NM_153577	0.00662241	-1.30994	E11.5 * Wnt1 down vs E11.5 * Ctrl
10586491	Dapk2	NM_010019	0.000655767	-1.31003	E11.5 * Wnt1 down vs E11.5 * Ctrl
10458090	Reep5	NM_007874	0.00209281	-1.31031	E11.5 * Wnt1 down vs E11.5 * Ctrl
10537880		---	0.0062942	-1.31032	E11.5 * Wnt1 down vs E11.5 * Ctrl
10400057	Arl4a	NM_001039515	0.000220513	-1.31058	E11.5 * Wnt1 down vs E11.5 * Ctrl
10447130	Pkdcc	NM_134117	0.00107578	-1.31073	E11.5 * Wnt1 down vs E11.5 * Ctrl
10557009	Eef2k	NM_007908	5.82175e-005	-1.31075	E11.5 * Wnt1 down vs E11.5 * Ctrl
10388545	Abr	NM_198895	0.00195233	-1.31081	E11.5 * Wnt1 down vs E11.5 * Ctrl
10359446	AI848100	BC138379	0.00178666	-1.31086	E11.5 * Wnt1 down vs E11.5 * Ctrl
10374236	Upp1	NM_009477	0.00618538	-1.31092	E11.5 * Wnt1 down vs E11.5 * Ctrl
10506786	Zcchc11	NM_175472	0.000590564	-1.31093	E11.5 * Wnt1 down vs E11.5 * Ctrl
10500982	I830077J02Rik	NM_001033780	0.00714922	-1.31108	E11.5 * Wnt1 down vs E11.5 * Ctrl
10418048	ENSMUSG0000C	ENSMUST00000	0.00352789	-1.31131	E11.5 * Wnt1 down vs E11.5 * Ctrl
10587829	Plod2	NM_001142916	0.000223567	-1.31148	E11.5 * Wnt1 down vs E11.5 * Ctrl
10607124	Chrdl1	NM_001114385	0.00586423	-1.31155	E11.5 * Wnt1 down vs E11.5 * Ctrl
10513608	Alad	NM_008525	0.00146505	-1.31188	E11.5 * Wnt1 down vs E11.5 * Ctrl
10354368	Col5a2	BC055077	0.00179341	-1.31231	E11.5 * Wnt1 down vs E11.5 * Ctrl
10596747	Sema3f	NM_011349	0.000132135	-1.31257	E11.5 * Wnt1 down vs E11.5 * Ctrl
10569437	Phlda2	NM_009434	0.00011389	-1.31273	E11.5 * Wnt1 down vs E11.5 * Ctrl

10595718	Chst2	NM_018763	0.00383349	-1.31323	E11.5 * Wnt1 down vs E11.5 * Ctrl
10399198	Ncoa4	NM_019744	5.65788e-005	-1.31337	E11.5 * Wnt1 down vs E11.5 * Ctrl
10566926	Rnf141	NM_025999	0.0070669	-1.3139	E11.5 * Wnt1 down vs E11.5 * Ctrl
10481186	Sardh	NM_138665	0.000788129	-1.31397	E11.5 * Wnt1 down vs E11.5 * Ctrl
10438907	Gp5	NM_008148	0.00442571	-1.31435	E11.5 * Wnt1 down vs E11.5 * Ctrl
10516335	Tekt2	NM_011902	0.00692923	-1.31483	E11.5 * Wnt1 down vs E11.5 * Ctrl
10518300	Tnfrsf1b	NM_011610	0.00495958	-1.31512	E11.5 * Wnt1 down vs E11.5 * Ctrl
10566264	EG436003	NM_001127686	0.000705699	-1.3159	E11.5 * Wnt1 down vs E11.5 * Ctrl
10559172	Krtap5-3	ENSMUST00000(	0.00783117	-1.31596	E11.5 * Wnt1 down vs E11.5 * Ctrl
10394805	5730507C01Rik	ENSMUST00000(	0.00130795	-1.31623	E11.5 * Wnt1 down vs E11.5 * Ctrl
10410099	Cdc14b	NM_172587	0.00161611	-1.3163	E11.5 * Wnt1 down vs E11.5 * Ctrl
10367434	ENSMUSG0000C	AK086046	0.00600649	-1.3164	E11.5 * Wnt1 down vs E11.5 * Ctrl
10408185	Btn2a2	NM_175938	0.000258837	-1.31671	E11.5 * Wnt1 down vs E11.5 * Ctrl
10404152	Fam65b	NM_029679	0.007576	-1.31685	E11.5 * Wnt1 down vs E11.5 * Ctrl
10522712	Rest	NM_011263	0.0018142	-1.31686	E11.5 * Wnt1 down vs E11.5 * Ctrl
10353460	Kcnq5	NM_023872	0.00213059	-1.31714	E11.5 * Wnt1 down vs E11.5 * Ctrl
10592535	Sorl1	NM_011436	8.06031e-005	-1.31723	E11.5 * Wnt1 down vs E11.5 * Ctrl
10600355	Snora70	NR_002899	0.000530807	-1.31731	E11.5 * Wnt1 down vs E11.5 * Ctrl
10602756	Smpx	NM_025357	0.00328549	-1.31752	E11.5 * Wnt1 down vs E11.5 * Ctrl
10544982	Nt5c3	NM_026004	0.00364824	-1.31763	E11.5 * Wnt1 down vs E11.5 * Ctrl
10512067	Ddx58	NM_172689	0.000176105	-1.3178	E11.5 * Wnt1 down vs E11.5 * Ctrl
10510178	OTTMUSG00000	NM_001037926	0.000687979	-1.31802	E11.5 * Wnt1 down vs E11.5 * Ctrl
10434285		---	0.00189368	-1.31805	E11.5 * Wnt1 down vs E11.5 * Ctrl
10544348	Trpv6	NM_022413	7.58029e-005	-1.31824	E11.5 * Wnt1 down vs E11.5 * Ctrl
10606864	Tceal5	NM_177919	0.00135941	-1.31834	E11.5 * Wnt1 down vs E11.5 * Ctrl
10415608	Ift88	NM_009376	0.00818594	-1.31836	E11.5 * Wnt1 down vs E11.5 * Ctrl
10411804	Mast4	ENSMUST00000(	4.34033e-005	-1.31848	E11.5 * Wnt1 down vs E11.5 * Ctrl
10517967	Fblim1	NM_133754	0.00706424	-1.31919	E11.5 * Wnt1 down vs E11.5 * Ctrl
10387797	Bcl6b	NM_007528	0.00648509	-1.31984	E11.5 * Wnt1 down vs E11.5 * Ctrl
10384233	Tns3	NM_001083587	0.0019797	-1.32003	E11.5 * Wnt1 down vs E11.5 * Ctrl
10411464	Fcho2	NM_172591	0.000821941	-1.32032	E11.5 * Wnt1 down vs E11.5 * Ctrl
10446771	Lclat1	NM_001081071	8.0172e-005	-1.32033	E11.5 * Wnt1 down vs E11.5 * Ctrl
10592772	Abcg4	NM_138955	9.44037e-005	-1.32069	E11.5 * Wnt1 down vs E11.5 * Ctrl
10504849	Stx17	NM_026343	0.00491725	-1.32073	E11.5 * Wnt1 down vs E11.5 * Ctrl
10552245	Tshz3	NM_172298	0.000452725	-1.32084	E11.5 * Wnt1 down vs E11.5 * Ctrl
10607189	Amot	NM_153319	0.00782983	-1.32086	E11.5 * Wnt1 down vs E11.5 * Ctrl
10600921	Stard8	NM_199018	0.000247333	-1.32094	E11.5 * Wnt1 down vs E11.5 * Ctrl
10591094	Fat3	NM_001080814	0.00202137	-1.32105	E11.5 * Wnt1 down vs E11.5 * Ctrl
10466745	Tjp2	NM_011597	0.00121724	-1.32131	E11.5 * Wnt1 down vs E11.5 * Ctrl
10594969	Unc13c	NM_001081153	0.000105183	-1.32175	E11.5 * Wnt1 down vs E11.5 * Ctrl
10413932	E130203B14Rik	NM_178791	0.00459949	-1.32182	E11.5 * Wnt1 down vs E11.5 * Ctrl
10544660	Osbpl3	NM_027881	0.00015043	-1.32187	E11.5 * Wnt1 down vs E11.5 * Ctrl
10526098	Scand3	NM_183088	0.00345008	-1.322	E11.5 * Wnt1 down vs E11.5 * Ctrl
10492424		---	0.00435608	-1.32203	E11.5 * Wnt1 down vs E11.5 * Ctrl
10414590	Ear6	NM_053111	0.00509398	-1.32218	E11.5 * Wnt1 down vs E11.5 * Ctrl
10445119	H2-M3	NM_013819	0.00383209	-1.3225	E11.5 * Wnt1 down vs E11.5 * Ctrl
10511446	Asph	NM_023066	8.96951e-005	-1.32266	E11.5 * Wnt1 down vs E11.5 * Ctrl
10586433	Rbpms2	NM_028030	0.00405912	-1.32294	E11.5 * Wnt1 down vs E11.5 * Ctrl
10479979	Slc25a36	NM_138756	0.0013048	-1.32323	E11.5 * Wnt1 down vs E11.5 * Ctrl
10504178	4933409K07Rik	BC059060	0.000446607	-1.32337	E11.5 * Wnt1 down vs E11.5 * Ctrl
10578253	Sgcz	NM_145841	0.0022436	-1.32353	E11.5 * Wnt1 down vs E11.5 * Ctrl
10483809	Nfe2l2	NM_010902	0.00650283	-1.3239	E11.5 * Wnt1 down vs E11.5 * Ctrl
10415413	BC030046	BC151043	0.00488978	-1.32421	E11.5 * Wnt1 down vs E11.5 * Ctrl
10458569	Nr3c1	NM_008173	0.00305867	-1.32462	E11.5 * Wnt1 down vs E11.5 * Ctrl
10601771	Armcx1	NM_030066	0.000655837	-1.32498	E11.5 * Wnt1 down vs E11.5 * Ctrl
10349102	Bcl2	NM_009741	0.00145847	-1.32515	E11.5 * Wnt1 down vs E11.5 * Ctrl
10504172	4933409K07Rik	BC059060	0.000580578	-1.32572	E11.5 * Wnt1 down vs E11.5 * Ctrl
10504188	Ccl19	NM_011888	0.00283484	-1.32585	E11.5 * Wnt1 down vs E11.5 * Ctrl
10404783	Edn1	NM_010104	0.00376788	-1.32588	E11.5 * Wnt1 down vs E11.5 * Ctrl
10465224	ENSMUSG0000C	ENSMUST00000(	0.00332634	-1.32622	E11.5 * Wnt1 down vs E11.5 * Ctrl
10582303	Cyba	NM_007806	0.0064265	-1.327	E11.5 * Wnt1 down vs E11.5 * Ctrl
10428604	Tnfrsf11b	NM_008764	0.00158052	-1.32706	E11.5 * Wnt1 down vs E11.5 * Ctrl
10566583	EG668139	AK172683	0.00540248	-1.32729	E11.5 * Wnt1 down vs E11.5 * Ctrl
10463277	Nkx2-3	NM_008699	0.00121055	-1.32743	E11.5 * Wnt1 down vs E11.5 * Ctrl
10505120	Palm2	NM_172868	0.00223137	-1.32764	E11.5 * Wnt1 down vs E11.5 * Ctrl
10446473	Lama1	NM_008480	0.000120144	-1.32765	E11.5 * Wnt1 down vs E11.5 * Ctrl

10420362	Gjb2	NM_008125	0.00248483	-1.32768	E11.5 * Wnt1 down vs E11.5 * Ctrl
10404429	Serpinb9	NM_009256	0.00676726	-1.32784	E11.5 * Wnt1 down vs E11.5 * Ctrl
10531338		---	0.00206397	-1.32797	E11.5 * Wnt1 down vs E11.5 * Ctrl
10398240	Eml1	NM_001043335	0.00105208	-1.32805	E11.5 * Wnt1 down vs E11.5 * Ctrl
10461402	Fth1	NM_010239	2.99447e-009	-1.32828	E11.5 * Wnt1 down vs E11.5 * Ctrl
10348739	Sned1	NM_172463	0.0029024	-1.32835	E11.5 * Wnt1 down vs E11.5 * Ctrl
10557326	Il4ra	NM_001008700	0.00523995	-1.32844	E11.5 * Wnt1 down vs E11.5 * Ctrl
10500938	Wnt2b	NM_009520	0.00610163	-1.32856	E11.5 * Wnt1 down vs E11.5 * Ctrl
10572861	F2rl3	NM_007975	0.00208693	-1.3287	E11.5 * Wnt1 down vs E11.5 * Ctrl
10595702	1190002N15Rik	NM_001033145	0.0022677	-1.32904	E11.5 * Wnt1 down vs E11.5 * Ctrl
10424439	Efr3a	NM_133766	0.00196477	-1.32911	E11.5 * Wnt1 down vs E11.5 * Ctrl
10384154	Myo1g	NM_178440	0.000647039	-1.32923	E11.5 * Wnt1 down vs E11.5 * Ctrl
10494351	Mtmr11	NM_181409	0.00259642	-1.32954	E11.5 * Wnt1 down vs E11.5 * Ctrl
10483178	Cobll1	NM_177025	0.0014793	-1.32958	E11.5 * Wnt1 down vs E11.5 * Ctrl
10561702	Kcnk6	NM_001033525	0.000184257	-1.32991	E11.5 * Wnt1 down vs E11.5 * Ctrl
10503148		---	0.00321386	-1.33017	E11.5 * Wnt1 down vs E11.5 * Ctrl
10544417	Epha1	NM_023580	0.00336875	-1.33044	E11.5 * Wnt1 down vs E11.5 * Ctrl
10506225	Cachd1	NM_198037	0.0053376	-1.33062	E11.5 * Wnt1 down vs E11.5 * Ctrl
10360185		---	0.00443957	-1.33153	E11.5 * Wnt1 down vs E11.5 * Ctrl
10548752		---	0.00828578	-1.33172	E11.5 * Wnt1 down vs E11.5 * Ctrl
10568361	Yipf5	NM_023311	0.00214743	-1.33253	E11.5 * Wnt1 down vs E11.5 * Ctrl
10504148	4933409K07Rik	BC059060	0.000819599	-1.33259	E11.5 * Wnt1 down vs E11.5 * Ctrl
10601874	Tceal3	NM_001029978	0.00112478	-1.33297	E11.5 * Wnt1 down vs E11.5 * Ctrl
10531529	Cnot6l	NM_178854	9.68297e-005	-1.33319	E11.5 * Wnt1 down vs E11.5 * Ctrl
10528799	Speer4a	NM_029376	0.00794274	-1.33339	E11.5 * Wnt1 down vs E11.5 * Ctrl
10360563	Smyd3	NM_027188	0.00504264	-1.33341	E11.5 * Wnt1 down vs E11.5 * Ctrl
10461979	Aldh1a1	NM_013467	0.00169135	-1.33369	E11.5 * Wnt1 down vs E11.5 * Ctrl
10541910	Vwf	NM_011708	0.000302929	-1.33369	E11.5 * Wnt1 down vs E11.5 * Ctrl
10512327	4933409K07Rik	BC072647	0.00091858	-1.33374	E11.5 * Wnt1 down vs E11.5 * Ctrl
10347748	Acsl3	NM_028817	0.00018138	-1.33403	E11.5 * Wnt1 down vs E11.5 * Ctrl
10353844	Neurl3	NM_153408	0.000433479	-1.33427	E11.5 * Wnt1 down vs E11.5 * Ctrl
10447923	Tcte2	NM_022311	4.7102e-005	-1.33468	E11.5 * Wnt1 down vs E11.5 * Ctrl
10541885	Scnn1a	NM_011324	0.0014867	-1.33469	E11.5 * Wnt1 down vs E11.5 * Ctrl
10407416	Calml3	NM_027416	0.00712498	-1.33478	E11.5 * Wnt1 down vs E11.5 * Ctrl
10496862		---	0.000707417	-1.33494	E11.5 * Wnt1 down vs E11.5 * Ctrl
10409202		---	0.000297603	-1.33501	E11.5 * Wnt1 down vs E11.5 * Ctrl
10421418	Epb4.9	NM_013514	8.49272e-005	-1.33515	E11.5 * Wnt1 down vs E11.5 * Ctrl
10523260	Shroom3	NM_015756	0.000278049	-1.33577	E11.5 * Wnt1 down vs E11.5 * Ctrl
10438749		---	0.00460681	-1.33666	E11.5 * Wnt1 down vs E11.5 * Ctrl
10355967	Ap1s3	NM_183027	0.00249206	-1.33699	E11.5 * Wnt1 down vs E11.5 * Ctrl
10405755		---	0.00086023	-1.33714	E11.5 * Wnt1 down vs E11.5 * Ctrl
10413434	D14Abb1e	NM_001114879	0.00062607	-1.33752	E11.5 * Wnt1 down vs E11.5 * Ctrl
10392142	Cd79b	NM_008339	0.00731131	-1.33769	E11.5 * Wnt1 down vs E11.5 * Ctrl
10494889	Dennd2c	NM_177857	0.000606592	-1.33778	E11.5 * Wnt1 down vs E11.5 * Ctrl
10493086	Hdgf	NM_008231	0.00114175	-1.33836	E11.5 * Wnt1 down vs E11.5 * Ctrl
10504159	Ccl19	NM_011888	0.00419383	-1.33892	E11.5 * Wnt1 down vs E11.5 * Ctrl
10512322	Ccl19	NM_011888	0.00419383	-1.33892	E11.5 * Wnt1 down vs E11.5 * Ctrl
10500754	Sycp1	NM_011516	0.000119107	-1.33903	E11.5 * Wnt1 down vs E11.5 * Ctrl
10503615		---	0.000438261	-1.33913	E11.5 * Wnt1 down vs E11.5 * Ctrl
10591660	Epor	NM_010149	4.5189e-006	-1.33917	E11.5 * Wnt1 down vs E11.5 * Ctrl
10400170	Prkd1	NM_008858	0.00230343	-1.3392	E11.5 * Wnt1 down vs E11.5 * Ctrl
10512930	Ppp3r2	NM_001004025	0.00570378	-1.3392	E11.5 * Wnt1 down vs E11.5 * Ctrl
10364072	Ggt5	NM_011820	2.76718e-005	-1.33932	E11.5 * Wnt1 down vs E11.5 * Ctrl
10374325	EG432534	ENSMUST00000(	0.0057847	-1.34031	E11.5 * Wnt1 down vs E11.5 * Ctrl
10495449	Col11a1	NM_007729	4.88483e-006	-1.34046	E11.5 * Wnt1 down vs E11.5 * Ctrl
10519879	Speer4f	NM_027609	0.000432048	-1.34135	E11.5 * Wnt1 down vs E11.5 * Ctrl
10383214	Rnf213	AK173199	0.000238995	-1.34142	E11.5 * Wnt1 down vs E11.5 * Ctrl
10504775	Col15a1	NM_009928	0.00027957	-1.34157	E11.5 * Wnt1 down vs E11.5 * Ctrl
10602722	Spin2	NM_001005370	0.00326915	-1.34187	E11.5 * Wnt1 down vs E11.5 * Ctrl
10474381	Kif18a	NM_139303	0.00125967	-1.34215	E11.5 * Wnt1 down vs E11.5 * Ctrl
10596428	1110001D15Rik	NM_026763	0.00458064	-1.34239	E11.5 * Wnt1 down vs E11.5 * Ctrl
10490384	Lama5	NM_001081171	8.65876e-005	-1.34247	E11.5 * Wnt1 down vs E11.5 * Ctrl
10485213	Cd82	NM_007656	0.00131794	-1.34291	E11.5 * Wnt1 down vs E11.5 * Ctrl
10489204	Tgm2	NM_009373	0.000577551	-1.34309	E11.5 * Wnt1 down vs E11.5 * Ctrl
10592289	Ccdc15	NM_001081429	0.0012089	-1.34328	E11.5 * Wnt1 down vs E11.5 * Ctrl
10549276	Bhlhe41	NM_024469	7.72928e-005	-1.34391	E11.5 * Wnt1 down vs E11.5 * Ctrl

10585331	Exph5	NM_176846	0.00097366	-1.34441	E11.5 * Wnt1 down vs E11.5 * Ctrl
10413416	Il17rd	NM_134437	0.00146726	-1.34536	E11.5 * Wnt1 down vs E11.5 * Ctrl
10488090	Tasp1	NM_175225	0.000247892	-1.34552	E11.5 * Wnt1 down vs E11.5 * Ctrl
10528679	Wdr86	NM_001081441	0.000681468	-1.3456	E11.5 * Wnt1 down vs E11.5 * Ctrl
10461078	Atl3	NM_146091	0.000180831	-1.34615	E11.5 * Wnt1 down vs E11.5 * Ctrl
10391404	D830013H23Rik	AK052868	0.00761294	-1.34624	E11.5 * Wnt1 down vs E11.5 * Ctrl
10492310	Mbnl1	NM_020007	1.05692e-005	-1.34642	E11.5 * Wnt1 down vs E11.5 * Ctrl
10511588	Tmem67	NM_177861	0.00292857	-1.34651	E11.5 * Wnt1 down vs E11.5 * Ctrl
10352936	Sox17	NM_011441	0.00140201	-1.34661	E11.5 * Wnt1 down vs E11.5 * Ctrl
10569485	Tnfrsf26	NM_175649	0.00468567	-1.34681	E11.5 * Wnt1 down vs E11.5 * Ctrl
10590690	Dync2h1	NM_029851	8.56372e-006	-1.34715	E11.5 * Wnt1 down vs E11.5 * Ctrl
10500570	Hao3	NM_019545	0.000315298	-1.3474	E11.5 * Wnt1 down vs E11.5 * Ctrl
10557960	Tgfb1i1	NM_009365	0.001779	-1.34839	E11.5 * Wnt1 down vs E11.5 * Ctrl
10552500	Klk8	NM_008940	0.000953749	-1.34845	E11.5 * Wnt1 down vs E11.5 * Ctrl
10375578	Flt4	NM_008029	0.00116801	-1.34909	E11.5 * Wnt1 down vs E11.5 * Ctrl
10474619	Fmn1	NM_010230	0.00491283	-1.34915	E11.5 * Wnt1 down vs E11.5 * Ctrl
10579012	Csgalnact1	NM_172753	0.00857466	-1.34945	E11.5 * Wnt1 down vs E11.5 * Ctrl
10533034	Ccdc60	NM_177759	0.00275969	-1.34988	E11.5 * Wnt1 down vs E11.5 * Ctrl
10512372	Ccl19	NM_011888	0.00223483	-1.35016	E11.5 * Wnt1 down vs E11.5 * Ctrl
10530393	Gabrg1	NM_010252	0.000814574	-1.35028	E11.5 * Wnt1 down vs E11.5 * Ctrl
10578322	Pdgfrl	NM_026840	0.00873024	-1.35043	E11.5 * Wnt1 down vs E11.5 * Ctrl
10503484	Fam82b	NM_025476	0.00608699	-1.35053	E11.5 * Wnt1 down vs E11.5 * Ctrl
10537558	621968	ENSMUST00000	0.00545877	-1.35055	E11.5 * Wnt1 down vs E11.5 * Ctrl
10596465	Acy1	NM_025371	1.67942e-005	-1.35117	E11.5 * Wnt1 down vs E11.5 * Ctrl
10474860	Chst14	NM_028117	0.000153524	-1.35143	E11.5 * Wnt1 down vs E11.5 * Ctrl
10410235		---	0.00567808	-1.35155	E11.5 * Wnt1 down vs E11.5 * Ctrl
10572897	Hmox1	NM_010442	0.00131406	-1.35206	E11.5 * Wnt1 down vs E11.5 * Ctrl
10591161	Zfp558	ENSMUST00000	0.00774315	-1.35218	E11.5 * Wnt1 down vs E11.5 * Ctrl
10373325	Gpr182	NM_007412	0.000308811	-1.35232	E11.5 * Wnt1 down vs E11.5 * Ctrl
10427622		---	0.00704916	-1.35306	E11.5 * Wnt1 down vs E11.5 * Ctrl
10490872	Lrrcc1	NM_028915	0.00176413	-1.35326	E11.5 * Wnt1 down vs E11.5 * Ctrl
10504139	4933409K07Rik	BC059060	0.000513106	-1.35342	E11.5 * Wnt1 down vs E11.5 * Ctrl
10363786	Ank3	NM_146005	0.00108982	-1.35356	E11.5 * Wnt1 down vs E11.5 * Ctrl
10360848	Mosc2	NM_133684	2.89467e-006	-1.35438	E11.5 * Wnt1 down vs E11.5 * Ctrl
10506254	Raver2	NM_183024	0.000636734	-1.35455	E11.5 * Wnt1 down vs E11.5 * Ctrl
10467578	Pik3ap1	NM_031376	0.00011702	-1.35551	E11.5 * Wnt1 down vs E11.5 * Ctrl
10523021	Slc4a4	NM_018760	0.00143039	-1.35563	E11.5 * Wnt1 down vs E11.5 * Ctrl
10513592	Wdr31	NM_023597	0.00135209	-1.35597	E11.5 * Wnt1 down vs E11.5 * Ctrl
10474371	ENSMUSG00000	ENSMUST00000	0.00193932	-1.3561	E11.5 * Wnt1 down vs E11.5 * Ctrl
10427845	F830212C03Rik	AK157350	0.00323356	-1.35682	E11.5 * Wnt1 down vs E11.5 * Ctrl
10410475	BC018507	ENSMUST00000	5.67757e-005	-1.35708	E11.5 * Wnt1 down vs E11.5 * Ctrl
10454398	AW554918	NM_001033532	0.00258319	-1.35716	E11.5 * Wnt1 down vs E11.5 * Ctrl
10380260	Trim25	NM_009546	0.00211043	-1.35777	E11.5 * Wnt1 down vs E11.5 * Ctrl
10469514	Bmi1	NM_007552	0.00337366	-1.35777	E11.5 * Wnt1 down vs E11.5 * Ctrl
10549473	Caprin2	NM_181541	3.30574e-005	-1.35801	E11.5 * Wnt1 down vs E11.5 * Ctrl
10377662	Ybx2	NM_016875	0.000417098	-1.35841	E11.5 * Wnt1 down vs E11.5 * Ctrl
10355152	Fzd5	NM_022721	0.0043918	-1.35843	E11.5 * Wnt1 down vs E11.5 * Ctrl
10593799	Scaper	NM_001081341	6.11459e-005	-1.35958	E11.5 * Wnt1 down vs E11.5 * Ctrl
10408574		---	0.00224644	-1.35974	E11.5 * Wnt1 down vs E11.5 * Ctrl
10417887	Zmynd17	BC119816	0.00834187	-1.36008	E11.5 * Wnt1 down vs E11.5 * Ctrl
10594103	2410076I21Rik	ENSMUST00000	0.000257328	-1.3602	E11.5 * Wnt1 down vs E11.5 * Ctrl
10547322	Cacna1c	NM_001159533	1.31679e-005	-1.3602	E11.5 * Wnt1 down vs E11.5 * Ctrl
10459496	Ccbe1	NM_178793	2.54233e-006	-1.36031	E11.5 * Wnt1 down vs E11.5 * Ctrl
10528664	Smarcd3	NM_025891	0.000942496	-1.36037	E11.5 * Wnt1 down vs E11.5 * Ctrl
10605181	Renbp	NM_023132	0.00427775	-1.36083	E11.5 * Wnt1 down vs E11.5 * Ctrl
10530269	Rbm47	NM_178446	0.00270867	-1.36086	E11.5 * Wnt1 down vs E11.5 * Ctrl
10361186	Sertad4	NM_198247	9.09896e-005	-1.36146	E11.5 * Wnt1 down vs E11.5 * Ctrl
10425761		---	0.00016409	-1.36158	E11.5 * Wnt1 down vs E11.5 * Ctrl
10596072	3222402P14Rik	BC151170	0.000221647	-1.36161	E11.5 * Wnt1 down vs E11.5 * Ctrl
10515771	Tie1	NM_011587	0.00032721	-1.36168	E11.5 * Wnt1 down vs E11.5 * Ctrl
10415742	Mipep	NM_027436	0.000393954	-1.36233	E11.5 * Wnt1 down vs E11.5 * Ctrl
10455738	Snx2	NM_026386	4.90687e-006	-1.36248	E11.5 * Wnt1 down vs E11.5 * Ctrl
10584591		---	0.000115316	-1.36281	E11.5 * Wnt1 down vs E11.5 * Ctrl
10382438	Cd300a	NM_170758	0.00143511	-1.36287	E11.5 * Wnt1 down vs E11.5 * Ctrl
10379034	Tlcd1	NM_026708	0.0027165	-1.36333	E11.5 * Wnt1 down vs E11.5 * Ctrl
10351679	Cd84	NM_013489	0.00461257	-1.36371	E11.5 * Wnt1 down vs E11.5 * Ctrl

10446425		---	0.00361027	-1.3641	E11.5 * Wnt1 down vs E11.5 * Ctrl
10404132	Cmah	NM_001111110	0.00788985	-1.3643	E11.5 * Wnt1 down vs E11.5 * Ctrl
10464328	Pnliprp2	NM_011128	0.000224961	-1.36439	E11.5 * Wnt1 down vs E11.5 * Ctrl
10428536	Trps1	NM_032000	0.00104943	-1.3646	E11.5 * Wnt1 down vs E11.5 * Ctrl
10559200	Tnni2	NM_009405	0.000139126	-1.36469	E11.5 * Wnt1 down vs E11.5 * Ctrl
10419286	ENSMUSG0000C	ENSMUST00000	0.00654049	-1.36503	E11.5 * Wnt1 down vs E11.5 * Ctrl
10439830		---	0.00875684	-1.36623	E11.5 * Wnt1 down vs E11.5 * Ctrl
10421293	Ppp3cc	NM_008915	0.00242532	-1.36631	E11.5 * Wnt1 down vs E11.5 * Ctrl
10605303	Dnase1l1	NM_027109	8.11143e-005	-1.36641	E11.5 * Wnt1 down vs E11.5 * Ctrl
10477942	Rbl1	NM_011249	0.0030814	-1.36747	E11.5 * Wnt1 down vs E11.5 * Ctrl
10584855	Scn2b	NM_001014761	0.000184932	-1.36756	E11.5 * Wnt1 down vs E11.5 * Ctrl
10360412	Olfr419	NM_146715	0.000700645	-1.36764	E11.5 * Wnt1 down vs E11.5 * Ctrl
10406407	Arrdc3	NM_001042591	0.00561145	-1.36787	E11.5 * Wnt1 down vs E11.5 * Ctrl
10440393	Samsn1	NM_023380	0.000374954	-1.36879	E11.5 * Wnt1 down vs E11.5 * Ctrl
10605370	Mpp1	NM_008621	0.00101332	-1.36885	E11.5 * Wnt1 down vs E11.5 * Ctrl
10381588	Grn	NM_008175	0.00159547	-1.36891	E11.5 * Wnt1 down vs E11.5 * Ctrl
10537179	Bpgm	NM_007563	0.000649877	-1.36913	E11.5 * Wnt1 down vs E11.5 * Ctrl
10485948	Grem1	NM_011824	0.00589121	-1.36961	E11.5 * Wnt1 down vs E11.5 * Ctrl
10457407		---	4.906e-006	-1.36967	E11.5 * Wnt1 down vs E11.5 * Ctrl
10500610	Fam46c	NM_001142952	0.00667661	-1.37013	E11.5 * Wnt1 down vs E11.5 * Ctrl
10575844	Cdh13	NM_019707	0.00329226	-1.37044	E11.5 * Wnt1 down vs E11.5 * Ctrl
10492864	Sh3d19	NM_001082414	9.28569e-005	-1.37069	E11.5 * Wnt1 down vs E11.5 * Ctrl
10552363	I1C0022H11Rik	NM_001034893	0.000233517	-1.37083	E11.5 * Wnt1 down vs E11.5 * Ctrl
10596095	Ephb1	NM_173447	0.000202751	-1.37094	E11.5 * Wnt1 down vs E11.5 * Ctrl
10552469	Klk13	NM_001039042	1.28693e-005	-1.37199	E11.5 * Wnt1 down vs E11.5 * Ctrl
10573939	Lpcat2	NM_173014	0.00126384	-1.3722	E11.5 * Wnt1 down vs E11.5 * Ctrl
10578690	Neil3	NM_146208	0.000144993	-1.37231	E11.5 * Wnt1 down vs E11.5 * Ctrl
10368356	Akap7	NM_018747	0.000339278	-1.37273	E11.5 * Wnt1 down vs E11.5 * Ctrl
10401028	Sgpp1	NM_030750	0.00575051	-1.37286	E11.5 * Wnt1 down vs E11.5 * Ctrl
10578241	A730069N07Rik	NM_178735	3.53219e-005	-1.37321	E11.5 * Wnt1 down vs E11.5 * Ctrl
10531479	4932430I15Rik	ENSMUST00000	0.000191948	-1.37338	E11.5 * Wnt1 down vs E11.5 * Ctrl
10440425		---	0.00507205	-1.37352	E11.5 * Wnt1 down vs E11.5 * Ctrl
10396278	Daam1	NM_026102	5.34698e-005	-1.37407	E11.5 * Wnt1 down vs E11.5 * Ctrl
10364262	Itgb2	NM_008404	0.000587758	-1.37515	E11.5 * Wnt1 down vs E11.5 * Ctrl
10423230	Cdh9	NM_009869	0.00110477	-1.37533	E11.5 * Wnt1 down vs E11.5 * Ctrl
10425410	Grap2	NM_010815	0.000643775	-1.37582	E11.5 * Wnt1 down vs E11.5 * Ctrl
10487208	Atp8b4	NM_001080944	0.000161524	-1.37623	E11.5 * Wnt1 down vs E11.5 * Ctrl
10552358	I1C0022H11Rik	NM_001034893	0.000334299	-1.37635	E11.5 * Wnt1 down vs E11.5 * Ctrl
10365420	AI597468	NM_001013028	7.18874e-006	-1.37666	E11.5 * Wnt1 down vs E11.5 * Ctrl
10420171	Adcy4	NM_080435	0.00808225	-1.37687	E11.5 * Wnt1 down vs E11.5 * Ctrl
10602105	Col4a5	NM_007736	0.000327785	-1.37695	E11.5 * Wnt1 down vs E11.5 * Ctrl
10539517	Dysf	NM_021469	1.05266e-005	-1.37707	E11.5 * Wnt1 down vs E11.5 * Ctrl
10419744	Slc7a7	NM_011405	0.00411802	-1.37718	E11.5 * Wnt1 down vs E11.5 * Ctrl
10346348	2810022L02Rik	NM_144882	0.000389716	-1.37761	E11.5 * Wnt1 down vs E11.5 * Ctrl
10500911	Mov10	NM_008619	0.000928327	-1.3777	E11.5 * Wnt1 down vs E11.5 * Ctrl
10428302	Klf10	NM_013692	7.40865e-005	-1.37791	E11.5 * Wnt1 down vs E11.5 * Ctrl
10356278	Sp110	NM_175397	0.000128605	-1.37802	E11.5 * Wnt1 down vs E11.5 * Ctrl
10560329	Hif3a	NM_016868	0.000302026	-1.37839	E11.5 * Wnt1 down vs E11.5 * Ctrl
10391119	1110036O03Rik	NM_176830	3.92384e-006	-1.3785	E11.5 * Wnt1 down vs E11.5 * Ctrl
10544763	Hoxa4	NM_008265	0.00015536	-1.37927	E11.5 * Wnt1 down vs E11.5 * Ctrl
10372988	Slc16a7	NM_011391	0.00442616	-1.37999	E11.5 * Wnt1 down vs E11.5 * Ctrl
10381603	Fzd2	NM_020510	0.00335181	-1.38	E11.5 * Wnt1 down vs E11.5 * Ctrl
10346722	Nbeal1	NM_173444	0.000471835	-1.38033	E11.5 * Wnt1 down vs E11.5 * Ctrl
10578324	Mtus1	NM_001005863	2.88209e-005	-1.38053	E11.5 * Wnt1 down vs E11.5 * Ctrl
10524878	BC023744	NM_001033311	0.00138852	-1.38063	E11.5 * Wnt1 down vs E11.5 * Ctrl
10472514	Nostrin	NM_181547	0.00376245	-1.38121	E11.5 * Wnt1 down vs E11.5 * Ctrl
10399965	F730043M19Rik	ENSMUST00000(	0.00373659	-1.38161	E11.5 * Wnt1 down vs E11.5 * Ctrl
10568355	Pycard	NM_023258	0.00341353	-1.38161	E11.5 * Wnt1 down vs E11.5 * Ctrl
10399148	Rapgef5	NM_175930	0.00285495	-1.38175	E11.5 * Wnt1 down vs E11.5 * Ctrl
10594661	Tpm1	NM_024427	7.06093e-005	-1.3818	E11.5 * Wnt1 down vs E11.5 * Ctrl
10416700	Pcdh17	NM_001013753	0.00317427	-1.38258	E11.5 * Wnt1 down vs E11.5 * Ctrl
10476560	Ism1	NM_001126490	0.00552798	-1.38301	E11.5 * Wnt1 down vs E11.5 * Ctrl
10553354	Nav2	NM_175272	6.24184e-005	-1.38352	E11.5 * Wnt1 down vs E11.5 * Ctrl
10599736	Fhl1	NM_001077361	7.48645e-006	-1.38381	E11.5 * Wnt1 down vs E11.5 * Ctrl
10473809	Sfpi1	NM_011355	2.66476e-005	-1.38408	E11.5 * Wnt1 down vs E11.5 * Ctrl
10550877	Kcnn4	NM_008433	4.45148e-005	-1.38409	E11.5 * Wnt1 down vs E11.5 * Ctrl

10583669	AB124611	AB124611	0.00309471	-1.38489	E11.5 * Wnt1 down vs E11.5 * Ctrl
10548875	Art4	NM_026639	8.35166e-005	-1.38527	E11.5 * Wnt1 down vs E11.5 * Ctrl
10394789	B430203G13Rik	AK046603	9.9922e-005	-1.38534	E11.5 * Wnt1 down vs E11.5 * Ctrl
10528008	Steap2	NM_001103157	0.00140941	-1.38545	E11.5 * Wnt1 down vs E11.5 * Ctrl
10361139	Traf5	NM_011633	0.000414263	-1.38607	E11.5 * Wnt1 down vs E11.5 * Ctrl
10362630		---	0.00370467	-1.38634	E11.5 * Wnt1 down vs E11.5 * Ctrl
10514000	Mpdz	NM_010820	7.98664e-006	-1.38651	E11.5 * Wnt1 down vs E11.5 * Ctrl
10473432	Tnks1bp1	NM_001081260	0.000303159	-1.38663	E11.5 * Wnt1 down vs E11.5 * Ctrl
10358982	Mr1	NM_008209	0.00615351	-1.3867	E11.5 * Wnt1 down vs E11.5 * Ctrl
10516481	Gja4	NM_008120	0.000230837	-1.38672	E11.5 * Wnt1 down vs E11.5 * Ctrl
10506154	Alg6	NM_001081264	0.00378113	-1.38688	E11.5 * Wnt1 down vs E11.5 * Ctrl
10539773	Gfpt1	NM_013528	0.00040034	-1.38726	E11.5 * Wnt1 down vs E11.5 * Ctrl
10381122	Fkbp10	NM_010221	0.00214077	-1.3873	E11.5 * Wnt1 down vs E11.5 * Ctrl
10530592	Fryl	NM_177136	0.000272409	-1.38769	E11.5 * Wnt1 down vs E11.5 * Ctrl
10372280	EG628870	XM_893664	0.000294589	-1.38797	E11.5 * Wnt1 down vs E11.5 * Ctrl
10581538	Nqo1	NM_008706	0.00699245	-1.38807	E11.5 * Wnt1 down vs E11.5 * Ctrl
10416785	4930517O19Rik	ENSMUST00000(	0.00106633	-1.38851	E11.5 * Wnt1 down vs E11.5 * Ctrl
10513160	Ptpn3	NM_011207	0.000428108	-1.38876	E11.5 * Wnt1 down vs E11.5 * Ctrl
10531994	Mpa2l	NM_194336	9.43944e-005	-1.3888	E11.5 * Wnt1 down vs E11.5 * Ctrl
10458999	Fbn2	NM_010181	0.000398237	-1.38883	E11.5 * Wnt1 down vs E11.5 * Ctrl
10346878	Zdbf2	ENSMUST00000	6.47799e-005	-1.38891	E11.5 * Wnt1 down vs E11.5 * Ctrl
10451613	Foxp4	NM_001110824	0.00280485	-1.38945	E11.5 * Wnt1 down vs E11.5 * Ctrl
10399671	Hpcal1	NM_016677	0.000143021	-1.38954	E11.5 * Wnt1 down vs E11.5 * Ctrl
10498992	Tlr2	NM_011905	0.00146723	-1.39013	E11.5 * Wnt1 down vs E11.5 * Ctrl
10473406	Prg3	NM_016914	0.000349927	-1.3903	E11.5 * Wnt1 down vs E11.5 * Ctrl
10449419	Tead3	NM_001098226	0.00293181	-1.39165	E11.5 * Wnt1 down vs E11.5 * Ctrl
10485378	2600010E01Rik	NM_001083810	0.001768	-1.39166	E11.5 * Wnt1 down vs E11.5 * Ctrl
10588263	Slco2a1	NM_033314	0.00702084	-1.39166	E11.5 * Wnt1 down vs E11.5 * Ctrl
10551185	Tgfb1	NM_011577	0.00106149	-1.39198	E11.5 * Wnt1 down vs E11.5 * Ctrl
10427049	6030408B16Rik	ENSMUST00000(	0.000290359	-1.39212	E11.5 * Wnt1 down vs E11.5 * Ctrl
10440186	Crybg3	NM_174848	0.00419358	-1.39226	E11.5 * Wnt1 down vs E11.5 * Ctrl
10347792		---	0.00409433	-1.39246	E11.5 * Wnt1 down vs E11.5 * Ctrl
10482766	Rprm	NM_023396	0.000537931	-1.39265	E11.5 * Wnt1 down vs E11.5 * Ctrl
10383152		---	5.80254e-006	-1.39305	E11.5 * Wnt1 down vs E11.5 * Ctrl
10564520	Rpl23	AF357384	0.00500198	-1.3935	E11.5 * Wnt1 down vs E11.5 * Ctrl
10437673	Tnp2	NM_013694	0.0011812	-1.39433	E11.5 * Wnt1 down vs E11.5 * Ctrl
10427303	Hoxc4	NM_013553	0.00148605	-1.3944	E11.5 * Wnt1 down vs E11.5 * Ctrl
10490854	OTTMUSG00000	NM_001037926	0.00205173	-1.39453	E11.5 * Wnt1 down vs E11.5 * Ctrl
10540359	Cntn4	NM_001109749	0.00045548	-1.39463	E11.5 * Wnt1 down vs E11.5 * Ctrl
10369690	Tet1	AK129421	1.63713e-005	-1.39483	E11.5 * Wnt1 down vs E11.5 * Ctrl
10462195	Kank1	NM_181404	0.000321981	-1.39504	E11.5 * Wnt1 down vs E11.5 * Ctrl
10528332	Napepld	NM_178728	5.36855e-006	-1.39509	E11.5 * Wnt1 down vs E11.5 * Ctrl
10398432		---	0.00160174	-1.3952	E11.5 * Wnt1 down vs E11.5 * Ctrl
10446619	Myom1	NM_010867	0.00113363	-1.39551	E11.5 * Wnt1 down vs E11.5 * Ctrl
10491623	4932438A13Rik	NM_172679	0.00454219	-1.39553	E11.5 * Wnt1 down vs E11.5 * Ctrl
10478744		---	0.00813397	-1.39563	E11.5 * Wnt1 down vs E11.5 * Ctrl
10414805	Trav13d-4	ENSMUST00000	0.00398536	-1.39615	E11.5 * Wnt1 down vs E11.5 * Ctrl
10384770	5730522E02Rik	ENSMUST00000	0.000146996	-1.39657	E11.5 * Wnt1 down vs E11.5 * Ctrl
10369264	Oit3	NM_010959	2.32041e-005	-1.39719	E11.5 * Wnt1 down vs E11.5 * Ctrl
10372796	Hmga2	NM_010441	0.000254734	-1.39747	E11.5 * Wnt1 down vs E11.5 * Ctrl
10510167	OTTMUSG00000	NM_001037926	0.000966029	-1.39822	E11.5 * Wnt1 down vs E11.5 * Ctrl
10389134	Slfn9	NM_172796	5.02369e-005	-1.3988	E11.5 * Wnt1 down vs E11.5 * Ctrl
10357003	Rnf152	NM_178779	0.00529393	-1.39918	E11.5 * Wnt1 down vs E11.5 * Ctrl
10597973	Lztfl1	NM_033322	0.000202127	-1.40035	E11.5 * Wnt1 down vs E11.5 * Ctrl
10564539	Mctp2	NM_001024703	0.000451143	-1.40087	E11.5 * Wnt1 down vs E11.5 * Ctrl
10353524	Ogfrl1	NM_001081079	0.000343467	-1.40092	E11.5 * Wnt1 down vs E11.5 * Ctrl
10366004	Atp2b1	NM_026482	1.22484e-009	-1.4013	E11.5 * Wnt1 down vs E11.5 * Ctrl
10403081	Wdr60	NM_146039	0.000138307	-1.40135	E11.5 * Wnt1 down vs E11.5 * Ctrl
10366886	Arhgap9	NM_146011	0.000276182	-1.40164	E11.5 * Wnt1 down vs E11.5 * Ctrl
10418244	Fam116a	NM_001134465	0.00152696	-1.40253	E11.5 * Wnt1 down vs E11.5 * Ctrl
10406982	Adamts6	NM_001081020	0.0033782	-1.40264	E11.5 * Wnt1 down vs E11.5 * Ctrl
10506767	Echdc2	NM_026728	0.00011146	-1.40365	E11.5 * Wnt1 down vs E11.5 * Ctrl
10404301	Prl3a1	NM_025896	0.000230004	-1.40376	E11.5 * Wnt1 down vs E11.5 * Ctrl
10395428	Dgkb	NM_178681	0.00383488	-1.40377	E11.5 * Wnt1 down vs E11.5 * Ctrl
10524310	Ttc28	BC002262	0.00872753	-1.40402	E11.5 * Wnt1 down vs E11.5 * Ctrl
10549879	Usp29	NM_021323	0.00112089	-1.40429	E11.5 * Wnt1 down vs E11.5 * Ctrl

10455118	Pcdhb18	NM_053143	0.00184197	-1.40454	E11.5 * Wnt1 down vs E11.5 * Ctrl
10508972	434166	BC080727	0.000481705	-1.40457	E11.5 * Wnt1 down vs E11.5 * Ctrl
10522925	Prol1	NM_008644	0.000204833	-1.40479	E11.5 * Wnt1 down vs E11.5 * Ctrl
10607870	Tlr7	NM_133211	0.000557952	-1.4049	E11.5 * Wnt1 down vs E11.5 * Ctrl
10589884	Bcl2a1c	NM_007535	0.000220336	-1.40515	E11.5 * Wnt1 down vs E11.5 * Ctrl
10369116	Mcm9	NM_027830	0.00212683	-1.40562	E11.5 * Wnt1 down vs E11.5 * Ctrl
10379389	Adap2	NM_172133	0.000201783	-1.40568	E11.5 * Wnt1 down vs E11.5 * Ctrl
10607562	Cnksr2	NM_177751	0.000538466	-1.4064	E11.5 * Wnt1 down vs E11.5 * Ctrl
10530100	Arap2	NM_178407	0.002444	-1.40647	E11.5 * Wnt1 down vs E11.5 * Ctrl
10436550		---	0.0028425	-1.40689	E11.5 * Wnt1 down vs E11.5 * Ctrl
10479165	Edn3	NM_007903	6.89849e-006	-1.40692	E11.5 * Wnt1 down vs E11.5 * Ctrl
10584827	Mpzl2	NM_007962	0.00178612	-1.40725	E11.5 * Wnt1 down vs E11.5 * Ctrl
10498386	Igsf10	ENSMUST00000	7.22446e-005	-1.40739	E11.5 * Wnt1 down vs E11.5 * Ctrl
10579776	Arhgap10	NM_030113	1.71684e-005	-1.40745	E11.5 * Wnt1 down vs E11.5 * Ctrl
10583163	Trpc6	NM_013838	0.000202013	-1.40751	E11.5 * Wnt1 down vs E11.5 * Ctrl
10466200	Ms4a7	NM_027836	0.00305677	-1.40776	E11.5 * Wnt1 down vs E11.5 * Ctrl
10544798	Hoxa10	NM_008263	8.76563e-005	-1.40822	E11.5 * Wnt1 down vs E11.5 * Ctrl
10556082	Ppfibp2	NM_008905	0.000355398	-1.40829	E11.5 * Wnt1 down vs E11.5 * Ctrl
10424252	Wdyhv1	NM_029734	0.00248912	-1.40835	E11.5 * Wnt1 down vs E11.5 * Ctrl
10435345	Mylk	NM_139300	9.82762e-005	-1.4084	E11.5 * Wnt1 down vs E11.5 * Ctrl
10561055	Ceacam2	NM_001113368	0.0048413	-1.40841	E11.5 * Wnt1 down vs E11.5 * Ctrl
10524312	Ttc28	ENSMUST00000	0.000766227	-1.40876	E11.5 * Wnt1 down vs E11.5 * Ctrl
10357008	Pign	NM_013784	3.06189e-006	-1.40886	E11.5 * Wnt1 down vs E11.5 * Ctrl
10597279	Ccrl2	NM_017466	0.00598358	-1.40911	E11.5 * Wnt1 down vs E11.5 * Ctrl
10497079	Ptger3	NM_011196	7.32709e-005	-1.40919	E11.5 * Wnt1 down vs E11.5 * Ctrl
10418848	Wdfy4	NM_001146022	0.000266185	-1.40938	E11.5 * Wnt1 down vs E11.5 * Ctrl
10586446		---	0.00106242	-1.40965	E11.5 * Wnt1 down vs E11.5 * Ctrl
10361381	Syne1	NM_001079686	2.16702e-006	-1.41084	E11.5 * Wnt1 down vs E11.5 * Ctrl
10593834	Rfpl3s	NM_183111	0.00244228	-1.41147	E11.5 * Wnt1 down vs E11.5 * Ctrl
10411611	Naip5	NM_010870	0.00110377	-1.41239	E11.5 * Wnt1 down vs E11.5 * Ctrl
10437885	Myh11	NM_013607	0.00634746	-1.41254	E11.5 * Wnt1 down vs E11.5 * Ctrl
10482880	Baz2b	NM_001001182	5.2886e-007	-1.41286	E11.5 * Wnt1 down vs E11.5 * Ctrl
10456745	Smad7	NM_001042660	0.000337842	-1.41304	E11.5 * Wnt1 down vs E11.5 * Ctrl
10382200	Ccdc46	NM_029606	3.16429e-005	-1.41308	E11.5 * Wnt1 down vs E11.5 * Ctrl
10385903	Pdlim4	NM_019417	9.57517e-005	-1.41335	E11.5 * Wnt1 down vs E11.5 * Ctrl
10600698	5430427O19Rik	BC115576	0.00621907	-1.41354	E11.5 * Wnt1 down vs E11.5 * Ctrl
10398388		---	0.000560687	-1.41458	E11.5 * Wnt1 down vs E11.5 * Ctrl
10560190	Ehd2	NM_153068	0.000405379	-1.41462	E11.5 * Wnt1 down vs E11.5 * Ctrl
10389786	Hlf	NM_172563	7.86348e-005	-1.41462	E11.5 * Wnt1 down vs E11.5 * Ctrl
10389929	Cacna1g	NM_009783	0.000482008	-1.41514	E11.5 * Wnt1 down vs E11.5 * Ctrl
10434778	Rtp4	NM_023386	0.00153461	-1.41531	E11.5 * Wnt1 down vs E11.5 * Ctrl
10497731	Ccdc39	NM_026222	0.000558221	-1.41577	E11.5 * Wnt1 down vs E11.5 * Ctrl
10385526	9930111J21Rik	NM_173434	0.00147579	-1.41602	E11.5 * Wnt1 down vs E11.5 * Ctrl
10399046	Vipr2	NM_009511	0.00379172	-1.41611	E11.5 * Wnt1 down vs E11.5 * Ctrl
10399232	ENSMUSG00000	ENSMUST00000	1.3715e-006	-1.41612	E11.5 * Wnt1 down vs E11.5 * Ctrl
10491627	4932438A13Rik	NM_172679	0.00567322	-1.41658	E11.5 * Wnt1 down vs E11.5 * Ctrl
10354563	Dnahc7b	ENSMUST00000	0.00864074	-1.41658	E11.5 * Wnt1 down vs E11.5 * Ctrl
10590060	Ctdspl	NM_133710	7.0687e-006	-1.41697	E11.5 * Wnt1 down vs E11.5 * Ctrl
10561104	Axl	NM_009465	0.000594632	-1.41768	E11.5 * Wnt1 down vs E11.5 * Ctrl
10384780	Fancl	NM_025923	0.00814449	-1.41846	E11.5 * Wnt1 down vs E11.5 * Ctrl
10368527	Hint3	NM_025798	0.00313819	-1.41873	E11.5 * Wnt1 down vs E11.5 * Ctrl
10495596	Frrs1	NM_001113478	0.00196851	-1.41911	E11.5 * Wnt1 down vs E11.5 * Ctrl
10453233	Slc8a1	NM_011406	9.76491e-005	-1.41913	E11.5 * Wnt1 down vs E11.5 * Ctrl
10454632	Camk4	NM_009793	0.00307189	-1.41949	E11.5 * Wnt1 down vs E11.5 * Ctrl
10428534	Trps1	NM_032000	0.000441535	-1.41951	E11.5 * Wnt1 down vs E11.5 * Ctrl
10482500	Rnd3	NM_028810	2.88784e-005	-1.41959	E11.5 * Wnt1 down vs E11.5 * Ctrl
10390186	Abi3	NM_025659	2.01388e-005	-1.42115	E11.5 * Wnt1 down vs E11.5 * Ctrl
10504106	Il11ra1	NM_010549	7.85291e-005	-1.42126	E11.5 * Wnt1 down vs E11.5 * Ctrl
10573924	Mmp2	NM_008610	0.00135389	-1.42183	E11.5 * Wnt1 down vs E11.5 * Ctrl
10449280	EG383229	XM_356935	0.00654421	-1.42214	E11.5 * Wnt1 down vs E11.5 * Ctrl
10466573	Ostf1	NM_017375	0.00178149	-1.42242	E11.5 * Wnt1 down vs E11.5 * Ctrl
10466624	Aldh1a7	NM_011921	0.00781235	-1.42383	E11.5 * Wnt1 down vs E11.5 * Ctrl
10356293	A630001G21Rik	BC052931	0.00107615	-1.42435	E11.5 * Wnt1 down vs E11.5 * Ctrl
10422962	1110020G09Rik	NM_001085410	4.95513e-006	-1.42476	E11.5 * Wnt1 down vs E11.5 * Ctrl
10498647	B3galnt1	NM_020026	6.12769e-007	-1.42544	E11.5 * Wnt1 down vs E11.5 * Ctrl
10507101	OTTMUSG00000	NM_001085549	0.00020329	-1.42641	E11.5 * Wnt1 down vs E11.5 * Ctrl

10435457	Parp9	NM_030253	0.000187205	-1.42648	E11.5 * Wnt1 down vs E11.5 * Ctrl
10461587	Ms4a4a	XM_889011	0.00516102	-1.42683	E11.5 * Wnt1 down vs E11.5 * Ctrl
10469167	Sfmbt2	NM_177386	1.84261e-008	-1.42724	E11.5 * Wnt1 down vs E11.5 * Ctrl
10356968	Pam	NM_013626	0.000285567	-1.42746	E11.5 * Wnt1 down vs E11.5 * Ctrl
10392177	Icam2	NM_010494	8.60902e-005	-1.42766	E11.5 * Wnt1 down vs E11.5 * Ctrl
10475218		---	0.00454075	-1.42785	E11.5 * Wnt1 down vs E11.5 * Ctrl
10606445	Rps6ka6	NM_025949	2.98503e-005	-1.42786	E11.5 * Wnt1 down vs E11.5 * Ctrl
10542885	2810474O19Rik	NM_026054	0.000775758	-1.4284	E11.5 * Wnt1 down vs E11.5 * Ctrl
10599693	Ncrna00086	BC052359	0.00498168	-1.42858	E11.5 * Wnt1 down vs E11.5 * Ctrl
10597074		---	1.39605e-005	-1.42906	E11.5 * Wnt1 down vs E11.5 * Ctrl
10431935	Amigo2	NM_178114	0.00221555	-1.42928	E11.5 * Wnt1 down vs E11.5 * Ctrl
10356333	Snord82	NR_002851	0.00350119	-1.42951	E11.5 * Wnt1 down vs E11.5 * Ctrl
10455123	Pcdhb19	NM_053144	0.00373127	-1.42962	E11.5 * Wnt1 down vs E11.5 * Ctrl
10566333	9230105E10Rik	NM_001146007	8.36597e-006	-1.43042	E11.5 * Wnt1 down vs E11.5 * Ctrl
10538142	Gimap5	NM_175035	0.000226814	-1.43043	E11.5 * Wnt1 down vs E11.5 * Ctrl
10570639	6820431F20Rik	BC025151	0.000254151	-1.4308	E11.5 * Wnt1 down vs E11.5 * Ctrl
10607658	Reps2	NM_178256	0.00126662	-1.43087	E11.5 * Wnt1 down vs E11.5 * Ctrl
10489878	Ptgis	NM_008968	8.72624e-005	-1.43181	E11.5 * Wnt1 down vs E11.5 * Ctrl
10434229	Cldn5	NM_013805	0.000217888	-1.43181	E11.5 * Wnt1 down vs E11.5 * Ctrl
10481592	Dnm1	NM_010065	0.000404377	-1.43201	E11.5 * Wnt1 down vs E11.5 * Ctrl
10455873	Slc12a2	NM_009194	3.84276e-005	-1.43231	E11.5 * Wnt1 down vs E11.5 * Ctrl
10501164	Csf1	NM_007778	0.00794374	-1.43248	E11.5 * Wnt1 down vs E11.5 * Ctrl
10376074	P4ha2	NM_001136076	0.00628765	-1.43364	E11.5 * Wnt1 down vs E11.5 * Ctrl
10445767	Treml2	NM_001033405	0.00157823	-1.43383	E11.5 * Wnt1 down vs E11.5 * Ctrl
10597239	Pth1r	NM_011199	2.40513e-005	-1.43394	E11.5 * Wnt1 down vs E11.5 * Ctrl
10346072		---	0.000132727	-1.43667	E11.5 * Wnt1 down vs E11.5 * Ctrl
10487987	Lrrn4	NM_177303	0.000243305	-1.43728	E11.5 * Wnt1 down vs E11.5 * Ctrl
10544133	Parp12	NM_172893	0.00109841	-1.43755	E11.5 * Wnt1 down vs E11.5 * Ctrl
10353258	4930444P10Rik	ENSMUST00000	0.000533893	-1.4376	E11.5 * Wnt1 down vs E11.5 * Ctrl
10390117	Itga3	NM_013565	0.000297989	-1.43761	E11.5 * Wnt1 down vs E11.5 * Ctrl
10603567	Dynlt3	NM_025975	0.00148726	-1.43822	E11.5 * Wnt1 down vs E11.5 * Ctrl
10558410	Ptpre	NM_011212	0.00106405	-1.43832	E11.5 * Wnt1 down vs E11.5 * Ctrl
10582868	Sp110	NM_175397	0.00023491	-1.43861	E11.5 * Wnt1 down vs E11.5 * Ctrl
10548333	Cd69	NM_001033122	0.00216041	-1.43949	E11.5 * Wnt1 down vs E11.5 * Ctrl
10444028	Kank3	NM_030697	0.000258736	-1.44034	E11.5 * Wnt1 down vs E11.5 * Ctrl
10407420	Net1	NM_019671	0.000454696	-1.44035	E11.5 * Wnt1 down vs E11.5 * Ctrl
10410743	Ankrd32	NM_134071	0.00202172	-1.44071	E11.5 * Wnt1 down vs E11.5 * Ctrl
10514133	Ttc39b	NM_027238	0.000265296	-1.44077	E11.5 * Wnt1 down vs E11.5 * Ctrl
10504137	4933409K07Rik	BC072647	0.00221878	-1.44098	E11.5 * Wnt1 down vs E11.5 * Ctrl
10504201	4933409K07Rik	BC072647	0.00221878	-1.44098	E11.5 * Wnt1 down vs E11.5 * Ctrl
10512350	4933409K07Rik	BC072647	0.00221878	-1.44098	E11.5 * Wnt1 down vs E11.5 * Ctrl
10512352	4933409K07Rik	BC072647	0.00221878	-1.44098	E11.5 * Wnt1 down vs E11.5 * Ctrl
10531952	Abcg3	NM_030239	0.000222636	-1.44127	E11.5 * Wnt1 down vs E11.5 * Ctrl
10361250	Camk1g	NM_144817	0.000105651	-1.4414	E11.5 * Wnt1 down vs E11.5 * Ctrl
10502565	Clca2	NM_030601	0.00180376	-1.44141	E11.5 * Wnt1 down vs E11.5 * Ctrl
10416734		---	0.00606985	-1.44173	E11.5 * Wnt1 down vs E11.5 * Ctrl
10377429	Snord118	X04239	0.0023797	-1.4419	E11.5 * Wnt1 down vs E11.5 * Ctrl
10399428	Snord118	X04239	0.0023797	-1.4419	E11.5 * Wnt1 down vs E11.5 * Ctrl
10604135	Dppa3	NM_139218	0.000148142	-1.4424	E11.5 * Wnt1 down vs E11.5 * Ctrl
10462535	B430203M17Rik	NM_177096	0.00784693	-1.4424	E11.5 * Wnt1 down vs E11.5 * Ctrl
10422598	Sepp1	NM_009155	0.00322081	-1.44275	E11.5 * Wnt1 down vs E11.5 * Ctrl
10598175	Ear10	NM_053112	0.00049388	-1.44325	E11.5 * Wnt1 down vs E11.5 * Ctrl
10536220	Col1a2	NM_007743	1.41461e-005	-1.4435	E11.5 * Wnt1 down vs E11.5 * Ctrl
10471486	Eng	NM_001146350	2.07295e-005	-1.44355	E11.5 * Wnt1 down vs E11.5 * Ctrl
10372618	Frs2	NM_177798	0.000373714	-1.44374	E11.5 * Wnt1 down vs E11.5 * Ctrl
10497862	Trpc3	NM_019510	0.000199349	-1.44421	E11.5 * Wnt1 down vs E11.5 * Ctrl
10497548	Fndc3b	NM_173182	0.000430963	-1.44448	E11.5 * Wnt1 down vs E11.5 * Ctrl
10476740	Slc24a3	NM_053195	0.0015051	-1.4447	E11.5 * Wnt1 down vs E11.5 * Ctrl
10587023	Rab27a	NM_023635	3.28012e-006	-1.44527	E11.5 * Wnt1 down vs E11.5 * Ctrl
10421932	Pcdh9	NM_001081377	0.00571637	-1.44608	E11.5 * Wnt1 down vs E11.5 * Ctrl
10395277	Sypl	NM_013635	5.10604e-006	-1.44625	E11.5 * Wnt1 down vs E11.5 * Ctrl
10467013	Prkg1	NM_001013833	7.26713e-005	-1.44653	E11.5 * Wnt1 down vs E11.5 * Ctrl
10505788	Acer2	BC051923	0.0011406	-1.44664	E11.5 * Wnt1 down vs E11.5 * Ctrl
10385513	9930111J21Rik	NM_173434	0.00187664	-1.44675	E11.5 * Wnt1 down vs E11.5 * Ctrl
10571325	Mfhas1	NM_001081279	1.64919e-006	-1.44682	E11.5 * Wnt1 down vs E11.5 * Ctrl
10505517	Tlr4	NM_021297	0.000139704	-1.44744	E11.5 * Wnt1 down vs E11.5 * Ctrl

10541555	Clec4a1	NM_199311	0.00645402	-1.44787	E11.5 * Wnt1 down vs E11.5 * Ctrl
10356520	Col6a3	AF064749	0.000312839	-1.44875	E11.5 * Wnt1 down vs E11.5 * Ctrl
10554059	ENSMUSG0000C	ENSMUST00000(	0.00344504	-1.4495	E11.5 * Wnt1 down vs E11.5 * Ctrl
10386110	Hand1	NM_008213	7.93937e-005	-1.4495	E11.5 * Wnt1 down vs E11.5 * Ctrl
10433633	Mkl2	NM_181860	0.000787496	-1.45012	E11.5 * Wnt1 down vs E11.5 * Ctrl
10556581	ENSMUSG0000C	ENSMUST00000(	0.00260915	-1.45113	E11.5 * Wnt1 down vs E11.5 * Ctrl
10395733	Npas3	NM_013780	0.00197253	-1.4514	E11.5 * Wnt1 down vs E11.5 * Ctrl
10429402	Gpr20	NM_173365	0.000123922	-1.45168	E11.5 * Wnt1 down vs E11.5 * Ctrl
10435641	Fstl1	NM_008047	2.56071e-005	-1.45185	E11.5 * Wnt1 down vs E11.5 * Ctrl
10462231	Dmrt2	NM_145831	0.00725653	-1.45216	E11.5 * Wnt1 down vs E11.5 * Ctrl
10413047	Plau	NM_008873	0.0044841	-1.45256	E11.5 * Wnt1 down vs E11.5 * Ctrl
10436608	Cxadr	NM_009988	5.35374e-006	-1.45312	E11.5 * Wnt1 down vs E11.5 * Ctrl
10377534	A030009H04Rik	AB041807	2.42612e-005	-1.45345	E11.5 * Wnt1 down vs E11.5 * Ctrl
10402399	Serpina1a	NM_009243	0.00121269	-1.45395	E11.5 * Wnt1 down vs E11.5 * Ctrl
10466224	Ms4a3	NM_133246	0.00559219	-1.45442	E11.5 * Wnt1 down vs E11.5 * Ctrl
10467871	Dnmbp	NM_028029	0.00134471	-1.45445	E11.5 * Wnt1 down vs E11.5 * Ctrl
10404612	Rreb1	NM_001013392	0.000447875	-1.45469	E11.5 * Wnt1 down vs E11.5 * Ctrl
10593198	Fam55b	NM_030069	0.00283016	-1.45533	E11.5 * Wnt1 down vs E11.5 * Ctrl
10384579	Ugp2	NM_139297	3.29368e-005	-1.45571	E11.5 * Wnt1 down vs E11.5 * Ctrl
10542221		---	0.000103461	-1.45583	E11.5 * Wnt1 down vs E11.5 * Ctrl
10366707	Avpr1a	NM_016847	0.000479264	-1.45622	E11.5 * Wnt1 down vs E11.5 * Ctrl
10591092	Fat3	NM_001080814	8.56956e-005	-1.45653	E11.5 * Wnt1 down vs E11.5 * Ctrl
10387743	Slc2a4	NM_009204	4.85073e-005	-1.45685	E11.5 * Wnt1 down vs E11.5 * Ctrl
10531972	5830443L24Rik	NM_029509	0.000737747	-1.45711	E11.5 * Wnt1 down vs E11.5 * Ctrl
10580990	Cdh8	NM_001039154	0.00024305	-1.45809	E11.5 * Wnt1 down vs E11.5 * Ctrl
10387368	Tmem88	NM_025915	4.89429e-005	-1.4582	E11.5 * Wnt1 down vs E11.5 * Ctrl
10363082	Lilrb4	NM_013532	9.28794e-005	-1.45849	E11.5 * Wnt1 down vs E11.5 * Ctrl
10457587	Zfp521	NM_145492	4.02187e-006	-1.45896	E11.5 * Wnt1 down vs E11.5 * Ctrl
10462507	Papss2	NM_011864	8.09486e-005	-1.45902	E11.5 * Wnt1 down vs E11.5 * Ctrl
10346695	Nbeal1	NM_173444	1.30746e-006	-1.45931	E11.5 * Wnt1 down vs E11.5 * Ctrl
10557853	B230325K18Rik	ENSMUST00000(	0.000894197	-1.46008	E11.5 * Wnt1 down vs E11.5 * Ctrl
10395978	Gm527	BC099503	0.000304873	-1.46135	E11.5 * Wnt1 down vs E11.5 * Ctrl
10358272	Lhx9	NM_001042577	1.29318e-005	-1.4621	E11.5 * Wnt1 down vs E11.5 * Ctrl
10512949	Abca1	NM_013454	7.48023e-006	-1.46211	E11.5 * Wnt1 down vs E11.5 * Ctrl
10531166	Adamts3	NM_001081401	0.000229017	-1.46243	E11.5 * Wnt1 down vs E11.5 * Ctrl
10424676	Ly6e	NM_008529	0.00816132	-1.46333	E11.5 * Wnt1 down vs E11.5 * Ctrl
10435525	Iqcb1	NM_177128	0.000110841	-1.46439	E11.5 * Wnt1 down vs E11.5 * Ctrl
10531790	Nkx6-1	NM_144955	1.51886e-005	-1.46675	E11.5 * Wnt1 down vs E11.5 * Ctrl
10437639	Emp2	NM_007929	0.000227549	-1.46764	E11.5 * Wnt1 down vs E11.5 * Ctrl
10376239	Tcp1	AF357392	0.00566481	-1.46832	E11.5 * Wnt1 down vs E11.5 * Ctrl
10545086	Snca	NM_001042451	0.00124095	-1.46853	E11.5 * Wnt1 down vs E11.5 * Ctrl
10543952	9330158H04Rik	ENSMUST00000	0.000676392	-1.4686	E11.5 * Wnt1 down vs E11.5 * Ctrl
10430929	Tbrg3	BC095996	0.00409368	-1.46862	E11.5 * Wnt1 down vs E11.5 * Ctrl
10372844	Rassf3	NM_138956	0.000609816	-1.46893	E11.5 * Wnt1 down vs E11.5 * Ctrl
10485405	Cd44	NM_009851	3.50363e-005	-1.46977	E11.5 * Wnt1 down vs E11.5 * Ctrl
10533462	Rad9b	NM_144912	0.00504483	-1.46994	E11.5 * Wnt1 down vs E11.5 * Ctrl
10402730	Ppp1r13b	NM_011625	0.000424107	-1.47011	E11.5 * Wnt1 down vs E11.5 * Ctrl
10556244	Ipo7	AF357383	0.00055863	-1.47042	E11.5 * Wnt1 down vs E11.5 * Ctrl
10503315	E130016E03Rik	NM_001039556	4.94424e-005	-1.47183	E11.5 * Wnt1 down vs E11.5 * Ctrl
10400510	Clec14a	NM_025809	0.00392395	-1.47231	E11.5 * Wnt1 down vs E11.5 * Ctrl
10519913	Magi2	NM_015823	0.00204149	-1.47236	E11.5 * Wnt1 down vs E11.5 * Ctrl
10411226	F2rl1	NM_007974	0.00152728	-1.47258	E11.5 * Wnt1 down vs E11.5 * Ctrl
10351482	1700015E13Rik	BC099563	4.17433e-005	-1.4733	E11.5 * Wnt1 down vs E11.5 * Ctrl
10448836	Tmem204	NM_001001183	5.3575e-005	-1.47334	E11.5 * Wnt1 down vs E11.5 * Ctrl
10377982	Kif1c	NM_153103	0.0038709	-1.47359	E11.5 * Wnt1 down vs E11.5 * Ctrl
10461856	Gna14	NM_008137	0.00351217	-1.47379	E11.5 * Wnt1 down vs E11.5 * Ctrl
10544815	Hibadh	NM_145567	0.000230389	-1.47383	E11.5 * Wnt1 down vs E11.5 * Ctrl
10402409	Serpina1e	NM_009247	0.000792951	-1.47407	E11.5 * Wnt1 down vs E11.5 * Ctrl
10462281	Vldlr	NM_013703	0.000495077	-1.4742	E11.5 * Wnt1 down vs E11.5 * Ctrl
10485597	Depdc7	NM_144804	0.00250845	-1.47522	E11.5 * Wnt1 down vs E11.5 * Ctrl
10406672	Arsb	NM_009712	0.000173983	-1.47579	E11.5 * Wnt1 down vs E11.5 * Ctrl
10346564	Casp8	NM_009812	0.00169223	-1.4758	E11.5 * Wnt1 down vs E11.5 * Ctrl
10555438	Fchsd2	NM_199012	2.38306e-007	-1.47661	E11.5 * Wnt1 down vs E11.5 * Ctrl
10538547	Fkbp9	NM_012056	1.84964e-005	-1.47694	E11.5 * Wnt1 down vs E11.5 * Ctrl
10366746	Lrig3	NM_177152	0.00247127	-1.47704	E11.5 * Wnt1 down vs E11.5 * Ctrl
10389214	Ccl9	NM_011338	0.000233301	-1.47731	E11.5 * Wnt1 down vs E11.5 * Ctrl

10417167	Clybl	NM_029556	0.000105709	-1.47774	E11.5 * Wnt1 down vs E11.5 * Ctrl
10493449	Thbs3	NM_013691	1.03967e-006	-1.47808	E11.5 * Wnt1 down vs E11.5 * Ctrl
10359419		---	0.00718244	-1.47874	E11.5 * Wnt1 down vs E11.5 * Ctrl
10361509	Syne1	NM_001079686	6.40579e-006	-1.47884	E11.5 * Wnt1 down vs E11.5 * Ctrl
10538848	OTTMUSG00000	XR_032708	0.000210028	-1.47946	E11.5 * Wnt1 down vs E11.5 * Ctrl
10544827		---	0.00111152	-1.48072	E11.5 * Wnt1 down vs E11.5 * Ctrl
10493382	Pklr	NM_013631	0.000134166	-1.48153	E11.5 * Wnt1 down vs E11.5 * Ctrl
10352576	Esrrg	NM_011935	0.000168612	-1.48255	E11.5 * Wnt1 down vs E11.5 * Ctrl
10607924	Amelx	NM_001081978	0.000167127	-1.48292	E11.5 * Wnt1 down vs E11.5 * Ctrl
10413100	Myst4	NM_017479	3.6745e-007	-1.48424	E11.5 * Wnt1 down vs E11.5 * Ctrl
10437160	Ets2	NM_011809	2.73686e-005	-1.4845	E11.5 * Wnt1 down vs E11.5 * Ctrl
10361270	Cd46	NM_010778	0.00432229	-1.48525	E11.5 * Wnt1 down vs E11.5 * Ctrl
10416689	Olfm4	NM_001030294	0.00159244	-1.4856	E11.5 * Wnt1 down vs E11.5 * Ctrl
10381006	Thra	NM_178060	0.000865602	-1.48605	E11.5 * Wnt1 down vs E11.5 * Ctrl
10510129	Dhrs3	NM_011303	0.000581477	-1.48649	E11.5 * Wnt1 down vs E11.5 * Ctrl
10354649	Pgap1	ENSMUST00000	0.00300088	-1.48656	E11.5 * Wnt1 down vs E11.5 * Ctrl
10477069	Tcf15	NM_009328	0.000171354	-1.48759	E11.5 * Wnt1 down vs E11.5 * Ctrl
10542181	Clec9a	NM_172732	0.000724165	-1.48818	E11.5 * Wnt1 down vs E11.5 * Ctrl
10486119	Plcb2	NM_177568	0.000162016	-1.48858	E11.5 * Wnt1 down vs E11.5 * Ctrl
10416653	Kbtbd7	NM_001024135	0.00332759	-1.48958	E11.5 * Wnt1 down vs E11.5 * Ctrl
10600093	Zfp185	NM_009549	6.12611e-006	-1.48993	E11.5 * Wnt1 down vs E11.5 * Ctrl
10391332	Ptrf	NM_008986	6.98711e-005	-1.49073	E11.5 * Wnt1 down vs E11.5 * Ctrl
10396950		---	0.000517681	-1.49087	E11.5 * Wnt1 down vs E11.5 * Ctrl
10603417	Gata1	NM_008089	2.10024e-006	-1.49118	E11.5 * Wnt1 down vs E11.5 * Ctrl
10384486	Etaa1	NM_026576	0.000472292	-1.49215	E11.5 * Wnt1 down vs E11.5 * Ctrl
10479294		---	0.00165988	-1.49228	E11.5 * Wnt1 down vs E11.5 * Ctrl
10419559	Rnase12	NM_001011875	0.000385953	-1.49295	E11.5 * Wnt1 down vs E11.5 * Ctrl
10482866	Tanc1	NM_198294	3.19748e-005	-1.49386	E11.5 * Wnt1 down vs E11.5 * Ctrl
10592816	Hmbs	NM_013551	1.51207e-005	-1.49514	E11.5 * Wnt1 down vs E11.5 * Ctrl
10385883	Slc22a21	NM_019723	0.000866867	-1.49604	E11.5 * Wnt1 down vs E11.5 * Ctrl
10501608	Vcam1	NM_011693	0.000103616	-1.4964	E11.5 * Wnt1 down vs E11.5 * Ctrl
10512919	Grin3a	NM_001033351	3.35833e-005	-1.4964	E11.5 * Wnt1 down vs E11.5 * Ctrl
10408861	9530008L14Rik	NM_175417	0.000228156	-1.49646	E11.5 * Wnt1 down vs E11.5 * Ctrl
10484999	Ddb2	NM_028119	2.00124e-006	-1.4965	E11.5 * Wnt1 down vs E11.5 * Ctrl
10566934	Lyve1	NM_053247	0.000438861	-1.49701	E11.5 * Wnt1 down vs E11.5 * Ctrl
10577164	Gas6	NM_019521	0.00204744	-1.49757	E11.5 * Wnt1 down vs E11.5 * Ctrl
10453676		---	0.00095464	-1.49836	E11.5 * Wnt1 down vs E11.5 * Ctrl
10406334	Mctp1	NM_030174	1.87119e-006	-1.49842	E11.5 * Wnt1 down vs E11.5 * Ctrl
10439710	Phldb2	NM_153412	1.12104e-007	-1.49872	E11.5 * Wnt1 down vs E11.5 * Ctrl
10448117	Has1	NM_008215	0.000816981	-1.49949	E11.5 * Wnt1 down vs E11.5 * Ctrl
10503376	OTTMUSG00000	DQ351292	0.00258627	-1.49949	E11.5 * Wnt1 down vs E11.5 * Ctrl
10432636	BC004728	NM_174992	0.000391197	-1.49974	E11.5 * Wnt1 down vs E11.5 * Ctrl
10380135		---	0.00126835	-1.5003	E11.5 * Wnt1 down vs E11.5 * Ctrl
10573451	Syce2	NM_027954	9.57833e-008	-1.50034	E11.5 * Wnt1 down vs E11.5 * Ctrl
10472970	Hoxd4	NM_010469	7.15931e-005	-1.50069	E11.5 * Wnt1 down vs E11.5 * Ctrl
10587659	EG547109	NM_001034906	0.00195208	-1.50085	E11.5 * Wnt1 down vs E11.5 * Ctrl
10370037	Mmp11	NM_008606	0.000396749	-1.50168	E11.5 * Wnt1 down vs E11.5 * Ctrl
10542470	Mgst1	NM_019946	0.00117386	-1.50169	E11.5 * Wnt1 down vs E11.5 * Ctrl
10500272	Gm129	NM_001033302	0.00353618	-1.50199	E11.5 * Wnt1 down vs E11.5 * Ctrl
10498379		---	0.00159749	-1.50203	E11.5 * Wnt1 down vs E11.5 * Ctrl
10584589		---	0.00034333	-1.50269	E11.5 * Wnt1 down vs E11.5 * Ctrl
10470283	Egfl7	NM_198724	4.6885e-005	-1.50274	E11.5 * Wnt1 down vs E11.5 * Ctrl
10410756	Ankrd32	NM_134071	0.00108168	-1.50302	E11.5 * Wnt1 down vs E11.5 * Ctrl
10598004	Ccr1	NM_009912	0.000881545	-1.50381	E11.5 * Wnt1 down vs E11.5 * Ctrl
10579812	Ednra	NM_010332	7.52346e-005	-1.5043	E11.5 * Wnt1 down vs E11.5 * Ctrl
10575976	Crispld2	NM_030209	0.000196231	-1.50463	E11.5 * Wnt1 down vs E11.5 * Ctrl
10443027	A930001N09Rik	NM_029870	1.05297e-005	-1.50631	E11.5 * Wnt1 down vs E11.5 * Ctrl
10369750	ENSMUSG00000	ENSMUST00000	0.0077216	-1.5064	E11.5 * Wnt1 down vs E11.5 * Ctrl
10354418	Obfc2a	NM_028696	0.00211077	-1.5071	E11.5 * Wnt1 down vs E11.5 * Ctrl
10410973		---	1.74136e-005	-1.50767	E11.5 * Wnt1 down vs E11.5 * Ctrl
10372139	Nts	NM_024435	0.000323752	-1.50799	E11.5 * Wnt1 down vs E11.5 * Ctrl
10535841	Slc46a3	NM_027872	0.0025431	-1.50851	E11.5 * Wnt1 down vs E11.5 * Ctrl
10530145	Tlr1	NM_030682	0.00202636	-1.5091	E11.5 * Wnt1 down vs E11.5 * Ctrl
10498383	Igsf10	ENSMUST00000	0.000607003	-1.50951	E11.5 * Wnt1 down vs E11.5 * Ctrl
10547769	Ptpn6	NM_013545	8.54429e-006	-1.51007	E11.5 * Wnt1 down vs E11.5 * Ctrl
10420216	Sdr39u1	NM_001082975	0.00108126	-1.51053	E11.5 * Wnt1 down vs E11.5 * Ctrl

10508663	Laptm5	NM_010686	0.00546738	-1.51061	E11.5 * Wnt1 down vs E11.5 * Ctrl
10542164	Clec12a	NM_177686	0.000186383	-1.51112	E11.5 * Wnt1 down vs E11.5 * Ctrl
10367945	Phactr2	NM_001033257	0.00220877	-1.51163	E11.5 * Wnt1 down vs E11.5 * Ctrl
10588786	Ube1l	NM_023738	3.54446e-005	-1.51245	E11.5 * Wnt1 down vs E11.5 * Ctrl
10402606	Rtl1	NM_184109	4.79044e-005	-1.5126	E11.5 * Wnt1 down vs E11.5 * Ctrl
10428511	Csmd3	NM_001081391	0.00217016	-1.51292	E11.5 * Wnt1 down vs E11.5 * Ctrl
10544002	Creb3l2	NM_178661	3.00384e-006	-1.51349	E11.5 * Wnt1 down vs E11.5 * Ctrl
10492402	Kcnab1	NM_010597	2.09191e-005	-1.51374	E11.5 * Wnt1 down vs E11.5 * Ctrl
10604956		---	0.000401801	-1.51396	E11.5 * Wnt1 down vs E11.5 * Ctrl
10604542	Hs6st2	NM_001077202	2.42605e-005	-1.51511	E11.5 * Wnt1 down vs E11.5 * Ctrl
10409282	Ror2	NM_013846	6.26809e-005	-1.51564	E11.5 * Wnt1 down vs E11.5 * Ctrl
10362615	Traf3ip2	NM_134000	8.59725e-006	-1.51627	E11.5 * Wnt1 down vs E11.5 * Ctrl
10549282	Itpr2	NM_019923	8.04744e-007	-1.51735	E11.5 * Wnt1 down vs E11.5 * Ctrl
10409994	EG435366	ENSMUST00000	0.00283557	-1.51821	E11.5 * Wnt1 down vs E11.5 * Ctrl
10447848	Mas1	NM_008552	0.000477846	-1.5198	E11.5 * Wnt1 down vs E11.5 * Ctrl
10419156	Ear10	NM_053112	8.94533e-005	-1.51996	E11.5 * Wnt1 down vs E11.5 * Ctrl
10390299	Pnpo	NM_134021	0.000155898	-1.52009	E11.5 * Wnt1 down vs E11.5 * Ctrl
10482301	A930041I02Rik	NM_178778	5.66758e-005	-1.5207	E11.5 * Wnt1 down vs E11.5 * Ctrl
10458661		---	2.59972e-005	-1.52077	E11.5 * Wnt1 down vs E11.5 * Ctrl
10395869	4921506M07Rik	BC100489	0.00601348	-1.52186	E11.5 * Wnt1 down vs E11.5 * Ctrl
10385500	Irgm1	NM_008326	7.24057e-005	-1.52544	E11.5 * Wnt1 down vs E11.5 * Ctrl
10523727	Pkd2	NM_008861	1.51539e-006	-1.52583	E11.5 * Wnt1 down vs E11.5 * Ctrl
10439268	Dtx3l	NM_001013371	0.000378185	-1.52645	E11.5 * Wnt1 down vs E11.5 * Ctrl
10452980	Eif2ak2	NM_011163	0.00100292	-1.52661	E11.5 * Wnt1 down vs E11.5 * Ctrl
10584325	Vsig2	NM_020518	2.04574e-005	-1.52663	E11.5 * Wnt1 down vs E11.5 * Ctrl
10380419	Col1a1	NM_007742	3.49493e-007	-1.52688	E11.5 * Wnt1 down vs E11.5 * Ctrl
10357137	Gli2	NM_001081125	7.16883e-005	-1.52722	E11.5 * Wnt1 down vs E11.5 * Ctrl
10531610	Rasgef1b	NM_145839	0.00140986	-1.52737	E11.5 * Wnt1 down vs E11.5 * Ctrl
10576911	Efnb2	NM_010111	0.000102057	-1.52792	E11.5 * Wnt1 down vs E11.5 * Ctrl
10366196	Ppfia2	NM_177373	2.95753e-005	-1.52836	E11.5 * Wnt1 down vs E11.5 * Ctrl
10401149	Plek2	NM_013738	0.000127925	-1.52871	E11.5 * Wnt1 down vs E11.5 * Ctrl
10380629	Hoxb8	NM_010461	2.83742e-005	-1.52905	E11.5 * Wnt1 down vs E11.5 * Ctrl
10362811	Sesn1	NM_001013370	1.23629e-005	-1.5292	E11.5 * Wnt1 down vs E11.5 * Ctrl
10359970	Nos1ap	NM_001109985	2.42663e-005	-1.52925	E11.5 * Wnt1 down vs E11.5 * Ctrl
10590686	Ddi1	NM_027942	0.00139247	-1.53071	E11.5 * Wnt1 down vs E11.5 * Ctrl
10554156	Fam174b	ENSMUST00000	2.49396e-005	-1.53181	E11.5 * Wnt1 down vs E11.5 * Ctrl
10562651	C330019L16Rik	NM_001034857	0.00269741	-1.53277	E11.5 * Wnt1 down vs E11.5 * Ctrl
10382435	Gprc5c	NM_001110337	3.03193e-005	-1.53304	E11.5 * Wnt1 down vs E11.5 * Ctrl
10490777	Zfhx4	NM_030708	1.08447e-006	-1.53362	E11.5 * Wnt1 down vs E11.5 * Ctrl
10379511	Ccl2	NM_011333	5.56291e-006	-1.53365	E11.5 * Wnt1 down vs E11.5 * Ctrl
10420747	Gata4	NM_008092	9.23571e-005	-1.53461	E11.5 * Wnt1 down vs E11.5 * Ctrl
10398907	Pld4	NM_178911	3.11375e-005	-1.53472	E11.5 * Wnt1 down vs E11.5 * Ctrl
10385872	Slc22a5	NM_011396	0.000250133	-1.53526	E11.5 * Wnt1 down vs E11.5 * Ctrl
10598586	Xk	NM_023500	7.86195e-005	-1.5355	E11.5 * Wnt1 down vs E11.5 * Ctrl
10347291	Il8rb	NM_009909	0.000322073	-1.53588	E11.5 * Wnt1 down vs E11.5 * Ctrl
10497337	Car1	NM_009799	0.00152124	-1.53591	E11.5 * Wnt1 down vs E11.5 * Ctrl
10503241	Gdf6	NM_013526	0.00243062	-1.53607	E11.5 * Wnt1 down vs E11.5 * Ctrl
10369132	Fam184a	NM_001081428	3.01151e-006	-1.53662	E11.5 * Wnt1 down vs E11.5 * Ctrl
10362138	Vnn1	NM_011704	0.000289298	-1.53664	E11.5 * Wnt1 down vs E11.5 * Ctrl
10436519	Robo1	NM_019413	3.83214e-006	-1.53787	E11.5 * Wnt1 down vs E11.5 * Ctrl
10435841	Ccdc52	NM_144550	7.91542e-005	-1.53816	E11.5 * Wnt1 down vs E11.5 * Ctrl
10553450	Nell1	NM_001037906	0.00101461	-1.5393	E11.5 * Wnt1 down vs E11.5 * Ctrl
10576581	Kcnk1	NM_008430	0.00110758	-1.53954	E11.5 * Wnt1 down vs E11.5 * Ctrl
10519484	Steap2	AK141701	0.00176918	-1.53984	E11.5 * Wnt1 down vs E11.5 * Ctrl
10405733	EG630579	NM_001039239	0.000815488	-1.5399	E11.5 * Wnt1 down vs E11.5 * Ctrl
10514421	Lrrc19	NM_175305	0.00073617	-1.53993	E11.5 * Wnt1 down vs E11.5 * Ctrl
10526693	Zcwpw1	NM_001005426	0.000836237	-1.54026	E11.5 * Wnt1 down vs E11.5 * Ctrl
10396778	Mpp5	NM_019579	9.98821e-007	-1.54076	E11.5 * Wnt1 down vs E11.5 * Ctrl
10591164		---	0.00102333	-1.54116	E11.5 * Wnt1 down vs E11.5 * Ctrl
10463911	Add3	NM_013758	1.15387e-005	-1.54137	E11.5 * Wnt1 down vs E11.5 * Ctrl
10463404	Fam178a	NM_001081225	2.07638e-007	-1.54161	E11.5 * Wnt1 down vs E11.5 * Ctrl
10407097	Pde4d	NM_011056	4.32807e-009	-1.54172	E11.5 * Wnt1 down vs E11.5 * Ctrl
10583870	Bmper	NM_028472	2.28276e-005	-1.54255	E11.5 * Wnt1 down vs E11.5 * Ctrl
10447799	Igf2r	NM_010515	4.01665e-008	-1.54364	E11.5 * Wnt1 down vs E11.5 * Ctrl
10554800	Rab38	NM_028238	6.81368e-008	-1.54376	E11.5 * Wnt1 down vs E11.5 * Ctrl
10472846	Pdk1	NM_172665	0.00624819	-1.5442	E11.5 * Wnt1 down vs E11.5 * Ctrl

10541564	Clec4a3	NM_153197	0.000206705	-1.54484	E11.5 * Wnt1 down vs E11.5 * Ctrl
10575052	Cdh1	NM_009864	0.00191726	-1.54498	E11.5 * Wnt1 down vs E11.5 * Ctrl
10428124	Rgs22	ENSMUST00000	0.000204188	-1.54539	E11.5 * Wnt1 down vs E11.5 * Ctrl
10594840	Gcom1	NM_001033208	0.000177276	-1.54554	E11.5 * Wnt1 down vs E11.5 * Ctrl
10443949	Adamts10	NM_172619	0.000218484	-1.54585	E11.5 * Wnt1 down vs E11.5 * Ctrl
10363070	Gp49a	NM_008147	0.00112074	-1.54588	E11.5 * Wnt1 down vs E11.5 * Ctrl
10472240	Tanc1	NM_198294	5.53324e-007	-1.54591	E11.5 * Wnt1 down vs E11.5 * Ctrl
10527940	Pftk1	NM_011074	2.05505e-005	-1.54612	E11.5 * Wnt1 down vs E11.5 * Ctrl
10492957	Cd1d2	NM_007640	1.00396e-005	-1.54643	E11.5 * Wnt1 down vs E11.5 * Ctrl
10519815	Cacna2d1	NM_001110843	0.000106921	-1.54702	E11.5 * Wnt1 down vs E11.5 * Ctrl
10365991	Epyc	NM_007884	6.37445e-005	-1.54729	E11.5 * Wnt1 down vs E11.5 * Ctrl
10398326	Meg3	NR_003633	0.000627204	-1.54794	E11.5 * Wnt1 down vs E11.5 * Ctrl
10397575	Nrxn3	NM_172544	3.15866e-005	-1.54873	E11.5 * Wnt1 down vs E11.5 * Ctrl
10456329	5330437I02Rik	NM_177028	0.000181705	-1.54905	E11.5 * Wnt1 down vs E11.5 * Ctrl
10460968	Rasgrp2	NM_011242	1.24286e-006	-1.55005	E11.5 * Wnt1 down vs E11.5 * Ctrl
10354647	Pgap1	ENSMUST00000	0.000707439	-1.55043	E11.5 * Wnt1 down vs E11.5 * Ctrl
10475532	Sqrdl	NM_021507	0.00468206	-1.55094	E11.5 * Wnt1 down vs E11.5 * Ctrl
10578829	Palld	NM_001081390	0.00183677	-1.55425	E11.5 * Wnt1 down vs E11.5 * Ctrl
10534679	Trim56	NM_201373	0.00450526	-1.55461	E11.5 * Wnt1 down vs E11.5 * Ctrl
10584334	Siae	NM_011734	0.000557194	-1.55499	E11.5 * Wnt1 down vs E11.5 * Ctrl
10471929	Arhgap15	NM_153820	0.00153734	-1.55519	E11.5 * Wnt1 down vs E11.5 * Ctrl
10469505	Commd3	NM_147778	0.000129199	-1.55566	E11.5 * Wnt1 down vs E11.5 * Ctrl
10403871	Aoah	NM_012054	3.65195e-005	-1.55585	E11.5 * Wnt1 down vs E11.5 * Ctrl
10409059		---	0.000416922	-1.55587	E11.5 * Wnt1 down vs E11.5 * Ctrl
10560618	Apoc1	NM_007469	0.000178525	-1.55617	E11.5 * Wnt1 down vs E11.5 * Ctrl
10423024	Capsl	NM_029341	0.000676221	-1.55638	E11.5 * Wnt1 down vs E11.5 * Ctrl
10440344	Robo2	NM_175549	0.000199149	-1.55678	E11.5 * Wnt1 down vs E11.5 * Ctrl
10441093	Erg	NM_133659	7.84795e-007	-1.55693	E11.5 * Wnt1 down vs E11.5 * Ctrl
10397633	Flrt2	NM_201518	0.00012585	-1.55753	E11.5 * Wnt1 down vs E11.5 * Ctrl
10439612	Boc	NM_172506	5.45269e-006	-1.55799	E11.5 * Wnt1 down vs E11.5 * Ctrl
10394860	B230354O11Rik	AK046228	0.00179028	-1.55824	E11.5 * Wnt1 down vs E11.5 * Ctrl
10546452	Adamts9	NM_175314	4.11507e-007	-1.55871	E11.5 * Wnt1 down vs E11.5 * Ctrl
10499113	Gm1019	NM_001001650	7.07003e-006	-1.55889	E11.5 * Wnt1 down vs E11.5 * Ctrl
10436196		---	6.79553e-005	-1.55894	E11.5 * Wnt1 down vs E11.5 * Ctrl
10398428		---	7.34049e-005	-1.55935	E11.5 * Wnt1 down vs E11.5 * Ctrl
10550388	Gng8	NM_010320	0.00146605	-1.55946	E11.5 * Wnt1 down vs E11.5 * Ctrl
10497051	Negr1	NM_001039094	0.000775166	-1.55999	E11.5 * Wnt1 down vs E11.5 * Ctrl
10538706	Mmrn1	BC137623	0.00332202	-1.56007	E11.5 * Wnt1 down vs E11.5 * Ctrl
10354588	Stk17b	NM_133810	0.00297049	-1.56106	E11.5 * Wnt1 down vs E11.5 * Ctrl
10538802	A930038C07Rik	NM_172399	0.000606058	-1.56109	E11.5 * Wnt1 down vs E11.5 * Ctrl
10368373	C030003D03Rik	NM_029881	0.000100395	-1.56218	E11.5 * Wnt1 down vs E11.5 * Ctrl
10531185	Adamts3	NM_001081401	2.59172e-005	-1.56271	E11.5 * Wnt1 down vs E11.5 * Ctrl
10376245	Gria1	NM_001113325	1.51719e-006	-1.56285	E11.5 * Wnt1 down vs E11.5 * Ctrl
10457077	Fbxo15	NM_015798	0.00095521	-1.56311	E11.5 * Wnt1 down vs E11.5 * Ctrl
10547100	Plxnd1	NM_026376	0.000537218	-1.56352	E11.5 * Wnt1 down vs E11.5 * Ctrl
10366546	Cpm	NM_027468	0.000617352	-1.56356	E11.5 * Wnt1 down vs E11.5 * Ctrl
10559558	Tnni3	NM_009406	7.75227e-006	-1.5644	E11.5 * Wnt1 down vs E11.5 * Ctrl
10483110	Ifih1	NM_027835	5.85663e-005	-1.56472	E11.5 * Wnt1 down vs E11.5 * Ctrl
10397975	Ifi27l1	NM_026790	3.81865e-005	-1.56585	E11.5 * Wnt1 down vs E11.5 * Ctrl
10442069	Lix1	NM_025681	0.00405791	-1.56587	E11.5 * Wnt1 down vs E11.5 * Ctrl
10607943		---	0.000171606	-1.56613	E11.5 * Wnt1 down vs E11.5 * Ctrl
10369702	Tet1	ENSMUST00000	2.88837e-006	-1.56662	E11.5 * Wnt1 down vs E11.5 * Ctrl
10424543	Wisp1	NM_018865	0.000314275	-1.56665	E11.5 * Wnt1 down vs E11.5 * Ctrl
10546010	Arhgap25	NM_001037727	0.00152368	-1.56694	E11.5 * Wnt1 down vs E11.5 * Ctrl
10466530	Pcsk5	BC013068	1.08678e-005	-1.56739	E11.5 * Wnt1 down vs E11.5 * Ctrl
10514219	Scarna8	AF357402	0.00300548	-1.56766	E11.5 * Wnt1 down vs E11.5 * Ctrl
10584317	Esam	NM_027102	5.84831e-005	-1.56791	E11.5 * Wnt1 down vs E11.5 * Ctrl
10520612	Khk	NM_008439	0.000150486	-1.56831	E11.5 * Wnt1 down vs E11.5 * Ctrl
10371379	Nuak1	NM_001004363	8.95933e-005	-1.56836	E11.5 * Wnt1 down vs E11.5 * Ctrl
10462140	Dock8	NM_028785	0.000386135	-1.56913	E11.5 * Wnt1 down vs E11.5 * Ctrl
10513208	Svep1	NM_022814	3.67033e-006	-1.56997	E11.5 * Wnt1 down vs E11.5 * Ctrl
10352448	Dusp10	NM_022019	0.000184919	-1.57017	E11.5 * Wnt1 down vs E11.5 * Ctrl
10454601	Tslp	NM_021367	1.5979e-005	-1.57023	E11.5 * Wnt1 down vs E11.5 * Ctrl
10463410	Fam178a	NM_001081225	0.000147303	-1.57068	E11.5 * Wnt1 down vs E11.5 * Ctrl
10454192	Ttr	NM_013697	0.00209126	-1.57148	E11.5 * Wnt1 down vs E11.5 * Ctrl
10409978	EG630579	NM_001039239	0.000317185	-1.57237	E11.5 * Wnt1 down vs E11.5 * Ctrl

10431749	Adamts20	NM_177431	3.23289e-005	-1.57435	E11.5 * Wnt1 down vs E11.5 * Ctrl
10375360	Ebf1	NM_007897	0.00246342	-1.57475	E11.5 * Wnt1 down vs E11.5 * Ctrl
10467068	Sgms1	NM_144792	1.917e-005	-1.57566	E11.5 * Wnt1 down vs E11.5 * Ctrl
10495206	Slc16a4	NM_146136	6.46347e-007	-1.5762	E11.5 * Wnt1 down vs E11.5 * Ctrl
10425066	Csf2rb	NM_007780	5.26259e-005	-1.57625	E11.5 * Wnt1 down vs E11.5 * Ctrl
10404059	Hist1h1c	NM_015786	0.000114438	-1.57666	E11.5 * Wnt1 down vs E11.5 * Ctrl
10607486	Ptchd1	NM_001093750	3.11222e-007	-1.57765	E11.5 * Wnt1 down vs E11.5 * Ctrl
10408812	Mak	NM_001145803	0.000409705	-1.57836	E11.5 * Wnt1 down vs E11.5 * Ctrl
10463476	Kazald1	NM_178929	0.0001856	-1.57893	E11.5 * Wnt1 down vs E11.5 * Ctrl
10563085	Fcgrt	NM_010189	7.24711e-008	-1.57919	E11.5 * Wnt1 down vs E11.5 * Ctrl
10388902	Lgals9	NM_010708	2.46392e-005	-1.57939	E11.5 * Wnt1 down vs E11.5 * Ctrl
10407591	Chrm3	NM_033269	0.000423273	-1.57943	E11.5 * Wnt1 down vs E11.5 * Ctrl
10400483	Slc25a21	NM_172577	1.51085e-005	-1.57999	E11.5 * Wnt1 down vs E11.5 * Ctrl
10437236	B230307C23Rik	ENSMUST00000	0.00618109	-1.58045	E11.5 * Wnt1 down vs E11.5 * Ctrl
10404885	Gmpr	NM_025508	7.71925e-007	-1.58078	E11.5 * Wnt1 down vs E11.5 * Ctrl
10430993	1700001L05Rik	ENSMUST00000	0.000579907	-1.58113	E11.5 * Wnt1 down vs E11.5 * Ctrl
10553788	Atp10a	NM_009728	3.88827e-007	-1.58238	E11.5 * Wnt1 down vs E11.5 * Ctrl
10604564	Gpc4	NM_008150	0.00011125	-1.58269	E11.5 * Wnt1 down vs E11.5 * Ctrl
10501222	Gstm2	NM_008183	0.000933598	-1.58321	E11.5 * Wnt1 down vs E11.5 * Ctrl
10584208	Cdon	NM_021339	9.37183e-008	-1.58335	E11.5 * Wnt1 down vs E11.5 * Ctrl
10542993	Pon3	NM_173006	4.32466e-006	-1.58508	E11.5 * Wnt1 down vs E11.5 * Ctrl
10515981		---	0.00468238	-1.58576	E11.5 * Wnt1 down vs E11.5 * Ctrl
10495830	Sec24d	NM_027135	4.91038e-005	-1.58602	E11.5 * Wnt1 down vs E11.5 * Ctrl
10530692	Kdr	NM_010612	4.25898e-005	-1.58652	E11.5 * Wnt1 down vs E11.5 * Ctrl
10363860	Slc16a9	NM_025807	0.000952778	-1.58746	E11.5 * Wnt1 down vs E11.5 * Ctrl
10606658	Trmt2b	NM_172540	0.00081081	-1.58763	E11.5 * Wnt1 down vs E11.5 * Ctrl
10496251	Bdh2	NM_027208	1.78455e-005	-1.58924	E11.5 * Wnt1 down vs E11.5 * Ctrl
10601270	ENSMUSG0000C	ENSMUST00000	0.000186424	-1.58934	E11.5 * Wnt1 down vs E11.5 * Ctrl
10497381	Cyp7b1	NM_007825	1.30905e-005	-1.58952	E11.5 * Wnt1 down vs E11.5 * Ctrl
10358389	Rgs2	NM_009061	0.000115849	-1.59023	E11.5 * Wnt1 down vs E11.5 * Ctrl
10401607	Pgf	NM_008827	0.000586654	-1.59059	E11.5 * Wnt1 down vs E11.5 * Ctrl
10455108	Pcdhb16	NM_053141	0.00724824	-1.59087	E11.5 * Wnt1 down vs E11.5 * Ctrl
10576051	Foxc2	NM_013519	0.00767316	-1.59145	E11.5 * Wnt1 down vs E11.5 * Ctrl
10561212	Ltbp4	NM_175641	3.71148e-005	-1.59155	E11.5 * Wnt1 down vs E11.5 * Ctrl
10361790	Fuca2	NM_025799	4.51152e-005	-1.59172	E11.5 * Wnt1 down vs E11.5 * Ctrl
10517517	C1qa	NM_007572	2.29305e-005	-1.59254	E11.5 * Wnt1 down vs E11.5 * Ctrl
10554521	Pde8a	NM_008803	0.000108355	-1.59268	E11.5 * Wnt1 down vs E11.5 * Ctrl
10571530	Fat1	NM_001081286	3.76774e-007	-1.59297	E11.5 * Wnt1 down vs E11.5 * Ctrl
10416230	Tnfrsf10b	NM_020275	0.00228933	-1.59365	E11.5 * Wnt1 down vs E11.5 * Ctrl
10496182	Cxxc4	NM_001004367	5.12986e-006	-1.5942	E11.5 * Wnt1 down vs E11.5 * Ctrl
10535936		---	0.000577977	-1.59462	E11.5 * Wnt1 down vs E11.5 * Ctrl
10455071	Pcdhb7	NM_053132	0.000664682	-1.5948	E11.5 * Wnt1 down vs E11.5 * Ctrl
10600326	Tktl1	NM_031379	0.00364494	-1.59513	E11.5 * Wnt1 down vs E11.5 * Ctrl
10474419	Lgr4	NM_172671	4.17357e-005	-1.5954	E11.5 * Wnt1 down vs E11.5 * Ctrl
10519811	Speer8-ps1	NR_001584	4.28302e-005	-1.59587	E11.5 * Wnt1 down vs E11.5 * Ctrl
10362968	Bves	NM_024285	7.78412e-006	-1.59647	E11.5 * Wnt1 down vs E11.5 * Ctrl
10584315		---	0.00776653	-1.59731	E11.5 * Wnt1 down vs E11.5 * Ctrl
10404294	Prl3b1	NM_008865	1.61156e-006	-1.59738	E11.5 * Wnt1 down vs E11.5 * Ctrl
10406598	Serinc5	NM_172588	0.00124531	-1.59848	E11.5 * Wnt1 down vs E11.5 * Ctrl
10591114	Fat3	NM_001080814	0.0026465	-1.59945	E11.5 * Wnt1 down vs E11.5 * Ctrl
10505717	Adamtsl1	NM_029967	0.000628354	-1.59947	E11.5 * Wnt1 down vs E11.5 * Ctrl
10404982	Barx1	NM_007526	0.000813521	-1.60075	E11.5 * Wnt1 down vs E11.5 * Ctrl
10366266	Pawr	NM_054056	0.00283412	-1.60116	E11.5 * Wnt1 down vs E11.5 * Ctrl
10470562	ENSMUSG0000C	ENSMUST00000(	0.00154291	-1.6017	E11.5 * Wnt1 down vs E11.5 * Ctrl
10479397	Ntsr1	NM_018766	0.000610734	-1.60252	E11.5 * Wnt1 down vs E11.5 * Ctrl
10531189	Adamts3	NM_001081401	0.000996739	-1.60284	E11.5 * Wnt1 down vs E11.5 * Ctrl
10463875	Sorcs3	NM_025696	4.33728e-005	-1.60337	E11.5 * Wnt1 down vs E11.5 * Ctrl
10554969	Odz4	NM_011858	8.4867e-005	-1.60342	E11.5 * Wnt1 down vs E11.5 * Ctrl
10565759	Uvrag	NM_178635	0.000777914	-1.60422	E11.5 * Wnt1 down vs E11.5 * Ctrl
10566366	AI451617	NM_199146	0.00238242	-1.60434	E11.5 * Wnt1 down vs E11.5 * Ctrl
10466248	Stx3	NM_001025307	5.50408e-006	-1.6059	E11.5 * Wnt1 down vs E11.5 * Ctrl
10363090	Rfx6	NM_001159389	1.5173e-005	-1.60676	E11.5 * Wnt1 down vs E11.5 * Ctrl
10353731	4930444G20Rik	NM_053264	0.00558089	-1.60723	E11.5 * Wnt1 down vs E11.5 * Ctrl
10477717	Procr	NM_011171	0.000322461	-1.60841	E11.5 * Wnt1 down vs E11.5 * Ctrl
10422496	Gpr183	NM_183031	1.37228e-005	-1.60862	E11.5 * Wnt1 down vs E11.5 * Ctrl
10604944	Gabre	NM_017369	0.000211514	-1.60878	E11.5 * Wnt1 down vs E11.5 * Ctrl

10423109	Adamts12	NM_175501	3.0762e-007	-1.60917	E11.5 * Wnt1 down vs E11.5 * Ctrl
10599598	A630012P03Rik	ENSMUST00000	2.69445e-005	-1.60951	E11.5 * Wnt1 down vs E11.5 * Ctrl
10458875	Dtwd2	NM_026854	0.00285952	-1.61164	E11.5 * Wnt1 down vs E11.5 * Ctrl
10424082	Aard	NM_175503	0.000290133	-1.612	E11.5 * Wnt1 down vs E11.5 * Ctrl
10407598	Ryr2	NM_023868	4.24317e-008	-1.61292	E11.5 * Wnt1 down vs E11.5 * Ctrl
10554574	Tm6sf1	NM_145375	0.00168608	-1.61295	E11.5 * Wnt1 down vs E11.5 * Ctrl
10555862	Trim34	NM_030684	7.35452e-005	-1.61424	E11.5 * Wnt1 down vs E11.5 * Ctrl
10544801	Hoxa11	NM_010450	2.21965e-005	-1.61457	E11.5 * Wnt1 down vs E11.5 * Ctrl
10459288	Adrb2	NM_007420	0.000575139	-1.61479	E11.5 * Wnt1 down vs E11.5 * Ctrl
10443786	Pde9a	NM_008804	2.32048e-006	-1.61491	E11.5 * Wnt1 down vs E11.5 * Ctrl
10453811	AK220484	NM_001083628	6.21339e-005	-1.61495	E11.5 * Wnt1 down vs E11.5 * Ctrl
10422013	Klf12	NM_010636	4.37814e-005	-1.61556	E11.5 * Wnt1 down vs E11.5 * Ctrl
10430372	Rac2	NM_009008	1.22221e-006	-1.6166	E11.5 * Wnt1 down vs E11.5 * Ctrl
10564237	ENSMUSG0000C	ENSMUST00000	0.000350052	-1.61925	E11.5 * Wnt1 down vs E11.5 * Ctrl
10350516	Ptgs2	NM_011198	0.00655154	-1.61929	E11.5 * Wnt1 down vs E11.5 * Ctrl
10549504	Dennd5b	NM_177192	0.000890764	-1.6196	E11.5 * Wnt1 down vs E11.5 * Ctrl
10401068	Spnb1	NM_013675	3.499e-006	-1.62066	E11.5 * Wnt1 down vs E11.5 * Ctrl
10508012	Rspo1	NM_138683	0.000178154	-1.62096	E11.5 * Wnt1 down vs E11.5 * Ctrl
10569618	Ano1	NM_178642	0.00105766	-1.624	E11.5 * Wnt1 down vs E11.5 * Ctrl
10528385	Reln	NM_011261	2.47619e-006	-1.62438	E11.5 * Wnt1 down vs E11.5 * Ctrl
10359339	Rabgap1l	NM_013862	2.82452e-005	-1.62532	E11.5 * Wnt1 down vs E11.5 * Ctrl
10579894	Hhip	NM_020259	0.00160468	-1.62538	E11.5 * Wnt1 down vs E11.5 * Ctrl
10521616	C1qtnf7	NM_001135172	2.13543e-005	-1.62597	E11.5 * Wnt1 down vs E11.5 * Ctrl
10346607	Fzd7	NM_008057	7.72285e-008	-1.62644	E11.5 * Wnt1 down vs E11.5 * Ctrl
10557033	Eef2k	ENSMUST00000	3.81706e-005	-1.62667	E11.5 * Wnt1 down vs E11.5 * Ctrl
10601848	6530401D17Rik	NR_003641	0.00284348	-1.62672	E11.5 * Wnt1 down vs E11.5 * Ctrl
10376201	Gpx3	NM_001083929	2.33379e-006	-1.6274	E11.5 * Wnt1 down vs E11.5 * Ctrl
10447317	Epas1	NM_010137	0.000510445	-1.62908	E11.5 * Wnt1 down vs E11.5 * Ctrl
10578904	Cpe	NM_013494	9.13375e-008	-1.63053	E11.5 * Wnt1 down vs E11.5 * Ctrl
10462132	E030010A14Rik	NM_183160	1.12694e-006	-1.63095	E11.5 * Wnt1 down vs E11.5 * Ctrl
10383168		---	3.45262e-006	-1.6316	E11.5 * Wnt1 down vs E11.5 * Ctrl
10588819	1700021K14Rik	NM_001122635	0.000228034	-1.63194	E11.5 * Wnt1 down vs E11.5 * Ctrl
10455054	Pcdhb3	NM_053128	0.000811767	-1.632	E11.5 * Wnt1 down vs E11.5 * Ctrl
10408898		---	0.00114285	-1.63277	E11.5 * Wnt1 down vs E11.5 * Ctrl
10469695	Apbb1ip	NM_019456	1.78291e-005	-1.63454	E11.5 * Wnt1 down vs E11.5 * Ctrl
10495416	Vav3	NM_020505	5.13128e-005	-1.6362	E11.5 * Wnt1 down vs E11.5 * Ctrl
10406663	Arsb	NM_009712	0.000572446	-1.63629	E11.5 * Wnt1 down vs E11.5 * Ctrl
10369154	Man1a	NM_008548	0.00112303	-1.63712	E11.5 * Wnt1 down vs E11.5 * Ctrl
10414102	Mmrn2	NM_153127	0.000699116	-1.6375	E11.5 * Wnt1 down vs E11.5 * Ctrl
10536494	Cav2	NM_016900	2.28786e-005	-1.63863	E11.5 * Wnt1 down vs E11.5 * Ctrl
10353729	4930444G20Rik	NM_053264	0.00658781	-1.63887	E11.5 * Wnt1 down vs E11.5 * Ctrl
10433782	Efcab1	NM_025769	2.47309e-005	-1.63904	E11.5 * Wnt1 down vs E11.5 * Ctrl
10422844	Gdnf	NM_010275	1.31747e-005	-1.64056	E11.5 * Wnt1 down vs E11.5 * Ctrl
10383765		---	0.00608103	-1.64065	E11.5 * Wnt1 down vs E11.5 * Ctrl
10359929	Ddr2	NM_022563	0.000110354	-1.64339	E11.5 * Wnt1 down vs E11.5 * Ctrl
10455139	Pcdhb22	NM_053147	0.000117256	-1.64457	E11.5 * Wnt1 down vs E11.5 * Ctrl
10436182	Cd47	NM_010581	7.45346e-006	-1.64507	E11.5 * Wnt1 down vs E11.5 * Ctrl
10354247	Fhl2	NM_010212	1.23117e-005	-1.64515	E11.5 * Wnt1 down vs E11.5 * Ctrl
10536472	Mdfic	NM_175088	0.000155519	-1.64523	E11.5 * Wnt1 down vs E11.5 * Ctrl
10514926	B230314M03Rik	ENSMUST00000	4.73337e-006	-1.64523	E11.5 * Wnt1 down vs E11.5 * Ctrl
10506335	Pde4b	NM_019840	3.33088e-005	-1.64591	E11.5 * Wnt1 down vs E11.5 * Ctrl
10546510	Lrig1	NM_008377	1.32339e-005	-1.64601	E11.5 * Wnt1 down vs E11.5 * Ctrl
10478048	Lbp	NM_008489	3.97477e-006	-1.64707	E11.5 * Wnt1 down vs E11.5 * Ctrl
10380285	Tmem100	NM_026433	0.000644475	-1.64727	E11.5 * Wnt1 down vs E11.5 * Ctrl
10436590	2810055G20Rik	AK148800	0.00191765	-1.64777	E11.5 * Wnt1 down vs E11.5 * Ctrl
10360382	Ifi204	NM_008329	0.00420336	-1.64948	E11.5 * Wnt1 down vs E11.5 * Ctrl
10444258	Psmb8	NM_010724	7.11481e-005	-1.65047	E11.5 * Wnt1 down vs E11.5 * Ctrl
10548940	Lmo3	NM_207222	0.00115786	-1.651	E11.5 * Wnt1 down vs E11.5 * Ctrl
10388958	Evi2a	NM_001033711	0.000787687	-1.65122	E11.5 * Wnt1 down vs E11.5 * Ctrl
10406205	Erap1	NM_030711	0.000229116	-1.65248	E11.5 * Wnt1 down vs E11.5 * Ctrl
10559248	Tspan32	NM_020286	9.65251e-008	-1.65319	E11.5 * Wnt1 down vs E11.5 * Ctrl
10536505	Met	NM_008591	0.000202488	-1.65322	E11.5 * Wnt1 down vs E11.5 * Ctrl
10373223	Lrp1	NM_008512	0.000661904	-1.65335	E11.5 * Wnt1 down vs E11.5 * Ctrl
10408280	Lrrc16a	NM_026825	7.09597e-007	-1.65374	E11.5 * Wnt1 down vs E11.5 * Ctrl
10561453	Zfp36	NM_011756	0.000405591	-1.655	E11.5 * Wnt1 down vs E11.5 * Ctrl
10355227	1110028C15Rik	NM_001122738	7.06504e-006	-1.65502	E11.5 * Wnt1 down vs E11.5 * Ctrl

10522060	Fam114a1	NM_026667	5.86156e-005	-1.65563	E11.5 * Wnt1 down vs E11.5 * Ctrl
10358210	Nr5a2	NM_001159769	0.000436215	-1.65607	E11.5 * Wnt1 down vs E11.5 * Ctrl
10428522	Csmd3	NM_001081391	0.00210871	-1.65608	E11.5 * Wnt1 down vs E11.5 * Ctrl
10434741		---	0.00298223	-1.65696	E11.5 * Wnt1 down vs E11.5 * Ctrl
10470555	Gbgt1	NM_139197	3.90907e-005	-1.65736	E11.5 * Wnt1 down vs E11.5 * Ctrl
10488147	Flrt3	NM_178382	2.0978e-006	-1.65784	E11.5 * Wnt1 down vs E11.5 * Ctrl
10493307		---	0.00466232	-1.65833	E11.5 * Wnt1 down vs E11.5 * Ctrl
10443980	Myo1f	NM_053214	0.000720959	-1.6591	E11.5 * Wnt1 down vs E11.5 * Ctrl
10406551	Ssbp2	NM_024272	1.14644e-008	-1.65937	E11.5 * Wnt1 down vs E11.5 * Ctrl
10354576	Dnahc7a	ENSMUST00000(	0.0049556	-1.65947	E11.5 * Wnt1 down vs E11.5 * Ctrl
10526441	Upk3b	NM_175309	0.000161611	-1.66283	E11.5 * Wnt1 down vs E11.5 * Ctrl
10363887	Bicc1	NM_031397	0.0014891	-1.66376	E11.5 * Wnt1 down vs E11.5 * Ctrl
10531201	Adamts3	NM_001081401	6.52177e-005	-1.66448	E11.5 * Wnt1 down vs E11.5 * Ctrl
10515803	1110020C03Rik	BC062805	1.03985e-005	-1.66476	E11.5 * Wnt1 down vs E11.5 * Ctrl
10434782	Lpp	NM_178665	1.20585e-005	-1.66482	E11.5 * Wnt1 down vs E11.5 * Ctrl
10503659	Epha7	NM_010141	1.65917e-008	-1.66529	E11.5 * Wnt1 down vs E11.5 * Ctrl
10599348	Gria3	NM_016886	7.79287e-007	-1.66582	E11.5 * Wnt1 down vs E11.5 * Ctrl
10397715		---	0.00132132	-1.6678	E11.5 * Wnt1 down vs E11.5 * Ctrl
10409866	Ctla2b	NM_007797	0.000168521	-1.66838	E11.5 * Wnt1 down vs E11.5 * Ctrl
10606600	Pcdh19	NM_001105245	3.80975e-005	-1.6692	E11.5 * Wnt1 down vs E11.5 * Ctrl
10573457	Klf1	NM_010635	1.45727e-005	-1.66979	E11.5 * Wnt1 down vs E11.5 * Ctrl
10541491	1700063H04Rik	ENSMUST00000(	1.49581e-006	-1.66992	E11.5 * Wnt1 down vs E11.5 * Ctrl
10368585	Nkain2	NM_001013411	7.07333e-005	-1.67272	E11.5 * Wnt1 down vs E11.5 * Ctrl
10505779	Acer2	NM_139306	2.9405e-005	-1.67305	E11.5 * Wnt1 down vs E11.5 * Ctrl
10450145	Psmb9	NM_013585	6.91724e-005	-1.67328	E11.5 * Wnt1 down vs E11.5 * Ctrl
10351347	Creg1	NM_011804	9.59895e-006	-1.67336	E11.5 * Wnt1 down vs E11.5 * Ctrl
10474642	BC052040	NM_001145898	3.25118e-005	-1.67478	E11.5 * Wnt1 down vs E11.5 * Ctrl
10527963		---	3.27009e-005	-1.67719	E11.5 * Wnt1 down vs E11.5 * Ctrl
10373179	Gli1	NM_010296	3.21313e-006	-1.68086	E11.5 * Wnt1 down vs E11.5 * Ctrl
10519324	Cdk6	NM_009873	1.14847e-006	-1.68387	E11.5 * Wnt1 down vs E11.5 * Ctrl
10507137	Pdzk1ip1	NM_026018	4.88337e-007	-1.68423	E11.5 * Wnt1 down vs E11.5 * Ctrl
10607945	4933400A11Rik	NR_003635	0.000189378	-1.68452	E11.5 * Wnt1 down vs E11.5 * Ctrl
10457686	Dsc2	NM_013505	4.62601e-007	-1.68509	E11.5 * Wnt1 down vs E11.5 * Ctrl
10586722	F830001A07Rik	AK045567	0.000557572	-1.6851	E11.5 * Wnt1 down vs E11.5 * Ctrl
10359870	Pbx1	NM_183355	5.32655e-005	-1.68567	E11.5 * Wnt1 down vs E11.5 * Ctrl
10527051	Sdk1	NM_177879	9.80707e-006	-1.68597	E11.5 * Wnt1 down vs E11.5 * Ctrl
10489377	Serinc3	NM_012032	1.35722e-010	-1.68723	E11.5 * Wnt1 down vs E11.5 * Ctrl
10448094	Lnpep	NM_172827	3.54494e-006	-1.68737	E11.5 * Wnt1 down vs E11.5 * Ctrl
10589886	4930520O04Rik	AK040958	8.56408e-005	-1.68788	E11.5 * Wnt1 down vs E11.5 * Ctrl
10555087		---	0.00296723	-1.6887	E11.5 * Wnt1 down vs E11.5 * Ctrl
10365845	Fgd6	NM_053072	2.43415e-006	-1.68919	E11.5 * Wnt1 down vs E11.5 * Ctrl
10482509	Rbm43	NM_001141981	5.08184e-005	-1.69087	E11.5 * Wnt1 down vs E11.5 * Ctrl
10602385	Pfkfb1	NM_008824	0.00160495	-1.69135	E11.5 * Wnt1 down vs E11.5 * Ctrl
10385118	Dock2	NM_033374	0.000129345	-1.6915	E11.5 * Wnt1 down vs E11.5 * Ctrl
10468691	Ablim1	NM_178688	7.0516e-007	-1.69233	E11.5 * Wnt1 down vs E11.5 * Ctrl
10531980	BC057170	NM_172777	0.000261967	-1.69261	E11.5 * Wnt1 down vs E11.5 * Ctrl
10414262	Ear2	NM_007895	5.74584e-005	-1.69262	E11.5 * Wnt1 down vs E11.5 * Ctrl
10359201	Ralgps2	NM_023884	4.78527e-010	-1.6927	E11.5 * Wnt1 down vs E11.5 * Ctrl
10578989	Psd3	NM_177698	1.88863e-005	-1.69276	E11.5 * Wnt1 down vs E11.5 * Ctrl
10566943	Mrvi1	NM_010826	3.55424e-005	-1.6931	E11.5 * Wnt1 down vs E11.5 * Ctrl
10562709	Cd33	NM_001111058	2.18896e-007	-1.69341	E11.5 * Wnt1 down vs E11.5 * Ctrl
10456296	Malt1	NM_172833	1.59053e-005	-1.69356	E11.5 * Wnt1 down vs E11.5 * Ctrl
10497451	Cpa3	NM_007753	0.00680333	-1.6936	E11.5 * Wnt1 down vs E11.5 * Ctrl
10348244	Inpp5d	NM_010566	5.24471e-006	-1.6941	E11.5 * Wnt1 down vs E11.5 * Ctrl
10348632	Twist2	NM_007855	2.67378e-005	-1.69467	E11.5 * Wnt1 down vs E11.5 * Ctrl
10427052	Krt7	NM_033073	0.000408549	-1.69529	E11.5 * Wnt1 down vs E11.5 * Ctrl
10425092	Cyth4	NM_028195	3.04464e-006	-1.69853	E11.5 * Wnt1 down vs E11.5 * Ctrl
10450496	Lst1	NM_010734	7.48072e-005	-1.69861	E11.5 * Wnt1 down vs E11.5 * Ctrl
10439312	Cd86	NM_019388	0.00320802	-1.69937	E11.5 * Wnt1 down vs E11.5 * Ctrl
10511817	Klhl32	ENSMUST00000(	0.00459071	-1.70234	E11.5 * Wnt1 down vs E11.5 * Ctrl
10511560	Esrp1	NM_194055	0.00033602	-1.70758	E11.5 * Wnt1 down vs E11.5 * Ctrl
10531173	Adamts3	NM_001081401	0.000239129	-1.71211	E11.5 * Wnt1 down vs E11.5 * Ctrl
10502081	Enpep	NM_007934	4.33138e-006	-1.71237	E11.5 * Wnt1 down vs E11.5 * Ctrl
10435305	Itgb5	NM_001145884	6.42295e-008	-1.71302	E11.5 * Wnt1 down vs E11.5 * Ctrl
10446253	Vav1	NM_011691	0.000114511	-1.71384	E11.5 * Wnt1 down vs E11.5 * Ctrl
10580349	Mylk3	NM_175441	9.81385e-006	-1.71559	E11.5 * Wnt1 down vs E11.5 * Ctrl

10474201	Lmo2	NM_008505	4.80605e-006	-1.71628	E11.5 * Wnt1 down vs E11.5 * Ctrl
10401673	Tgfb3	NM_009368	0.000523823	-1.71795	E11.5 * Wnt1 down vs E11.5 * Ctrl
10408220	Hist1h2ac	NM_178189	0.00644191	-1.71822	E11.5 * Wnt1 down vs E11.5 * Ctrl
10534493	Ccl24	NM_019577	8.66189e-005	-1.71853	E11.5 * Wnt1 down vs E11.5 * Ctrl
10551347	Blvrb	NM_144923	4.66657e-007	-1.71883	E11.5 * Wnt1 down vs E11.5 * Ctrl
10453797	AK220484	NM_001083628	1.501e-005	-1.72098	E11.5 * Wnt1 down vs E11.5 * Ctrl
10603573	Sytl5	NM_177704	9.75458e-006	-1.72238	E11.5 * Wnt1 down vs E11.5 * Ctrl
10439058	Lrrc33	NM_146069	5.22115e-006	-1.72306	E11.5 * Wnt1 down vs E11.5 * Ctrl
10509441	Ece1	NM_199307	0.000121323	-1.72584	E11.5 * Wnt1 down vs E11.5 * Ctrl
10439895	Alcam	NM_009655	2.98053e-005	-1.7269	E11.5 * Wnt1 down vs E11.5 * Ctrl
10513152	Ptpn3	NM_011207	0.00817637	-1.72698	E11.5 * Wnt1 down vs E11.5 * Ctrl
10572527	EG665858	ENSMUST00000	0.00208708	-1.72829	E11.5 * Wnt1 down vs E11.5 * Ctrl
10394731	Rock2	ENSMUST00000	0.000163933	-1.72866	E11.5 * Wnt1 down vs E11.5 * Ctrl
10488378	Thbd	NM_009378	0.000432955	-1.73006	E11.5 * Wnt1 down vs E11.5 * Ctrl
10496569	Gbp6	NM_145545	8.64549e-006	-1.7314	E11.5 * Wnt1 down vs E11.5 * Ctrl
10594066	Loxl1	NM_010729	0.000165593	-1.73168	E11.5 * Wnt1 down vs E11.5 * Ctrl
10493604	She	NM_172530	9.19777e-007	-1.73184	E11.5 * Wnt1 down vs E11.5 * Ctrl
10571444	Slc7a2	NM_007514	9.36479e-005	-1.73315	E11.5 * Wnt1 down vs E11.5 * Ctrl
10439126		---	2.83605e-005	-1.73326	E11.5 * Wnt1 down vs E11.5 * Ctrl
10549162	St8sia1	NM_011374	8.13036e-006	-1.73378	E11.5 * Wnt1 down vs E11.5 * Ctrl
10513154	Ptpn3	NM_011207	0.0002494	-1.73504	E11.5 * Wnt1 down vs E11.5 * Ctrl
10602221		---	0.00114017	-1.73522	E11.5 * Wnt1 down vs E11.5 * Ctrl
10539894	Mgll	NM_011844	0.000162567	-1.73555	E11.5 * Wnt1 down vs E11.5 * Ctrl
10590631	Ccr2	NM_009915	0.0002185	-1.73598	E11.5 * Wnt1 down vs E11.5 * Ctrl
10410039	Ptch1	NM_008957	8.85577e-006	-1.7394	E11.5 * Wnt1 down vs E11.5 * Ctrl
10541587	Clec4a2	NM_011999	0.000377975	-1.73957	E11.5 * Wnt1 down vs E11.5 * Ctrl
10607467	Sat1	NM_009121	9.59915e-005	-1.7407	E11.5 * Wnt1 down vs E11.5 * Ctrl
10364444	Madcam1	NM_013591	0.000161776	-1.74075	E11.5 * Wnt1 down vs E11.5 * Ctrl
10498852	Rxfp1	NM_212452	5.78994e-007	-1.74108	E11.5 * Wnt1 down vs E11.5 * Ctrl
10385477		---	0.00225148	-1.74398	E11.5 * Wnt1 down vs E11.5 * Ctrl
10497580		---	0.00879565	-1.74403	E11.5 * Wnt1 down vs E11.5 * Ctrl
10404359	Mboat1	NM_153546	3.07795e-006	-1.74687	E11.5 * Wnt1 down vs E11.5 * Ctrl
10604608		---	0.000331265	-1.74688	E11.5 * Wnt1 down vs E11.5 * Ctrl
10604694	Mtap7d3	NM_177293	0.000447103	-1.74802	E11.5 * Wnt1 down vs E11.5 * Ctrl
10554140	Zic4	BC116191	1.70105e-006	-1.74843	E11.5 * Wnt1 down vs E11.5 * Ctrl
10548899	Rerg	NM_181988	3.0517e-005	-1.74981	E11.5 * Wnt1 down vs E11.5 * Ctrl
10499536	Efna1	NM_010107	2.35168e-007	-1.75058	E11.5 * Wnt1 down vs E11.5 * Ctrl
10420891	Scara3	NM_172604	4.66251e-005	-1.75059	E11.5 * Wnt1 down vs E11.5 * Ctrl
10396270	Dact1	NM_021532	0.000106441	-1.75111	E11.5 * Wnt1 down vs E11.5 * Ctrl
10441902	Smoc2	NM_022315	1.42029e-005	-1.75345	E11.5 * Wnt1 down vs E11.5 * Ctrl
10459071	2010002N04Rik	NM_134133	6.27497e-006	-1.75429	E11.5 * Wnt1 down vs E11.5 * Ctrl
10519855	Cacna2d1	NM_001110843	8.50926e-005	-1.75568	E11.5 * Wnt1 down vs E11.5 * Ctrl
10435565	Hcls1	NM_008225	2.19525e-005	-1.75602	E11.5 * Wnt1 down vs E11.5 * Ctrl
10407126	Plk2	NM_152804	3.03081e-005	-1.75705	E11.5 * Wnt1 down vs E11.5 * Ctrl
10510150	OTTMUSG00000	NM_001014397	0.00132374	-1.75732	E11.5 * Wnt1 down vs E11.5 * Ctrl
10521796		---	0.0019814	-1.76265	E11.5 * Wnt1 down vs E11.5 * Ctrl
10591978	Ntm	NM_172290	1.49265e-005	-1.76267	E11.5 * Wnt1 down vs E11.5 * Ctrl
10531179	Adamts3	NM_001081401	8.05363e-005	-1.76276	E11.5 * Wnt1 down vs E11.5 * Ctrl
10400304	Egln3	NM_028133	0.000806506	-1.76391	E11.5 * Wnt1 down vs E11.5 * Ctrl
10484203	2610301F02Rik	ENSMUST00000	4.67795e-007	-1.76448	E11.5 * Wnt1 down vs E11.5 * Ctrl
10360377	AI607873	BC150711	0.000774159	-1.76486	E11.5 * Wnt1 down vs E11.5 * Ctrl
10534906	OTTMUSG00000	ENSMUST00000	0.000127327	-1.76711	E11.5 * Wnt1 down vs E11.5 * Ctrl
10606366	Zcchc5	NM_199468	0.000533197	-1.76742	E11.5 * Wnt1 down vs E11.5 * Ctrl
10354598	Hecw2	NM_001001883	2.84976e-006	-1.76854	E11.5 * Wnt1 down vs E11.5 * Ctrl
10591090	Fat3	NM_001080814	6.22059e-005	-1.77062	E11.5 * Wnt1 down vs E11.5 * Ctrl
10503835	Rragd	NM_027491	2.14402e-006	-1.77072	E11.5 * Wnt1 down vs E11.5 * Ctrl
10458843	Sema6a	NM_018744	3.50154e-007	-1.77216	E11.5 * Wnt1 down vs E11.5 * Ctrl
10422022	ENSMUSG00000	ENSMUST00000	0.000494236	-1.77291	E11.5 * Wnt1 down vs E11.5 * Ctrl
10399924	Pik3cg	NM_020272	4.04837e-006	-1.77332	E11.5 * Wnt1 down vs E11.5 * Ctrl
10478928	Tshz2	NM_080455	4.64546e-009	-1.77399	E11.5 * Wnt1 down vs E11.5 * Ctrl
10386211	3100002J23Rik	ENSMUST00000	0.00144544	-1.77473	E11.5 * Wnt1 down vs E11.5 * Ctrl
10526797	Stag3	NM_016964	6.32597e-007	-1.77632	E11.5 * Wnt1 down vs E11.5 * Ctrl
10523128	Ppbp	NM_023785	7.76361e-005	-1.77787	E11.5 * Wnt1 down vs E11.5 * Ctrl
10603182	Arhgap6	NM_009707	4.69336e-005	-1.7788	E11.5 * Wnt1 down vs E11.5 * Ctrl
10538715	C130092O11Rik	BC107398	7.10858e-006	-1.77937	E11.5 * Wnt1 down vs E11.5 * Ctrl
10399691	Id2	NM_010496	1.54474e-006	-1.78129	E11.5 * Wnt1 down vs E11.5 * Ctrl

10492438	EG624866	NM_001037923	5.07981e-006	-1.78136	E11.5 * Wnt1 down vs E11.5 * Ctrl
10528821	ENSMUSG0000C	NM_001134299	3.48587e-007	-1.78155	E11.5 * Wnt1 down vs E11.5 * Ctrl
10554599	Adamtsl3	ENSMUST00000(	2.27449e-005	-1.7823	E11.5 * Wnt1 down vs E11.5 * Ctrl
10427906	D130046C19Rik	AK051399	0.00138267	-1.783	E11.5 * Wnt1 down vs E11.5 * Ctrl
10425749	Fam109b	NM_177391	3.11688e-005	-1.7831	E11.5 * Wnt1 down vs E11.5 * Ctrl
10368566	Tpd52l1	NM_009413	0.000167565	-1.78379	E11.5 * Wnt1 down vs E11.5 * Ctrl
10539211	Lrrtm4	NR_027323	6.71211e-007	-1.78901	E11.5 * Wnt1 down vs E11.5 * Ctrl
10507500	Slc6a9	NM_008135	2.73111e-007	-1.79211	E11.5 * Wnt1 down vs E11.5 * Ctrl
10555323	P4ha3	NM_177161	3.79291e-005	-1.79406	E11.5 * Wnt1 down vs E11.5 * Ctrl
10455128	Pcdhb20	NM_053145	0.000513603	-1.79412	E11.5 * Wnt1 down vs E11.5 * Ctrl
10506488	Ppap2b	NM_080555	0.000427852	-1.79736	E11.5 * Wnt1 down vs E11.5 * Ctrl
10567580	Igsf6	NM_030691	2.61844e-005	-1.79857	E11.5 * Wnt1 down vs E11.5 * Ctrl
10406782	Fam169a	NM_001100458	0.000487806	-1.79899	E11.5 * Wnt1 down vs E11.5 * Ctrl
10372807	Msrb3	NM_177092	4.84785e-006	-1.79917	E11.5 * Wnt1 down vs E11.5 * Ctrl
10462922	Plce1	NM_019588	2.18402e-009	-1.8012	E11.5 * Wnt1 down vs E11.5 * Ctrl
10544788	Hoxa9	NM_010456	1.53611e-005	-1.80179	E11.5 * Wnt1 down vs E11.5 * Ctrl
10354003	Mgat4a	NM_173870	0.00114236	-1.80179	E11.5 * Wnt1 down vs E11.5 * Ctrl
10489569	Pltp	NM_011125	8.57924e-008	-1.80222	E11.5 * Wnt1 down vs E11.5 * Ctrl
10547621	Apobec1	NM_031159	8.08676e-008	-1.8036	E11.5 * Wnt1 down vs E11.5 * Ctrl
10596115	Ephb1	AK082061	5.44944e-005	-1.80388	E11.5 * Wnt1 down vs E11.5 * Ctrl
10375055	F830116E18Rik	NM_001033981	0.000329531	-1.80539	E11.5 * Wnt1 down vs E11.5 * Ctrl
10495967	Tifa	NM_145133	6.96986e-007	-1.8067	E11.5 * Wnt1 down vs E11.5 * Ctrl
10528804	ENSMUSG0000C	NM_001134299	8.70339e-007	-1.80725	E11.5 * Wnt1 down vs E11.5 * Ctrl
10458894	Lox	NM_010728	0.000476957	-1.80803	E11.5 * Wnt1 down vs E11.5 * Ctrl
10363132	Nepn	NM_025684	0.000105819	-1.81175	E11.5 * Wnt1 down vs E11.5 * Ctrl
10427816	Pdzd2	NM_001081064	1.43583e-006	-1.81405	E11.5 * Wnt1 down vs E11.5 * Ctrl
10412298	Itga1	NM_001033228	0.000915638	-1.81484	E11.5 * Wnt1 down vs E11.5 * Ctrl
10369792	Arid5b	NM_023598	0.000113372	-1.81614	E11.5 * Wnt1 down vs E11.5 * Ctrl
10570963	Zmat4	NM_177086	1.73444e-006	-1.81621	E11.5 * Wnt1 down vs E11.5 * Ctrl
10368947	Aim1	NM_172393	2.9206e-005	-1.81811	E11.5 * Wnt1 down vs E11.5 * Ctrl
10536845	Flnc	NM_001081185	2.75954e-007	-1.81837	E11.5 * Wnt1 down vs E11.5 * Ctrl
10395606	ENSMUSG0000C	ENSMUST00000	0.000273043	-1.82012	E11.5 * Wnt1 down vs E11.5 * Ctrl
10474700	Thbs1	NM_011580	1.5808e-006	-1.82059	E11.5 * Wnt1 down vs E11.5 * Ctrl
10485633	ENSMUSG0000C	ENSMUST00000(	0.00318131	-1.82112	E11.5 * Wnt1 down vs E11.5 * Ctrl
10463263	Lztfl1	NM_033322	0.000185346	-1.8217	E11.5 * Wnt1 down vs E11.5 * Ctrl
10369844	Bicc1	NM_031397	6.65589e-007	-1.82259	E11.5 * Wnt1 down vs E11.5 * Ctrl
10531183	Adamts3	NM_001081401	8.73483e-006	-1.82557	E11.5 * Wnt1 down vs E11.5 * Ctrl
10566326	Trim12	NM_023835	0.000403968	-1.82765	E11.5 * Wnt1 down vs E11.5 * Ctrl
10542172	Clec1b	NM_019985	6.05224e-007	-1.82807	E11.5 * Wnt1 down vs E11.5 * Ctrl
10414537	Ang	NM_007447	1.00685e-005	-1.82908	E11.5 * Wnt1 down vs E11.5 * Ctrl
10368720	Slc16a10	NM_001114332	9.55419e-009	-1.8299	E11.5 * Wnt1 down vs E11.5 * Ctrl
10598976	Timp1	NM_001044384	1.30057e-005	-1.83055	E11.5 * Wnt1 down vs E11.5 * Ctrl
10366645	1700006J14Rik	ENSMUST00000(	2.43602e-005	-1.8311	E11.5 * Wnt1 down vs E11.5 * Ctrl
10497265	Fabp4	NM_024406	0.000787885	-1.83127	E11.5 * Wnt1 down vs E11.5 * Ctrl
10594199	Thsd4	NM_001040426	4.23317e-006	-1.83231	E11.5 * Wnt1 down vs E11.5 * Ctrl
10522749	Lphn3	NM_198702	2.18993e-006	-1.83241	E11.5 * Wnt1 down vs E11.5 * Ctrl
10502451	Bmpr1b	NM_007560	3.43713e-005	-1.83246	E11.5 * Wnt1 down vs E11.5 * Ctrl
10427918	Fam105a	BC052328	1.24665e-006	-1.83262	E11.5 * Wnt1 down vs E11.5 * Ctrl
10408329	Gmnn	AF068780	0.00153715	-1.83351	E11.5 * Wnt1 down vs E11.5 * Ctrl
10375145	Lcp2	NM_010696	1.85364e-005	-1.83493	E11.5 * Wnt1 down vs E11.5 * Ctrl
10588899	Gpx1	NM_008160	0.000231021	-1.83509	E11.5 * Wnt1 down vs E11.5 * Ctrl
10459391	Fech	NM_007998	8.20535e-008	-1.83673	E11.5 * Wnt1 down vs E11.5 * Ctrl
10471912	Kynu	NM_027552	0.000284627	-1.83852	E11.5 * Wnt1 down vs E11.5 * Ctrl
10377938	Eno3	NM_007933	6.82442e-007	-1.83886	E11.5 * Wnt1 down vs E11.5 * Ctrl
10398319	Dlk1	NM_010052	0.000596063	-1.83904	E11.5 * Wnt1 down vs E11.5 * Ctrl
10560624	Apoe	NM_009696	2.50051e-007	-1.84073	E11.5 * Wnt1 down vs E11.5 * Ctrl
10416974	Gpc6	NM_001079844	1.03961e-006	-1.84458	E11.5 * Wnt1 down vs E11.5 * Ctrl
10503520	Ttpa	NM_015767	2.84589e-005	-1.84646	E11.5 * Wnt1 down vs E11.5 * Ctrl
10565958	P2ry6	NM_183168	2.24278e-006	-1.84782	E11.5 * Wnt1 down vs E11.5 * Ctrl
10593832		---	0.00174927	-1.85015	E11.5 * Wnt1 down vs E11.5 * Ctrl
10368317	Enpp3	NM_134005	3.26031e-008	-1.85134	E11.5 * Wnt1 down vs E11.5 * Ctrl
10514128	Ttc39b	NM_027238	0.000385054	-1.85285	E11.5 * Wnt1 down vs E11.5 * Ctrl
10528476		---	0.00249778	-1.8531	E11.5 * Wnt1 down vs E11.5 * Ctrl
10447602	Ezr	NM_009510	1.28552e-006	-1.85533	E11.5 * Wnt1 down vs E11.5 * Ctrl
10360834	Hlx	NM_008250	6.29888e-007	-1.85577	E11.5 * Wnt1 down vs E11.5 * Ctrl
10594110	Neo1	NM_008684	1.11239e-010	-1.85667	E11.5 * Wnt1 down vs E11.5 * Ctrl

10461369	Ahnak	NM_009643	1.36764e-006	-1.85682	E11.5 * Wnt1 down vs E11.5 * Ctrl
10467041	Asah2	NM_018830	1.27414e-007	-1.85711	E11.5 * Wnt1 down vs E11.5 * Ctrl
10435697	Popdc2	NM_001081984	9.59008e-006	-1.85812	E11.5 * Wnt1 down vs E11.5 * Ctrl
10586718	9530091C08Rik	NM_177159	0.00154169	-1.85878	E11.5 * Wnt1 down vs E11.5 * Ctrl
10543273	Cttnbp2	NM_080285	8.67488e-006	-1.85891	E11.5 * Wnt1 down vs E11.5 * Ctrl
10472757	Cybrd1	NM_028593	1.2533e-006	-1.85995	E11.5 * Wnt1 down vs E11.5 * Ctrl
10467258	Myof	NM_001099634	1.57636e-007	-1.86009	E11.5 * Wnt1 down vs E11.5 * Ctrl
10521678	Cd38	NM_007646	1.41287e-007	-1.86123	E11.5 * Wnt1 down vs E11.5 * Ctrl
10455647	Tnfaip8	NM_134131	8.1793e-005	-1.86208	E11.5 * Wnt1 down vs E11.5 * Ctrl
10389022	Myo1d	NM_177390	0.000161153	-1.86319	E11.5 * Wnt1 down vs E11.5 * Ctrl
10441791	D17Ertd663e	ENSMUST00000	1.59824e-007	-1.86565	E11.5 * Wnt1 down vs E11.5 * Ctrl
10538305	5730596B20Rik	ENSMUST00000	3.62556e-005	-1.86617	E11.5 * Wnt1 down vs E11.5 * Ctrl
10403743	Inhba	NM_008380	1.84229e-005	-1.86625	E11.5 * Wnt1 down vs E11.5 * Ctrl
10502830	Nexn	NM_199465	5.69293e-005	-1.86969	E11.5 * Wnt1 down vs E11.5 * Ctrl
10436596	2810055G20Rik	ENSMUST00000	1.98306e-005	-1.87087	E11.5 * Wnt1 down vs E11.5 * Ctrl
10496971	Asb17	NM_025758	2.63652e-007	-1.87148	E11.5 * Wnt1 down vs E11.5 * Ctrl
10570855	Plat	NM_008872	3.01818e-005	-1.87199	E11.5 * Wnt1 down vs E11.5 * Ctrl
10345212	Khdrbs2	NM_133235	2.01459e-005	-1.87209	E11.5 * Wnt1 down vs E11.5 * Ctrl
10521759	Slit2	NM_178804	2.20035e-007	-1.87487	E11.5 * Wnt1 down vs E11.5 * Ctrl
10570894	Ank1	NM_001110783	1.73048e-010	-1.87549	E11.5 * Wnt1 down vs E11.5 * Ctrl
10353309	Jph1	AK081751	0.00290821	-1.87586	E11.5 * Wnt1 down vs E11.5 * Ctrl
10473384	Slc43a3	NM_021398	4.38595e-007	-1.87799	E11.5 * Wnt1 down vs E11.5 * Ctrl
10500335	Fcgr1	NM_010186	1.25956e-005	-1.87854	E11.5 * Wnt1 down vs E11.5 * Ctrl
10442087	4930546H06Rik	BC171984	1.69179e-005	-1.87909	E11.5 * Wnt1 down vs E11.5 * Ctrl
10549108	Abcc9	NM_021041	7.28407e-007	-1.88054	E11.5 * Wnt1 down vs E11.5 * Ctrl
10531191	Adamts3	NM_001081401	0.00167836	-1.88102	E11.5 * Wnt1 down vs E11.5 * Ctrl
10410931	Vcan	NM_001081249	1.28899e-006	-1.8815	E11.5 * Wnt1 down vs E11.5 * Ctrl
10458303	Ecscr	NM_001033141	4.20052e-007	-1.88167	E11.5 * Wnt1 down vs E11.5 * Ctrl
10505674	Cntln	NM_175275	1.33214e-010	-1.88358	E11.5 * Wnt1 down vs E11.5 * Ctrl
10423568	5730407I07Rik	ENSMUST00000	3.25357e-005	-1.88597	E11.5 * Wnt1 down vs E11.5 * Ctrl
10568024	Coro1a	NM_009898	2.3142e-007	-1.88604	E11.5 * Wnt1 down vs E11.5 * Ctrl
10521622	4833430A08Rik	AK076499	9.98604e-005	-1.88703	E11.5 * Wnt1 down vs E11.5 * Ctrl
10543791	Podxl	NM_013723	3.42499e-006	-1.88876	E11.5 * Wnt1 down vs E11.5 * Ctrl
10494114	Selenbp1	NM_009150	6.95181e-005	-1.8889	E11.5 * Wnt1 down vs E11.5 * Ctrl
10362102	ENSMUSG00000	ENSMUST00000	7.15058e-006	-1.88932	E11.5 * Wnt1 down vs E11.5 * Ctrl
10406504	Edil3	NM_001037987	0.000254302	-1.8898	E11.5 * Wnt1 down vs E11.5 * Ctrl
10462853	Cyp26a1	NM_007811	2.61185e-007	-1.88996	E11.5 * Wnt1 down vs E11.5 * Ctrl
10435961	ENSMUSG00000	ENSMUST00000	6.55673e-006	-1.89107	E11.5 * Wnt1 down vs E11.5 * Ctrl
10579054	4930467E23Rik	ENSMUST00000	0.00170446	-1.89122	E11.5 * Wnt1 down vs E11.5 * Ctrl
10418410	Prkcd	NM_011103	1.56725e-006	-1.8913	E11.5 * Wnt1 down vs E11.5 * Ctrl
10499870	Lor	NM_008508	0.00107431	-1.89133	E11.5 * Wnt1 down vs E11.5 * Ctrl
10579958	Il15	NM_008357	1.30605e-006	-1.89153	E11.5 * Wnt1 down vs E11.5 * Ctrl
10361926	Map3k5	NM_008580	1.16412e-005	-1.89374	E11.5 * Wnt1 down vs E11.5 * Ctrl
10476021	Sirpa	NM_007547	3.15103e-007	-1.89761	E11.5 * Wnt1 down vs E11.5 * Ctrl
10599696	Ddx26b	NM_172779	3.52616e-006	-1.89809	E11.5 * Wnt1 down vs E11.5 * Ctrl
10478936		---	0.0057427	-1.899	E11.5 * Wnt1 down vs E11.5 * Ctrl
10519747	Sema3e	NM_011348	2.51608e-006	-1.8993	E11.5 * Wnt1 down vs E11.5 * Ctrl
10433492	Atf7ip2	NM_153123	6.94933e-006	-1.9011	E11.5 * Wnt1 down vs E11.5 * Ctrl
10544089	Zc3hav1	NM_028421	6.58369e-008	-1.90289	E11.5 * Wnt1 down vs E11.5 * Ctrl
10455721	Sncaip	NM_026408	4.35315e-005	-1.90665	E11.5 * Wnt1 down vs E11.5 * Ctrl
10441787	2810051F02Rik	BC009123	3.34807e-007	-1.90665	E11.5 * Wnt1 down vs E11.5 * Ctrl
10419465	Ccnb1ip1	NM_001111119	1.09297e-006	-1.90802	E11.5 * Wnt1 down vs E11.5 * Ctrl
10577361			9.50167e-005	-1.90886	E11.5 * Wnt1 down vs E11.5 * Ctrl
10556076	Olfml1	NM_172907	5.82618e-005	-1.90906	E11.5 * Wnt1 down vs E11.5 * Ctrl
10406905	Ccdc125	NM_183115	5.71324e-005	-1.91157	E11.5 * Wnt1 down vs E11.5 * Ctrl
10513101		---	0.000831376	-1.91433	E11.5 * Wnt1 down vs E11.5 * Ctrl
10398442		---	0.00201556	-1.91728	E11.5 * Wnt1 down vs E11.5 * Ctrl
10353213	Msc	NM_010827	9.87286e-006	-1.9174	E11.5 * Wnt1 down vs E11.5 * Ctrl
10530615	Ociad2	NM_026950	0.000342865	-1.92149	E11.5 * Wnt1 down vs E11.5 * Ctrl
10577388	4930467E23Rik	ENSMUST00000	0.000619967	-1.92373	E11.5 * Wnt1 down vs E11.5 * Ctrl
10600857	Heph	NM_010417	4.38927e-006	-1.92756	E11.5 * Wnt1 down vs E11.5 * Ctrl
10368598	Rlbp1l2	NM_175448	7.53567e-005	-1.92913	E11.5 * Wnt1 down vs E11.5 * Ctrl
10464251	Atrnl1	NM_181415	3.78295e-007	-1.93121	E11.5 * Wnt1 down vs E11.5 * Ctrl
10418506	Stab1	NM_138672	1.14029e-008	-1.93209	E11.5 * Wnt1 down vs E11.5 * Ctrl
10344750	Sgk3	NM_133220	0.000651917	-1.93224	E11.5 * Wnt1 down vs E11.5 * Ctrl
10352143	D230039L06Rik	NM_177724	2.0761e-006	-1.93229	E11.5 * Wnt1 down vs E11.5 * Ctrl

10531193	Adamts3	NM_001081401	2.91195e-005	-1.93302	E11.5 * Wnt1 down vs E11.5 * Ctrl
10476321	Prnd	NM_023043	5.71504e-005	-1.93348	E11.5 * Wnt1 down vs E11.5 * Ctrl
10456046	Pdgfrb	NM_001146268	1.11177e-005	-1.93478	E11.5 * Wnt1 down vs E11.5 * Ctrl
10528815	5031410I06Rik	NM_207657	8.57319e-008	-1.93707	E11.5 * Wnt1 down vs E11.5 * Ctrl
10590031	Itga9	NM_133721	5.29917e-006	-1.94145	E11.5 * Wnt1 down vs E11.5 * Ctrl
10582466	Sult5a1	NM_020564	1.3499e-007	-1.94228	E11.5 * Wnt1 down vs E11.5 * Ctrl
10471721	Ptgs1	NM_008969	7.62951e-008	-1.94324	E11.5 * Wnt1 down vs E11.5 * Ctrl
10493820	S100a6	NM_011313	0.000199498	-1.94602	E11.5 * Wnt1 down vs E11.5 * Ctrl
10505489	Pappa	NM_021362	6.77817e-007	-1.94741	E11.5 * Wnt1 down vs E11.5 * Ctrl
10440091	Col8a1	NM_007739	0.000471278	-1.94948	E11.5 * Wnt1 down vs E11.5 * Ctrl
10355806	Tuba4a	NM_009447	5.75536e-007	-1.95081	E11.5 * Wnt1 down vs E11.5 * Ctrl
10492888		---	1.95123e-005	-1.95121	E11.5 * Wnt1 down vs E11.5 * Ctrl
10384423	Cobl	NM_172496	1.83618e-007	-1.9517	E11.5 * Wnt1 down vs E11.5 * Ctrl
10492428	Tiparp	NM_178892	1.30541e-007	-1.95356	E11.5 * Wnt1 down vs E11.5 * Ctrl
10591120	Fat3	NM_001080814	6.5071e-006	-1.95493	E11.5 * Wnt1 down vs E11.5 * Ctrl
10581605	Hp	NM_017370	0.000144051	-1.95867	E11.5 * Wnt1 down vs E11.5 * Ctrl
10528810	Speer4a	NM_029376	1.55349e-008	-1.96151	E11.5 * Wnt1 down vs E11.5 * Ctrl
10500283	Car14	NM_011797	1.87364e-006	-1.96797	E11.5 * Wnt1 down vs E11.5 * Ctrl
10372293	EG628870	XM_893664	1.08143e-009	-1.96945	E11.5 * Wnt1 down vs E11.5 * Ctrl
10367591	Myct1	NM_026793	0.000113622	-1.97231	E11.5 * Wnt1 down vs E11.5 * Ctrl
10570634	4930467E23Rik	ENSMUST00000(	0.0013271	-1.97364	E11.5 * Wnt1 down vs E11.5 * Ctrl
10344935	Kcnb2	NM_001098528	1.35521e-005	-1.97366	E11.5 * Wnt1 down vs E11.5 * Ctrl
10554863	Sytl2	NM_001040085	5.59699e-006	-1.97396	E11.5 * Wnt1 down vs E11.5 * Ctrl
10604576	Gpc3	NM_016697	3.59754e-006	-1.97508	E11.5 * Wnt1 down vs E11.5 * Ctrl
10491414	Usp13	NM_001013024	2.12689e-006	-1.97564	E11.5 * Wnt1 down vs E11.5 * Ctrl
10374727	Bcl11a	NM_016707	3.28584e-007	-1.97582	E11.5 * Wnt1 down vs E11.5 * Ctrl
10545401	Vamp5	NM_016872	6.66253e-007	-1.97694	E11.5 * Wnt1 down vs E11.5 * Ctrl
10513774		---	1.1735e-005	-1.97796	E11.5 * Wnt1 down vs E11.5 * Ctrl
10575548		---	0.00027785	-1.9786	E11.5 * Wnt1 down vs E11.5 * Ctrl
10356995		---	7.05572e-005	-1.97946	E11.5 * Wnt1 down vs E11.5 * Ctrl
10502890	St6galnac3	NM_011372	8.61844e-008	-1.98314	E11.5 * Wnt1 down vs E11.5 * Ctrl
10368409	Lama2	NM_008481	9.2235e-010	-1.98841	E11.5 * Wnt1 down vs E11.5 * Ctrl
10600852		---	7.94644e-006	-1.98848	E11.5 * Wnt1 down vs E11.5 * Ctrl
10560242	C5ar1	NM_007577	5.83031e-005	-1.98857	E11.5 * Wnt1 down vs E11.5 * Ctrl
10398356		---	1.5103e-005	-1.98876	E11.5 * Wnt1 down vs E11.5 * Ctrl
10544774	Hoxa6	NM_010454	3.90605e-006	-1.98913	E11.5 * Wnt1 down vs E11.5 * Ctrl
10433274	Vasn	NM_139307	4.3576e-006	-1.99158	E11.5 * Wnt1 down vs E11.5 * Ctrl
10415396	Nfatc4	NM_023699	2.46883e-006	-1.99178	E11.5 * Wnt1 down vs E11.5 * Ctrl
10441003	Runx1	NM_001111023	5.65597e-006	-1.99402	E11.5 * Wnt1 down vs E11.5 * Ctrl
10603346	Plp2	NM_019755	2.83758e-006	-1.99521	E11.5 * Wnt1 down vs E11.5 * Ctrl
10427280	Hoxc10	NM_010462	8.66921e-007	-1.99624	E11.5 * Wnt1 down vs E11.5 * Ctrl
10495976	Pitx2	NM_011098	6.65648e-008	-2.00029	E11.5 * Wnt1 down vs E11.5 * Ctrl
10533401	Cux2	ENSMUST00000	2.20093e-006	-2.0031	E11.5 * Wnt1 down vs E11.5 * Ctrl
10352150		---	8.83246e-007	-2.00419	E11.5 * Wnt1 down vs E11.5 * Ctrl
10497608	Mds1	NM_021442	1.04276e-005	-2.00515	E11.5 * Wnt1 down vs E11.5 * Ctrl
10390746		---	5.89195e-005	-2.00707	E11.5 * Wnt1 down vs E11.5 * Ctrl
10354677	Ankrd44	NM_001081433	1.19703e-006	-2.00722	E11.5 * Wnt1 down vs E11.5 * Ctrl
10587733	Ctsh	NM_007801	3.84576e-005	-2.00733	E11.5 * Wnt1 down vs E11.5 * Ctrl
10513166	Ptpn3	NM_011207	1.28899e-006	-2.00797	E11.5 * Wnt1 down vs E11.5 * Ctrl
10473022	Plp2	NM_019755	5.6776e-005	-2.00895	E11.5 * Wnt1 down vs E11.5 * Ctrl
10599174	Il13ra1	NM_133990	8.74662e-008	-2.00932	E11.5 * Wnt1 down vs E11.5 * Ctrl
10389025	Myo1d	NM_177390	7.36587e-005	-2.01076	E11.5 * Wnt1 down vs E11.5 * Ctrl
10358421	Rgs18	NM_022881	4.91323e-008	-2.01542	E11.5 * Wnt1 down vs E11.5 * Ctrl
10538842	Gng12	NM_025278	5.40377e-009	-2.01894	E11.5 * Wnt1 down vs E11.5 * Ctrl
10605493	Prrg1	BC032926	8.10391e-007	-2.02272	E11.5 * Wnt1 down vs E11.5 * Ctrl
10366052	Kitl	NM_013598	4.48495e-007	-2.02368	E11.5 * Wnt1 down vs E11.5 * Ctrl
10406928	Cd180	NM_008533	1.2412e-005	-2.02579	E11.5 * Wnt1 down vs E11.5 * Ctrl
10355500	Igfbp5	NM_010518	4.08631e-005	-2.02618	E11.5 * Wnt1 down vs E11.5 * Ctrl
10427297	Hoxc5	NM_175730	3.38347e-008	-2.02624	E11.5 * Wnt1 down vs E11.5 * Ctrl
10466606	Anxa1	NM_010730	0.00261137	-2.02905	E11.5 * Wnt1 down vs E11.5 * Ctrl
10565712	Acer3	NM_025408	1.52847e-006	-2.03464	E11.5 * Wnt1 down vs E11.5 * Ctrl
10579060	4930467E23Rik	ENSMUST00000(	0.00115191	-2.03564	E11.5 * Wnt1 down vs E11.5 * Ctrl
10344990	Crispld1	NM_031402	3.31043e-009	-2.03578	E11.5 * Wnt1 down vs E11.5 * Ctrl
10430555	Dmc1	NM_010059	0.000144473	-2.03927	E11.5 * Wnt1 down vs E11.5 * Ctrl
10438708	Masp1	NM_008555	1.24858e-005	-2.04713	E11.5 * Wnt1 down vs E11.5 * Ctrl
10472962	Hoxd9	NM_013555	5.60707e-005	-2.05094	E11.5 * Wnt1 down vs E11.5 * Ctrl

10416004	Zfp395	NM_199029	8.00604e-005	-2.0516	E11.5 * Wnt1 down vs E11.5 * Ctrl
10409061		---	0.000982597	-2.05338	E11.5 * Wnt1 down vs E11.5 * Ctrl
10458731	Mcc	NM_001085373	2.57016e-008	-2.05516	E11.5 * Wnt1 down vs E11.5 * Ctrl
10513145	Ptpn3	NM_011207	4.59904e-005	-2.05626	E11.5 * Wnt1 down vs E11.5 * Ctrl
10407281	Esm1	NM_023612	1.18641e-007	-2.05919	E11.5 * Wnt1 down vs E11.5 * Ctrl
10423902	Zfpm2	NM_011766	6.66618e-006	-2.06112	E11.5 * Wnt1 down vs E11.5 * Ctrl
10369752	Lrrtm3	NM_178678	4.2513e-006	-2.06121	E11.5 * Wnt1 down vs E11.5 * Ctrl
10419082	5730469M10Rik	BC056635	1.9283e-007	-2.06835	E11.5 * Wnt1 down vs E11.5 * Ctrl
10457091	Neto1	NM_144946	5.51224e-005	-2.06903	E11.5 * Wnt1 down vs E11.5 * Ctrl
10506301	Lepr	NM_001122899	1.71841e-007	-2.07099	E11.5 * Wnt1 down vs E11.5 * Ctrl
10462237	Smarca2	NM_011416	6.13635e-010	-2.07202	E11.5 * Wnt1 down vs E11.5 * Ctrl
10462818	Hhex	NM_008245	2.30653e-005	-2.07204	E11.5 * Wnt1 down vs E11.5 * Ctrl
10345762	Il1r1	NM_008362	1.98243e-006	-2.07342	E11.5 * Wnt1 down vs E11.5 * Ctrl
10483046	Dpp4	NM_010074	5.73463e-006	-2.07422	E11.5 * Wnt1 down vs E11.5 * Ctrl
10606694	Btk	NM_013482	6.82291e-006	-2.07919	E11.5 * Wnt1 down vs E11.5 * Ctrl
10528794	5031410I06Rik	NM_207657	3.55658e-009	-2.07953	E11.5 * Wnt1 down vs E11.5 * Ctrl
10523468	Bmp2k	NM_080708	5.4416e-006	-2.08279	E11.5 * Wnt1 down vs E11.5 * Ctrl
10433096	Nfe2	NM_008685	3.66102e-008	-2.08285	E11.5 * Wnt1 down vs E11.5 * Ctrl
10453759	ENSMUSG0000C	ENSMUST00000(	8.89652e-009	-2.0834	E11.5 * Wnt1 down vs E11.5 * Ctrl
10487040	Fbn1	NM_007993	3.37665e-007	-2.08472	E11.5 * Wnt1 down vs E11.5 * Ctrl
10542355	Emp1	NM_010128	1.37177e-005	-2.08654	E11.5 * Wnt1 down vs E11.5 * Ctrl
10477600		---	1.04793e-008	-2.08755	E11.5 * Wnt1 down vs E11.5 * Ctrl
10426315	Lrrk2	NM_025730	1.69176e-008	-2.08763	E11.5 * Wnt1 down vs E11.5 * Ctrl
10599581	2610018G03Rik	NM_133729	1.16659e-008	-2.08834	E11.5 * Wnt1 down vs E11.5 * Ctrl
10447946	4930474M22Rik	BC089489	1.30824e-006	-2.0965	E11.5 * Wnt1 down vs E11.5 * Ctrl
10513805	Dbc1	NM_019967	3.94436e-007	-2.09666	E11.5 * Wnt1 down vs E11.5 * Ctrl
10355403	Fn1	NM_010233	1.467e-006	-2.09881	E11.5 * Wnt1 down vs E11.5 * Ctrl
10506201	Ror1	NM_013845	7.05972e-009	-2.09895	E11.5 * Wnt1 down vs E11.5 * Ctrl
10506058	Inadl	NM_172696	2.93759e-009	-2.103	E11.5 * Wnt1 down vs E11.5 * Ctrl
10442327	Cldn6	NM_018777	3.05167e-006	-2.10357	E11.5 * Wnt1 down vs E11.5 * Ctrl
10546661	Foxp1	NM_053202	1.52264e-008	-2.10377	E11.5 * Wnt1 down vs E11.5 * Ctrl
10365559	Igf1	NM_010512	1.28174e-006	-2.10664	E11.5 * Wnt1 down vs E11.5 * Ctrl
10580033	Cd97	NM_011925	1.21836e-005	-2.1068	E11.5 * Wnt1 down vs E11.5 * Ctrl
10570957	Sfrp1	NM_013834	7.88289e-006	-2.10732	E11.5 * Wnt1 down vs E11.5 * Ctrl
10436304	Abi3bp	NM_001014423	6.36069e-007	-2.1077	E11.5 * Wnt1 down vs E11.5 * Ctrl
10384656	B3gnt2	NM_016888	2.35998e-006	-2.11078	E11.5 * Wnt1 down vs E11.5 * Ctrl
10453394	Six2	NM_011380	7.35907e-008	-2.11321	E11.5 * Wnt1 down vs E11.5 * Ctrl
10352548	Slc30a10	NM_001033286	7.79103e-007	-2.11367	E11.5 * Wnt1 down vs E11.5 * Ctrl
10551736	Ppp1r14a	NM_026731	0.000411349	-2.11369	E11.5 * Wnt1 down vs E11.5 * Ctrl
10514255	Mllt3	NM_027326	3.24409e-011	-2.1148	E11.5 * Wnt1 down vs E11.5 * Ctrl
10419154	Ear1	NM_007894	5.52756e-005	-2.11599	E11.5 * Wnt1 down vs E11.5 * Ctrl
10502776	Lphn2	NM_001081298	1.81486e-011	-2.1163	E11.5 * Wnt1 down vs E11.5 * Ctrl
10502783	ENSMUSG0000C	ENSMUST00000(	8.77381e-005	-2.11757	E11.5 * Wnt1 down vs E11.5 * Ctrl
10411359	Plp2	NM_019755	4.31819e-006	-2.11946	E11.5 * Wnt1 down vs E11.5 * Ctrl
10376950	Pmp22	NM_008885	6.86352e-009	-2.1196	E11.5 * Wnt1 down vs E11.5 * Ctrl
10490856	Ralyl	NM_178631	5.48495e-007	-2.12008	E11.5 * Wnt1 down vs E11.5 * Ctrl
10532085	Tgfbr3	NM_011578	2.72064e-008	-2.12401	E11.5 * Wnt1 down vs E11.5 * Ctrl
10479973	ENSMUSG0000C	ENSMUST00000(	0.0026049	-2.12473	E11.5 * Wnt1 down vs E11.5 * Ctrl
10434806	Lpp	NM_178665	1.75878e-006	-2.12853	E11.5 * Wnt1 down vs E11.5 * Ctrl
10455112	Pcdhb17	NM_053142	8.03472e-006	-2.1323	E11.5 * Wnt1 down vs E11.5 * Ctrl
10537834	Arhgef5	NM_133674	6.12774e-008	-2.13677	E11.5 * Wnt1 down vs E11.5 * Ctrl
10475890	Mertk	NM_008587	3.65412e-007	-2.14011	E11.5 * Wnt1 down vs E11.5 * Ctrl
10455761	Prdm6	NM_001033281	7.23654e-005	-2.14232	E11.5 * Wnt1 down vs E11.5 * Ctrl
10540207	Adamts9	ENSMUST00000(	1.07167e-006	-2.14337	E11.5 * Wnt1 down vs E11.5 * Ctrl
10545707	Actg2	NM_009610	8.68694e-005	-2.14536	E11.5 * Wnt1 down vs E11.5 * Ctrl
10398358		---	0.0012378	-2.14614	E11.5 * Wnt1 down vs E11.5 * Ctrl
10502552	Clca1	NM_009899	0.00016331	-2.14674	E11.5 * Wnt1 down vs E11.5 * Ctrl
10398408		---	0.00150073	-2.15633	E11.5 * Wnt1 down vs E11.5 * Ctrl
10605503		---	0.00629994	-2.15858	E11.5 * Wnt1 down vs E11.5 * Ctrl
10396402	Prkch	NM_008856	1.61708e-007	-2.15962	E11.5 * Wnt1 down vs E11.5 * Ctrl
10445291		---	0.00013398	-2.16203	E11.5 * Wnt1 down vs E11.5 * Ctrl
10478525	Wfdc2	NM_026323	2.82723e-006	-2.16267	E11.5 * Wnt1 down vs E11.5 * Ctrl
10465916	Gm98	BC157942	3.73114e-007	-2.16719	E11.5 * Wnt1 down vs E11.5 * Ctrl
10528207	Cd36	NM_001159557	5.29422e-007	-2.1696	E11.5 * Wnt1 down vs E11.5 * Ctrl
10398360		---	0.000230644	-2.17602	E11.5 * Wnt1 down vs E11.5 * Ctrl
10592420	AW551984	NM_178737	1.20593e-008	-2.17645	E11.5 * Wnt1 down vs E11.5 * Ctrl

10583021	Pdgfd	NM_027924	5.65263e-006	-2.17762	E11.5 * Wnt1 down vs E11.5 * Ctrl
10384504	Meis1	NM_010789	5.16064e-009	-2.18513	E11.5 * Wnt1 down vs E11.5 * Ctrl
10514275	Ptplad2	NM_025760	5.00556e-006	-2.18715	E11.5 * Wnt1 down vs E11.5 * Ctrl
10503334	Gem	NM_010276	2.02174e-005	-2.18814	E11.5 * Wnt1 down vs E11.5 * Ctrl
10598507	Slc38a5	NM_172479	3.73385e-007	-2.18903	E11.5 * Wnt1 down vs E11.5 * Ctrl
10372028	Plxnc1	NM_018797	5.13794e-009	-2.19006	E11.5 * Wnt1 down vs E11.5 * Ctrl
10351546	Apoa2	NM_013474	0.00333965	-2.19317	E11.5 * Wnt1 down vs E11.5 * Ctrl
10502766	Lphn2	NM_001081298	0.000640595	-2.19383	E11.5 * Wnt1 down vs E11.5 * Ctrl
10368970	Prdm1	NM_007548	4.8063e-007	-2.19394	E11.5 * Wnt1 down vs E11.5 * Ctrl
10392560	Abca9	NM_147220	1.35694e-008	-2.19491	E11.5 * Wnt1 down vs E11.5 * Ctrl
10511429	Car8	NM_007592	2.26387e-006	-2.19503	E11.5 * Wnt1 down vs E11.5 * Ctrl
10485643	AI314831	NR_015462	3.29077e-005	-2.20558	E11.5 * Wnt1 down vs E11.5 * Ctrl
10360070	Fcer1g	NM_010185	4.29408e-006	-2.20621	E11.5 * Wnt1 down vs E11.5 * Ctrl
10545409	Vamp8	NM_016794	1.36201e-006	-2.2163	E11.5 * Wnt1 down vs E11.5 * Ctrl
10380637	Hoxb6	NM_008269	1.83716e-008	-2.21649	E11.5 * Wnt1 down vs E11.5 * Ctrl
10574027	Mt1	NM_013602	4.2385e-005	-2.21734	E11.5 * Wnt1 down vs E11.5 * Ctrl
10429128	Sla	NM_001029841	5.35088e-005	-2.21812	E11.5 * Wnt1 down vs E11.5 * Ctrl
10400844	Pygl	NM_133198	5.14093e-008	-2.22209	E11.5 * Wnt1 down vs E11.5 * Ctrl
10407122		---	0.00455997	-2.22317	E11.5 * Wnt1 down vs E11.5 * Ctrl
10466210	Ms4a6d	NM_026835	2.38548e-007	-2.22454	E11.5 * Wnt1 down vs E11.5 * Ctrl
10423556	Pgcp	NM_018755	2.50962e-007	-2.22862	E11.5 * Wnt1 down vs E11.5 * Ctrl
10436392	Cpox	NM_007757	3.85056e-007	-2.22891	E11.5 * Wnt1 down vs E11.5 * Ctrl
10590842	ENSMUSG0000C	ENSMUST00000(	0.0026411	-2.22909	E11.5 * Wnt1 down vs E11.5 * Ctrl
10434802	Lpp	BC005613	3.61892e-005	-2.23068	E11.5 * Wnt1 down vs E11.5 * Ctrl
10415651	Fgf9	NM_013518	5.61352e-007	-2.23275	E11.5 * Wnt1 down vs E11.5 * Ctrl
10601360	Atp7a	NM_001109757	1.4023e-009	-2.23344	E11.5 * Wnt1 down vs E11.5 * Ctrl
10350840	Angptl1	NM_028333	1.88229e-006	-2.24121	E11.5 * Wnt1 down vs E11.5 * Ctrl
10527638	Alox5ap	NM_009663	6.03301e-006	-2.24582	E11.5 * Wnt1 down vs E11.5 * Ctrl
10366476	Ptprb	NM_029928	3.1343e-008	-2.24639	E11.5 * Wnt1 down vs E11.5 * Ctrl
10427904	Fbxl7	BC050864	5.4879e-006	-2.24703	E11.5 * Wnt1 down vs E11.5 * Ctrl
10367822	Rab32	NM_026405	7.78561e-006	-2.24979	E11.5 * Wnt1 down vs E11.5 * Ctrl
10498972	Rbm46	NM_001146328	2.11134e-006	-2.25632	E11.5 * Wnt1 down vs E11.5 * Ctrl
10406519	Hapln1	NM_013500	7.14686e-006	-2.25724	E11.5 * Wnt1 down vs E11.5 * Ctrl
10544768	Hoxa5	NM_010453	3.11037e-005	-2.25731	E11.5 * Wnt1 down vs E11.5 * Ctrl
10548761	Hebp1	NM_013546	2.81067e-006	-2.26057	E11.5 * Wnt1 down vs E11.5 * Ctrl
10359861	Mgst3	NM_025569	3.25186e-006	-2.26076	E11.5 * Wnt1 down vs E11.5 * Ctrl
10529875	Ldb2	NM_001077398	2.63993e-007	-2.26221	E11.5 * Wnt1 down vs E11.5 * Ctrl
10424140	Col14a1	NM_181277	1.17642e-006	-2.27948	E11.5 * Wnt1 down vs E11.5 * Ctrl
10502863	Ak5	NM_001081277	4.7413e-008	-2.28025	E11.5 * Wnt1 down vs E11.5 * Ctrl
10463070	Entpd1	NM_009848	3.06238e-005	-2.28044	E11.5 * Wnt1 down vs E11.5 * Ctrl
10563441	Emp3	NM_010129	5.83508e-007	-2.28096	E11.5 * Wnt1 down vs E11.5 * Ctrl
10542594	ENSMUSG0000C	AY512955	4.68094e-006	-2.28126	E11.5 * Wnt1 down vs E11.5 * Ctrl
10362113	ENSMUSG0000C	ENSMUST00000	4.16025e-006	-2.28643	E11.5 * Wnt1 down vs E11.5 * Ctrl
10519998	Lrrc17	NM_028977	4.51076e-008	-2.28843	E11.5 * Wnt1 down vs E11.5 * Ctrl
10498018	Pcdh18	NM_130448	2.40042e-009	-2.28987	E11.5 * Wnt1 down vs E11.5 * Ctrl
10527713	Rxfp2	NM_080468	1.95994e-010	-2.29008	E11.5 * Wnt1 down vs E11.5 * Ctrl
10418355	Cacna1d	NM_028981	1.20216e-009	-2.30064	E11.5 * Wnt1 down vs E11.5 * Ctrl
10583529	Icam4	NM_023892	1.19746e-008	-2.30116	E11.5 * Wnt1 down vs E11.5 * Ctrl
10463428		---	0.000518766	-2.30867	E11.5 * Wnt1 down vs E11.5 * Ctrl
10413482	Wnt5a	NM_009524	2.43138e-005	-2.30904	E11.5 * Wnt1 down vs E11.5 * Ctrl
10408525	1700018A04Rik	ENSMUST00000(	1.30636e-007	-2.30922	E11.5 * Wnt1 down vs E11.5 * Ctrl
10495794	Pde5a	NM_153422	4.82148e-012	-2.31032	E11.5 * Wnt1 down vs E11.5 * Ctrl
10379530	Ccl12	NM_011331	1.75093e-006	-2.31325	E11.5 * Wnt1 down vs E11.5 * Ctrl
10502748	Lphn2	NM_001081298	3.47359e-010	-2.31357	E11.5 * Wnt1 down vs E11.5 * Ctrl
10453049	Cdc42ep3	NM_026514	6.67103e-006	-2.31523	E11.5 * Wnt1 down vs E11.5 * Ctrl
10576046	Foxf1a	NM_010426	1.79001e-005	-2.31945	E11.5 * Wnt1 down vs E11.5 * Ctrl
10591135	Fat3	NM_001080814	2.0393e-005	-2.32139	E11.5 * Wnt1 down vs E11.5 * Ctrl
10537849	Arhgef5	NM_133674	5.25302e-008	-2.32242	E11.5 * Wnt1 down vs E11.5 * Ctrl
10529824	Prom1	NM_008935	2.9028e-010	-2.32287	E11.5 * Wnt1 down vs E11.5 * Ctrl
10523579	Arhgap24	NM_029270	1.26643e-009	-2.323	E11.5 * Wnt1 down vs E11.5 * Ctrl
10440099	St3gal6	NM_018784	1.12273e-007	-2.32479	E11.5 * Wnt1 down vs E11.5 * Ctrl
10506031	Nfia	NM_010905	2.79533e-006	-2.32621	E11.5 * Wnt1 down vs E11.5 * Ctrl
10485840	Ryr3	NM_177652	1.86606e-011	-2.33295	E11.5 * Wnt1 down vs E11.5 * Ctrl
10376778	Mfap4	NM_029568	9.8852e-008	-2.33311	E11.5 * Wnt1 down vs E11.5 * Ctrl
10578880	Tll1	NM_009390	2.56765e-006	-2.33455	E11.5 * Wnt1 down vs E11.5 * Ctrl
10583809	Cnn1	NM_009922	9.67663e-005	-2.3347	E11.5 * Wnt1 down vs E11.5 * Ctrl

10549420	Tmtc1	NM_198967	1.41288e-008	-2.33494	E11.5 * Wnt1 down vs E11.5 * Ctrl
10513362	OTTMUSG00000	ENSMUST00000	1.77662e-008	-2.3396	E11.5 * Wnt1 down vs E11.5 * Ctrl
10502778	Lphn2	NM_001081298	1.14636e-006	-2.34363	E11.5 * Wnt1 down vs E11.5 * Ctrl
10601980	Mum1l1	NM_175541	5.07211e-006	-2.3442	E11.5 * Wnt1 down vs E11.5 * Ctrl
10351867	Aim2	NM_001013779	3.7998e-005	-2.34775	E11.5 * Wnt1 down vs E11.5 * Ctrl
10592067	Fli1	NM_008026	6.65034e-007	-2.34968	E11.5 * Wnt1 down vs E11.5 * Ctrl
10402390	Serpina1b	NM_009244	0.00302558	-2.35125	E11.5 * Wnt1 down vs E11.5 * Ctrl
10584593		---	2.84458e-005	-2.35457	E11.5 * Wnt1 down vs E11.5 * Ctrl
10568392	Rgs10	NM_026418	3.19237e-009	-2.36699	E11.5 * Wnt1 down vs E11.5 * Ctrl
10599680	3830403N18Rik	NM_027510	1.08763e-007	-2.37282	E11.5 * Wnt1 down vs E11.5 * Ctrl
10484389	Tfpi	NM_011576	8.19091e-011	-2.37722	E11.5 * Wnt1 down vs E11.5 * Ctrl
10601993	D330045A20Rik	NM_175326	1.29904e-006	-2.37879	E11.5 * Wnt1 down vs E11.5 * Ctrl
10501802	Tmem56	NM_178936	4.71898e-008	-2.37913	E11.5 * Wnt1 down vs E11.5 * Ctrl
10531197	Adamts3	NM_001081401	5.30789e-007	-2.3836	E11.5 * Wnt1 down vs E11.5 * Ctrl
10346015	Col3a1	NM_009930	3.56884e-008	-2.38811	E11.5 * Wnt1 down vs E11.5 * Ctrl
10474295	Wt1	NM_144783	2.4701e-008	-2.39126	E11.5 * Wnt1 down vs E11.5 * Ctrl
10497590	Evi1	NM_007963	3.02564e-006	-2.39973	E11.5 * Wnt1 down vs E11.5 * Ctrl
10519983	Fgl2	NM_008013	2.34571e-007	-2.40814	E11.5 * Wnt1 down vs E11.5 * Ctrl
10544487	Tpk1	NM_013861	5.40514e-006	-2.40876	E11.5 * Wnt1 down vs E11.5 * Ctrl
10406736	F2rl2	NM_010170	1.07277e-006	-2.41016	E11.5 * Wnt1 down vs E11.5 * Ctrl
10527959		---	5.38144e-005	-2.412	E11.5 * Wnt1 down vs E11.5 * Ctrl
10360040	Fcgr3	NM_010188	5.52664e-008	-2.41449	E11.5 * Wnt1 down vs E11.5 * Ctrl
10604528	Mbnl3	NM_134163	6.81571e-007	-2.4147	E11.5 * Wnt1 down vs E11.5 * Ctrl
10500276	BC028528	BC028528	2.14432e-009	-2.4154	E11.5 * Wnt1 down vs E11.5 * Ctrl
10549877		---	3.62346e-006	-2.41754	E11.5 * Wnt1 down vs E11.5 * Ctrl
10382106	Gm885	NM_001033435	1.09894e-006	-2.41942	E11.5 * Wnt1 down vs E11.5 * Ctrl
10530536	Tec	NM_001113460	6.27302e-007	-2.42189	E11.5 * Wnt1 down vs E11.5 * Ctrl
10447383	Epcam	NM_008532	0.000142578	-2.42395	E11.5 * Wnt1 down vs E11.5 * Ctrl
10357833	Atp2b4	NM_213616	1.99487e-008	-2.4254	E11.5 * Wnt1 down vs E11.5 * Ctrl
10581961	Adamts18	NM_172466	3.83054e-007	-2.42574	E11.5 * Wnt1 down vs E11.5 * Ctrl
10404649	Dsp	NM_023842	1.4065e-007	-2.42621	E11.5 * Wnt1 down vs E11.5 * Ctrl
10546434	Adamts9	NM_175314	1.95122e-006	-2.42811	E11.5 * Wnt1 down vs E11.5 * Ctrl
10363762	Tmem26	NM_177794	4.28329e-008	-2.43003	E11.5 * Wnt1 down vs E11.5 * Ctrl
10402705	Gm266	NM_001033248	1.36527e-005	-2.43196	E11.5 * Wnt1 down vs E11.5 * Ctrl
10517508	C1qb	NM_009777	6.80175e-005	-2.43267	E11.5 * Wnt1 down vs E11.5 * Ctrl
10577641	1810011O10Rik	NM_026931	7.66024e-005	-2.43796	E11.5 * Wnt1 down vs E11.5 * Ctrl
10487011	Gatm	NM_025961	4.97235e-009	-2.43844	E11.5 * Wnt1 down vs E11.5 * Ctrl
10360398	Ifi202b	NM_008327	0.000106147	-2.44102	E11.5 * Wnt1 down vs E11.5 * Ctrl
10404404	Foxf2	NM_010225	1.67824e-006	-2.44284	E11.5 * Wnt1 down vs E11.5 * Ctrl
10398368		---	3.95509e-008	-2.44745	E11.5 * Wnt1 down vs E11.5 * Ctrl
10413928	1810011H11Rik	AK007434	8.78762e-006	-2.44883	E11.5 * Wnt1 down vs E11.5 * Ctrl
10492798	Sfrp2	NM_009144	2.49894e-009	-2.45444	E11.5 * Wnt1 down vs E11.5 * Ctrl
10440340		---	1.29541e-008	-2.45972	E11.5 * Wnt1 down vs E11.5 * Ctrl
10525030	Tbx5	NM_011537	2.93892e-007	-2.46545	E11.5 * Wnt1 down vs E11.5 * Ctrl
10374366	Egfr	NM_207655	1.22892e-007	-2.4689	E11.5 * Wnt1 down vs E11.5 * Ctrl
10584561	9030425E11Rik	NM_133733	5.67544e-005	-2.47412	E11.5 * Wnt1 down vs E11.5 * Ctrl
10433484	Atf7ip2	NM_153123	2.94296e-005	-2.47635	E11.5 * Wnt1 down vs E11.5 * Ctrl
10363696	D630028G08Rik	AK038706	5.79616e-007	-2.47695	E11.5 * Wnt1 down vs E11.5 * Ctrl
10586118	Calml4	NM_138304	0.000282019	-2.477	E11.5 * Wnt1 down vs E11.5 * Ctrl
10454172	Dsg2	NM_007883	4.42321e-007	-2.47931	E11.5 * Wnt1 down vs E11.5 * Ctrl
10502240	Npnt	NM_033525	2.0488e-007	-2.4822	E11.5 * Wnt1 down vs E11.5 * Ctrl
10469457	Plxdc2	NM_026162	4.41667e-011	-2.48603	E11.5 * Wnt1 down vs E11.5 * Ctrl
10591125	Fat3	NM_001080814	1.11501e-005	-2.48678	E11.5 * Wnt1 down vs E11.5 * Ctrl
10362314	Ptprk	NM_008983	3.86833e-010	-2.48733	E11.5 * Wnt1 down vs E11.5 * Ctrl
10602372	Alas2	NM_009653	1.16916e-009	-2.48834	E11.5 * Wnt1 down vs E11.5 * Ctrl
10436600		---	0.0061147	-2.49403	E11.5 * Wnt1 down vs E11.5 * Ctrl
10399801	Sntg2	NM_172951	3.66041e-008	-2.49419	E11.5 * Wnt1 down vs E11.5 * Ctrl
10460541	Cd248	NM_054042	1.36141e-006	-2.49651	E11.5 * Wnt1 down vs E11.5 * Ctrl
10559816		---	1.06849e-006	-2.51315	E11.5 * Wnt1 down vs E11.5 * Ctrl
10502780	Lphn2	NM_001081298	3.88934e-010	-2.52121	E11.5 * Wnt1 down vs E11.5 * Ctrl
10407350	Fgf10	NM_008002	4.29882e-007	-2.52575	E11.5 * Wnt1 down vs E11.5 * Ctrl
10455919	Adamts19	NM_175506	1.2756e-008	-2.52608	E11.5 * Wnt1 down vs E11.5 * Ctrl
10564527	Nr2f2	NM_009697	1.23268e-010	-2.52785	E11.5 * Wnt1 down vs E11.5 * Ctrl
10358434	Pla2g4a	NM_008869	4.64871e-007	-2.52851	E11.5 * Wnt1 down vs E11.5 * Ctrl
10543306	Tspan12	NM_173007	2.5793e-008	-2.52851	E11.5 * Wnt1 down vs E11.5 * Ctrl
10351111	Dnm3os	NR_002870	3.4148e-007	-2.52876	E11.5 * Wnt1 down vs E11.5 * Ctrl

10501586	S1pr1	NM_007901	1.20034e-006	-2.52886	E11.5 * Wnt1 down vs E11.5 * Ctrl
10587446	Myo6	NM_001039546	3.48142e-008	-2.52891	E11.5 * Wnt1 down vs E11.5 * Ctrl
10398354		---	0.000140656	-2.53208	E11.5 * Wnt1 down vs E11.5 * Ctrl
10595633	Bcl2a1d	NM_007536	3.37604e-007	-2.53477	E11.5 * Wnt1 down vs E11.5 * Ctrl
10362294	Arhgap18	NM_176837	8.26903e-008	-2.53631	E11.5 * Wnt1 down vs E11.5 * Ctrl
10536499	Cav1	NM_007616	5.3569e-005	-2.53697	E11.5 * Wnt1 down vs E11.5 * Ctrl
10564818	Anpep	NM_008486	2.75021e-005	-2.54143	E11.5 * Wnt1 down vs E11.5 * Ctrl
10398364		---	4.02793e-005	-2.55172	E11.5 * Wnt1 down vs E11.5 * Ctrl
10502774	Lphn2	NM_001081298	1.68086e-011	-2.55451	E11.5 * Wnt1 down vs E11.5 * Ctrl
10531195	Adamts3	NM_001081401	9.26187e-005	-2.5568	E11.5 * Wnt1 down vs E11.5 * Ctrl
10521700	ENSMUSG0000(	ENSMUST00000(	0.000111521	-2.55787	E11.5 * Wnt1 down vs E11.5 * Ctrl
10374333	Ikzf1	NM_001025597	4.57586e-008	-2.55954	E11.5 * Wnt1 down vs E11.5 * Ctrl
10506820		---	0.000126003	-2.56325	E11.5 * Wnt1 down vs E11.5 * Ctrl
10432918	Krt8	NM_031170	3.6308e-006	-2.56375	E11.5 * Wnt1 down vs E11.5 * Ctrl
10372652	Lyz1	NM_013590	5.99831e-006	-2.56939	E11.5 * Wnt1 down vs E11.5 * Ctrl
10571601	Pdlim3	NM_016798	5.7959e-009	-2.58143	E11.5 * Wnt1 down vs E11.5 * Ctrl
10492689	Pdgfc	NM_019971	3.52005e-008	-2.59002	E11.5 * Wnt1 down vs E11.5 * Ctrl
10351623	F11r	NM_172647	6.5183e-006	-2.59078	E11.5 * Wnt1 down vs E11.5 * Ctrl
10587690	Bcl2a1b	NM_007534	4.34441e-007	-2.59367	E11.5 * Wnt1 down vs E11.5 * Ctrl
10555297	Kcne3	NM_020574	7.82837e-009	-2.5942	E11.5 * Wnt1 down vs E11.5 * Ctrl
10445046	Trim10	NM_011280	1.42908e-009	-2.59984	E11.5 * Wnt1 down vs E11.5 * Ctrl
10535807	Flt1	NM_010228	1.40882e-011	-2.59994	E11.5 * Wnt1 down vs E11.5 * Ctrl
10352152	Kif26b	NM_177757	6.05607e-008	-2.60797	E11.5 * Wnt1 down vs E11.5 * Ctrl
10571788	Vegfc	NM_009506	1.70657e-006	-2.61154	E11.5 * Wnt1 down vs E11.5 * Ctrl
10591116	Fat3	NM_001080814	5.64302e-009	-2.61708	E11.5 * Wnt1 down vs E11.5 * Ctrl
10548038	Ntf3	NM_008742	8.37605e-007	-2.62061	E11.5 * Wnt1 down vs E11.5 * Ctrl
10398362	Rian	AF357355	7.22833e-005	-2.63144	E11.5 * Wnt1 down vs E11.5 * Ctrl
10531203	Adamts3	NM_001081401	1.17134e-007	-2.63523	E11.5 * Wnt1 down vs E11.5 * Ctrl
10606016	Il2rg	NM_013563	3.68152e-006	-2.6389	E11.5 * Wnt1 down vs E11.5 * Ctrl
10352867	Plxna2	NM_008882	7.65424e-010	-2.63975	E11.5 * Wnt1 down vs E11.5 * Ctrl
10514177	Bnc2	NM_172870	2.77136e-008	-2.63977	E11.5 * Wnt1 down vs E11.5 * Ctrl
10344837	Prex2	NM_029525	1.18566e-007	-2.63994	E11.5 * Wnt1 down vs E11.5 * Ctrl
10475544	Sema6d	NM_199241	1.12484e-007	-2.64264	E11.5 * Wnt1 down vs E11.5 * Ctrl
10541075	Cxcl12	NM_001012477	1.47866e-005	-2.6563	E11.5 * Wnt1 down vs E11.5 * Ctrl
10435501	Stfa1	NM_001082543	7.06091e-007	-2.66156	E11.5 * Wnt1 down vs E11.5 * Ctrl
10486664	Epb4.2	NM_013513	6.52076e-008	-2.66343	E11.5 * Wnt1 down vs E11.5 * Ctrl
10400143	Stxbp6	NM_144552	1.92615e-008	-2.664	E11.5 * Wnt1 down vs E11.5 * Ctrl
10523062	Alb	NM_009654	0.00338407	-2.66824	E11.5 * Wnt1 down vs E11.5 * Ctrl
10515848	Ermap	NM_013848	2.46545e-009	-2.66881	E11.5 * Wnt1 down vs E11.5 * Ctrl
10494548	Gja5	NM_008121	1.47841e-007	-2.67139	E11.5 * Wnt1 down vs E11.5 * Ctrl
10543239	Tcfec	NM_031198	7.23033e-006	-2.67165	E11.5 * Wnt1 down vs E11.5 * Ctrl
10591131	Fat3	NM_001080814	8.56749e-007	-2.67299	E11.5 * Wnt1 down vs E11.5 * Ctrl
10559207	Lsp1	NM_019391	3.98028e-008	-2.683	E11.5 * Wnt1 down vs E11.5 * Ctrl
10514070	2310067E19Rik	BC066147	4.16849e-007	-2.69651	E11.5 * Wnt1 down vs E11.5 * Ctrl
10594044	Islr	NM_012043	3.68703e-007	-2.70029	E11.5 * Wnt1 down vs E11.5 * Ctrl
10439500	Upk1b	NM_178924	4.33415e-005	-2.70617	E11.5 * Wnt1 down vs E11.5 * Ctrl
10384458	Plek	NM_019549	1.01173e-006	-2.70941	E11.5 * Wnt1 down vs E11.5 * Ctrl
10396936	Smoc1	NM_001146217	7.0494e-007	-2.72149	E11.5 * Wnt1 down vs E11.5 * Ctrl
10501903	Synpo2	NM_080451	5.55181e-008	-2.72276	E11.5 * Wnt1 down vs E11.5 * Ctrl
10412345	Parp8	NM_001081009	9.42949e-009	-2.72703	E11.5 * Wnt1 down vs E11.5 * Ctrl
10453057	Cyp1b1	NM_009994	5.56259e-007	-2.73613	E11.5 * Wnt1 down vs E11.5 * Ctrl
10587683	Bcl2a1a	NM_009742	5.69606e-006	-2.73694	E11.5 * Wnt1 down vs E11.5 * Ctrl
10586744	Anxa2	NM_007585	6.4942e-006	-2.74144	E11.5 * Wnt1 down vs E11.5 * Ctrl
10510239	ENSMUSG0000(	ENSMUST00000	5.12468e-005	-2.74162	E11.5 * Wnt1 down vs E11.5 * Ctrl
10584595	2610203C20Rik	ENSMUST00000(	5.21345e-006	-2.74277	E11.5 * Wnt1 down vs E11.5 * Ctrl
10604347	Smarca1	NM_053123	3.72519e-009	-2.75041	E11.5 * Wnt1 down vs E11.5 * Ctrl
10566358	Trim30	NM_009099	9.75086e-007	-2.75068	E11.5 * Wnt1 down vs E11.5 * Ctrl
10467840	C130021O09Rik	AK081501	6.41758e-005	-2.7507	E11.5 * Wnt1 down vs E11.5 * Ctrl
10578123	Rbpms	NM_019733	2.3642e-010	-2.75414	E11.5 * Wnt1 down vs E11.5 * Ctrl
10436598	2810055G20Rik	ENSMUST00000(	0.00185599	-2.75503	E11.5 * Wnt1 down vs E11.5 * Ctrl
10597743	Cx3cr1	NM_009987	6.42699e-007	-2.76766	E11.5 * Wnt1 down vs E11.5 * Ctrl
10446282	Emr1	NM_010130	7.65655e-007	-2.76907	E11.5 * Wnt1 down vs E11.5 * Ctrl
10389231	Ccl3	NM_011337	1.0197e-005	-2.77889	E11.5 * Wnt1 down vs E11.5 * Ctrl
10411235	Iqgap2	NM_027711	1.66969e-007	-2.77902	E11.5 * Wnt1 down vs E11.5 * Ctrl
10453747	Colec12	NM_130449	1.16912e-007	-2.78066	E11.5 * Wnt1 down vs E11.5 * Ctrl
10606355	Cysltr1	NM_021476	3.13496e-008	-2.78126	E11.5 * Wnt1 down vs E11.5 * Ctrl

10559796	Peg3	NM_008817	4.96487e-007	-2.78931	E11.5 * Wnt1 down vs E11.5 * Ctrl
10461721	Mpeg1	NM_010821	1.48653e-006	-2.79224	E11.5 * Wnt1 down vs E11.5 * Ctrl
10344952	Rdh10	NM_133832	2.46072e-005	-2.80633	E11.5 * Wnt1 down vs E11.5 * Ctrl
10362091	Raet1d	NM_020030	6.30845e-005	-2.80796	E11.5 * Wnt1 down vs E11.5 * Ctrl
10458828	Cdo1	NM_033037	4.20565e-009	-2.80904	E11.5 * Wnt1 down vs E11.5 * Ctrl
10559649	Cox6b2	NM_183405	7.01799e-006	-2.81352	E11.5 * Wnt1 down vs E11.5 * Ctrl
10407286	BC067074	ENSMUST00000(	1.40267e-005	-2.81742	E11.5 * Wnt1 down vs E11.5 * Ctrl
10467319	Rbp4	NM_001159487	1.63838e-006	-2.81852	E11.5 * Wnt1 down vs E11.5 * Ctrl
10536324	Asb4	NM_023048	2.75767e-007	-2.82069	E11.5 * Wnt1 down vs E11.5 * Ctrl
10427336	Nckap1l	NM_153505	4.89254e-007	-2.82712	E11.5 * Wnt1 down vs E11.5 * Ctrl
10499132	Mab21l2	NM_011839	5.45513e-007	-2.82734	E11.5 * Wnt1 down vs E11.5 * Ctrl
10351455	Rgs5	NM_009063	9.19387e-006	-2.82929	E11.5 * Wnt1 down vs E11.5 * Ctrl
10484201	2610301F02Rik	ENSMUST00000(	1.3638e-006	-2.83402	E11.5 * Wnt1 down vs E11.5 * Ctrl
10495685	Arhgap29	NM_172525	6.28566e-007	-2.84143	E11.5 * Wnt1 down vs E11.5 * Ctrl
10513158	Ptpn3	NM_011207	2.61313e-005	-2.84856	E11.5 * Wnt1 down vs E11.5 * Ctrl
10554808	Fzd4	NM_008055	1.07129e-007	-2.85547	E11.5 * Wnt1 down vs E11.5 * Ctrl
10514054	Nfib	NM_001113209	7.84345e-011	-2.86497	E11.5 * Wnt1 down vs E11.5 * Ctrl
10360415	Grem2	NM_011825	1.03206e-008	-2.86686	E11.5 * Wnt1 down vs E11.5 * Ctrl
10543959	Ptn	NM_008973	6.10991e-008	-2.90762	E11.5 * Wnt1 down vs E11.5 * Ctrl
10453857	Gata6	NM_010258	3.15115e-007	-2.90787	E11.5 * Wnt1 down vs E11.5 * Ctrl
10506050	Nfia	NM_001122952	3.84943e-007	-2.90844	E11.5 * Wnt1 down vs E11.5 * Ctrl
10498119	Frem2	NM_172862	3.42164e-008	-2.91066	E11.5 * Wnt1 down vs E11.5 * Ctrl
10496872	Eltd1	NM_133222	9.43022e-006	-2.93135	E11.5 * Wnt1 down vs E11.5 * Ctrl
10513957	Ptprd	NM_011211	4.57414e-012	-2.93177	E11.5 * Wnt1 down vs E11.5 * Ctrl
10484207	2610301F02Rik	NM_001025576	1.46287e-011	-2.93207	E11.5 * Wnt1 down vs E11.5 * Ctrl
10523078	Afp	NM_007423	0.00121003	-2.94356	E11.5 * Wnt1 down vs E11.5 * Ctrl
10380571	Gngt2	NM_023121	2.57234e-007	-2.94459	E11.5 * Wnt1 down vs E11.5 * Ctrl
10428707	Has2	NM_008216	7.46032e-009	-2.95226	E11.5 * Wnt1 down vs E11.5 * Ctrl
10427286	Hoxc9	NM_008272	6.75696e-010	-2.9548	E11.5 * Wnt1 down vs E11.5 * Ctrl
10513739	Tnc	NM_011607	1.0895e-007	-2.95676	E11.5 * Wnt1 down vs E11.5 * Ctrl
10412335	Isl1	NM_021459	4.02903e-006	-2.96105	E11.5 * Wnt1 down vs E11.5 * Ctrl
10423548	Sdc2	NM_008304	1.71471e-008	-2.96641	E11.5 * Wnt1 down vs E11.5 * Ctrl
10522288	Shisa3	NM_001033415	4.37162e-010	-2.96683	E11.5 * Wnt1 down vs E11.5 * Ctrl
10565204	Bnc1	NM_007562	8.56835e-009	-2.99923	E11.5 * Wnt1 down vs E11.5 * Ctrl
10536444	Foxp2	NM_053242	2.08994e-008	-3.01973	E11.5 * Wnt1 down vs E11.5 * Ctrl
10502772	Lphn2	NM_001081298	3.75138e-010	-3.02214	E11.5 * Wnt1 down vs E11.5 * Ctrl
10519857	Hgf	NM_010427	8.51602e-010	-3.02981	E11.5 * Wnt1 down vs E11.5 * Ctrl
10440019	Tmem45a	NM_019631	3.60944e-006	-3.05555	E11.5 * Wnt1 down vs E11.5 * Ctrl
10590635	Ccr5	NM_009917	3.59193e-007	-3.05562	E11.5 * Wnt1 down vs E11.5 * Ctrl
10598013	Ccr5	NM_009917	3.59193e-007	-3.05562	E11.5 * Wnt1 down vs E11.5 * Ctrl
10427075	Krt18	NM_010664	1.42901e-007	-3.06492	E11.5 * Wnt1 down vs E11.5 * Ctrl
10354374	Slc40a1	NM_016917	3.07819e-009	-3.07248	E11.5 * Wnt1 down vs E11.5 * Ctrl
10513630	Ambp	NM_007443	0.00017866	-3.09066	E11.5 * Wnt1 down vs E11.5 * Ctrl
10548879	Mgp	NM_008597	3.31469e-007	-3.09761	E11.5 * Wnt1 down vs E11.5 * Ctrl
10381298	Ramp2	NM_019444	4.81561e-007	-3.11305	E11.5 * Wnt1 down vs E11.5 * Ctrl
10546454	Adamts9	NM_175314	2.27195e-008	-3.11814	E11.5 * Wnt1 down vs E11.5 * Ctrl
10503054	Lrrc7	NM_001081358	1.82412e-005	-3.12009	E11.5 * Wnt1 down vs E11.5 * Ctrl
10509002	Rhd	NM_011270	4.32345e-010	-3.12484	E11.5 * Wnt1 down vs E11.5 * Ctrl
10538126	Gimap4	NM_174990	8.95405e-008	-3.12815	E11.5 * Wnt1 down vs E11.5 * Ctrl
10421172	Slc25a37	NM_026331	3.52189e-008	-3.1359	E11.5 * Wnt1 down vs E11.5 * Ctrl
10569335	H19	NR_001592	4.20723e-005	-3.1472	E11.5 * Wnt1 down vs E11.5 * Ctrl
10358339	Cfh	NM_009888	5.65588e-009	-3.164	E11.5 * Wnt1 down vs E11.5 * Ctrl
10369842		---	0.000117399	-3.16476	E11.5 * Wnt1 down vs E11.5 * Ctrl
10441864	Mllt4	NM_010806	4.86927e-013	-3.16906	E11.5 * Wnt1 down vs E11.5 * Ctrl
10502770	Lphn2	NM_001081298	1.64893e-005	-3.17319	E11.5 * Wnt1 down vs E11.5 * Ctrl
10546430	Adamts9	NM_175314	5.29048e-006	-3.1794	E11.5 * Wnt1 down vs E11.5 * Ctrl
10483800		---	2.55005e-007	-3.19028	E11.5 * Wnt1 down vs E11.5 * Ctrl
10503382	Runx1t1	NM_001111027	1.50993e-007	-3.19594	E11.5 * Wnt1 down vs E11.5 * Ctrl
10601569	Pcdh11x	NM_001081385	5.37855e-005	-3.19998	E11.5 * Wnt1 down vs E11.5 * Ctrl
10348451	Cxcr7	NM_007722	5.60198e-009	-3.20186	E11.5 * Wnt1 down vs E11.5 * Ctrl
10467838		---	8.16899e-006	-3.22464	E11.5 * Wnt1 down vs E11.5 * Ctrl
10578264	Msr1	NM_031195	1.945e-008	-3.22615	E11.5 * Wnt1 down vs E11.5 * Ctrl
10595211	Col12a1	NM_007730	6.75703e-008	-3.23069	E11.5 * Wnt1 down vs E11.5 * Ctrl
10408693	F13a1	NM_028784	2.76632e-007	-3.23212	E11.5 * Wnt1 down vs E11.5 * Ctrl
10522530	Kit	NM_001122733	2.63153e-010	-3.23791	E11.5 * Wnt1 down vs E11.5 * Ctrl
10607484	Ptchd1	NM_001093750	4.92524e-006	-3.25995	E11.5 * Wnt1 down vs E11.5 * Ctrl

10591118	Fat3	NM_001080814	2.18686e-006	-3.2818	E11.5 * Wnt1 down vs E11.5 * Ctrl
10531177	Adamts3	NM_001081401	2.37193e-008	-3.28722	E11.5 * Wnt1 down vs E11.5 * Ctrl
10467124	Acta2	NM_007392	0.000621655	-3.30344	E11.5 * Wnt1 down vs E11.5 * Ctrl
10591127	Fat3	NM_001080814	1.03616e-005	-3.32069	E11.5 * Wnt1 down vs E11.5 * Ctrl
10405587	Tgfbi	NM_009369	2.96516e-007	-3.32688	E11.5 * Wnt1 down vs E11.5 * Ctrl
10427293	Hoxc6	NM_010465	2.42624e-006	-3.32765	E11.5 * Wnt1 down vs E11.5 * Ctrl
10491732	Fat4	NM_183221	1.27372e-007	-3.33231	E11.5 * Wnt1 down vs E11.5 * Ctrl
10546736	Cntn3	NM_008779	1.4001e-008	-3.33277	E11.5 * Wnt1 down vs E11.5 * Ctrl
10412260	Fst	NM_008046	2.32158e-008	-3.34918	E11.5 * Wnt1 down vs E11.5 * Ctrl
10546450	Adamts9	NM_175314	1.88254e-006	-3.35177	E11.5 * Wnt1 down vs E11.5 * Ctrl
10351463	Rgs5	ENSMUST00000(	7.43924e-005	-3.35355	E11.5 * Wnt1 down vs E11.5 * Ctrl
10398366		---	6.29349e-005	-3.38797	E11.5 * Wnt1 down vs E11.5 * Ctrl
10568553	4631426J05Rik	NM_029935	4.37998e-008	-3.39148	E11.5 * Wnt1 down vs E11.5 * Ctrl
10344897	Sulf1	NM_172294	3.36143e-007	-3.40074	E11.5 * Wnt1 down vs E11.5 * Ctrl
10491319	Kcnmb2	NM_028231	2.73993e-008	-3.41686	E11.5 * Wnt1 down vs E11.5 * Ctrl
10355893	Epha4	NM_007936	1.64513e-010	-3.42621	E11.5 * Wnt1 down vs E11.5 * Ctrl
10591129	Fat3	NM_001080814	3.128e-005	-3.42676	E11.5 * Wnt1 down vs E11.5 * Ctrl
10419151	Ear1	NM_007894	1.73318e-005	-3.43295	E11.5 * Wnt1 down vs E11.5 * Ctrl
10545101	Ptgds2	NM_019455	5.45475e-008	-3.43875	E11.5 * Wnt1 down vs E11.5 * Ctrl
10456071	Csf1r	NM_001037859	7.42772e-008	-3.44221	E11.5 * Wnt1 down vs E11.5 * Ctrl
10591739	Acp5	NM_001102404	2.241e-009	-3.47015	E11.5 * Wnt1 down vs E11.5 * Ctrl
10506108		---	3.30891e-005	-3.47028	E11.5 * Wnt1 down vs E11.5 * Ctrl
10376326	Irgm2	NM_019440	1.30945e-009	-3.47102	E11.5 * Wnt1 down vs E11.5 * Ctrl
10584600		---	7.99058e-008	-3.47362	E11.5 * Wnt1 down vs E11.5 * Ctrl
10361771	Plagl1	NM_009538	3.35262e-007	-3.49904	E11.5 * Wnt1 down vs E11.5 * Ctrl
10514049	Nfib	NM_001113209	8.95107e-012	-3.51268	E11.5 * Wnt1 down vs E11.5 * Ctrl
10517165	Cd52	NM_013706	2.47767e-007	-3.51969	E11.5 * Wnt1 down vs E11.5 * Ctrl
10575034	Cdh3	NM_001037809	1.11727e-008	-3.52194	E11.5 * Wnt1 down vs E11.5 * Ctrl
10498653	1110032A04Rik	NM_133675	1.11406e-006	-3.53072	E11.5 * Wnt1 down vs E11.5 * Ctrl
10604393	ENSMUSG0000C	NM_019680	1.89882e-011	-3.53353	E11.5 * Wnt1 down vs E11.5 * Ctrl
10513256	Lpar1	NM_010336	4.86178e-008	-3.53537	E11.5 * Wnt1 down vs E11.5 * Ctrl
10604585		---	1.07062e-008	-3.54171	E11.5 * Wnt1 down vs E11.5 * Ctrl
10572130	Lpl	NM_008509	1.89835e-010	-3.54233	E11.5 * Wnt1 down vs E11.5 * Ctrl
10521972	Pcdh7	NM_018764	2.52353e-011	-3.54238	E11.5 * Wnt1 down vs E11.5 * Ctrl
10523376	Fras1	NM_175473	4.05292e-009	-3.58786	E11.5 * Wnt1 down vs E11.5 * Ctrl
10358224	Ptprc	NM_001111316	3.06225e-007	-3.59132	E11.5 * Wnt1 down vs E11.5 * Ctrl
10443108	Syngap1	XM_985548	5.16329e-005	-3.59442	E11.5 * Wnt1 down vs E11.5 * Ctrl
10419261	Bmp4	NM_007554	2.74281e-009	-3.59473	E11.5 * Wnt1 down vs E11.5 * Ctrl
10607848	Egfl6	NM_019397	5.58497e-007	-3.5966	E11.5 * Wnt1 down vs E11.5 * Ctrl
10386996	Myocd	NM_145136	1.29096e-008	-3.60006	E11.5 * Wnt1 down vs E11.5 * Ctrl
10472953	Hoxd11	NM_008273	1.09408e-007	-3.60918	E11.5 * Wnt1 down vs E11.5 * Ctrl
10376956	Hs3st3a1	NM_178870	5.26421e-008	-3.60952	E11.5 * Wnt1 down vs E11.5 * Ctrl
10372648	Lyz2	NM_017372	7.08376e-008	-3.62571	E11.5 * Wnt1 down vs E11.5 * Ctrl
10553274	Saa2	NM_011314	1.028e-006	-3.63493	E11.5 * Wnt1 down vs E11.5 * Ctrl
10493831	S100a8	NM_013650	6.77553e-006	-3.6438	E11.5 * Wnt1 down vs E11.5 * Ctrl
10349648	Ctse	NM_007799	1.85369e-010	-3.64703	E11.5 * Wnt1 down vs E11.5 * Ctrl
10499189	Fcrls	NM_030707	7.66532e-008	-3.65557	E11.5 * Wnt1 down vs E11.5 * Ctrl
10380174	Mpo	NM_010824	6.64104e-011	-3.66391	E11.5 * Wnt1 down vs E11.5 * Ctrl
10519717	Sema3a	NM_009152	7.12525e-009	-3.67443	E11.5 * Wnt1 down vs E11.5 * Ctrl
10473399	Prg2	NM_008920	7.991e-008	-3.68248	E11.5 * Wnt1 down vs E11.5 * Ctrl
10469404	Cacnb2	NM_023116	1.94365e-009	-3.68282	E11.5 * Wnt1 down vs E11.5 * Ctrl
10366153	Rassf9	NM_146240	2.48045e-008	-3.69508	E11.5 * Wnt1 down vs E11.5 * Ctrl
10408935	ENSMUSG0000C	ENSMUST00000(	0.000849093	-3.69737	E11.5 * Wnt1 down vs E11.5 * Ctrl
10517513	C1qc	NM_007574	7.76681e-009	-3.72837	E11.5 * Wnt1 down vs E11.5 * Ctrl
10594855	Cgnl1	NM_026599	1.44429e-008	-3.73112	E11.5 * Wnt1 down vs E11.5 * Ctrl
10362674	Rnu3a	NR_002842	0.000122682	-3.73447	E11.5 * Wnt1 down vs E11.5 * Ctrl
10427290	Hoxc8	NM_010466	3.43007e-008	-3.74118	E11.5 * Wnt1 down vs E11.5 * Ctrl
10447190	Plekhh2	NM_177606	4.26812e-011	-3.75682	E11.5 * Wnt1 down vs E11.5 * Ctrl
10569341	H19	NR_001592	1.44252e-005	-3.78252	E11.5 * Wnt1 down vs E11.5 * Ctrl
10409876	Ctla2a	NM_007796	1.14533e-006	-3.78823	E11.5 * Wnt1 down vs E11.5 * Ctrl
10505954	Tek	NM_013690	1.68818e-009	-3.79266	E11.5 * Wnt1 down vs E11.5 * Ctrl
10498921	Tdo2	NM_019911	5.8429e-008	-3.82762	E11.5 * Wnt1 down vs E11.5 * Ctrl
10391649	Slc4a1	NM_011403	1.14866e-011	-3.84048	E11.5 * Wnt1 down vs E11.5 * Ctrl
10581013	Cdh11	NM_009866	8.33906e-010	-3.84745	E11.5 * Wnt1 down vs E11.5 * Ctrl
10530029	Lgi2	NM_144945	9.15854e-010	-3.85418	E11.5 * Wnt1 down vs E11.5 * Ctrl
10405179	S1pr3	NM_010101	6.08864e-011	-3.88048	E11.5 * Wnt1 down vs E11.5 * Ctrl

10537298	Chrm2	NM_203491	3.88954e-006	-3.88393	E11.5 * Wnt1 down vs E11.5 * Ctrl
10544583	Gimap6	NM_153175	1.03082e-009	-3.89416	E11.5 * Wnt1 down vs E11.5 * Ctrl
10511363	Penk	NM_001002927	2.10355e-008	-3.91111	E11.5 * Wnt1 down vs E11.5 * Ctrl
10578136		---	3.74156e-006	-3.92328	E11.5 * Wnt1 down vs E11.5 * Ctrl
10531931	Sparcl1	NM_010097	7.08302e-010	-3.93638	E11.5 * Wnt1 down vs E11.5 * Ctrl
10423971	Pkhd1l1	NM_138674	1.1348e-015	-3.94478	E11.5 * Wnt1 down vs E11.5 * Ctrl
10368495	Rspo3	NM_028351	3.05317e-011	-3.97415	E11.5 * Wnt1 down vs E11.5 * Ctrl
10542592	ENSMUSG0000C	AK143453	1.47834e-009	-4.0179	E11.5 * Wnt1 down vs E11.5 * Ctrl
10392221	Pecam1	NM_008816	3.19602e-010	-4.03471	E11.5 * Wnt1 down vs E11.5 * Ctrl
10496324	Slc39a8	NM_001135149	3.30061e-010	-4.03605	E11.5 * Wnt1 down vs E11.5 * Ctrl
10422436	Dock9	NM_001081039	1.99414e-011	-4.05304	E11.5 * Wnt1 down vs E11.5 * Ctrl
10534389	Cldn13	NM_020504	3.46507e-007	-4.05635	E11.5 * Wnt1 down vs E11.5 * Ctrl
10542575	Pde3a	NM_018779	1.55081e-007	-4.06968	E11.5 * Wnt1 down vs E11.5 * Ctrl
10484197	2610301F02Rik	ENSMUST00000	3.12945e-009	-4.07679	E11.5 * Wnt1 down vs E11.5 * Ctrl
10591123	Fat3	NM_001080814	1.51144e-006	-4.07836	E11.5 * Wnt1 down vs E11.5 * Ctrl
10394534	Osr1	NM_011859	6.35663e-006	-4.0876	E11.5 * Wnt1 down vs E11.5 * Ctrl
10472965	Hoxd8	NM_008276	4.88667e-009	-4.08811	E11.5 * Wnt1 down vs E11.5 * Ctrl
10483074	Gcg	NM_008100	1.08428e-008	-4.09123	E11.5 * Wnt1 down vs E11.5 * Ctrl
10558769	Ifitm1	NM_026820	2.74292e-008	-4.09157	E11.5 * Wnt1 down vs E11.5 * Ctrl
10396419		---	2.6106e-005	-4.09356	E11.5 * Wnt1 down vs E11.5 * Ctrl
10554789	Ctsc	NM_009982	7.7733e-009	-4.11393	E11.5 * Wnt1 down vs E11.5 * Ctrl
10346164	Sdpr	NM_138741	2.11824e-008	-4.14101	E11.5 * Wnt1 down vs E11.5 * Ctrl
10445268	Gpr116	NM_001081178	5.15928e-010	-4.18092	E11.5 * Wnt1 down vs E11.5 * Ctrl
10366446	Tspan8	NM_146010	3.66047e-005	-4.18684	E11.5 * Wnt1 down vs E11.5 * Ctrl
10514088	Frem1	NM_177863	8.99722e-011	-4.19029	E11.5 * Wnt1 down vs E11.5 * Ctrl
10606369	Itm2a	NM_008409	1.39146e-006	-4.23981	E11.5 * Wnt1 down vs E11.5 * Ctrl
10365983	Lum	NM_008524	3.0368e-005	-4.24127	E11.5 * Wnt1 down vs E11.5 * Ctrl
10467826	LOC545291	NM_001081257	8.21169e-009	-4.25357	E11.5 * Wnt1 down vs E11.5 * Ctrl
10423520	Sema5a	NM_009154	1.34922e-009	-4.276	E11.5 * Wnt1 down vs E11.5 * Ctrl
10435948	Ccdc80	NM_026439	6.67469e-009	-4.31469	E11.5 * Wnt1 down vs E11.5 * Ctrl
10498935	Gucy1b3	NM_017469	1.92711e-007	-4.36592	E11.5 * Wnt1 down vs E11.5 * Ctrl
10574415	1700047G07Rik	ENSMUST00000	8.13026e-006	-4.36632	E11.5 * Wnt1 down vs E11.5 * Ctrl
10546432	Adamts9	NM_175314	2.27103e-005	-4.37819	E11.5 * Wnt1 down vs E11.5 * Ctrl
10498952	Gucy1a3	NM_021896	3.53592e-010	-4.45699	E11.5 * Wnt1 down vs E11.5 * Ctrl
10569344	Igf2	NM_001122737	3.19061e-007	-4.47961	E11.5 * Wnt1 down vs E11.5 * Ctrl
10398380		---	1.87683e-006	-4.48061	E11.5 * Wnt1 down vs E11.5 * Ctrl
10486041	Meis2	NM_001136072	1.32707e-007	-4.56972	E11.5 * Wnt1 down vs E11.5 * Ctrl
10603551	Cybb	NM_007807	9.36509e-008	-4.63069	E11.5 * Wnt1 down vs E11.5 * Ctrl
10568436	Fgfr2	NM_010207	3.40678e-008	-4.63356	E11.5 * Wnt1 down vs E11.5 * Ctrl
10490775	ENSMUSG0000C	ENSMUST00000	5.32962e-007	-4.644	E11.5 * Wnt1 down vs E11.5 * Ctrl
10576049	Foxf1a	ENSMUST00000	5.48839e-007	-4.66023	E11.5 * Wnt1 down vs E11.5 * Ctrl
10501063	Cd53	NM_007651	1.00107e-008	-4.67284	E11.5 * Wnt1 down vs E11.5 * Ctrl
10369615	Srgn	NM_011157	6.65956e-009	-4.71637	E11.5 * Wnt1 down vs E11.5 * Ctrl
10499861	S100a9	NM_009114	6.02032e-008	-4.74833	E11.5 * Wnt1 down vs E11.5 * Ctrl
10406254	Ell2	NM_138953	4.92693e-010	-4.7821	E11.5 * Wnt1 down vs E11.5 * Ctrl
10427402	Ghr	NM_010284	3.68505e-010	-4.80293	E11.5 * Wnt1 down vs E11.5 * Ctrl
10352905	Cd34	NM_001111059	8.51875e-009	-4.80528	E11.5 * Wnt1 down vs E11.5 * Ctrl
10554129	B130024G19Rik	BC070425	1.76818e-009	-4.83304	E11.5 * Wnt1 down vs E11.5 * Ctrl
10424105	Colec10	NM_173422	3.99356e-012	-4.94443	E11.5 * Wnt1 down vs E11.5 * Ctrl
10433776	Snai2	NM_011415	2.12413e-009	-4.98241	E11.5 * Wnt1 down vs E11.5 * Ctrl
10488382	Cd93	NM_010740	1.35494e-009	-5.01195	E11.5 * Wnt1 down vs E11.5 * Ctrl
10428376	Angpt1	NM_009640	1.79155e-009	-5.06396	E11.5 * Wnt1 down vs E11.5 * Ctrl
10356880	St8sia4	NM_009183	4.61691e-012	-5.17321	E11.5 * Wnt1 down vs E11.5 * Ctrl
10578950	ENSMUSG0000C	ENSMUST00000	4.31954e-009	-5.18243	E11.5 * Wnt1 down vs E11.5 * Ctrl
10422728	Dab2	NM_023118	4.6894e-009	-5.3352	E11.5 * Wnt1 down vs E11.5 * Ctrl
10369023	Fam162b	BC147085	4.01758e-008	-5.3541	E11.5 * Wnt1 down vs E11.5 * Ctrl
10500559	Hsd3b6	NM_013821	7.44482e-009	-5.38102	E11.5 * Wnt1 down vs E11.5 * Ctrl
10536635	A430107O13Rik	NM_001081351	8.96996e-010	-5.40969	E11.5 * Wnt1 down vs E11.5 * Ctrl
10537296		---	3.53411e-007	-5.42702	E11.5 * Wnt1 down vs E11.5 * Ctrl
10490923	Car2	NM_009801	1.44569e-009	-5.44561	E11.5 * Wnt1 down vs E11.5 * Ctrl
10423917		---	0.000162142	-5.53708	E11.5 * Wnt1 down vs E11.5 * Ctrl
10385770	Olfr1372-ps1	BC055827	1.06394e-007	-5.56066	E11.5 * Wnt1 down vs E11.5 * Ctrl
10439299	Stfa3	NM_025288	9.20211e-008	-5.58181	E11.5 * Wnt1 down vs E11.5 * Ctrl
10563611	Saa1	NM_009117	8.43752e-007	-5.60776	E11.5 * Wnt1 down vs E11.5 * Ctrl
10438445	Klhl6	NM_183390	1.67459e-011	-5.64057	E11.5 * Wnt1 down vs E11.5 * Ctrl
10365974	Dcn	NM_007833	2.49393e-006	-5.71167	E11.5 * Wnt1 down vs E11.5 * Ctrl

10592533		---	6.40079e-010	-5.8347	E11.5 * Wnt1 down vs E11.5 * Ctrl
10601519	Klhl4	NM_172781	1.41568e-008	-5.87175	E11.5 * Wnt1 down vs E11.5 * Ctrl
10569017	Ifitm3	NM_025378	5.41359e-007	-5.9167	E11.5 * Wnt1 down vs E11.5 * Ctrl
10523451	Anxa3	NM_013470	1.42104e-008	-5.91803	E11.5 * Wnt1 down vs E11.5 * Ctrl
10473444	Aplnr	NM_011784	6.55862e-011	-5.95756	E11.5 * Wnt1 down vs E11.5 * Ctrl
10522503	Pdgfra	NM_011058	1.40176e-007	-6.02308	E11.5 * Wnt1 down vs E11.5 * Ctrl
10586865	Aldh1a2	NM_009022	4.44652e-008	-6.02903	E11.5 * Wnt1 down vs E11.5 * Ctrl
10450484	Aif1	NM_019467	1.21749e-007	-6.04333	E11.5 * Wnt1 down vs E11.5 * Ctrl
10404606	Ly86	NM_010745	6.56118e-009	-6.06171	E11.5 * Wnt1 down vs E11.5 * Ctrl
10559790	Zim1	NM_011769	7.83362e-010	-6.1427	E11.5 * Wnt1 down vs E11.5 * Ctrl
10484371	Calcrl	NM_018782	1.42018e-010	-6.20101	E11.5 * Wnt1 down vs E11.5 * Ctrl
10512757	Hemgn	NM_053149	2.08736e-011	-6.35108	E11.5 * Wnt1 down vs E11.5 * Ctrl
10351905	Spna1	NM_011465	1.07168e-012	-6.38078	E11.5 * Wnt1 down vs E11.5 * Ctrl
10523134	Pf4	NM_019932	1.71199e-008	-6.41335	E11.5 * Wnt1 down vs E11.5 * Ctrl
10514185		---	1.05381e-005	-6.43171	E11.5 * Wnt1 down vs E11.5 * Ctrl
10446965	Rasgrp3	NM_207246	2.10608e-010	-6.45534	E11.5 * Wnt1 down vs E11.5 * Ctrl
10466800	Pgm5	NM_175013	1.25165e-007	-6.45588	E11.5 * Wnt1 down vs E11.5 * Ctrl
10451677		---	2.23279e-011	-6.47948	E11.5 * Wnt1 down vs E11.5 * Ctrl
10523756		---	2.23279e-011	-6.47948	E11.5 * Wnt1 down vs E11.5 * Ctrl
10537504		---	2.23279e-011	-6.47948	E11.5 * Wnt1 down vs E11.5 * Ctrl
10574438	Cdh5	NM_009868	2.14826e-008	-6.50756	E11.5 * Wnt1 down vs E11.5 * Ctrl
10531724	Plac8	NM_139198	2.43952e-006	-6.60837	E11.5 * Wnt1 down vs E11.5 * Ctrl
10359504	Dnm3os	NR_002870	3.32665e-007	-6.63718	E11.5 * Wnt1 down vs E11.5 * Ctrl
10461614	Ms4a6c	NM_028595	1.65397e-010	-6.72105	E11.5 * Wnt1 down vs E11.5 * Ctrl
10542981	Gmfg	NM_022024	3.71837e-009	-6.76922	E11.5 * Wnt1 down vs E11.5 * Ctrl
10544383	Kel	NM_032540	1.28846e-014	-6.99346	E11.5 * Wnt1 down vs E11.5 * Ctrl
10368240	Tcf21	NM_011545	1.4355e-008	-7.05481	E11.5 * Wnt1 down vs E11.5 * Ctrl
10484283	Pde1a	NM_016744	9.75054e-011	-7.11387	E11.5 * Wnt1 down vs E11.5 * Ctrl
10523359	Cxcl13	NM_018866	7.82275e-010	-7.16871	E11.5 * Wnt1 down vs E11.5 * Ctrl
10601942	Nrk	NM_013724	5.03741e-008	-7.19942	E11.5 * Wnt1 down vs E11.5 * Ctrl
10571840	Hpgd	NM_008278	2.35441e-009	-7.30751	E11.5 * Wnt1 down vs E11.5 * Ctrl
10500780	Nr1h5	NM_198658	1.24489e-010	-7.43618	E11.5 * Wnt1 down vs E11.5 * Ctrl
10397645	Gpr65	NM_008152	6.27868e-009	-7.45339	E11.5 * Wnt1 down vs E11.5 * Ctrl
10548892	Arhgdib	NM_007486	8.69219e-008	-7.75856	E11.5 * Wnt1 down vs E11.5 * Ctrl
10357488	Cd55	NM_010016	3.15794e-010	-8.0657	E11.5 * Wnt1 down vs E11.5 * Ctrl
10570434	Ifitm1	NM_026820	9.80197e-010	-8.29073	E11.5 * Wnt1 down vs E11.5 * Ctrl
10416437	Lcp1	NM_008879	2.6651e-011	-8.31511	E11.5 * Wnt1 down vs E11.5 * Ctrl
10461622	Ms4a6b	NM_027209	1.06976e-008	-8.43349	E11.5 * Wnt1 down vs E11.5 * Ctrl
10422760	Fyb	NM_011815	4.76039e-011	-8.58855	E11.5 * Wnt1 down vs E11.5 * Ctrl
10445192	Rhag	NM_011269	1.12535e-011	-8.66038	E11.5 * Wnt1 down vs E11.5 * Ctrl
10587231	Bmp5	NM_007555	5.46439e-009	-8.82071	E11.5 * Wnt1 down vs E11.5 * Ctrl
10604175	Fam70a	NM_172930	3.6809e-010	-8.82926	E11.5 * Wnt1 down vs E11.5 * Ctrl
10480090	Itga8	NM_001001309	1.33793e-010	-9.04913	E11.5 * Wnt1 down vs E11.5 * Ctrl
10496359	Emcn	NM_016885	3.11701e-012	-9.54448	E11.5 * Wnt1 down vs E11.5 * Ctrl
10494271	Ctss	NM_021281	4.71185e-010	-10.0091	E11.5 * Wnt1 down vs E11.5 * Ctrl
10573054	Gypa	NM_010369	2.02039e-010	-10.0152	E11.5 * Wnt1 down vs E11.5 * Ctrl
10551883	Tyrobp	NM_011662	3.54359e-011	-10.0535	E11.5 * Wnt1 down vs E11.5 * Ctrl
10547657	C3ar1	NM_009779	9.38993e-011	-10.1163	E11.5 * Wnt1 down vs E11.5 * Ctrl
10472958	Hoxd10	NM_013554	5.55426e-008	-10.5749	E11.5 * Wnt1 down vs E11.5 * Ctrl
10600024	Gpr50	NM_010340	1.50918e-009	-10.6583	E11.5 * Wnt1 down vs E11.5 * Ctrl
10469358	Mrc1	NM_008625	9.07102e-012	-11.0292	E11.5 * Wnt1 down vs E11.5 * Ctrl
10347931	C130026I21Rik	BC007193	1.24234e-009	-12.1604	E11.5 * Wnt1 down vs E11.5 * Ctrl
10500796		---	1.34066e-010	-12.3214	E11.5 * Wnt1 down vs E11.5 * Ctrl
10439292	BC100530	NM_001082546	2.34568e-009	-12.6251	E11.5 * Wnt1 down vs E11.5 * Ctrl
10574023	Mt2	NM_008630	8.54264e-011	-15.6768	E11.5 * Wnt1 down vs E11.5 * Ctrl
10566272	Hbb-y	NM_008221	3.99355e-007	-31.0044	E11.5 * Wnt1 down vs E11.5 * Ctrl
10375046	Hba-x	NM_010405	1.40534e-009	-47.8866	E11.5 * Wnt1 down vs E11.5 * Ctrl
10375058	Hba-a2	NM_001083955	1.2189e-009	-50.6126	E11.5 * Wnt1 down vs E11.5 * Ctrl
10566258	Hbb-b1	ENSMUST00000(	7.33325e-010	-53.1168	E11.5 * Wnt1 down vs E11.5 * Ctrl
10375051	Hba-a1	NM_008218	1.60102e-009	-53.4619	E11.5 * Wnt1 down vs E11.5 * Ctrl
10566254	Hbb-b1	NM_008220	8.2729e-010	-59.3173	E11.5 * Wnt1 down vs E11.5 * Ctrl
10566268	Hbb-bh1	NM_008219	1.5701e-011	-121.789	E11.5 * Wnt1 down vs E11.5 * Ctrl

S11 vs C11

Probeset ID	Gene Symbol	RefSeq	p-value	Fold-change	Comparison

10593756	Chrna3	NM_145129	1.42855e-017	31.0444	E11.5 * Sox10 up vs E11.5 * Ctrl
10357103	Cdh19	NM_001081386	8.50558e-013	24.2301	E11.5 * Sox10 up vs E11.5 * Ctrl
10547227	Ret	NM_001080780	4.15503e-016	22.982	E11.5 * Sox10 up vs E11.5 * Ctrl
10515095	Elavl4	NM_010488	1.19519e-015	18.3919	E11.5 * Sox10 up vs E11.5 * Ctrl
10576332	Tubb3	NM_023279	3.40598e-012	17.8591	E11.5 * Sox10 up vs E11.5 * Ctrl
10595033	Scg3	NM_009130	3.35504e-016	16.5488	E11.5 * Sox10 up vs E11.5 * Ctrl
10536667	Ptprz1	NM_001081306	1.46002e-015	16.1907	E11.5 * Sox10 up vs E11.5 * Ctrl
10492169	Mab21l1	NM_010750	4.59575e-015	14.5006	E11.5 * Sox10 up vs E11.5 * Ctrl
10427862	Cdh6	NM_007666	3.03876e-016	13.286	E11.5 * Sox10 up vs E11.5 * Ctrl
10431812	Nell2	NM_016743	4.26103e-015	12.729	E11.5 * Sox10 up vs E11.5 * Ctrl
10470412	Dbh	NM_138942	3.87969e-014	11.9563	E11.5 * Sox10 up vs E11.5 * Ctrl
10373467	Erbb3	NM_010153	6.05005e-013	11.7862	E11.5 * Sox10 up vs E11.5 * Ctrl
10464471	Gal	NM_010253	7.98352e-011	10.8923	E11.5 * Sox10 up vs E11.5 * Ctrl
10531869	Mapk10	NM_009158	3.81004e-014	10.8553	E11.5 * Sox10 up vs E11.5 * Ctrl
10431659	Kif21a	NM_001109040	1.69686e-015	9.65558	E11.5 * Sox10 up vs E11.5 * Ctrl
10417628	Cadps	NM_012061	8.99827e-013	9.43107	E11.5 * Sox10 up vs E11.5 * Ctrl
10490818	Stmn2	NM_025285	1.56112e-013	9.20625	E11.5 * Sox10 up vs E11.5 * Ctrl
10543802	Plxna4	NM_175750	1.68247e-014	9.01822	E11.5 * Sox10 up vs E11.5 * Ctrl
10522895	Csn3	NM_007786	1.18321e-011	8.9885	E11.5 * Sox10 up vs E11.5 * Ctrl
10605113	L1cam	NM_008478	3.6917e-014	8.98042	E11.5 * Sox10 up vs E11.5 * Ctrl
10463737	Ina	AK144917	3.88912e-011	8.95211	E11.5 * Sox10 up vs E11.5 * Ctrl
10523231	Art3	NM_181728	2.30623e-014	8.77677	E11.5 * Sox10 up vs E11.5 * Ctrl
10432675	I730030J21Rik	ENSMUST00000(	7.61061e-009	8.59491	E11.5 * Sox10 up vs E11.5 * Ctrl
10402020	Eml5	NM_001081191	2.51928e-015	8.51556	E11.5 * Sox10 up vs E11.5 * Ctrl
10372324	Syt1	NM_009306	5.72974e-012	8.51273	E11.5 * Sox10 up vs E11.5 * Ctrl
10499431	Syt11	NM_018804	7.62949e-014	7.9546	E11.5 * Sox10 up vs E11.5 * Ctrl
10345016	Tcfap2b	NM_001025305	2.13579e-012	7.71177	E11.5 * Sox10 up vs E11.5 * Ctrl
10593767	Chrnb4	NM_148944	1.43229e-011	7.70393	E11.5 * Sox10 up vs E11.5 * Ctrl
10585484	Chrna5	NM_176844	4.07404e-012	7.30208	E11.5 * Sox10 up vs E11.5 * Ctrl
10367982	Gpr126	NM_001002268	2.34462e-013	7.28619	E11.5 * Sox10 up vs E11.5 * Ctrl
10423243	Cdh10	NM_009865	2.85677e-011	7.23871	E11.5 * Sox10 up vs E11.5 * Ctrl
10428698	Sntb1	NM_016667	1.00286e-013	7.23379	E11.5 * Sox10 up vs E11.5 * Ctrl
10575693	Vat1l	NM_173016	1.18346e-012	7.09111	E11.5 * Sox10 up vs E11.5 * Ctrl
10464370	Slc18a2	NM_172523	3.41059e-013	7.05644	E11.5 * Sox10 up vs E11.5 * Ctrl
10463732	Ina	NM_146100	5.9778e-015	6.95836	E11.5 * Sox10 up vs E11.5 * Ctrl
10597817	Cck	NM_031161	1.81906e-009	6.67985	E11.5 * Sox10 up vs E11.5 * Ctrl
10494043	Tdrkh	NM_028307	2.13837e-013	6.6519	E11.5 * Sox10 up vs E11.5 * Ctrl
10601888	Plp1	NM_011123	4.40775e-011	6.61103	E11.5 * Sox10 up vs E11.5 * Ctrl
10554900	Dlg2	NM_011807	1.21455e-013	6.42699	E11.5 * Sox10 up vs E11.5 * Ctrl
10408798	Tcfap2a	NM_011547	1.80483e-012	6.36505	E11.5 * Sox10 up vs E11.5 * Ctrl
10368999	Grik2	NM_010349	1.15238e-012	6.33903	E11.5 * Sox10 up vs E11.5 * Ctrl
10422164	Ednrb	NM_007904	3.3722e-015	6.29359	E11.5 * Sox10 up vs E11.5 * Ctrl
10439514	Gap43	NM_008083	2.77244e-014	6.24286	E11.5 * Sox10 up vs E11.5 * Ctrl
10498885	Gria2	NM_013540	3.15653e-011	6.22541	E11.5 * Sox10 up vs E11.5 * Ctrl
10363224	Fabp7	NM_021272	2.28508e-012	6.14351	E11.5 * Sox10 up vs E11.5 * Ctrl
10522388	Slc10a4	NM_173403	1.12036e-011	5.97466	E11.5 * Sox10 up vs E11.5 * Ctrl
10491477	Sox2	NM_011443	1.84543e-009	5.93813	E11.5 * Sox10 up vs E11.5 * Ctrl
10353192	Eya1	NM_010164	1.43604e-011	5.80427	E11.5 * Sox10 up vs E11.5 * Ctrl
10492640	Fstl5	NM_178673	1.72417e-011	5.68055	E11.5 * Sox10 up vs E11.5 * Ctrl
10458663	Dpysl3	NM_009468	1.32373e-013	5.64483	E11.5 * Sox10 up vs E11.5 * Ctrl
10368175	Pde7b	NM_013875	1.72844e-011	5.63926	E11.5 * Sox10 up vs E11.5 * Ctrl
10540298	Chl1	NM_007697	8.69657e-013	5.58508	E11.5 * Sox10 up vs E11.5 * Ctrl
10400926	Rtn1	NM_153457	1.33097e-011	5.52782	E11.5 * Sox10 up vs E11.5 * Ctrl
10508052	Grik3	NM_001081097	1.52131e-010	5.5106	E11.5 * Sox10 up vs E11.5 * Ctrl
10530306	Phox2b	NM_008888	1.15544e-011	5.43972	E11.5 * Sox10 up vs E11.5 * Ctrl
10522208	Uchl1	NM_011670	3.96755e-010	5.38989	E11.5 * Sox10 up vs E11.5 * Ctrl
10586591	Car12	NM_178396	6.84604e-012	5.38943	E11.5 * Sox10 up vs E11.5 * Ctrl
10362372	9330159F19Rik	BC138282	1.23927e-010	5.31468	E11.5 * Sox10 up vs E11.5 * Ctrl
10423836	Cthrc1	NM_026778	5.28545e-014	5.26788	E11.5 * Sox10 up vs E11.5 * Ctrl
10443463	Cdkn1a	NM_007669	9.83068e-011	5.25802	E11.5 * Sox10 up vs E11.5 * Ctrl
10485955	Scg5	NM_009162	3.88907e-013	5.23895	E11.5 * Sox10 up vs E11.5 * Ctrl
10584549	Scn3b	NM_178227	1.23832e-011	5.23049	E11.5 * Sox10 up vs E11.5 * Ctrl
10603151	Gpm6b	NM_023122	2.28498e-014	5.1687	E11.5 * Sox10 up vs E11.5 * Ctrl
10408629	1300014I06Rik	NM_025831	1.51899e-011	5.10588	E11.5 * Sox10 up vs E11.5 * Ctrl
10605437	Pls3	NM_145629	3.25618e-013	5.10199	E11.5 * Sox10 up vs E11.5 * Ctrl
10395553	Nrcam	NM_176930	3.11368e-012	5.02772	E11.5 * Sox10 up vs E11.5 * Ctrl

10514510	Cyp2j6	NM_010008	7.1451e-012	5.0022	E11.5 * Sox10 up vs E11.5 * Ctrl
10411527	Cartpt	NM_013732	1.16595e-007	4.91073	E11.5 * Sox10 up vs E11.5 * Ctrl
10514520	Cyp2j9	NM_028979	4.59091e-011	4.89445	E11.5 * Sox10 up vs E11.5 * Ctrl
10472809	Dlx1	NM_010053	1.28456e-012	4.85003	E11.5 * Sox10 up vs E11.5 * Ctrl
10476512	Snap25	NM_011428	3.72287e-012	4.77474	E11.5 * Sox10 up vs E11.5 * Ctrl
10416340	Gfra2	NM_008115	4.15648e-012	4.75431	E11.5 * Sox10 up vs E11.5 * Ctrl
10465820	Gng3	NM_010316	3.32024e-010	4.65961	E11.5 * Sox10 up vs E11.5 * Ctrl
10520950	Pdlim1	NM_016861	3.52146e-011	4.63135	E11.5 * Sox10 up vs E11.5 * Ctrl
10569646	Ccnd1	NM_007631	3.92476e-013	4.57095	E11.5 * Sox10 up vs E11.5 * Ctrl
10402708	Ckb	NM_021273	8.23454e-011	4.47947	E11.5 * Sox10 up vs E11.5 * Ctrl
10529903	Fam184b	NM_021416	3.73078e-010	4.45933	E11.5 * Sox10 up vs E11.5 * Ctrl
10483803	6720416L17Rik	ENSMUST00000	4.88264e-011	4.42593	E11.5 * Sox10 up vs E11.5 * Ctrl
10492136	Dclk1	NM_019978	4.14056e-013	4.39523	E11.5 * Sox10 up vs E11.5 * Ctrl
10543058	Dlx5	NM_010056	3.53384e-008	4.38633	E11.5 * Sox10 up vs E11.5 * Ctrl
10491136	Tnik	BC137799	3.3858e-015	4.38043	E11.5 * Sox10 up vs E11.5 * Ctrl
10458052	Epb4.1l4a	NM_013512	1.14318e-012	4.33362	E11.5 * Sox10 up vs E11.5 * Ctrl
10562223	Fxyd3	NM_008557	2.47495e-012	4.32217	E11.5 * Sox10 up vs E11.5 * Ctrl
10505998	Fggy	NM_001113412	1.33311e-007	4.30463	E11.5 * Sox10 up vs E11.5 * Ctrl
10346882	Adam23	NM_011780	6.12772e-011	4.24644	E11.5 * Sox10 up vs E11.5 * Ctrl
10467420	Pdlim1	NM_016861	1.29345e-010	4.24297	E11.5 * Sox10 up vs E11.5 * Ctrl
10584259	Fez1	NM_183171	8.07002e-014	4.19258	E11.5 * Sox10 up vs E11.5 * Ctrl
10351400	Fam78b	NM_175461	2.28233e-011	4.18394	E11.5 * Sox10 up vs E11.5 * Ctrl
10468452	Sorcs1	NM_021377	6.80181e-013	4.14835	E11.5 * Sox10 up vs E11.5 * Ctrl
10603896	Klhl13	NM_026167	1.83427e-013	4.14466	E11.5 * Sox10 up vs E11.5 * Ctrl
10363541	Ass1	NM_007494	1.44276e-009	4.13774	E11.5 * Sox10 up vs E11.5 * Ctrl
10354529	1700019D03Rik	NM_144953	1.92669e-009	4.13709	E11.5 * Sox10 up vs E11.5 * Ctrl
10605874	Eda2r	NM_175540	2.16459e-007	4.11695	E11.5 * Sox10 up vs E11.5 * Ctrl
10474096	Lrrc4c	NM_178725	4.24112e-012	4.09756	E11.5 * Sox10 up vs E11.5 * Ctrl
10485718	Ano3	NM_001128103	3.48932e-008	4.09222	E11.5 * Sox10 up vs E11.5 * Ctrl
10485979	Gjd2	NM_010290	4.00074e-007	4.0421	E11.5 * Sox10 up vs E11.5 * Ctrl
10406461	C130071C03Rik	ENSMUST00000(	2.56303e-009	4.02192	E11.5 * Sox10 up vs E11.5 * Ctrl
10505994	Fggy	NM_001113412	3.43819e-006	3.99917	E11.5 * Sox10 up vs E11.5 * Ctrl
10471994	Kif5c	NM_008449	5.82096e-011	3.99646	E11.5 * Sox10 up vs E11.5 * Ctrl
10471154	Ass1	NM_007494	3.87452e-009	3.96457	E11.5 * Sox10 up vs E11.5 * Ctrl
10366163	Slc6a15	NM_175328	3.17231e-009	3.95564	E11.5 * Sox10 up vs E11.5 * Ctrl
10533055	1500001A10Rik	NM_026886	4.91942e-013	3.95365	E11.5 * Sox10 up vs E11.5 * Ctrl
10346150	Tmeff2	NM_019790	4.90869e-013	3.93506	E11.5 * Sox10 up vs E11.5 * Ctrl
10359113	Fam163a	NM_177838	9.78232e-009	3.80496	E11.5 * Sox10 up vs E11.5 * Ctrl
10452766		---	0.000413383	3.77585	E11.5 * Sox10 up vs E11.5 * Ctrl
10413419	Arhgef3	NM_027871	4.25734e-011	3.76358	E11.5 * Sox10 up vs E11.5 * Ctrl
10599880	Slitrk2	NM_198863	1.73025e-010	3.71594	E11.5 * Sox10 up vs E11.5 * Ctrl
10548030	Cd9	NM_007657	1.25737e-009	3.64557	E11.5 * Sox10 up vs E11.5 * Ctrl
10485357		---	0.00328903	3.59003	E11.5 * Sox10 up vs E11.5 * Ctrl
10473125	Itga4	NM_010576	5.2366e-009	3.57835	E11.5 * Sox10 up vs E11.5 * Ctrl
10571865	Scrg1	NM_009136	1.4164e-008	3.54266	E11.5 * Sox10 up vs E11.5 * Ctrl
10425240	Micall1	NM_177461	6.90408e-011	3.52916	E11.5 * Sox10 up vs E11.5 * Ctrl
10385271	Ccng1	NM_009831	5.95932e-012	3.51425	E11.5 * Sox10 up vs E11.5 * Ctrl
10362363	6330407J23Rik	NM_026138	8.19392e-011	3.51164	E11.5 * Sox10 up vs E11.5 * Ctrl
10350438	Kcnt2	NM_001081027	6.82205e-008	3.49584	E11.5 * Sox10 up vs E11.5 * Ctrl
10362003	F730021E23Rik	AK155606	3.01716e-006	3.48545	E11.5 * Sox10 up vs E11.5 * Ctrl
10373407	Mbc2	NM_011843	2.41706e-009	3.46719	E11.5 * Sox10 up vs E11.5 * Ctrl
10502510	Lmo4	NM_010723	1.02982e-010	3.45077	E11.5 * Sox10 up vs E11.5 * Ctrl
10447056	Qpct	NM_027455	3.36614e-010	3.42306	E11.5 * Sox10 up vs E11.5 * Ctrl
10535956	Stard13	NM_146258	1.46457e-008	3.4058	E11.5 * Sox10 up vs E11.5 * Ctrl
10371578	Ascl1	NM_008553	1.60232e-006	3.37418	E11.5 * Sox10 up vs E11.5 * Ctrl
10604751	Fgf13	NM_010200	5.23065e-008	3.36616	E11.5 * Sox10 up vs E11.5 * Ctrl
10503234	Rlbp1l1	NM_028940	2.43844e-012	3.36471	E11.5 * Sox10 up vs E11.5 * Ctrl
10350733	Rgs16	NM_011267	2.31812e-007	3.35342	E11.5 * Sox10 up vs E11.5 * Ctrl
10598626	Tspan7	NM_019634	3.13338e-011	3.34276	E11.5 * Sox10 up vs E11.5 * Ctrl
10439651	Cd200	NM_010818	1.35689e-009	3.33993	E11.5 * Sox10 up vs E11.5 * Ctrl
10479698	Myt1	NM_008665	2.7969e-009	3.33163	E11.5 * Sox10 up vs E11.5 * Ctrl
10492341	4631416L12Rik	NM_001081295	2.01268e-009	3.31969	E11.5 * Sox10 up vs E11.5 * Ctrl
10503214	Chd7	NM_001081417	1.29187e-009	3.30499	E11.5 * Sox10 up vs E11.5 * Ctrl
10363146	Slc35f1	NM_178675	1.32216e-008	3.29566	E11.5 * Sox10 up vs E11.5 * Ctrl
10549222	Bcat1	NM_001024468	1.32685e-009	3.27989	E11.5 * Sox10 up vs E11.5 * Ctrl
10503218	Chd7	NM_001081417	1.76939e-006	3.27963	E11.5 * Sox10 up vs E11.5 * Ctrl

10478374	Gdap1l1	NM_144891	1.62877e-010	3.27813	E11.5 * Sox10 up vs E11.5 * Ctrl
10362538	Lama4	NM_010681	5.01288e-014	3.26558	E11.5 * Sox10 up vs E11.5 * Ctrl
10344679	St18	NM_173868	7.24621e-010	3.2336	E11.5 * Sox10 up vs E11.5 * Ctrl
10388430	Serpinf1	NM_011340	9.24223e-013	3.21296	E11.5 * Sox10 up vs E11.5 * Ctrl
10584674	Mcam	NM_023061	2.03922e-011	3.19261	E11.5 * Sox10 up vs E11.5 * Ctrl
10487139	Shc4	NM_199022	5.95917e-012	3.18364	E11.5 * Sox10 up vs E11.5 * Ctrl
10411519	Mtap1b	NM_008634	4.50387e-011	3.17182	E11.5 * Sox10 up vs E11.5 * Ctrl
10564163	Snord116	NR_002895	1.81765e-005	3.16352	E11.5 * Sox10 up vs E11.5 * Ctrl
10564167	Snord116	NR_002895	1.81765e-005	3.16352	E11.5 * Sox10 up vs E11.5 * Ctrl
10564171	Snord116	NR_002895	1.81765e-005	3.16352	E11.5 * Sox10 up vs E11.5 * Ctrl
10564173	Snord116	NR_002895	1.81765e-005	3.16352	E11.5 * Sox10 up vs E11.5 * Ctrl
10564175	Snord116	NR_002895	1.81765e-005	3.16352	E11.5 * Sox10 up vs E11.5 * Ctrl
10564179	Snord116	NR_002895	1.81765e-005	3.16352	E11.5 * Sox10 up vs E11.5 * Ctrl
10564181	Snord116	NR_002895	1.81765e-005	3.16352	E11.5 * Sox10 up vs E11.5 * Ctrl
10564185	Snord116	NR_002895	1.81765e-005	3.16352	E11.5 * Sox10 up vs E11.5 * Ctrl
10564187	Snord116	NR_002895	1.81765e-005	3.16352	E11.5 * Sox10 up vs E11.5 * Ctrl
10564189	Snord116	NR_002895	1.81765e-005	3.16352	E11.5 * Sox10 up vs E11.5 * Ctrl
10564191	Snord116	NR_002895	1.81765e-005	3.16352	E11.5 * Sox10 up vs E11.5 * Ctrl
10564193	Snord116	NR_002895	1.81765e-005	3.16352	E11.5 * Sox10 up vs E11.5 * Ctrl
10564195	Snord116	NR_002895	1.81765e-005	3.16352	E11.5 * Sox10 up vs E11.5 * Ctrl
10564197	Snord116	NR_002895	1.81765e-005	3.16352	E11.5 * Sox10 up vs E11.5 * Ctrl
10564199	Snord116	NR_002895	1.81765e-005	3.16352	E11.5 * Sox10 up vs E11.5 * Ctrl
10564201	Snord116	AF241256	1.81765e-005	3.16352	E11.5 * Sox10 up vs E11.5 * Ctrl
10564205	Snord116	AF241256	1.81765e-005	3.16352	E11.5 * Sox10 up vs E11.5 * Ctrl
10564207	Snord116	AF241256	1.81765e-005	3.16352	E11.5 * Sox10 up vs E11.5 * Ctrl
10530499	EG545758	NM_001024147	1.1601e-009	3.1553	E11.5 * Sox10 up vs E11.5 * Ctrl
10416090	Stmn4	NM_019675	3.10027e-008	3.15497	E11.5 * Sox10 up vs E11.5 * Ctrl
10381154	Cnp	NM_009923	2.5254e-010	3.14199	E11.5 * Sox10 up vs E11.5 * Ctrl
10465943	Dagla	NM_198114	2.09954e-007	3.1255	E11.5 * Sox10 up vs E11.5 * Ctrl
10568107		---	2.29878e-005	3.11343	E11.5 * Sox10 up vs E11.5 * Ctrl
10471945	Zeb2	NM_015753	2.15453e-009	3.10927	E11.5 * Sox10 up vs E11.5 * Ctrl
10395702	Akap6	NM_198111	4.9991e-012	3.10829	E11.5 * Sox10 up vs E11.5 * Ctrl
10514352	Elavl2	NM_207685	1.39758e-009	3.10734	E11.5 * Sox10 up vs E11.5 * Ctrl
10468722	Gfra1	NM_010279	2.26765e-010	3.10687	E11.5 * Sox10 up vs E11.5 * Ctrl
10431017	Ttll1	NM_178869	6.93095e-012	3.1037	E11.5 * Sox10 up vs E11.5 * Ctrl
10506433	Dab1	NM_177259	2.38539e-010	3.0951	E11.5 * Sox10 up vs E11.5 * Ctrl
10564161	Snord116	NR_002895	1.77155e-005	3.07992	E11.5 * Sox10 up vs E11.5 * Ctrl
10503259	Trp53inp1	NM_021897	2.29862e-011	3.07422	E11.5 * Sox10 up vs E11.5 * Ctrl
10359908	Rgs4	NM_009062	3.43138e-008	3.05718	E11.5 * Sox10 up vs E11.5 * Ctrl
10573979	Gnao1	NM_010308	1.71072e-011	3.04702	E11.5 * Sox10 up vs E11.5 * Ctrl
10594447	Map2k1	NM_008927	2.31394e-011	3.03955	E11.5 * Sox10 up vs E11.5 * Ctrl
10564177	Snord116	AF241256	2.05805e-005	3.03756	E11.5 * Sox10 up vs E11.5 * Ctrl
10396125	Atl1	NM_178628	1.56939e-008	3.03006	E11.5 * Sox10 up vs E11.5 * Ctrl
10556509	Spon1	NM_145584	8.54197e-012	2.96533	E11.5 * Sox10 up vs E11.5 * Ctrl
10503206	Chd7	NM_001081417	4.27755e-009	2.9576	E11.5 * Sox10 up vs E11.5 * Ctrl
10503200	Chd7	NM_001081417	1.08528e-008	2.9354	E11.5 * Sox10 up vs E11.5 * Ctrl
10599884		---	0.000508496	2.93389	E11.5 * Sox10 up vs E11.5 * Ctrl
10440534	Adamts5	NM_011782	7.90377e-008	2.9333	E11.5 * Sox10 up vs E11.5 * Ctrl
10430458	Sox10	NM_011437	7.60788e-008	2.92426	E11.5 * Sox10 up vs E11.5 * Ctrl
10599001	Agtr2	NM_007429	0.000113313	2.91435	E11.5 * Sox10 up vs E11.5 * Ctrl
10402061	Eml5	BC049855	1.20032e-007	2.91185	E11.5 * Sox10 up vs E11.5 * Ctrl
10439321	Slc15a2	NM_021301	6.09197e-005	2.9079	E11.5 * Sox10 up vs E11.5 * Ctrl
10503202	Chd7	NM_001081417	8.13631e-010	2.90528	E11.5 * Sox10 up vs E11.5 * Ctrl
10404792	Phactr1	NM_198419	1.92735e-010	2.90181	E11.5 * Sox10 up vs E11.5 * Ctrl
10344973	Gdap1	NM_010267	4.2064e-007	2.89072	E11.5 * Sox10 up vs E11.5 * Ctrl
10384378	Ddc	NM_016672	4.27875e-011	2.88924	E11.5 * Sox10 up vs E11.5 * Ctrl
10462683	Pcgf5	NM_029508	3.54048e-008	2.88808	E11.5 * Sox10 up vs E11.5 * Ctrl
10360418	Rgs7	NM_011880	1.05137e-007	2.88193	E11.5 * Sox10 up vs E11.5 * Ctrl
10505996	Fggy	NM_001113412	7.86691e-008	2.85606	E11.5 * Sox10 up vs E11.5 * Ctrl
10492628	Serpini1	NM_009250	2.05964e-008	2.85524	E11.5 * Sox10 up vs E11.5 * Ctrl
10355984	Serpine2	NM_009255	1.93179e-013	2.85478	E11.5 * Sox10 up vs E11.5 * Ctrl
10357736	Nfasc	NM_182716	3.14355e-008	2.84222	E11.5 * Sox10 up vs E11.5 * Ctrl
10439695	Tagln3	NM_019754	9.91639e-010	2.82078	E11.5 * Sox10 up vs E11.5 * Ctrl
10434845	Il1rap	NM_008364	5.11139e-011	2.82039	E11.5 * Sox10 up vs E11.5 * Ctrl
10393379	Mxra7	NM_026280	4.84437e-010	2.81329	E11.5 * Sox10 up vs E11.5 * Ctrl
10440258	Epha3	NM_010140	8.02197e-013	2.81119	E11.5 * Sox10 up vs E11.5 * Ctrl

10521498	Crmp1	NM_007765	2.33484e-009	2.80994	E11.5 * Sox10 up vs E11.5 * Ctrl
10503194	Chd7	NM_001081417	3.71317e-007	2.80285	E11.5 * Sox10 up vs E11.5 * Ctrl
10423855	Rims2	NM_053271	3.08325e-009	2.79603	E11.5 * Sox10 up vs E11.5 * Ctrl
10354506	Mfsd6	NM_133829	1.70778e-007	2.78993	E11.5 * Sox10 up vs E11.5 * Ctrl
10422244	Slitrk6	NM_175499	7.07162e-009	2.77787	E11.5 * Sox10 up vs E11.5 * Ctrl
10497663	Slc7a14	NM_172861	4.09329e-009	2.76978	E11.5 * Sox10 up vs E11.5 * Ctrl
10482448	Zeb2	NM_015753	2.97491e-012	2.76911	E11.5 * Sox10 up vs E11.5 * Ctrl
10355836	Resp18	NM_009049	2.2232e-008	2.76549	E11.5 * Sox10 up vs E11.5 * Ctrl
10532753	Coro1c	NM_011779	1.74387e-009	2.76363	E11.5 * Sox10 up vs E11.5 * Ctrl
10501924	Ndst3	NM_031186	6.36321e-009	2.75923	E11.5 * Sox10 up vs E11.5 * Ctrl
10462613	Ifit2	NM_008332	2.9038e-011	2.75798	E11.5 * Sox10 up vs E11.5 * Ctrl
10457963	Gpr17	NM_001025381	1.16143e-007	2.75727	E11.5 * Sox10 up vs E11.5 * Ctrl
10564159	Snord116	AF241256	4.26278e-005	2.74214	E11.5 * Sox10 up vs E11.5 * Ctrl
10503198	Chd7	NM_001081417	4.45609e-008	2.73828	E11.5 * Sox10 up vs E11.5 * Ctrl
10435733	Igsf11	NM_170599	3.46992e-009	2.73133	E11.5 * Sox10 up vs E11.5 * Ctrl
10416175	Nefl	NM_010910	7.04373e-009	2.72728	E11.5 * Sox10 up vs E11.5 * Ctrl
10525016	Tbx3	NM_011535	1.58885e-012	2.72507	E11.5 * Sox10 up vs E11.5 * Ctrl
10572989	Slc10a7	NM_029736	1.2648e-011	2.72379	E11.5 * Sox10 up vs E11.5 * Ctrl
10374197	Ramp3	NM_019511	5.14411e-009	2.71099	E11.5 * Sox10 up vs E11.5 * Ctrl
10408850	Nedd9	NM_001111324	2.08077e-011	2.71022	E11.5 * Sox10 up vs E11.5 * Ctrl
10431697	Abcd2	NM_011994	5.05826e-007	2.70918	E11.5 * Sox10 up vs E11.5 * Ctrl
10494069	Tnrc4	NM_172434	8.86275e-006	2.70492	E11.5 * Sox10 up vs E11.5 * Ctrl
10564183	Snord116	AF241256	4.38386e-005	2.7032	E11.5 * Sox10 up vs E11.5 * Ctrl
10485198	Tspan18	NM_183180	1.26647e-009	2.69698	E11.5 * Sox10 up vs E11.5 * Ctrl
10352092	Zfp238	NM_001012330	2.5825e-010	2.68609	E11.5 * Sox10 up vs E11.5 * Ctrl
10362596	Fyn	NM_001122893	7.1676e-017	2.68161	E11.5 * Sox10 up vs E11.5 * Ctrl
10577190	Rasa3	NM_009025	1.36436e-009	2.67359	E11.5 * Sox10 up vs E11.5 * Ctrl
10430447	1700088E04Rik	NM_138581	7.59741e-008	2.67096	E11.5 * Sox10 up vs E11.5 * Ctrl
10539310	Pcgf1	NM_197992	1.43549e-007	2.65194	E11.5 * Sox10 up vs E11.5 * Ctrl
10399973	Hdac9	NM_024124	6.28336e-011	2.63992	E11.5 * Sox10 up vs E11.5 * Ctrl
10588755	Camkv	NM_145621	1.11592e-007	2.6364	E11.5 * Sox10 up vs E11.5 * Ctrl
10553917	Apba2	NM_007461	1.61335e-007	2.6189	E11.5 * Sox10 up vs E11.5 * Ctrl
10603289	Clcn5	NM_016691	2.30749e-012	2.6098	E11.5 * Sox10 up vs E11.5 * Ctrl
10522976	Rufy3	NM_027530	1.15943e-012	2.60722	E11.5 * Sox10 up vs E11.5 * Ctrl
10522285	C330024D21Rik	ENSMUST00000	0.00253377	2.60557	E11.5 * Sox10 up vs E11.5 * Ctrl
10458534	Pcdh1	NM_029357	4.6232e-008	2.60115	E11.5 * Sox10 up vs E11.5 * Ctrl
10499138	Dclk2	NM_027539	1.26311e-010	2.59986	E11.5 * Sox10 up vs E11.5 * Ctrl
10500114	Mllt11	NM_019914	1.60715e-011	2.59715	E11.5 * Sox10 up vs E11.5 * Ctrl
10495781	Bcar3	NM_013867	3.36788e-007	2.59422	E11.5 * Sox10 up vs E11.5 * Ctrl
10518947	Ajap1	NM_001099299	5.14658e-009	2.59227	E11.5 * Sox10 up vs E11.5 * Ctrl
10607877	Prps2	NM_026662	6.18491e-010	2.5873	E11.5 * Sox10 up vs E11.5 * Ctrl
10425257	Polr2f	NM_027231	8.82247e-007	2.57051	E11.5 * Sox10 up vs E11.5 * Ctrl
10458645	Ppp2r2b	NM_028392	5.03364e-011	2.57008	E11.5 * Sox10 up vs E11.5 * Ctrl
10365290	Chst11	NM_021439	1.65551e-008	2.55863	E11.5 * Sox10 up vs E11.5 * Ctrl
10503178	Chd7	NM_001081417	2.93839e-010	2.5471	E11.5 * Sox10 up vs E11.5 * Ctrl
10428619	Enpp2	NM_015744	3.25959e-008	2.54065	E11.5 * Sox10 up vs E11.5 * Ctrl
10503212	Chd7	NM_001081417	1.02412e-006	2.53968	E11.5 * Sox10 up vs E11.5 * Ctrl
10546685	Eif4e3	NM_025829	3.19895e-011	2.53866	E11.5 * Sox10 up vs E11.5 * Ctrl
10389590	Gdpd1	NM_025638	2.36828e-009	2.53851	E11.5 * Sox10 up vs E11.5 * Ctrl
10472820	Itga6	NM_008397	3.21586e-009	2.53648	E11.5 * Sox10 up vs E11.5 * Ctrl
10585048	Cadm1	NM_207675	1.01124e-011	2.53542	E11.5 * Sox10 up vs E11.5 * Ctrl
10371332	Aldh1l2	NM_153543	2.92178e-009	2.51278	E11.5 * Sox10 up vs E11.5 * Ctrl
10506360	Sgip1	NM_144906	2.11552e-011	2.50072	E11.5 * Sox10 up vs E11.5 * Ctrl
10424559	Khdrbs3	NM_010158	9.30259e-012	2.50017	E11.5 * Sox10 up vs E11.5 * Ctrl
10448967	Sox8	NM_011447	2.00581e-008	2.49864	E11.5 * Sox10 up vs E11.5 * Ctrl
10415132	Cmtm5	NM_026066	3.72455e-009	2.49521	E11.5 * Sox10 up vs E11.5 * Ctrl
10568668	Adam12	NM_007400	1.57656e-008	2.49343	E11.5 * Sox10 up vs E11.5 * Ctrl
10490665	Stmn3	NM_009133	1.8278e-008	2.49255	E11.5 * Sox10 up vs E11.5 * Ctrl
10481711	Stxbp1	NM_001113569	6.45469e-009	2.4911	E11.5 * Sox10 up vs E11.5 * Ctrl
10492021	Postn	NM_015784	1.69491e-009	2.48911	E11.5 * Sox10 up vs E11.5 * Ctrl
10507840	Heyl	NM_013905	1.5551e-006	2.48445	E11.5 * Sox10 up vs E11.5 * Ctrl
10455826	Megf10	NM_001001979	1.51058e-007	2.47828	E11.5 * Sox10 up vs E11.5 * Ctrl
10594301	Coro2b	NM_175484	4.18788e-008	2.47238	E11.5 * Sox10 up vs E11.5 * Ctrl
10503222	Chd7	NM_001081417	6.11386e-009	2.47054	E11.5 * Sox10 up vs E11.5 * Ctrl
10555027	Gab2	NM_010248	5.84695e-011	2.46857	E11.5 * Sox10 up vs E11.5 * Ctrl
10564165	Snord116	AF241256	0.000139768	2.46855	E11.5 * Sox10 up vs E11.5 * Ctrl

10479950	Cugbp2	NM_001110231	1.34035e-011	2.45452	E11.5 * Sox10 up vs E11.5 * Ctrl
10503176	Chd7	NM_001081417	3.74838e-009	2.45338	E11.5 * Sox10 up vs E11.5 * Ctrl
10521602	Cpeb2	NM_175937	5.71868e-007	2.45079	E11.5 * Sox10 up vs E11.5 * Ctrl
10403604	Lyst	NM_010748	2.28085e-010	2.44121	E11.5 * Sox10 up vs E11.5 * Ctrl
10385391	Cyfip2	NM_133769	1.22112e-009	2.43925	E11.5 * Sox10 up vs E11.5 * Ctrl
10468992	Frmd4a	NM_172475	3.25112e-010	2.439	E11.5 * Sox10 up vs E11.5 * Ctrl
10538482	Adcyap1r1	NM_007407	9.16724e-008	2.4329	E11.5 * Sox10 up vs E11.5 * Ctrl
10492165		---	3.10988e-005	2.42132	E11.5 * Sox10 up vs E11.5 * Ctrl
10418835	Slc18a3	NM_021712	7.44609e-006	2.41736	E11.5 * Sox10 up vs E11.5 * Ctrl
10574166	Cpne2	NM_153507	1.5169e-010	2.40879	E11.5 * Sox10 up vs E11.5 * Ctrl
10503196	Chd7	NM_001081417	2.85869e-008	2.408	E11.5 * Sox10 up vs E11.5 * Ctrl
10399360	Rhob	NM_007483	1.33582e-008	2.40675	E11.5 * Sox10 up vs E11.5 * Ctrl
10503208	Chd7	NM_001081417	1.22002e-007	2.40312	E11.5 * Sox10 up vs E11.5 * Ctrl
10503180	Chd7	NM_001081417	1.53593e-008	2.40271	E11.5 * Sox10 up vs E11.5 * Ctrl
10454369	Fhod3	NM_175276	3.47903e-008	2.39608	E11.5 * Sox10 up vs E11.5 * Ctrl
10531437	Scarb2	NM_007644	9.93544e-009	2.39222	E11.5 * Sox10 up vs E11.5 * Ctrl
10548051	Kcna6	NM_013568	4.83485e-008	2.39119	E11.5 * Sox10 up vs E11.5 * Ctrl
10417798	Kcnk5	NM_021542	4.37557e-007	2.38826	E11.5 * Sox10 up vs E11.5 * Ctrl
10503220	Chd7	NM_001081417	6.12023e-007	2.38733	E11.5 * Sox10 up vs E11.5 * Ctrl
10498710	Bche	NM_009738	1.66697e-007	2.38636	E11.5 * Sox10 up vs E11.5 * Ctrl
10562532	Zfp536	NM_172385	2.78642e-008	2.38457	E11.5 * Sox10 up vs E11.5 * Ctrl
10503172	Chd7	NM_001081417	0.000137491	2.38436	E11.5 * Sox10 up vs E11.5 * Ctrl
10567289	Syt17	NM_138649	1.20363e-008	2.37333	E11.5 * Sox10 up vs E11.5 * Ctrl
10457606	Kctd1	NM_134112	5.77809e-008	2.37252	E11.5 * Sox10 up vs E11.5 * Ctrl
10457644	Cdh2	NM_007664	8.82979e-013	2.37119	E11.5 * Sox10 up vs E11.5 * Ctrl
10566723	Lmo1	NM_057173	3.36058e-010	2.36436	E11.5 * Sox10 up vs E11.5 * Ctrl
10570516	Kbtbd11	NM_029116	5.37278e-011	2.36066	E11.5 * Sox10 up vs E11.5 * Ctrl
10422537	Nalcn	NM_177393	6.98227e-009	2.36003	E11.5 * Sox10 up vs E11.5 * Ctrl
10503216	Chd7	NM_001081417	4.32706e-009	2.35838	E11.5 * Sox10 up vs E11.5 * Ctrl
10423358	March11	NM_177597	0.000554442	2.3505	E11.5 * Sox10 up vs E11.5 * Ctrl
10595371	Hmgn3	NM_026122	1.21495e-007	2.34392	E11.5 * Sox10 up vs E11.5 * Ctrl
10446553	Epb4.1l3	NM_013813	8.69233e-012	2.34093	E11.5 * Sox10 up vs E11.5 * Ctrl
10358587	Hmcn1	NM_001024720	2.75489e-005	2.33864	E11.5 * Sox10 up vs E11.5 * Ctrl
10602068	Mid2	NM_011845	1.67081e-010	2.33466	E11.5 * Sox10 up vs E11.5 * Ctrl
10406434	Mef2c	NM_025282	5.73966e-011	2.3339	E11.5 * Sox10 up vs E11.5 * Ctrl
10602805	Mtap7d2	NM_001081124	1.71123e-008	2.33005	E11.5 * Sox10 up vs E11.5 * Ctrl
10523758	Lrrc8b	NM_001033550	3.54346e-008	2.3219	E11.5 * Sox10 up vs E11.5 * Ctrl
10486201	OTTMUSG00000	ENSMUST00000(	3.39156e-006	2.31245	E11.5 * Sox10 up vs E11.5 * Ctrl
10519612	9330182L06Rik	NM_172706	8.31918e-008	2.30872	E11.5 * Sox10 up vs E11.5 * Ctrl
10503190	Chd7	NM_001081417	5.60767e-008	2.30798	E11.5 * Sox10 up vs E11.5 * Ctrl
10407481	Pfkp	NM_019703	2.56275e-008	2.30296	E11.5 * Sox10 up vs E11.5 * Ctrl
10397002	Sipa1l1	NM_172579	1.27494e-009	2.30133	E11.5 * Sox10 up vs E11.5 * Ctrl
10545538	Ctnna2	NM_009819	1.60142e-007	2.29762	E11.5 * Sox10 up vs E11.5 * Ctrl
10544906	Ggct	NM_026637	3.96802e-008	2.29553	E11.5 * Sox10 up vs E11.5 * Ctrl
10476628	Otor	NM_020595	2.99128e-007	2.29398	E11.5 * Sox10 up vs E11.5 * Ctrl
10345777	Il1rl2	NM_133193	5.51812e-006	2.29123	E11.5 * Sox10 up vs E11.5 * Ctrl
10553537	Luzp2	NM_178705	7.45986e-006	2.28956	E11.5 * Sox10 up vs E11.5 * Ctrl
10607156	Dcx	NM_001110222	2.20306e-007	2.28758	E11.5 * Sox10 up vs E11.5 * Ctrl
10503166	Chd7	NM_001081417	6.77308e-006	2.28652	E11.5 * Sox10 up vs E11.5 * Ctrl
10556463	Arntl	NM_007489	1.60883e-009	2.28391	E11.5 * Sox10 up vs E11.5 * Ctrl
10353632	Bai3	NM_175642	6.86045e-009	2.28192	E11.5 * Sox10 up vs E11.5 * Ctrl
10366043	Dusp6	NM_026268	1.95265e-006	2.28119	E11.5 * Sox10 up vs E11.5 * Ctrl
10503210	Chd7	NM_001081417	1.82489e-007	2.28058	E11.5 * Sox10 up vs E11.5 * Ctrl
10383309		---	0.000321492	2.27939	E11.5 * Sox10 up vs E11.5 * Ctrl
10426244	Mapk8ip2	NM_021921	1.43583e-006	2.27213	E11.5 * Sox10 up vs E11.5 * Ctrl
10457820	Nol4	NM_199024	2.83266e-007	2.27203	E11.5 * Sox10 up vs E11.5 * Ctrl
10410547	Nkd2	NM_028186	4.91241e-007	2.2713	E11.5 * Sox10 up vs E11.5 * Ctrl
10399505	Greb1	NM_015764	1.33421e-007	2.26937	E11.5 * Sox10 up vs E11.5 * Ctrl
10375313	Ccnjl	NM_001045530	3.7533e-008	2.2685	E11.5 * Sox10 up vs E11.5 * Ctrl
10472984	Hoxd3	NM_010468	2.23504e-008	2.26495	E11.5 * Sox10 up vs E11.5 * Ctrl
10485745	Ano3	NM_001128103	0.000114088	2.2554	E11.5 * Sox10 up vs E11.5 * Ctrl
10500802	Atg4a	NM_174875	1.52692e-006	2.24327	E11.5 * Sox10 up vs E11.5 * Ctrl
10361807	Hivep2	NM_010437	6.99415e-007	2.24034	E11.5 * Sox10 up vs E11.5 * Ctrl
10503184	Chd7	NM_001081417	2.43076e-010	2.23923	E11.5 * Sox10 up vs E11.5 * Ctrl
10542414	Ptpro	NM_011216	1.52504e-007	2.23082	E11.5 * Sox10 up vs E11.5 * Ctrl
10493798	S100a16	NM_026416	3.74636e-007	2.22651	E11.5 * Sox10 up vs E11.5 * Ctrl

10452571	Ptprm	NM_008984	1.82581e-007	2.22024	E11.5 * Sox10 up vs E11.5 * Ctrl
10501971	Ank2	NM_178655	9.84001e-010	2.21546	E11.5 * Sox10 up vs E11.5 * Ctrl
10355456	Mreg	NM_001005423	1.18113e-006	2.21288	E11.5 * Sox10 up vs E11.5 * Ctrl
10607089	Acsl4	NM_207625	1.43359e-008	2.20921	E11.5 * Sox10 up vs E11.5 * Ctrl
10349826	ENSMUSG0000C	ENSMUST00000(	0.000447663	2.20551	E11.5 * Sox10 up vs E11.5 * Ctrl
10591706	Elavl3	NM_010487	1.02935e-006	2.20127	E11.5 * Sox10 up vs E11.5 * Ctrl
10603051	Ap1s2	NM_026887	5.82185e-005	2.19843	E11.5 * Sox10 up vs E11.5 * Ctrl
10503182	Chd7	NM_001081417	2.34807e-010	2.19201	E11.5 * Sox10 up vs E11.5 * Ctrl
10518751	Car6	NM_009802	0.000335354	2.18872	E11.5 * Sox10 up vs E11.5 * Ctrl
10564169	Snord116	AF241256	0.000377637	2.18859	E11.5 * Sox10 up vs E11.5 * Ctrl
10555197	Mtap6	NM_010837	3.20197e-009	2.18197	E11.5 * Sox10 up vs E11.5 * Ctrl
10497673	Zmat3	NM_009517	1.65202e-007	2.17721	E11.5 * Sox10 up vs E11.5 * Ctrl
10348493	Lrrfip1	NM_008515	5.95187e-011	2.17704	E11.5 * Sox10 up vs E11.5 * Ctrl
10360920	Tgfb2	NM_009367	6.27399e-015	2.16896	E11.5 * Sox10 up vs E11.5 * Ctrl
10368997	C130030K03Rik	AK048022	0.00143082	2.16828	E11.5 * Sox10 up vs E11.5 * Ctrl
10394593	Fam49a	NM_029758	4.09693e-007	2.16607	E11.5 * Sox10 up vs E11.5 * Ctrl
10345241	Dst	NM_134448	7.10839e-012	2.16437	E11.5 * Sox10 up vs E11.5 * Ctrl
10440522	Adamts1	NM_009621	7.81028e-009	2.16381	E11.5 * Sox10 up vs E11.5 * Ctrl
10476759	Rin2	NM_028724	3.2517e-008	2.16128	E11.5 * Sox10 up vs E11.5 * Ctrl
10358535	Hmcn1	NM_001024720	8.08789e-007	2.15851	E11.5 * Sox10 up vs E11.5 * Ctrl
10461057	Rcor2	NM_054048	2.98831e-006	2.15661	E11.5 * Sox10 up vs E11.5 * Ctrl
10595013	Tmod2	NM_016711	7.29885e-010	2.15638	E11.5 * Sox10 up vs E11.5 * Ctrl
10422227	Spry2	NM_011897	7.12523e-008	2.15268	E11.5 * Sox10 up vs E11.5 * Ctrl
10503186	Chd7	NM_001081417	4.63503e-009	2.15094	E11.5 * Sox10 up vs E11.5 * Ctrl
10574350	Mmp15	NM_008609	2.79327e-007	2.14375	E11.5 * Sox10 up vs E11.5 * Ctrl
10593497	Zc3h12c	ENSMUST00000(	2.97408e-007	2.14224	E11.5 * Sox10 up vs E11.5 * Ctrl
10575019	ENSMUSG0000C	ENSMUST00000(	0.00231203	2.1367	E11.5 * Sox10 up vs E11.5 * Ctrl
10587299	Ick	NM_019987	4.84829e-010	2.13669	E11.5 * Sox10 up vs E11.5 * Ctrl
10605431	Rab39b	NM_175122	1.08482e-006	2.13508	E11.5 * Sox10 up vs E11.5 * Ctrl
10598996	EG331392	NM_198633	2.92141e-007	2.13036	E11.5 * Sox10 up vs E11.5 * Ctrl
10552075	Lgi4	NM_144556	1.7421e-007	2.12805	E11.5 * Sox10 up vs E11.5 * Ctrl
10350136	Csrp1	NM_007791	4.93155e-008	2.12511	E11.5 * Sox10 up vs E11.5 * Ctrl
10358557	Hmcn1	NM_001024720	1.90825e-006	2.12366	E11.5 * Sox10 up vs E11.5 * Ctrl
10527158	Fscn1	NM_007984	0.000244416	2.11828	E11.5 * Sox10 up vs E11.5 * Ctrl
10518585	Kif1b	NM_207682	1.19769e-010	2.11542	E11.5 * Sox10 up vs E11.5 * Ctrl
10363455	Pcbd1	NM_025273	5.91175e-007	2.11355	E11.5 * Sox10 up vs E11.5 * Ctrl
10607113	Rgs3	NM_134257	1.95435e-007	2.11279	E11.5 * Sox10 up vs E11.5 * Ctrl
10490129	Bmp7	NM_007557	1.2244e-007	2.11193	E11.5 * Sox10 up vs E11.5 * Ctrl
10358547	Hmcn1	NM_001024720	2.66412e-005	2.11165	E11.5 * Sox10 up vs E11.5 * Ctrl
10497817	Anxa5	NM_009673	8.22706e-009	2.10705	E11.5 * Sox10 up vs E11.5 * Ctrl
10408359	Nrsn1	NM_009513	2.17593e-006	2.10564	E11.5 * Sox10 up vs E11.5 * Ctrl
10470564	Ralgds	NM_001145835	8.04957e-007	2.10461	E11.5 * Sox10 up vs E11.5 * Ctrl
10458685	Jakmip2	ENSMUST00000(	2.02969e-005	2.10383	E11.5 * Sox10 up vs E11.5 * Ctrl
10535866	Ubl3	NM_011908	3.94323e-011	2.10212	E11.5 * Sox10 up vs E11.5 * Ctrl
10375265	Atp10b	NM_176999	9.47984e-008	2.09708	E11.5 * Sox10 up vs E11.5 * Ctrl
10540227	Kbtbd8	NM_001008785	2.01144e-005	2.09669	E11.5 * Sox10 up vs E11.5 * Ctrl
10518484	Fbxo44	NM_173401	5.50795e-008	2.09326	E11.5 * Sox10 up vs E11.5 * Ctrl
10582580		---	0.000161498	2.08881	E11.5 * Sox10 up vs E11.5 * Ctrl
10564211	Snrpn	NM_013670	1.48642e-011	2.08436	E11.5 * Sox10 up vs E11.5 * Ctrl
10460118	Socs6	NM_018821	1.84406e-007	2.08403	E11.5 * Sox10 up vs E11.5 * Ctrl
10358525	Hmcn1	NM_001024720	9.90032e-007	2.0766	E11.5 * Sox10 up vs E11.5 * Ctrl
10474064	Trp53i11	NM_001025246	1.9895e-005	2.07398	E11.5 * Sox10 up vs E11.5 * Ctrl
10399421	Mycn	NM_008709	5.36004e-008	2.0731	E11.5 * Sox10 up vs E11.5 * Ctrl
10356712	Kif1a	NM_008440	2.53058e-009	2.07085	E11.5 * Sox10 up vs E11.5 * Ctrl
10520527	Dpysl5	NM_023047	2.1541e-007	2.07011	E11.5 * Sox10 up vs E11.5 * Ctrl
10496727	Ddah1	NM_026993	2.69499e-009	2.06975	E11.5 * Sox10 up vs E11.5 * Ctrl
10432540	Lima1	NM_001113545	1.82744e-011	2.06902	E11.5 * Sox10 up vs E11.5 * Ctrl
10577586	Ap3m2	NM_001122820	7.56392e-009	2.06814	E11.5 * Sox10 up vs E11.5 * Ctrl
10466410	Psat1	NM_177420	2.09982e-008	2.06764	E11.5 * Sox10 up vs E11.5 * Ctrl
10372338	EG368203	NM_203660	0.000283142	2.06714	E11.5 * Sox10 up vs E11.5 * Ctrl
10407072	Elovl7	NM_029001	5.77012e-005	2.0657	E11.5 * Sox10 up vs E11.5 * Ctrl
10466976	Gldc	NM_138595	9.21042e-008	2.06378	E11.5 * Sox10 up vs E11.5 * Ctrl
10406817	Enc1	NM_007930	1.6355e-008	2.06368	E11.5 * Sox10 up vs E11.5 * Ctrl
10566668	Socs6	NM_018821	0.000103469	2.06056	E11.5 * Sox10 up vs E11.5 * Ctrl
10501456	Fam102b	ENSMUST00000(	3.7877e-008	2.05941	E11.5 * Sox10 up vs E11.5 * Ctrl
10455942	A730017C20Rik	NM_173759	1.89447e-006	2.055	E11.5 * Sox10 up vs E11.5 * Ctrl

10549200	Sox5	NM_011444	3.07179e-007	2.0545	E11.5 * Sox10 up vs E11.5 * Ctrl
10503902	Cnr1	NM_007726	5.81664e-007	2.05337	E11.5 * Sox10 up vs E11.5 * Ctrl
10421810	1190002H23Rik	NM_025427	2.624e-007	2.05261	E11.5 * Sox10 up vs E11.5 * Ctrl
10602090	Atg4a	NM_174875	5.93693e-007	2.04926	E11.5 * Sox10 up vs E11.5 * Ctrl
10492826	Fbxw7	NM_080428	4.07368e-011	2.04918	E11.5 * Sox10 up vs E11.5 * Ctrl
10452734	Alk	NM_007439	2.86926e-008	2.04862	E11.5 * Sox10 up vs E11.5 * Ctrl
10498210	Nbea	NM_030595	1.95937e-011	2.04817	E11.5 * Sox10 up vs E11.5 * Ctrl
10490559	Chrna4	NM_015730	4.91565e-006	2.03996	E11.5 * Sox10 up vs E11.5 * Ctrl
10503174	Chd7	NM_001081417	7.0244e-008	2.03915	E11.5 * Sox10 up vs E11.5 * Ctrl
10503188	Chd7	NM_001081417	3.41158e-006	2.03882	E11.5 * Sox10 up vs E11.5 * Ctrl
10538356	Chn2	NM_023543	1.79931e-007	2.03359	E11.5 * Sox10 up vs E11.5 * Ctrl
10595496	Snap91	NM_013669	3.56733e-006	2.03	E11.5 * Sox10 up vs E11.5 * Ctrl
10499093		---	2.48055e-006	2.02848	E11.5 * Sox10 up vs E11.5 * Ctrl
10406456	AY512938	AY512938	0.000131238	2.02812	E11.5 * Sox10 up vs E11.5 * Ctrl
10494735	Gdap2	NM_010269	2.77136e-007	2.02369	E11.5 * Sox10 up vs E11.5 * Ctrl
10495285	Sort1	NM_019972	2.47966e-010	2.02269	E11.5 * Sox10 up vs E11.5 * Ctrl
10365471	Fbxo7	NM_153195	8.03913e-010	2.01677	E11.5 * Sox10 up vs E11.5 * Ctrl
10412066	Rab3c	NM_023852	1.76728e-006	2.01097	E11.5 * Sox10 up vs E11.5 * Ctrl
10421100	Nefm	NM_008691	3.88777e-010	2.00735	E11.5 * Sox10 up vs E11.5 * Ctrl
10495623	ENSMUSG0000C	ENSMUST00000	0.000240809	2.00455	E11.5 * Sox10 up vs E11.5 * Ctrl
10503204	Chd7	NM_001081417	3.7069e-007	2.00316	E11.5 * Sox10 up vs E11.5 * Ctrl
10392910	C630004H02Rik	BC024617	0.00012033	2.00299	E11.5 * Sox10 up vs E11.5 * Ctrl
10469138	4930412O13Rik	NR_024257	0.000298248	2.0007	E11.5 * Sox10 up vs E11.5 * Ctrl
10358577	Hmcn1	NM_001024720	2.11941e-005	1.99838	E11.5 * Sox10 up vs E11.5 * Ctrl
10548735	Dusp16	NM_130447	6.04733e-010	1.99644	E11.5 * Sox10 up vs E11.5 * Ctrl
10594652	Lactb	NM_030717	7.00417e-006	1.99551	E11.5 * Sox10 up vs E11.5 * Ctrl
10593492	Zc3h12c	AK220416	2.73226e-010	1.99508	E11.5 * Sox10 up vs E11.5 * Ctrl
10399457	Akr1b3	NM_009658	9.47114e-009	1.99327	E11.5 * Sox10 up vs E11.5 * Ctrl
10347781	9430031J16Rik	BC082310	1.53734e-006	1.99287	E11.5 * Sox10 up vs E11.5 * Ctrl
10564482	Synm	NM_201639	9.0395e-009	1.97983	E11.5 * Sox10 up vs E11.5 * Ctrl
10462918	Tmem20	NM_175507	1.69055e-009	1.97874	E11.5 * Sox10 up vs E11.5 * Ctrl
10534960	Gje1	NM_080450	0.000117206	1.97618	E11.5 * Sox10 up vs E11.5 * Ctrl
10436623	Chodl	NM_139134	0.000312077	1.97608	E11.5 * Sox10 up vs E11.5 * Ctrl
10418895	Zfp488	NM_001013777	7.17356e-008	1.97293	E11.5 * Sox10 up vs E11.5 * Ctrl
10556553	Insc	NM_173767	8.58547e-007	1.9723	E11.5 * Sox10 up vs E11.5 * Ctrl
10467139	Lipa	NM_021460	9.53134e-009	1.96696	E11.5 * Sox10 up vs E11.5 * Ctrl
10601152	Nlgn3	NM_172932	2.52865e-008	1.96426	E11.5 * Sox10 up vs E11.5 * Ctrl
10359480	Dnm3	NM_001038619	3.92813e-007	1.96399	E11.5 * Sox10 up vs E11.5 * Ctrl
10582582		---	0.00831065	1.96174	E11.5 * Sox10 up vs E11.5 * Ctrl
10582584		---	0.00831065	1.96174	E11.5 * Sox10 up vs E11.5 * Ctrl
10404975	Id4	NM_031166	2.29482e-007	1.96102	E11.5 * Sox10 up vs E11.5 * Ctrl
10526191	Gatsl2	BC026208	1.00182e-006	1.96095	E11.5 * Sox10 up vs E11.5 * Ctrl
10428857	Mtss1	NM_001146180	1.254e-008	1.95913	E11.5 * Sox10 up vs E11.5 * Ctrl
10481845	Fam125b	BC059907	1.23799e-007	1.95615	E11.5 * Sox10 up vs E11.5 * Ctrl
10397912	9030205A07Rik	AB257853	3.27118e-008	1.9558	E11.5 * Sox10 up vs E11.5 * Ctrl
10448925	Cacna1h	NM_021415	1.49874e-006	1.9558	E11.5 * Sox10 up vs E11.5 * Ctrl
10358549	Hmcn1	NM_001024720	3.69866e-006	1.95444	E11.5 * Sox10 up vs E11.5 * Ctrl
10358613	Hmcn1	NM_001024720	1.04794e-005	1.95393	E11.5 * Sox10 up vs E11.5 * Ctrl
10436456	Pros1	NM_011173	2.9622e-007	1.9527	E11.5 * Sox10 up vs E11.5 * Ctrl
10503170	Chd7	NM_001081417	1.85755e-009	1.95221	E11.5 * Sox10 up vs E11.5 * Ctrl
10471424	Fam102a	NM_153560	2.19014e-006	1.95078	E11.5 * Sox10 up vs E11.5 * Ctrl
10503192	Chd7	NM_001081417	8.25719e-007	1.94264	E11.5 * Sox10 up vs E11.5 * Ctrl
10595298	Filip1	NM_001081243	6.01437e-005	1.94157	E11.5 * Sox10 up vs E11.5 * Ctrl
10407841	Hecw1	NM_001081348	8.87033e-005	1.93859	E11.5 * Sox10 up vs E11.5 * Ctrl
10362186	Moxd1	NM_021509	4.53292e-005	1.93755	E11.5 * Sox10 up vs E11.5 * Ctrl
10497920	Ankrd50	NM_001033198	2.02261e-009	1.93647	E11.5 * Sox10 up vs E11.5 * Ctrl
10347036	Mtap2	NM_001039934	6.87967e-010	1.93505	E11.5 * Sox10 up vs E11.5 * Ctrl
10599654	Cxx1c	NM_028375	2.59951e-006	1.93479	E11.5 * Sox10 up vs E11.5 * Ctrl
10358521	Hmcn1	NM_001024720	0.000565918	1.93478	E11.5 * Sox10 up vs E11.5 * Ctrl
10370242	Pcbp3	NM_021568	2.2695e-008	1.93313	E11.5 * Sox10 up vs E11.5 * Ctrl
10436978	Cbr3	NM_173047	0.000414487	1.93222	E11.5 * Sox10 up vs E11.5 * Ctrl
10448081	Rgmb	NM_178615	3.49297e-011	1.92751	E11.5 * Sox10 up vs E11.5 * Ctrl
10403413	Idi1	NM_145360	0.000367848	1.92068	E11.5 * Sox10 up vs E11.5 * Ctrl
10374406	Cnrip1	NM_029861	5.24098e-008	1.91944	E11.5 * Sox10 up vs E11.5 * Ctrl
10458555	Spry4	NM_011898	2.54934e-006	1.91926	E11.5 * Sox10 up vs E11.5 * Ctrl
10355974	Wdfy1	NM_001111279	5.77765e-008	1.91649	E11.5 * Sox10 up vs E11.5 * Ctrl

10391084	Hap1	NM_010404	5.53947e-007	1.91555	E11.5 * Sox10 up vs E11.5 * Ctrl
10455374	Stk32a	NM_178749	3.33285e-007	1.91486	E11.5 * Sox10 up vs E11.5 * Ctrl
10565570	4632434I11Rik	NM_001080995	2.09346e-006	1.91034	E11.5 * Sox10 up vs E11.5 * Ctrl
10462702	Hectd2	NM_172637	4.70055e-006	1.90913	E11.5 * Sox10 up vs E11.5 * Ctrl
10358597	Hmcn1	NM_001024720	5.74468e-005	1.90749	E11.5 * Sox10 up vs E11.5 * Ctrl
10381939	Tanc2	NM_181071	3.95405e-008	1.90655	E11.5 * Sox10 up vs E11.5 * Ctrl
10492335	Rap2b	NM_028712	2.95129e-008	1.90342	E11.5 * Sox10 up vs E11.5 * Ctrl
10509127	Fuca1	NM_024243	1.00057e-007	1.90234	E11.5 * Sox10 up vs E11.5 * Ctrl
10366391	Kcnc2	NM_001025581	1.91185e-006	1.89938	E11.5 * Sox10 up vs E11.5 * Ctrl
10456237	St8sia3	NM_009182	6.75141e-006	1.89775	E11.5 * Sox10 up vs E11.5 * Ctrl
10474373	Kcna4	NM_021275	9.44713e-005	1.89773	E11.5 * Sox10 up vs E11.5 * Ctrl
10557992	Bag3	NM_013863	3.61748e-008	1.89632	E11.5 * Sox10 up vs E11.5 * Ctrl
10588509	Pcbp4	NM_021567	0.000234644	1.89545	E11.5 * Sox10 up vs E11.5 * Ctrl
10425265	ENSMUSG0000(	ENSMUST00000	5.56527e-005	1.89519	E11.5 * Sox10 up vs E11.5 * Ctrl
10358555	Hmcn1	NM_001024720	9.07266e-006	1.8934	E11.5 * Sox10 up vs E11.5 * Ctrl
10454286	Mapre2	NM_153058	3.07438e-010	1.89308	E11.5 * Sox10 up vs E11.5 * Ctrl
10459671	Dcc	NM_007831	1.29953e-008	1.89251	E11.5 * Sox10 up vs E11.5 * Ctrl
10577315	Angpt2	NM_007426	4.10095e-008	1.89136	E11.5 * Sox10 up vs E11.5 * Ctrl
10485402	Fjx1	NM_010218	1.04136e-010	1.88904	E11.5 * Sox10 up vs E11.5 * Ctrl
10472893	B230120H23Rik	NM_023057	7.63984e-007	1.88882	E11.5 * Sox10 up vs E11.5 * Ctrl
10362005	Ahi1	NM_026203	7.76454e-007	1.88868	E11.5 * Sox10 up vs E11.5 * Ctrl
10427496	Egflam	NM_178748	9.73721e-009	1.88593	E11.5 * Sox10 up vs E11.5 * Ctrl
10567546	Crym	NM_016669	1.38354e-007	1.88435	E11.5 * Sox10 up vs E11.5 * Ctrl
10415784	Trim13	NM_023233	0.000297246	1.8839	E11.5 * Sox10 up vs E11.5 * Ctrl
10485309	E530001K10Rik	ENSMUST00000(	0.000835833	1.8834	E11.5 * Sox10 up vs E11.5 * Ctrl
10491780	Hspa4l	NM_011020	6.96698e-006	1.8828	E11.5 * Sox10 up vs E11.5 * Ctrl
10513061	Ctnnal1	NM_018761	1.25894e-008	1.88011	E11.5 * Sox10 up vs E11.5 * Ctrl
10420988	Dpysl2	NM_009955	1.7811e-005	1.8762	E11.5 * Sox10 up vs E11.5 * Ctrl
10506452	AY512949	AY512949	0.000180523	1.8736	E11.5 * Sox10 up vs E11.5 * Ctrl
10406852	Cnn3	NM_028044	5.4667e-005	1.87237	E11.5 * Sox10 up vs E11.5 * Ctrl
10514466	Jun	NM_010591	5.75912e-005	1.86881	E11.5 * Sox10 up vs E11.5 * Ctrl
10591853	Tbx20	NM_194263	1.1064e-005	1.86204	E11.5 * Sox10 up vs E11.5 * Ctrl
10503168	Chd7	NM_001081417	3.05323e-007	1.86195	E11.5 * Sox10 up vs E11.5 * Ctrl
10357590	Dyrk3	NM_145508	1.38414e-005	1.86182	E11.5 * Sox10 up vs E11.5 * Ctrl
10367746	Sash1	NM_175155	9.6173e-008	1.861	E11.5 * Sox10 up vs E11.5 * Ctrl
10540233	Fam19a1	NM_182808	3.75694e-006	1.8588	E11.5 * Sox10 up vs E11.5 * Ctrl
10505008	Slc44a1	NM_133891	6.28435e-010	1.85623	E11.5 * Sox10 up vs E11.5 * Ctrl
10358633	Hmcn1	NM_001024720	0.000206872	1.85424	E11.5 * Sox10 up vs E11.5 * Ctrl
10590383	Deb1	NM_026794	1.0852e-008	1.85395	E11.5 * Sox10 up vs E11.5 * Ctrl
10598071		---	0.00130496	1.84951	E11.5 * Sox10 up vs E11.5 * Ctrl
10358635	Hmcn1	NM_001024720	4.72402e-005	1.84499	E11.5 * Sox10 up vs E11.5 * Ctrl
10549594	Ttyh1	NM_021324	3.03089e-008	1.8448	E11.5 * Sox10 up vs E11.5 * Ctrl
10378855	Ssh2	ENSMUST00000(	0.00556045	1.84242	E11.5 * Sox10 up vs E11.5 * Ctrl
10606174	Nap1l2	NM_008671	0.000128094	1.84168	E11.5 * Sox10 up vs E11.5 * Ctrl
10553773	Gabrb3	NM_008071	1.26028e-007	1.83902	E11.5 * Sox10 up vs E11.5 * Ctrl
10372557	Gm239	NM_001033333	1.05238e-005	1.83044	E11.5 * Sox10 up vs E11.5 * Ctrl
10607302	Gnl3l	NM_198110	6.97903e-010	1.83023	E11.5 * Sox10 up vs E11.5 * Ctrl
10425945	Fbln1	NM_010180	2.11048e-006	1.8284	E11.5 * Sox10 up vs E11.5 * Ctrl
10350697	Nmnat2	NM_175460	8.71497e-006	1.82796	E11.5 * Sox10 up vs E11.5 * Ctrl
10358533	Hmcn1	NM_001024720	6.56566e-006	1.82381	E11.5 * Sox10 up vs E11.5 * Ctrl
10504518	Polr1e	NM_022811	8.51622e-009	1.82242	E11.5 * Sox10 up vs E11.5 * Ctrl
10576639	Nrp1	NM_008737	2.03984e-007	1.82158	E11.5 * Sox10 up vs E11.5 * Ctrl
10454580	Bin1	NM_009668	2.93169e-005	1.82139	E11.5 * Sox10 up vs E11.5 * Ctrl
10571312	Dusp4	NM_176933	2.31885e-005	1.82033	E11.5 * Sox10 up vs E11.5 * Ctrl
10368227	Ube2q2	NM_180600	1.80269e-007	1.81861	E11.5 * Sox10 up vs E11.5 * Ctrl
10594048	Islr2	NM_177193	6.80538e-006	1.8175	E11.5 * Sox10 up vs E11.5 * Ctrl
10571815	Gpm6a	NM_153581	0.00013693	1.8173	E11.5 * Sox10 up vs E11.5 * Ctrl
10482762	Idi1	NM_145360	0.000208667	1.81632	E11.5 * Sox10 up vs E11.5 * Ctrl
10358531	Hmcn1	NM_001024720	2.51085e-005	1.81315	E11.5 * Sox10 up vs E11.5 * Ctrl
10476443	Plcb4	NM_013829	7.85889e-008	1.8126	E11.5 * Sox10 up vs E11.5 * Ctrl
10469151	Itih5	NM_172471	6.7998e-008	1.81229	E11.5 * Sox10 up vs E11.5 * Ctrl
10508651	Sdc3	NM_011520	6.40653e-006	1.81141	E11.5 * Sox10 up vs E11.5 * Ctrl
10598833	Chst7	NM_021715	0.000416643	1.81079	E11.5 * Sox10 up vs E11.5 * Ctrl
10438017	Fgd4	NM_139232	1.2791e-006	1.80864	E11.5 * Sox10 up vs E11.5 * Ctrl
10378833	Ssh2	NM_177710	8.19647e-008	1.80584	E11.5 * Sox10 up vs E11.5 * Ctrl
10595831	Zbtb38	NM_175537	1.15614e-006	1.80367	E11.5 * Sox10 up vs E11.5 * Ctrl

10488387	Napb	NM_019632	3.81631e-007	1.80348	E11.5 * Sox10 up vs E11.5 * Ctrl
10358637	Hmcn1	NM_001024720	9.3891e-006	1.80329	E11.5 * Sox10 up vs E11.5 * Ctrl
10419288	Gch1	NM_008102	2.29702e-006	1.80234	E11.5 * Sox10 up vs E11.5 * Ctrl
10358529	Hmcn1	NM_001024720	5.1213e-006	1.80121	E11.5 * Sox10 up vs E11.5 * Ctrl
10476355	Chgb	NM_007694	8.00325e-005	1.79915	E11.5 * Sox10 up vs E11.5 * Ctrl
10582162	Cotl1	NM_028071	0.000688234	1.79895	E11.5 * Sox10 up vs E11.5 * Ctrl
10408613	Tubb2b	NM_023716	8.34231e-005	1.79733	E11.5 * Sox10 up vs E11.5 * Ctrl
10439092	1700021K19Rik	BC060601	5.20962e-008	1.79733	E11.5 * Sox10 up vs E11.5 * Ctrl
10350951	Tnr	NM_022312	5.11929e-006	1.79486	E11.5 * Sox10 up vs E11.5 * Ctrl
10416887	Slain1	NM_198014	2.64228e-005	1.79486	E11.5 * Sox10 up vs E11.5 * Ctrl
10581914	Chst5	NM_019950	8.24854e-007	1.79331	E11.5 * Sox10 up vs E11.5 * Ctrl
10595392	Elovl4	NM_148941	0.000202723	1.79276	E11.5 * Sox10 up vs E11.5 * Ctrl
10441456	Synj2	NM_011523	2.43581e-007	1.79245	E11.5 * Sox10 up vs E11.5 * Ctrl
10449034	Fam173a	BC096050	5.62478e-007	1.78876	E11.5 * Sox10 up vs E11.5 * Ctrl
10421526	Rb1	NM_009029	1.13823e-006	1.78793	E11.5 * Sox10 up vs E11.5 * Ctrl
10596812	6230427J02Rik	NM_026597	3.91126e-007	1.78695	E11.5 * Sox10 up vs E11.5 * Ctrl
10504234	Unc13b	NM_001081413	3.19543e-008	1.7868	E11.5 * Sox10 up vs E11.5 * Ctrl
10516932	Sesn2	NM_144907	3.60576e-005	1.78621	E11.5 * Sox10 up vs E11.5 * Ctrl
10585494	Ube2q2	NM_180600	8.6035e-008	1.78612	E11.5 * Sox10 up vs E11.5 * Ctrl
10501963	Ugt8a	NM_011674	0.000452719	1.78595	E11.5 * Sox10 up vs E11.5 * Ctrl
10414417	Peli2	NM_033602	2.02876e-007	1.78451	E11.5 * Sox10 up vs E11.5 * Ctrl
10371092	Atcay	NM_178662	4.81947e-005	1.78449	E11.5 * Sox10 up vs E11.5 * Ctrl
10596200	Tmem108	NM_178638	0.000844192	1.78268	E11.5 * Sox10 up vs E11.5 * Ctrl
10568651	Dhx32	NM_133941	1.88898e-007	1.78265	E11.5 * Sox10 up vs E11.5 * Ctrl
10409660	Gkap1	NM_019832	3.94224e-005	1.77772	E11.5 * Sox10 up vs E11.5 * Ctrl
10368981	Lin28b	NM_001031772	1.38257e-006	1.77769	E11.5 * Sox10 up vs E11.5 * Ctrl
10408838	Elovl2	NM_019423	1.54109e-005	1.77727	E11.5 * Sox10 up vs E11.5 * Ctrl
10471675	Glo1	NM_025374	3.9725e-006	1.77388	E11.5 * Sox10 up vs E11.5 * Ctrl
10415911	Kif13b	NM_001081177	5.66651e-006	1.77251	E11.5 * Sox10 up vs E11.5 * Ctrl
10522655	C530008M17Rik	ENSMUST00000	0.00336662	1.77073	E11.5 * Sox10 up vs E11.5 * Ctrl
10358601	Hmcn1	NM_001024720	4.29181e-005	1.768	E11.5 * Sox10 up vs E11.5 * Ctrl
10474814	Disp2	NM_170593	3.7125e-005	1.7673	E11.5 * Sox10 up vs E11.5 * Ctrl
10409021	Tpmt	NM_016785	8.39044e-007	1.76695	E11.5 * Sox10 up vs E11.5 * Ctrl
10453518	Nrxn1	NM_020252	6.63246e-005	1.76624	E11.5 * Sox10 up vs E11.5 * Ctrl
10438626	Etv5	NM_023794	2.42682e-005	1.76523	E11.5 * Sox10 up vs E11.5 * Ctrl
10474467	Muc15	NM_172979	0.00431602	1.7646	E11.5 * Sox10 up vs E11.5 * Ctrl
10596148	Trf	NM_133977	7.09727e-007	1.76415	E11.5 * Sox10 up vs E11.5 * Ctrl
10389581	Ypel2	NM_001005341	9.69209e-008	1.76391	E11.5 * Sox10 up vs E11.5 * Ctrl
10582275	Slc7a5	NM_011404	0.000280573	1.76383	E11.5 * Sox10 up vs E11.5 * Ctrl
10392388	Prkca	NM_011101	1.41948e-007	1.76214	E11.5 * Sox10 up vs E11.5 * Ctrl
10358551	Hmcn1	NM_001024720	6.31058e-005	1.7612	E11.5 * Sox10 up vs E11.5 * Ctrl
10564262	Mkrn3	NM_011746	0.000151016	1.76009	E11.5 * Sox10 up vs E11.5 * Ctrl
10514561	E130114P18Rik	ENSMUST00000(	1.60447e-005	1.75823	E11.5 * Sox10 up vs E11.5 * Ctrl
10354494	Nab1	NM_008667	9.80434e-007	1.75801	E11.5 * Sox10 up vs E11.5 * Ctrl
10368101	D10Bwg1379e	NM_001033258	1.10376e-006	1.7563	E11.5 * Sox10 up vs E11.5 * Ctrl
10358575	Hmcn1	NM_001024720	3.02107e-005	1.75559	E11.5 * Sox10 up vs E11.5 * Ctrl
10358648	Hmcn1	NM_001024720	3.62925e-005	1.75514	E11.5 * Sox10 up vs E11.5 * Ctrl
10451679	Daam2	NM_001008231	1.77844e-006	1.75352	E11.5 * Sox10 up vs E11.5 * Ctrl
10464583	Gstp1	NM_013541	2.75615e-006	1.75127	E11.5 * Sox10 up vs E11.5 * Ctrl
10499091	Dear1	NM_001040461	1.4161e-005	1.75002	E11.5 * Sox10 up vs E11.5 * Ctrl
10603316	2010204K13Rik	AF038507	9.97935e-007	1.74939	E11.5 * Sox10 up vs E11.5 * Ctrl
10578521	Snx25	NM_207213	2.44811e-007	1.7486	E11.5 * Sox10 up vs E11.5 * Ctrl
10590844	9030420J04Rik	BC137891	8.48079e-008	1.74844	E11.5 * Sox10 up vs E11.5 * Ctrl
10433445	Abat	NM_172961	8.82007e-005	1.74769	E11.5 * Sox10 up vs E11.5 * Ctrl
10392943	Hn1	NM_008258	1.24603e-006	1.74611	E11.5 * Sox10 up vs E11.5 * Ctrl
10356020	Dock10	NM_175291	1.36981e-005	1.74604	E11.5 * Sox10 up vs E11.5 * Ctrl
10543859	Akr1b3	NM_009658	3.33623e-009	1.74386	E11.5 * Sox10 up vs E11.5 * Ctrl
10558001	Inpp5f	NM_178641	7.82617e-007	1.73951	E11.5 * Sox10 up vs E11.5 * Ctrl
10469577	ENSMUSG0000C	ENSMUST00000	0.00012346	1.73935	E11.5 * Sox10 up vs E11.5 * Ctrl
10439009	Apod	NM_007470	2.41117e-005	1.7383	E11.5 * Sox10 up vs E11.5 * Ctrl
10503161	Chd7	NM_001081417	4.00151e-008	1.73793	E11.5 * Sox10 up vs E11.5 * Ctrl
10592471	Gramd1b	NM_172768	3.88008e-006	1.7379	E11.5 * Sox10 up vs E11.5 * Ctrl
10563303	Bax	NM_007527	1.67515e-006	1.73741	E11.5 * Sox10 up vs E11.5 * Ctrl
10495854	Prss12	NM_008939	2.75986e-007	1.73695	E11.5 * Sox10 up vs E11.5 * Ctrl
10441565	Rps6ka2	NM_011299	9.6352e-007	1.73695	E11.5 * Sox10 up vs E11.5 * Ctrl
10392056	Cyb561	NM_007805	4.3389e-005	1.7352	E11.5 * Sox10 up vs E11.5 * Ctrl

10426656	Prph	NM_013639	3.22017e-006	1.73498	E11.5 * Sox10 up vs E11.5 * Ctrl
10430519	Csnk1e	NM_013767	1.45885e-006	1.73495	E11.5 * Sox10 up vs E11.5 * Ctrl
10358605	Hmcn1	NM_001024720	1.54042e-005	1.73481	E11.5 * Sox10 up vs E11.5 * Ctrl
10391146	Acly	NM_134037	8.54817e-006	1.73463	E11.5 * Sox10 up vs E11.5 * Ctrl
10464283	ENSMUSG0000C	ENSMUST00000(	1.4315e-006	1.73443	E11.5 * Sox10 up vs E11.5 * Ctrl
10354768	Akr1b3	NM_009658	2.68763e-009	1.73356	E11.5 * Sox10 up vs E11.5 * Ctrl
10520304	Actr3b	NM_001004365	1.04825e-005	1.73346	E11.5 * Sox10 up vs E11.5 * Ctrl
10412038	Zswim6	NM_145456	1.11785e-009	1.73205	E11.5 * Sox10 up vs E11.5 * Ctrl
10399470	Trib2	NM_144551	1.39385e-007	1.73119	E11.5 * Sox10 up vs E11.5 * Ctrl
10421046	Dock5	NM_177780	2.24081e-009	1.7288	E11.5 * Sox10 up vs E11.5 * Ctrl
10483719	Chn1	NM_001113246	6.65749e-008	1.72464	E11.5 * Sox10 up vs E11.5 * Ctrl
10571860	Hand2	NM_010402	3.62542e-007	1.72452	E11.5 * Sox10 up vs E11.5 * Ctrl
10358607	Hmcn1	NM_001024720	6.49967e-006	1.72381	E11.5 * Sox10 up vs E11.5 * Ctrl
10599120	Dock11	NM_001009947	9.92168e-007	1.72213	E11.5 * Sox10 up vs E11.5 * Ctrl
10350896	Astn1	NM_007495	3.06686e-006	1.72195	E11.5 * Sox10 up vs E11.5 * Ctrl
10369783	Zfp365	NM_178679	3.55396e-007	1.72128	E11.5 * Sox10 up vs E11.5 * Ctrl
10603706	Med4	NM_026119	8.56454e-005	1.72093	E11.5 * Sox10 up vs E11.5 * Ctrl
10358595	Hmcn1	NM_001024720	0.000103558	1.72044	E11.5 * Sox10 up vs E11.5 * Ctrl
10495316	Psrc1	NM_019976	7.16321e-006	1.72022	E11.5 * Sox10 up vs E11.5 * Ctrl
10496091	Lef1	NM_010703	2.98747e-006	1.71769	E11.5 * Sox10 up vs E11.5 * Ctrl
10519949	A630072M18Rik	BC020055	0.000599991	1.71752	E11.5 * Sox10 up vs E11.5 * Ctrl
10498284	Wwtr1	NM_133784	6.09017e-008	1.71685	E11.5 * Sox10 up vs E11.5 * Ctrl
10595805	Rasa2	NM_053268	4.28373e-007	1.71655	E11.5 * Sox10 up vs E11.5 * Ctrl
10540333	Cntn6	NM_017383	6.50657e-005	1.71593	E11.5 * Sox10 up vs E11.5 * Ctrl
10358599	Hmcn1	NM_001024720	0.000112263	1.7157	E11.5 * Sox10 up vs E11.5 * Ctrl
10494200	Cdc42se1	NM_172395	4.22126e-006	1.71555	E11.5 * Sox10 up vs E11.5 * Ctrl
10533633	Diablo	NM_023232	9.9446e-009	1.71394	E11.5 * Sox10 up vs E11.5 * Ctrl
10593245	Htr3b	NM_020274	7.22144e-005	1.7115	E11.5 * Sox10 up vs E11.5 * Ctrl
10434165	Arvcf	NM_033474	1.60182e-006	1.70807	E11.5 * Sox10 up vs E11.5 * Ctrl
10379936	Tbx2	NM_009324	6.54312e-005	1.70761	E11.5 * Sox10 up vs E11.5 * Ctrl
10528527	Fam126a	NM_053090	5.72527e-007	1.70353	E11.5 * Sox10 up vs E11.5 * Ctrl
10435769	Zbtb20	NM_019778	3.04663e-007	1.70321	E11.5 * Sox10 up vs E11.5 * Ctrl
10600688	Map3k7ip3	NM_025729	8.3936e-007	1.7022	E11.5 * Sox10 up vs E11.5 * Ctrl
10474141	Slc1a2	NM_001077514	1.47608e-005	1.70193	E11.5 * Sox10 up vs E11.5 * Ctrl
10358579	Hmcn1	NM_001024720	2.50085e-005	1.69913	E11.5 * Sox10 up vs E11.5 * Ctrl
10407792	Gpr137b-ps	NR_003568	0.00190528	1.6981	E11.5 * Sox10 up vs E11.5 * Ctrl
10404848	Jarid2	NM_021878	3.76336e-010	1.69728	E11.5 * Sox10 up vs E11.5 * Ctrl
10521467	Ccdc96	NM_025725	9.83961e-006	1.69569	E11.5 * Sox10 up vs E11.5 * Ctrl
10553897	Mtmr10	NM_172742	0.000109938	1.69242	E11.5 * Sox10 up vs E11.5 * Ctrl
10588037	Rbp1	NM_011254	5.12321e-008	1.6923	E11.5 * Sox10 up vs E11.5 * Ctrl
10408047	Zfp389	NR_026798	0.000804498	1.69177	E11.5 * Sox10 up vs E11.5 * Ctrl
10549842	Zfp667	NM_001024928	9.93653e-006	1.6917	E11.5 * Sox10 up vs E11.5 * Ctrl
10578493	Tlr3	NM_126166	0.000264385	1.6903	E11.5 * Sox10 up vs E11.5 * Ctrl
10400357	Baz1a	NM_013815	7.18452e-006	1.68953	E11.5 * Sox10 up vs E11.5 * Ctrl
10449644	Glo1	NM_025374	2.97334e-007	1.68913	E11.5 * Sox10 up vs E11.5 * Ctrl
10355225	ENSMUSG0000C	ENSMUST00000(	8.71022e-005	1.68868	E11.5 * Sox10 up vs E11.5 * Ctrl
10541260	Cecr2	NM_001128151	5.22628e-005	1.68852	E11.5 * Sox10 up vs E11.5 * Ctrl
10473244	Zfp804a	NM_175513	1.00486e-005	1.68799	E11.5 * Sox10 up vs E11.5 * Ctrl
10480329	Dnajc1	NM_007869	6.55909e-006	1.68791	E11.5 * Sox10 up vs E11.5 * Ctrl
10358490	Hmcn1	NM_001024720	1.32881e-006	1.68482	E11.5 * Sox10 up vs E11.5 * Ctrl
10468311	Sh3pxd2a	NM_008018	2.81996e-005	1.6848	E11.5 * Sox10 up vs E11.5 * Ctrl
10396476	Rhoj	NM_023275	1.31144e-005	1.68475	E11.5 * Sox10 up vs E11.5 * Ctrl
10588283	Rab6b	NM_173781	0.000508102	1.68405	E11.5 * Sox10 up vs E11.5 * Ctrl
10351259	Slc19a2	NM_054087	0.000589291	1.68266	E11.5 * Sox10 up vs E11.5 * Ctrl
10381250	Tubg1	NM_134024	4.39696e-005	1.68223	E11.5 * Sox10 up vs E11.5 * Ctrl
10358581	Hmcn1	NM_001024720	8.10734e-005	1.68213	E11.5 * Sox10 up vs E11.5 * Ctrl
10395414	Tmem195	NM_178767	0.00103351	1.68185	E11.5 * Sox10 up vs E11.5 * Ctrl
10359917	Hsd17b7	NM_010476	0.000361985	1.68172	E11.5 * Sox10 up vs E11.5 * Ctrl
10593671	Dmxl2	NM_172771	2.12012e-007	1.68109	E11.5 * Sox10 up vs E11.5 * Ctrl
10347970	Cab39	NM_133781	6.35359e-009	1.6795	E11.5 * Sox10 up vs E11.5 * Ctrl
10462005	Tmem2	NM_031997	9.02747e-008	1.67902	E11.5 * Sox10 up vs E11.5 * Ctrl
10543145	Thsd7a	ENSMUST00000	2.25195e-006	1.67847	E11.5 * Sox10 up vs E11.5 * Ctrl
10575777	4933407C03Rik	BC158118	4.23758e-006	1.67776	E11.5 * Sox10 up vs E11.5 * Ctrl
10527306	Lmtk2	NM_001081109	1.64986e-007	1.67634	E11.5 * Sox10 up vs E11.5 * Ctrl
10445879	Kcnh8	NM_001031811	0.00042495	1.67588	E11.5 * Sox10 up vs E11.5 * Ctrl
10363130	Gopc	NM_053187	0.000500355	1.67578	E11.5 * Sox10 up vs E11.5 * Ctrl

10406856	Gm807	ENSMUST00000	4.51795e-005	1.67426	E11.5 * Sox10 up vs E11.5 * Ctrl
10358609	Hmcn1	NM_001024720	4.82975e-005	1.67421	E11.5 * Sox10 up vs E11.5 * Ctrl
10493114	Nes	NM_016701	0.00099055	1.674	E11.5 * Sox10 up vs E11.5 * Ctrl
10354472	Gls	NM_001081081	0.000190209	1.67153	E11.5 * Sox10 up vs E11.5 * Ctrl
10518408	Plod1	NM_011122	4.73181e-005	1.67078	E11.5 * Sox10 up vs E11.5 * Ctrl
10350742	Rnasel	NM_011882	0.000978161	1.66992	E11.5 * Sox10 up vs E11.5 * Ctrl
10380341	Spag9	NM_027569	6.93098e-009	1.66938	E11.5 * Sox10 up vs E11.5 * Ctrl
10358652	Hmcn1	NM_001024720	8.59631e-006	1.66896	E11.5 * Sox10 up vs E11.5 * Ctrl
10449041	Metrn	NM_133719	5.60494e-005	1.66879	E11.5 * Sox10 up vs E11.5 * Ctrl
10605820	Zc4h2	NM_001003916	4.98723e-007	1.66715	E11.5 * Sox10 up vs E11.5 * Ctrl
10536805	Fam71f1	NM_207258	1.37233e-005	1.66668	E11.5 * Sox10 up vs E11.5 * Ctrl
10579925	Gab1	NM_021356	2.24318e-008	1.66509	E11.5 * Sox10 up vs E11.5 * Ctrl
10374106	Ykt6	NM_019661	0.000120077	1.66476	E11.5 * Sox10 up vs E11.5 * Ctrl
10415332	Rec8	NM_020002	3.29933e-007	1.66474	E11.5 * Sox10 up vs E11.5 * Ctrl
10483604	Slc25a12	NM_172436	2.97312e-006	1.66445	E11.5 * Sox10 up vs E11.5 * Ctrl
10527229	2810453I06Rik	NM_026050	2.0974e-006	1.66236	E11.5 * Sox10 up vs E11.5 * Ctrl
10358571	Hmcn1	NM_001024720	4.57432e-005	1.6603	E11.5 * Sox10 up vs E11.5 * Ctrl
10482059	Ggta1	NM_010283	3.67679e-005	1.65963	E11.5 * Sox10 up vs E11.5 * Ctrl
10475643	Fgf7	NM_008008	0.00135912	1.65899	E11.5 * Sox10 up vs E11.5 * Ctrl
10481056	Notch1	NM_008714	0.000164295	1.65839	E11.5 * Sox10 up vs E11.5 * Ctrl
10381934	Tanc2	NM_181071	7.96911e-005	1.65829	E11.5 * Sox10 up vs E11.5 * Ctrl
10447502	Adcyap1	NM_009625	0.000190818	1.65827	E11.5 * Sox10 up vs E11.5 * Ctrl
10566767	St5	NM_001001326	6.17664e-006	1.65814	E11.5 * Sox10 up vs E11.5 * Ctrl
10451167	Tmem63b	NM_198167	9.45873e-006	1.65776	E11.5 * Sox10 up vs E11.5 * Ctrl
10481272	1700007K13Rik	BC099566	4.2932e-005	1.65716	E11.5 * Sox10 up vs E11.5 * Ctrl
10442887	Fbxl16	BC132383	0.000137762	1.65601	E11.5 * Sox10 up vs E11.5 * Ctrl
10394611	Nbas	ENSMUST00000	3.19461e-007	1.65576	E11.5 * Sox10 up vs E11.5 * Ctrl
10358565	Hmcn1	NM_001024720	0.000226286	1.65524	E11.5 * Sox10 up vs E11.5 * Ctrl
10518147	Pdpn	NM_010329	0.000301135	1.65466	E11.5 * Sox10 up vs E11.5 * Ctrl
10469322	Vim	NM_011701	3.81508e-010	1.65348	E11.5 * Sox10 up vs E11.5 * Ctrl
10452295	Tubb4	NM_009451	0.00018155	1.65331	E11.5 * Sox10 up vs E11.5 * Ctrl
10385236	Akr1b3	NM_009658	3.03058e-008	1.65231	E11.5 * Sox10 up vs E11.5 * Ctrl
10352503	Bpnt1	NM_011794	3.02663e-005	1.6516	E11.5 * Sox10 up vs E11.5 * Ctrl
10451838	Slc5a7	NM_022025	0.000219725	1.64864	E11.5 * Sox10 up vs E11.5 * Ctrl
10493519	Shc1	NM_001113331	0.00119609	1.64859	E11.5 * Sox10 up vs E11.5 * Ctrl
10528143	Ppp1r14b	NM_008889	3.42715e-009	1.64744	E11.5 * Sox10 up vs E11.5 * Ctrl
10375263	ENSMUSG00000	ENSMUST00000	0.00167386	1.64711	E11.5 * Sox10 up vs E11.5 * Ctrl
10362829	Ostm1	NM_172416	4.25289e-006	1.6464	E11.5 * Sox10 up vs E11.5 * Ctrl
10566846	Dennd5a	NM_021494	3.14877e-009	1.64432	E11.5 * Sox10 up vs E11.5 * Ctrl
10384138	Tmed4	NM_134020	0.000268167	1.64401	E11.5 * Sox10 up vs E11.5 * Ctrl
10575775	4933407C03Rik	BC158118	2.35092e-006	1.6438	E11.5 * Sox10 up vs E11.5 * Ctrl
10468762	4930506M07Rik	NM_001114312	3.26215e-005	1.64364	E11.5 * Sox10 up vs E11.5 * Ctrl
10375432	C030019I05Rik	NM_177075	0.00224261	1.64364	E11.5 * Sox10 up vs E11.5 * Ctrl
10593668	Dmxl2	NM_172771	9.00354e-005	1.6403	E11.5 * Sox10 up vs E11.5 * Ctrl
10550316	Tmem160	NM_026938	5.05442e-007	1.63929	E11.5 * Sox10 up vs E11.5 * Ctrl
10377018	Myh3	NM_001099635	7.67431e-008	1.63851	E11.5 * Sox10 up vs E11.5 * Ctrl
10426016	Gtse1	NM_013882	1.98954e-007	1.63763	E11.5 * Sox10 up vs E11.5 * Ctrl
10362394	Hddc2	NM_027168	4.53546e-005	1.63656	E11.5 * Sox10 up vs E11.5 * Ctrl
10569370	Th	NM_009377	3.07844e-006	1.63601	E11.5 * Sox10 up vs E11.5 * Ctrl
10544186	Mkrn1	NM_018810	9.64184e-008	1.63592	E11.5 * Sox10 up vs E11.5 * Ctrl
10369835	Phyhipl	NM_178621	2.34406e-005	1.63534	E11.5 * Sox10 up vs E11.5 * Ctrl
10358627	Hmcn1	NM_001024720	4.87656e-005	1.63382	E11.5 * Sox10 up vs E11.5 * Ctrl
10446777	Ehd3	NM_020578	3.28272e-005	1.63213	E11.5 * Sox10 up vs E11.5 * Ctrl
10605797	Arhgef9	NM_001033329	1.03105e-007	1.63198	E11.5 * Sox10 up vs E11.5 * Ctrl
10488709	8430427H17Rik	NM_001134300	3.94408e-006	1.63173	E11.5 * Sox10 up vs E11.5 * Ctrl
10519770	Pclo	NM_011995	3.17027e-005	1.63018	E11.5 * Sox10 up vs E11.5 * Ctrl
10405343	Tspan17	NM_028841	8.90931e-006	1.6299	E11.5 * Sox10 up vs E11.5 * Ctrl
10395457	Etv1	NM_007960	1.67853e-005	1.62954	E11.5 * Sox10 up vs E11.5 * Ctrl
10459552	Spire1	NM_194355	2.63159e-008	1.62953	E11.5 * Sox10 up vs E11.5 * Ctrl
10476301	Smox	NM_145533	0.00011655	1.62731	E11.5 * Sox10 up vs E11.5 * Ctrl
10358754	EG639787	XR_002131	2.92571e-005	1.62721	E11.5 * Sox10 up vs E11.5 * Ctrl
10462035	Ldhb	NM_008492	9.74567e-008	1.62701	E11.5 * Sox10 up vs E11.5 * Ctrl
10571567	Sorbs2	NM_172752	1.02981e-007	1.62651	E11.5 * Sox10 up vs E11.5 * Ctrl
10429295	Kcnk9	NM_001033876	5.52423e-006	1.62639	E11.5 * Sox10 up vs E11.5 * Ctrl
10503643	Ndufaf4	NM_026742	6.98865e-008	1.62588	E11.5 * Sox10 up vs E11.5 * Ctrl
10533858	Eif2b1	NM_145371	1.01153e-008	1.62583	E11.5 * Sox10 up vs E11.5 * Ctrl

10466938	5033414D02Rik	NM_026362	0.000591275	1.62541	E11.5 * Sox10 up vs E11.5 * Ctrl
10468668	Afap1l2	NM_146102	0.000501973	1.62457	E11.5 * Sox10 up vs E11.5 * Ctrl
10561369	BC089491	NM_175033	8.46827e-006	1.62441	E11.5 * Sox10 up vs E11.5 * Ctrl
10568109	Asphd1	NM_001039645	4.11441e-005	1.62436	E11.5 * Sox10 up vs E11.5 * Ctrl
10379795	Ap1gbp1	NM_001115009	6.95315e-007	1.62407	E11.5 * Sox10 up vs E11.5 * Ctrl
10358650	Hmcn1	NM_001024720	2.14772e-005	1.62302	E11.5 * Sox10 up vs E11.5 * Ctrl
10504692	Tmod1	NM_021883	4.29705e-005	1.62291	E11.5 * Sox10 up vs E11.5 * Ctrl
10423471	Ctnnd2	NM_008729	6.83188e-006	1.62289	E11.5 * Sox10 up vs E11.5 * Ctrl
10398996	Crip2	NM_024223	0.000390565	1.62066	E11.5 * Sox10 up vs E11.5 * Ctrl
10378549	Rtn4rl1	NM_177708	1.03654e-005	1.62011	E11.5 * Sox10 up vs E11.5 * Ctrl
10560919	Atp1a3	NM_144921	0.00129207	1.62	E11.5 * Sox10 up vs E11.5 * Ctrl
10421648	Slc25a30	NM_026232	7.09622e-007	1.6195	E11.5 * Sox10 up vs E11.5 * Ctrl
10358537	Hmcn1	NM_001024720	1.52073e-006	1.6193	E11.5 * Sox10 up vs E11.5 * Ctrl
10394627	Nbas	BC057020	7.767e-009	1.6189	E11.5 * Sox10 up vs E11.5 * Ctrl
10395394	4930579E17Rik	NM_178629	0.000122641	1.61798	E11.5 * Sox10 up vs E11.5 * Ctrl
10538519	Gsbs	NM_011153	0.000152525	1.61795	E11.5 * Sox10 up vs E11.5 * Ctrl
10605465	Prkx	NM_016979	0.000238818	1.61734	E11.5 * Sox10 up vs E11.5 * Ctrl
10506150	Foxd3	NM_010425	0.00104044	1.6171	E11.5 * Sox10 up vs E11.5 * Ctrl
10366951	Ndufa4l2	NM_001098789	0.000791832	1.61592	E11.5 * Sox10 up vs E11.5 * Ctrl
10479230	Phactr3	NM_028806	9.38676e-005	1.61576	E11.5 * Sox10 up vs E11.5 * Ctrl
10404702	Gcnt2	NM_023887	1.80852e-006	1.61476	E11.5 * Sox10 up vs E11.5 * Ctrl
10367641	Mthfd1l	NM_172308	6.65558e-009	1.61424	E11.5 * Sox10 up vs E11.5 * Ctrl
10457942	Syt4	NM_009308	0.000143656	1.61393	E11.5 * Sox10 up vs E11.5 * Ctrl
10406934	Etv1	NM_007960	2.32319e-006	1.61361	E11.5 * Sox10 up vs E11.5 * Ctrl
10345172	ENSMUSG0000C	ENSMUST00000	0.00133473	1.61139	E11.5 * Sox10 up vs E11.5 * Ctrl
10597323	Arpp21	NM_033264	1.54558e-007	1.61022	E11.5 * Sox10 up vs E11.5 * Ctrl
10392449	Wipi1	NM_145940	2.76617e-005	1.60739	E11.5 * Sox10 up vs E11.5 * Ctrl
10439955	Fam55c	NM_001134457	7.86541e-005	1.60722	E11.5 * Sox10 up vs E11.5 * Ctrl
10358569	Hmcn1	NM_001024720	7.8178e-005	1.60673	E11.5 * Sox10 up vs E11.5 * Ctrl
10451054	Enpp4	NM_199016	3.67159e-005	1.60523	E11.5 * Sox10 up vs E11.5 * Ctrl
10358583	Hmcn1	NM_001024720	5.7558e-005	1.60418	E11.5 * Sox10 up vs E11.5 * Ctrl
10477994	Ctnnbl1	NM_025680	4.08303e-007	1.6035	E11.5 * Sox10 up vs E11.5 * Ctrl
10460057	Tshz1	NM_001081300	5.80111e-006	1.60304	E11.5 * Sox10 up vs E11.5 * Ctrl
10468217	Actr1a	NM_016860	1.76622e-005	1.60255	E11.5 * Sox10 up vs E11.5 * Ctrl
10568735	Ebf3	NM_001113415	3.44545e-006	1.60083	E11.5 * Sox10 up vs E11.5 * Ctrl
10468200	Cuedc2	NM_024192	2.55543e-007	1.60042	E11.5 * Sox10 up vs E11.5 * Ctrl
10495625	Dpyd	NM_170778	7.212e-005	1.59892	E11.5 * Sox10 up vs E11.5 * Ctrl
10402283	Itpk1	NM_172584	0.0033891	1.59824	E11.5 * Sox10 up vs E11.5 * Ctrl
10512065	OTTMUSG00000	XR_033416	0.000249272	1.59738	E11.5 * Sox10 up vs E11.5 * Ctrl
10354741	Rftn2	NM_028713	2.72577e-007	1.59676	E11.5 * Sox10 up vs E11.5 * Ctrl
10373964	Thoc5	NM_172438	6.18786e-006	1.59663	E11.5 * Sox10 up vs E11.5 * Ctrl
10391513	Dusp3	NM_028207	0.000607446	1.59639	E11.5 * Sox10 up vs E11.5 * Ctrl
10559146	Brsk2	NM_029426	9.05657e-006	1.59619	E11.5 * Sox10 up vs E11.5 * Ctrl
10358654	Hmcn1	NM_001024720	1.14162e-005	1.59505	E11.5 * Sox10 up vs E11.5 * Ctrl
10441361	Tiam2	NM_001122998	9.49894e-006	1.5945	E11.5 * Sox10 up vs E11.5 * Ctrl
10590860	9030420J04Rik	BC137891	2.28763e-005	1.59444	E11.5 * Sox10 up vs E11.5 * Ctrl
10470696	Med27	NM_026896	0.000208927	1.59432	E11.5 * Sox10 up vs E11.5 * Ctrl
10490794	Pkia	NM_008862	7.60767e-005	1.5943	E11.5 * Sox10 up vs E11.5 * Ctrl
10358553	Hmcn1	NM_001024720	0.00381187	1.59418	E11.5 * Sox10 up vs E11.5 * Ctrl
10358567	Hmcn1	NM_001024720	3.83825e-005	1.59393	E11.5 * Sox10 up vs E11.5 * Ctrl
10375322	4933415A04Rik	ENSMUST00000	9.65475e-006	1.59318	E11.5 * Sox10 up vs E11.5 * Ctrl
10390635	1810046J19Rik	NM_025559	1.55642e-005	1.59303	E11.5 * Sox10 up vs E11.5 * Ctrl
10361642	Lrp11	NM_172784	0.000485563	1.59249	E11.5 * Sox10 up vs E11.5 * Ctrl
10435789	Zbtb20	ENSMUST00000	0.000109983	1.5909	E11.5 * Sox10 up vs E11.5 * Ctrl
10528648	Abcf2	NM_013853	7.10063e-007	1.59069	E11.5 * Sox10 up vs E11.5 * Ctrl
10381082	Rara	NM_009024	0.000130228	1.58972	E11.5 * Sox10 up vs E11.5 * Ctrl
10564220	Gkap1	NM_019832	6.50342e-005	1.58925	E11.5 * Sox10 up vs E11.5 * Ctrl
10472162	Gpd2	NM_010274	6.29496e-006	1.58923	E11.5 * Sox10 up vs E11.5 * Ctrl
10593927	Scamp5	NM_020270	7.22893e-005	1.58906	E11.5 * Sox10 up vs E11.5 * Ctrl
10386844	Zswim7	BC109343	0.000459268	1.58861	E11.5 * Sox10 up vs E11.5 * Ctrl
10538753	Atoh1	BC010820	0.000323418	1.58822	E11.5 * Sox10 up vs E11.5 * Ctrl
10487748	4930402H24Rik	BC052447	1.41838e-007	1.58809	E11.5 * Sox10 up vs E11.5 * Ctrl
10494060	Mrpl9	NM_030116	6.39932e-007	1.58742	E11.5 * Sox10 up vs E11.5 * Ctrl
10567022	Btbd10	NM_133700	5.34608e-005	1.58675	E11.5 * Sox10 up vs E11.5 * Ctrl
10417713	Rarb	NM_011243	4.83196e-006	1.58674	E11.5 * Sox10 up vs E11.5 * Ctrl
10388884	Nlk	NM_008702	8.75336e-008	1.5861	E11.5 * Sox10 up vs E11.5 * Ctrl

10575497	Mtss1l	NM_198625	0.000201358	1.58535	E11.5 * Sox10 up vs E11.5 * Ctrl
10349637	2700049P18Rik	NM_175382	9.60112e-005	1.58529	E11.5 * Sox10 up vs E11.5 * Ctrl
10370766	Gamt	NM_010255	0.000123259	1.58509	E11.5 * Sox10 up vs E11.5 * Ctrl
10600836	Msn	NM_010833	1.54734e-008	1.58505	E11.5 * Sox10 up vs E11.5 * Ctrl
10589913	Dync1li1	NM_146229	1.85221e-006	1.58469	E11.5 * Sox10 up vs E11.5 * Ctrl
10530100	Arap2	NM_178407	0.000109334	1.58404	E11.5 * Sox10 up vs E11.5 * Ctrl
10400141	Zbed4	NM_181412	0.000252779	1.58378	E11.5 * Sox10 up vs E11.5 * Ctrl
10508907	Lin28	NM_145833	0.00181927	1.5833	E11.5 * Sox10 up vs E11.5 * Ctrl
10411306	Polk	NM_012048	2.53518e-008	1.58271	E11.5 * Sox10 up vs E11.5 * Ctrl
10501358	Sars	NM_011319	1.24717e-007	1.5823	E11.5 * Sox10 up vs E11.5 * Ctrl
10459827	Rnf165	ENSMUST00000(	3.85549e-007	1.58201	E11.5 * Sox10 up vs E11.5 * Ctrl
10455238	Ndfip1	NM_022996	1.15247e-008	1.58188	E11.5 * Sox10 up vs E11.5 * Ctrl
10364502	Palm	NM_023128	8.51374e-005	1.58177	E11.5 * Sox10 up vs E11.5 * Ctrl
10564203		---	0.00833481	1.58118	E11.5 * Sox10 up vs E11.5 * Ctrl
10587085	BC031353	NM_001113283	1.77086e-006	1.58003	E11.5 * Sox10 up vs E11.5 * Ctrl
10416044	Ccdc25	NM_145944	0.000547619	1.57919	E11.5 * Sox10 up vs E11.5 * Ctrl
10385375	Thg1l	NM_001080969	0.000127291	1.57825	E11.5 * Sox10 up vs E11.5 * Ctrl
10424404	Pvt1	NR_003368	0.000785782	1.57738	E11.5 * Sox10 up vs E11.5 * Ctrl
10461022	Ppp1r14b	NM_008889	1.36935e-009	1.57711	E11.5 * Sox10 up vs E11.5 * Ctrl
10358631	Hmcn1	NM_001024720	0.00123511	1.57578	E11.5 * Sox10 up vs E11.5 * Ctrl
10545379	Usp39	NM_138592	1.20305e-007	1.57531	E11.5 * Sox10 up vs E11.5 * Ctrl
10459329	2700046A07Rik	ENSMUST00000(	0.000273846	1.5752	E11.5 * Sox10 up vs E11.5 * Ctrl
10460645	Map3k11	NM_022012	0.00305476	1.57386	E11.5 * Sox10 up vs E11.5 * Ctrl
10431051	Scube1	NM_022723	2.47814e-005	1.57338	E11.5 * Sox10 up vs E11.5 * Ctrl
10357345	E030049G20Rik	NM_172484	0.00033135	1.57315	E11.5 * Sox10 up vs E11.5 * Ctrl
10396740	Gphn	NM_172952	5.82558e-006	1.57265	E11.5 * Sox10 up vs E11.5 * Ctrl
10521709	Lap3	NM_024434	1.04144e-006	1.5725	E11.5 * Sox10 up vs E11.5 * Ctrl
10420787	Mtmr9	NM_177594	6.60877e-006	1.57219	E11.5 * Sox10 up vs E11.5 * Ctrl
10573172	Clgn	NM_009904	5.42571e-005	1.57146	E11.5 * Sox10 up vs E11.5 * Ctrl
10454077	Taf4b	NM_001100449	4.27402e-006	1.57095	E11.5 * Sox10 up vs E11.5 * Ctrl
10569129	Sct	NM_011328	7.88735e-005	1.57079	E11.5 * Sox10 up vs E11.5 * Ctrl
10524955	Tesc	NM_021344	6.22818e-005	1.56921	E11.5 * Sox10 up vs E11.5 * Ctrl
10550400	Pnmal2	NM_001099636	3.42529e-005	1.5685	E11.5 * Sox10 up vs E11.5 * Ctrl
10581289	Atp6v0d1	NM_013477	9.09992e-005	1.56761	E11.5 * Sox10 up vs E11.5 * Ctrl
10354229	2610017I09Rik	BC058417	0.000782803	1.56737	E11.5 * Sox10 up vs E11.5 * Ctrl
10420385	N6amt2	NM_026526	9.0073e-006	1.56675	E11.5 * Sox10 up vs E11.5 * Ctrl
10511725	Cyb5r4	NM_024195	2.31554e-006	1.56584	E11.5 * Sox10 up vs E11.5 * Ctrl
10540248	Mitf	NM_001113198	0.000286803	1.56571	E11.5 * Sox10 up vs E11.5 * Ctrl
10420846	Fzd3	NM_021458	5.03029e-007	1.56489	E11.5 * Sox10 up vs E11.5 * Ctrl
10590663	Gria4	NM_019691	4.50304e-006	1.56462	E11.5 * Sox10 up vs E11.5 * Ctrl
10381574	Rundc3a	NM_016759	1.7812e-005	1.56442	E11.5 * Sox10 up vs E11.5 * Ctrl
10497996	Ikzf5	NM_175115	6.04221e-006	1.56437	E11.5 * Sox10 up vs E11.5 * Ctrl
10561376	Dll3	NM_007866	1.45138e-005	1.56397	E11.5 * Sox10 up vs E11.5 * Ctrl
10389719	Scpep1	NM_029023	4.43166e-007	1.56328	E11.5 * Sox10 up vs E11.5 * Ctrl
10478897	Ptpn1	NM_011201	3.30499e-007	1.56259	E11.5 * Sox10 up vs E11.5 * Ctrl
10538338	Creb5	NM_172728	1.71798e-007	1.56254	E11.5 * Sox10 up vs E11.5 * Ctrl
10345715	Map4k4	NM_008696	3.35479e-007	1.56254	E11.5 * Sox10 up vs E11.5 * Ctrl
10358656	Hmcn1	NM_001024720	3.91573e-006	1.56233	E11.5 * Sox10 up vs E11.5 * Ctrl
10375175	Slit3	NM_011412	2.88613e-006	1.56226	E11.5 * Sox10 up vs E11.5 * Ctrl
10552276	Ube2h	NM_009459	0.000109312	1.56206	E11.5 * Sox10 up vs E11.5 * Ctrl
10456522	Tcf4	NM_013685	5.68772e-009	1.56132	E11.5 * Sox10 up vs E11.5 * Ctrl
10594001	Arid3b	NM_019689	1.15923e-006	1.56111	E11.5 * Sox10 up vs E11.5 * Ctrl
10425158	Pdxp	NM_020271	0.00012185	1.56086	E11.5 * Sox10 up vs E11.5 * Ctrl
10546476	Magi1	NM_001029850	4.864e-007	1.56076	E11.5 * Sox10 up vs E11.5 * Ctrl
10416541	Enox1	NM_172813	2.96843e-007	1.55888	E11.5 * Sox10 up vs E11.5 * Ctrl
10587107	Myo5a	NM_010864	5.14531e-009	1.55875	E11.5 * Sox10 up vs E11.5 * Ctrl
10483626	Dlx2	NM_010054	1.07192e-005	1.55844	E11.5 * Sox10 up vs E11.5 * Ctrl
10358523	Hmcn1	NM_001024720	5.87803e-005	1.55785	E11.5 * Sox10 up vs E11.5 * Ctrl
10575249	Txnl4b	NM_175646	7.18094e-006	1.5575	E11.5 * Sox10 up vs E11.5 * Ctrl
10358585	Hmcn1	NM_001024720	0.00010673	1.5571	E11.5 * Sox10 up vs E11.5 * Ctrl
10585601	Snupn	NM_178374	1.48429e-005	1.55604	E11.5 * Sox10 up vs E11.5 * Ctrl
10590909	Endod1	NM_028013	3.71912e-006	1.55581	E11.5 * Sox10 up vs E11.5 * Ctrl
10520650	Cad	NM_023525	3.55325e-005	1.5556	E11.5 * Sox10 up vs E11.5 * Ctrl
10578027	Mak16	NM_026453	7.76114e-005	1.55523	E11.5 * Sox10 up vs E11.5 * Ctrl
10348432	Agap1	NM_178119	8.54935e-005	1.55502	E11.5 * Sox10 up vs E11.5 * Ctrl
10548043	Kcna5	NM_145983	0.00314925	1.55399	E11.5 * Sox10 up vs E11.5 * Ctrl

10434934	Bdh1	NM_175177	7.67206e-005	1.55374	E11.5 * Sox10 up vs E11.5 * Ctrl
10394770	Odc1	NM_013614	9.76711e-008	1.55361	E11.5 * Sox10 up vs E11.5 * Ctrl
10358573	Hmcn1	NM_001024720	0.000172662	1.55254	E11.5 * Sox10 up vs E11.5 * Ctrl
10528484	Srpk2	NM_009274	3.48635e-007	1.5525	E11.5 * Sox10 up vs E11.5 * Ctrl
10526977	Eif3b	NM_133916	3.24927e-005	1.55197	E11.5 * Sox10 up vs E11.5 * Ctrl
10600390	Gdi1	NM_010273	3.57664e-006	1.55159	E11.5 * Sox10 up vs E11.5 * Ctrl
10565852	Rnf169	AK173319	1.58424e-006	1.55156	E11.5 * Sox10 up vs E11.5 * Ctrl
10446763	Lbh	NM_029999	6.27818e-008	1.55115	E11.5 * Sox10 up vs E11.5 * Ctrl
10604057	06-Sep	NM_019942	3.78572e-005	1.55108	E11.5 * Sox10 up vs E11.5 * Ctrl
10457118	Rttn	NM_175542	3.50497e-005	1.55098	E11.5 * Sox10 up vs E11.5 * Ctrl
10542896	Bicd1	NM_009753	3.52648e-005	1.55067	E11.5 * Sox10 up vs E11.5 * Ctrl
10456346	Sec11c	NM_025468	1.77681e-005	1.55065	E11.5 * Sox10 up vs E11.5 * Ctrl
10370072	Prmt2	NM_133182	0.000228606	1.55064	E11.5 * Sox10 up vs E11.5 * Ctrl
10600210	Slc6a8	NM_133987	3.63869e-005	1.55033	E11.5 * Sox10 up vs E11.5 * Ctrl
10531866	Mapk10	NM_009158	4.19526e-005	1.54927	E11.5 * Sox10 up vs E11.5 * Ctrl
10478364	Tox2	NM_001098799	1.02444e-005	1.54917	E11.5 * Sox10 up vs E11.5 * Ctrl
10517373	Rcan3	NM_022980	1.2338e-008	1.5479	E11.5 * Sox10 up vs E11.5 * Ctrl
10464504	Lrp5	NM_008513	0.00192102	1.5478	E11.5 * Sox10 up vs E11.5 * Ctrl
10451580	Bysl	NM_016859	7.97026e-006	1.54748	E11.5 * Sox10 up vs E11.5 * Ctrl
10350113	Arl8a	NM_026823	7.66408e-005	1.54733	E11.5 * Sox10 up vs E11.5 * Ctrl
10429160	St3gal1	NM_009177	8.40934e-005	1.54702	E11.5 * Sox10 up vs E11.5 * Ctrl
10575376	Ftsjd1	NM_146215	1.99372e-006	1.54666	E11.5 * Sox10 up vs E11.5 * Ctrl
10424781	Grina	NM_023168	3.38213e-005	1.54627	E11.5 * Sox10 up vs E11.5 * Ctrl
10607004	Psmd10	NM_016883	1.90269e-005	1.54624	E11.5 * Sox10 up vs E11.5 * Ctrl
10518428	Clcn6	NM_011929	0.000537091	1.54498	E11.5 * Sox10 up vs E11.5 * Ctrl
10558971	Ap2a2	NM_007459	5.06592e-006	1.54491	E11.5 * Sox10 up vs E11.5 * Ctrl
10565018	Iqgap1	NM_016721	5.07314e-007	1.54467	E11.5 * Sox10 up vs E11.5 * Ctrl
10352119	Pppde1	NM_024282	7.17039e-007	1.54442	E11.5 * Sox10 up vs E11.5 * Ctrl
10525439	P2rx4	NM_011026	0.000782952	1.54417	E11.5 * Sox10 up vs E11.5 * Ctrl
10490972	Trim55	NM_001081281	0.00435209	1.54368	E11.5 * Sox10 up vs E11.5 * Ctrl
10558481	Dpysl4	NM_011993	6.75547e-005	1.54349	E11.5 * Sox10 up vs E11.5 * Ctrl
10513869	Megf9	NM_172694	1.94246e-008	1.54315	E11.5 * Sox10 up vs E11.5 * Ctrl
10507273	Pik3r3	NM_181585	1.07665e-008	1.54311	E11.5 * Sox10 up vs E11.5 * Ctrl
10566516	Rrp8	NM_025897	3.67748e-005	1.54059	E11.5 * Sox10 up vs E11.5 * Ctrl
10372891	Srgap1	NM_001081037	3.17299e-006	1.5403	E11.5 * Sox10 up vs E11.5 * Ctrl
10458767	Trim36	NM_178872	0.000104976	1.53931	E11.5 * Sox10 up vs E11.5 * Ctrl
10524588	Ankrd13a	NM_026718	2.46984e-005	1.53858	E11.5 * Sox10 up vs E11.5 * Ctrl
10524909	Nos1	NM_008712	1.67741e-005	1.5385	E11.5 * Sox10 up vs E11.5 * Ctrl
10408450	Sox4	NM_009238	0.000329046	1.53776	E11.5 * Sox10 up vs E11.5 * Ctrl
10466712	Mamdc2	NM_174857	2.54068e-005	1.53715	E11.5 * Sox10 up vs E11.5 * Ctrl
10604633	Cxx1b	NM_001018063	4.01447e-008	1.53708	E11.5 * Sox10 up vs E11.5 * Ctrl
10392369	Cacng4	NM_019431	1.43558e-005	1.53614	E11.5 * Sox10 up vs E11.5 * Ctrl
10564417	Aldh1a3	NM_053080	0.000213404	1.53601	E11.5 * Sox10 up vs E11.5 * Ctrl
10355996	Slc25a5	NM_007451	5.7316e-007	1.53561	E11.5 * Sox10 up vs E11.5 * Ctrl
10351825	Tagln2	NM_178598	5.72149e-005	1.53557	E11.5 * Sox10 up vs E11.5 * Ctrl
10528227	Gnai1	NM_010305	8.00236e-006	1.53546	E11.5 * Sox10 up vs E11.5 * Ctrl
10523595	Ptpn13	NM_011204	3.72036e-008	1.53531	E11.5 * Sox10 up vs E11.5 * Ctrl
10594631	Aph1b	NM_177583	4.25264e-006	1.53463	E11.5 * Sox10 up vs E11.5 * Ctrl
10596583	Dock3	NM_153413	1.97606e-005	1.53375	E11.5 * Sox10 up vs E11.5 * Ctrl
10499643	Chrnb2	NM_009602	6.15115e-005	1.53368	E11.5 * Sox10 up vs E11.5 * Ctrl
10563390	Cyth2	NM_011181	0.000256508	1.5331	E11.5 * Sox10 up vs E11.5 * Ctrl
10554960	Fam181b	NM_021427	7.96079e-005	1.53287	E11.5 * Sox10 up vs E11.5 * Ctrl
10358539	Hmcn1	NM_001024720	0.00217078	1.5327	E11.5 * Sox10 up vs E11.5 * Ctrl
10545217	LOC100046973	ENSMUST00000	0.00365056	1.53252	E11.5 * Sox10 up vs E11.5 * Ctrl
10480492	Cacna1b	NM_001042528	2.18941e-007	1.53246	E11.5 * Sox10 up vs E11.5 * Ctrl
10472289	Tank	NM_011529	5.33413e-005	1.53213	E11.5 * Sox10 up vs E11.5 * Ctrl
10409345	Cltb	NM_028870	0.000140603	1.53152	E11.5 * Sox10 up vs E11.5 * Ctrl
10504008	Chmp5	NM_029814	6.88368e-007	1.53004	E11.5 * Sox10 up vs E11.5 * Ctrl
10536483	Tes	NM_207176	4.36872e-011	1.52964	E11.5 * Sox10 up vs E11.5 * Ctrl
10550915	Cadm4	NM_153112	0.000915112	1.52936	E11.5 * Sox10 up vs E11.5 * Ctrl
10570000	Gpi1	NM_008155	0.000667018	1.52922	E11.5 * Sox10 up vs E11.5 * Ctrl
10358623	Hmcn1	NM_001024720	0.000157077	1.5288	E11.5 * Sox10 up vs E11.5 * Ctrl
10581547	Nob1	NM_026277	1.8626e-005	1.52864	E11.5 * Sox10 up vs E11.5 * Ctrl
10495659	Cnn3	NM_028044	1.73016e-008	1.52746	E11.5 * Sox10 up vs E11.5 * Ctrl
10456501	Rnmt	NM_026440	4.96904e-006	1.52715	E11.5 * Sox10 up vs E11.5 * Ctrl
10358611	Hmcn1	NM_001024720	0.000133306	1.52646	E11.5 * Sox10 up vs E11.5 * Ctrl

10400023	Tspan13	NM_025359	4.09468e-006	1.52625	E11.5 * Sox10 up vs E11.5 * Ctrl
10358664	Hmcn1	NM_001024720	1.08619e-005	1.5259	E11.5 * Sox10 up vs E11.5 * Ctrl
10491962	Foxo1	NM_019739	1.42772e-006	1.52586	E11.5 * Sox10 up vs E11.5 * Ctrl
10579872	Tpd52	NM_001025263	0.000122074	1.52583	E11.5 * Sox10 up vs E11.5 * Ctrl
10394286	2410017P09Rik	BC067010	0.000900058	1.52568	E11.5 * Sox10 up vs E11.5 * Ctrl
10434758	St6gal1	NM_145933	8.02161e-007	1.52448	E11.5 * Sox10 up vs E11.5 * Ctrl
10360479	Cep170	NM_001099637	4.50176e-007	1.52309	E11.5 * Sox10 up vs E11.5 * Ctrl
10358660	Hmcn1	NM_001024720	0.000918321	1.52246	E11.5 * Sox10 up vs E11.5 * Ctrl
10555118	Pak1	NM_011035	2.22316e-006	1.5224	E11.5 * Sox10 up vs E11.5 * Ctrl
10570437	Fbxo25	NM_025785	1.98862e-005	1.52198	E11.5 * Sox10 up vs E11.5 * Ctrl
10419578	Ndrg2	NM_013864	5.26437e-005	1.5219	E11.5 * Sox10 up vs E11.5 * Ctrl
10384795	Cyb5d1	NM_001045525	0.00564332	1.5208	E11.5 * Sox10 up vs E11.5 * Ctrl
10467425	Sorbs1	NM_178362	1.63805e-006	1.52048	E11.5 * Sox10 up vs E11.5 * Ctrl
10470834	Spna2	NM_001076554	2.30561e-006	1.52044	E11.5 * Sox10 up vs E11.5 * Ctrl
10506274	Dnajc6	NM_198412	2.42835e-006	1.51994	E11.5 * Sox10 up vs E11.5 * Ctrl
10511881	Manea	NM_172865	1.62361e-005	1.51991	E11.5 * Sox10 up vs E11.5 * Ctrl
10452613	Arhgap28	NM_172964	4.13217e-007	1.51879	E11.5 * Sox10 up vs E11.5 * Ctrl
10436487	Vgll3	AK007165	0.000866473	1.51837	E11.5 * Sox10 up vs E11.5 * Ctrl
10491083	Aadacl1	NM_178772	9.87442e-005	1.51822	E11.5 * Sox10 up vs E11.5 * Ctrl
10357133		---	1.90841e-008	1.51768	E11.5 * Sox10 up vs E11.5 * Ctrl
10486396	Ehd4	NM_133838	0.00477573	1.51743	E11.5 * Sox10 up vs E11.5 * Ctrl
10573115	Rnf150	NM_177378	1.34712e-005	1.51696	E11.5 * Sox10 up vs E11.5 * Ctrl
10384985	Rhbdf1	NM_010117	6.64225e-005	1.51668	E11.5 * Sox10 up vs E11.5 * Ctrl
10519951	Pion	NM_175437	0.000299135	1.51625	E11.5 * Sox10 up vs E11.5 * Ctrl
10441497	Tulp4	NM_054040	0.000921578	1.51607	E11.5 * Sox10 up vs E11.5 * Ctrl
10362941	Prep	NM_011156	4.44375e-006	1.51598	E11.5 * Sox10 up vs E11.5 * Ctrl
10561343	Josd1	NM_028792	0.000816768	1.51549	E11.5 * Sox10 up vs E11.5 * Ctrl
10426798	Smarcd1	NM_031842	7.0103e-005	1.51512	E11.5 * Sox10 up vs E11.5 * Ctrl
10570180	EG434280	ENSMUST00000(	2.67327e-005	1.51482	E11.5 * Sox10 up vs E11.5 * Ctrl
10490551	Nkain4	NM_021426	3.37228e-005	1.51366	E11.5 * Sox10 up vs E11.5 * Ctrl
10394749	Nol10	NM_001008421	1.16108e-005	1.51348	E11.5 * Sox10 up vs E11.5 * Ctrl
10430020	Vps28	NM_025842	0.00012085	1.5134	E11.5 * Sox10 up vs E11.5 * Ctrl
10402585	Wars	NM_011710	5.82571e-006	1.51293	E11.5 * Sox10 up vs E11.5 * Ctrl
10554094	Igf1r	NM_010513	0.000108914	1.51287	E11.5 * Sox10 up vs E11.5 * Ctrl
10566457	Apbb1	NM_009685	5.20115e-005	1.51204	E11.5 * Sox10 up vs E11.5 * Ctrl
10464586	Gstp2	NM_181796	1.22439e-005	1.51186	E11.5 * Sox10 up vs E11.5 * Ctrl
10540880	Syn2	NM_001111015	2.06812e-005	1.51184	E11.5 * Sox10 up vs E11.5 * Ctrl
10352554		---	0.00032385	1.51143	E11.5 * Sox10 up vs E11.5 * Ctrl
10355141	Klf7	NM_033563	2.39209e-007	1.5109	E11.5 * Sox10 up vs E11.5 * Ctrl
10530201	Ugdh	NM_009466	2.39783e-005	1.51065	E11.5 * Sox10 up vs E11.5 * Ctrl
10427772	Tars	NM_033074	3.11203e-005	1.51	E11.5 * Sox10 up vs E11.5 * Ctrl
10363575	Dna2	NM_177372	5.93231e-008	1.50993	E11.5 * Sox10 up vs E11.5 * Ctrl
10543686	Ube2h	NM_009459	0.000229913	1.50992	E11.5 * Sox10 up vs E11.5 * Ctrl
10575512	Cog4	NM_133973	7.37856e-006	1.50895	E11.5 * Sox10 up vs E11.5 * Ctrl
10590445	Snrk	NM_133741	9.72013e-006	1.5086	E11.5 * Sox10 up vs E11.5 * Ctrl
10361023	Prox1	NM_008937	0.00142728	1.50856	E11.5 * Sox10 up vs E11.5 * Ctrl
10499108	Glt28d2	NM_177130	0.000177487	1.50843	E11.5 * Sox10 up vs E11.5 * Ctrl
10472794	Metapl1	NM_025633	0.000228728	1.50814	E11.5 * Sox10 up vs E11.5 * Ctrl
10358668	Hmcn1	NM_001024720	7.06355e-006	1.50736	E11.5 * Sox10 up vs E11.5 * Ctrl
10455372	C330007P06Rik	NM_029951	5.27853e-005	1.50735	E11.5 * Sox10 up vs E11.5 * Ctrl
10434348	Eif2b5	NM_172265	8.38452e-006	1.50732	E11.5 * Sox10 up vs E11.5 * Ctrl
10569429	Cdkn1c	NM_009876	1.00912e-008	1.50636	E11.5 * Sox10 up vs E11.5 * Ctrl
10463138	Dppa2	NM_028615	0.000358575	1.50629	E11.5 * Sox10 up vs E11.5 * Ctrl
10579607	B3gnt3	BC026418	0.00493536	1.50585	E11.5 * Sox10 up vs E11.5 * Ctrl
10595094	2310046A06Rik	BC089626	0.00227359	1.50576	E11.5 * Sox10 up vs E11.5 * Ctrl
10523479	Slc25a5	NM_007451	9.64535e-007	1.50561	E11.5 * Sox10 up vs E11.5 * Ctrl
10607587	Pdha1	NM_008810	8.96611e-006	1.50543	E11.5 * Sox10 up vs E11.5 * Ctrl
10495343	Wdr47	NM_181400	0.000781772	1.50504	E11.5 * Sox10 up vs E11.5 * Ctrl
10435271	Heg1	NM_175256	1.02027e-006	1.505	E11.5 * Sox10 up vs E11.5 * Ctrl
10376747	Kcnj12	NM_010603	0.000153289	1.50397	E11.5 * Sox10 up vs E11.5 * Ctrl
10413726	Tnnc1	NM_009393	0.00918489	1.50386	E11.5 * Sox10 up vs E11.5 * Ctrl
10356154	Sphkap	NM_172430	0.00254683	1.5038	E11.5 * Sox10 up vs E11.5 * Ctrl
10356833	Stk25	NM_021537	0.000434623	1.50303	E11.5 * Sox10 up vs E11.5 * Ctrl
10476314	Prnp	NM_011170	2.35192e-006	1.5025	E11.5 * Sox10 up vs E11.5 * Ctrl
10470027	Npdc1	NM_008721	0.000115692	1.50191	E11.5 * Sox10 up vs E11.5 * Ctrl
10469936	Nrarp	NM_025980	3.82045e-006	1.50144	E11.5 * Sox10 up vs E11.5 * Ctrl

10373388	Obfc2b	NM_027257	0.000227461	1.50131	E11.5 * Sox10 up vs E11.5 * Ctrl
10411882	Nln	NM_029447	1.07048e-005	1.50128	E11.5 * Sox10 up vs E11.5 * Ctrl
10465521	Plcb3	NM_008874	5.55818e-006	1.50063	E11.5 * Sox10 up vs E11.5 * Ctrl
10367122	Baz2a	NM_054078	1.6382e-008	1.50044	E11.5 * Sox10 up vs E11.5 * Ctrl
10583145	Tmem123	NM_133739	7.25596e-007	1.49995	E11.5 * Sox10 up vs E11.5 * Ctrl
10494595	Notch2	NM_010928	4.99257e-005	1.49989	E11.5 * Sox10 up vs E11.5 * Ctrl
10363901	Etv5	NM_023794	1.9e-005	1.49985	E11.5 * Sox10 up vs E11.5 * Ctrl
10484888	Ptprj	NM_008982	0.000332893	1.49959	E11.5 * Sox10 up vs E11.5 * Ctrl
10378914	Myo18a	NM_011586	9.75163e-008	1.49953	E11.5 * Sox10 up vs E11.5 * Ctrl
10358527	Hmcn1	NM_001024720	0.00069359	1.49935	E11.5 * Sox10 up vs E11.5 * Ctrl
10527516	Wasf3	NM_145155	0.000127256	1.49934	E11.5 * Sox10 up vs E11.5 * Ctrl
10458461	Hdac3	NM_010411	1.55357e-006	1.49897	E11.5 * Sox10 up vs E11.5 * Ctrl
10368577	Rnf217	NM_001146349	0.000102621	1.49891	E11.5 * Sox10 up vs E11.5 * Ctrl
10352815	Irf6	NM_016851	9.45387e-005	1.49763	E11.5 * Sox10 up vs E11.5 * Ctrl
10374529	AV249152	NM_145425	4.36692e-005	1.49759	E11.5 * Sox10 up vs E11.5 * Ctrl
10440929	Gart	NM_010256	1.28013e-006	1.49697	E11.5 * Sox10 up vs E11.5 * Ctrl
10540391	Trnt1	NM_027296	6.33339e-005	1.49687	E11.5 * Sox10 up vs E11.5 * Ctrl
10358625	Hmcn1	NM_001024720	0.00195034	1.49678	E11.5 * Sox10 up vs E11.5 * Ctrl
10534120	Asl	NM_133768	1.98871e-005	1.49648	E11.5 * Sox10 up vs E11.5 * Ctrl
10350146	Phlda3	NM_013750	0.000139638	1.49637	E11.5 * Sox10 up vs E11.5 * Ctrl
10358615	Hmcn1	NM_001024720	0.000325784	1.49586	E11.5 * Sox10 up vs E11.5 * Ctrl
10540544	Thumpd3	NM_008188	1.40495e-006	1.49578	E11.5 * Sox10 up vs E11.5 * Ctrl
10380815	Psmb3	NM_011971	0.000839994	1.49567	E11.5 * Sox10 up vs E11.5 * Ctrl
10445325	Rcan2	NM_030598	0.00232065	1.49557	E11.5 * Sox10 up vs E11.5 * Ctrl
10388861	Tmem199	NM_199199	0.000894765	1.49544	E11.5 * Sox10 up vs E11.5 * Ctrl
10583992	Igsf9b	NM_001129787	1.49281e-006	1.49501	E11.5 * Sox10 up vs E11.5 * Ctrl
10593966	Csk	NM_007783	0.00199374	1.495	E11.5 * Sox10 up vs E11.5 * Ctrl
10358591	Hmcn1	NM_001024720	0.000258073	1.49489	E11.5 * Sox10 up vs E11.5 * Ctrl
10376021	08-Sep	NM_033144	1.59333e-005	1.49461	E11.5 * Sox10 up vs E11.5 * Ctrl
10485388	Ldlrad3	NM_178886	0.000111367	1.4945	E11.5 * Sox10 up vs E11.5 * Ctrl
10493309	Rit1	NM_009069	0.00154344	1.49408	E11.5 * Sox10 up vs E11.5 * Ctrl
10358603	Hmcn1	NM_001024720	0.000388744	1.49369	E11.5 * Sox10 up vs E11.5 * Ctrl
10481368	Trub2	NM_145520	3.47377e-005	1.49347	E11.5 * Sox10 up vs E11.5 * Ctrl
10361152	Gstp2	NM_181796	1.01979e-005	1.49325	E11.5 * Sox10 up vs E11.5 * Ctrl
10437590	Carhsp1	NM_025821	2.98208e-007	1.4929	E11.5 * Sox10 up vs E11.5 * Ctrl
10409767	Golm1	NM_027307	9.78481e-005	1.49258	E11.5 * Sox10 up vs E11.5 * Ctrl
10419073	Tspan14	NM_145928	3.02676e-005	1.49238	E11.5 * Sox10 up vs E11.5 * Ctrl
10444932	2310014H01Rik	NM_001146711	7.67374e-005	1.49203	E11.5 * Sox10 up vs E11.5 * Ctrl
10456400	Tubb6	NM_026473	2.07021e-007	1.49126	E11.5 * Sox10 up vs E11.5 * Ctrl
10404763	Tmem170b	XM_886379	2.72844e-005	1.49122	E11.5 * Sox10 up vs E11.5 * Ctrl
10571399	Zdhhc2	NM_178395	0.000802059	1.49103	E11.5 * Sox10 up vs E11.5 * Ctrl
10358593	Hmcn1	NM_001024720	0.000437138	1.49066	E11.5 * Sox10 up vs E11.5 * Ctrl
10497309	Snx16	NM_029068	0.00578064	1.49053	E11.5 * Sox10 up vs E11.5 * Ctrl
10363231	Smpdl3a	NM_020561	0.000337425	1.49006	E11.5 * Sox10 up vs E11.5 * Ctrl
10578222	Dlc1	NM_015802	4.60255e-005	1.49005	E11.5 * Sox10 up vs E11.5 * Ctrl
10436666	Jam2	NM_023844	4.74854e-005	1.48969	E11.5 * Sox10 up vs E11.5 * Ctrl
10479948	Cugbp2	NM_001110228	0.00427309	1.48936	E11.5 * Sox10 up vs E11.5 * Ctrl
10371904	1110012L19Rik	NM_026787	0.00409605	1.48888	E11.5 * Sox10 up vs E11.5 * Ctrl
10501629	Cdc14a	NM_001080818	5.72019e-007	1.48884	E11.5 * Sox10 up vs E11.5 * Ctrl
10438702	St6gal1	NM_145933	0.00100408	1.48881	E11.5 * Sox10 up vs E11.5 * Ctrl
10418185	D14Ertd449e	NM_025311	0.000174213	1.48836	E11.5 * Sox10 up vs E11.5 * Ctrl
10393970	Fasn	NM_007988	0.00310757	1.48823	E11.5 * Sox10 up vs E11.5 * Ctrl
10358457	Bex4	NM_212457	3.1924e-005	1.4877	E11.5 * Sox10 up vs E11.5 * Ctrl
10365482	Timp3	NM_011595	0.00147749	1.48764	E11.5 * Sox10 up vs E11.5 * Ctrl
10484894	Ptprj	NM_008982	0.000244248	1.48709	E11.5 * Sox10 up vs E11.5 * Ctrl
10570178	A230072I06Rik	ENSMUST00000(	0.00624946	1.48686	E11.5 * Sox10 up vs E11.5 * Ctrl
10567355	Gprc5b	NM_022420	1.32305e-005	1.48649	E11.5 * Sox10 up vs E11.5 * Ctrl
10368011	Vta1	NM_025418	1.62072e-006	1.48615	E11.5 * Sox10 up vs E11.5 * Ctrl
10392207	Tex2	NM_198292	0.00019329	1.48591	E11.5 * Sox10 up vs E11.5 * Ctrl
10578623	Wwc2	NM_133791	2.71897e-007	1.48558	E11.5 * Sox10 up vs E11.5 * Ctrl
10455656	Hsd17b4	NM_008292	3.54082e-008	1.48536	E11.5 * Sox10 up vs E11.5 * Ctrl
10435057	Pcyt1a	NM_009981	4.4827e-005	1.48527	E11.5 * Sox10 up vs E11.5 * Ctrl
10518726	Slc25a33	NM_027460	2.62317e-006	1.48525	E11.5 * Sox10 up vs E11.5 * Ctrl
10479749	Rpp38	NM_001013376	0.00882615	1.48513	E11.5 * Sox10 up vs E11.5 * Ctrl
10411274	Sv2c	NM_029210	8.03454e-005	1.48505	E11.5 * Sox10 up vs E11.5 * Ctrl
10535080	Cox19	NM_197980	7.79657e-005	1.48492	E11.5 * Sox10 up vs E11.5 * Ctrl

10358561	Hmcn1	NM_001024720	0.000767059	1.48407	E11.5 * Sox10 up vs E11.5 * Ctrl
10358541	Hmcn1	NM_001024720	0.00762955	1.48382	E11.5 * Sox10 up vs E11.5 * Ctrl
10467730	Morn4	NM_198108	0.00840981	1.48283	E11.5 * Sox10 up vs E11.5 * Ctrl
10599215	Slc25a5	NM_007451	8.25207e-007	1.4828	E11.5 * Sox10 up vs E11.5 * Ctrl
10495094	6530418L21Rik	BC052371	4.09742e-005	1.48265	E11.5 * Sox10 up vs E11.5 * Ctrl
10396919	4933426M11Rik	BC040401	5.6074e-007	1.4813	E11.5 * Sox10 up vs E11.5 * Ctrl
10582916		---	0.00649806	1.48099	E11.5 * Sox10 up vs E11.5 * Ctrl
10394778	Hpcal1	NM_016677	0.000905263	1.48087	E11.5 * Sox10 up vs E11.5 * Ctrl
10469613	Thnsl1	NM_177588	4.0847e-005	1.47976	E11.5 * Sox10 up vs E11.5 * Ctrl
10515399	Plk3	NM_013807	0.00439164	1.47968	E11.5 * Sox10 up vs E11.5 * Ctrl
10447341	Rhoq	NM_145491	6.12419e-005	1.47965	E11.5 * Sox10 up vs E11.5 * Ctrl
10558454	Glrx3	NM_023140	0.000121688	1.47911	E11.5 * Sox10 up vs E11.5 * Ctrl
10358589	Hmcn1	NM_001024720	0.000327466	1.47805	E11.5 * Sox10 up vs E11.5 * Ctrl
10373814	Pes1	NM_022889	0.000163483	1.47804	E11.5 * Sox10 up vs E11.5 * Ctrl
10595466	Pgm3	NM_028352	8.19919e-006	1.47801	E11.5 * Sox10 up vs E11.5 * Ctrl
10418198	D14Ertd449e	NM_025311	0.000155937	1.47778	E11.5 * Sox10 up vs E11.5 * Ctrl
10573128	Tbc1d9	NM_001111304	6.22221e-009	1.47765	E11.5 * Sox10 up vs E11.5 * Ctrl
10417813	Ecd	NM_027475	1.28034e-007	1.47722	E11.5 * Sox10 up vs E11.5 * Ctrl
10358563	Hmcn1	NM_001024720	0.000138021	1.47696	E11.5 * Sox10 up vs E11.5 * Ctrl
10394929	EG668662	AK132630	0.000684587	1.47693	E11.5 * Sox10 up vs E11.5 * Ctrl
10355960	Scg2	NM_009129	0.00604018	1.47632	E11.5 * Sox10 up vs E11.5 * Ctrl
10534575	Prkrip1	NM_025774	3.13282e-006	1.47627	E11.5 * Sox10 up vs E11.5 * Ctrl
10399419	Tubb2b	NM_023716	0.000177683	1.47582	E11.5 * Sox10 up vs E11.5 * Ctrl
10412100	Map3k1	NM_011945	0.000160302	1.47576	E11.5 * Sox10 up vs E11.5 * Ctrl
10545672	Mthfd2	NM_008638	0.000210337	1.47492	E11.5 * Sox10 up vs E11.5 * Ctrl
10490159	Pmepa1	NM_022995	3.83124e-005	1.47481	E11.5 * Sox10 up vs E11.5 * Ctrl
10510464	Lzic	NM_026963	7.62881e-006	1.47477	E11.5 * Sox10 up vs E11.5 * Ctrl
10388898	1810009O10Rik	BC027022	0.00444148	1.47456	E11.5 * Sox10 up vs E11.5 * Ctrl
10387757	Rai12	NM_018740	0.0002753	1.47413	E11.5 * Sox10 up vs E11.5 * Ctrl
10406823		---	0.00454893	1.47411	E11.5 * Sox10 up vs E11.5 * Ctrl
10378870	Git1	NM_001004144	0.000912006	1.4737	E11.5 * Sox10 up vs E11.5 * Ctrl
10379223	1810012P15Rik	NM_001076681	0.000399967	1.47325	E11.5 * Sox10 up vs E11.5 * Ctrl
10499168	Kirrel	NM_130867	0.00147647	1.47275	E11.5 * Sox10 up vs E11.5 * Ctrl
10365833	Usp44	NM_183199	0.000662196	1.47259	E11.5 * Sox10 up vs E11.5 * Ctrl
10418002	EG620119	XM_884506	0.000468481	1.47257	E11.5 * Sox10 up vs E11.5 * Ctrl
10556178	Tub	NM_021885	4.64704e-005	1.47243	E11.5 * Sox10 up vs E11.5 * Ctrl
10365518	Nt5dc3	NM_175331	0.000591371	1.47232	E11.5 * Sox10 up vs E11.5 * Ctrl
10379543	Tmem132e	NM_023438	0.000415099	1.47151	E11.5 * Sox10 up vs E11.5 * Ctrl
10394158	Kif3c	NM_008445	7.54752e-005	1.47078	E11.5 * Sox10 up vs E11.5 * Ctrl
10460517	Brms1	NM_134155	0.000495911	1.47061	E11.5 * Sox10 up vs E11.5 * Ctrl
10594590	Snx1	NM_019727	2.30981e-006	1.47049	E11.5 * Sox10 up vs E11.5 * Ctrl
10360460	Chml	NM_021350	0.000615876	1.46877	E11.5 * Sox10 up vs E11.5 * Ctrl
10409338	Nop16	NM_178605	0.000290821	1.46848	E11.5 * Sox10 up vs E11.5 * Ctrl
10461093	Pla2g16	NM_139269	0.00153217	1.46827	E11.5 * Sox10 up vs E11.5 * Ctrl
10579335	Pgpep1	NM_023217	6.34114e-006	1.46793	E11.5 * Sox10 up vs E11.5 * Ctrl
10405211	Gadd45g	NM_011817	0.00207839	1.46742	E11.5 * Sox10 up vs E11.5 * Ctrl
10572730	Zfp617	NM_133358	0.00958889	1.46672	E11.5 * Sox10 up vs E11.5 * Ctrl
10499062	Fhdc1	NM_001033301	0.00548966	1.46666	E11.5 * Sox10 up vs E11.5 * Ctrl
10385283	Gabrg2	NM_008073	0.00167543	1.46556	E11.5 * Sox10 up vs E11.5 * Ctrl
10566714	Ric3	NM_001038624	0.000100906	1.46532	E11.5 * Sox10 up vs E11.5 * Ctrl
10417065	Rap2a	NM_029519	1.43201e-006	1.46517	E11.5 * Sox10 up vs E11.5 * Ctrl
10371230	Gna11	NM_010301	0.000102096	1.465	E11.5 * Sox10 up vs E11.5 * Ctrl
10449920	Zfp811	NM_183177	0.00184202	1.4648	E11.5 * Sox10 up vs E11.5 * Ctrl
10426397	Cntn1	NM_007727	0.000298203	1.46462	E11.5 * Sox10 up vs E11.5 * Ctrl
10442914	0610011F06Rik	NM_026686	1.10684e-005	1.46439	E11.5 * Sox10 up vs E11.5 * Ctrl
10418210	D14Ertd449e	NM_025311	0.000182896	1.46439	E11.5 * Sox10 up vs E11.5 * Ctrl
10363905	Zwint	NM_025635	1.25815e-007	1.46339	E11.5 * Sox10 up vs E11.5 * Ctrl
10472436	B3galt1	NM_020283	0.000239672	1.46312	E11.5 * Sox10 up vs E11.5 * Ctrl
10354031	Tsga10	NM_207228	0.00164176	1.46307	E11.5 * Sox10 up vs E11.5 * Ctrl
10413255	Duxbl	NM_183389	0.000449755	1.463	E11.5 * Sox10 up vs E11.5 * Ctrl
10413265	Duxbl	NM_183389	0.000449755	1.463	E11.5 * Sox10 up vs E11.5 * Ctrl
10491300	Skil	NM_011386	1.21251e-005	1.46273	E11.5 * Sox10 up vs E11.5 * Ctrl
10379654	Ap2b1	NM_001035854	8.57493e-008	1.46266	E11.5 * Sox10 up vs E11.5 * Ctrl
10367734	Ust	NM_177387	0.000169418	1.46229	E11.5 * Sox10 up vs E11.5 * Ctrl
10568529	Ikzf5	NM_175115	0.00248884	1.46219	E11.5 * Sox10 up vs E11.5 * Ctrl
10485151	Mapk8ip1	NM_011162	0.000191319	1.46215	E11.5 * Sox10 up vs E11.5 * Ctrl

10580537	Aktip	NM_010241	0.000199766	1.46198	E11.5 * Sox10 up vs E11.5 * Ctrl
10512226	Wdr40a	NM_026893	6.09039e-005	1.46138	E11.5 * Sox10 up vs E11.5 * Ctrl
10504703	AU014645	NM_001033201	1.28778e-006	1.46129	E11.5 * Sox10 up vs E11.5 * Ctrl
10512308	Sigmar1	NM_011014	0.00051085	1.4612	E11.5 * Sox10 up vs E11.5 * Ctrl
10456587	Mro	NM_027741	8.90289e-005	1.46087	E11.5 * Sox10 up vs E11.5 * Ctrl
10524941	Fbxo21	NM_145564	2.60234e-005	1.46083	E11.5 * Sox10 up vs E11.5 * Ctrl
10593937	Mpi	NM_025837	1.44518e-005	1.4605	E11.5 * Sox10 up vs E11.5 * Ctrl
10524882	Wsb2	NM_021539	0.000106339	1.46037	E11.5 * Sox10 up vs E11.5 * Ctrl
10477297	Kif3b	NM_008444	7.63669e-005	1.46028	E11.5 * Sox10 up vs E11.5 * Ctrl
10372116	Csl	NM_027945	6.41134e-007	1.46002	E11.5 * Sox10 up vs E11.5 * Ctrl
10509777	Iffo2	NM_183148	8.7299e-005	1.45993	E11.5 * Sox10 up vs E11.5 * Ctrl
10501909	Mettl14	NM_201638	1.34603e-007	1.4599	E11.5 * Sox10 up vs E11.5 * Ctrl
10512391	Vcp	NM_009503	7.22416e-008	1.4595	E11.5 * Sox10 up vs E11.5 * Ctrl
10599498	Utp14a	NM_028276	1.32833e-007	1.45949	E11.5 * Sox10 up vs E11.5 * Ctrl
10524684	Msi1	NM_008629	0.00341775	1.45924	E11.5 * Sox10 up vs E11.5 * Ctrl
10587604	Rwdd2a	NM_027100	0.000237359	1.45905	E11.5 * Sox10 up vs E11.5 * Ctrl
10346235	Hibch	NM_146108	0.00051592	1.45862	E11.5 * Sox10 up vs E11.5 * Ctrl
10416037	Pbk	NM_023209	0.000544444	1.45853	E11.5 * Sox10 up vs E11.5 * Ctrl
10359851	Uck2	NM_030724	4.21804e-005	1.45787	E11.5 * Sox10 up vs E11.5 * Ctrl
10382701	Sap30bp	NM_020483	0.000222588	1.45787	E11.5 * Sox10 up vs E11.5 * Ctrl
10394625		---	0.00205228	1.45698	E11.5 * Sox10 up vs E11.5 * Ctrl
10517425	Lypla2	NM_011942	0.00500603	1.45695	E11.5 * Sox10 up vs E11.5 * Ctrl
10437580		---	0.00187248	1.45678	E11.5 * Sox10 up vs E11.5 * Ctrl
10601850	Bex4	NM_212457	1.69703e-005	1.45677	E11.5 * Sox10 up vs E11.5 * Ctrl
10538082	Atp6v0e2	NM_133764	0.00013203	1.45674	E11.5 * Sox10 up vs E11.5 * Ctrl
10349239	Mki67ip	NM_026472	9.9666e-006	1.45613	E11.5 * Sox10 up vs E11.5 * Ctrl
10570144	Arhgef7	NM_001113518	5.24067e-007	1.45553	E11.5 * Sox10 up vs E11.5 * Ctrl
10476189	Mrps26	NM_207207	0.000924185	1.45508	E11.5 * Sox10 up vs E11.5 * Ctrl
10463535	Nolc1	NM_053086	2.11479e-006	1.45462	E11.5 * Sox10 up vs E11.5 * Ctrl
10538290	Snx10	NM_028035	5.46868e-005	1.45402	E11.5 * Sox10 up vs E11.5 * Ctrl
10562989	Cpt1c	NM_153679	3.40567e-005	1.4534	E11.5 * Sox10 up vs E11.5 * Ctrl
10526113	Crcp	NM_007761	0.00681585	1.45318	E11.5 * Sox10 up vs E11.5 * Ctrl
10555953	Fxc1	NM_019502	3.27581e-005	1.45291	E11.5 * Sox10 up vs E11.5 * Ctrl
10422052	Commd6	NM_001033132	0.0003072	1.45258	E11.5 * Sox10 up vs E11.5 * Ctrl
10503508	Ggh	NM_010281	0.000106172	1.45255	E11.5 * Sox10 up vs E11.5 * Ctrl
10503523	Ggh	NM_010281	0.000106172	1.45255	E11.5 * Sox10 up vs E11.5 * Ctrl
10552311	OTTMUSG00000	XR_030737	2.19083e-005	1.45118	E11.5 * Sox10 up vs E11.5 * Ctrl
10510391	Srm	NM_009272	7.24846e-005	1.45087	E11.5 * Sox10 up vs E11.5 * Ctrl
10345230	Rab23	NM_008999	8.91545e-006	1.45072	E11.5 * Sox10 up vs E11.5 * Ctrl
10397332	Eif2b2	NM_145445	0.00253702	1.45022	E11.5 * Sox10 up vs E11.5 * Ctrl
10488589	Fam110a	NM_028666	8.68368e-005	1.45019	E11.5 * Sox10 up vs E11.5 * Ctrl
10509838	Padi2	NM_008812	0.00324916	1.44983	E11.5 * Sox10 up vs E11.5 * Ctrl
10451665	Apobec2	NM_009694	0.00122532	1.44935	E11.5 * Sox10 up vs E11.5 * Ctrl
10355214	Idh1	NM_010497	1.01298e-006	1.4493	E11.5 * Sox10 up vs E11.5 * Ctrl
10384452	Ublcp1	NM_024475	0.000555528	1.44927	E11.5 * Sox10 up vs E11.5 * Ctrl
10425116	Cdc42ep1	NM_027219	0.00404519	1.44892	E11.5 * Sox10 up vs E11.5 * Ctrl
10463799	EG329070	ENSMUST00000	2.54298e-006	1.44883	E11.5 * Sox10 up vs E11.5 * Ctrl
10497214	Tpd52	NM_001025261	0.00294993	1.44877	E11.5 * Sox10 up vs E11.5 * Ctrl
10380226	Cuedc1	NM_198013	0.0018592	1.44872	E11.5 * Sox10 up vs E11.5 * Ctrl
10413243	Duxbl	NM_183389	0.000582328	1.44797	E11.5 * Sox10 up vs E11.5 * Ctrl
10546346	Chchd4	NM_133928	0.000107341	1.44767	E11.5 * Sox10 up vs E11.5 * Ctrl
10405729	Selk	NM_019979	0.000375579	1.44735	E11.5 * Sox10 up vs E11.5 * Ctrl
10426835	Dip2b	NM_001159361	4.13978e-006	1.44732	E11.5 * Sox10 up vs E11.5 * Ctrl
10375234	Nudcd2	NM_026023	3.01185e-008	1.44711	E11.5 * Sox10 up vs E11.5 * Ctrl
10386636	Usp22	NM_001004143	6.03119e-007	1.44693	E11.5 * Sox10 up vs E11.5 * Ctrl
10456378	Chmp1b	NM_024190	0.00164988	1.44678	E11.5 * Sox10 up vs E11.5 * Ctrl
10535449	E130309D02Rik	NM_172726	0.000272247	1.44676	E11.5 * Sox10 up vs E11.5 * Ctrl
10358733	Rgl1	NM_016846	0.000160931	1.44614	E11.5 * Sox10 up vs E11.5 * Ctrl
10388465	Doc2b	NM_007873	0.000133277	1.44607	E11.5 * Sox10 up vs E11.5 * Ctrl
10450957	Cenpq	NM_031863	0.000599826	1.44605	E11.5 * Sox10 up vs E11.5 * Ctrl
10455227	Rnf14	NM_020012	5.39459e-008	1.44604	E11.5 * Sox10 up vs E11.5 * Ctrl
10604637	Cxx1b	NM_001018063	2.38387e-007	1.44573	E11.5 * Sox10 up vs E11.5 * Ctrl
10380415	Cdc34	BC094502	0.000617371	1.44573	E11.5 * Sox10 up vs E11.5 * Ctrl
10424607	Ptp4a3	NM_008975	0.00114285	1.44535	E11.5 * Sox10 up vs E11.5 * Ctrl
10603000	Rai2	NM_198409	0.00709819	1.44527	E11.5 * Sox10 up vs E11.5 * Ctrl
10404827	Nol7	NM_023554	2.3789e-005	1.44478	E11.5 * Sox10 up vs E11.5 * Ctrl

10445241	Tnfrsf21	NM_178589	6.10692e-005	1.44421	E11.5 * Sox10 up vs E11.5 * Ctrl
10357220	Tmem177	NM_175106	0.00317905	1.44397	E11.5 * Sox10 up vs E11.5 * Ctrl
10516371	Eif2c1	NM_153403	0.000168404	1.44367	E11.5 * Sox10 up vs E11.5 * Ctrl
10355958	Ube2v1	NM_023230	0.000139268	1.44336	E11.5 * Sox10 up vs E11.5 * Ctrl
10457834		---	0.00229022	1.4432	E11.5 * Sox10 up vs E11.5 * Ctrl
10372682	Slc35e3	NM_029875	0.00757681	1.44307	E11.5 * Sox10 up vs E11.5 * Ctrl
10452556	Rab12	NM_024448	0.00147124	1.44273	E11.5 * Sox10 up vs E11.5 * Ctrl
10440840	1110004E09Rik	BC019533	4.26047e-006	1.44234	E11.5 * Sox10 up vs E11.5 * Ctrl
10492325	Ube2v1	NM_023230	6.51712e-005	1.44234	E11.5 * Sox10 up vs E11.5 * Ctrl
10375893	Sar1b	NM_025535	4.99235e-007	1.44205	E11.5 * Sox10 up vs E11.5 * Ctrl
10527965	Cldn12	NM_022890	0.010133	1.44167	E11.5 * Sox10 up vs E11.5 * Ctrl
10414325	Cgrrf1	NM_026832	0.0016797	1.44114	E11.5 * Sox10 up vs E11.5 * Ctrl
10492590	Ppm1l	NM_178726	0.000325186	1.44087	E11.5 * Sox10 up vs E11.5 * Ctrl
10540105	Tmem43	NM_028766	0.000480052	1.44037	E11.5 * Sox10 up vs E11.5 * Ctrl
10534531	Ywhag	NM_018871	0.000672894	1.43988	E11.5 * Sox10 up vs E11.5 * Ctrl
10542791	Ppfibp1	NM_026221	3.48077e-005	1.43962	E11.5 * Sox10 up vs E11.5 * Ctrl
10497285	Impa1	NM_018864	0.00141053	1.43933	E11.5 * Sox10 up vs E11.5 * Ctrl
10489694	Zfp334	NM_178411	0.000212836	1.43908	E11.5 * Sox10 up vs E11.5 * Ctrl
10500582	Wdr3	NM_175552	5.45203e-005	1.43856	E11.5 * Sox10 up vs E11.5 * Ctrl
10516064	Mfsd2	NM_029662	0.00242619	1.43841	E11.5 * Sox10 up vs E11.5 * Ctrl
10442816	Lmf1	NM_029624	3.8984e-005	1.43696	E11.5 * Sox10 up vs E11.5 * Ctrl
10545910	Pcyox1	NM_025823	1.40621e-005	1.43629	E11.5 * Sox10 up vs E11.5 * Ctrl
10566804	Tmem9b	NM_020050	0.000128765	1.4359	E11.5 * Sox10 up vs E11.5 * Ctrl
10589130	Celsr3	NM_080437	0.000188934	1.43514	E11.5 * Sox10 up vs E11.5 * Ctrl
10469070	Nudt5	NM_016918	6.18053e-005	1.43507	E11.5 * Sox10 up vs E11.5 * Ctrl
10573998	Ogfod1	NM_177767	0.00348936	1.43458	E11.5 * Sox10 up vs E11.5 * Ctrl
10486875	Frmd5	NM_172673	0.000139182	1.43396	E11.5 * Sox10 up vs E11.5 * Ctrl
10358559	Hmcn1	NM_001024720	5.69378e-005	1.43391	E11.5 * Sox10 up vs E11.5 * Ctrl
10375973	Taf13	NM_025444	6.31335e-005	1.43372	E11.5 * Sox10 up vs E11.5 * Ctrl
10375880	Nhp2	NM_026631	0.000407494	1.43366	E11.5 * Sox10 up vs E11.5 * Ctrl
10409330	4833439L19Rik	BC033445	0.00482466	1.43361	E11.5 * Sox10 up vs E11.5 * Ctrl
10358670	Hmcn1	NM_001024720	0.000104259	1.43323	E11.5 * Sox10 up vs E11.5 * Ctrl
10520842	Bre	NM_181279	1.45118e-005	1.43299	E11.5 * Sox10 up vs E11.5 * Ctrl
10504218	Dnajb5	NM_019874	0.000326956	1.43288	E11.5 * Sox10 up vs E11.5 * Ctrl
10379891	Ppm1d	NM_016910	3.33661e-006	1.43279	E11.5 * Sox10 up vs E11.5 * Ctrl
10358666	Hmcn1	NM_001024720	2.91627e-005	1.43254	E11.5 * Sox10 up vs E11.5 * Ctrl
10589041	Impdh2	NM_011830	1.18172e-005	1.4325	E11.5 * Sox10 up vs E11.5 * Ctrl
10448034	Dll1	NM_007865	0.000186527	1.43242	E11.5 * Sox10 up vs E11.5 * Ctrl
10376854	Pigl	NM_001039536	3.72231e-005	1.43198	E11.5 * Sox10 up vs E11.5 * Ctrl
10606554	Nap1l3	NM_138742	0.000629703	1.43181	E11.5 * Sox10 up vs E11.5 * Ctrl
10575685	Nudt7	NM_024437	0.00708342	1.43174	E11.5 * Sox10 up vs E11.5 * Ctrl
10391454	Vat1	NM_012037	0.00111356	1.43169	E11.5 * Sox10 up vs E11.5 * Ctrl
10383799	Tcn2	NM_015749	1.4734e-005	1.4316	E11.5 * Sox10 up vs E11.5 * Ctrl
10381744	Arf2	NM_007477	5.01992e-005	1.43057	E11.5 * Sox10 up vs E11.5 * Ctrl
10578810	Clcn3	NM_173874	5.55426e-006	1.43054	E11.5 * Sox10 up vs E11.5 * Ctrl
10585214	Cryab	NM_009964	0.00421741	1.4305	E11.5 * Sox10 up vs E11.5 * Ctrl
10411171	Pde8b	NM_172263	7.76945e-006	1.43004	E11.5 * Sox10 up vs E11.5 * Ctrl
10531146	Mkrn1-ps1	AF494488	0.000486699	1.42985	E11.5 * Sox10 up vs E11.5 * Ctrl
10478447	Stk4	NM_021420	1.06524e-007	1.42947	E11.5 * Sox10 up vs E11.5 * Ctrl
10410959	Atg10	NM_025770	0.00281281	1.4292	E11.5 * Sox10 up vs E11.5 * Ctrl
10363498	Ppa1	NM_026438	4.2675e-007	1.42905	E11.5 * Sox10 up vs E11.5 * Ctrl
10445496	Yipf3	NM_145353	0.0067888	1.42904	E11.5 * Sox10 up vs E11.5 * Ctrl
10431170	5031439G07Rik	NM_001033273	0.000182432	1.42902	E11.5 * Sox10 up vs E11.5 * Ctrl
10561335	Prkcz	NM_008860	0.00797245	1.42893	E11.5 * Sox10 up vs E11.5 * Ctrl
10388227	Cyb5d2	NM_001024926	0.00207037	1.42877	E11.5 * Sox10 up vs E11.5 * Ctrl
10489660	Elmo2	NM_207706	0.000514849	1.4285	E11.5 * Sox10 up vs E11.5 * Ctrl
10467956	Bloc1s2	BC065806	0.00129202	1.42844	E11.5 * Sox10 up vs E11.5 * Ctrl
10490838	Fabp5	NM_010634	5.53895e-006	1.4278	E11.5 * Sox10 up vs E11.5 * Ctrl
10448865	Gnptg	NM_172529	8.2608e-007	1.42755	E11.5 * Sox10 up vs E11.5 * Ctrl
10547521	Atp6v1e1	NM_007510	0.000443145	1.42748	E11.5 * Sox10 up vs E11.5 * Ctrl
10405757	2010111I01Rik	NM_028079	5.61348e-005	1.42709	E11.5 * Sox10 up vs E11.5 * Ctrl
10381345	Psme3	NM_011192	1.85767e-005	1.42688	E11.5 * Sox10 up vs E11.5 * Ctrl
10504551	Rg9mtd3	NM_027266	1.97415e-005	1.42674	E11.5 * Sox10 up vs E11.5 * Ctrl
10518167	Trappc2	NM_025432	0.00814362	1.42591	E11.5 * Sox10 up vs E11.5 * Ctrl
10518812	Camta1	NM_001081557	0.000259884	1.42588	E11.5 * Sox10 up vs E11.5 * Ctrl
10497321	Pgam1	NM_023418	1.55515e-005	1.42495	E11.5 * Sox10 up vs E11.5 * Ctrl

10571849	Fbxo8	NM_015791	0.000567936	1.42436	E11.5 * Sox10 up vs E11.5 * Ctrl
10582981	Tfdp1	NM_009361	7.48367e-006	1.42398	E11.5 * Sox10 up vs E11.5 * Ctrl
10503966	Aco1	NM_007386	2.32547e-005	1.42344	E11.5 * Sox10 up vs E11.5 * Ctrl
10471474	Ak1	NM_021515	0.00192224	1.42316	E11.5 * Sox10 up vs E11.5 * Ctrl
10404380	Dusp22	NM_001037955	1.15064e-005	1.4226	E11.5 * Sox10 up vs E11.5 * Ctrl
10494551	Acp6	NM_019800	0.000754816	1.42221	E11.5 * Sox10 up vs E11.5 * Ctrl
10587315	Gsta4	NM_010357	7.07985e-005	1.42205	E11.5 * Sox10 up vs E11.5 * Ctrl
10395466	Dock4	NM_172803	1.24854e-005	1.42194	E11.5 * Sox10 up vs E11.5 * Ctrl
10596543	Rad54l2	NM_030730	0.00297015	1.42185	E11.5 * Sox10 up vs E11.5 * Ctrl
10461844	Gnaq	NM_008139	3.18023e-005	1.42171	E11.5 * Sox10 up vs E11.5 * Ctrl
10538420	Gars	NM_180678	1.13087e-007	1.42163	E11.5 * Sox10 up vs E11.5 * Ctrl
10393387	Jmjd6	NM_033398	1.99394e-005	1.4216	E11.5 * Sox10 up vs E11.5 * Ctrl
10559415	Leng1	NM_027203	0.000344313	1.42157	E11.5 * Sox10 up vs E11.5 * Ctrl
10542397	H2afj	NM_177688	1.15112e-007	1.42152	E11.5 * Sox10 up vs E11.5 * Ctrl
10418766	Ankrd28	NM_001024604	0.000123827	1.42118	E11.5 * Sox10 up vs E11.5 * Ctrl
10431154	Phf21b	NM_001081166	0.000117689	1.42114	E11.5 * Sox10 up vs E11.5 * Ctrl
10551065	D930028M14Rik	ENSMUST00000(	0.000240026	1.42105	E11.5 * Sox10 up vs E11.5 * Ctrl
10420672	Dleu7	NM_173419	0.00286269	1.42071	E11.5 * Sox10 up vs E11.5 * Ctrl
10592126	Fam118b	NM_194257	8.09316e-006	1.42018	E11.5 * Sox10 up vs E11.5 * Ctrl
10545041	Nap1l5	NM_021432	0.0013126	1.41978	E11.5 * Sox10 up vs E11.5 * Ctrl
10397882	Chga	NM_007693	0.000477042	1.41961	E11.5 * Sox10 up vs E11.5 * Ctrl
10592593	Tecta	NM_009347	0.000442143	1.41951	E11.5 * Sox10 up vs E11.5 * Ctrl
10348580	Klhl30	NM_027551	0.000392245	1.41911	E11.5 * Sox10 up vs E11.5 * Ctrl
10490061	Bcas1	NM_029815	1.64279e-005	1.41896	E11.5 * Sox10 up vs E11.5 * Ctrl
10476817	Atpaf1	NM_181040	0.00052401	1.41884	E11.5 * Sox10 up vs E11.5 * Ctrl
10353934	Actr1b	NM_146107	0.00297053	1.41813	E11.5 * Sox10 up vs E11.5 * Ctrl
10589800	Clasp2	NM_001114347	5.19377e-008	1.41793	E11.5 * Sox10 up vs E11.5 * Ctrl
10599321	Zbtb33	NM_020256	0.00177256	1.41783	E11.5 * Sox10 up vs E11.5 * Ctrl
10457888	5730494M16Rik	NM_001004361	2.3479e-008	1.4177	E11.5 * Sox10 up vs E11.5 * Ctrl
10379968	Tubd1	NM_019756	0.00788627	1.41749	E11.5 * Sox10 up vs E11.5 * Ctrl
10556005	Ilk	NM_010562	3.40607e-006	1.41745	E11.5 * Sox10 up vs E11.5 * Ctrl
10598359	Syp	NM_009305	0.000156537	1.41714	E11.5 * Sox10 up vs E11.5 * Ctrl
10537712	Gstk1	NM_029555	0.000427489	1.41703	E11.5 * Sox10 up vs E11.5 * Ctrl
10519886	Sema3c	NM_013657	4.51141e-006	1.41682	E11.5 * Sox10 up vs E11.5 * Ctrl
10605055	Haus7	NM_028633	0.000521388	1.41638	E11.5 * Sox10 up vs E11.5 * Ctrl
10600114	Pnma3	NM_153169	0.000967148	1.41618	E11.5 * Sox10 up vs E11.5 * Ctrl
10401997	Ptpn21	NM_011877	2.24589e-005	1.41566	E11.5 * Sox10 up vs E11.5 * Ctrl
10504349	Creb3	NM_013497	8.15677e-005	1.41538	E11.5 * Sox10 up vs E11.5 * Ctrl
10355176	4921521F21Rik	BC051128	0.000639701	1.41528	E11.5 * Sox10 up vs E11.5 * Ctrl
10579799	Tmem184c	NM_145599	4.57273e-005	1.41497	E11.5 * Sox10 up vs E11.5 * Ctrl
10476814	Insm1	NM_016889	0.0031817	1.41455	E11.5 * Sox10 up vs E11.5 * Ctrl
10450226	Ppt2	NM_019441	0.0064722	1.41433	E11.5 * Sox10 up vs E11.5 * Ctrl
10534102	Gusb	NM_010368	0.000486402	1.41379	E11.5 * Sox10 up vs E11.5 * Ctrl
10562360	Gpi1	NM_008155	0.000123225	1.41375	E11.5 * Sox10 up vs E11.5 * Ctrl
10410124	Ctsl	NM_009984	4.00827e-007	1.41363	E11.5 * Sox10 up vs E11.5 * Ctrl
10585905	Parp6	NM_029922	1.48643e-005	1.41361	E11.5 * Sox10 up vs E11.5 * Ctrl
10566730	Stk33	ENSMUST00000	0.00049403	1.41337	E11.5 * Sox10 up vs E11.5 * Ctrl
10492890	Lrba	NM_030695	1.50469e-005	1.41298	E11.5 * Sox10 up vs E11.5 * Ctrl
10482109	Rbm18	NR_027515	1.20548e-005	1.41272	E11.5 * Sox10 up vs E11.5 * Ctrl
10361007	Smyd2	NM_026796	1.08697e-006	1.41246	E11.5 * Sox10 up vs E11.5 * Ctrl
10542757	Stk38l	NM_172734	0.004948	1.41232	E11.5 * Sox10 up vs E11.5 * Ctrl
10569181	Lrdd	NM_022654	0.000248683	1.41099	E11.5 * Sox10 up vs E11.5 * Ctrl
10384145	H2afv	BC028539	0.000410796	1.41087	E11.5 * Sox10 up vs E11.5 * Ctrl
10549097	Ldhb	NM_008492	2.81538e-007	1.41071	E11.5 * Sox10 up vs E11.5 * Ctrl
10510687	Acot7	NM_133348	0.00015301	1.41063	E11.5 * Sox10 up vs E11.5 * Ctrl
10478772	Arfgef2	NM_001085495	1.62141e-005	1.41018	E11.5 * Sox10 up vs E11.5 * Ctrl
10596053	Pccb	NM_025835	0.00227003	1.4092	E11.5 * Sox10 up vs E11.5 * Ctrl
10399636	Mrto4	NM_023536	3.28356e-005	1.40911	E11.5 * Sox10 up vs E11.5 * Ctrl
10548745	Gpr19	NM_008157	0.000299781	1.40896	E11.5 * Sox10 up vs E11.5 * Ctrl
10474073	C230071H18Rik	BC147668	0.000211031	1.40836	E11.5 * Sox10 up vs E11.5 * Ctrl
10434577	Vps8	NM_001081366	1.00218e-008	1.40832	E11.5 * Sox10 up vs E11.5 * Ctrl
10527148	Slc29a4	NM_146257	1.12758e-005	1.40817	E11.5 * Sox10 up vs E11.5 * Ctrl
10353117	Slco5a1	NM_172841	0.00019076	1.40795	E11.5 * Sox10 up vs E11.5 * Ctrl
10390283	Cdk5rap3	NM_030248	0.000814447	1.40794	E11.5 * Sox10 up vs E11.5 * Ctrl
10571705	Irf2	NM_008391	0.00109828	1.40731	E11.5 * Sox10 up vs E11.5 * Ctrl
10433672	Rrn3	NM_001039521	4.76946e-006	1.40729	E11.5 * Sox10 up vs E11.5 * Ctrl

10473737	Mtch2	NM_019758	1.24142e-005	1.40665	E11.5 * Sox10 up vs E11.5 * Ctrl
10373113	Kif5a	NM_008447	0.003219	1.40637	E11.5 * Sox10 up vs E11.5 * Ctrl
10599232	Nkap	NM_025937	0.00187744	1.40635	E11.5 * Sox10 up vs E11.5 * Ctrl
10433971	Rimbp3	NM_001033338	0.0011075	1.40627	E11.5 * Sox10 up vs E11.5 * Ctrl
10387111	2310004I24Rik	NM_025510	0.000243582	1.40611	E11.5 * Sox10 up vs E11.5 * Ctrl
10486469	Vps39	NM_147153	4.49623e-005	1.40599	E11.5 * Sox10 up vs E11.5 * Ctrl
10601701	Tmem35	NM_026239	0.000498155	1.40578	E11.5 * Sox10 up vs E11.5 * Ctrl
10578916	Sc4mol	NM_025436	0.00136561	1.40577	E11.5 * Sox10 up vs E11.5 * Ctrl
10536216	Gng11	NM_025331	0.0011621	1.40532	E11.5 * Sox10 up vs E11.5 * Ctrl
10535273	Gna12	NM_010302	0.000822972	1.40527	E11.5 * Sox10 up vs E11.5 * Ctrl
10406941	Sgtb	NM_144838	0.00615208	1.40511	E11.5 * Sox10 up vs E11.5 * Ctrl
10404928	C78339	NM_001033192	1.58694e-006	1.40445	E11.5 * Sox10 up vs E11.5 * Ctrl
10447490	Pja2	NM_001025309	3.7346e-007	1.40443	E11.5 * Sox10 up vs E11.5 * Ctrl
10398972	Mta1	NM_054081	0.000785374	1.4041	E11.5 * Sox10 up vs E11.5 * Ctrl
10558723	Psmd13	NM_011875	0.00108451	1.40336	E11.5 * Sox10 up vs E11.5 * Ctrl
10501121	Fam40a	NM_153563	0.000149426	1.40335	E11.5 * Sox10 up vs E11.5 * Ctrl
10377215	Gas7	NM_008088	6.08289e-006	1.40311	E11.5 * Sox10 up vs E11.5 * Ctrl
10557705	Phkg2	NM_026888	3.42984e-005	1.40302	E11.5 * Sox10 up vs E11.5 * Ctrl
10468990	Parl	NM_001005767	0.001023	1.40283	E11.5 * Sox10 up vs E11.5 * Ctrl
10376017	ENSMUSG0000C	ENSMUST00000(	0.00897854	1.40277	E11.5 * Sox10 up vs E11.5 * Ctrl
10530319	Atp8a1	NM_001038999	3.48462e-007	1.40261	E11.5 * Sox10 up vs E11.5 * Ctrl
10592336	Spa17	NM_011449	0.00605198	1.40217	E11.5 * Sox10 up vs E11.5 * Ctrl
10487476	1500011K16Rik	ENSMUST00000(	0.000510457	1.40209	E11.5 * Sox10 up vs E11.5 * Ctrl
10365729	Pctk2	NM_146239	1.44955e-006	1.4019	E11.5 * Sox10 up vs E11.5 * Ctrl
10563602	Saa4	NM_011316	0.000543894	1.40187	E11.5 * Sox10 up vs E11.5 * Ctrl
10481830	Zbtb43	NM_027947	0.00206075	1.40161	E11.5 * Sox10 up vs E11.5 * Ctrl
10392347	Pitpnc1	NM_145823	1.87257e-006	1.40128	E11.5 * Sox10 up vs E11.5 * Ctrl
10430748	Rangap1	NM_011241	0.00114229	1.40114	E11.5 * Sox10 up vs E11.5 * Ctrl
10602401	Fgd1	NM_008001	0.000136023	1.40113	E11.5 * Sox10 up vs E11.5 * Ctrl
10557420	Tufm	NM_172745	0.00493656	1.40079	E11.5 * Sox10 up vs E11.5 * Ctrl
10376019	OTTMUSG00000	XM_621250	0.0094222	1.40035	E11.5 * Sox10 up vs E11.5 * Ctrl
10462587	Stambpl1	NM_029682	0.00457835	1.39985	E11.5 * Sox10 up vs E11.5 * Ctrl
10475247	Tmem62	NM_175285	0.00919512	1.3995	E11.5 * Sox10 up vs E11.5 * Ctrl
10360580	Tfb2m	NM_008249	7.50536e-005	1.39908	E11.5 * Sox10 up vs E11.5 * Ctrl
10472095	Bloc1s2	BC065806	0.000347451	1.39889	E11.5 * Sox10 up vs E11.5 * Ctrl
10571214	Rnf122	NM_175136	0.00129446	1.39873	E11.5 * Sox10 up vs E11.5 * Ctrl
10407222	Dhx29	NM_172594	7.0424e-005	1.39856	E11.5 * Sox10 up vs E11.5 * Ctrl
10507203	Atpaf1	NM_181040	0.000326596	1.3985	E11.5 * Sox10 up vs E11.5 * Ctrl
10581625	2400003C14Rik	BC039052	0.000152295	1.39847	E11.5 * Sox10 up vs E11.5 * Ctrl
10344895	EG665262	XR_032183	0.000135117	1.39836	E11.5 * Sox10 up vs E11.5 * Ctrl
10537157	2310005E10Rik	NM_172398	5.16895e-005	1.39816	E11.5 * Sox10 up vs E11.5 * Ctrl
10364455	Cdc34	NM_177613	0.000552395	1.39745	E11.5 * Sox10 up vs E11.5 * Ctrl
10510254	Fv1	NM_010244	0.00119931	1.39731	E11.5 * Sox10 up vs E11.5 * Ctrl
10599562	1100001E04Rik	NM_001081123	0.0014661	1.39713	E11.5 * Sox10 up vs E11.5 * Ctrl
10558948	Cd151	NM_009842	0.00232486	1.39663	E11.5 * Sox10 up vs E11.5 * Ctrl
10412909	Fdft1	NM_010191	0.00139791	1.39647	E11.5 * Sox10 up vs E11.5 * Ctrl
10371319	ENSMUSG0000C	ENSMUST00000(	0.00879094	1.39631	E11.5 * Sox10 up vs E11.5 * Ctrl
10476893	Gzf1	NM_028986	0.00356606	1.39606	E11.5 * Sox10 up vs E11.5 * Ctrl
10442691	Clcn7	NM_011930	0.0047274	1.39565	E11.5 * Sox10 up vs E11.5 * Ctrl
10423293	Myo10	NM_019472	0.000364893	1.39551	E11.5 * Sox10 up vs E11.5 * Ctrl
10382912	09-Sep	NM_001113486	3.03696e-005	1.3954	E11.5 * Sox10 up vs E11.5 * Ctrl
10533869	Ccdc92	NM_144819	0.000902999	1.39536	E11.5 * Sox10 up vs E11.5 * Ctrl
10396064	Txndc9	NM_172054	0.000154678	1.39511	E11.5 * Sox10 up vs E11.5 * Ctrl
10364293	Ube2g2	NM_019803	0.00339641	1.3947	E11.5 * Sox10 up vs E11.5 * Ctrl
10544148	Jhdm1d	NM_001033430	0.0011516	1.39464	E11.5 * Sox10 up vs E11.5 * Ctrl
10398936	Pacs2	NM_001081170	0.000757398	1.39459	E11.5 * Sox10 up vs E11.5 * Ctrl
10554233	Aen	NM_026531	0.00573986	1.39449	E11.5 * Sox10 up vs E11.5 * Ctrl
10371396	Mterfd3	NM_028832	0.00977121	1.39429	E11.5 * Sox10 up vs E11.5 * Ctrl
10362922	Atg5	NM_053069	6.36363e-005	1.39411	E11.5 * Sox10 up vs E11.5 * Ctrl
10463704	As3mt	NM_020577	9.93546e-006	1.39402	E11.5 * Sox10 up vs E11.5 * Ctrl
10408168	Abt1	NM_013924	0.000946353	1.39391	E11.5 * Sox10 up vs E11.5 * Ctrl
10373702	Pisd-ps1	NR_003517	0.00561797	1.39379	E11.5 * Sox10 up vs E11.5 * Ctrl
10555205	Gdpd5	NM_201352	0.0012198	1.39372	E11.5 * Sox10 up vs E11.5 * Ctrl
10400165	Tfb2m	NM_008249	0.000141809	1.39343	E11.5 * Sox10 up vs E11.5 * Ctrl
10549552	Prpf31	NM_027328	0.000143187	1.39318	E11.5 * Sox10 up vs E11.5 * Ctrl
10527133	Wipi2	NM_178398	0.000338247	1.39282	E11.5 * Sox10 up vs E11.5 * Ctrl

10543697	Zc3hc1	NM_172735	0.00255028	1.39251	E11.5 * Sox10 up vs E11.5 * Ctrl
10429908	Bop1	NM_013481	0.00058764	1.39246	E11.5 * Sox10 up vs E11.5 * Ctrl
10393926	Dcxr	NM_026428	0.00110838	1.39222	E11.5 * Sox10 up vs E11.5 * Ctrl
10404531	Psmg4	NM_001101430	0.00195466	1.3919	E11.5 * Sox10 up vs E11.5 * Ctrl
10453887	Cables1	NM_001146287	4.0518e-005	1.39181	E11.5 * Sox10 up vs E11.5 * Ctrl
10602474	Phf8	NM_001113354	0.00136289	1.39167	E11.5 * Sox10 up vs E11.5 * Ctrl
10582262	Klhdc4	NM_145605	0.00494463	1.39122	E11.5 * Sox10 up vs E11.5 * Ctrl
10587627	Cyb5r4	NM_024195	4.13907e-005	1.39096	E11.5 * Sox10 up vs E11.5 * Ctrl
10452918	Strn	NM_011500	1.69442e-005	1.39095	E11.5 * Sox10 up vs E11.5 * Ctrl
10437174	Wrb	NM_207301	0.00150838	1.39079	E11.5 * Sox10 up vs E11.5 * Ctrl
10565910	Plekhb1	NM_013746	0.0030448	1.39036	E11.5 * Sox10 up vs E11.5 * Ctrl
10365601	Gnptab	NM_001004164	3.8104e-005	1.3903	E11.5 * Sox10 up vs E11.5 * Ctrl
10434384	Ap2m1	NM_009679	0.0036249	1.38962	E11.5 * Sox10 up vs E11.5 * Ctrl
10579554	Unc13a	NM_001029873	0.000224457	1.38883	E11.5 * Sox10 up vs E11.5 * Ctrl
10540085	Fbln2	NM_007992	0.00138515	1.38879	E11.5 * Sox10 up vs E11.5 * Ctrl
10380381	Tob1	NM_009427	0.000548845	1.38874	E11.5 * Sox10 up vs E11.5 * Ctrl
10578984	Tufm	NM_172745	0.00413671	1.38872	E11.5 * Sox10 up vs E11.5 * Ctrl
10372668	Mdm2	NM_010786	3.62861e-005	1.38864	E11.5 * Sox10 up vs E11.5 * Ctrl
10581737	Ddx19b	NM_172284	0.000256178	1.38819	E11.5 * Sox10 up vs E11.5 * Ctrl
10384961	Stc2	NM_011491	0.00320334	1.38815	E11.5 * Sox10 up vs E11.5 * Ctrl
10460573	Eif1ad	NM_027236	3.73756e-005	1.38793	E11.5 * Sox10 up vs E11.5 * Ctrl
10394791	LOC236260	ENSMUST00000(	0.00795087	1.38785	E11.5 * Sox10 up vs E11.5 * Ctrl
10399379	Pgk1	NM_008828	3.8192e-009	1.38773	E11.5 * Sox10 up vs E11.5 * Ctrl
10498313	Pgk1	NM_008828	3.8192e-009	1.38773	E11.5 * Sox10 up vs E11.5 * Ctrl
10415980	Fbxo16	NM_015795	0.000684385	1.38731	E11.5 * Sox10 up vs E11.5 * Ctrl
10472989	Hoxd1	NM_010467	0.00719797	1.38722	E11.5 * Sox10 up vs E11.5 * Ctrl
10372497	Thap2	NM_025780	0.00362288	1.38716	E11.5 * Sox10 up vs E11.5 * Ctrl
10573566	Dhps	NM_001039514	7.60487e-006	1.38696	E11.5 * Sox10 up vs E11.5 * Ctrl
10529041	Preb	NM_016703	0.00301445	1.38677	E11.5 * Sox10 up vs E11.5 * Ctrl
10571907	Mfap3l	NM_027756	0.000394153	1.38671	E11.5 * Sox10 up vs E11.5 * Ctrl
10501244	Ampd2	NM_028779	0.000372659	1.38656	E11.5 * Sox10 up vs E11.5 * Ctrl
10529549	Tbc1d14	NM_001113362	3.45184e-005	1.38632	E11.5 * Sox10 up vs E11.5 * Ctrl
10456836	St8sia5	NM_153124	0.000320282	1.38575	E11.5 * Sox10 up vs E11.5 * Ctrl
10425293	Cby1	NM_028634	1.01138e-005	1.38574	E11.5 * Sox10 up vs E11.5 * Ctrl
10395538	Pnpla8	NM_026164	1.10266e-005	1.3854	E11.5 * Sox10 up vs E11.5 * Ctrl
10386604	Shmt1	NM_009171	0.00109611	1.38536	E11.5 * Sox10 up vs E11.5 * Ctrl
10381697	Hexim1	NM_138753	3.96301e-005	1.38525	E11.5 * Sox10 up vs E11.5 * Ctrl
10432362	Rhebl1	NM_026967	0.00684605	1.3851	E11.5 * Sox10 up vs E11.5 * Ctrl
10446739	Clip4	NM_030179	0.000701851	1.38498	E11.5 * Sox10 up vs E11.5 * Ctrl
10522250	Tmem33	NM_028975	3.87862e-005	1.38388	E11.5 * Sox10 up vs E11.5 * Ctrl
10472136	Galnt13	NM_173030	0.00194691	1.38349	E11.5 * Sox10 up vs E11.5 * Ctrl
10457536	Ankrd29	ENSMUST00000	0.000675122	1.38333	E11.5 * Sox10 up vs E11.5 * Ctrl
10361799	Adat2	NM_025748	0.000336034	1.38328	E11.5 * Sox10 up vs E11.5 * Ctrl
10376033	Kif3a	NM_008443	5.79022e-007	1.38315	E11.5 * Sox10 up vs E11.5 * Ctrl
10533929	Scarb1	NM_016741	0.00245063	1.38315	E11.5 * Sox10 up vs E11.5 * Ctrl
10577508	Ckap2	NM_001004140	0.000533795	1.38286	E11.5 * Sox10 up vs E11.5 * Ctrl
10529344	Poln	NM_181857	1.94594e-005	1.38279	E11.5 * Sox10 up vs E11.5 * Ctrl
10576844	BC068157	NM_207203	0.000425252	1.38264	E11.5 * Sox10 up vs E11.5 * Ctrl
10480672	Ssna1	NM_023464	0.00488397	1.38216	E11.5 * Sox10 up vs E11.5 * Ctrl
10593205	Rexo2	NM_024233	4.43103e-006	1.38193	E11.5 * Sox10 up vs E11.5 * Ctrl
10510176	OTTMUSG00000	NM_001039233	0.000157978	1.38189	E11.5 * Sox10 up vs E11.5 * Ctrl
10378649	Slc43a2	NM_173388	0.000353792	1.38148	E11.5 * Sox10 up vs E11.5 * Ctrl
10402715	Bag5	NM_027404	2.8775e-005	1.38133	E11.5 * Sox10 up vs E11.5 * Ctrl
10398727	Klc1	NM_001025360	0.000580816	1.38087	E11.5 * Sox10 up vs E11.5 * Ctrl
10362490	Tspyl4	NM_030203	0.000253101	1.38074	E11.5 * Sox10 up vs E11.5 * Ctrl
10529689	Wdr1	NM_011715	0.000282368	1.38068	E11.5 * Sox10 up vs E11.5 * Ctrl
10428407	Tmem74	NM_175502	0.0015381	1.38065	E11.5 * Sox10 up vs E11.5 * Ctrl
10571344	D8Ertd82e	NM_172911	0.000615139	1.38065	E11.5 * Sox10 up vs E11.5 * Ctrl
10476702	Sec23b	NM_019787	2.06468e-006	1.38061	E11.5 * Sox10 up vs E11.5 * Ctrl
10409222	Shc3	NM_009167	0.000295977	1.38041	E11.5 * Sox10 up vs E11.5 * Ctrl
10359235	Rasal2	NM_177644	9.99881e-007	1.38032	E11.5 * Sox10 up vs E11.5 * Ctrl
10358662	Hmcn1	NM_001024720	0.000386347	1.38017	E11.5 * Sox10 up vs E11.5 * Ctrl
10540911	Tsen2	NM_199033	0.000320067	1.38011	E11.5 * Sox10 up vs E11.5 * Ctrl
10555108	Clns1a	NM_023671	2.74943e-007	1.38001	E11.5 * Sox10 up vs E11.5 * Ctrl
10469020	Bend7	NM_178663	8.7455e-006	1.37948	E11.5 * Sox10 up vs E11.5 * Ctrl
10500683	Rangrf	NM_021329	0.00334102	1.37929	E11.5 * Sox10 up vs E11.5 * Ctrl

10558248	Bub3	NM_009774	7.86213e-007	1.37922	E11.5 * Sox10 up vs E11.5 * Ctrl
10347232	Xrcc5	NM_009533	8.83854e-006	1.3791	E11.5 * Sox10 up vs E11.5 * Ctrl
10589004	Qars	NM_133794	3.14412e-005	1.37896	E11.5 * Sox10 up vs E11.5 * Ctrl
10369630	Ddx21	NM_019553	9.67438e-005	1.37886	E11.5 * Sox10 up vs E11.5 * Ctrl
10474977	1500003O03Rik	NM_019769	1.54473e-005	1.37885	E11.5 * Sox10 up vs E11.5 * Ctrl
10569890	Aida	NM_181732	0.000796587	1.3788	E11.5 * Sox10 up vs E11.5 * Ctrl
10460108	Gnpnat1	NM_019425	0.000338721	1.37869	E11.5 * Sox10 up vs E11.5 * Ctrl
10499438	Msto1	NM_144898	0.00629068	1.37868	E11.5 * Sox10 up vs E11.5 * Ctrl
10403076		---	0.000119784	1.37834	E11.5 * Sox10 up vs E11.5 * Ctrl
10506269	Ak3l1	NM_009647	0.00144181	1.37829	E11.5 * Sox10 up vs E11.5 * Ctrl
10512291	Dctn3	NM_016890	1.79991e-005	1.37818	E11.5 * Sox10 up vs E11.5 * Ctrl
10426093	Zbed4	NM_181412	1.46915e-005	1.37804	E11.5 * Sox10 up vs E11.5 * Ctrl
10450622	2610110G12Rik	NM_001142744	0.00637825	1.37796	E11.5 * Sox10 up vs E11.5 * Ctrl
10581266	Tppp3	NM_026481	0.000445452	1.37795	E11.5 * Sox10 up vs E11.5 * Ctrl
10470322	Surf2	NM_013678	9.42111e-006	1.37791	E11.5 * Sox10 up vs E11.5 * Ctrl
10416640	Mtrf1	NM_145960	0.00145464	1.37762	E11.5 * Sox10 up vs E11.5 * Ctrl
10358629	Hmcn1	NM_001024720	0.00632728	1.37744	E11.5 * Sox10 up vs E11.5 * Ctrl
10373571	1110012D08Rik	BC037624	0.00113308	1.37737	E11.5 * Sox10 up vs E11.5 * Ctrl
10505894	Mtap	NM_024433	2.90134e-005	1.37732	E11.5 * Sox10 up vs E11.5 * Ctrl
10353899	Sema4c	NM_001126047	0.000668683	1.37722	E11.5 * Sox10 up vs E11.5 * Ctrl
10496417	Rg9mtd2	NM_175389	0.000553556	1.37714	E11.5 * Sox10 up vs E11.5 * Ctrl
10439218	Pdia5	NM_028295	5.97103e-006	1.37702	E11.5 * Sox10 up vs E11.5 * Ctrl
10417787	Gng2	NM_010315	1.30293e-005	1.37679	E11.5 * Sox10 up vs E11.5 * Ctrl
10488687	Pdrg1	NM_178939	6.7129e-005	1.37636	E11.5 * Sox10 up vs E11.5 * Ctrl
10528038	Adam22	NM_001007220	0.000104676	1.37618	E11.5 * Sox10 up vs E11.5 * Ctrl
10557156	Plk1	NM_011121	0.000545892	1.37582	E11.5 * Sox10 up vs E11.5 * Ctrl
10399430	Ddx1	NM_134040	2.2081e-007	1.37559	E11.5 * Sox10 up vs E11.5 * Ctrl
10583942	Thyn1	NM_144543	4.0882e-006	1.37546	E11.5 * Sox10 up vs E11.5 * Ctrl
10370471	Agpat3	NM_053014	6.99502e-006	1.37519	E11.5 * Sox10 up vs E11.5 * Ctrl
10445338	Enpp5	NM_032003	0.00137384	1.37487	E11.5 * Sox10 up vs E11.5 * Ctrl
10368918	Sobp	NM_175407	5.87447e-005	1.37477	E11.5 * Sox10 up vs E11.5 * Ctrl
10580469	Cbln1	NM_019626	0.00115279	1.37461	E11.5 * Sox10 up vs E11.5 * Ctrl
10459241	Afap1l1	NM_178928	0.000203603	1.37444	E11.5 * Sox10 up vs E11.5 * Ctrl
10404941	Aof1	NM_172262	0.000339661	1.37421	E11.5 * Sox10 up vs E11.5 * Ctrl
10416899	Ndfip2	NM_029561	9.09111e-005	1.37419	E11.5 * Sox10 up vs E11.5 * Ctrl
10529656	Nsg1	NM_010942	5.09181e-005	1.37408	E11.5 * Sox10 up vs E11.5 * Ctrl
10397450	Vash1	NM_177354	0.00045447	1.37403	E11.5 * Sox10 up vs E11.5 * Ctrl
10402318	Prima1	NM_133364	0.00717224	1.37384	E11.5 * Sox10 up vs E11.5 * Ctrl
10416510	Nufip1	NM_013745	0.000313832	1.37337	E11.5 * Sox10 up vs E11.5 * Ctrl
10544462	Fam115a	NM_029930	0.000199738	1.37328	E11.5 * Sox10 up vs E11.5 * Ctrl
10516051	Cap1	NM_007598	0.0102638	1.37322	E11.5 * Sox10 up vs E11.5 * Ctrl
10519693	Sema3d	NM_028882	0.00876897	1.37292	E11.5 * Sox10 up vs E11.5 * Ctrl
10492522	Schip1	NM_001113421	0.000113541	1.37224	E11.5 * Sox10 up vs E11.5 * Ctrl
10369885	Cisd1	NM_134007	0.00363913	1.37217	E11.5 * Sox10 up vs E11.5 * Ctrl
10517706	Mrto4	NM_023536	1.33998e-005	1.37203	E11.5 * Sox10 up vs E11.5 * Ctrl
10476005	A730036I17Rik	ENSMUST00000(	0.00217446	1.37165	E11.5 * Sox10 up vs E11.5 * Ctrl
10345357	Imp4	NM_178601	0.00019277	1.37161	E11.5 * Sox10 up vs E11.5 * Ctrl
10560304	Calm3	NM_007590	0.000134387	1.37161	E11.5 * Sox10 up vs E11.5 * Ctrl
10564313	Mphosph10	NM_026483	0.000211413	1.37093	E11.5 * Sox10 up vs E11.5 * Ctrl
10456357	Pmaip1	NM_021451	0.00464721	1.37081	E11.5 * Sox10 up vs E11.5 * Ctrl
10432256	Arf3	NM_007478	0.000125741	1.37052	E11.5 * Sox10 up vs E11.5 * Ctrl
10383556	Fn3krp	NM_181420	0.00630525	1.37022	E11.5 * Sox10 up vs E11.5 * Ctrl
10529957	Gpr125	ENSMUST00000(	4.52247e-005	1.36989	E11.5 * Sox10 up vs E11.5 * Ctrl
10581560	Psmd7	NM_010817	1.0204e-005	1.36949	E11.5 * Sox10 up vs E11.5 * Ctrl
10553163	Nomo1	NM_153057	0.000745038	1.36915	E11.5 * Sox10 up vs E11.5 * Ctrl
10485830	Tmem85	NM_026519	0.000939589	1.36912	E11.5 * Sox10 up vs E11.5 * Ctrl
10504743	Nans	NM_053179	0.000656396	1.36887	E11.5 * Sox10 up vs E11.5 * Ctrl
10585699	Fabp5	NM_010634	2.12097e-005	1.36836	E11.5 * Sox10 up vs E11.5 * Ctrl
10375730		---	0.00319936	1.368	E11.5 * Sox10 up vs E11.5 * Ctrl
10467230	Ide	NM_031156	0.000247816	1.36769	E11.5 * Sox10 up vs E11.5 * Ctrl
10585417	Idh3a	NM_029573	0.00103709	1.36768	E11.5 * Sox10 up vs E11.5 * Ctrl
10545780	Exoc6b	NM_177077	3.5522e-005	1.36759	E11.5 * Sox10 up vs E11.5 * Ctrl
10415021	Abhd4	NM_134076	8.65387e-005	1.36743	E11.5 * Sox10 up vs E11.5 * Ctrl
10367582	Vip	NM_011702	0.00927614	1.36729	E11.5 * Sox10 up vs E11.5 * Ctrl
10531290	Rchy1	NM_026557	0.00593932	1.36713	E11.5 * Sox10 up vs E11.5 * Ctrl
10454735	Fam53c	BC057111	0.00326934	1.36644	E11.5 * Sox10 up vs E11.5 * Ctrl

10523058	Eif5a	NM_181582	0.00189152	1.36621	E11.5 * Sox10 up vs E11.5 * Ctrl
10344624	Lypla1	NM_008866	0.000756039	1.36615	E11.5 * Sox10 up vs E11.5 * Ctrl
10485624	Prrg4	NM_178695	0.00209573	1.36604	E11.5 * Sox10 up vs E11.5 * Ctrl
10510286	Mad2l2	NM_027985	0.000189889	1.3658	E11.5 * Sox10 up vs E11.5 * Ctrl
10528546	Gabarapl2	NM_026693	0.000143336	1.36579	E11.5 * Sox10 up vs E11.5 * Ctrl
10559261	Cd81	NM_133655	6.63135e-006	1.36575	E11.5 * Sox10 up vs E11.5 * Ctrl
10534456	Hip1	NM_146001	0.000765562	1.36546	E11.5 * Sox10 up vs E11.5 * Ctrl
10345025	Iars	NM_172015	0.000784129	1.36507	E11.5 * Sox10 up vs E11.5 * Ctrl
10548163	Tulp3	NM_011657	0.000282779	1.36496	E11.5 * Sox10 up vs E11.5 * Ctrl
10375485		---	3.57885e-005	1.36352	E11.5 * Sox10 up vs E11.5 * Ctrl
10515943	Ctps	NM_016748	3.0215e-005	1.36347	E11.5 * Sox10 up vs E11.5 * Ctrl
10467749	Crtac1	NM_145123	1.4183e-005	1.36339	E11.5 * Sox10 up vs E11.5 * Ctrl
10576305	Tcf25	NM_001037878	5.79537e-005	1.36329	E11.5 * Sox10 up vs E11.5 * Ctrl
10438517	Alg3	NM_145939	8.70976e-005	1.36292	E11.5 * Sox10 up vs E11.5 * Ctrl
10517328	Tmem50a	NM_027935	8.09757e-007	1.36288	E11.5 * Sox10 up vs E11.5 * Ctrl
10535852	Slc7a1	NM_007513	0.000254945	1.3628	E11.5 * Sox10 up vs E11.5 * Ctrl
10554588	Sh3gl3	NM_017400	0.00752172	1.36278	E11.5 * Sox10 up vs E11.5 * Ctrl
10544837	1200009O22Rik	BC043099	6.43228e-005	1.36268	E11.5 * Sox10 up vs E11.5 * Ctrl
10590983	Panx1	NM_019482	0.000242857	1.36267	E11.5 * Sox10 up vs E11.5 * Ctrl
10526853	Fam20c	NM_030565	0.00508475	1.36266	E11.5 * Sox10 up vs E11.5 * Ctrl
10562578	Pop4	NM_025390	0.000470373	1.36229	E11.5 * Sox10 up vs E11.5 * Ctrl
10489065	Ndrg3	NM_013865	3.83866e-005	1.36161	E11.5 * Sox10 up vs E11.5 * Ctrl
10384691	0610010F05Rik	NM_027860	9.26106e-005	1.36119	E11.5 * Sox10 up vs E11.5 * Ctrl
10392856	Nat9	NM_025400	0.00507826	1.36113	E11.5 * Sox10 up vs E11.5 * Ctrl
10425461	Adsl	NM_009634	1.24255e-005	1.36088	E11.5 * Sox10 up vs E11.5 * Ctrl
10556528	Pde3b	NM_011055	0.00059195	1.36082	E11.5 * Sox10 up vs E11.5 * Ctrl
10406067	Clptm1l	NM_146047	0.00260084	1.36068	E11.5 * Sox10 up vs E11.5 * Ctrl
10555550	Clpb	NM_009191	9.41349e-006	1.36062	E11.5 * Sox10 up vs E11.5 * Ctrl
10549057	Recql	NM_023042	0.000444	1.36037	E11.5 * Sox10 up vs E11.5 * Ctrl
10491551	Exosc9	NM_019393	0.000437048	1.35995	E11.5 * Sox10 up vs E11.5 * Ctrl
10528507	Pus7	NM_178403	8.56929e-005	1.35973	E11.5 * Sox10 up vs E11.5 * Ctrl
10473097	Plekha3	NM_031256	0.0001757	1.35972	E11.5 * Sox10 up vs E11.5 * Ctrl
10476725	Gm561	NM_001033297	0.00894411	1.35946	E11.5 * Sox10 up vs E11.5 * Ctrl
10535184	Psmg3	NM_025604	0.00110653	1.35941	E11.5 * Sox10 up vs E11.5 * Ctrl
10445909	Kat2b	NM_020005	0.000112263	1.35916	E11.5 * Sox10 up vs E11.5 * Ctrl
10403765	Vps41	NM_172120	1.76638e-005	1.35895	E11.5 * Sox10 up vs E11.5 * Ctrl
10384398	Grb10	NM_010345	0.000109025	1.35877	E11.5 * Sox10 up vs E11.5 * Ctrl
10434629	Map3k13	NM_172821	0.00200212	1.35832	E11.5 * Sox10 up vs E11.5 * Ctrl
10494007	Them4	NM_029431	0.00421009	1.35822	E11.5 * Sox10 up vs E11.5 * Ctrl
10419049	Nrg3	NM_008734	0.00957154	1.35786	E11.5 * Sox10 up vs E11.5 * Ctrl
10491385	Actl6a	NM_019673	2.83729e-005	1.35771	E11.5 * Sox10 up vs E11.5 * Ctrl
10381260	Tubg2	NM_134028	0.000145434	1.35761	E11.5 * Sox10 up vs E11.5 * Ctrl
10355916	Pax3	NM_008781	4.39961e-005	1.35736	E11.5 * Sox10 up vs E11.5 * Ctrl
10433735	Abcc1	NM_008576	0.000384894	1.35706	E11.5 * Sox10 up vs E11.5 * Ctrl
10537102	Exoc4	NM_009148	5.59911e-005	1.35704	E11.5 * Sox10 up vs E11.5 * Ctrl
10463254	D19Ertd386e	NM_177464	7.72304e-005	1.35703	E11.5 * Sox10 up vs E11.5 * Ctrl
10401172	Vti1b	NM_016800	1.14719e-006	1.35699	E11.5 * Sox10 up vs E11.5 * Ctrl
10409737	Agtpbp1	NM_023328	3.0879e-005	1.35686	E11.5 * Sox10 up vs E11.5 * Ctrl
10419170	Txndc16	NM_172597	0.00353088	1.35641	E11.5 * Sox10 up vs E11.5 * Ctrl
10458906	Ppic	NM_008908	4.64428e-007	1.35611	E11.5 * Sox10 up vs E11.5 * Ctrl
10374119	Ogdh	NM_010956	0.00122067	1.35602	E11.5 * Sox10 up vs E11.5 * Ctrl
10368484	ENSMUSG0000C	ENSMUST00000(	0.000183766	1.35548	E11.5 * Sox10 up vs E11.5 * Ctrl
10400006	Ahr	NM_013464	0.000133361	1.35541	E11.5 * Sox10 up vs E11.5 * Ctrl
10394498	Wdr35	NM_172470	0.00064863	1.35528	E11.5 * Sox10 up vs E11.5 * Ctrl
10413461	Erc2	NM_177814	0.00347577	1.35504	E11.5 * Sox10 up vs E11.5 * Ctrl
10400395	Ppp2r3c	NM_021529	0.00932606	1.35453	E11.5 * Sox10 up vs E11.5 * Ctrl
10352242	Parp1	NM_007415	0.00030925	1.35443	E11.5 * Sox10 up vs E11.5 * Ctrl
10407742	Actn2	NM_033268	1.71918e-005	1.35422	E11.5 * Sox10 up vs E11.5 * Ctrl
10551881	0610010E21Rik	NM_001033140	0.000240859	1.35417	E11.5 * Sox10 up vs E11.5 * Ctrl
10527233	Cyth3	NM_011182	1.63308e-005	1.35399	E11.5 * Sox10 up vs E11.5 * Ctrl
10512640	Gne	NM_015828	5.104e-005	1.35372	E11.5 * Sox10 up vs E11.5 * Ctrl
10471535	Fam129b	NM_146119	0.000418943	1.35366	E11.5 * Sox10 up vs E11.5 * Ctrl
10514658	Itgb3bp	ENSMUST00000(	2.1749e-005	1.35361	E11.5 * Sox10 up vs E11.5 * Ctrl
10428222	Ncald	NM_134094	0.000109465	1.35341	E11.5 * Sox10 up vs E11.5 * Ctrl
10478424	Ywhab	NM_018753	9.82161e-008	1.35337	E11.5 * Sox10 up vs E11.5 * Ctrl
10415844	Ctsb	NM_007798	0.000553983	1.35333	E11.5 * Sox10 up vs E11.5 * Ctrl

10457359	Mpp7	NM_001081287	0.0016993	1.35328	E11.5 * Sox10 up vs E11.5 * Ctrl
10571384	Efha2	ENSMUST00000(	0.00120799	1.35299	E11.5 * Sox10 up vs E11.5 * Ctrl
10409365	Gprin1	NM_012014	0.000113532	1.35287	E11.5 * Sox10 up vs E11.5 * Ctrl
10583788	Prkcsh	NM_008925	0.00308679	1.35284	E11.5 * Sox10 up vs E11.5 * Ctrl
10561527	Actn4	NM_021895	0.00196273	1.3526	E11.5 * Sox10 up vs E11.5 * Ctrl
10457546	Osbpl1a	NM_207530	4.12206e-005	1.35223	E11.5 * Sox10 up vs E11.5 * Ctrl
10515396	9530048O09Rik	NR_024078	0.00829125	1.35213	E11.5 * Sox10 up vs E11.5 * Ctrl
10521440	Afap1	NM_027373	0.000124668	1.3521	E11.5 * Sox10 up vs E11.5 * Ctrl
10530806	Ppat	BC023841	1.37541e-006	1.35204	E11.5 * Sox10 up vs E11.5 * Ctrl
10386789	Ulk2	NM_013881	5.60043e-007	1.35182	E11.5 * Sox10 up vs E11.5 * Ctrl
10555570	Phox2a	NM_008887	0.0053049	1.35175	E11.5 * Sox10 up vs E11.5 * Ctrl
10437712	Zc3h7a	NM_145931	0.000424368	1.35159	E11.5 * Sox10 up vs E11.5 * Ctrl
10583929	B3gat1	NM_029792	0.00029928	1.35127	E11.5 * Sox10 up vs E11.5 * Ctrl
10428232	Rrm2b	NM_199476	0.000101247	1.35026	E11.5 * Sox10 up vs E11.5 * Ctrl
10592850	Trappc4	NM_021789	0.00186832	1.35023	E11.5 * Sox10 up vs E11.5 * Ctrl
10498837	Etfdh	NM_025794	1.75392e-007	1.34992	E11.5 * Sox10 up vs E11.5 * Ctrl
10509163	Id3	NM_008321	0.00290842	1.34977	E11.5 * Sox10 up vs E11.5 * Ctrl
10474112	Traf6	NM_009424	0.000204495	1.34975	E11.5 * Sox10 up vs E11.5 * Ctrl
10530851	Mtch2	NM_019758	0.00021913	1.34975	E11.5 * Sox10 up vs E11.5 * Ctrl
10517263	Fam54b	BC026808	5.55715e-005	1.34953	E11.5 * Sox10 up vs E11.5 * Ctrl
10389391	OTTMUSG00000	ENSMUST00000(	0.00791638	1.34912	E11.5 * Sox10 up vs E11.5 * Ctrl
10400126	Lrrn3	NM_010733	3.73471e-005	1.34899	E11.5 * Sox10 up vs E11.5 * Ctrl
10501649	Rtcd1	NM_025517	1.86049e-007	1.34884	E11.5 * Sox10 up vs E11.5 * Ctrl
10464045	Acsl5	NM_027976	0.000466376	1.34878	E11.5 * Sox10 up vs E11.5 * Ctrl
10555568	Rpl31	BC092139	0.00911808	1.34877	E11.5 * Sox10 up vs E11.5 * Ctrl
10467842	Got1	NM_010324	0.000106724	1.34846	E11.5 * Sox10 up vs E11.5 * Ctrl
10539653	Tprkb	NM_176842	0.000569264	1.34812	E11.5 * Sox10 up vs E11.5 * Ctrl
10459835		---	0.00606291	1.34791	E11.5 * Sox10 up vs E11.5 * Ctrl
10428157	Rnf19a	NM_013923	0.000104251	1.34777	E11.5 * Sox10 up vs E11.5 * Ctrl
10442396	Abca3	NM_013855	4.37574e-005	1.34763	E11.5 * Sox10 up vs E11.5 * Ctrl
10363599	Rufy2	NM_027425	0.000116502	1.34718	E11.5 * Sox10 up vs E11.5 * Ctrl
10436369	Filip1l	NM_030163	3.26667e-006	1.34667	E11.5 * Sox10 up vs E11.5 * Ctrl
10498775	Golim4	NM_175193	5.46045e-005	1.34652	E11.5 * Sox10 up vs E11.5 * Ctrl
10361200	AA408296	NM_145415	2.19171e-006	1.34618	E11.5 * Sox10 up vs E11.5 * Ctrl
10550638	Rtn2	NM_013648	1.48649e-005	1.34604	E11.5 * Sox10 up vs E11.5 * Ctrl
10557450	Bola2	NM_175103	3.20236e-005	1.34526	E11.5 * Sox10 up vs E11.5 * Ctrl
10457205	Crem	NM_001110856	0.00219022	1.34524	E11.5 * Sox10 up vs E11.5 * Ctrl
10478967	Cstf1	NM_024199	0.00284382	1.345	E11.5 * Sox10 up vs E11.5 * Ctrl
10384936	Acyp2	NM_029344	0.000119041	1.34492	E11.5 * Sox10 up vs E11.5 * Ctrl
10373728	Patz1	NM_019574	0.00537172	1.34474	E11.5 * Sox10 up vs E11.5 * Ctrl
10529547		---	0.000987447	1.34466	E11.5 * Sox10 up vs E11.5 * Ctrl
10580365	4921524J17Rik	NM_025722	0.00121092	1.34452	E11.5 * Sox10 up vs E11.5 * Ctrl
10368556	Hey2	NM_013904	0.00505212	1.34447	E11.5 * Sox10 up vs E11.5 * Ctrl
10392983	Slc25a19	NM_026071	0.000711046	1.34424	E11.5 * Sox10 up vs E11.5 * Ctrl
10584741	Slc37a4	NM_008063	0.00644696	1.34402	E11.5 * Sox10 up vs E11.5 * Ctrl
10346000	Gulp1	NM_028450	0.00473396	1.34354	E11.5 * Sox10 up vs E11.5 * Ctrl
10468275	Pcgf6	NM_027654	1.32532e-005	1.34352	E11.5 * Sox10 up vs E11.5 * Ctrl
10441064	Dscr3	NM_007834	0.00793463	1.34325	E11.5 * Sox10 up vs E11.5 * Ctrl
10591960	Vps26b	NM_178027	0.00306015	1.34288	E11.5 * Sox10 up vs E11.5 * Ctrl
10574033	Nup93	NM_172410	1.67477e-008	1.34267	E11.5 * Sox10 up vs E11.5 * Ctrl
10456248	Onecut2	NM_194268	0.00126798	1.34264	E11.5 * Sox10 up vs E11.5 * Ctrl
10538354	1200009O22Rik	BC043099	5.72461e-006	1.3425	E11.5 * Sox10 up vs E11.5 * Ctrl
10507529	Med8	NM_020000	0.00032852	1.34247	E11.5 * Sox10 up vs E11.5 * Ctrl
10347734	Sgpp2	NM_001004173	0.000206985	1.34214	E11.5 * Sox10 up vs E11.5 * Ctrl
10593225	Zbtb16	NM_001033324	0.00154988	1.34211	E11.5 * Sox10 up vs E11.5 * Ctrl
10493193	Cct3	NM_009836	8.92418e-005	1.34206	E11.5 * Sox10 up vs E11.5 * Ctrl
10400805	Nin	NM_008697	3.11721e-006	1.3419	E11.5 * Sox10 up vs E11.5 * Ctrl
10558910	Rangrf	NM_021329	0.0048861	1.34188	E11.5 * Sox10 up vs E11.5 * Ctrl
10574204	Arl2bp	NM_024191	5.75068e-006	1.34178	E11.5 * Sox10 up vs E11.5 * Ctrl
10570870	1700041G16Rik	ENSMUST00000(	0.00296074	1.34176	E11.5 * Sox10 up vs E11.5 * Ctrl
10592237	Ei24	NM_007915	0.00719222	1.34161	E11.5 * Sox10 up vs E11.5 * Ctrl
10352348	Cnih4	NM_030131	1.64331e-005	1.34149	E11.5 * Sox10 up vs E11.5 * Ctrl
10427877	ENSMUSG00000	AK157781	0.00657606	1.34146	E11.5 * Sox10 up vs E11.5 * Ctrl
10560408	Psg17	NM_007677	0.00707664	1.34134	E11.5 * Sox10 up vs E11.5 * Ctrl
10367413	Dnajc14	NM_028873	0.000203602	1.34074	E11.5 * Sox10 up vs E11.5 * Ctrl
10450055	H2-Ke2	NM_010385	0.000324266	1.3407	E11.5 * Sox10 up vs E11.5 * Ctrl

10601903	Zcchc18	NM_001035510	0.0104043	1.34039	E11.5 * Sox10 up vs E11.5 * Ctrl
10458183	Gfra3	NM_010280	0.00184648	1.34023	E11.5 * Sox10 up vs E11.5 * Ctrl
10470529	Olfm1	NM_019498	3.75147e-005	1.34008	E11.5 * Sox10 up vs E11.5 * Ctrl
10589494	Cspg5	NM_013884	0.00139272	1.34001	E11.5 * Sox10 up vs E11.5 * Ctrl
10397094	Psen1	NM_008943	4.03068e-005	1.34001	E11.5 * Sox10 up vs E11.5 * Ctrl
10495111	Wdr77	NM_027432	2.83449e-005	1.3399	E11.5 * Sox10 up vs E11.5 * Ctrl
10546855	Srgap3	NM_080448	7.01947e-006	1.33965	E11.5 * Sox10 up vs E11.5 * Ctrl
10533386	Sh2b3	NM_008507	0.000121331	1.33946	E11.5 * Sox10 up vs E11.5 * Ctrl
10464877	Dpp3	NM_133803	0.000848403	1.33887	E11.5 * Sox10 up vs E11.5 * Ctrl
10580309	1500041N16Rik	NM_026399	5.82517e-005	1.33883	E11.5 * Sox10 up vs E11.5 * Ctrl
10586604	Rps27l	NM_026467	0.00020516	1.33859	E11.5 * Sox10 up vs E11.5 * Ctrl
10499777	Ints3	NM_145540	0.00039354	1.33846	E11.5 * Sox10 up vs E11.5 * Ctrl
10561907	0610010E21Rik	NM_001033140	2.71188e-005	1.33841	E11.5 * Sox10 up vs E11.5 * Ctrl
10388018	Dhx33	NM_178367	0.000709473	1.33803	E11.5 * Sox10 up vs E11.5 * Ctrl
10422179	Pou4f1	NM_011143	0.00128625	1.33798	E11.5 * Sox10 up vs E11.5 * Ctrl
10514896	2210012G02Rik	NM_025617	0.00133342	1.33787	E11.5 * Sox10 up vs E11.5 * Ctrl
10430113	D15Wsu169e	BC060637	0.00330644	1.33775	E11.5 * Sox10 up vs E11.5 * Ctrl
10601390	Pgk1	NM_008828	2.40982e-008	1.33773	E11.5 * Sox10 up vs E11.5 * Ctrl
10344637	Atp6v1h	NM_133826	1.30101e-005	1.33771	E11.5 * Sox10 up vs E11.5 * Ctrl
10369301	Chst3	NM_016803	9.83815e-005	1.33765	E11.5 * Sox10 up vs E11.5 * Ctrl
10358617	Hmcn1	NM_001024720	0.00878547	1.33757	E11.5 * Sox10 up vs E11.5 * Ctrl
10349208	Cntnap5a	NM_001077425	0.00289444	1.33728	E11.5 * Sox10 up vs E11.5 * Ctrl
10369295	D10Ertd641e	NM_025514	0.000154518	1.33702	E11.5 * Sox10 up vs E11.5 * Ctrl
10453900	Riok3	NM_024182	0.000244981	1.33694	E11.5 * Sox10 up vs E11.5 * Ctrl
10544858	Chn2	NM_023543	0.00587604	1.33684	E11.5 * Sox10 up vs E11.5 * Ctrl
10593196	ENSMUSG0000(	ENSMUST00000(	0.000381958	1.33647	E11.5 * Sox10 up vs E11.5 * Ctrl
10465844	Asrgl1	NM_025610	1.70195e-005	1.33636	E11.5 * Sox10 up vs E11.5 * Ctrl
10505109	BC026590	BC026590	0.000518588	1.33618	E11.5 * Sox10 up vs E11.5 * Ctrl
10408975	Kif13a	NM_010617	0.000611496	1.33616	E11.5 * Sox10 up vs E11.5 * Ctrl
10382376	Ttyh2	NM_053273	0.00228858	1.33613	E11.5 * Sox10 up vs E11.5 * Ctrl
10536541	St7	NM_022332	0.00046301	1.33611	E11.5 * Sox10 up vs E11.5 * Ctrl
10440738	Tiam1	NM_009384	0.000466278	1.33595	E11.5 * Sox10 up vs E11.5 * Ctrl
10388749	Traf4	NM_009423	0.000860463	1.33564	E11.5 * Sox10 up vs E11.5 * Ctrl
10582712	Egln1	NM_053207	3.07565e-006	1.33564	E11.5 * Sox10 up vs E11.5 * Ctrl
10546163	Mcm2	NM_008564	0.00206299	1.33543	E11.5 * Sox10 up vs E11.5 * Ctrl
10477353	Mapre1	NM_007896	1.38079e-006	1.33535	E11.5 * Sox10 up vs E11.5 * Ctrl
10421197	Chmp7	NM_134078	0.00266149	1.33512	E11.5 * Sox10 up vs E11.5 * Ctrl
10559312	Dhcr7	NM_007856	0.00182637	1.335	E11.5 * Sox10 up vs E11.5 * Ctrl
10518561	Apitd1	NM_027263	0.00303223	1.33491	E11.5 * Sox10 up vs E11.5 * Ctrl
10507557	Ebna1bp2	NM_026932	1.46512e-005	1.33468	E11.5 * Sox10 up vs E11.5 * Ctrl
10426098	Creld2	NM_029720	2.90438e-006	1.33466	E11.5 * Sox10 up vs E11.5 * Ctrl
10579437	Ccdc124	NM_026964	0.00390303	1.33449	E11.5 * Sox10 up vs E11.5 * Ctrl
10431872	Slc38a1	ENSMUST00000	9.88477e-005	1.33426	E11.5 * Sox10 up vs E11.5 * Ctrl
10409616	Spock1	NM_009262	0.000300315	1.33402	E11.5 * Sox10 up vs E11.5 * Ctrl
10363669	Dnajc12	NM_013888	0.00103841	1.3337	E11.5 * Sox10 up vs E11.5 * Ctrl
10468929	Nmt2	NM_008708	0.000141274	1.33346	E11.5 * Sox10 up vs E11.5 * Ctrl
10596545	Rad54l2	NM_030730	7.77378e-006	1.33304	E11.5 * Sox10 up vs E11.5 * Ctrl
10397818	Cpsf2	NM_016856	9.81079e-008	1.33299	E11.5 * Sox10 up vs E11.5 * Ctrl
10510482	Clstn1	NM_023051	0.00558123	1.33284	E11.5 * Sox10 up vs E11.5 * Ctrl
10418247	E430028B21Rik	NM_178668	0.00109348	1.33278	E11.5 * Sox10 up vs E11.5 * Ctrl
10494662	Ywhah	NM_011738	2.60061e-006	1.33214	E11.5 * Sox10 up vs E11.5 * Ctrl
10488303	Crnkl1	NM_025820	0.000305469	1.33194	E11.5 * Sox10 up vs E11.5 * Ctrl
10485656	Elp4	NM_023876	0.00350225	1.33178	E11.5 * Sox10 up vs E11.5 * Ctrl
10507040	Spata6	NM_026470	0.0021138	1.33163	E11.5 * Sox10 up vs E11.5 * Ctrl
10509858	Sdhb	NM_023374	0.00132047	1.33149	E11.5 * Sox10 up vs E11.5 * Ctrl
10558707	Ric8	NM_053194	0.00091073	1.33149	E11.5 * Sox10 up vs E11.5 * Ctrl
10409464	Dbn1	NM_019813	0.00247124	1.33148	E11.5 * Sox10 up vs E11.5 * Ctrl
10358259	Nek7	NM_021605	0.000360093	1.33138	E11.5 * Sox10 up vs E11.5 * Ctrl
10434252	Hira	NM_010435	0.000188209	1.3308	E11.5 * Sox10 up vs E11.5 * Ctrl
10390175	Ngfr	NM_033217	0.000820605	1.33075	E11.5 * Sox10 up vs E11.5 * Ctrl
10371846	Apaf1	NM_001042558	5.25897e-005	1.33028	E11.5 * Sox10 up vs E11.5 * Ctrl
10592084	St3gal4	NM_009178	0.000806116	1.33025	E11.5 * Sox10 up vs E11.5 * Ctrl
10580274	Rnaseh2a	NM_027187	0.00126848	1.33001	E11.5 * Sox10 up vs E11.5 * Ctrl
10447097	Gemin6	NM_026053	0.00547747	1.32952	E11.5 * Sox10 up vs E11.5 * Ctrl
10480950	D2Bwg1335e	NM_026828	0.00102073	1.32945	E11.5 * Sox10 up vs E11.5 * Ctrl
10401753	AI413782	NM_001142580	0.000836124	1.32941	E11.5 * Sox10 up vs E11.5 * Ctrl

10480381	Arhgap21	NM_001128084	1.2553e-007	1.32938	E11.5 * Sox10 up vs E11.5 * Ctrl
10524314	Pitpnb	NM_019640	2.97933e-006	1.32938	E11.5 * Sox10 up vs E11.5 * Ctrl
10477644	Trp53inp2	NM_178111	0.000631687	1.32935	E11.5 * Sox10 up vs E11.5 * Ctrl
10418218	Slmap	NM_032008	1.4273e-005	1.32925	E11.5 * Sox10 up vs E11.5 * Ctrl
10438460	Parl	NM_001005767	0.00346843	1.32887	E11.5 * Sox10 up vs E11.5 * Ctrl
10586174		---	0.00159098	1.32864	E11.5 * Sox10 up vs E11.5 * Ctrl
10596637	Mapkapk3	NM_178907	0.00286354	1.32855	E11.5 * Sox10 up vs E11.5 * Ctrl
10572290	Nr2c2ap	NM_001025586	0.00375066	1.32842	E11.5 * Sox10 up vs E11.5 * Ctrl
10383970	Kremen1	NM_032396	0.000791259	1.32823	E11.5 * Sox10 up vs E11.5 * Ctrl
10433996	Snap29	NM_023348	0.000308681	1.32812	E11.5 * Sox10 up vs E11.5 * Ctrl
10465011	Sart1	NM_016882	0.000103409	1.32799	E11.5 * Sox10 up vs E11.5 * Ctrl
10514398	5830433M19Rik	BC020067	0.00123437	1.3278	E11.5 * Sox10 up vs E11.5 * Ctrl
10581940	Kars	NM_001130868	0.00011448	1.32752	E11.5 * Sox10 up vs E11.5 * Ctrl
10413752	Bap1	NM_027088	0.00930043	1.32739	E11.5 * Sox10 up vs E11.5 * Ctrl
10595836	E030011O05Rik	ENSMUST00000(	0.00174179	1.32737	E11.5 * Sox10 up vs E11.5 * Ctrl
10505200	Hsdl2	NM_024255	0.00020018	1.3273	E11.5 * Sox10 up vs E11.5 * Ctrl
10393774	Nploc4	NM_199469	0.000154282	1.32714	E11.5 * Sox10 up vs E11.5 * Ctrl
10425822	Pnpla3	NM_054088	0.000687228	1.32682	E11.5 * Sox10 up vs E11.5 * Ctrl
10358672	Hmcn1	NM_001024720	0.00243852	1.32669	E11.5 * Sox10 up vs E11.5 * Ctrl
10485225	Ext2	NM_010163	0.000194219	1.32662	E11.5 * Sox10 up vs E11.5 * Ctrl
10567941	Eif3c	NM_146200	5.69657e-008	1.32657	E11.5 * Sox10 up vs E11.5 * Ctrl
10440388	Hspa13	NR_027492	0.00627863	1.32647	E11.5 * Sox10 up vs E11.5 * Ctrl
10374485	Peli1	NM_023324	0.000663883	1.32639	E11.5 * Sox10 up vs E11.5 * Ctrl
10385175	Wwc1	NM_170779	0.00029853	1.32601	E11.5 * Sox10 up vs E11.5 * Ctrl
10521907	1810013D10Rik	NM_001145433	0.00958809	1.32598	E11.5 * Sox10 up vs E11.5 * Ctrl
10383671	Drg1	NM_007879	3.20234e-006	1.32581	E11.5 * Sox10 up vs E11.5 * Ctrl
10557231	EG628696	XR_004960	0.00966157	1.32544	E11.5 * Sox10 up vs E11.5 * Ctrl
10373036	Os9	NM_177614	0.00199303	1.32512	E11.5 * Sox10 up vs E11.5 * Ctrl
10449394	Taf11	NM_026836	3.624e-006	1.32493	E11.5 * Sox10 up vs E11.5 * Ctrl
10600377	Atp6ap1	NM_018794	0.00561644	1.3249	E11.5 * Sox10 up vs E11.5 * Ctrl
10561679	Psmd8	NM_026545	0.00023029	1.32484	E11.5 * Sox10 up vs E11.5 * Ctrl
10375083	Stk10	NM_009288	0.00014414	1.32473	E11.5 * Sox10 up vs E11.5 * Ctrl
10405074	Nol8	NM_001081350	0.000325096	1.32461	E11.5 * Sox10 up vs E11.5 * Ctrl
10447354	Txndc14	NM_025868	0.00110956	1.32438	E11.5 * Sox10 up vs E11.5 * Ctrl
10511207	Cpsf3l	NM_028020	0.000162645	1.32435	E11.5 * Sox10 up vs E11.5 * Ctrl
10591967	Jam3	NM_023277	0.000317291	1.3243	E11.5 * Sox10 up vs E11.5 * Ctrl
10507418	Eif2b3	NM_001111277	0.00179024	1.32424	E11.5 * Sox10 up vs E11.5 * Ctrl
10381668	Nmt1	NM_008707	1.08917e-005	1.32407	E11.5 * Sox10 up vs E11.5 * Ctrl
10389010	5730455P16Rik	NM_027472	0.00393373	1.3237	E11.5 * Sox10 up vs E11.5 * Ctrl
10435752	Lsamp	NM_175548	0.000198723	1.32368	E11.5 * Sox10 up vs E11.5 * Ctrl
10358658	Hmcn1	NM_001024720	0.00127593	1.32349	E11.5 * Sox10 up vs E11.5 * Ctrl
10395142	Sh3yl1	NM_013709	0.000215836	1.32336	E11.5 * Sox10 up vs E11.5 * Ctrl
10347774	Mrpl44	NM_001081210	0.000338427	1.32318	E11.5 * Sox10 up vs E11.5 * Ctrl
10578149	Leprotl1	NM_026609	2.17964e-006	1.32314	E11.5 * Sox10 up vs E11.5 * Ctrl
10524105	Chfr	NM_172717	6.8581e-005	1.32308	E11.5 * Sox10 up vs E11.5 * Ctrl
10400189	EG636070	XR_030785	0.00393016	1.32258	E11.5 * Sox10 up vs E11.5 * Ctrl
10516246	Cdca8	NM_026560	2.81464e-005	1.32257	E11.5 * Sox10 up vs E11.5 * Ctrl
10482824	Acvr1	NM_001110204	3.04133e-006	1.32245	E11.5 * Sox10 up vs E11.5 * Ctrl
10553280	Gtf2h1	NM_008186	0.000180389	1.32228	E11.5 * Sox10 up vs E11.5 * Ctrl
10357630	Srgap2	NM_001081011	0.000970772	1.32184	E11.5 * Sox10 up vs E11.5 * Ctrl
10425226	Eif3l	NM_145139	2.39401e-006	1.32154	E11.5 * Sox10 up vs E11.5 * Ctrl
10541091	Gemin6	NM_026053	0.000227583	1.3215	E11.5 * Sox10 up vs E11.5 * Ctrl
10593270	Ttc12	NM_172770	0.000113307	1.32126	E11.5 * Sox10 up vs E11.5 * Ctrl
10596680	Sema3b	NM_001042779	0.00186507	1.32125	E11.5 * Sox10 up vs E11.5 * Ctrl
10421661	Gtf2f2	NM_026816	2.05918e-005	1.32124	E11.5 * Sox10 up vs E11.5 * Ctrl
10362904	Rtn4ip1	NM_130892	0.00140761	1.32113	E11.5 * Sox10 up vs E11.5 * Ctrl
10416666	Sugt1	NM_026474	0.000169139	1.32094	E11.5 * Sox10 up vs E11.5 * Ctrl
10487371	Ciao1	NM_025296	0.000850544	1.3206	E11.5 * Sox10 up vs E11.5 * Ctrl
10497752	Carhsp1	NM_025821	0.00713082	1.32004	E11.5 * Sox10 up vs E11.5 * Ctrl
10452892	Fam98a	NM_133747	0.000533803	1.31976	E11.5 * Sox10 up vs E11.5 * Ctrl
10373569	Rpsa	NM_011029	0.00823663	1.31972	E11.5 * Sox10 up vs E11.5 * Ctrl
10399555	Kcnf1	NM_201531	0.00880846	1.31927	E11.5 * Sox10 up vs E11.5 * Ctrl
10494804	Casq2	NM_009814	0.0032713	1.31883	E11.5 * Sox10 up vs E11.5 * Ctrl
10607317	Tsr2	NM_175146	0.00310375	1.31877	E11.5 * Sox10 up vs E11.5 * Ctrl
10441815	Sod2	NM_013671	0.000323424	1.31853	E11.5 * Sox10 up vs E11.5 * Ctrl
10550632	Opa3	NM_207525	0.00203521	1.31842	E11.5 * Sox10 up vs E11.5 * Ctrl

10367634	Akap12	NM_031185	2.41318e-005	1.31836	E11.5 * Sox10 up vs E11.5 * Ctrl
10431948	Rpap3	NM_028003	0.00155121	1.31833	E11.5 * Sox10 up vs E11.5 * Ctrl
10516305	Mtap7d1	NM_144941	6.78325e-006	1.31808	E11.5 * Sox10 up vs E11.5 * Ctrl
10410929	Agk	NM_023538	0.00365254	1.31796	E11.5 * Sox10 up vs E11.5 * Ctrl
10380986	Psmd3	NM_009439	0.000772493	1.31785	E11.5 * Sox10 up vs E11.5 * Ctrl
10447431	Foxn2	NM_180974	4.30988e-006	1.31738	E11.5 * Sox10 up vs E11.5 * Ctrl
10502881	St6galnac5	NM_012028	0.000788597	1.31732	E11.5 * Sox10 up vs E11.5 * Ctrl
10575102	Cirh1a	NM_011574	0.000457557	1.31715	E11.5 * Sox10 up vs E11.5 * Ctrl
10510604	Dnajc11	NM_172704	0.001335	1.3164	E11.5 * Sox10 up vs E11.5 * Ctrl
10375499	Snord96a	AJ132685	0.00437255	1.31621	E11.5 * Sox10 up vs E11.5 * Ctrl
10394809	EG668645	XR_033945	0.00938162	1.31618	E11.5 * Sox10 up vs E11.5 * Ctrl
10394816	EG668645	XR_033945	0.00938162	1.31618	E11.5 * Sox10 up vs E11.5 * Ctrl
10350247	Kif21b	NM_001039472	0.000351754	1.31605	E11.5 * Sox10 up vs E11.5 * Ctrl
10468533	Gpam	NM_008149	0.000177204	1.31605	E11.5 * Sox10 up vs E11.5 * Ctrl
10582295	Odc1	NM_013614	0.00011156	1.31603	E11.5 * Sox10 up vs E11.5 * Ctrl
10543004	Pon2	NM_183308	0.00100267	1.31586	E11.5 * Sox10 up vs E11.5 * Ctrl
10425267	Pick1	NM_008837	0.00157853	1.31582	E11.5 * Sox10 up vs E11.5 * Ctrl
10400095	Ifrd1	NM_013562	0.0014574	1.31578	E11.5 * Sox10 up vs E11.5 * Ctrl
10420659	6330409N04Rik	NM_025697	2.99277e-005	1.31556	E11.5 * Sox10 up vs E11.5 * Ctrl
10487969	Trmt6	NM_175113	0.00154164	1.31551	E11.5 * Sox10 up vs E11.5 * Ctrl
10348653	Gpc1	NM_016696	0.00200541	1.31515	E11.5 * Sox10 up vs E11.5 * Ctrl
10446470	ENSMUSG0000C	ENSMUST00000(	0.00314073	1.31493	E11.5 * Sox10 up vs E11.5 * Ctrl
10496397	Mapksp1	NM_019920	0.00010729	1.31484	E11.5 * Sox10 up vs E11.5 * Ctrl
10511149	Mrpl20	NM_025570	0.00263767	1.31414	E11.5 * Sox10 up vs E11.5 * Ctrl
10462346	Rcl1	NM_021525	2.13689e-005	1.3141	E11.5 * Sox10 up vs E11.5 * Ctrl
10383575	Tbcd	NM_029878	7.87047e-006	1.31388	E11.5 * Sox10 up vs E11.5 * Ctrl
10457733	B4galt6	NM_019737	0.00044904	1.31382	E11.5 * Sox10 up vs E11.5 * Ctrl
10430389	Mfng	NM_008595	0.00103317	1.31374	E11.5 * Sox10 up vs E11.5 * Ctrl
10591726	Ecsit	NM_012029	0.000888484	1.31369	E11.5 * Sox10 up vs E11.5 * Ctrl
10548194	Fkbp4	NM_010219	0.00226653	1.31363	E11.5 * Sox10 up vs E11.5 * Ctrl
10537463	Agk	NM_023538	7.37139e-007	1.31329	E11.5 * Sox10 up vs E11.5 * Ctrl
10498038	Elf2	NM_023502	3.74212e-005	1.31308	E11.5 * Sox10 up vs E11.5 * Ctrl
10582599	Nup133	NM_172288	0.000142546	1.31299	E11.5 * Sox10 up vs E11.5 * Ctrl
10524668	Coq5	NM_026504	0.00269489	1.31289	E11.5 * Sox10 up vs E11.5 * Ctrl
10584142	Ets1	NM_011808	0.000470417	1.31286	E11.5 * Sox10 up vs E11.5 * Ctrl
10409265	Auh	NM_016709	0.00186767	1.31279	E11.5 * Sox10 up vs E11.5 * Ctrl
10589087	Prkar2a	NM_008924	0.000476334	1.31249	E11.5 * Sox10 up vs E11.5 * Ctrl
10373950	Nipsnap1	NM_008698	0.000132409	1.31208	E11.5 * Sox10 up vs E11.5 * Ctrl
10345350		---	0.000700537	1.31205	E11.5 * Sox10 up vs E11.5 * Ctrl
10564692	Mrpl46	NM_023331	0.00211716	1.31156	E11.5 * Sox10 up vs E11.5 * Ctrl
10351269	4930455F23Rik	NM_029115	0.000882283	1.31132	E11.5 * Sox10 up vs E11.5 * Ctrl
10526614	Actl6b	NM_031404	0.00333957	1.31111	E11.5 * Sox10 up vs E11.5 * Ctrl
10406229	Pcsk1	NM_013628	0.00548705	1.31107	E11.5 * Sox10 up vs E11.5 * Ctrl
10425559	L3mbtl2	NM_145993	6.30187e-005	1.31095	E11.5 * Sox10 up vs E11.5 * Ctrl
10461237	Bscl2	NM_001136064	0.000235651	1.31087	E11.5 * Sox10 up vs E11.5 * Ctrl
10576873	Elavl1	NM_010485	2.66367e-005	1.31079	E11.5 * Sox10 up vs E11.5 * Ctrl
10576274	Zfp276	NM_020497	0.00243031	1.31066	E11.5 * Sox10 up vs E11.5 * Ctrl
10407543	Gtpbp4	NM_027000	8.1595e-006	1.3106	E11.5 * Sox10 up vs E11.5 * Ctrl
10501183	4933431E20Rik	BC060136	0.00559248	1.3103	E11.5 * Sox10 up vs E11.5 * Ctrl
10470705	Rapgef1	NM_001039087	0.00107989	1.31009	E11.5 * Sox10 up vs E11.5 * Ctrl
10539606	Cct7	NM_007638	2.73057e-005	1.31	E11.5 * Sox10 up vs E11.5 * Ctrl
10405619	5133401N09Rik	NM_198004	0.000164635	1.30995	E11.5 * Sox10 up vs E11.5 * Ctrl
10427494		---	0.00632412	1.30943	E11.5 * Sox10 up vs E11.5 * Ctrl
10465804	Polr2g	NM_026329	9.61267e-005	1.30916	E11.5 * Sox10 up vs E11.5 * Ctrl
10436941	Mrps6	NM_080456	0.0050313	1.30909	E11.5 * Sox10 up vs E11.5 * Ctrl
10360934	Rrp15	NM_026041	5.03066e-005	1.30882	E11.5 * Sox10 up vs E11.5 * Ctrl
10402665	Cdc42bpb	NM_183016	0.00208402	1.30878	E11.5 * Sox10 up vs E11.5 * Ctrl
10433462	Pmm2	NM_016881	0.00247398	1.3087	E11.5 * Sox10 up vs E11.5 * Ctrl
10373577	Ormdl2	NM_024180	0.00191773	1.30862	E11.5 * Sox10 up vs E11.5 * Ctrl
10512728	BC057893	NM_173033	0.000426763	1.30821	E11.5 * Sox10 up vs E11.5 * Ctrl
10543551	Rbm28	NM_133925	7.39005e-005	1.3082	E11.5 * Sox10 up vs E11.5 * Ctrl
10564849	2610034B18Rik	NM_027420	5.00392e-005	1.3082	E11.5 * Sox10 up vs E11.5 * Ctrl
10415725	Spata13	BC138455	0.00289438	1.30806	E11.5 * Sox10 up vs E11.5 * Ctrl
10381776	Mapt	NM_001038609	0.00493967	1.308	E11.5 * Sox10 up vs E11.5 * Ctrl
10363563	Slc25a16	NM_175194	0.00769362	1.30724	E11.5 * Sox10 up vs E11.5 * Ctrl
10561854	Tbcb	NM_025548	0.000499533	1.30721	E11.5 * Sox10 up vs E11.5 * Ctrl

10596951	Arih2	NM_011790	0.000973877	1.30694	E11.5 * Sox10 up vs E11.5 * Ctrl
10606835	Bex2	NM_009749	0.00403353	1.30673	E11.5 * Sox10 up vs E11.5 * Ctrl
10586384	Pdcd7	NM_016688	0.000782311	1.30663	E11.5 * Sox10 up vs E11.5 * Ctrl
10585509	Fbxo22	NM_028049	0.00334086	1.30653	E11.5 * Sox10 up vs E11.5 * Ctrl
10459844	Haus1	NM_146089	0.000283966	1.30646	E11.5 * Sox10 up vs E11.5 * Ctrl
10539882	Ruvbl1	NM_019685	5.22401e-005	1.30643	E11.5 * Sox10 up vs E11.5 * Ctrl
10383289	Baiap2	NM_130862	0.00156253	1.30634	E11.5 * Sox10 up vs E11.5 * Ctrl
10499045	Trim2	NM_030706	7.52896e-005	1.30621	E11.5 * Sox10 up vs E11.5 * Ctrl
10432404	Tuba1a	NM_011653	6.61728e-005	1.306	E11.5 * Sox10 up vs E11.5 * Ctrl
10519472	ENSMUSG0000C	NM_001042670	0.00730423	1.30537	E11.5 * Sox10 up vs E11.5 * Ctrl
10499981	Oaz3	NM_016901	0.00496772	1.30537	E11.5 * Sox10 up vs E11.5 * Ctrl
10352396	Trp53bp2	NM_173378	2.46935e-005	1.30439	E11.5 * Sox10 up vs E11.5 * Ctrl
10391755	Ccdc43	NM_025918	9.41634e-005	1.30428	E11.5 * Sox10 up vs E11.5 * Ctrl
10439732	Plcxd2	NM_001134480	0.00414691	1.30418	E11.5 * Sox10 up vs E11.5 * Ctrl
10458607	Lars	NM_134137	2.92982e-005	1.30403	E11.5 * Sox10 up vs E11.5 * Ctrl
10511226	Acap3	NM_207223	0.000591411	1.30377	E11.5 * Sox10 up vs E11.5 * Ctrl
10484431	Txndc14	NM_025868	0.0013521	1.30372	E11.5 * Sox10 up vs E11.5 * Ctrl
10507379	Zswim5	NM_001029912	0.00129252	1.3036	E11.5 * Sox10 up vs E11.5 * Ctrl
10573713	Heatr3	NM_172757	0.000300601	1.30356	E11.5 * Sox10 up vs E11.5 * Ctrl
10424349	Sqle	NM_009270	0.000413966	1.30351	E11.5 * Sox10 up vs E11.5 * Ctrl
10574718	Tmem208	NM_025486	0.00141146	1.30328	E11.5 * Sox10 up vs E11.5 * Ctrl
10483228	Scn3a	NM_018732	0.00859505	1.3031	E11.5 * Sox10 up vs E11.5 * Ctrl
10350848	2810025M15Rik	BC055845	0.00027198	1.30304	E11.5 * Sox10 up vs E11.5 * Ctrl
10419966	Zfhx2	NM_001039198	0.00738044	1.30302	E11.5 * Sox10 up vs E11.5 * Ctrl
10456764	Smad2	NM_010754	1.41924e-005	1.30286	E11.5 * Sox10 up vs E11.5 * Ctrl
10521725	Med28	NM_025895	0.00330114	1.30283	E11.5 * Sox10 up vs E11.5 * Ctrl
10449452	Fkbp5	NM_010220	0.0019394	1.30276	E11.5 * Sox10 up vs E11.5 * Ctrl
10370483	Rrp1	NM_010925	0.0012313	1.30263	E11.5 * Sox10 up vs E11.5 * Ctrl
10511139	Ssu72	NM_026899	0.000376323	1.30262	E11.5 * Sox10 up vs E11.5 * Ctrl
10481804	Ralgps1	NM_175211	0.00106501	1.30253	E11.5 * Sox10 up vs E11.5 * Ctrl
10452257	Slc25a23	NM_025877	0.00574482	1.30248	E11.5 * Sox10 up vs E11.5 * Ctrl
10347873	Agfg1	NM_010472	3.69298e-005	1.30245	E11.5 * Sox10 up vs E11.5 * Ctrl
10574595		---	1.98671e-005	1.30241	E11.5 * Sox10 up vs E11.5 * Ctrl
10587503	Sh3bgrl2	NM_172507	0.00522783	1.30222	E11.5 * Sox10 up vs E11.5 * Ctrl
10547540	Mical3	BC043122	0.0075593	1.30222	E11.5 * Sox10 up vs E11.5 * Ctrl
10581729	Ddx19a	NM_007916	0.0010337	1.30208	E11.5 * Sox10 up vs E11.5 * Ctrl
10452110	2410015M20Rik	NM_153152	0.00191661	1.30167	E11.5 * Sox10 up vs E11.5 * Ctrl
10599627	Hprt1	NM_013556	2.91671e-005	1.30151	E11.5 * Sox10 up vs E11.5 * Ctrl
10412076	Gemin8	NM_146238	0.00332224	1.30142	E11.5 * Sox10 up vs E11.5 * Ctrl
10450814	Ppp1r11	NM_029632	0.000117832	1.30141	E11.5 * Sox10 up vs E11.5 * Ctrl
10524004	Pcgf3	NM_172716	1.19115e-005	1.30139	E11.5 * Sox10 up vs E11.5 * Ctrl
10362767	Mical1	NM_138315	0.00185798	1.3013	E11.5 * Sox10 up vs E11.5 * Ctrl
10604922	BC023829	NM_001033328	0.000156096	1.30095	E11.5 * Sox10 up vs E11.5 * Ctrl
10460490	Dpp3	NM_133803	0.000866894	1.3009	E11.5 * Sox10 up vs E11.5 * Ctrl
10351636	Refbp2	NM_019484	0.00574338	1.30072	E11.5 * Sox10 up vs E11.5 * Ctrl
10407307	Mocs2	NM_013826	0.00171293	1.30066	E11.5 * Sox10 up vs E11.5 * Ctrl
10458498	Arap3	NM_139206	0.000535113	1.30041	E11.5 * Sox10 up vs E11.5 * Ctrl
10401891	Ston2	NM_175367	0.00143577	1.30029	E11.5 * Sox10 up vs E11.5 * Ctrl
10430804	Pppde2	NM_134095	0.00170629	1.3002	E11.5 * Sox10 up vs E11.5 * Ctrl
10423629	Pop1	NM_152894	0.00126471	1.30005	E11.5 * Sox10 up vs E11.5 * Ctrl
10568217	Dctpp1	NM_023203	0.00470675	1.3	E11.5 * Sox10 up vs E11.5 * Ctrl
10375038	Mpg	NM_010822	0.00998464	1.29987	E11.5 * Sox10 up vs E11.5 * Ctrl
10478778	Arfgef2	NM_001085495	0.00192848	1.2998	E11.5 * Sox10 up vs E11.5 * Ctrl
10487945	Prei4	NM_028802	0.00666641	1.29977	E11.5 * Sox10 up vs E11.5 * Ctrl
10598994	Atp1b3	NM_007502	0.000189775	1.29975	E11.5 * Sox10 up vs E11.5 * Ctrl
10409557	H2afy	NM_012015	1.48057e-006	1.29967	E11.5 * Sox10 up vs E11.5 * Ctrl
10545940	Gmcl1	NM_011818	1.42079e-007	1.29966	E11.5 * Sox10 up vs E11.5 * Ctrl
10427035	Nr4a1	NM_010444	0.00176688	1.29962	E11.5 * Sox10 up vs E11.5 * Ctrl
10345058	Tmem14a	NM_029398	0.00233798	1.29945	E11.5 * Sox10 up vs E11.5 * Ctrl
10439471	Ktelc1	NM_172380	0.000691972	1.29924	E11.5 * Sox10 up vs E11.5 * Ctrl
10527430	Arpc1a	NM_019767	1.0493e-006	1.29912	E11.5 * Sox10 up vs E11.5 * Ctrl
10597627	Oxsr1	NM_133985	0.000176164	1.29888	E11.5 * Sox10 up vs E11.5 * Ctrl
10487392	Kcnip3	NM_019789	0.00423094	1.29885	E11.5 * Sox10 up vs E11.5 * Ctrl
10519203	A230069A22Rik	NM_001033394	0.00424166	1.29877	E11.5 * Sox10 up vs E11.5 * Ctrl
10448765	Mapk8ip3	NM_013931	4.65536e-005	1.2987	E11.5 * Sox10 up vs E11.5 * Ctrl
10534974	Mcm7	NM_008568	0.0045492	1.29868	E11.5 * Sox10 up vs E11.5 * Ctrl

10523012	Dck	NM_007832	0.00448201	1.29865	E11.5 * Sox10 up vs E11.5 * Ctrl
10412667	Ptprg	NM_008981	0.000703133	1.29839	E11.5 * Sox10 up vs E11.5 * Ctrl
10439566	Atp6v1a	NM_007508	4.17122e-005	1.29818	E11.5 * Sox10 up vs E11.5 * Ctrl
10443561	1300018I05Rik	NM_028791	0.000324906	1.29793	E11.5 * Sox10 up vs E11.5 * Ctrl
10547553	Mical3	NM_153396	0.000723375	1.29781	E11.5 * Sox10 up vs E11.5 * Ctrl
10604630	Mospd1	NM_027409	0.00108041	1.29769	E11.5 * Sox10 up vs E11.5 * Ctrl
10369525	2010107G23Rik	NM_027251	0.00286502	1.29748	E11.5 * Sox10 up vs E11.5 * Ctrl
10448707	Tbl3	NM_145396	0.00102375	1.29742	E11.5 * Sox10 up vs E11.5 * Ctrl
10584941	Bace1	NM_011792	0.00289963	1.29726	E11.5 * Sox10 up vs E11.5 * Ctrl
10405001	Bicd2	NM_001039179	0.00146247	1.29704	E11.5 * Sox10 up vs E11.5 * Ctrl
10396694	Churc1	NM_206534	0.00185832	1.29693	E11.5 * Sox10 up vs E11.5 * Ctrl
10433721	Nde1	NM_023317	0.000124849	1.29666	E11.5 * Sox10 up vs E11.5 * Ctrl
10461723	4632417K18Rik	NM_026640	0.00813859	1.29653	E11.5 * Sox10 up vs E11.5 * Ctrl
10413238	Cphx	NM_175342	0.00716633	1.29641	E11.5 * Sox10 up vs E11.5 * Ctrl
10413260	Cphx	NM_175342	0.00716633	1.29641	E11.5 * Sox10 up vs E11.5 * Ctrl
10573348	Cacna1a	NM_007578	0.000560685	1.29629	E11.5 * Sox10 up vs E11.5 * Ctrl
10587746	Tmem41b	NM_153525	0.000714825	1.29611	E11.5 * Sox10 up vs E11.5 * Ctrl
10491329	Zmat3	NM_009517	0.00133534	1.29608	E11.5 * Sox10 up vs E11.5 * Ctrl
10396800	Plekhh1	ENSMUST00000(	1.68209e-005	1.29595	E11.5 * Sox10 up vs E11.5 * Ctrl
10573217	Ddx39	NM_197982	5.42998e-005	1.2958	E11.5 * Sox10 up vs E11.5 * Ctrl
10454564	Ercc3	NM_133658	0.000118811	1.29577	E11.5 * Sox10 up vs E11.5 * Ctrl
10474411	Lin7c	NM_011699	9.78564e-008	1.29559	E11.5 * Sox10 up vs E11.5 * Ctrl
10542522	Plekha5	NM_144920	0.000298023	1.29536	E11.5 * Sox10 up vs E11.5 * Ctrl
10406226	Hnrnpa1l2	XM_619124	0.00176367	1.29512	E11.5 * Sox10 up vs E11.5 * Ctrl
10525804	Atp6v0a2	NM_011596	0.00298786	1.29506	E11.5 * Sox10 up vs E11.5 * Ctrl
10457054	Zadh2	NM_146090	0.000913473	1.29483	E11.5 * Sox10 up vs E11.5 * Ctrl
10603373	Pqbp1	NM_019478	0.000157863	1.29481	E11.5 * Sox10 up vs E11.5 * Ctrl
10376813	Cytsb	NM_001029936	0.000469239	1.29478	E11.5 * Sox10 up vs E11.5 * Ctrl
10492456	Rsrc1	NM_025822	0.00777168	1.29447	E11.5 * Sox10 up vs E11.5 * Ctrl
10471967	Mbd5	NM_029924	0.00233631	1.29429	E11.5 * Sox10 up vs E11.5 * Ctrl
10454851	Cxxc5	NM_133687	0.00158599	1.29426	E11.5 * Sox10 up vs E11.5 * Ctrl
10380641	Hoxb5	NM_008268	3.11681e-005	1.29355	E11.5 * Sox10 up vs E11.5 * Ctrl
10544570	Pcnp	NM_001024622	0.0027914	1.29318	E11.5 * Sox10 up vs E11.5 * Ctrl
10363173	Gja1	NM_010288	6.14891e-005	1.29267	E11.5 * Sox10 up vs E11.5 * Ctrl
10601473	Apool	NM_026565	0.00288849	1.29259	E11.5 * Sox10 up vs E11.5 * Ctrl
10500685	Atp1a1	NM_144900	0.000226347	1.29244	E11.5 * Sox10 up vs E11.5 * Ctrl
10415377	2610027L16Rik	NM_026403	0.000327597	1.29214	E11.5 * Sox10 up vs E11.5 * Ctrl
10541711	Phb2	NM_007531	0.000142886	1.29201	E11.5 * Sox10 up vs E11.5 * Ctrl
10574184	Rspry1	NM_026274	0.000184939	1.29198	E11.5 * Sox10 up vs E11.5 * Ctrl
10436053	Dppa2	NM_028615	0.000407707	1.29198	E11.5 * Sox10 up vs E11.5 * Ctrl
10543835	Chchd3	NM_025336	0.000221652	1.29195	E11.5 * Sox10 up vs E11.5 * Ctrl
10430201	Myh9	NM_022410	0.00107008	1.29189	E11.5 * Sox10 up vs E11.5 * Ctrl
10576403	Urb2	NM_001029876	0.0012745	1.29181	E11.5 * Sox10 up vs E11.5 * Ctrl
10521136	Whsc1	NM_001081102	1.64719e-005	1.29177	E11.5 * Sox10 up vs E11.5 * Ctrl
10434467	Psmd2	NR_027485	0.000826518	1.29175	E11.5 * Sox10 up vs E11.5 * Ctrl
10506643	Tmem59	NM_029565	0.000167719	1.29159	E11.5 * Sox10 up vs E11.5 * Ctrl
10402650	2810452K22Rik	NM_026048	0.000915415	1.29124	E11.5 * Sox10 up vs E11.5 * Ctrl
10481621	1110008P14Rik	NM_198001	0.000752475	1.29124	E11.5 * Sox10 up vs E11.5 * Ctrl
10475245	AV039307	AK006022	0.00247136	1.291	E11.5 * Sox10 up vs E11.5 * Ctrl
10524703	Pxn	NM_011223	0.00149433	1.29098	E11.5 * Sox10 up vs E11.5 * Ctrl
10582719	Sipa1l2	NM_001081337	0.00336102	1.29098	E11.5 * Sox10 up vs E11.5 * Ctrl
10508454	Bsdc1	NM_133889	0.00966966	1.29097	E11.5 * Sox10 up vs E11.5 * Ctrl
10450212	Egfl8	NM_152922	9.06903e-005	1.29081	E11.5 * Sox10 up vs E11.5 * Ctrl
10493703	Jtb	NM_206924	0.0020068	1.2908	E11.5 * Sox10 up vs E11.5 * Ctrl
10396306	1200003C05Rik	NM_024205	0.000678228	1.29071	E11.5 * Sox10 up vs E11.5 * Ctrl
10390211	Igf2bp1	NM_009951	0.00908936	1.29045	E11.5 * Sox10 up vs E11.5 * Ctrl
10459643	4930503L19Rik	NM_172967	0.00486329	1.28993	E11.5 * Sox10 up vs E11.5 * Ctrl
10374228	OTTMUSG00000	NM_001037928	0.00467498	1.28988	E11.5 * Sox10 up vs E11.5 * Ctrl
10479514	Col20a1	BC016112	0.00519174	1.28974	E11.5 * Sox10 up vs E11.5 * Ctrl
10593130	Sidt2	NM_172257	0.0006241	1.2896	E11.5 * Sox10 up vs E11.5 * Ctrl
10379901	Bcas3	NM_138681	0.000397002	1.28945	E11.5 * Sox10 up vs E11.5 * Ctrl
10362392	Psmd2	NR_027485	0.000258682	1.28936	E11.5 * Sox10 up vs E11.5 * Ctrl
10471505	Sh2d3c	NM_013781	0.0019316	1.28922	E11.5 * Sox10 up vs E11.5 * Ctrl
10480921	Qsox2	NM_153559	0.000420452	1.28919	E11.5 * Sox10 up vs E11.5 * Ctrl
10432986	Aaas	NM_153416	0.00142529	1.28914	E11.5 * Sox10 up vs E11.5 * Ctrl
10406898	Taf9	NM_027592	0.000391914	1.28911	E11.5 * Sox10 up vs E11.5 * Ctrl

10579830	Rbmxrt	NM_009033	1.38456e-005	1.28909	E11.5 * Sox10 up vs E11.5 * Ctrl
10465895	Fads2	NM_019699	0.0021525	1.28889	E11.5 * Sox10 up vs E11.5 * Ctrl
10369290	Ddit4	NM_029083	4.27478e-005	1.28864	E11.5 * Sox10 up vs E11.5 * Ctrl
10417183	Pcca	NM_144844	0.00101104	1.28863	E11.5 * Sox10 up vs E11.5 * Ctrl
10380648	Hoxb4	NM_010459	0.000788975	1.28818	E11.5 * Sox10 up vs E11.5 * Ctrl
10579825	Pou4f2	NM_138944	3.2841e-005	1.28799	E11.5 * Sox10 up vs E11.5 * Ctrl
10524621	Oasl2	NM_011854	0.000590598	1.28793	E11.5 * Sox10 up vs E11.5 * Ctrl
10414256	Rbmxrt	NM_009033	1.6002e-005	1.28779	E11.5 * Sox10 up vs E11.5 * Ctrl
10430265	Eif3d	NM_018749	0.0041664	1.28778	E11.5 * Sox10 up vs E11.5 * Ctrl
10487340	Ncaph	NM_144818	3.23709e-005	1.28768	E11.5 * Sox10 up vs E11.5 * Ctrl
10367292	Cs	NM_026444	7.92105e-005	1.28741	E11.5 * Sox10 up vs E11.5 * Ctrl
10505705	Sh3gl2	NM_019535	0.00124346	1.28728	E11.5 * Sox10 up vs E11.5 * Ctrl
10587194	Gnb5	NM_138719	0.00865102	1.28665	E11.5 * Sox10 up vs E11.5 * Ctrl
10486552	Lrrc57	NM_001159612	0.000120388	1.28654	E11.5 * Sox10 up vs E11.5 * Ctrl
10540168	Fgd5	NM_172731	0.000192146	1.28642	E11.5 * Sox10 up vs E11.5 * Ctrl
10493103	Isg20l2	NM_177663	0.0070795	1.28602	E11.5 * Sox10 up vs E11.5 * Ctrl
10573893	Fto	NM_011936	0.00734458	1.28574	E11.5 * Sox10 up vs E11.5 * Ctrl
10572146	Atp6v1b2	NM_007509	0.00245185	1.28554	E11.5 * Sox10 up vs E11.5 * Ctrl
10377889	Mink1	NM_001045959	0.00278888	1.28546	E11.5 * Sox10 up vs E11.5 * Ctrl
10532857	Gltp	NM_019821	0.00227028	1.28513	E11.5 * Sox10 up vs E11.5 * Ctrl
10406881	Smn1	NM_011420	0.000249266	1.28498	E11.5 * Sox10 up vs E11.5 * Ctrl
10355813	Ptprn	NM_008985	0.00147326	1.28477	E11.5 * Sox10 up vs E11.5 * Ctrl
10373740	Pik3ip1	NM_178149	0.00275859	1.28473	E11.5 * Sox10 up vs E11.5 * Ctrl
10466304	Dtx4	NM_172442	0.000431384	1.28412	E11.5 * Sox10 up vs E11.5 * Ctrl
10473045	Rbm45	NM_153405	0.000210198	1.28342	E11.5 * Sox10 up vs E11.5 * Ctrl
10559996	100042997	XR_034250	0.000518821	1.28335	E11.5 * Sox10 up vs E11.5 * Ctrl
10589438	Mtap4	NM_008633	0.000124508	1.28332	E11.5 * Sox10 up vs E11.5 * Ctrl
10523772	Lrrc8d	NM_178701	0.0013282	1.28304	E11.5 * Sox10 up vs E11.5 * Ctrl
10507238	Lrrc41	NM_153521	0.00407271	1.28285	E11.5 * Sox10 up vs E11.5 * Ctrl
10548585	Csda	NM_139117	0.00173829	1.28267	E11.5 * Sox10 up vs E11.5 * Ctrl
10578300	Mtmr7	NM_001040699	0.00210858	1.28255	E11.5 * Sox10 up vs E11.5 * Ctrl
10416371	P2ry5	NM_175116	0.0012479	1.28241	E11.5 * Sox10 up vs E11.5 * Ctrl
10561356	Psmc4	NM_011874	0.000409887	1.28199	E11.5 * Sox10 up vs E11.5 * Ctrl
10451763	Satb1	NM_009122	8.1072e-006	1.28198	E11.5 * Sox10 up vs E11.5 * Ctrl
10443021	Atp6v0e	NM_025272	0.000166054	1.28189	E11.5 * Sox10 up vs E11.5 * Ctrl
10444420	Agpat1	NM_018862	0.00476916	1.28156	E11.5 * Sox10 up vs E11.5 * Ctrl
10392328	Nol11	NM_133702	0.00253221	1.28141	E11.5 * Sox10 up vs E11.5 * Ctrl
10466818	Cbwd1	NM_146097	0.00840183	1.28124	E11.5 * Sox10 up vs E11.5 * Ctrl
10435676	Gsk3b	NM_019827	2.11504e-005	1.28118	E11.5 * Sox10 up vs E11.5 * Ctrl
10386230	Rnf187	AB030190	0.00282619	1.28111	E11.5 * Sox10 up vs E11.5 * Ctrl
10608136		---	0.0011316	1.281	E11.5 * Sox10 up vs E11.5 * Ctrl
10498296	Commd2	NM_175095	8.82263e-006	1.2809	E11.5 * Sox10 up vs E11.5 * Ctrl
10517731	Igsf21	NM_198610	0.00898655	1.28082	E11.5 * Sox10 up vs E11.5 * Ctrl
10475965	Polr1b	NM_009086	0.000228762	1.2799	E11.5 * Sox10 up vs E11.5 * Ctrl
10471191	Exosc2	NM_144886	0.00140382	1.27985	E11.5 * Sox10 up vs E11.5 * Ctrl
10479649	Prpf6	NM_133701	2.2106e-005	1.27971	E11.5 * Sox10 up vs E11.5 * Ctrl
10518453	Chchd2	NM_024166	0.00745674	1.2797	E11.5 * Sox10 up vs E11.5 * Ctrl
10578922	Klhl2	NM_178633	0.0034241	1.27947	E11.5 * Sox10 up vs E11.5 * Ctrl
10571048	Lsm1	NM_026032	0.00412565	1.27933	E11.5 * Sox10 up vs E11.5 * Ctrl
10559983	Zscan4-ps2	ENSMUST00000(	0.000937421	1.27907	E11.5 * Sox10 up vs E11.5 * Ctrl
10465772	Slc3a2	NM_008577	0.00482143	1.27907	E11.5 * Sox10 up vs E11.5 * Ctrl
10400866	Trim9	NM_053167	0.00653607	1.27897	E11.5 * Sox10 up vs E11.5 * Ctrl
10501319	Celsr2	NM_017392	0.00615776	1.27879	E11.5 * Sox10 up vs E11.5 * Ctrl
10503845	Ube2j1	NM_019586	0.000425302	1.27862	E11.5 * Sox10 up vs E11.5 * Ctrl
10579550	Unc13a	BC058348	0.00650821	1.27852	E11.5 * Sox10 up vs E11.5 * Ctrl
10447589	Fbl	NM_007991	6.47059e-005	1.27836	E11.5 * Sox10 up vs E11.5 * Ctrl
10546944	Tmem111	NM_175101	0.000920138	1.27824	E11.5 * Sox10 up vs E11.5 * Ctrl
10511803	2610029I01Rik	ENSMUST00000(	0.00702032	1.2782	E11.5 * Sox10 up vs E11.5 * Ctrl
10365260	Txnrd1	NM_001042523	5.25976e-006	1.27785	E11.5 * Sox10 up vs E11.5 * Ctrl
10553993	Snrpa1	NM_021336	1.96805e-005	1.27772	E11.5 * Sox10 up vs E11.5 * Ctrl
10377681	Dullard	NM_026017	0.000392724	1.27751	E11.5 * Sox10 up vs E11.5 * Ctrl
10500412	Gpr89	NM_026229	0.000152735	1.27741	E11.5 * Sox10 up vs E11.5 * Ctrl
10539342	Rtkn	NM_133641	0.00134456	1.27699	E11.5 * Sox10 up vs E11.5 * Ctrl
10346298	Coq10b	NM_001039710	0.00400852	1.27697	E11.5 * Sox10 up vs E11.5 * Ctrl
10605172	Ard1a	NM_019870	0.0024464	1.27696	E11.5 * Sox10 up vs E11.5 * Ctrl
10497090	Ankrd13c	NM_001013806	0.000184038	1.27687	E11.5 * Sox10 up vs E11.5 * Ctrl

10356339	Pde6d	NM_008801	0.0100616	1.27684	E11.5 * Sox10 up vs E11.5 * Ctrl
10434925	Hes1	NM_008235	0.000211866	1.2768	E11.5 * Sox10 up vs E11.5 * Ctrl
10584071	Prdm10	NM_001080817	8.3809e-005	1.27679	E11.5 * Sox10 up vs E11.5 * Ctrl
10557628	Zfp689	NM_175163	0.0014472	1.27672	E11.5 * Sox10 up vs E11.5 * Ctrl
10597354	Pdcd6ip	NM_011052	0.000207217	1.27651	E11.5 * Sox10 up vs E11.5 * Ctrl
10546853	Srgap3	NM_080448	0.000883144	1.27629	E11.5 * Sox10 up vs E11.5 * Ctrl
10411899	2410002O22Rik	NM_025879	0.00195308	1.27628	E11.5 * Sox10 up vs E11.5 * Ctrl
10514732	Slc35d1	NM_177732	0.00463961	1.276	E11.5 * Sox10 up vs E11.5 * Ctrl
10488912	Edem2	NM_145537	0.00262922	1.2759	E11.5 * Sox10 up vs E11.5 * Ctrl
10483648	Ola1	NM_025942	0.00276323	1.2756	E11.5 * Sox10 up vs E11.5 * Ctrl
10360076	Ndufs2	NM_153064	0.000354212	1.27552	E11.5 * Sox10 up vs E11.5 * Ctrl
10476582	Macrod2	NM_028387	3.12444e-005	1.27539	E11.5 * Sox10 up vs E11.5 * Ctrl
10575476	Vac14	NM_146216	0.0011438	1.2753	E11.5 * Sox10 up vs E11.5 * Ctrl
10574676	Nol3	NM_030152	0.00362242	1.27517	E11.5 * Sox10 up vs E11.5 * Ctrl
10575630	Cntnap4	NM_130457	0.00830704	1.27516	E11.5 * Sox10 up vs E11.5 * Ctrl
10359677	Blzf1	NM_025505	0.00714897	1.27515	E11.5 * Sox10 up vs E11.5 * Ctrl
10505132	Akap2	NM_001035533	9.70454e-005	1.27511	E11.5 * Sox10 up vs E11.5 * Ctrl
10373454	Pa2g4	NM_011119	0.000104024	1.27495	E11.5 * Sox10 up vs E11.5 * Ctrl
10369877	Ube2d1	NM_145420	0.000203997	1.27479	E11.5 * Sox10 up vs E11.5 * Ctrl
10533729	Vps37b	NM_177876	0.00519266	1.27468	E11.5 * Sox10 up vs E11.5 * Ctrl
10433219	Nat15	NM_029090	0.00119227	1.27468	E11.5 * Sox10 up vs E11.5 * Ctrl
10475708	Blvra	NM_026678	0.00117642	1.2744	E11.5 * Sox10 up vs E11.5 * Ctrl
10516894	Rcc1	NM_133878	0.0050898	1.27438	E11.5 * Sox10 up vs E11.5 * Ctrl
10477897	Dlgap4	NM_146128	8.17523e-008	1.27438	E11.5 * Sox10 up vs E11.5 * Ctrl
10597871	Higd1a	NM_019814	0.00474119	1.27413	E11.5 * Sox10 up vs E11.5 * Ctrl
10483770	Lnp	NM_001110209	0.00313357	1.274	E11.5 * Sox10 up vs E11.5 * Ctrl
10407955	Epdr1	NM_134065	0.00805194	1.27389	E11.5 * Sox10 up vs E11.5 * Ctrl
10446074	Uhrf1	NM_010931	0.00349185	1.27377	E11.5 * Sox10 up vs E11.5 * Ctrl
10413250	Cphx	NM_175342	0.00974324	1.27372	E11.5 * Sox10 up vs E11.5 * Ctrl
10500054	Psmd4	NM_008951	0.00299412	1.27334	E11.5 * Sox10 up vs E11.5 * Ctrl
10481453	Dolk	NM_177648	0.00343957	1.27324	E11.5 * Sox10 up vs E11.5 * Ctrl
10349512	Ubxn4	NM_026390	2.77372e-006	1.27322	E11.5 * Sox10 up vs E11.5 * Ctrl
10545450	Tgoln1	NM_009443	0.000163796	1.27311	E11.5 * Sox10 up vs E11.5 * Ctrl
10392936	Nt5c	NM_015807	0.00018871	1.273	E11.5 * Sox10 up vs E11.5 * Ctrl
10441342	1810020G14Rik	ENSMUST00000	0.00149995	1.27295	E11.5 * Sox10 up vs E11.5 * Ctrl
10604451	Enox2	NM_145951	0.000487012	1.27274	E11.5 * Sox10 up vs E11.5 * Ctrl
10369761	Reep3	NM_178606	0.000134516	1.2725	E11.5 * Sox10 up vs E11.5 * Ctrl
10411332	Hmgcr	NM_008255	0.00889933	1.27236	E11.5 * Sox10 up vs E11.5 * Ctrl
10469581	Etl4	NM_001081006	0.000111259	1.27228	E11.5 * Sox10 up vs E11.5 * Ctrl
10380660	Hoxb2	NM_134032	0.00275338	1.27173	E11.5 * Sox10 up vs E11.5 * Ctrl
10449312	ENSMUSG00000	NM_001122657	0.00261957	1.2717	E11.5 * Sox10 up vs E11.5 * Ctrl
10396161	Tmx1	NM_028339	0.00285403	1.27145	E11.5 * Sox10 up vs E11.5 * Ctrl
10415159	Thtpa	NM_153083	0.00210589	1.27125	E11.5 * Sox10 up vs E11.5 * Ctrl
10447551	5730437N04Rik	NM_027457	0.00843456	1.27084	E11.5 * Sox10 up vs E11.5 * Ctrl
10516778	Zcchc17	NM_153160	0.000959108	1.27082	E11.5 * Sox10 up vs E11.5 * Ctrl
10605740	Eif2s3x	NM_012010	0.000282726	1.27049	E11.5 * Sox10 up vs E11.5 * Ctrl
10535502	Jtv1	NM_146165	0.00180976	1.27048	E11.5 * Sox10 up vs E11.5 * Ctrl
10516678	Kpna6	NM_008468	0.00297421	1.27028	E11.5 * Sox10 up vs E11.5 * Ctrl
10603843	Syn1	NM_013680	0.0022122	1.27006	E11.5 * Sox10 up vs E11.5 * Ctrl
10591947	Acad8	NM_025862	8.44916e-005	1.26995	E11.5 * Sox10 up vs E11.5 * Ctrl
10456723	BC031181	BC016084	0.00354394	1.2699	E11.5 * Sox10 up vs E11.5 * Ctrl
10446334	Glcci1	NM_133236	0.00758387	1.26985	E11.5 * Sox10 up vs E11.5 * Ctrl
10520800	Gpn1	NM_133756	4.9598e-005	1.26957	E11.5 * Sox10 up vs E11.5 * Ctrl
10496621	Gtf2b	NM_145546	0.00123452	1.26941	E11.5 * Sox10 up vs E11.5 * Ctrl
10438478	Abcc5	NM_013790	8.17866e-005	1.26939	E11.5 * Sox10 up vs E11.5 * Ctrl
10582669	Ttc13	NM_145607	0.00019182	1.26922	E11.5 * Sox10 up vs E11.5 * Ctrl
10529313	Letm1	NM_019694	0.000346878	1.26894	E11.5 * Sox10 up vs E11.5 * Ctrl
10401181	Rdh11	NM_021557	0.00195899	1.26888	E11.5 * Sox10 up vs E11.5 * Ctrl
10436348	Tomm70a	NM_138599	0.000502468	1.26876	E11.5 * Sox10 up vs E11.5 * Ctrl
10454786	Ctnna1	NM_009818	0.000364425	1.26875	E11.5 * Sox10 up vs E11.5 * Ctrl
10409645	Ubqln1	NM_152234	5.98199e-006	1.26874	E11.5 * Sox10 up vs E11.5 * Ctrl
10376094		---	0.000163465	1.26837	E11.5 * Sox10 up vs E11.5 * Ctrl
10401136	EG230765	XR_032827	0.0015309	1.26836	E11.5 * Sox10 up vs E11.5 * Ctrl
10542445	Strap	NM_011499	1.81511e-006	1.26823	E11.5 * Sox10 up vs E11.5 * Ctrl
10430593	Josd1	NM_028792	0.00115772	1.26821	E11.5 * Sox10 up vs E11.5 * Ctrl
10547758	Emg1	NM_013536	0.000232915	1.26818	E11.5 * Sox10 up vs E11.5 * Ctrl

10577623	Gins4	NM_024240	0.00529766	1.26812	E11.5 * Sox10 up vs E11.5 * Ctrl
10375290	Slu7	NM_148673	0.00109318	1.268	E11.5 * Sox10 up vs E11.5 * Ctrl
10525751	Ddx55	NM_026409	0.00219748	1.26796	E11.5 * Sox10 up vs E11.5 * Ctrl
10373313	Nab2	NM_008668	0.00941156	1.2678	E11.5 * Sox10 up vs E11.5 * Ctrl
10419810	Psmb5	NM_011186	0.000318303	1.26778	E11.5 * Sox10 up vs E11.5 * Ctrl
10514366	Glrx3	NM_023140	0.00908165	1.26776	E11.5 * Sox10 up vs E11.5 * Ctrl
10461164	Wdr74	NM_134139	0.00266838	1.26773	E11.5 * Sox10 up vs E11.5 * Ctrl
10525726	2810006K23Rik	NM_028310	0.00656742	1.26766	E11.5 * Sox10 up vs E11.5 * Ctrl
10499705	Hax1	NM_011826	0.00013394	1.26762	E11.5 * Sox10 up vs E11.5 * Ctrl
10460631	Rela	NM_009045	0.00283761	1.26758	E11.5 * Sox10 up vs E11.5 * Ctrl
10545576	Mrpl19	NM_026490	0.00269636	1.26752	E11.5 * Sox10 up vs E11.5 * Ctrl
10534021	Rimbp2	NM_001081388	1.39944e-005	1.2675	E11.5 * Sox10 up vs E11.5 * Ctrl
10475264	Ccndbp1	NM_010761	0.00554979	1.26744	E11.5 * Sox10 up vs E11.5 * Ctrl
10393320	Ube2o	NM_173755	0.000475813	1.26735	E11.5 * Sox10 up vs E11.5 * Ctrl
10585721	Scamp2	NM_022813	0.00234466	1.26707	E11.5 * Sox10 up vs E11.5 * Ctrl
10421012	Ppp2r2a	NM_028032	2.2594e-005	1.26693	E11.5 * Sox10 up vs E11.5 * Ctrl
10573401	Trmt1	NM_198020	0.000675789	1.26675	E11.5 * Sox10 up vs E11.5 * Ctrl
10406245		---	9.06346e-005	1.2666	E11.5 * Sox10 up vs E11.5 * Ctrl
10543118	Glcci1	NM_133236	0.00160038	1.26626	E11.5 * Sox10 up vs E11.5 * Ctrl
10578746	BC088983	BC088983	0.00537876	1.26619	E11.5 * Sox10 up vs E11.5 * Ctrl
10512884		---	0.00487211	1.26618	E11.5 * Sox10 up vs E11.5 * Ctrl
10593842	Tspan3	NM_019793	2.50136e-006	1.26596	E11.5 * Sox10 up vs E11.5 * Ctrl
10395074	Myt1l	NM_001093775	0.000157295	1.26592	E11.5 * Sox10 up vs E11.5 * Ctrl
10417053	Mbnl2	NM_175341	0.000122948	1.26583	E11.5 * Sox10 up vs E11.5 * Ctrl
10558740	OTTMUSG00000	XM_001480612	0.00467714	1.2657	E11.5 * Sox10 up vs E11.5 * Ctrl
10606868	Bex1	NM_009052	0.00645975	1.26553	E11.5 * Sox10 up vs E11.5 * Ctrl
10474870	Rpusd2	NM_173450	0.000294185	1.26527	E11.5 * Sox10 up vs E11.5 * Ctrl
10496475	Adh5	NM_007410	9.9408e-008	1.2652	E11.5 * Sox10 up vs E11.5 * Ctrl
10478938	Hax1	NM_011826	2.81963e-005	1.26516	E11.5 * Sox10 up vs E11.5 * Ctrl
10501397	Tmem167b	NM_026198	0.00602929	1.26448	E11.5 * Sox10 up vs E11.5 * Ctrl
10527268	Pms2	NM_008886	0.00981387	1.26447	E11.5 * Sox10 up vs E11.5 * Ctrl
10585860	Adpgk	NM_028121	0.00239783	1.26407	E11.5 * Sox10 up vs E11.5 * Ctrl
10388492	Vps53	NM_026664	2.61835e-005	1.26351	E11.5 * Sox10 up vs E11.5 * Ctrl
10581926	Adat1	NM_013925	0.00870402	1.26339	E11.5 * Sox10 up vs E11.5 * Ctrl
10423346	Zfp622	NM_144523	0.000196974	1.26333	E11.5 * Sox10 up vs E11.5 * Ctrl
10369086	Gopc	NM_053187	7.42835e-006	1.26305	E11.5 * Sox10 up vs E11.5 * Ctrl
10438603	Igf2bp2	NM_183029	0.00242727	1.26301	E11.5 * Sox10 up vs E11.5 * Ctrl
10433691	Ntan1	NM_010946	0.000286329	1.26298	E11.5 * Sox10 up vs E11.5 * Ctrl
10456021	Camk2a	NM_177407	0.00294793	1.26293	E11.5 * Sox10 up vs E11.5 * Ctrl
10433953	Ypel1	NM_023249	0.00167003	1.26285	E11.5 * Sox10 up vs E11.5 * Ctrl
10539159	Suclg1	NM_019879	0.00288863	1.26281	E11.5 * Sox10 up vs E11.5 * Ctrl
10522661	C530008M17Rik	AK122469	0.00529796	1.2628	E11.5 * Sox10 up vs E11.5 * Ctrl
10477012	Fkbp1a	NM_008019	0.000709567	1.26254	E11.5 * Sox10 up vs E11.5 * Ctrl
10496789	Lpar3	NM_022983	0.00108208	1.26252	E11.5 * Sox10 up vs E11.5 * Ctrl
10432492	Faim2	NM_028224	0.00930665	1.26246	E11.5 * Sox10 up vs E11.5 * Ctrl
10495869	Tram1l1	NM_146140	0.00447701	1.26235	E11.5 * Sox10 up vs E11.5 * Ctrl
10411782	Pik3r1	NM_001077495	0.000852626	1.26217	E11.5 * Sox10 up vs E11.5 * Ctrl
10446594		---	0.00834338	1.26175	E11.5 * Sox10 up vs E11.5 * Ctrl
10423333	Fam134b	NM_001034851	0.00282555	1.26163	E11.5 * Sox10 up vs E11.5 * Ctrl
10580986	Rbmxrt	NM_009033	0.000549812	1.26157	E11.5 * Sox10 up vs E11.5 * Ctrl
10485294	Hsd17b12	NM_019657	0.00781612	1.26126	E11.5 * Sox10 up vs E11.5 * Ctrl
10490569	Kcnq2	NM_010611	0.000999152	1.26113	E11.5 * Sox10 up vs E11.5 * Ctrl
10577560	Ikbkb	NM_010546	0.000105397	1.26108	E11.5 * Sox10 up vs E11.5 * Ctrl
10379410	Rhot1	NM_021536	0.000725791	1.26094	E11.5 * Sox10 up vs E11.5 * Ctrl
10384885	Spnb2	NM_175836	4.11218e-005	1.26077	E11.5 * Sox10 up vs E11.5 * Ctrl
10568099	Tmem219	NM_026827	0.00207008	1.26075	E11.5 * Sox10 up vs E11.5 * Ctrl
10477187	Tpx2	NM_001141977	0.00120432	1.26066	E11.5 * Sox10 up vs E11.5 * Ctrl
10542824	Mrps35	NM_145573	0.0062915	1.26032	E11.5 * Sox10 up vs E11.5 * Ctrl
10453512	Kpna2	NM_010655	0.000626449	1.26023	E11.5 * Sox10 up vs E11.5 * Ctrl
10603618		---	0.00824293	1.26023	E11.5 * Sox10 up vs E11.5 * Ctrl
10497503	Kpna2	NM_010655	0.000342172	1.2602	E11.5 * Sox10 up vs E11.5 * Ctrl
10379564	Lig3	NM_010716	0.000446365	1.26019	E11.5 * Sox10 up vs E11.5 * Ctrl
10467766	Loxl4	ENSMUST00000	0.00669005	1.2599	E11.5 * Sox10 up vs E11.5 * Ctrl
10531899	Klhl8	NM_178741	0.00269519	1.25977	E11.5 * Sox10 up vs E11.5 * Ctrl
10376959	Elac2	NM_023479	0.00196484	1.25973	E11.5 * Sox10 up vs E11.5 * Ctrl
10585022	Zfp259	NM_011752	0.000828222	1.25953	E11.5 * Sox10 up vs E11.5 * Ctrl

10544243	Mrps33	NM_010270	0.000615856	1.25951	E11.5 * Sox10 up vs E11.5 * Ctrl
10401968	Galc	NM_008079	0.00854687	1.25931	E11.5 * Sox10 up vs E11.5 * Ctrl
10445229		---	0.00860991	1.25913	E11.5 * Sox10 up vs E11.5 * Ctrl
10386359	Guk1	NM_008193	0.00878249	1.25901	E11.5 * Sox10 up vs E11.5 * Ctrl
10605659	OTTMUSG00000	ENSMUST00000	0.00781063	1.25883	E11.5 * Sox10 up vs E11.5 * Ctrl
10594988	Mapk6	NM_015806	7.13772e-006	1.2588	E11.5 * Sox10 up vs E11.5 * Ctrl
10571288	Gtf2e2	NM_026584	7.57806e-005	1.25875	E11.5 * Sox10 up vs E11.5 * Ctrl
10420730	Fdft1	NM_010191	0.00126479	1.25874	E11.5 * Sox10 up vs E11.5 * Ctrl
10521163	Gm1673	BC147444	0.00271232	1.25851	E11.5 * Sox10 up vs E11.5 * Ctrl
10373027	Tspan31	NM_025982	0.00134806	1.2585	E11.5 * Sox10 up vs E11.5 * Ctrl
10452000	Dpp9	NM_172624	0.00453062	1.25842	E11.5 * Sox10 up vs E11.5 * Ctrl
10442435	Rnps1	NM_009070	0.0025753	1.25771	E11.5 * Sox10 up vs E11.5 * Ctrl
10355454	EG383528	XR_033954	0.00611633	1.25727	E11.5 * Sox10 up vs E11.5 * Ctrl
10345666	Pdcl3	NM_026850	0.000472846	1.25704	E11.5 * Sox10 up vs E11.5 * Ctrl
10458213	Etf1	NM_144866	3.38144e-005	1.25694	E11.5 * Sox10 up vs E11.5 * Ctrl
10532892	1500011B03Rik	ENSMUST00000	5.50155e-005	1.25691	E11.5 * Sox10 up vs E11.5 * Ctrl
10402519	Atg2b	NM_029654	0.000232022	1.25688	E11.5 * Sox10 up vs E11.5 * Ctrl
10560315	Ppp5c	NM_011155	0.00633697	1.25682	E11.5 * Sox10 up vs E11.5 * Ctrl
10468309	2310014D11Rik	AK009333	0.00277444	1.25665	E11.5 * Sox10 up vs E11.5 * Ctrl
10496110	Papss1	NM_011863	0.00547959	1.25657	E11.5 * Sox10 up vs E11.5 * Ctrl
10517488	Ephb2	NM_010142	0.00473602	1.25655	E11.5 * Sox10 up vs E11.5 * Ctrl
10374348	OTTMUSG00000	XR_032931	0.000424302	1.25645	E11.5 * Sox10 up vs E11.5 * Ctrl
10575184	Wwp2	NM_025830	0.00552691	1.25644	E11.5 * Sox10 up vs E11.5 * Ctrl
10525296	Brap	NM_028227	0.000132706	1.25633	E11.5 * Sox10 up vs E11.5 * Ctrl
10391768	Eftud2	NM_011431	0.00292225	1.25621	E11.5 * Sox10 up vs E11.5 * Ctrl
10433264	Glis2	NM_031184	0.00199222	1.25601	E11.5 * Sox10 up vs E11.5 * Ctrl
10542200	Gabarapl1	NM_020590	0.00145877	1.25561	E11.5 * Sox10 up vs E11.5 * Ctrl
10521927	Tbc1d19	NM_144517	0.0011048	1.25561	E11.5 * Sox10 up vs E11.5 * Ctrl
10368782	Fig4	NM_133999	0.00658677	1.25554	E11.5 * Sox10 up vs E11.5 * Ctrl
10539861	Rpn1	NM_133933	9.84185e-005	1.25552	E11.5 * Sox10 up vs E11.5 * Ctrl
10591357	Eif3g	NM_016876	0.0018296	1.25533	E11.5 * Sox10 up vs E11.5 * Ctrl
10508420	Yars	NM_134151	0.00478855	1.25525	E11.5 * Sox10 up vs E11.5 * Ctrl
10404578	Cdyl	NM_009881	0.000460878	1.25513	E11.5 * Sox10 up vs E11.5 * Ctrl
10418053	Kcnma1	NM_010610	0.00476005	1.25494	E11.5 * Sox10 up vs E11.5 * Ctrl
10399228	EG627245	XR_034503	0.00772107	1.25485	E11.5 * Sox10 up vs E11.5 * Ctrl
10419216	Gnpnat1	NM_019425	0.00343342	1.25481	E11.5 * Sox10 up vs E11.5 * Ctrl
10571653	Actg1	NM_009609	1.30496e-005	1.25481	E11.5 * Sox10 up vs E11.5 * Ctrl
10459730	Me2	NM_145494	0.00125586	1.25466	E11.5 * Sox10 up vs E11.5 * Ctrl
10461497	Ddb1	NM_015735	0.00203648	1.25464	E11.5 * Sox10 up vs E11.5 * Ctrl
10546805	Ddx18	NM_025860	0.00037506	1.25454	E11.5 * Sox10 up vs E11.5 * Ctrl
10541002	D6Wsu116e	BC056942	0.00046046	1.25447	E11.5 * Sox10 up vs E11.5 * Ctrl
10363392	Dnajb12	NM_019965	0.00383655	1.25446	E11.5 * Sox10 up vs E11.5 * Ctrl
10598882	Uba1	NM_009457	2.56048e-006	1.25446	E11.5 * Sox10 up vs E11.5 * Ctrl
10380238	Mrps23	NM_024174	0.00572793	1.25428	E11.5 * Sox10 up vs E11.5 * Ctrl
10503915	Chchd2	NM_024166	0.00912902	1.25417	E11.5 * Sox10 up vs E11.5 * Ctrl
10347106	Rpe	NM_025683	0.000314373	1.25405	E11.5 * Sox10 up vs E11.5 * Ctrl
10434643	Psmb3	NM_011971	0.000341414	1.25399	E11.5 * Sox10 up vs E11.5 * Ctrl
10422608	Oxct1	NM_024188	4.03474e-007	1.25391	E11.5 * Sox10 up vs E11.5 * Ctrl
10437765	Cpped1	NM_146067	0.00977126	1.25376	E11.5 * Sox10 up vs E11.5 * Ctrl
10446596	Dlgap1	NM_177639	0.00797795	1.25339	E11.5 * Sox10 up vs E11.5 * Ctrl
10411199	Aggf1	NM_025630	0.00218986	1.25325	E11.5 * Sox10 up vs E11.5 * Ctrl
10349249	Clasp1	NM_001081276	0.000797067	1.25325	E11.5 * Sox10 up vs E11.5 * Ctrl
10431113	Sult4a1	NM_013873	0.00975701	1.25323	E11.5 * Sox10 up vs E11.5 * Ctrl
10511661	Otud6b	NM_152812	0.000879895	1.25227	E11.5 * Sox10 up vs E11.5 * Ctrl
10588669	Rassf1	NM_019713	0.00857958	1.25225	E11.5 * Sox10 up vs E11.5 * Ctrl
10388971	Utp6	NM_144826	9.15848e-005	1.25223	E11.5 * Sox10 up vs E11.5 * Ctrl
10458913	Cep120	NM_178686	0.000963397	1.25223	E11.5 * Sox10 up vs E11.5 * Ctrl
10511580	Ppm2c	NM_001098230	0.00085862	1.25208	E11.5 * Sox10 up vs E11.5 * Ctrl
10514221	Adfp	NM_007408	0.00143595	1.25184	E11.5 * Sox10 up vs E11.5 * Ctrl
10418171	Zcchc24	NM_001101433	0.00253261	1.25183	E11.5 * Sox10 up vs E11.5 * Ctrl
10412913	EG667344	XR_031629	0.00701744	1.25179	E11.5 * Sox10 up vs E11.5 * Ctrl
10434029	Lztr1	NM_025808	0.00166298	1.25147	E11.5 * Sox10 up vs E11.5 * Ctrl
10591009	Med17	NM_144933	0.000420867	1.2511	E11.5 * Sox10 up vs E11.5 * Ctrl
10526381	Mdh2	NM_008617	0.000811908	1.251	E11.5 * Sox10 up vs E11.5 * Ctrl
10569203	Chid1	NM_001142681	0.00289199	1.25092	E11.5 * Sox10 up vs E11.5 * Ctrl
10366310	Osbpl8	NM_175489	0.00868794	1.25083	E11.5 * Sox10 up vs E11.5 * Ctrl

10465625	Otub1	NM_134150	0.00503153	1.25058	E11.5 * Sox10 up vs E11.5 * Ctrl
10560260	Sae1	NM_019748	0.00422845	1.25052	E11.5 * Sox10 up vs E11.5 * Ctrl
10517421	Pnrc2	NM_026383	0.00011697	1.2505	E11.5 * Sox10 up vs E11.5 * Ctrl
10384123	Ddx56	NM_026538	0.000794049	1.25031	E11.5 * Sox10 up vs E11.5 * Ctrl
10422628	Plcxd3	NM_177355	0.00356459	1.25031	E11.5 * Sox10 up vs E11.5 * Ctrl
10392070	Strada	NM_028126	0.00378999	1.25009	E11.5 * Sox10 up vs E11.5 * Ctrl
10434869	Ccdc50	NM_026202	4.49017e-005	1.25004	E11.5 * Sox10 up vs E11.5 * Ctrl
10439936	Nfkbiz	NM_030612	0.000952646	1.24983	E11.5 * Sox10 up vs E11.5 * Ctrl
10484563	Olfr1043	NM_146577	0.00579745	1.24978	E11.5 * Sox10 up vs E11.5 * Ctrl
10397891	D230037D09Rik	NM_177140	0.00420472	1.24964	E11.5 * Sox10 up vs E11.5 * Ctrl
10503911	Polr1d	NM_181730	2.26608e-005	1.24962	E11.5 * Sox10 up vs E11.5 * Ctrl
10349771	EG638532	XM_914588	0.00818573	1.24957	E11.5 * Sox10 up vs E11.5 * Ctrl
10379075	2610507B11Rik	NM_001002004	2.12902e-005	1.2494	E11.5 * Sox10 up vs E11.5 * Ctrl
10398483	Dync1h1	NM_030238	0.000503243	1.24927	E11.5 * Sox10 up vs E11.5 * Ctrl
10441511			0.0013476	1.24925	E11.5 * Sox10 up vs E11.5 * Ctrl
10388786	Supt6h	NM_009297	6.05931e-005	1.2492	E11.5 * Sox10 up vs E11.5 * Ctrl
10550098	Wdr12	NM_021312	0.00118963	1.24913	E11.5 * Sox10 up vs E11.5 * Ctrl
10477370	Tomm20	NM_024214	0.00387877	1.24898	E11.5 * Sox10 up vs E11.5 * Ctrl
10346810	Pard3b	NM_001081050	0.00443943	1.24893	E11.5 * Sox10 up vs E11.5 * Ctrl
10599612	Phf6	NM_027642	0.000899547	1.24891	E11.5 * Sox10 up vs E11.5 * Ctrl
10437311	Trap1	NM_026508	0.000282693	1.2489	E11.5 * Sox10 up vs E11.5 * Ctrl
10348817	02-Sep	NM_001159719	0.0003628	1.24874	E11.5 * Sox10 up vs E11.5 * Ctrl
10438098	Sdf2l1	NM_022324	0.00338504	1.24863	E11.5 * Sox10 up vs E11.5 * Ctrl
10525766	Gtf2h3	NM_181410	0.00831563	1.24829	E11.5 * Sox10 up vs E11.5 * Ctrl
10478341	Ift52	NM_172150	0.000170236	1.24825	E11.5 * Sox10 up vs E11.5 * Ctrl
10468231	Arl3	NM_019718	0.000324989	1.24784	E11.5 * Sox10 up vs E11.5 * Ctrl
10459837	8030462N17Rik	NM_178670	0.00482896	1.24775	E11.5 * Sox10 up vs E11.5 * Ctrl
10491977	2810046L04Rik	NM_173382	0.005143	1.24729	E11.5 * Sox10 up vs E11.5 * Ctrl
10543080	Rnps1	NM_009070	0.00476488	1.24717	E11.5 * Sox10 up vs E11.5 * Ctrl
10453792	Thoc1	BC024951	0.00350905	1.24717	E11.5 * Sox10 up vs E11.5 * Ctrl
10590343	Trak1	NM_175114	0.0093124	1.24715	E11.5 * Sox10 up vs E11.5 * Ctrl
10358191	Camsap1l1	NM_001081360	1.11874e-005	1.24676	E11.5 * Sox10 up vs E11.5 * Ctrl
10592891	Phldb1	NM_153537	0.00367356	1.24672	E11.5 * Sox10 up vs E11.5 * Ctrl
10507933	Inpp5b	NM_008385	0.000342558	1.24645	E11.5 * Sox10 up vs E11.5 * Ctrl
10513397	1110054O05Rik	NM_001013577	0.000122214	1.24639	E11.5 * Sox10 up vs E11.5 * Ctrl
10532538	Asphd2	NM_028386	0.00941337	1.24624	E11.5 * Sox10 up vs E11.5 * Ctrl
10440037	Nit2	NM_023175	0.00832288	1.24615	E11.5 * Sox10 up vs E11.5 * Ctrl
10500295	Plekho1	NM_023320	0.00116401	1.24606	E11.5 * Sox10 up vs E11.5 * Ctrl
10448064	Prdm9	NM_144809	0.00256943	1.24575	E11.5 * Sox10 up vs E11.5 * Ctrl
10360225	Timm23	NM_016897	1.55106e-005	1.24558	E11.5 * Sox10 up vs E11.5 * Ctrl
10416069	Timm23	NM_016897	1.55106e-005	1.24558	E11.5 * Sox10 up vs E11.5 * Ctrl
10526193		---	0.00353916	1.24533	E11.5 * Sox10 up vs E11.5 * Ctrl
10472764	Dync1i2	NM_010064	0.000100727	1.24513	E11.5 * Sox10 up vs E11.5 * Ctrl
10486712	Zscan29	ENSMUST00000	0.0104105	1.24506	E11.5 * Sox10 up vs E11.5 * Ctrl
10401805	Snw1	NM_025507	1.91871e-005	1.24499	E11.5 * Sox10 up vs E11.5 * Ctrl
10467470	Aldh18a1	NM_019698	0.0009802	1.2448	E11.5 * Sox10 up vs E11.5 * Ctrl
10423512	Fam173b	NM_026546	0.000671805	1.2444	E11.5 * Sox10 up vs E11.5 * Ctrl
10467941	Cwf19l1	NM_001081077	0.00191185	1.24433	E11.5 * Sox10 up vs E11.5 * Ctrl
10566405	Mapksp1	NM_019920	0.00128925	1.2441	E11.5 * Sox10 up vs E11.5 * Ctrl
10345913	Nck2	NM_010879	0.00136864	1.24408	E11.5 * Sox10 up vs E11.5 * Ctrl
10576934	Fam155a	NM_173446	0.00799635	1.24401	E11.5 * Sox10 up vs E11.5 * Ctrl
10449386	D17Wsu92e	NM_001033279	0.000163671	1.24385	E11.5 * Sox10 up vs E11.5 * Ctrl
10563829	Mrps33	NM_010270	0.000666248	1.24371	E11.5 * Sox10 up vs E11.5 * Ctrl
10504450	Glipr2	NM_027450	0.000399967	1.24361	E11.5 * Sox10 up vs E11.5 * Ctrl
10605831	Las1l	NM_152822	0.000267744	1.24354	E11.5 * Sox10 up vs E11.5 * Ctrl
10536176	Ccdc132	NM_024260	0.00620701	1.24314	E11.5 * Sox10 up vs E11.5 * Ctrl
10583647	Dnm2	NM_001039520	0.00216488	1.24295	E11.5 * Sox10 up vs E11.5 * Ctrl
10511069	Gnb1	NM_008142	0.000152495	1.24222	E11.5 * Sox10 up vs E11.5 * Ctrl
10558090	Tacc2	NM_001004468	0.000602726	1.24203	E11.5 * Sox10 up vs E11.5 * Ctrl
10503448	Mmp16	NM_019724	0.00026499	1.24203	E11.5 * Sox10 up vs E11.5 * Ctrl
10539669	Add2	NM_013458	0.000219602	1.24195	E11.5 * Sox10 up vs E11.5 * Ctrl
10378334	Tax1bp3	NM_029564	0.000272767	1.24184	E11.5 * Sox10 up vs E11.5 * Ctrl
10369604	Vps26a	NM_133672	0.00193191	1.24178	E11.5 * Sox10 up vs E11.5 * Ctrl
10354832	Ppil3	NM_027351	0.000260508	1.24135	E11.5 * Sox10 up vs E11.5 * Ctrl
10354792	1110034B05Rik	NR_004860	0.00237949	1.24124	E11.5 * Sox10 up vs E11.5 * Ctrl
10597531	Rbms3	NM_178660	9.80194e-005	1.24119	E11.5 * Sox10 up vs E11.5 * Ctrl

10578763	Sap30	NM_021788	5.25348e-005	1.24106	E11.5 * Sox10 up vs E11.5 * Ctrl
10515295	Mast2	NM_001042743	0.000154628	1.24094	E11.5 * Sox10 up vs E11.5 * Ctrl
10502440		---	0.010198	1.24092	E11.5 * Sox10 up vs E11.5 * Ctrl
10585610	Ptpn9	NM_019651	0.000168418	1.2407	E11.5 * Sox10 up vs E11.5 * Ctrl
10455801	Phax	NM_019996	8.12334e-005	1.24067	E11.5 * Sox10 up vs E11.5 * Ctrl
10533007	Ccdc64	NM_001080808	0.000405227	1.24042	E11.5 * Sox10 up vs E11.5 * Ctrl
10569163	Cend1	NM_021316	0.00921501	1.24024	E11.5 * Sox10 up vs E11.5 * Ctrl
10391577	Hdac5	NM_001077696	0.00534024	1.24021	E11.5 * Sox10 up vs E11.5 * Ctrl
10511311	Tmem68	NM_028097	0.00845077	1.24013	E11.5 * Sox10 up vs E11.5 * Ctrl
10496888		---	0.00886485	1.23983	E11.5 * Sox10 up vs E11.5 * Ctrl
10546396	Mrps25	NM_025578	0.00455402	1.23982	E11.5 * Sox10 up vs E11.5 * Ctrl
10407709	Mtr	NM_001081128	0.00754356	1.23976	E11.5 * Sox10 up vs E11.5 * Ctrl
10352066	Sdccag8	NM_029756	0.000738132	1.23974	E11.5 * Sox10 up vs E11.5 * Ctrl
10525542	Bcl7a	NM_029850	0.00961315	1.23968	E11.5 * Sox10 up vs E11.5 * Ctrl
10558903	Taldo1	NM_011528	0.00213054	1.2396	E11.5 * Sox10 up vs E11.5 * Ctrl
10372583	Rab3ip	NM_001003950	0.000196978	1.23914	E11.5 * Sox10 up vs E11.5 * Ctrl
10373873	Sf3a1	NM_026175	0.00687424	1.23907	E11.5 * Sox10 up vs E11.5 * Ctrl
10472994	Mtx2	NM_016804	8.02921e-005	1.23895	E11.5 * Sox10 up vs E11.5 * Ctrl
10562064	Etv2	NM_007959	0.00500841	1.23868	E11.5 * Sox10 up vs E11.5 * Ctrl
10356628	Hdac4	NM_207225	0.00491916	1.23863	E11.5 * Sox10 up vs E11.5 * Ctrl
10377987	Rabep1	NM_019400	8.42385e-006	1.23841	E11.5 * Sox10 up vs E11.5 * Ctrl
10424400	Myc	NM_010849	0.00798849	1.2383	E11.5 * Sox10 up vs E11.5 * Ctrl
10596137	Srprb	NM_009275	0.00260053	1.23808	E11.5 * Sox10 up vs E11.5 * Ctrl
10346533	Nif3l1	NM_022988	0.00213288	1.23798	E11.5 * Sox10 up vs E11.5 * Ctrl
10392098	Ftsj3	NM_025310	0.00102998	1.23793	E11.5 * Sox10 up vs E11.5 * Ctrl
10437586	Tmem186	NM_025708	0.00737279	1.23788	E11.5 * Sox10 up vs E11.5 * Ctrl
10545720	Stambp	NM_024239	0.00512483	1.23781	E11.5 * Sox10 up vs E11.5 * Ctrl
10530625	OTTMUSG00000	XR_034092	0.00350692	1.23768	E11.5 * Sox10 up vs E11.5 * Ctrl
10424411	Tsg101	NM_021884	0.000438502	1.23752	E11.5 * Sox10 up vs E11.5 * Ctrl
10578477	Fam149a	NM_153535	0.00312863	1.23737	E11.5 * Sox10 up vs E11.5 * Ctrl
10418796	Dph3	NM_172254	0.000215877	1.23733	E11.5 * Sox10 up vs E11.5 * Ctrl
10553301	Ldha	NM_010699	0.00470673	1.2371	E11.5 * Sox10 up vs E11.5 * Ctrl
10419223	Fermt2	NM_146054	0.000199127	1.23698	E11.5 * Sox10 up vs E11.5 * Ctrl
10465604	Stip1	NM_016737	0.000144133	1.23688	E11.5 * Sox10 up vs E11.5 * Ctrl
10567423	Dcun1d3	NM_173408	0.00116804	1.23683	E11.5 * Sox10 up vs E11.5 * Ctrl
10402835	Nudt14	NM_025399	0.00262339	1.23675	E11.5 * Sox10 up vs E11.5 * Ctrl
10494832	5730470L24Rik	NM_025679	0.000175736	1.23663	E11.5 * Sox10 up vs E11.5 * Ctrl
10526838	Got2	NM_010325	0.00122183	1.23661	E11.5 * Sox10 up vs E11.5 * Ctrl
10389164	Pex12	NM_134025	0.00301126	1.2365	E11.5 * Sox10 up vs E11.5 * Ctrl
10492078	Alg5	NM_025442	0.0011696	1.23648	E11.5 * Sox10 up vs E11.5 * Ctrl
10462724	Tnks2	ENSMUST00000	0.000140295	1.2363	E11.5 * Sox10 up vs E11.5 * Ctrl
10378668	Pitpna	NM_008850	9.06088e-006	1.2363	E11.5 * Sox10 up vs E11.5 * Ctrl
10546567	A130022J15Rik	NM_175313	0.00525469	1.23604	E11.5 * Sox10 up vs E11.5 * Ctrl
10576373	Gas8	NM_018855	0.000107099	1.23595	E11.5 * Sox10 up vs E11.5 * Ctrl
10543650	Tnpo3	NM_177296	2.39839e-006	1.23591	E11.5 * Sox10 up vs E11.5 * Ctrl
10370025	Smarcb1	NM_011418	0.000753061	1.2358	E11.5 * Sox10 up vs E11.5 * Ctrl
10589196	Uqcrc1	NM_025407	0.00356663	1.2357	E11.5 * Sox10 up vs E11.5 * Ctrl
10389865	Nme1	NM_008704	7.64618e-006	1.23563	E11.5 * Sox10 up vs E11.5 * Ctrl
10454478	Polr2d	NM_027002	0.0074068	1.23538	E11.5 * Sox10 up vs E11.5 * Ctrl
10586017	Uaca	NM_028283	0.00255797	1.23523	E11.5 * Sox10 up vs E11.5 * Ctrl
10428238	Ubr5	NM_001081359	0.000209913	1.2351	E11.5 * Sox10 up vs E11.5 * Ctrl
10384603	Mdh1	NM_008618	3.29025e-005	1.23509	E11.5 * Sox10 up vs E11.5 * Ctrl
10467328	5730455O13Rik	NM_001081075	0.00332943	1.235	E11.5 * Sox10 up vs E11.5 * Ctrl
10522075	Klhl5	NM_175174	0.000549682	1.23467	E11.5 * Sox10 up vs E11.5 * Ctrl
10363000	Gpx4	NM_001037741	0.00184437	1.2346	E11.5 * Sox10 up vs E11.5 * Ctrl
10530310	Bend4	XM_001477289	0.00425748	1.23458	E11.5 * Sox10 up vs E11.5 * Ctrl
10521031	Ywhah	NM_011738	0.000615127	1.23431	E11.5 * Sox10 up vs E11.5 * Ctrl
10584535	EG235279	XR_034073	0.00572525	1.23419	E11.5 * Sox10 up vs E11.5 * Ctrl
10431726	Yaf2	NM_024189	0.000360677	1.23418	E11.5 * Sox10 up vs E11.5 * Ctrl
10447356	Socs5	NM_019654	0.0022666	1.23379	E11.5 * Sox10 up vs E11.5 * Ctrl
10425632	Xrcc6	NM_010247	0.00169381	1.23313	E11.5 * Sox10 up vs E11.5 * Ctrl
10403466	Dip2c	NM_001081426	0.00465258	1.23306	E11.5 * Sox10 up vs E11.5 * Ctrl
10561673	Spred3	NM_182927	0.00285682	1.23276	E11.5 * Sox10 up vs E11.5 * Ctrl
10350823	Abl2	NM_001136104	0.00247593	1.23275	E11.5 * Sox10 up vs E11.5 * Ctrl
10402096	Ttc7b	NM_001033213	0.001202	1.2327	E11.5 * Sox10 up vs E11.5 * Ctrl
10483381	Stk39	NM_016866	0.000204262	1.23266	E11.5 * Sox10 up vs E11.5 * Ctrl

10358155	2310006M14Rik	AK009188	0.00970253	1.23264	E11.5 * Sox10 up vs E11.5 * Ctrl
10562096	Tmem147	NM_027215	0.00259651	1.23232	E11.5 * Sox10 up vs E11.5 * Ctrl
10582814	Tomm20	NM_024214	0.00227881	1.23227	E11.5 * Sox10 up vs E11.5 * Ctrl
10364675	Gpx4	NM_008162	0.00224632	1.23227	E11.5 * Sox10 up vs E11.5 * Ctrl
10465686	Rtn3	NM_001003934	6.32535e-005	1.23207	E11.5 * Sox10 up vs E11.5 * Ctrl
10558436	BC005624	BC005624	0.00840328	1.23181	E11.5 * Sox10 up vs E11.5 * Ctrl
10369586	Supv3l1	NM_181423	0.00254501	1.2315	E11.5 * Sox10 up vs E11.5 * Ctrl
10542108	Tom1	NM_011622	0.00229304	1.2311	E11.5 * Sox10 up vs E11.5 * Ctrl
10349404	Mgat5	NM_145128	0.00205615	1.23099	E11.5 * Sox10 up vs E11.5 * Ctrl
10389269	Aatf	NM_019816	0.000487297	1.23098	E11.5 * Sox10 up vs E11.5 * Ctrl
10581111	Nae1	NM_144931	0.000481186	1.23093	E11.5 * Sox10 up vs E11.5 * Ctrl
10469951	Rnf208	NM_176834	0.00358841	1.23091	E11.5 * Sox10 up vs E11.5 * Ctrl
10412227	Snx18	NM_130796	0.00127193	1.23072	E11.5 * Sox10 up vs E11.5 * Ctrl
10503882	Rngtt	NM_011884	0.00278249	1.23059	E11.5 * Sox10 up vs E11.5 * Ctrl
10526391	2900083I11Rik	NM_021403	0.00508986	1.23031	E11.5 * Sox10 up vs E11.5 * Ctrl
10593591	Acat1	NM_144784	3.98336e-005	1.23022	E11.5 * Sox10 up vs E11.5 * Ctrl
10458293	Dnajc18	NM_029669	0.00400005	1.23013	E11.5 * Sox10 up vs E11.5 * Ctrl
10416082	Trim35	NM_029979	0.000539357	1.23003	E11.5 * Sox10 up vs E11.5 * Ctrl
10472440	Tax1bp3	NM_029564	0.00029573	1.23002	E11.5 * Sox10 up vs E11.5 * Ctrl
10590549	Exosc7	NM_001081188	0.000823582	1.22995	E11.5 * Sox10 up vs E11.5 * Ctrl
10450605	Tubb5	NM_011655	0.00239116	1.22987	E11.5 * Sox10 up vs E11.5 * Ctrl
10575550	Aars	NM_146217	0.00137241	1.22986	E11.5 * Sox10 up vs E11.5 * Ctrl
10437499	3930401K13Rik	NM_001079814	0.00591694	1.22969	E11.5 * Sox10 up vs E11.5 * Ctrl
10404840	Cd83	NM_009856	0.00404482	1.22962	E11.5 * Sox10 up vs E11.5 * Ctrl
10592856	Ccdc84	NM_201372	0.00398683	1.22956	E11.5 * Sox10 up vs E11.5 * Ctrl
10438959	Lsg1	NM_178069	0.00117999	1.22945	E11.5 * Sox10 up vs E11.5 * Ctrl
10598422	Gripap1	NM_207670	0.00231887	1.2292	E11.5 * Sox10 up vs E11.5 * Ctrl
10445941	100043485	XR_033353	0.00650357	1.22915	E11.5 * Sox10 up vs E11.5 * Ctrl
10558134	Plekha1	NM_133942	0.0025168	1.22905	E11.5 * Sox10 up vs E11.5 * Ctrl
10518361	Smarca5	NM_053124	0.000334824	1.22902	E11.5 * Sox10 up vs E11.5 * Ctrl
10448803	Hn1l	NM_198937	0.000749435	1.22899	E11.5 * Sox10 up vs E11.5 * Ctrl
10538087	Lrrc61	NM_177736	0.00575298	1.22884	E11.5 * Sox10 up vs E11.5 * Ctrl
10499483	Fdps	NM_134469	0.00181331	1.22884	E11.5 * Sox10 up vs E11.5 * Ctrl
10458398	Hars	NM_008214	0.00068304	1.22877	E11.5 * Sox10 up vs E11.5 * Ctrl
10395005	Kidins220	NM_001081378	0.000130702	1.22874	E11.5 * Sox10 up vs E11.5 * Ctrl
10533327	Mapkapk5	NM_010765	0.000493099	1.22861	E11.5 * Sox10 up vs E11.5 * Ctrl
10435226	Snx4	NM_080557	0.00332245	1.22835	E11.5 * Sox10 up vs E11.5 * Ctrl
10474588	2900064A13Rik	BC115477	0.00641051	1.22829	E11.5 * Sox10 up vs E11.5 * Ctrl
10458547	Gnpda1	NM_011937	0.00209012	1.22826	E11.5 * Sox10 up vs E11.5 * Ctrl
10414093	Glud1	NM_008133	0.0021576	1.22823	E11.5 * Sox10 up vs E11.5 * Ctrl
10532157	Tmed5	NM_028876	0.00368512	1.22816	E11.5 * Sox10 up vs E11.5 * Ctrl
10384811	Ccdc104	NM_025740	0.00595319	1.22787	E11.5 * Sox10 up vs E11.5 * Ctrl
10500388	Polr3c	NM_028925	0.00680378	1.22786	E11.5 * Sox10 up vs E11.5 * Ctrl
10555695	Rrm1	NM_009103	3.44097e-005	1.22775	E11.5 * Sox10 up vs E11.5 * Ctrl
10526261	Stx1a	NM_016801	0.00380609	1.22749	E11.5 * Sox10 up vs E11.5 * Ctrl
10604844	Sms	NM_009214	0.000378565	1.22707	E11.5 * Sox10 up vs E11.5 * Ctrl
10550815	V1rd9	NM_030735	0.00779072	1.22701	E11.5 * Sox10 up vs E11.5 * Ctrl
10436446	Gps1	NM_145370	0.00936171	1.22672	E11.5 * Sox10 up vs E11.5 * Ctrl
10469145	Kin	NM_025280	0.00196817	1.22654	E11.5 * Sox10 up vs E11.5 * Ctrl
10392284	Kpna2	NM_010655	0.00130015	1.22653	E11.5 * Sox10 up vs E11.5 * Ctrl
10353803	Ugcgl1	NM_198899	0.00112221	1.2265	E11.5 * Sox10 up vs E11.5 * Ctrl
10348180	Eif4e2	NM_001039169	0.0017528	1.22635	E11.5 * Sox10 up vs E11.5 * Ctrl
10422518	Tmtc4	NM_028651	0.00165213	1.22624	E11.5 * Sox10 up vs E11.5 * Ctrl
10502537	Sh3glb1	NM_019464	1.26208e-005	1.22589	E11.5 * Sox10 up vs E11.5 * Ctrl
10531133	Grsf1	NM_178700	2.3891e-006	1.22586	E11.5 * Sox10 up vs E11.5 * Ctrl
10431266	Cerk	NM_145475	0.00133172	1.22577	E11.5 * Sox10 up vs E11.5 * Ctrl
10523297	Ccng2	NM_007635	0.00904197	1.22564	E11.5 * Sox10 up vs E11.5 * Ctrl
10357242	Dbi	NM_001037999	0.00831674	1.22545	E11.5 * Sox10 up vs E11.5 * Ctrl
10406795	Gfm2	NM_177266	0.000223768	1.22521	E11.5 * Sox10 up vs E11.5 * Ctrl
10414374	Ktn1	NM_008477	0.000173056	1.2252	E11.5 * Sox10 up vs E11.5 * Ctrl
10577226	2610019F03Rik	NM_173744	0.00285029	1.22514	E11.5 * Sox10 up vs E11.5 * Ctrl
10350489	Uchl5	NM_019562	5.28682e-005	1.22495	E11.5 * Sox10 up vs E11.5 * Ctrl
10418804	Timm23	NM_016897	3.71048e-005	1.22494	E11.5 * Sox10 up vs E11.5 * Ctrl
10494857	Nras	NM_010937	8.15784e-005	1.22491	E11.5 * Sox10 up vs E11.5 * Ctrl
10521461	Grpel1	NM_024478	0.00098211	1.2248	E11.5 * Sox10 up vs E11.5 * Ctrl
10589511	Scap	NM_001001144	0.00437963	1.22475	E11.5 * Sox10 up vs E11.5 * Ctrl

10553967	Pcsk6	ENSMUST00000	0.00814936	1.22473	E11.5 * Sox10 up vs E11.5 * Ctrl
10464070	Vti1a	NM_016862	0.00578694	1.22473	E11.5 * Sox10 up vs E11.5 * Ctrl
10558458	Ppp2r2d	NM_026391	0.00154542	1.22466	E11.5 * Sox10 up vs E11.5 * Ctrl
10516605	Hdac1	NM_008228	0.000100749	1.22447	E11.5 * Sox10 up vs E11.5 * Ctrl
10381187	Atp6v0a1	NM_016920	0.00144658	1.22439	E11.5 * Sox10 up vs E11.5 * Ctrl
10497773	Mccc1	NM_023644	0.00352023	1.22435	E11.5 * Sox10 up vs E11.5 * Ctrl
10420035	Ipo4	NM_024267	0.00524009	1.22429	E11.5 * Sox10 up vs E11.5 * Ctrl
10590325	Ctnnb1	NM_007614	0.00281787	1.22415	E11.5 * Sox10 up vs E11.5 * Ctrl
10447708	Qk	NM_021881	4.37373e-005	1.2241	E11.5 * Sox10 up vs E11.5 * Ctrl
10588903	Usp4	NM_011678	7.484e-005	1.22396	E11.5 * Sox10 up vs E11.5 * Ctrl
10481525	Tor1a	NM_144884	0.00730373	1.22383	E11.5 * Sox10 up vs E11.5 * Ctrl
10479765	Suv39h2	NM_022724	0.000270665	1.22382	E11.5 * Sox10 up vs E11.5 * Ctrl
10391567	Tmem101	NM_029649	0.00221587	1.22382	E11.5 * Sox10 up vs E11.5 * Ctrl
10535508	AU022870	BC048077	0.00825973	1.22381	E11.5 * Sox10 up vs E11.5 * Ctrl
10463836	Gsto1	NM_010362	0.00590369	1.2238	E11.5 * Sox10 up vs E11.5 * Ctrl
10579860	Smad1	NM_008539	0.000445125	1.22342	E11.5 * Sox10 up vs E11.5 * Ctrl
10445214	Mut	NM_008650	0.00448134	1.22337	E11.5 * Sox10 up vs E11.5 * Ctrl
10356379	Ecel1	NM_021306	0.00801292	1.22334	E11.5 * Sox10 up vs E11.5 * Ctrl
10375941	Vdac1	NM_011694	0.00462356	1.22313	E11.5 * Sox10 up vs E11.5 * Ctrl
10452937	Heatr5b	NM_001081179	0.000661764	1.2231	E11.5 * Sox10 up vs E11.5 * Ctrl
10402336	Ddx24	NM_001159502	0.00170092	1.22309	E11.5 * Sox10 up vs E11.5 * Ctrl
10606926	1700025D03Rik	NM_026469	1.35464e-005	1.22304	E11.5 * Sox10 up vs E11.5 * Ctrl
10534324	Limk1	NM_010717	0.00291287	1.22299	E11.5 * Sox10 up vs E11.5 * Ctrl
10371307	Nfyb	NM_010914	0.00969904	1.22275	E11.5 * Sox10 up vs E11.5 * Ctrl
10378508	Tsr1	NM_177325	0.000614465	1.22258	E11.5 * Sox10 up vs E11.5 * Ctrl
10413502	Actr8	NM_027493	0.00421162	1.22206	E11.5 * Sox10 up vs E11.5 * Ctrl
10526181	Gatsl2	NM_030719	0.00738691	1.22197	E11.5 * Sox10 up vs E11.5 * Ctrl
10360806	Capn2	NM_009794	0.00904745	1.22195	E11.5 * Sox10 up vs E11.5 * Ctrl
10385343	Ttc1	NM_133795	0.000398799	1.22173	E11.5 * Sox10 up vs E11.5 * Ctrl
10604100	Ndufa1	NM_019443	0.000193588	1.22171	E11.5 * Sox10 up vs E11.5 * Ctrl
10390574	Fbxl20	NM_028149	0.00481099	1.2217	E11.5 * Sox10 up vs E11.5 * Ctrl
10431874	Slc38a1	NM_134086	0.00116654	1.22136	E11.5 * Sox10 up vs E11.5 * Ctrl
10554463	Hddc3	NM_026812	0.00274838	1.22112	E11.5 * Sox10 up vs E11.5 * Ctrl
10361828	Cited2	NM_010828	0.00367463	1.22111	E11.5 * Sox10 up vs E11.5 * Ctrl
10532187	Gak	NM_153569	0.000217486	1.2211	E11.5 * Sox10 up vs E11.5 * Ctrl
10455989	Rbm22	NM_025776	0.000222416	1.22105	E11.5 * Sox10 up vs E11.5 * Ctrl
10414623	E130112L23Rik	NM_198249	0.00159957	1.22101	E11.5 * Sox10 up vs E11.5 * Ctrl
10421672	1200011I18Rik	NM_026177	0.00602626	1.22077	E11.5 * Sox10 up vs E11.5 * Ctrl
10418480	Gnl3	NM_153547	0.00155083	1.22071	E11.5 * Sox10 up vs E11.5 * Ctrl
10502232	Scye1	NM_007926	0.00162405	1.22045	E11.5 * Sox10 up vs E11.5 * Ctrl
10556571	Rpl19	NM_009078	0.000223742	1.22032	E11.5 * Sox10 up vs E11.5 * Ctrl
10516982	Stx12	NM_133887	0.000468365	1.22022	E11.5 * Sox10 up vs E11.5 * Ctrl
10389701	Akap1	NM_009648	0.000856663	1.22019	E11.5 * Sox10 up vs E11.5 * Ctrl
10375667	Rnf130	NM_021540	0.00560521	1.22009	E11.5 * Sox10 up vs E11.5 * Ctrl
10607694	Syap1	NM_025932	0.0046676	1.21999	E11.5 * Sox10 up vs E11.5 * Ctrl
10554445	Prc1	NM_145150	0.00725087	1.21998	E11.5 * Sox10 up vs E11.5 * Ctrl
10493585	Ube2q1	NM_027315	0.000626059	1.21979	E11.5 * Sox10 up vs E11.5 * Ctrl
10569071	Hras1	NM_001130444	0.000293995	1.21963	E11.5 * Sox10 up vs E11.5 * Ctrl
10360884	Iars2	NM_198653	0.00139557	1.2196	E11.5 * Sox10 up vs E11.5 * Ctrl
10550925	Zfp428	NM_146183	0.000157738	1.21954	E11.5 * Sox10 up vs E11.5 * Ctrl
10558847	Phrf1	NM_001081118	0.0035491	1.21953	E11.5 * Sox10 up vs E11.5 * Ctrl
10406718	Wdr41	NM_172590	0.00926832	1.21947	E11.5 * Sox10 up vs E11.5 * Ctrl
10493120	Gpatch4	NM_025663	0.00193134	1.21946	E11.5 * Sox10 up vs E11.5 * Ctrl
10399121	Ptprn2	NM_011215	0.00223152	1.21942	E11.5 * Sox10 up vs E11.5 * Ctrl
10498064	Setd7	NM_080793	0.00287259	1.21908	E11.5 * Sox10 up vs E11.5 * Ctrl
10447004	Hdac1	NM_008228	0.000268446	1.21883	E11.5 * Sox10 up vs E11.5 * Ctrl
10477649	Acss2	NM_019811	0.00651619	1.2185	E11.5 * Sox10 up vs E11.5 * Ctrl
10477630	Dynlrb1	NM_025947	2.74737e-009	1.21847	E11.5 * Sox10 up vs E11.5 * Ctrl
10448292	Thoc6	NM_001008425	0.00335319	1.21831	E11.5 * Sox10 up vs E11.5 * Ctrl
10384183	AB182283	NM_001081652	0.000559339	1.21811	E11.5 * Sox10 up vs E11.5 * Ctrl
10425966	Atxn10	NM_016843	0.000327534	1.21788	E11.5 * Sox10 up vs E11.5 * Ctrl
10544891	Nod1	NM_172729	0.00647683	1.21784	E11.5 * Sox10 up vs E11.5 * Ctrl
10446907	Ttc27	NM_152817	0.0100522	1.21763	E11.5 * Sox10 up vs E11.5 * Ctrl
10388234	Gsg2	NM_010353	0.00306643	1.21749	E11.5 * Sox10 up vs E11.5 * Ctrl
10433536	Clec16a	NM_177562	0.00096748	1.21746	E11.5 * Sox10 up vs E11.5 * Ctrl
10532463	Pus1	NM_001025561	0.00286156	1.21724	E11.5 * Sox10 up vs E11.5 * Ctrl

10549447	Ipo8	NM_001081113	0.00768458	1.21675	E11.5 * Sox10 up vs E11.5 * Ctrl
10586274	2010321M09Rik	NM_175153	0.0012049	1.21669	E11.5 * Sox10 up vs E11.5 * Ctrl
10388132	4933427D14Rik	BC043106	0.000591941	1.21631	E11.5 * Sox10 up vs E11.5 * Ctrl
10451637	1700122O11Rik	ENSMUST00000(	0.000637569	1.21625	E11.5 * Sox10 up vs E11.5 * Ctrl
10607724	Zrsr2	NM_178794	0.000705767	1.21613	E11.5 * Sox10 up vs E11.5 * Ctrl
10547140	Tmcc1	ENSMUST00000(	0.000397574	1.21597	E11.5 * Sox10 up vs E11.5 * Ctrl
10438726	Masp1	ENSMUST00000(	0.00611022	1.21588	E11.5 * Sox10 up vs E11.5 * Ctrl
10424825	Cyc1	NM_025567	0.00474715	1.21584	E11.5 * Sox10 up vs E11.5 * Ctrl
10460312	Cdk2ap2	NM_026373	0.00631639	1.21569	E11.5 * Sox10 up vs E11.5 * Ctrl
10373542	Dgka	NM_016811	0.0035869	1.21563	E11.5 * Sox10 up vs E11.5 * Ctrl
10563615	Hps5	NM_001005247	0.00356933	1.21548	E11.5 * Sox10 up vs E11.5 * Ctrl
10363368	Timm23	NM_016897	5.23115e-005	1.21499	E11.5 * Sox10 up vs E11.5 * Ctrl
10574632	Cbfb	NM_022309	0.000272265	1.2149	E11.5 * Sox10 up vs E11.5 * Ctrl
10559513	Cab39	NM_133781	0.000974451	1.21477	E11.5 * Sox10 up vs E11.5 * Ctrl
10502522	Hs2st1	NM_011828	3.35841e-006	1.21449	E11.5 * Sox10 up vs E11.5 * Ctrl
10485361	B230118H07Rik	NM_026592	0.00768729	1.21434	E11.5 * Sox10 up vs E11.5 * Ctrl
10362171	Stx7	NM_016797	0.00266773	1.21429	E11.5 * Sox10 up vs E11.5 * Ctrl
10426030	Trmu	NM_028063	0.00503346	1.21428	E11.5 * Sox10 up vs E11.5 * Ctrl
10409204	ENSMUSG0000C	XM_001474265	8.85225e-005	1.2141	E11.5 * Sox10 up vs E11.5 * Ctrl
10360506	Akt3	NM_011785	0.000416209	1.21404	E11.5 * Sox10 up vs E11.5 * Ctrl
10467216	Cpeb3	NM_198300	0.00564546	1.21392	E11.5 * Sox10 up vs E11.5 * Ctrl
10534585	Sh2b2	NM_018825	0.00465937	1.21333	E11.5 * Sox10 up vs E11.5 * Ctrl
10385616	Rufy1	NM_172557	0.00552233	1.21312	E11.5 * Sox10 up vs E11.5 * Ctrl
10568260	Zfp629	NM_177226	0.00180809	1.21302	E11.5 * Sox10 up vs E11.5 * Ctrl
10537062	Mest	NM_008590	0.000166192	1.21297	E11.5 * Sox10 up vs E11.5 * Ctrl
10497842	Bbs7	NM_027810	0.000834158	1.21296	E11.5 * Sox10 up vs E11.5 * Ctrl
10384349	Polr2c	NM_009090	0.00891775	1.2129	E11.5 * Sox10 up vs E11.5 * Ctrl
10587211	Leo1	NM_001039522	0.000169345	1.21276	E11.5 * Sox10 up vs E11.5 * Ctrl
10557585	Zfp553	NM_146201	0.00695706	1.21269	E11.5 * Sox10 up vs E11.5 * Ctrl
10355037	Wdr12	NM_021312	0.00175476	1.21228	E11.5 * Sox10 up vs E11.5 * Ctrl
10548176	5930416I19Rik	NR_027360	0.007131	1.2117	E11.5 * Sox10 up vs E11.5 * Ctrl
10585706	Cox5a	NM_007747	0.00793703	1.21165	E11.5 * Sox10 up vs E11.5 * Ctrl
10415574	Ccni	NM_017367	0.000413528	1.21146	E11.5 * Sox10 up vs E11.5 * Ctrl
10460732	Znhit2	NM_013859	0.00434431	1.21123	E11.5 * Sox10 up vs E11.5 * Ctrl
10490826	Zbtb10	ENSMUST00000(	0.00655255	1.21108	E11.5 * Sox10 up vs E11.5 * Ctrl
10427910	Fam105b	NM_001013792	0.00673106	1.21081	E11.5 * Sox10 up vs E11.5 * Ctrl
10495243	Gstm5	NM_010360	0.000802156	1.21075	E11.5 * Sox10 up vs E11.5 * Ctrl
10581159	Fam96b	NM_026753	0.00498777	1.21072	E11.5 * Sox10 up vs E11.5 * Ctrl
10423185	Rnasen	NM_001130149	0.00303441	1.21048	E11.5 * Sox10 up vs E11.5 * Ctrl
10476648	Dstn	NM_019771	0.000949961	1.21044	E11.5 * Sox10 up vs E11.5 * Ctrl
10383485	Gps1	NM_145370	0.00656111	1.2104	E11.5 * Sox10 up vs E11.5 * Ctrl
10488472	2310001A20Rik	AJ310638	0.00411136	1.21036	E11.5 * Sox10 up vs E11.5 * Ctrl
10578582	D030016E14Rik	NM_177240	0.00107227	1.21035	E11.5 * Sox10 up vs E11.5 * Ctrl
10540241	Arl6ip5	NM_022992	0.00866151	1.21021	E11.5 * Sox10 up vs E11.5 * Ctrl
10448700	Gfer	NM_023040	0.00152567	1.20985	E11.5 * Sox10 up vs E11.5 * Ctrl
10461257	Ubxn1	NM_146093	0.00541329	1.20977	E11.5 * Sox10 up vs E11.5 * Ctrl
10397719	Tdp1	NM_028354	0.00213441	1.20974	E11.5 * Sox10 up vs E11.5 * Ctrl
10572880	Tom1	NM_011622	0.0017402	1.20969	E11.5 * Sox10 up vs E11.5 * Ctrl
10357280	Insig2	NM_133748	0.00736817	1.20968	E11.5 * Sox10 up vs E11.5 * Ctrl
10346255	Ormdl1	NM_145517	0.0103489	1.20946	E11.5 * Sox10 up vs E11.5 * Ctrl
10594551	Zfp609	NM_172536	0.00484321	1.20925	E11.5 * Sox10 up vs E11.5 * Ctrl
10585474	Psma4	NM_011966	0.00147852	1.20909	E11.5 * Sox10 up vs E11.5 * Ctrl
10397752	Calm1	NM_009790	1.17699e-006	1.20907	E11.5 * Sox10 up vs E11.5 * Ctrl
10466848	D19Bwg1357e	NM_177474	4.86398e-005	1.20881	E11.5 * Sox10 up vs E11.5 * Ctrl
10360130	Nit1	NM_012049	0.00879905	1.20866	E11.5 * Sox10 up vs E11.5 * Ctrl
10358726	Tsen15	NM_025677	0.00596967	1.20849	E11.5 * Sox10 up vs E11.5 * Ctrl
10499265	Prcc	NM_033573	0.00612822	1.20838	E11.5 * Sox10 up vs E11.5 * Ctrl
10602840	Sh3kbp1	NM_021389	1.66684e-005	1.20828	E11.5 * Sox10 up vs E11.5 * Ctrl
10384672	Ahsa2	NM_172391	0.000613275	1.20816	E11.5 * Sox10 up vs E11.5 * Ctrl
10586477	Ppib	NM_011149	0.000566673	1.20814	E11.5 * Sox10 up vs E11.5 * Ctrl
10549388	Pthlh	NM_008970	0.00713995	1.20776	E11.5 * Sox10 up vs E11.5 * Ctrl
10522265	Slc30a9	NM_178651	0.00782799	1.20764	E11.5 * Sox10 up vs E11.5 * Ctrl
10490946	Hsp90aa1	NM_010480	0.000298524	1.20752	E11.5 * Sox10 up vs E11.5 * Ctrl
10380773	4933428G20Rik	NM_021493	0.00368239	1.20741	E11.5 * Sox10 up vs E11.5 * Ctrl
10360270	Atp1a2	NM_178405	0.000527631	1.20733	E11.5 * Sox10 up vs E11.5 * Ctrl
10385159	Rars	NM_025936	1.70367e-007	1.20731	E11.5 * Sox10 up vs E11.5 * Ctrl

10536297	Ppp1r9a	NM_181595	0.000898286	1.20715	E11.5 * Sox10 up vs E11.5 * Ctrl
10484520	4833423E24Rik	BC120879	0.00405722	1.2071	E11.5 * Sox10 up vs E11.5 * Ctrl
10505927		---	0.00732124	1.20698	E11.5 * Sox10 up vs E11.5 * Ctrl
10425757	1500032L24Rik	BC092006	0.00054421	1.20694	E11.5 * Sox10 up vs E11.5 * Ctrl
10524640	2210016L21Rik	BC031162	0.00730166	1.2067	E11.5 * Sox10 up vs E11.5 * Ctrl
10546349	Xpc	NM_009531	5.6985e-005	1.20648	E11.5 * Sox10 up vs E11.5 * Ctrl
10511113	Cdc2l1	NM_007661	3.33571e-005	1.20644	E11.5 * Sox10 up vs E11.5 * Ctrl
10545394	Rnf181	NM_025607	0.0089099	1.20622	E11.5 * Sox10 up vs E11.5 * Ctrl
10465263	Dpf2	NM_011262	0.000840073	1.20595	E11.5 * Sox10 up vs E11.5 * Ctrl
10539279	Aup1	NM_007517	0.00359238	1.20577	E11.5 * Sox10 up vs E11.5 * Ctrl
10510142	Vmn2r-ps14	NR_002888	0.00134793	1.20576	E11.5 * Sox10 up vs E11.5 * Ctrl
10483584	Mettl8	NM_145524	0.00654302	1.20563	E11.5 * Sox10 up vs E11.5 * Ctrl
10373924	Ascc2	NM_029291	0.000501147	1.20552	E11.5 * Sox10 up vs E11.5 * Ctrl
10388276	Olfr382	NM_146443	0.00388641	1.20499	E11.5 * Sox10 up vs E11.5 * Ctrl
10504926	Rnf20	NM_182999	5.15841e-005	1.20468	E11.5 * Sox10 up vs E11.5 * Ctrl
10370446	Trappc10	NM_001081055	0.010023	1.20464	E11.5 * Sox10 up vs E11.5 * Ctrl
10592734	Cbl	NM_007619	0.0020483	1.20455	E11.5 * Sox10 up vs E11.5 * Ctrl
10527732	Fry	ENSMUST00000(	0.00370371	1.2042	E11.5 * Sox10 up vs E11.5 * Ctrl
10496036	Col25a1	NM_029838	0.00775937	1.20383	E11.5 * Sox10 up vs E11.5 * Ctrl
10384529	Cep68	NM_172260	0.000572128	1.20372	E11.5 * Sox10 up vs E11.5 * Ctrl
10567407	Thumpd1	NM_145585	0.00152577	1.20371	E11.5 * Sox10 up vs E11.5 * Ctrl
10515012	Prpf38a	NM_172697	0.00116134	1.20356	E11.5 * Sox10 up vs E11.5 * Ctrl
10569458	Cars	NM_013742	0.000196493	1.20351	E11.5 * Sox10 up vs E11.5 * Ctrl
10410134	EG665612	XR_033575	0.00297236	1.20346	E11.5 * Sox10 up vs E11.5 * Ctrl
10414433	6720456H20Rik	NM_172600	0.00291696	1.20321	E11.5 * Sox10 up vs E11.5 * Ctrl
10358717	1700025G04Rik	BC034723	0.00039287	1.20313	E11.5 * Sox10 up vs E11.5 * Ctrl
10515836	Ccnb1	NM_172301	0.00937469	1.20308	E11.5 * Sox10 up vs E11.5 * Ctrl
10349316	Tmem185b	NM_146103	0.00728665	1.203	E11.5 * Sox10 up vs E11.5 * Ctrl
10517312	Tmem57	NM_025382	0.0032352	1.20294	E11.5 * Sox10 up vs E11.5 * Ctrl
10468489	Xpnpep1	NM_133216	0.00128885	1.20293	E11.5 * Sox10 up vs E11.5 * Ctrl
10367843	Utrn	NM_011682	0.00281503	1.20292	E11.5 * Sox10 up vs E11.5 * Ctrl
10577534	Vdac3	NM_011696	0.000611034	1.20288	E11.5 * Sox10 up vs E11.5 * Ctrl
10405094	Iars	NM_172015	0.000185019	1.20248	E11.5 * Sox10 up vs E11.5 * Ctrl
10580457	N4bp1	BC004022	0.00756415	1.20217	E11.5 * Sox10 up vs E11.5 * Ctrl
10451238	Polr1c	NM_009085	0.00505199	1.20208	E11.5 * Sox10 up vs E11.5 * Ctrl
10379467	Psmd11	NM_178616	0.000112644	1.20189	E11.5 * Sox10 up vs E11.5 * Ctrl
10496742	2410004B18Rik	NM_025555	0.00239296	1.20188	E11.5 * Sox10 up vs E11.5 * Ctrl
10382010	Wdr68	NM_027946	0.000644898	1.20168	E11.5 * Sox10 up vs E11.5 * Ctrl
10361846	Reps1	NM_009048	0.0051198	1.20166	E11.5 * Sox10 up vs E11.5 * Ctrl
10442155	Ppp2r1a	NM_016891	0.0056731	1.20134	E11.5 * Sox10 up vs E11.5 * Ctrl
10569886	Trappc5	NM_025701	0.00459515	1.2013	E11.5 * Sox10 up vs E11.5 * Ctrl
10584954	Pcsk7	NM_008794	0.00443663	1.20115	E11.5 * Sox10 up vs E11.5 * Ctrl
10363845	Ccdc6	NM_001111121	0.00437467	1.20081	E11.5 * Sox10 up vs E11.5 * Ctrl
10424624	Bai1	NM_174991	0.00830958	1.20064	E11.5 * Sox10 up vs E11.5 * Ctrl
10382069	Psmc5	NM_008950	1.30073e-005	1.20055	E11.5 * Sox10 up vs E11.5 * Ctrl
10596857	Apeh	NM_146226	0.00551316	1.20042	E11.5 * Sox10 up vs E11.5 * Ctrl
10431802	Twf1	NM_008971	0.000495956	1.20019	E11.5 * Sox10 up vs E11.5 * Ctrl
10514374	EG664886	XR_030634	0.00549675	1.20015	E11.5 * Sox10 up vs E11.5 * Ctrl
10523529	Cops4	NM_012001	0.000266538	1.20009	E11.5 * Sox10 up vs E11.5 * Ctrl
10454655	Apc	NM_007462	0.00214738	1.20002	E11.5 * Sox10 up vs E11.5 * Ctrl
10425161	Lgals1	NM_008495	0.000626579	1.19997	E11.5 * Sox10 up vs E11.5 * Ctrl
10405971	Nsun2	NM_145354	9.90587e-005	1.19973	E11.5 * Sox10 up vs E11.5 * Ctrl
10379998	Trim37	NM_197987	0.00090738	1.19963	E11.5 * Sox10 up vs E11.5 * Ctrl
10461334	Mta2	NM_011842	0.000985042	1.1994	E11.5 * Sox10 up vs E11.5 * Ctrl
10421694		---	0.00624022	1.19928	E11.5 * Sox10 up vs E11.5 * Ctrl
10591139	Naalad2	NM_028279	0.00547612	1.19925	E11.5 * Sox10 up vs E11.5 * Ctrl
10401244	Actn1	NM_134156	0.0027139	1.19921	E11.5 * Sox10 up vs E11.5 * Ctrl
10465336	Mrpl49	NM_026246	0.00945803	1.19921	E11.5 * Sox10 up vs E11.5 * Ctrl
10535282	Card11	NM_175362	0.00604183	1.1992	E11.5 * Sox10 up vs E11.5 * Ctrl
10461354	Tut1	NM_197993	0.00961488	1.19913	E11.5 * Sox10 up vs E11.5 * Ctrl
10502419	Rap1gds1	NM_001040690	0.00592851	1.19874	E11.5 * Sox10 up vs E11.5 * Ctrl
10348866	Atg4b	NM_174874	0.00441426	1.19874	E11.5 * Sox10 up vs E11.5 * Ctrl
10382139	Psmd12	NM_025894	1.8012e-006	1.19861	E11.5 * Sox10 up vs E11.5 * Ctrl
10557432	G730046D07Rik	AK144875	0.0061347	1.19828	E11.5 * Sox10 up vs E11.5 * Ctrl
10360454	Opn3	NM_010098	0.00974343	1.19827	E11.5 * Sox10 up vs E11.5 * Ctrl
10593103	Rnf214	NM_178709	0.00230971	1.19821	E11.5 * Sox10 up vs E11.5 * Ctrl

10400538	Trappc6b	NM_030057	0.00486671	1.19797	E11.5 * Sox10 up vs E11.5 * Ctrl
10454709	Kif20a	NM_009004	7.2297e-005	1.19795	E11.5 * Sox10 up vs E11.5 * Ctrl
10580370	Dnaja2	NM_019794	3.68287e-006	1.19784	E11.5 * Sox10 up vs E11.5 * Ctrl
10529873	Rab2a	NM_021518	3.31449e-005	1.19775	E11.5 * Sox10 up vs E11.5 * Ctrl
10452905		---	0.00188122	1.19754	E11.5 * Sox10 up vs E11.5 * Ctrl
10416269	Ppp3cc	ENSMUST00000(	0.00405617	1.19729	E11.5 * Sox10 up vs E11.5 * Ctrl
10407921	Vdac3	NM_011696	0.000424771	1.1972	E11.5 * Sox10 up vs E11.5 * Ctrl
10407926	Vdac3	NM_011696	0.000424771	1.1972	E11.5 * Sox10 up vs E11.5 * Ctrl
10393222	Srp68	NM_146032	0.00573509	1.19698	E11.5 * Sox10 up vs E11.5 * Ctrl
10547531	Bid	NM_007544	0.00855457	1.19697	E11.5 * Sox10 up vs E11.5 * Ctrl
10510305	Frap1	NM_020009	0.00765689	1.19691	E11.5 * Sox10 up vs E11.5 * Ctrl
10393021	Recql5	NM_130454	0.00244829	1.19669	E11.5 * Sox10 up vs E11.5 * Ctrl
10370259	Col18a1	NM_009929	0.00479503	1.19668	E11.5 * Sox10 up vs E11.5 * Ctrl
10572637	Fam125a	NM_028617	0.00590489	1.19622	E11.5 * Sox10 up vs E11.5 * Ctrl
10403756	1600012F09Rik	NM_025904	0.00522405	1.19595	E11.5 * Sox10 up vs E11.5 * Ctrl
10407916	1600012F09Rik	NM_025904	0.00522405	1.19595	E11.5 * Sox10 up vs E11.5 * Ctrl
10374564	Cct4	NM_009837	0.000132164	1.1958	E11.5 * Sox10 up vs E11.5 * Ctrl
10380524	Slc35b1	NM_016752	0.000373497	1.1958	E11.5 * Sox10 up vs E11.5 * Ctrl
10537078	Mkln1	NM_013791	0.00057428	1.19577	E11.5 * Sox10 up vs E11.5 * Ctrl
10471438	Dpm2	NM_010073	0.00551491	1.19558	E11.5 * Sox10 up vs E11.5 * Ctrl
10397741	Psmc1	NM_008947	0.00178584	1.19557	E11.5 * Sox10 up vs E11.5 * Ctrl
10445558	BC048355	NM_207161	0.0099386	1.19542	E11.5 * Sox10 up vs E11.5 * Ctrl
10393408	Tmc6	NM_145439	0.00463141	1.19516	E11.5 * Sox10 up vs E11.5 * Ctrl
10467637	Arhgap19	NM_027667	0.00613189	1.19511	E11.5 * Sox10 up vs E11.5 * Ctrl
10552125	Pepd	NM_008820	0.00609278	1.19492	E11.5 * Sox10 up vs E11.5 * Ctrl
10424485	Phf20l1	NM_001081409	0.00959504	1.19476	E11.5 * Sox10 up vs E11.5 * Ctrl
10352947	Mrpl15	NM_025300	0.000215538	1.19471	E11.5 * Sox10 up vs E11.5 * Ctrl
10347460	Ttll4	NM_001014974	0.000166938	1.1947	E11.5 * Sox10 up vs E11.5 * Ctrl
10546586	Tmf1	NM_001081111	0.00695155	1.1946	E11.5 * Sox10 up vs E11.5 * Ctrl
10355742	Abcb6	NM_023732	0.00449204	1.19451	E11.5 * Sox10 up vs E11.5 * Ctrl
10597103	Dhx30	NM_133347	0.00058983	1.19431	E11.5 * Sox10 up vs E11.5 * Ctrl
10434675	Dnajb11	NM_026400	0.00219406	1.1943	E11.5 * Sox10 up vs E11.5 * Ctrl
10365830		---	0.0025115	1.19429	E11.5 * Sox10 up vs E11.5 * Ctrl
10500808	Olfml3	NM_133859	0.00710532	1.19426	E11.5 * Sox10 up vs E11.5 * Ctrl
10535750	Mtif3	NM_029581	0.00692136	1.1942	E11.5 * Sox10 up vs E11.5 * Ctrl
10460157	Cpt1a	NM_013495	0.0100332	1.1941	E11.5 * Sox10 up vs E11.5 * Ctrl
10346323	02-Mar	NM_175439	0.00746891	1.19385	E11.5 * Sox10 up vs E11.5 * Ctrl
10473793	Psmc3	NM_008948	4.7646e-005	1.19378	E11.5 * Sox10 up vs E11.5 * Ctrl
10441038	Hlcs	NM_139145	0.0094167	1.1937	E11.5 * Sox10 up vs E11.5 * Ctrl
10448192	EG624960	XR_033753	0.00311521	1.1935	E11.5 * Sox10 up vs E11.5 * Ctrl
10499839	Snapin	NM_133854	6.35492e-005	1.19337	E11.5 * Sox10 up vs E11.5 * Ctrl
10436708	Usp16	NM_024258	0.00832428	1.19304	E11.5 * Sox10 up vs E11.5 * Ctrl
10418927	Bmpr1a	NM_009758	0.000319268	1.19291	E11.5 * Sox10 up vs E11.5 * Ctrl
10479411	Ogfr	NM_031373	0.0013922	1.19266	E11.5 * Sox10 up vs E11.5 * Ctrl
10522335	Atp10d	NR_003966	0.00908034	1.19242	E11.5 * Sox10 up vs E11.5 * Ctrl
10490773	Hnrnph2	NM_019868	0.00483321	1.19232	E11.5 * Sox10 up vs E11.5 * Ctrl
10531323	G3bp2	NM_011816	0.000918202	1.19217	E11.5 * Sox10 up vs E11.5 * Ctrl
10481634	Slc25a25	NM_146118	0.00472355	1.1921	E11.5 * Sox10 up vs E11.5 * Ctrl
10502787	Rpsa	NM_011029	0.00853828	1.19205	E11.5 * Sox10 up vs E11.5 * Ctrl
10541114	Rasgef1a	BC138284	0.0102358	1.19198	E11.5 * Sox10 up vs E11.5 * Ctrl
10549979	100042997	XR_034250	0.00127573	1.19187	E11.5 * Sox10 up vs E11.5 * Ctrl
10365297	D10Wsu102e	BC094241	0.00312659	1.19166	E11.5 * Sox10 up vs E11.5 * Ctrl
10498802	Rapgef2	NM_001099624	0.00174873	1.1916	E11.5 * Sox10 up vs E11.5 * Ctrl
10382565	Mrps7	NM_025305	0.000510311	1.19158	E11.5 * Sox10 up vs E11.5 * Ctrl
10480605	Cobra1	NM_021393	0.000658479	1.19136	E11.5 * Sox10 up vs E11.5 * Ctrl
10393823	P4hb	NM_011032	0.00575566	1.19125	E11.5 * Sox10 up vs E11.5 * Ctrl
10557631	Prr14	NM_145589	0.000140351	1.19106	E11.5 * Sox10 up vs E11.5 * Ctrl
10376096	Acsl6	NM_001033598	0.00155559	1.19092	E11.5 * Sox10 up vs E11.5 * Ctrl
10386095	Fam114a2	NM_026342	0.00360361	1.19087	E11.5 * Sox10 up vs E11.5 * Ctrl
10457844	Zfp191	NM_021559	0.000438175	1.19079	E11.5 * Sox10 up vs E11.5 * Ctrl
10580771	Ciapin1	NM_134141	0.000450931	1.19075	E11.5 * Sox10 up vs E11.5 * Ctrl
10367337	Rnf41	NM_026259	0.00131347	1.19071	E11.5 * Sox10 up vs E11.5 * Ctrl
10462136	Cycs	NM_007808	0.00876263	1.19067	E11.5 * Sox10 up vs E11.5 * Ctrl
10393309	Prpsap1	BC029621	0.00646541	1.19059	E11.5 * Sox10 up vs E11.5 * Ctrl
10575144	Nip7	NM_025391	0.0057284	1.19034	E11.5 * Sox10 up vs E11.5 * Ctrl
10599200	Pgrmc1	NM_016783	0.010275	1.19006	E11.5 * Sox10 up vs E11.5 * Ctrl

10347254	Smarcal1	NM_018817	0.000496886	1.18973	E11.5 * Sox10 up vs E11.5 * Ctrl
10467859	Cox15	NM_144874	0.00733525	1.18939	E11.5 * Sox10 up vs E11.5 * Ctrl
10439991	Tfg	NM_019678	4.12601e-005	1.18924	E11.5 * Sox10 up vs E11.5 * Ctrl
10375501		---	0.00993254	1.18921	E11.5 * Sox10 up vs E11.5 * Ctrl
10429972	Cpsf1	NM_053193	0.000467783	1.18919	E11.5 * Sox10 up vs E11.5 * Ctrl
10524124	Golga3	NM_008146	0.000480036	1.18889	E11.5 * Sox10 up vs E11.5 * Ctrl
10350684	Arpc5	NM_026369	0.00938843	1.18886	E11.5 * Sox10 up vs E11.5 * Ctrl
10479560	Rtel1	NM_001001882	0.00404933	1.18857	E11.5 * Sox10 up vs E11.5 * Ctrl
10578539	Slc25a4	NM_007450	4.0236e-005	1.18856	E11.5 * Sox10 up vs E11.5 * Ctrl
10574996	Prmt7	NM_145404	0.00170984	1.18824	E11.5 * Sox10 up vs E11.5 * Ctrl
10587012	Ccpg1	NM_001114328	0.0089596	1.18799	E11.5 * Sox10 up vs E11.5 * Ctrl
10558345	Dock1	NM_001033420	0.00161125	1.18799	E11.5 * Sox10 up vs E11.5 * Ctrl
10580534		---	0.00542441	1.18784	E11.5 * Sox10 up vs E11.5 * Ctrl
10478754	Arfgef2	NM_001085495	0.00136428	1.18771	E11.5 * Sox10 up vs E11.5 * Ctrl
10529425	Nop14	NM_029278	0.00058998	1.1874	E11.5 * Sox10 up vs E11.5 * Ctrl
10555414	Rab6	NM_024287	0.00785701	1.1874	E11.5 * Sox10 up vs E11.5 * Ctrl
10604763	Arpc1b	NM_023142	0.00969769	1.18739	E11.5 * Sox10 up vs E11.5 * Ctrl
10522676	Srp72	NM_025691	0.0010053	1.18725	E11.5 * Sox10 up vs E11.5 * Ctrl
10420532	Atp8a2	NM_015803	0.00603286	1.18721	E11.5 * Sox10 up vs E11.5 * Ctrl
10397311	Dlst	NM_030225	0.00475234	1.1872	E11.5 * Sox10 up vs E11.5 * Ctrl
10578019	Nudc	NM_010948	0.00220045	1.18716	E11.5 * Sox10 up vs E11.5 * Ctrl
10603702	Llph	NM_025431	0.00201666	1.1869	E11.5 * Sox10 up vs E11.5 * Ctrl
10546113	Sec61a1	NM_016906	0.000556571	1.1869	E11.5 * Sox10 up vs E11.5 * Ctrl
10367772	Samd5	NM_177271	0.00771003	1.18669	E11.5 * Sox10 up vs E11.5 * Ctrl
10607524	Sms	NM_009214	0.00084357	1.18667	E11.5 * Sox10 up vs E11.5 * Ctrl
10515293	Llph	NM_025431	0.00613503	1.18648	E11.5 * Sox10 up vs E11.5 * Ctrl
10486616	Ubr1	NM_009461	0.00386601	1.18633	E11.5 * Sox10 up vs E11.5 * Ctrl
10545658	Wdr54	NM_023790	0.000453984	1.18628	E11.5 * Sox10 up vs E11.5 * Ctrl
10533849	Rilpl1	NM_021430	0.00612028	1.18593	E11.5 * Sox10 up vs E11.5 * Ctrl
10352178	Sccpdh	NM_178653	0.00858529	1.18584	E11.5 * Sox10 up vs E11.5 * Ctrl
10591423	Fdx1l	NM_001039824	0.00902779	1.18577	E11.5 * Sox10 up vs E11.5 * Ctrl
10580391	Itfg1	NM_028007	0.000279328	1.18564	E11.5 * Sox10 up vs E11.5 * Ctrl
10499394	Lmna	NM_001002011	0.00900032	1.18556	E11.5 * Sox10 up vs E11.5 * Ctrl
10585201	Timm8b	NM_013897	0.00176799	1.1855	E11.5 * Sox10 up vs E11.5 * Ctrl
10512851	Erp44	NM_029572	0.0054549	1.18537	E11.5 * Sox10 up vs E11.5 * Ctrl
10523206	Uso1	NM_019490	0.000387841	1.18532	E11.5 * Sox10 up vs E11.5 * Ctrl
10478997	Tcfap2c	NM_009335	0.00147957	1.18525	E11.5 * Sox10 up vs E11.5 * Ctrl
10356657	Ndufa10	NM_024197	0.000546754	1.18487	E11.5 * Sox10 up vs E11.5 * Ctrl
10551760	Zfp84	NM_023750	0.00823873	1.18484	E11.5 * Sox10 up vs E11.5 * Ctrl
10502165	Sec24b	NM_207209	1.20803e-005	1.18483	E11.5 * Sox10 up vs E11.5 * Ctrl
10472300	Psmd14	NM_021526	0.00118776	1.1843	E11.5 * Sox10 up vs E11.5 * Ctrl
10569441	Nap1l4	NM_008672	0.00184141	1.18424	E11.5 * Sox10 up vs E11.5 * Ctrl
10435784	BC002163	NR_002445	0.00115834	1.18405	E11.5 * Sox10 up vs E11.5 * Ctrl
10405662	Mak10	NM_030153	0.000186238	1.18372	E11.5 * Sox10 up vs E11.5 * Ctrl
10365230	Tdg	NM_172552	0.00210565	1.18372	E11.5 * Sox10 up vs E11.5 * Ctrl
10357164	Epb4.1l5	NM_145506	0.000560214	1.18371	E11.5 * Sox10 up vs E11.5 * Ctrl
10435162	Lrch3	NM_001081255	0.00349562	1.18365	E11.5 * Sox10 up vs E11.5 * Ctrl
10368930	1700021F05Rik	NM_026411	0.0023069	1.1836	E11.5 * Sox10 up vs E11.5 * Ctrl
10582201	Cox4nb	NM_010926	0.00101411	1.18353	E11.5 * Sox10 up vs E11.5 * Ctrl
10560842	Zfp94	NM_009568	0.00271509	1.18347	E11.5 * Sox10 up vs E11.5 * Ctrl
10391242	Rab5c	NM_024456	0.00316287	1.18306	E11.5 * Sox10 up vs E11.5 * Ctrl
10451918	Ubxn6	NM_024432	0.00115999	1.183	E11.5 * Sox10 up vs E11.5 * Ctrl
10501265	Gnai3	NM_010306	0.000122159	1.18268	E11.5 * Sox10 up vs E11.5 * Ctrl
10553833	Ndn	NM_010882	0.00971436	1.18261	E11.5 * Sox10 up vs E11.5 * Ctrl
10597920	Zdhhc3	NM_026917	0.000578772	1.18248	E11.5 * Sox10 up vs E11.5 * Ctrl
10430945	Poldip3	NM_178627	0.00263954	1.1824	E11.5 * Sox10 up vs E11.5 * Ctrl
10371676	Utp20	NM_175158	0.00116852	1.1822	E11.5 * Sox10 up vs E11.5 * Ctrl
10476056	AU015228	NM_001033197	0.00858408	1.18209	E11.5 * Sox10 up vs E11.5 * Ctrl
10529118	Eif2b4	NM_001127356	0.00669979	1.18204	E11.5 * Sox10 up vs E11.5 * Ctrl
10515536	Ipo13	NM_146152	0.00433243	1.18191	E11.5 * Sox10 up vs E11.5 * Ctrl
10418720	Mettl6	NM_025907	0.00479987	1.18187	E11.5 * Sox10 up vs E11.5 * Ctrl
10403511	Heatr1	NM_144835	0.000432787	1.18182	E11.5 * Sox10 up vs E11.5 * Ctrl
10487359	Itpripl1	BC130218	0.000146884	1.18174	E11.5 * Sox10 up vs E11.5 * Ctrl
10546217	Chchd6	NM_025351	0.00312736	1.18164	E11.5 * Sox10 up vs E11.5 * Ctrl
10390653	Med24	NM_011869	0.00151612	1.18138	E11.5 * Sox10 up vs E11.5 * Ctrl
10593159	Pafah1b2	NM_008775	0.00160201	1.18138	E11.5 * Sox10 up vs E11.5 * Ctrl

10468022	Mrpl43	NM_053164	0.000920169	1.18134	E11.5 * Sox10 up vs E11.5 * Ctrl
10493247	Ubqln4	NM_033526	0.00534428	1.18104	E11.5 * Sox10 up vs E11.5 * Ctrl
10591369	Dnmt1	NM_010066	0.000840754	1.18084	E11.5 * Sox10 up vs E11.5 * Ctrl
10601760	Hnrnph2	NM_019868	0.00482366	1.18074	E11.5 * Sox10 up vs E11.5 * Ctrl
10360764	Enah	NM_010135	0.00118337	1.18067	E11.5 * Sox10 up vs E11.5 * Ctrl
10527012	Lfng	NM_008494	0.00264087	1.18048	E11.5 * Sox10 up vs E11.5 * Ctrl
10372965	Usp15	NM_027604	0.000361214	1.18046	E11.5 * Sox10 up vs E11.5 * Ctrl
10471608	Cep110	NM_012018	0.0041318	1.18043	E11.5 * Sox10 up vs E11.5 * Ctrl
10589503	2610002I17Rik	NM_001081381	0.00624709	1.18035	E11.5 * Sox10 up vs E11.5 * Ctrl
10576696	Insr	NM_010568	0.00114509	1.18031	E11.5 * Sox10 up vs E11.5 * Ctrl
10488482	Acss1	NM_080575	0.00537831	1.18007	E11.5 * Sox10 up vs E11.5 * Ctrl
10562251	Scn1b	NM_011322	0.00131803	1.17995	E11.5 * Sox10 up vs E11.5 * Ctrl
10529794	EG636612	XR_001803	0.00425406	1.17979	E11.5 * Sox10 up vs E11.5 * Ctrl
10543369	Cadps2	NM_153163	0.00551796	1.17975	E11.5 * Sox10 up vs E11.5 * Ctrl
10497587	1600012F09Rik	NM_025904	0.00449183	1.17967	E11.5 * Sox10 up vs E11.5 * Ctrl
10455578	Commd10	NM_178377	0.00753318	1.17965	E11.5 * Sox10 up vs E11.5 * Ctrl
10596347	Atp2c1	NM_175025	0.0008436	1.17961	E11.5 * Sox10 up vs E11.5 * Ctrl
10565968	Atg16l2	NM_001111111	0.00611849	1.17954	E11.5 * Sox10 up vs E11.5 * Ctrl
10446928	Ltbp1	NM_019919	0.00601501	1.17945	E11.5 * Sox10 up vs E11.5 * Ctrl
10462100	Sarnp	NM_025364	0.00430059	1.17912	E11.5 * Sox10 up vs E11.5 * Ctrl
10509620	Capzb	NM_001037761	0.0097537	1.17898	E11.5 * Sox10 up vs E11.5 * Ctrl
10528170	Cycs	NM_007808	0.000609362	1.17869	E11.5 * Sox10 up vs E11.5 * Ctrl
10459405	Nars	NM_001142950	0.000138531	1.17865	E11.5 * Sox10 up vs E11.5 * Ctrl
10433352	Ubn1	NM_026666	0.00444451	1.17854	E11.5 * Sox10 up vs E11.5 * Ctrl
10541191	Rad52	NM_011236	0.00635748	1.17822	E11.5 * Sox10 up vs E11.5 * Ctrl
10606609	Tspan6	NM_019656	0.0068328	1.17814	E11.5 * Sox10 up vs E11.5 * Ctrl
10594480	Rab11a	NM_017382	0.000592756	1.1781	E11.5 * Sox10 up vs E11.5 * Ctrl
10475420	Trim69	NM_080510	0.00741999	1.17791	E11.5 * Sox10 up vs E11.5 * Ctrl
10472212	Pkp4	NM_026361	0.00184655	1.17784	E11.5 * Sox10 up vs E11.5 * Ctrl
10493770	Ilf2	NM_026374	0.00335058	1.17783	E11.5 * Sox10 up vs E11.5 * Ctrl
10352459	Rab3gap2	BC057872	0.00862775	1.1777	E11.5 * Sox10 up vs E11.5 * Ctrl
10367708	Pcmt1	NM_008786	0.00181292	1.17758	E11.5 * Sox10 up vs E11.5 * Ctrl
10547251	Bms1	NM_194339	0.00528345	1.17752	E11.5 * Sox10 up vs E11.5 * Ctrl
10368883	Tdg	NM_011561	0.00298232	1.17742	E11.5 * Sox10 up vs E11.5 * Ctrl
10496737	Bcl10	NM_009740	0.000951186	1.17727	E11.5 * Sox10 up vs E11.5 * Ctrl
10476668	Csrp2bp	NM_181417	0.00443157	1.17705	E11.5 * Sox10 up vs E11.5 * Ctrl
10439878	Psmc1	NM_008947	0.00907983	1.17705	E11.5 * Sox10 up vs E11.5 * Ctrl
10597288	Mlh1	NM_026810	0.00962644	1.17672	E11.5 * Sox10 up vs E11.5 * Ctrl
10420131	Rabggta	NM_019519	0.00519748	1.1765	E11.5 * Sox10 up vs E11.5 * Ctrl
10437687	Litaf	NM_019980	0.00123083	1.17647	E11.5 * Sox10 up vs E11.5 * Ctrl
10434233	Ufd1l	NM_011672	0.000235237	1.17609	E11.5 * Sox10 up vs E11.5 * Ctrl
10605919	Pja1	NM_001083110	0.000199251	1.17577	E11.5 * Sox10 up vs E11.5 * Ctrl
10603431	Suv39h1	NM_011514	0.00510981	1.17575	E11.5 * Sox10 up vs E11.5 * Ctrl
10375926	Ppp2ca	NM_019411	9.10092e-005	1.1757	E11.5 * Sox10 up vs E11.5 * Ctrl
10348558	Scly	NM_016717	0.00559727	1.17548	E11.5 * Sox10 up vs E11.5 * Ctrl
10506680	Tmem48	NM_028355	0.00719409	1.17529	E11.5 * Sox10 up vs E11.5 * Ctrl
10570373	Tfdp1	NM_009361	0.00127871	1.17507	E11.5 * Sox10 up vs E11.5 * Ctrl
10441987	Chd1	NM_007690	0.000155277	1.17504	E11.5 * Sox10 up vs E11.5 * Ctrl
10437748	Gspt1	NM_146066	0.000109081	1.17486	E11.5 * Sox10 up vs E11.5 * Ctrl
10556169	Eif3f	NM_025344	0.00226113	1.17473	E11.5 * Sox10 up vs E11.5 * Ctrl
10402841	Brf1	NM_028193	0.00776729	1.17471	E11.5 * Sox10 up vs E11.5 * Ctrl
10462791	March5	NM_027314	0.00483438	1.17466	E11.5 * Sox10 up vs E11.5 * Ctrl
10387992	Nup88	NM_172394	0.000185253	1.17455	E11.5 * Sox10 up vs E11.5 * Ctrl
10461898	Rfk	NM_019437	0.00771668	1.17454	E11.5 * Sox10 up vs E11.5 * Ctrl
10575129	Vps4a	NM_126165	0.00151563	1.17449	E11.5 * Sox10 up vs E11.5 * Ctrl
10403903	Trim27	NM_009054	0.00277794	1.17423	E11.5 * Sox10 up vs E11.5 * Ctrl
10464836	Actn3	NM_013456	0.00510315	1.17422	E11.5 * Sox10 up vs E11.5 * Ctrl
10511258	Fam132a	NM_026125	0.00451998	1.17394	E11.5 * Sox10 up vs E11.5 * Ctrl
10513583	Cdc26	NM_139291	0.00671888	1.17388	E11.5 * Sox10 up vs E11.5 * Ctrl
10390153	Myst2	NM_177619	0.00270969	1.17385	E11.5 * Sox10 up vs E11.5 * Ctrl
10450669	Prr3	NM_145487	9.79897e-005	1.17377	E11.5 * Sox10 up vs E11.5 * Ctrl
10528268	Ptpn12	NM_011203	0.00116246	1.17355	E11.5 * Sox10 up vs E11.5 * Ctrl
10564663	Tdg	NM_172552	0.0100359	1.17332	E11.5 * Sox10 up vs E11.5 * Ctrl
10492671	Ppid	NM_026352	0.000956915	1.17315	E11.5 * Sox10 up vs E11.5 * Ctrl
10601915	BC031748	BC031748	0.00836761	1.17307	E11.5 * Sox10 up vs E11.5 * Ctrl
10516640	Eif3i	NM_018799	0.00349613	1.17277	E11.5 * Sox10 up vs E11.5 * Ctrl

10446693	Wdr43	AK129031	0.000496548	1.17248	E11.5 * Sox10 up vs E11.5 * Ctrl
10533285	Ptpn11	NM_011202	0.00306341	1.1723	E11.5 * Sox10 up vs E11.5 * Ctrl
10394288	Itsn2	NM_011365	0.00600287	1.17224	E11.5 * Sox10 up vs E11.5 * Ctrl
10368229	Tbpl1	NM_011603	0.00987341	1.17183	E11.5 * Sox10 up vs E11.5 * Ctrl
10533659	Clip1	NM_019765	0.00024176	1.17167	E11.5 * Sox10 up vs E11.5 * Ctrl
10413138	Vdac2	NM_011695	0.00100988	1.17141	E11.5 * Sox10 up vs E11.5 * Ctrl
10594645	Rab8b	NM_173413	0.00727346	1.17139	E11.5 * Sox10 up vs E11.5 * Ctrl
10460833	Ehd1	NM_010119	0.00136221	1.17112	E11.5 * Sox10 up vs E11.5 * Ctrl
10551496	Paf1	NM_019458	0.00114243	1.17107	E11.5 * Sox10 up vs E11.5 * Ctrl
10586920	Rfx7	NM_001033536	0.00182919	1.17106	E11.5 * Sox10 up vs E11.5 * Ctrl
10388269		---	0.00130658	1.17093	E11.5 * Sox10 up vs E11.5 * Ctrl
10422852	Rpl19	NM_009078	1.72281e-005	1.17078	E11.5 * Sox10 up vs E11.5 * Ctrl
10452496	Ralbp1	NM_009067	0.00404039	1.17065	E11.5 * Sox10 up vs E11.5 * Ctrl
10480057	Rbm17	NM_152824	0.00105476	1.17049	E11.5 * Sox10 up vs E11.5 * Ctrl
10566225	Olfr616	NM_147099	0.00401787	1.16999	E11.5 * Sox10 up vs E11.5 * Ctrl
10363706	Jmjd1c	NM_207221	0.0089112	1.16985	E11.5 * Sox10 up vs E11.5 * Ctrl
10474545	Slc12a6	NM_133649	0.0074391	1.16979	E11.5 * Sox10 up vs E11.5 * Ctrl
10577070	Tubgcp3	NM_198031	0.00217634	1.16944	E11.5 * Sox10 up vs E11.5 * Ctrl
10460112		---	0.00949241	1.16903	E11.5 * Sox10 up vs E11.5 * Ctrl
10447395	Msh2	NM_008628	0.00247352	1.16867	E11.5 * Sox10 up vs E11.5 * Ctrl
10583697	Smarca4	NM_011417	0.00425448	1.16859	E11.5 * Sox10 up vs E11.5 * Ctrl
10482323	Ppp6c	NM_024209	0.00769976	1.16825	E11.5 * Sox10 up vs E11.5 * Ctrl
10520025	Psmc2	NM_011188	9.78517e-005	1.16802	E11.5 * Sox10 up vs E11.5 * Ctrl
10502058	BC002199	NM_145964	0.000597204	1.16771	E11.5 * Sox10 up vs E11.5 * Ctrl
10589723	Lrrfip2	NM_027742	0.00809182	1.1675	E11.5 * Sox10 up vs E11.5 * Ctrl
10362036	Hbs1l	NM_019702	7.77892e-005	1.16743	E11.5 * Sox10 up vs E11.5 * Ctrl
10605090	Idh3g	NM_008323	0.00149511	1.16738	E11.5 * Sox10 up vs E11.5 * Ctrl
10493891	Ywhaz	NM_011740	3.14511e-006	1.16734	E11.5 * Sox10 up vs E11.5 * Ctrl
10512136	Bag1	NM_009736	0.000469433	1.16707	E11.5 * Sox10 up vs E11.5 * Ctrl
10489831	Stau1	NM_001109906	0.00198904	1.16651	E11.5 * Sox10 up vs E11.5 * Ctrl
10545932	Snrnp27	NM_025665	0.00602945	1.16604	E11.5 * Sox10 up vs E11.5 * Ctrl
10438112	Pi4ka	NM_001001983	0.00062693	1.16595	E11.5 * Sox10 up vs E11.5 * Ctrl
10470268	Pmpca	NM_173180	0.00464308	1.16567	E11.5 * Sox10 up vs E11.5 * Ctrl
10531970	EG545790	XM_620236	0.000785654	1.16546	E11.5 * Sox10 up vs E11.5 * Ctrl
10579874	Abce1	NM_015751	0.00269477	1.16527	E11.5 * Sox10 up vs E11.5 * Ctrl
10430871	Tdg	NM_011561	0.00331106	1.16509	E11.5 * Sox10 up vs E11.5 * Ctrl
10433910	Top3b	NM_011624	0.00541126	1.16508	E11.5 * Sox10 up vs E11.5 * Ctrl
10432593	Csrnp2	NM_153407	4.52638e-005	1.16462	E11.5 * Sox10 up vs E11.5 * Ctrl
10564736	Polg	NM_017462	0.00671397	1.16462	E11.5 * Sox10 up vs E11.5 * Ctrl
10508376	Trim62	NM_178110	0.00243831	1.16414	E11.5 * Sox10 up vs E11.5 * Ctrl
10535331	Mmd2	NM_175217	0.0103567	1.16411	E11.5 * Sox10 up vs E11.5 * Ctrl
10389674	Dynll2	NM_026556	0.000673034	1.16401	E11.5 * Sox10 up vs E11.5 * Ctrl
10576506	Gnpat	NM_010322	0.00824272	1.16388	E11.5 * Sox10 up vs E11.5 * Ctrl
10353420	Mcm3	NM_008563	0.00208452	1.16369	E11.5 * Sox10 up vs E11.5 * Ctrl
10440600	Cct8	NM_009840	0.000152441	1.16358	E11.5 * Sox10 up vs E11.5 * Ctrl
10446402	Ppp4r1	NM_146081	0.00724555	1.1633	E11.5 * Sox10 up vs E11.5 * Ctrl
10348004	Psmd1	NM_027357	3.79781e-005	1.16315	E11.5 * Sox10 up vs E11.5 * Ctrl
10421981	Dis3	NM_028315	0.00436824	1.16304	E11.5 * Sox10 up vs E11.5 * Ctrl
10382284	Prkar1a	NM_021880	0.00262332	1.16293	E11.5 * Sox10 up vs E11.5 * Ctrl
10384662	Commd1	NM_144514	0.00102437	1.16279	E11.5 * Sox10 up vs E11.5 * Ctrl
10517465	Kdm1	NM_133872	0.000132116	1.16273	E11.5 * Sox10 up vs E11.5 * Ctrl
10356800	Hdlbp	NM_133808	2.77959e-005	1.16271	E11.5 * Sox10 up vs E11.5 * Ctrl
10527559	Polr1d	NM_009087	0.00838686	1.16242	E11.5 * Sox10 up vs E11.5 * Ctrl
10454298	Zfp397	NM_027007	0.000945413	1.16236	E11.5 * Sox10 up vs E11.5 * Ctrl
10569898	Evi5l	NM_001039578	0.00241298	1.16226	E11.5 * Sox10 up vs E11.5 * Ctrl
10579969	Zfp330	NM_145600	0.00117059	1.16202	E11.5 * Sox10 up vs E11.5 * Ctrl
10395788	Srp54c	NM_001100110	0.000132441	1.16201	E11.5 * Sox10 up vs E11.5 * Ctrl
10378802	Blmh	NM_178645	0.000173826	1.16192	E11.5 * Sox10 up vs E11.5 * Ctrl
10512111	Smu1	NM_021535	0.000546141	1.16159	E11.5 * Sox10 up vs E11.5 * Ctrl
10417664	Rpl19	NM_009078	0.00154713	1.16141	E11.5 * Sox10 up vs E11.5 * Ctrl
10405693	Dapk1	NM_029653	1.55518e-005	1.16134	E11.5 * Sox10 up vs E11.5 * Ctrl
10484318	Nckap1	NM_016965	0.00337919	1.16133	E11.5 * Sox10 up vs E11.5 * Ctrl
10384192	Tbrg4	NM_134011	0.00262299	1.1613	E11.5 * Sox10 up vs E11.5 * Ctrl
10405125	Fbxw17	NM_175401	0.00894406	1.16088	E11.5 * Sox10 up vs E11.5 * Ctrl
10371877	Slc25a3	NM_133668	0.00012707	1.16073	E11.5 * Sox10 up vs E11.5 * Ctrl
10596190	Bfsp2	NM_001002896	0.00795225	1.16068	E11.5 * Sox10 up vs E11.5 * Ctrl

10589756	Epm2aip1	NM_175266	0.00516326	1.16064	E11.5 * Sox10 up vs E11.5 * Ctrl
10520417	Gm1040	ENSMUST00000(	0.00772339	1.1605	E11.5 * Sox10 up vs E11.5 * Ctrl
10392183	Ern1	NM_023913	0.00580913	1.16039	E11.5 * Sox10 up vs E11.5 * Ctrl
10421922		---	0.00839845	1.16007	E11.5 * Sox10 up vs E11.5 * Ctrl
10573637	Phkb	NM_199446	0.00637315	1.16006	E11.5 * Sox10 up vs E11.5 * Ctrl
10416940	Tpm3	ENSMUST00000(	2.36193e-005	1.15995	E11.5 * Sox10 up vs E11.5 * Ctrl
10570625	Rpl19	NM_009078	3.46237e-005	1.15964	E11.5 * Sox10 up vs E11.5 * Ctrl
10488031		---	0.00578717	1.15902	E11.5 * Sox10 up vs E11.5 * Ctrl
10424340	Ndufb9	NM_023172	0.00200712	1.15895	E11.5 * Sox10 up vs E11.5 * Ctrl
10559270	Tssc4	NM_020285	0.00763364	1.15884	E11.5 * Sox10 up vs E11.5 * Ctrl
10503401	Tmem55a	NM_028264	0.00209343	1.15855	E11.5 * Sox10 up vs E11.5 * Ctrl
10565479	l7Rn6	NM_026304	0.00822326	1.1581	E11.5 * Sox10 up vs E11.5 * Ctrl
10479026	Rae1	NM_175112	0.000573907	1.15791	E11.5 * Sox10 up vs E11.5 * Ctrl
10472313	Tbr1	NM_009322	0.000418412	1.15779	E11.5 * Sox10 up vs E11.5 * Ctrl
10526726	Zkscan1	NM_133906	0.00596773	1.15772	E11.5 * Sox10 up vs E11.5 * Ctrl
10590438	C730027P07Rik	NM_177743	0.00714843	1.15767	E11.5 * Sox10 up vs E11.5 * Ctrl
10423842	Wdsof1	NM_198606	0.00288366	1.15726	E11.5 * Sox10 up vs E11.5 * Ctrl
10428052	Cct5	NM_007637	1.41858e-006	1.1567	E11.5 * Sox10 up vs E11.5 * Ctrl
10456995	Txnl4a	NM_178604	0.00756458	1.15665	E11.5 * Sox10 up vs E11.5 * Ctrl
10542229		---	0.00882251	1.15658	E11.5 * Sox10 up vs E11.5 * Ctrl
10411646	Gtf2h2	NM_022011	0.00673045	1.15649	E11.5 * Sox10 up vs E11.5 * Ctrl
10386058	Sparc	NM_009242	0.00296219	1.15645	E11.5 * Sox10 up vs E11.5 * Ctrl
10521587	Dnaja1	NM_008298	0.00016974	1.15588	E11.5 * Sox10 up vs E11.5 * Ctrl
10418991	Gcap14	NM_027045	0.0096986	1.15585	E11.5 * Sox10 up vs E11.5 * Ctrl
10395739	Srp54b	NM_001100109	4.40481e-005	1.15585	E11.5 * Sox10 up vs E11.5 * Ctrl
10385790	Hspa4	NM_008300	0.000326392	1.15551	E11.5 * Sox10 up vs E11.5 * Ctrl
10347917	Rpl19	NM_009078	0.000291733	1.15539	E11.5 * Sox10 up vs E11.5 * Ctrl
10498415	Dhx36	NM_028136	0.00898348	1.15526	E11.5 * Sox10 up vs E11.5 * Ctrl
10519392	Krit1	NM_030675	0.00534827	1.15503	E11.5 * Sox10 up vs E11.5 * Ctrl
10456194	Napg	NM_028017	0.00581273	1.15463	E11.5 * Sox10 up vs E11.5 * Ctrl
10522668	Paics	NM_025939	0.00231371	1.15458	E11.5 * Sox10 up vs E11.5 * Ctrl
10461268	1810009A15Rik	BC047099	0.00524064	1.15454	E11.5 * Sox10 up vs E11.5 * Ctrl
10520815	Slc4a1ap	NM_009206	0.00509457	1.15421	E11.5 * Sox10 up vs E11.5 * Ctrl
10503150	Rab2a	NM_021518	2.7266e-006	1.15404	E11.5 * Sox10 up vs E11.5 * Ctrl
10589982	Azi2	NM_013727	0.00614332	1.15355	E11.5 * Sox10 up vs E11.5 * Ctrl
10581363	Cenpt	NM_177150	0.00400829	1.15317	E11.5 * Sox10 up vs E11.5 * Ctrl
10604501	Hnrnpf	NM_133834	0.000357567	1.15315	E11.5 * Sox10 up vs E11.5 * Ctrl
10408684	Tmed10	NM_026775	1.51704e-006	1.15225	E11.5 * Sox10 up vs E11.5 * Ctrl
10398695	Mark3	NM_021516	0.00215551	1.15213	E11.5 * Sox10 up vs E11.5 * Ctrl
10414522	Apex1	NM_009687	0.00251388	1.15191	E11.5 * Sox10 up vs E11.5 * Ctrl
10532901	OTTMUSG00000	XR_033041	0.00226608	1.1519	E11.5 * Sox10 up vs E11.5 * Ctrl
10565288	Fam108c	NM_133722	0.0072533	1.15188	E11.5 * Sox10 up vs E11.5 * Ctrl
10429944	Scrt1	NM_130893	0.00543625	1.15121	E11.5 * Sox10 up vs E11.5 * Ctrl
10490370	Psma7	NM_011969	4.97702e-006	1.15111	E11.5 * Sox10 up vs E11.5 * Ctrl
10362861	Scml4	NM_172938	0.00557534	1.15105	E11.5 * Sox10 up vs E11.5 * Ctrl
10482017	Rab14	NM_026697	0.00104195	1.15084	E11.5 * Sox10 up vs E11.5 * Ctrl
10477572	Chmp4b	NM_029362	0.00511091	1.15081	E11.5 * Sox10 up vs E11.5 * Ctrl
10590253	Rpsa	NM_011029	0.0100469	1.15077	E11.5 * Sox10 up vs E11.5 * Ctrl
10510624	Klhl21	NM_001033352	0.00783028	1.15051	E11.5 * Sox10 up vs E11.5 * Ctrl
10598638	Mid1ip1	NM_026524	0.0080091	1.15046	E11.5 * Sox10 up vs E11.5 * Ctrl
10499879	Rpl3	NM_013762	0.000837836	1.15029	E11.5 * Sox10 up vs E11.5 * Ctrl
10499881	Rpl3	NM_013762	0.000837836	1.15029	E11.5 * Sox10 up vs E11.5 * Ctrl
10557148	Dctn5	NM_021608	0.00306299	1.15012	E11.5 * Sox10 up vs E11.5 * Ctrl
10530434	Commd8	NM_178599	0.00521914	1.15003	E11.5 * Sox10 up vs E11.5 * Ctrl
10521174	Rnf4	NM_011278	0.00633339	1.14958	E11.5 * Sox10 up vs E11.5 * Ctrl
10380833	Rpl19	NM_009078	7.53713e-005	1.14946	E11.5 * Sox10 up vs E11.5 * Ctrl
10414025	Gdf10	NM_145741	0.00486257	1.14925	E11.5 * Sox10 up vs E11.5 * Ctrl
10407811	Hnrnpf	NM_133834	2.17947e-005	1.14884	E11.5 * Sox10 up vs E11.5 * Ctrl
10378154	Zzef1	NM_001045536	0.00788421	1.14875	E11.5 * Sox10 up vs E11.5 * Ctrl
10487154	Secisbp2l	NM_177608	0.0073074	1.14869	E11.5 * Sox10 up vs E11.5 * Ctrl
10440406	Nrip1	NM_173440	0.00760568	1.14861	E11.5 * Sox10 up vs E11.5 * Ctrl
10489266	Chd6	NM_173368	0.0059853	1.14858	E11.5 * Sox10 up vs E11.5 * Ctrl
10352514	Eprs	NM_029735	7.09948e-005	1.14848	E11.5 * Sox10 up vs E11.5 * Ctrl
10385495	Cdk2ap1	NM_013812	0.00772771	1.14794	E11.5 * Sox10 up vs E11.5 * Ctrl
10498623	Kpna4	NM_008467	0.00698237	1.14771	E11.5 * Sox10 up vs E11.5 * Ctrl
10504470	Melk	NM_010790	0.00903789	1.14764	E11.5 * Sox10 up vs E11.5 * Ctrl

10468037		---	0.0071134	1.14752	E11.5 * Sox10 up vs E11.5 * Ctrl
10512709	Mcart1	NM_001009949	0.00258526	1.14751	E11.5 * Sox10 up vs E11.5 * Ctrl
10375980	Aff4	NM_033565	0.00359401	1.14745	E11.5 * Sox10 up vs E11.5 * Ctrl
10482229	Psmb7	NM_011187	0.00233815	1.14727	E11.5 * Sox10 up vs E11.5 * Ctrl
10483756	Atp5g3	NM_175015	0.00304644	1.14727	E11.5 * Sox10 up vs E11.5 * Ctrl
10407570	Zmynd11	NM_144516	0.00430928	1.14724	E11.5 * Sox10 up vs E11.5 * Ctrl
10425611	Aco2	NM_080633	0.00923509	1.14707	E11.5 * Sox10 up vs E11.5 * Ctrl
10446207	Clpp	NM_017393	0.00896469	1.14706	E11.5 * Sox10 up vs E11.5 * Ctrl
10493565	Adar	NM_001038587	0.00226351	1.14658	E11.5 * Sox10 up vs E11.5 * Ctrl
10358359	Cdc73	NM_145991	0.00152969	1.14617	E11.5 * Sox10 up vs E11.5 * Ctrl
10555063	Ints4	NM_027256	0.00992643	1.14616	E11.5 * Sox10 up vs E11.5 * Ctrl
10473272	Zc3h15	NM_026934	0.003887	1.14584	E11.5 * Sox10 up vs E11.5 * Ctrl
10515352	Akr1a4	NM_021473	0.0028176	1.14559	E11.5 * Sox10 up vs E11.5 * Ctrl
10605222	Irak1	NM_008363	0.00170946	1.14559	E11.5 * Sox10 up vs E11.5 * Ctrl
10429739	Puf60	NM_028364	0.00894379	1.14549	E11.5 * Sox10 up vs E11.5 * Ctrl
10453006	Cebpz	NM_001024806	0.00838329	1.14522	E11.5 * Sox10 up vs E11.5 * Ctrl
10560000	Tpm3	NM_022314	0.00321151	1.14522	E11.5 * Sox10 up vs E11.5 * Ctrl
10561140	Mrpl41	NM_001031808	0.00459467	1.14521	E11.5 * Sox10 up vs E11.5 * Ctrl
10525718	Arl6ip4	NM_144509	0.00320754	1.14507	E11.5 * Sox10 up vs E11.5 * Ctrl
10577866	Ash2l	NM_011791	1.69356e-005	1.14484	E11.5 * Sox10 up vs E11.5 * Ctrl
10447695		---	0.00482159	1.14481	E11.5 * Sox10 up vs E11.5 * Ctrl
10499425	2700060E02Rik	NM_026528	0.000160315	1.14471	E11.5 * Sox10 up vs E11.5 * Ctrl
10425354	Mgat3	NM_010795	0.00777767	1.14454	E11.5 * Sox10 up vs E11.5 * Ctrl
10479627	Tpd52l2	NM_025482	0.00650068	1.14451	E11.5 * Sox10 up vs E11.5 * Ctrl
10460196	1810055G02Rik	NM_028077	0.00481654	1.14418	E11.5 * Sox10 up vs E11.5 * Ctrl
10531488	Ccni	NM_017367	0.00595608	1.1441	E11.5 * Sox10 up vs E11.5 * Ctrl
10502714	Bxdc5	NM_027371	0.00127334	1.14396	E11.5 * Sox10 up vs E11.5 * Ctrl
10599461	Calm2	NM_007589	0.00254132	1.14396	E11.5 * Sox10 up vs E11.5 * Ctrl
10447594	Dynlt1	NM_009342	2.42321e-005	1.14364	E11.5 * Sox10 up vs E11.5 * Ctrl
10454296	Rpl19	NM_009078	0.000263336	1.14329	E11.5 * Sox10 up vs E11.5 * Ctrl
10473574	Olfr1151	NM_146638	0.00965833	1.14304	E11.5 * Sox10 up vs E11.5 * Ctrl
10601164	Nono	NM_023144	0.00509131	1.14269	E11.5 * Sox10 up vs E11.5 * Ctrl
10458823		---	0.000256962	1.14241	E11.5 * Sox10 up vs E11.5 * Ctrl
10510270	Mthfr	NM_010840	0.00515803	1.14238	E11.5 * Sox10 up vs E11.5 * Ctrl
10487629	Idh3b	NM_130884	0.0016671	1.1423	E11.5 * Sox10 up vs E11.5 * Ctrl
10386495	Tom1l2	NM_153080	0.00971564	1.14181	E11.5 * Sox10 up vs E11.5 * Ctrl
10397476	2310044G17Rik	BC026384	0.00600438	1.14174	E11.5 * Sox10 up vs E11.5 * Ctrl
10508974	Pafah2	NM_133880	0.0100887	1.14142	E11.5 * Sox10 up vs E11.5 * Ctrl
10509463	Eif4g3	NM_172703	0.00469122	1.14125	E11.5 * Sox10 up vs E11.5 * Ctrl
10548057	Ndufa9	NM_025358	0.00521793	1.14096	E11.5 * Sox10 up vs E11.5 * Ctrl
10514590	Dock7	NM_026082	0.000630283	1.14085	E11.5 * Sox10 up vs E11.5 * Ctrl
10459705	Smad4	NM_008540	0.000210144	1.13997	E11.5 * Sox10 up vs E11.5 * Ctrl
10580756	2310065K24Rik	NM_028221	0.00313929	1.13958	E11.5 * Sox10 up vs E11.5 * Ctrl
10372687	Nup107	NM_134010	0.0063053	1.13905	E11.5 * Sox10 up vs E11.5 * Ctrl
10466374	Tle4	NM_011600	0.0068174	1.13888	E11.5 * Sox10 up vs E11.5 * Ctrl
10457872	Slc39a6	NM_139143	0.00642367	1.13881	E11.5 * Sox10 up vs E11.5 * Ctrl
10560630	Tomm40	NM_001109748	0.0080202	1.1388	E11.5 * Sox10 up vs E11.5 * Ctrl
10356577	Ilkap	NM_023343	0.00993118	1.13826	E11.5 * Sox10 up vs E11.5 * Ctrl
10406417	Actg1	NM_009609	0.00129911	1.13823	E11.5 * Sox10 up vs E11.5 * Ctrl
10415119	Pabpn1	NM_019402	0.00102403	1.13759	E11.5 * Sox10 up vs E11.5 * Ctrl
10473919	Ckap5	NM_029437	0.000675017	1.13634	E11.5 * Sox10 up vs E11.5 * Ctrl
10578287	Cnot7	NM_011135	0.00496493	1.13598	E11.5 * Sox10 up vs E11.5 * Ctrl
10420089	Nedd8	NM_008683	0.0014908	1.13596	E11.5 * Sox10 up vs E11.5 * Ctrl
10422718	Ttc33	NM_026213	0.00864511	1.13573	E11.5 * Sox10 up vs E11.5 * Ctrl
10446109	Safb	ENSMUST00000(	0.000809674	1.13571	E11.5 * Sox10 up vs E11.5 * Ctrl
10486322		---	0.0090155	1.13506	E11.5 * Sox10 up vs E11.5 * Ctrl
10372750	Cand1	NM_027994	0.000496029	1.13495	E11.5 * Sox10 up vs E11.5 * Ctrl
10593367	Dlat	NM_145614	0.00348969	1.13489	E11.5 * Sox10 up vs E11.5 * Ctrl
10478196	Top1	NM_009408	0.000658651	1.13471	E11.5 * Sox10 up vs E11.5 * Ctrl
10531286	Vdac2	NM_011695	0.00159121	1.13449	E11.5 * Sox10 up vs E11.5 * Ctrl
10560282	Grlf1	NM_172739	0.0090284	1.13405	E11.5 * Sox10 up vs E11.5 * Ctrl
10501143	Ahcyl1	NM_145542	0.0017815	1.13356	E11.5 * Sox10 up vs E11.5 * Ctrl
10413304	Arf4	NM_007479	0.00208345	1.13351	E11.5 * Sox10 up vs E11.5 * Ctrl
10466402	Eif4a1	NM_144958	0.00297585	1.13349	E11.5 * Sox10 up vs E11.5 * Ctrl
10356859	Dtymk	NM_001105667	0.0017219	1.13326	E11.5 * Sox10 up vs E11.5 * Ctrl
10503134	Sdcbp	NM_001098227	4.38653e-005	1.13315	E11.5 * Sox10 up vs E11.5 * Ctrl

10435149	Fyttd1	NM_027226	0.000314006	1.13299	E11.5 * Sox10 up vs E11.5 * Ctrl
10412844	Top2b	NM_009409	0.00588906	1.13241	E11.5 * Sox10 up vs E11.5 * Ctrl
10450435	Csnk2b	NM_009975	0.010303	1.13227	E11.5 * Sox10 up vs E11.5 * Ctrl
10586844	Adam10	NM_007399	0.00318407	1.13169	E11.5 * Sox10 up vs E11.5 * Ctrl
10505182	Gng10	NM_025277	0.00172602	1.13165	E11.5 * Sox10 up vs E11.5 * Ctrl
10438308	Ranbp1	NM_011239	0.0026855	1.13155	E11.5 * Sox10 up vs E11.5 * Ctrl
10539177	Eif4a3	NM_138669	0.00214014	1.13143	E11.5 * Sox10 up vs E11.5 * Ctrl
10451778	EG328839	ENSMUST00000(	0.00918676	1.13137	E11.5 * Sox10 up vs E11.5 * Ctrl
10522716	Polr2b	NM_153798	0.000343229	1.13055	E11.5 * Sox10 up vs E11.5 * Ctrl
10378848	Hsp90aa1	NM_010480	0.00132081	1.13007	E11.5 * Sox10 up vs E11.5 * Ctrl
10559853	Clcn4-2	NM_011334	0.00787698	1.12999	E11.5 * Sox10 up vs E11.5 * Ctrl
10481900	Rabepk	NM_145522	0.00771554	1.12994	E11.5 * Sox10 up vs E11.5 * Ctrl
10426439	Pphln1	NM_146062	0.00527371	1.1292	E11.5 * Sox10 up vs E11.5 * Ctrl
10463557	Gbf1	NM_178930	0.0043823	1.12907	E11.5 * Sox10 up vs E11.5 * Ctrl
10454469	Ammecr1l	NM_153515	0.00140168	1.12851	E11.5 * Sox10 up vs E11.5 * Ctrl
10417689	Psmd6	NM_025550	0.0030732	1.12837	E11.5 * Sox10 up vs E11.5 * Ctrl
10583254	Cwc15	NM_023153	0.00195412	1.12829	E11.5 * Sox10 up vs E11.5 * Ctrl
10485013	1110051M20Rik	NM_175123	0.00659178	1.12765	E11.5 * Sox10 up vs E11.5 * Ctrl
10427885	Hnrnpa1	NM_010447	0.00296397	1.12738	E11.5 * Sox10 up vs E11.5 * Ctrl
10368928	Rpl41	NM_018860	0.000273379	1.12709	E11.5 * Sox10 up vs E11.5 * Ctrl
10412098	Rpl41	NM_018860	0.000273379	1.12709	E11.5 * Sox10 up vs E11.5 * Ctrl
10591867	Rpl41	NM_018860	0.000273379	1.12709	E11.5 * Sox10 up vs E11.5 * Ctrl
10541104	Hnrnpf	NM_133834	1.8211e-005	1.12685	E11.5 * Sox10 up vs E11.5 * Ctrl
10443550	Rnf8	NM_021419	0.00700402	1.12662	E11.5 * Sox10 up vs E11.5 * Ctrl
10377851	Psmb6	NM_008946	0.00276478	1.12628	E11.5 * Sox10 up vs E11.5 * Ctrl
10388010	C1qbp	NM_007573	0.000296203	1.12606	E11.5 * Sox10 up vs E11.5 * Ctrl
10385034	Rpsa	NM_011029	0.00536643	1.12594	E11.5 * Sox10 up vs E11.5 * Ctrl
10345981	Ercc5	NM_011729	0.00684952	1.1255	E11.5 * Sox10 up vs E11.5 * Ctrl
10394892	Cpsf3	NM_018813	0.00745735	1.12521	E11.5 * Sox10 up vs E11.5 * Ctrl
10543031	Slc25a13	NM_015829	0.00909654	1.12512	E11.5 * Sox10 up vs E11.5 * Ctrl
10452867	Dpy30	NM_001146222	0.00257527	1.12431	E11.5 * Sox10 up vs E11.5 * Ctrl
10435212	Osbpl11	NM_176840	0.00721058	1.12422	E11.5 * Sox10 up vs E11.5 * Ctrl
10400581	Fkbp3	NM_013902	0.00502303	1.12406	E11.5 * Sox10 up vs E11.5 * Ctrl
10504056	Ubap1	NM_023305	0.00682157	1.12251	E11.5 * Sox10 up vs E11.5 * Ctrl
10390352	Kpnb1	NM_008379	0.00139575	1.1221	E11.5 * Sox10 up vs E11.5 * Ctrl
10360863	Mark1	NM_145515	0.00976845	1.12182	E11.5 * Sox10 up vs E11.5 * Ctrl
10371987	Metap2	NM_019648	0.00132832	1.12098	E11.5 * Sox10 up vs E11.5 * Ctrl
10539741	Aak1	NM_001040106	0.00148282	1.12096	E11.5 * Sox10 up vs E11.5 * Ctrl
10544501	Ezh2	NM_007971	0.00664699	1.12087	E11.5 * Sox10 up vs E11.5 * Ctrl
10529142	Ppm1g	NM_008014	0.00772773	1.12061	E11.5 * Sox10 up vs E11.5 * Ctrl
10348929	Hnrnpf	NM_133834	4.71325e-005	1.12	E11.5 * Sox10 up vs E11.5 * Ctrl
10411491	Tnpo1	NM_178716	0.00547938	1.11989	E11.5 * Sox10 up vs E11.5 * Ctrl
10459375	Txnl1	NM_016792	0.00736992	1.11984	E11.5 * Sox10 up vs E11.5 * Ctrl
10500736	Vangl1	NM_177545	0.00916151	1.11964	E11.5 * Sox10 up vs E11.5 * Ctrl
10541067	Eif4a3	NM_138669	0.00233085	1.11952	E11.5 * Sox10 up vs E11.5 * Ctrl
10526120	Tpst1	NM_001130476	0.00941277	1.11942	E11.5 * Sox10 up vs E11.5 * Ctrl
10580704	Amfr	NM_011787	0.000294648	1.11872	E11.5 * Sox10 up vs E11.5 * Ctrl
10494910	Bcas2	NM_026602	0.00305848	1.11848	E11.5 * Sox10 up vs E11.5 * Ctrl
10586368	Clpx	NM_011802	0.00945912	1.11831	E11.5 * Sox10 up vs E11.5 * Ctrl
10525352	Ppp1cc	NM_013636	0.000453794	1.11795	E11.5 * Sox10 up vs E11.5 * Ctrl
10548785	Dynlt1	NM_009342	0.000196411	1.11783	E11.5 * Sox10 up vs E11.5 * Ctrl
10527598	Pomp	NM_025624	0.00787106	1.11727	E11.5 * Sox10 up vs E11.5 * Ctrl
10600324	Rpl3	NM_013762	7.59315e-005	1.11686	E11.5 * Sox10 up vs E11.5 * Ctrl
10478409	Pkig	NM_001039390	0.0081503	1.11618	E11.5 * Sox10 up vs E11.5 * Ctrl
10347163	Spag16	NM_029160	0.00576496	1.11558	E11.5 * Sox10 up vs E11.5 * Ctrl
10527888	Gatad1	NM_026033	0.00678709	1.11532	E11.5 * Sox10 up vs E11.5 * Ctrl
10348775	Ppp1r7	NM_023200	0.000255784	1.11438	E11.5 * Sox10 up vs E11.5 * Ctrl
10402615	Hsp90aa1	NM_010480	0.00250677	1.11412	E11.5 * Sox10 up vs E11.5 * Ctrl
10556764	Ppp1cc	NM_013636	0.000881083	1.11397	E11.5 * Sox10 up vs E11.5 * Ctrl
10380689	Cbx1	NM_007622	0.00695699	1.11361	E11.5 * Sox10 up vs E11.5 * Ctrl
10482181	Strbp	NM_009261	0.00688527	1.11344	E11.5 * Sox10 up vs E11.5 * Ctrl
10356351	Rpl6	NM_011290	0.00155014	1.11175	E11.5 * Sox10 up vs E11.5 * Ctrl
10462333	Cdc37l1	NM_025950	0.0102271	1.11154	E11.5 * Sox10 up vs E11.5 * Ctrl
10518642	Ube4b	NM_022022	0.00854598	1.11051	E11.5 * Sox10 up vs E11.5 * Ctrl
10524718	Rplp0	NM_007475	0.00481321	1.10959	E11.5 * Sox10 up vs E11.5 * Ctrl
10520010	Pmpcb	NM_028431	0.00407441	1.10913	E11.5 * Sox10 up vs E11.5 * Ctrl

10488929	Eif6	NM_010579	0.00961677	1.10899	E11.5 * Sox10 up vs E11.5 * Ctrl
10393754	Actg1	NM_009609	0.00273048	1.10838	E11.5 * Sox10 up vs E11.5 * Ctrl
10360794	Fbxo28	NM_175127	0.00267809	1.10771	E11.5 * Sox10 up vs E11.5 * Ctrl
10598721	Rpl3	NM_013762	6.69461e-006	1.10693	E11.5 * Sox10 up vs E11.5 * Ctrl
10568417	1110007A13Rik	BC038342	0.00475253	1.1064	E11.5 * Sox10 up vs E11.5 * Ctrl
10372600	Cct2	NM_007636	0.00148181	1.10443	E11.5 * Sox10 up vs E11.5 * Ctrl
10430669	Rpl3	NM_013762	1.26297e-005	1.10343	E11.5 * Sox10 up vs E11.5 * Ctrl
10432162	2310037I24Rik	BC003301	0.00294027	1.10323	E11.5 * Sox10 up vs E11.5 * Ctrl
10371981	Ccdc38	NM_175488	9.94768e-005	1.102	E11.5 * Sox10 up vs E11.5 * Ctrl
10571182	4921537P18Rik	NM_027618	0.00733064	1.10113	E11.5 * Sox10 up vs E11.5 * Ctrl
10546054	Rpl3	NM_013762	0.000172941	1.10088	E11.5 * Sox10 up vs E11.5 * Ctrl
10543054	Shfm1	NM_009169	0.000857408	1.09982	E11.5 * Sox10 up vs E11.5 * Ctrl
10525187	Rpl6	NM_011290	0.00226153	1.09979	E11.5 * Sox10 up vs E11.5 * Ctrl
10351703	Copa	NM_009938	0.00853096	1.09966	E11.5 * Sox10 up vs E11.5 * Ctrl
10549341	4933424B01Rik	NM_138757	0.00879578	1.09947	E11.5 * Sox10 up vs E11.5 * Ctrl
10533483	Atp2a2	NM_001110140	0.000860428	1.09918	E11.5 * Sox10 up vs E11.5 * Ctrl
10385686	Hnrnpab	NM_001048061	0.00206739	1.09833	E11.5 * Sox10 up vs E11.5 * Ctrl
10506424	Actg1	NM_009609	0.00600872	1.09741	E11.5 * Sox10 up vs E11.5 * Ctrl
10515154	Rpl3	NM_013762	0.000339723	1.09726	E11.5 * Sox10 up vs E11.5 * Ctrl
10395831	Brms1l	NM_001037756	0.00495724	1.09658	E11.5 * Sox10 up vs E11.5 * Ctrl
10369890	Cisd1	BC013522	0.00997445	1.09256	E11.5 * Sox10 up vs E11.5 * Ctrl
10506893	Nrd1	NM_146150	0.000912116	1.09192	E11.5 * Sox10 up vs E11.5 * Ctrl
10525657	Denr	NM_026603	0.00983542	1.08934	E11.5 * Sox10 up vs E11.5 * Ctrl
10378739	Ywhae	NM_009536	0.000531448	1.08891	E11.5 * Sox10 up vs E11.5 * Ctrl
10607388	Rpl7a	NM_013721	0.00926252	1.08685	E11.5 * Sox10 up vs E11.5 * Ctrl
10471256	Nup214	NM_172268	0.00514662	1.08528	E11.5 * Sox10 up vs E11.5 * Ctrl
10436694	Rplp0	NM_007475	0.0097698	1.08495	E11.5 * Sox10 up vs E11.5 * Ctrl
10416915	Rpl41	NM_018860	0.00075882	1.08324	E11.5 * Sox10 up vs E11.5 * Ctrl
10446785	Spast	NM_016962	0.00431712	1.08272	E11.5 * Sox10 up vs E11.5 * Ctrl
10485514	Caprin1	NM_016739	0.00276572	1.08253	E11.5 * Sox10 up vs E11.5 * Ctrl
10417749	Ube2e1	NM_009455	0.007394	1.08113	E11.5 * Sox10 up vs E11.5 * Ctrl
10366301	Rpl6	NM_011290	0.00480377	1.07881	E11.5 * Sox10 up vs E11.5 * Ctrl
10587546	Rpl7a	NM_013721	0.00883845	1.07859	E11.5 * Sox10 up vs E11.5 * Ctrl
10400470	Cox6c	NM_053071	0.000355346	1.07644	E11.5 * Sox10 up vs E11.5 * Ctrl
10568532	Rpl7a	NM_013721	0.00945959	1.07337	E11.5 * Sox10 up vs E11.5 * Ctrl
10544015	Ybx1	NM_011732	0.00681132	1.06808	E11.5 * Sox10 up vs E11.5 * Ctrl
10567020	Rpl7a	BC091731	0.00613864	1.06639	E11.5 * Sox10 up vs E11.5 * Ctrl
10411363	5730427N09Rik	NM_021552	0.00283535	1.0653	E11.5 * Sox10 up vs E11.5 * Ctrl
10427241	Pcbp2	NM_001103165	0.00195212	1.06373	E11.5 * Sox10 up vs E11.5 * Ctrl
10418498	Rpl7a	NM_013721	0.00453212	1.06297	E11.5 * Sox10 up vs E11.5 * Ctrl
10594752	Rpl7a	NM_013721	0.0021192	1.05857	E11.5 * Sox10 up vs E11.5 * Ctrl
10432527	Lass5	NM_028015	0.00274635	1.05564	E11.5 * Sox10 up vs E11.5 * Ctrl
10451301	Rpl11	NM_025919	0.000590133	1.05507	E11.5 * Sox10 up vs E11.5 * Ctrl
10457731	Rpl35	NM_025592	0.00482915	1.05471	E11.5 * Sox10 up vs E11.5 * Ctrl
10404262	Rps8	NM_009098	0.00428771	1.05268	E11.5 * Sox10 up vs E11.5 * Ctrl
10428554	Eif3h	NM_080635	0.00672349	1.05186	E11.5 * Sox10 up vs E11.5 * Ctrl
10414313	Ubb	NM_011664	0.00205979	1.05139	E11.5 * Sox10 up vs E11.5 * Ctrl
10482267	Rpl35	NM_025592	0.00610755	1.04904	E11.5 * Sox10 up vs E11.5 * Ctrl
10350816	Rpl35	NM_025592	0.00513875	1.04888	E11.5 * Sox10 up vs E11.5 * Ctrl
10376864	Ubb	NM_011664	0.00435238	1.04814	E11.5 * Sox10 up vs E11.5 * Ctrl
10454097	Rpl11	NM_025919	0.0026912	1.04633	E11.5 * Sox10 up vs E11.5 * Ctrl
10594142	Rpl11	NM_025919	0.000833882	1.04359	E11.5 * Sox10 up vs E11.5 * Ctrl
10517457	Rpl11	NM_025919	0.00311984	1.03971	E11.5 * Sox10 up vs E11.5 * Ctrl
10502745	Rpl11	NM_025919	0.00804183	1.03837	E11.5 * Sox10 up vs E11.5 * Ctrl
10550052	Rps5	NM_009095	0.00704566	1.03418	E11.5 * Sox10 up vs E11.5 * Ctrl
10607324	Rpl21	NM_019647	0.0066424	-1.03485	E11.5 * Sox10 down vs E11.5 * Ctrl
10424553	EG668319	NM_001111313	0.0086322	-1.03518	E11.5 * Sox10 down vs E11.5 * Ctrl
10401695	Rpl21	BC106116	0.00988692	-1.03764	E11.5 * Sox10 down vs E11.5 * Ctrl
10579839	EG668319	NM_001111313	0.00232648	-1.04034	E11.5 * Sox10 down vs E11.5 * Ctrl
10455338	Rpl21	NM_019647	0.00386719	-1.04065	E11.5 * Sox10 down vs E11.5 * Ctrl
10599433	EG668319	NM_001111313	0.00331227	-1.04218	E11.5 * Sox10 down vs E11.5 * Ctrl
10392996	EG668319	NM_001111313	0.00226763	-1.04358	E11.5 * Sox10 down vs E11.5 * Ctrl
10567823	EG668319	NM_001111313	0.00288746	-1.04548	E11.5 * Sox10 down vs E11.5 * Ctrl
10562663	EG668668	NM_001081036	0.0094484	-1.04739	E11.5 * Sox10 down vs E11.5 * Ctrl
10546292	Rpl21	BC094410	0.0104396	-1.04984	E11.5 * Sox10 down vs E11.5 * Ctrl
10531144	EG668319	NM_001111313	0.0061238	-1.0501	E11.5 * Sox10 down vs E11.5 * Ctrl

10595604	Syncrip	NM_019796	0.00630366	-1.05019	E11.5 * Sox10 down vs E11.5 * Ctrl
10423963	Eny2	NM_175009	0.0084243	-1.05397	E11.5 * Sox10 down vs E11.5 * Ctrl
10473006	EG668319	NM_001111313	0.00490079	-1.05657	E11.5 * Sox10 down vs E11.5 * Ctrl
10543317	Rpl21	NM_019647	0.00122644	-1.05955	E11.5 * Sox10 down vs E11.5 * Ctrl
10412665	Rpl21	BC138299	0.00204199	-1.06427	E11.5 * Sox10 down vs E11.5 * Ctrl
10598636	Ube2i	NM_011665	0.00649022	-1.07122	E11.5 * Sox10 down vs E11.5 * Ctrl
10359525	Bat2d	NM_001081290	0.00254969	-1.07462	E11.5 * Sox10 down vs E11.5 * Ctrl
10389882	3300001P08Rik	NM_026313	0.00871398	-1.07758	E11.5 * Sox10 down vs E11.5 * Ctrl
10380210	Sfrs1	NM_173374	0.00111727	-1.08064	E11.5 * Sox10 down vs E11.5 * Ctrl
10507011	Dmrta2	NM_172296	0.00693129	-1.08449	E11.5 * Sox10 down vs E11.5 * Ctrl
10515337	Nasp	NM_016777	0.00910253	-1.08616	E11.5 * Sox10 down vs E11.5 * Ctrl
10404020	V1rh2	NM_145844	0.00309582	-1.08729	E11.5 * Sox10 down vs E11.5 * Ctrl
10547436	Wnk1	NM_198703	0.00965346	-1.08786	E11.5 * Sox10 down vs E11.5 * Ctrl
10577352	Defb12	NM_152802	0.0103083	-1.09248	E11.5 * Sox10 down vs E11.5 * Ctrl
10414817	Tcra	U07662	0.00777916	-1.09407	E11.5 * Sox10 down vs E11.5 * Ctrl
10461071	2700081O15Rik	NM_175381	0.00612561	-1.0942	E11.5 * Sox10 down vs E11.5 * Ctrl
10428912		---	0.00722737	-1.09603	E11.5 * Sox10 down vs E11.5 * Ctrl
10557206	Rbbp6	NM_175023	0.0028213	-1.09689	E11.5 * Sox10 down vs E11.5 * Ctrl
10367477	Olfr774	NM_207620	0.00425658	-1.10057	E11.5 * Sox10 down vs E11.5 * Ctrl
10405163	Spin1	NM_146043	0.00469452	-1.10434	E11.5 * Sox10 down vs E11.5 * Ctrl
10511629	ENSMUSG0000C	NR_003968	0.00915574	-1.10455	E11.5 * Sox10 down vs E11.5 * Ctrl
10598020		---	0.000975134	-1.10459	E11.5 * Sox10 down vs E11.5 * Ctrl
10357965	Lgr6	NM_001033409	0.00458267	-1.10492	E11.5 * Sox10 down vs E11.5 * Ctrl
10591773	Hmgn2	NM_016957	0.000959527	-1.10562	E11.5 * Sox10 down vs E11.5 * Ctrl
10349100	EG626075	XR_032823	0.0100074	-1.10596	E11.5 * Sox10 down vs E11.5 * Ctrl
10577666	Adam18	NM_010084	0.00945055	-1.1065	E11.5 * Sox10 down vs E11.5 * Ctrl
10541581	Clec4b1	NM_027218	0.00481023	-1.10756	E11.5 * Sox10 down vs E11.5 * Ctrl
10522472	Fip1l1	NM_001159573	0.00659021	-1.10785	E11.5 * Sox10 down vs E11.5 * Ctrl
10500813	Hipk1	NM_010432	0.00553817	-1.10921	E11.5 * Sox10 down vs E11.5 * Ctrl
10517141	Hmgn2	NM_016957	0.000601771	-1.1094	E11.5 * Sox10 down vs E11.5 * Ctrl
10405400	Nsd1	NM_008739	0.00794975	-1.10965	E11.5 * Sox10 down vs E11.5 * Ctrl
10530666	Lnx1	NM_010727	0.00551431	-1.11142	E11.5 * Sox10 down vs E11.5 * Ctrl
10448004	Phf10	NM_024250	0.00412463	-1.1116	E11.5 * Sox10 down vs E11.5 * Ctrl
10579659	Hmgn2	NM_016957	0.000830567	-1.11203	E11.5 * Sox10 down vs E11.5 * Ctrl
10506500	Usp24	ENSMUST00000(	0.00380122	-1.11299	E11.5 * Sox10 down vs E11.5 * Ctrl
10395532		---	0.00877076	-1.11302	E11.5 * Sox10 down vs E11.5 * Ctrl
10436873	Son	NM_178880	0.00955216	-1.11334	E11.5 * Sox10 down vs E11.5 * Ctrl
10417601	Ptma	NM_008972	0.000426349	-1.11417	E11.5 * Sox10 down vs E11.5 * Ctrl
10566097	Nup98	NM_022979	0.00888622	-1.11485	E11.5 * Sox10 down vs E11.5 * Ctrl
10522024	Tbc1d1	NM_019636	0.00749688	-1.11518	E11.5 * Sox10 down vs E11.5 * Ctrl
10508490	Sfrs5	NM_001079695	0.00408783	-1.11597	E11.5 * Sox10 down vs E11.5 * Ctrl
10477966	Manbal	NM_026968	0.00798546	-1.11603	E11.5 * Sox10 down vs E11.5 * Ctrl
10555729	Olfr555	NM_147103	0.00717044	-1.11654	E11.5 * Sox10 down vs E11.5 * Ctrl
10492045	Hnrnpa3	NM_053263	0.0089936	-1.11662	E11.5 * Sox10 down vs E11.5 * Ctrl
10458138	Brd8	NM_030147	0.00678501	-1.11706	E11.5 * Sox10 down vs E11.5 * Ctrl
10499716	Ubap2l	NM_028475	0.00230448	-1.11719	E11.5 * Sox10 down vs E11.5 * Ctrl
10396237	2700049A03Rik	BC150744	0.000655346	-1.11757	E11.5 * Sox10 down vs E11.5 * Ctrl
10418616	Dnahc1	BC023155	0.00921179	-1.11757	E11.5 * Sox10 down vs E11.5 * Ctrl
10414581	AY358078	AY358078	0.00875683	-1.1177	E11.5 * Sox10 down vs E11.5 * Ctrl
10435714	Tmem39a	NM_026407	0.00590606	-1.11806	E11.5 * Sox10 down vs E11.5 * Ctrl
10348078	Ptma	NM_008972	8.77112e-005	-1.11841	E11.5 * Sox10 down vs E11.5 * Ctrl
10473008	Hnrnpa3	NM_146130	0.00678166	-1.11925	E11.5 * Sox10 down vs E11.5 * Ctrl
10447141	Eml4	NM_001114361	0.00815271	-1.11997	E11.5 * Sox10 down vs E11.5 * Ctrl
10360848	Mosc2	NM_133684	0.0091404	-1.1212	E11.5 * Sox10 down vs E11.5 * Ctrl
10350535	Tpr	NM_133780	0.00824829	-1.12146	E11.5 * Sox10 down vs E11.5 * Ctrl
10602501	Huwe1	NM_021523	0.000368951	-1.12195	E11.5 * Sox10 down vs E11.5 * Ctrl
10598067		---	0.00167938	-1.12209	E11.5 * Sox10 down vs E11.5 * Ctrl
10440180	Olfr205	NM_001011736	0.00471461	-1.12224	E11.5 * Sox10 down vs E11.5 * Ctrl
10560744	V1rd15	NM_203489	0.0094336	-1.12265	E11.5 * Sox10 down vs E11.5 * Ctrl
10489018	Rbm39	NM_133242	0.00214398	-1.12298	E11.5 * Sox10 down vs E11.5 * Ctrl
10438942	Tmem44	NM_172614	0.00684943	-1.12349	E11.5 * Sox10 down vs E11.5 * Ctrl
10363007	Ascc3	NM_001146089	0.00151011	-1.12668	E11.5 * Sox10 down vs E11.5 * Ctrl
10598029	ND1	ENSMUST00000(	0.00135595	-1.12673	E11.5 * Sox10 down vs E11.5 * Ctrl
10597493	Stt3b	NM_024222	0.00187229	-1.12683	E11.5 * Sox10 down vs E11.5 * Ctrl
10579663	Eps15l1	NM_007944	0.00312656	-1.12722	E11.5 * Sox10 down vs E11.5 * Ctrl
10534964	Rps25	NM_024266	0.00869309	-1.12732	E11.5 * Sox10 down vs E11.5 * Ctrl

10397257	Vsx2	NM_007701	0.00601065	-1.12776	E11.5 * Sox10 down vs E11.5 * Ctrl
10580516	EG625801	XR_031896	0.00603799	-1.12797	E11.5 * Sox10 down vs E11.5 * Ctrl
10516103	Macf1	NM_009600	0.00298706	-1.12869	E11.5 * Sox10 down vs E11.5 * Ctrl
10598025		---	0.000453851	-1.1288	E11.5 * Sox10 down vs E11.5 * Ctrl
10503570	Sfrs18	NM_025669	0.00712556	-1.12903	E11.5 * Sox10 down vs E11.5 * Ctrl
10492558	Smc4	NM_133786	0.00757312	-1.12973	E11.5 * Sox10 down vs E11.5 * Ctrl
10437454	Anks3	NM_028301	0.00135654	-1.12983	E11.5 * Sox10 down vs E11.5 * Ctrl
10367501		---	0.00396151	-1.13008	E11.5 * Sox10 down vs E11.5 * Ctrl
10390707	Top2a	NM_011623	0.00475181	-1.13064	E11.5 * Sox10 down vs E11.5 * Ctrl
10576965		---	0.00436013	-1.13101	E11.5 * Sox10 down vs E11.5 * Ctrl
10436804	Mrap	NM_029844	0.00726933	-1.13192	E11.5 * Sox10 down vs E11.5 * Ctrl
10576386	Rhou	NM_133955	0.00932791	-1.13194	E11.5 * Sox10 down vs E11.5 * Ctrl
10583753	EG624219	NM_001080940	0.00678516	-1.13241	E11.5 * Sox10 down vs E11.5 * Ctrl
10502982	Tnni3k	NM_177066	0.0076927	-1.13316	E11.5 * Sox10 down vs E11.5 * Ctrl
10443438	Pnpla1	NM_001034885	0.0025135	-1.13318	E11.5 * Sox10 down vs E11.5 * Ctrl
10493842	Pglyrp3	NM_207247	0.00425995	-1.13325	E11.5 * Sox10 down vs E11.5 * Ctrl
10366667	Gns	NM_029364	0.00310496	-1.13342	E11.5 * Sox10 down vs E11.5 * Ctrl
10455342		---	0.00359493	-1.13361	E11.5 * Sox10 down vs E11.5 * Ctrl
10529226	Rbks	NM_153196	0.00377147	-1.13414	E11.5 * Sox10 down vs E11.5 * Ctrl
10360589	Ahctf1	NM_026375	0.000872148	-1.13493	E11.5 * Sox10 down vs E11.5 * Ctrl
10405999	8030423J24Rik	ENSMUST00000(	0.00605203	-1.13498	E11.5 * Sox10 down vs E11.5 * Ctrl
10551293	Cyp2f2	NM_007817	0.00825276	-1.13584	E11.5 * Sox10 down vs E11.5 * Ctrl
10417004	Dzip1	NM_025943	0.00020047	-1.13627	E11.5 * Sox10 down vs E11.5 * Ctrl
10502898	Ube2l3	NM_009456	0.00354313	-1.13651	E11.5 * Sox10 down vs E11.5 * Ctrl
10458046	D0H4S114	NM_053078	0.00325264	-1.13686	E11.5 * Sox10 down vs E11.5 * Ctrl
10437332	Crebbp	NM_001025432	0.00067888	-1.13808	E11.5 * Sox10 down vs E11.5 * Ctrl
10344939	Terf1	NM_009352	0.00701517	-1.13871	E11.5 * Sox10 down vs E11.5 * Ctrl
10396926	Sfrs5	NM_001079695	0.00273399	-1.13878	E11.5 * Sox10 down vs E11.5 * Ctrl
10537957	Krba1	NM_133922	0.0088325	-1.1388	E11.5 * Sox10 down vs E11.5 * Ctrl
10489936	Adnp	NM_009628	0.00355042	-1.13888	E11.5 * Sox10 down vs E11.5 * Ctrl
10371959	Elk3	NM_013508	0.00882494	-1.13917	E11.5 * Sox10 down vs E11.5 * Ctrl
10387568	Tnfsf12-tnfsf13	NM_001034097	0.00579709	-1.13934	E11.5 * Sox10 down vs E11.5 * Ctrl
10587075		---	0.00532635	-1.14002	E11.5 * Sox10 down vs E11.5 * Ctrl
10599781	Htatsf1	NM_028242	0.00851771	-1.14016	E11.5 * Sox10 down vs E11.5 * Ctrl
10479249	Cdh26	NM_198656	0.00332587	-1.14026	E11.5 * Sox10 down vs E11.5 * Ctrl
10352556	100042375	XR_032441	0.00857989	-1.14056	E11.5 * Sox10 down vs E11.5 * Ctrl
10452508	Twsg1	NM_023053	0.00299961	-1.14066	E11.5 * Sox10 down vs E11.5 * Ctrl
10541290	Pex26	NM_028730	0.00855012	-1.14095	E11.5 * Sox10 down vs E11.5 * Ctrl
10370303	Adarb1	NR_004429	0.00772807	-1.14238	E11.5 * Sox10 down vs E11.5 * Ctrl
10355069	Ino80d	NM_001114609	0.000219845	-1.14254	E11.5 * Sox10 down vs E11.5 * Ctrl
10594825	Aqp9	NM_022026	0.00606218	-1.14273	E11.5 * Sox10 down vs E11.5 * Ctrl
10600547	Tbl1x	NM_020601	0.00250886	-1.14278	E11.5 * Sox10 down vs E11.5 * Ctrl
10408915	Dtnbp1	NM_025772	0.0032994	-1.14305	E11.5 * Sox10 down vs E11.5 * Ctrl
10580905	Cnot1	NM_153164	9.55397e-005	-1.1432	E11.5 * Sox10 down vs E11.5 * Ctrl
10578941	Trim60	NM_153097	0.00899966	-1.14352	E11.5 * Sox10 down vs E11.5 * Ctrl
10409152	1110007C09Rik	NM_026738	0.00762813	-1.14389	E11.5 * Sox10 down vs E11.5 * Ctrl
10394477	Laptm4a	NM_008640	0.000558721	-1.14389	E11.5 * Sox10 down vs E11.5 * Ctrl
10557315	4930571K23Rik	NM_001145759	0.00903855	-1.14396	E11.5 * Sox10 down vs E11.5 * Ctrl
10570697		---	0.0087154	-1.14424	E11.5 * Sox10 down vs E11.5 * Ctrl
10598069	CYTB	ENSMUST00000(	6.47874e-006	-1.14509	E11.5 * Sox10 down vs E11.5 * Ctrl
10377319	Myh10	NM_175260	0.00832415	-1.14554	E11.5 * Sox10 down vs E11.5 * Ctrl
10438530	Clcn2	NM_009900	0.000478362	-1.14568	E11.5 * Sox10 down vs E11.5 * Ctrl
10607425	EG434881	NM_001037167	0.00755424	-1.14589	E11.5 * Sox10 down vs E11.5 * Ctrl
10382795	OTTMUSG00000	XR_034731	0.00538127	-1.14659	E11.5 * Sox10 down vs E11.5 * Ctrl
10375732	Cby3	ENSMUST00000(	0.00265209	-1.14701	E11.5 * Sox10 down vs E11.5 * Ctrl
10374500	Vps54	NM_139061	0.000276873	-1.14728	E11.5 * Sox10 down vs E11.5 * Ctrl
10408175	Btn1a1	NM_013483	0.0036429	-1.14736	E11.5 * Sox10 down vs E11.5 * Ctrl
10495873	Ptma	NM_008972	0.00292514	-1.14753	E11.5 * Sox10 down vs E11.5 * Ctrl
10398392		---	0.00601878	-1.14863	E11.5 * Sox10 down vs E11.5 * Ctrl
10506843	Cc2d1b	NM_177045	0.00866122	-1.14864	E11.5 * Sox10 down vs E11.5 * Ctrl
10353727		---	0.00910159	-1.14869	E11.5 * Sox10 down vs E11.5 * Ctrl
10450325	Cfb	NM_008198	0.006333	-1.14878	E11.5 * Sox10 down vs E11.5 * Ctrl
10601988	Trap1a	NM_011635	0.00435141	-1.14884	E11.5 * Sox10 down vs E11.5 * Ctrl
10544610	Igf2bp3	NM_023670	0.00945097	-1.14885	E11.5 * Sox10 down vs E11.5 * Ctrl
10404593		---	0.00542939	-1.14942	E11.5 * Sox10 down vs E11.5 * Ctrl
10357064	Vps4b	NM_009190	0.00671879	-1.15019	E11.5 * Sox10 down vs E11.5 * Ctrl

10453256	Kcng3	NM_153512	0.00530463	-1.15046	E11.5 * Sox10 down vs E11.5 * Ctrl
10366238	Ppp1r12a	NM_027892	0.000215331	-1.15096	E11.5 * Sox10 down vs E11.5 * Ctrl
10557587	Zfp771	NM_177362	0.00305138	-1.15189	E11.5 * Sox10 down vs E11.5 * Ctrl
10515201	Cyp4b1	NM_007823	0.00263717	-1.15239	E11.5 * Sox10 down vs E11.5 * Ctrl
10494583	Sec22b	NM_011342	0.000786707	-1.15275	E11.5 * Sox10 down vs E11.5 * Ctrl
10526195	Gtf2ird2	NM_053266	0.000898109	-1.15283	E11.5 * Sox10 down vs E11.5 * Ctrl
10577973	Adrb3	NM_013462	0.00790207	-1.15344	E11.5 * Sox10 down vs E11.5 * Ctrl
10492803		---	0.00486075	-1.15361	E11.5 * Sox10 down vs E11.5 * Ctrl
10368380	L3mbtl3	NM_172787	0.00284031	-1.15374	E11.5 * Sox10 down vs E11.5 * Ctrl
10537397	Luc7l2	NM_138680	0.000224096	-1.15384	E11.5 * Sox10 down vs E11.5 * Ctrl
10532040	Zfp644	NM_026856	0.00353445	-1.15438	E11.5 * Sox10 down vs E11.5 * Ctrl
10425177	Triobp	NM_001039156	0.00332415	-1.15453	E11.5 * Sox10 down vs E11.5 * Ctrl
10434799		---	0.00825996	-1.15453	E11.5 * Sox10 down vs E11.5 * Ctrl
10387029	Dnahc9	NM_001099633	0.00240495	-1.15529	E11.5 * Sox10 down vs E11.5 * Ctrl
10570614	Defb6	NM_054074	0.00887958	-1.15592	E11.5 * Sox10 down vs E11.5 * Ctrl
10558285	Zranb1	NM_207302	0.00812639	-1.15609	E11.5 * Sox10 down vs E11.5 * Ctrl
10552632	Shank1	ENSMUST00000	0.00332336	-1.15636	E11.5 * Sox10 down vs E11.5 * Ctrl
10388086	Nlrp1c	NM_001039234	0.00971225	-1.15658	E11.5 * Sox10 down vs E11.5 * Ctrl
10569057	Rnh1	NM_145135	0.001941	-1.15675	E11.5 * Sox10 down vs E11.5 * Ctrl
10431014	Vkorc1l1	NM_027121	0.00893127	-1.15687	E11.5 * Sox10 down vs E11.5 * Ctrl
10582390	Aprt	NM_009698	0.00573329	-1.15707	E11.5 * Sox10 down vs E11.5 * Ctrl
10607712	Grpr	NM_008177	0.00282655	-1.15725	E11.5 * Sox10 down vs E11.5 * Ctrl
10446136	Tmem146	BC116633	0.00416164	-1.15748	E11.5 * Sox10 down vs E11.5 * Ctrl
10527832	Pds5b	NM_175310	0.000392687	-1.15757	E11.5 * Sox10 down vs E11.5 * Ctrl
10416421	Lrch1	NM_001033439	0.00699788	-1.15827	E11.5 * Sox10 down vs E11.5 * Ctrl
10422912	2410089E03Rik	BC058107	3.16979e-005	-1.15939	E11.5 * Sox10 down vs E11.5 * Ctrl
10529052	Slc5a6	NM_177870	0.00837902	-1.16067	E11.5 * Sox10 down vs E11.5 * Ctrl
10461867	Vps13a	AK015803	0.00815302	-1.16079	E11.5 * Sox10 down vs E11.5 * Ctrl
10528183	Speer4e	NM_001122661	0.00037394	-1.1611	E11.5 * Sox10 down vs E11.5 * Ctrl
10471677	Dab2ip	NM_001114124	0.00308665	-1.16116	E11.5 * Sox10 down vs E11.5 * Ctrl
10530151	Tlr6	NM_011604	0.00696747	-1.16119	E11.5 * Sox10 down vs E11.5 * Ctrl
10376895		---	0.00304072	-1.16123	E11.5 * Sox10 down vs E11.5 * Ctrl
10419296	Wdhd1	NM_172598	0.00354533	-1.16134	E11.5 * Sox10 down vs E11.5 * Ctrl
10406403		---	0.00350093	-1.16136	E11.5 * Sox10 down vs E11.5 * Ctrl
10601691	Arl13a	BC100522	0.00415542	-1.16139	E11.5 * Sox10 down vs E11.5 * Ctrl
10542632	Iapp	NM_010491	0.0041928	-1.16151	E11.5 * Sox10 down vs E11.5 * Ctrl
10438178	4930451C15Rik	NM_029053	0.00993163	-1.16159	E11.5 * Sox10 down vs E11.5 * Ctrl
10401296	Slc8a3	NM_080440	0.00841176	-1.16165	E11.5 * Sox10 down vs E11.5 * Ctrl
10519942	Magi2	ENSMUST00000(	0.00509791	-1.16168	E11.5 * Sox10 down vs E11.5 * Ctrl
10420775		---	0.00798259	-1.16194	E11.5 * Sox10 down vs E11.5 * Ctrl
10457637		---	0.0077377	-1.16198	E11.5 * Sox10 down vs E11.5 * Ctrl
10526106	Vkorc1l1	NM_027121	0.000542559	-1.16223	E11.5 * Sox10 down vs E11.5 * Ctrl
10399198	Ncoa4	NM_019744	0.00475077	-1.16228	E11.5 * Sox10 down vs E11.5 * Ctrl
10571371	Tusc3	NM_030254	0.0012462	-1.1624	E11.5 * Sox10 down vs E11.5 * Ctrl
10426875	Atf1	NM_007497	0.00437392	-1.1627	E11.5 * Sox10 down vs E11.5 * Ctrl
10364984	Zbtb7a	NM_010731	0.0067532	-1.16281	E11.5 * Sox10 down vs E11.5 * Ctrl
10479938	Echdc3	NM_024208	0.00468644	-1.16284	E11.5 * Sox10 down vs E11.5 * Ctrl
10548779	Pbp2	NM_029595	0.0101655	-1.16288	E11.5 * Sox10 down vs E11.5 * Ctrl
10507817	Bmp8b	NM_007559	0.00882509	-1.16292	E11.5 * Sox10 down vs E11.5 * Ctrl
10372082	Nudt4	NM_027722	0.00873231	-1.16326	E11.5 * Sox10 down vs E11.5 * Ctrl
10527575	Pan3	NM_028291	0.00250779	-1.16373	E11.5 * Sox10 down vs E11.5 * Ctrl
10385642	9630041N07Rik	NM_173387	0.00376931	-1.16405	E11.5 * Sox10 down vs E11.5 * Ctrl
10440717	Krtap6-1	ENSMUST00000(	0.00482517	-1.16406	E11.5 * Sox10 down vs E11.5 * Ctrl
10563116	Flt3l	NM_013520	0.00184352	-1.16462	E11.5 * Sox10 down vs E11.5 * Ctrl
10391914	Sh3d20	NM_183288	0.00328687	-1.16473	E11.5 * Sox10 down vs E11.5 * Ctrl
10453272	Zfp36l2	NM_001001806	0.00793967	-1.16531	E11.5 * Sox10 down vs E11.5 * Ctrl
10567964	Cln3	NM_001146311	0.00640294	-1.16532	E11.5 * Sox10 down vs E11.5 * Ctrl
10527694	B3galtl	NM_001081204	0.00607231	-1.1657	E11.5 * Sox10 down vs E11.5 * Ctrl
10365134		---	0.00219922	-1.16582	E11.5 * Sox10 down vs E11.5 * Ctrl
10381172	Stat5a	NM_011488	0.00228462	-1.16583	E11.5 * Sox10 down vs E11.5 * Ctrl
10413839	Ncoa4	NM_001033988	0.00182105	-1.16602	E11.5 * Sox10 down vs E11.5 * Ctrl
10477058	Scrt2	ENSMUST00000(	0.00130018	-1.16607	E11.5 * Sox10 down vs E11.5 * Ctrl
10383423	Anapc11	NM_025389	0.00744282	-1.16648	E11.5 * Sox10 down vs E11.5 * Ctrl
10601404	EG331493	NM_001033541	0.00800815	-1.1668	E11.5 * Sox10 down vs E11.5 * Ctrl
10374020	Rhbdd3	NM_177370	0.000452705	-1.16733	E11.5 * Sox10 down vs E11.5 * Ctrl
10373651	Olfr814	ENSMUST00000(	0.00577526	-1.16788	E11.5 * Sox10 down vs E11.5 * Ctrl

10348150	Chrnd	NM_021600	0.00534218	-1.16835	E11.5 * Sox10 down vs E11.5 * Ctrl
10603026	Ctps2	NM_018737	0.0026789	-1.16844	E11.5 * Sox10 down vs E11.5 * Ctrl
10577948	Brf2	NM_025686	0.0093296	-1.16871	E11.5 * Sox10 down vs E11.5 * Ctrl
10508797	ENSMUSG0000C	ENSMUST00000(	0.00145747	-1.1691	E11.5 * Sox10 down vs E11.5 * Ctrl
10503303	1110037F02Rik	NM_001081183	0.00382497	-1.16926	E11.5 * Sox10 down vs E11.5 * Ctrl
10344772	6030422M02Rik	NM_177722	0.00922645	-1.16941	E11.5 * Sox10 down vs E11.5 * Ctrl
10577421	Defcr-rs1	NM_007844	0.00475171	-1.16946	E11.5 * Sox10 down vs E11.5 * Ctrl
10513320	Ptgr1	NM_025968	0.00333626	-1.16959	E11.5 * Sox10 down vs E11.5 * Ctrl
10401114	Rab15	NM_134050	0.00263831	-1.16964	E11.5 * Sox10 down vs E11.5 * Ctrl
10409905	Ctsr	NM_020284	0.00904472	-1.16976	E11.5 * Sox10 down vs E11.5 * Ctrl
10577757	Adam9	NM_007404	0.00653418	-1.16996	E11.5 * Sox10 down vs E11.5 * Ctrl
10548884	Erp27	NM_026983	0.00831699	-1.17012	E11.5 * Sox10 down vs E11.5 * Ctrl
10366038	Galnt4	NM_015737	0.00600051	-1.17021	E11.5 * Sox10 down vs E11.5 * Ctrl
10490304	Sycp2	NM_177191	0.00292945	-1.1704	E11.5 * Sox10 down vs E11.5 * Ctrl
10346651	Bmpr2	NM_007561	0.000243945	-1.17068	E11.5 * Sox10 down vs E11.5 * Ctrl
10598369	Prickle3	NM_175097	0.00157901	-1.17095	E11.5 * Sox10 down vs E11.5 * Ctrl
10554045	Adamts17	NM_001033877	0.000879962	-1.17112	E11.5 * Sox10 down vs E11.5 * Ctrl
10345626	Eif5b	NM_198303	0.00885831	-1.17142	E11.5 * Sox10 down vs E11.5 * Ctrl
10453721	Wac	NM_153085	0.0039258	-1.17158	E11.5 * Sox10 down vs E11.5 * Ctrl
10461526	Vwce	NM_027913	0.00931571	-1.17158	E11.5 * Sox10 down vs E11.5 * Ctrl
10605499	3426406K10Rik	AK132212	0.000903951	-1.17181	E11.5 * Sox10 down vs E11.5 * Ctrl
10434815	Trp63	NM_001127259	0.0084851	-1.17237	E11.5 * Sox10 down vs E11.5 * Ctrl
10353878	Ankrd23	NM_153502	0.00172513	-1.17256	E11.5 * Sox10 down vs E11.5 * Ctrl
10503711	Casp8ap2	NM_011997	0.0043758	-1.17266	E11.5 * Sox10 down vs E11.5 * Ctrl
10539617	Alms1	NM_145223	0.00871651	-1.17268	E11.5 * Sox10 down vs E11.5 * Ctrl
10361338	Ipcef1	NM_001033391	0.00834526	-1.17272	E11.5 * Sox10 down vs E11.5 * Ctrl
10516823	Epb4.1	NM_183428	2.37425e-005	-1.17298	E11.5 * Sox10 down vs E11.5 * Ctrl
10430647		---	0.00481627	-1.17299	E11.5 * Sox10 down vs E11.5 * Ctrl
10525780	Tctn2	NM_026486	0.00669412	-1.17322	E11.5 * Sox10 down vs E11.5 * Ctrl
10585318	Rdx	NM_001104617	0.00505368	-1.17398	E11.5 * Sox10 down vs E11.5 * Ctrl
10520648		---	0.00275592	-1.17469	E11.5 * Sox10 down vs E11.5 * Ctrl
10539873	Gata2	NM_008090	0.0103857	-1.17579	E11.5 * Sox10 down vs E11.5 * Ctrl
10535329	Papolb	NM_019943	0.00727953	-1.17587	E11.5 * Sox10 down vs E11.5 * Ctrl
10385747	Phf15	NM_199299	0.00825986	-1.17643	E11.5 * Sox10 down vs E11.5 * Ctrl
10551365	Prx	NM_019412	0.00355925	-1.1766	E11.5 * Sox10 down vs E11.5 * Ctrl
10455954	EG240327	NM_001033767	0.00357476	-1.17677	E11.5 * Sox10 down vs E11.5 * Ctrl
10492442		---	0.000641801	-1.177	E11.5 * Sox10 down vs E11.5 * Ctrl
10462480	Cstf2t	NM_031249	0.0043729	-1.17731	E11.5 * Sox10 down vs E11.5 * Ctrl
10484575	Olfr1048	NM_147014	0.00224784	-1.17751	E11.5 * Sox10 down vs E11.5 * Ctrl
10561247	Shkbp1	NM_138676	0.00980333	-1.17761	E11.5 * Sox10 down vs E11.5 * Ctrl
10398426		---	0.00993005	-1.17769	E11.5 * Sox10 down vs E11.5 * Ctrl
10383953	Emid1	NM_080595	0.00429027	-1.17776	E11.5 * Sox10 down vs E11.5 * Ctrl
10552488	Klk10	NM_133712	0.00117474	-1.17803	E11.5 * Sox10 down vs E11.5 * Ctrl
10395596	Foxg1	NM_008241	0.000404243	-1.17813	E11.5 * Sox10 down vs E11.5 * Ctrl
10366725	Fam19a2	NM_182807	0.00493237	-1.17836	E11.5 * Sox10 down vs E11.5 * Ctrl
10519805	Speer4d	NM_025759	0.00977875	-1.17862	E11.5 * Sox10 down vs E11.5 * Ctrl
10603245		---	0.00469558	-1.17866	E11.5 * Sox10 down vs E11.5 * Ctrl
10414493	Olfr748	NM_001011837	0.00612876	-1.17869	E11.5 * Sox10 down vs E11.5 * Ctrl
10602592	Hsd17b10	NM_016763	0.00610182	-1.17876	E11.5 * Sox10 down vs E11.5 * Ctrl
10550365	Prkd2	NM_178900	0.00822699	-1.17887	E11.5 * Sox10 down vs E11.5 * Ctrl
10563355	Sec1	NM_019934	0.00719624	-1.17898	E11.5 * Sox10 down vs E11.5 * Ctrl
10532248	Rnf212	ENSMUST00000(	0.00661037	-1.17913	E11.5 * Sox10 down vs E11.5 * Ctrl
10394392	Nfyc	NM_008692	0.00274428	-1.17917	E11.5 * Sox10 down vs E11.5 * Ctrl
10414333	Samd4	NM_001037221	0.0075308	-1.1793	E11.5 * Sox10 down vs E11.5 * Ctrl
10576561	2310079N02Rik	NM_025636	0.00897666	-1.1796	E11.5 * Sox10 down vs E11.5 * Ctrl
10438425	Olfr167	NM_146935	0.00195472	-1.18008	E11.5 * Sox10 down vs E11.5 * Ctrl
10598038	Prf1	X60165	0.00102805	-1.18045	E11.5 * Sox10 down vs E11.5 * Ctrl
10440681	Krtap16-1	NM_130870	0.00293423	-1.18053	E11.5 * Sox10 down vs E11.5 * Ctrl
10557655	Srcap	ENSMUST00000(	0.00745392	-1.18059	E11.5 * Sox10 down vs E11.5 * Ctrl
10608089	Ube1y1	NM_011667	0.00693717	-1.18068	E11.5 * Sox10 down vs E11.5 * Ctrl
10387620	Amac1	NM_019871	0.00379395	-1.18148	E11.5 * Sox10 down vs E11.5 * Ctrl
10394448	Pum2	NM_030723	0.000979784	-1.18152	E11.5 * Sox10 down vs E11.5 * Ctrl
10526232	Wbscr27	NM_024479	0.00140021	-1.18193	E11.5 * Sox10 down vs E11.5 * Ctrl
10464917	Cnih2	NM_009920	0.00522154	-1.18194	E11.5 * Sox10 down vs E11.5 * Ctrl
10564713	Mfge8	NM_008594	0.00392564	-1.18217	E11.5 * Sox10 down vs E11.5 * Ctrl
10567229	Smg1	NM_001031814	0.000811805	-1.18231	E11.5 * Sox10 down vs E11.5 * Ctrl

10525955	Fzd10	NM_175284	0.00473929	-1.18234	E11.5 * Sox10 down vs E11.5 * Ctrl
10457223	Cdh11	NM_009866	0.000138338	-1.18262	E11.5 * Sox10 down vs E11.5 * Ctrl
10600480	4930408F14Rik	BC117726	0.00156993	-1.18277	E11.5 * Sox10 down vs E11.5 * Ctrl
10603230	4930408F14Rik	BC117726	0.00156993	-1.18277	E11.5 * Sox10 down vs E11.5 * Ctrl
10605353	4930408F14Rik	BC117726	0.00156993	-1.18277	E11.5 * Sox10 down vs E11.5 * Ctrl
10479887	Sec61a2	NM_021305	0.00542431	-1.18279	E11.5 * Sox10 down vs E11.5 * Ctrl
10443039	Bnip1	NM_172149	0.00572341	-1.18285	E11.5 * Sox10 down vs E11.5 * Ctrl
10374413		---	0.00335851	-1.18288	E11.5 * Sox10 down vs E11.5 * Ctrl
10590427	Ccbp2	NM_021609	0.00815363	-1.18303	E11.5 * Sox10 down vs E11.5 * Ctrl
10421877	Diap3	NM_019670	0.00321775	-1.18321	E11.5 * Sox10 down vs E11.5 * Ctrl
10411105	ENSMUSG0000C	ENSMUST00000	0.00858753	-1.18384	E11.5 * Sox10 down vs E11.5 * Ctrl
10436209	Cblb	NM_001033238	0.00807768	-1.18395	E11.5 * Sox10 down vs E11.5 * Ctrl
10375515	Ifi47	NM_008330	0.000161337	-1.18413	E11.5 * Sox10 down vs E11.5 * Ctrl
10449914	9030612M13Rik	NM_172458	0.00313101	-1.18424	E11.5 * Sox10 down vs E11.5 * Ctrl
10408064	Olfr11	NM_146542	0.000940113	-1.18464	E11.5 * Sox10 down vs E11.5 * Ctrl
10450872	Olfr99	NM_146515	0.0044762	-1.1848	E11.5 * Sox10 down vs E11.5 * Ctrl
10468593	Nrap	NM_008733	0.00207271	-1.18483	E11.5 * Sox10 down vs E11.5 * Ctrl
10603308		---	0.0068696	-1.18498	E11.5 * Sox10 down vs E11.5 * Ctrl
10429462	1700016M24Rik	BC111897	0.00261419	-1.18557	E11.5 * Sox10 down vs E11.5 * Ctrl
10569539	Mrgprg	NM_203492	0.00276764	-1.18583	E11.5 * Sox10 down vs E11.5 * Ctrl
10598126	Hhipl2	BC034362	0.00362603	-1.18599	E11.5 * Sox10 down vs E11.5 * Ctrl
10550715	Trappc6a	NM_025960	0.00210962	-1.18611	E11.5 * Sox10 down vs E11.5 * Ctrl
10463513	4930505N22Rik	ENSMUST00000	0.00829579	-1.18611	E11.5 * Sox10 down vs E11.5 * Ctrl
10454653		---	0.00373384	-1.18653	E11.5 * Sox10 down vs E11.5 * Ctrl
10511282	Tnfrsf4	NM_011659	0.000868882	-1.18663	E11.5 * Sox10 down vs E11.5 * Ctrl
10579538	Tmem221	NM_001100462	0.00185991	-1.18667	E11.5 * Sox10 down vs E11.5 * Ctrl
10480321	A930004D18Rik	ENSMUST00000	0.00625114	-1.18682	E11.5 * Sox10 down vs E11.5 * Ctrl
10430974	Arfgap3	NM_025445	0.00221578	-1.18691	E11.5 * Sox10 down vs E11.5 * Ctrl
10375472	Timd4	NM_178759	0.00493409	-1.18696	E11.5 * Sox10 down vs E11.5 * Ctrl
10575021	Zfp90	NM_011764	1.78941e-005	-1.18708	E11.5 * Sox10 down vs E11.5 * Ctrl
10383758	Tug1	NR_002321	0.00483767	-1.18726	E11.5 * Sox10 down vs E11.5 * Ctrl
10457385	Ccny	NM_026484	0.000637831	-1.18737	E11.5 * Sox10 down vs E11.5 * Ctrl
10351099	Tnfsf18	NM_183391	0.00520194	-1.18742	E11.5 * Sox10 down vs E11.5 * Ctrl
10394846	LOC100043371	ENSMUST00000	0.00869241	-1.18745	E11.5 * Sox10 down vs E11.5 * Ctrl
10602269		---	0.00407268	-1.18745	E11.5 * Sox10 down vs E11.5 * Ctrl
10480238	St8sia6	NM_145838	0.0062689	-1.1875	E11.5 * Sox10 down vs E11.5 * Ctrl
10352798	Kcnh1	NM_010600	0.00634978	-1.18798	E11.5 * Sox10 down vs E11.5 * Ctrl
10345141	Lmbrd1	NM_026719	0.00356708	-1.18852	E11.5 * Sox10 down vs E11.5 * Ctrl
10494411	Rnu1b1	NR_004412	0.00182111	-1.18982	E11.5 * Sox10 down vs E11.5 * Ctrl
10494417	Rnu1b1	NR_004412	0.00182111	-1.18982	E11.5 * Sox10 down vs E11.5 * Ctrl
10500356	Rnu1b1	NR_004412	0.00182111	-1.18982	E11.5 * Sox10 down vs E11.5 * Ctrl
10353630	Prf1	X60165	0.000723324	-1.18984	E11.5 * Sox10 down vs E11.5 * Ctrl
10509122	Cnr2	NM_009924	0.00653556	-1.19003	E11.5 * Sox10 down vs E11.5 * Ctrl
10348424	Sh3bp4	NM_133816	0.00471141	-1.19019	E11.5 * Sox10 down vs E11.5 * Ctrl
10489372	0610008F07Rik	BC025862	0.00697657	-1.19022	E11.5 * Sox10 down vs E11.5 * Ctrl
10537710	Tas2r144	NM_001001453	0.00551934	-1.19032	E11.5 * Sox10 down vs E11.5 * Ctrl
10399689		---	0.0070949	-1.1904	E11.5 * Sox10 down vs E11.5 * Ctrl
10372656	Cpsf6	NM_001013391	0.00431786	-1.19041	E11.5 * Sox10 down vs E11.5 * Ctrl
10476108	Ebf4	NM_001110513	0.00956221	-1.19088	E11.5 * Sox10 down vs E11.5 * Ctrl
10592772	Abcg4	NM_138955	0.00276612	-1.19094	E11.5 * Sox10 down vs E11.5 * Ctrl
10433101	Gpr84	NM_030720	0.00972937	-1.19096	E11.5 * Sox10 down vs E11.5 * Ctrl
10507872		---	0.00363619	-1.191	E11.5 * Sox10 down vs E11.5 * Ctrl
10417517	ENSMUSG0000C	ENSMUST00000	0.0102821	-1.19121	E11.5 * Sox10 down vs E11.5 * Ctrl
10529285	Nkx1-1	ENSMUST00000	0.00958503	-1.19134	E11.5 * Sox10 down vs E11.5 * Ctrl
10572466	Pde4c	NM_201607	0.00233171	-1.19149	E11.5 * Sox10 down vs E11.5 * Ctrl
10473356	Ube2l6	NM_019949	0.00908149	-1.19155	E11.5 * Sox10 down vs E11.5 * Ctrl
10565591	OTTMUSG00000	ENSMUST00000	0.000967518	-1.19175	E11.5 * Sox10 down vs E11.5 * Ctrl
10598059	ND4L	ENSMUST00000	0.00113706	-1.19202	E11.5 * Sox10 down vs E11.5 * Ctrl
10499552	Efna4	NM_007910	0.00564797	-1.19228	E11.5 * Sox10 down vs E11.5 * Ctrl
10562500	Dpy19l3	NM_178704	0.000107047	-1.19244	E11.5 * Sox10 down vs E11.5 * Ctrl
10383511	Tex19.1	AF285590	0.00440422	-1.19285	E11.5 * Sox10 down vs E11.5 * Ctrl
10405745		---	0.00460917	-1.19324	E11.5 * Sox10 down vs E11.5 * Ctrl
10509178	Asap3	NM_001008232	0.00796489	-1.19353	E11.5 * Sox10 down vs E11.5 * Ctrl
10548000	Ltbr	NM_010736	0.00117972	-1.19421	E11.5 * Sox10 down vs E11.5 * Ctrl
10538526	Avl9	NM_030235	0.00560373	-1.19451	E11.5 * Sox10 down vs E11.5 * Ctrl
10429843	Plec1	BC024074	0.00094866	-1.19468	E11.5 * Sox10 down vs E11.5 * Ctrl

10529613	Evc	NM_021292	0.000118701	-1.19474	E11.5 * Sox10 down vs E11.5 * Ctrl
10576027	ENSMUSG00000	ENSMUST00000	0.000873772	-1.1949	E11.5 * Sox10 down vs E11.5 * Ctrl
10553403	Htatip2	NM_001146049	0.00929588	-1.19504	E11.5 * Sox10 down vs E11.5 * Ctrl
10584819		---	0.00312634	-1.19535	E11.5 * Sox10 down vs E11.5 * Ctrl
10445781	Trem2	NM_031254	0.00209687	-1.19551	E11.5 * Sox10 down vs E11.5 * Ctrl
10386402	Zkscan17	NM_172941	0.00155457	-1.19574	E11.5 * Sox10 down vs E11.5 * Ctrl
10569100		---	0.00323631	-1.19631	E11.5 * Sox10 down vs E11.5 * Ctrl
10504865	Invs	NM_010569	0.00687179	-1.19641	E11.5 * Sox10 down vs E11.5 * Ctrl
10480288	Nebl	BC119802	0.00509434	-1.19662	E11.5 * Sox10 down vs E11.5 * Ctrl
10414245	4930503E14Rik	NM_029131	0.0104066	-1.19679	E11.5 * Sox10 down vs E11.5 * Ctrl
10436087	Retnlb	NM_023881	0.00196723	-1.19687	E11.5 * Sox10 down vs E11.5 * Ctrl
10409014		---	0.01034	-1.19697	E11.5 * Sox10 down vs E11.5 * Ctrl
10410576	Cep72	NM_028959	0.00832982	-1.19702	E11.5 * Sox10 down vs E11.5 * Ctrl
10498477	E130311K13Rik	NM_177856	0.00491763	-1.19752	E11.5 * Sox10 down vs E11.5 * Ctrl
10416355	Rcbtb2	NM_134083	0.0093367	-1.19781	E11.5 * Sox10 down vs E11.5 * Ctrl
10398224	Cyp46a1	NM_010010	0.00566851	-1.19789	E11.5 * Sox10 down vs E11.5 * Ctrl
10381502	4930417O22Rik	ENSMUST00000	0.00689394	-1.1979	E11.5 * Sox10 down vs E11.5 * Ctrl
10463082	Cc2d2b	XM_001000795	0.002433	-1.19798	E11.5 * Sox10 down vs E11.5 * Ctrl
10601235	Ogt	NM_139144	0.000110759	-1.19803	E11.5 * Sox10 down vs E11.5 * Ctrl
10597933		---	0.000166207	-1.19879	E11.5 * Sox10 down vs E11.5 * Ctrl
10378126	Ankfy1	NM_009671	0.0017063	-1.19903	E11.5 * Sox10 down vs E11.5 * Ctrl
10389207	Ccl5	NM_013653	0.00196029	-1.19915	E11.5 * Sox10 down vs E11.5 * Ctrl
10592217	Stt3a	NM_008408	0.00378004	-1.19938	E11.5 * Sox10 down vs E11.5 * Ctrl
10376394	Olfr324	NM_001011743	0.00980408	-1.19968	E11.5 * Sox10 down vs E11.5 * Ctrl
10443946	1700029I08Rik	NM_183282	0.00601055	-1.20049	E11.5 * Sox10 down vs E11.5 * Ctrl
10466216	Ms4a2	NM_013516	0.00312025	-1.20063	E11.5 * Sox10 down vs E11.5 * Ctrl
10450529	Dpcr1	NM_001033366	0.00893561	-1.20086	E11.5 * Sox10 down vs E11.5 * Ctrl
10484801	Olfr1226	NM_146967	0.00394087	-1.20092	E11.5 * Sox10 down vs E11.5 * Ctrl
10518967	1190007F08Rik	ENSMUST00000	0.000813804	-1.20099	E11.5 * Sox10 down vs E11.5 * Ctrl
10493816	S100a5	NM_011312	0.00221038	-1.20101	E11.5 * Sox10 down vs E11.5 * Ctrl
10420614	Sacs	NM_172809	0.00388106	-1.20124	E11.5 * Sox10 down vs E11.5 * Ctrl
10376144	Fnip1	NM_173753	0.00846134	-1.20144	E11.5 * Sox10 down vs E11.5 * Ctrl
10346168	Stat4	NM_011487	0.00880802	-1.20146	E11.5 * Sox10 down vs E11.5 * Ctrl
10528191	Speer4d	NM_025759	0.00123759	-1.20172	E11.5 * Sox10 down vs E11.5 * Ctrl
10473217		---	0.0019497	-1.20174	E11.5 * Sox10 down vs E11.5 * Ctrl
10582458	Ankrd11	NM_001081379	0.00530149	-1.20175	E11.5 * Sox10 down vs E11.5 * Ctrl
10519578	Abcb4	NM_008830	0.00913696	-1.20198	E11.5 * Sox10 down vs E11.5 * Ctrl
10591884	Glb1l2	NM_153803	0.00504085	-1.20237	E11.5 * Sox10 down vs E11.5 * Ctrl
10423731		---	0.000746752	-1.20253	E11.5 * Sox10 down vs E11.5 * Ctrl
10388042	6330403K07Rik	NM_134022	0.00127294	-1.20272	E11.5 * Sox10 down vs E11.5 * Ctrl
10588154	Stag1	NM_009282	0.0047293	-1.203	E11.5 * Sox10 down vs E11.5 * Ctrl
10520833	Mrpl33	NM_025796	0.00692897	-1.20367	E11.5 * Sox10 down vs E11.5 * Ctrl
10507870		---	0.00379496	-1.20382	E11.5 * Sox10 down vs E11.5 * Ctrl
10379721	Ccl4	NM_013652	0.000980024	-1.20446	E11.5 * Sox10 down vs E11.5 * Ctrl
10417124	B930095G15Rik	BC096543	0.000917069	-1.20451	E11.5 * Sox10 down vs E11.5 * Ctrl
10399314	Gm1964	NM_001033488	0.00502859	-1.20468	E11.5 * Sox10 down vs E11.5 * Ctrl
10578649	Odz3	NM_011857	0.00864462	-1.20545	E11.5 * Sox10 down vs E11.5 * Ctrl
10373846	Sec14l3	NM_001029937	0.00910867	-1.20551	E11.5 * Sox10 down vs E11.5 * Ctrl
10363379	Cbara1	NM_144822	0.00831066	-1.20567	E11.5 * Sox10 down vs E11.5 * Ctrl
10461160		---	0.00787559	-1.20569	E11.5 * Sox10 down vs E11.5 * Ctrl
10375751	Adamts2	NM_175643	0.00471525	-1.20573	E11.5 * Sox10 down vs E11.5 * Ctrl
10391742	Gpatch8	NM_001159492	0.00710253	-1.20586	E11.5 * Sox10 down vs E11.5 * Ctrl
10601854	Wbp5	NM_011712	0.00032741	-1.20594	E11.5 * Sox10 down vs E11.5 * Ctrl
10438572	2510009E07Rik	NM_001001881	0.000645369	-1.20602	E11.5 * Sox10 down vs E11.5 * Ctrl
10536898	Irf5	NM_012057	0.00194994	-1.20603	E11.5 * Sox10 down vs E11.5 * Ctrl
10412719	Synpr	NM_028052	0.00359978	-1.20635	E11.5 * Sox10 down vs E11.5 * Ctrl
10505064	Tmem38b	NM_028053	0.00465901	-1.20663	E11.5 * Sox10 down vs E11.5 * Ctrl
10483633	Sp3	NM_001018042	0.00998367	-1.20682	E11.5 * Sox10 down vs E11.5 * Ctrl
10534988		---	0.0011669	-1.20721	E11.5 * Sox10 down vs E11.5 * Ctrl
10576034	Irf8	NM_008320	0.000964443	-1.20751	E11.5 * Sox10 down vs E11.5 * Ctrl
10538438	C330043M08Rik	NM_001142781	0.00110483	-1.20757	E11.5 * Sox10 down vs E11.5 * Ctrl
10516784		---	0.00163913	-1.2077	E11.5 * Sox10 down vs E11.5 * Ctrl
10578262		---	4.69672e-005	-1.20775	E11.5 * Sox10 down vs E11.5 * Ctrl
10531342	U90926	U90926	0.000476244	-1.20779	E11.5 * Sox10 down vs E11.5 * Ctrl
10411804	Mast4	ENSMUST00000	0.000790096	-1.20796	E11.5 * Sox10 down vs E11.5 * Ctrl
10544798	Hoxa10	NM_008263	0.00631698	-1.208	E11.5 * Sox10 down vs E11.5 * Ctrl

10397359	Batf	NM_016767	0.000595339	-1.20815	E11.5 * Sox10 down vs E11.5 * Ctrl
10396383	Slc38a6	BC157917	0.00307768	-1.20818	E11.5 * Sox10 down vs E11.5 * Ctrl
10454912	Ankhd1	NM_175375	0.00235942	-1.2082	E11.5 * Sox10 down vs E11.5 * Ctrl
10396956	Pcnx	NM_018814	0.00100983	-1.20856	E11.5 * Sox10 down vs E11.5 * Ctrl
10426261	Shank3	NM_021423	0.00135607	-1.20919	E11.5 * Sox10 down vs E11.5 * Ctrl
10446656	Lpin2	NM_022882	0.00548243	-1.20933	E11.5 * Sox10 down vs E11.5 * Ctrl
10506110	L1td1	NM_001081202	0.00649907	-1.20933	E11.5 * Sox10 down vs E11.5 * Ctrl
10528236		---	0.00145248	-1.20939	E11.5 * Sox10 down vs E11.5 * Ctrl
10357932	Ppp1r12b	NM_001081307	0.000176101	-1.20959	E11.5 * Sox10 down vs E11.5 * Ctrl
10358299	EG214403	NM_001029977	0.00299648	-1.21	E11.5 * Sox10 down vs E11.5 * Ctrl
10477286	Pofut1	NM_080463	0.00137379	-1.21052	E11.5 * Sox10 down vs E11.5 * Ctrl
10396278	Daam1	NM_026102	0.00250219	-1.2106	E11.5 * Sox10 down vs E11.5 * Ctrl
10605542	2410003J06Rik	NM_001113734	0.000728095	-1.21062	E11.5 * Sox10 down vs E11.5 * Ctrl
10418702	Sh3bp5	NM_011894	0.000197352	-1.21068	E11.5 * Sox10 down vs E11.5 * Ctrl
10377859	Pld2	NM_008876	0.00258518	-1.21091	E11.5 * Sox10 down vs E11.5 * Ctrl
10350046	Kdm5b	NM_152895	4.75103e-005	-1.21111	E11.5 * Sox10 down vs E11.5 * Ctrl
10447773	Slc22a3	NM_011395	0.00647347	-1.21165	E11.5 * Sox10 down vs E11.5 * Ctrl
10519879	Speer4f	NM_027609	0.00644468	-1.21168	E11.5 * Sox10 down vs E11.5 * Ctrl
10522925	Prol1	NM_008644	0.00948057	-1.21176	E11.5 * Sox10 down vs E11.5 * Ctrl
10512487	Rmrp	NR_001460	0.0102484	-1.21205	E11.5 * Sox10 down vs E11.5 * Ctrl
10491056	Tbl1xr1	NM_030732	0.00395268	-1.21206	E11.5 * Sox10 down vs E11.5 * Ctrl
10500103	Gabpb2	NM_029885	9.01492e-005	-1.21206	E11.5 * Sox10 down vs E11.5 * Ctrl
10399189	Sp8	NM_177082	0.000190662	-1.21215	E11.5 * Sox10 down vs E11.5 * Ctrl
10554063	Adamts17	NM_001033877	0.00727204	-1.21215	E11.5 * Sox10 down vs E11.5 * Ctrl
10547795	Atn1	NM_007881	0.00801605	-1.21233	E11.5 * Sox10 down vs E11.5 * Ctrl
10369748	EG631906	ENSMUST00000(	0.000362183	-1.2124	E11.5 * Sox10 down vs E11.5 * Ctrl
10582337	Fam38a	ENSMUST00000(	0.00293459	-1.21265	E11.5 * Sox10 down vs E11.5 * Ctrl
10563657		---	0.00623812	-1.21314	E11.5 * Sox10 down vs E11.5 * Ctrl
10441344	Rbm16	BC075621	0.00409	-1.21331	E11.5 * Sox10 down vs E11.5 * Ctrl
10595871	Clstn2	NM_022319	0.000209172	-1.2135	E11.5 * Sox10 down vs E11.5 * Ctrl
10538547	Fkbp9	NM_012056	0.00466956	-1.21353	E11.5 * Sox10 down vs E11.5 * Ctrl
10497222	Zfp704	NM_133218	0.00478668	-1.21358	E11.5 * Sox10 down vs E11.5 * Ctrl
10355329	Bard1	NM_007525	0.00519773	-1.21363	E11.5 * Sox10 down vs E11.5 * Ctrl
10402063	Foxn3	BC029185	0.000772029	-1.21365	E11.5 * Sox10 down vs E11.5 * Ctrl
10346799	Icos	NM_017480	0.00387598	-1.21412	E11.5 * Sox10 down vs E11.5 * Ctrl
10413161	Cnot1	NM_153164	0.000318043	-1.21438	E11.5 * Sox10 down vs E11.5 * Ctrl
10499766	Slc27a3	NM_011988	0.00501241	-1.21441	E11.5 * Sox10 down vs E11.5 * Ctrl
10362245	Epb4.1l2	NM_013511	3.51416e-005	-1.21457	E11.5 * Sox10 down vs E11.5 * Ctrl
10546272	Iqsec1	NM_001134384	0.00190762	-1.21457	E11.5 * Sox10 down vs E11.5 * Ctrl
10396511	Syne2	NM_001005510	0.00189428	-1.21462	E11.5 * Sox10 down vs E11.5 * Ctrl
10513162	Ptpn3	NM_011207	0.00920362	-1.21524	E11.5 * Sox10 down vs E11.5 * Ctrl
10405743		---	0.00227531	-1.21524	E11.5 * Sox10 down vs E11.5 * Ctrl
10465244	Malat1	NR_002847	0.00295839	-1.21526	E11.5 * Sox10 down vs E11.5 * Ctrl
10344821	Cspp1	NM_026493	0.00636613	-1.21528	E11.5 * Sox10 down vs E11.5 * Ctrl
10368473		---	0.000421087	-1.21569	E11.5 * Sox10 down vs E11.5 * Ctrl
10440727	Krtap8-1	ENSMUST00000(	0.00673759	-1.21605	E11.5 * Sox10 down vs E11.5 * Ctrl
10605651	4930415L06Rik	ENSMUST00000(	0.0016746	-1.21619	E11.5 * Sox10 down vs E11.5 * Ctrl
10391685	Fam171a2	NM_199200	0.00166865	-1.2162	E11.5 * Sox10 down vs E11.5 * Ctrl
10552475	Klk12	NM_027097	0.00415646	-1.21623	E11.5 * Sox10 down vs E11.5 * Ctrl
10603860	Cfp	NM_008823	0.0103236	-1.21624	E11.5 * Sox10 down vs E11.5 * Ctrl
10414154		---	0.00342487	-1.21635	E11.5 * Sox10 down vs E11.5 * Ctrl
10601503	Dach2	NM_033605	0.00345564	-1.21651	E11.5 * Sox10 down vs E11.5 * Ctrl
10409924	Cts8	NM_019541	0.00280842	-1.21658	E11.5 * Sox10 down vs E11.5 * Ctrl
10445688	Ccnd3	NM_001081636	0.00366249	-1.21664	E11.5 * Sox10 down vs E11.5 * Ctrl
10470584	Tsc1	NM_022887	0.000749051	-1.21668	E11.5 * Sox10 down vs E11.5 * Ctrl
10435237	Zfp148	NM_011749	0.000449059	-1.21687	E11.5 * Sox10 down vs E11.5 * Ctrl
10408490	Exoc2	NM_025588	5.82334e-005	-1.21692	E11.5 * Sox10 down vs E11.5 * Ctrl
10382848	1110005A03Rik	BC026936	0.00107488	-1.21725	E11.5 * Sox10 down vs E11.5 * Ctrl
10494413	Rnu1b1	NR_004412	0.00019262	-1.21735	E11.5 * Sox10 down vs E11.5 * Ctrl
10494421	Rnu1b1	NR_004412	0.00019262	-1.21735	E11.5 * Sox10 down vs E11.5 * Ctrl
10500343	Rnu1b1	NR_004412	0.00019262	-1.21735	E11.5 * Sox10 down vs E11.5 * Ctrl
10500358	Rnu1b1	NR_004412	0.00019262	-1.21735	E11.5 * Sox10 down vs E11.5 * Ctrl
10512937	Rnu1b1	NR_004412	0.00019262	-1.21735	E11.5 * Sox10 down vs E11.5 * Ctrl
10411464	Fcho2	NM_172591	0.00623687	-1.21775	E11.5 * Sox10 down vs E11.5 * Ctrl
10607059	Irs4	NM_010572	0.00198181	-1.21781	E11.5 * Sox10 down vs E11.5 * Ctrl
10374771		---	0.0100241	-1.21795	E11.5 * Sox10 down vs E11.5 * Ctrl

10490852		---	0.00566301	-1.21797	E11.5 * Sox10 down vs E11.5 * Ctrl
10464171	Tdrd1	NM_001002238	0.000473349	-1.21806	E11.5 * Sox10 down vs E11.5 * Ctrl
10372342	Nav3	NM_001081035	0.00519685	-1.21849	E11.5 * Sox10 down vs E11.5 * Ctrl
10539692	Tgfa	NM_031199	0.00689845	-1.21857	E11.5 * Sox10 down vs E11.5 * Ctrl
10536170	Pigt	AB057593	0.00161254	-1.21858	E11.5 * Sox10 down vs E11.5 * Ctrl
10545208	Gm189	ENSMUST00000	0.00221024	-1.21934	E11.5 * Sox10 down vs E11.5 * Ctrl
10428302	Klf10	NM_013692	0.00258375	-1.21937	E11.5 * Sox10 down vs E11.5 * Ctrl
10544219	Braf	NM_139294	0.00802063	-1.21968	E11.5 * Sox10 down vs E11.5 * Ctrl
10471819	Crb2	ENSMUST00000(	0.00892746	-1.21968	E11.5 * Sox10 down vs E11.5 * Ctrl
10389738	Dgke	NM_019505	0.006098	-1.21989	E11.5 * Sox10 down vs E11.5 * Ctrl
10501879	Usp53	NM_133857	0.00997469	-1.22008	E11.5 * Sox10 down vs E11.5 * Ctrl
10459510		---	0.00686085	-1.2203	E11.5 * Sox10 down vs E11.5 * Ctrl
10587829	Plod2	NM_001142916	0.00171007	-1.22035	E11.5 * Sox10 down vs E11.5 * Ctrl
10571321	Ppp1r3b	NM_177741	0.00336725	-1.22035	E11.5 * Sox10 down vs E11.5 * Ctrl
10481304	Gfi1b	NM_008114	0.00887757	-1.22039	E11.5 * Sox10 down vs E11.5 * Ctrl
10555186	Wnt11	NM_009519	0.00209813	-1.22044	E11.5 * Sox10 down vs E11.5 * Ctrl
10415742	Mipep	NM_027436	0.00661021	-1.22047	E11.5 * Sox10 down vs E11.5 * Ctrl
10384782	Vrk2	NM_027260	0.00486696	-1.22069	E11.5 * Sox10 down vs E11.5 * Ctrl
10405741	100041437	XR_030564	0.0014448	-1.22078	E11.5 * Sox10 down vs E11.5 * Ctrl
10581214	Lrrc29	NM_177449	0.0104208	-1.22081	E11.5 * Sox10 down vs E11.5 * Ctrl
10424381		---	0.000843664	-1.22142	E11.5 * Sox10 down vs E11.5 * Ctrl
10577337	Defb40	NM_183039	0.00858127	-1.22148	E11.5 * Sox10 down vs E11.5 * Ctrl
10360858	C130074G19Rik	NM_178692	0.00839423	-1.22148	E11.5 * Sox10 down vs E11.5 * Ctrl
10485372	Rag1	NM_009019	0.00929973	-1.22153	E11.5 * Sox10 down vs E11.5 * Ctrl
10489241	Gm826	NM_001033411	0.00345072	-1.22155	E11.5 * Sox10 down vs E11.5 * Ctrl
10602772	Rps6ka3	NM_148945	0.00661141	-1.22161	E11.5 * Sox10 down vs E11.5 * Ctrl
10351603	Arhgap30	NM_001005508	0.00736591	-1.22178	E11.5 * Sox10 down vs E11.5 * Ctrl
10487238	Hdc	NM_008230	0.00342784	-1.22186	E11.5 * Sox10 down vs E11.5 * Ctrl
10450374	D17H6S56E-5	L78788	0.00596741	-1.22194	E11.5 * Sox10 down vs E11.5 * Ctrl
10351500		---	0.00364266	-1.22199	E11.5 * Sox10 down vs E11.5 * Ctrl
10516590	Zbtb8a	NM_028603	0.00872169	-1.22201	E11.5 * Sox10 down vs E11.5 * Ctrl
10427461	Ptger4	NM_001136079	0.00605551	-1.22203	E11.5 * Sox10 down vs E11.5 * Ctrl
10400052	4930579E17Rik	NM_178629	0.00433422	-1.2221	E11.5 * Sox10 down vs E11.5 * Ctrl
10372385	Zdhhc17	NM_172554	0.00327643	-1.22213	E11.5 * Sox10 down vs E11.5 * Ctrl
10519060	Tnfrsf14	NM_178931	0.00210202	-1.22217	E11.5 * Sox10 down vs E11.5 * Ctrl
10542857	Far2	NM_178797	0.00212467	-1.22218	E11.5 * Sox10 down vs E11.5 * Ctrl
10542691	Lrmp	NM_008511	0.00807896	-1.22228	E11.5 * Sox10 down vs E11.5 * Ctrl
10434283	LOC635992	ENSMUST00000(	0.000663396	-1.22234	E11.5 * Sox10 down vs E11.5 * Ctrl
10500100	Tnfaip8l2	NM_027206	0.00620171	-1.22245	E11.5 * Sox10 down vs E11.5 * Ctrl
10577412	6820431F20Rik	BC025151	0.000124227	-1.22259	E11.5 * Sox10 down vs E11.5 * Ctrl
10439667	BC016579	NM_145389	0.00118421	-1.22265	E11.5 * Sox10 down vs E11.5 * Ctrl
10391378	Ezh1	NM_007970	2.77834e-005	-1.2227	E11.5 * Sox10 down vs E11.5 * Ctrl
10351515	Rnu1b1	NR_004412	0.000476165	-1.22283	E11.5 * Sox10 down vs E11.5 * Ctrl
10554650	1700010L04Rik	ENSMUST00000(	0.00654321	-1.22292	E11.5 * Sox10 down vs E11.5 * Ctrl
10417912	Usp54	NM_030180	0.00277443	-1.22293	E11.5 * Sox10 down vs E11.5 * Ctrl
10406086	Tert	NM_009354	0.00510854	-1.22298	E11.5 * Sox10 down vs E11.5 * Ctrl
10460237	Unc93b1	NM_019449	0.00408539	-1.22303	E11.5 * Sox10 down vs E11.5 * Ctrl
10549473	Caprin2	NM_181541	0.000859206	-1.22331	E11.5 * Sox10 down vs E11.5 * Ctrl
10374621	Ahsa2	NM_172391	8.18813e-005	-1.2234	E11.5 * Sox10 down vs E11.5 * Ctrl
10559420	Tmc4	NM_181820	0.000687763	-1.22403	E11.5 * Sox10 down vs E11.5 * Ctrl
10497613	ENSMUSG0000C	ENSMUST00000(	0.000947324	-1.22453	E11.5 * Sox10 down vs E11.5 * Ctrl
10544497	Tarbp2	NM_009319	0.00320378	-1.22533	E11.5 * Sox10 down vs E11.5 * Ctrl
10425430	Tnrc6b	NM_144812	0.00388281	-1.2254	E11.5 * Sox10 down vs E11.5 * Ctrl
10549733	A430110N23Rik	NM_173008	0.00999421	-1.22564	E11.5 * Sox10 down vs E11.5 * Ctrl
10440463	LOC436427	XR_034439	0.000711829	-1.22632	E11.5 * Sox10 down vs E11.5 * Ctrl
10587686		---	0.00240043	-1.22643	E11.5 * Sox10 down vs E11.5 * Ctrl
10595628		---	0.00240043	-1.22643	E11.5 * Sox10 down vs E11.5 * Ctrl
10359734	Iqwd1	ENSMUST00000(	0.00232601	-1.22668	E11.5 * Sox10 down vs E11.5 * Ctrl
10345037	Paqr8	NM_028829	7.2486e-006	-1.22676	E11.5 * Sox10 down vs E11.5 * Ctrl
10537676	1700034O15Rik	BC048592	0.00280804	-1.2268	E11.5 * Sox10 down vs E11.5 * Ctrl
10597554	Zcwpw2	BC104378	0.0029893	-1.22701	E11.5 * Sox10 down vs E11.5 * Ctrl
10373502	Ikzf4	NM_011772	0.00197765	-1.22708	E11.5 * Sox10 down vs E11.5 * Ctrl
10550605	Eml2	BC055476	0.00116594	-1.22719	E11.5 * Sox10 down vs E11.5 * Ctrl
10586880	Suhw4	NM_146224	0.00579432	-1.2272	E11.5 * Sox10 down vs E11.5 * Ctrl
10373340	Rbms2	NM_001039080	0.00725895	-1.22722	E11.5 * Sox10 down vs E11.5 * Ctrl
10540897	Pparg	NM_001127330	0.0090824	-1.22726	E11.5 * Sox10 down vs E11.5 * Ctrl

10547993		---	0.00599842	-1.22793	E11.5 * Sox10 down vs E11.5 * Ctrl
10516765	Serinc2	NM_172702	0.00367621	-1.22798	E11.5 * Sox10 down vs E11.5 * Ctrl
10468307	EG629389	XM_894271	0.00958296	-1.22813	E11.5 * Sox10 down vs E11.5 * Ctrl
10404287	Prl3c1	NM_013766	0.00290942	-1.22829	E11.5 * Sox10 down vs E11.5 * Ctrl
10366004	Atp2b1	NM_026482	2.78769e-007	-1.22839	E11.5 * Sox10 down vs E11.5 * Ctrl
10531794	Wdfy3	AB093277	0.00494497	-1.22856	E11.5 * Sox10 down vs E11.5 * Ctrl
10545588	Hk2	NM_013820	0.00297114	-1.22869	E11.5 * Sox10 down vs E11.5 * Ctrl
10586306	Igdcc4	NM_020043	0.00474103	-1.22908	E11.5 * Sox10 down vs E11.5 * Ctrl
10579089	D330038O06Rik	NM_177899	0.00231644	-1.22911	E11.5 * Sox10 down vs E11.5 * Ctrl
10588701	Hyal3	NM_178020	0.00310938	-1.22937	E11.5 * Sox10 down vs E11.5 * Ctrl
10353624	ND4L	ENSMUST00000(	0.00154329	-1.22953	E11.5 * Sox10 down vs E11.5 * Ctrl
10542140	Klrb1f	NM_153094	0.0046403	-1.22953	E11.5 * Sox10 down vs E11.5 * Ctrl
10513160	Ptpn3	NM_011207	0.00812258	-1.22959	E11.5 * Sox10 down vs E11.5 * Ctrl
10522712	Rest	NM_011263	0.00798164	-1.23047	E11.5 * Sox10 down vs E11.5 * Ctrl
10563659	Spty2d1	NM_175318	0.00525776	-1.23055	E11.5 * Sox10 down vs E11.5 * Ctrl
10378240	P2rx1	NM_008771	0.000651078	-1.23068	E11.5 * Sox10 down vs E11.5 * Ctrl
10503615		---	0.00372189	-1.23089	E11.5 * Sox10 down vs E11.5 * Ctrl
10474860	Chst14	NM_028117	0.00200525	-1.23119	E11.5 * Sox10 down vs E11.5 * Ctrl
10497399	Pde7a	NM_001122759	0.00060181	-1.23119	E11.5 * Sox10 down vs E11.5 * Ctrl
10371356	Appl2	NM_145220	0.00162379	-1.23163	E11.5 * Sox10 down vs E11.5 * Ctrl
10445078	Gabbr1	NM_019439	0.00514429	-1.23175	E11.5 * Sox10 down vs E11.5 * Ctrl
10487321	2010106G01Rik	NM_023220	0.00139042	-1.23188	E11.5 * Sox10 down vs E11.5 * Ctrl
10432939	Csad	NM_144942	0.00504887	-1.23216	E11.5 * Sox10 down vs E11.5 * Ctrl
10591988	Adamts15	NM_001024139	0.00938062	-1.23246	E11.5 * Sox10 down vs E11.5 * Ctrl
10518346	OTTMUSG00000	NM_001085522	0.0103808	-1.23314	E11.5 * Sox10 down vs E11.5 * Ctrl
10588942	Lamb2	NM_008483	0.00635793	-1.23314	E11.5 * Sox10 down vs E11.5 * Ctrl
10573419	Lyl1	NM_008535	0.00111408	-1.23317	E11.5 * Sox10 down vs E11.5 * Ctrl
10591660	Epor	NM_010149	7.33203e-005	-1.23352	E11.5 * Sox10 down vs E11.5 * Ctrl
10345752	Il1r2	NM_010555	0.00454338	-1.23375	E11.5 * Sox10 down vs E11.5 * Ctrl
10558295	Zranb1	ENSMUST00000	0.00788422	-1.23395	E11.5 * Sox10 down vs E11.5 * Ctrl
10574727	Slc9a5	NM_001081332	0.000523582	-1.23397	E11.5 * Sox10 down vs E11.5 * Ctrl
10526459	Rasa4	NM_133914	0.00643507	-1.23416	E11.5 * Sox10 down vs E11.5 * Ctrl
10566822	Scube2	NM_020052	0.00063712	-1.23418	E11.5 * Sox10 down vs E11.5 * Ctrl
10433480	Rpl39l	NM_026594	0.000660175	-1.23421	E11.5 * Sox10 down vs E11.5 * Ctrl
10437928	0610037P05Rik	BC011300	0.000413466	-1.23438	E11.5 * Sox10 down vs E11.5 * Ctrl
10455738	Snx2	NM_026386	0.000132859	-1.23442	E11.5 * Sox10 down vs E11.5 * Ctrl
10550263	Slc8a2	NM_148946	0.00296394	-1.23452	E11.5 * Sox10 down vs E11.5 * Ctrl
10403750	2810021B07Rik	NM_025479	0.00296283	-1.23554	E11.5 * Sox10 down vs E11.5 * Ctrl
10569313	6330512M04Rik	NM_177265	0.0041418	-1.23561	E11.5 * Sox10 down vs E11.5 * Ctrl
10510178	OTTMUSG00000	NM_001037926	0.00320452	-1.23563	E11.5 * Sox10 down vs E11.5 * Ctrl
10535091	4930432F04Rik	BC016220	0.000640371	-1.23563	E11.5 * Sox10 down vs E11.5 * Ctrl
10461745		---	0.00732576	-1.23588	E11.5 * Sox10 down vs E11.5 * Ctrl
10593430	Sik2	NM_178710	0.00133932	-1.23599	E11.5 * Sox10 down vs E11.5 * Ctrl
10543333	Aass	NM_013930	0.000722791	-1.23602	E11.5 * Sox10 down vs E11.5 * Ctrl
10421581	Lrch1	NM_001033439	0.000361712	-1.23602	E11.5 * Sox10 down vs E11.5 * Ctrl
10493292	2810403A07Rik	AK173057	0.000132444	-1.23607	E11.5 * Sox10 down vs E11.5 * Ctrl
10476353		---	0.00592168	-1.23612	E11.5 * Sox10 down vs E11.5 * Ctrl
10585543	EG665005	XR_030731	0.00697652	-1.2362	E11.5 * Sox10 down vs E11.5 * Ctrl
10456812	Hdhd2	ENSMUST00000(	0.00925293	-1.23633	E11.5 * Sox10 down vs E11.5 * Ctrl
10477311	Asxl1	NM_001039939	0.000680586	-1.23639	E11.5 * Sox10 down vs E11.5 * Ctrl
10497622	Lrrc34	NM_027941	0.000290689	-1.23664	E11.5 * Sox10 down vs E11.5 * Ctrl
10407173	Il6st	NM_010560	0.000324901	-1.23674	E11.5 * Sox10 down vs E11.5 * Ctrl
10367024	Tac2	NM_009312	0.00628947	-1.237	E11.5 * Sox10 down vs E11.5 * Ctrl
10446473	Lama1	NM_008480	0.000851694	-1.23708	E11.5 * Sox10 down vs E11.5 * Ctrl
10508249	OTTMUSG00000	NM_001099319	0.00573839	-1.23716	E11.5 * Sox10 down vs E11.5 * Ctrl
10544756	Hoxa3	NM_010452	6.03805e-005	-1.23722	E11.5 * Sox10 down vs E11.5 * Ctrl
10356423	Usp40	NM_001033291	0.00213364	-1.23755	E11.5 * Sox10 down vs E11.5 * Ctrl
10594404	Smad3	NM_016769	0.00385179	-1.2376	E11.5 * Sox10 down vs E11.5 * Ctrl
10411229	F2r	NM_010169	0.000223671	-1.23768	E11.5 * Sox10 down vs E11.5 * Ctrl
10464328	Pnliprp2	NM_011128	0.00281488	-1.23795	E11.5 * Sox10 down vs E11.5 * Ctrl
10531348	Ppef2	NM_011148	2.84606e-005	-1.23813	E11.5 * Sox10 down vs E11.5 * Ctrl
10448878	Baiap3	BC158116	0.000316537	-1.23815	E11.5 * Sox10 down vs E11.5 * Ctrl
10513592	Wdr31	NM_023597	0.00973515	-1.23824	E11.5 * Sox10 down vs E11.5 * Ctrl
10415248	Pck2	NM_028994	0.00048475	-1.23905	E11.5 * Sox10 down vs E11.5 * Ctrl
10551696	Rasgrp4	NM_145149	0.000366634	-1.23925	E11.5 * Sox10 down vs E11.5 * Ctrl
10580782	Dok4	NM_053246	0.000298914	-1.23929	E11.5 * Sox10 down vs E11.5 * Ctrl

10382425	Gprc5c	NM_001110337	0.000985972	-1.23935	E11.5 * Sox10 down vs E11.5 * Ctrl
10600953	Eda	NM_010099	0.00996844	-1.23949	E11.5 * Sox10 down vs E11.5 * Ctrl
10447294	Prkce	NM_011104	0.00247267	-1.23949	E11.5 * Sox10 down vs E11.5 * Ctrl
10555389	Ucp2	NM_011671	0.00815104	-1.23965	E11.5 * Sox10 down vs E11.5 * Ctrl
10437210	Bace2	NM_019517	0.00117853	-1.23968	E11.5 * Sox10 down vs E11.5 * Ctrl
10559837	Vmn2r29	NR_003555	0.00412152	-1.23978	E11.5 * Sox10 down vs E11.5 * Ctrl
10500534	Hsd3b4	NM_001111336	0.00303456	-1.23983	E11.5 * Sox10 down vs E11.5 * Ctrl
10500539	Hsd3b4	NM_001111336	0.00303456	-1.23983	E11.5 * Sox10 down vs E11.5 * Ctrl
10499811	Npr1	NM_008727	0.00379637	-1.24018	E11.5 * Sox10 down vs E11.5 * Ctrl
10439854	Bbx	NM_027444	0.0032756	-1.24068	E11.5 * Sox10 down vs E11.5 * Ctrl
10504132	Ccl19	NM_011888	0.00391273	-1.24079	E11.5 * Sox10 down vs E11.5 * Ctrl
10590801	Birc3	NM_007464	0.00286756	-1.24095	E11.5 * Sox10 down vs E11.5 * Ctrl
10587792	Plscr1	NM_011636	0.00688422	-1.24095	E11.5 * Sox10 down vs E11.5 * Ctrl
10353844	Neurl3	NM_153408	0.00257093	-1.24096	E11.5 * Sox10 down vs E11.5 * Ctrl
10503584	Coq3	NM_172687	0.00363619	-1.24113	E11.5 * Sox10 down vs E11.5 * Ctrl
10481349	Ntng2	NM_133501	6.50263e-005	-1.24113	E11.5 * Sox10 down vs E11.5 * Ctrl
10569702	Cacng8	NM_133190	0.00711209	-1.24129	E11.5 * Sox10 down vs E11.5 * Ctrl
10398344	Rtl1	NM_184109	0.00449047	-1.24153	E11.5 * Sox10 down vs E11.5 * Ctrl
10524844	Taok3	NM_001081308	0.00123394	-1.24181	E11.5 * Sox10 down vs E11.5 * Ctrl
10596255		---	0.00535329	-1.24199	E11.5 * Sox10 down vs E11.5 * Ctrl
10412417	3110070M22Rik	ENSMUST00000(	0.000873693	-1.24217	E11.5 * Sox10 down vs E11.5 * Ctrl
10495562	Lrrc39	NM_175413	0.000502557	-1.24228	E11.5 * Sox10 down vs E11.5 * Ctrl
10349237		---	0.00343783	-1.24249	E11.5 * Sox10 down vs E11.5 * Ctrl
10530406	Gabra2	NM_008066	0.00602521	-1.2425	E11.5 * Sox10 down vs E11.5 * Ctrl
10464858	Bbs1	NM_001033128	0.00505353	-1.24271	E11.5 * Sox10 down vs E11.5 * Ctrl
10530421	Gabra4	NM_010251	0.00888741	-1.24286	E11.5 * Sox10 down vs E11.5 * Ctrl
10356248	C130026I21Rik	NM_175219	0.00229367	-1.24286	E11.5 * Sox10 down vs E11.5 * Ctrl
10399751	Colec11	NM_027866	0.00724082	-1.2429	E11.5 * Sox10 down vs E11.5 * Ctrl
10582626	Abcb10	NM_019552	0.00102027	-1.24319	E11.5 * Sox10 down vs E11.5 * Ctrl
10466745	Tjp2	NM_011597	0.00453861	-1.24321	E11.5 * Sox10 down vs E11.5 * Ctrl
10578324	Mtus1	NM_001005863	0.000621836	-1.24347	E11.5 * Sox10 down vs E11.5 * Ctrl
10585625	Sin3a	NM_011378	7.28432e-005	-1.24373	E11.5 * Sox10 down vs E11.5 * Ctrl
10590073	Vill	NM_011700	0.00490213	-1.244	E11.5 * Sox10 down vs E11.5 * Ctrl
10565735	A630091E08Rik	ENSMUST00000(	0.00643601	-1.24413	E11.5 * Sox10 down vs E11.5 * Ctrl
10585535	Isl2	NM_027397	0.00337828	-1.24415	E11.5 * Sox10 down vs E11.5 * Ctrl
10552156	Rhpn2	NM_027897	0.0099515	-1.24433	E11.5 * Sox10 down vs E11.5 * Ctrl
10578904	Cpe	NM_013494	0.000285165	-1.24471	E11.5 * Sox10 down vs E11.5 * Ctrl
10538811	Prdm5	NM_027547	0.00261517	-1.24487	E11.5 * Sox10 down vs E11.5 * Ctrl
10564938	Fes	NM_010194	2.74899e-005	-1.24524	E11.5 * Sox10 down vs E11.5 * Ctrl
10509280	Hspg2	NM_008305	0.000592003	-1.24529	E11.5 * Sox10 down vs E11.5 * Ctrl
10545127	4930597O21Rik	ENSMUST00000(	0.000185451	-1.24547	E11.5 * Sox10 down vs E11.5 * Ctrl
10347593	Speg	NM_007463	0.000871064	-1.24558	E11.5 * Sox10 down vs E11.5 * Ctrl
10515819		---	0.000783736	-1.24578	E11.5 * Sox10 down vs E11.5 * Ctrl
10513884	Tle1	NM_011599	2.06177e-005	-1.24592	E11.5 * Sox10 down vs E11.5 * Ctrl
10549506	Dennd5b	NM_177192	0.00236799	-1.24595	E11.5 * Sox10 down vs E11.5 * Ctrl
10472467		---	0.000309708	-1.246	E11.5 * Sox10 down vs E11.5 * Ctrl
10473432	Tnks1bp1	NM_001081260	0.00409401	-1.24603	E11.5 * Sox10 down vs E11.5 * Ctrl
10428453	Csmd3	NM_001081391	0.000707941	-1.24624	E11.5 * Sox10 down vs E11.5 * Ctrl
10595171	E330016A19Rik	BC052196	0.00434997	-1.24664	E11.5 * Sox10 down vs E11.5 * Ctrl
10362711	9030224M15Rik	NM_177793	0.00541789	-1.24667	E11.5 * Sox10 down vs E11.5 * Ctrl
10532241	Slc26a1	NM_174870	3.24325e-005	-1.24699	E11.5 * Sox10 down vs E11.5 * Ctrl
10436169	Ift57	NM_028680	0.00680477	-1.24724	E11.5 * Sox10 down vs E11.5 * Ctrl
10362536		---	0.00013451	-1.2474	E11.5 * Sox10 down vs E11.5 * Ctrl
10524331	C130026L21Rik	ENSMUST00000(	0.00875915	-1.24753	E11.5 * Sox10 down vs E11.5 * Ctrl
10599348	Gria3	NM_016886	0.00163013	-1.24768	E11.5 * Sox10 down vs E11.5 * Ctrl
10577395	6820431F20Rik	BC058969	0.000718596	-1.24824	E11.5 * Sox10 down vs E11.5 * Ctrl
10504094	Galt	NM_016658	8.56183e-005	-1.24852	E11.5 * Sox10 down vs E11.5 * Ctrl
10564704	Hapln3	NM_178255	0.00953717	-1.24877	E11.5 * Sox10 down vs E11.5 * Ctrl
10528177	ENSMUSG0000C	NM_198666	5.25902e-005	-1.24879	E11.5 * Sox10 down vs E11.5 * Ctrl
10415176	Dhrs4	NM_030686	0.00691697	-1.24915	E11.5 * Sox10 down vs E11.5 * Ctrl
10571246	5930422O12Rik	ENSMUST00000(	0.00332398	-1.24917	E11.5 * Sox10 down vs E11.5 * Ctrl
10398052	Serpina3h	NM_001034870	0.000351901	-1.24917	E11.5 * Sox10 down vs E11.5 * Ctrl
10368644	Fam26f	NM_175449	0.00711173	-1.24931	E11.5 * Sox10 down vs E11.5 * Ctrl
10458569	Nr3c1	NM_008173	0.00908861	-1.24938	E11.5 * Sox10 down vs E11.5 * Ctrl
10417561	Fam107a	NM_183187	0.00331013	-1.24967	E11.5 * Sox10 down vs E11.5 * Ctrl
10468885	Zfp826	ENSMUST00000(	0.00435801	-1.24987	E11.5 * Sox10 down vs E11.5 * Ctrl

10521626	Cc2d2a	NM_172274	0.0020862	-1.2499	E11.5 * Sox10 down vs E11.5 * Ctrl
10420694	Ints6	NM_008715	1.26166e-005	-1.25007	E11.5 * Sox10 down vs E11.5 * Ctrl
10451372	Ptk7	NM_175168	8.24709e-005	-1.2501	E11.5 * Sox10 down vs E11.5 * Ctrl
10504188	Ccl19	NM_011888	0.00852045	-1.25031	E11.5 * Sox10 down vs E11.5 * Ctrl
10493789	S100a13	NM_009113	0.000270477	-1.25039	E11.5 * Sox10 down vs E11.5 * Ctrl
10559238	Igf2as	NR_002855	0.00167977	-1.2504	E11.5 * Sox10 down vs E11.5 * Ctrl
10427049	6030408B16Rik	ENSMUST00000	0.00381843	-1.25085	E11.5 * Sox10 down vs E11.5 * Ctrl
10512011	Zfp292	NM_013889	8.49981e-005	-1.25151	E11.5 * Sox10 down vs E11.5 * Ctrl
10511755	Wwp1	NM_177327	0.00389316	-1.2517	E11.5 * Sox10 down vs E11.5 * Ctrl
10550509	Pglyrp1	NM_009402	0.00598905	-1.25175	E11.5 * Sox10 down vs E11.5 * Ctrl
10359086	Cep350	BC089561	0.000845823	-1.25182	E11.5 * Sox10 down vs E11.5 * Ctrl
10385870	Irf1	NM_008390	0.000191827	-1.25186	E11.5 * Sox10 down vs E11.5 * Ctrl
10463277	Nkx2-3	NM_008699	0.00407966	-1.2519	E11.5 * Sox10 down vs E11.5 * Ctrl
10444306	H2-Eb2	NM_001033978	0.00381034	-1.25191	E11.5 * Sox10 down vs E11.5 * Ctrl
10475567	Slc24a5	NM_175034	0.0027937	-1.25196	E11.5 * Sox10 down vs E11.5 * Ctrl
10398665	Tnfaip2	NM_009396	0.00211397	-1.25233	E11.5 * Sox10 down vs E11.5 * Ctrl
10412123	Ncf2	NM_010877	0.000517196	-1.2526	E11.5 * Sox10 down vs E11.5 * Ctrl
10354111	Aff3	NM_010678	0.00244051	-1.25271	E11.5 * Sox10 down vs E11.5 * Ctrl
10373073	D10Ertd610e	NM_028027	0.000982294	-1.25272	E11.5 * Sox10 down vs E11.5 * Ctrl
10598034	ND2	ENSMUST00000	3.15772e-005	-1.25274	E11.5 * Sox10 down vs E11.5 * Ctrl
10548701	Lrp6	NM_008514	0.00122035	-1.25275	E11.5 * Sox10 down vs E11.5 * Ctrl
10555873	Olfr652	NM_147048	0.0018387	-1.25288	E11.5 * Sox10 down vs E11.5 * Ctrl
10534202	Ncf1	NM_010876	0.0024074	-1.25289	E11.5 * Sox10 down vs E11.5 * Ctrl
10536827	Ccdc136	BC006583	0.000253183	-1.25295	E11.5 * Sox10 down vs E11.5 * Ctrl
10537184	Cald1	NM_145575	0.0022197	-1.25301	E11.5 * Sox10 down vs E11.5 * Ctrl
10603440	Was	NM_009515	0.00203612	-1.25318	E11.5 * Sox10 down vs E11.5 * Ctrl
10394852	EG668698	ENSMUST00000	0.00367896	-1.25328	E11.5 * Sox10 down vs E11.5 * Ctrl
10585851	Hcn4	NM_001081192	0.00138548	-1.25341	E11.5 * Sox10 down vs E11.5 * Ctrl
10420631	Ebpl	NM_026598	0.00374327	-1.25359	E11.5 * Sox10 down vs E11.5 * Ctrl
10400649	Pole2	NM_011133	0.00357634	-1.25361	E11.5 * Sox10 down vs E11.5 * Ctrl
10347748	Acsl3	NM_028817	0.000849706	-1.25364	E11.5 * Sox10 down vs E11.5 * Ctrl
10483178	Cobll1	NM_177025	0.00482817	-1.25368	E11.5 * Sox10 down vs E11.5 * Ctrl
10410016	Fancc	NM_007985	0.000106388	-1.25374	E11.5 * Sox10 down vs E11.5 * Ctrl
10361234	Hsd11b1	NM_008288	0.0022934	-1.25463	E11.5 * Sox10 down vs E11.5 * Ctrl
10585874	Hexa	NM_010421	0.00207661	-1.25468	E11.5 * Sox10 down vs E11.5 * Ctrl
10569437	Phlda2	NM_009434	0.000330351	-1.25471	E11.5 * Sox10 down vs E11.5 * Ctrl
10555425	Fam168a	BC079886	1.58212e-005	-1.25476	E11.5 * Sox10 down vs E11.5 * Ctrl
10435832	Gm608	NM_001029889	5.9302e-005	-1.25491	E11.5 * Sox10 down vs E11.5 * Ctrl
10574429		---	0.00883743	-1.25492	E11.5 * Sox10 down vs E11.5 * Ctrl
10414548	Rnase6	NM_030098	0.00314632	-1.25553	E11.5 * Sox10 down vs E11.5 * Ctrl
10445185	Pigt	AB057593	0.00427118	-1.25567	E11.5 * Sox10 down vs E11.5 * Ctrl
10420889	1110020C17Rik	ENSMUST00000	0.000829189	-1.25604	E11.5 * Sox10 down vs E11.5 * Ctrl
10490169	Ppp4r1l	ENSMUST00000	0.00194895	-1.25604	E11.5 * Sox10 down vs E11.5 * Ctrl
10370210	Col6a1	NM_009933	0.00156506	-1.25609	E11.5 * Sox10 down vs E11.5 * Ctrl
10607183	Lhfpl1	NM_178358	0.00822652	-1.25614	E11.5 * Sox10 down vs E11.5 * Ctrl
10367436	Cd63	NM_001042580	0.00125589	-1.25673	E11.5 * Sox10 down vs E11.5 * Ctrl
10395163	Lamb1-1	NM_008482	1.37635e-005	-1.25677	E11.5 * Sox10 down vs E11.5 * Ctrl
10490731	Sox18	NM_009236	0.00974642	-1.25681	E11.5 * Sox10 down vs E11.5 * Ctrl
10489253	Zhx3	NM_177263	0.00101219	-1.25702	E11.5 * Sox10 down vs E11.5 * Ctrl
10344789	Cspp1	NM_026493	0.00718371	-1.25715	E11.5 * Sox10 down vs E11.5 * Ctrl
10518847	Phf13	NM_172705	1.67448e-005	-1.25737	E11.5 * Sox10 down vs E11.5 * Ctrl
10455098	Pcdhb14	NM_053139	0.00173221	-1.25738	E11.5 * Sox10 down vs E11.5 * Ctrl
10416707		---	0.00764332	-1.25744	E11.5 * Sox10 down vs E11.5 * Ctrl
10431738	Prickle1	NM_001033217	0.00151485	-1.25756	E11.5 * Sox10 down vs E11.5 * Ctrl
10600825	Zc3h12b	NM_001034907	0.00555927	-1.25782	E11.5 * Sox10 down vs E11.5 * Ctrl
10348537	Ramp1	NM_016894	0.00361605	-1.25783	E11.5 * Sox10 down vs E11.5 * Ctrl
10557372	D430042O09Rik	ENSMUST00000	0.00158768	-1.25788	E11.5 * Sox10 down vs E11.5 * Ctrl
10485546	D430041D05Rik	NM_001033347	0.00797034	-1.25817	E11.5 * Sox10 down vs E11.5 * Ctrl
10550627	Gpr4	NM_175668	0.00456087	-1.25823	E11.5 * Sox10 down vs E11.5 * Ctrl
10412607	Abhd6	NM_025341	0.00844208	-1.25838	E11.5 * Sox10 down vs E11.5 * Ctrl
10507655		---	0.000417335	-1.25875	E11.5 * Sox10 down vs E11.5 * Ctrl
10579525	Plvap	NM_032398	0.00890897	-1.25921	E11.5 * Sox10 down vs E11.5 * Ctrl
10564343	Tjp1	NM_009386	1.34937e-006	-1.25927	E11.5 * Sox10 down vs E11.5 * Ctrl
10510167	OTTMUSG00000	NM_001037926	0.00848467	-1.2595	E11.5 * Sox10 down vs E11.5 * Ctrl
10414990		---	0.00865584	-1.25954	E11.5 * Sox10 down vs E11.5 * Ctrl
10474045	Chst1	NM_023850	0.00310902	-1.25964	E11.5 * Sox10 down vs E11.5 * Ctrl

10375838	Col23a1	NM_153393	0.00858171	-1.25979	E11.5 * Sox10 down vs E11.5 * Ctrl
10390458	Pcgf2	NM_009545	0.00379474	-1.25987	E11.5 * Sox10 down vs E11.5 * Ctrl
10383518	Hexdc	NM_001146073	0.000803112	-1.26005	E11.5 * Sox10 down vs E11.5 * Ctrl
10389070		---	0.00265872	-1.26008	E11.5 * Sox10 down vs E11.5 * Ctrl
10416522	Tsc22d1	NM_207652	9.55051e-006	-1.26025	E11.5 * Sox10 down vs E11.5 * Ctrl
10511723		---	0.00246053	-1.26033	E11.5 * Sox10 down vs E11.5 * Ctrl
10411082	Thbs4	NM_011582	0.00711765	-1.26051	E11.5 * Sox10 down vs E11.5 * Ctrl
10442240	Zfp760	NM_001008501	0.00640393	-1.26056	E11.5 * Sox10 down vs E11.5 * Ctrl
10404731	Tmem14c	NM_025387	0.000557291	-1.26077	E11.5 * Sox10 down vs E11.5 * Ctrl
10596166	1300017J02Rik	BC021390	0.000206197	-1.26096	E11.5 * Sox10 down vs E11.5 * Ctrl
10554586	4833418N17Rik	AK080712	0.00444606	-1.2611	E11.5 * Sox10 down vs E11.5 * Ctrl
10413398	Il17rd	NM_134437	0.00603809	-1.26139	E11.5 * Sox10 down vs E11.5 * Ctrl
10353002		---	0.0096048	-1.26175	E11.5 * Sox10 down vs E11.5 * Ctrl
10360412	Olfr419	NM_146715	0.00403554	-1.26182	E11.5 * Sox10 down vs E11.5 * Ctrl
10554150	Rgma	NM_177740	0.0010494	-1.26191	E11.5 * Sox10 down vs E11.5 * Ctrl
10424439	Efr3a	NM_133766	0.00503482	-1.26224	E11.5 * Sox10 down vs E11.5 * Ctrl
10432640	Bin2	ENSMUST00000	0.00469041	-1.26227	E11.5 * Sox10 down vs E11.5 * Ctrl
10399383	Kcns3	NM_173417	0.000744664	-1.26227	E11.5 * Sox10 down vs E11.5 * Ctrl
10555009		---	0.000967578	-1.26236	E11.5 * Sox10 down vs E11.5 * Ctrl
10562649		---	0.00377587	-1.26246	E11.5 * Sox10 down vs E11.5 * Ctrl
10540059	Slc41a3	NM_027868	0.00287557	-1.26268	E11.5 * Sox10 down vs E11.5 * Ctrl
10593799	Scaper	NM_001081341	0.000430973	-1.26269	E11.5 * Sox10 down vs E11.5 * Ctrl
10601771	Armcx1	NM_030066	0.00172545	-1.26274	E11.5 * Sox10 down vs E11.5 * Ctrl
10415806	Fam124a	ENSMUST00000	0.00292672	-1.26273	E11.5 * Sox10 down vs E11.5 * Ctrl
10359648	Scyl3	NM_028776	1.47435e-005	-1.2629	E11.5 * Sox10 down vs E11.5 * Ctrl
10416057	Clu	NM_013492	0.00711227	-1.26319	E11.5 * Sox10 down vs E11.5 * Ctrl
10602180	Tmem164	NM_177592	0.00687973	-1.26352	E11.5 * Sox10 down vs E11.5 * Ctrl
10590799	C330006D17Rik	ENSMUST00000	0.00345479	-1.26374	E11.5 * Sox10 down vs E11.5 * Ctrl
10505481	8030463A06Rik	ENSMUST00000	0.00273309	-1.26387	E11.5 * Sox10 down vs E11.5 * Ctrl
10491564	4932438A13Rik	NM_172679	0.00301927	-1.26403	E11.5 * Sox10 down vs E11.5 * Ctrl
10474399	Bdnf	NM_001048139	2.78092e-005	-1.2642	E11.5 * Sox10 down vs E11.5 * Ctrl
10569102	Irf7	NM_016850	0.0022888	-1.2648	E11.5 * Sox10 down vs E11.5 * Ctrl
10373325	Gpr182	NM_007412	0.00143674	-1.26485	E11.5 * Sox10 down vs E11.5 * Ctrl
10561431	Plekhg2	NM_138752	0.0083189	-1.26491	E11.5 * Sox10 down vs E11.5 * Ctrl
10461402	Fth1	NM_010239	1.44155e-008	-1.265	E11.5 * Sox10 down vs E11.5 * Ctrl
10543600	Kcp	NM_001029985	0.000282021	-1.26522	E11.5 * Sox10 down vs E11.5 * Ctrl
10604032		---	0.00325403	-1.26547	E11.5 * Sox10 down vs E11.5 * Ctrl
10379044	Rab34	NM_033475	0.00167946	-1.26548	E11.5 * Sox10 down vs E11.5 * Ctrl
10361246	G0s2	NM_008059	0.00129233	-1.26554	E11.5 * Sox10 down vs E11.5 * Ctrl
10606248	Magee2	NM_053206	0.00555831	-1.26567	E11.5 * Sox10 down vs E11.5 * Ctrl
10487645	Cpxm1	NM_019696	0.0061884	-1.26595	E11.5 * Sox10 down vs E11.5 * Ctrl
10357535	Pfkfb2	NM_008825	0.00438046	-1.26646	E11.5 * Sox10 down vs E11.5 * Ctrl
10575160	Nfat5	NM_133957	6.4381e-005	-1.26653	E11.5 * Sox10 down vs E11.5 * Ctrl
10578253	Sgcz	NM_145841	0.00463274	-1.26684	E11.5 * Sox10 down vs E11.5 * Ctrl
10598101	Maml2	NM_001013813	0.00137843	-1.26686	E11.5 * Sox10 down vs E11.5 * Ctrl
10530269	Rbm47	NM_178446	0.00987276	-1.26693	E11.5 * Sox10 down vs E11.5 * Ctrl
10383152		---	9.70555e-005	-1.26711	E11.5 * Sox10 down vs E11.5 * Ctrl
10519344		---	0.0021109	-1.26711	E11.5 * Sox10 down vs E11.5 * Ctrl
10401586	Prox2	NM_175198	0.000514447	-1.26719	E11.5 * Sox10 down vs E11.5 * Ctrl
10465314	Capn1	NM_007600	0.00874136	-1.2672	E11.5 * Sox10 down vs E11.5 * Ctrl
10548146	Tead4	NM_011567	0.000384766	-1.2674	E11.5 * Sox10 down vs E11.5 * Ctrl
10507479	Tmem53	NM_026837	0.00125434	-1.26753	E11.5 * Sox10 down vs E11.5 * Ctrl
10543253	Wnt2	NM_023653	0.0030439	-1.26778	E11.5 * Sox10 down vs E11.5 * Ctrl
10425138	Sh3bp1	NM_009164	4.51838e-005	-1.26818	E11.5 * Sox10 down vs E11.5 * Ctrl
10498500	Vmn2r1	NM_019918	0.00662574	-1.26823	E11.5 * Sox10 down vs E11.5 * Ctrl
10384365	4930512M02Rik	ENSMUST00000	0.00109556	-1.26845	E11.5 * Sox10 down vs E11.5 * Ctrl
10414271	Ptger2	NM_008964	0.00528397	-1.26857	E11.5 * Sox10 down vs E11.5 * Ctrl
10606436	Nsbp1	NM_016710	0.00570252	-1.26858	E11.5 * Sox10 down vs E11.5 * Ctrl
10384770	5730522E02Rik	ENSMUST00000	0.00153063	-1.26862	E11.5 * Sox10 down vs E11.5 * Ctrl
10584855	Scn2b	NM_001014761	0.00111274	-1.26868	E11.5 * Sox10 down vs E11.5 * Ctrl
10488060	Jag1	NM_013822	0.00973343	-1.2688	E11.5 * Sox10 down vs E11.5 * Ctrl
10502071	5730508B09Rik	NM_027482	0.00471133	-1.26902	E11.5 * Sox10 down vs E11.5 * Ctrl
10362803	Cd164	NM_016898	0.000142461	-1.26903	E11.5 * Sox10 down vs E11.5 * Ctrl
10357480	Daf2	NM_007827	0.00130738	-1.26924	E11.5 * Sox10 down vs E11.5 * Ctrl
10595768	Pls1	NM_001033210	0.00217657	-1.26927	E11.5 * Sox10 down vs E11.5 * Ctrl
10436830	Ifnar2	NM_010509	0.00224603	-1.26943	E11.5 * Sox10 down vs E11.5 * Ctrl

10598178	Disp1	NM_026866	0.000618694	-1.26982	E11.5 * Sox10 down vs E11.5 * Ctrl
10574259	Gpr56	NM_018882	0.000898417	-1.26984	E11.5 * Sox10 down vs E11.5 * Ctrl
10481186	Sardh	NM_138665	0.0013466	-1.26993	E11.5 * Sox10 down vs E11.5 * Ctrl
10548367	Clec1a	NM_175526	0.0015761	-1.27012	E11.5 * Sox10 down vs E11.5 * Ctrl
10445640	Trerf1	NM_001097623	0.00172422	-1.27014	E11.5 * Sox10 down vs E11.5 * Ctrl
10545096	2410003J06Rik	NM_028025	0.000607491	-1.27033	E11.5 * Sox10 down vs E11.5 * Ctrl
10351443	Lmx1a	NM_033652	0.00986065	-1.27065	E11.5 * Sox10 down vs E11.5 * Ctrl
10576413		---	0.000125448	-1.27085	E11.5 * Sox10 down vs E11.5 * Ctrl
10571922	Nek1	NM_175089	0.00752838	-1.27088	E11.5 * Sox10 down vs E11.5 * Ctrl
10544348	Trpv6	NM_022413	0.000156084	-1.27098	E11.5 * Sox10 down vs E11.5 * Ctrl
10471464	St6galnac6	NM_016973	0.000481514	-1.27112	E11.5 * Sox10 down vs E11.5 * Ctrl
10536908	Tspan33	NM_146173	0.000273445	-1.27128	E11.5 * Sox10 down vs E11.5 * Ctrl
10595560	Tbx18	NM_023814	0.000202117	-1.27137	E11.5 * Sox10 down vs E11.5 * Ctrl
10565193	Hdgfrp3	NM_013886	0.000342465	-1.27155	E11.5 * Sox10 down vs E11.5 * Ctrl
10553559	Siglech	NM_178706	0.00997595	-1.27174	E11.5 * Sox10 down vs E11.5 * Ctrl
10455472	Dcp2	NM_027490	0.000896599	-1.27192	E11.5 * Sox10 down vs E11.5 * Ctrl
10411215	Crhbp	NM_198408	0.0098032	-1.27208	E11.5 * Sox10 down vs E11.5 * Ctrl
10462881	Gpr120	NM_181748	0.0024854	-1.27209	E11.5 * Sox10 down vs E11.5 * Ctrl
10554061	Adamts17	NM_001033877	0.000224276	-1.27213	E11.5 * Sox10 down vs E11.5 * Ctrl
10459225		---	0.00598944	-1.27215	E11.5 * Sox10 down vs E11.5 * Ctrl
10398350		---	0.000561075	-1.2725	E11.5 * Sox10 down vs E11.5 * Ctrl
10587383	Cd109	NM_153098	0.000140659	-1.27263	E11.5 * Sox10 down vs E11.5 * Ctrl
10382438	Cd300a	NM_170758	0.00527695	-1.27314	E11.5 * Sox10 down vs E11.5 * Ctrl
10474518		---	0.00368446	-1.27316	E11.5 * Sox10 down vs E11.5 * Ctrl
10475866	Bcl2l11	NM_207680	0.00126014	-1.27316	E11.5 * Sox10 down vs E11.5 * Ctrl
10505627	LOC100040144	AK085025	0.00341486	-1.27407	E11.5 * Sox10 down vs E11.5 * Ctrl
10380398	Chad	NM_007689	0.000136778	-1.2741	E11.5 * Sox10 down vs E11.5 * Ctrl
10606864	Tceal5	NM_177919	0.00222833	-1.27411	E11.5 * Sox10 down vs E11.5 * Ctrl
10526546		---	0.00578592	-1.27426	E11.5 * Sox10 down vs E11.5 * Ctrl
10473584		---	0.00190778	-1.27432	E11.5 * Sox10 down vs E11.5 * Ctrl
10363701		---	0.00400655	-1.27437	E11.5 * Sox10 down vs E11.5 * Ctrl
10440909	4932438H23Rik	ENSMUST00000	0.00012498	-1.27449	E11.5 * Sox10 down vs E11.5 * Ctrl
10372421	Trhde	NM_146241	0.00350062	-1.27459	E11.5 * Sox10 down vs E11.5 * Ctrl
10553042	Rasip1	NM_028544	0.000941041	-1.27482	E11.5 * Sox10 down vs E11.5 * Ctrl
10502284	Tet2	NM_001040400	0.00108435	-1.27502	E11.5 * Sox10 down vs E11.5 * Ctrl
10555235	Arrb1	NM_177231	0.000111266	-1.27508	E11.5 * Sox10 down vs E11.5 * Ctrl
10607124	Chrdl1	NM_001114385	0.00768976	-1.27512	E11.5 * Sox10 down vs E11.5 * Ctrl
10597918		---	0.00469141	-1.27562	E11.5 * Sox10 down vs E11.5 * Ctrl
10589541	Kif9	NM_010628	0.000324393	-1.27566	E11.5 * Sox10 down vs E11.5 * Ctrl
10464084	Tcf7l2	NM_001142918	4.02611e-005	-1.2759	E11.5 * Sox10 down vs E11.5 * Ctrl
10384579	Ugp2	NM_139297	0.000965372	-1.27599	E11.5 * Sox10 down vs E11.5 * Ctrl
10462130	Fam122a	NM_026520	0.00317394	-1.27627	E11.5 * Sox10 down vs E11.5 * Ctrl
10605370	Mpp1	NM_008621	0.00402988	-1.27633	E11.5 * Sox10 down vs E11.5 * Ctrl
10495987	EG435755	DQ851564	0.00551766	-1.27731	E11.5 * Sox10 down vs E11.5 * Ctrl
10446986	Crim1	NM_015800	0.00190474	-1.27758	E11.5 * Sox10 down vs E11.5 * Ctrl
10538299	Hoxa3	NM_010452	0.00104862	-1.27763	E11.5 * Sox10 down vs E11.5 * Ctrl
10428103	Stk3	NM_019635	0.00029014	-1.27768	E11.5 * Sox10 down vs E11.5 * Ctrl
10535898	Gnpda1	NM_011937	0.00844402	-1.27776	E11.5 * Sox10 down vs E11.5 * Ctrl
10476237	Hspa12b	NM_028306	0.00384425	-1.2778	E11.5 * Sox10 down vs E11.5 * Ctrl
10418848	Wdfy4	NM_001146022	0.0024224	-1.27784	E11.5 * Sox10 down vs E11.5 * Ctrl
10519988	AI847670	NM_177869	0.00693966	-1.27788	E11.5 * Sox10 down vs E11.5 * Ctrl
10590267	Snora62	NR_002902	0.00169574	-1.27821	E11.5 * Sox10 down vs E11.5 * Ctrl
10587558	Dopey1	NM_177208	0.00381718	-1.27822	E11.5 * Sox10 down vs E11.5 * Ctrl
10490854	OTTMUSG00000	NM_001037926	0.00989499	-1.27862	E11.5 * Sox10 down vs E11.5 * Ctrl
10548105	Ccnd2	NM_009829	0.00110341	-1.27864	E11.5 * Sox10 down vs E11.5 * Ctrl
10538590	Herc5	ENSMUST00000	0.00264783	-1.27864	E11.5 * Sox10 down vs E11.5 * Ctrl
10590060	Ctdspl	NM_133710	0.000129621	-1.27873	E11.5 * Sox10 down vs E11.5 * Ctrl
10400057	Arl4a	NM_001039515	0.000284462	-1.27893	E11.5 * Sox10 down vs E11.5 * Ctrl
10508436	Sync	NM_023485	0.00252354	-1.27915	E11.5 * Sox10 down vs E11.5 * Ctrl
10593050	Il10ra	NM_008348	0.00635454	-1.27978	E11.5 * Sox10 down vs E11.5 * Ctrl
10532103	Glmn	NM_133248	0.0017354	-1.27997	E11.5 * Sox10 down vs E11.5 * Ctrl
10494643	Hmgcs2	NM_008256	0.00545955	-1.28008	E11.5 * Sox10 down vs E11.5 * Ctrl
10347697	Slc4a3	NM_009208	0.00227446	-1.28024	E11.5 * Sox10 down vs E11.5 * Ctrl
10506786	Zcchc11	NM_175472	0.000725383	-1.28035	E11.5 * Sox10 down vs E11.5 * Ctrl
10433792		---	0.000882362	-1.28042	E11.5 * Sox10 down vs E11.5 * Ctrl
10463227	EG628994	BC089359	0.00354132	-1.28051	E11.5 * Sox10 down vs E11.5 * Ctrl

10377537	Chd3	NM_146019	8.48618e-007	-1.2807	E11.5 * Sox10 down vs E11.5 * Ctrl
10548375	Clec7a	NM_020008	0.00119928	-1.28078	E11.5 * Sox10 down vs E11.5 * Ctrl
10504159	Ccl19	NM_011888	0.00789349	-1.28093	E11.5 * Sox10 down vs E11.5 * Ctrl
10512322	Ccl19	NM_011888	0.00789349	-1.28093	E11.5 * Sox10 down vs E11.5 * Ctrl
10482766	Rprm	NM_023396	0.00309419	-1.28112	E11.5 * Sox10 down vs E11.5 * Ctrl
10437330	Crebbp	ENSMUST00000	1.86756e-007	-1.28123	E11.5 * Sox10 down vs E11.5 * Ctrl
10548661		---	0.00500988	-1.2813	E11.5 * Sox10 down vs E11.5 * Ctrl
10351509	Fcgr4	NM_144559	0.00596166	-1.28133	E11.5 * Sox10 down vs E11.5 * Ctrl
10458983	March3	NM_177115	0.00411578	-1.28182	E11.5 * Sox10 down vs E11.5 * Ctrl
10559644	Tmem224	NM_177887	0.00735482	-1.28235	E11.5 * Sox10 down vs E11.5 * Ctrl
10537179	Bpgm	NM_007563	0.00240384	-1.28257	E11.5 * Sox10 down vs E11.5 * Ctrl
10580795	Ccdc102a	NM_001033533	0.00242064	-1.2831	E11.5 * Sox10 down vs E11.5 * Ctrl
10531479	4932430I15Rik	ENSMUST00000	0.000872988	-1.28345	E11.5 * Sox10 down vs E11.5 * Ctrl
10384044	Myl7	NM_022879	0.000131274	-1.28348	E11.5 * Sox10 down vs E11.5 * Ctrl
10503148		---	0.00506592	-1.2836	E11.5 * Sox10 down vs E11.5 * Ctrl
10404152	Fam65b	NM_029679	0.00901405	-1.28433	E11.5 * Sox10 down vs E11.5 * Ctrl
10472970	Hoxd4	NM_010469	0.00257882	-1.28447	E11.5 * Sox10 down vs E11.5 * Ctrl
10544062	D630045J12Rik	ENSMUST00000	7.48151e-005	-1.28486	E11.5 * Sox10 down vs E11.5 * Ctrl
10434229	Cldn5	NM_013805	0.00243843	-1.28488	E11.5 * Sox10 down vs E11.5 * Ctrl
10385893	Slc22a4	NM_019687	0.00129847	-1.28489	E11.5 * Sox10 down vs E11.5 * Ctrl
10433717		---	0.000382687	-1.28507	E11.5 * Sox10 down vs E11.5 * Ctrl
10476880	A530006G24Rik	XR_034699	0.00271013	-1.28515	E11.5 * Sox10 down vs E11.5 * Ctrl
10391332	Ptrf	NM_008986	0.00220125	-1.28531	E11.5 * Sox10 down vs E11.5 * Ctrl
10607116	Ammecr1	NM_019496	0.00224482	-1.28542	E11.5 * Sox10 down vs E11.5 * Ctrl
10606026	Zmym3	NM_019831	0.00138357	-1.28556	E11.5 * Sox10 down vs E11.5 * Ctrl
10374083	Aebp1	NM_009636	0.00411069	-1.2856	E11.5 * Sox10 down vs E11.5 * Ctrl
10388545	Abr	NM_198895	0.00205753	-1.28582	E11.5 * Sox10 down vs E11.5 * Ctrl
10598528	Ssxb9	NM_199066	0.00129631	-1.28611	E11.5 * Sox10 down vs E11.5 * Ctrl
10403229	Itgb8	NM_177290	0.00134316	-1.28634	E11.5 * Sox10 down vs E11.5 * Ctrl
10492091	Smad9	NM_019483	0.00223938	-1.28637	E11.5 * Sox10 down vs E11.5 * Ctrl
10344879	A830018L16Rik	NM_177173	0.00234064	-1.28652	E11.5 * Sox10 down vs E11.5 * Ctrl
10383196		---	0.0102003	-1.28713	E11.5 * Sox10 down vs E11.5 * Ctrl
10428089	Nipal2	NM_145469	0.00619156	-1.28715	E11.5 * Sox10 down vs E11.5 * Ctrl
10495933	4930422G04Rik	BC079891	0.00287978	-1.28715	E11.5 * Sox10 down vs E11.5 * Ctrl
10500754	Sycp1	NM_011516	0.000246467	-1.28744	E11.5 * Sox10 down vs E11.5 * Ctrl
10428604	Tnfrsf11b	NM_008764	0.00224256	-1.28747	E11.5 * Sox10 down vs E11.5 * Ctrl
10485622	Qser1	NM_001123327	0.00268383	-1.28752	E11.5 * Sox10 down vs E11.5 * Ctrl
10515385	Urod	NM_009478	0.00019483	-1.28774	E11.5 * Sox10 down vs E11.5 * Ctrl
10539517	Dysf	NM_021469	6.05535e-005	-1.28797	E11.5 * Sox10 down vs E11.5 * Ctrl
10418244	Fam116a	NM_001134465	0.00724795	-1.28803	E11.5 * Sox10 down vs E11.5 * Ctrl
10544417	Epha1	NM_023580	0.00484584	-1.28816	E11.5 * Sox10 down vs E11.5 * Ctrl
10459066	LOC100044195	BC150900	0.00122408	-1.28853	E11.5 * Sox10 down vs E11.5 * Ctrl
10552208	Nudt19	NM_033080	0.00150753	-1.28858	E11.5 * Sox10 down vs E11.5 * Ctrl
10471486	Eng	NM_001146350	0.00039159	-1.28869	E11.5 * Sox10 down vs E11.5 * Ctrl
10416709	Tdrd3	NM_172605	5.45673e-005	-1.2891	E11.5 * Sox10 down vs E11.5 * Ctrl
10377802		---	0.000910289	-1.28922	E11.5 * Sox10 down vs E11.5 * Ctrl
10399232	ENSMUSG0000C	ENSMUST00000	2.13623e-005	-1.28941	E11.5 * Sox10 down vs E11.5 * Ctrl
10586446		---	0.00576416	-1.2896	E11.5 * Sox10 down vs E11.5 * Ctrl
10364072	Ggt5	NM_011820	5.75725e-005	-1.28964	E11.5 * Sox10 down vs E11.5 * Ctrl
10577963	Got1l1	NM_029674	0.000655941	-1.28966	E11.5 * Sox10 down vs E11.5 * Ctrl
10518335	OTTMUSG0000C	NM_001083918	0.000846501	-1.28977	E11.5 * Sox10 down vs E11.5 * Ctrl
10480601	A830007P12Rik	NM_146115	0.00351546	-1.28987	E11.5 * Sox10 down vs E11.5 * Ctrl
10584325	Vsig2	NM_020518	0.0012237	-1.29005	E11.5 * Sox10 down vs E11.5 * Ctrl
10379060	Proca1	NM_001045516	0.000495327	-1.2902	E11.5 * Sox10 down vs E11.5 * Ctrl
10472235	Dapl1	NM_029723	0.00561678	-1.29042	E11.5 * Sox10 down vs E11.5 * Ctrl
10569485	Tnfrsf26	NM_175649	0.00821303	-1.29059	E11.5 * Sox10 down vs E11.5 * Ctrl
10546024	Prokr1	NM_021381	0.00114904	-1.29078	E11.5 * Sox10 down vs E11.5 * Ctrl
10443080	Syngap1	XM_985548	0.00318914	-1.29082	E11.5 * Sox10 down vs E11.5 * Ctrl
10512334	100038993	NM_001100596	9.2205e-005	-1.29091	E11.5 * Sox10 down vs E11.5 * Ctrl
10512356	100038993	NM_001100596	9.2205e-005	-1.29091	E11.5 * Sox10 down vs E11.5 * Ctrl
10511819	Klhl32	NM_001033531	0.00214661	-1.29103	E11.5 * Sox10 down vs E11.5 * Ctrl
10428449		---	0.00405188	-1.29121	E11.5 * Sox10 down vs E11.5 * Ctrl
10400971	Six4	NM_011382	0.00228629	-1.2914	E11.5 * Sox10 down vs E11.5 * Ctrl
10592251	Pknox2	NM_001029838	0.00459675	-1.29156	E11.5 * Sox10 down vs E11.5 * Ctrl
10512372	Ccl19	NM_011888	0.00431362	-1.29162	E11.5 * Sox10 down vs E11.5 * Ctrl
10452648	Emilin2	NM_145158	0.00601719	-1.29277	E11.5 * Sox10 down vs E11.5 * Ctrl

10575844	Cdh13	NM_019707	0.00813806	-1.29284	E11.5 * Sox10 down vs E11.5 * Ctrl
10498371	P2ry12	NM_027571	0.00404933	-1.29288	E11.5 * Sox10 down vs E11.5 * Ctrl
10518069	Efhd2	NM_025994	0.00613752	-1.29309	E11.5 * Sox10 down vs E11.5 * Ctrl
10584827	Mpzl2	NM_007962	0.00794407	-1.29315	E11.5 * Sox10 down vs E11.5 * Ctrl
10398388		---	0.00341044	-1.29318	E11.5 * Sox10 down vs E11.5 * Ctrl
10368356	Akap7	NM_018747	0.00112458	-1.29318	E11.5 * Sox10 down vs E11.5 * Ctrl
10556039	Olfr17	NM_020598	0.00979773	-1.29344	E11.5 * Sox10 down vs E11.5 * Ctrl
10463224	Marveld1	BC054384	0.000435883	-1.29417	E11.5 * Sox10 down vs E11.5 * Ctrl
10558088		---	0.0011681	-1.29464	E11.5 * Sox10 down vs E11.5 * Ctrl
10394858		---	0.0050495	-1.2947	E11.5 * Sox10 down vs E11.5 * Ctrl
10385500	Irgm1	NM_008326	0.00274608	-1.29495	E11.5 * Sox10 down vs E11.5 * Ctrl
10564624	St8sia2	NM_009181	0.00369515	-1.2951	E11.5 * Sox10 down vs E11.5 * Ctrl
10503654		---	0.00381827	-1.29551	E11.5 * Sox10 down vs E11.5 * Ctrl
10512384	BC049635	NM_177785	0.000260463	-1.29574	E11.5 * Sox10 down vs E11.5 * Ctrl
10490872	Lrrcc1	NM_028915	0.00335855	-1.29575	E11.5 * Sox10 down vs E11.5 * Ctrl
10504775	Col15a1	NM_009928	0.00047139	-1.29575	E11.5 * Sox10 down vs E11.5 * Ctrl
10483131	Kcnh7	NM_133207	0.00304262	-1.29605	E11.5 * Sox10 down vs E11.5 * Ctrl
10602105	Col4a5	NM_007736	0.00109684	-1.29616	E11.5 * Sox10 down vs E11.5 * Ctrl
10512129	B4galt1	NM_022305	0.00602599	-1.2969	E11.5 * Sox10 down vs E11.5 * Ctrl
10417704	Lrrc3b	NM_146052	0.00205002	-1.29695	E11.5 * Sox10 down vs E11.5 * Ctrl
10559890	Vmn2r43	NM_198961	0.00128189	-1.29695	E11.5 * Sox10 down vs E11.5 * Ctrl
10384154	Myo1g	NM_178440	0.000787357	-1.29696	E11.5 * Sox10 down vs E11.5 * Ctrl
10579776	Arhgap10	NM_030113	0.000136341	-1.29733	E11.5 * Sox10 down vs E11.5 * Ctrl
10359375	Gpr52	NM_001146330	0.00395881	-1.29751	E11.5 * Sox10 down vs E11.5 * Ctrl
10350630	Fam129a	NM_022018	0.00586582	-1.29799	E11.5 * Sox10 down vs E11.5 * Ctrl
10537410	Tbxas1	NM_011539	0.000346314	-1.2981	E11.5 * Sox10 down vs E11.5 * Ctrl
10582868	Sp110	NM_175397	0.00210674	-1.29848	E11.5 * Sox10 down vs E11.5 * Ctrl
10578241	A730069N07Rik	NM_178735	0.000124365	-1.29864	E11.5 * Sox10 down vs E11.5 * Ctrl
10478678	Cd40	NM_170701	0.000172922	-1.29901	E11.5 * Sox10 down vs E11.5 * Ctrl
10387797	Bcl6b	NM_007528	0.00614173	-1.29928	E11.5 * Sox10 down vs E11.5 * Ctrl
10474223	Cd59b	NM_181858	0.00913062	-1.29938	E11.5 * Sox10 down vs E11.5 * Ctrl
10555438	Fchsd2	NM_199012	1.08071e-005	-1.29954	E11.5 * Sox10 down vs E11.5 * Ctrl
10522784	Hmgn2	BC083085	0.00444587	-1.2997	E11.5 * Sox10 down vs E11.5 * Ctrl
10398240	Eml1	NM_001043335	0.00115421	-1.29996	E11.5 * Sox10 down vs E11.5 * Ctrl
10600921	Stard8	NM_199018	0.000229366	-1.30021	E11.5 * Sox10 down vs E11.5 * Ctrl
10551872	AI428936	NM_153577	0.00514769	-1.30029	E11.5 * Sox10 down vs E11.5 * Ctrl
10453231	Slc8a1	NM_011406	0.00677336	-1.30034	E11.5 * Sox10 down vs E11.5 * Ctrl
10483439	Lrp2	NM_001081088	9.25998e-005	-1.30061	E11.5 * Sox10 down vs E11.5 * Ctrl
10497548	Fndc3b	NM_173182	0.00355077	-1.30068	E11.5 * Sox10 down vs E11.5 * Ctrl
10449731	U2af1	NM_024187	0.0101938	-1.30082	E11.5 * Sox10 down vs E11.5 * Ctrl
10436865	Ifngr2	NM_008338	0.00847061	-1.30124	E11.5 * Sox10 down vs E11.5 * Ctrl
10556426	Parva	NM_020606	0.00223538	-1.30128	E11.5 * Sox10 down vs E11.5 * Ctrl
10398424		---	5.16155e-005	-1.30135	E11.5 * Sox10 down vs E11.5 * Ctrl
10604735	Rbmx	NM_011252	0.00116473	-1.30139	E11.5 * Sox10 down vs E11.5 * Ctrl
10495927		---	0.00743188	-1.30162	E11.5 * Sox10 down vs E11.5 * Ctrl
10349051	Tnfrsf11a	NM_009399	0.0019758	-1.30184	E11.5 * Sox10 down vs E11.5 * Ctrl
10484205	2610301F02Rik	ENSMUST00000(	0.00496829	-1.30215	E11.5 * Sox10 down vs E11.5 * Ctrl
10408861	9530008L14Rik	NM_175417	0.00396786	-1.30226	E11.5 * Sox10 down vs E11.5 * Ctrl
10583207	Maml2	NM_001013813	0.0072719	-1.30305	E11.5 * Sox10 down vs E11.5 * Ctrl
10512499	Tpm2	NM_009416	0.000243885	-1.30334	E11.5 * Sox10 down vs E11.5 * Ctrl
10541246	Il17ra	NM_008359	0.00937559	-1.30338	E11.5 * Sox10 down vs E11.5 * Ctrl
10453811	AK220484	NM_001083628	0.00498788	-1.30338	E11.5 * Sox10 down vs E11.5 * Ctrl
10361156	Rcor3	NM_144814	0.00121112	-1.3034	E11.5 * Sox10 down vs E11.5 * Ctrl
10456383	Impa2	NM_053261	7.79223e-005	-1.30394	E11.5 * Sox10 down vs E11.5 * Ctrl
10549758	4632433K11Rik	NM_029849	0.000649274	-1.30395	E11.5 * Sox10 down vs E11.5 * Ctrl
10601874	Tceal3	NM_001029978	0.00123979	-1.30417	E11.5 * Sox10 down vs E11.5 * Ctrl
10433403	A2bp1	NM_021477	0.00236148	-1.30434	E11.5 * Sox10 down vs E11.5 * Ctrl
10600786	Klhl15	NM_001039060	0.000297933	-1.30438	E11.5 * Sox10 down vs E11.5 * Ctrl
10469828	Psd4	NM_177611	0.00234498	-1.30495	E11.5 * Sox10 down vs E11.5 * Ctrl
10592719	Oaf	NM_178644	2.01251e-005	-1.30582	E11.5 * Sox10 down vs E11.5 * Ctrl
10412830	Ngly1	NM_021504	0.000638109	-1.306	E11.5 * Sox10 down vs E11.5 * Ctrl
10396485	Syne2	NM_001005510	7.03189e-006	-1.30602	E11.5 * Sox10 down vs E11.5 * Ctrl
10350102	Ptpn7	NM_177081	0.000635633	-1.30623	E11.5 * Sox10 down vs E11.5 * Ctrl
10505788	Acer2	BC051923	0.00705944	-1.30645	E11.5 * Sox10 down vs E11.5 * Ctrl
10413434	D14Abb1e	NM_001114879	0.000721073	-1.30668	E11.5 * Sox10 down vs E11.5 * Ctrl
10501051	Cept1	NM_133869	0.000551675	-1.30681	E11.5 * Sox10 down vs E11.5 * Ctrl

10602625	Iqsec2	NM_001005475	0.000131433	-1.30758	E11.5 * Sox10 down vs E11.5 * Ctrl
10430572		---	0.00476517	-1.30762	E11.5 * Sox10 down vs E11.5 * Ctrl
10415408	BC030046	BC151043	0.00400791	-1.30778	E11.5 * Sox10 down vs E11.5 * Ctrl
10550795	Nlrp5	NM_011860	0.00265336	-1.30802	E11.5 * Sox10 down vs E11.5 * Ctrl
10579852	Mmaa	NM_133823	0.000212986	-1.30837	E11.5 * Sox10 down vs E11.5 * Ctrl
10434942	Dlg1	NM_007862	7.22274e-005	-1.30873	E11.5 * Sox10 down vs E11.5 * Ctrl
10547396	Wnt5b	NM_009525	0.00526335	-1.3088	E11.5 * Sox10 down vs E11.5 * Ctrl
10527605	C130038G02Rik	NM_029920	0.000240703	-1.30946	E11.5 * Sox10 down vs E11.5 * Ctrl
10599296	Rhox6	NM_008955	0.000523956	-1.3095	E11.5 * Sox10 down vs E11.5 * Ctrl
10390186	Abi3	NM_025659	0.000146632	-1.30954	E11.5 * Sox10 down vs E11.5 * Ctrl
10529937	Kcnip4	NM_030265	0.0049539	-1.31001	E11.5 * Sox10 down vs E11.5 * Ctrl
10568765	Tcerg1l	NM_183289	8.08669e-005	-1.31007	E11.5 * Sox10 down vs E11.5 * Ctrl
10410235		---	0.00742612	-1.31014	E11.5 * Sox10 down vs E11.5 * Ctrl
10575213	Zfhx3	NM_007496	0.00137156	-1.31062	E11.5 * Sox10 down vs E11.5 * Ctrl
10557960	Tgfb1i1	NM_009365	0.0022741	-1.31071	E11.5 * Sox10 down vs E11.5 * Ctrl
10360235	Casq1	NM_009813	0.00614599	-1.31076	E11.5 * Sox10 down vs E11.5 * Ctrl
10389786	Hlf	NM_172563	0.000420751	-1.31113	E11.5 * Sox10 down vs E11.5 * Ctrl
10523021	Slc4a4	NM_018760	0.00208306	-1.31114	E11.5 * Sox10 down vs E11.5 * Ctrl
10565152	9330120H11Rik	ENSMUST00000(	0.00838615	-1.31116	E11.5 * Sox10 down vs E11.5 * Ctrl
10425866	Parvg	NM_022321	0.00462043	-1.31144	E11.5 * Sox10 down vs E11.5 * Ctrl
10586433	Rbpms2	NM_028030	0.00317264	-1.31159	E11.5 * Sox10 down vs E11.5 * Ctrl
10496457	Adh6b	ENSMUST00000(	0.00574434	-1.31167	E11.5 * Sox10 down vs E11.5 * Ctrl
10508972	434166	BC080727	0.00173862	-1.31174	E11.5 * Sox10 down vs E11.5 * Ctrl
10560618	Apoc1	NM_007469	0.00512395	-1.31207	E11.5 * Sox10 down vs E11.5 * Ctrl
10589884	Bcl2a1c	NM_007535	0.000871305	-1.31209	E11.5 * Sox10 down vs E11.5 * Ctrl
10513381	Rod1	NM_144904	4.53062e-007	-1.31219	E11.5 * Sox10 down vs E11.5 * Ctrl
10426284	Acr	NM_013455	0.000354012	-1.31256	E11.5 * Sox10 down vs E11.5 * Ctrl
10455118	Pcdhb18	NM_053143	0.00546525	-1.31296	E11.5 * Sox10 down vs E11.5 * Ctrl
10433797	Prkdc	NM_011159	0.000131474	-1.31304	E11.5 * Sox10 down vs E11.5 * Ctrl
10487987	Lrrn4	NM_177303	0.00153679	-1.31345	E11.5 * Sox10 down vs E11.5 * Ctrl
10488090	Tasp1	NM_175225	0.00028856	-1.3139	E11.5 * Sox10 down vs E11.5 * Ctrl
10420362	Gjb2	NM_008125	0.00199984	-1.31395	E11.5 * Sox10 down vs E11.5 * Ctrl
10413100	Myst4	NM_017479	1.17342e-005	-1.31398	E11.5 * Sox10 down vs E11.5 * Ctrl
10555832	Olfr632	NM_147119	0.00564886	-1.314	E11.5 * Sox10 down vs E11.5 * Ctrl
10476941		---	0.0102063	-1.3141	E11.5 * Sox10 down vs E11.5 * Ctrl
10376513	Nlrp3	NM_145827	0.00552924	-1.3141	E11.5 * Sox10 down vs E11.5 * Ctrl
10576973	Col4a1	NM_009931	5.20365e-006	-1.31471	E11.5 * Sox10 down vs E11.5 * Ctrl
10356968	Pam	NM_013626	0.00147668	-1.31479	E11.5 * Sox10 down vs E11.5 * Ctrl
10510230	OTTMUSG00000	NM_001039209	0.000636543	-1.31492	E11.5 * Sox10 down vs E11.5 * Ctrl
10419744	Slc7a7	NM_011405	0.00736614	-1.31495	E11.5 * Sox10 down vs E11.5 * Ctrl
10579347	Ifi30	NM_023065	0.00239324	-1.31513	E11.5 * Sox10 down vs E11.5 * Ctrl
10504905	E130309F12Rik	NM_178756	0.00268858	-1.31565	E11.5 * Sox10 down vs E11.5 * Ctrl
10601412	Lpar4	NM_175271	4.67554e-005	-1.31567	E11.5 * Sox10 down vs E11.5 * Ctrl
10346348	2810022L02Rik	NM_144882	0.000822012	-1.31588	E11.5 * Sox10 down vs E11.5 * Ctrl
10566264	EG436003	NM_001127686	0.000398157	-1.31592	E11.5 * Sox10 down vs E11.5 * Ctrl
10445071	Zfp57	NM_001013745	6.93055e-005	-1.31615	E11.5 * Sox10 down vs E11.5 * Ctrl
10541910	Vwf	NM_011708	0.000250405	-1.31665	E11.5 * Sox10 down vs E11.5 * Ctrl
10380699	Copz2	NM_019877	0.00208685	-1.31685	E11.5 * Sox10 down vs E11.5 * Ctrl
10515242	Nsun4	NM_028142	0.00269242	-1.31694	E11.5 * Sox10 down vs E11.5 * Ctrl
10364529	Prtn3	NM_011178	0.00716534	-1.31699	E11.5 * Sox10 down vs E11.5 * Ctrl
10344725	Adhfe1	NM_175236	8.37942e-005	-1.31727	E11.5 * Sox10 down vs E11.5 * Ctrl
10350077	Syt2	NM_009307	0.000647379	-1.31748	E11.5 * Sox10 down vs E11.5 * Ctrl
10532711	Cmklr1	NM_008153	0.000932644	-1.31769	E11.5 * Sox10 down vs E11.5 * Ctrl
10544779	Hoxa7	NM_010455	7.48469e-005	-1.31783	E11.5 * Sox10 down vs E11.5 * Ctrl
10395277	Sypl	NM_013635	5.42922e-005	-1.31821	E11.5 * Sox10 down vs E11.5 * Ctrl
10344707	Pcmtd1	NM_183028	6.70376e-005	-1.31822	E11.5 * Sox10 down vs E11.5 * Ctrl
10414805	Trav13d-4	ENSMUST00000	0.00874003	-1.31828	E11.5 * Sox10 down vs E11.5 * Ctrl
10533034	Ccdc60	NM_177759	0.00308108	-1.3183	E11.5 * Sox10 down vs E11.5 * Ctrl
10480891	Ubac1	NM_133835	0.000474068	-1.31852	E11.5 * Sox10 down vs E11.5 * Ctrl
10560139	B430211C08Rik	NM_001039146	5.57068e-005	-1.31863	E11.5 * Sox10 down vs E11.5 * Ctrl
10436802		---	0.00919239	-1.3194	E11.5 * Sox10 down vs E11.5 * Ctrl
10544638	Tra2a	AB052758	0.00889697	-1.31949	E11.5 * Sox10 down vs E11.5 * Ctrl
10602925	Phka2	NM_172783	0.000550123	-1.31965	E11.5 * Sox10 down vs E11.5 * Ctrl
10445753	Trem3	NM_021407	0.0012052	-1.31981	E11.5 * Sox10 down vs E11.5 * Ctrl
10548905	Eps8	NM_007945	0.000155511	-1.3199	E11.5 * Sox10 down vs E11.5 * Ctrl
10477069	Tcf15	NM_009328	0.00199965	-1.31997	E11.5 * Sox10 down vs E11.5 * Ctrl

10582303	Cyba	NM_007806	0.0047028	-1.32	E11.5 * Sox10 down vs E11.5 * Ctrl
10498992	Tlr2	NM_011905	0.00317295	-1.32002	E11.5 * Sox10 down vs E11.5 * Ctrl
10447799	Igf2r	NM_010515	3.81544e-006	-1.32005	E11.5 * Sox10 down vs E11.5 * Ctrl
10584057	Zbtb44	NM_001115130	0.00104566	-1.32036	E11.5 * Sox10 down vs E11.5 * Ctrl
10531181	Adamts3	NM_001081401	0.00836887	-1.32036	E11.5 * Sox10 down vs E11.5 * Ctrl
10546803	Itpr1	NM_010585	0.00284667	-1.3209	E11.5 * Sox10 down vs E11.5 * Ctrl
10539773	Gfpt1	NM_013528	0.000898144	-1.32092	E11.5 * Sox10 down vs E11.5 * Ctrl
10409202		---	0.000227987	-1.32092	E11.5 * Sox10 down vs E11.5 * Ctrl
10378523	Smg6	NM_001002764	0.00055473	-1.32109	E11.5 * Sox10 down vs E11.5 * Ctrl
10356278	Sp110	NM_175397	0.000255818	-1.32127	E11.5 * Sox10 down vs E11.5 * Ctrl
10595480	Me1	NM_008615	0.0034217	-1.32135	E11.5 * Sox10 down vs E11.5 * Ctrl
10573939	Lpcat2	NM_173014	0.00200055	-1.32154	E11.5 * Sox10 down vs E11.5 * Ctrl
10361381	Syne1	NM_001079686	1.07249e-005	-1.32183	E11.5 * Sox10 down vs E11.5 * Ctrl
10560655	Bcam	NM_020486	0.000677745	-1.32189	E11.5 * Sox10 down vs E11.5 * Ctrl
10580191	Nfix	NM_001081981	0.000451941	-1.32227	E11.5 * Sox10 down vs E11.5 * Ctrl
10369264	Oit3	NM_010959	7.24088e-005	-1.32286	E11.5 * Sox10 down vs E11.5 * Ctrl
10591112	Fat3	NM_001080814	7.70909e-005	-1.32291	E11.5 * Sox10 down vs E11.5 * Ctrl
10357043	Bcl2	NM_009741	2.01931e-005	-1.32294	E11.5 * Sox10 down vs E11.5 * Ctrl
10568174	Spn	NM_009259	2.10617e-005	-1.32309	E11.5 * Sox10 down vs E11.5 * Ctrl
10566583	EG668139	AK172683	0.00370376	-1.32332	E11.5 * Sox10 down vs E11.5 * Ctrl
10492699	Ctso	NM_177662	0.000329514	-1.32356	E11.5 * Sox10 down vs E11.5 * Ctrl
10390103	Pdk2	NM_133667	0.000918098	-1.32366	E11.5 * Sox10 down vs E11.5 * Ctrl
10542221		---	0.000782989	-1.32371	E11.5 * Sox10 down vs E11.5 * Ctrl
10530393	Gabrg1	NM_010252	0.0008178	-1.32416	E11.5 * Sox10 down vs E11.5 * Ctrl
10410164	Cntnap3	NM_001081129	0.000290568	-1.32457	E11.5 * Sox10 down vs E11.5 * Ctrl
10436550		---	0.00670564	-1.32465	E11.5 * Sox10 down vs E11.5 * Ctrl
10491617	4932438A13Rik	NM_172679	0.00287929	-1.32481	E11.5 * Sox10 down vs E11.5 * Ctrl
10404359	Mboat1	NM_153546	0.00126277	-1.325	E11.5 * Sox10 down vs E11.5 * Ctrl
10578025		---	0.000806715	-1.32529	E11.5 * Sox10 down vs E11.5 * Ctrl
10414590	Ear6	NM_053111	0.00302979	-1.32555	E11.5 * Sox10 down vs E11.5 * Ctrl
10500570	Hao3	NM_019545	0.000283923	-1.32603	E11.5 * Sox10 down vs E11.5 * Ctrl
10573747	Adcy7	NM_007406	0.00274934	-1.32621	E11.5 * Sox10 down vs E11.5 * Ctrl
10594661	Tpm1	NM_024427	0.000136984	-1.32646	E11.5 * Sox10 down vs E11.5 * Ctrl
10466963	9930021J03Rik	NM_172836	0.00191702	-1.32649	E11.5 * Sox10 down vs E11.5 * Ctrl
10348244	Inpp5d	NM_010566	0.00113503	-1.32659	E11.5 * Sox10 down vs E11.5 * Ctrl
10512067	Ddx58	NM_172689	7.32515e-005	-1.32686	E11.5 * Sox10 down vs E11.5 * Ctrl
10559200	Tnni2	NM_009405	0.000178807	-1.32736	E11.5 * Sox10 down vs E11.5 * Ctrl
10607283	Maged2	NM_030700	0.00326342	-1.32781	E11.5 * Sox10 down vs E11.5 * Ctrl
10457407		---	7.35278e-006	-1.32805	E11.5 * Sox10 down vs E11.5 * Ctrl
10558596	Paox	NM_153783	0.00080716	-1.32854	E11.5 * Sox10 down vs E11.5 * Ctrl
10375886	D930048N14Rik	BC095931	4.19401e-005	-1.32855	E11.5 * Sox10 down vs E11.5 * Ctrl
10511588	Tmem67	NM_177861	0.00249567	-1.32916	E11.5 * Sox10 down vs E11.5 * Ctrl
10553354	Nav2	NM_175272	0.000116914	-1.32949	E11.5 * Sox10 down vs E11.5 * Ctrl
10455961	Iigp1	NM_001146275	0.000424555	-1.32964	E11.5 * Sox10 down vs E11.5 * Ctrl
10433114	Itga5	NM_010577	0.00932068	-1.32996	E11.5 * Sox10 down vs E11.5 * Ctrl
10497862	Trpc3	NM_019510	0.00100702	-1.33006	E11.5 * Sox10 down vs E11.5 * Ctrl
10407416	Calml3	NM_027416	0.00500951	-1.33018	E11.5 * Sox10 down vs E11.5 * Ctrl
10584762	Bcl9l	NM_030256	0.000354653	-1.33027	E11.5 * Sox10 down vs E11.5 * Ctrl
10382228	Axin2	NM_015732	1.60458e-005	-1.33046	E11.5 * Sox10 down vs E11.5 * Ctrl
10403727	Gli3	NM_008130	0.00101662	-1.33091	E11.5 * Sox10 down vs E11.5 * Ctrl
10540408	Itpr1	NM_010585	0.000122824	-1.33167	E11.5 * Sox10 down vs E11.5 * Ctrl
10604333	Wdr40b	NM_178739	0.00101762	-1.33186	E11.5 * Sox10 down vs E11.5 * Ctrl
10467871	Dnmbp	NM_028029	0.00560006	-1.33263	E11.5 * Sox10 down vs E11.5 * Ctrl
10578681		---	0.00022434	-1.33345	E11.5 * Sox10 down vs E11.5 * Ctrl
10362432	Trdn	NM_029726	0.00183143	-1.33347	E11.5 * Sox10 down vs E11.5 * Ctrl
10422348	Ugcgl2	NM_001081252	0.00239746	-1.33362	E11.5 * Sox10 down vs E11.5 * Ctrl
10519555	Abcb1b	NM_011075	0.00909313	-1.3337	E11.5 * Sox10 down vs E11.5 * Ctrl
10472514	Nostrin	NM_181547	0.00516437	-1.33407	E11.5 * Sox10 down vs E11.5 * Ctrl
10476924	Cst9	NM_009979	0.000590042	-1.33442	E11.5 * Sox10 down vs E11.5 * Ctrl
10351482	1700015E13Rik	BC099563	0.000366113	-1.33457	E11.5 * Sox10 down vs E11.5 * Ctrl
10514000	Mpdz	NM_010820	1.47151e-005	-1.33499	E11.5 * Sox10 down vs E11.5 * Ctrl
10568714	Mki67	NM_001081117	1.63083e-005	-1.33558	E11.5 * Sox10 down vs E11.5 * Ctrl
10538848	OTTMUSG00000	XR_032708	0.00154699	-1.33571	E11.5 * Sox10 down vs E11.5 * Ctrl
10495763	Gclm	NM_008129	0.00864269	-1.33578	E11.5 * Sox10 down vs E11.5 * Ctrl
10433658		---	0.000580377	-1.33611	E11.5 * Sox10 down vs E11.5 * Ctrl
10394829		---	0.000131601	-1.33661	E11.5 * Sox10 down vs E11.5 * Ctrl

10594103	2410076I21Rik	ENSMUST00000(	0.000238454	-1.33666	E11.5 * Sox10 down vs E11.5 * Ctrl
10594969	Unc13c	NM_001081153	3.64888e-005	-1.33669	E11.5 * Sox10 down vs E11.5 * Ctrl
10490384	Lama5	NM_001081171	5.12292e-005	-1.33703	E11.5 * Sox10 down vs E11.5 * Ctrl
10525932	Tmem132c	NM_175432	0.00016894	-1.33713	E11.5 * Sox10 down vs E11.5 * Ctrl
10433639	Mkl2	NM_153588	2.62733e-006	-1.33736	E11.5 * Sox10 down vs E11.5 * Ctrl
10357003	Rnf152	NM_178779	0.00863029	-1.33745	E11.5 * Sox10 down vs E11.5 * Ctrl
10592535	Sorl1	NM_011436	2.36153e-005	-1.33758	E11.5 * Sox10 down vs E11.5 * Ctrl
10560329	Hif3a	NM_016868	0.000398069	-1.33776	E11.5 * Sox10 down vs E11.5 * Ctrl
10365420	AI597468	NM_001013028	9.64985e-006	-1.33788	E11.5 * Sox10 down vs E11.5 * Ctrl
10596465	Acy1	NM_025371	1.174e-005	-1.33788	E11.5 * Sox10 down vs E11.5 * Ctrl
10570639	6820431F20Rik	BC025151	0.000858652	-1.33789	E11.5 * Sox10 down vs E11.5 * Ctrl
10507484	Eri3	NM_080469	0.00322362	-1.33813	E11.5 * Sox10 down vs E11.5 * Ctrl
10586110	Cln6	NM_001033175	3.88873e-005	-1.33854	E11.5 * Sox10 down vs E11.5 * Ctrl
10587023	Rab27a	NM_023635	2.03126e-005	-1.33867	E11.5 * Sox10 down vs E11.5 * Ctrl
10390117	Itga3	NM_013565	0.00106655	-1.33947	E11.5 * Sox10 down vs E11.5 * Ctrl
10600093	Zfp185	NM_009549	7.88981e-005	-1.33948	E11.5 * Sox10 down vs E11.5 * Ctrl
10514421	Lrrc19	NM_175305	0.00760706	-1.33998	E11.5 * Sox10 down vs E11.5 * Ctrl
10563872	Gabra5	NM_176942	0.00929606	-1.34061	E11.5 * Sox10 down vs E11.5 * Ctrl
10552469	Klk13	NM_001039042	1.41207e-005	-1.34083	E11.5 * Sox10 down vs E11.5 * Ctrl
10605392	F8	NM_007977	0.00273614	-1.34093	E11.5 * Sox10 down vs E11.5 * Ctrl
10607189	Amot	NM_153319	0.00357113	-1.34105	E11.5 * Sox10 down vs E11.5 * Ctrl
10375578	Flt4	NM_008029	0.000799149	-1.34115	E11.5 * Sox10 down vs E11.5 * Ctrl
10557009	Eef2k	NM_007908	1.23981e-005	-1.34139	E11.5 * Sox10 down vs E11.5 * Ctrl
10382200	Ccdc46	NM_029606	8.25853e-005	-1.34185	E11.5 * Sox10 down vs E11.5 * Ctrl
10448124	Fpr1	NM_013521	0.00166583	-1.34224	E11.5 * Sox10 down vs E11.5 * Ctrl
10416689	Olfm4	NM_001030294	0.00775404	-1.34254	E11.5 * Sox10 down vs E11.5 * Ctrl
10596428	1110001D15Rik	NM_026763	0.00287638	-1.34256	E11.5 * Sox10 down vs E11.5 * Ctrl
10363090	Rfx6	NM_001159389	0.000752198	-1.34278	E11.5 * Sox10 down vs E11.5 * Ctrl
10548899	Rerg	NM_181988	0.00431052	-1.3428	E11.5 * Sox10 down vs E11.5 * Ctrl
10410115	1110018J18Rik	NM_025370	0.00344907	-1.34287	E11.5 * Sox10 down vs E11.5 * Ctrl
10591094	Fat3	NM_001080814	0.000760221	-1.34292	E11.5 * Sox10 down vs E11.5 * Ctrl
10568355	Pycard	NM_023258	0.00405528	-1.34302	E11.5 * Sox10 down vs E11.5 * Ctrl
10556244	Ipo7	AF357383	0.00267989	-1.34385	E11.5 * Sox10 down vs E11.5 * Ctrl
10387368	Tmem88	NM_025915	0.000263974	-1.34447	E11.5 * Sox10 down vs E11.5 * Ctrl
10474381	Kif18a	NM_139303	0.000697022	-1.34458	E11.5 * Sox10 down vs E11.5 * Ctrl
10526098	Scand3	NM_183088	0.00135743	-1.34475	E11.5 * Sox10 down vs E11.5 * Ctrl
10346695	Nbeal1	NM_173444	9.62558e-006	-1.3448	E11.5 * Sox10 down vs E11.5 * Ctrl
10409629	Klhl3	ENSMUST00000(	0.000870883	-1.34502	E11.5 * Sox10 down vs E11.5 * Ctrl
10607169	Trpc5	NM_009428	0.00152535	-1.3453	E11.5 * Sox10 down vs E11.5 * Ctrl
10432178	Snora2b	AF357413	0.00364589	-1.34536	E11.5 * Sox10 down vs E11.5 * Ctrl
10383214	Rnf213	AK173199	0.000115982	-1.34555	E11.5 * Sox10 down vs E11.5 * Ctrl
10562729		---	2.53548e-005	-1.34596	E11.5 * Sox10 down vs E11.5 * Ctrl
10346107	Dnahc7b	ENSMUST00000(	0.00294282	-1.34637	E11.5 * Sox10 down vs E11.5 * Ctrl
10500434	Bcl9	NM_029933	5.29593e-005	-1.34677	E11.5 * Sox10 down vs E11.5 * Ctrl
10454632	Camk4	NM_009793	0.00575434	-1.34812	E11.5 * Sox10 down vs E11.5 * Ctrl
10472042	OTTMUSG00000	AF390105	0.000503633	-1.34815	E11.5 * Sox10 down vs E11.5 * Ctrl
10534889	Agfg2	NM_178162	1.8583e-006	-1.34911	E11.5 * Sox10 down vs E11.5 * Ctrl
10598538	Ssxb1	NM_026492	0.00126153	-1.34921	E11.5 * Sox10 down vs E11.5 * Ctrl
10355871	Obsl1	NM_178884	0.000572525	-1.35023	E11.5 * Sox10 down vs E11.5 * Ctrl
10476945	Cst7	NM_009977	0.00190775	-1.35048	E11.5 * Sox10 down vs E11.5 * Ctrl
10559221	Tnnt3	NM_011620	0.00373511	-1.35063	E11.5 * Sox10 down vs E11.5 * Ctrl
10604932	Cd99l2	NM_138309	0.00377029	-1.35136	E11.5 * Sox10 down vs E11.5 * Ctrl
10461384		---	0.0046573	-1.35138	E11.5 * Sox10 down vs E11.5 * Ctrl
10583163	Trpc6	NM_013838	0.000347397	-1.35145	E11.5 * Sox10 down vs E11.5 * Ctrl
10357008	Pign	NM_013784	6.07822e-006	-1.35208	E11.5 * Sox10 down vs E11.5 * Ctrl
10424252	Wdyhv1	NM_029734	0.00380816	-1.35208	E11.5 * Sox10 down vs E11.5 * Ctrl
10394805	5730507C01Rik	ENSMUST00000(	0.000339854	-1.35242	E11.5 * Sox10 down vs E11.5 * Ctrl
10549530		---	0.00794279	-1.35254	E11.5 * Sox10 down vs E11.5 * Ctrl
10485213	Cd82	NM_007656	0.000626031	-1.35283	E11.5 * Sox10 down vs E11.5 * Ctrl
10453629	9430020K01Rik	NM_001081963	0.000407543	-1.35292	E11.5 * Sox10 down vs E11.5 * Ctrl
10531994	Mpa2l	NM_194336	0.000110406	-1.35295	E11.5 * Sox10 down vs E11.5 * Ctrl
10548875	Art4	NM_026639	8.9726e-005	-1.3534	E11.5 * Sox10 down vs E11.5 * Ctrl
10554800	Rab38	NM_028238	2.31759e-006	-1.35349	E11.5 * Sox10 down vs E11.5 * Ctrl
10363082	Lilrb4	NM_013532	0.000385779	-1.35362	E11.5 * Sox10 down vs E11.5 * Ctrl
10429402	Gpr20	NM_173365	0.000447861	-1.35377	E11.5 * Sox10 down vs E11.5 * Ctrl
10502785	ENSMUSG0000C	ENSMUST00000(	0.00492245	-1.35386	E11.5 * Sox10 down vs E11.5 * Ctrl

10557229	LOC100134980	BC016578	0.00016809	-1.3539	E11.5 * Sox10 down vs E11.5 * Ctrl
10388902	Lgals9	NM_010708	0.000642762	-1.35396	E11.5 * Sox10 down vs E11.5 * Ctrl
10360090	Ppox	NM_008911	0.000232626	-1.35408	E11.5 * Sox10 down vs E11.5 * Ctrl
10504849	Stx17	NM_026343	0.00165144	-1.35416	E11.5 * Sox10 down vs E11.5 * Ctrl
10399965	F730043M19Rik	ENSMUST00000(	0.00366653	-1.35419	E11.5 * Sox10 down vs E11.5 * Ctrl
10346722	Nbeal1	NM_173444	0.000445082	-1.35465	E11.5 * Sox10 down vs E11.5 * Ctrl
10497731	Ccdc39	NM_026222	0.000987262	-1.35479	E11.5 * Sox10 down vs E11.5 * Ctrl
10474048	Syt13	NM_030725	1.63147e-006	-1.35511	E11.5 * Sox10 down vs E11.5 * Ctrl
10530592	Fryl	NM_177136	0.000289825	-1.35578	E11.5 * Sox10 down vs E11.5 * Ctrl
10461408	Rab3il1	NM_144538	0.000109923	-1.35615	E11.5 * Sox10 down vs E11.5 * Ctrl
10398332		---	1.32749e-005	-1.35625	E11.5 * Sox10 down vs E11.5 * Ctrl
10396606	Syne2	NM_001005510	0.00208849	-1.35628	E11.5 * Sox10 down vs E11.5 * Ctrl
10512022	Mobkl2b	ENSMUST00000(	0.0100811	-1.35628	E11.5 * Sox10 down vs E11.5 * Ctrl
10603247		---	0.000856553	-1.35649	E11.5 * Sox10 down vs E11.5 * Ctrl
10382435	Gprc5c	NM_001110337	0.000400628	-1.35706	E11.5 * Sox10 down vs E11.5 * Ctrl
10413943	Lrrc18	NM_026253	1.33712e-005	-1.35716	E11.5 * Sox10 down vs E11.5 * Ctrl
10483246		---	0.00106754	-1.35723	E11.5 * Sox10 down vs E11.5 * Ctrl
10403748		---	4.72503e-005	-1.35731	E11.5 * Sox10 down vs E11.5 * Ctrl
10569456		---	0.000730085	-1.35829	E11.5 * Sox10 down vs E11.5 * Ctrl
10426437		---	0.00430471	-1.35838	E11.5 * Sox10 down vs E11.5 * Ctrl
10459288	Adrb2	NM_007420	0.00909152	-1.35845	E11.5 * Sox10 down vs E11.5 * Ctrl
10491629	4932438A13Rik	NM_172679	0.000179407	-1.35846	E11.5 * Sox10 down vs E11.5 * Ctrl
10381601	Grn	M86736	0.000462674	-1.35852	E11.5 * Sox10 down vs E11.5 * Ctrl
10438749		---	0.00194313	-1.35868	E11.5 * Sox10 down vs E11.5 * Ctrl
10439268	Dtx3l	NM_001013371	0.00288943	-1.35895	E11.5 * Sox10 down vs E11.5 * Ctrl
10553299	Ifitm2	NM_030694	0.00754957	-1.35903	E11.5 * Sox10 down vs E11.5 * Ctrl
10530692	Kdr	NM_010612	0.000972655	-1.35921	E11.5 * Sox10 down vs E11.5 * Ctrl
10468746	Hspa12a	NM_175199	0.000544966	-1.35923	E11.5 * Sox10 down vs E11.5 * Ctrl
10377534	A030009H04Rik	AB041807	9.03632e-005	-1.35954	E11.5 * Sox10 down vs E11.5 * Ctrl
10584067	Zbtb44	NM_001115130	2.44205e-005	-1.35954	E11.5 * Sox10 down vs E11.5 * Ctrl
10428753		---	0.000171134	-1.35981	E11.5 * Sox10 down vs E11.5 * Ctrl
10490903	Car13	NM_024495	0.00457447	-1.35995	E11.5 * Sox10 down vs E11.5 * Ctrl
10470283	Egfl7	NM_198724	0.000357801	-1.3602	E11.5 * Sox10 down vs E11.5 * Ctrl
10356170		---	0.00146454	-1.36021	E11.5 * Sox10 down vs E11.5 * Ctrl
10379389	Adap2	NM_172133	0.000276749	-1.3603	E11.5 * Sox10 down vs E11.5 * Ctrl
10543494	Grm8	NM_008174	0.00915009	-1.36031	E11.5 * Sox10 down vs E11.5 * Ctrl
10516221	D130007C19Rik	AK051152	0.000150251	-1.36035	E11.5 * Sox10 down vs E11.5 * Ctrl
10430834	Naga	NM_008669	0.00487712	-1.36058	E11.5 * Sox10 down vs E11.5 * Ctrl
10602722	Spin2	NM_001005370	0.00141448	-1.36072	E11.5 * Sox10 down vs E11.5 * Ctrl
10435457	Parp9	NM_030253	0.000371843	-1.36075	E11.5 * Sox10 down vs E11.5 * Ctrl
10379944	Tbx4	NM_011536	0.00220268	-1.36088	E11.5 * Sox10 down vs E11.5 * Ctrl
10505517	Tlr4	NM_021297	0.000396196	-1.36154	E11.5 * Sox10 down vs E11.5 * Ctrl
10456745	Smad7	NM_001042660	0.000507912	-1.36164	E11.5 * Sox10 down vs E11.5 * Ctrl
10504106	Il11ra1	NM_010549	0.000143866	-1.36185	E11.5 * Sox10 down vs E11.5 * Ctrl
10556302	Ampd3	NM_009667	0.00504942	-1.36244	E11.5 * Sox10 down vs E11.5 * Ctrl
10568001	Sult1a1	NM_133670	0.000434304	-1.36273	E11.5 * Sox10 down vs E11.5 * Ctrl
10378216	Atp2a3	NM_016745	3.05219e-005	-1.36292	E11.5 * Sox10 down vs E11.5 * Ctrl
10560190	Ehd2	NM_153068	0.000605586	-1.363	E11.5 * Sox10 down vs E11.5 * Ctrl
10590690	Dync2h1	NM_029851	2.62631e-006	-1.36317	E11.5 * Sox10 down vs E11.5 * Ctrl
10391404	D830013H23Rik	AK052868	0.00375629	-1.3632	E11.5 * Sox10 down vs E11.5 * Ctrl
10395103	Pxdn	NM_181395	0.000149991	-1.36339	E11.5 * Sox10 down vs E11.5 * Ctrl
10447130	Pkdcc	NM_134117	0.000185763	-1.36339	E11.5 * Sox10 down vs E11.5 * Ctrl
10369688	Tet1	ENSMUST00000(	0.00223889	-1.36376	E11.5 * Sox10 down vs E11.5 * Ctrl
10424731	Gsdmd	NM_026960	0.00750803	-1.3639	E11.5 * Sox10 down vs E11.5 * Ctrl
10400510	Clec14a	NM_025809	0.0103661	-1.36416	E11.5 * Sox10 down vs E11.5 * Ctrl
10370037	Mmp11	NM_008606	0.0020476	-1.36448	E11.5 * Sox10 down vs E11.5 * Ctrl
10528008	Steap2	NM_001103157	0.00121523	-1.36449	E11.5 * Sox10 down vs E11.5 * Ctrl
10363786	Ank3	NM_146005	0.000503011	-1.36453	E11.5 * Sox10 down vs E11.5 * Ctrl
10488299	OTTMUSG00000	NM_001037929	0.000669096	-1.36461	E11.5 * Sox10 down vs E11.5 * Ctrl
10432408	C1ql4	NM_001024702	0.00362722	-1.36482	E11.5 * Sox10 down vs E11.5 * Ctrl
10532517	Cryba4	NM_021351	0.00454875	-1.36506	E11.5 * Sox10 down vs E11.5 * Ctrl
10462507	Papss2	NM_011864	0.000268099	-1.36516	E11.5 * Sox10 down vs E11.5 * Ctrl
10346564	Casp8	NM_009812	0.00514749	-1.36522	E11.5 * Sox10 down vs E11.5 * Ctrl
10406205	Erap1	NM_030711	0.00568769	-1.36603	E11.5 * Sox10 down vs E11.5 * Ctrl
10492310	Mbnl1	NM_020007	2.96399e-006	-1.36608	E11.5 * Sox10 down vs E11.5 * Ctrl
10492864	Sh3d19	NM_001082414	5.3203e-005	-1.36619	E11.5 * Sox10 down vs E11.5 * Ctrl

10376074	P4ha2	NM_001136076	0.0100576	-1.36632	E11.5 * Sox10 down vs E11.5 * Ctrl
10552500	Klk8	NM_008940	0.000375454	-1.36642	E11.5 * Sox10 down vs E11.5 * Ctrl
10531790	Nkx6-1	NM_144955	6.29765e-005	-1.36647	E11.5 * Sox10 down vs E11.5 * Ctrl
10503315	E130016E03Rik	NM_001039556	0.000203298	-1.36669	E11.5 * Sox10 down vs E11.5 * Ctrl
10353311	Jph1	NM_020604	0.00238666	-1.36672	E11.5 * Sox10 down vs E11.5 * Ctrl
10511789	Nkain3	NM_172987	0.000455819	-1.36688	E11.5 * Sox10 down vs E11.5 * Ctrl
10579765		---	0.000256006	-1.36705	E11.5 * Sox10 down vs E11.5 * Ctrl
10552358	I1C0022H11Rik	NM_001034893	0.000219462	-1.36756	E11.5 * Sox10 down vs E11.5 * Ctrl
10597945	Tmem158	NM_001002267	0.00017442	-1.36769	E11.5 * Sox10 down vs E11.5 * Ctrl
10454398	AW554918	NM_001033532	0.00129001	-1.36776	E11.5 * Sox10 down vs E11.5 * Ctrl
10358982	Mr1	NM_008209	0.00526315	-1.36789	E11.5 * Sox10 down vs E11.5 * Ctrl
10546421	Prickle2	NM_001134461	0.00123788	-1.36835	E11.5 * Sox10 down vs E11.5 * Ctrl
10392683	ENSMUSG0000C	ENSMUST00000	0.000315727	-1.36934	E11.5 * Sox10 down vs E11.5 * Ctrl
10467013	Prkg1	NM_001013833	0.000179075	-1.36941	E11.5 * Sox10 down vs E11.5 * Ctrl
10514193		---	0.000398337	-1.36969	E11.5 * Sox10 down vs E11.5 * Ctrl
10564667	Ntrk3	NM_008746	0.0101812	-1.36985	E11.5 * Sox10 down vs E11.5 * Ctrl
10604135	Dppa3	NM_139218	0.000318162	-1.37069	E11.5 * Sox10 down vs E11.5 * Ctrl
10576581	Kcnk1	NM_008430	0.00648623	-1.37085	E11.5 * Sox10 down vs E11.5 * Ctrl
10602756	Smpx	NM_025357	0.000696766	-1.371	E11.5 * Sox10 down vs E11.5 * Ctrl
10514779	Prkaa2	NM_178143	0.000863605	-1.37122	E11.5 * Sox10 down vs E11.5 * Ctrl
10597279	Ccrl2	NM_017466	0.00661361	-1.37138	E11.5 * Sox10 down vs E11.5 * Ctrl
10584589		---	0.00160359	-1.37202	E11.5 * Sox10 down vs E11.5 * Ctrl
10436788	Hunk	NM_015755	0.000164064	-1.37202	E11.5 * Sox10 down vs E11.5 * Ctrl
10391119	1110036O03Rik	NM_176830	2.16856e-006	-1.37209	E11.5 * Sox10 down vs E11.5 * Ctrl
10578690	Neil3	NM_146208	7.52305e-005	-1.37281	E11.5 * Sox10 down vs E11.5 * Ctrl
10431943	100043354	XR_033584	0.00504134	-1.37293	E11.5 * Sox10 down vs E11.5 * Ctrl
10400708		---	0.000297457	-1.373	E11.5 * Sox10 down vs E11.5 * Ctrl
10498647	B3galnt1	NM_020026	1.03892e-006	-1.37313	E11.5 * Sox10 down vs E11.5 * Ctrl
10575534	St3gal2	NM_009179	4.16178e-006	-1.37329	E11.5 * Sox10 down vs E11.5 * Ctrl
10353947	Tmem131	NM_018872	3.84513e-008	-1.37347	E11.5 * Sox10 down vs E11.5 * Ctrl
10548752		---	0.00266594	-1.37402	E11.5 * Sox10 down vs E11.5 * Ctrl
10457225	Map3k8	NM_007746	0.000488749	-1.37406	E11.5 * Sox10 down vs E11.5 * Ctrl
10421418	Epb4.9	NM_013514	1.62274e-005	-1.37416	E11.5 * Sox10 down vs E11.5 * Ctrl
10463476	Kazald1	NM_178929	0.00218482	-1.37465	E11.5 * Sox10 down vs E11.5 * Ctrl
10566543	Dchs1	ENSMUST00000	0.00552203	-1.37471	E11.5 * Sox10 down vs E11.5 * Ctrl
10489204	Tgm2	NM_009373	0.000160786	-1.37476	E11.5 * Sox10 down vs E11.5 * Ctrl
10379630	Slfn2	NM_011408	0.00977238	-1.3748	E11.5 * Sox10 down vs E11.5 * Ctrl
10404612	Rreb1	NM_001013392	0.000996093	-1.37536	E11.5 * Sox10 down vs E11.5 * Ctrl
10445542		---	0.00373531	-1.37553	E11.5 * Sox10 down vs E11.5 * Ctrl
10581992	Maf	NM_001025577	0.00231147	-1.37603	E11.5 * Sox10 down vs E11.5 * Ctrl
10398382		---	0.000122946	-1.37764	E11.5 * Sox10 down vs E11.5 * Ctrl
10368199	Myb	NM_010848	0.000179027	-1.37802	E11.5 * Sox10 down vs E11.5 * Ctrl
10516481	Gja4	NM_008120	0.0001477	-1.37831	E11.5 * Sox10 down vs E11.5 * Ctrl
10552363	I1C0022H11Rik	NM_001034893	0.00010484	-1.37887	E11.5 * Sox10 down vs E11.5 * Ctrl
10381408	Ifi35	NM_027320	8.36798e-005	-1.37921	E11.5 * Sox10 down vs E11.5 * Ctrl
10410173	Hiatl1	NM_133680	0.00589842	-1.37922	E11.5 * Sox10 down vs E11.5 * Ctrl
10408185	Btn2a2	NM_175938	2.9549e-005	-1.37933	E11.5 * Sox10 down vs E11.5 * Ctrl
10606362		---	0.000139874	-1.37963	E11.5 * Sox10 down vs E11.5 * Ctrl
10443027	A930001N09Rik	NM_029870	6.43592e-005	-1.37996	E11.5 * Sox10 down vs E11.5 * Ctrl
10375137	Kcnmb1	NM_031169	0.00207742	-1.38003	E11.5 * Sox10 down vs E11.5 * Ctrl
10473809	Sfpi1	NM_011355	1.44401e-005	-1.38016	E11.5 * Sox10 down vs E11.5 * Ctrl
10391504	Meox1	NM_010791	0.00413326	-1.38021	E11.5 * Sox10 down vs E11.5 * Ctrl
10375229		---	0.00722698	-1.38042	E11.5 * Sox10 down vs E11.5 * Ctrl
10528332	Napepld	NM_178728	3.62806e-006	-1.38061	E11.5 * Sox10 down vs E11.5 * Ctrl
10387536	Cd68	NM_009853	0.00380741	-1.38065	E11.5 * Sox10 down vs E11.5 * Ctrl
10427095	Tenc1	NM_153533	0.000132765	-1.38081	E11.5 * Sox10 down vs E11.5 * Ctrl
10367475		---	4.84582e-005	-1.3814	E11.5 * Sox10 down vs E11.5 * Ctrl
10596072	3222402P14Rik	BC151170	7.58794e-005	-1.38145	E11.5 * Sox10 down vs E11.5 * Ctrl
10472860	Rapgef4	NM_019688	0.000809957	-1.38163	E11.5 * Sox10 down vs E11.5 * Ctrl
10359377	Zbtb37	NM_173424	6.25348e-006	-1.38184	E11.5 * Sox10 down vs E11.5 * Ctrl
10412394	Nnt	NR_003544	5.07419e-005	-1.38219	E11.5 * Sox10 down vs E11.5 * Ctrl
10390032	Acsf2	NM_153807	0.00201792	-1.38268	E11.5 * Sox10 down vs E11.5 * Ctrl
10351347	Creg1	NM_011804	0.000471453	-1.38274	E11.5 * Sox10 down vs E11.5 * Ctrl
10440186	Crybg3	NM_174848	0.00295721	-1.38471	E11.5 * Sox10 down vs E11.5 * Ctrl
10436608	Cxadr	NM_009988	1.16598e-005	-1.38492	E11.5 * Sox10 down vs E11.5 * Ctrl
10603567	Dynlt3	NM_025975	0.00205539	-1.38509	E11.5 * Sox10 down vs E11.5 * Ctrl

10580056	Lphn1	NM_181039	0.000141642	-1.38544	E11.5 * Sox10 down vs E11.5 * Ctrl
10549276	Bhlhe41	NM_024469	1.416e-005	-1.3855	E11.5 * Sox10 down vs E11.5 * Ctrl
10485070	Mdk	NM_010784	0.000271203	-1.38591	E11.5 * Sox10 down vs E11.5 * Ctrl
10460968	Rasgrp2	NM_011242	1.58133e-005	-1.38631	E11.5 * Sox10 down vs E11.5 * Ctrl
10371740	Ano4	NM_178773	0.000263548	-1.38671	E11.5 * Sox10 down vs E11.5 * Ctrl
10476740	Slc24a3	NM_053195	0.00221709	-1.38673	E11.5 * Sox10 down vs E11.5 * Ctrl
10605616	Il1rapl1	BC119580	0.00263047	-1.38732	E11.5 * Sox10 down vs E11.5 * Ctrl
10598603			9.18013e-005	-1.38744	E11.5 * Sox10 down vs E11.5 * Ctrl
10557033	Eef2k	ENSMUST00000(	0.000803918	-1.38766	E11.5 * Sox10 down vs E11.5 * Ctrl
10399820	Acp1	NM_001110239	0.00143566	-1.38789	E11.5 * Sox10 down vs E11.5 * Ctrl
10544660	Osbpl3	NM_027881	1.51317e-005	-1.3879	E11.5 * Sox10 down vs E11.5 * Ctrl
10517967	Fblim1	NM_133754	0.00133941	-1.38834	E11.5 * Sox10 down vs E11.5 * Ctrl
10558410	Ptpre	NM_011212	0.00140063	-1.38872	E11.5 * Sox10 down vs E11.5 * Ctrl
10462195	Kank1	NM_181404	0.000197848	-1.38895	E11.5 * Sox10 down vs E11.5 * Ctrl
10372208	Acss3	NM_001142804	0.000606859	-1.38899	E11.5 * Sox10 down vs E11.5 * Ctrl
10599736	Fhl1	NM_001077361	3.10479e-006	-1.38923	E11.5 * Sox10 down vs E11.5 * Ctrl
10563858	Gabrg3	NM_008074	0.00484358	-1.38945	E11.5 * Sox10 down vs E11.5 * Ctrl
10371379	Nuak1	NM_001004363	0.000826783	-1.38946	E11.5 * Sox10 down vs E11.5 * Ctrl
10494565	Fmo5	NM_010232	0.000104911	-1.38965	E11.5 * Sox10 down vs E11.5 * Ctrl
10543952	9330158H04Rik	ENSMUST00000	0.00137235	-1.39015	E11.5 * Sox10 down vs E11.5 * Ctrl
10394938		---	0.000633685	-1.39018	E11.5 * Sox10 down vs E11.5 * Ctrl
10399657		---	0.000633685	-1.39018	E11.5 * Sox10 down vs E11.5 * Ctrl
10567995	Nupr1	NM_019738	0.000113415	-1.3906	E11.5 * Sox10 down vs E11.5 * Ctrl
10492402	Kcnab1	NM_010597	0.000107159	-1.39084	E11.5 * Sox10 down vs E11.5 * Ctrl
10511446	Asph	NM_023066	7.94966e-006	-1.3914	E11.5 * Sox10 down vs E11.5 * Ctrl
10561104	Axl	NM_009465	0.00053643	-1.3916	E11.5 * Sox10 down vs E11.5 * Ctrl
10525487	4932422M17Rik	ENSMUST00000(	0.00251801	-1.39203	E11.5 * Sox10 down vs E11.5 * Ctrl
10377662	Ybx2	NM_016875	0.000111006	-1.39224	E11.5 * Sox10 down vs E11.5 * Ctrl
10344815	Cspp1	NM_026493	0.0022856	-1.39232	E11.5 * Sox10 down vs E11.5 * Ctrl
10363856	2310015B20Rik	ENSMUST00000(	0.00600946	-1.39271	E11.5 * Sox10 down vs E11.5 * Ctrl
10551169	B3gnt8	NM_146184	0.00756905	-1.39296	E11.5 * Sox10 down vs E11.5 * Ctrl
10439830		---	0.0038549	-1.39302	E11.5 * Sox10 down vs E11.5 * Ctrl
10531952	Abcg3	NM_030239	0.000293999	-1.39321	E11.5 * Sox10 down vs E11.5 * Ctrl
10346072		---	0.000159286	-1.39439	E11.5 * Sox10 down vs E11.5 * Ctrl
10413047	Plau	NM_008873	0.00618818	-1.39444	E11.5 * Sox10 down vs E11.5 * Ctrl
10472982		---	0.00636867	-1.39449	E11.5 * Sox10 down vs E11.5 * Ctrl
10550877	Kcnn4	NM_008433	1.7438e-005	-1.39479	E11.5 * Sox10 down vs E11.5 * Ctrl
10448094	Lnpep	NM_172827	0.000191516	-1.39494	E11.5 * Sox10 down vs E11.5 * Ctrl
10376201	Gpx3	NM_001083929	6.74613e-005	-1.39505	E11.5 * Sox10 down vs E11.5 * Ctrl
10410408	Adcy2	NM_153534	0.002238	-1.39549	E11.5 * Sox10 down vs E11.5 * Ctrl
10395287	Atxn7l1	NM_028139	2.24087e-006	-1.39574	E11.5 * Sox10 down vs E11.5 * Ctrl
10428511	Csmd3	NM_001081391	0.00609481	-1.39591	E11.5 * Sox10 down vs E11.5 * Ctrl
10372796	Hmga2	NM_010441	0.000140417	-1.39624	E11.5 * Sox10 down vs E11.5 * Ctrl
10564539	Mctp2	NM_001024703	0.000271448	-1.39642	E11.5 * Sox10 down vs E11.5 * Ctrl
10361509	Syne1	NM_001079686	1.73123e-005	-1.39647	E11.5 * Sox10 down vs E11.5 * Ctrl
10383756	Ifitm2	NM_030694	0.00760594	-1.39659	E11.5 * Sox10 down vs E11.5 * Ctrl
10499914	Lce1b	NM_026822	0.00318399	-1.39659	E11.5 * Sox10 down vs E11.5 * Ctrl
10568638	Uros	NM_009479	2.54854e-006	-1.3969	E11.5 * Sox10 down vs E11.5 * Ctrl
10463911	Add3	NM_013758	8.28387e-005	-1.39706	E11.5 * Sox10 down vs E11.5 * Ctrl
10595140		---	0.00312144	-1.39761	E11.5 * Sox10 down vs E11.5 * Ctrl
10591092	Fat3	NM_001080814	0.000137603	-1.3978	E11.5 * Sox10 down vs E11.5 * Ctrl
10570002	EG546032	XM_486078	0.00116328	-1.39793	E11.5 * Sox10 down vs E11.5 * Ctrl
10382321	Kcnj2	NM_008425	0.00473852	-1.39803	E11.5 * Sox10 down vs E11.5 * Ctrl
10587616	Prss35	NM_178738	0.0043341	-1.39896	E11.5 * Sox10 down vs E11.5 * Ctrl
10504127	Ccl21a	NM_011124	0.000495203	-1.39906	E11.5 * Sox10 down vs E11.5 * Ctrl
10504154	Ccl21a	NM_011124	0.000495203	-1.39906	E11.5 * Sox10 down vs E11.5 * Ctrl
10504183	Ccl21a	NM_011124	0.000495203	-1.39906	E11.5 * Sox10 down vs E11.5 * Ctrl
10512377	Ccl21a	NM_011124	0.000495203	-1.39906	E11.5 * Sox10 down vs E11.5 * Ctrl
10366886	Arhgap9	NM_146011	0.00015644	-1.39921	E11.5 * Sox10 down vs E11.5 * Ctrl
10490491	Gata5	NM_008093	6.08492e-006	-1.39948	E11.5 * Sox10 down vs E11.5 * Ctrl
10510129	Dhrs3	NM_011303	0.00129027	-1.39989	E11.5 * Sox10 down vs E11.5 * Ctrl
10420171	Adcy4	NM_080435	0.00375826	-1.40005	E11.5 * Sox10 down vs E11.5 * Ctrl
10388488	Fam101b	ENSMUST00000(	0.000508258	-1.40008	E11.5 * Sox10 down vs E11.5 * Ctrl
10505779	Acer2	NM_139306	0.000829476	-1.40018	E11.5 * Sox10 down vs E11.5 * Ctrl
10454235	Asxl3	ENSMUST00000	3.6092e-005	-1.4003	E11.5 * Sox10 down vs E11.5 * Ctrl
10356293	A630001G21Rik	BC052931	0.000930064	-1.40061	E11.5 * Sox10 down vs E11.5 * Ctrl

10404402	Foxq1	NM_008239	0.00228361	-1.40076	E11.5 * Sox10 down vs E11.5 * Ctrl
10493812	S100a4	NM_011311	0.00251505	-1.40088	E11.5 * Sox10 down vs E11.5 * Ctrl
10366707	Avpr1a	NM_016847	0.000684141	-1.40097	E11.5 * Sox10 down vs E11.5 * Ctrl
10461078	Atl3	NM_146091	2.67324e-005	-1.40129	E11.5 * Sox10 down vs E11.5 * Ctrl
10494151		---	0.00199907	-1.40171	E11.5 * Sox10 down vs E11.5 * Ctrl
10503118	Fam110b	NM_173426	0.00269579	-1.40208	E11.5 * Sox10 down vs E11.5 * Ctrl
10445119	H2-M3	NM_013819	0.00052705	-1.40211	E11.5 * Sox10 down vs E11.5 * Ctrl
10598750	Gpr34	NM_011823	0.00367685	-1.40222	E11.5 * Sox10 down vs E11.5 * Ctrl
10523513	4930522N08Rik	ENSMUST00000	0.0042866	-1.40286	E11.5 * Sox10 down vs E11.5 * Ctrl
10542894		---	0.000623916	-1.4031	E11.5 * Sox10 down vs E11.5 * Ctrl
10604542	Hs6st2	NM_001077202	9.58747e-005	-1.40382	E11.5 * Sox10 down vs E11.5 * Ctrl
10399671	Hpcal1	NM_016677	5.45924e-005	-1.40445	E11.5 * Sox10 down vs E11.5 * Ctrl
10491313	Cldn11	NM_008770	0.000102093	-1.40449	E11.5 * Sox10 down vs E11.5 * Ctrl
10515771	Tie1	NM_011587	7.09765e-005	-1.40451	E11.5 * Sox10 down vs E11.5 * Ctrl
10506767	Echdc2	NM_026728	5.6277e-005	-1.40498	E11.5 * Sox10 down vs E11.5 * Ctrl
10552245	Tshz3	NM_172298	3.54274e-005	-1.40544	E11.5 * Sox10 down vs E11.5 * Ctrl
10449280	EG383229	XM_356935	0.00528274	-1.4057	E11.5 * Sox10 down vs E11.5 * Ctrl
10422962	1110020G09Rik	NM_001085410	3.60946e-006	-1.40581	E11.5 * Sox10 down vs E11.5 * Ctrl
10381588	Grn	NM_008175	0.000473536	-1.40611	E11.5 * Sox10 down vs E11.5 * Ctrl
10402117	Rps6ka5	NM_153587	0.00535494	-1.40631	E11.5 * Sox10 down vs E11.5 * Ctrl
10411609		---	0.000229077	-1.4065	E11.5 * Sox10 down vs E11.5 * Ctrl
10586491	Dapk2	NM_010019	3.9683e-005	-1.40653	E11.5 * Sox10 down vs E11.5 * Ctrl
10473312	Fam171b	NM_175514	0.00170486	-1.40659	E11.5 * Sox10 down vs E11.5 * Ctrl
10481857	Pbx3	NM_016768	8.12171e-006	-1.40661	E11.5 * Sox10 down vs E11.5 * Ctrl
10403579	Prl2c5	NM_181852	0.0028893	-1.40783	E11.5 * Sox10 down vs E11.5 * Ctrl
10493382	Pklr	NM_013631	0.000261499	-1.40806	E11.5 * Sox10 down vs E11.5 * Ctrl
10389214	Ccl9	NM_011338	0.000415107	-1.40813	E11.5 * Sox10 down vs E11.5 * Ctrl
10362499	Frk	NM_001159544	0.0002729	-1.40856	E11.5 * Sox10 down vs E11.5 * Ctrl
10512919	Grin3a	NM_001033351	8.72757e-005	-1.40891	E11.5 * Sox10 down vs E11.5 * Ctrl
10549762	Ccdc106	NM_146178	0.00021333	-1.40973	E11.5 * Sox10 down vs E11.5 * Ctrl
10561055	Ceacam2	NM_001113368	0.00300038	-1.40975	E11.5 * Sox10 down vs E11.5 * Ctrl
10348739	Sned1	NM_172463	0.00037228	-1.40976	E11.5 * Sox10 down vs E11.5 * Ctrl
10459335	Fam38b	BC147606	0.00768313	-1.4098	E11.5 * Sox10 down vs E11.5 * Ctrl
10355514	Tns1	NM_027884	0.000172592	-1.41038	E11.5 * Sox10 down vs E11.5 * Ctrl
10447923	Tcte2	NM_022311	3.50864e-006	-1.41049	E11.5 * Sox10 down vs E11.5 * Ctrl
10605303	Dnase1l1	NM_027109	1.50038e-005	-1.41086	E11.5 * Sox10 down vs E11.5 * Ctrl
10531189	Adamts3	NM_001081401	0.00601729	-1.41115	E11.5 * Sox10 down vs E11.5 * Ctrl
10557862	Itgam	NM_001082960	0.00433334	-1.41119	E11.5 * Sox10 down vs E11.5 * Ctrl
10587554	Tpbg	NM_011627	0.000836846	-1.41195	E11.5 * Sox10 down vs E11.5 * Ctrl
10453233	Slc8a1	NM_011406	5.78146e-005	-1.41244	E11.5 * Sox10 down vs E11.5 * Ctrl
10406672	Arsb	NM_009712	0.000282111	-1.41255	E11.5 * Sox10 down vs E11.5 * Ctrl
10421924	Pcdh9	NM_001081377	0.000586922	-1.41277	E11.5 * Sox10 down vs E11.5 * Ctrl
10415991	Zfp395	NM_199029	0.000553075	-1.4131	E11.5 * Sox10 down vs E11.5 * Ctrl
10427898	Fbxl7	NM_176959	0.000157132	-1.41368	E11.5 * Sox10 down vs E11.5 * Ctrl
10599693	Ncrna00086	BC052359	0.00387942	-1.41375	E11.5 * Sox10 down vs E11.5 * Ctrl
10544815	Hibadh	NM_145567	0.000350022	-1.41392	E11.5 * Sox10 down vs E11.5 * Ctrl
10538142	Gimap5	NM_175035	0.000163265	-1.41472	E11.5 * Sox10 down vs E11.5 * Ctrl
10459496	Ccbe1	NM_178793	2.81521e-007	-1.41473	E11.5 * Sox10 down vs E11.5 * Ctrl
10485597	Depdc7	NM_144804	0.00344525	-1.41604	E11.5 * Sox10 down vs E11.5 * Ctrl
10398432		---	0.000656966	-1.41629	E11.5 * Sox10 down vs E11.5 * Ctrl
10425410	Grap2	NM_010815	0.000161593	-1.41696	E11.5 * Sox10 down vs E11.5 * Ctrl
10582839	ENSMUSG00000	ENSMUST00000	0.00325298	-1.41718	E11.5 * Sox10 down vs E11.5 * Ctrl
10369116	Mcm9	NM_027830	0.00104355	-1.41795	E11.5 * Sox10 down vs E11.5 * Ctrl
10604564	Gpc4	NM_008150	0.000714623	-1.41799	E11.5 * Sox10 down vs E11.5 * Ctrl
10405755		---	9.01744e-005	-1.41805	E11.5 * Sox10 down vs E11.5 * Ctrl
10514926	B230314M03Rik	ENSMUST00000	9.59832e-005	-1.4184	E11.5 * Sox10 down vs E11.5 * Ctrl
10425092	Cyth4	NM_028195	0.000119622	-1.41845	E11.5 * Sox10 down vs E11.5 * Ctrl
10461587	Ms4a4a	XM_889011	0.00368069	-1.41846	E11.5 * Sox10 down vs E11.5 * Ctrl
10486119	Plcb2	NM_177568	0.000285372	-1.41897	E11.5 * Sox10 down vs E11.5 * Ctrl
10596747	Sema3f	NM_011349	4.80961e-006	-1.419	E11.5 * Sox10 down vs E11.5 * Ctrl
10534906	OTTMUSG00000	ENSMUST00000	0.00399975	-1.41915	E11.5 * Sox10 down vs E11.5 * Ctrl
10488623	Sox12	NM_011438	4.08072e-005	-1.4194	E11.5 * Sox10 down vs E11.5 * Ctrl
10425808	Tspo	NM_009775	0.000129973	-1.41971	E11.5 * Sox10 down vs E11.5 * Ctrl
10361250	Camk1g	NM_144817	8.1989e-005	-1.42053	E11.5 * Sox10 down vs E11.5 * Ctrl
10528548	Kcnh2	NM_013569	1.22791e-005	-1.4206	E11.5 * Sox10 down vs E11.5 * Ctrl
10469066	Ccdc3	NM_028804	0.00466495	-1.42087	E11.5 * Sox10 down vs E11.5 * Ctrl

10491613	4932438A13Rik	NM_172679	0.00557001	-1.42114	E11.5 * Sox10 down vs E11.5 * Ctrl
10394833	2410018L13Rik	BC063063	0.000267916	-1.42138	E11.5 * Sox10 down vs E11.5 * Ctrl
10585803	Stra6	NM_009291	0.000743742	-1.4214	E11.5 * Sox10 down vs E11.5 * Ctrl
10585381	Slc35f2	NM_028060	0.00457126	-1.42147	E11.5 * Sox10 down vs E11.5 * Ctrl
10439523	ENSMUSG00000	ENSMUST00000	0.00191222	-1.42219	E11.5 * Sox10 down vs E11.5 * Ctrl
10369690	Tet1	AK129421	4.21616e-006	-1.42238	E11.5 * Sox10 down vs E11.5 * Ctrl
10568536	Cpxm2	NM_018867	0.000803733	-1.4224	E11.5 * Sox10 down vs E11.5 * Ctrl
10552351		---	0.000407843	-1.4225	E11.5 * Sox10 down vs E11.5 * Ctrl
10512949	Abca1	NM_013454	8.18283e-006	-1.42256	E11.5 * Sox10 down vs E11.5 * Ctrl
10366376	Caps2	NM_178278	0.0052627	-1.42305	E11.5 * Sox10 down vs E11.5 * Ctrl
10376897		---	0.00156777	-1.42306	E11.5 * Sox10 down vs E11.5 * Ctrl
10523128	Ppbp	NM_023785	0.002815	-1.42401	E11.5 * Sox10 down vs E11.5 * Ctrl
10573451	Syce2	NM_027954	2.28631e-007	-1.42408	E11.5 * Sox10 down vs E11.5 * Ctrl
10407420	Net1	NM_019671	0.000332485	-1.42431	E11.5 * Sox10 down vs E11.5 * Ctrl
10479165	Edn3	NM_007903	2.17774e-006	-1.42444	E11.5 * Sox10 down vs E11.5 * Ctrl
10533145	Tpcn1	NM_145853	0.000234173	-1.42447	E11.5 * Sox10 down vs E11.5 * Ctrl
10355967	Ap1s3	NM_183027	0.000287728	-1.42476	E11.5 * Sox10 down vs E11.5 * Ctrl
10362138	Vnn1	NM_011704	0.000859047	-1.42489	E11.5 * Sox10 down vs E11.5 * Ctrl
10487208	Atp8b4	NM_001080944	3.00675e-005	-1.4249	E11.5 * Sox10 down vs E11.5 * Ctrl
10404301	Prl3a1	NM_025896	8.06843e-005	-1.42513	E11.5 * Sox10 down vs E11.5 * Ctrl
10482500	Rnd3	NM_028810	1.25458e-005	-1.42563	E11.5 * Sox10 down vs E11.5 * Ctrl
10385872	Slc22a5	NM_011396	0.000730455	-1.42564	E11.5 * Sox10 down vs E11.5 * Ctrl
10554574	Tm6sf1	NM_145375	0.00825123	-1.42592	E11.5 * Sox10 down vs E11.5 * Ctrl
10364262	Itgb2	NM_008404	0.000121315	-1.42593	E11.5 * Sox10 down vs E11.5 * Ctrl
10482880	Baz2b	NM_001001182	1.71295e-007	-1.42602	E11.5 * Sox10 down vs E11.5 * Ctrl
10350749	EG433365	NM_001008426	0.00504094	-1.42613	E11.5 * Sox10 down vs E11.5 * Ctrl
10531338		---	0.000178709	-1.42616	E11.5 * Sox10 down vs E11.5 * Ctrl
10360185		---	0.000510373	-1.42632	E11.5 * Sox10 down vs E11.5 * Ctrl
10406334	Mctp1	NM_030174	3.8102e-006	-1.42679	E11.5 * Sox10 down vs E11.5 * Ctrl
10430372	Rac2	NM_009008	1.67858e-005	-1.42685	E11.5 * Sox10 down vs E11.5 * Ctrl
10503376	OTTMUSG00000	DQ351292	0.00401896	-1.42755	E11.5 * Sox10 down vs E11.5 * Ctrl
10495929		---	0.00840266	-1.42786	E11.5 * Sox10 down vs E11.5 * Ctrl
10384486	Etaa1	NM_026576	0.000718584	-1.42807	E11.5 * Sox10 down vs E11.5 * Ctrl
10404024	Hist1h4h	NM_153173	0.00791555	-1.42827	E11.5 * Sox10 down vs E11.5 * Ctrl
10439547	Gramd1c	NM_153528	0.00346673	-1.42862	E11.5 * Sox10 down vs E11.5 * Ctrl
10414218	4930503E14Rik	NM_029131	0.00761373	-1.42883	E11.5 * Sox10 down vs E11.5 * Ctrl
10446619	Myom1	NM_010867	0.000361852	-1.42913	E11.5 * Sox10 down vs E11.5 * Ctrl
10595702	1190002N15Rik	NM_001033145	0.000194425	-1.42945	E11.5 * Sox10 down vs E11.5 * Ctrl
10451287	Isg15	NM_015783	0.00132345	-1.42964	E11.5 * Sox10 down vs E11.5 * Ctrl
10394789	B430203G13Rik	AK046603	1.9725e-005	-1.43019	E11.5 * Sox10 down vs E11.5 * Ctrl
10358272	Lhx9	NM_001042577	1.14252e-005	-1.43253	E11.5 * Sox10 down vs E11.5 * Ctrl
10542885	2810474O19Rik	NM_026054	0.000409875	-1.43257	E11.5 * Sox10 down vs E11.5 * Ctrl
10372139	Nts	NM_024435	0.000575494	-1.43291	E11.5 * Sox10 down vs E11.5 * Ctrl
10354588	Stk17b	NM_133810	0.00778972	-1.43376	E11.5 * Sox10 down vs E11.5 * Ctrl
10390299	Pnpo	NM_134021	0.000335043	-1.43381	E11.5 * Sox10 down vs E11.5 * Ctrl
10435525	Iqcb1	NM_177128	0.000100553	-1.43393	E11.5 * Sox10 down vs E11.5 * Ctrl
10404284	Prl3d3	NM_172156	0.00068295	-1.43402	E11.5 * Sox10 down vs E11.5 * Ctrl
10477942	Rbl1	NM_011249	0.000610244	-1.43407	E11.5 * Sox10 down vs E11.5 * Ctrl
10376239	Tcp1	AF357392	0.00551331	-1.4345	E11.5 * Sox10 down vs E11.5 * Ctrl
10455135	Pcdhb21	NM_053146	0.00121035	-1.43505	E11.5 * Sox10 down vs E11.5 * Ctrl
10456492	D18Ertd653e	NM_172631	0.000314432	-1.43594	E11.5 * Sox10 down vs E11.5 * Ctrl
10536494	Cav2	NM_016900	0.000247311	-1.43699	E11.5 * Sox10 down vs E11.5 * Ctrl
10592816	Hmbs	NM_013551	2.23044e-005	-1.4372	E11.5 * Sox10 down vs E11.5 * Ctrl
10362968	Bves	NM_024285	5.54767e-005	-1.43775	E11.5 * Sox10 down vs E11.5 * Ctrl
10449419	Tead3	NM_001098226	0.000839116	-1.4389	E11.5 * Sox10 down vs E11.5 * Ctrl
10385526	9930111J21Rik	NM_173434	0.000593998	-1.43907	E11.5 * Sox10 down vs E11.5 * Ctrl
10368343	Arg1	NM_007482	0.00262027	-1.43928	E11.5 * Sox10 down vs E11.5 * Ctrl
10479294		---	0.00204762	-1.43935	E11.5 * Sox10 down vs E11.5 * Ctrl
10470555	Gbgt1	NM_139197	0.000457655	-1.43975	E11.5 * Sox10 down vs E11.5 * Ctrl
10415013		---	2.14552e-005	-1.43982	E11.5 * Sox10 down vs E11.5 * Ctrl
10359970	Nos1ap	NM_001109985	5.8462e-005	-1.43999	E11.5 * Sox10 down vs E11.5 * Ctrl
10491623	4932438A13Rik	NM_172679	0.00145948	-1.44012	E11.5 * Sox10 down vs E11.5 * Ctrl
10407591	Chrm3	NM_033269	0.00155837	-1.44031	E11.5 * Sox10 down vs E11.5 * Ctrl
10598175	Ear10	NM_053112	0.000286737	-1.44056	E11.5 * Sox10 down vs E11.5 * Ctrl
10540359	Cntn4	NM_001109749	0.000104966	-1.44066	E11.5 * Sox10 down vs E11.5 * Ctrl
10354418	Obfc2a	NM_028696	0.00302083	-1.44085	E11.5 * Sox10 down vs E11.5 * Ctrl

10606445	Rps6ka6	NM_025949	1.11421e-005	-1.44143	E11.5 * Sox10 down vs E11.5 * Ctrl
10492424		---	0.000307001	-1.4417	E11.5 * Sox10 down vs E11.5 * Ctrl
10445767	Treml2	NM_001033405	0.000818414	-1.44204	E11.5 * Sox10 down vs E11.5 * Ctrl
10523190	9130213B05Rik	BC006604	0.00020652	-1.44295	E11.5 * Sox10 down vs E11.5 * Ctrl
10606182		---	0.00382674	-1.44363	E11.5 * Sox10 down vs E11.5 * Ctrl
10427052	Krt7	NM_033073	0.00410699	-1.44368	E11.5 * Sox10 down vs E11.5 * Ctrl
10363070	Gp49a	NM_008147	0.00246985	-1.44398	E11.5 * Sox10 down vs E11.5 * Ctrl
10578557	Ccdc111	NM_001001184	0.000359458	-1.44408	E11.5 * Sox10 down vs E11.5 * Ctrl
10607870	Tlr7	NM_133211	0.00015115	-1.44447	E11.5 * Sox10 down vs E11.5 * Ctrl
10531166	Adamts3	NM_001081401	0.000167143	-1.44462	E11.5 * Sox10 down vs E11.5 * Ctrl
10467578	Pik3ap1	NM_031376	8.23475e-006	-1.44501	E11.5 * Sox10 down vs E11.5 * Ctrl
10463410	Fam178a	NM_001081225	0.000507771	-1.44522	E11.5 * Sox10 down vs E11.5 * Ctrl
10462535	B430203M17Rik	NM_177096	0.00493237	-1.44552	E11.5 * Sox10 down vs E11.5 * Ctrl
10455873	Slc12a2	NM_009194	1.45896e-005	-1.44578	E11.5 * Sox10 down vs E11.5 * Ctrl
10354649	Pgap1	ENSMUST00000	0.00307662	-1.4471	E11.5 * Sox10 down vs E11.5 * Ctrl
10416700	Pcdh17	NM_001013753	0.000679509	-1.44772	E11.5 * Sox10 down vs E11.5 * Ctrl
10501608	Vcam1	NM_011693	0.000124157	-1.44774	E11.5 * Sox10 down vs E11.5 * Ctrl
10428124	Rgs22	ENSMUST00000	0.000471401	-1.44893	E11.5 * Sox10 down vs E11.5 * Ctrl
10572861	F2rl3	NM_007975	0.00012132	-1.44933	E11.5 * Sox10 down vs E11.5 * Ctrl
10424582		---	0.0073048	-1.44956	E11.5 * Sox10 down vs E11.5 * Ctrl
10575209		---	0.000905255	-1.44999	E11.5 * Sox10 down vs E11.5 * Ctrl
10506154	Alg6	NM_001081264	0.000872068	-1.45013	E11.5 * Sox10 down vs E11.5 * Ctrl
10590686	Ddi1	NM_027942	0.00233523	-1.45055	E11.5 * Sox10 down vs E11.5 * Ctrl
10500666	Ptgfrn	NM_011197	0.00302531	-1.45066	E11.5 * Sox10 down vs E11.5 * Ctrl
10435345	Mylk	NM_139300	2.15197e-005	-1.45092	E11.5 * Sox10 down vs E11.5 * Ctrl
10435841	Ccdc52	NM_144550	0.000168396	-1.45109	E11.5 * Sox10 down vs E11.5 * Ctrl
10556082	Ppfibp2	NM_008905	8.72519e-005	-1.45112	E11.5 * Sox10 down vs E11.5 * Ctrl
10528679	Wdr86	NM_001081441	4.44918e-005	-1.45134	E11.5 * Sox10 down vs E11.5 * Ctrl
10473406	Prg3	NM_016914	5.84799e-005	-1.45136	E11.5 * Sox10 down vs E11.5 * Ctrl
10584591		---	8.50229e-006	-1.45201	E11.5 * Sox10 down vs E11.5 * Ctrl
10550388	Gng8	NM_010320	0.00323772	-1.45269	E11.5 * Sox10 down vs E11.5 * Ctrl
10457686	Dsc2	NM_013505	9.87127e-006	-1.45313	E11.5 * Sox10 down vs E11.5 * Ctrl
10405605	Smad5	NM_008541	4.08537e-007	-1.45385	E11.5 * Sox10 down vs E11.5 * Ctrl
10359446	AI848100	BC138379	5.65506e-005	-1.45392	E11.5 * Sox10 down vs E11.5 * Ctrl
10478048	Lbp	NM_008489	4.23479e-005	-1.45421	E11.5 * Sox10 down vs E11.5 * Ctrl
10531175	Adamts3	NM_001081401	0.00394345	-1.45502	E11.5 * Sox10 down vs E11.5 * Ctrl
10409282	Ror2	NM_013846	8.95856e-005	-1.4552	E11.5 * Sox10 down vs E11.5 * Ctrl
10511290	Tnfrsf18	NM_009400	0.000277184	-1.45524	E11.5 * Sox10 down vs E11.5 * Ctrl
10344811	Cspp1	NM_026493	0.00157588	-1.45672	E11.5 * Sox10 down vs E11.5 * Ctrl
10369132	Fam184a	NM_001081428	6.26634e-006	-1.45709	E11.5 * Sox10 down vs E11.5 * Ctrl
10368373	C030003D03Rik	NM_029881	0.000263142	-1.45724	E11.5 * Sox10 down vs E11.5 * Ctrl
10520612	Khk	NM_008439	0.000406073	-1.45856	E11.5 * Sox10 down vs E11.5 * Ctrl
10526441	Upk3b	NM_175309	0.00119969	-1.45859	E11.5 * Sox10 down vs E11.5 * Ctrl
10481592	Dnm1	NM_010065	0.000138478	-1.45894	E11.5 * Sox10 down vs E11.5 * Ctrl
10461856	Gna14	NM_008137	0.00263614	-1.45901	E11.5 * Sox10 down vs E11.5 * Ctrl
10432294	C430014K11Rik	ENSMUST00000	7.40814e-006	-1.45919	E11.5 * Sox10 down vs E11.5 * Ctrl
10425066	Csf2rb	NM_007780	0.000167153	-1.45928	E11.5 * Sox10 down vs E11.5 * Ctrl
10593834	Rfpl3s	NM_183111	0.000694939	-1.46006	E11.5 * Sox10 down vs E11.5 * Ctrl
10571325	Mfhas1	NM_001081279	5.64704e-007	-1.4601	E11.5 * Sox10 down vs E11.5 * Ctrl
10431894	Slc38a2	NM_175121	1.70891e-006	-1.46011	E11.5 * Sox10 down vs E11.5 * Ctrl
10603417	Gata1	NM_008089	1.80956e-006	-1.46028	E11.5 * Sox10 down vs E11.5 * Ctrl
10399046	Vipr2	NM_009511	0.00123717	-1.46036	E11.5 * Sox10 down vs E11.5 * Ctrl
10597973	Lztfl1	NM_033322	3.29714e-005	-1.46092	E11.5 * Sox10 down vs E11.5 * Ctrl
10572800	Klf2	NM_008452	0.00155844	-1.46125	E11.5 * Sox10 down vs E11.5 * Ctrl
10571530	Fat1	NM_001081286	1.90546e-006	-1.46168	E11.5 * Sox10 down vs E11.5 * Ctrl
10549504	Dennd5b	NM_177192	0.00328421	-1.46207	E11.5 * Sox10 down vs E11.5 * Ctrl
10564235		---	0.0060033	-1.46263	E11.5 * Sox10 down vs E11.5 * Ctrl
10584317	Esam	NM_027102	0.000155678	-1.4628	E11.5 * Sox10 down vs E11.5 * Ctrl
10506225	Cachd1	NM_198037	0.000346547	-1.46332	E11.5 * Sox10 down vs E11.5 * Ctrl
10395978	Gm527	BC099503	0.000158374	-1.46386	E11.5 * Sox10 down vs E11.5 * Ctrl
10482509	Rbm43	NM_001141981	0.000542656	-1.46407	E11.5 * Sox10 down vs E11.5 * Ctrl
10350471		---	0.00255445	-1.46419	E11.5 * Sox10 down vs E11.5 * Ctrl
10409866	Ctla2b	NM_007797	0.00119752	-1.46481	E11.5 * Sox10 down vs E11.5 * Ctrl
10406419	Lysmd3	NM_030257	0.0012489	-1.46489	E11.5 * Sox10 down vs E11.5 * Ctrl
10489878	Ptgis	NM_008968	2.36435e-005	-1.46514	E11.5 * Sox10 down vs E11.5 * Ctrl
10528788	ENSMUSG0000C	ENSMUST00000	0.00207415	-1.46527	E11.5 * Sox10 down vs E11.5 * Ctrl

10428388	Rspo2	NM_172815	0.00111194	-1.46617	E11.5 * Sox10 down vs E11.5 * Ctrl
10463404	Fam178a	NM_001081225	4.10082e-007	-1.46629	E11.5 * Sox10 down vs E11.5 * Ctrl
10353524	Ogfrl1	NM_001081079	5.40671e-005	-1.4671	E11.5 * Sox10 down vs E11.5 * Ctrl
10531529	Cnot6l	NM_178854	2.23374e-006	-1.46716	E11.5 * Sox10 down vs E11.5 * Ctrl
10582078		---	0.00303169	-1.46759	E11.5 * Sox10 down vs E11.5 * Ctrl
10413416	Il17rd	NM_134437	8.43741e-005	-1.46808	E11.5 * Sox10 down vs E11.5 * Ctrl
10356520	Col6a3	AF064749	0.000121348	-1.46827	E11.5 * Sox10 down vs E11.5 * Ctrl
10407097	Pde4d	NM_011056	8.54555e-009	-1.46836	E11.5 * Sox10 down vs E11.5 * Ctrl
10528664	Smarcd3	NM_025891	6.92879e-005	-1.46861	E11.5 * Sox10 down vs E11.5 * Ctrl
10384233	Tns3	NM_001083587	6.37175e-005	-1.46895	E11.5 * Sox10 down vs E11.5 * Ctrl
10457077	Fbxo15	NM_015798	0.00182503	-1.46917	E11.5 * Sox10 down vs E11.5 * Ctrl
10551250		---	0.00204854	-1.46959	E11.5 * Sox10 down vs E11.5 * Ctrl
10407049	3830408C21Rik	ENSMUST00000	0.000210317	-1.47028	E11.5 * Sox10 down vs E11.5 * Ctrl
10583669	AB124611	AB124611	0.000480326	-1.47105	E11.5 * Sox10 down vs E11.5 * Ctrl
10412699		---	0.0050997	-1.47117	E11.5 * Sox10 down vs E11.5 * Ctrl
10523260	Shroom3	NM_015756	7.84566e-006	-1.47134	E11.5 * Sox10 down vs E11.5 * Ctrl
10357856		---	0.00066397	-1.47148	E11.5 * Sox10 down vs E11.5 * Ctrl
10544827		---	0.000732758	-1.47172	E11.5 * Sox10 down vs E11.5 * Ctrl
10542181	Clec9a	NM_172732	0.000517276	-1.47251	E11.5 * Sox10 down vs E11.5 * Ctrl
10544801	Hoxa11	NM_010450	9.76501e-005	-1.47266	E11.5 * Sox10 down vs E11.5 * Ctrl
10410973		---	1.59122e-005	-1.47302	E11.5 * Sox10 down vs E11.5 * Ctrl
10374350		---	0.000212644	-1.47311	E11.5 * Sox10 down vs E11.5 * Ctrl
10461150		---	0.00302725	-1.47317	E11.5 * Sox10 down vs E11.5 * Ctrl
10504121	4933409K07Rik	BC059060	0.00364764	-1.47319	E11.5 * Sox10 down vs E11.5 * Ctrl
10436182	Cd47	NM_010581	5.15279e-005	-1.47355	E11.5 * Sox10 down vs E11.5 * Ctrl
10543591	Opn1sw	NM_007538	0.00128338	-1.47355	E11.5 * Sox10 down vs E11.5 * Ctrl
10446425		---	0.00036753	-1.47393	E11.5 * Sox10 down vs E11.5 * Ctrl
10466224	Ms4a3	NM_133246	0.00277789	-1.47423	E11.5 * Sox10 down vs E11.5 * Ctrl
10588007	Tfdp2	NM_178667	3.43642e-009	-1.47444	E11.5 * Sox10 down vs E11.5 * Ctrl
10491885	Pcdh10	NM_001098171	0.000641947	-1.47484	E11.5 * Sox10 down vs E11.5 * Ctrl
10573924	Mmp2	NM_008610	0.000337845	-1.475	E11.5 * Sox10 down vs E11.5 * Ctrl
10513141	Ptpn3	NM_011207	0.000490162	-1.47529	E11.5 * Sox10 down vs E11.5 * Ctrl
10390269		---	0.00576695	-1.47582	E11.5 * Sox10 down vs E11.5 * Ctrl
10364444	Madcam1	NM_013591	0.00183022	-1.47647	E11.5 * Sox10 down vs E11.5 * Ctrl
10489377	Serinc3	NM_012032	2.7444e-009	-1.47647	E11.5 * Sox10 down vs E11.5 * Ctrl
10379615	Slfn5	NM_183201	0.0014784	-1.47812	E11.5 * Sox10 down vs E11.5 * Ctrl
10419162	4930503E14Rik	NM_029131	0.00389651	-1.47883	E11.5 * Sox10 down vs E11.5 * Ctrl
10510150	OTTMUSG00000	NM_001014397	0.0102129	-1.479	E11.5 * Sox10 down vs E11.5 * Ctrl
10353061		---	0.00811249	-1.47904	E11.5 * Sox10 down vs E11.5 * Ctrl
10344807	Cspp1	NM_026493	2.23522e-005	-1.47945	E11.5 * Sox10 down vs E11.5 * Ctrl
10548940	Lmo3	NM_207222	0.00434081	-1.47973	E11.5 * Sox10 down vs E11.5 * Ctrl
10404407	Foxc1	NM_008592	0.000195573	-1.48037	E11.5 * Sox10 down vs E11.5 * Ctrl
10564237	ENSMUSG00000	ENSMUST00000	0.00112597	-1.48062	E11.5 * Sox10 down vs E11.5 * Ctrl
10386110	Hand1	NM_008213	2.27816e-005	-1.48085	E11.5 * Sox10 down vs E11.5 * Ctrl
10462281	Vldlr	NM_013703	0.0002472	-1.48087	E11.5 * Sox10 down vs E11.5 * Ctrl
10524310	Ttc28	BC002262	0.00207344	-1.48119	E11.5 * Sox10 down vs E11.5 * Ctrl
10580990	Cdh8	NM_001039154	8.79126e-005	-1.48121	E11.5 * Sox10 down vs E11.5 * Ctrl
10422598	Sepp1	NM_009155	0.00115917	-1.48134	E11.5 * Sox10 down vs E11.5 * Ctrl
10394860	B230354O11Rik	AK046228	0.00267376	-1.48144	E11.5 * Sox10 down vs E11.5 * Ctrl
10471929	Arhgap15	NM_153820	0.00221986	-1.48209	E11.5 * Sox10 down vs E11.5 * Ctrl
10548727		---	0.00978735	-1.4824	E11.5 * Sox10 down vs E11.5 * Ctrl
10495206	Slc16a4	NM_146136	1.61084e-006	-1.48255	E11.5 * Sox10 down vs E11.5 * Ctrl
10564233		---	0.00180435	-1.4827	E11.5 * Sox10 down vs E11.5 * Ctrl
10355536	Tns1	NM_027884	0.00701867	-1.48293	E11.5 * Sox10 down vs E11.5 * Ctrl
10517517	C1qa	NM_007572	6.30298e-005	-1.48336	E11.5 * Sox10 down vs E11.5 * Ctrl
10389022	Myo1d	NM_177390	0.00385305	-1.48349	E11.5 * Sox10 down vs E11.5 * Ctrl
10416732	Snora30	AF357389	0.00257874	-1.48363	E11.5 * Sox10 down vs E11.5 * Ctrl
10557703	Snora30	AF357389	0.00257874	-1.48363	E11.5 * Sox10 down vs E11.5 * Ctrl
10506335	Pde4b	NM_019840	0.000171777	-1.48368	E11.5 * Sox10 down vs E11.5 * Ctrl
10383168		---	1.73833e-005	-1.48475	E11.5 * Sox10 down vs E11.5 * Ctrl
10359339	Rabgap1l	NM_013862	0.000113867	-1.48476	E11.5 * Sox10 down vs E11.5 * Ctrl
10380558		---	3.66854e-005	-1.48484	E11.5 * Sox10 down vs E11.5 * Ctrl
10438769	Cldn1	NM_016674	0.00626593	-1.48551	E11.5 * Sox10 down vs E11.5 * Ctrl
10484999	Ddb2	NM_028119	1.14093e-006	-1.48557	E11.5 * Sox10 down vs E11.5 * Ctrl
10581538	Nqo1	NM_008706	0.00113537	-1.48581	E11.5 * Sox10 down vs E11.5 * Ctrl
10389300	Dhrs11	NM_177564	0.000424509	-1.48658	E11.5 * Sox10 down vs E11.5 * Ctrl

10527940	Pftk1	NM_011074	2.6945e-005	-1.48766	E11.5 * Sox10 down vs E11.5 * Ctrl
10425761		---	6.15568e-006	-1.48799	E11.5 * Sox10 down vs E11.5 * Ctrl
10385513	9930111J21Rik	NM_173434	0.000610043	-1.48818	E11.5 * Sox10 down vs E11.5 * Ctrl
10539211	Lrrtm4	NR_027323	2.25836e-005	-1.48852	E11.5 * Sox10 down vs E11.5 * Ctrl
10443786	Pde9a	NM_008804	8.83227e-006	-1.48869	E11.5 * Sox10 down vs E11.5 * Ctrl
10531201	Adamts3	NM_001081401	0.000355894	-1.48878	E11.5 * Sox10 down vs E11.5 * Ctrl
10533462	Rad9b	NM_144912	0.00248939	-1.49021	E11.5 * Sox10 down vs E11.5 * Ctrl
10404294	Prl3b1	NM_008865	4.67331e-006	-1.49038	E11.5 * Sox10 down vs E11.5 * Ctrl
10607486	Ptchd1	NM_001093750	6.85975e-007	-1.49071	E11.5 * Sox10 down vs E11.5 * Ctrl
10346878	Zdbf2	ENSMUST00000	3.97789e-006	-1.49116	E11.5 * Sox10 down vs E11.5 * Ctrl
10565958	P2ry6	NM_183168	0.000104047	-1.49213	E11.5 * Sox10 down vs E11.5 * Ctrl
10578322	Pdgfrl	NM_026840	0.00067926	-1.49253	E11.5 * Sox10 down vs E11.5 * Ctrl
10435565	Hcls1	NM_008225	0.00031637	-1.49278	E11.5 * Sox10 down vs E11.5 * Ctrl
10421932	Pcdh9	NM_001081377	0.00198852	-1.49385	E11.5 * Sox10 down vs E11.5 * Ctrl
10544763	Hoxa4	NM_008265	8.11447e-006	-1.49401	E11.5 * Sox10 down vs E11.5 * Ctrl
10500938	Wnt2b	NM_009520	0.000245513	-1.49426	E11.5 * Sox10 down vs E11.5 * Ctrl
10440393	Samsn1	NM_023380	1.76826e-005	-1.49426	E11.5 * Sox10 down vs E11.5 * Ctrl
10466573	Ostf1	NM_017375	0.000349524	-1.49435	E11.5 * Sox10 down vs E11.5 * Ctrl
10380285	Tmem100	NM_026433	0.0021154	-1.49463	E11.5 * Sox10 down vs E11.5 * Ctrl
10605181	Renbp	NM_023132	0.000309544	-1.49522	E11.5 * Sox10 down vs E11.5 * Ctrl
10504375	Npr2	NM_173788	0.000993267	-1.49525	E11.5 * Sox10 down vs E11.5 * Ctrl
10372807	Msrb3	NM_177092	0.000123717	-1.4955	E11.5 * Sox10 down vs E11.5 * Ctrl
10446253	Vav1	NM_011691	0.000849534	-1.49559	E11.5 * Sox10 down vs E11.5 * Ctrl
10396778	Mpp5	NM_019579	1.05221e-006	-1.49566	E11.5 * Sox10 down vs E11.5 * Ctrl
10557853	B230325K18Rik	ENSMUST00000	0.000297137	-1.49566	E11.5 * Sox10 down vs E11.5 * Ctrl
10572527	EG665858	ENSMUST00000	0.0102608	-1.49601	E11.5 * Sox10 down vs E11.5 * Ctrl
10495596	Frrs1	NM_001113478	0.000361185	-1.4961	E11.5 * Sox10 down vs E11.5 * Ctrl
10458843	Sema6a	NM_018744	9.04927e-006	-1.49723	E11.5 * Sox10 down vs E11.5 * Ctrl
10508663	Laptm5	NM_010686	0.00403826	-1.49748	E11.5 * Sox10 down vs E11.5 * Ctrl
10585377		---	0.000374866	-1.49771	E11.5 * Sox10 down vs E11.5 * Ctrl
10420747	Gata4	NM_008092	8.42455e-005	-1.49841	E11.5 * Sox10 down vs E11.5 * Ctrl
10403081	Wdr60	NM_146039	1.10089e-005	-1.49879	E11.5 * Sox10 down vs E11.5 * Ctrl
10406504	Edil3	NM_001037987	0.00525303	-1.49883	E11.5 * Sox10 down vs E11.5 * Ctrl
10410756	Ankrd32	NM_134071	0.000661071	-1.49891	E11.5 * Sox10 down vs E11.5 * Ctrl
10347291	Il8rb	NM_009909	0.000298674	-1.49898	E11.5 * Sox10 down vs E11.5 * Ctrl
10361139	Traf5	NM_011633	2.75917e-005	-1.4992	E11.5 * Sox10 down vs E11.5 * Ctrl
10497337	Car1	NM_009799	0.00141641	-1.49949	E11.5 * Sox10 down vs E11.5 * Ctrl
10366266	Pawr	NM_054056	0.00503087	-1.49949	E11.5 * Sox10 down vs E11.5 * Ctrl
10434285		---	3.32573e-005	-1.49993	E11.5 * Sox10 down vs E11.5 * Ctrl
10433633	Mkl2	NM_181860	0.000204894	-1.49995	E11.5 * Sox10 down vs E11.5 * Ctrl
10413932	E130203B14Rik	NM_178791	0.000124644	-1.50118	E11.5 * Sox10 down vs E11.5 * Ctrl
10555087		---	0.0100417	-1.50129	E11.5 * Sox10 down vs E11.5 * Ctrl
10524312	Ttc28	ENSMUST00000	8.95084e-005	-1.50165	E11.5 * Sox10 down vs E11.5 * Ctrl
10401673	Tgfb3	NM_009368	0.00291747	-1.50208	E11.5 * Sox10 down vs E11.5 * Ctrl
10546010	Arhgap25	NM_001037727	0.00194	-1.5023	E11.5 * Sox10 down vs E11.5 * Ctrl
10547469	Hsn2	NM_001037155	0.00122085	-1.503	E11.5 * Sox10 down vs E11.5 * Ctrl
10528385	Reln	NM_011261	8.23501e-006	-1.50327	E11.5 * Sox10 down vs E11.5 * Ctrl
10562651	C330019L16Rik	NM_001034857	0.00231591	-1.50373	E11.5 * Sox10 down vs E11.5 * Ctrl
10509000		---	0.000138932	-1.50375	E11.5 * Sox10 down vs E11.5 * Ctrl
10594066	Loxl1	NM_010729	0.0012122	-1.50397	E11.5 * Sox10 down vs E11.5 * Ctrl
10506254	Raver2	NM_183024	1.9836e-005	-1.50437	E11.5 * Sox10 down vs E11.5 * Ctrl
10536505	Met	NM_008591	0.000694652	-1.50507	E11.5 * Sox10 down vs E11.5 * Ctrl
10597239	Pth1r	NM_011199	2.8182e-006	-1.50698	E11.5 * Sox10 down vs E11.5 * Ctrl
10554521	Pde8a	NM_008803	0.000189264	-1.5075	E11.5 * Sox10 down vs E11.5 * Ctrl
10402606	Rtl1	NM_184109	2.62069e-005	-1.5076	E11.5 * Sox10 down vs E11.5 * Ctrl
10434743		---	0.00174632	-1.50771	E11.5 * Sox10 down vs E11.5 * Ctrl
10458661		---	1.59243e-005	-1.50801	E11.5 * Sox10 down vs E11.5 * Ctrl
10448836	Tmem204	NM_001001183	1.46202e-005	-1.50802	E11.5 * Sox10 down vs E11.5 * Ctrl
10354647	Pgap1	ENSMUST00000	0.00069691	-1.50825	E11.5 * Sox10 down vs E11.5 * Ctrl
10485405	Cd44	NM_009851	8.39112e-006	-1.50999	E11.5 * Sox10 down vs E11.5 * Ctrl
10548333	Cd69	NM_001033122	0.00046044	-1.51125	E11.5 * Sox10 down vs E11.5 * Ctrl
10366293	Csrp2	NM_007792	2.57634e-005	-1.51182	E11.5 * Sox10 down vs E11.5 * Ctrl
10495449	Col11a1	NM_007729	3.59168e-008	-1.51202	E11.5 * Sox10 down vs E11.5 * Ctrl
10375358	Ebf1	ENSMUST00000	0.00858366	-1.51216	E11.5 * Sox10 down vs E11.5 * Ctrl
10559649	Cox6b2	NM_183405	0.0102949	-1.51255	E11.5 * Sox10 down vs E11.5 * Ctrl
10372280	EG628870	XM_893664	1.52154e-005	-1.51289	E11.5 * Sox10 down vs E11.5 * Ctrl

10369750	ENSMUSG0000C	ENSMUST00000(	0.00470194	-1.5129	E11.5 * Sox10 down vs E11.5 * Ctrl
10519811	Speer8-ps1	NR_001584	7.33395e-005	-1.51344	E11.5 * Sox10 down vs E11.5 * Ctrl
10458875	Dtwd2	NM_026854	0.00477661	-1.51358	E11.5 * Sox10 down vs E11.5 * Ctrl
10387743	Slc2a4	NM_009204	8.65194e-006	-1.51371	E11.5 * Sox10 down vs E11.5 * Ctrl
10493086	Hdgf	NM_008231	2.33138e-005	-1.51399	E11.5 * Sox10 down vs E11.5 * Ctrl
10566934	Lyve1	NM_053247	0.000187269	-1.51414	E11.5 * Sox10 down vs E11.5 * Ctrl
10407598	Ryr2	NM_023868	1.06343e-007	-1.51495	E11.5 * Sox10 down vs E11.5 * Ctrl
10417167	Clybl	NM_029556	2.90909e-005	-1.51511	E11.5 * Sox10 down vs E11.5 * Ctrl
10406663	Arsb	NM_009712	0.00132254	-1.51556	E11.5 * Sox10 down vs E11.5 * Ctrl
10349102	Bcl2	NM_009741	2.18157e-005	-1.51578	E11.5 * Sox10 down vs E11.5 * Ctrl
10566943	Mrvi1	NM_010826	0.000184677	-1.51635	E11.5 * Sox10 down vs E11.5 * Ctrl
10437160	Ets2	NM_011809	7.34981e-006	-1.51863	E11.5 * Sox10 down vs E11.5 * Ctrl
10566333	9230105E10Rik	NM_001146007	6.61217e-007	-1.51901	E11.5 * Sox10 down vs E11.5 * Ctrl
10554059	ENSMUSG0000C	ENSMUST00000(	0.000839085	-1.51919	E11.5 * Sox10 down vs E11.5 * Ctrl
10531185	Adamts3	NM_001081401	2.52573e-005	-1.51987	E11.5 * Sox10 down vs E11.5 * Ctrl
10494351	Mtmr11	NM_181409	4.95689e-005	-1.52145	E11.5 * Sox10 down vs E11.5 * Ctrl
10489246	Mafb	NM_010658	0.00091632	-1.52165	E11.5 * Sox10 down vs E11.5 * Ctrl
10359624	Prrx1	NM_175686	0.0055909	-1.5218	E11.5 * Sox10 down vs E11.5 * Ctrl
10547100	Plxnd1	NM_026376	0.000517253	-1.52184	E11.5 * Sox10 down vs E11.5 * Ctrl
10399148	Rapgef5	NM_175930	0.000190796	-1.52208	E11.5 * Sox10 down vs E11.5 * Ctrl
10421911	Pcdh20	NM_178685	0.00555406	-1.52279	E11.5 * Sox10 down vs E11.5 * Ctrl
10410475	BC018507	ENSMUST00000(	8.10731e-007	-1.52292	E11.5 * Sox10 down vs E11.5 * Ctrl
10547769	Ptpn6	NM_013545	3.22824e-006	-1.52306	E11.5 * Sox10 down vs E11.5 * Ctrl
10406551	Ssbp2	NM_024272	5.14236e-008	-1.52316	E11.5 * Sox10 down vs E11.5 * Ctrl
10414102	Mmrn2	NM_153127	0.00145697	-1.52369	E11.5 * Sox10 down vs E11.5 * Ctrl
10447317	Epas1	NM_010137	0.000998336	-1.52379	E11.5 * Sox10 down vs E11.5 * Ctrl
10491625	4932438A13Rik	NM_172679	0.000591435	-1.52478	E11.5 * Sox10 down vs E11.5 * Ctrl
10456329	5330437I02Rik	NM_177028	0.000133844	-1.52637	E11.5 * Sox10 down vs E11.5 * Ctrl
10598586	Xk	NM_023500	4.59397e-005	-1.52705	E11.5 * Sox10 down vs E11.5 * Ctrl
10369702	Tet1	ENSMUST00000(	2.55109e-006	-1.52887	E11.5 * Sox10 down vs E11.5 * Ctrl
10439058	Lrrc33	NM_146069	3.63968e-005	-1.52901	E11.5 * Sox10 down vs E11.5 * Ctrl
10397975	Ifi27l1	NM_026790	3.35703e-005	-1.52923	E11.5 * Sox10 down vs E11.5 * Ctrl
10420216	Sdr39u1	NM_001082975	0.000484192	-1.52947	E11.5 * Sox10 down vs E11.5 * Ctrl
10369171	9530009G21Rik	AK156585	0.00119181	-1.53012	E11.5 * Sox10 down vs E11.5 * Ctrl
10500911	Mov10	NM_008619	3.65422e-005	-1.53043	E11.5 * Sox10 down vs E11.5 * Ctrl
10395293	Atxn7l1	NM_001033436	6.34362e-006	-1.53177	E11.5 * Sox10 down vs E11.5 * Ctrl
10405058	Omd	NM_012050	0.00117602	-1.53192	E11.5 * Sox10 down vs E11.5 * Ctrl
10561212	Ltbp4	NM_175641	4.48955e-005	-1.53212	E11.5 * Sox10 down vs E11.5 * Ctrl
10504123	4933409K07Rik	BC059060	0.00323382	-1.53233	E11.5 * Sox10 down vs E11.5 * Ctrl
10504125	4933409K07Rik	BC059060	0.00323382	-1.53233	E11.5 * Sox10 down vs E11.5 * Ctrl
10476299		---	0.00709132	-1.53278	E11.5 * Sox10 down vs E11.5 * Ctrl
10354368	Col5a2	BC055077	1.50431e-005	-1.533	E11.5 * Sox10 down vs E11.5 * Ctrl
10459227		---	0.00215404	-1.53371	E11.5 * Sox10 down vs E11.5 * Ctrl
10595718	Chst2	NM_018763	4.56037e-005	-1.53382	E11.5 * Sox10 down vs E11.5 * Ctrl
10454601	Tslp	NM_021367	1.3673e-005	-1.53461	E11.5 * Sox10 down vs E11.5 * Ctrl
10398428		---	5.43264e-005	-1.53469	E11.5 * Sox10 down vs E11.5 * Ctrl
10492957	Cd1d2	NM_007640	5.8062e-006	-1.53514	E11.5 * Sox10 down vs E11.5 * Ctrl
10358408	Rgs1	NM_015811	0.00821725	-1.53516	E11.5 * Sox10 down vs E11.5 * Ctrl
10495967	Tifa	NM_145133	1.24337e-005	-1.53604	E11.5 * Sox10 down vs E11.5 * Ctrl
10579012	Csgalnact1	NM_172753	0.000356065	-1.5362	E11.5 * Sox10 down vs E11.5 * Ctrl
10423024	Capsl	NM_029341	0.000491501	-1.53666	E11.5 * Sox10 down vs E11.5 * Ctrl
10406982	Adamts6	NM_001081020	0.000289898	-1.53729	E11.5 * Sox10 down vs E11.5 * Ctrl
10544788	Hoxa9	NM_010456	0.000182465	-1.53763	E11.5 * Sox10 down vs E11.5 * Ctrl
10457587	Zfp521	NM_145492	4.03613e-007	-1.53788	E11.5 * Sox10 down vs E11.5 * Ctrl
10507101	OTTMUSG00000	NM_001085549	1.52349e-005	-1.53817	E11.5 * Sox10 down vs E11.5 * Ctrl
10462132	E030010A14Rik	NM_183160	2.25575e-006	-1.53875	E11.5 * Sox10 down vs E11.5 * Ctrl
10432636	BC004728	NM_174992	0.000119459	-1.53932	E11.5 * Sox10 down vs E11.5 * Ctrl
10381122	Fkbp10	NM_010221	0.000114128	-1.53983	E11.5 * Sox10 down vs E11.5 * Ctrl
10428536	Trps1	NM_032000	2.6542e-005	-1.54086	E11.5 * Sox10 down vs E11.5 * Ctrl
10447848	Mas1	NM_008552	0.000194263	-1.54174	E11.5 * Sox10 down vs E11.5 * Ctrl
10499536	Efna1	NM_010107	2.23433e-006	-1.54191	E11.5 * Sox10 down vs E11.5 * Ctrl
10423230	Cdh9	NM_009869	3.59552e-005	-1.54215	E11.5 * Sox10 down vs E11.5 * Ctrl
10428534	Trps1	NM_032000	3.04659e-005	-1.54269	E11.5 * Sox10 down vs E11.5 * Ctrl
10544538		---	0.00993053	-1.54284	E11.5 * Sox10 down vs E11.5 * Ctrl
10344809	Cspp1	NM_026493	0.00910289	-1.54303	E11.5 * Sox10 down vs E11.5 * Ctrl
10527051	Sdk1	NM_177879	3.4487e-005	-1.54328	E11.5 * Sox10 down vs E11.5 * Ctrl

10419156	Ear10	NM_053112	3.19195e-005	-1.54335	E11.5 * Sox10 down vs E11.5 * Ctrl
10547322	Cacna1c	NM_001159533	1.10687e-007	-1.54364	E11.5 * Sox10 down vs E11.5 * Ctrl
10380859	Crkrs	BC057057	0.00170005	-1.54377	E11.5 * Sox10 down vs E11.5 * Ctrl
10412298	Itga1	NM_001033228	0.00566479	-1.54435	E11.5 * Sox10 down vs E11.5 * Ctrl
10555862	Trim34	NM_030684	9.86473e-005	-1.54449	E11.5 * Sox10 down vs E11.5 * Ctrl
10428517	Csmd3	NM_001081391	4.76204e-005	-1.54486	E11.5 * Sox10 down vs E11.5 * Ctrl
10443980	Myo1f	NM_053214	0.00142581	-1.54517	E11.5 * Sox10 down vs E11.5 * Ctrl
10538802	A930038C07Rik	NM_172399	0.000415819	-1.54536	E11.5 * Sox10 down vs E11.5 * Ctrl
10405733	EG630579	NM_001039239	0.000431037	-1.54554	E11.5 * Sox10 down vs E11.5 * Ctrl
10424543	Wisp1	NM_018865	0.00022276	-1.54681	E11.5 * Sox10 down vs E11.5 * Ctrl
10496862		---	6.48723e-006	-1.54731	E11.5 * Sox10 down vs E11.5 * Ctrl
10466530	Pcsk5	BC013068	7.17005e-006	-1.54757	E11.5 * Sox10 down vs E11.5 * Ctrl
10439016	4930444G20Rik	NM_053264	0.00970337	-1.54767	E11.5 * Sox10 down vs E11.5 * Ctrl
10534493	Ccl24	NM_019577	0.00034229	-1.54834	E11.5 * Sox10 down vs E11.5 * Ctrl
10431749	Adamts20	NM_177431	2.28583e-005	-1.55102	E11.5 * Sox10 down vs E11.5 * Ctrl
10372988	Slc16a7	NM_011391	0.00021869	-1.55139	E11.5 * Sox10 down vs E11.5 * Ctrl
10436519	Robo1	NM_019413	1.39243e-006	-1.55239	E11.5 * Sox10 down vs E11.5 * Ctrl
10406276		---	0.00259058	-1.55254	E11.5 * Sox10 down vs E11.5 * Ctrl
10359419		---	0.00201823	-1.55322	E11.5 * Sox10 down vs E11.5 * Ctrl
10419559	Rnase12	NM_001011875	8.55012e-005	-1.55398	E11.5 * Sox10 down vs E11.5 * Ctrl
10362615	Traf3ip2	NM_134000	2.10186e-006	-1.55493	E11.5 * Sox10 down vs E11.5 * Ctrl
10434778	Rtp4	NM_023386	0.000105506	-1.55566	E11.5 * Sox10 down vs E11.5 * Ctrl
10355227	1110028C15Rik	NM_001122738	1.42242e-005	-1.55567	E11.5 * Sox10 down vs E11.5 * Ctrl
10372844	Rassf3	NM_138956	9.20468e-005	-1.55682	E11.5 * Sox10 down vs E11.5 * Ctrl
10554156	Fam174b	ENSMUST00000	8.1737e-006	-1.55689	E11.5 * Sox10 down vs E11.5 * Ctrl
10491627	4932438A13Rik	NM_172679	0.000564813	-1.55725	E11.5 * Sox10 down vs E11.5 * Ctrl
10580349	Mylk3	NM_175441	3.89584e-005	-1.55754	E11.5 * Sox10 down vs E11.5 * Ctrl
10554599	Adamtsl3	ENSMUST00000	0.000164468	-1.55758	E11.5 * Sox10 down vs E11.5 * Ctrl
10447602	Ezr	NM_009510	2.40671e-005	-1.55889	E11.5 * Sox10 down vs E11.5 * Ctrl
10462140	Dock8	NM_028785	0.000240419	-1.55938	E11.5 * Sox10 down vs E11.5 * Ctrl
10500283	Car14	NM_011797	8.31747e-005	-1.55976	E11.5 * Sox10 down vs E11.5 * Ctrl
10537880		---	6.04106e-005	-1.55991	E11.5 * Sox10 down vs E11.5 * Ctrl
10600169	Bgn	NM_007542	4.51056e-006	-1.56171	E11.5 * Sox10 down vs E11.5 * Ctrl
10410766	Nr2f1	NM_010151	9.15568e-008	-1.56276	E11.5 * Sox10 down vs E11.5 * Ctrl
10549102	Kcnj8	NM_008428	0.0055625	-1.56305	E11.5 * Sox10 down vs E11.5 * Ctrl
10368585	Nkain2	NM_001013411	0.000145209	-1.56324	E11.5 * Sox10 down vs E11.5 * Ctrl
10551185	Tgfb1	NM_011577	3.62768e-005	-1.56346	E11.5 * Sox10 down vs E11.5 * Ctrl
10531179	Adamts3	NM_001081401	0.000395261	-1.56358	E11.5 * Sox10 down vs E11.5 * Ctrl
10498166	ENSMUSG0000C	ENSMUST00000	0.00447311	-1.56369	E11.5 * Sox10 down vs E11.5 * Ctrl
10532741	Tmem119	NM_146162	0.000139136	-1.56401	E11.5 * Sox10 down vs E11.5 * Ctrl
10594840	Gcom1	NM_001033208	7.1954e-005	-1.5641	E11.5 * Sox10 down vs E11.5 * Ctrl
10554074	Adamts17	NM_001033877	0.00253857	-1.56411	E11.5 * Sox10 down vs E11.5 * Ctrl
10482301	A930041I02Rik	NM_178778	1.43821e-005	-1.56442	E11.5 * Sox10 down vs E11.5 * Ctrl
10381898	Mrc2	NM_008626	0.000323929	-1.56444	E11.5 * Sox10 down vs E11.5 * Ctrl
10553788	Atp10a	NM_009728	2.34851e-007	-1.5645	E11.5 * Sox10 down vs E11.5 * Ctrl
10366196	Ppfia2	NM_177373	8.01528e-006	-1.56583	E11.5 * Sox10 down vs E11.5 * Ctrl
10601270	ENSMUSG0000C	ENSMUST00000	0.00013308	-1.56707	E11.5 * Sox10 down vs E11.5 * Ctrl
10560624	Apoe	NM_009696	4.07583e-006	-1.568	E11.5 * Sox10 down vs E11.5 * Ctrl
10528804	ENSMUSG0000C	NM_001134299	9.20122e-006	-1.56813	E11.5 * Sox10 down vs E11.5 * Ctrl
10602385	Pfkfb1	NM_008824	0.00308547	-1.56818	E11.5 * Sox10 down vs E11.5 * Ctrl
10542164	Clec12a	NM_177686	4.15411e-005	-1.56902	E11.5 * Sox10 down vs E11.5 * Ctrl
10493449	Thbs3	NM_013691	8.26232e-008	-1.56937	E11.5 * Sox10 down vs E11.5 * Ctrl
10469457	Plxdc2	NM_026162	1.66769e-007	-1.57013	E11.5 * Sox10 down vs E11.5 * Ctrl
10367591	Myct1	NM_026793	0.00211407	-1.57028	E11.5 * Sox10 down vs E11.5 * Ctrl
10407124	AI452195	ENSMUST00000	0.00766118	-1.57054	E11.5 * Sox10 down vs E11.5 * Ctrl
10588786	Ube1l	NM_023738	6.8556e-006	-1.57061	E11.5 * Sox10 down vs E11.5 * Ctrl
10499113	Gm1019	NM_001001650	2.75977e-006	-1.57089	E11.5 * Sox10 down vs E11.5 * Ctrl
10408574		---	4.15372e-005	-1.57095	E11.5 * Sox10 down vs E11.5 * Ctrl
10365845	Fgd6	NM_053072	6.07715e-006	-1.57168	E11.5 * Sox10 down vs E11.5 * Ctrl
10490777	Zfhx4	NM_030708	2.50058e-007	-1.57197	E11.5 * Sox10 down vs E11.5 * Ctrl
10466248	Stx3	NM_001025307	4.33023e-006	-1.57255	E11.5 * Sox10 down vs E11.5 * Ctrl
10523727	Pkd2	NM_008861	3.0233e-007	-1.5727	E11.5 * Sox10 down vs E11.5 * Ctrl
10459071	2010002N04Rik	NM_134133	3.10399e-005	-1.57287	E11.5 * Sox10 down vs E11.5 * Ctrl
10469167	Sfmbt2	NM_177386	3.31946e-010	-1.57303	E11.5 * Sox10 down vs E11.5 * Ctrl
10519913	Magi2	NM_015823	0.00032236	-1.57333	E11.5 * Sox10 down vs E11.5 * Ctrl
10476560	Ism1	NM_001126490	0.000236948	-1.57339	E11.5 * Sox10 down vs E11.5 * Ctrl

10607924	Amelx	NM_001081978	2.07725e-005	-1.57505	E11.5 * Sox10 down vs E11.5 * Ctrl
10436602		---	0.00336419	-1.5754	E11.5 * Sox10 down vs E11.5 * Ctrl
10531972	5830443L24Rik	NM_029509	7.16973e-005	-1.57574	E11.5 * Sox10 down vs E11.5 * Ctrl
10541555	Clec4a1	NM_199311	0.00087557	-1.57726	E11.5 * Sox10 down vs E11.5 * Ctrl
10403871	Aoah	NM_012054	1.21068e-005	-1.58278	E11.5 * Sox10 down vs E11.5 * Ctrl
10380419	Col1a1	NM_007742	5.62304e-008	-1.58308	E11.5 * Sox10 down vs E11.5 * Ctrl
10584208	Cdon	NM_021339	4.06292e-008	-1.58351	E11.5 * Sox10 down vs E11.5 * Ctrl
10354247	Fhl2	NM_010212	1.3967e-005	-1.58447	E11.5 * Sox10 down vs E11.5 * Ctrl
10530641	Usp46	NM_177561	0.000682549	-1.58466	E11.5 * Sox10 down vs E11.5 * Ctrl
10501222	Gstm2	NM_008183	0.000524315	-1.58489	E11.5 * Sox10 down vs E11.5 * Ctrl
10515803	1110020C03Rik	BC062805	1.51042e-005	-1.58492	E11.5 * Sox10 down vs E11.5 * Ctrl
10458999	Fbn2	NM_010181	7.03875e-006	-1.58517	E11.5 * Sox10 down vs E11.5 * Ctrl
10507137	Pdzk1ip1	NM_026018	9.58236e-007	-1.58517	E11.5 * Sox10 down vs E11.5 * Ctrl
10427303	Hoxc4	NM_013553	4.28389e-005	-1.5861	E11.5 * Sox10 down vs E11.5 * Ctrl
10400483	Slc25a21	NM_172577	6.65816e-006	-1.58623	E11.5 * Sox10 down vs E11.5 * Ctrl
10385903	Pdlim4	NM_019417	2.15933e-006	-1.58724	E11.5 * Sox10 down vs E11.5 * Ctrl
10561453	Zfp36	NM_011756	0.000480225	-1.58757	E11.5 * Sox10 down vs E11.5 * Ctrl
10526211		---	0.00607682	-1.58759	E11.5 * Sox10 down vs E11.5 * Ctrl
10531610	Rasgef1b	NM_145839	0.000391547	-1.58786	E11.5 * Sox10 down vs E11.5 * Ctrl
10513154	Ptpn3	NM_011207	0.000638585	-1.58889	E11.5 * Sox10 down vs E11.5 * Ctrl
10542172	Clec1b	NM_019985	5.86754e-006	-1.58929	E11.5 * Sox10 down vs E11.5 * Ctrl
10498379		---	0.000309293	-1.58965	E11.5 * Sox10 down vs E11.5 * Ctrl
10493307		---	0.00533228	-1.58985	E11.5 * Sox10 down vs E11.5 * Ctrl
10433075	5730585A16Rik	ENSMUST00000(	0.00678208	-1.58991	E11.5 * Sox10 down vs E11.5 * Ctrl
10354003	Mgat4a	NM_173870	0.00405996	-1.59033	E11.5 * Sox10 down vs E11.5 * Ctrl
10531980	BC057170	NM_172777	0.000441847	-1.59068	E11.5 * Sox10 down vs E11.5 * Ctrl
10472240	Tanc1	NM_198294	1.12374e-007	-1.59146	E11.5 * Sox10 down vs E11.5 * Ctrl
10482918		---	0.00258521	-1.59153	E11.5 * Sox10 down vs E11.5 * Ctrl
10587659	EG547109	NM_001034906	0.000374853	-1.59176	E11.5 * Sox10 down vs E11.5 * Ctrl
10439126		---	7.68445e-005	-1.59396	E11.5 * Sox10 down vs E11.5 * Ctrl
10423796		---	0.00961877	-1.59423	E11.5 * Sox10 down vs E11.5 * Ctrl
10519324	Cdk6	NM_009873	1.9169e-006	-1.59425	E11.5 * Sox10 down vs E11.5 * Ctrl
10549162	St8sia1	NM_011374	2.33361e-005	-1.59453	E11.5 * Sox10 down vs E11.5 * Ctrl
10456437		---	0.0023751	-1.59472	E11.5 * Sox10 down vs E11.5 * Ctrl
10360382	Ifi204	NM_008329	0.00427868	-1.59506	E11.5 * Sox10 down vs E11.5 * Ctrl
10546510	Lrig1	NM_008377	1.27434e-005	-1.59649	E11.5 * Sox10 down vs E11.5 * Ctrl
10568024	Coro1a	NM_009898	3.75236e-006	-1.59709	E11.5 * Sox10 down vs E11.5 * Ctrl
10563893	ENSMUSG0000C	ENSMUST00000(	0.00770169	-1.59778	E11.5 * Sox10 down vs E11.5 * Ctrl
10599598	A630012P03Rik	ENSMUST00000	1.56416e-005	-1.598	E11.5 * Sox10 down vs E11.5 * Ctrl
10405063	Ogn	NM_008760	2.52392e-005	-1.59833	E11.5 * Sox10 down vs E11.5 * Ctrl
10445944	St6gal2	NM_172829	0.000774711	-1.5986	E11.5 * Sox10 down vs E11.5 * Ctrl
10536220	Col1a2	NM_007743	4.01768e-007	-1.59863	E11.5 * Sox10 down vs E11.5 * Ctrl
10586722	F830001A07Rik	AK045567	0.000777521	-1.59868	E11.5 * Sox10 down vs E11.5 * Ctrl
10383233	Rnf213	AK173199	0.00183678	-1.59881	E11.5 * Sox10 down vs E11.5 * Ctrl
10469695	Apbb1ip	NM_019456	1.42066e-005	-1.59922	E11.5 * Sox10 down vs E11.5 * Ctrl
10604956		---	6.71156e-005	-1.59949	E11.5 * Sox10 down vs E11.5 * Ctrl
10563441	Emp3	NM_010129	0.000125527	-1.59961	E11.5 * Sox10 down vs E11.5 * Ctrl
10575976	Crispld2	NM_030209	2.5547e-005	-1.59978	E11.5 * Sox10 down vs E11.5 * Ctrl
10380135		---	0.000202103	-1.60025	E11.5 * Sox10 down vs E11.5 * Ctrl
10472962	Hoxd9	NM_013555	0.00136729	-1.60038	E11.5 * Sox10 down vs E11.5 * Ctrl
10479397	Ntsr1	NM_018766	0.000348399	-1.601	E11.5 * Sox10 down vs E11.5 * Ctrl
10409059		---	0.000125384	-1.60237	E11.5 * Sox10 down vs E11.5 * Ctrl
10398319	Dlk1	NM_010052	0.00262285	-1.60318	E11.5 * Sox10 down vs E11.5 * Ctrl
10376245	Gria1	NM_001113325	3.56764e-007	-1.60323	E11.5 * Sox10 down vs E11.5 * Ctrl
10607943		---	5.47804e-005	-1.60329	E11.5 * Sox10 down vs E11.5 * Ctrl
10398326	Meg3	NR_003633	0.000171402	-1.60451	E11.5 * Sox10 down vs E11.5 * Ctrl
10365574	Pmch	NM_029971	0.00185631	-1.60642	E11.5 * Sox10 down vs E11.5 * Ctrl
10583870	Bmper	NM_028472	4.12511e-006	-1.60719	E11.5 * Sox10 down vs E11.5 * Ctrl
10361790	Fuca2	NM_025799	1.74783e-005	-1.6102	E11.5 * Sox10 down vs E11.5 * Ctrl
10455128	Pcdhb20	NM_053145	0.00157501	-1.61039	E11.5 * Sox10 down vs E11.5 * Ctrl
10372618	Frs2	NM_177798	1.51093e-005	-1.61065	E11.5 * Sox10 down vs E11.5 * Ctrl
10531193	Adamts3	NM_001081401	0.00034536	-1.61142	E11.5 * Sox10 down vs E11.5 * Ctrl
10389929	Cacna1g	NM_009783	1.11836e-005	-1.61148	E11.5 * Sox10 down vs E11.5 * Ctrl
10366052	Kitl	NM_013598	1.75595e-005	-1.61169	E11.5 * Sox10 down vs E11.5 * Ctrl
10474201	Lmo2	NM_008505	8.9292e-006	-1.61243	E11.5 * Sox10 down vs E11.5 * Ctrl
10398907	Pld4	NM_178911	4.67202e-006	-1.61248	E11.5 * Sox10 down vs E11.5 * Ctrl

10365991	Epyc	NM_007884	1.24308e-005	-1.61281	E11.5 * Sox10 down vs E11.5 * Ctrl
10435305	Itgb5	NM_001145884	1.19808e-007	-1.61415	E11.5 * Sox10 down vs E11.5 * Ctrl
10474700	Thbs1	NM_011580	9.29165e-006	-1.6143	E11.5 * Sox10 down vs E11.5 * Ctrl
10531183	Adamts3	NM_001081401	4.78609e-005	-1.61457	E11.5 * Sox10 down vs E11.5 * Ctrl
10434741		---	0.00268342	-1.61508	E11.5 * Sox10 down vs E11.5 * Ctrl
10395428	Dgkb	NM_178681	0.00012767	-1.6154	E11.5 * Sox10 down vs E11.5 * Ctrl
10362102	ENSMUSG0000C	ENSMUST00000	6.8693e-005	-1.61546	E11.5 * Sox10 down vs E11.5 * Ctrl
10519484	Steap2	AK141701	0.000434745	-1.61546	E11.5 * Sox10 down vs E11.5 * Ctrl
10346607	Fzd7	NM_008057	3.9835e-008	-1.61563	E11.5 * Sox10 down vs E11.5 * Ctrl
10607658	Reps2	NM_178256	4.95088e-005	-1.61628	E11.5 * Sox10 down vs E11.5 * Ctrl
10541491	1700063H04Rik	ENSMUST00000	1.47466e-006	-1.61647	E11.5 * Sox10 down vs E11.5 * Ctrl
10375055	F830116E18Rik	NM_001033981	0.00107417	-1.6171	E11.5 * Sox10 down vs E11.5 * Ctrl
10528821	ENSMUSG0000C	NM_001134299	1.38161e-006	-1.61758	E11.5 * Sox10 down vs E11.5 * Ctrl
10350516	Ptgs2	NM_011198	0.00426171	-1.61836	E11.5 * Sox10 down vs E11.5 * Ctrl
10401068	Spnb1	NM_013675	1.67997e-006	-1.61861	E11.5 * Sox10 down vs E11.5 * Ctrl
10383198		---	0.000670212	-1.62049	E11.5 * Sox10 down vs E11.5 * Ctrl
10455071	Pcdhb7	NM_053132	0.00027178	-1.62212	E11.5 * Sox10 down vs E11.5 * Ctrl
10478928	Tshz2	NM_080455	1.7182e-008	-1.62301	E11.5 * Sox10 down vs E11.5 * Ctrl
10436196		---	1.38601e-005	-1.62322	E11.5 * Sox10 down vs E11.5 * Ctrl
10397715		---	0.0011759	-1.62514	E11.5 * Sox10 down vs E11.5 * Ctrl
10468691	Ablim1	NM_178688	8.2482e-007	-1.62523	E11.5 * Sox10 down vs E11.5 * Ctrl
10355806	Tuba4a	NM_009447	9.96669e-006	-1.6274	E11.5 * Sox10 down vs E11.5 * Ctrl
10474419	Lgr4	NM_172671	1.33606e-005	-1.6277	E11.5 * Sox10 down vs E11.5 * Ctrl
10385883	Slc22a21	NM_019723	8.52215e-005	-1.6283	E11.5 * Sox10 down vs E11.5 * Ctrl
10560242	C5ar1	NM_007577	0.000725992	-1.63011	E11.5 * Sox10 down vs E11.5 * Ctrl
10463263	Lztfl1	NM_033322	0.000631176	-1.63016	E11.5 * Sox10 down vs E11.5 * Ctrl
10482866	Tanc1	NM_198294	1.74614e-006	-1.63128	E11.5 * Sox10 down vs E11.5 * Ctrl
10495830	Sec24d	NM_027135	1.30124e-005	-1.6323	E11.5 * Sox10 down vs E11.5 * Ctrl
10483110	Ifih1	NM_027835	1.1349e-005	-1.63267	E11.5 * Sox10 down vs E11.5 * Ctrl
10362811	Sesn1	NM_001013370	1.15995e-006	-1.63272	E11.5 * Sox10 down vs E11.5 * Ctrl
10559558	Tnni3	NM_009406	1.29316e-006	-1.63273	E11.5 * Sox10 down vs E11.5 * Ctrl
10511333	Plag1	NM_019969	0.00010762	-1.6343	E11.5 * Sox10 down vs E11.5 * Ctrl
10497051	Negr1	NM_001039094	0.000179179	-1.63431	E11.5 * Sox10 down vs E11.5 * Ctrl
10547621	Apobec1	NM_031159	3.29779e-007	-1.6354	E11.5 * Sox10 down vs E11.5 * Ctrl
10474642	BC052040	NM_001145898	2.62723e-005	-1.63622	E11.5 * Sox10 down vs E11.5 * Ctrl
10531195	Adamts3	NM_001081401	0.0089403	-1.63678	E11.5 * Sox10 down vs E11.5 * Ctrl
10491601	4932438A13Rik	NM_172679	0.00556376	-1.63687	E11.5 * Sox10 down vs E11.5 * Ctrl
10467068	Sgms1	NM_144792	3.80362e-006	-1.63738	E11.5 * Sox10 down vs E11.5 * Ctrl
10599696	Ddx26b	NM_172779	2.89596e-005	-1.63847	E11.5 * Sox10 down vs E11.5 * Ctrl
10422844	Gdnf	NM_010275	6.51268e-006	-1.63879	E11.5 * Sox10 down vs E11.5 * Ctrl
10554140	Zic4	BC116191	3.16224e-006	-1.64066	E11.5 * Sox10 down vs E11.5 * Ctrl
10392177	Icam2	NM_010494	1.18973e-006	-1.64069	E11.5 * Sox10 down vs E11.5 * Ctrl
10452980	Eif2ak2	NM_011163	0.000139975	-1.64069	E11.5 * Sox10 down vs E11.5 * Ctrl
10591114	Fat3	NM_001080814	0.00105773	-1.64125	E11.5 * Sox10 down vs E11.5 * Ctrl
10555323	P4ha3	NM_177161	9.83717e-005	-1.64362	E11.5 * Sox10 down vs E11.5 * Ctrl
10464107	Ppnr	X65588	0.000134622	-1.6439	E11.5 * Sox10 down vs E11.5 * Ctrl
10368720	Slc16a10	NM_001114332	4.76335e-008	-1.64545	E11.5 * Sox10 down vs E11.5 * Ctrl
10455123	Pcdhb19	NM_053144	0.000140904	-1.64573	E11.5 * Sox10 down vs E11.5 * Ctrl
10541564	Clec4a3	NM_153197	2.69381e-005	-1.64949	E11.5 * Sox10 down vs E11.5 * Ctrl
10385118	Dock2	NM_033374	0.00010808	-1.65032	E11.5 * Sox10 down vs E11.5 * Ctrl
10586454	D030028M11Rik	AK050877	3.46615e-005	-1.65157	E11.5 * Sox10 down vs E11.5 * Ctrl
10600326	Tktl1	NM_031379	0.00127634	-1.65427	E11.5 * Sox10 down vs E11.5 * Ctrl
10473022	Plp2	NM_019755	0.000625174	-1.65447	E11.5 * Sox10 down vs E11.5 * Ctrl
10541587	Clec4a2	NM_011999	0.000478213	-1.65552	E11.5 * Sox10 down vs E11.5 * Ctrl
10521616	C1qtnf7	NM_001135172	7.00975e-006	-1.65565	E11.5 * Sox10 down vs E11.5 * Ctrl
10395606	ENSMUSG0000C	ENSMUST00000	0.000679902	-1.65621	E11.5 * Sox10 down vs E11.5 * Ctrl
10538715	C130092O11Rik	BC107398	1.44305e-005	-1.65707	E11.5 * Sox10 down vs E11.5 * Ctrl
10448117	Has1	NM_008215	5.91283e-005	-1.65719	E11.5 * Sox10 down vs E11.5 * Ctrl
10589886	4930520O04Rik	AK040958	6.30066e-005	-1.6572	E11.5 * Sox10 down vs E11.5 * Ctrl
10401149	Plek2	NM_013738	1.08492e-005	-1.65781	E11.5 * Sox10 down vs E11.5 * Ctrl
10436449		---	2.19575e-005	-1.65795	E11.5 * Sox10 down vs E11.5 * Ctrl
10493604	She	NM_172530	1.10903e-006	-1.65797	E11.5 * Sox10 down vs E11.5 * Ctrl
10397575	Nrxn3	NM_172544	3.16426e-006	-1.65827	E11.5 * Sox10 down vs E11.5 * Ctrl
10600308		---	0.00919149	-1.65866	E11.5 * Sox10 down vs E11.5 * Ctrl
10584315		---	0.0029533	-1.65981	E11.5 * Sox10 down vs E11.5 * Ctrl
10601880	BC065397	ENSMUST00000	0.000196475	-1.65993	E11.5 * Sox10 down vs E11.5 * Ctrl

10388958	Evi2a	NM_001033711	0.000405408	-1.6606	E11.5 * Sox10 down vs E11.5 * Ctrl
10420668		---	0.00336355	-1.66216	E11.5 * Sox10 down vs E11.5 * Ctrl
10422013	Klf12	NM_010636	1.17883e-005	-1.66278	E11.5 * Sox10 down vs E11.5 * Ctrl
10409978	EG630579	NM_001039239	5.53439e-005	-1.66329	E11.5 * Sox10 down vs E11.5 * Ctrl
10356333	Snord82	NR_002851	0.000105052	-1.66346	E11.5 * Sox10 down vs E11.5 * Ctrl
10570634	4930467E23Rik	ENSMUST00000(	0.00642825	-1.664	E11.5 * Sox10 down vs E11.5 * Ctrl
10487011	Gatm	NM_025961	2.13449e-006	-1.66419	E11.5 * Sox10 down vs E11.5 * Ctrl
10596115	Ephb1	AK082061	0.000118749	-1.66485	E11.5 * Sox10 down vs E11.5 * Ctrl
10512327	4933409K07Rik	BC072647	1.53074e-006	-1.66556	E11.5 * Sox10 down vs E11.5 * Ctrl
10440344	Robo2	NM_175549	2.47891e-005	-1.66751	E11.5 * Sox10 down vs E11.5 * Ctrl
10435641	Fstl1	NM_008047	3.33591e-007	-1.66837	E11.5 * Sox10 down vs E11.5 * Ctrl
10508012	Rspo1	NM_138683	5.3075e-005	-1.66848	E11.5 * Sox10 down vs E11.5 * Ctrl
10423109	Adamts12	NM_175501	5.48025e-008	-1.66858	E11.5 * Sox10 down vs E11.5 * Ctrl
10439710	Phldb2	NM_153412	2.37878e-009	-1.66922	E11.5 * Sox10 down vs E11.5 * Ctrl
10408898		---	0.0004532	-1.66947	E11.5 * Sox10 down vs E11.5 * Ctrl
10563077	Rcn3	NM_026555	0.00418298	-1.6707	E11.5 * Sox10 down vs E11.5 * Ctrl
10562709	Cd33	NM_001111058	1.31051e-007	-1.67165	E11.5 * Sox10 down vs E11.5 * Ctrl
10360834	Hlx	NM_008250	2.49887e-006	-1.67184	E11.5 * Sox10 down vs E11.5 * Ctrl
10358389	Rgs2	NM_009061	2.10468e-005	-1.67185	E11.5 * Sox10 down vs E11.5 * Ctrl
10416785	4930517O19Rik	ENSMUST00000(	7.59379e-006	-1.67209	E11.5 * Sox10 down vs E11.5 * Ctrl
10498386	Igsf10	ENSMUST00000(	3.77218e-007	-1.67243	E11.5 * Sox10 down vs E11.5 * Ctrl
10598004	Ccr1	NM_009912	5.77915e-005	-1.67302	E11.5 * Sox10 down vs E11.5 * Ctrl
10521678	Cd38	NM_007646	6.12335e-007	-1.67326	E11.5 * Sox10 down vs E11.5 * Ctrl
10474371	ENSMUSG0000C	ENSMUST00000	7.54794e-006	-1.67459	E11.5 * Sox10 down vs E11.5 * Ctrl
10568601	BC002195	BC002195	0.0096574	-1.67557	E11.5 * Sox10 down vs E11.5 * Ctrl
10398338		---	0.000937242	-1.67737	E11.5 * Sox10 down vs E11.5 * Ctrl
10439612	Boc	NM_172506	4.18472e-007	-1.678	E11.5 * Sox10 down vs E11.5 * Ctrl
10367945	Phactr2	NM_001033257	0.000192522	-1.67814	E11.5 * Sox10 down vs E11.5 * Ctrl
10579060	4930467E23Rik	ENSMUST00000(	0.00677848	-1.67962	E11.5 * Sox10 down vs E11.5 * Ctrl
10441093	Erg	NM_133659	5.05341e-008	-1.67971	E11.5 * Sox10 down vs E11.5 * Ctrl
10515981		---	0.00120248	-1.68127	E11.5 * Sox10 down vs E11.5 * Ctrl
10543791	Podxl	NM_013723	1.51474e-005	-1.68155	E11.5 * Sox10 down vs E11.5 * Ctrl
10427280	Hoxc10	NM_010462	1.02893e-005	-1.68195	E11.5 * Sox10 down vs E11.5 * Ctrl
10551347	Blvrb	NM_144923	3.41247e-007	-1.68208	E11.5 * Sox10 down vs E11.5 * Ctrl
10354576	Dnahc7a	ENSMUST00000(	0.00258431	-1.68271	E11.5 * Sox10 down vs E11.5 * Ctrl
10531173	Adamts3	NM_001081401	0.000173405	-1.68336	E11.5 * Sox10 down vs E11.5 * Ctrl
10413808		---	0.00995403	-1.68423	E11.5 * Sox10 down vs E11.5 * Ctrl
10400170	Prkd1	NM_008858	5.41606e-006	-1.68425	E11.5 * Sox10 down vs E11.5 * Ctrl
10483081	Fap	NM_007986	1.32966e-005	-1.68572	E11.5 * Sox10 down vs E11.5 * Ctrl
10383204		---	0.00198087	-1.68619	E11.5 * Sox10 down vs E11.5 * Ctrl
10404885	Gmpr	NM_025508	6.84006e-008	-1.68684	E11.5 * Sox10 down vs E11.5 * Ctrl
10369752	Lrrtm3	NM_178678	6.54484e-005	-1.68694	E11.5 * Sox10 down vs E11.5 * Ctrl
10434782	Lpp	NM_178665	4.35649e-006	-1.68704	E11.5 * Sox10 down vs E11.5 * Ctrl
10361926	Map3k5	NM_008580	4.5703e-005	-1.68917	E11.5 * Sox10 down vs E11.5 * Ctrl
10603182	Arhgap6	NM_009707	6.10847e-005	-1.69012	E11.5 * Sox10 down vs E11.5 * Ctrl
10548139		---	1.05813e-005	-1.69055	E11.5 * Sox10 down vs E11.5 * Ctrl
10361186	Sertad4	NM_198247	1.11887e-007	-1.69084	E11.5 * Sox10 down vs E11.5 * Ctrl
10497265	Fabp4	NM_024406	0.00143297	-1.69088	E11.5 * Sox10 down vs E11.5 * Ctrl
10600355	Snora70	NR_002899	2.92286e-007	-1.69159	E11.5 * Sox10 down vs E11.5 * Ctrl
10463875	Sorcs3	NM_025696	6.73594e-006	-1.69262	E11.5 * Sox10 down vs E11.5 * Ctrl
10554969	Odz4	NM_011858	1.38856e-005	-1.69344	E11.5 * Sox10 down vs E11.5 * Ctrl
10498383	Igsf10	ENSMUST00000(	3.14796e-005	-1.69415	E11.5 * Sox10 down vs E11.5 * Ctrl
10528815	5031410I06Rik	NM_207657	5.88894e-007	-1.69533	E11.5 * Sox10 down vs E11.5 * Ctrl
10543273	Cttnbp2	NM_080285	2.36731e-005	-1.69565	E11.5 * Sox10 down vs E11.5 * Ctrl
10503659	Epha7	NM_010141	4.39285e-009	-1.6973	E11.5 * Sox10 down vs E11.5 * Ctrl
10579054	4930467E23Rik	ENSMUST00000(	0.00402345	-1.69769	E11.5 * Sox10 down vs E11.5 * Ctrl
10386211	3100002J23Rik	ENSMUST00000(	0.00160739	-1.69769	E11.5 * Sox10 down vs E11.5 * Ctrl
10472846	Pdk1	NM_172665	0.000915985	-1.69783	E11.5 * Sox10 down vs E11.5 * Ctrl
10498972	Rbm46	NM_001146328	0.000100467	-1.69812	E11.5 * Sox10 down vs E11.5 * Ctrl
10584229		---	0.000514982	-1.69817	E11.5 * Sox10 down vs E11.5 * Ctrl
10485633	ENSMUSG0000C	ENSMUST00000(	0.004708	-1.69829	E11.5 * Sox10 down vs E11.5 * Ctrl
10603346	Plp2	NM_019755	2.49116e-005	-1.69845	E11.5 * Sox10 down vs E11.5 * Ctrl
10485643	AI314831	NR_015462	0.000741288	-1.6992	E11.5 * Sox10 down vs E11.5 * Ctrl
10504172	4933409K07Rik	BC059060	3.92248e-007	-1.69962	E11.5 * Sox10 down vs E11.5 * Ctrl
10504169	4933409K07Rik	BC072647	6.56106e-007	-1.69998	E11.5 * Sox10 down vs E11.5 * Ctrl
10483046	Dpp4	NM_010074	8.00732e-005	-1.70035	E11.5 * Sox10 down vs E11.5 * Ctrl

10427816	Pdzd2	NM_001081064	2.48708e-006	-1.70093	E11.5 * Sox10 down vs E11.5 * Ctrl
10517508	C1qb	NM_009777	0.00311665	-1.70097	E11.5 * Sox10 down vs E11.5 * Ctrl
10450496	Lst1	NM_010734	3.70022e-005	-1.70152	E11.5 * Sox10 down vs E11.5 * Ctrl
10504139	4933409K07Rik	BC059060	7.46733e-007	-1.70174	E11.5 * Sox10 down vs E11.5 * Ctrl
10498852	Rxfp1	NM_212452	4.25435e-007	-1.70273	E11.5 * Sox10 down vs E11.5 * Ctrl
10353731	4930444G20Rik	NM_053264	0.00152263	-1.7037	E11.5 * Sox10 down vs E11.5 * Ctrl
10375145	Lcp2	NM_010696	3.58986e-005	-1.70394	E11.5 * Sox10 down vs E11.5 * Ctrl
10406598	Serinc5	NM_172588	0.000234811	-1.70505	E11.5 * Sox10 down vs E11.5 * Ctrl
10352576	Esrrg	NM_011935	3.5855e-006	-1.70515	E11.5 * Sox10 down vs E11.5 * Ctrl
10531928		---	0.000723585	-1.7059	E11.5 * Sox10 down vs E11.5 * Ctrl
10559248	Tspan32	NM_020286	1.92484e-008	-1.70694	E11.5 * Sox10 down vs E11.5 * Ctrl
10400304	Egln3	NM_028133	0.000751966	-1.70812	E11.5 * Sox10 down vs E11.5 * Ctrl
10363860	Slc16a9	NM_025807	0.000141871	-1.71033	E11.5 * Sox10 down vs E11.5 * Ctrl
10377938	Eno3	NM_007933	1.36236e-006	-1.71131	E11.5 * Sox10 down vs E11.5 * Ctrl
10492438	EG624866	NM_001037923	5.35566e-006	-1.71178	E11.5 * Sox10 down vs E11.5 * Ctrl
10597074		---	5.26535e-008	-1.71221	E11.5 * Sox10 down vs E11.5 * Ctrl
10403945	LOC100041230	BC139425	0.00824568	-1.71234	E11.5 * Sox10 down vs E11.5 * Ctrl
10504178	4933409K07Rik	BC059060	2.10751e-007	-1.7128	E11.5 * Sox10 down vs E11.5 * Ctrl
10406782	Fam169a	NM_001100458	0.000581333	-1.71286	E11.5 * Sox10 down vs E11.5 * Ctrl
10496182	Cxxc4	NM_001004367	4.42645e-007	-1.71416	E11.5 * Sox10 down vs E11.5 * Ctrl
10535936		---	8.17238e-005	-1.71652	E11.5 * Sox10 down vs E11.5 * Ctrl
10563085	Fcgrt	NM_010189	3.6198e-009	-1.71745	E11.5 * Sox10 down vs E11.5 * Ctrl
10566366	AI451617	NM_199146	0.000475827	-1.71763	E11.5 * Sox10 down vs E11.5 * Ctrl
10546452	Adamts9	NM_175314	1.53559e-008	-1.71809	E11.5 * Sox10 down vs E11.5 * Ctrl
10604694	Mtap7d3	NM_177293	0.000325354	-1.71878	E11.5 * Sox10 down vs E11.5 * Ctrl
10576911	Efnb2	NM_010111	3.84095e-006	-1.71891	E11.5 * Sox10 down vs E11.5 * Ctrl
10398356		---	8.74884e-005	-1.71902	E11.5 * Sox10 down vs E11.5 * Ctrl
10360377	AI607873	BC150711	0.000650853	-1.72038	E11.5 * Sox10 down vs E11.5 * Ctrl
10357137	Gli2	NM_001081125	2.43314e-006	-1.72172	E11.5 * Sox10 down vs E11.5 * Ctrl
10415413	BC030046	BC151043	7.27948e-006	-1.72196	E11.5 * Sox10 down vs E11.5 * Ctrl
10504148	4933409K07Rik	BC059060	5.87185e-007	-1.72321	E11.5 * Sox10 down vs E11.5 * Ctrl
10509441	Ece1	NM_199307	6.4927e-005	-1.7233	E11.5 * Sox10 down vs E11.5 * Ctrl
10496251	Bdh2	NM_027208	1.40287e-006	-1.7236	E11.5 * Sox10 down vs E11.5 * Ctrl
10598976	Timp1	NM_001044384	1.94011e-005	-1.72419	E11.5 * Sox10 down vs E11.5 * Ctrl
10502830	Nexn	NM_199465	0.000115022	-1.72439	E11.5 * Sox10 down vs E11.5 * Ctrl
10495416	Vav3	NM_020505	8.6185e-006	-1.72581	E11.5 * Sox10 down vs E11.5 * Ctrl
10461369	Ahnak	NM_009643	2.66753e-006	-1.72684	E11.5 * Sox10 down vs E11.5 * Ctrl
10496569	Gbp6	NM_145545	4.26755e-006	-1.72847	E11.5 * Sox10 down vs E11.5 * Ctrl
10538305	5730596B20Rik	ENSMUST00000(	6.88183e-005	-1.72921	E11.5 * Sox10 down vs E11.5 * Ctrl
10462922	Plce1	NM_019588	2.33196e-009	-1.72925	E11.5 * Sox10 down vs E11.5 * Ctrl
10451613	Foxp4	NM_001110824	1.52537e-005	-1.73086	E11.5 * Sox10 down vs E11.5 * Ctrl
10368317	Enpp3	NM_134005	5.89897e-008	-1.73246	E11.5 * Sox10 down vs E11.5 * Ctrl
10425749	Fam109b	NM_177391	2.63977e-005	-1.73278	E11.5 * Sox10 down vs E11.5 * Ctrl
10577388	4930467E23Rik	ENSMUST00000(	0.00147295	-1.73325	E11.5 * Sox10 down vs E11.5 * Ctrl
10416734		---	0.000137496	-1.73325	E11.5 * Sox10 down vs E11.5 * Ctrl
10416974	Gpc6	NM_001079844	1.65798e-006	-1.73365	E11.5 * Sox10 down vs E11.5 * Ctrl
10502451	Bmpr1b	NM_007560	4.47186e-005	-1.73696	E11.5 * Sox10 down vs E11.5 * Ctrl
10459391	Fech	NM_007998	1.16565e-007	-1.73813	E11.5 * Sox10 down vs E11.5 * Ctrl
10462853	Cyp26a1	NM_007811	6.2725e-007	-1.73944	E11.5 * Sox10 down vs E11.5 * Ctrl
10435961	ENSMUSG0000C	ENSMUST00000(	1.4536e-005	-1.74013	E11.5 * Sox10 down vs E11.5 * Ctrl
10553450	Nell1	NM_001037906	5.73351e-005	-1.7402	E11.5 * Sox10 down vs E11.5 * Ctrl
10420891	Scara3	NM_172604	2.58578e-005	-1.74142	E11.5 * Sox10 down vs E11.5 * Ctrl
10497451	Cpa3	NM_007753	0.00307409	-1.7418	E11.5 * Sox10 down vs E11.5 * Ctrl
10577361			0.000213802	-1.74212	E11.5 * Sox10 down vs E11.5 * Ctrl
10513208	Svep1	NM_022814	1.38915e-007	-1.74466	E11.5 * Sox10 down vs E11.5 * Ctrl
10430555	Dmc1	NM_010059	0.000719373	-1.7448	E11.5 * Sox10 down vs E11.5 * Ctrl
10422496	Gpr183	NM_183031	1.09232e-006	-1.74523	E11.5 * Sox10 down vs E11.5 * Ctrl
10545086	Snca	NM_001042451	2.14974e-005	-1.74524	E11.5 * Sox10 down vs E11.5 * Ctrl
10542355	Emp1	NM_010128	0.000116814	-1.7463	E11.5 * Sox10 down vs E11.5 * Ctrl
10411359	Plp2	NM_019755	5.03531e-005	-1.74761	E11.5 * Sox10 down vs E11.5 * Ctrl
10593198	Fam55b	NM_030069	5.06794e-005	-1.74766	E11.5 * Sox10 down vs E11.5 * Ctrl
10522060	Fam114a1	NM_026667	9.89213e-006	-1.74915	E11.5 * Sox10 down vs E11.5 * Ctrl
10455108	Pcdhb16	NM_053141	0.00121083	-1.74944	E11.5 * Sox10 down vs E11.5 * Ctrl
10491414	Usp13	NM_001013024	9.08688e-006	-1.74987	E11.5 * Sox10 down vs E11.5 * Ctrl
10444258	Psmb8	NM_010724	1.13128e-005	-1.75004	E11.5 * Sox10 down vs E11.5 * Ctrl
10398352		---	0.000915864	-1.75066	E11.5 * Sox10 down vs E11.5 * Ctrl

10582388		---	0.00113821	-1.75237	E11.5 * Sox10 down vs E11.5 * Ctrl
10492428	Tiparp	NM_178892	4.94201e-007	-1.753	E11.5 * Sox10 down vs E11.5 * Ctrl
10591090	Fat3	NM_001080814	3.74694e-005	-1.75404	E11.5 * Sox10 down vs E11.5 * Ctrl
10549282	Itpr2	NM_019923	8.73622e-009	-1.75435	E11.5 * Sox10 down vs E11.5 * Ctrl
10571444	Slc7a2	NM_007514	3.85923e-005	-1.75453	E11.5 * Sox10 down vs E11.5 * Ctrl
10514219	Scarna8	AF357402	0.0002887	-1.75462	E11.5 * Sox10 down vs E11.5 * Ctrl
10467258	Myof	NM_001099634	2.32427e-007	-1.75484	E11.5 * Sox10 down vs E11.5 * Ctrl
10379511	Ccl2	NM_011333	9.97387e-008	-1.75512	E11.5 * Sox10 down vs E11.5 * Ctrl
10472398	Scn2a1	NM_001099298	0.0098356	-1.7557	E11.5 * Sox10 down vs E11.5 * Ctrl
10410743	Ankrd32	NM_134071	2.18544e-005	-1.75592	E11.5 * Sox10 down vs E11.5 * Ctrl
10488378	Thbd	NM_009378	0.000186473	-1.75593	E11.5 * Sox10 down vs E11.5 * Ctrl
10408280	Lrrc16a	NM_026825	8.02464e-008	-1.75597	E11.5 * Sox10 down vs E11.5 * Ctrl
10390746		---	0.000234568	-1.75796	E11.5 * Sox10 down vs E11.5 * Ctrl
10353729	4930444G20Rik	NM_053264	0.00161449	-1.75925	E11.5 * Sox10 down vs E11.5 * Ctrl
10582464		---	0.00776706	-1.76171	E11.5 * Sox10 down vs E11.5 * Ctrl
10368566	Tpd52l1	NM_009413	0.000108444	-1.76248	E11.5 * Sox10 down vs E11.5 * Ctrl
10606600	Pcdh19	NM_001105245	6.29613e-006	-1.76363	E11.5 * Sox10 down vs E11.5 * Ctrl
10488147	Flrt3	NM_178382	2.42314e-007	-1.76377	E11.5 * Sox10 down vs E11.5 * Ctrl
10399691	Id2	NM_010496	8.4213e-007	-1.76613	E11.5 * Sox10 down vs E11.5 * Ctrl
10352448	Dusp10	NM_022019	8.91989e-006	-1.76639	E11.5 * Sox10 down vs E11.5 * Ctrl
10492888		---	5.1331e-005	-1.76714	E11.5 * Sox10 down vs E11.5 * Ctrl
10606770	Zmat1	NM_175446	1.17636e-005	-1.76844	E11.5 * Sox10 down vs E11.5 * Ctrl
10565712	Acer3	NM_025408	8.53922e-006	-1.76914	E11.5 * Sox10 down vs E11.5 * Ctrl
10418506	Stab1	NM_138672	3.01378e-008	-1.76957	E11.5 * Sox10 down vs E11.5 * Ctrl
10408812	Mak	NM_001145803	2.49275e-005	-1.76985	E11.5 * Sox10 down vs E11.5 * Ctrl
10443949	Adamts10	NM_172619	7.09532e-006	-1.7701	E11.5 * Sox10 down vs E11.5 * Ctrl
10345762	Il1r1	NM_008362	1.43081e-005	-1.77023	E11.5 * Sox10 down vs E11.5 * Ctrl
10462818	Hhex	NM_008245	0.000134497	-1.77079	E11.5 * Sox10 down vs E11.5 * Ctrl
10404059	Hist1h1c	NM_015786	5.33371e-006	-1.7714	E11.5 * Sox10 down vs E11.5 * Ctrl
10603573	Sytl5	NM_177704	2.56715e-006	-1.77395	E11.5 * Sox10 down vs E11.5 * Ctrl
10470562	ENSMUSG0000C	ENSMUST00000(	0.000161474	-1.77448	E11.5 * Sox10 down vs E11.5 * Ctrl
10354598	Hecw2	NM_001001883	1.22333e-006	-1.77518	E11.5 * Sox10 down vs E11.5 * Ctrl
10570585		---	0.000736845	-1.77638	E11.5 * Sox10 down vs E11.5 * Ctrl
10538706	Mmrn1	BC137623	0.00024419	-1.7765	E11.5 * Sox10 down vs E11.5 * Ctrl
10539894	Mgll	NM_011844	5.66954e-005	-1.77682	E11.5 * Sox10 down vs E11.5 * Ctrl
10503835	Rragd	NM_027491	9.0912e-007	-1.77783	E11.5 * Sox10 down vs E11.5 * Ctrl
10453797	AK220484	NM_001083628	3.7801e-006	-1.77838	E11.5 * Sox10 down vs E11.5 * Ctrl
10604608		---	0.000132715	-1.77936	E11.5 * Sox10 down vs E11.5 * Ctrl
10490856	Ralyl	NM_178631	5.28424e-006	-1.78204	E11.5 * Sox10 down vs E11.5 * Ctrl
10502081	Enpep	NM_007934	8.74352e-007	-1.78275	E11.5 * Sox10 down vs E11.5 * Ctrl
10542993	Pon3	NM_173006	1.3317e-007	-1.78373	E11.5 * Sox10 down vs E11.5 * Ctrl
10418410	Prkcd	NM_011103	2.14281e-006	-1.78634	E11.5 * Sox10 down vs E11.5 * Ctrl
10398442		---	0.00282509	-1.78858	E11.5 * Sox10 down vs E11.5 * Ctrl
10526797	Stag3	NM_016964	2.45119e-007	-1.7888	E11.5 * Sox10 down vs E11.5 * Ctrl
10399924	Pik3cg	NM_020272	1.58052e-006	-1.78932	E11.5 * Sox10 down vs E11.5 * Ctrl
10435697	Popdc2	NM_001081984	9.15862e-006	-1.78961	E11.5 * Sox10 down vs E11.5 * Ctrl
10414537	Ang	NM_007447	7.16612e-006	-1.79075	E11.5 * Sox10 down vs E11.5 * Ctrl
10433782	Efcab1	NM_025769	1.9148e-006	-1.79152	E11.5 * Sox10 down vs E11.5 * Ctrl
10353989		---	0.00174506	-1.79199	E11.5 * Sox10 down vs E11.5 * Ctrl
10579894	Hhip	NM_020259	0.000192214	-1.79215	E11.5 * Sox10 down vs E11.5 * Ctrl
10455139	Pcdhb22	NM_053147	1.16007e-005	-1.79234	E11.5 * Sox10 down vs E11.5 * Ctrl
10607945	4933400A11Rik	NR_003635	3.19452e-005	-1.79451	E11.5 * Sox10 down vs E11.5 * Ctrl
10384656	B3gnt2	NM_016888	1.68953e-005	-1.79476	E11.5 * Sox10 down vs E11.5 * Ctrl
10354563	Dnahc7b	ENSMUST00000(	8.08591e-005	-1.79562	E11.5 * Sox10 down vs E11.5 * Ctrl
10578989	Psd3	NM_177698	2.8095e-006	-1.79586	E11.5 * Sox10 down vs E11.5 * Ctrl
10604944	Gabre	NM_017369	1.32793e-005	-1.79752	E11.5 * Sox10 down vs E11.5 * Ctrl
10352548	Slc30a10	NM_001033286	5.89285e-006	-1.79842	E11.5 * Sox10 down vs E11.5 * Ctrl
10544002	Creb3l2	NM_178661	1.98628e-008	-1.79844	E11.5 * Sox10 down vs E11.5 * Ctrl
10441003	Runx1	NM_001111023	1.59217e-005	-1.79874	E11.5 * Sox10 down vs E11.5 * Ctrl
10504137	4933409K07Rik	BC072647	1.5758e-005	-1.80085	E11.5 * Sox10 down vs E11.5 * Ctrl
10504201	4933409K07Rik	BC072647	1.5758e-005	-1.80085	E11.5 * Sox10 down vs E11.5 * Ctrl
10512350	4933409K07Rik	BC072647	1.5758e-005	-1.80085	E11.5 * Sox10 down vs E11.5 * Ctrl
10512352	4933409K07Rik	BC072647	1.5758e-005	-1.80085	E11.5 * Sox10 down vs E11.5 * Ctrl
10424082	Aard	NM_175503	1.90898e-005	-1.80267	E11.5 * Sox10 down vs E11.5 * Ctrl
10430929	Tbrg3	BC095996	6.28263e-005	-1.80403	E11.5 * Sox10 down vs E11.5 * Ctrl
10373223	Lrp1	NM_008512	8.37261e-005	-1.80424	E11.5 * Sox10 down vs E11.5 * Ctrl

10389134	Slfn9	NM_172796	3.52002e-008	-1.80459	E11.5 * Sox10 down vs E11.5 * Ctrl
10500335	Fcgr1	NM_010186	1.2463e-005	-1.80463	E11.5 * Sox10 down vs E11.5 * Ctrl
10361270	Cd46	NM_010778	8.81664e-005	-1.80672	E11.5 * Sox10 down vs E11.5 * Ctrl
10536472	Mdfic	NM_175088	1.37072e-005	-1.80824	E11.5 * Sox10 down vs E11.5 * Ctrl
10439312	Cd86	NM_019388	0.000819511	-1.8094	E11.5 * Sox10 down vs E11.5 * Ctrl
10505674	Cntln	NM_175275	1.30615e-010	-1.80968	E11.5 * Sox10 down vs E11.5 * Ctrl
10599174	Il13ra1	NM_133990	2.81199e-007	-1.81018	E11.5 * Sox10 down vs E11.5 * Ctrl
10605493	Prrg1	BC032926	2.73226e-006	-1.81022	E11.5 * Sox10 down vs E11.5 * Ctrl
10473384	Slc43a3	NM_021398	4.03344e-007	-1.81055	E11.5 * Sox10 down vs E11.5 * Ctrl
10513166	Ptpn3	NM_011207	3.77199e-006	-1.81093	E11.5 * Sox10 down vs E11.5 * Ctrl
10359201	Ralgps2	NM_023884	3.81201e-011	-1.813	E11.5 * Sox10 down vs E11.5 * Ctrl
10450145	Psmb9	NM_013585	7.48258e-006	-1.81388	E11.5 * Sox10 down vs E11.5 * Ctrl
10436590	2810055G20Rik	AK148800	0.000254653	-1.81405	E11.5 * Sox10 down vs E11.5 * Ctrl
10409994	EG435366	ENSMUST00000	7.71775e-005	-1.81428	E11.5 * Sox10 down vs E11.5 * Ctrl
10404649	Dsp	NM_023842	6.71116e-006	-1.81496	E11.5 * Sox10 down vs E11.5 * Ctrl
10569618	Ano1	NM_178642	9.2024e-005	-1.81618	E11.5 * Sox10 down vs E11.5 * Ctrl
10365559	Igf1	NM_010512	7.44718e-006	-1.81635	E11.5 * Sox10 down vs E11.5 * Ctrl
10477717	Procr	NM_011171	1.79385e-005	-1.8166	E11.5 * Sox10 down vs E11.5 * Ctrl
10494114	Selenbp1	NM_009150	6.71609e-005	-1.81665	E11.5 * Sox10 down vs E11.5 * Ctrl
10484307	Frzb	NM_011356	2.98191e-005	-1.81977	E11.5 * Sox10 down vs E11.5 * Ctrl
10419465	Ccnb1ip1	NM_001111119	1.20678e-006	-1.82074	E11.5 * Sox10 down vs E11.5 * Ctrl
10591978	Ntm	NM_172290	3.8023e-006	-1.82296	E11.5 * Sox10 down vs E11.5 * Ctrl
10521759	Slit2	NM_178804	1.69006e-007	-1.82355	E11.5 * Sox10 down vs E11.5 * Ctrl
10579812	Ednra	NM_010332	5.14075e-007	-1.82449	E11.5 * Sox10 down vs E11.5 * Ctrl
10583203	Phxr4	BC107288	0.00158176	-1.82696	E11.5 * Sox10 down vs E11.5 * Ctrl
10428522	Csmd3	NM_001081391	0.000280293	-1.82729	E11.5 * Sox10 down vs E11.5 * Ctrl
10439895	Alcam	NM_009655	5.02401e-006	-1.82735	E11.5 * Sox10 down vs E11.5 * Ctrl
10489569	Pltp	NM_011125	2.75256e-008	-1.82817	E11.5 * Sox10 down vs E11.5 * Ctrl
10455647	Tnfaip8	NM_134131	5.61469e-005	-1.82947	E11.5 * Sox10 down vs E11.5 * Ctrl
10394731	Rock2	ENSMUST00000	3.18359e-005	-1.83039	E11.5 * Sox10 down vs E11.5 * Ctrl
10380629	Hoxb8	NM_010461	2.4057e-007	-1.83099	E11.5 * Sox10 down vs E11.5 * Ctrl
10502783	ENSMUSG0000C	ENSMUST00000	0.000350742	-1.83108	E11.5 * Sox10 down vs E11.5 * Ctrl
10583809	Cnn1	NM_009922	0.00106866	-1.83159	E11.5 * Sox10 down vs E11.5 * Ctrl
10582466	Sult5a1	NM_020564	1.84819e-007	-1.83176	E11.5 * Sox10 down vs E11.5 * Ctrl
10556611		---	0.00179954	-1.83209	E11.5 * Sox10 down vs E11.5 * Ctrl
10396950		---	4.11079e-006	-1.83284	E11.5 * Sox10 down vs E11.5 * Ctrl
10456296	Malt1	NM_172833	1.57564e-006	-1.83377	E11.5 * Sox10 down vs E11.5 * Ctrl
10397633	Flrt2	NM_201518	2.24469e-006	-1.83435	E11.5 * Sox10 down vs E11.5 * Ctrl
10495976	Pitx2	NM_011098	1.50803e-007	-1.83497	E11.5 * Sox10 down vs E11.5 * Ctrl
10580955		---	0.00316127	-1.83507	E11.5 * Sox10 down vs E11.5 * Ctrl
10366746	Lrig3	NM_177152	2.59256e-005	-1.83672	E11.5 * Sox10 down vs E11.5 * Ctrl
10529824	Prom1	NM_008935	8.65226e-009	-1.83717	E11.5 * Sox10 down vs E11.5 * Ctrl
10581605	Hp	NM_017370	0.000196721	-1.83725	E11.5 * Sox10 down vs E11.5 * Ctrl
10384423	Cobl	NM_172496	2.46947e-007	-1.84132	E11.5 * Sox10 down vs E11.5 * Ctrl
10570963	Zmat4	NM_177086	6.05585e-007	-1.84151	E11.5 * Sox10 down vs E11.5 * Ctrl
10590631	Ccr2	NM_009915	4.25065e-005	-1.84201	E11.5 * Sox10 down vs E11.5 * Ctrl
10344750	Sgk3	NM_133220	0.000700638	-1.84202	E11.5 * Sox10 down vs E11.5 * Ctrl
10423568	5730407I07Rik	ENSMUST00000	2.40927e-005	-1.84204	E11.5 * Sox10 down vs E11.5 * Ctrl
10600852		---	1.37714e-005	-1.84268	E11.5 * Sox10 down vs E11.5 * Ctrl
10344935	Kcnb2	NM_001098528	2.06256e-005	-1.84296	E11.5 * Sox10 down vs E11.5 * Ctrl
10467041	Asah2	NM_018830	6.48851e-008	-1.8431	E11.5 * Sox10 down vs E11.5 * Ctrl
10531191	Adamts3	NM_001081401	0.00128709	-1.84338	E11.5 * Sox10 down vs E11.5 * Ctrl
10528794	5031410I06Rik	NM_207657	1.48643e-008	-1.84569	E11.5 * Sox10 down vs E11.5 * Ctrl
10476321	Prnd	NM_023043	6.06498e-005	-1.84612	E11.5 * Sox10 down vs E11.5 * Ctrl
10466606	Anxa1	NM_010730	0.0044298	-1.84718	E11.5 * Sox10 down vs E11.5 * Ctrl
10506201	Ror1	NM_013845	3.25797e-008	-1.84996	E11.5 * Sox10 down vs E11.5 * Ctrl
10368947	Aim1	NM_172393	1.0482e-005	-1.85122	E11.5 * Sox10 down vs E11.5 * Ctrl
10507500	Slc6a9	NM_008135	6.08466e-008	-1.85248	E11.5 * Sox10 down vs E11.5 * Ctrl
10372652	Lyz1	NM_013590	0.000229213	-1.85357	E11.5 * Sox10 down vs E11.5 * Ctrl
10426315	Lrrk2	NM_025730	6.47534e-008	-1.85697	E11.5 * Sox10 down vs E11.5 * Ctrl
10503334	Gem	NM_010276	0.000110439	-1.85737	E11.5 * Sox10 down vs E11.5 * Ctrl
10482517	Nmi	NM_001141949	0.000100521	-1.85744	E11.5 * Sox10 down vs E11.5 * Ctrl
10528810	Speer4a	NM_029376	1.90063e-008	-1.85948	E11.5 * Sox10 down vs E11.5 * Ctrl
10467038	EG625995	BC096400	0.00307968	-1.86039	E11.5 * Sox10 down vs E11.5 * Ctrl
10432918	Krt8	NM_031170	0.000138258	-1.86164	E11.5 * Sox10 down vs E11.5 * Ctrl
10588819	1700021K14Rik	NM_001122635	1.06189e-005	-1.86217	E11.5 * Sox10 down vs E11.5 * Ctrl

10497381	Cyp7b1	NM_007825	2.0443e-007	-1.86226	E11.5 * Sox10 down vs E11.5 * Ctrl
10536845	Flnc	NM_001081185	7.26561e-008	-1.86545	E11.5 * Sox10 down vs E11.5 * Ctrl
10441902	Smoc2	NM_022315	2.05875e-006	-1.8681	E11.5 * Sox10 down vs E11.5 * Ctrl
10594110	Neo1	NM_008684	3.96926e-011	-1.86826	E11.5 * Sox10 down vs E11.5 * Ctrl
10363887	Bicc1	NM_031397	0.000141716	-1.86926	E11.5 * Sox10 down vs E11.5 * Ctrl
10441791	D17Ertd663e	ENSMUST00000	6.70958e-008	-1.86968	E11.5 * Sox10 down vs E11.5 * Ctrl
10594199	Thsd4	NM_001040426	1.334e-006	-1.87123	E11.5 * Sox10 down vs E11.5 * Ctrl
10455112	Pcdhb17	NM_053142	2.95775e-005	-1.87179	E11.5 * Sox10 down vs E11.5 * Ctrl
10513158	Ptpn3	NM_011207	0.00152476	-1.87227	E11.5 * Sox10 down vs E11.5 * Ctrl
10439500	Upk1b	NM_178924	0.00150278	-1.8732	E11.5 * Sox10 down vs E11.5 * Ctrl
10586718	9530091C08Rik	NM_177159	0.000816948	-1.8738	E11.5 * Sox10 down vs E11.5 * Ctrl
10522749	Lphn3	NM_198702	6.3807e-007	-1.87681	E11.5 * Sox10 down vs E11.5 * Ctrl
10505717	Adamtsl1	NM_029967	2.02534e-005	-1.87724	E11.5 * Sox10 down vs E11.5 * Ctrl
10502890	St6galnac3	NM_011372	1.05255e-007	-1.87799	E11.5 * Sox10 down vs E11.5 * Ctrl
10567580	Igsf6	NM_030691	5.93055e-006	-1.87809	E11.5 * Sox10 down vs E11.5 * Ctrl
10590842	ENSMUSG0000C	ENSMUST00000(	0.00837691	-1.8784	E11.5 * Sox10 down vs E11.5 * Ctrl
10514128	Ttc39b	NM_027238	0.000170344	-1.87966	E11.5 * Sox10 down vs E11.5 * Ctrl
10368970	Prdm1	NM_007548	2.84235e-006	-1.87996	E11.5 * Sox10 down vs E11.5 * Ctrl
10395273	Gdap10	BC052902	5.90515e-005	-1.88089	E11.5 * Sox10 down vs E11.5 * Ctrl
10580033	Cd97	NM_011925	3.37466e-005	-1.883	E11.5 * Sox10 down vs E11.5 * Ctrl
10359870	Pbx1	NM_183355	3.25717e-006	-1.88489	E11.5 * Sox10 down vs E11.5 * Ctrl
10459768		---	0.000662933	-1.88509	E11.5 * Sox10 down vs E11.5 * Ctrl
10464251	Atrnl1	NM_181415	2.62997e-007	-1.88597	E11.5 * Sox10 down vs E11.5 * Ctrl
10505489	Pappa	NM_021362	5.25514e-007	-1.89066	E11.5 * Sox10 down vs E11.5 * Ctrl
10363696	D630028G08Rik	AK038706	1.49466e-005	-1.89081	E11.5 * Sox10 down vs E11.5 * Ctrl
10455054	Pcdhb3	NM_053128	3.78569e-005	-1.8922	E11.5 * Sox10 down vs E11.5 * Ctrl
10497580		---	0.0022458	-1.89235	E11.5 * Sox10 down vs E11.5 * Ctrl
10587733	Ctsh	NM_007801	4.79457e-005	-1.89236	E11.5 * Sox10 down vs E11.5 * Ctrl
10570894	Ank1	NM_001110783	5.68875e-011	-1.895	E11.5 * Sox10 down vs E11.5 * Ctrl
10401317	EG214321	NM_001038995	5.87889e-005	-1.8959	E11.5 * Sox10 down vs E11.5 * Ctrl
10476021	Sirpa	NM_007547	1.39142e-007	-1.89796	E11.5 * Sox10 down vs E11.5 * Ctrl
10549108	Abcc9	NM_021041	2.62966e-007	-1.90249	E11.5 * Sox10 down vs E11.5 * Ctrl
10534862	Pcolce	NM_008788	4.41663e-005	-1.9033	E11.5 * Sox10 down vs E11.5 * Ctrl
10457091	Neto1	NM_144946	9.44524e-005	-1.90416	E11.5 * Sox10 down vs E11.5 * Ctrl
10588899	Gpx1	NM_008160	6.95813e-005	-1.90417	E11.5 * Sox10 down vs E11.5 * Ctrl
10579958	Il15	NM_008357	5.21393e-007	-1.90496	E11.5 * Sox10 down vs E11.5 * Ctrl
10496971	Asb17	NM_025758	7.98069e-008	-1.90822	E11.5 * Sox10 down vs E11.5 * Ctrl
10368598	Rlbp1l2	NM_175448	4.54324e-005	-1.90891	E11.5 * Sox10 down vs E11.5 * Ctrl
10366476	Ptprb	NM_029928	2.15785e-007	-1.91181	E11.5 * Sox10 down vs E11.5 * Ctrl
10366645	1700006J14Rik	ENSMUST00000(	5.57338e-006	-1.91294	E11.5 * Sox10 down vs E11.5 * Ctrl
10403743	Inhba	NM_008380	5.82382e-006	-1.91351	E11.5 * Sox10 down vs E11.5 * Ctrl
10396270	Dact1	NM_021532	1.18667e-005	-1.91403	E11.5 * Sox10 down vs E11.5 * Ctrl
10478525	Wfdc2	NM_026323	9.1756e-006	-1.91414	E11.5 * Sox10 down vs E11.5 * Ctrl
10461721	Mpeg1	NM_010821	9.88899e-005	-1.91414	E11.5 * Sox10 down vs E11.5 * Ctrl
10423902	Zfpm2	NM_011766	1.03417e-005	-1.91547	E11.5 * Sox10 down vs E11.5 * Ctrl
10349510		---	0.00126509	-1.91585	E11.5 * Sox10 down vs E11.5 * Ctrl
10496438	Adh1	NM_007409	0.00392742	-1.91719	E11.5 * Sox10 down vs E11.5 * Ctrl
10407281	Esm1	NM_023612	1.87266e-007	-1.91746	E11.5 * Sox10 down vs E11.5 * Ctrl
10352143	D230039L06Rik	NM_177724	1.07199e-006	-1.91986	E11.5 * Sox10 down vs E11.5 * Ctrl
10422022	ENSMUSG0000C	ENSMUST00000	7.94175e-005	-1.92113	E11.5 * Sox10 down vs E11.5 * Ctrl
10419082	5730469M10Rik	BC056635	3.10386e-007	-1.92264	E11.5 * Sox10 down vs E11.5 * Ctrl
10447946	4930474M22Rik	BC089489	2.50836e-006	-1.92417	E11.5 * Sox10 down vs E11.5 * Ctrl
10512332	4930466F19Rik	ENSMUST00000	0.00268251	-1.92443	E11.5 * Sox10 down vs E11.5 * Ctrl
10512354	4930466F19Rik	ENSMUST00000	0.00268251	-1.92443	E11.5 * Sox10 down vs E11.5 * Ctrl
10523579	Arhgap24	NM_029270	1.37154e-008	-1.92708	E11.5 * Sox10 down vs E11.5 * Ctrl
10575548		---	0.000216324	-1.92723	E11.5 * Sox10 down vs E11.5 * Ctrl
10545707	Actg2	NM_009610	0.000192406	-1.92869	E11.5 * Sox10 down vs E11.5 * Ctrl
10511429	Car8	NM_007592	8.00753e-006	-1.92928	E11.5 * Sox10 down vs E11.5 * Ctrl
10523468	Bmp2k	NM_080708	8.75116e-006	-1.92977	E11.5 * Sox10 down vs E11.5 * Ctrl
10472757	Cybrd1	NM_028593	2.82486e-007	-1.93075	E11.5 * Sox10 down vs E11.5 * Ctrl
10369844	Bicc1	NM_031397	9.55337e-008	-1.93265	E11.5 * Sox10 down vs E11.5 * Ctrl
10383765		---	0.000415161	-1.93402	E11.5 * Sox10 down vs E11.5 * Ctrl
10433096	Nfe2	NM_008685	5.99904e-008	-1.93447	E11.5 * Sox10 down vs E11.5 * Ctrl
10527963		---	1.04769e-006	-1.93522	E11.5 * Sox10 down vs E11.5 * Ctrl
10433492	Atf7ip2	NM_153123	2.38949e-006	-1.93632	E11.5 * Sox10 down vs E11.5 * Ctrl
10573457	Klf1	NM_010635	3.66241e-007	-1.93897	E11.5 * Sox10 down vs E11.5 * Ctrl

10345212	Khdrbs2	NM_133235	5.40256e-006	-1.93902	E11.5 * Sox10 down vs E11.5 * Ctrl
10392560	Abca9	NM_147220	5.0881e-008	-1.93947	E11.5 * Sox10 down vs E11.5 * Ctrl
10404982	Barx1	NM_007526	1.66996e-005	-1.93997	E11.5 * Sox10 down vs E11.5 * Ctrl
10478936		---	0.00285348	-1.94395	E11.5 * Sox10 down vs E11.5 * Ctrl
10521796		---	0.000300515	-1.94751	E11.5 * Sox10 down vs E11.5 * Ctrl
10427906	D130046C19Rik	AK051399	0.000234979	-1.94769	E11.5 * Sox10 down vs E11.5 * Ctrl
10400844	Pygl	NM_133198	2.09368e-007	-1.94772	E11.5 * Sox10 down vs E11.5 * Ctrl
10441787	2810051F02Rik	BC009123	9.84461e-008	-1.94886	E11.5 * Sox10 down vs E11.5 * Ctrl
10406928	Cd180	NM_008533	1.10741e-005	-1.94911	E11.5 * Sox10 down vs E11.5 * Ctrl
10570855	Plat	NM_008872	7.47975e-006	-1.95091	E11.5 * Sox10 down vs E11.5 * Ctrl
10414262	Ear2	NM_007895	2.1191e-006	-1.95116	E11.5 * Sox10 down vs E11.5 * Ctrl
10544089	Zc3hav1	NM_028421	1.75354e-008	-1.95121	E11.5 * Sox10 down vs E11.5 * Ctrl
10545401	Vamp5	NM_016872	3.73399e-007	-1.95262	E11.5 * Sox10 down vs E11.5 * Ctrl
10455721	Sncaip	NM_026408	1.4457e-005	-1.95595	E11.5 * Sox10 down vs E11.5 * Ctrl
10528476		---	0.000772574	-1.95607	E11.5 * Sox10 down vs E11.5 * Ctrl
10433274	Vasn	NM_139307	2.73336e-006	-1.95729	E11.5 * Sox10 down vs E11.5 * Ctrl
10513805	Dbc1	NM_019967	5.43431e-007	-1.96255	E11.5 * Sox10 down vs E11.5 * Ctrl
10398362	Rian	AF357355	0.00100658	-1.96469	E11.5 * Sox10 down vs E11.5 * Ctrl
10536324	Asb4	NM_023048	1.71178e-005	-1.96593	E11.5 * Sox10 down vs E11.5 * Ctrl
10591120	Fat3	NM_001080814	2.78793e-006	-1.96644	E11.5 * Sox10 down vs E11.5 * Ctrl
10568392	Rgs10	NM_026418	3.01838e-008	-1.96687	E11.5 * Sox10 down vs E11.5 * Ctrl
10506058	Inadl	NM_172696	4.02647e-009	-1.97127	E11.5 * Sox10 down vs E11.5 * Ctrl
10376950	Pmp22	NM_008885	1.06811e-008	-1.97178	E11.5 * Sox10 down vs E11.5 * Ctrl
10458894	Lox	NM_010728	7.18021e-005	-1.97272	E11.5 * Sox10 down vs E11.5 * Ctrl
10598507	Slc38a5	NM_172479	9.19416e-007	-1.97277	E11.5 * Sox10 down vs E11.5 * Ctrl
10532085	Tgfbr3	NM_011578	4.27895e-008	-1.97344	E11.5 * Sox10 down vs E11.5 * Ctrl
10353309	Jph1	AK081751	0.000961035	-1.97473	E11.5 * Sox10 down vs E11.5 * Ctrl
10438708	Masp1	NM_008555	1.03231e-005	-1.97792	E11.5 * Sox10 down vs E11.5 * Ctrl
10380571	Gngt2	NM_023121	2.24736e-005	-1.97815	E11.5 * Sox10 down vs E11.5 * Ctrl
10372293	EG628870	XM_893664	4.0447e-010	-1.97984	E11.5 * Sox10 down vs E11.5 * Ctrl
10418355	Cacna1d	NM_028981	6.62106e-009	-1.98105	E11.5 * Sox10 down vs E11.5 * Ctrl
10398360		---	0.000401458	-1.98118	E11.5 * Sox10 down vs E11.5 * Ctrl
10362294	Arhgap18	NM_176837	1.50403e-006	-1.98204	E11.5 * Sox10 down vs E11.5 * Ctrl
10458303	Ecscr	NM_001033141	7.04632e-008	-1.98224	E11.5 * Sox10 down vs E11.5 * Ctrl
10502552	Clca1	NM_009899	0.000240948	-1.98419	E11.5 * Sox10 down vs E11.5 * Ctrl
10453759	ENSMUSG0000C	ENSMUST00000	9.07432e-009	-1.98484	E11.5 * Sox10 down vs E11.5 * Ctrl
10519747	Sema3e	NM_011348	4.91673e-007	-1.9935	E11.5 * Sox10 down vs E11.5 * Ctrl
10554808	Fzd4	NM_008055	6.73073e-006	-1.99376	E11.5 * Sox10 down vs E11.5 * Ctrl
10436392	Cpox	NM_007757	1.0206e-006	-1.99513	E11.5 * Sox10 down vs E11.5 * Ctrl
10604576	Gpc3	NM_016697	1.38761e-006	-1.99729	E11.5 * Sox10 down vs E11.5 * Ctrl
10578829	Palld	NM_001081390	1.57606e-005	-1.99844	E11.5 * Sox10 down vs E11.5 * Ctrl
10503520	Ttpa	NM_015767	3.67084e-006	-1.99935	E11.5 * Sox10 down vs E11.5 * Ctrl
10369792	Arid5b	NM_023598	1.23949e-005	-2.0001	E11.5 * Sox10 down vs E11.5 * Ctrl
10554863	Sytl2	NM_001040085	2.03878e-006	-2.0047	E11.5 * Sox10 down vs E11.5 * Ctrl
10427918	Fam105a	BC052328	1.01269e-007	-2.00978	E11.5 * Sox10 down vs E11.5 * Ctrl
10352150		---	3.62735e-007	-2.0153	E11.5 * Sox10 down vs E11.5 * Ctrl
10591135	Fat3	NM_001080814	6.74262e-005	-2.01567	E11.5 * Sox10 down vs E11.5 * Ctrl
10360070	Fcer1g	NM_010185	7.70365e-006	-2.01827	E11.5 * Sox10 down vs E11.5 * Ctrl
10600857	Heph	NM_010417	9.2749e-007	-2.01921	E11.5 * Sox10 down vs E11.5 * Ctrl
10465916	Gm98	BC157942	5.28665e-007	-2.0193	E11.5 * Sox10 down vs E11.5 * Ctrl
10368409	Lama2	NM_008481	2.79727e-010	-2.02105	E11.5 * Sox10 down vs E11.5 * Ctrl
10359929	Ddr2	NM_022563	1.27708e-006	-2.02267	E11.5 * Sox10 down vs E11.5 * Ctrl
10466210	Ms4a6d	NM_026835	4.82291e-007	-2.02488	E11.5 * Sox10 down vs E11.5 * Ctrl
10602221		---	6.79788e-005	-2.02604	E11.5 * Sox10 down vs E11.5 * Ctrl
10382106	Gm885	NM_001033435	6.34443e-006	-2.03129	E11.5 * Sox10 down vs E11.5 * Ctrl
10434806	Lpp	NM_178665	1.68797e-006	-2.03228	E11.5 * Sox10 down vs E11.5 * Ctrl
10531197	Adamts3	NM_001081401	2.50894e-006	-2.03689	E11.5 * Sox10 down vs E11.5 * Ctrl
10408329	Gmnn	AF068780	0.0002256	-2.03727	E11.5 * Sox10 down vs E11.5 * Ctrl
10381603	Fzd2	NM_020510	8.42943e-007	-2.03869	E11.5 * Sox10 down vs E11.5 * Ctrl
10358421	Rgs18	NM_022881	1.60712e-008	-2.04658	E11.5 * Sox10 down vs E11.5 * Ctrl
10546661	Foxp1	NM_053202	1.02742e-008	-2.04917	E11.5 * Sox10 down vs E11.5 * Ctrl
10367822	Rab32	NM_026405	1.41518e-005	-2.05001	E11.5 * Sox10 down vs E11.5 * Ctrl
10474295	Wt1	NM_144783	1.19225e-007	-2.05245	E11.5 * Sox10 down vs E11.5 * Ctrl
10519983	Fgl2	NM_008013	1.1456e-006	-2.05406	E11.5 * Sox10 down vs E11.5 * Ctrl
10360040	Fcgr3	NM_010188	2.93395e-007	-2.0544	E11.5 * Sox10 down vs E11.5 * Ctrl
10351623	F11r	NM_172647	6.06012e-005	-2.05583	E11.5 * Sox10 down vs E11.5 * Ctrl

10537834	Arhgef5	NM_133674	4.96131e-008	-2.05928	E11.5 * Sox10 down vs E11.5 * Ctrl
10538842	Gng12	NM_025278	1.57271e-009	-2.05975	E11.5 * Sox10 down vs E11.5 * Ctrl
10462237	Smarca2	NM_011416	2.70477e-010	-2.06456	E11.5 * Sox10 down vs E11.5 * Ctrl
10389231	Ccl3	NM_011337	0.000166009	-2.06741	E11.5 * Sox10 down vs E11.5 * Ctrl
10549420	Tmtc1	NM_198967	4.28759e-008	-2.06812	E11.5 * Sox10 down vs E11.5 * Ctrl
10348632	Twist2	NM_007855	3.32112e-007	-2.06975	E11.5 * Sox10 down vs E11.5 * Ctrl
10530536	Tec	NM_001113460	2.76372e-006	-2.07199	E11.5 * Sox10 down vs E11.5 * Ctrl
10530615	Ociad2	NM_026950	6.39449e-005	-2.07514	E11.5 * Sox10 down vs E11.5 * Ctrl
10544768	Hoxa5	NM_010453	4.7866e-005	-2.0754	E11.5 * Sox10 down vs E11.5 * Ctrl
10389025	Myo1d	NM_177390	2.37289e-005	-2.07574	E11.5 * Sox10 down vs E11.5 * Ctrl
10424140	Col14a1	NM_181277	2.17593e-006	-2.07694	E11.5 * Sox10 down vs E11.5 * Ctrl
10601360	Atp7a	NM_001109757	1.97546e-009	-2.0797	E11.5 * Sox10 down vs E11.5 * Ctrl
10590031	Itga9	NM_133721	7.82065e-007	-2.08129	E11.5 * Sox10 down vs E11.5 * Ctrl
10398358		---	0.00100578	-2.08167	E11.5 * Sox10 down vs E11.5 * Ctrl
10527959		---	0.000166322	-2.0819	E11.5 * Sox10 down vs E11.5 * Ctrl
10484389	Tfpi	NM_011576	3.01057e-010	-2.08495	E11.5 * Sox10 down vs E11.5 * Ctrl
10475890	Mertk	NM_008587	2.4598e-007	-2.08629	E11.5 * Sox10 down vs E11.5 * Ctrl
10401607	Pgf	NM_008827	2.91533e-006	-2.08866	E11.5 * Sox10 down vs E11.5 * Ctrl
10398368		---	1.90945e-007	-2.09049	E11.5 * Sox10 down vs E11.5 * Ctrl
10484203	2610301F02Rik	ENSMUST00000(	7.71097e-009	-2.09283	E11.5 * Sox10 down vs E11.5 * Ctrl
10513145	Ptpn3	NM_011207	1.78831e-005	-2.093	E11.5 * Sox10 down vs E11.5 * Ctrl
10398408		---	0.00119871	-2.09533	E11.5 * Sox10 down vs E11.5 * Ctrl
10544774	Hoxa6	NM_010454	7.67946e-007	-2.09705	E11.5 * Sox10 down vs E11.5 * Ctrl
10471721	Ptgs1	NM_008969	8.27828e-009	-2.09736	E11.5 * Sox10 down vs E11.5 * Ctrl
10606694	Btk	NM_013482	2.78054e-006	-2.09957	E11.5 * Sox10 down vs E11.5 * Ctrl
10354677	Ankrd44	NM_001081433	2.45936e-007	-2.10486	E11.5 * Sox10 down vs E11.5 * Ctrl
10436304	Abi3bp	NM_001014423	2.91788e-007	-2.10487	E11.5 * Sox10 down vs E11.5 * Ctrl
10502766	Lphn2	NM_001081298	0.00056724	-2.10757	E11.5 * Sox10 down vs E11.5 * Ctrl
10566326	Trim12	NM_023835	2.74134e-005	-2.10914	E11.5 * Sox10 down vs E11.5 * Ctrl
10445291		---	9.26098e-005	-2.11586	E11.5 * Sox10 down vs E11.5 * Ctrl
10578880	Tll1	NM_009390	4.81194e-006	-2.11742	E11.5 * Sox10 down vs E11.5 * Ctrl
10533401	Cux2	ENSMUST00000	4.00692e-007	-2.11944	E11.5 * Sox10 down vs E11.5 * Ctrl
10494548	Gja5	NM_008121	1.57031e-006	-2.12604	E11.5 * Sox10 down vs E11.5 * Ctrl
10427297	Hoxc5	NM_175730	6.12751e-009	-2.12811	E11.5 * Sox10 down vs E11.5 * Ctrl
10384504	Meis1	NM_010789	3.24236e-009	-2.13374	E11.5 * Sox10 down vs E11.5 * Ctrl
10548761	Hebp1	NM_013546	2.98867e-006	-2.13579	E11.5 * Sox10 down vs E11.5 * Ctrl
10527638	Alox5ap	NM_009663	5.82359e-006	-2.13663	E11.5 * Sox10 down vs E11.5 * Ctrl
10537849	Arhgef5	NM_133674	8.30113e-008	-2.1378	E11.5 * Sox10 down vs E11.5 * Ctrl
10583021	Pdgfd	NM_027924	3.47261e-006	-2.1389	E11.5 * Sox10 down vs E11.5 * Ctrl
10459766	Scarna17	AF357342	4.2906e-005	-2.14318	E11.5 * Sox10 down vs E11.5 * Ctrl
10461642	Scarna17	AF357342	4.2906e-005	-2.14318	E11.5 * Sox10 down vs E11.5 * Ctrl
10513362	OTTMUSG00000	ENSMUST00000	3.01019e-008	-2.14404	E11.5 * Sox10 down vs E11.5 * Ctrl
10416004	Zfp395	NM_199029	2.1876e-005	-2.14604	E11.5 * Sox10 down vs E11.5 * Ctrl
10506301	Lepr	NM_001122899	4.10597e-008	-2.14747	E11.5 * Sox10 down vs E11.5 * Ctrl
10528207	Cd36	NM_001159557	2.66967e-007	-2.15315	E11.5 * Sox10 down vs E11.5 * Ctrl
10410039	Ptch1	NM_008957	8.73319e-008	-2.15477	E11.5 * Sox10 down vs E11.5 * Ctrl
10458731	Mcc	NM_001085373	4.83062e-009	-2.15503	E11.5 * Sox10 down vs E11.5 * Ctrl
10479973	ENSMUSG00000	ENSMUST00000(	0.00136923	-2.15515	E11.5 * Sox10 down vs E11.5 * Ctrl
10427075	Krt18	NM_010664	5.46689e-006	-2.15604	E11.5 * Sox10 down vs E11.5 * Ctrl
10379530	Ccl12	NM_011331	2.18507e-006	-2.15878	E11.5 * Sox10 down vs E11.5 * Ctrl
10514177	Bnc2	NM_172870	2.15924e-007	-2.15907	E11.5 * Sox10 down vs E11.5 * Ctrl
10395320	Twist1	NM_011658	0.000236244	-2.15909	E11.5 * Sox10 down vs E11.5 * Ctrl
10428509	Csmd3	NM_001081391	0.0103642	-2.16098	E11.5 * Sox10 down vs E11.5 * Ctrl
10415651	Fgf9	NM_013518	4.10431e-007	-2.16253	E11.5 * Sox10 down vs E11.5 * Ctrl
10374727	Bcl11a	NM_016707	3.00327e-008	-2.16629	E11.5 * Sox10 down vs E11.5 * Ctrl
10574027	Mt1	NM_013602	2.87197e-005	-2.16761	E11.5 * Sox10 down vs E11.5 * Ctrl
10373179	Gli1	NM_010296	1.11696e-008	-2.16841	E11.5 * Sox10 down vs E11.5 * Ctrl
10433484	Atf7ip2	NM_153123	7.22977e-005	-2.17316	E11.5 * Sox10 down vs E11.5 * Ctrl
10544487	Tpk1	NM_013861	9.86342e-006	-2.17847	E11.5 * Sox10 down vs E11.5 * Ctrl
10527713	Rxfp2	NM_080468	1.77254e-010	-2.18352	E11.5 * Sox10 down vs E11.5 * Ctrl
10366446	Tspan8	NM_146010	0.00383816	-2.18363	E11.5 * Sox10 down vs E11.5 * Ctrl
10502776	Lphn2	NM_001081298	4.11571e-012	-2.18646	E11.5 * Sox10 down vs E11.5 * Ctrl
10593832		---	0.000121338	-2.19187	E11.5 * Sox10 down vs E11.5 * Ctrl
10427904	Fbxl7	BC050864	3.65813e-006	-2.19289	E11.5 * Sox10 down vs E11.5 * Ctrl
10599680	3830403N18Rik	NM_027510	1.51014e-007	-2.19683	E11.5 * Sox10 down vs E11.5 * Ctrl
10406519	Hapln1	NM_013500	4.81238e-006	-2.20158	E11.5 * Sox10 down vs E11.5 * Ctrl

10536499	Cav1	NM_007616	0.000137566	-2.20505	E11.5 * Sox10 down vs E11.5 * Ctrl
10374777	Efemp1	NM_146015	3.2201e-006	-2.21154	E11.5 * Sox10 down vs E11.5 * Ctrl
10406905	Ccdc125	NM_183115	3.16933e-006	-2.21215	E11.5 * Sox10 down vs E11.5 * Ctrl
10429128	Sla	NM_001029841	2.77973e-005	-2.21333	E11.5 * Sox10 down vs E11.5 * Ctrl
10587690	Bcl2a1b	NM_007534	1.66852e-006	-2.21523	E11.5 * Sox10 down vs E11.5 * Ctrl
10455761	Prdm6	NM_001033281	2.36404e-005	-2.21542	E11.5 * Sox10 down vs E11.5 * Ctrl
10502863	Ak5	NM_001081277	3.18677e-008	-2.21621	E11.5 * Sox10 down vs E11.5 * Ctrl
10357833	Atp2b4	NM_213616	3.30002e-008	-2.21768	E11.5 * Sox10 down vs E11.5 * Ctrl
10344990	Crispld1	NM_031402	3.05032e-010	-2.21928	E11.5 * Sox10 down vs E11.5 * Ctrl
10477600		---	1.56115e-009	-2.21971	E11.5 * Sox10 down vs E11.5 * Ctrl
10540207	Adamts9	ENSMUST00000(	2.82405e-007	-2.22009	E11.5 * Sox10 down vs E11.5 * Ctrl
10601980	Mum1l1	NM_175541	4.70328e-006	-2.23071	E11.5 * Sox10 down vs E11.5 * Ctrl
10559207	Lsp1	NM_019391	2.24704e-007	-2.23162	E11.5 * Sox10 down vs E11.5 * Ctrl
10396402	Prkch	NM_008856	4.17419e-008	-2.23274	E11.5 * Sox10 down vs E11.5 * Ctrl
10380637	Hoxb6	NM_008269	6.88889e-009	-2.23401	E11.5 * Sox10 down vs E11.5 * Ctrl
10440091	Col8a1	NM_007739	4.25903e-005	-2.23813	E11.5 * Sox10 down vs E11.5 * Ctrl
10400143	Stxbp6	NM_144552	9.5608e-008	-2.24053	E11.5 * Sox10 down vs E11.5 * Ctrl
10423556	Pgcp	NM_018755	1.0067e-007	-2.24256	E11.5 * Sox10 down vs E11.5 * Ctrl
10592420	AW551984	NM_178737	3.12443e-009	-2.24285	E11.5 * Sox10 down vs E11.5 * Ctrl
10355403	Fn1	NM_010233	2.37789e-007	-2.24362	E11.5 * Sox10 down vs E11.5 * Ctrl
10356995		---	5.90219e-006	-2.24476	E11.5 * Sox10 down vs E11.5 * Ctrl
10502240	Npnt	NM_033525	3.7407e-007	-2.24613	E11.5 * Sox10 down vs E11.5 * Ctrl
10354374	Slc40a1	NM_016917	8.42255e-008	-2.24706	E11.5 * Sox10 down vs E11.5 * Ctrl
10497608	Mds1	NM_021442	8.75332e-007	-2.24714	E11.5 * Sox10 down vs E11.5 * Ctrl
10501802	Tmem56	NM_178936	4.79407e-008	-2.24731	E11.5 * Sox10 down vs E11.5 * Ctrl
10495794	Pde5a	NM_153422	3.01865e-012	-2.24951	E11.5 * Sox10 down vs E11.5 * Ctrl
10599581	2610018G03Rik	NM_133729	1.40731e-009	-2.24991	E11.5 * Sox10 down vs E11.5 * Ctrl
10586118	Calml4	NM_138304	0.000418871	-2.2513	E11.5 * Sox10 down vs E11.5 * Ctrl
10363762	Tmem26	NM_177794	5.70697e-008	-2.25184	E11.5 * Sox10 down vs E11.5 * Ctrl
10606366	Zcchc5	NM_199468	8.52836e-006	-2.25201	E11.5 * Sox10 down vs E11.5 * Ctrl
10440099	St3gal6	NM_018784	7.83215e-008	-2.25337	E11.5 * Sox10 down vs E11.5 * Ctrl
10514255	Mllt3	NM_027326	4.30989e-012	-2.25498	E11.5 * Sox10 down vs E11.5 * Ctrl
10353213	Msc	NM_010827	3.52102e-007	-2.25605	E11.5 * Sox10 down vs E11.5 * Ctrl
10514275	Ptplad2	NM_025760	1.48901e-006	-2.25746	E11.5 * Sox10 down vs E11.5 * Ctrl
10500276	BC028528	BC028528	2.5254e-009	-2.25834	E11.5 * Sox10 down vs E11.5 * Ctrl
10352152	Kif26b	NM_177757	1.94751e-007	-2.26085	E11.5 * Sox10 down vs E11.5 * Ctrl
10501586	S1pr1	NM_007901	2.43504e-006	-2.26224	E11.5 * Sox10 down vs E11.5 * Ctrl
10375360	Ebf1	NM_007897	5.4394e-006	-2.26523	E11.5 * Sox10 down vs E11.5 * Ctrl
10595633	Bcl2a1d	NM_007536	6.99196e-007	-2.26745	E11.5 * Sox10 down vs E11.5 * Ctrl
10591125	Fat3	NM_001080814	1.71452e-005	-2.26747	E11.5 * Sox10 down vs E11.5 * Ctrl
10570957	Sfrp1	NM_013834	1.27701e-006	-2.26767	E11.5 * Sox10 down vs E11.5 * Ctrl
10419154	Ear1	NM_007894	1.04868e-005	-2.27489	E11.5 * Sox10 down vs E11.5 * Ctrl
10384458	Plek	NM_019549	4.25547e-006	-2.27687	E11.5 * Sox10 down vs E11.5 * Ctrl
10413928	1810011H11Rik	AK007434	1.06926e-005	-2.27689	E11.5 * Sox10 down vs E11.5 * Ctrl
10543306	Tspan12	NM_173007	5.01546e-008	-2.2775	E11.5 * Sox10 down vs E11.5 * Ctrl
10442087	4930546H06Rik	BC171984	3.6747e-007	-2.28397	E11.5 * Sox10 down vs E11.5 * Ctrl
10604347	Smarca1	NM_053123	2.11655e-008	-2.28565	E11.5 * Sox10 down vs E11.5 * Ctrl
10506031	Nfia	NM_010905	1.64048e-006	-2.28695	E11.5 * Sox10 down vs E11.5 * Ctrl
10463070	Entpd1	NM_009848	1.45489e-005	-2.28782	E11.5 * Sox10 down vs E11.5 * Ctrl
10597743	Cx3cr1	NM_009987	3.19929e-006	-2.29192	E11.5 * Sox10 down vs E11.5 * Ctrl
10453394	Six2	NM_011380	8.48346e-009	-2.29369	E11.5 * Sox10 down vs E11.5 * Ctrl
10410931	Vcan	NM_001081249	1.99732e-008	-2.29651	E11.5 * Sox10 down vs E11.5 * Ctrl
10456046	Pdgfrb	NM_001146268	3.64508e-007	-2.29693	E11.5 * Sox10 down vs E11.5 * Ctrl
10372028	Plxnc1	NM_018797	9.14385e-010	-2.30939	E11.5 * Sox10 down vs E11.5 * Ctrl
10374333	Ikzf1	NM_001025597	8.24895e-008	-2.31308	E11.5 * Sox10 down vs E11.5 * Ctrl
10576046	Foxf1a	NM_010426	8.91855e-006	-2.31538	E11.5 * Sox10 down vs E11.5 * Ctrl
10502778	Lphn2	NM_001081298	6.01851e-007	-2.31962	E11.5 * Sox10 down vs E11.5 * Ctrl
10436596	2810055G20Rik	ENSMUST00000(	3.18802e-007	-2.32198	E11.5 * Sox10 down vs E11.5 * Ctrl
10454172	Dsg2	NM_007883	4.85209e-007	-2.32287	E11.5 * Sox10 down vs E11.5 * Ctrl
10434802	Lpp	BC005613	1.05612e-005	-2.32402	E11.5 * Sox10 down vs E11.5 * Ctrl
10549877		---	2.82928e-006	-2.32554	E11.5 * Sox10 down vs E11.5 * Ctrl
10556076	Olfml1	NM_172907	1.48006e-006	-2.33577	E11.5 * Sox10 down vs E11.5 * Ctrl
10543239	Tcfec	NM_031198	1.70946e-005	-2.33733	E11.5 * Sox10 down vs E11.5 * Ctrl
10601993	D330045A20Rik	NM_175326	6.6754e-007	-2.35801	E11.5 * Sox10 down vs E11.5 * Ctrl
10399801	Sntg2	NM_172951	3.49514e-008	-2.35862	E11.5 * Sox10 down vs E11.5 * Ctrl
10502748	Lphn2	NM_001081298	9.99703e-011	-2.36658	E11.5 * Sox10 down vs E11.5 * Ctrl

10409061		---	0.000109714	-2.36697	E11.5 * Sox10 down vs E11.5 * Ctrl
10587683	Bcl2a1a	NM_009742	1.48819e-005	-2.36897	E11.5 * Sox10 down vs E11.5 * Ctrl
10555297	Kcne3	NM_020574	1.17652e-008	-2.37227	E11.5 * Sox10 down vs E11.5 * Ctrl
10409876	Ctla2a	NM_007796	5.98717e-005	-2.37879	E11.5 * Sox10 down vs E11.5 * Ctrl
10519998	Lrrc17	NM_028977	1.06498e-008	-2.38152	E11.5 * Sox10 down vs E11.5 * Ctrl
10467124	Acta2	NM_007392	0.00421763	-2.38552	E11.5 * Sox10 down vs E11.5 * Ctrl
10460541	Cd248	NM_054042	1.12711e-006	-2.38634	E11.5 * Sox10 down vs E11.5 * Ctrl
10521700	ENSMUSG0000C	ENSMUST00000(	0.000120829	-2.38981	E11.5 * Sox10 down vs E11.5 * Ctrl
10406845	Foxd1	NM_008242	0.000289591	-2.39434	E11.5 * Sox10 down vs E11.5 * Ctrl
10506820		---	0.000136515	-2.39434	E11.5 * Sox10 down vs E11.5 * Ctrl
10435501	Stfa1	NM_001082543	1.21824e-006	-2.39698	E11.5 * Sox10 down vs E11.5 * Ctrl
10499132	Mab21l2	NM_011839	1.89498e-006	-2.39725	E11.5 * Sox10 down vs E11.5 * Ctrl
10587446	Myo6	NM_001039546	3.15806e-008	-2.39792	E11.5 * Sox10 down vs E11.5 * Ctrl
10546736	Cntn3	NM_008779	3.20215e-007	-2.40048	E11.5 * Sox10 down vs E11.5 * Ctrl
10545101	Ptgds2	NM_019455	1.5193e-006	-2.4044	E11.5 * Sox10 down vs E11.5 * Ctrl
10407350	Fgf10	NM_008002	3.65886e-007	-2.40704	E11.5 * Sox10 down vs E11.5 * Ctrl
10453049	Cdc42ep3	NM_026514	1.85236e-006	-2.40989	E11.5 * Sox10 down vs E11.5 * Ctrl
10583529	Icam4	NM_023892	2.45934e-009	-2.41378	E11.5 * Sox10 down vs E11.5 * Ctrl
10586744	Anxa2	NM_007585	1.34737e-005	-2.41641	E11.5 * Sox10 down vs E11.5 * Ctrl
10453747	Colec12	NM_130449	3.01559e-007	-2.42358	E11.5 * Sox10 down vs E11.5 * Ctrl
10513774		---	2.68622e-007	-2.42488	E11.5 * Sox10 down vs E11.5 * Ctrl
10346015	Col3a1	NM_009930	1.20446e-008	-2.42787	E11.5 * Sox10 down vs E11.5 * Ctrl
10498018	Pcdh18	NM_130448	3.99316e-010	-2.43078	E11.5 * Sox10 down vs E11.5 * Ctrl
10453057	Cyp1b1	NM_009994	1.10635e-006	-2.43104	E11.5 * Sox10 down vs E11.5 * Ctrl
10360398	Ifi202b	NM_008327	5.67398e-005	-2.4343	E11.5 * Sox10 down vs E11.5 * Ctrl
10535807	Flt1	NM_010228	1.49221e-011	-2.4351	E11.5 * Sox10 down vs E11.5 * Ctrl
10504203	4930578G10Rik	ENSMUST00000(	0.00254902	-2.43738	E11.5 * Sox10 down vs E11.5 * Ctrl
10395409	Meox2	NM_008584	2.16078e-005	-2.43794	E11.5 * Sox10 down vs E11.5 * Ctrl
10564818	Anpep	NM_008486	2.19877e-005	-2.43844	E11.5 * Sox10 down vs E11.5 * Ctrl
10406736	F2rl2	NM_010170	4.15465e-007	-2.43844	E11.5 * Sox10 down vs E11.5 * Ctrl
10531203	Adamts3	NM_001081401	1.25339e-007	-2.46311	E11.5 * Sox10 down vs E11.5 * Ctrl
10498119	Frem2	NM_172862	1.22678e-007	-2.46472	E11.5 * Sox10 down vs E11.5 * Ctrl
10396936	Smoc1	NM_001146217	1.0951e-006	-2.46491	E11.5 * Sox10 down vs E11.5 * Ctrl
10501903	Synpo2	NM_080451	8.65846e-008	-2.46973	E11.5 * Sox10 down vs E11.5 * Ctrl
10446282	Emr1	NM_010130	1.36078e-006	-2.4773	E11.5 * Sox10 down vs E11.5 * Ctrl
10542594	ENSMUSG0000C	AY512955	7.01513e-007	-2.48293	E11.5 * Sox10 down vs E11.5 * Ctrl
10545409	Vamp8	NM_016794	1.22589e-007	-2.48402	E11.5 * Sox10 down vs E11.5 * Ctrl
10360415	Grem2	NM_011825	2.80925e-008	-2.486	E11.5 * Sox10 down vs E11.5 * Ctrl
10455919	Adamts19	NM_175506	6.56649e-009	-2.49136	E11.5 * Sox10 down vs E11.5 * Ctrl
10506488	Ppap2b	NM_080555	2.38314e-006	-2.4917	E11.5 * Sox10 down vs E11.5 * Ctrl
10453857	Gata6	NM_010258	9.12501e-007	-2.49266	E11.5 * Sox10 down vs E11.5 * Ctrl
10538126	Gimap4	NM_174990	5.84595e-007	-2.49495	E11.5 * Sox10 down vs E11.5 * Ctrl
10362091	Raet1d	NM_020030	0.000106747	-2.49761	E11.5 * Sox10 down vs E11.5 * Ctrl
10445046	Trim10	NM_011280	1.03144e-009	-2.49982	E11.5 * Sox10 down vs E11.5 * Ctrl
10427286	Hoxc9	NM_008272	2.52793e-009	-2.50049	E11.5 * Sox10 down vs E11.5 * Ctrl
10344952	Rdh10	NM_133832	4.19741e-005	-2.50067	E11.5 * Sox10 down vs E11.5 * Ctrl
10386996	Myocd	NM_145136	3.55655e-007	-2.50393	E11.5 * Sox10 down vs E11.5 * Ctrl
10514070	2310067E19Rik	BC066147	4.65142e-007	-2.50791	E11.5 * Sox10 down vs E11.5 * Ctrl
10362314	Ptprk	NM_008983	1.36288e-010	-2.51391	E11.5 * Sox10 down vs E11.5 * Ctrl
10415396	Nfatc4	NM_023699	3.03886e-008	-2.52823	E11.5 * Sox10 down vs E11.5 * Ctrl
10503054	Lrrc7	NM_001081358	7.20777e-005	-2.53663	E11.5 * Sox10 down vs E11.5 * Ctrl
10358434	Pla2g4a	NM_008869	1.92848e-007	-2.54261	E11.5 * Sox10 down vs E11.5 * Ctrl
10376778	Mfap4	NM_029568	1.25095e-008	-2.54442	E11.5 * Sox10 down vs E11.5 * Ctrl
10584593		---	5.3889e-006	-2.54681	E11.5 * Sox10 down vs E11.5 * Ctrl
10566358	Trim30	NM_009099	1.11826e-006	-2.54851	E11.5 * Sox10 down vs E11.5 * Ctrl
10551736	Ppp1r14a	NM_026731	2.51084e-005	-2.54975	E11.5 * Sox10 down vs E11.5 * Ctrl
10604528	Mbnl3	NM_134163	1.47275e-007	-2.55022	E11.5 * Sox10 down vs E11.5 * Ctrl
10502780	Lphn2	NM_001081298	1.33953e-010	-2.55262	E11.5 * Sox10 down vs E11.5 * Ctrl
10487040	Fbn1	NM_007993	6.77274e-009	-2.55811	E11.5 * Sox10 down vs E11.5 * Ctrl
10362113	ENSMUSG0000C	ENSMUST00000	4.33184e-007	-2.55903	E11.5 * Sox10 down vs E11.5 * Ctrl
10537298	Chrm2	NM_203491	9.0529e-005	-2.56427	E11.5 * Sox10 down vs E11.5 * Ctrl
10578264	Msr1	NM_031195	1.24785e-007	-2.57151	E11.5 * Sox10 down vs E11.5 * Ctrl
10577641	1810011O10Rik	NM_026931	2.13627e-005	-2.57246	E11.5 * Sox10 down vs E11.5 * Ctrl
10359861	Mgst3	NM_025569	2.32075e-007	-2.59833	E11.5 * Sox10 down vs E11.5 * Ctrl
10458828	Cdo1	NM_033037	4.7578e-009	-2.60381	E11.5 * Sox10 down vs E11.5 * Ctrl
10591116	Fat3	NM_001080814	2.5253e-009	-2.60504	E11.5 * Sox10 down vs E11.5 * Ctrl

10485840	Ryr3	NM_177652	1.40591e-012	-2.60747	E11.5 * Sox10 down vs E11.5 * Ctrl
10392221	Pecam1	NM_008816	1.70567e-008	-2.61588	E11.5 * Sox10 down vs E11.5 * Ctrl
10578123	Rbpms	NM_019733	1.93986e-010	-2.61654	E11.5 * Sox10 down vs E11.5 * Ctrl
10565204	Bnc1	NM_007562	1.85874e-008	-2.63013	E11.5 * Sox10 down vs E11.5 * Ctrl
10606355	Cysltr1	NM_021476	2.73361e-008	-2.63076	E11.5 * Sox10 down vs E11.5 * Ctrl
10529875	Ldb2	NM_001077398	1.37065e-008	-2.63277	E11.5 * Sox10 down vs E11.5 * Ctrl
10398366		---	0.000299326	-2.6334	E11.5 * Sox10 down vs E11.5 * Ctrl
10581961	Adamts18	NM_172466	5.42924e-008	-2.64294	E11.5 * Sox10 down vs E11.5 * Ctrl
10506108		---	0.000193106	-2.64378	E11.5 * Sox10 down vs E11.5 * Ctrl
10355500	Igfbp5	NM_010518	5.38766e-007	-2.65019	E11.5 * Sox10 down vs E11.5 * Ctrl
10495685	Arhgap29	NM_172525	6.35188e-007	-2.65382	E11.5 * Sox10 down vs E11.5 * Ctrl
10509002	Rhd	NM_011270	1.36584e-009	-2.65579	E11.5 * Sox10 down vs E11.5 * Ctrl
10572130	Lpl	NM_008509	2.25204e-009	-2.6669	E11.5 * Sox10 down vs E11.5 * Ctrl
10347724	Slc4a3	ENSMUST00000	0.00100598	-2.66841	E11.5 * Sox10 down vs E11.5 * Ctrl
10515848	Ermap	NM_013848	1.02184e-009	-2.67011	E11.5 * Sox10 down vs E11.5 * Ctrl
10412260	Fst	NM_008046	1.33112e-007	-2.67296	E11.5 * Sox10 down vs E11.5 * Ctrl
10427336	Nckap1l	NM_153505	4.20591e-007	-2.67643	E11.5 * Sox10 down vs E11.5 * Ctrl
10559816		---	2.06728e-007	-2.68817	E11.5 * Sox10 down vs E11.5 * Ctrl
10486664	Epb4.2	NM_013513	2.46991e-008	-2.69106	E11.5 * Sox10 down vs E11.5 * Ctrl
10408525	1700018A04Rik	ENSMUST00000	6.67698e-009	-2.69371	E11.5 * Sox10 down vs E11.5 * Ctrl
10441864	Mllt4	NM_010806	1.50553e-012	-2.7008	E11.5 * Sox10 down vs E11.5 * Ctrl
10434804		---	0.000612775	-2.70422	E11.5 * Sox10 down vs E11.5 * Ctrl
10543959	Ptn	NM_008973	6.36437e-008	-2.7051	E11.5 * Sox10 down vs E11.5 * Ctrl
10571601	Pdlim3	NM_016798	1.26572e-009	-2.71025	E11.5 * Sox10 down vs E11.5 * Ctrl
10352867	Plxna2	NM_008882	2.12441e-010	-2.71878	E11.5 * Sox10 down vs E11.5 * Ctrl
10606016	Il2rg	NM_013563	1.18847e-006	-2.72247	E11.5 * Sox10 down vs E11.5 * Ctrl
10467838		---	2.12865e-005	-2.72333	E11.5 * Sox10 down vs E11.5 * Ctrl
10374366	Egfr	NM_207655	1.40752e-008	-2.73099	E11.5 * Sox10 down vs E11.5 * Ctrl
10423548	Sdc2	NM_008304	1.99026e-008	-2.73266	E11.5 * Sox10 down vs E11.5 * Ctrl
10351455	Rgs5	NM_009063	6.40771e-006	-2.73284	E11.5 * Sox10 down vs E11.5 * Ctrl
10548879	Mgp	NM_008597	5.78146e-007	-2.74468	E11.5 * Sox10 down vs E11.5 * Ctrl
10569335	H19	NR_001592	7.61576e-005	-2.74774	E11.5 * Sox10 down vs E11.5 * Ctrl
10421172	Slc25a37	NM_026331	7.01143e-008	-2.75085	E11.5 * Sox10 down vs E11.5 * Ctrl
10517513	C1qc	NM_007574	8.93656e-008	-2.75856	E11.5 * Sox10 down vs E11.5 * Ctrl
10559796	Peg3	NM_008817	2.5209e-007	-2.75952	E11.5 * Sox10 down vs E11.5 * Ctrl
10499870	Lor	NM_008508	6.30175e-006	-2.76228	E11.5 * Sox10 down vs E11.5 * Ctrl
10564527	Nr2f2	NM_009697	1.42555e-011	-2.76843	E11.5 * Sox10 down vs E11.5 * Ctrl
10605503		---	0.000455694	-2.77277	E11.5 * Sox10 down vs E11.5 * Ctrl
10412345	Parp8	NM_001081009	3.11181e-009	-2.7801	E11.5 * Sox10 down vs E11.5 * Ctrl
10575034	Cdh3	NM_001037809	6.57982e-008	-2.78314	E11.5 * Sox10 down vs E11.5 * Ctrl
10492689	Pdgfc	NM_019971	5.68737e-009	-2.79235	E11.5 * Sox10 down vs E11.5 * Ctrl
10602372	Alas2	NM_009653	9.81212e-011	-2.79492	E11.5 * Sox10 down vs E11.5 * Ctrl
10546434	Adamts9	NM_175314	1.50859e-007	-2.79743	E11.5 * Sox10 down vs E11.5 * Ctrl
10525030	Tbx5	NM_011537	2.52107e-008	-2.79843	E11.5 * Sox10 down vs E11.5 * Ctrl
10506050	Nfia	NM_001122952	2.59895e-007	-2.80574	E11.5 * Sox10 down vs E11.5 * Ctrl
10348451	Cxcr7	NM_007722	1.09094e-008	-2.8112	E11.5 * Sox10 down vs E11.5 * Ctrl
10604393	ENSMUSG00000	NM_019680	1.12122e-010	-2.8144	E11.5 * Sox10 down vs E11.5 * Ctrl
10522288	Shisa3	NM_001033415	3.32772e-010	-2.8247	E11.5 * Sox10 down vs E11.5 * Ctrl
10590635	Ccr5	NM_009917	3.82926e-007	-2.82725	E11.5 * Sox10 down vs E11.5 * Ctrl
10598013	Ccr5	NM_009917	3.82926e-007	-2.82725	E11.5 * Sox10 down vs E11.5 * Ctrl
10408693	F13a1	NM_028784	5.26974e-007	-2.82958	E11.5 * Sox10 down vs E11.5 * Ctrl
10484207	2610301F02Rik	NM_001025576	9.29233e-012	-2.83064	E11.5 * Sox10 down vs E11.5 * Ctrl
10443108	Syngap1	XM_985548	0.00019563	-2.83725	E11.5 * Sox10 down vs E11.5 * Ctrl
10456071	Csf1r	NM_001037859	2.33975e-007	-2.86716	E11.5 * Sox10 down vs E11.5 * Ctrl
10514054	Nfib	NM_001113209	2.93908e-011	-2.88383	E11.5 * Sox10 down vs E11.5 * Ctrl
10351867	Aim2	NM_001013779	1.7108e-006	-2.88661	E11.5 * Sox10 down vs E11.5 * Ctrl
10381298	Ramp2	NM_019444	4.52733e-007	-2.90882	E11.5 * Sox10 down vs E11.5 * Ctrl
10510239	ENSMUSG00000	ENSMUST00000	1.40423e-005	-2.91068	E11.5 * Sox10 down vs E11.5 * Ctrl
10407122		---	0.000269605	-2.91353	E11.5 * Sox10 down vs E11.5 * Ctrl
10483074	Gcg	NM_008100	1.44795e-007	-2.92169	E11.5 * Sox10 down vs E11.5 * Ctrl
10544583	Gimap6	NM_153175	9.22638e-009	-2.92823	E11.5 * Sox10 down vs E11.5 * Ctrl
10427290	Hoxc8	NM_010466	1.85395e-007	-2.93697	E11.5 * Sox10 down vs E11.5 * Ctrl
10440019	Tmem45a	NM_019631	2.53479e-006	-2.93831	E11.5 * Sox10 down vs E11.5 * Ctrl
10571788	Vegfc	NM_009506	1.87247e-007	-2.94781	E11.5 * Sox10 down vs E11.5 * Ctrl
10502774	Lphn2	NM_001081298	9.71524e-013	-2.94966	E11.5 * Sox10 down vs E11.5 * Ctrl
10594044	Islr	NM_012043	5.66284e-008	-2.95171	E11.5 * Sox10 down vs E11.5 * Ctrl

10358339	Cfh	NM_009888	5.2329e-009	-2.95632	E11.5 * Sox10 down vs E11.5 * Ctrl
10502772	Lphn2	NM_001081298	1.94381e-010	-2.96529	E11.5 * Sox10 down vs E11.5 * Ctrl
10591131	Fat3	NM_001080814	1.08442e-007	-2.97806	E11.5 * Sox10 down vs E11.5 * Ctrl
10469404	Cacnb2	NM_023116	8.19725e-009	-2.97835	E11.5 * Sox10 down vs E11.5 * Ctrl
10407286	BC067074	ENSMUST00000(	3.73865e-006	-2.9819	E11.5 * Sox10 down vs E11.5 * Ctrl
10521972	Pcdh7	NM_018764	7.17362e-011	-2.98843	E11.5 * Sox10 down vs E11.5 * Ctrl
10445268	Gpr116	NM_001081178	6.61715e-009	-3.00667	E11.5 * Sox10 down vs E11.5 * Ctrl
10351463	Rgs5	ENSMUST00000(	9.70918e-005	-3.00863	E11.5 * Sox10 down vs E11.5 * Ctrl
10522530	Kit	NM_001122733	2.54806e-010	-3.00931	E11.5 * Sox10 down vs E11.5 * Ctrl
10344837	Prex2	NM_029525	1.01463e-008	-3.01582	E11.5 * Sox10 down vs E11.5 * Ctrl
10440340		---	3.47105e-010	-3.03779	E11.5 * Sox10 down vs E11.5 * Ctrl
10411235	Iqgap2	NM_027711	2.49647e-008	-3.0452	E11.5 * Sox10 down vs E11.5 * Ctrl
10519857	Hgf	NM_010427	3.15301e-010	-3.05809	E11.5 * Sox10 down vs E11.5 * Ctrl
10436598	2810055G20Rik	ENSMUST00000(	0.000488515	-3.05871	E11.5 * Sox10 down vs E11.5 * Ctrl
10499189	Fcrls	NM_030707	2.08243e-007	-3.05877	E11.5 * Sox10 down vs E11.5 * Ctrl
10372648	Lyz2	NM_017372	1.65798e-007	-3.07928	E11.5 * Sox10 down vs E11.5 * Ctrl
10592067	Fli1	NM_008026	9.42107e-009	-3.08513	E11.5 * Sox10 down vs E11.5 * Ctrl
10475544	Sema6d	NM_199241	7.4604e-009	-3.08633	E11.5 * Sox10 down vs E11.5 * Ctrl
10576051	Foxc2	NM_013519	1.24428e-006	-3.08681	E11.5 * Sox10 down vs E11.5 * Ctrl
10350840	Angptl1	NM_028333	1.36757e-008	-3.09515	E11.5 * Sox10 down vs E11.5 * Ctrl
10607484	Ptchd1	NM_001093750	3.69989e-006	-3.10779	E11.5 * Sox10 down vs E11.5 * Ctrl
10497590	Evi1	NM_007963	6.10792e-008	-3.11012	E11.5 * Sox10 down vs E11.5 * Ctrl
10498921	Tdo2	NM_019911	2.02609e-007	-3.11205	E11.5 * Sox10 down vs E11.5 * Ctrl
10412335	Isl1	NM_021459	1.10873e-006	-3.11475	E11.5 * Sox10 down vs E11.5 * Ctrl
10591127	Fat3	NM_001080814	9.00047e-006	-3.11824	E11.5 * Sox10 down vs E11.5 * Ctrl
10594855	Cgnl1	NM_026599	3.9459e-008	-3.11969	E11.5 * Sox10 down vs E11.5 * Ctrl
10402705	Gm266	NM_001033248	3.6787e-007	-3.12801	E11.5 * Sox10 down vs E11.5 * Ctrl
10568553	4631426J05Rik	NM_029935	4.36021e-008	-3.13554	E11.5 * Sox10 down vs E11.5 * Ctrl
10351111	Dnm3os	NR_002870	1.067e-008	-3.14558	E11.5 * Sox10 down vs E11.5 * Ctrl
10404404	Foxf2	NM_010225	3.52861e-008	-3.15483	E11.5 * Sox10 down vs E11.5 * Ctrl
10517165	Cd52	NM_013706	3.22209e-007	-3.16403	E11.5 * Sox10 down vs E11.5 * Ctrl
10591118	Fat3	NM_001080814	1.43546e-006	-3.16734	E11.5 * Sox10 down vs E11.5 * Ctrl
10427293	Hoxc6	NM_010465	1.80074e-006	-3.17138	E11.5 * Sox10 down vs E11.5 * Ctrl
10601569	Pcdh11x	NM_001081385	2.89613e-005	-3.17723	E11.5 * Sox10 down vs E11.5 * Ctrl
10467840	C130021O09Rik	AK081501	7.88925e-006	-3.17739	E11.5 * Sox10 down vs E11.5 * Ctrl
10496872	Eltd1	NM_133222	1.97244e-006	-3.17902	E11.5 * Sox10 down vs E11.5 * Ctrl
10472953	Hoxd11	NM_008273	1.64043e-007	-3.19536	E11.5 * Sox10 down vs E11.5 * Ctrl
10607848	Egfl6	NM_019397	7.63531e-007	-3.20978	E11.5 * Sox10 down vs E11.5 * Ctrl
10398364		---	1.75385e-006	-3.2146	E11.5 * Sox10 down vs E11.5 * Ctrl
10591123	Fat3	NM_001080814	5.81538e-006	-3.21517	E11.5 * Sox10 down vs E11.5 * Ctrl
10436600		---	0.000598139	-3.21568	E11.5 * Sox10 down vs E11.5 * Ctrl
10361771	Plagl1	NM_009538	3.46132e-007	-3.21717	E11.5 * Sox10 down vs E11.5 * Ctrl
10514185		---	0.000518858	-3.21751	E11.5 * Sox10 down vs E11.5 * Ctrl
10413482	Wnt5a	NM_009524	2.48463e-007	-3.22622	E11.5 * Sox10 down vs E11.5 * Ctrl
10513957	Ptprd	NM_011211	5.63496e-013	-3.23109	E11.5 * Sox10 down vs E11.5 * Ctrl
10492798	Sfrp2	NM_009144	2.95316e-011	-3.23198	E11.5 * Sox10 down vs E11.5 * Ctrl
10584561	9030425E11Rik	NM_133733	1.66147e-006	-3.23373	E11.5 * Sox10 down vs E11.5 * Ctrl
10428707	Has2	NM_008216	1.08712e-009	-3.23551	E11.5 * Sox10 down vs E11.5 * Ctrl
10514049	Nfib	NM_001113209	9.01572e-012	-3.23689	E11.5 * Sox10 down vs E11.5 * Ctrl
10484201	2610301F02Rik	ENSMUST00000(	1.39215e-007	-3.25064	E11.5 * Sox10 down vs E11.5 * Ctrl
10491319	Kcnmb2	NM_028231	1.96112e-008	-3.25467	E11.5 * Sox10 down vs E11.5 * Ctrl
10531931	Sparcl1	NM_010097	2.0376e-009	-3.26301	E11.5 * Sox10 down vs E11.5 * Ctrl
10513101		---	8.93207e-007	-3.28703	E11.5 * Sox10 down vs E11.5 * Ctrl
10505954	Tek	NM_013690	3.06553e-009	-3.29116	E11.5 * Sox10 down vs E11.5 * Ctrl
10604585		---	9.68827e-009	-3.29427	E11.5 * Sox10 down vs E11.5 * Ctrl
10422436	Dock9	NM_001081039	6.99913e-011	-3.29552	E11.5 * Sox10 down vs E11.5 * Ctrl
10472965	Hoxd8	NM_008276	1.7017e-008	-3.30409	E11.5 * Sox10 down vs E11.5 * Ctrl
10513739	Tnc	NM_011607	1.36137e-008	-3.30861	E11.5 * Sox10 down vs E11.5 * Ctrl
10488382	Cd93	NM_010740	2.56259e-008	-3.32172	E11.5 * Sox10 down vs E11.5 * Ctrl
10569017	Ifitm3	NM_025378	2.08152e-005	-3.33103	E11.5 * Sox10 down vs E11.5 * Ctrl
10398380		---	9.79298e-006	-3.3562	E11.5 * Sox10 down vs E11.5 * Ctrl
10523376	Fras1	NM_175473	3.26748e-009	-3.37043	E11.5 * Sox10 down vs E11.5 * Ctrl
10591739	Acp5	NM_001102404	1.26955e-009	-3.37193	E11.5 * Sox10 down vs E11.5 * Ctrl
10447190	Plekhh2	NM_177606	5.41986e-011	-3.37465	E11.5 * Sox10 down vs E11.5 * Ctrl
10558769	Ifitm1	NM_026820	7.17002e-008	-3.3898	E11.5 * Sox10 down vs E11.5 * Ctrl
10584595	2610203C20Rik	ENSMUST00000(	2.51723e-007	-3.39918	E11.5 * Sox10 down vs E11.5 * Ctrl

10376956	Hs3st3a1	NM_178870	4.04455e-008	-3.40817	E11.5 * Sox10 down vs E11.5 * Ctrl
10483800		---	5.60728e-008	-3.41125	E11.5 * Sox10 down vs E11.5 * Ctrl
10501063	Cd53	NM_007651	7.70441e-008	-3.41136	E11.5 * Sox10 down vs E11.5 * Ctrl
10496324	Slc39a8	NM_001135149	7.40252e-010	-3.41397	E11.5 * Sox10 down vs E11.5 * Ctrl
10534389	Cldn13	NM_020504	7.49367e-007	-3.41423	E11.5 * Sox10 down vs E11.5 * Ctrl
10424105	Colec10	NM_173422	5.31113e-011	-3.43785	E11.5 * Sox10 down vs E11.5 * Ctrl
10581013	Cdh11	NM_009866	1.08282e-009	-3.4405	E11.5 * Sox10 down vs E11.5 * Ctrl
10391649	Slc4a1	NM_011403	1.46446e-011	-3.44292	E11.5 * Sox10 down vs E11.5 * Ctrl
10352905	Cd34	NM_001111059	7.48589e-008	-3.44391	E11.5 * Sox10 down vs E11.5 * Ctrl
10398354		---	3.48284e-006	-3.44973	E11.5 * Sox10 down vs E11.5 * Ctrl
10473399	Prg2	NM_008920	6.50819e-008	-3.45448	E11.5 * Sox10 down vs E11.5 * Ctrl
10346164	Sdpr	NM_138741	4.92592e-008	-3.46645	E11.5 * Sox10 down vs E11.5 * Ctrl
10376326	Irgm2	NM_019440	5.34099e-010	-3.47667	E11.5 * Sox10 down vs E11.5 * Ctrl
10548038	Ntf3	NM_008742	1.47909e-008	-3.49261	E11.5 * Sox10 down vs E11.5 * Ctrl
10542575	Pde3a	NM_018779	2.75031e-007	-3.49962	E11.5 * Sox10 down vs E11.5 * Ctrl
10503382	Runx1t1	NM_001111027	2.51554e-008	-3.50701	E11.5 * Sox10 down vs E11.5 * Ctrl
10344897	Sulf1	NM_172294	1.07412e-007	-3.51502	E11.5 * Sox10 down vs E11.5 * Ctrl
10584600		---	2.78912e-008	-3.54952	E11.5 * Sox10 down vs E11.5 * Ctrl
10419151	Ear1	NM_007894	5.75723e-006	-3.58525	E11.5 * Sox10 down vs E11.5 * Ctrl
10553274	Saa2	NM_011314	5.22695e-007	-3.59022	E11.5 * Sox10 down vs E11.5 * Ctrl
10349648	Ctse	NM_007799	8.81167e-011	-3.59515	E11.5 * Sox10 down vs E11.5 * Ctrl
10368495	Rspo3	NM_028351	3.40902e-011	-3.5973	E11.5 * Sox10 down vs E11.5 * Ctrl
10519717	Sema3a	NM_009152	3.69311e-009	-3.59956	E11.5 * Sox10 down vs E11.5 * Ctrl
10358224	Ptprc	NM_001111316	1.19795e-007	-3.63818	E11.5 * Sox10 down vs E11.5 * Ctrl
10493831	S100a8	NM_013650	3.05022e-006	-3.66497	E11.5 * Sox10 down vs E11.5 * Ctrl
10523134	Pf4	NM_019932	6.12227e-007	-3.6731	E11.5 * Sox10 down vs E11.5 * Ctrl
10380174	Mpo	NM_010824	2.41574e-011	-3.70399	E11.5 * Sox10 down vs E11.5 * Ctrl
10366153	Rassf9	NM_146240	1.0052e-008	-3.71499	E11.5 * Sox10 down vs E11.5 * Ctrl
10498935	Gucy1b3	NM_017469	3.36468e-007	-3.73139	E11.5 * Sox10 down vs E11.5 * Ctrl
10362674	Rnu3a	NR_002842	6.35727e-005	-3.73733	E11.5 * Sox10 down vs E11.5 * Ctrl
10554789	Ctsc	NM_009982	8.00992e-009	-3.74324	E11.5 * Sox10 down vs E11.5 * Ctrl
10355893	Epha4	NM_007936	2.45549e-011	-3.77061	E11.5 * Sox10 down vs E11.5 * Ctrl
10405179	S1pr3	NM_010101	3.25685e-011	-3.77632	E11.5 * Sox10 down vs E11.5 * Ctrl
10591129	Fat3	NM_001080814	6.76641e-006	-3.77842	E11.5 * Sox10 down vs E11.5 * Ctrl
10498653	1110032A04Rik	NM_133675	2.63229e-007	-3.78776	E11.5 * Sox10 down vs E11.5 * Ctrl
10423971	Pkhd1l1	NM_138674	6.26841e-016	-3.81836	E11.5 * Sox10 down vs E11.5 * Ctrl
10536444	Foxp2	NM_053242	7.11971e-010	-3.82555	E11.5 * Sox10 down vs E11.5 * Ctrl
10405587	Tgfbi	NM_009369	3.33262e-008	-3.82603	E11.5 * Sox10 down vs E11.5 * Ctrl
10603551	Cybb	NM_007807	2.10708e-007	-3.82733	E11.5 * Sox10 down vs E11.5 * Ctrl
10369842		---	1.1944e-005	-3.84918	E11.5 * Sox10 down vs E11.5 * Ctrl
10541075	Cxcl12	NM_001012477	1.64336e-007	-3.8559	E11.5 * Sox10 down vs E11.5 * Ctrl
10606369	Itm2a	NM_008409	1.36366e-006	-3.87122	E11.5 * Sox10 down vs E11.5 * Ctrl
10530029	Lgi2	NM_144945	3.41906e-010	-3.89495	E11.5 * Sox10 down vs E11.5 * Ctrl
10595211	Col12a1	NM_007730	4.38845e-009	-3.89611	E11.5 * Sox10 down vs E11.5 * Ctrl
10546430	Adamts9	NM_175314	3.32209e-007	-3.94599	E11.5 * Sox10 down vs E11.5 * Ctrl
10568436	Fgfr2	NM_010207	5.81455e-008	-3.95297	E11.5 * Sox10 down vs E11.5 * Ctrl
10498952	Gucy1a3	NM_021896	4.39693e-010	-3.95571	E11.5 * Sox10 down vs E11.5 * Ctrl
10578136		---	1.60941e-006	-3.96114	E11.5 * Sox10 down vs E11.5 * Ctrl
10446965	Rasgrp3	NM_207246	4.63374e-009	-3.97075	E11.5 * Sox10 down vs E11.5 * Ctrl
10559790	Zim1	NM_011769	1.16755e-008	-3.97266	E11.5 * Sox10 down vs E11.5 * Ctrl
10546454	Adamts9	NM_175314	7.85159e-010	-3.97283	E11.5 * Sox10 down vs E11.5 * Ctrl
10491732	Fat4	NM_183221	8.69322e-009	-4.02844	E11.5 * Sox10 down vs E11.5 * Ctrl
10419261	Bmp4	NM_007554	3.43195e-010	-4.06215	E11.5 * Sox10 down vs E11.5 * Ctrl
10513256	Lpar1	NM_010336	5.31202e-009	-4.07764	E11.5 * Sox10 down vs E11.5 * Ctrl
10604175	Fam70a	NM_172930	4.44549e-008	-4.08281	E11.5 * Sox10 down vs E11.5 * Ctrl
10467826	LOC545291	NM_001081257	4.92444e-009	-4.09196	E11.5 * Sox10 down vs E11.5 * Ctrl
10396419		---	1.27105e-005	-4.10006	E11.5 * Sox10 down vs E11.5 * Ctrl
10569344	Igf2	NM_001122737	2.92397e-007	-4.10764	E11.5 * Sox10 down vs E11.5 * Ctrl
10537296		---	1.21846e-006	-4.15988	E11.5 * Sox10 down vs E11.5 * Ctrl
10423520	Sema5a	NM_009154	6.55527e-010	-4.20304	E11.5 * Sox10 down vs E11.5 * Ctrl
10542592	ENSMUSG0000C	AK143453	3.76844e-010	-4.23348	E11.5 * Sox10 down vs E11.5 * Ctrl
10427402	Ghr	NM_010284	4.3513e-010	-4.26137	E11.5 * Sox10 down vs E11.5 * Ctrl
10397645	Gpr65	NM_008152	1.63863e-007	-4.26456	E11.5 * Sox10 down vs E11.5 * Ctrl
10569341	H19	NR_001592	2.54662e-006	-4.28589	E11.5 * Sox10 down vs E11.5 * Ctrl
10574438	Cdh5	NM_009868	2.13384e-007	-4.2929	E11.5 * Sox10 down vs E11.5 * Ctrl
10514088	Frem1	NM_177863	2.90456e-011	-4.29655	E11.5 * Sox10 down vs E11.5 * Ctrl

10433776	Snai2	NM_011415	2.76213e-009	-4.35899	E11.5 * Sox10 down vs E11.5 * Ctrl
10531177	Adamts3	NM_001081401	6.0849e-010	-4.37845	E11.5 * Sox10 down vs E11.5 * Ctrl
10546432	Adamts9	NM_175314	1.01492e-005	-4.4344	E11.5 * Sox10 down vs E11.5 * Ctrl
10484197	2610301F02Rik	ENSMUST00000(	5.82914e-010	-4.45613	E11.5 * Sox10 down vs E11.5 * Ctrl
10484371	Calcrl	NM_018782	8.22082e-010	-4.46082	E11.5 * Sox10 down vs E11.5 * Ctrl
10511363	Penk	NM_001002927	2.41885e-009	-4.52269	E11.5 * Sox10 down vs E11.5 * Ctrl
10369615	Srgn	NM_011157	3.97657e-009	-4.52623	E11.5 * Sox10 down vs E11.5 * Ctrl
10450484	Aif1	NM_019467	4.33283e-007	-4.55402	E11.5 * Sox10 down vs E11.5 * Ctrl
10500559	Hsd3b6	NM_013821	1.16678e-008	-4.57241	E11.5 * Sox10 down vs E11.5 * Ctrl
10490923	Car2	NM_009801	2.11969e-009	-4.66295	E11.5 * Sox10 down vs E11.5 * Ctrl
10576049	Foxf1a	ENSMUST00000(	2.42989e-007	-4.66669	E11.5 * Sox10 down vs E11.5 * Ctrl
10356880	St8sia4	NM_009183	3.78296e-012	-4.75889	E11.5 * Sox10 down vs E11.5 * Ctrl
10416437	Lcp1	NM_008879	6.3793e-010	-4.76913	E11.5 * Sox10 down vs E11.5 * Ctrl
10499861	S100a9	NM_009114	2.48624e-008	-4.7748	E11.5 * Sox10 down vs E11.5 * Ctrl
10404606	Ly86	NM_010745	1.69728e-008	-4.78324	E11.5 * Sox10 down vs E11.5 * Ctrl
10502770	Lphn2	NM_001081298	2.22955e-007	-4.81555	E11.5 * Sox10 down vs E11.5 * Ctrl
10563611	Saa1	NM_009117	1.08877e-006	-4.84621	E11.5 * Sox10 down vs E11.5 * Ctrl
10422728	Dab2	NM_023118	4.13e-009	-4.86284	E11.5 * Sox10 down vs E11.5 * Ctrl
10490775	ENSMUSG0000C	ENSMUST00000(	1.58714e-007	-4.89209	E11.5 * Sox10 down vs E11.5 * Ctrl
10548892	Arhgdib	NM_007486	8.35621e-007	-4.90331	E11.5 * Sox10 down vs E11.5 * Ctrl
10435948	Ccdc80	NM_026439	9.23557e-010	-4.91221	E11.5 * Sox10 down vs E11.5 * Ctrl
10578950	ENSMUSG0000C	ENSMUST00000(	2.78769e-009	-4.91252	E11.5 * Sox10 down vs E11.5 * Ctrl
10601519	Klhl4	NM_172781	2.28625e-008	-4.92639	E11.5 * Sox10 down vs E11.5 * Ctrl
10368240	Tcf21	NM_011545	7.58982e-008	-4.95967	E11.5 * Sox10 down vs E11.5 * Ctrl
10406254	Ell2	NM_138953	1.38806e-010	-5.00331	E11.5 * Sox10 down vs E11.5 * Ctrl
10438445	Klhl6	NM_183390	1.71973e-011	-5.02826	E11.5 * Sox10 down vs E11.5 * Ctrl
10500780	Nr1h5	NM_198658	8.92468e-010	-5.04268	E11.5 * Sox10 down vs E11.5 * Ctrl
10592533		---	8.23046e-010	-5.04681	E11.5 * Sox10 down vs E11.5 * Ctrl
10486041	Meis2	NM_001136072	2.52245e-008	-5.0704	E11.5 * Sox10 down vs E11.5 * Ctrl
10571840	Hpgd	NM_008278	1.35888e-008	-5.0748	E11.5 * Sox10 down vs E11.5 * Ctrl
10523451	Anxa3	NM_013470	1.88849e-008	-5.08581	E11.5 * Sox10 down vs E11.5 * Ctrl
10551883	Tyrobp	NM_011662	1.5028e-009	-5.09825	E11.5 * Sox10 down vs E11.5 * Ctrl
10566272	Hbb-y	NM_008221	0.000470412	-5.15246	E11.5 * Sox10 down vs E11.5 * Ctrl
10365974	Dcn	NM_007833	2.32847e-006	-5.15402	E11.5 * Sox10 down vs E11.5 * Ctrl
10365983	Lum	NM_008524	3.60167e-006	-5.18261	E11.5 * Sox10 down vs E11.5 * Ctrl
10586865	Aldh1a2	NM_009022	5.3223e-008	-5.24151	E11.5 * Sox10 down vs E11.5 * Ctrl
10473444	Aplnr	NM_011784	6.83215e-011	-5.2821	E11.5 * Sox10 down vs E11.5 * Ctrl
10472958	Hoxd10	NM_013554	1.60178e-006	-5.31234	E11.5 * Sox10 down vs E11.5 * Ctrl
10461614	Ms4a6c	NM_028595	3.60603e-010	-5.36986	E11.5 * Sox10 down vs E11.5 * Ctrl
10523359	Cxcl13	NM_018866	2.35898e-009	-5.44001	E11.5 * Sox10 down vs E11.5 * Ctrl
10531724	Plac8	NM_139198	3.89079e-006	-5.44789	E11.5 * Sox10 down vs E11.5 * Ctrl
10428376	Angpt1	NM_009640	3.90701e-010	-5.49232	E11.5 * Sox10 down vs E11.5 * Ctrl
10536635	A430107O13Rik	NM_001081351	3.2466e-010	-5.50308	E11.5 * Sox10 down vs E11.5 * Ctrl
10546450	Adamts9	NM_175314	1.19812e-008	-5.53412	E11.5 * Sox10 down vs E11.5 * Ctrl
10451677		---	2.93729e-011	-5.54445	E11.5 * Sox10 down vs E11.5 * Ctrl
10523756		---	2.93729e-011	-5.54445	E11.5 * Sox10 down vs E11.5 * Ctrl
10537504		---	2.93729e-011	-5.54445	E11.5 * Sox10 down vs E11.5 * Ctrl
10466800	Pgm5	NM_175013	1.53429e-007	-5.5616	E11.5 * Sox10 down vs E11.5 * Ctrl
10385770	Olfr1372-ps1	BC055827	4.52315e-008	-5.57908	E11.5 * Sox10 down vs E11.5 * Ctrl
10574415	1700047G07Rik	ENSMUST00000(	6.04048e-007	-5.64059	E11.5 * Sox10 down vs E11.5 * Ctrl
10601942	Nrk	NM_013724	1.12643e-007	-5.64509	E11.5 * Sox10 down vs E11.5 * Ctrl
10542981	Gmfg	NM_022024	5.64485e-009	-5.65262	E11.5 * Sox10 down vs E11.5 * Ctrl
10369023	Fam162b	BC147085	1.14648e-008	-5.65496	E11.5 * Sox10 down vs E11.5 * Ctrl
10554129	B130024G19Rik	BC070425	1.82831e-010	-5.75347	E11.5 * Sox10 down vs E11.5 * Ctrl
10422760	Fyb	NM_011815	2.9494e-010	-5.78396	E11.5 * Sox10 down vs E11.5 * Ctrl
10394534	Osr1	NM_011859	2.1962e-007	-5.79921	E11.5 * Sox10 down vs E11.5 * Ctrl
10512757	Hemgn	NM_053149	1.55487e-011	-5.85506	E11.5 * Sox10 down vs E11.5 * Ctrl
10351905	Spna1	NM_011465	5.99484e-013	-6.10264	E11.5 * Sox10 down vs E11.5 * Ctrl
10439299	Stfa3	NM_025288	1.92964e-008	-6.18149	E11.5 * Sox10 down vs E11.5 * Ctrl
10357488	Cd55	NM_010016	7.33204e-010	-6.24881	E11.5 * Sox10 down vs E11.5 * Ctrl
10522503	Pdgfra	NM_011058	4.55738e-008	-6.28812	E11.5 * Sox10 down vs E11.5 * Ctrl
10484283	Pde1a	NM_016744	9.33463e-011	-6.30479	E11.5 * Sox10 down vs E11.5 * Ctrl
10461622	Ms4a6b	NM_027209	2.75174e-008	-6.37151	E11.5 * Sox10 down vs E11.5 * Ctrl
10544383	Kel	NM_032540	8.64593e-015	-6.5051	E11.5 * Sox10 down vs E11.5 * Ctrl
10570434	Ifitm1	NM_026820	1.9994e-009	-6.5132	E11.5 * Sox10 down vs E11.5 * Ctrl
10547657	C3ar1	NM_009779	6.34865e-010	-6.5211	E11.5 * Sox10 down vs E11.5 * Ctrl

10480090	Itga8	NM_001001309	4.55309e-010	-6.54842	E11.5 * Sox10 down vs E11.5 * Ctrl
10494271	Ctss	NM_021281	2.29271e-009	-6.75572	E11.5 * Sox10 down vs E11.5 * Ctrl
10587231	Bmp5	NM_007555	1.20456e-008	-6.78747	E11.5 * Sox10 down vs E11.5 * Ctrl
10496359	Emcn	NM_016885	7.89718e-012	-7.1768	E11.5 * Sox10 down vs E11.5 * Ctrl
10500796		---	1.03092e-009	-7.49488	E11.5 * Sox10 down vs E11.5 * Ctrl
10347931	C130026I21Rik	BC007193	7.84939e-009	-7.59822	E11.5 * Sox10 down vs E11.5 * Ctrl
10445192	Rhag	NM_011269	9.62904e-012	-7.7113	E11.5 * Sox10 down vs E11.5 * Ctrl
10469358	Mrc1	NM_008625	3.02113e-011	-7.80227	E11.5 * Sox10 down vs E11.5 * Ctrl
10600024	Gpr50	NM_010340	2.72401e-009	-8.29513	E11.5 * Sox10 down vs E11.5 * Ctrl
10423917		---	7.65092e-006	-8.65404	E11.5 * Sox10 down vs E11.5 * Ctrl
10359504	Dnm3os	NR_002870	2.77644e-008	-8.70662	E11.5 * Sox10 down vs E11.5 * Ctrl
10574023	Mt2	NM_008630	7.02559e-010	-9.0011	E11.5 * Sox10 down vs E11.5 * Ctrl
10573054	Gypa	NM_010369	1.28306e-010	-9.29867	E11.5 * Sox10 down vs E11.5 * Ctrl
10375046	Hba-x	NM_010405	3.01694e-007	-10.6769	E11.5 * Sox10 down vs E11.5 * Ctrl
10566254	Hbb-b1	NM_008220	2.29806e-007	-11.6445	E11.5 * Sox10 down vs E11.5 * Ctrl
10566258	Hbb-b1	ENSMUST00000(	1.41703e-007	-11.7301	E11.5 * Sox10 down vs E11.5 * Ctrl
10439292	BC100530	NM_001082546	7.32318e-010	-13.3566	E11.5 * Sox10 down vs E11.5 * Ctrl
10375051	Hba-a1	NM_008218	1.42568e-007	-13.6785	E11.5 * Sox10 down vs E11.5 * Ctrl
10375058	Hba-a2	NM_001083955	8.48747e-008	-13.9448	E11.5 * Sox10 down vs E11.5 * Ctrl
10566268	Hbb-bh1	NM_008219	6.28451e-010	-30.2574	E11.5 * Sox10 down vs E11.5 * Ctrl

W11 vs S11

Probeset ID	Gene Symbol	RefSeq	p-value	Fold-change	Comparison
10406845	Foxd1	NM_008242	1.06825e-006	4.43936	E11.5 * Wnt1 up vs E11.5 * Sox10
10359624	Prrx1	NM_175686	7.85618e-006	2.41211	E11.5 * Wnt1 up vs E11.5 * Sox10
10374777	Efemp1	NM_146015	1.68387e-005	1.97954	E11.5 * Wnt1 up vs E11.5 * Sox10
10604961	Gabra3	NM_008067	1.19612e-005	1.62065	E11.5 * Wnt1 up vs E11.5 * Sox10
10483081	Fap	NM_007986	6.11863e-005	1.56946	E11.5 * Wnt1 up vs E11.5 * Sox10
10418300	Cacna2d3	NM_009785	4.75558e-005	1.5382	E11.5 * Wnt1 up vs E11.5 * Sox10
10542369	EG668137	NM_001142734	8.79081e-005	1.52643	E11.5 * Wnt1 up vs E11.5 * Sox10
10427796	Npr3	NM_008728	3.01705e-005	1.43848	E11.5 * Wnt1 up vs E11.5 * Sox10
10535017	Gal3st4	NM_001033416	4.50238e-005	1.40859	E11.5 * Wnt1 up vs E11.5 * Sox10
10410766	Nr2f1	NM_010151	2.884e-006	1.39689	E11.5 * Wnt1 up vs E11.5 * Sox10
10440258	Epha3	NM_010140	6.42963e-006	1.34751	E11.5 * Wnt1 up vs E11.5 * Sox10
10395129	Tmem18	NM_172049	4.02787e-005	1.33119	E11.5 * Wnt1 up vs E11.5 * Sox10
10414025	Gdf10	NM_145741	0.00012492	1.24395	E11.5 * Wnt1 up vs E11.5 * Sox10
10520234	2010209O12Rik	NM_133913	6.2418e-005	1.23603	E11.5 * Wnt1 up vs E11.5 * Sox10
10513869	Megf9	NM_172694	8.79596e-005	1.23126	E11.5 * Wnt1 up vs E11.5 * Sox10
10504424	Reck	NM_016678	8.62674e-005	1.22364	E11.5 * Wnt1 up vs E11.5 * Sox10
10578262		---	3.19624e-005	1.21652	E11.5 * Wnt1 up vs E11.5 * Sox10
10437330	Crebbp	ENSMUST00000	1.6752e-005	1.18246	E11.5 * Wnt1 up vs E11.5 * Sox10
10581910	Tmem170	NM_025781	6.52998e-005	1.17136	E11.5 * Wnt1 up vs E11.5 * Sox10
10367393	Wibg	NM_030100	4.186e-005	1.15889	E11.5 * Wnt1 up vs E11.5 * Sox10
10465649	Mark2	NM_007928	8.24369e-005	1.11486	E11.5 * Wnt1 up vs E11.5 * Sox10
10550052	Rps5	NM_009095	4.9987e-005	-1.06324	E11.5 * Wnt1 down vs E11.5 * Sox10
10408684	Tmed10	NM_026775	7.36797e-005	-1.10395	E11.5 * Wnt1 down vs E11.5 * Sox10
10366004	Atp2b1	NM_026482	4.17805e-005	-1.14076	E11.5 * Wnt1 down vs E11.5 * Sox10
10437392	Magmas	NM_025571	0.000124983	-1.14544	E11.5 * Wnt1 down vs E11.5 * Sox10
10395739	Srp54b	NM_001100109	1.40352e-005	-1.17538	E11.5 * Wnt1 down vs E11.5 * Sox10
10395788	Srp54c	NM_001100110	2.27304e-005	-1.19605	E11.5 * Wnt1 down vs E11.5 * Sox10
10368670	Amd1	NM_009665	5.9218e-005	-1.1964	E11.5 * Wnt1 down vs E11.5 * Sox10
10405693	Dapk1	NM_029653	2.05131e-006	-1.19662	E11.5 * Wnt1 down vs E11.5 * Sox10
10375234	Nudcd2	NM_026023	0.00010129	-1.19821	E11.5 * Wnt1 down vs E11.5 * Sox10
10357133		---	0.000103372	-1.21712	E11.5 * Wnt1 down vs E11.5 * Sox10
10375893	Sar1b	NM_025535	7.22255e-005	-1.26228	E11.5 * Wnt1 down vs E11.5 * Sox10
10542397	H2afj	NM_177688	1.34479e-005	-1.26291	E11.5 * Wnt1 down vs E11.5 * Sox10
10599627	Hprt1	NM_013556	2.45264e-005	-1.30728	E11.5 * Wnt1 down vs E11.5 * Sox10
10578904	Cpe	NM_013494	3.77055e-005	-1.30998	E11.5 * Wnt1 down vs E11.5 * Sox10
10597354	Pdcd6ip	NM_011052	6.97532e-005	-1.31462	E11.5 * Wnt1 down vs E11.5 * Sox10
10607143	Capn6	NM_007603	0.000118499	-1.31477	E11.5 * Wnt1 down vs E11.5 * Sox10
10426016	Gtse1	NM_013882	7.86591e-005	-1.3342	E11.5 * Wnt1 down vs E11.5 * Sox10
10355984	Serpine2	NM_009255	1.82956e-006	-1.35654	E11.5 * Wnt1 down vs E11.5 * Sox10
10449644	Glo1	NM_025374	9.99336e-005	-1.36214	E11.5 * Wnt1 down vs E11.5 * Sox10
10394812		---	1.03421e-005	-1.40148	E11.5 * Wnt1 down vs E11.5 * Sox10
10415332	Rec8	NM_020002	3.33932e-005	-1.40241	E11.5 * Wnt1 down vs E11.5 * Sox10

10565852	Rnf169	AK173319	1.64563e-005	-1.42764	E11.5 * Wnt1 down vs E11.5 * Sox10
10424105	Colec10	NM_173422	0.000127562	-1.43824	E11.5 * Wnt1 down vs E11.5 * Sox10
10487011	Gatm	NM_025961	4.81421e-005	-1.46524	E11.5 * Wnt1 down vs E11.5 * Sox10
10411306	Polk	NM_012048	2.00172e-007	-1.47532	E11.5 * Wnt1 down vs E11.5 * Sox10
10531869	Mapk10	NM_009158	0.000122534	-1.5139	E11.5 * Wnt1 down vs E11.5 * Sox10
10369783	Zfp365	NM_178679	6.4553e-006	-1.52625	E11.5 * Wnt1 down vs E11.5 * Sox10
10384145	H2afv	BC028539	3.79458e-005	-1.55598	E11.5 * Wnt1 down vs E11.5 * Sox10
10596637	Mapkapk3	NM_178907	5.59909e-005	-1.56538	E11.5 * Wnt1 down vs E11.5 * Sox10
10469457	Plxdc2	NM_026162	1.33091e-007	-1.58333	E11.5 * Wnt1 down vs E11.5 * Sox10
10359480	Dnm3	NM_001038619	1.66468e-005	-1.6252	E11.5 * Wnt1 down vs E11.5 * Sox10
10492640	Fstl5	NM_178673	5.86455e-005	-1.66399	E11.5 * Wnt1 down vs E11.5 * Sox10
10519886	Sema3c	NM_013657	4.61139e-008	-1.6679	E11.5 * Wnt1 down vs E11.5 * Sox10
10495316	Psrc1	NM_019976	9.18041e-006	-1.69948	E11.5 * Wnt1 down vs E11.5 * Sox10
10472893	B230120H23Rik	NM_023057	4.23804e-006	-1.73014	E11.5 * Wnt1 down vs E11.5 * Sox10
10416437	Lcp1	NM_008879	0.000123417	-1.74353	E11.5 * Wnt1 down vs E11.5 * Sox10
10503259	Trp53inp1	NM_021897	1.65272e-007	-1.7631	E11.5 * Wnt1 down vs E11.5 * Sox10
10442327	Cldn6	NM_018777	2.6515e-005	-1.76918	E11.5 * Wnt1 down vs E11.5 * Sox10
10565570	4632434I11Rik	NM_001080995	6.58022e-006	-1.79461	E11.5 * Wnt1 down vs E11.5 * Sox10
10407126	Plk2	NM_152804	6.98428e-006	-1.83079	E11.5 * Wnt1 down vs E11.5 * Sox10
10481272	1700007K13Rik	BC099566	2.5565e-006	-1.92606	E11.5 * Wnt1 down vs E11.5 * Sox10
10416230	Tnfrsf10b	NM_020275	5.32439e-005	-1.95445	E11.5 * Wnt1 down vs E11.5 * Sox10
10551883	Tyrobp	NM_011662	4.94282e-005	-1.97195	E11.5 * Wnt1 down vs E11.5 * Sox10
10425257	Polr2f	NM_027231	1.83274e-005	-2.05542	E11.5 * Wnt1 down vs E11.5 * Sox10
10604175	Fam70a	NM_172930	4.45268e-005	-2.16254	E11.5 * Wnt1 down vs E11.5 * Sox10
10530100	Arap2	NM_178407	2.41693e-007	-2.22792	E11.5 * Wnt1 down vs E11.5 * Sox10
10467319	Rbp4	NM_001159487	5.78784e-006	-2.38044	E11.5 * Wnt1 down vs E11.5 * Sox10
10485745	Ano3	NM_001128103	2.93343e-005	-2.5388	E11.5 * Wnt1 down vs E11.5 * Sox10
10430447	1700088E04Rik	NM_138581	1.04669e-007	-2.60484	E11.5 * Wnt1 down vs E11.5 * Sox10
10605874	Eda2r	NM_175540	2.60763e-006	-3.14677	E11.5 * Wnt1 down vs E11.5 * Sox10
10425240	Micall1	NM_177461	1.31109e-010	-3.3271	E11.5 * Wnt1 down vs E11.5 * Sox10
10385271	Ccng1	NM_009831	6.50571e-012	-3.48595	E11.5 * Wnt1 down vs E11.5 * Sox10
10566268	Hbb-bh1	NM_008219	3.09622e-005	-4.0251	E11.5 * Wnt1 down vs E11.5 * Sox10
10375046	Hba-x	NM_010405	4.9479e-005	-4.48505	E11.5 * Wnt1 down vs E11.5 * Sox10
10566258	Hbb-b1	ENSMUST00000	3.66923e-005	-4.52825	E11.5 * Wnt1 down vs E11.5 * Sox10
10485718	Ano3	NM_001128103	8.2266e-009	-4.84739	E11.5 * Wnt1 down vs E11.5 * Sox10
10566254	Hbb-b1	NM_008220	2.46268e-005	-5.09403	E11.5 * Wnt1 down vs E11.5 * Sox10
10443463	Cdkn1a	NM_007669	2.8372e-011	-6.17501	E11.5 * Wnt1 down vs E11.5 * Sox10

W15 vs S15

Probeset ID	Gene Symbol	RefSeq	p-value	Fold-change	Comparison
10564163	Snord116	NR_002895	9.23217e-007	4.4914	E15.5 * Wnt1 up vs E15.5 * Sox10
10564167	Snord116	NR_002895	9.23217e-007	4.4914	E15.5 * Wnt1 up vs E15.5 * Sox10
10564171	Snord116	NR_002895	9.23217e-007	4.4914	E15.5 * Wnt1 up vs E15.5 * Sox10
10564173	Snord116	NR_002895	9.23217e-007	4.4914	E15.5 * Wnt1 up vs E15.5 * Sox10
10564175	Snord116	NR_002895	9.23217e-007	4.4914	E15.5 * Wnt1 up vs E15.5 * Sox10
10564179	Snord116	NR_002895	9.23217e-007	4.4914	E15.5 * Wnt1 up vs E15.5 * Sox10
10564181	Snord116	NR_002895	9.23217e-007	4.4914	E15.5 * Wnt1 up vs E15.5 * Sox10
10564185	Snord116	NR_002895	9.23217e-007	4.4914	E15.5 * Wnt1 up vs E15.5 * Sox10
10564187	Snord116	NR_002895	9.23217e-007	4.4914	E15.5 * Wnt1 up vs E15.5 * Sox10
10564189	Snord116	NR_002895	9.23217e-007	4.4914	E15.5 * Wnt1 up vs E15.5 * Sox10
10564191	Snord116	NR_002895	9.23217e-007	4.4914	E15.5 * Wnt1 up vs E15.5 * Sox10
10564193	Snord116	NR_002895	9.23217e-007	4.4914	E15.5 * Wnt1 up vs E15.5 * Sox10
10564195	Snord116	NR_002895	9.23217e-007	4.4914	E15.5 * Wnt1 up vs E15.5 * Sox10
10564197	Snord116	NR_002895	9.23217e-007	4.4914	E15.5 * Wnt1 up vs E15.5 * Sox10
10564199	Snord116	NR_002895	9.23217e-007	4.4914	E15.5 * Wnt1 up vs E15.5 * Sox10
10564201	Snord116	AF241256	9.23217e-007	4.4914	E15.5 * Wnt1 up vs E15.5 * Sox10
10564205	Snord116	AF241256	9.23217e-007	4.4914	E15.5 * Wnt1 up vs E15.5 * Sox10
10564207	Snord116	AF241256	9.23217e-007	4.4914	E15.5 * Wnt1 up vs E15.5 * Sox10
10564165	Snord116	AF241256	5.77436e-007	4.49031	E15.5 * Wnt1 up vs E15.5 * Sox10
10472398	Scn2a1	NM_001099298	1.54135e-006	4.45106	E15.5 * Wnt1 up vs E15.5 * Sox10
10564161	Snord116	NR_002895	7.40581e-007	4.44278	E15.5 * Wnt1 up vs E15.5 * Sox10
10564177	Snord116	AF241256	8.1004e-007	4.4046	E15.5 * Wnt1 up vs E15.5 * Sox10
10564169	Snord116	AF241256	4.68105e-007	4.37933	E15.5 * Wnt1 up vs E15.5 * Sox10

10564159	Snord116	AF241256	6.64353e-007	4.34272	E15.5 * Wnt1 up vs E15.5 * Sox10
10564183	Snord116	AF241256	6.2471e-007	4.30258	E15.5 * Wnt1 up vs E15.5 * Sox10
10421934	Klhl1	NM_053105	3.65971e-010	3.91372	E15.5 * Wnt1 up vs E15.5 * Sox10
10472384	Scn2a1	NM_001099298	7.61648e-008	3.88985	E15.5 * Wnt1 up vs E15.5 * Sox10
10428509	Csmd3	NM_001081391	0.000158309	3.78558	E15.5 * Wnt1 up vs E15.5 * Sox10
10472400	Scn2a1	NM_001099298	8.34814e-005	3.74816	E15.5 * Wnt1 up vs E15.5 * Sox10
10428515	Csmd3	NM_001081391	3.605e-007	3.72218	E15.5 * Wnt1 up vs E15.5 * Sox10
10503416	Calb1	NM_009788	7.40222e-011	3.66818	E15.5 * Wnt1 up vs E15.5 * Sox10
10564011	Snord115	AF357427	3.17709e-009	3.49577	E15.5 * Wnt1 up vs E15.5 * Sox10
10472372	Scn2a1	NM_001099298	6.89819e-009	3.42322	E15.5 * Wnt1 up vs E15.5 * Sox10
10563913		---	5.51642e-009	3.38529	E15.5 * Wnt1 up vs E15.5 * Sox10
10563917		---	5.51642e-009	3.38529	E15.5 * Wnt1 up vs E15.5 * Sox10
10563923	100040985	XM_001475615	5.51642e-009	3.38529	E15.5 * Wnt1 up vs E15.5 * Sox10
10563937	Snord115	AF357427	5.38857e-008	3.32803	E15.5 * Wnt1 up vs E15.5 * Sox10
10563947	Snord115	AF357427	5.38857e-008	3.32803	E15.5 * Wnt1 up vs E15.5 * Sox10
10563951	Snord115	AF357427	5.38857e-008	3.32803	E15.5 * Wnt1 up vs E15.5 * Sox10
10563953	Snord115	AF357427	5.38857e-008	3.32803	E15.5 * Wnt1 up vs E15.5 * Sox10
10563957	Snord115	AF357427	5.38857e-008	3.32803	E15.5 * Wnt1 up vs E15.5 * Sox10
10563995	Snord115	AF357427	5.38857e-008	3.32803	E15.5 * Wnt1 up vs E15.5 * Sox10
10563997	Snord115	AF357427	5.38857e-008	3.32803	E15.5 * Wnt1 up vs E15.5 * Sox10
10563999	Snord115	AF357427	5.38857e-008	3.32803	E15.5 * Wnt1 up vs E15.5 * Sox10
10564001	Snord115	AF357427	5.38857e-008	3.32803	E15.5 * Wnt1 up vs E15.5 * Sox10
10564003	Snord115	AF357427	5.38857e-008	3.32803	E15.5 * Wnt1 up vs E15.5 * Sox10
10564007	Snord115	AF357427	5.38857e-008	3.32803	E15.5 * Wnt1 up vs E15.5 * Sox10
10564029	Snord115	AF357427	5.38857e-008	3.32803	E15.5 * Wnt1 up vs E15.5 * Sox10
10564031	Snord115	AF357427	5.38857e-008	3.32803	E15.5 * Wnt1 up vs E15.5 * Sox10
10564035		---	5.38857e-008	3.32803	E15.5 * Wnt1 up vs E15.5 * Sox10
10564039		---	5.38857e-008	3.32803	E15.5 * Wnt1 up vs E15.5 * Sox10
10564051		---	5.38857e-008	3.32803	E15.5 * Wnt1 up vs E15.5 * Sox10
10564059		---	5.38857e-008	3.32803	E15.5 * Wnt1 up vs E15.5 * Sox10
10564061		---	5.38857e-008	3.32803	E15.5 * Wnt1 up vs E15.5 * Sox10
10564063		---	5.38857e-008	3.32803	E15.5 * Wnt1 up vs E15.5 * Sox10
10564065	100040985	XM_001475615	5.38857e-008	3.32803	E15.5 * Wnt1 up vs E15.5 * Sox10
10564067		---	5.38857e-008	3.32803	E15.5 * Wnt1 up vs E15.5 * Sox10
10564071		---	5.38857e-008	3.32803	E15.5 * Wnt1 up vs E15.5 * Sox10
10564075		---	5.38857e-008	3.32803	E15.5 * Wnt1 up vs E15.5 * Sox10
10564081		---	5.38857e-008	3.32803	E15.5 * Wnt1 up vs E15.5 * Sox10
10564083		---	5.38857e-008	3.32803	E15.5 * Wnt1 up vs E15.5 * Sox10
10564131		---	5.38857e-008	3.32803	E15.5 * Wnt1 up vs E15.5 * Sox10
10564133		---	5.38857e-008	3.32803	E15.5 * Wnt1 up vs E15.5 * Sox10
10564141		---	5.38857e-008	3.32803	E15.5 * Wnt1 up vs E15.5 * Sox10
10564145		---	5.38857e-008	3.32803	E15.5 * Wnt1 up vs E15.5 * Sox10
10564149		---	5.38857e-008	3.32803	E15.5 * Wnt1 up vs E15.5 * Sox10
10564151		---	5.38857e-008	3.32803	E15.5 * Wnt1 up vs E15.5 * Sox10
10564153		---	5.38857e-008	3.32803	E15.5 * Wnt1 up vs E15.5 * Sox10
10564155		---	5.38857e-008	3.32803	E15.5 * Wnt1 up vs E15.5 * Sox10
10564041		---	2.53768e-008	3.30017	E15.5 * Wnt1 up vs E15.5 * Sox10
10423287	Cdh18	NM_001081299	4.53399e-005	3.20925	E15.5 * Wnt1 up vs E15.5 * Sox10
10472380	Scn2a1	NM_001099298	2.85687e-006	3.17621	E15.5 * Wnt1 up vs E15.5 * Sox10
10501468	Ntng1	NM_030699	4.87323e-009	3.16544	E15.5 * Wnt1 up vs E15.5 * Sox10
10472378	Scn2a1	NM_001099298	7.4977e-005	3.08737	E15.5 * Wnt1 up vs E15.5 * Sox10
10472386	Scn2a1	NM_001099298	1.319e-007	3.0851	E15.5 * Wnt1 up vs E15.5 * Sox10
10472402	Scn2a1	NM_001099298	2.48838e-008	3.07615	E15.5 * Wnt1 up vs E15.5 * Sox10
10398360		---	2.69563e-006	3.05786	E15.5 * Wnt1 up vs E15.5 * Sox10
10483324	Scn9a	NM_018852	1.05091e-007	2.99374	E15.5 * Wnt1 up vs E15.5 * Sox10
10536363	Tac1	NM_009311	1.53902e-005	2.97131	E15.5 * Wnt1 up vs E15.5 * Sox10
10358928	Cacna1e	NM_009782	3.27347e-010	2.94801	E15.5 * Wnt1 up vs E15.5 * Sox10
10563935		---	2.13245e-008	2.89901	E15.5 * Wnt1 up vs E15.5 * Sox10
10564045		---	2.13245e-008	2.89901	E15.5 * Wnt1 up vs E15.5 * Sox10
10564047		---	2.13245e-008	2.89901	E15.5 * Wnt1 up vs E15.5 * Sox10
10483326	Scn9a	NM_018852	4.83817e-008	2.8611	E15.5 * Wnt1 up vs E15.5 * Sox10
10366457	Ptprr	NM_011217	2.67422e-009	2.85487	E15.5 * Wnt1 up vs E15.5 * Sox10
10563993		---	6.95379e-009	2.81469	E15.5 * Wnt1 up vs E15.5 * Sox10
10564021		---	6.95379e-009	2.81469	E15.5 * Wnt1 up vs E15.5 * Sox10
10564025		---	6.95379e-009	2.81469	E15.5 * Wnt1 up vs E15.5 * Sox10
10601569	Pcdh11x	NM_001081385	8.84672e-005	2.81431	E15.5 * Wnt1 up vs E15.5 * Sox10

10453518	Nrxn1	NM_020252	7.76111e-008	2.80793	E15.5 * Wnt1 up vs E15.5 * Sox10
10350506	Fam5c	NM_153539	2.91859e-009	2.80506	E15.5 * Wnt1 up vs E15.5 * Sox10
10563965		---	7.37823e-010	2.79847	E15.5 * Wnt1 up vs E15.5 * Sox10
10563967		---	7.37823e-010	2.79847	E15.5 * Wnt1 up vs E15.5 * Sox10
10563971		---	7.37823e-010	2.79847	E15.5 * Wnt1 up vs E15.5 * Sox10
10564043		---	7.37823e-010	2.79847	E15.5 * Wnt1 up vs E15.5 * Sox10
10418092	A830039N20Rik	BC038501	2.76967e-006	2.78973	E15.5 * Wnt1 up vs E15.5 * Sox10
10492174	Tm4sf4	NM_145539	4.72393e-006	2.78416	E15.5 * Wnt1 up vs E15.5 * Sox10
10564089		---	2.19883e-007	2.76268	E15.5 * Wnt1 up vs E15.5 * Sox10
10592420	AW551984	NM_178737	1.61609e-010	2.7488	E15.5 * Wnt1 up vs E15.5 * Sox10
10418921	Sncg	NM_011430	3.5025e-008	2.74419	E15.5 * Wnt1 up vs E15.5 * Sox10
10564135		---	4.15653e-010	2.73965	E15.5 * Wnt1 up vs E15.5 * Sox10
10564027		---	4.19105e-007	2.73125	E15.5 * Wnt1 up vs E15.5 * Sox10
10564077		---	4.19105e-007	2.73125	E15.5 * Wnt1 up vs E15.5 * Sox10
10564079		---	4.19105e-007	2.73125	E15.5 * Wnt1 up vs E15.5 * Sox10
10564085		---	4.19105e-007	2.73125	E15.5 * Wnt1 up vs E15.5 * Sox10
10564087		---	4.19105e-007	2.73125	E15.5 * Wnt1 up vs E15.5 * Sox10
10564091		---	4.19105e-007	2.73125	E15.5 * Wnt1 up vs E15.5 * Sox10
10564093		---	4.19105e-007	2.73125	E15.5 * Wnt1 up vs E15.5 * Sox10
10564095		---	4.19105e-007	2.73125	E15.5 * Wnt1 up vs E15.5 * Sox10
10564097		---	4.19105e-007	2.73125	E15.5 * Wnt1 up vs E15.5 * Sox10
10564099		---	4.19105e-007	2.73125	E15.5 * Wnt1 up vs E15.5 * Sox10
10564101		---	4.19105e-007	2.73125	E15.5 * Wnt1 up vs E15.5 * Sox10
10564103		---	4.19105e-007	2.73125	E15.5 * Wnt1 up vs E15.5 * Sox10
10564105		---	4.19105e-007	2.73125	E15.5 * Wnt1 up vs E15.5 * Sox10
10564107		---	4.19105e-007	2.73125	E15.5 * Wnt1 up vs E15.5 * Sox10
10564113		---	4.19105e-007	2.73125	E15.5 * Wnt1 up vs E15.5 * Sox10
10564115		---	4.19105e-007	2.73125	E15.5 * Wnt1 up vs E15.5 * Sox10
10564119		---	4.19105e-007	2.73125	E15.5 * Wnt1 up vs E15.5 * Sox10
10564121		---	4.19105e-007	2.73125	E15.5 * Wnt1 up vs E15.5 * Sox10
10564123		---	4.19105e-007	2.73125	E15.5 * Wnt1 up vs E15.5 * Sox10
10564125		---	4.19105e-007	2.73125	E15.5 * Wnt1 up vs E15.5 * Sox10
10564127		---	4.19105e-007	2.73125	E15.5 * Wnt1 up vs E15.5 * Sox10
10564129		---	4.19105e-007	2.73125	E15.5 * Wnt1 up vs E15.5 * Sox10
10564139		---	4.19105e-007	2.73125	E15.5 * Wnt1 up vs E15.5 * Sox10
10357300	Dpp10	NM_199021	1.73674e-013	2.72977	E15.5 * Wnt1 up vs E15.5 * Sox10
10472396	Scn2a1	NM_001099298	8.2527e-007	2.72495	E15.5 * Wnt1 up vs E15.5 * Sox10
10564143		---	7.50468e-010	2.71734	E15.5 * Wnt1 up vs E15.5 * Sox10
10564147		---	7.50468e-010	2.71734	E15.5 * Wnt1 up vs E15.5 * Sox10
10564013	Snord115	AF357427	8.67839e-009	2.71053	E15.5 * Wnt1 up vs E15.5 * Sox10
10564017	Snord115	AF357427	8.67839e-009	2.71053	E15.5 * Wnt1 up vs E15.5 * Sox10
10398366		---	0.000240372	2.69666	E15.5 * Wnt1 up vs E15.5 * Sox10
10472364	Scn2a1	NM_001099298	2.55629e-005	2.68912	E15.5 * Wnt1 up vs E15.5 * Sox10
10439362	Stxbp5l	NM_172440	1.76532e-008	2.68618	E15.5 * Wnt1 up vs E15.5 * Sox10
10350951	Tnr	NM_022312	9.97919e-009	2.66436	E15.5 * Wnt1 up vs E15.5 * Sox10
10564069		---	1.63403e-010	2.66267	E15.5 * Wnt1 up vs E15.5 * Sox10
10564073		---	1.63403e-010	2.66267	E15.5 * Wnt1 up vs E15.5 * Sox10
10584549	Scn3b	NM_178227	1.30305e-008	2.6577	E15.5 * Wnt1 up vs E15.5 * Sox10
10564023		---	2.22742e-006	2.63599	E15.5 * Wnt1 up vs E15.5 * Sox10
10511363	Penk	NM_001002927	6.97445e-007	2.61118	E15.5 * Wnt1 up vs E15.5 * Sox10
10366144	Mgat4c	NM_026243	6.5403e-008	2.60395	E15.5 * Wnt1 up vs E15.5 * Sox10
10564019		---	6.83622e-008	2.58731	E15.5 * Wnt1 up vs E15.5 * Sox10
10398380		---	0.000122699	2.57579	E15.5 * Wnt1 up vs E15.5 * Sox10
10374356	Vstm2a	NM_145967	5.65796e-007	2.57318	E15.5 * Wnt1 up vs E15.5 * Sox10
10436636	Ncam2	NM_001113208	1.04383e-006	2.55894	E15.5 * Wnt1 up vs E15.5 * Sox10
10355836	Resp18	NM_009049	6.3801e-008	2.54835	E15.5 * Wnt1 up vs E15.5 * Sox10
10402604		---	4.04657e-007	2.54196	E15.5 * Wnt1 up vs E15.5 * Sox10
10564033		---	2.63014e-006	2.53941	E15.5 * Wnt1 up vs E15.5 * Sox10
10423274	Cdh18	NM_001081299	3.00699e-006	2.53435	E15.5 * Wnt1 up vs E15.5 * Sox10
10418835	Slc18a3	NM_021712	4.24983e-006	2.53029	E15.5 * Wnt1 up vs E15.5 * Sox10
10440465		---	0.000812515	2.52201	E15.5 * Wnt1 up vs E15.5 * Sox10
10548043	Kcna5	NM_145983	3.19264e-006	2.51117	E15.5 * Wnt1 up vs E15.5 * Sox10
10599187	Zcchc12	NM_028325	3.75385e-008	2.50075	E15.5 * Wnt1 up vs E15.5 * Sox10
10355960	Scg2	NM_009129	3.06645e-006	2.45794	E15.5 * Wnt1 up vs E15.5 * Sox10
10472374	Scn9a	BC172147	1.79538e-005	2.4546	E15.5 * Wnt1 up vs E15.5 * Sox10
10444135	AA388235	NM_001013793	7.72618e-005	2.42593	E15.5 * Wnt1 up vs E15.5 * Sox10

10564005		---	2.15425e-005	2.41385	E15.5 * Wnt1 up vs E15.5 * Sox10
10456353	Grp	NM_175012	1.31814e-006	2.4095	E15.5 * Wnt1 up vs E15.5 * Sox10
10512332	4930466F19Rik	ENSMUST00000	0.000242649	2.40631	E15.5 * Wnt1 up vs E15.5 * Sox10
10512354	4930466F19Rik	ENSMUST00000	0.000242649	2.40631	E15.5 * Wnt1 up vs E15.5 * Sox10
10428522	Csmd3	NM_001081391	6.30128e-006	2.40529	E15.5 * Wnt1 up vs E15.5 * Sox10
10519770	Pclo	NM_011995	3.80751e-008	2.39399	E15.5 * Wnt1 up vs E15.5 * Sox10
10501555	Amy1	NM_007446	8.80045e-005	2.38856	E15.5 * Wnt1 up vs E15.5 * Sox10
10553450	Nell1	NM_001037906	3.99873e-007	2.37879	E15.5 * Wnt1 up vs E15.5 * Sox10
10588283	Rab6b	NM_173781	3.34612e-006	2.3608	E15.5 * Wnt1 up vs E15.5 * Sox10
10360053	Pcp4l1	NM_025557	2.02322e-006	2.3591	E15.5 * Wnt1 up vs E15.5 * Sox10
10504123	4933409K07Rik	BC059060	5.31102e-006	2.35184	E15.5 * Wnt1 up vs E15.5 * Sox10
10504125	4933409K07Rik	BC059060	5.31102e-006	2.35184	E15.5 * Wnt1 up vs E15.5 * Sox10
10563931		---	2.70568e-006	2.33653	E15.5 * Wnt1 up vs E15.5 * Sox10
10440621	Grik1	NM_146072	4.57241e-008	2.32906	E15.5 * Wnt1 up vs E15.5 * Sox10
10563955	100040985	XM_001475615	1.25908e-005	2.32874	E15.5 * Wnt1 up vs E15.5 * Sox10
10392484	Abca8b	NM_013851	7.2824e-006	2.32343	E15.5 * Wnt1 up vs E15.5 * Sox10
10344879	A830018L16Rik	NM_177173	6.11398e-009	2.32277	E15.5 * Wnt1 up vs E15.5 * Sox10
10411274	Sv2c	NM_029210	1.32688e-008	2.32065	E15.5 * Wnt1 up vs E15.5 * Sox10
10451838	Slc5a7	NM_022025	9.15558e-007	2.31385	E15.5 * Wnt1 up vs E15.5 * Sox10
10436658	7120432I05Rik	AK148667	0.000673447	2.30801	E15.5 * Wnt1 up vs E15.5 * Sox10
10560919	Atp1a3	NM_144921	7.21139e-006	2.29656	E15.5 * Wnt1 up vs E15.5 * Sox10
10564137		---	3.12505e-006	2.29113	E15.5 * Wnt1 up vs E15.5 * Sox10
10508614	Fabp3	NM_010174	6.01221e-008	2.28276	E15.5 * Wnt1 up vs E15.5 * Sox10
10408268	Scgn	NM_145399	0.000411338	2.26944	E15.5 * Wnt1 up vs E15.5 * Sox10
10483228	Scn3a	NM_018732	1.90535e-007	2.26666	E15.5 * Wnt1 up vs E15.5 * Sox10
10563858	Gabrg3	NM_008074	8.93813e-007	2.26284	E15.5 * Wnt1 up vs E15.5 * Sox10
10476594	Macrod2	NM_001013802	0.00130385	2.25781	E15.5 * Wnt1 up vs E15.5 * Sox10
10547641	Slc2a3	NM_011401	2.62086e-008	2.25717	E15.5 * Wnt1 up vs E15.5 * Sox10
10416090	Stmn4	NM_019675	2.09606e-006	2.25058	E15.5 * Wnt1 up vs E15.5 * Sox10
10599841	C230004F18Rik	ENSMUST00000(	4.97162e-006	2.24761	E15.5 * Wnt1 up vs E15.5 * Sox10
10472368	Scn2a1	NM_001099298	1.15754e-005	2.24563	E15.5 * Wnt1 up vs E15.5 * Sox10
10504121	4933409K07Rik	BC059060	4.46936e-006	2.23025	E15.5 * Wnt1 up vs E15.5 * Sox10
10594048	Islr2	NM_177193	2.3053e-007	2.2236	E15.5 * Wnt1 up vs E15.5 * Sox10
10531304	Cdkl2	NM_016912	9.10872e-009	2.21724	E15.5 * Wnt1 up vs E15.5 * Sox10
10455080	Pcdhb9	NM_053134	7.60111e-005	2.21259	E15.5 * Wnt1 up vs E15.5 * Sox10
10440216	Epha6	NM_007938	3.15793e-007	2.20618	E15.5 * Wnt1 up vs E15.5 * Sox10
10366196	Ppfia2	NM_177373	8.63521e-009	2.206	E15.5 * Wnt1 up vs E15.5 * Sox10
10519693	Sema3d	NM_028882	2.52813e-006	2.20357	E15.5 * Wnt1 up vs E15.5 * Sox10
10504148	4933409K07Rik	BC059060	5.85751e-009	2.19848	E15.5 * Wnt1 up vs E15.5 * Sox10
10457929	Rit2	NM_009065	3.24695e-007	2.19231	E15.5 * Wnt1 up vs E15.5 * Sox10
10504178	4933409K07Rik	BC059060	1.88352e-009	2.18318	E15.5 * Wnt1 up vs E15.5 * Sox10
10355259	Myl1	NM_021285	1.56282e-005	2.17242	E15.5 * Wnt1 up vs E15.5 * Sox10
10453231	Slc8a1	NM_011406	2.29704e-007	2.15798	E15.5 * Wnt1 up vs E15.5 * Sox10
10492536		---	1.98177e-005	2.14453	E15.5 * Wnt1 up vs E15.5 * Sox10
10555303	Pgm2l1	NM_027629	2.02424e-006	2.14192	E15.5 * Wnt1 up vs E15.5 * Sox10
10462113	Apba1	NM_177034	1.35515e-007	2.14116	E15.5 * Wnt1 up vs E15.5 * Sox10
10398358		---	0.000744843	2.13949	E15.5 * Wnt1 up vs E15.5 * Sox10
10428517	Csmd3	NM_001081391	8.22688e-008	2.13859	E15.5 * Wnt1 up vs E15.5 * Sox10
10540233	Fam19a1	NM_182808	3.49243e-007	2.13762	E15.5 * Wnt1 up vs E15.5 * Sox10
10483215	Scn3a	NM_018732	1.00982e-006	2.1321	E15.5 * Wnt1 up vs E15.5 * Sox10
10455942	A730017C20Rik	NM_173759	1.14479e-006	2.12166	E15.5 * Wnt1 up vs E15.5 * Sox10
10357472	Cxcr4	NM_009911	2.5924e-006	2.11983	E15.5 * Wnt1 up vs E15.5 * Sox10
10504169	4933409K07Rik	BC072647	9.35004e-009	2.11206	E15.5 * Wnt1 up vs E15.5 * Sox10
10431625	Syt10	NM_018803	2.11072e-006	2.1044	E15.5 * Wnt1 up vs E15.5 * Sox10
10502881	St6galnac5	NM_012028	1.62504e-008	2.10237	E15.5 * Wnt1 up vs E15.5 * Sox10
10398364		---	0.000218872	2.08774	E15.5 * Wnt1 up vs E15.5 * Sox10
10371502	Fabp3	NM_010174	9.91278e-009	2.07861	E15.5 * Wnt1 up vs E15.5 * Sox10
10472382	Scn2a1	NM_001099298	5.57552e-006	2.07708	E15.5 * Wnt1 up vs E15.5 * Sox10
10408359	Nrsn1	NM_009513	2.8083e-006	2.07133	E15.5 * Wnt1 up vs E15.5 * Sox10
10371321	Slc41a2	NM_177388	1.92631e-006	2.06782	E15.5 * Wnt1 up vs E15.5 * Sox10
10604175	Fam70a	NM_172930	8.20765e-005	2.06565	E15.5 * Wnt1 up vs E15.5 * Sox10
10418053	Kcnma1	NM_010610	4.05524e-008	2.06406	E15.5 * Wnt1 up vs E15.5 * Sox10
10359689	Atp1b1	NM_009721	1.39407e-005	2.05976	E15.5 * Wnt1 up vs E15.5 * Sox10
10537458	EG434008	NM_001004182	1.50519e-005	2.05703	E15.5 * Wnt1 up vs E15.5 * Sox10
10358999	Xpr1	NM_011273	1.26413e-010	2.04577	E15.5 * Wnt1 up vs E15.5 * Sox10
10512327	4933409K07Rik	BC072647	2.53431e-008	2.04318	E15.5 * Wnt1 up vs E15.5 * Sox10

10347188	A830006F12Rik	NM_177164	2.69021e-006	2.04261	E15.5 * Wnt1 up vs E15.5 * Sox10
10407481	Pfkp	NM_019703	1.7883e-007	2.04152	E15.5 * Wnt1 up vs E15.5 * Sox10
10593245	Htr3b	NM_020274	3.64232e-006	2.03792	E15.5 * Wnt1 up vs E15.5 * Sox10
10367582	Vip	NM_011702	7.83546e-006	2.03725	E15.5 * Wnt1 up vs E15.5 * Sox10
10600604	Dmd	NM_007868	6.57316e-007	2.02995	E15.5 * Wnt1 up vs E15.5 * Sox10
10399308	Fkbp1b	NM_016863	0.000134102	2.02923	E15.5 * Wnt1 up vs E15.5 * Sox10
10437205	Pcp4	NM_008791	7.02869e-005	2.02829	E15.5 * Wnt1 up vs E15.5 * Sox10
10411527	Cartpt	NM_013732	0.00065362	2.02662	E15.5 * Wnt1 up vs E15.5 * Sox10
10474814	Disp2	NM_170593	3.65551e-006	2.02577	E15.5 * Wnt1 up vs E15.5 * Sox10
10497051	Negr1	NM_001039094	4.31321e-006	2.02322	E15.5 * Wnt1 up vs E15.5 * Sox10
10545086	Snca	NM_001042451	1.64927e-006	2.01821	E15.5 * Wnt1 up vs E15.5 * Sox10
10376245	Gria1	NM_001113325	2.4343e-009	2.01647	E15.5 * Wnt1 up vs E15.5 * Sox10
10460123	9330132A10Rik	BC098197	2.97383e-006	2.00727	E15.5 * Wnt1 up vs E15.5 * Sox10
10349208	Cntnap5a	NM_001077425	5.73643e-007	2.00418	E15.5 * Wnt1 up vs E15.5 * Sox10
10591123	Fat3	NM_001080814	0.000908619	2.0027	E15.5 * Wnt1 up vs E15.5 * Sox10
10526559	Ache	NM_009599	3.54082e-005	2.00011	E15.5 * Wnt1 up vs E15.5 * Sox10
10398368		---	4.09553e-007	1.99847	E15.5 * Wnt1 up vs E15.5 * Sox10
10428513	Csmd3	NM_001081391	7.68513e-005	1.99575	E15.5 * Wnt1 up vs E15.5 * Sox10
10406782	Fam169a	NM_001100458	5.85709e-005	1.99252	E15.5 * Wnt1 up vs E15.5 * Sox10
10344935	Kcnb2	NM_001098528	5.96381e-006	1.985	E15.5 * Wnt1 up vs E15.5 * Sox10
10506274	Dnajc6	NM_198412	5.7449e-009	1.98461	E15.5 * Wnt1 up vs E15.5 * Sox10
10421932	Pcdh9	NM_001081377	1.48526e-005	1.98293	E15.5 * Wnt1 up vs E15.5 * Sox10
10395702	Akap6	NM_198111	4.45002e-009	1.97636	E15.5 * Wnt1 up vs E15.5 * Sox10
10397482	Tmem63c	NM_172583	1.24226e-007	1.97577	E15.5 * Wnt1 up vs E15.5 * Sox10
10456237	St8sia3	NM_009182	3.53307e-006	1.97168	E15.5 * Wnt1 up vs E15.5 * Sox10
10360349	Cadm3	NM_053199	3.01165e-007	1.96798	E15.5 * Wnt1 up vs E15.5 * Sox10
10520318	Dpp6	NM_010075	3.12798e-010	1.96643	E15.5 * Wnt1 up vs E15.5 * Sox10
10363786	Ank3	NM_146005	1.2405e-007	1.96523	E15.5 * Wnt1 up vs E15.5 * Sox10
10474373	Kcna4	NM_021275	6.17083e-005	1.95222	E15.5 * Wnt1 up vs E15.5 * Sox10
10563915		---	2.53034e-007	1.95204	E15.5 * Wnt1 up vs E15.5 * Sox10
10563929		---	2.53034e-007	1.95204	E15.5 * Wnt1 up vs E15.5 * Sox10
10572070	Npy1r	NM_010934	2.1252e-006	1.95032	E15.5 * Wnt1 up vs E15.5 * Sox10
10607156	Dcx	NM_001110222	2.86913e-006	1.94588	E15.5 * Wnt1 up vs E15.5 * Sox10
10537300	1700111E14Rik	ENSMUST00000(	0.000395956	1.94284	E15.5 * Wnt1 up vs E15.5 * Sox10
10546736	Cntn3	NM_008779	7.8439e-006	1.94265	E15.5 * Wnt1 up vs E15.5 * Sox10
10363921	Pcdh15	NM_023115	3.30699e-006	1.93793	E15.5 * Wnt1 up vs E15.5 * Sox10
10398378		---	0.00482684	1.93791	E15.5 * Wnt1 up vs E15.5 * Sox10
10606554	Nap1l3	NM_138742	1.2567e-006	1.93592	E15.5 * Wnt1 up vs E15.5 * Sox10
10472136	Galnt13	NM_173030	2.09618e-006	1.93192	E15.5 * Wnt1 up vs E15.5 * Sox10
10395428	Dgkb	NM_178681	4.74515e-006	1.9311	E15.5 * Wnt1 up vs E15.5 * Sox10
10530870	Epha5	NM_007937	7.01648e-007	1.9288	E15.5 * Wnt1 up vs E15.5 * Sox10
10584561	9030425E11Rik	NM_133733	0.000606879	1.92721	E15.5 * Wnt1 up vs E15.5 * Sox10
10607484	Ptchd1	NM_001093750	0.000845764	1.92189	E15.5 * Wnt1 up vs E15.5 * Sox10
10472366	Scn2a1	NM_001099298	0.00022607	1.91843	E15.5 * Wnt1 up vs E15.5 * Sox10
10585823	LOC665268	XM_001479935	0.000228257	1.91008	E15.5 * Wnt1 up vs E15.5 * Sox10
10366163	Slc6a15	NM_175328	2.61979e-005	1.90741	E15.5 * Wnt1 up vs E15.5 * Sox10
10360664	ENSMUSG0000C	ENSMUST00000(	2.26689e-006	1.90651	E15.5 * Wnt1 up vs E15.5 * Sox10
10504139	4933409K07Rik	BC059060	7.15181e-008	1.90566	E15.5 * Wnt1 up vs E15.5 * Sox10
10428453	Csmd3	NM_001081391	4.94895e-009	1.90158	E15.5 * Wnt1 up vs E15.5 * Sox10
10456363	Gnal	NM_010307	0.000124904	1.9006	E15.5 * Wnt1 up vs E15.5 * Sox10
10493995	S100a10	NM_009112	5.16161e-005	1.90044	E15.5 * Wnt1 up vs E15.5 * Sox10
10457942	Syt4	NM_009308	7.01997e-006	1.9003	E15.5 * Wnt1 up vs E15.5 * Sox10
10504137	4933409K07Rik	BC072647	6.17227e-006	1.89871	E15.5 * Wnt1 up vs E15.5 * Sox10
10504201	4933409K07Rik	BC072647	6.17227e-006	1.89871	E15.5 * Wnt1 up vs E15.5 * Sox10
10512350	4933409K07Rik	BC072647	6.17227e-006	1.89871	E15.5 * Wnt1 up vs E15.5 * Sox10
10512352	4933409K07Rik	BC072647	6.17227e-006	1.89871	E15.5 * Wnt1 up vs E15.5 * Sox10
10438639	Dgkg	NM_138650	2.29957e-007	1.89624	E15.5 * Wnt1 up vs E15.5 * Sox10
10529937	Kcnip4	NM_030265	1.62934e-006	1.89465	E15.5 * Wnt1 up vs E15.5 * Sox10
10579550	Unc13a	BC058348	8.94204e-007	1.89345	E15.5 * Wnt1 up vs E15.5 * Sox10
10398459	Ppp2r5c	NM_001135001	0.000189043	1.89091	E15.5 * Wnt1 up vs E15.5 * Sox10
10563939		---	0.000695283	1.89066	E15.5 * Wnt1 up vs E15.5 * Sox10
10494372	Sv2a	NM_022030	0.000233071	1.8898	E15.5 * Wnt1 up vs E15.5 * Sox10
10492136	Dclk1	NM_019978	3.15616e-008	1.88877	E15.5 * Wnt1 up vs E15.5 * Sox10
10369752	Lrrtm3	NM_178678	8.64473e-006	1.88633	E15.5 * Wnt1 up vs E15.5 * Sox10
10529953	ENSMUSG0000C	AK036806	0.00491691	1.88589	E15.5 * Wnt1 up vs E15.5 * Sox10
10475324	Ckmt1	NM_009897	0.000144645	1.88414	E15.5 * Wnt1 up vs E15.5 * Sox10

10406823		---	7.75089e-005	1.88384	E15.5 * Wnt1 up vs E15.5 * Sox10
10583163	Trpc6	NM_013838	1.16499e-007	1.87901	E15.5 * Wnt1 up vs E15.5 * Sox10
10504172	4933409K07Rik	BC059060	4.76301e-008	1.87701	E15.5 * Wnt1 up vs E15.5 * Sox10
10400302		---	0.0036074	1.87648	E15.5 * Wnt1 up vs E15.5 * Sox10
10605113	L1cam	NM_008478	5.45543e-007	1.87255	E15.5 * Wnt1 up vs E15.5 * Sox10
10572024	Spock3	NM_023689	8.417e-006	1.87189	E15.5 * Wnt1 up vs E15.5 * Sox10
10483131	Kcnh7	NM_133207	5.54808e-007	1.87096	E15.5 * Wnt1 up vs E15.5 * Sox10
10569278	5530400B01Rik	ENSMUST00000	0.000115672	1.87074	E15.5 * Wnt1 up vs E15.5 * Sox10
10385283	Gabrg2	NM_008073	1.79434e-005	1.87041	E15.5 * Wnt1 up vs E15.5 * Sox10
10532630	Adrbk2	NM_177078	5.72154e-005	1.8673	E15.5 * Wnt1 up vs E15.5 * Sox10
10449920	Zfp811	NM_183177	2.11049e-005	1.86547	E15.5 * Wnt1 up vs E15.5 * Sox10
10398354		---	0.00237238	1.86155	E15.5 * Wnt1 up vs E15.5 * Sox10
10485580	Cstf3	NM_145529	7.32685e-005	1.86068	E15.5 * Wnt1 up vs E15.5 * Sox10
10463737	Ina	AK144917	0.000149102	1.85899	E15.5 * Wnt1 up vs E15.5 * Sox10
10502451	Bmpr1b	NM_007560	1.33718e-005	1.85843	E15.5 * Wnt1 up vs E15.5 * Sox10
10564667	Ntrk3	NM_008746	4.40731e-005	1.85459	E15.5 * Wnt1 up vs E15.5 * Sox10
10602198	Pak3	NM_008778	2.47306e-006	1.85217	E15.5 * Wnt1 up vs E15.5 * Sox10
10356154	Sphkap	NM_172430	7.3521e-005	1.8517	E15.5 * Wnt1 up vs E15.5 * Sox10
10347734	Sgpp2	NM_001004173	5.8484e-008	1.85007	E15.5 * Wnt1 up vs E15.5 * Sox10
10485309	E530001K10Rik	ENSMUST00000	0.00109321	1.84496	E15.5 * Wnt1 up vs E15.5 * Sox10
10366229	Lin7a	NM_001039354	4.35451e-006	1.84338	E15.5 * Wnt1 up vs E15.5 * Sox10
10372342	Nav3	NM_001081035	7.04252e-008	1.84306	E15.5 * Wnt1 up vs E15.5 * Sox10
10354003	Mgat4a	NM_173870	0.000494421	1.83911	E15.5 * Wnt1 up vs E15.5 * Sox10
10492720	Mtap9	NM_001081230	6.75334e-005	1.83779	E15.5 * Wnt1 up vs E15.5 * Sox10
10606789	Tceal6	NM_025355	0.00100748	1.83742	E15.5 * Wnt1 up vs E15.5 * Sox10
10573172	Clgn	NM_009904	2.24489e-006	1.83523	E15.5 * Wnt1 up vs E15.5 * Sox10
10492628	Serpini1	NM_009250	1.34402e-005	1.83475	E15.5 * Wnt1 up vs E15.5 * Sox10
10353729	4930444G20Rik	NM_053264	0.000921183	1.83391	E15.5 * Wnt1 up vs E15.5 * Sox10
10395971		---	0.000547667	1.83294	E15.5 * Wnt1 up vs E15.5 * Sox10
10556067	Syt9	NM_021889	4.13947e-007	1.8324	E15.5 * Wnt1 up vs E15.5 * Sox10
10463732	Ina	NM_146100	3.72683e-008	1.83083	E15.5 * Wnt1 up vs E15.5 * Sox10
10426656	Prph	NM_013639	1.13802e-006	1.82767	E15.5 * Wnt1 up vs E15.5 * Sox10
10351056	Ankrd45	BC049713	9.6995e-005	1.8218	E15.5 * Wnt1 up vs E15.5 * Sox10
10498965	Npy2r	NM_008731	0.00188441	1.81721	E15.5 * Wnt1 up vs E15.5 * Sox10
10353731	4930444G20Rik	NM_053264	0.000604064	1.81712	E15.5 * Wnt1 up vs E15.5 * Sox10
10501971	Ank2	NM_178655	4.02894e-008	1.81547	E15.5 * Wnt1 up vs E15.5 * Sox10
10396270	Dact1	NM_021532	2.93706e-005	1.81476	E15.5 * Wnt1 up vs E15.5 * Sox10
10395005	Kidins220	NM_001081378	4.77013e-010	1.81356	E15.5 * Wnt1 up vs E15.5 * Sox10
10447294	Prkce	NM_011104	7.57555e-008	1.81001	E15.5 * Wnt1 up vs E15.5 * Sox10
10426397	Cntn1	NM_007727	3.46377e-006	1.80421	E15.5 * Wnt1 up vs E15.5 * Sox10
10595496	Snap91	NM_013669	2.59856e-005	1.80382	E15.5 * Wnt1 up vs E15.5 * Sox10
10606864	Tceal5	NM_177919	3.05942e-007	1.80229	E15.5 * Wnt1 up vs E15.5 * Sox10
10575693	Vat1l	NM_173016	5.76565e-006	1.79908	E15.5 * Wnt1 up vs E15.5 * Sox10
10421924	Pcdh9	NM_001081377	2.8612e-006	1.79902	E15.5 * Wnt1 up vs E15.5 * Sox10
10422585	Fgf14	NM_207667	2.53817e-005	1.79902	E15.5 * Wnt1 up vs E15.5 * Sox10
10497012	BC007180	BC138236	3.6768e-008	1.79545	E15.5 * Wnt1 up vs E15.5 * Sox10
10533401	Cux2	ENSMUST00000	7.69021e-006	1.7885	E15.5 * Wnt1 up vs E15.5 * Sox10
10366391	Kcnc2	NM_001025581	5.91336e-006	1.78713	E15.5 * Wnt1 up vs E15.5 * Sox10
10592515	Ubash3b	NM_176860	1.25428e-006	1.78601	E15.5 * Wnt1 up vs E15.5 * Sox10
10564111		---	8.7987e-008	1.78572	E15.5 * Wnt1 up vs E15.5 * Sox10
10468691	Ablim1	NM_178688	9.72708e-008	1.7851	E15.5 * Wnt1 up vs E15.5 * Sox10
10373113	Kif5a	NM_008447	3.21219e-005	1.78167	E15.5 * Wnt1 up vs E15.5 * Sox10
10407841	Hecw1	NM_001081348	0.000320121	1.78085	E15.5 * Wnt1 up vs E15.5 * Sox10
10601903	Zcchc18	NM_001035510	4.58745e-005	1.77689	E15.5 * Wnt1 up vs E15.5 * Sox10
10472370	Scn2a1	NM_001099298	3.01431e-005	1.77591	E15.5 * Wnt1 up vs E15.5 * Sox10
10473058	Osbpl6	NM_145525	5.26027e-007	1.77571	E15.5 * Wnt1 up vs E15.5 * Sox10
10400607	Mdga2	AK163637	0.000188565	1.77031	E15.5 * Wnt1 up vs E15.5 * Sox10
10544875	Scrn1	NM_027268	0.000190756	1.76206	E15.5 * Wnt1 up vs E15.5 * Sox10
10498720	Zbbx	NM_172515	1.28198e-007	1.75967	E15.5 * Wnt1 up vs E15.5 * Sox10
10472376	Scn2a1	NM_001099298	7.80831e-006	1.75628	E15.5 * Wnt1 up vs E15.5 * Sox10
10587241	Hmgcll1	NM_173731	5.82465e-005	1.75199	E15.5 * Wnt1 up vs E15.5 * Sox10
10484371	Calcrl	NM_018782	9.06115e-005	1.75084	E15.5 * Wnt1 up vs E15.5 * Sox10
10505568	Frmd3	NM_172869	1.57323e-005	1.73989	E15.5 * Wnt1 up vs E15.5 * Sox10
10388465	Doc2b	NM_007873	1.86132e-006	1.73684	E15.5 * Wnt1 up vs E15.5 * Sox10
10523511	Prkg2	NM_008926	0.000663178	1.73537	E15.5 * Wnt1 up vs E15.5 * Sox10
10564209		---	0.000108295	1.73467	E15.5 * Wnt1 up vs E15.5 * Sox10

10564203		---	0.00242945	1.73463	E15.5 * Wnt1 up vs E15.5 * Sox10
10555197	Mtap6	NM_010837	2.65635e-007	1.73243	E15.5 * Wnt1 up vs E15.5 * Sox10
10545041	Nap1l5	NM_021432	2.04582e-005	1.73239	E15.5 * Wnt1 up vs E15.5 * Sox10
10421100	Nefm	NM_008691	8.81605e-009	1.73109	E15.5 * Wnt1 up vs E15.5 * Sox10
10423230	Cdh9	NM_009869	2.86651e-006	1.72796	E15.5 * Wnt1 up vs E15.5 * Sox10
10498707	Slitrk3	NM_198864	2.11164e-006	1.72704	E15.5 * Wnt1 up vs E15.5 * Sox10
10606174	Nap1l2	NM_008671	0.000361216	1.72561	E15.5 * Wnt1 up vs E15.5 * Sox10
10510725	Chd5	NM_001081376	8.95621e-008	1.72529	E15.5 * Wnt1 up vs E15.5 * Sox10
10400926	Rtn1	NM_153457	2.03552e-005	1.7247	E15.5 * Wnt1 up vs E15.5 * Sox10
10398326	Meg3	NR_003633	4.28947e-005	1.72434	E15.5 * Wnt1 up vs E15.5 * Sox10
10563945		---	1.97184e-006	1.72348	E15.5 * Wnt1 up vs E15.5 * Sox10
10480492	Cacna1b	NM_001042528	1.1217e-008	1.71825	E15.5 * Wnt1 up vs E15.5 * Sox10
10580247	Mast1	NM_019945	3.72732e-006	1.71707	E15.5 * Wnt1 up vs E15.5 * Sox10
10481772	Garnl3	NM_178888	7.71257e-007	1.71655	E15.5 * Wnt1 up vs E15.5 * Sox10
10381776	Mapt	NM_001038609	1.03649e-005	1.71374	E15.5 * Wnt1 up vs E15.5 * Sox10
10405343	Tspan17	NM_028841	3.05032e-006	1.71227	E15.5 * Wnt1 up vs E15.5 * Sox10
10490856	Ralyl	NM_178631	1.16502e-005	1.71193	E15.5 * Wnt1 up vs E15.5 * Sox10
10598493	Pcsk1n	NM_013892	4.20189e-007	1.70775	E15.5 * Wnt1 up vs E15.5 * Sox10
10439895	Alcam	NM_009655	1.93098e-005	1.70349	E15.5 * Wnt1 up vs E15.5 * Sox10
10485979	Gjd2	NM_010290	0.00453875	1.70056	E15.5 * Wnt1 up vs E15.5 * Sox10
10481857	Pbx3	NM_016768	4.36898e-008	1.69978	E15.5 * Wnt1 up vs E15.5 * Sox10
10490818	Stmn2	NM_025285	1.38283e-005	1.69892	E15.5 * Wnt1 up vs E15.5 * Sox10
10580990	Cdh8	NM_001039154	3.75457e-006	1.6982	E15.5 * Wnt1 up vs E15.5 * Sox10
10454632	Camk4	NM_009793	4.89963e-005	1.69719	E15.5 * Wnt1 up vs E15.5 * Sox10
10355024	Ica1l	NM_027407	1.81076e-006	1.69381	E15.5 * Wnt1 up vs E15.5 * Sox10
10483679	Gpr155	NM_001080707	4.3336e-006	1.69333	E15.5 * Wnt1 up vs E15.5 * Sox10
10420532	Atp8a2	NM_015803	1.04565e-007	1.69259	E15.5 * Wnt1 up vs E15.5 * Sox10
10368495	Rspo3	NM_028351	2.6952e-006	1.69195	E15.5 * Wnt1 up vs E15.5 * Sox10
10553773	Gabrb3	NM_008071	7.49176e-007	1.69161	E15.5 * Wnt1 up vs E15.5 * Sox10
10535577	Tmem130	NM_177735	2.96654e-006	1.69008	E15.5 * Wnt1 up vs E15.5 * Sox10
10493555	Kcnn3	NM_080466	9.69595e-007	1.68636	E15.5 * Wnt1 up vs E15.5 * Sox10
10399419	Tubb2b	NM_023716	8.99614e-006	1.68546	E15.5 * Wnt1 up vs E15.5 * Sox10
10563905		---	1.64489e-009	1.68472	E15.5 * Wnt1 up vs E15.5 * Sox10
10563907		---	1.64489e-009	1.68472	E15.5 * Wnt1 up vs E15.5 * Sox10
10551065	D930028M14Rik	ENSMUST00000(	4.23374e-006	1.68403	E15.5 * Wnt1 up vs E15.5 * Sox10
10600114	Pnma3	NM_153169	2.25998e-005	1.68216	E15.5 * Wnt1 up vs E15.5 * Sox10
10399208	Tmem196	ENSMUST00000(	0.0043903	1.68117	E15.5 * Wnt1 up vs E15.5 * Sox10
10561335	Prkcz	NM_008860	0.000509916	1.67922	E15.5 * Wnt1 up vs E15.5 * Sox10
10568109	Asphd1	NM_001039645	2.16453e-005	1.67556	E15.5 * Wnt1 up vs E15.5 * Sox10
10522208	Uchl1	NM_011670	0.000355637	1.67459	E15.5 * Wnt1 up vs E15.5 * Sox10
10520527	Dpysl5	NM_023047	1.17078e-005	1.67324	E15.5 * Wnt1 up vs E15.5 * Sox10
10488387	Napb	NM_019632	1.93161e-006	1.67181	E15.5 * Wnt1 up vs E15.5 * Sox10
10412466	Hmgcs1	NM_145942	7.26867e-007	1.67161	E15.5 * Wnt1 up vs E15.5 * Sox10
10592535	Sorl1	NM_011436	3.10561e-008	1.67081	E15.5 * Wnt1 up vs E15.5 * Sox10
10603814	Slc9a7	NM_177353	2.22778e-005	1.66806	E15.5 * Wnt1 up vs E15.5 * Sox10
10585484	Chrna5	NM_176844	7.4751e-005	1.66763	E15.5 * Wnt1 up vs E15.5 * Sox10
10396712	Fut8	NM_016893	6.04156e-006	1.66529	E15.5 * Wnt1 up vs E15.5 * Sox10
10398356		---	0.000161125	1.66248	E15.5 * Wnt1 up vs E15.5 * Sox10
10439016	4930444G20Rik	NM_053264	0.00370134	1.66118	E15.5 * Wnt1 up vs E15.5 * Sox10
10445078	Gabbr1	NM_019439	1.25364e-006	1.66094	E15.5 * Wnt1 up vs E15.5 * Sox10
10365682	Anks1b	NM_001128086	7.55034e-005	1.65739	E15.5 * Wnt1 up vs E15.5 * Sox10
10397912	9030205A07Rik	AB257853	1.0464e-006	1.65686	E15.5 * Wnt1 up vs E15.5 * Sox10
10424113	Mal2	NM_178920	0.00198324	1.65594	E15.5 * Wnt1 up vs E15.5 * Sox10
10461856	Gna14	NM_008137	0.000251687	1.65443	E15.5 * Wnt1 up vs E15.5 * Sox10
10484503	Lrrc55	NM_001033346	0.000779335	1.65306	E15.5 * Wnt1 up vs E15.5 * Sox10
10395074	Myt1l	NM_001093775	3.20827e-008	1.65223	E15.5 * Wnt1 up vs E15.5 * Sox10
10606069	4930444G20Rik	NM_053264	0.00360603	1.65127	E15.5 * Wnt1 up vs E15.5 * Sox10
10494007	Them4	NM_029431	6.85159e-005	1.64904	E15.5 * Wnt1 up vs E15.5 * Sox10
10590865	Cntn5	NM_001033359	2.00916e-006	1.64875	E15.5 * Wnt1 up vs E15.5 * Sox10
10408613	Tubb2b	NM_023716	0.000380098	1.64575	E15.5 * Wnt1 up vs E15.5 * Sox10
10360972	Kcnk2	NM_010607	5.36094e-006	1.64546	E15.5 * Wnt1 up vs E15.5 * Sox10
10591118	Fat3	NM_001080814	0.00401581	1.64404	E15.5 * Wnt1 up vs E15.5 * Sox10
10573979	Gnao1	NM_010308	5.72998e-007	1.64315	E15.5 * Wnt1 up vs E15.5 * Sox10
10553635	Nipa1	NM_153578	4.43235e-006	1.64312	E15.5 * Wnt1 up vs E15.5 * Sox10
10526614	Actl6b	NM_031404	1.49273e-005	1.64223	E15.5 * Wnt1 up vs E15.5 * Sox10
10490602	Eef1a2	NM_007906	5.81149e-007	1.64211	E15.5 * Wnt1 up vs E15.5 * Sox10

10603768	Efhc2	NM_028916	1.00349e-005	1.64097	E15.5 * Wnt1 up vs E15.5 * Sox10
10498647	B3galnt1	NM_020026	4.10511e-009	1.64074	E15.5 * Wnt1 up vs E15.5 * Sox10
10416175	Nefl	NM_010910	2.98244e-005	1.64029	E15.5 * Wnt1 up vs E15.5 * Sox10
10520304	Actr3b	NM_001004365	3.22906e-005	1.63999	E15.5 * Wnt1 up vs E15.5 * Sox10
10354141	Lonrf2	NM_001029878	0.000605618	1.63819	E15.5 * Wnt1 up vs E15.5 * Sox10
10506335	Pde4b	NM_019840	1.88362e-005	1.63599	E15.5 * Wnt1 up vs E15.5 * Sox10
10563975		---	2.58194e-008	1.63588	E15.5 * Wnt1 up vs E15.5 * Sox10
10563981		---	2.58194e-008	1.63588	E15.5 * Wnt1 up vs E15.5 * Sox10
10563987		---	2.58194e-008	1.63588	E15.5 * Wnt1 up vs E15.5 * Sox10
10564009		---	2.58194e-008	1.63588	E15.5 * Wnt1 up vs E15.5 * Sox10
10545538	Ctnna2	NM_009819	5.92654e-005	1.63486	E15.5 * Wnt1 up vs E15.5 * Sox10
10406423	Mblac2	NM_028372	0.000390604	1.6338	E15.5 * Wnt1 up vs E15.5 * Sox10
10490665	Stmn3	NM_009133	2.68928e-005	1.6326	E15.5 * Wnt1 up vs E15.5 * Sox10
10564053	100040985	XM_001475615	3.4264e-009	1.63187	E15.5 * Wnt1 up vs E15.5 * Sox10
10564055		---	3.4264e-009	1.63187	E15.5 * Wnt1 up vs E15.5 * Sox10
10362005	Ahi1	NM_026203	1.48428e-005	1.63173	E15.5 * Wnt1 up vs E15.5 * Sox10
10400126	Lrrn3	NM_010733	1.42169e-007	1.63024	E15.5 * Wnt1 up vs E15.5 * Sox10
10526564	Ufsp1	NM_027356	0.00012993	1.62997	E15.5 * Wnt1 up vs E15.5 * Sox10
10492006	Trpc4	NM_016984	7.78007e-007	1.62948	E15.5 * Wnt1 up vs E15.5 * Sox10
10413461	Erc2	NM_177814	6.14384e-005	1.62913	E15.5 * Wnt1 up vs E15.5 * Sox10
10518812	Camta1	NM_001081557	1.14341e-005	1.62436	E15.5 * Wnt1 up vs E15.5 * Sox10
10482030	Stom	NM_013515	0.00427246	1.62348	E15.5 * Wnt1 up vs E15.5 * Sox10
10568982	BC066028	NM_001001180	8.80736e-005	1.62029	E15.5 * Wnt1 up vs E15.5 * Sox10
10495623	ENSMUSG0000(	ENSMUST00000(	0.0044139	1.61984	E15.5 * Wnt1 up vs E15.5 * Sox10
10607658	Reps2	NM_178256	4.88493e-005	1.61732	E15.5 * Wnt1 up vs E15.5 * Sox10
10428511	Csmd3	NM_001081391	0.000384332	1.61515	E15.5 * Wnt1 up vs E15.5 * Sox10
10375019	Nsg2	NM_008741	2.81097e-005	1.61444	E15.5 * Wnt1 up vs E15.5 * Sox10
10441195	Dscam	NM_031174	4.90435e-005	1.61369	E15.5 * Wnt1 up vs E15.5 * Sox10
10578904	Cpe	NM_013494	5.33092e-008	1.61211	E15.5 * Wnt1 up vs E15.5 * Sox10
10476592	Macrod2	NM_001013802	0.00138626	1.61114	E15.5 * Wnt1 up vs E15.5 * Sox10
10445338	Enpp5	NM_032003	3.49511e-005	1.611	E15.5 * Wnt1 up vs E15.5 * Sox10
10599530	Rab33a	NM_011228	0.000228895	1.60683	E15.5 * Wnt1 up vs E15.5 * Sox10
10344973	Gdap1	NM_010267	0.00152372	1.6022	E15.5 * Wnt1 up vs E15.5 * Sox10
10501483	Ntng1	ENSMUST00000(	3.4889e-006	1.60175	E15.5 * Wnt1 up vs E15.5 * Sox10
10430282	Cacng2	NM_007583	6.69867e-005	1.59994	E15.5 * Wnt1 up vs E15.5 * Sox10
10355813	Ptprn	NM_008985	3.43733e-006	1.59887	E15.5 * Wnt1 up vs E15.5 * Sox10
10400609	Mdga2	NM_207010	3.92733e-005	1.59839	E15.5 * Wnt1 up vs E15.5 * Sox10
10602805	Mtap7d2	NM_001081124	1.82442e-005	1.59782	E15.5 * Wnt1 up vs E15.5 * Sox10
10445325	Rcan2	NM_030598	0.000702981	1.5978	E15.5 * Wnt1 up vs E15.5 * Sox10
10366712	Ppm1h	NM_001110218	2.00359e-006	1.5976	E15.5 * Wnt1 up vs E15.5 * Sox10
10441091	6530402D11Rik	AK078339	1.17246e-005	1.59654	E15.5 * Wnt1 up vs E15.5 * Sox10
10455108	Pcdhb16	NM_053141	0.00455055	1.59468	E15.5 * Wnt1 up vs E15.5 * Sox10
10368598	Rlbp1l2	NM_175448	0.000924914	1.59266	E15.5 * Wnt1 up vs E15.5 * Sox10
10604292	Odz1	NM_011855	0.000167345	1.59245	E15.5 * Wnt1 up vs E15.5 * Sox10
10353632	Bai3	NM_175642	7.07362e-006	1.5913	E15.5 * Wnt1 up vs E15.5 * Sox10
10540509	Grm7	NM_177328	1.49204e-005	1.5908	E15.5 * Wnt1 up vs E15.5 * Sox10
10505705	Sh3gl2	NM_019535	3.41341e-006	1.58897	E15.5 * Wnt1 up vs E15.5 * Sox10
10465820	Gng3	NM_010316	0.000376336	1.58687	E15.5 * Wnt1 up vs E15.5 * Sox10
10527732	Fry	ENSMUST00000(	5.54459e-007	1.58673	E15.5 * Wnt1 up vs E15.5 * Sox10
10345212	Khdrbs2	NM_133235	0.000217722	1.58593	E15.5 * Wnt1 up vs E15.5 * Sox10
10453233	Slc8a1	NM_011406	2.62272e-006	1.58487	E15.5 * Wnt1 up vs E15.5 * Sox10
10471994	Kif5c	NM_008449	4.21939e-005	1.58287	E15.5 * Wnt1 up vs E15.5 * Sox10
10468762	4930506M07Rik	NM_001114312	7.36806e-005	1.58152	E15.5 * Wnt1 up vs E15.5 * Sox10
10593668	Dmxl2	NM_172771	0.000189475	1.58041	E15.5 * Wnt1 up vs E15.5 * Sox10
10458767	Trim36	NM_178872	6.11704e-005	1.57731	E15.5 * Wnt1 up vs E15.5 * Sox10
10601874	Tceal3	NM_001029978	7.21579e-006	1.57658	E15.5 * Wnt1 up vs E15.5 * Sox10
10564157		---	0.000872957	1.57609	E15.5 * Wnt1 up vs E15.5 * Sox10
10496975	Slc44a5	NM_001081263	0.00169324	1.57383	E15.5 * Wnt1 up vs E15.5 * Sox10
10428376	Angpt1	NM_009640	0.00114727	1.5731	E15.5 * Wnt1 up vs E15.5 * Sox10
10433709	2900011O08Rik	NM_144518	1.9827e-005	1.57277	E15.5 * Wnt1 up vs E15.5 * Sox10
10376444	Hist3h2ba	NM_030082	0.000816147	1.56983	E15.5 * Wnt1 up vs E15.5 * Sox10
10376216	Slc36a1	NM_153139	0.000361645	1.56944	E15.5 * Wnt1 up vs E15.5 * Sox10
10429083	Kcnq3	NM_152923	1.05294e-007	1.56819	E15.5 * Wnt1 up vs E15.5 * Sox10
10452935	Heatr5b	BC019508	0.00065659	1.56793	E15.5 * Wnt1 up vs E15.5 * Sox10
10406934	Etv1	NM_007960	4.68414e-006	1.56792	E15.5 * Wnt1 up vs E15.5 * Sox10
10375360	Ebf1	NM_007897	0.00162478	1.56687	E15.5 * Wnt1 up vs E15.5 * Sox10

10368881	Armc2	NM_001034858	0.00367728	1.56432	E15.5 * Wnt1 up vs E15.5 * Sox10
10417124	B930095G15Rik	BC096543	8.78678e-008	1.56335	E15.5 * Wnt1 up vs E15.5 * Sox10
10393594	D11Bwg0517e	NM_001039167	0.000113074	1.56259	E15.5 * Wnt1 up vs E15.5 * Sox10
10469767	Nxph2	NM_008752	0.000463642	1.56119	E15.5 * Wnt1 up vs E15.5 * Sox10
10369835	Phyhipl	NM_178621	6.48935e-005	1.56079	E15.5 * Wnt1 up vs E15.5 * Sox10
10356712	Kif1a	NM_008440	1.16179e-006	1.56032	E15.5 * Wnt1 up vs E15.5 * Sox10
10443690	Glp1r	NM_021332	0.000747874	1.55935	E15.5 * Wnt1 up vs E15.5 * Sox10
10417787	Gng2	NM_010315	2.99683e-007	1.55928	E15.5 * Wnt1 up vs E15.5 * Sox10
10589800	Clasp2	NM_001114347	2.40545e-009	1.55888	E15.5 * Wnt1 up vs E15.5 * Sox10
10412066	Rab3c	NM_023852	0.000212421	1.55599	E15.5 * Wnt1 up vs E15.5 * Sox10
10448557	Abca3	NM_013855	0.000604558	1.55524	E15.5 * Wnt1 up vs E15.5 * Sox10
10347073	Unc80	FJ210934	3.79886e-006	1.55425	E15.5 * Wnt1 up vs E15.5 * Sox10
10471842		---	0.000912043	1.55358	E15.5 * Wnt1 up vs E15.5 * Sox10
10601343	Magee1	NM_053201	0.00157763	1.55333	E15.5 * Wnt1 up vs E15.5 * Sox10
10594800	B230380D07Rik	NM_172772	0.00467821	1.55173	E15.5 * Wnt1 up vs E15.5 * Sox10
10521972	Pcdh7	NM_018764	6.56205e-006	1.5513	E15.5 * Wnt1 up vs E15.5 * Sox10
10514779	Prkaa2	NM_178143	4.17391e-005	1.55043	E15.5 * Wnt1 up vs E15.5 * Sox10
10392601	Abca6	NM_147218	0.00020627	1.54852	E15.5 * Wnt1 up vs E15.5 * Sox10
10428388	Rspo2	NM_172815	0.000364435	1.54788	E15.5 * Wnt1 up vs E15.5 * Sox10
10347781	9430031J16Rik	BC082310	0.000188884	1.54755	E15.5 * Wnt1 up vs E15.5 * Sox10
10424082	Aard	NM_175503	0.000359673	1.54654	E15.5 * Wnt1 up vs E15.5 * Sox10
10462922	Plce1	NM_019588	4.48118e-008	1.54472	E15.5 * Wnt1 up vs E15.5 * Sox10
10588380	Cpne4	NM_028719	1.14585e-005	1.54433	E15.5 * Wnt1 up vs E15.5 * Sox10
10348451	Cxcr7	NM_007722	0.000201866	1.54362	E15.5 * Wnt1 up vs E15.5 * Sox10
10439651	Cd200	NM_010818	0.000192668	1.54263	E15.5 * Wnt1 up vs E15.5 * Sox10
10548030	Cd9	NM_007657	0.000348583	1.54195	E15.5 * Wnt1 up vs E15.5 * Sox10
10353460	Kcnq5	NM_023872	1.93624e-005	1.54136	E15.5 * Wnt1 up vs E15.5 * Sox10
10411519	Mtap1b	NM_008634	9.49036e-006	1.54101	E15.5 * Wnt1 up vs E15.5 * Sox10
10564211	Snrpn	NM_013670	1.6264e-008	1.54069	E15.5 * Wnt1 up vs E15.5 * Sox10
10476512	Snap25	NM_011428	3.38115e-005	1.54062	E15.5 * Wnt1 up vs E15.5 * Sox10
10519612	9330182L06Rik	NM_172706	0.000131606	1.54041	E15.5 * Wnt1 up vs E15.5 * Sox10
10425852	Parvb	NM_133167	0.00103877	1.5404	E15.5 * Wnt1 up vs E15.5 * Sox10
10423505	Cmbl	NM_181588	0.000405996	1.54018	E15.5 * Wnt1 up vs E15.5 * Sox10
10455128	Pcdhb20	NM_053145	0.00324949	1.54009	E15.5 * Wnt1 up vs E15.5 * Sox10
10395961	Lrfn5	NM_178714	7.25991e-005	1.53833	E15.5 * Wnt1 up vs E15.5 * Sox10
10377368	Rpl26	NM_009080	0.00231574	1.53703	E15.5 * Wnt1 up vs E15.5 * Sox10
10395805	1700047I17Rik1	BC049669	5.7338e-006	1.53436	E15.5 * Wnt1 up vs E15.5 * Sox10
10503902	Cnr1	NM_007726	0.000156593	1.53413	E15.5 * Wnt1 up vs E15.5 * Sox10
10455259	Arhgap26	NM_175164	1.25e-005	1.53296	E15.5 * Wnt1 up vs E15.5 * Sox10
10396125	Atl1	NM_178628	0.000561107	1.53268	E15.5 * Wnt1 up vs E15.5 * Sox10
10372988	Slc16a7	NM_011391	0.000294543	1.5296	E15.5 * Wnt1 up vs E15.5 * Sox10
10514352	Elavl2	NM_207685	0.000134223	1.5288	E15.5 * Wnt1 up vs E15.5 * Sox10
10573348	Cacna1a	NM_007578	4.20516e-006	1.52802	E15.5 * Wnt1 up vs E15.5 * Sox10
10474096	Lrrc4c	NM_178725	1.55357e-005	1.52797	E15.5 * Wnt1 up vs E15.5 * Sox10
10428407	Tmem74	NM_175502	0.00015022	1.52635	E15.5 * Wnt1 up vs E15.5 * Sox10
10373542	Dgka	NM_016811	2.63127e-006	1.52596	E15.5 * Wnt1 up vs E15.5 * Sox10
10442069	Lix1	NM_025681	0.00389538	1.525	E15.5 * Wnt1 up vs E15.5 * Sox10
10583195	ENSMUSG0000C	ENSMUST00000	0.000887104	1.52379	E15.5 * Wnt1 up vs E15.5 * Sox10
10488617	Nrsn2	NM_001009948	0.000370055	1.52245	E15.5 * Wnt1 up vs E15.5 * Sox10
10563909		---	1.14834e-007	1.52234	E15.5 * Wnt1 up vs E15.5 * Sox10
10602704	Klf8	NM_173780	3.54295e-007	1.51974	E15.5 * Wnt1 up vs E15.5 * Sox10
10350438	Kcnt2	NM_001081027	0.00407409	1.51953	E15.5 * Wnt1 up vs E15.5 * Sox10
10601834	Gprasp1	NM_026081	0.000538682	1.51867	E15.5 * Wnt1 up vs E15.5 * Sox10
10598359	Syp	NM_009305	2.69062e-005	1.51638	E15.5 * Wnt1 up vs E15.5 * Sox10
10483624	Dlx1as	NR_002854	0.000423953	1.51615	E15.5 * Wnt1 up vs E15.5 * Sox10
10490794	Pkia	NM_008862	0.000226032	1.51589	E15.5 * Wnt1 up vs E15.5 * Sox10
10389617	Ppm1e	NM_177167	3.73624e-005	1.51394	E15.5 * Wnt1 up vs E15.5 * Sox10
10504375	Npr2	NM_173788	0.000786124	1.51282	E15.5 * Wnt1 up vs E15.5 * Sox10
10362372	9330159F19Rik	BC138282	0.00101346	1.5128	E15.5 * Wnt1 up vs E15.5 * Sox10
10604347	Smarca1	NM_053123	6.18054e-005	1.51231	E15.5 * Wnt1 up vs E15.5 * Sox10
10523190	9130213B05Rik	BC006604	6.54606e-005	1.5119	E15.5 * Wnt1 up vs E15.5 * Sox10
10476326	Cds2	NM_138651	3.53521e-005	1.51155	E15.5 * Wnt1 up vs E15.5 * Sox10
10546855	Srgap3	NM_080448	1.22131e-007	1.51148	E15.5 * Wnt1 up vs E15.5 * Sox10
10552929	Lin7b	NM_011698	0.000951556	1.51127	E15.5 * Wnt1 up vs E15.5 * Sox10
10593499	AI593442	NM_178906	3.79032e-005	1.50893	E15.5 * Wnt1 up vs E15.5 * Sox10
10535053	Prkar1b	NM_008923	1.54218e-005	1.50806	E15.5 * Wnt1 up vs E15.5 * Sox10

10462281	Vldlr	NM_013703	0.000162623	1.50804	E15.5 * Wnt1 up vs E15.5 * Sox10
10504692	Tmod1	NM_021883	0.000211821	1.50803	E15.5 * Wnt1 up vs E15.5 * Sox10
10458828	Cdo1	NM_033037	8.91218e-005	1.50788	E15.5 * Wnt1 up vs E15.5 * Sox10
10473349	Ypel4	NM_001005342	0.000185821	1.50756	E15.5 * Wnt1 up vs E15.5 * Sox10
10549879	Usp29	NM_021323	0.000117754	1.50725	E15.5 * Wnt1 up vs E15.5 * Sox10
10439514	Gap43	NM_008083	7.07723e-006	1.50715	E15.5 * Wnt1 up vs E15.5 * Sox10
10495613	4833424O15Rik	NM_029425	0.000595201	1.5054	E15.5 * Wnt1 up vs E15.5 * Sox10
10396314	Lrrc9	NM_001142728	0.000131539	1.50509	E15.5 * Wnt1 up vs E15.5 * Sox10
10491627	4932438A13Rik	NM_172679	0.00110027	1.50465	E15.5 * Wnt1 up vs E15.5 * Sox10
10496262	Nhedc2	NM_178877	0.000382459	1.50253	E15.5 * Wnt1 up vs E15.5 * Sox10
10555118	Pak1	NM_011035	3.3465e-006	1.50002	E15.5 * Wnt1 up vs E15.5 * Sox10
10520544	Mapre3	NM_133350	0.000784286	1.49904	E15.5 * Wnt1 up vs E15.5 * Sox10
10476596	Macrod2	NM_001013802	0.000203219	1.49877	E15.5 * Wnt1 up vs E15.5 * Sox10
10542414	Ptpro	NM_011216	0.000259315	1.49867	E15.5 * Wnt1 up vs E15.5 * Sox10
10490802	Fam164a	NM_173181	0.00405084	1.4976	E15.5 * Wnt1 up vs E15.5 * Sox10
10563899		---	1.59124e-009	1.4961	E15.5 * Wnt1 up vs E15.5 * Sox10
10452295	Tubb4	NM_009451	0.00124167	1.49513	E15.5 * Wnt1 up vs E15.5 * Sox10
10595033	Scg3	NM_009130	2.79465e-005	1.49501	E15.5 * Wnt1 up vs E15.5 * Sox10
10356880	St8sia4	NM_009183	7.15975e-005	1.49308	E15.5 * Wnt1 up vs E15.5 * Sox10
10593634	Elmod1	NM_177769	0.0015723	1.49268	E15.5 * Wnt1 up vs E15.5 * Sox10
10536611	Kcnd2	NM_019697	0.000354128	1.49234	E15.5 * Wnt1 up vs E15.5 * Sox10
10395770	1700047I17Rik1	BC048158	3.89528e-006	1.49218	E15.5 * Wnt1 up vs E15.5 * Sox10
10395780	1700047I17Rik1	BC048158	3.89528e-006	1.49218	E15.5 * Wnt1 up vs E15.5 * Sox10
10604906	Ids	NM_010498	2.26667e-005	1.49184	E15.5 * Wnt1 up vs E15.5 * Sox10
10443383	Tmhs	NM_026571	0.00173285	1.4913	E15.5 * Wnt1 up vs E15.5 * Sox10
10439695	Tagln3	NM_019754	7.96163e-005	1.4907	E15.5 * Wnt1 up vs E15.5 * Sox10
10547807	Eno2	NM_013509	0.00011998	1.49033	E15.5 * Wnt1 up vs E15.5 * Sox10
10366725	Fam19a2	NM_182807	1.19365e-006	1.48983	E15.5 * Wnt1 up vs E15.5 * Sox10
10578679	ENSMUSG0000C	ENSMUST00000(	1.20626e-005	1.4897	E15.5 * Wnt1 up vs E15.5 * Sox10
10577996	Unc5d	NM_153135	0.000521404	1.48945	E15.5 * Wnt1 up vs E15.5 * Sox10
10385203	Odz2	NM_011856	7.65694e-005	1.48913	E15.5 * Wnt1 up vs E15.5 * Sox10
10486201	OTTMUSG00000	ENSMUST00000(	0.00353254	1.48727	E15.5 * Wnt1 up vs E15.5 * Sox10
10532586	Myo18b	BC145218	0.00135523	1.48626	E15.5 * Wnt1 up vs E15.5 * Sox10
10395273	Gdap10	BC052902	0.00323358	1.48555	E15.5 * Wnt1 up vs E15.5 * Sox10
10397633	Flrt2	NM_201518	0.000195004	1.48533	E15.5 * Wnt1 up vs E15.5 * Sox10
10440849	Synj1	ENSMUST00000(	1.9801e-006	1.48502	E15.5 * Wnt1 up vs E15.5 * Sox10
10352980	Sntg1	NM_027671	4.90982e-005	1.48468	E15.5 * Wnt1 up vs E15.5 * Sox10
10564235		---	0.00467696	1.48437	E15.5 * Wnt1 up vs E15.5 * Sox10
10497663	Slc7a14	NM_172861	0.000210853	1.48393	E15.5 * Wnt1 up vs E15.5 * Sox10
10458685	Jakmip2	ENSMUST00000(	0.00499914	1.48293	E15.5 * Wnt1 up vs E15.5 * Sox10
10559580	Syt5	NM_016908	0.000104272	1.48289	E15.5 * Wnt1 up vs E15.5 * Sox10
10399121	Ptprn2	NM_011215	3.30671e-006	1.48264	E15.5 * Wnt1 up vs E15.5 * Sox10
10603843	Syn1	NM_013680	2.52269e-005	1.48207	E15.5 * Wnt1 up vs E15.5 * Sox10
10554166	Akap13	NM_029332	2.61403e-007	1.48203	E15.5 * Wnt1 up vs E15.5 * Sox10
10361642	Lrp11	NM_172784	0.00189049	1.48187	E15.5 * Wnt1 up vs E15.5 * Sox10
10455292	2900055J20Rik	ENSMUST00000(	0.000315646	1.48103	E15.5 * Wnt1 up vs E15.5 * Sox10
10384233	Tns3	NM_001083587	5.33622e-005	1.47909	E15.5 * Wnt1 up vs E15.5 * Sox10
10384797	Ccdc85a	NM_181577	1.79499e-005	1.47803	E15.5 * Wnt1 up vs E15.5 * Sox10
10601412	Lpar4	NM_175271	1.00979e-006	1.4755	E15.5 * Wnt1 up vs E15.5 * Sox10
10578957		---	0.00266613	1.47527	E15.5 * Wnt1 up vs E15.5 * Sox10
10430968	A4galt	NM_001004150	0.00040338	1.47373	E15.5 * Wnt1 up vs E15.5 * Sox10
10444756	Atp6v1g2	NM_023179	0.000765454	1.47281	E15.5 * Wnt1 up vs E15.5 * Sox10
10454441	Syt4	NM_009308	0.00275592	1.47269	E15.5 * Wnt1 up vs E15.5 * Sox10
10401987	Kcnk10	NM_029911	0.000716734	1.47141	E15.5 * Wnt1 up vs E15.5 * Sox10
10501374	5330417C22Rik	NM_001033304	4.34364e-006	1.47124	E15.5 * Wnt1 up vs E15.5 * Sox10
10537880		---	0.000234884	1.47081	E15.5 * Wnt1 up vs E15.5 * Sox10
10442580	Rps2	NM_008503	1.45299e-005	1.47003	E15.5 * Wnt1 up vs E15.5 * Sox10
10598138	Spry3	NM_001030293	0.00253207	1.46941	E15.5 * Wnt1 up vs E15.5 * Sox10
10564233		---	0.00220062	1.46749	E15.5 * Wnt1 up vs E15.5 * Sox10
10529656	Nsg1	NM_010942	7.11259e-006	1.46648	E15.5 * Wnt1 up vs E15.5 * Sox10
10540298	Chl1	NM_007697	0.000102155	1.46624	E15.5 * Wnt1 up vs E15.5 * Sox10
10420785	Mtmr9	ENSMUST00000(	0.00379415	1.46597	E15.5 * Wnt1 up vs E15.5 * Sox10
10555235	Arrb1	NM_177231	8.46678e-007	1.46499	E15.5 * Wnt1 up vs E15.5 * Sox10
10436196		---	0.000153591	1.46485	E15.5 * Wnt1 up vs E15.5 * Sox10
10524909	Nos1	NM_008712	5.8779e-005	1.46455	E15.5 * Wnt1 up vs E15.5 * Sox10
10508436	Sync	NM_023485	5.64188e-005	1.46421	E15.5 * Wnt1 up vs E15.5 * Sox10

10571353	6430573F11Rik	BC052184	0.000215764	1.46302	E15.5 * Wnt1 up vs E15.5 * Sox10
10409737	Agtpbp1	NM_023328	2.9362e-006	1.46063	E15.5 * Wnt1 up vs E15.5 * Sox10
10364712	Cirbp	NM_007705	0.00202845	1.45907	E15.5 * Wnt1 up vs E15.5 * Sox10
10546853	Srgap3	NM_080448	1.38075e-005	1.45893	E15.5 * Wnt1 up vs E15.5 * Sox10
10524312	Ttc28	ENSMUST00000	0.000184278	1.45777	E15.5 * Wnt1 up vs E15.5 * Sox10
10356177	Dner	NM_152915	0.000992074	1.45742	E15.5 * Wnt1 up vs E15.5 * Sox10
10362171	Stx7	NM_016797	5.67377e-006	1.4571	E15.5 * Wnt1 up vs E15.5 * Sox10
10356305	Htr2b	NM_008311	0.000644492	1.457	E15.5 * Wnt1 up vs E15.5 * Sox10
10519913	Magi2	NM_015823	0.00151778	1.45687	E15.5 * Wnt1 up vs E15.5 * Sox10
10448247	Zfp40	NM_009555	0.00376292	1.4567	E15.5 * Wnt1 up vs E15.5 * Sox10
10599736	Fhl1	NM_001077361	6.50841e-007	1.45653	E15.5 * Wnt1 up vs E15.5 * Sox10
10462957	Tbc1d12	NM_145952	0.00141651	1.45606	E15.5 * Wnt1 up vs E15.5 * Sox10
10366707	Avpr1a	NM_016847	0.000271676	1.4548	E15.5 * Wnt1 up vs E15.5 * Sox10
10381187	Atp6v0a1	NM_016920	3.84928e-006	1.45424	E15.5 * Wnt1 up vs E15.5 * Sox10
10423855	Rims2	NM_053271	0.000304594	1.45397	E15.5 * Wnt1 up vs E15.5 * Sox10
10363415	Spock2	NM_052994	0.000265429	1.45392	E15.5 * Wnt1 up vs E15.5 * Sox10
10485840	Ryr3	NM_177652	3.18176e-007	1.45259	E15.5 * Wnt1 up vs E15.5 * Sox10
10366346	Phlda1	NM_009344	1.5025e-005	1.45202	E15.5 * Wnt1 up vs E15.5 * Sox10
10522976	Rufy3	NM_027530	2.86478e-007	1.44961	E15.5 * Wnt1 up vs E15.5 * Sox10
10604380	Zdhhc9	NM_172465	9.89142e-005	1.44901	E15.5 * Wnt1 up vs E15.5 * Sox10
10541968	Ano2	NM_153589	2.11477e-005	1.44781	E15.5 * Wnt1 up vs E15.5 * Sox10
10411395	Rgnef	NM_012026	8.14823e-005	1.44772	E15.5 * Wnt1 up vs E15.5 * Sox10
10398727	Klc1	NM_001025360	0.000171874	1.44753	E15.5 * Wnt1 up vs E15.5 * Sox10
10595013	Tmod2	NM_016711	6.01715e-006	1.44741	E15.5 * Wnt1 up vs E15.5 * Sox10
10390117	Itga3	NM_013565	0.000135698	1.44729	E15.5 * Wnt1 up vs E15.5 * Sox10
10359480	Dnm3	NM_001038619	0.000252734	1.44622	E15.5 * Wnt1 up vs E15.5 * Sox10
10528090	Rundc3b	NM_198620	0.000790628	1.4458	E15.5 * Wnt1 up vs E15.5 * Sox10
10529923	Lcorl	NM_172153	0.000430403	1.44566	E15.5 * Wnt1 up vs E15.5 * Sox10
10567289	Syt17	NM_138649	0.00018872	1.44553	E15.5 * Wnt1 up vs E15.5 * Sox10
10360418	Rgs7	NM_011880	0.00393864	1.44514	E15.5 * Wnt1 up vs E15.5 * Sox10
10601846	2900062L11Rik	NR_003642	0.000861974	1.44484	E15.5 * Wnt1 up vs E15.5 * Sox10
10495343	Wdr47	NM_181400	0.0018031	1.44469	E15.5 * Wnt1 up vs E15.5 * Sox10
10571567	Sorbs2	NM_172752	2.9169e-006	1.44381	E15.5 * Wnt1 up vs E15.5 * Sox10
10411156	Scamp1	NM_029153	8.59057e-007	1.44334	E15.5 * Wnt1 up vs E15.5 * Sox10
10368373	C030003D03Rik	NM_029881	0.000334314	1.44289	E15.5 * Wnt1 up vs E15.5 * Sox10
10505030	Fsd1l	NM_176966	0.0009588	1.44166	E15.5 * Wnt1 up vs E15.5 * Sox10
10547282	Zfp9	NM_011763	0.000643646	1.4407	E15.5 * Wnt1 up vs E15.5 * Sox10
10531653	BC062109	BC055321	0.00330546	1.44065	E15.5 * Wnt1 up vs E15.5 * Sox10
10559837	Vmn2r29	NR_003555	4.51092e-005	1.44042	E15.5 * Wnt1 up vs E15.5 * Sox10
10363696	D630028G08Rik	AK038706	0.00240225	1.43945	E15.5 * Wnt1 up vs E15.5 * Sox10
10419416	3632451O06Rik	NM_026142	0.00140547	1.43899	E15.5 * Wnt1 up vs E15.5 * Sox10
10417048	Hs6st3	NM_015820	7.27565e-005	1.4389	E15.5 * Wnt1 up vs E15.5 * Sox10
10560911	Rabac1	NM_010261	0.000128791	1.43778	E15.5 * Wnt1 up vs E15.5 * Sox10
10502419	Rap1gds1	NM_001040690	1.42609e-005	1.43773	E15.5 * Wnt1 up vs E15.5 * Sox10
10491625	4932438A13Rik	NM_172679	0.00196823	1.43754	E15.5 * Wnt1 up vs E15.5 * Sox10
10473244	Zfp804a	NM_175513	0.000385834	1.4362	E15.5 * Wnt1 up vs E15.5 * Sox10
10359861	Mgst3	NM_025569	0.00342721	1.43551	E15.5 * Wnt1 up vs E15.5 * Sox10
10412345	Parp8	NM_001081009	0.000399082	1.43521	E15.5 * Wnt1 up vs E15.5 * Sox10
10411782	Pik3r1	NM_001077495	1.31449e-005	1.43416	E15.5 * Wnt1 up vs E15.5 * Sox10
10439881	5330426P16Rik	ENSMUST00000(	0.000188133	1.43392	E15.5 * Wnt1 up vs E15.5 * Sox10
10496854	Ttll7	NM_027594	0.000587547	1.43325	E15.5 * Wnt1 up vs E15.5 * Sox10
10438358	05-Sep	NM_213614	8.07763e-007	1.43252	E15.5 * Wnt1 up vs E15.5 * Sox10
10491136	Tnik	BC137799	4.77873e-007	1.43209	E15.5 * Wnt1 up vs E15.5 * Sox10
10490569	Kcnq2	NM_010611	1.63912e-005	1.43174	E15.5 * Wnt1 up vs E15.5 * Sox10
10389738	Dgke	NM_019505	4.54564e-005	1.43019	E15.5 * Wnt1 up vs E15.5 * Sox10
10345580	Inpp4a	NM_030266	5.69765e-005	1.43013	E15.5 * Wnt1 up vs E15.5 * Sox10
10591116	Fat3	NM_001080814	0.000212333	1.42923	E15.5 * Wnt1 up vs E15.5 * Sox10
10355474	March4	NM_001045533	0.000323493	1.42903	E15.5 * Wnt1 up vs E15.5 * Sox10
10588876	Nicn1	NM_025449	0.0025259	1.42783	E15.5 * Wnt1 up vs E15.5 * Sox10
10431051	Scube1	NM_022723	0.000265587	1.42706	E15.5 * Wnt1 up vs E15.5 * Sox10
10356968	Pam	NM_013626	0.000157423	1.42639	E15.5 * Wnt1 up vs E15.5 * Sox10
10446376	Man2a1	NM_008549	1.06103e-005	1.42627	E15.5 * Wnt1 up vs E15.5 * Sox10
10525195	AU042671	ENSMUST00000(	0.00092588	1.42536	E15.5 * Wnt1 up vs E15.5 * Sox10
10406229	Pcsk1	NM_013628	0.000769915	1.42349	E15.5 * Wnt1 up vs E15.5 * Sox10
10511290	Tnfrsf18	NM_009400	0.000477082	1.42307	E15.5 * Wnt1 up vs E15.5 * Sox10
10455071	Pcdhb7	NM_053132	0.00344999	1.42253	E15.5 * Wnt1 up vs E15.5 * Sox10

10481592	Dnm1	NM_010065	0.000264011	1.42239	E15.5 * Wnt1 up vs E15.5 * Sox10
10591706	Elavl3	NM_010487	0.00259639	1.42168	E15.5 * Wnt1 up vs E15.5 * Sox10
10448094	Lnpep	NM_172827	0.000114059	1.42137	E15.5 * Wnt1 up vs E15.5 * Sox10
10465314	Capn1	NM_007600	0.00048004	1.42133	E15.5 * Wnt1 up vs E15.5 * Sox10
10534041	Stx2	NM_007941	0.000132078	1.41993	E15.5 * Wnt1 up vs E15.5 * Sox10
10417628	Cadps	NM_012061	0.00216029	1.41987	E15.5 * Wnt1 up vs E15.5 * Sox10
10563897		---	0.00177059	1.41945	E15.5 * Wnt1 up vs E15.5 * Sox10
10433403	A2bp1	NM_021477	0.000248848	1.41799	E15.5 * Wnt1 up vs E15.5 * Sox10
10348493	Lrrfip1	NM_008515	1.57736e-006	1.41761	E15.5 * Wnt1 up vs E15.5 * Sox10
10576934	Fam155a	NM_173446	0.000218744	1.41749	E15.5 * Wnt1 up vs E15.5 * Sox10
10594679	Tln2	NM_001081242	2.82642e-005	1.41732	E15.5 * Wnt1 up vs E15.5 * Sox10
10441680	Pde10a	NM_011866	1.18866e-006	1.41659	E15.5 * Wnt1 up vs E15.5 * Sox10
10485582	Tcp11l1	NM_177190	0.00040427	1.41616	E15.5 * Wnt1 up vs E15.5 * Sox10
10392056	Cyb561	NM_007805	0.00250791	1.4148	E15.5 * Wnt1 up vs E15.5 * Sox10
10456161	Htr4	NM_008313	3.82212e-005	1.41475	E15.5 * Wnt1 up vs E15.5 * Sox10
10535065	Adap1	NM_172723	0.000197663	1.41454	E15.5 * Wnt1 up vs E15.5 * Sox10
10553501	Slc17a6	NM_080853	0.00184745	1.41396	E15.5 * Wnt1 up vs E15.5 * Sox10
10524889	Ksr2	NM_001114545	0.000103196	1.41231	E15.5 * Wnt1 up vs E15.5 * Sox10
10431711	Slc2a13	NM_001033633	0.000329911	1.41134	E15.5 * Wnt1 up vs E15.5 * Sox10
10441361	Tiam2	NM_001122998	0.000206223	1.41062	E15.5 * Wnt1 up vs E15.5 * Sox10
10558468	Jakmip3	BC119557	0.000619243	1.41041	E15.5 * Wnt1 up vs E15.5 * Sox10
10407370	4833420G17Rik	NM_001113550	0.000318813	1.41017	E15.5 * Wnt1 up vs E15.5 * Sox10
10588592	Cacna2d2	NM_020263	1.82176e-005	1.41012	E15.5 * Wnt1 up vs E15.5 * Sox10
10583179	Pgr	NM_008829	0.000175366	1.40984	E15.5 * Wnt1 up vs E15.5 * Sox10
10344990	Crispld1	NM_031402	9.90672e-006	1.40947	E15.5 * Wnt1 up vs E15.5 * Sox10
10498885	Gria2	NM_013540	0.0038245	1.40864	E15.5 * Wnt1 up vs E15.5 * Sox10
10604932	Cd99l2	NM_138309	0.00147043	1.40838	E15.5 * Wnt1 up vs E15.5 * Sox10
10350113	Arl8a	NM_026823	0.000704123	1.40812	E15.5 * Wnt1 up vs E15.5 * Sox10
10572146	Atp6v1b2	NM_007509	0.000187919	1.40795	E15.5 * Wnt1 up vs E15.5 * Sox10
10489985	Atp9a	NM_015731	0.00183919	1.40785	E15.5 * Wnt1 up vs E15.5 * Sox10
10411332	Hmgcr	NM_008255	0.000720598	1.40766	E15.5 * Wnt1 up vs E15.5 * Sox10
10569163	Cend1	NM_021316	0.000290303	1.40691	E15.5 * Wnt1 up vs E15.5 * Sox10
10506296		---	0.00379142	1.40594	E15.5 * Wnt1 up vs E15.5 * Sox10
10498952	Gucy1a3	NM_021896	0.00216666	1.40457	E15.5 * Wnt1 up vs E15.5 * Sox10
10345241	Dst	NM_134448	3.16528e-007	1.40344	E15.5 * Wnt1 up vs E15.5 * Sox10
10452793	Galnt14	NM_027864	0.000449677	1.40333	E15.5 * Wnt1 up vs E15.5 * Sox10
10473312	Fam171b	NM_175514	0.00180538	1.40299	E15.5 * Wnt1 up vs E15.5 * Sox10
10418817	Chat	NM_009891	6.9076e-005	1.40222	E15.5 * Wnt1 up vs E15.5 * Sox10
10348537	Ramp1	NM_016894	0.000150299	1.40208	E15.5 * Wnt1 up vs E15.5 * Sox10
10472408	Csrnp3	NM_153409	0.00100386	1.40107	E15.5 * Wnt1 up vs E15.5 * Sox10
10400057	Arl4a	NM_001039515	1.24126e-005	1.40101	E15.5 * Wnt1 up vs E15.5 * Sox10
10351111	Dnm3os	NR_002870	0.00357406	1.40098	E15.5 * Wnt1 up vs E15.5 * Sox10
10542894		---	0.000648897	1.4009	E15.5 * Wnt1 up vs E15.5 * Sox10
10559681	D430041B17Rik	NM_172737	8.60557e-006	1.40046	E15.5 * Wnt1 up vs E15.5 * Sox10
10434446	Ece2	NM_139293	0.000104416	1.40001	E15.5 * Wnt1 up vs E15.5 * Sox10
10521995	3110047P20Rik	NM_177006	0.00483533	1.3998	E15.5 * Wnt1 up vs E15.5 * Sox10
10540880	Syn2	NM_001111015	0.000163418	1.39934	E15.5 * Wnt1 up vs E15.5 * Sox10
10602428	Wnk3	ENSMUST00000(	0.0017439	1.39897	E15.5 * Wnt1 up vs E15.5 * Sox10
10542401	BC049715	NM_178776	0.00308568	1.39862	E15.5 * Wnt1 up vs E15.5 * Sox10
10543369	Cadps2	NM_153163	1.12523e-005	1.39755	E15.5 * Wnt1 up vs E15.5 * Sox10
10564237	ENSMUSG00000	ENSMUST00000(	0.00369054	1.39741	E15.5 * Wnt1 up vs E15.5 * Sox10
10521498	Crmp1	NM_007765	0.00070506	1.39677	E15.5 * Wnt1 up vs E15.5 * Sox10
10497505	Nlgn1	NM_138666	0.00187792	1.39662	E15.5 * Wnt1 up vs E15.5 * Sox10
10562204	Fxyd7	NM_022007	1.81647e-005	1.39507	E15.5 * Wnt1 up vs E15.5 * Sox10
10349184	Cdh7	NM_172853	3.80445e-006	1.39297	E15.5 * Wnt1 up vs E15.5 * Sox10
10486875	Frmd5	NM_172673	0.000315938	1.39064	E15.5 * Wnt1 up vs E15.5 * Sox10
10518835	Camta1	NM_001081557	0.0016267	1.39048	E15.5 * Wnt1 up vs E15.5 * Sox10
10483353	Scn7a	NM_009135	0.00452426	1.39035	E15.5 * Wnt1 up vs E15.5 * Sox10
10395457	Etv1	NM_007960	0.000715988	1.39024	E15.5 * Wnt1 up vs E15.5 * Sox10
10584572	Hspa8	NM_031165	2.51324e-006	1.39018	E15.5 * Wnt1 up vs E15.5 * Sox10
10532542	Sez6l	NM_019982	0.000175264	1.38896	E15.5 * Wnt1 up vs E15.5 * Sox10
10557535	Sez6l2	NM_144926	0.000661019	1.38831	E15.5 * Wnt1 up vs E15.5 * Sox10
10496519	Unc5c	NM_009472	1.25653e-005	1.38821	E15.5 * Wnt1 up vs E15.5 * Sox10
10452734	Alk	NM_007439	0.000186321	1.38794	E15.5 * Wnt1 up vs E15.5 * Sox10
10584883	Fxyd6	NM_022004	5.13748e-005	1.3874	E15.5 * Wnt1 up vs E15.5 * Sox10
10415700	Mtmr6	NM_144843	0.00496919	1.38604	E15.5 * Wnt1 up vs E15.5 * Sox10

10482336	Lrp1b	NM_053011	3.29527e-006	1.38599	E15.5 * Wnt1 up vs E15.5 * Sox10
10362861	Scml4	NM_172938	2.51658e-006	1.38596	E15.5 * Wnt1 up vs E15.5 * Sox10
10381006	Thra	NM_178060	0.00232372	1.38584	E15.5 * Wnt1 up vs E15.5 * Sox10
10530319	Atp8a1	NM_001038999	5.47319e-007	1.38519	E15.5 * Wnt1 up vs E15.5 * Sox10
10385234		---	0.000227264	1.38512	E15.5 * Wnt1 up vs E15.5 * Sox10
10589130	Celsr3	NM_080437	0.000479294	1.38493	E15.5 * Wnt1 up vs E15.5 * Sox10
10593671	Dmxl2	NM_172771	4.13695e-005	1.38467	E15.5 * Wnt1 up vs E15.5 * Sox10
10554667	Tmc3	NM_177695	6.86441e-006	1.38462	E15.5 * Wnt1 up vs E15.5 * Sox10
10408937	Atxn1	NM_009124	0.00336455	1.38422	E15.5 * Wnt1 up vs E15.5 * Sox10
10362147	Taar1	NM_053205	0.000627842	1.38371	E15.5 * Wnt1 up vs E15.5 * Sox10
10543952	9330158H04Rik	ENSMUST00000	0.0015345	1.38362	E15.5 * Wnt1 up vs E15.5 * Sox10
10470834	Spna2	NM_001076554	3.71477e-005	1.3834	E15.5 * Wnt1 up vs E15.5 * Sox10
10372421	Trhde	NM_146241	0.00034887	1.38339	E15.5 * Wnt1 up vs E15.5 * Sox10
10396278	Daam1	NM_026102	2.20005e-005	1.38273	E15.5 * Wnt1 up vs E15.5 * Sox10
10552030	C630016N16Rik	AK049951	0.000772091	1.38269	E15.5 * Wnt1 up vs E15.5 * Sox10
10571371	Tusc3	NM_030254	5.28764e-007	1.38266	E15.5 * Wnt1 up vs E15.5 * Sox10
10345571	Cnga3	NM_009918	0.000217673	1.38263	E15.5 * Wnt1 up vs E15.5 * Sox10
10432294	C430014K11Rik	ENSMUST00000	3.82063e-005	1.38242	E15.5 * Wnt1 up vs E15.5 * Sox10
10543219	Gpr85	NM_145066	0.000101505	1.38207	E15.5 * Wnt1 up vs E15.5 * Sox10
10578300	Mtmr7	NM_001040699	0.000241406	1.38152	E15.5 * Wnt1 up vs E15.5 * Sox10
10391987		---	8.04928e-006	1.38134	E15.5 * Wnt1 up vs E15.5 * Sox10
10607302	Gnl3l	NM_198110	1.76626e-006	1.38126	E15.5 * Wnt1 up vs E15.5 * Sox10
10428443	Kcnv1	NM_026200	0.00363185	1.38092	E15.5 * Wnt1 up vs E15.5 * Sox10
10346150	Tmeff2	NM_019790	3.86594e-005	1.3803	E15.5 * Wnt1 up vs E15.5 * Sox10
10457686	Dsc2	NM_013505	4.87418e-005	1.37851	E15.5 * Wnt1 up vs E15.5 * Sox10
10483299	Scn1a	NM_018733	1.13445e-006	1.37782	E15.5 * Wnt1 up vs E15.5 * Sox10
10543802	Plxna4	NM_175750	0.000363316	1.3776	E15.5 * Wnt1 up vs E15.5 * Sox10
10512915	2810432L12Rik	NM_025944	0.00267383	1.37722	E15.5 * Wnt1 up vs E15.5 * Sox10
10356379	Ecel1	NM_021306	0.000233901	1.37695	E15.5 * Wnt1 up vs E15.5 * Sox10
10563921		---	4.75874e-007	1.37488	E15.5 * Wnt1 up vs E15.5 * Sox10
10563925		---	4.75874e-007	1.37488	E15.5 * Wnt1 up vs E15.5 * Sox10
10463911	Add3	NM_013758	0.000133461	1.37474	E15.5 * Wnt1 up vs E15.5 * Sox10
10451213	Rsph9	NM_029338	0.00313641	1.37458	E15.5 * Wnt1 up vs E15.5 * Sox10
10376998	A530088H08Rik	EU447173	0.00302496	1.37452	E15.5 * Wnt1 up vs E15.5 * Sox10
10445071	Zfp57	NM_001013745	1.54145e-005	1.37419	E15.5 * Wnt1 up vs E15.5 * Sox10
10557033	Eef2k	ENSMUST00000	0.00105514	1.3728	E15.5 * Wnt1 up vs E15.5 * Sox10
10592471	Gramd1b	NM_172768	0.000922467	1.37217	E15.5 * Wnt1 up vs E15.5 * Sox10
10383564	Fn3k	NM_001038699	0.00015144	1.3719	E15.5 * Wnt1 up vs E15.5 * Sox10
10355214	Idh1	NM_010497	6.31568e-006	1.3718	E15.5 * Wnt1 up vs E15.5 * Sox10
10566993	Galntl4	NM_173739	0.00188787	1.37173	E15.5 * Wnt1 up vs E15.5 * Sox10
10441642	Brp44l	NM_018819	0.0014318	1.37172	E15.5 * Wnt1 up vs E15.5 * Sox10
10595953	Fam62c	NM_177775	4.03396e-005	1.37028	E15.5 * Wnt1 up vs E15.5 * Sox10
10606835	Bex2	NM_009749	0.00121419	1.37006	E15.5 * Wnt1 up vs E15.5 * Sox10
10512315	Ccl27a	NM_001048179	0.000884082	1.36945	E15.5 * Wnt1 up vs E15.5 * Sox10
10499045	Trim2	NM_030706	1.43064e-005	1.36872	E15.5 * Wnt1 up vs E15.5 * Sox10
10410995	Rasgrf2	NM_009027	6.86935e-005	1.3686	E15.5 * Wnt1 up vs E15.5 * Sox10
10599853	Ldoc1	NM_001018087	0.00497561	1.3682	E15.5 * Wnt1 up vs E15.5 * Sox10
10585905	Parp6	NM_029922	4.36215e-005	1.36645	E15.5 * Wnt1 up vs E15.5 * Sox10
10549171	5730419I09Rik	NM_029081	0.000115833	1.36635	E15.5 * Wnt1 up vs E15.5 * Sox10
10539211	Lrrtm4	NR_027323	0.000246999	1.36593	E15.5 * Wnt1 up vs E15.5 * Sox10
10549162	St8sia1	NM_011374	0.00100522	1.36566	E15.5 * Wnt1 up vs E15.5 * Sox10
10547719	Clstn3	NM_153508	0.000256387	1.36498	E15.5 * Wnt1 up vs E15.5 * Sox10
10600419	Plxna3	NM_008883	0.000783237	1.36479	E15.5 * Wnt1 up vs E15.5 * Sox10
10525487	4932422M17Rik	ENSMUST00000	0.00389524	1.36474	E15.5 * Wnt1 up vs E15.5 * Sox10
10601360	Atp7a	NM_001109757	5.1199e-005	1.36424	E15.5 * Wnt1 up vs E15.5 * Sox10
10458843	Sema6a	NM_018744	0.00013562	1.36322	E15.5 * Wnt1 up vs E15.5 * Sox10
10384504	Meis1	NM_010789	0.000107814	1.36294	E15.5 * Wnt1 up vs E15.5 * Sox10
10568298	Stx1b	NM_024414	0.00136934	1.36223	E15.5 * Wnt1 up vs E15.5 * Sox10
10346330	Plcl1	NM_001114663	0.000401183	1.36185	E15.5 * Wnt1 up vs E15.5 * Sox10
10587012	Ccpg1	NM_001114328	8.79061e-005	1.36173	E15.5 * Wnt1 up vs E15.5 * Sox10
10552245	Tshz3	NM_172298	9.51641e-005	1.36114	E15.5 * Wnt1 up vs E15.5 * Sox10
10544829	Jazf1	NM_173406	6.20182e-005	1.3597	E15.5 * Wnt1 up vs E15.5 * Sox10
10457895	Brunol4	NM_001146292	0.000847654	1.35962	E15.5 * Wnt1 up vs E15.5 * Sox10
10387194	Odf4	NM_145746	0.000349779	1.35959	E15.5 * Wnt1 up vs E15.5 * Sox10
10495163	Tmem77	NM_026013	0.0016496	1.35865	E15.5 * Wnt1 up vs E15.5 * Sox10
10349876	Plekha6	NM_182930	0.000272617	1.35834	E15.5 * Wnt1 up vs E15.5 * Sox10

10511580	Ppm2c	NM_001098230	5.06031e-005	1.35823	E15.5 * Wnt1 up vs E15.5 * Sox10
10601701	Tmem35	NM_026239	0.00121634	1.3576	E15.5 * Wnt1 up vs E15.5 * Sox10
10455118	Pcdhb18	NM_053143	0.0025162	1.35619	E15.5 * Wnt1 up vs E15.5 * Sox10
10369210	Serinc1	NM_019760	8.71072e-006	1.35593	E15.5 * Wnt1 up vs E15.5 * Sox10
10569972	Lass4	NM_026058	0.00372	1.35592	E15.5 * Wnt1 up vs E15.5 * Sox10
10565102	Ap3b2	NM_021492	0.000739576	1.35511	E15.5 * Wnt1 up vs E15.5 * Sox10
10361358	Rgs17	NM_019958	0.000223812	1.3546	E15.5 * Wnt1 up vs E15.5 * Sox10
10376163	Rapgef6	NM_175258	9.9018e-007	1.35406	E15.5 * Wnt1 up vs E15.5 * Sox10
10440314	Cadm2	NM_178721	0.000359244	1.35366	E15.5 * Wnt1 up vs E15.5 * Sox10
10601771	Armcx1	NM_030066	0.000191369	1.35328	E15.5 * Wnt1 up vs E15.5 * Sox10
10456756	Zbtb7c	NM_145356	0.000221368	1.35299	E15.5 * Wnt1 up vs E15.5 * Sox10
10360344	Darc	NM_010045	0.000669701	1.35242	E15.5 * Wnt1 up vs E15.5 * Sox10
10456248	Onecut2	NM_194268	0.00105621	1.35187	E15.5 * Wnt1 up vs E15.5 * Sox10
10449258	Arhgdig	NM_008113	0.00240586	1.35115	E15.5 * Wnt1 up vs E15.5 * Sox10
10497689	Gnb4	NM_013531	8.53966e-005	1.35101	E15.5 * Wnt1 up vs E15.5 * Sox10
10422075	Mycbp2	NM_207215	1.85373e-006	1.35078	E15.5 * Wnt1 up vs E15.5 * Sox10
10362363	6330407J23Rik	NM_026138	0.00105775	1.35013	E15.5 * Wnt1 up vs E15.5 * Sox10
10602896	Gpr64	NM_178712	0.000185237	1.34841	E15.5 * Wnt1 up vs E15.5 * Sox10
10480490		---	0.00312051	1.34779	E15.5 * Wnt1 up vs E15.5 * Sox10
10593799	Scaper	NM_001081341	4.24195e-005	1.3471	E15.5 * Wnt1 up vs E15.5 * Sox10
10361191	Syt14	NM_181546	0.000236433	1.34653	E15.5 * Wnt1 up vs E15.5 * Sox10
10350697	Nmnat2	NM_175460	0.00481033	1.34595	E15.5 * Wnt1 up vs E15.5 * Sox10
10434436	Vwa5b2	NM_001144953	0.00224818	1.34579	E15.5 * Wnt1 up vs E15.5 * Sox10
10384838	C230094A16Rik	NM_146016	0.00183486	1.34506	E15.5 * Wnt1 up vs E15.5 * Sox10
10538373	Prr15	NM_030024	0.00303267	1.34491	E15.5 * Wnt1 up vs E15.5 * Sox10
10427369	Pde1b	NM_008800	8.64718e-005	1.34437	E15.5 * Wnt1 up vs E15.5 * Sox10
10492516	Iqcj	NM_177585	0.000246682	1.34315	E15.5 * Wnt1 up vs E15.5 * Sox10
10503593	6230409E13Rik	NM_175234	0.000166009	1.34302	E15.5 * Wnt1 up vs E15.5 * Sox10
10367775	Stxbp5	NM_001081344	0.000555529	1.34285	E15.5 * Wnt1 up vs E15.5 * Sox10
10579554	Unc13a	NM_001029873	0.000589701	1.34238	E15.5 * Wnt1 up vs E15.5 * Sox10
10352439	Susd4	NM_144796	0.000434329	1.34192	E15.5 * Wnt1 up vs E15.5 * Sox10
10574384	Ndrg4	NM_145602	0.000462113	1.34179	E15.5 * Wnt1 up vs E15.5 * Sox10
10361790	Fuca2	NM_025799	0.00152557	1.3413	E15.5 * Wnt1 up vs E15.5 * Sox10
10587023	Rab27a	NM_023635	1.94081e-005	1.34036	E15.5 * Wnt1 up vs E15.5 * Sox10
10596583	Dock3	NM_153413	0.000727478	1.33979	E15.5 * Wnt1 up vs E15.5 * Sox10
10510422	Casz1	NM_001159344	4.08686e-005	1.33943	E15.5 * Wnt1 up vs E15.5 * Sox10
10472418	Scn9a	ENSMUST00000	0.000714357	1.33927	E15.5 * Wnt1 up vs E15.5 * Sox10
10416340	Gfra2	NM_008115	0.00129003	1.3384	E15.5 * Wnt1 up vs E15.5 * Sox10
10362115	1110021L09Rik	NM_183116	0.000234037	1.33828	E15.5 * Wnt1 up vs E15.5 * Sox10
10521759	Slit2	NM_178804	0.000405543	1.33716	E15.5 * Wnt1 up vs E15.5 * Sox10
10534021	Rimbp2	NM_001081388	1.45989e-006	1.33657	E15.5 * Wnt1 up vs E15.5 * Sox10
10442219	Zfp52	NM_144515	0.00314423	1.33641	E15.5 * Wnt1 up vs E15.5 * Sox10
10607888	Frmpd4	NM_001033330	0.00147406	1.33573	E15.5 * Wnt1 up vs E15.5 * Sox10
10416541	Enox1	NM_172813	3.6706e-005	1.33556	E15.5 * Wnt1 up vs E15.5 * Sox10
10370242	Pcbp3	NM_021568	0.000228215	1.33555	E15.5 * Wnt1 up vs E15.5 * Sox10
10454254	Dtna	NM_207650	0.000121626	1.3355	E15.5 * Wnt1 up vs E15.5 * Sox10
10593756	Chrna3	NM_145129	0.000512476	1.33525	E15.5 * Wnt1 up vs E15.5 * Sox10
10541049	March8	NM_027920	0.000503694	1.33471	E15.5 * Wnt1 up vs E15.5 * Sox10
10583847	Bbs9	NM_178415	0.000189802	1.33441	E15.5 * Wnt1 up vs E15.5 * Sox10
10416112	Pnma2	NM_175498	0.00268259	1.3342	E15.5 * Wnt1 up vs E15.5 * Sox10
10466248	Stx3	NM_001025307	0.000408819	1.33397	E15.5 * Wnt1 up vs E15.5 * Sox10
10413771	Capn7	NM_009796	0.000258259	1.33364	E15.5 * Wnt1 up vs E15.5 * Sox10
10502224	Sgms2	NM_028943	0.00362283	1.33335	E15.5 * Wnt1 up vs E15.5 * Sox10
10515894	1700041C02Rik	NM_029286	0.000659025	1.33251	E15.5 * Wnt1 up vs E15.5 * Sox10
10563943		---	0.00163972	1.33152	E15.5 * Wnt1 up vs E15.5 * Sox10
10581266	Tppp3	NM_026481	0.00114869	1.33127	E15.5 * Wnt1 up vs E15.5 * Sox10
10427303	Hoxc4	NM_013553	0.00285636	1.32959	E15.5 * Wnt1 up vs E15.5 * Sox10
10464030	Adra2a	NM_007417	0.00155415	1.32958	E15.5 * Wnt1 up vs E15.5 * Sox10
10538408	2410066E13Rik	BC042507	0.000360872	1.3294	E15.5 * Wnt1 up vs E15.5 * Sox10
10506360	Sgip1	NM_144906	3.8546e-005	1.3289	E15.5 * Wnt1 up vs E15.5 * Sox10
10590306	Entpd3	NM_178676	0.00171959	1.32882	E15.5 * Wnt1 up vs E15.5 * Sox10
10392560	Abca9	NM_147220	0.000497806	1.32841	E15.5 * Wnt1 up vs E15.5 * Sox10
10565089	Cpeb1	NM_007755	0.000399683	1.32823	E15.5 * Wnt1 up vs E15.5 * Sox10
10481711	Stxbp1	NM_001113569	0.00181523	1.32779	E15.5 * Wnt1 up vs E15.5 * Sox10
10602009	Rnf128	NM_023270	0.000192825	1.32765	E15.5 * Wnt1 up vs E15.5 * Sox10
10458663	Dpysl3	NM_009468	0.000482723	1.32764	E15.5 * Wnt1 up vs E15.5 * Sox10

10576495	Trim67	BC094596	0.000272938	1.32759	E15.5 * Wnt1 up vs E15.5 * Sox10
10564624	St8sia2	NM_009181	0.00192258	1.32723	E15.5 * Wnt1 up vs E15.5 * Sox10
10428534	Trps1	NM_032000	0.00147331	1.32717	E15.5 * Wnt1 up vs E15.5 * Sox10
10598996	EG331392	NM_198633	0.0042986	1.32626	E15.5 * Wnt1 up vs E15.5 * Sox10
10544089	Zc3hav1	NM_028421	0.000273786	1.32556	E15.5 * Wnt1 up vs E15.5 * Sox10
10504234	Unc13b	NM_001081413	0.000115176	1.32519	E15.5 * Wnt1 up vs E15.5 * Sox10
10498210	Nbea	NM_030595	2.86743e-006	1.32464	E15.5 * Wnt1 up vs E15.5 * Sox10
10351140	Kifap3	NM_010629	1.26807e-005	1.32462	E15.5 * Wnt1 up vs E15.5 * Sox10
10578324	Mtus1	NM_001005863	6.14178e-005	1.32317	E15.5 * Wnt1 up vs E15.5 * Sox10
10608288		---	0.000325177	1.32296	E15.5 * Wnt1 up vs E15.5 * Sox10
10608300		---	0.000325177	1.32296	E15.5 * Wnt1 up vs E15.5 * Sox10
10561369	BC089491	NM_175033	0.00151946	1.32283	E15.5 * Wnt1 up vs E15.5 * Sox10
10454235	Asxl3	ENSMUST00000	0.000219261	1.3228	E15.5 * Wnt1 up vs E15.5 * Sox10
10463875	Sorcs3	NM_025696	0.00243426	1.32203	E15.5 * Wnt1 up vs E15.5 * Sox10
10455019	Pcdha10	NM_009961	3.90737e-005	1.32155	E15.5 * Wnt1 up vs E15.5 * Sox10
10598626	Tspan7	NM_019634	0.000791265	1.32083	E15.5 * Wnt1 up vs E15.5 * Sox10
10485828	Slc12a6	NM_133648	0.000414915	1.31995	E15.5 * Wnt1 up vs E15.5 * Sox10
10375002	Cpeb4	NM_026252	2.80331e-006	1.31892	E15.5 * Wnt1 up vs E15.5 * Sox10
10458052	Epb4.1l4a	NM_013512	0.000552729	1.3186	E15.5 * Wnt1 up vs E15.5 * Sox10
10527233	Cyth3	NM_011182	4.32345e-005	1.31786	E15.5 * Wnt1 up vs E15.5 * Sox10
10476633	Pcsk2	NM_008792	0.00318319	1.31773	E15.5 * Wnt1 up vs E15.5 * Sox10
10392388	Prkca	NM_011101	0.000338269	1.31748	E15.5 * Wnt1 up vs E15.5 * Sox10
10428020	March6	NM_172606	7.43531e-005	1.31692	E15.5 * Wnt1 up vs E15.5 * Sox10
10541288	ENSMUSG0000C	ENSMUST00000	0.0012205	1.31637	E15.5 * Wnt1 up vs E15.5 * Sox10
10547521	Atp6v1e1	NM_007510	0.00337493	1.31634	E15.5 * Wnt1 up vs E15.5 * Sox10
10600082	Nsdhl	NM_010941	0.0037106	1.31621	E15.5 * Wnt1 up vs E15.5 * Sox10
10600390	Gdi1	NM_010273	0.000415477	1.31575	E15.5 * Wnt1 up vs E15.5 * Sox10
10365830		---	5.89325e-005	1.31472	E15.5 * Wnt1 up vs E15.5 * Sox10
10438103	Ccdc116	NM_029779	5.28028e-005	1.31378	E15.5 * Wnt1 up vs E15.5 * Sox10
10604038	Sfrs17b	NM_001081956	0.00342826	1.31369	E15.5 * Wnt1 up vs E15.5 * Sox10
10383684	Limk2	NM_010718	1.08657e-005	1.31335	E15.5 * Wnt1 up vs E15.5 * Sox10
10347018	Pth2r	NM_139270	0.00194784	1.313	E15.5 * Wnt1 up vs E15.5 * Sox10
10515095	Elavl4	NM_010488	0.00276012	1.31283	E15.5 * Wnt1 up vs E15.5 * Sox10
10458913	Cep120	NM_178686	0.000183695	1.31264	E15.5 * Wnt1 up vs E15.5 * Sox10
10422059	Kctd12	NM_177715	0.00239248	1.31254	E15.5 * Wnt1 up vs E15.5 * Sox10
10454655	Apc	NM_007462	6.70899e-005	1.31196	E15.5 * Wnt1 up vs E15.5 * Sox10
10604961	Gabra3	NM_008067	0.00234268	1.31175	E15.5 * Wnt1 up vs E15.5 * Sox10
10403604	Lyst	NM_010748	0.000260379	1.31164	E15.5 * Wnt1 up vs E15.5 * Sox10
10565634	Myo7a	NM_008663	4.00895e-005	1.31141	E15.5 * Wnt1 up vs E15.5 * Sox10
10381939	Tanc2	NM_181071	0.000502001	1.31111	E15.5 * Wnt1 up vs E15.5 * Sox10
10512847	Alg2	NM_019998	0.000809474	1.31102	E15.5 * Wnt1 up vs E15.5 * Sox10
10409616	Spock1	NM_009262	0.000528376	1.31026	E15.5 * Wnt1 up vs E15.5 * Sox10
10379153	Aldoc	NM_009657	0.00431312	1.30978	E15.5 * Wnt1 up vs E15.5 * Sox10
10576062	Jph3	NM_020605	0.00152292	1.30975	E15.5 * Wnt1 up vs E15.5 * Sox10
10465424	Nrxn2	NM_020253	0.00295928	1.30968	E15.5 * Wnt1 up vs E15.5 * Sox10
10597309	Stac	NM_016853	0.00370083	1.30943	E15.5 * Wnt1 up vs E15.5 * Sox10
10606026	Zmym3	NM_019831	0.000784513	1.3094	E15.5 * Wnt1 up vs E15.5 * Sox10
10555009		---	0.000277616	1.30934	E15.5 * Wnt1 up vs E15.5 * Sox10
10408610	Tubb2a	NM_009450	0.000151829	1.30916	E15.5 * Wnt1 up vs E15.5 * Sox10
10480676	Grin1	NM_008169	1.23974e-005	1.30866	E15.5 * Wnt1 up vs E15.5 * Sox10
10426479	Ano6	NM_175344	0.000324749	1.30861	E15.5 * Wnt1 up vs E15.5 * Sox10
10437210	Bace2	NM_019517	0.000169997	1.30826	E15.5 * Wnt1 up vs E15.5 * Sox10
10456206	Wdr7	NM_001014981	3.67655e-005	1.30808	E15.5 * Wnt1 up vs E15.5 * Sox10
10543120	Ica1	NM_010492	0.00343184	1.30765	E15.5 * Wnt1 up vs E15.5 * Sox10
10426924	Slc4a8	NM_021530	0.00123394	1.30757	E15.5 * Wnt1 up vs E15.5 * Sox10
10539472	Nagk	NM_019542	0.0022166	1.30699	E15.5 * Wnt1 up vs E15.5 * Sox10
10547322	Cacna1c	NM_001159533	2.83576e-005	1.30685	E15.5 * Wnt1 up vs E15.5 * Sox10
10500469	Pde4dip	NM_001039376	3.60722e-005	1.30662	E15.5 * Wnt1 up vs E15.5 * Sox10
10499483	Fdps	NM_134469	0.000202442	1.30653	E15.5 * Wnt1 up vs E15.5 * Sox10
10599174	Il13ra1	NM_133990	0.0010499	1.30631	E15.5 * Wnt1 up vs E15.5 * Sox10
10364194	Lss	NM_146006	0.00121019	1.30596	E15.5 * Wnt1 up vs E15.5 * Sox10
10533869	Ccdc92	NM_144819	0.0046768	1.30593	E15.5 * Wnt1 up vs E15.5 * Sox10
10495993	Elovl6	NM_130450	0.00265587	1.30567	E15.5 * Wnt1 up vs E15.5 * Sox10
10593384	Dixdc1	NM_178118	3.54511e-005	1.30528	E15.5 * Wnt1 up vs E15.5 * Sox10
10441073	Kcnj6	NM_010606	0.000492086	1.30511	E15.5 * Wnt1 up vs E15.5 * Sox10
10518585	Kif1b	NM_207682	3.67343e-005	1.30431	E15.5 * Wnt1 up vs E15.5 * Sox10

10345350		---	0.000852001	1.30375	E15.5 * Wnt1 up vs E15.5 * Sox10
10591092	Fat3	NM_001080814	0.00108982	1.3027	E15.5 * Wnt1 up vs E15.5 * Sox10
10355141	Klf7	NM_033563	3.6469e-005	1.30265	E15.5 * Wnt1 up vs E15.5 * Sox10
10418879	Mapk8	NM_016700	0.000159941	1.30235	E15.5 * Wnt1 up vs E15.5 * Sox10
10554900	Dlg2	NM_011807	0.00148921	1.30193	E15.5 * Wnt1 up vs E15.5 * Sox10
10519815	Cacna2d1	NM_001110843	0.00400977	1.30127	E15.5 * Wnt1 up vs E15.5 * Sox10
10477637	Map1lc3a	NM_025735	0.003756	1.30125	E15.5 * Wnt1 up vs E15.5 * Sox10
10375572	Olfr1382	NM_001011790	0.00313031	1.3008	E15.5 * Wnt1 up vs E15.5 * Sox10
10483381	Stk39	NM_016866	2.14584e-005	1.30035	E15.5 * Wnt1 up vs E15.5 * Sox10
10399854	Slc26a4	NM_011867	0.00181331	1.3003	E15.5 * Wnt1 up vs E15.5 * Sox10
10396454	Syt16	NM_172804	9.26347e-005	1.29969	E15.5 * Wnt1 up vs E15.5 * Sox10
10347036	Mtap2	NM_001039934	4.46152e-005	1.29946	E15.5 * Wnt1 up vs E15.5 * Sox10
10526792	0910001L09Rik	NM_001081108	2.96375e-005	1.29925	E15.5 * Wnt1 up vs E15.5 * Sox10
10539669	Add2	NM_013458	3.52106e-005	1.29882	E15.5 * Wnt1 up vs E15.5 * Sox10
10377534	A030009H04Rik	AB041807	0.000402177	1.29881	E15.5 * Wnt1 up vs E15.5 * Sox10
10440158	Olfr191	NM_001011807	0.00300967	1.29819	E15.5 * Wnt1 up vs E15.5 * Sox10
10549420	Tmtc1	NM_198967	0.00187443	1.29796	E15.5 * Wnt1 up vs E15.5 * Sox10
10468980	Fam107b	NM_025626	0.00116307	1.29788	E15.5 * Wnt1 up vs E15.5 * Sox10
10469622	Gpr158	NM_001004761	0.00159943	1.29777	E15.5 * Wnt1 up vs E15.5 * Sox10
10377431	Vamp2	NM_009497	0.000795642	1.29744	E15.5 * Wnt1 up vs E15.5 * Sox10
10571384	Efha2	ENSMUST00000(	0.00365991	1.2974	E15.5 * Wnt1 up vs E15.5 * Sox10
10372385	Zdhhc17	NM_172554	0.000422992	1.29698	E15.5 * Wnt1 up vs E15.5 * Sox10
10444521	Zbtb12	NM_198886	0.000996926	1.29667	E15.5 * Wnt1 up vs E15.5 * Sox10
10551410	Zfp60	NM_009560	0.00265927	1.29624	E15.5 * Wnt1 up vs E15.5 * Sox10
10487748	4930402H24Rik	BC052447	9.17729e-005	1.29549	E15.5 * Wnt1 up vs E15.5 * Sox10
10351971	Fmn2	NM_019445	0.00213525	1.29515	E15.5 * Wnt1 up vs E15.5 * Sox10
10500114	Mllt11	NM_019914	0.000122556	1.29452	E15.5 * Wnt1 up vs E15.5 * Sox10
10366825	Agap2	NM_001033263	0.00238308	1.29387	E15.5 * Wnt1 up vs E15.5 * Sox10
10367634	Akap12	NM_031185	4.97919e-005	1.29381	E15.5 * Wnt1 up vs E15.5 * Sox10
10395466	Dock4	NM_172803	0.000281686	1.29338	E15.5 * Wnt1 up vs E15.5 * Sox10
10458046	D0H4S114	NM_053078	5.44497e-006	1.2926	E15.5 * Wnt1 up vs E15.5 * Sox10
10375216	Pank3	NM_145962	0.001052	1.29244	E15.5 * Wnt1 up vs E15.5 * Sox10
10366293	Csrp2	NM_007792	0.00192446	1.29227	E15.5 * Wnt1 up vs E15.5 * Sox10
10599719	Slc9a6	NM_172780	0.000838139	1.2922	E15.5 * Wnt1 up vs E15.5 * Sox10
10396862	Strm	AF031663	0.000472097	1.29201	E15.5 * Wnt1 up vs E15.5 * Sox10
10412667	Ptprg	NM_008981	0.000835221	1.29144	E15.5 * Wnt1 up vs E15.5 * Sox10
10532150	Fam69a	NM_026062	0.00262396	1.29089	E15.5 * Wnt1 up vs E15.5 * Sox10
10368484	ENSMUSG0000C	ENSMUST00000(	0.000852085	1.29086	E15.5 * Wnt1 up vs E15.5 * Sox10
10498309	Pfn2	NM_019410	0.00407073	1.28982	E15.5 * Wnt1 up vs E15.5 * Sox10
10550638	Rtn2	NM_013648	7.419e-005	1.28942	E15.5 * Wnt1 up vs E15.5 * Sox10
10386388	Snap47	NM_144521	0.00200194	1.289	E15.5 * Wnt1 up vs E15.5 * Sox10
10547410	Erc1	NM_053204	8.96776e-005	1.28888	E15.5 * Wnt1 up vs E15.5 * Sox10
10550274	Meis3	NM_008627	0.000269054	1.28831	E15.5 * Wnt1 up vs E15.5 * Sox10
10368918	Sobp	NM_175407	0.00049748	1.28802	E15.5 * Wnt1 up vs E15.5 * Sox10
10372383	Zdhhc17	BC051527	0.0039123	1.28788	E15.5 * Wnt1 up vs E15.5 * Sox10
10453373	Prepl	NM_145984	0.00246534	1.28741	E15.5 * Wnt1 up vs E15.5 * Sox10
10537851	Cntnap2	NM_001004357	0.000511975	1.28721	E15.5 * Wnt1 up vs E15.5 * Sox10
10591643	Rab3d	NM_031874	0.000195047	1.28674	E15.5 * Wnt1 up vs E15.5 * Sox10
10560139	B430211C08Rik	NM_001039146	0.000139911	1.28619	E15.5 * Wnt1 up vs E15.5 * Sox10
10491629	4932438A13Rik	NM_172679	0.000994658	1.28616	E15.5 * Wnt1 up vs E15.5 * Sox10
10402020	Eml5	NM_001081191	0.00071228	1.28597	E15.5 * Wnt1 up vs E15.5 * Sox10
10421418	Epb4.9	NM_013514	0.000170513	1.28581	E15.5 * Wnt1 up vs E15.5 * Sox10
10552508	Klk7	NM_011872	0.000203128	1.28577	E15.5 * Wnt1 up vs E15.5 * Sox10
10441422	Zdhhc14	NM_146073	0.00194935	1.28576	E15.5 * Wnt1 up vs E15.5 * Sox10
10549536	Amn1	NM_001113424	0.00222066	1.28548	E15.5 * Wnt1 up vs E15.5 * Sox10
10599435	Ocrl	NM_177215	0.000105881	1.28507	E15.5 * Wnt1 up vs E15.5 * Sox10
10357946	Ppp1r12b	NM_001081307	0.00337166	1.28505	E15.5 * Wnt1 up vs E15.5 * Sox10
10388042	6330403K07Rik	NM_134022	8.47256e-005	1.2849	E15.5 * Wnt1 up vs E15.5 * Sox10
10361509	Syne1	NM_001079686	0.000291402	1.28489	E15.5 * Wnt1 up vs E15.5 * Sox10
10527982	A330021E22Rik	NM_172447	0.00113431	1.28471	E15.5 * Wnt1 up vs E15.5 * Sox10
10379013	Flot2	NM_008028	0.00243647	1.28428	E15.5 * Wnt1 up vs E15.5 * Sox10
10532578	Myo18b	BC145218	0.00115348	1.28417	E15.5 * Wnt1 up vs E15.5 * Sox10
10372005	Vezt	NM_172538	0.00387869	1.28362	E15.5 * Wnt1 up vs E15.5 * Sox10
10607486	Ptchd1	NM_001093750	0.000111916	1.28332	E15.5 * Wnt1 up vs E15.5 * Sox10
10597323	Arpp21	NM_033264	0.000186935	1.28297	E15.5 * Wnt1 up vs E15.5 * Sox10
10421709	EG629678	NM_001037935	0.00343471	1.28243	E15.5 * Wnt1 up vs E15.5 * Sox10

10521481	Jakmip1	NM_178394	0.00230242	1.2822	E15.5 * Wnt1 up vs E15.5 * Sox10
10361381	Syne1	NM_001079686	3.66748e-005	1.2819	E15.5 * Wnt1 up vs E15.5 * Sox10
10451039	Slc25a27	NM_028711	0.00150723	1.28187	E15.5 * Wnt1 up vs E15.5 * Sox10
10377725	Dlg4	NM_007864	0.00340628	1.28186	E15.5 * Wnt1 up vs E15.5 * Sox10
10472764	Dync1i2	NM_010064	2.91895e-005	1.28152	E15.5 * Wnt1 up vs E15.5 * Sox10
10496182	Cxxc4	NM_001004367	0.00119789	1.28126	E15.5 * Wnt1 up vs E15.5 * Sox10
10477536	5430413K10Rik	BC083121	0.00104998	1.28097	E15.5 * Wnt1 up vs E15.5 * Sox10
10527920	Cyp51	NM_020010	0.00181451	1.28027	E15.5 * Wnt1 up vs E15.5 * Sox10
10484941	Madd	NM_145527	2.81954e-005	1.27969	E15.5 * Wnt1 up vs E15.5 * Sox10
10529797		---	0.00283684	1.27966	E15.5 * Wnt1 up vs E15.5 * Sox10
10491363	Mfn1	NM_024200	1.45223e-005	1.27961	E15.5 * Wnt1 up vs E15.5 * Sox10
10459671	Dcc	NM_007831	0.000481514	1.27949	E15.5 * Wnt1 up vs E15.5 * Sox10
10455098	Pcdhb14	NM_053139	0.00097538	1.27943	E15.5 * Wnt1 up vs E15.5 * Sox10
10459262	Ablim3	NM_198649	0.00372626	1.27927	E15.5 * Wnt1 up vs E15.5 * Sox10
10526261	Stx1a	NM_016801	0.000967932	1.27899	E15.5 * Wnt1 up vs E15.5 * Sox10
10443786	Pde9a	NM_008804	0.000912161	1.27844	E15.5 * Wnt1 up vs E15.5 * Sox10
10447490	Pja2	NM_001025309	1.52034e-005	1.27814	E15.5 * Wnt1 up vs E15.5 * Sox10
10491564	4932438A13Rik	NM_172679	0.00217678	1.27777	E15.5 * Wnt1 up vs E15.5 * Sox10
10552252	1600014C10Rik	NM_001085385	1.3745e-005	1.27727	E15.5 * Wnt1 up vs E15.5 * Sox10
10425726	03-Sep	NM_011889	0.000158214	1.27676	E15.5 * Wnt1 up vs E15.5 * Sox10
10496338	Ppp3ca	NM_008913	9.69618e-005	1.27625	E15.5 * Wnt1 up vs E15.5 * Sox10
10594969	Unc13c	NM_001081153	0.000199949	1.2761	E15.5 * Wnt1 up vs E15.5 * Sox10
10361186	Sertad4	NM_198247	0.000434612	1.27595	E15.5 * Wnt1 up vs E15.5 * Sox10
10475027	Tyro3	NM_019392	3.48342e-005	1.27541	E15.5 * Wnt1 up vs E15.5 * Sox10
10509238	Htr1d	NM_008309	0.00196761	1.2749	E15.5 * Wnt1 up vs E15.5 * Sox10
10538658	Herc3	NM_028705	0.00018057	1.27488	E15.5 * Wnt1 up vs E15.5 * Sox10
10524621	Oasl2	NM_011854	0.000848692	1.27415	E15.5 * Wnt1 up vs E15.5 * Sox10
10395227	Cog5	ENSMUST00000(	0.00341833	1.27388	E15.5 * Wnt1 up vs E15.5 * Sox10
10395142	Sh3yl1	NM_013709	0.00077829	1.27373	E15.5 * Wnt1 up vs E15.5 * Sox10
10591517	Cdkn2d	NM_009878	0.000432702	1.27372	E15.5 * Wnt1 up vs E15.5 * Sox10
10439239	Dirc2	NM_153550	0.0032999	1.27343	E15.5 * Wnt1 up vs E15.5 * Sox10
10440340		---	0.00478704	1.27241	E15.5 * Wnt1 up vs E15.5 * Sox10
10379654	Ap2b1	NM_001035854	1.72022e-005	1.27228	E15.5 * Wnt1 up vs E15.5 * Sox10
10564037		---	0.000387449	1.27184	E15.5 * Wnt1 up vs E15.5 * Sox10
10523021	Slc4a4	NM_018760	0.00485765	1.27153	E15.5 * Wnt1 up vs E15.5 * Sox10
10572485	Rab3a	NM_009001	0.00091087	1.27044	E15.5 * Wnt1 up vs E15.5 * Sox10
10460157	Cpt1a	NM_013495	0.00128122	1.27037	E15.5 * Wnt1 up vs E15.5 * Sox10
10533633	Diablo	NM_023232	0.000114201	1.26955	E15.5 * Wnt1 up vs E15.5 * Sox10
10572050	March1	NM_175188	0.00105294	1.2691	E15.5 * Wnt1 up vs E15.5 * Sox10
10513362	OTTMUSG00000	ENSMUST00000	0.0041729	1.26874	E15.5 * Wnt1 up vs E15.5 * Sox10
10592023	Aplp2	NM_001102455	0.000858487	1.26873	E15.5 * Wnt1 up vs E15.5 * Sox10
10359024	EG433367	ENSMUST00000(	0.00238268	1.26848	E15.5 * Wnt1 up vs E15.5 * Sox10
10495869	Tram1l1	NM_146140	0.00389413	1.26846	E15.5 * Wnt1 up vs E15.5 * Sox10
10515335	RP23-233B9.8	NM_001037916	0.00359116	1.26825	E15.5 * Wnt1 up vs E15.5 * Sox10
10463140	Lcor	NM_172154	3.93267e-005	1.26806	E15.5 * Wnt1 up vs E15.5 * Sox10
10419198	Ero1l	NM_015774	8.42438e-005	1.26711	E15.5 * Wnt1 up vs E15.5 * Sox10
10477854	Epb4.1l1	NM_013510	0.000364263	1.26577	E15.5 * Wnt1 up vs E15.5 * Sox10
10528038	Adam22	NM_001007220	0.00148516	1.26565	E15.5 * Wnt1 up vs E15.5 * Sox10
10446553	Epb4.1l3	NM_013813	6.31595e-005	1.26528	E15.5 * Wnt1 up vs E15.5 * Sox10
10418991	Gcap14	NM_027045	0.000253649	1.26506	E15.5 * Wnt1 up vs E15.5 * Sox10
10516658	Ccdc28b	NM_025455	9.31855e-005	1.26451	E15.5 * Wnt1 up vs E15.5 * Sox10
10546977	Atp2b2	NM_009723	0.000753239	1.26432	E15.5 * Wnt1 up vs E15.5 * Sox10
10384885	Spnb2	NM_175836	3.80336e-005	1.26301	E15.5 * Wnt1 up vs E15.5 * Sox10
10434418	Vwa5b2	NM_182636	0.000238999	1.26274	E15.5 * Wnt1 up vs E15.5 * Sox10
10553646	Herc2	NM_010418	1.98026e-006	1.26229	E15.5 * Wnt1 up vs E15.5 * Sox10
10469720	Acbd5	NM_001102437	0.00373513	1.26218	E15.5 * Wnt1 up vs E15.5 * Sox10
10563941		---	0.000521283	1.26141	E15.5 * Wnt1 up vs E15.5 * Sox10
10600765	Pcyt1b	NM_211138	0.000559393	1.26101	E15.5 * Wnt1 up vs E15.5 * Sox10
10391043	Krt9	NM_201255	0.00272969	1.26087	E15.5 * Wnt1 up vs E15.5 * Sox10
10404195	D130043K22Rik	NM_001081051	0.000146547	1.25935	E15.5 * Wnt1 up vs E15.5 * Sox10
10367546	Oprm1	NM_001039652	0.00191664	1.25935	E15.5 * Wnt1 up vs E15.5 * Sox10
10493449	Thbs3	NM_013691	0.000142963	1.25932	E15.5 * Wnt1 up vs E15.5 * Sox10
10356172	5033414K04Rik	BC080290	0.00459876	1.25889	E15.5 * Wnt1 up vs E15.5 * Sox10
10470529	Olfm1	NM_019498	0.000376769	1.25848	E15.5 * Wnt1 up vs E15.5 * Sox10
10606064	Rgag4	NM_183318	0.0012008	1.25782	E15.5 * Wnt1 up vs E15.5 * Sox10
10517373	Rcan3	NM_022980	2.46846e-005	1.25765	E15.5 * Wnt1 up vs E15.5 * Sox10

10424349	Sqle	NM_009270	0.00140501	1.25692	E15.5 * Wnt1 up vs E15.5 * Sox10
10607259	Tro	NM_001002272	1.80142e-006	1.25638	E15.5 * Wnt1 up vs E15.5 * Sox10
10369541	Hk1	NM_010438	0.000328622	1.25568	E15.5 * Wnt1 up vs E15.5 * Sox10
10460127	Dok6	NM_001039173	0.00260867	1.25552	E15.5 * Wnt1 up vs E15.5 * Sox10
10395293	Atxn7l1	NM_001033436	0.00227771	1.25486	E15.5 * Wnt1 up vs E15.5 * Sox10
10593196	ENSMUSG0000C	ENSMUST00000(	0.00274471	1.25448	E15.5 * Wnt1 up vs E15.5 * Sox10
10477725	Mmp24	NM_010808	0.00130377	1.25433	E15.5 * Wnt1 up vs E15.5 * Sox10
10589784	Dclk3	NM_172928	0.00308831	1.25403	E15.5 * Wnt1 up vs E15.5 * Sox10
10401068	Spnb1	NM_013675	0.00241146	1.25388	E15.5 * Wnt1 up vs E15.5 * Sox10
10380341	Spag9	NM_027569	9.18105e-005	1.25325	E15.5 * Wnt1 up vs E15.5 * Sox10
10401708	Ngb	NM_022414	0.000885871	1.25271	E15.5 * Wnt1 up vs E15.5 * Sox10
10597531	Rbms3	NM_178660	6.51003e-005	1.25266	E15.5 * Wnt1 up vs E15.5 * Sox10
10570437	Fbxo25	NM_025785	0.00458449	1.2523	E15.5 * Wnt1 up vs E15.5 * Sox10
10384423	Cobl	NM_172496	0.00451352	1.25003	E15.5 * Wnt1 up vs E15.5 * Sox10
10429222	Fam135b	NM_177819	0.00497959	1.24996	E15.5 * Wnt1 up vs E15.5 * Sox10
10482500	Rnd3	NM_028810	0.00103789	1.24924	E15.5 * Wnt1 up vs E15.5 * Sox10
10590888	EG665154	XR_031035	0.00187116	1.24902	E15.5 * Wnt1 up vs E15.5 * Sox10
10404702	Gcnt2	NM_023887	0.00277452	1.24885	E15.5 * Wnt1 up vs E15.5 * Sox10
10398350		---	0.00110835	1.24846	E15.5 * Wnt1 up vs E15.5 * Sox10
10604505	6720401G13Rik	NR_015505	0.00230611	1.24717	E15.5 * Wnt1 up vs E15.5 * Sox10
10500685	Atp1a1	NM_144900	0.00083409	1.24709	E15.5 * Wnt1 up vs E15.5 * Sox10
10533007	Ccdc64	NM_001080808	0.000328519	1.24685	E15.5 * Wnt1 up vs E15.5 * Sox10
10488033	Pak7	NM_172858	0.000455122	1.24666	E15.5 * Wnt1 up vs E15.5 * Sox10
10345141	Lmbrd1	NM_026719	0.000542046	1.24635	E15.5 * Wnt1 up vs E15.5 * Sox10
10457250	Arhgap12	NM_001039692	0.00054578	1.24615	E15.5 * Wnt1 up vs E15.5 * Sox10
10528548	Kcnh2	NM_013569	0.00104222	1.24579	E15.5 * Wnt1 up vs E15.5 * Sox10
10435752	Lsamp	NM_175548	0.00158896	1.2454	E15.5 * Wnt1 up vs E15.5 * Sox10
10509014	D4Wsu53e	BC043057	0.000744974	1.24477	E15.5 * Wnt1 up vs E15.5 * Sox10
10530215	1110003E01Rik	NM_133697	0.000931235	1.24449	E15.5 * Wnt1 up vs E15.5 * Sox10
10597162	Klhl18	NM_177771	0.000251152	1.24444	E15.5 * Wnt1 up vs E15.5 * Sox10
10549849	Zfp78	NM_001025163	0.00440158	1.24427	E15.5 * Wnt1 up vs E15.5 * Sox10
10384064	Camk2b	NM_007595	9.03864e-005	1.24213	E15.5 * Wnt1 up vs E15.5 * Sox10
10584855	Scn2b	NM_001014761	0.00232505	1.2413	E15.5 * Wnt1 up vs E15.5 * Sox10
10534168	Auts2	NM_177047	0.000280318	1.24128	E15.5 * Wnt1 up vs E15.5 * Sox10
10346843	Nrp2	NM_001077403	0.00163539	1.24099	E15.5 * Wnt1 up vs E15.5 * Sox10
10404885	Gmpr	NM_025508	0.000832947	1.24099	E15.5 * Wnt1 up vs E15.5 * Sox10
10380641	Hoxb5	NM_008268	0.000178903	1.24051	E15.5 * Wnt1 up vs E15.5 * Sox10
10376096	Acsl6	NM_001033598	0.000277973	1.23939	E15.5 * Wnt1 up vs E15.5 * Sox10
10546024	Prokr1	NM_021381	0.00414607	1.23925	E15.5 * Wnt1 up vs E15.5 * Sox10
10578138	Dctn6	NM_011722	0.00156902	1.23867	E15.5 * Wnt1 up vs E15.5 * Sox10
10589350	Shisa5	NM_025858	9.00744e-005	1.23828	E15.5 * Wnt1 up vs E15.5 * Sox10
10359235	Rasal2	NM_177644	8.67199e-005	1.23795	E15.5 * Wnt1 up vs E15.5 * Sox10
10525236	AU042671	BC051044	0.00485068	1.23787	E15.5 * Wnt1 up vs E15.5 * Sox10
10578872		---	0.00253062	1.23726	E15.5 * Wnt1 up vs E15.5 * Sox10
10374406	Cnrip1	NM_029861	0.00417458	1.23696	E15.5 * Wnt1 up vs E15.5 * Sox10
10548116	Ccnd2	BC049086	0.0016789	1.23683	E15.5 * Wnt1 up vs E15.5 * Sox10
10568024	Coro1a	NM_009898	0.00499636	1.23681	E15.5 * Wnt1 up vs E15.5 * Sox10
10533403	Cux2	NM_007804	0.000485856	1.23647	E15.5 * Wnt1 up vs E15.5 * Sox10
10490838	Fabp5	NM_010634	0.000876703	1.23588	E15.5 * Wnt1 up vs E15.5 * Sox10
10585699	Fabp5	NM_010634	0.000863893	1.2355	E15.5 * Wnt1 up vs E15.5 * Sox10
10349404	Mgat5	NM_145128	0.0018046	1.23548	E15.5 * Wnt1 up vs E15.5 * Sox10
10363599	Rufy2	NM_027425	0.00219595	1.23508	E15.5 * Wnt1 up vs E15.5 * Sox10
10590597	Sacm1l	NM_030692	0.00301256	1.23484	E15.5 * Wnt1 up vs E15.5 * Sox10
10376201	Gpx3	NM_001083929	0.00332084	1.2344	E15.5 * Wnt1 up vs E15.5 * Sox10
10547553	Mical3	NM_153396	0.00368663	1.23436	E15.5 * Wnt1 up vs E15.5 * Sox10
10410973		---	0.00370975	1.23316	E15.5 * Wnt1 up vs E15.5 * Sox10
10577240	Csmd1	NM_053171	0.00110349	1.23298	E15.5 * Wnt1 up vs E15.5 * Sox10
10399470	Trib2	NM_144551	0.00246002	1.23217	E15.5 * Wnt1 up vs E15.5 * Sox10
10414485	Olfr264	ENSMUST00000(	0.000555889	1.23141	E15.5 * Wnt1 up vs E15.5 * Sox10
10496862		---	0.00499353	1.23138	E15.5 * Wnt1 up vs E15.5 * Sox10
10592772	Abcg4	NM_138955	0.000713891	1.23127	E15.5 * Wnt1 up vs E15.5 * Sox10
10454856	Psd2	NM_028707	0.00337037	1.23127	E15.5 * Wnt1 up vs E15.5 * Sox10
10405029		---	0.00287309	1.23096	E15.5 * Wnt1 up vs E15.5 * Sox10
10477058	Scrt2	ENSMUST00000(	9.26741e-005	1.23036	E15.5 * Wnt1 up vs E15.5 * Sox10
10446756	Ypel5	NM_027166	0.00383525	1.23005	E15.5 * Wnt1 up vs E15.5 * Sox10
10578810	Clcn3	NM_173874	0.00111035	1.22978	E15.5 * Wnt1 up vs E15.5 * Sox10

10400023	Tspan13	NM_025359	0.00317687	1.22959	E15.5 * Wnt1 up vs E15.5 * Sox10
10515168	Cyp4x1	NM_001003947	0.00197211	1.2295	E15.5 * Wnt1 up vs E15.5 * Sox10
10513818	Stmn1	NM_019641	1.74304e-007	1.22928	E15.5 * Wnt1 up vs E15.5 * Sox10
10401343	Map3k9	NM_177395	0.0035815	1.22889	E15.5 * Wnt1 up vs E15.5 * Sox10
10465244	Malat1	NR_002847	0.00200484	1.22829	E15.5 * Wnt1 up vs E15.5 * Sox10
10578681		---	0.00341861	1.22822	E15.5 * Wnt1 up vs E15.5 * Sox10
10550514	Nova2	NM_001029877	0.00408675	1.22731	E15.5 * Wnt1 up vs E15.5 * Sox10
10464015	Shoc2	NM_019658	0.000140693	1.22656	E15.5 * Wnt1 up vs E15.5 * Sox10
10508986	Stmn1	NM_019641	9.20993e-008	1.22645	E15.5 * Wnt1 up vs E15.5 * Sox10
10351013	Rc3h1	NM_001024952	0.00298789	1.22632	E15.5 * Wnt1 up vs E15.5 * Sox10
10553833	Ndn	NM_010882	0.00271615	1.2259	E15.5 * Wnt1 up vs E15.5 * Sox10
10533055	1500001A10Rik	NM_026886	0.00232816	1.22558	E15.5 * Wnt1 up vs E15.5 * Sox10
10442468	Caskin1	NM_027937	0.00116703	1.22469	E15.5 * Wnt1 up vs E15.5 * Sox10
10433008	Map3k12	NM_009582	0.000962423	1.22388	E15.5 * Wnt1 up vs E15.5 * Sox10
10372028	Plxnc1	NM_018797	0.0038633	1.22331	E15.5 * Wnt1 up vs E15.5 * Sox10
10458794	Ccdc112	ENSMUST00000(	0.00106379	1.22303	E15.5 * Wnt1 up vs E15.5 * Sox10
10499470	Rusc1	NM_028188	0.00401831	1.22256	E15.5 * Wnt1 up vs E15.5 * Sox10
10382096	2310007L24Rik	ENSMUST00000(	0.00199468	1.22229	E15.5 * Wnt1 up vs E15.5 * Sox10
10596347	Atp2c1	NM_175025	0.000151465	1.22219	E15.5 * Wnt1 up vs E15.5 * Sox10
10515994	Smap2	NM_133716	0.00391821	1.22207	E15.5 * Wnt1 up vs E15.5 * Sox10
10563895		---	0.0036856	1.22203	E15.5 * Wnt1 up vs E15.5 * Sox10
10486262	Ltk	NM_203345	0.000236135	1.22179	E15.5 * Wnt1 up vs E15.5 * Sox10
10453114	Dhx57	NM_198942	0.000676718	1.22103	E15.5 * Wnt1 up vs E15.5 * Sox10
10382010	Wdr68	NM_027946	0.000316762	1.22067	E15.5 * Wnt1 up vs E15.5 * Sox10
10422980	Lmbrd2	NM_177178	0.00173742	1.21982	E15.5 * Wnt1 up vs E15.5 * Sox10
10389786	Hlf	NM_172563	0.00468672	1.21922	E15.5 * Wnt1 up vs E15.5 * Sox10
10589438	Mtap4	NM_008633	0.000992802	1.21797	E15.5 * Wnt1 up vs E15.5 * Sox10
10365601	Gnptab	NM_001004164	0.00336813	1.2172	E15.5 * Wnt1 up vs E15.5 * Sox10
10479925	5430407P10Rik	NM_001159657	0.000477875	1.216	E15.5 * Wnt1 up vs E15.5 * Sox10
10389674	Dynll2	NM_026556	6.75649e-005	1.21548	E15.5 * Wnt1 up vs E15.5 * Sox10
10464084	Tcf7l2	NM_001142918	0.000338244	1.21537	E15.5 * Wnt1 up vs E15.5 * Sox10
10529549	Tbc1d14	NM_001113362	0.00312675	1.21534	E15.5 * Wnt1 up vs E15.5 * Sox10
10465686	Rtn3	NM_001003934	0.000128943	1.21409	E15.5 * Wnt1 up vs E15.5 * Sox10
10363868	Fam13c	NM_024244	0.0021055	1.21374	E15.5 * Wnt1 up vs E15.5 * Sox10
10555414	Rab6	NM_024287	0.00358356	1.21367	E15.5 * Wnt1 up vs E15.5 * Sox10
10503019	4930566N20Rik	ENSMUST00000(	0.000939309	1.21218	E15.5 * Wnt1 up vs E15.5 * Sox10
10434302	Klhl24	NM_029436	0.00231972	1.21168	E15.5 * Wnt1 up vs E15.5 * Sox10
10578045	Nrg1	NM_178591	0.00162111	1.2116	E15.5 * Wnt1 up vs E15.5 * Sox10
10350684	Arpc5	NM_026369	0.00491622	1.21132	E15.5 * Wnt1 up vs E15.5 * Sox10
10587653	Snx14	NM_172926	0.00326045	1.21096	E15.5 * Wnt1 up vs E15.5 * Sox10
10585048	Cadm1	NM_207675	0.00105163	1.2099	E15.5 * Wnt1 up vs E15.5 * Sox10
10563911		---	0.00166924	1.2087	E15.5 * Wnt1 up vs E15.5 * Sox10
10360764	Enah	NM_010135	0.000397657	1.20832	E15.5 * Wnt1 up vs E15.5 * Sox10
10418702	Sh3bp5	NM_011894	0.000223304	1.2076	E15.5 * Wnt1 up vs E15.5 * Sox10
10407598	Ryr2	NM_023868	0.000520369	1.20681	E15.5 * Wnt1 up vs E15.5 * Sox10
10437330	Crebbp	ENSMUST00000	4.84353e-006	1.20655	E15.5 * Wnt1 up vs E15.5 * Sox10
10433717		---	0.00396156	1.20456	E15.5 * Wnt1 up vs E15.5 * Sox10
10586505	Herc1	BC004027	0.000457686	1.20455	E15.5 * Wnt1 up vs E15.5 * Sox10
10382788	Galr2	NM_010254	0.00132435	1.20408	E15.5 * Wnt1 up vs E15.5 * Sox10
10436608	Cxadr	NM_009988	0.00207453	1.20396	E15.5 * Wnt1 up vs E15.5 * Sox10
10559853	Clcn4-2	NM_011334	0.000353184	1.2029	E15.5 * Wnt1 up vs E15.5 * Sox10
10534489	Ccl26	NM_001013412	0.00114176	1.20272	E15.5 * Wnt1 up vs E15.5 * Sox10
10404380	Dusp22	NM_001037955	0.00386871	1.20194	E15.5 * Wnt1 up vs E15.5 * Sox10
10401114	Rab15	NM_134050	0.000774113	1.20146	E15.5 * Wnt1 up vs E15.5 * Sox10
10485395	Trim44	NM_020267	0.00026125	1.20108	E15.5 * Wnt1 up vs E15.5 * Sox10
10479887	Sec61a2	NM_021305	0.00300732	1.20086	E15.5 * Wnt1 up vs E15.5 * Sox10
10601235	Ogt	NM_139144	9.7781e-005	1.20084	E15.5 * Wnt1 up vs E15.5 * Sox10
10453636	Svil	NM_153153	0.0034125	1.20073	E15.5 * Wnt1 up vs E15.5 * Sox10
10545096	2410003J06Rik	NM_028025	0.00475348	1.20001	E15.5 * Wnt1 up vs E15.5 * Sox10
10400538	Trappc6b	NM_030057	0.00458691	1.1999	E15.5 * Wnt1 up vs E15.5 * Sox10
10599461	Calm2	NM_007589	0.000209465	1.19956	E15.5 * Wnt1 up vs E15.5 * Sox10
10464917	Cnih2	NM_009920	0.00293772	1.19937	E15.5 * Wnt1 up vs E15.5 * Sox10
10344637	Atp6v1h	NM_133826	0.00111476	1.19911	E15.5 * Wnt1 up vs E15.5 * Sox10
10407097	Pde4d	NM_011056	5.62027e-005	1.19812	E15.5 * Wnt1 up vs E15.5 * Sox10
10359201	Ralgps2	NM_023884	7.46558e-005	1.19786	E15.5 * Wnt1 up vs E15.5 * Sox10
10606475	Hdx	NM_001080549	0.00211143	1.19566	E15.5 * Wnt1 up vs E15.5 * Sox10

10364601	Abca7	NM_013850	0.00372439	1.19565	E15.5 * Wnt1 up vs E15.5 * Sox10
10542310	Cdkn1b	NM_009875	0.00301183	1.19527	E15.5 * Wnt1 up vs E15.5 * Sox10
10395976	Dnajb6	NM_011847	0.00386639	1.19523	E15.5 * Wnt1 up vs E15.5 * Sox10
10536827	Ccdc136	BC006583	0.00180871	1.19501	E15.5 * Wnt1 up vs E15.5 * Sox10
10354404	Dnajb6	NM_001037940	0.00412399	1.19483	E15.5 * Wnt1 up vs E15.5 * Sox10
10401244	Actn1	NM_134156	0.00314927	1.19472	E15.5 * Wnt1 up vs E15.5 * Sox10
10516103	Macf1	NM_009600	0.000119094	1.19467	E15.5 * Wnt1 up vs E15.5 * Sox10
10603492	Porcn	NM_016913	0.00283174	1.19285	E15.5 * Wnt1 up vs E15.5 * Sox10
10386636	Usp22	NM_001004143	0.00112826	1.19206	E15.5 * Wnt1 up vs E15.5 * Sox10
10386495	Tom1l2	NM_153080	0.00142876	1.19181	E15.5 * Wnt1 up vs E15.5 * Sox10
10429802	Plec1	NM_011117	0.00195845	1.19176	E15.5 * Wnt1 up vs E15.5 * Sox10
10434733	Eif4a2	NM_013506	0.00460256	1.18974	E15.5 * Wnt1 up vs E15.5 * Sox10
10398332		---	0.00226825	1.18968	E15.5 * Wnt1 up vs E15.5 * Sox10
10387014	Map2k4	NM_009157	0.00126311	1.18887	E15.5 * Wnt1 up vs E15.5 * Sox10
10455227	Rnf14	NM_020012	0.000235185	1.18869	E15.5 * Wnt1 up vs E15.5 * Sox10
10388337	Pafah1b1	NM_013625	0.000278294	1.18837	E15.5 * Wnt1 up vs E15.5 * Sox10
10417212	Itgbl1	NM_145467	0.00255842	1.18826	E15.5 * Wnt1 up vs E15.5 * Sox10
10481868	Dnajb6	NM_011847	0.0046592	1.18817	E15.5 * Wnt1 up vs E15.5 * Sox10
10594315	Fem1b	NM_010193	1.90995e-005	1.18795	E15.5 * Wnt1 up vs E15.5 * Sox10
10445781	Trem2	NM_031254	0.00281265	1.18712	E15.5 * Wnt1 up vs E15.5 * Sox10
10463704	As3mt	NM_020577	0.00381388	1.18694	E15.5 * Wnt1 up vs E15.5 * Sox10
10371188	Brunol5	NM_176954	0.00422043	1.18679	E15.5 * Wnt1 up vs E15.5 * Sox10
10578262		---	0.000124957	1.18636	E15.5 * Wnt1 up vs E15.5 * Sox10
10448765	Mapk8ip3	NM_013931	0.00205657	1.18557	E15.5 * Wnt1 up vs E15.5 * Sox10
10597377	Fbxl2	NM_178624	0.00109713	1.18511	E15.5 * Wnt1 up vs E15.5 * Sox10
10595081	Tinag	NM_012033	0.00388201	1.1842	E15.5 * Wnt1 up vs E15.5 * Sox10
10552044	Dmkn	NM_172899	0.00361967	1.18419	E15.5 * Wnt1 up vs E15.5 * Sox10
10583773	BC018242	NM_144935	0.000534084	1.18306	E15.5 * Wnt1 up vs E15.5 * Sox10
10428238	Ubr5	NM_001081359	0.00146856	1.18287	E15.5 * Wnt1 up vs E15.5 * Sox10
10352194	Cdc42bpa	BC158017	0.000815632	1.18266	E15.5 * Wnt1 up vs E15.5 * Sox10
10443764	Slc37a1	NM_153062	0.000310506	1.18238	E15.5 * Wnt1 up vs E15.5 * Sox10
10502732	Prkacb	NM_011100	0.00224829	1.18157	E15.5 * Wnt1 up vs E15.5 * Sox10
10573823	Chd9	NM_177224	0.00283594	1.18052	E15.5 * Wnt1 up vs E15.5 * Sox10
10504891	Tmeff1	NM_021436	0.00221837	1.18003	E15.5 * Wnt1 up vs E15.5 * Sox10
10462039	Trpm3	NM_001035244	0.00112118	1.17951	E15.5 * Wnt1 up vs E15.5 * Sox10
10598678	Usp9x	NM_009481	0.000440701	1.17943	E15.5 * Wnt1 up vs E15.5 * Sox10
10514255	Mllt3	NM_027326	0.000703786	1.17852	E15.5 * Wnt1 up vs E15.5 * Sox10
10368409	Lama2	NM_008481	0.00271388	1.17692	E15.5 * Wnt1 up vs E15.5 * Sox10
10517328	Tmem50a	NM_027935	0.00062626	1.17603	E15.5 * Wnt1 up vs E15.5 * Sox10
10395287	Atxn7l1	NM_028139	0.00231244	1.17531	E15.5 * Wnt1 up vs E15.5 * Sox10
10453256	Kcng3	NM_153512	0.00203095	1.17437	E15.5 * Wnt1 up vs E15.5 * Sox10
10457872	Slc39a6	NM_139143	0.00144866	1.17411	E15.5 * Wnt1 up vs E15.5 * Sox10
10604633	Cxx1b	NM_001018063	0.00134078	1.17389	E15.5 * Wnt1 up vs E15.5 * Sox10
10552632	Shank1	ENSMUST00000	0.00172553	1.17222	E15.5 * Wnt1 up vs E15.5 * Sox10
10372478	Rab21	NM_024454	0.00148262	1.17219	E15.5 * Wnt1 up vs E15.5 * Sox10
10516982	Stx12	NM_133887	0.00280013	1.17212	E15.5 * Wnt1 up vs E15.5 * Sox10
10418616	Dnahc1	BC023155	0.000727562	1.17178	E15.5 * Wnt1 up vs E15.5 * Sox10
10415262	Wdr23	NM_133734	0.00425907	1.17171	E15.5 * Wnt1 up vs E15.5 * Sox10
10478077	B230339M05Rik	NM_177658	0.00224841	1.17156	E15.5 * Wnt1 up vs E15.5 * Sox10
10362896	Cd24a	NM_009846	0.000718101	1.17144	E15.5 * Wnt1 up vs E15.5 * Sox10
10422912	2410089E03Rik	BC058107	1.58783e-005	1.17112	E15.5 * Wnt1 up vs E15.5 * Sox10
10482880	Baz2b	NM_001001182	0.000840781	1.17079	E15.5 * Wnt1 up vs E15.5 * Sox10
10374908	Rtn4	NM_194054	0.000588065	1.16861	E15.5 * Wnt1 up vs E15.5 * Sox10
10427895	Basp1	NM_027395	4.46965e-005	1.16765	E15.5 * Wnt1 up vs E15.5 * Sox10
10472155	Kcnj3	NM_008426	0.00450238	1.16676	E15.5 * Wnt1 up vs E15.5 * Sox10
10504094	Galt	NM_016658	0.00203587	1.16637	E15.5 * Wnt1 up vs E15.5 * Sox10
10437080	Ttc3	NM_009441	8.07638e-005	1.16609	E15.5 * Wnt1 up vs E15.5 * Sox10
10573128	Tbc1d9	NM_001111304	0.000252572	1.16556	E15.5 * Wnt1 up vs E15.5 * Sox10
10594110	Neo1	NM_008684	0.00055013	1.1655	E15.5 * Wnt1 up vs E15.5 * Sox10
10373396	EG667952	ENSMUST00000(	0.00388624	1.16321	E15.5 * Wnt1 up vs E15.5 * Sox10
10429949	Rpl29	NM_009082	0.00473938	1.16294	E15.5 * Wnt1 up vs E15.5 * Sox10
10471953	Acvr2a	NM_007396	0.00113152	1.16275	E15.5 * Wnt1 up vs E15.5 * Sox10
10597933		---	0.000845901	1.16264	E15.5 * Wnt1 up vs E15.5 * Sox10
10604637	Cxx1b	NM_001018063	0.00212009	1.16126	E15.5 * Wnt1 up vs E15.5 * Sox10
10406551	Ssbp2	NM_024272	0.00226747	1.16109	E15.5 * Wnt1 up vs E15.5 * Sox10
10475625	Eid1	NM_025613	0.000850917	1.15982	E15.5 * Wnt1 up vs E15.5 * Sox10

10437992	Dnm1l	NM_152816	0.00381404	1.15921	E15.5 * Wnt1 up vs E15.5 * Sox10
10360506	Akt3	NM_011785	0.00358476	1.15885	E15.5 * Wnt1 up vs E15.5 * Sox10
10588195	9630041A04Rik	ENSMUST00000	0.00343423	1.15847	E15.5 * Wnt1 up vs E15.5 * Sox10
10362149	Taar2	NM_001007266	0.000567135	1.15768	E15.5 * Wnt1 up vs E15.5 * Sox10
10397752	Calm1	NM_009790	2.14902e-005	1.1575	E15.5 * Wnt1 up vs E15.5 * Sox10
10417895	Ppp3cb	NM_008914	0.00318694	1.15684	E15.5 * Wnt1 up vs E15.5 * Sox10
10376033	Kif3a	NM_008443	0.00197175	1.15374	E15.5 * Wnt1 up vs E15.5 * Sox10
10514668	Jak1	NM_146145	0.000425256	1.15233	E15.5 * Wnt1 up vs E15.5 * Sox10
10358191	Camsap1l1	NM_001081360	0.000782226	1.15232	E15.5 * Wnt1 up vs E15.5 * Sox10
10590690	Dync2h1	NM_029851	0.00407004	1.15087	E15.5 * Wnt1 up vs E15.5 * Sox10
10515051	Osbpl9	NM_133885	0.00265854	1.15077	E15.5 * Wnt1 up vs E15.5 * Sox10
10601778	Armcx3	NM_027870	0.00325444	1.15036	E15.5 * Wnt1 up vs E15.5 * Sox10
10454286	Mapre2	NM_153058	0.0042736	1.14964	E15.5 * Wnt1 up vs E15.5 * Sox10
10563961		---	0.00102948	1.14885	E15.5 * Wnt1 up vs E15.5 * Sox10
10551009	Tmsb10	NM_025284	5.14271e-005	1.14858	E15.5 * Wnt1 up vs E15.5 * Sox10
10494413	Rnu1b1	NR_004412	0.00343471	1.14809	E15.5 * Wnt1 up vs E15.5 * Sox10
10494421	Rnu1b1	NR_004412	0.00343471	1.14809	E15.5 * Wnt1 up vs E15.5 * Sox10
10500343	Rnu1b1	NR_004412	0.00343471	1.14809	E15.5 * Wnt1 up vs E15.5 * Sox10
10500358	Rnu1b1	NR_004412	0.00343471	1.14809	E15.5 * Wnt1 up vs E15.5 * Sox10
10512937	Rnu1b1	NR_004412	0.00343471	1.14809	E15.5 * Wnt1 up vs E15.5 * Sox10
10455238	Ndfip1	NM_022996	0.00324568	1.14755	E15.5 * Wnt1 up vs E15.5 * Sox10
10582551	Dbndd1	NM_028146	0.00016579	1.1465	E15.5 * Wnt1 up vs E15.5 * Sox10
10367224	Stat2	NM_019963	0.00260344	1.14326	E15.5 * Wnt1 up vs E15.5 * Sox10
10553115	Lmtk3	NM_001005511	0.00292125	1.14201	E15.5 * Wnt1 up vs E15.5 * Sox10
10551417	Zfp59	NM_011762	0.000394426	1.14145	E15.5 * Wnt1 up vs E15.5 * Sox10
10391378	Ezh1	NM_007970	0.00133573	1.14084	E15.5 * Wnt1 up vs E15.5 * Sox10
10366004	Atp2b1	NM_026482	4.19982e-005	1.14068	E15.5 * Wnt1 up vs E15.5 * Sox10
10587107	Myo5a	NM_010864	0.00234996	1.13982	E15.5 * Wnt1 up vs E15.5 * Sox10
10448081	Rgmb	NM_178615	0.00283956	1.13797	E15.5 * Wnt1 up vs E15.5 * Sox10
10474411	Lin7c	NM_011699	0.00020715	1.13779	E15.5 * Wnt1 up vs E15.5 * Sox10
10407766	Lgals8	NM_018886	0.00368641	1.13643	E15.5 * Wnt1 up vs E15.5 * Sox10
10549923	Vmn2r48	NM_001105152	0.00274265	1.13633	E15.5 * Wnt1 up vs E15.5 * Sox10
10453451	Calm2	NM_007589	9.90521e-005	1.13604	E15.5 * Wnt1 up vs E15.5 * Sox10
10555425	Fam168a	BC079886	0.00289131	1.13561	E15.5 * Wnt1 up vs E15.5 * Sox10
10468089	Mgea5	NM_023799	0.00112766	1.13555	E15.5 * Wnt1 up vs E15.5 * Sox10
10389882	3300001P08Rik	NM_026313	0.000157948	1.13361	E15.5 * Wnt1 up vs E15.5 * Sox10
10396926	Sfrs5	NM_001079695	0.00352577	1.13357	E15.5 * Wnt1 up vs E15.5 * Sox10
10475879	Gm355	ENSMUST00000	0.00321977	1.13153	E15.5 * Wnt1 up vs E15.5 * Sox10
10545479	Tmsb10	NM_025284	1.54589e-005	1.13141	E15.5 * Wnt1 up vs E15.5 * Sox10
10503917	Akirin2	NM_001007589	0.000767121	1.13099	E15.5 * Wnt1 up vs E15.5 * Sox10
10463153	Morf4l1	NM_024431	0.00243842	1.13077	E15.5 * Wnt1 up vs E15.5 * Sox10
10478698	Eya2	NM_010165	0.00173845	1.13018	E15.5 * Wnt1 up vs E15.5 * Sox10
10592847	EG667952	ENSMUST00000	0.00105325	1.12917	E15.5 * Wnt1 up vs E15.5 * Sox10
10377537	Chd3	NM_146019	0.00117535	1.12802	E15.5 * Wnt1 up vs E15.5 * Sox10
10354372	EG667952	ENSMUST00000	0.00304233	1.1273	E15.5 * Wnt1 up vs E15.5 * Sox10
10442134	Vmn2r107	NM_001104569	0.00253833	1.12685	E15.5 * Wnt1 up vs E15.5 * Sox10
10482181	Strbp	NM_009261	0.00368459	1.12539	E15.5 * Wnt1 up vs E15.5 * Sox10
10577882	Hgsnat	NM_029884	0.00220767	1.12473	E15.5 * Wnt1 up vs E15.5 * Sox10
10427807	Sub1	NM_011294	5.72892e-005	1.12366	E15.5 * Wnt1 up vs E15.5 * Sox10
10598020		---	0.000278298	1.12205	E15.5 * Wnt1 up vs E15.5 * Sox10
10413951	Arhgap22	NM_153800	0.00353872	1.12064	E15.5 * Wnt1 up vs E15.5 * Sox10
10355069	Ino80d	NM_001114609	0.000877373	1.12034	E15.5 * Wnt1 up vs E15.5 * Sox10
10598036	COX1	ENSMUST00000	0.00462284	1.12008	E15.5 * Wnt1 up vs E15.5 * Sox10
10457888	5730494M16Rik	NM_001004361	0.00283385	1.11952	E15.5 * Wnt1 up vs E15.5 * Sox10
10438530	Clcn2	NM_009900	0.00232355	1.11809	E15.5 * Wnt1 up vs E15.5 * Sox10
10508490	Sfrs5	NM_001079695	0.00372619	1.11763	E15.5 * Wnt1 up vs E15.5 * Sox10
10607865	Tmsb4x	NM_021278	0.00255503	1.11284	E15.5 * Wnt1 up vs E15.5 * Sox10
10386743	Epn2	NM_010148	0.00256417	1.11093	E15.5 * Wnt1 up vs E15.5 * Sox10
10421488	Fndc3a	NM_207636	0.00389425	1.11075	E15.5 * Wnt1 up vs E15.5 * Sox10
10414313	Ubb	NM_011664	1.58264e-006	1.11065	E15.5 * Wnt1 up vs E15.5 * Sox10
10376864	Ubb	NM_011664	2.6099e-006	1.11044	E15.5 * Wnt1 up vs E15.5 * Sox10
10412011	Kif2a	NM_008442	0.000122655	1.10895	E15.5 * Wnt1 up vs E15.5 * Sox10
10473541	Olfr1125	ENSMUST00000	0.00254639	1.10706	E15.5 * Wnt1 up vs E15.5 * Sox10
10345037	Paqr8	NM_028829	0.00412908	1.10657	E15.5 * Wnt1 up vs E15.5 * Sox10
10598025		---	0.00266888	1.10183	E15.5 * Wnt1 up vs E15.5 * Sox10
10465649	Mark2	NM_007928	0.000395073	1.09623	E15.5 * Wnt1 up vs E15.5 * Sox10

10384522	Actr2	NM_146243	0.00209852	1.09576	E15.5 * Wnt1 up vs E15.5 * Sox10
10596769	Rbm5	NM_148930	0.00352288	1.09174	E15.5 * Wnt1 up vs E15.5 * Sox10
10503134	Sdcbp	NM_001098227	0.00144985	1.08844	E15.5 * Wnt1 up vs E15.5 * Sox10
10417601	Ptma	NM_008972	0.00332433	1.08686	E15.5 * Wnt1 up vs E15.5 * Sox10
10348078	Ptma	NM_008972	0.00141805	1.08498	E15.5 * Wnt1 up vs E15.5 * Sox10
10411776	H3f3a	NM_008210	0.000178954	1.08458	E15.5 * Wnt1 up vs E15.5 * Sox10
10598069	CYTB	ENSMUST00000	0.00152292	1.07921	E15.5 * Wnt1 up vs E15.5 * Sox10
10503150	Rab2a	NM_021518	0.00337501	1.06928	E15.5 * Wnt1 up vs E15.5 * Sox10
10594142	Rpl11	NM_025919	0.000180429	-1.05211	E15.5 * Wnt1 down vs E15.5 * Sox10
10450063	Rps18	NM_011296	0.00162833	-1.05244	E15.5 * Wnt1 down vs E15.5 * Sox10
10451301	Rpl11	NM_025919	0.0007205	-1.05375	E15.5 * Wnt1 down vs E15.5 * Sox10
10502745	Rpl11	NM_025919	0.000725362	-1.05392	E15.5 * Wnt1 down vs E15.5 * Sox10
10517457	Rpl11	NM_025919	0.000260522	-1.05436	E15.5 * Wnt1 down vs E15.5 * Sox10
10404354	Rps18	NM_011296	0.00147993	-1.05657	E15.5 * Wnt1 down vs E15.5 * Sox10
10581535	Rps18	NM_011296	0.000681621	-1.05704	E15.5 * Wnt1 down vs E15.5 * Sox10
10410506	Rpl9	NM_011292	0.00193919	-1.05726	E15.5 * Wnt1 down vs E15.5 * Sox10
10543464	Rpl7a	NM_013721	0.00496631	-1.05778	E15.5 * Wnt1 down vs E15.5 * Sox10
10407535	Rpl10a	NM_011287	0.004323	-1.0583	E15.5 * Wnt1 down vs E15.5 * Sox10
10530194	Rpl9	NM_011292	0.00232915	-1.05917	E15.5 * Wnt1 down vs E15.5 * Sox10
10454097	Rpl11	NM_025919	0.000333555	-1.06042	E15.5 * Wnt1 down vs E15.5 * Sox10
10586157	Rpl4	NM_024212	0.00109278	-1.06303	E15.5 * Wnt1 down vs E15.5 * Sox10
10428554	Eif3h	NM_080635	0.00146743	-1.06479	E15.5 * Wnt1 down vs E15.5 * Sox10
10491730	Rps23	NM_024175	0.00456187	-1.06701	E15.5 * Wnt1 down vs E15.5 * Sox10
10439402	Rpl9	NM_011292	0.00254515	-1.06849	E15.5 * Wnt1 down vs E15.5 * Sox10
10546054	Rpl3	NM_013762	0.00345469	-1.06893	E15.5 * Wnt1 down vs E15.5 * Sox10
10550052	Rps5	NM_009095	1.92921e-005	-1.06955	E15.5 * Wnt1 down vs E15.5 * Sox10
10490370	Psma7	NM_011969	0.0033052	-1.07209	E15.5 * Wnt1 down vs E15.5 * Sox10
10355173	Rpl10a	NM_011287	0.000568182	-1.07214	E15.5 * Wnt1 down vs E15.5 * Sox10
10405890	Rpl9	NM_011292	0.000893802	-1.07249	E15.5 * Wnt1 down vs E15.5 * Sox10
10496032	Rpl12	NM_009076	0.00204954	-1.07571	E15.5 * Wnt1 down vs E15.5 * Sox10
10443360	Rpl10a	NM_011287	0.000961512	-1.07673	E15.5 * Wnt1 down vs E15.5 * Sox10
10567020	Rpl7a	BC091731	0.00197776	-1.0786	E15.5 * Wnt1 down vs E15.5 * Sox10
10368504	Rpl12	NM_009076	0.00294694	-1.07933	E15.5 * Wnt1 down vs E15.5 * Sox10
10560103	Rps8	NM_009098	0.000623537	-1.08147	E15.5 * Wnt1 down vs E15.5 * Sox10
10528544	Rpl11	NM_025919	0.00270704	-1.08193	E15.5 * Wnt1 down vs E15.5 * Sox10
10411393	Rps18	NM_011296	8.86352e-005	-1.08434	E15.5 * Wnt1 down vs E15.5 * Sox10
10430669	Rpl3	NM_013762	5.25904e-005	-1.08963	E15.5 * Wnt1 down vs E15.5 * Sox10
10411363	5730427N09Rik	NM_021552	0.000233486	-1.08966	E15.5 * Wnt1 down vs E15.5 * Sox10
10598721	Rpl3	NM_013762	3.54482e-005	-1.09078	E15.5 * Wnt1 down vs E15.5 * Sox10
10502165	Sec24b	NM_207209	0.00408912	-1.09219	E15.5 * Wnt1 down vs E15.5 * Sox10
10406541	Rps23	NM_024175	0.00137344	-1.09261	E15.5 * Wnt1 down vs E15.5 * Sox10
10459705	Smad4	NM_008540	0.00418298	-1.09443	E15.5 * Wnt1 down vs E15.5 * Sox10
10371981	Ccdc38	NM_175488	0.000176717	-1.09575	E15.5 * Wnt1 down vs E15.5 * Sox10
10348004	Psmd1	NM_027357	0.00302281	-1.09583	E15.5 * Wnt1 down vs E15.5 * Sox10
10404262	Rps8	NM_009098	2.59148e-005	-1.09694	E15.5 * Wnt1 down vs E15.5 * Sox10
10396237	2700049A03Rik	BC150744	0.00242146	-1.0987	E15.5 * Wnt1 down vs E15.5 * Sox10
10462081	5730427N09Rik	NM_021552	0.00239781	-1.09969	E15.5 * Wnt1 down vs E15.5 * Sox10
10535927	Nme2	NM_008705	0.002138	-1.09977	E15.5 * Wnt1 down vs E15.5 * Sox10
10525657	Denr	NM_026603	0.00500175	-1.10002	E15.5 * Wnt1 down vs E15.5 * Sox10
10474485	Olfr1280	NM_146908	0.00360502	-1.1006	E15.5 * Wnt1 down vs E15.5 * Sox10
10572301	Mef2b	NM_008578	0.00282956	-1.10101	E15.5 * Wnt1 down vs E15.5 * Sox10
10601390	Pgk1	NM_008828	0.00239189	-1.10136	E15.5 * Wnt1 down vs E15.5 * Sox10
10373709	Eif4enif1	NM_023743	0.00272686	-1.10305	E15.5 * Wnt1 down vs E15.5 * Sox10
10607388	Rpl7a	NM_013721	0.00317845	-1.10312	E15.5 * Wnt1 down vs E15.5 * Sox10
10399379	Pgk1	NM_008828	0.00175641	-1.10314	E15.5 * Wnt1 down vs E15.5 * Sox10
10498313	Pgk1	NM_008828	0.00175641	-1.10314	E15.5 * Wnt1 down vs E15.5 * Sox10
10594752	Rpl7a	NM_013721	1.43077e-005	-1.10325	E15.5 * Wnt1 down vs E15.5 * Sox10
10549714	Rpl28	NM_009081	0.00258848	-1.1047	E15.5 * Wnt1 down vs E15.5 * Sox10
10531133	Grsf1	NM_178700	0.00207284	-1.10593	E15.5 * Wnt1 down vs E15.5 * Sox10
10543650	Tnpo3	NM_177296	0.00268334	-1.10643	E15.5 * Wnt1 down vs E15.5 * Sox10
10487513	Anapc1	NM_008569	0.00107128	-1.10697	E15.5 * Wnt1 down vs E15.5 * Sox10
10522716	Polr2b	NM_153798	0.00162172	-1.10708	E15.5 * Wnt1 down vs E15.5 * Sox10
10380732	Mrpl10	NM_026154	0.00173495	-1.10767	E15.5 * Wnt1 down vs E15.5 * Sox10
10394609	Rpl36	NM_018730	0.00128092	-1.10786	E15.5 * Wnt1 down vs E15.5 * Sox10
10432162	2310037I24Rik	BC003301	0.00209651	-1.10839	E15.5 * Wnt1 down vs E15.5 * Sox10
10378783	Rpl36	NM_018730	0.000619757	-1.10864	E15.5 * Wnt1 down vs E15.5 * Sox10

10515383	Rpl36	NM_018730	0.000928886	-1.11049	E15.5 * Wnt1 down vs E15.5 * Sox10
10594946	Rpl36	NM_018730	0.000479098	-1.11119	E15.5 * Wnt1 down vs E15.5 * Sox10
10428192	Pabpc1	NM_008774	0.00189469	-1.11266	E15.5 * Wnt1 down vs E15.5 * Sox10
10478196	Top1	NM_009408	0.00241279	-1.11308	E15.5 * Wnt1 down vs E15.5 * Sox10
10471358	Rpl36	NM_018730	0.000573806	-1.11322	E15.5 * Wnt1 down vs E15.5 * Sox10
10382139	Psmd12	NM_025894	0.000335386	-1.11538	E15.5 * Wnt1 down vs E15.5 * Sox10
10603011	Rbbp7	NM_009031	9.51576e-005	-1.11732	E15.5 * Wnt1 down vs E15.5 * Sox10
10501649	Rtcd1	NM_025517	0.00345605	-1.11754	E15.5 * Wnt1 down vs E15.5 * Sox10
10446131	Rpl36	NM_018730	0.000339944	-1.11829	E15.5 * Wnt1 down vs E15.5 * Sox10
10574033	Nup93	NM_172410	0.00065296	-1.11858	E15.5 * Wnt1 down vs E15.5 * Sox10
10426648	Rpl36	NM_018730	0.000164245	-1.11885	E15.5 * Wnt1 down vs E15.5 * Sox10
10463826	6330577E15Rik	NM_026377	0.00130399	-1.11889	E15.5 * Wnt1 down vs E15.5 * Sox10
10452658	Smchd1	NM_028887	0.00497737	-1.11986	E15.5 * Wnt1 down vs E15.5 * Sox10
10592138	Rpl36	NM_018730	0.00113745	-1.11987	E15.5 * Wnt1 down vs E15.5 * Sox10
10546801	Rpl36	NM_018730	9.44617e-005	-1.12003	E15.5 * Wnt1 down vs E15.5 * Sox10
10515154	Rpl3	NM_013762	5.05368e-005	-1.12011	E15.5 * Wnt1 down vs E15.5 * Sox10
10466342	Olfr1453	NM_146700	0.00299934	-1.12037	E15.5 * Wnt1 down vs E15.5 * Sox10
10429657	Eef1d	NM_029663	0.00147231	-1.12043	E15.5 * Wnt1 down vs E15.5 * Sox10
10454296	Rpl19	NM_009078	0.00101349	-1.12123	E15.5 * Wnt1 down vs E15.5 * Sox10
10433073	ENSMUSG0000C	ENSMUST00000	0.00295861	-1.12125	E15.5 * Wnt1 down vs E15.5 * Sox10
10401805	Snw1	NM_025507	0.00474411	-1.12343	E15.5 * Wnt1 down vs E15.5 * Sox10
10377851	Psmb6	NM_008946	0.00316996	-1.12373	E15.5 * Wnt1 down vs E15.5 * Sox10
10526319	Baz1b	NM_011714	0.000276276	-1.12423	E15.5 * Wnt1 down vs E15.5 * Sox10
10490544	Ythdf1	NM_173761	0.00109395	-1.12491	E15.5 * Wnt1 down vs E15.5 * Sox10
10395163	Lamb1-1	NM_008482	0.00472044	-1.12496	E15.5 * Wnt1 down vs E15.5 * Sox10
10504047	Ube2r2	NM_026275	0.00486537	-1.12545	E15.5 * Wnt1 down vs E15.5 * Sox10
10441987	Chd1	NM_007690	0.00213142	-1.12554	E15.5 * Wnt1 down vs E15.5 * Sox10
10374564	Cct4	NM_009837	0.00387239	-1.12587	E15.5 * Wnt1 down vs E15.5 * Sox10
10578391	Smarce1	NM_020618	0.00469874	-1.12645	E15.5 * Wnt1 down vs E15.5 * Sox10
10564343	Tjp1	NM_009386	0.000962811	-1.12718	E15.5 * Wnt1 down vs E15.5 * Sox10
10422321	Dzip1	NM_025943	0.000873756	-1.12774	E15.5 * Wnt1 down vs E15.5 * Sox10
10399430	Ddx1	NM_134040	0.00339147	-1.12797	E15.5 * Wnt1 down vs E15.5 * Sox10
10503723	Mdn1	NM_001081392	0.00121536	-1.12816	E15.5 * Wnt1 down vs E15.5 * Sox10
10401519	Npc2	NM_023409	0.0049936	-1.12909	E15.5 * Wnt1 down vs E15.5 * Sox10
10566097	Nup98	NM_022979	0.00436884	-1.1292	E15.5 * Wnt1 down vs E15.5 * Sox10
10401637	Nek9	NM_145138	0.000643279	-1.12941	E15.5 * Wnt1 down vs E15.5 * Sox10
10487629	Idh3b	NM_130884	0.00303487	-1.1305	E15.5 * Wnt1 down vs E15.5 * Sox10
10496475	Adh5	NM_007410	0.000122623	-1.13218	E15.5 * Wnt1 down vs E15.5 * Sox10
10556764	Ppp1cc	NM_013636	0.000261534	-1.13229	E15.5 * Wnt1 down vs E15.5 * Sox10
10499879	Rpl3	NM_013762	0.00174991	-1.13605	E15.5 * Wnt1 down vs E15.5 * Sox10
10499881	Rpl3	NM_013762	0.00174991	-1.13605	E15.5 * Wnt1 down vs E15.5 * Sox10
10570625	Rpl19	NM_009078	0.00014207	-1.13701	E15.5 * Wnt1 down vs E15.5 * Sox10
10371877	Slc25a3	NM_133668	0.000473819	-1.13767	E15.5 * Wnt1 down vs E15.5 * Sox10
10422852	Rpl19	NM_009078	0.000130349	-1.13772	E15.5 * Wnt1 down vs E15.5 * Sox10
10492671	Ppid	NM_026352	0.00462713	-1.13776	E15.5 * Wnt1 down vs E15.5 * Sox10
10552740	Nup62	NM_053074	0.00120479	-1.13779	E15.5 * Wnt1 down vs E15.5 * Sox10
10380833	Rpl19	NM_009078	0.00015244	-1.13824	E15.5 * Wnt1 down vs E15.5 * Sox10
10421456	Xpo7	NM_023045	0.000446546	-1.13838	E15.5 * Wnt1 down vs E15.5 * Sox10
10405971	Nsun2	NM_145354	0.00184335	-1.13867	E15.5 * Wnt1 down vs E15.5 * Sox10
10370127	Pcnt	NM_008787	0.00188034	-1.13963	E15.5 * Wnt1 down vs E15.5 * Sox10
10385159	Rars	NM_025936	1.09749e-005	-1.14057	E15.5 * Wnt1 down vs E15.5 * Sox10
10486522	Zfp106	NM_011743	0.00286507	-1.14093	E15.5 * Wnt1 down vs E15.5 * Sox10
10512111	Smu1	NM_021535	0.00153075	-1.14106	E15.5 * Wnt1 down vs E15.5 * Sox10
10388010	C1qbp	NM_007573	0.000110147	-1.14115	E15.5 * Wnt1 down vs E15.5 * Sox10
10496737	Bcl10	NM_009740	0.00451883	-1.14141	E15.5 * Wnt1 down vs E15.5 * Sox10
10428912		---	0.000469619	-1.14148	E15.5 * Wnt1 down vs E15.5 * Sox10
10525352	Ppp1cc	NM_013636	9.26181e-005	-1.14165	E15.5 * Wnt1 down vs E15.5 * Sox10
10566662	Olfr469	NM_146426	0.00107636	-1.14184	E15.5 * Wnt1 down vs E15.5 * Sox10
10366301	Rpl6	NM_011290	4.14199e-005	-1.14211	E15.5 * Wnt1 down vs E15.5 * Sox10
10522596	Tmem165	NM_011626	0.000749294	-1.1422	E15.5 * Wnt1 down vs E15.5 * Sox10
10378579	Prpf8	NM_138659	0.00331061	-1.14238	E15.5 * Wnt1 down vs E15.5 * Sox10
10454469	Ammecr1l	NM_153515	0.00062887	-1.14277	E15.5 * Wnt1 down vs E15.5 * Sox10
10408684	Tmed10	NM_026775	2.98334e-006	-1.14295	E15.5 * Wnt1 down vs E15.5 * Sox10
10345882	Mrps9	NM_023514	0.0018976	-1.143	E15.5 * Wnt1 down vs E15.5 * Sox10
10528340	Dnajc2	NM_009584	0.00449241	-1.14309	E15.5 * Wnt1 down vs E15.5 * Sox10
10383671	Drg1	NM_007879	0.00332746	-1.1434	E15.5 * Wnt1 down vs E15.5 * Sox10

10398195	Ccnk	NM_009832	0.00330914	-1.14351	E15.5 * Wnt1 down vs E15.5 * Sox10
10360225	Timm23	NM_016897	0.00147363	-1.14391	E15.5 * Wnt1 down vs E15.5 * Sox10
10416069	Timm23	NM_016897	0.00147363	-1.14391	E15.5 * Wnt1 down vs E15.5 * Sox10
10375125	Ssbp1	NM_212468	0.00201575	-1.14417	E15.5 * Wnt1 down vs E15.5 * Sox10
10407543	Gtpbp4	NM_027000	0.00426589	-1.14433	E15.5 * Wnt1 down vs E15.5 * Sox10
10409557	H2afy	NM_012015	0.00108981	-1.14467	E15.5 * Wnt1 down vs E15.5 * Sox10
10494306	Mcl1	NM_008562	0.00341745	-1.14496	E15.5 * Wnt1 down vs E15.5 * Sox10
10590892	Cep57	NM_026665	0.00151693	-1.14543	E15.5 * Wnt1 down vs E15.5 * Sox10
10554045	Adamts17	NM_001033877	0.00278449	-1.14563	E15.5 * Wnt1 down vs E15.5 * Sox10
10397818	Cpsf2	NM_016856	0.000329914	-1.14584	E15.5 * Wnt1 down vs E15.5 * Sox10
10498837	Etfdh	NM_025794	0.000717425	-1.14586	E15.5 * Wnt1 down vs E15.5 * Sox10
10477946	Rpn2	NM_019642	0.00262452	-1.14689	E15.5 * Wnt1 down vs E15.5 * Sox10
10578763	Sap30	NM_021788	0.0026975	-1.14704	E15.5 * Wnt1 down vs E15.5 * Sox10
10600324	Rpl3	NM_013762	7.82355e-006	-1.1473	E15.5 * Wnt1 down vs E15.5 * Sox10
10498519	Ssr3	NM_026155	0.00181907	-1.14781	E15.5 * Wnt1 down vs E15.5 * Sox10
10507644	Zmynd12	NM_001014900	0.00355462	-1.14818	E15.5 * Wnt1 down vs E15.5 * Sox10
10455801	Phax	NM_019996	0.00344843	-1.14827	E15.5 * Wnt1 down vs E15.5 * Sox10
10347917	Rpl19	NM_009078	0.000429871	-1.14827	E15.5 * Wnt1 down vs E15.5 * Sox10
10461108	Iscu	NM_025526	0.0035087	-1.14853	E15.5 * Wnt1 down vs E15.5 * Sox10
10555764	Olfr585	NM_147087	0.00359238	-1.14886	E15.5 * Wnt1 down vs E15.5 * Sox10
10536818	Calu	NM_184053	0.00309783	-1.14908	E15.5 * Wnt1 down vs E15.5 * Sox10
10439766	Pvrl3	NM_021496	0.000965078	-1.14926	E15.5 * Wnt1 down vs E15.5 * Sox10
10393559	Timp2	NM_011594	0.00143183	-1.14966	E15.5 * Wnt1 down vs E15.5 * Sox10
10406626	Homer1	NM_147176	0.00333853	-1.1503	E15.5 * Wnt1 down vs E15.5 * Sox10
10477325	Dnmt3b	NM_001003961	0.00455989	-1.1508	E15.5 * Wnt1 down vs E15.5 * Sox10
10567941	Eif3c	NM_146200	0.000133962	-1.15176	E15.5 * Wnt1 down vs E15.5 * Sox10
10370983	Plekhj1	NM_023900	0.00306291	-1.15259	E15.5 * Wnt1 down vs E15.5 * Sox10
10414497	Parp2	NM_009632	0.00479523	-1.15292	E15.5 * Wnt1 down vs E15.5 * Sox10
10400795	Sav1	NM_022028	0.00085266	-1.15296	E15.5 * Wnt1 down vs E15.5 * Sox10
10493394	Clk2	NM_007712	0.000282801	-1.15299	E15.5 * Wnt1 down vs E15.5 * Sox10
10415065	Lrp10	NM_022993	0.00239173	-1.15334	E15.5 * Wnt1 down vs E15.5 * Sox10
10437737	Rsl1d1	NM_025546	0.000671636	-1.15366	E15.5 * Wnt1 down vs E15.5 * Sox10
10530163	Rfc1	NM_011258	0.000603318	-1.15413	E15.5 * Wnt1 down vs E15.5 * Sox10
10499425	2700060E02Rik	NM_026528	8.93472e-005	-1.15448	E15.5 * Wnt1 down vs E15.5 * Sox10
10522668	Paics	NM_025939	0.00231878	-1.15453	E15.5 * Wnt1 down vs E15.5 * Sox10
10587211	Leo1	NM_001039522	0.00200398	-1.15479	E15.5 * Wnt1 down vs E15.5 * Sox10
10414767	Rps19	NM_023133	0.00037858	-1.15523	E15.5 * Wnt1 down vs E15.5 * Sox10
10414874	Rps19	NM_023133	0.00037858	-1.15523	E15.5 * Wnt1 down vs E15.5 * Sox10
10450103	H2-Ke6	NM_013543	0.00436956	-1.15613	E15.5 * Wnt1 down vs E15.5 * Sox10
10522676	Srp72	NM_025691	0.00354443	-1.15619	E15.5 * Wnt1 down vs E15.5 * Sox10
10455989	Rbm22	NM_025776	0.00299318	-1.15631	E15.5 * Wnt1 down vs E15.5 * Sox10
10503109	Chchd7	NM_181391	0.00145117	-1.15681	E15.5 * Wnt1 down vs E15.5 * Sox10
10572345	Homer3	NM_001146153	0.00404122	-1.15715	E15.5 * Wnt1 down vs E15.5 * Sox10
10369647	Ddx50	NM_053183	0.00415488	-1.15772	E15.5 * Wnt1 down vs E15.5 * Sox10
10428685		---	0.0011798	-1.15854	E15.5 * Wnt1 down vs E15.5 * Sox10
10593789	Etfa	NM_145615	0.000155131	-1.15869	E15.5 * Wnt1 down vs E15.5 * Sox10
10478447	Stk4	NM_021420	0.00112013	-1.15884	E15.5 * Wnt1 down vs E15.5 * Sox10
10520800	Gpn1	NM_133756	0.00311988	-1.15917	E15.5 * Wnt1 down vs E15.5 * Sox10
10505674	Cntln	NM_175275	0.000980484	-1.15971	E15.5 * Wnt1 down vs E15.5 * Sox10
10386171	Ndufs3	NM_026688	0.00297779	-1.15993	E15.5 * Wnt1 down vs E15.5 * Sox10
10428052	Cct5	NM_007637	1.11527e-006	-1.16022	E15.5 * Wnt1 down vs E15.5 * Sox10
10350838	2810417H13Rik	NM_026515	0.00215148	-1.16056	E15.5 * Wnt1 down vs E15.5 * Sox10
10469322	Vim	NM_011701	0.000459635	-1.16071	E15.5 * Wnt1 down vs E15.5 * Sox10
10360270	Atp1a2	NM_178405	0.00320738	-1.16131	E15.5 * Wnt1 down vs E15.5 * Sox10
10515337	Nasp	NM_016777	7.94078e-005	-1.16193	E15.5 * Wnt1 down vs E15.5 * Sox10
10549341	4933424B01Rik	NM_138757	0.000261239	-1.16294	E15.5 * Wnt1 down vs E15.5 * Sox10
10459375	Txnl1	NM_016792	0.000924745	-1.16317	E15.5 * Wnt1 down vs E15.5 * Sox10
10419611	Supt16h	NM_033618	0.000846673	-1.16401	E15.5 * Wnt1 down vs E15.5 * Sox10
10538927	Amd1	NM_009665	0.00407289	-1.1641	E15.5 * Wnt1 down vs E15.5 * Sox10
10441539	Rnaset2a	NM_001083938	2.74354e-005	-1.16423	E15.5 * Wnt1 down vs E15.5 * Sox10
10354768	Akr1b3	NM_009658	0.00259475	-1.1644	E15.5 * Wnt1 down vs E15.5 * Sox10
10398599	Rps19	NM_023133	0.00337654	-1.16482	E15.5 * Wnt1 down vs E15.5 * Sox10
10394862	Ddef2	NM_001135192	0.00310071	-1.16607	E15.5 * Wnt1 down vs E15.5 * Sox10
10363498	Ppa1	NM_026438	0.00193229	-1.16632	E15.5 * Wnt1 down vs E15.5 * Sox10
10346074	Wdr75	NM_028599	0.00127266	-1.16635	E15.5 * Wnt1 down vs E15.5 * Sox10
10518019	Ddi2	NM_001017966	1.33209e-005	-1.16666	E15.5 * Wnt1 down vs E15.5 * Sox10

10358717	1700025G04Rik	BC034723	0.00174348	-1.16672	E15.5 * Wnt1 down vs E15.5 * Sox10
10526302	Tbl2	NM_013763	0.000529	-1.16704	E15.5 * Wnt1 down vs E15.5 * Sox10
10543859	Akr1b3	NM_009658	0.00283075	-1.16713	E15.5 * Wnt1 down vs E15.5 * Sox10
10356351	Rpl6	NM_011290	5.33727e-005	-1.16718	E15.5 * Wnt1 down vs E15.5 * Sox10
10528332	Napepld	NM_178728	0.00333964	-1.16751	E15.5 * Wnt1 down vs E15.5 * Sox10
10412038	Zswim6	NM_145456	0.00134964	-1.16764	E15.5 * Wnt1 down vs E15.5 * Sox10
10369890	Cisd1	BC013522	0.000130366	-1.16777	E15.5 * Wnt1 down vs E15.5 * Sox10
10484912	Ndufs3	NM_026688	0.00412752	-1.16788	E15.5 * Wnt1 down vs E15.5 * Sox10
10446109	Safb	ENSMUST00000(	0.000139006	-1.1679	E15.5 * Wnt1 down vs E15.5 * Sox10
10577070	Tubgcp3	NM_198031	0.00231185	-1.16795	E15.5 * Wnt1 down vs E15.5 * Sox10
10518361	Smarca5	NM_053124	0.00317046	-1.16841	E15.5 * Wnt1 down vs E15.5 * Sox10
10521174	Rnf4	NM_011278	0.00300358	-1.16848	E15.5 * Wnt1 down vs E15.5 * Sox10
10568865	6430531B16Rik	NM_001033465	0.00186683	-1.16859	E15.5 * Wnt1 down vs E15.5 * Sox10
10440406	Nrip1	NM_173440	0.00347736	-1.16889	E15.5 * Wnt1 down vs E15.5 * Sox10
10546113	Sec61a1	NM_016906	0.00119506	-1.169	E15.5 * Wnt1 down vs E15.5 * Sox10
10417813	Ecd	NM_027475	0.00156239	-1.16953	E15.5 * Wnt1 down vs E15.5 * Sox10
10582201	Cox4nb	NM_010926	0.00178402	-1.16971	E15.5 * Wnt1 down vs E15.5 * Sox10
10538932	Amd1	NM_009665	0.00305618	-1.17071	E15.5 * Wnt1 down vs E15.5 * Sox10
10510365	Exosc10	NM_016699	0.00430512	-1.17125	E15.5 * Wnt1 down vs E15.5 * Sox10
10440661	Rpl13	NM_016738	0.00229011	-1.17143	E15.5 * Wnt1 down vs E15.5 * Sox10
10478928	Tshz2	NM_080455	0.00220007	-1.17146	E15.5 * Wnt1 down vs E15.5 * Sox10
10522024	Tbc1d1	NM_019636	0.000467867	-1.17154	E15.5 * Wnt1 down vs E15.5 * Sox10
10468789	Pdzd8	NM_001033222	0.00119513	-1.17161	E15.5 * Wnt1 down vs E15.5 * Sox10
10506736	Magoh	NM_010760	0.00321371	-1.17209	E15.5 * Wnt1 down vs E15.5 * Sox10
10385236	Akr1b3	NM_009658	0.00380884	-1.17224	E15.5 * Wnt1 down vs E15.5 * Sox10
10369761	Reep3	NM_178606	0.00397458	-1.17288	E15.5 * Wnt1 down vs E15.5 * Sox10
10594754	Foxb1	NM_022378	0.0047387	-1.17293	E15.5 * Wnt1 down vs E15.5 * Sox10
10385034	Rpsa	NM_011029	0.000557085	-1.17372	E15.5 * Wnt1 down vs E15.5 * Sox10
10579874	Abce1	NM_015751	0.00190671	-1.17387	E15.5 * Wnt1 down vs E15.5 * Sox10
10502522	Hs2st1	NM_011828	2.60359e-005	-1.1747	E15.5 * Wnt1 down vs E15.5 * Sox10
10481900	Rabepk	NM_145522	0.00106477	-1.17528	E15.5 * Wnt1 down vs E15.5 * Sox10
10603328	Ccdc22	NM_138603	0.00358613	-1.17572	E15.5 * Wnt1 down vs E15.5 * Sox10
10579830	Rbmxrt	NM_009033	0.000954504	-1.17645	E15.5 * Wnt1 down vs E15.5 * Sox10
10408952	Nup153	NM_175749	0.00432747	-1.17694	E15.5 * Wnt1 down vs E15.5 * Sox10
10365749	Lta4h	NM_008517	0.00276071	-1.17724	E15.5 * Wnt1 down vs E15.5 * Sox10
10542445	Strap	NM_011499	9.97677e-005	-1.17735	E15.5 * Wnt1 down vs E15.5 * Sox10
10583952	Ncapd3	NM_178113	0.00428387	-1.17743	E15.5 * Wnt1 down vs E15.5 * Sox10
10388971	Utp6	NM_144826	0.00150869	-1.17744	E15.5 * Wnt1 down vs E15.5 * Sox10
10414256	Rbmxrt	NM_009033	0.000985143	-1.17753	E15.5 * Wnt1 down vs E15.5 * Sox10
10566333	9230105E10Rik	NM_001146007	0.00492397	-1.1779	E15.5 * Wnt1 down vs E15.5 * Sox10
10391762	Gjc1	NM_001159382	2.21957e-005	-1.178	E15.5 * Wnt1 down vs E15.5 * Sox10
10461334	Mta2	NM_011842	0.00215078	-1.17868	E15.5 * Wnt1 down vs E15.5 * Sox10
10525187	Rpl6	NM_011290	1.54176e-005	-1.17881	E15.5 * Wnt1 down vs E15.5 * Sox10
10590648	Top2a	NM_011623	0.000809827	-1.17942	E15.5 * Wnt1 down vs E15.5 * Sox10
10581473	Slc7a6os	NM_001007567	0.00316015	-1.17977	E15.5 * Wnt1 down vs E15.5 * Sox10
10406245		---	0.00197123	-1.18002	E15.5 * Wnt1 down vs E15.5 * Sox10
10479775	Hspa14	NM_015765	0.00333868	-1.18084	E15.5 * Wnt1 down vs E15.5 * Sox10
10562368	4931406P16Rik	NM_172741	0.00209699	-1.18149	E15.5 * Wnt1 down vs E15.5 * Sox10
10407173	Il6st	NM_010560	0.00232518	-1.18159	E15.5 * Wnt1 down vs E15.5 * Sox10
10455656	Hsd17b4	NM_008292	0.000442886	-1.1818	E15.5 * Wnt1 down vs E15.5 * Sox10
10547492	Ccdc77	NM_026028	0.00334365	-1.18257	E15.5 * Wnt1 down vs E15.5 * Sox10
10590808	Yap1	NM_009534	0.00393695	-1.18291	E15.5 * Wnt1 down vs E15.5 * Sox10
10415662	Rcbtb1	NM_027764	0.000853377	-1.18293	E15.5 * Wnt1 down vs E15.5 * Sox10
10402795	Cdca4	NM_028023	0.00424995	-1.18298	E15.5 * Wnt1 down vs E15.5 * Sox10
10573637	Phkb	NM_199446	0.00274439	-1.18307	E15.5 * Wnt1 down vs E15.5 * Sox10
10404578	Cdyl	NM_009881	0.0045194	-1.18405	E15.5 * Wnt1 down vs E15.5 * Sox10
10394892	Cpsf3	NM_018813	0.000521263	-1.18417	E15.5 * Wnt1 down vs E15.5 * Sox10
10572932	Nat5	NM_001141965	0.00390291	-1.18463	E15.5 * Wnt1 down vs E15.5 * Sox10
10583942	Thyn1	NM_144543	0.00171688	-1.18465	E15.5 * Wnt1 down vs E15.5 * Sox10
10478341	Ift52	NM_172150	0.00166982	-1.1848	E15.5 * Wnt1 down vs E15.5 * Sox10
10359097	Tor1aip1	NM_144791	0.00379095	-1.18487	E15.5 * Wnt1 down vs E15.5 * Sox10
10487930	Pcna	NM_011045	7.2862e-006	-1.18519	E15.5 * Wnt1 down vs E15.5 * Sox10
10423346	Zfp622	NM_144523	0.00271655	-1.18537	E15.5 * Wnt1 down vs E15.5 * Sox10
10539177	Eif4a3	NM_138669	0.000141856	-1.18547	E15.5 * Wnt1 down vs E15.5 * Sox10
10377319	Myh10	NM_175260	0.00179079	-1.18547	E15.5 * Wnt1 down vs E15.5 * Sox10
10456579	Mex3c	NM_001039214	0.0018586	-1.18549	E15.5 * Wnt1 down vs E15.5 * Sox10

10368670	Amd1	NM_009665	9.78975e-005	-1.18577	E15.5 * Wnt1 down vs E15.5 * Sox10
10379564	Lig3	NM_010716	0.00462819	-1.18612	E15.5 * Wnt1 down vs E15.5 * Sox10
10603289	Clcn5	NM_016691	0.00135475	-1.18613	E15.5 * Wnt1 down vs E15.5 * Sox10
10579969	Zfp330	NM_145600	0.000400227	-1.18617	E15.5 * Wnt1 down vs E15.5 * Sox10
10525718	Arl6ip4	NM_144509	0.000513259	-1.18675	E15.5 * Wnt1 down vs E15.5 * Sox10
10446207	Clpp	NM_017393	0.00200959	-1.18682	E15.5 * Wnt1 down vs E15.5 * Sox10
10594835	Grinl1a	NM_178602	0.000301431	-1.18711	E15.5 * Wnt1 down vs E15.5 * Sox10
10352562	Gpatch2	NM_026367	0.00342053	-1.18724	E15.5 * Wnt1 down vs E15.5 * Sox10
10461391	Pcna	NM_011045	3.76626e-006	-1.18765	E15.5 * Wnt1 down vs E15.5 * Sox10
10429972	Cpsf1	NM_053193	0.000493115	-1.18794	E15.5 * Wnt1 down vs E15.5 * Sox10
10558090	Tacc2	NM_001004468	0.00355117	-1.18795	E15.5 * Wnt1 down vs E15.5 * Sox10
10473322	Cwc22	NM_030560	0.00095719	-1.18806	E15.5 * Wnt1 down vs E15.5 * Sox10
10473325	Cwc22	NM_030560	0.00095719	-1.18806	E15.5 * Wnt1 down vs E15.5 * Sox10
10473328	Cwc22	NM_030560	0.00095719	-1.18806	E15.5 * Wnt1 down vs E15.5 * Sox10
10473331	Cwc22	NM_030560	0.00095719	-1.18806	E15.5 * Wnt1 down vs E15.5 * Sox10
10473334	Cwc22	NM_030560	0.00095719	-1.18806	E15.5 * Wnt1 down vs E15.5 * Sox10
10473337	Cwc22	NM_030560	0.00095719	-1.18806	E15.5 * Wnt1 down vs E15.5 * Sox10
10473340	Cwc22	NM_030560	0.00095719	-1.18806	E15.5 * Wnt1 down vs E15.5 * Sox10
10582981	Tfdp1	NM_009361	0.00467979	-1.18816	E15.5 * Wnt1 down vs E15.5 * Sox10
10416588	1300010F03Rik	NM_027906	0.000683335	-1.18819	E15.5 * Wnt1 down vs E15.5 * Sox10
10434643	Psmb3	NM_011971	0.00301035	-1.18821	E15.5 * Wnt1 down vs E15.5 * Sox10
10419296	Wdhd1	NM_172598	0.00121556	-1.18856	E15.5 * Wnt1 down vs E15.5 * Sox10
10542397	H2afj	NM_177688	0.000266723	-1.18863	E15.5 * Wnt1 down vs E15.5 * Sox10
10574962	Nfatc3	NM_010901	0.000392121	-1.18869	E15.5 * Wnt1 down vs E15.5 * Sox10
10514000	Mpdz	NM_010820	0.00163978	-1.18959	E15.5 * Wnt1 down vs E15.5 * Sox10
10421046	Dock5	NM_177780	0.000805283	-1.18969	E15.5 * Wnt1 down vs E15.5 * Sox10
10482824	Acvr1	NM_001110204	0.000379044	-1.18995	E15.5 * Wnt1 down vs E15.5 * Sox10
10578019	Nudc	NM_010948	0.00198161	-1.19005	E15.5 * Wnt1 down vs E15.5 * Sox10
10448004	Phf10	NM_024250	6.20409e-005	-1.19013	E15.5 * Wnt1 down vs E15.5 * Sox10
10532937	Unc119b	NM_175352	0.00474961	-1.19035	E15.5 * Wnt1 down vs E15.5 * Sox10
10523529	Cops4	NM_012001	0.00039308	-1.1908	E15.5 * Wnt1 down vs E15.5 * Sox10
10569429	Cdkn1c	NM_009876	0.000164376	-1.19106	E15.5 * Wnt1 down vs E15.5 * Sox10
10571288	Gtf2e2	NM_026584	0.000897744	-1.19123	E15.5 * Wnt1 down vs E15.5 * Sox10
10590383	Deb1	NM_026794	0.00464678	-1.19124	E15.5 * Wnt1 down vs E15.5 * Sox10
10438959	Lsg1	NM_178069	0.00395726	-1.19169	E15.5 * Wnt1 down vs E15.5 * Sox10
10570744	Defb50	NM_199067	0.00249923	-1.19176	E15.5 * Wnt1 down vs E15.5 * Sox10
10507914	Sf3a3	NM_029157	0.00358106	-1.19194	E15.5 * Wnt1 down vs E15.5 * Sox10
10360076	Ndufs2	NM_153064	0.00435014	-1.19311	E15.5 * Wnt1 down vs E15.5 * Sox10
10536595	Lsm8	NM_133939	0.00318928	-1.19314	E15.5 * Wnt1 down vs E15.5 * Sox10
10368930	1700021F05Rik	NM_026411	0.00162145	-1.19323	E15.5 * Wnt1 down vs E15.5 * Sox10
10585249	Ppp2r1b	NM_001034085	0.0029551	-1.19336	E15.5 * Wnt1 down vs E15.5 * Sox10
10394823		---	0.00370346	-1.19383	E15.5 * Wnt1 down vs E15.5 * Sox10
10502232	Scye1	NM_007926	0.00377705	-1.19411	E15.5 * Wnt1 down vs E15.5 * Sox10
10445008	Lsm5	NM_025520	0.00392139	-1.19441	E15.5 * Wnt1 down vs E15.5 * Sox10
10476775	Nat5	NM_001141965	0.00417666	-1.1948	E15.5 * Wnt1 down vs E15.5 * Sox10
10446044	Hdgfrp2	NM_008233	0.00158542	-1.19489	E15.5 * Wnt1 down vs E15.5 * Sox10
10422854	Nup155	NM_133227	0.000399244	-1.1951	E15.5 * Wnt1 down vs E15.5 * Sox10
10504703	AU014645	NM_001033201	0.00198297	-1.19583	E15.5 * Wnt1 down vs E15.5 * Sox10
10371888	Tmpo	NM_001080129	0.00201271	-1.19586	E15.5 * Wnt1 down vs E15.5 * Sox10
10605421	Mtcp1	NM_001039373	0.000785233	-1.19623	E15.5 * Wnt1 down vs E15.5 * Sox10
10417070	Ipo5	NM_023579	6.62702e-007	-1.19636	E15.5 * Wnt1 down vs E15.5 * Sox10
10488482	Acss1	NM_080575	0.00311116	-1.19654	E15.5 * Wnt1 down vs E15.5 * Sox10
10478594	Ctsa	NM_008906	0.000730435	-1.19665	E15.5 * Wnt1 down vs E15.5 * Sox10
10417664	Rpl19	NM_009078	0.000339307	-1.19677	E15.5 * Wnt1 down vs E15.5 * Sox10
10352947	Mrpl15	NM_025300	0.000195118	-1.19701	E15.5 * Wnt1 down vs E15.5 * Sox10
10377380	1500010J02Rik	NR_026565	0.00178576	-1.19799	E15.5 * Wnt1 down vs E15.5 * Sox10
10496715	Znhit6	NM_001081094	2.1294e-005	-1.19877	E15.5 * Wnt1 down vs E15.5 * Sox10
10495773	Dnttip2	NM_153806	0.00264024	-1.19886	E15.5 * Wnt1 down vs E15.5 * Sox10
10533807	Cdk2ap1	NM_013812	0.00099617	-1.19897	E15.5 * Wnt1 down vs E15.5 * Sox10
10455578	Commd10	NM_178377	0.00405681	-1.19925	E15.5 * Wnt1 down vs E15.5 * Sox10
10500103	Gabpb2	NM_029885	0.000154987	-1.19932	E15.5 * Wnt1 down vs E15.5 * Sox10
10471072	Tor1b	NM_133673	0.000727691	-1.20017	E15.5 * Wnt1 down vs E15.5 * Sox10
10568859	Ndufab1	NM_028177	0.00356053	-1.20022	E15.5 * Wnt1 down vs E15.5 * Sox10
10367843	Utrn	NM_011682	0.00304489	-1.20049	E15.5 * Wnt1 down vs E15.5 * Sox10
10437748	Gspt1	NM_146066	3.10494e-005	-1.2005	E15.5 * Wnt1 down vs E15.5 * Sox10
10536376	Mios	NM_145374	0.00421176	-1.20153	E15.5 * Wnt1 down vs E15.5 * Sox10

10557793	Stx4a	NM_009294	0.00341206	-1.20158	E15.5 * Wnt1 down vs E15.5 * Sox10
10344939	Terf1	NM_009352	0.000535689	-1.20169	E15.5 * Wnt1 down vs E15.5 * Sox10
10541067	Eif4a3	NM_138669	3.00661e-005	-1.20202	E15.5 * Wnt1 down vs E15.5 * Sox10
10580986	Rbmxrt	NM_009033	0.00335688	-1.20217	E15.5 * Wnt1 down vs E15.5 * Sox10
10593492	Zc3h12c	AK220416	0.000906101	-1.20218	E15.5 * Wnt1 down vs E15.5 * Sox10
10518726	Slc25a33	NM_027460	0.00336472	-1.2025	E15.5 * Wnt1 down vs E15.5 * Sox10
10474437	Ccdc34	NM_026613	0.00206582	-1.20261	E15.5 * Wnt1 down vs E15.5 * Sox10
10433910	Top3b	NM_011624	0.00140057	-1.20264	E15.5 * Wnt1 down vs E15.5 * Sox10
10499639	Cks1b	NM_016904	0.00018793	-1.20267	E15.5 * Wnt1 down vs E15.5 * Sox10
10381668	Nmt1	NM_008707	0.000631501	-1.20283	E15.5 * Wnt1 down vs E15.5 * Sox10
10499705	Hax1	NM_011826	0.0011875	-1.20288	E15.5 * Wnt1 down vs E15.5 * Sox10
10475199	Snap23	NM_009222	0.00327746	-1.20319	E15.5 * Wnt1 down vs E15.5 * Sox10
10476276	Mavs	NM_144888	0.00192717	-1.20335	E15.5 * Wnt1 down vs E15.5 * Sox10
10402715	Bag5	NM_027404	0.00368055	-1.20338	E15.5 * Wnt1 down vs E15.5 * Sox10
10433639	Mkl2	NM_153588	0.00027325	-1.20351	E15.5 * Wnt1 down vs E15.5 * Sox10
10372668	Mdm2	NM_010786	0.00473931	-1.20371	E15.5 * Wnt1 down vs E15.5 * Sox10
10447675	Rnaset2a	NM_001083938	2.5138e-005	-1.20371	E15.5 * Wnt1 down vs E15.5 * Sox10
10462791	March5	NM_027314	0.00174572	-1.20408	E15.5 * Wnt1 down vs E15.5 * Sox10
10414333	Samd4	NM_001037221	0.00343214	-1.20419	E15.5 * Wnt1 down vs E15.5 * Sox10
10411646	Gtf2h2	NM_022011	0.00117162	-1.20419	E15.5 * Wnt1 down vs E15.5 * Sox10
10360848	Mosc2	NM_133684	0.000230233	-1.20422	E15.5 * Wnt1 down vs E15.5 * Sox10
10496796	Ssx2ip	NM_138744	0.00435296	-1.20446	E15.5 * Wnt1 down vs E15.5 * Sox10
10520010	Pmpcb	NM_028431	2.4787e-005	-1.2045	E15.5 * Wnt1 down vs E15.5 * Sox10
10555550	Clpb	NM_009191	0.0011266	-1.20476	E15.5 * Wnt1 down vs E15.5 * Sox10
10453082	Hnrpll	NM_144802	0.000108295	-1.20501	E15.5 * Wnt1 down vs E15.5 * Sox10
10533858	Eif2b1	NM_145371	0.000429545	-1.20512	E15.5 * Wnt1 down vs E15.5 * Sox10
10537292	1810058I24Rik	ENSMUST00000	0.00327404	-1.20521	E15.5 * Wnt1 down vs E15.5 * Sox10
10606689	Timm8a1	NM_013898	0.000478012	-1.20521	E15.5 * Wnt1 down vs E15.5 * Sox10
10473650	Nup160	NM_021512	0.00070853	-1.20527	E15.5 * Wnt1 down vs E15.5 * Sox10
10400762	Map4k5	NM_201519	0.00171739	-1.20556	E15.5 * Wnt1 down vs E15.5 * Sox10
10355037	Wdr12	NM_021312	0.00218413	-1.20559	E15.5 * Wnt1 down vs E15.5 * Sox10
10388613	Ccdc55	NM_001012309	0.000889414	-1.20561	E15.5 * Wnt1 down vs E15.5 * Sox10
10360003	Dusp12	NM_023173	0.00144034	-1.20567	E15.5 * Wnt1 down vs E15.5 * Sox10
10390059	Xylt2	NM_145828	0.00294891	-1.20596	E15.5 * Wnt1 down vs E15.5 * Sox10
10438308	Ranbp1	NM_011239	7.41783e-005	-1.20663	E15.5 * Wnt1 down vs E15.5 * Sox10
10435821	Nat13	NM_028108	1.53315e-005	-1.20699	E15.5 * Wnt1 down vs E15.5 * Sox10
10451372	Ptk7	NM_175168	0.000392283	-1.20778	E15.5 * Wnt1 down vs E15.5 * Sox10
10565794	Serpinh1	NM_009825	0.00214733	-1.2078	E15.5 * Wnt1 down vs E15.5 * Sox10
10556005	Ilk	NM_010562	0.00131778	-1.2078	E15.5 * Wnt1 down vs E15.5 * Sox10
10537246	Nup205	NM_027513	3.81017e-005	-1.20856	E15.5 * Wnt1 down vs E15.5 * Sox10
10562500	Dpy19l3	NM_178704	5.16482e-005	-1.20864	E15.5 * Wnt1 down vs E15.5 * Sox10
10478938	Hax1	NM_011826	0.000232253	-1.20879	E15.5 * Wnt1 down vs E15.5 * Sox10
10550102	Lig1	NM_001083188	0.00225233	-1.20918	E15.5 * Wnt1 down vs E15.5 * Sox10
10465783	Tmem179b	NM_026325	0.0011161	-1.20954	E15.5 * Wnt1 down vs E15.5 * Sox10
10386058	Sparc	NM_009242	0.000340317	-1.20974	E15.5 * Wnt1 down vs E15.5 * Sox10
10384123	Ddx56	NM_026538	0.00272636	-1.20978	E15.5 * Wnt1 down vs E15.5 * Sox10
10457587	Zfp521	NM_145492	0.00152398	-1.20982	E15.5 * Wnt1 down vs E15.5 * Sox10
10470322	Surf2	NM_013678	0.00129819	-1.20984	E15.5 * Wnt1 down vs E15.5 * Sox10
10573430	Gadd45gip1	NM_183358	0.00100899	-1.21006	E15.5 * Wnt1 down vs E15.5 * Sox10
10455813	Lmnb1	NM_010721	0.000555226	-1.21049	E15.5 * Wnt1 down vs E15.5 * Sox10
10366186	Ccdc59	NM_025602	0.00408472	-1.21073	E15.5 * Wnt1 down vs E15.5 * Sox10
10364744	Ndufs7	NM_029272	0.00433905	-1.21098	E15.5 * Wnt1 down vs E15.5 * Sox10
10591369	Dnmt1	NM_010066	0.00024651	-1.21107	E15.5 * Wnt1 down vs E15.5 * Sox10
10524150	Ankle2	NM_027922	0.00166291	-1.21109	E15.5 * Wnt1 down vs E15.5 * Sox10
10520842	Bre	NM_181279	0.00391101	-1.21109	E15.5 * Wnt1 down vs E15.5 * Sox10
10346340	9430016H08Rik	BC071241	0.00429186	-1.21111	E15.5 * Wnt1 down vs E15.5 * Sox10
10458213	Etf1	NM_144866	0.000189444	-1.21145	E15.5 * Wnt1 down vs E15.5 * Sox10
10354432	Myo1b	NM_010863	6.70785e-005	-1.21161	E15.5 * Wnt1 down vs E15.5 * Sox10
10549594	Ttyh1	NM_021324	0.0040578	-1.21167	E15.5 * Wnt1 down vs E15.5 * Sox10
10590253	Rpsa	NM_011029	0.00114424	-1.21194	E15.5 * Wnt1 down vs E15.5 * Sox10
10438753	Leprel1	NM_173379	0.00104656	-1.21221	E15.5 * Wnt1 down vs E15.5 * Sox10
10539080	St3gal5	NM_011375	0.000831984	-1.21276	E15.5 * Wnt1 down vs E15.5 * Sox10
10350489	Uchl5	NM_019562	8.72337e-005	-1.21277	E15.5 * Wnt1 down vs E15.5 * Sox10
10356657	Ndufa10	NM_024197	0.000170728	-1.2131	E15.5 * Wnt1 down vs E15.5 * Sox10
10391625	Ubtf	NM_011551	0.0030292	-1.21317	E15.5 * Wnt1 down vs E15.5 * Sox10
10582778		---	0.00139346	-1.21324	E15.5 * Wnt1 down vs E15.5 * Sox10

10558914	Rplp2	NM_026020	0.00100322	-1.21334	E15.5 * Wnt1 down vs E15.5 * Sox10
10512499	Tpm2	NM_009416	0.00307032	-1.214	E15.5 * Wnt1 down vs E15.5 * Sox10
10383799	Tcn2	NM_015749	0.00353647	-1.2142	E15.5 * Wnt1 down vs E15.5 * Sox10
10453006	Cebpz	NM_001024806	0.000616984	-1.21428	E15.5 * Wnt1 down vs E15.5 * Sox10
10561356	Psmc4	NM_011874	0.00290164	-1.21439	E15.5 * Wnt1 down vs E15.5 * Sox10
10463799	EG329070	ENSMUST00000	0.00138348	-1.21453	E15.5 * Wnt1 down vs E15.5 * Sox10
10494060	Mrpl9	NM_030116	0.00293267	-1.21483	E15.5 * Wnt1 down vs E15.5 * Sox10
10382200	Ccdc46	NM_029606	0.00272967	-1.21551	E15.5 * Wnt1 down vs E15.5 * Sox10
10597920	Zdhhc3	NM_026917	0.000147502	-1.21552	E15.5 * Wnt1 down vs E15.5 * Sox10
10386548	Pabpc4	NM_130881	0.0016084	-1.21598	E15.5 * Wnt1 down vs E15.5 * Sox10
10512935	Amd1	NM_009665	0.000901965	-1.21608	E15.5 * Wnt1 down vs E15.5 * Sox10
10405094	Iars	NM_172015	0.000104597	-1.2163	E15.5 * Wnt1 down vs E15.5 * Sox10
10529425	Nop14	NM_029278	0.000181998	-1.21654	E15.5 * Wnt1 down vs E15.5 * Sox10
10446693	Wdr43	AK129031	7.18617e-005	-1.21658	E15.5 * Wnt1 down vs E15.5 * Sox10
10485225	Ext2	NM_010163	0.00374922	-1.2167	E15.5 * Wnt1 down vs E15.5 * Sox10
10358816	Lamc1	NM_010683	0.00444079	-1.21706	E15.5 * Wnt1 down vs E15.5 * Sox10
10396919	4933426M11Rik	BC040401	0.000713347	-1.21706	E15.5 * Wnt1 down vs E15.5 * Sox10
10522009	Pgm1	NM_025700	0.00249015	-1.2172	E15.5 * Wnt1 down vs E15.5 * Sox10
10345230	Rab23	NM_008999	0.00300001	-1.21735	E15.5 * Wnt1 down vs E15.5 * Sox10
10403511	Heatr1	NM_144835	9.52709e-005	-1.21755	E15.5 * Wnt1 down vs E15.5 * Sox10
10385495	Cdk2ap1	NM_013812	0.000567214	-1.2176	E15.5 * Wnt1 down vs E15.5 * Sox10
10414522	Apex1	NM_009687	0.000154018	-1.2183	E15.5 * Wnt1 down vs E15.5 * Sox10
10564527	Nr2f2	NM_009697	0.00193628	-1.21864	E15.5 * Wnt1 down vs E15.5 * Sox10
10353032	Rpa3	NM_026632	0.00418964	-1.21869	E15.5 * Wnt1 down vs E15.5 * Sox10
10375485		---	0.00197001	-1.21888	E15.5 * Wnt1 down vs E15.5 * Sox10
10491385	Actl6a	NM_019673	0.00148809	-1.219	E15.5 * Wnt1 down vs E15.5 * Sox10
10529741	Rab28	NM_027295	0.000782122	-1.21903	E15.5 * Wnt1 down vs E15.5 * Sox10
10389245	Tada2l	NM_172562	0.00151753	-1.2192	E15.5 * Wnt1 down vs E15.5 * Sox10
10593856	C230081A13Rik	NM_172924	0.00444597	-1.21956	E15.5 * Wnt1 down vs E15.5 * Sox10
10386543	Pabpc4	NM_130881	0.000798456	-1.22009	E15.5 * Wnt1 down vs E15.5 * Sox10
10368748	Amd1	NM_009665	0.00015849	-1.22018	E15.5 * Wnt1 down vs E15.5 * Sox10
10461214	Tmem223	NM_025791	0.00439541	-1.22021	E15.5 * Wnt1 down vs E15.5 * Sox10
10440840	1110004E09Rik	BC019533	0.00148482	-1.22033	E15.5 * Wnt1 down vs E15.5 * Sox10
10430811	Nhp2l1	NM_011482	0.000552056	-1.22046	E15.5 * Wnt1 down vs E15.5 * Sox10
10564713	Mfge8	NM_008594	0.00106579	-1.22069	E15.5 * Wnt1 down vs E15.5 * Sox10
10540738	Fancd2	NM_001033244	0.00462232	-1.22118	E15.5 * Wnt1 down vs E15.5 * Sox10
10581940	Kars	NM_001130868	0.00218148	-1.22205	E15.5 * Wnt1 down vs E15.5 * Sox10
10586405	Spg21	NM_138584	0.00302897	-1.22244	E15.5 * Wnt1 down vs E15.5 * Sox10
10379953	4632419I22Rik	BC067002	0.000990786	-1.22267	E15.5 * Wnt1 down vs E15.5 * Sox10
10563583	Saal1	NM_030233	0.00195689	-1.2227	E15.5 * Wnt1 down vs E15.5 * Sox10
10505931	Ift74	NM_026319	0.00445154	-1.22339	E15.5 * Wnt1 down vs E15.5 * Sox10
10434348	Eif2b5	NM_172265	0.00473351	-1.2237	E15.5 * Wnt1 down vs E15.5 * Sox10
10472240	Tanc1	NM_198294	0.000758675	-1.22383	E15.5 * Wnt1 down vs E15.5 * Sox10
10541482	Nhp2l1	NM_011482	0.00124226	-1.22408	E15.5 * Wnt1 down vs E15.5 * Sox10
10359648	Scyl3	NM_028776	6.36925e-005	-1.22458	E15.5 * Wnt1 down vs E15.5 * Sox10
10445688	Ccnd3	NM_001081636	0.00289663	-1.22476	E15.5 * Wnt1 down vs E15.5 * Sox10
10537102	Exoc4	NM_009148	0.00203594	-1.22482	E15.5 * Wnt1 down vs E15.5 * Sox10
10394821	LOC677565	XR_034928	0.000846739	-1.2249	E15.5 * Wnt1 down vs E15.5 * Sox10
10429081	Snrpc	NM_011432	0.00193989	-1.22507	E15.5 * Wnt1 down vs E15.5 * Sox10
10347460	Ttll4	NM_001014974	4.56673e-005	-1.22527	E15.5 * Wnt1 down vs E15.5 * Sox10
10474671	Spred1	NM_033524	0.00306671	-1.22538	E15.5 * Wnt1 down vs E15.5 * Sox10
10473414	Ssrp1	NM_182990	0.000285177	-1.22551	E15.5 * Wnt1 down vs E15.5 * Sox10
10377215	Gas7	NM_008088	0.00086503	-1.22562	E15.5 * Wnt1 down vs E15.5 * Sox10
10382565	Mrps7	NM_025305	0.000131765	-1.22571	E15.5 * Wnt1 down vs E15.5 * Sox10
10595529	4922501C03Rik	NM_199316	0.00222757	-1.22621	E15.5 * Wnt1 down vs E15.5 * Sox10
10543213	B630005N14Rik	NM_175312	0.00259229	-1.22622	E15.5 * Wnt1 down vs E15.5 * Sox10
10444800	Nhp2l1	NM_011482	0.000703337	-1.22631	E15.5 * Wnt1 down vs E15.5 * Sox10
10558847	Phrf1	NM_001081118	0.00291575	-1.2264	E15.5 * Wnt1 down vs E15.5 * Sox10
10352709	Nsl1	NM_198654	0.00318787	-1.22647	E15.5 * Wnt1 down vs E15.5 * Sox10
10349316	Tmem185b	NM_146103	0.00375515	-1.22686	E15.5 * Wnt1 down vs E15.5 * Sox10
10456383	Impa2	NM_053261	0.00082172	-1.22701	E15.5 * Wnt1 down vs E15.5 * Sox10
10509965	Epha2	NM_010139	0.000411939	-1.22716	E15.5 * Wnt1 down vs E15.5 * Sox10
10517463	Luzp1	BC137786	0.00022811	-1.22717	E15.5 * Wnt1 down vs E15.5 * Sox10
10531428	Nup54	NM_183392	0.00497171	-1.22722	E15.5 * Wnt1 down vs E15.5 * Sox10
10575102	Cirh1a	NM_011574	0.00449795	-1.22724	E15.5 * Wnt1 down vs E15.5 * Sox10
10588243	Ryk	NM_013649	0.000655564	-1.22735	E15.5 * Wnt1 down vs E15.5 * Sox10

10581327	E130303B06Rik	BC052498	0.00349393	-1.22757	E15.5 * Wnt1 down vs E15.5 * Sox10
10585091	Nhp2l1	NM_011482	0.000607339	-1.22767	E15.5 * Wnt1 down vs E15.5 * Sox10
10554800	Rab38	NM_028238	0.000138784	-1.22783	E15.5 * Wnt1 down vs E15.5 * Sox10
10356800	Hdlbp	NM_133808	8.95967e-007	-1.22786	E15.5 * Wnt1 down vs E15.5 * Sox10
10556169	Eif3f	NM_025344	0.000308992	-1.22807	E15.5 * Wnt1 down vs E15.5 * Sox10
10502257	Gstcd	NM_026231	0.000544818	-1.22811	E15.5 * Wnt1 down vs E15.5 * Sox10
10459905	Setbp1	NM_053099	0.00496671	-1.22814	E15.5 * Wnt1 down vs E15.5 * Sox10
10459389	Amd1	NM_009665	0.000356586	-1.22887	E15.5 * Wnt1 down vs E15.5 * Sox10
10556571	Rpl19	NM_009078	0.000162108	-1.22893	E15.5 * Wnt1 down vs E15.5 * Sox10
10451434	Ppp2r5d	NM_009358	0.00165503	-1.22923	E15.5 * Wnt1 down vs E15.5 * Sox10
10544501	Ezh2	NM_007971	4.96768e-005	-1.22924	E15.5 * Wnt1 down vs E15.5 * Sox10
10425226	Eif3l	NM_145139	5.96804e-005	-1.22967	E15.5 * Wnt1 down vs E15.5 * Sox10
10395277	Sypl	NM_013635	0.000777263	-1.22968	E15.5 * Wnt1 down vs E15.5 * Sox10
10393408	Tmc6	NM_145439	0.00158213	-1.22999	E15.5 * Wnt1 down vs E15.5 * Sox10
10377681	Dullard	NM_026017	0.00156212	-1.23018	E15.5 * Wnt1 down vs E15.5 * Sox10
10402585	Wars	NM_011710	0.00336586	-1.2302	E15.5 * Wnt1 down vs E15.5 * Sox10
10435489	Ccdc58	NM_198645	0.000153879	-1.23027	E15.5 * Wnt1 down vs E15.5 * Sox10
10436519	Robo1	NM_019413	0.00208192	-1.23046	E15.5 * Wnt1 down vs E15.5 * Sox10
10421917	Pabpc4	NM_148917	0.00375239	-1.23099	E15.5 * Wnt1 down vs E15.5 * Sox10
10394392	Nfyc	NM_008692	0.000432145	-1.23107	E15.5 * Wnt1 down vs E15.5 * Sox10
10422962	1110020G09Rik	NM_001085410	0.0005113	-1.2311	E15.5 * Wnt1 down vs E15.5 * Sox10
10508036	Snip1	NM_175246	0.00294002	-1.23115	E15.5 * Wnt1 down vs E15.5 * Sox10
10513397	1110054O05Rik	NM_001013577	0.000208106	-1.23136	E15.5 * Wnt1 down vs E15.5 * Sox10
10499394	Lmna	NM_001002011	0.00234035	-1.23158	E15.5 * Wnt1 down vs E15.5 * Sox10
10384782	Vrk2	NM_027260	0.00361912	-1.2316	E15.5 * Wnt1 down vs E15.5 * Sox10
10385903	Pdlim4	NM_019417	0.0037623	-1.23166	E15.5 * Wnt1 down vs E15.5 * Sox10
10603151	Gpm6b	NM_023122	0.00137326	-1.23187	E15.5 * Wnt1 down vs E15.5 * Sox10
10429387	Ptplb	NM_023587	0.00494259	-1.23189	E15.5 * Wnt1 down vs E15.5 * Sox10
10604763	Arpc1b	NM_023142	0.00267959	-1.23234	E15.5 * Wnt1 down vs E15.5 * Sox10
10421309	Slc39a14	NM_001135151	0.00225705	-1.23239	E15.5 * Wnt1 down vs E15.5 * Sox10
10365716	1200009F10Rik	NM_026166	0.00236448	-1.23248	E15.5 * Wnt1 down vs E15.5 * Sox10
10412921	Nid2	NM_008695	0.000955644	-1.23258	E15.5 * Wnt1 down vs E15.5 * Sox10
10374880	Mtif2	NM_133767	0.00466181	-1.23269	E15.5 * Wnt1 down vs E15.5 * Sox10
10367931	Ltv1	NM_181470	0.00374753	-1.23285	E15.5 * Wnt1 down vs E15.5 * Sox10
10434758	St6gal1	NM_145933	0.000967643	-1.23288	E15.5 * Wnt1 down vs E15.5 * Sox10
10492480	Gfm1	NM_138591	0.000820193	-1.23298	E15.5 * Wnt1 down vs E15.5 * Sox10
10348180	Eif4e2	NM_001039169	0.00142704	-1.23315	E15.5 * Wnt1 down vs E15.5 * Sox10
10408212	Hist1h1e	NM_015787	0.00168766	-1.23318	E15.5 * Wnt1 down vs E15.5 * Sox10
10535502	Jtv1	NM_146165	0.00465314	-1.2333	E15.5 * Wnt1 down vs E15.5 * Sox10
10452709	Ndc80	NM_023294	0.00438609	-1.23333	E15.5 * Wnt1 down vs E15.5 * Sox10
10386125	Gemin5	NM_172558	0.0043134	-1.23354	E15.5 * Wnt1 down vs E15.5 * Sox10
10487577	Ckap2l	NM_181589	0.00463901	-1.23362	E15.5 * Wnt1 down vs E15.5 * Sox10
10450814	Ppp1r11	NM_029632	0.000886488	-1.23363	E15.5 * Wnt1 down vs E15.5 * Sox10
10364251	Pofut2	NM_030262	0.00193959	-1.2338	E15.5 * Wnt1 down vs E15.5 * Sox10
10504518	Polr1e	NM_022811	0.000835791	-1.23386	E15.5 * Wnt1 down vs E15.5 * Sox10
10423825	Fzd6	NM_008056	0.000164077	-1.23409	E15.5 * Wnt1 down vs E15.5 * Sox10
10375501		---	0.00279585	-1.23414	E15.5 * Wnt1 down vs E15.5 * Sox10
10492682	1110032E23Rik	NM_133187	3.54071e-005	-1.23419	E15.5 * Wnt1 down vs E15.5 * Sox10
10358668	Hmcn1	NM_001024720	0.00319838	-1.23424	E15.5 * Wnt1 down vs E15.5 * Sox10
10372082	Nudt4	NM_027722	0.000823861	-1.23437	E15.5 * Wnt1 down vs E15.5 * Sox10
10449163	Pigq	NM_011822	0.000854487	-1.23493	E15.5 * Wnt1 down vs E15.5 * Sox10
10503315	E130016E03Rik	NM_001039556	0.00465788	-1.23493	E15.5 * Wnt1 down vs E15.5 * Sox10
10525733	Setd8	NM_030241	0.000626071	-1.23507	E15.5 * Wnt1 down vs E15.5 * Sox10
10425632	Xrcc6	NM_010247	0.00159837	-1.2351	E15.5 * Wnt1 down vs E15.5 * Sox10
10466000	Tmem138	NM_028411	0.00172347	-1.23553	E15.5 * Wnt1 down vs E15.5 * Sox10
10366038	Galnt4	NM_015737	0.000660334	-1.2357	E15.5 * Wnt1 down vs E15.5 * Sox10
10516640	Eif3i	NM_018799	0.000370895	-1.23573	E15.5 * Wnt1 down vs E15.5 * Sox10
10367728	Map3k7ip2	NM_138667	0.00111786	-1.23591	E15.5 * Wnt1 down vs E15.5 * Sox10
10559270	Tssc4	NM_020285	0.000525729	-1.23593	E15.5 * Wnt1 down vs E15.5 * Sox10
10362581	Tube1	NM_028006	0.00189286	-1.23595	E15.5 * Wnt1 down vs E15.5 * Sox10
10579776	Arhgap10	NM_030113	0.000832483	-1.23649	E15.5 * Wnt1 down vs E15.5 * Sox10
10604501	Hnrnpf	NM_133834	6.59798e-006	-1.23653	E15.5 * Wnt1 down vs E15.5 * Sox10
10522973	Utp3	NM_023054	0.00443198	-1.23681	E15.5 * Wnt1 down vs E15.5 * Sox10
10368060	ENSMUSG0000C	ENSMUST00000	0.000775179	-1.237	E15.5 * Wnt1 down vs E15.5 * Sox10
10585721	Scamp2	NM_022813	0.00492963	-1.23715	E15.5 * Wnt1 down vs E15.5 * Sox10
10415377	2610027L16Rik	NM_026403	0.00153706	-1.23719	E15.5 * Wnt1 down vs E15.5 * Sox10

10511679	Decr1	NM_026172	0.00168921	-1.23728	E15.5 * Wnt1 down vs E15.5 * Sox10
10578690	Neil3	NM_146208	0.00229984	-1.23773	E15.5 * Wnt1 down vs E15.5 * Sox10
10523001	Mobkl1a	NM_026735	0.000700884	-1.23778	E15.5 * Wnt1 down vs E15.5 * Sox10
10347481	Cyp27a1	NM_024264	0.00164167	-1.23794	E15.5 * Wnt1 down vs E15.5 * Sox10
10461268	1810009A15Rik	BC047099	0.000231109	-1.23808	E15.5 * Wnt1 down vs E15.5 * Sox10
10499454	Dap3	NM_022994	0.00366891	-1.23812	E15.5 * Wnt1 down vs E15.5 * Sox10
10363545	Neurog3	NM_009719	0.00463501	-1.23815	E15.5 * Wnt1 down vs E15.5 * Sox10
10374706	Pus10	NM_028304	0.000379352	-1.23842	E15.5 * Wnt1 down vs E15.5 * Sox10
10437311	Trap1	NM_026508	0.000394393	-1.23875	E15.5 * Wnt1 down vs E15.5 * Sox10
10366277	E2f7	NM_178609	0.00121584	-1.23891	E15.5 * Wnt1 down vs E15.5 * Sox10
10603431	Suv39h1	NM_011514	0.000613187	-1.23932	E15.5 * Wnt1 down vs E15.5 * Sox10
10421981	Dis3	NM_028315	0.000271497	-1.23934	E15.5 * Wnt1 down vs E15.5 * Sox10
10504957	Smc2	NM_008017	0.000331564	-1.23941	E15.5 * Wnt1 down vs E15.5 * Sox10
10517336	Clic4	NM_013885	0.000169926	-1.23957	E15.5 * Wnt1 down vs E15.5 * Sox10
10587315	Gsta4	NM_010357	0.00439864	-1.23967	E15.5 * Wnt1 down vs E15.5 * Sox10
10416736	6720463M24Rik	NM_175265	0.00266443	-1.23979	E15.5 * Wnt1 down vs E15.5 * Sox10
10360105	Usp21	NM_013919	0.00326912	-1.2403	E15.5 * Wnt1 down vs E15.5 * Sox10
10455695	Srfbp1	NM_026040	0.00304497	-1.24034	E15.5 * Wnt1 down vs E15.5 * Sox10
10436561	Usp25	NM_013918	0.00344835	-1.24038	E15.5 * Wnt1 down vs E15.5 * Sox10
10492846	Pet112l	NM_144896	0.000180532	-1.24057	E15.5 * Wnt1 down vs E15.5 * Sox10
10602223	Alg13	NM_026247	0.000657922	-1.24091	E15.5 * Wnt1 down vs E15.5 * Sox10
10561837	Zfp146	NM_011980	0.00418475	-1.24101	E15.5 * Wnt1 down vs E15.5 * Sox10
10510464	Lzic	NM_026963	0.00185788	-1.24129	E15.5 * Wnt1 down vs E15.5 * Sox10
10349610	Lgtn	NM_001136070	0.000762818	-1.24142	E15.5 * Wnt1 down vs E15.5 * Sox10
10437970	2310008H04Rik	NM_146068	7.01922e-005	-1.24143	E15.5 * Wnt1 down vs E15.5 * Sox10
10424825	Cyc1	NM_025567	0.00231322	-1.24178	E15.5 * Wnt1 down vs E15.5 * Sox10
10588495	Dusp7	NM_153459	0.00321708	-1.24232	E15.5 * Wnt1 down vs E15.5 * Sox10
10488303	Crnkl1	NM_025820	0.00286203	-1.24263	E15.5 * Wnt1 down vs E15.5 * Sox10
10492798	Sfrp2	NM_009144	0.00398714	-1.24283	E15.5 * Wnt1 down vs E15.5 * Sox10
10393823	P4hb	NM_011032	0.00120527	-1.24293	E15.5 * Wnt1 down vs E15.5 * Sox10
10447431	Foxn2	NM_180974	5.37016e-005	-1.24305	E15.5 * Wnt1 down vs E15.5 * Sox10
10441791	D17Ertd663e	ENSMUST00000	0.00307021	-1.24307	E15.5 * Wnt1 down vs E15.5 * Sox10
10451238	Polr1c	NM_009085	0.00151724	-1.24321	E15.5 * Wnt1 down vs E15.5 * Sox10
10492558	Smc4	NM_133786	6.96414e-005	-1.24332	E15.5 * Wnt1 down vs E15.5 * Sox10
10466848	D19Bwg1357e	NM_177474	1.13972e-005	-1.24337	E15.5 * Wnt1 down vs E15.5 * Sox10
10551803	Zfp568	NM_001033355	0.000372621	-1.24338	E15.5 * Wnt1 down vs E15.5 * Sox10
10461257	Ubxn1	NM_146093	0.00204219	-1.24469	E15.5 * Wnt1 down vs E15.5 * Sox10
10605256	Flna	NM_010227	0.00470815	-1.24489	E15.5 * Wnt1 down vs E15.5 * Sox10
10586184	Tipin	NM_025372	0.000133523	-1.24499	E15.5 * Wnt1 down vs E15.5 * Sox10
10597288	Mlh1	NM_026810	0.00122648	-1.24509	E15.5 * Wnt1 down vs E15.5 * Sox10
10589420	Cdc25a	NM_007658	0.000521063	-1.24511	E15.5 * Wnt1 down vs E15.5 * Sox10
10590549	Exosc7	NM_001081188	0.000505466	-1.24525	E15.5 * Wnt1 down vs E15.5 * Sox10
10406881	Smn1	NM_011420	0.000794051	-1.24536	E15.5 * Wnt1 down vs E15.5 * Sox10
10400642	1110034A24Rik	BC062898	0.000971957	-1.2454	E15.5 * Wnt1 down vs E15.5 * Sox10
10449394	Taf11	NM_026836	5.18274e-005	-1.2454	E15.5 * Wnt1 down vs E15.5 * Sox10
10437687	Litaf	NM_019980	8.59187e-005	-1.24541	E15.5 * Wnt1 down vs E15.5 * Sox10
10458804	EG225468	XM_911153	0.000955751	-1.24579	E15.5 * Wnt1 down vs E15.5 * Sox10
10484425	2700094K13Rik	NM_001033166	0.000227452	-1.24587	E15.5 * Wnt1 down vs E15.5 * Sox10
10426477	Snrpc	NM_011432	0.00052422	-1.24598	E15.5 * Wnt1 down vs E15.5 * Sox10
10507500	Slc6a9	NM_008135	0.00240759	-1.24608	E15.5 * Wnt1 down vs E15.5 * Sox10
10537184	Cald1	NM_145575	0.00265731	-1.24615	E15.5 * Wnt1 down vs E15.5 * Sox10
10510201	Rex2	NM_009051	0.00181233	-1.24615	E15.5 * Wnt1 down vs E15.5 * Sox10
10521090	Tacc3	NM_001040435	0.000211693	-1.2463	E15.5 * Wnt1 down vs E15.5 * Sox10
10385239	Mat2b	NM_134017	7.46882e-005	-1.24637	E15.5 * Wnt1 down vs E15.5 * Sox10
10457054	Zadh2	NM_146090	0.00307499	-1.24648	E15.5 * Wnt1 down vs E15.5 * Sox10
10583634	Qtrt1	NM_021888	0.000722937	-1.24655	E15.5 * Wnt1 down vs E15.5 * Sox10
10528077	Dbf4	NM_013726	0.00188534	-1.24659	E15.5 * Wnt1 down vs E15.5 * Sox10
10391755	Ccdc43	NM_025918	0.000514122	-1.24702	E15.5 * Wnt1 down vs E15.5 * Sox10
10445558	BC048355	NM_207161	0.00242247	-1.24739	E15.5 * Wnt1 down vs E15.5 * Sox10
10523595	Ptpn13	NM_011204	7.09318e-005	-1.24761	E15.5 * Wnt1 down vs E15.5 * Sox10
10520080	Rint1	NM_177323	0.000661054	-1.24761	E15.5 * Wnt1 down vs E15.5 * Sox10
10412559	Slbp	NM_009193	0.00202132	-1.24785	E15.5 * Wnt1 down vs E15.5 * Sox10
10355742	Abcb6	NM_023732	0.000871815	-1.2479	E15.5 * Wnt1 down vs E15.5 * Sox10
10522075	Klhl5	NM_175174	0.000357833	-1.24791	E15.5 * Wnt1 down vs E15.5 * Sox10
10352242	Parp1	NM_007415	0.00375763	-1.24793	E15.5 * Wnt1 down vs E15.5 * Sox10
10470427	Wdr5	NM_080848	0.000602088	-1.24795	E15.5 * Wnt1 down vs E15.5 * Sox10

10459481	Lman1	NM_027400	0.00147956	-1.24827	E15.5 * Wnt1 down vs E15.5 * Sox10
10546834	Rad18	NM_021385	0.000109473	-1.24839	E15.5 * Wnt1 down vs E15.5 * Sox10
10579925	Gab1	NM_021356	0.000242321	-1.24876	E15.5 * Wnt1 down vs E15.5 * Sox10
10507557	Ebna1bp2	NM_026932	0.00020031	-1.24892	E15.5 * Wnt1 down vs E15.5 * Sox10
10384192	Tbrg4	NM_134011	8.884e-005	-1.24908	E15.5 * Wnt1 down vs E15.5 * Sox10
10603896	Klhl13	NM_026167	0.000861867	-1.24924	E15.5 * Wnt1 down vs E15.5 * Sox10
10401997	Ptpn21	NM_011877	0.00136555	-1.24927	E15.5 * Wnt1 down vs E15.5 * Sox10
10423629	Pop1	NM_152894	0.00422807	-1.24928	E15.5 * Wnt1 down vs E15.5 * Sox10
10557450	Bola2	NM_175103	0.000486285	-1.24953	E15.5 * Wnt1 down vs E15.5 * Sox10
10352119	Pppde1	NM_024282	0.000682528	-1.24984	E15.5 * Wnt1 down vs E15.5 * Sox10
10375083	Stk10	NM_009288	0.00109198	-1.24992	E15.5 * Wnt1 down vs E15.5 * Sox10
10603702	Llph	NM_025431	0.000221779	-1.25014	E15.5 * Wnt1 down vs E15.5 * Sox10
10503659	Epha7	NM_010141	9.83132e-005	-1.25031	E15.5 * Wnt1 down vs E15.5 * Sox10
10543835	Chchd3	NM_025336	0.000720188	-1.25097	E15.5 * Wnt1 down vs E15.5 * Sox10
10596652	Hemk1	NM_133984	0.000602564	-1.25115	E15.5 * Wnt1 down vs E15.5 * Sox10
10565996	Inppl1	NM_010567	0.00378676	-1.25152	E15.5 * Wnt1 down vs E15.5 * Sox10
10368199	Myb	NM_010848	0.00331676	-1.25169	E15.5 * Wnt1 down vs E15.5 * Sox10
10565018	Iqgap1	NM_016721	0.00049886	-1.25214	E15.5 * Wnt1 down vs E15.5 * Sox10
10502780	Lphn2	NM_001081298	0.00137098	-1.25214	E15.5 * Wnt1 down vs E15.5 * Sox10
10468519	Smndc1	NM_172429	0.00274449	-1.25217	E15.5 * Wnt1 down vs E15.5 * Sox10
10545379	Usp39	NM_138592	0.000255884	-1.25245	E15.5 * Wnt1 down vs E15.5 * Sox10
10447708	Qk	NM_021881	1.40246e-005	-1.2529	E15.5 * Wnt1 down vs E15.5 * Sox10
10375667	Rnf130	NM_021540	0.00234713	-1.25306	E15.5 * Wnt1 down vs E15.5 * Sox10
10456727	Dym	NM_027727	0.00101188	-1.25347	E15.5 * Wnt1 down vs E15.5 * Sox10
10420080	1810034K20Rik	NM_023397	0.00353104	-1.25362	E15.5 * Wnt1 down vs E15.5 * Sox10
10428763	Atad2	NM_027435	0.00017716	-1.2539	E15.5 * Wnt1 down vs E15.5 * Sox10
10573401	Trmt1	NM_198020	0.000969716	-1.25397	E15.5 * Wnt1 down vs E15.5 * Sox10
10490986	4632415L05Rik	BC023403	0.00249681	-1.25411	E15.5 * Wnt1 down vs E15.5 * Sox10
10420071	Tm9sf1	NM_028780	0.000323255	-1.25411	E15.5 * Wnt1 down vs E15.5 * Sox10
10507218	Mknk1	NM_021461	0.0036798	-1.25413	E15.5 * Wnt1 down vs E15.5 * Sox10
10582599	Nup133	NM_172288	0.000733557	-1.25426	E15.5 * Wnt1 down vs E15.5 * Sox10
10488697	Plagl2	NM_018807	0.000849774	-1.25444	E15.5 * Wnt1 down vs E15.5 * Sox10
10358656	Hmcn1	NM_001024720	0.00233629	-1.25447	E15.5 * Wnt1 down vs E15.5 * Sox10
10478825	Ddx27	NM_153065	0.000203028	-1.25521	E15.5 * Wnt1 down vs E15.5 * Sox10
10462346	Rcl1	NM_021525	0.000132043	-1.25586	E15.5 * Wnt1 down vs E15.5 * Sox10
10482496	Mmadhc	ENSMUST00000(	0.00483971	-1.25622	E15.5 * Wnt1 down vs E15.5 * Sox10
10471555	Angptl2	NM_011923	0.000349614	-1.2563	E15.5 * Wnt1 down vs E15.5 * Sox10
10503178	Chd7	NM_001081417	0.00188553	-1.25643	E15.5 * Wnt1 down vs E15.5 * Sox10
10358670	Hmcn1	NM_001024720	0.00442312	-1.25646	E15.5 * Wnt1 down vs E15.5 * Sox10
10596137	Srprb	NM_009275	0.0015665	-1.25678	E15.5 * Wnt1 down vs E15.5 * Sox10
10454310	Galnt1	NM_013814	0.000366514	-1.25714	E15.5 * Wnt1 down vs E15.5 * Sox10
10379127	Spag5	NM_017407	0.000781456	-1.25723	E15.5 * Wnt1 down vs E15.5 * Sox10
10471191	Exosc2	NM_144886	0.00246493	-1.25733	E15.5 * Wnt1 down vs E15.5 * Sox10
10352092	Zfp238	NM_001012330	0.00255211	-1.25776	E15.5 * Wnt1 down vs E15.5 * Sox10
10396645	Zbtb1	NM_178744	0.00400849	-1.2578	E15.5 * Wnt1 down vs E15.5 * Sox10
10357133		---	2.15085e-005	-1.25799	E15.5 * Wnt1 down vs E15.5 * Sox10
10448803	Hn1l	NM_198937	0.000295761	-1.2581	E15.5 * Wnt1 down vs E15.5 * Sox10
10535017	Gal3st4	NM_001033416	0.00160977	-1.25823	E15.5 * Wnt1 down vs E15.5 * Sox10
10551021	Rps19	NM_023133	0.00127943	-1.25829	E15.5 * Wnt1 down vs E15.5 * Sox10
10439276	Fam162a	NM_027342	0.00120906	-1.2588	E15.5 * Wnt1 down vs E15.5 * Sox10
10598994	Atp1b3	NM_007502	0.000598903	-1.25906	E15.5 * Wnt1 down vs E15.5 * Sox10
10462796	Kif11	NM_010615	0.000375392	-1.25921	E15.5 * Wnt1 down vs E15.5 * Sox10
10393844	Thoc4	NM_011568	0.00351839	-1.25949	E15.5 * Wnt1 down vs E15.5 * Sox10
10424779	Cks2	NM_025415	0.00051263	-1.25952	E15.5 * Wnt1 down vs E15.5 * Sox10
10568461	Rfwd3	NM_146218	0.000693863	-1.25965	E15.5 * Wnt1 down vs E15.5 * Sox10
10483698	Wipf1	NM_153138	0.00040396	-1.25966	E15.5 * Wnt1 down vs E15.5 * Sox10
10453272	Zfp36l2	NM_001001806	0.000357736	-1.26015	E15.5 * Wnt1 down vs E15.5 * Sox10
10580160	Mri1	NM_026423	0.00143343	-1.26083	E15.5 * Wnt1 down vs E15.5 * Sox10
10514576	Kank4	NM_172872	0.00455627	-1.26091	E15.5 * Wnt1 down vs E15.5 * Sox10
10410695	Rhobtb3	NM_028493	5.60685e-005	-1.26119	E15.5 * Wnt1 down vs E15.5 * Sox10
10421672	1200011I18Rik	NM_026177	0.00209697	-1.26148	E15.5 * Wnt1 down vs E15.5 * Sox10
10410766	Nr2f1	NM_010151	0.000131703	-1.26151	E15.5 * Wnt1 down vs E15.5 * Sox10
10506201	Ror1	NM_013845	0.00104885	-1.26205	E15.5 * Wnt1 down vs E15.5 * Sox10
10565486	Eed	NM_021876	0.000116546	-1.26229	E15.5 * Wnt1 down vs E15.5 * Sox10
10377953	Kif1c	NM_153103	0.000145382	-1.26257	E15.5 * Wnt1 down vs E15.5 * Sox10
10511113	Cdc2l1	NM_007661	3.16262e-006	-1.26265	E15.5 * Wnt1 down vs E15.5 * Sox10

10522051	Klf3	NM_008453	0.000328069	-1.26283	E15.5 * Wnt1 down vs E15.5 * Sox10
10505008	Slc44a1	NM_133891	6.89618e-005	-1.2629	E15.5 * Wnt1 down vs E15.5 * Sox10
10482172	Zbtb26	NM_199025	0.00116026	-1.26298	E15.5 * Wnt1 down vs E15.5 * Sox10
10521515	Evc2	NM_145920	0.00035593	-1.26333	E15.5 * Wnt1 down vs E15.5 * Sox10
10412267	Itga2	NM_008396	0.00337532	-1.2637	E15.5 * Wnt1 down vs E15.5 * Sox10
10560575	Relb	NM_009046	0.000820695	-1.2639	E15.5 * Wnt1 down vs E15.5 * Sox10
10487359	Itpripl1	BC130218	4.38151e-006	-1.26391	E15.5 * Wnt1 down vs E15.5 * Sox10
10406434	Mef2c	NM_025282	0.000240433	-1.26417	E15.5 * Wnt1 down vs E15.5 * Sox10
10497173	Pxmp3	NM_008994	0.00189733	-1.26425	E15.5 * Wnt1 down vs E15.5 * Sox10
10376950	Pmp22	NM_008885	0.00107574	-1.26435	E15.5 * Wnt1 down vs E15.5 * Sox10
10575961	Usp10	NM_009462	0.00307496	-1.26471	E15.5 * Wnt1 down vs E15.5 * Sox10
10439936	Nfkbiz	NM_030612	0.000618039	-1.26486	E15.5 * Wnt1 down vs E15.5 * Sox10
10532720	Sart3	NM_016926	0.0028599	-1.26512	E15.5 * Wnt1 down vs E15.5 * Sox10
10446441	Ddx11	NM_001003919	0.00102807	-1.26531	E15.5 * Wnt1 down vs E15.5 * Sox10
10570516	Kbtbd11	NM_029116	0.000247674	-1.26571	E15.5 * Wnt1 down vs E15.5 * Sox10
10363512	Sar1a	NM_009120	0.000333268	-1.26576	E15.5 * Wnt1 down vs E15.5 * Sox10
10448307	Tnfrsf12a	NM_013749	1.42308e-005	-1.26621	E15.5 * Wnt1 down vs E15.5 * Sox10
10462973	Hells	NM_008234	0.00344185	-1.26661	E15.5 * Wnt1 down vs E15.5 * Sox10
10375382	Clint1	NM_001045520	0.000105843	-1.26668	E15.5 * Wnt1 down vs E15.5 * Sox10
10435714	Tmem39a	NM_026407	7.70208e-006	-1.26672	E15.5 * Wnt1 down vs E15.5 * Sox10
10543709	Tmem209	NM_178625	0.00249504	-1.26691	E15.5 * Wnt1 down vs E15.5 * Sox10
10500388	Polr3c	NM_028925	0.00256981	-1.26742	E15.5 * Wnt1 down vs E15.5 * Sox10
10474875	Casc5	NM_029617	0.00174517	-1.26743	E15.5 * Wnt1 down vs E15.5 * Sox10
10605674	Pola1	NM_008892	0.00127303	-1.2678	E15.5 * Wnt1 down vs E15.5 * Sox10
10548585	Csda	NM_139117	0.00247702	-1.26793	E15.5 * Wnt1 down vs E15.5 * Sox10
10538253	Mpp6	NM_019939	0.000666663	-1.26804	E15.5 * Wnt1 down vs E15.5 * Sox10
10538547	Fkbp9	NM_012056	0.00103313	-1.26811	E15.5 * Wnt1 down vs E15.5 * Sox10
10424909	Hsf1	NM_008296	0.00252777	-1.26844	E15.5 * Wnt1 down vs E15.5 * Sox10
10459576	Cep76	NM_001081073	0.00449173	-1.26846	E15.5 * Wnt1 down vs E15.5 * Sox10
10471608	Cep110	NM_012018	0.00023162	-1.26852	E15.5 * Wnt1 down vs E15.5 * Sox10
10348929	Hnrnpf	NM_133834	7.96592e-009	-1.26868	E15.5 * Wnt1 down vs E15.5 * Sox10
10502787	Rpsa	NM_011029	0.000998864	-1.26872	E15.5 * Wnt1 down vs E15.5 * Sox10
10524659	Pop5	NM_026398	0.00322062	-1.26879	E15.5 * Wnt1 down vs E15.5 * Sox10
10547251	Bms1	NM_194339	0.000279607	-1.26885	E15.5 * Wnt1 down vs E15.5 * Sox10
10384398	Grb10	NM_010345	0.00101606	-1.26913	E15.5 * Wnt1 down vs E15.5 * Sox10
10582814	Tomm20	NM_024214	0.000813149	-1.26915	E15.5 * Wnt1 down vs E15.5 * Sox10
10541104	Hnrnpf	NM_133834	4.68292e-009	-1.26923	E15.5 * Wnt1 down vs E15.5 * Sox10
10534075	Psph	NM_133900	0.000902029	-1.26937	E15.5 * Wnt1 down vs E15.5 * Sox10
10581800	Rfwd3	NM_146218	0.000395962	-1.27034	E15.5 * Wnt1 down vs E15.5 * Sox10
10354542	Pms1	NM_153556	0.000682886	-1.27049	E15.5 * Wnt1 down vs E15.5 * Sox10
10480145	Rsu1	NM_009105	0.00120887	-1.27057	E15.5 * Wnt1 down vs E15.5 * Sox10
10485070	Mdk	NM_010784	0.00326059	-1.2708	E15.5 * Wnt1 down vs E15.5 * Sox10
10578109	Ubxn8	NM_178648	0.00284507	-1.27128	E15.5 * Wnt1 down vs E15.5 * Sox10
10475610	Dut	NM_023595	0.000214911	-1.27142	E15.5 * Wnt1 down vs E15.5 * Sox10
10407811	Hnrnpf	NM_133834	3.58213e-008	-1.27159	E15.5 * Wnt1 down vs E15.5 * Sox10
10441107	Psmg1	NM_019537	0.00125142	-1.27174	E15.5 * Wnt1 down vs E15.5 * Sox10
10528915	Tyms	NM_021288	0.00132987	-1.27195	E15.5 * Wnt1 down vs E15.5 * Sox10
10530201	Ugdh	NM_009466	0.0028541	-1.27236	E15.5 * Wnt1 down vs E15.5 * Sox10
10515293	Llph	NM_025431	0.000462245	-1.27238	E15.5 * Wnt1 down vs E15.5 * Sox10
10601335	2610029G23Rik	NM_026312	0.00141518	-1.27245	E15.5 * Wnt1 down vs E15.5 * Sox10
10488589	Fam110a	NM_028666	0.00334602	-1.27247	E15.5 * Wnt1 down vs E15.5 * Sox10
10491962	Foxo1	NM_019739	0.000455487	-1.27263	E15.5 * Wnt1 down vs E15.5 * Sox10
10462632	Kif20b	NM_183046	0.000612094	-1.27275	E15.5 * Wnt1 down vs E15.5 * Sox10
10371307	Nfyb	NM_010914	0.00295934	-1.27284	E15.5 * Wnt1 down vs E15.5 * Sox10
10451363	Srf	NM_020493	0.00480219	-1.27314	E15.5 * Wnt1 down vs E15.5 * Sox10
10409202		---	0.000785772	-1.27317	E15.5 * Wnt1 down vs E15.5 * Sox10
10498284	Wwtr1	NM_133784	0.000392151	-1.27329	E15.5 * Wnt1 down vs E15.5 * Sox10
10444505	Rdbp	NM_001045864	9.14729e-005	-1.27341	E15.5 * Wnt1 down vs E15.5 * Sox10
10588137	Dzip1l	NM_028258	0.000343974	-1.27368	E15.5 * Wnt1 down vs E15.5 * Sox10
10477929	1110008F13Rik	NM_026124	0.000194103	-1.27428	E15.5 * Wnt1 down vs E15.5 * Sox10
10437934	Rpsa	NM_011029	0.000923297	-1.27444	E15.5 * Wnt1 down vs E15.5 * Sox10
10512443	Stoml2	NM_023231	0.00282447	-1.27446	E15.5 * Wnt1 down vs E15.5 * Sox10
10468722	Gfra1	NM_010279	0.00421127	-1.27459	E15.5 * Wnt1 down vs E15.5 * Sox10
10362922	Atg5	NM_053069	0.00107751	-1.27468	E15.5 * Wnt1 down vs E15.5 * Sox10
10595680	Tbc1d2b	NM_194334	0.00262263	-1.27495	E15.5 * Wnt1 down vs E15.5 * Sox10
10479026	Rae1	NM_175112	3.66988e-006	-1.27522	E15.5 * Wnt1 down vs E15.5 * Sox10

10427606	Skp2	NM_145468	0.000631091	-1.27526	E15.5 * Wnt1 down vs E15.5 * Sox10
10516605	Hdac1	NM_008228	1.56138e-005	-1.27526	E15.5 * Wnt1 down vs E15.5 * Sox10
10417013	Dnajc3	NM_008929	8.69325e-006	-1.27583	E15.5 * Wnt1 down vs E15.5 * Sox10
10417034	Dnajc3	NM_008929	8.69325e-006	-1.27583	E15.5 * Wnt1 down vs E15.5 * Sox10
10351404	Tmco1	NM_001039483	0.00118865	-1.27626	E15.5 * Wnt1 down vs E15.5 * Sox10
10443009	Ergic1	NM_026170	0.00316994	-1.27663	E15.5 * Wnt1 down vs E15.5 * Sox10
10477370	Tomm20	NM_024214	0.00195378	-1.27711	E15.5 * Wnt1 down vs E15.5 * Sox10
10421029	Cdca2	NM_175384	5.82342e-005	-1.27737	E15.5 * Wnt1 down vs E15.5 * Sox10
10497920	Ankrd50	NM_001033198	0.000192865	-1.27747	E15.5 * Wnt1 down vs E15.5 * Sox10
10391119	1110036O03Rik	NM_176830	3.51334e-005	-1.27768	E15.5 * Wnt1 down vs E15.5 * Sox10
10391768	Eftud2	NM_011431	0.0017191	-1.27777	E15.5 * Wnt1 down vs E15.5 * Sox10
10521678	Cd38	NM_007646	0.00111573	-1.27819	E15.5 * Wnt1 down vs E15.5 * Sox10
10509246	Luzp1	NM_024452	2.21124e-005	-1.27837	E15.5 * Wnt1 down vs E15.5 * Sox10
10424400	Myc	NM_010849	0.00317579	-1.27868	E15.5 * Wnt1 down vs E15.5 * Sox10
10435457	Parp9	NM_030253	0.00230541	-1.27876	E15.5 * Wnt1 down vs E15.5 * Sox10
10512704	Exosc3	NM_025513	0.000669604	-1.27881	E15.5 * Wnt1 down vs E15.5 * Sox10
10504743	Nans	NM_053179	0.00419282	-1.27892	E15.5 * Wnt1 down vs E15.5 * Sox10
10541729	Cdca3	NM_013538	0.00185152	-1.27925	E15.5 * Wnt1 down vs E15.5 * Sox10
10416355	Rcbtb2	NM_134083	0.00106021	-1.27936	E15.5 * Wnt1 down vs E15.5 * Sox10
10556266	Wee1	NM_009516	0.00293519	-1.27996	E15.5 * Wnt1 down vs E15.5 * Sox10
10420877	Esco2	NM_028039	0.00429994	-1.28012	E15.5 * Wnt1 down vs E15.5 * Sox10
10529957	Gpr125	ENSMUST00000(	0.000445545	-1.28016	E15.5 * Wnt1 down vs E15.5 * Sox10
10583145	Tmem123	NM_133739	0.000148103	-1.28036	E15.5 * Wnt1 down vs E15.5 * Sox10
10446027	Chaf1a	NM_013733	6.30873e-005	-1.28057	E15.5 * Wnt1 down vs E15.5 * Sox10
10476668	Csrp2bp	NM_181417	0.000150248	-1.28071	E15.5 * Wnt1 down vs E15.5 * Sox10
10489355	Jph2	ENSMUST00000(	0.000444598	-1.28081	E15.5 * Wnt1 down vs E15.5 * Sox10
10477994	Ctnnbl1	NM_025680	0.000378367	-1.28086	E15.5 * Wnt1 down vs E15.5 * Sox10
10588669	Rassf1	NM_019713	0.00466146	-1.28087	E15.5 * Wnt1 down vs E15.5 * Sox10
10415960	Ints9	NM_153414	0.0015465	-1.28126	E15.5 * Wnt1 down vs E15.5 * Sox10
10424347	EG665565	XM_001473789	0.00382943	-1.28148	E15.5 * Wnt1 down vs E15.5 * Sox10
10494889	Dennd2c	NM_177857	0.00129197	-1.28164	E15.5 * Wnt1 down vs E15.5 * Sox10
10561907	0610010E21Rik	NM_001033140	0.000133542	-1.28199	E15.5 * Wnt1 down vs E15.5 * Sox10
10415163	Dhrs2	NM_027790	0.00217132	-1.28208	E15.5 * Wnt1 down vs E15.5 * Sox10
10420596	Tnfrsf19	NM_013869	8.89516e-005	-1.28252	E15.5 * Wnt1 down vs E15.5 * Sox10
10564698	Det1	NM_029585	0.000616615	-1.28287	E15.5 * Wnt1 down vs E15.5 * Sox10
10372121	Tmtc3	NM_001033332	0.000603821	-1.28307	E15.5 * Wnt1 down vs E15.5 * Sox10
10447417	Msh6	NM_010830	4.01749e-005	-1.2832	E15.5 * Wnt1 down vs E15.5 * Sox10
10503617	F730047E07Rik	NM_199467	0.00146825	-1.28363	E15.5 * Wnt1 down vs E15.5 * Sox10
10500295	Plekho1	NM_023320	0.000400802	-1.28389	E15.5 * Wnt1 down vs E15.5 * Sox10
10369252	10-Sep	NM_001024911	0.000426028	-1.28396	E15.5 * Wnt1 down vs E15.5 * Sox10
10472820	Itga6	NM_008397	0.00353013	-1.28402	E15.5 * Wnt1 down vs E15.5 * Sox10
10403361	Pitrm1	NM_145131	7.34909e-005	-1.28413	E15.5 * Wnt1 down vs E15.5 * Sox10
10380689	Cbx1	NM_007622	3.64667e-006	-1.28421	E15.5 * Wnt1 down vs E15.5 * Sox10
10473281	Itgav	NM_008402	0.000328538	-1.28432	E15.5 * Wnt1 down vs E15.5 * Sox10
10479411	Ogfr	NM_031373	6.08944e-005	-1.28435	E15.5 * Wnt1 down vs E15.5 * Sox10
10540544	Thumpd3	NM_008188	0.000208126	-1.2845	E15.5 * Wnt1 down vs E15.5 * Sox10
10581547	Nob1	NM_026277	0.00221186	-1.28472	E15.5 * Wnt1 down vs E15.5 * Sox10
10447004	Hdac1	NM_008228	2.64556e-005	-1.28522	E15.5 * Wnt1 down vs E15.5 * Sox10
10465604	Stip1	NM_016737	2.7687e-005	-1.28527	E15.5 * Wnt1 down vs E15.5 * Sox10
10369630	Ddx21	NM_019553	0.000873983	-1.28568	E15.5 * Wnt1 down vs E15.5 * Sox10
10469457	Plxdc2	NM_026162	0.000108656	-1.28591	E15.5 * Wnt1 down vs E15.5 * Sox10
10510629	Nol9	NM_028727	0.000939971	-1.28593	E15.5 * Wnt1 down vs E15.5 * Sox10
10491805	Plk4	NM_011495	0.000123176	-1.28626	E15.5 * Wnt1 down vs E15.5 * Sox10
10382998	Birc5	NM_001012273	0.0025897	-1.28645	E15.5 * Wnt1 down vs E15.5 * Sox10
10582811	Irf2bp2	BC048951	0.00180624	-1.28657	E15.5 * Wnt1 down vs E15.5 * Sox10
10465011	Sart1	NM_016882	0.000311446	-1.2866	E15.5 * Wnt1 down vs E15.5 * Sox10
10414417	Peli2	NM_033602	0.00110758	-1.28682	E15.5 * Wnt1 down vs E15.5 * Sox10
10462195	Kank1	NM_181404	0.00182415	-1.2871	E15.5 * Wnt1 down vs E15.5 * Sox10
10513824	Cdk5rap2	NM_145990	6.12048e-005	-1.28715	E15.5 * Wnt1 down vs E15.5 * Sox10
10594251	Kif23	NM_024245	0.000239806	-1.28717	E15.5 * Wnt1 down vs E15.5 * Sox10
10358490	Hmcn1	NM_001024720	0.00165364	-1.28737	E15.5 * Wnt1 down vs E15.5 * Sox10
10502714	Bxdc5	NM_027371	2.66563e-006	-1.28765	E15.5 * Wnt1 down vs E15.5 * Sox10
10427035	Nr4a1	NM_010444	0.00231067	-1.28766	E15.5 * Wnt1 down vs E15.5 * Sox10
10546066	Isy1	NM_133934	0.000157003	-1.28783	E15.5 * Wnt1 down vs E15.5 * Sox10
10407742	Actn2	NM_033268	0.000107867	-1.28793	E15.5 * Wnt1 down vs E15.5 * Sox10
10418905	3110001K24Rik	NM_029389	0.00399755	-1.28815	E15.5 * Wnt1 down vs E15.5 * Sox10

10431948	Rpap3	NM_028003	0.00293738	-1.28862	E15.5 * Wnt1 down vs E15.5 * Sox10
10495359	Clcc1	NM_145543	0.000592929	-1.28895	E15.5 * Wnt1 down vs E15.5 * Sox10
10440929	Gart	NM_010256	0.00017253	-1.2891	E15.5 * Wnt1 down vs E15.5 * Sox10
10490104	Aurka	NM_011497	0.00108786	-1.28929	E15.5 * Wnt1 down vs E15.5 * Sox10
10467941	Cwf19l1	NM_001081077	0.000570406	-1.28941	E15.5 * Wnt1 down vs E15.5 * Sox10
10460631	Rela	NM_009045	0.00168723	-1.28965	E15.5 * Wnt1 down vs E15.5 * Sox10
10535184	Psmg3	NM_025604	0.00436217	-1.29034	E15.5 * Wnt1 down vs E15.5 * Sox10
10548086	Rad51ap1	NM_009013	0.00182699	-1.29092	E15.5 * Wnt1 down vs E15.5 * Sox10
10597354	Pdcd6ip	NM_011052	0.000135959	-1.29093	E15.5 * Wnt1 down vs E15.5 * Sox10
10469151	Itih5	NM_172471	0.000595719	-1.29107	E15.5 * Wnt1 down vs E15.5 * Sox10
10563303	Bax	NM_007527	0.00266635	-1.29161	E15.5 * Wnt1 down vs E15.5 * Sox10
10352767	Nek2	NM_010892	0.00271633	-1.29176	E15.5 * Wnt1 down vs E15.5 * Sox10
10370013	Gstt2	NM_010361	0.00297422	-1.29179	E15.5 * Wnt1 down vs E15.5 * Sox10
10418720	Mettl6	NM_025907	0.000153561	-1.29184	E15.5 * Wnt1 down vs E15.5 * Sox10
10355996	Slc25a5	NM_007451	0.000149333	-1.29189	E15.5 * Wnt1 down vs E15.5 * Sox10
10545583	Pole4	NM_025882	0.00099883	-1.29196	E15.5 * Wnt1 down vs E15.5 * Sox10
10354832	Ppil3	NM_027351	5.07129e-005	-1.29232	E15.5 * Wnt1 down vs E15.5 * Sox10
10436951	1190017O12Rik	NM_138743	0.000303547	-1.29246	E15.5 * Wnt1 down vs E15.5 * Sox10
10520405	Gm1040	NM_001033457	0.002046	-1.29265	E15.5 * Wnt1 down vs E15.5 * Sox10
10356859	Dtymk	NM_001105667	1.49773e-006	-1.29313	E15.5 * Wnt1 down vs E15.5 * Sox10
10556280	Swap70	NM_009302	0.000507425	-1.29324	E15.5 * Wnt1 down vs E15.5 * Sox10
10573217	Ddx39	NM_197982	5.83686e-005	-1.29339	E15.5 * Wnt1 down vs E15.5 * Sox10
10521731	Ncapg	NM_019438	0.00033693	-1.29386	E15.5 * Wnt1 down vs E15.5 * Sox10
10469312	Pter	NM_008961	0.00237652	-1.29387	E15.5 * Wnt1 down vs E15.5 * Sox10
10447065	Fam82a1	NM_201361	0.000201385	-1.29413	E15.5 * Wnt1 down vs E15.5 * Sox10
10346607	Fzd7	NM_008057	4.96894e-005	-1.29423	E15.5 * Wnt1 down vs E15.5 * Sox10
10587829	Plod2	NM_001142916	0.000188009	-1.29491	E15.5 * Wnt1 down vs E15.5 * Sox10
10600341	Emd	NM_007927	0.000670462	-1.29497	E15.5 * Wnt1 down vs E15.5 * Sox10
10502299	Nfkb1	NM_008689	0.000635663	-1.2951	E15.5 * Wnt1 down vs E15.5 * Sox10
10604842		---	0.00200338	-1.29619	E15.5 * Wnt1 down vs E15.5 * Sox10
10459844	Haus1	NM_146089	0.000369802	-1.29632	E15.5 * Wnt1 down vs E15.5 * Sox10
10479950	Cugbp2	NM_001110231	5.60292e-005	-1.29636	E15.5 * Wnt1 down vs E15.5 * Sox10
10586110	Cln6	NM_001033175	0.000122924	-1.29638	E15.5 * Wnt1 down vs E15.5 * Sox10
10390707	Top2a	NM_011623	5.45026e-006	-1.29645	E15.5 * Wnt1 down vs E15.5 * Sox10
10405185	Cks2	NM_025415	0.000419058	-1.2969	E15.5 * Wnt1 down vs E15.5 * Sox10
10502191	Ostc	NM_025509	0.000427062	-1.297	E15.5 * Wnt1 down vs E15.5 * Sox10
10590494	Kif15	NM_010620	0.000909109	-1.29708	E15.5 * Wnt1 down vs E15.5 * Sox10
10534096	Chchd2	NM_024166	0.00290695	-1.2971	E15.5 * Wnt1 down vs E15.5 * Sox10
10515257	Rad54l	NM_009015	0.00041191	-1.29713	E15.5 * Wnt1 down vs E15.5 * Sox10
10474105	Rag2	NM_009020	0.00413744	-1.29715	E15.5 * Wnt1 down vs E15.5 * Sox10
10353167	Tram1	NM_028173	2.87929e-007	-1.29744	E15.5 * Wnt1 down vs E15.5 * Sox10
10379891	Ppm1d	NM_016910	0.000100196	-1.29756	E15.5 * Wnt1 down vs E15.5 * Sox10
10447799	Igf2r	NM_010515	7.64773e-006	-1.29805	E15.5 * Wnt1 down vs E15.5 * Sox10
10437765	Cpped1	NM_146067	0.00390063	-1.29836	E15.5 * Wnt1 down vs E15.5 * Sox10
10529689	Wdr1	NM_011715	0.00162043	-1.29911	E15.5 * Wnt1 down vs E15.5 * Sox10
10392098	Ftsj3	NM_025310	0.000173806	-1.29932	E15.5 * Wnt1 down vs E15.5 * Sox10
10514201	Haus6	AK173206	0.00180791	-1.29933	E15.5 * Wnt1 down vs E15.5 * Sox10
10601473	Apool	NM_026565	0.00249698	-1.29937	E15.5 * Wnt1 down vs E15.5 * Sox10
10469936	Nrarp	NM_025980	0.000332366	-1.29941	E15.5 * Wnt1 down vs E15.5 * Sox10
10507190	4732418C07Rik	BC059213	0.000552331	-1.29944	E15.5 * Wnt1 down vs E15.5 * Sox10
10523479	Slc25a5	NM_007451	0.00011628	-1.29967	E15.5 * Wnt1 down vs E15.5 * Sox10
10362245	Epb4.1l2	NM_013511	1.3098e-006	-1.29969	E15.5 * Wnt1 down vs E15.5 * Sox10
10566723	Lmo1	NM_057173	0.000330739	-1.29982	E15.5 * Wnt1 down vs E15.5 * Sox10
10391461	Brca1	NM_009764	0.000380306	-1.29995	E15.5 * Wnt1 down vs E15.5 * Sox10
10564313	Mphosph10	NM_026483	0.00102621	-1.30053	E15.5 * Wnt1 down vs E15.5 * Sox10
10363173	Gja1	NM_010288	4.84257e-005	-1.30069	E15.5 * Wnt1 down vs E15.5 * Sox10
10421661	Gtf2f2	NM_026816	3.68627e-005	-1.30135	E15.5 * Wnt1 down vs E15.5 * Sox10
10520445	Dnajb6	NM_001037941	0.00342507	-1.30173	E15.5 * Wnt1 down vs E15.5 * Sox10
10580457	N4bp1	BC004022	0.00053361	-1.30177	E15.5 * Wnt1 down vs E15.5 * Sox10
10384373	Fignl1	NM_021891	0.000569668	-1.30179	E15.5 * Wnt1 down vs E15.5 * Sox10
10607724	Zrsr2	NM_178794	4.42118e-005	-1.30181	E15.5 * Wnt1 down vs E15.5 * Sox10
10440522	Adamts1	NM_009621	0.00096042	-1.30204	E15.5 * Wnt1 down vs E15.5 * Sox10
10537770	Zyx	NM_011777	0.00269362	-1.30249	E15.5 * Wnt1 down vs E15.5 * Sox10
10566846	Dennd5a	NM_021494	6.8491e-006	-1.30272	E15.5 * Wnt1 down vs E15.5 * Sox10
10547177	Rassf4	NM_178045	0.00250697	-1.30289	E15.5 * Wnt1 down vs E15.5 * Sox10
10568328	Vkorc1	NM_178600	1.62876e-005	-1.3034	E15.5 * Wnt1 down vs E15.5 * Sox10

10558441	Mgmt	NM_008598	0.000781136	-1.30352	E15.5 * Wnt1 down vs E15.5 * Sox10
10497209	Mrps28	NM_025434	0.00234053	-1.30374	E15.5 * Wnt1 down vs E15.5 * Sox10
10418480	Gnl3	NM_153547	0.000130833	-1.30419	E15.5 * Wnt1 down vs E15.5 * Sox10
10398173	Vrk1	NM_011705	8.86912e-006	-1.30423	E15.5 * Wnt1 down vs E15.5 * Sox10
10371846	Apaf1	NM_001042558	0.000106492	-1.30431	E15.5 * Wnt1 down vs E15.5 * Sox10
10515744	Cdc20	NM_023223	0.00187759	-1.30454	E15.5 * Wnt1 down vs E15.5 * Sox10
10509820	Rcc2	NM_173867	0.003904	-1.30487	E15.5 * Wnt1 down vs E15.5 * Sox10
10513622	Pole3	NM_021498	0.00117513	-1.30588	E15.5 * Wnt1 down vs E15.5 * Sox10
10590844	9030420J04Rik	BC137891	0.000275029	-1.30591	E15.5 * Wnt1 down vs E15.5 * Sox10
10599215	Slc25a5	NM_007451	5.87484e-005	-1.30601	E15.5 * Wnt1 down vs E15.5 * Sox10
10546805	Ddx18	NM_025860	8.13238e-005	-1.30626	E15.5 * Wnt1 down vs E15.5 * Sox10
10538832	Mad2l1	NM_019499	0.00217652	-1.30645	E15.5 * Wnt1 down vs E15.5 * Sox10
10541711	Phb2	NM_007531	9.43856e-005	-1.3067	E15.5 * Wnt1 down vs E15.5 * Sox10
10539421	Mobkl1b	NM_145571	0.000728536	-1.30692	E15.5 * Wnt1 down vs E15.5 * Sox10
10385842	Rad50	NM_009012	0.00167567	-1.30734	E15.5 * Wnt1 down vs E15.5 * Sox10
10473250	Mrpl18	NM_026310	0.00153815	-1.30749	E15.5 * Wnt1 down vs E15.5 * Sox10
10425461	Adsl	NM_009634	5.2987e-005	-1.3075	E15.5 * Wnt1 down vs E15.5 * Sox10
10526217	Rfc2	NM_020022	0.00233284	-1.3075	E15.5 * Wnt1 down vs E15.5 * Sox10
10490212	Ctsz	NM_022325	3.80176e-005	-1.30756	E15.5 * Wnt1 down vs E15.5 * Sox10
10372687	Nup107	NM_134010	1.15557e-005	-1.30796	E15.5 * Wnt1 down vs E15.5 * Sox10
10485361	B230118H07Rik	NM_026592	0.000733006	-1.30812	E15.5 * Wnt1 down vs E15.5 * Sox10
10485667	Dnajc24	NM_026992	0.00241291	-1.30814	E15.5 * Wnt1 down vs E15.5 * Sox10
10456414	Psmg2	NM_134138	0.000509352	-1.30871	E15.5 * Wnt1 down vs E15.5 * Sox10
10532954	Mlec	NM_175403	0.000333006	-1.30919	E15.5 * Wnt1 down vs E15.5 * Sox10
10490894	E2f5	NM_007892	0.00161494	-1.30927	E15.5 * Wnt1 down vs E15.5 * Sox10
10437040	Chaf1b	NM_028083	0.00102007	-1.30942	E15.5 * Wnt1 down vs E15.5 * Sox10
10389816	Tom1l1	NM_028011	0.00187648	-1.30944	E15.5 * Wnt1 down vs E15.5 * Sox10
10501608	Vcam1	NM_011693	0.0018556	-1.30983	E15.5 * Wnt1 down vs E15.5 * Sox10
10367746	Sash1	NM_175155	0.000684379	-1.30989	E15.5 * Wnt1 down vs E15.5 * Sox10
10532944	Mlec	NM_175403	0.000168227	-1.31009	E15.5 * Wnt1 down vs E15.5 * Sox10
10408477	E2f3	NM_010093	0.000611643	-1.31027	E15.5 * Wnt1 down vs E15.5 * Sox10
10445796	AI314976	NM_207219	0.000275191	-1.31042	E15.5 * Wnt1 down vs E15.5 * Sox10
10399636	Mrto4	NM_023536	0.000323787	-1.31058	E15.5 * Wnt1 down vs E15.5 * Sox10
10409021	Tpmt	NM_016785	0.00141336	-1.31061	E15.5 * Wnt1 down vs E15.5 * Sox10
10394812		---	0.000100425	-1.31082	E15.5 * Wnt1 down vs E15.5 * Sox10
10578703	Spcs3	NM_029701	1.89221e-006	-1.31115	E15.5 * Wnt1 down vs E15.5 * Sox10
10379511	Ccl2	NM_011333	0.000287025	-1.31125	E15.5 * Wnt1 down vs E15.5 * Sox10
10515884	Ppih	NM_028677	8.74529e-005	-1.31138	E15.5 * Wnt1 down vs E15.5 * Sox10
10495945	4930422G04Rik	BC030185	4.12203e-005	-1.31192	E15.5 * Wnt1 down vs E15.5 * Sox10
10591357	Eif3g	NM_016876	0.000431943	-1.31193	E15.5 * Wnt1 down vs E15.5 * Sox10
10576873	Elavl1	NM_010485	2.55252e-005	-1.31225	E15.5 * Wnt1 down vs E15.5 * Sox10
10503222	Chd7	NM_001081417	0.00221392	-1.31315	E15.5 * Wnt1 down vs E15.5 * Sox10
10592593	Tecta	NM_009347	0.00319083	-1.31342	E15.5 * Wnt1 down vs E15.5 * Sox10
10347106	Rpe	NM_025683	5.34279e-005	-1.31357	E15.5 * Wnt1 down vs E15.5 * Sox10
10567546	Crym	NM_016669	0.000939773	-1.31377	E15.5 * Wnt1 down vs E15.5 * Sox10
10508711	Taf12	NM_025579	0.00431376	-1.3138	E15.5 * Wnt1 down vs E15.5 * Sox10
10572897	Hmox1	NM_010442	0.00169266	-1.31399	E15.5 * Wnt1 down vs E15.5 * Sox10
10401136	EG230765	XR_032827	0.000486638	-1.31456	E15.5 * Wnt1 down vs E15.5 * Sox10
10358652	Hmcn1	NM_001024720	0.00270877	-1.31486	E15.5 * Wnt1 down vs E15.5 * Sox10
10568714	Mki67	NM_001081117	2.92059e-005	-1.31491	E15.5 * Wnt1 down vs E15.5 * Sox10
10411306	Polk	NM_012048	1.15643e-005	-1.31492	E15.5 * Wnt1 down vs E15.5 * Sox10
10466745	Tjp2	NM_011597	0.000815287	-1.31508	E15.5 * Wnt1 down vs E15.5 * Sox10
10421269	Sorbs3	NM_011366	0.00131673	-1.31516	E15.5 * Wnt1 down vs E15.5 * Sox10
10439710	Phldb2	NM_153412	5.0468e-006	-1.31533	E15.5 * Wnt1 down vs E15.5 * Sox10
10549097	Ldhb	NM_008492	3.93844e-006	-1.3161	E15.5 * Wnt1 down vs E15.5 * Sox10
10599880	Slitrk2	NM_198863	0.00424288	-1.31652	E15.5 * Wnt1 down vs E15.5 * Sox10
10361767	Sf3b5	NM_175102	8.20624e-005	-1.31662	E15.5 * Wnt1 down vs E15.5 * Sox10
10485282	Alkbh3	NM_026944	0.000242527	-1.3167	E15.5 * Wnt1 down vs E15.5 * Sox10
10425905	Nup50	NM_016714	9.08103e-006	-1.31733	E15.5 * Wnt1 down vs E15.5 * Sox10
10556206	Rpl27a	AF357390	0.000321095	-1.31747	E15.5 * Wnt1 down vs E15.5 * Sox10
10352320	Tmem63a	NM_144794	0.000140837	-1.31759	E15.5 * Wnt1 down vs E15.5 * Sox10
10471503	Taf1d	BC110660	0.00430223	-1.31764	E15.5 * Wnt1 down vs E15.5 * Sox10
10570432	Rpl27a	AF357390	0.000371635	-1.31788	E15.5 * Wnt1 down vs E15.5 * Sox10
10471844	Nek6	NM_021606	0.00247874	-1.31882	E15.5 * Wnt1 down vs E15.5 * Sox10
10461452	Fen1	NM_007999	0.00108393	-1.31903	E15.5 * Wnt1 down vs E15.5 * Sox10
10362767	Mical1	NM_138315	0.00125119	-1.31944	E15.5 * Wnt1 down vs E15.5 * Sox10

10589041	Impdh2	NM_011830	0.000161798	-1.31989	E15.5 * Wnt1 down vs E15.5 * Sox10
10601705	Cenpi	NM_145924	0.000427335	-1.31998	E15.5 * Wnt1 down vs E15.5 * Sox10
10367772	Samd5	NM_177271	0.000179433	-1.32019	E15.5 * Wnt1 down vs E15.5 * Sox10
10376060	Irf1	NM_008390	0.00436677	-1.32024	E15.5 * Wnt1 down vs E15.5 * Sox10
10397763	9030617O03Rik	NM_145448	0.000954981	-1.32031	E15.5 * Wnt1 down vs E15.5 * Sox10
10454786	Ctnna1	NM_009818	8.58993e-005	-1.32043	E15.5 * Wnt1 down vs E15.5 * Sox10
10496204	Cenpe	NM_173762	1.43893e-005	-1.32055	E15.5 * Wnt1 down vs E15.5 * Sox10
10586174		---	0.00186049	-1.32098	E15.5 * Wnt1 down vs E15.5 * Sox10
10408798	Tcfap2a	NM_011547	0.00405785	-1.32108	E15.5 * Wnt1 down vs E15.5 * Sox10
10593270	Ttc12	NM_172770	0.000113341	-1.32125	E15.5 * Wnt1 down vs E15.5 * Sox10
10503915	Chchd2	NM_024166	0.00228497	-1.32143	E15.5 * Wnt1 down vs E15.5 * Sox10
10459590	Ptpn2	NM_001127177	0.00154183	-1.3215	E15.5 * Wnt1 down vs E15.5 * Sox10
10353004	Cks2	NM_025415	0.000193261	-1.3219	E15.5 * Wnt1 down vs E15.5 * Sox10
10582295	Odc1	NM_013614	9.50893e-005	-1.32206	E15.5 * Wnt1 down vs E15.5 * Sox10
10554013	Chsy1	NM_001081163	0.000405036	-1.32269	E15.5 * Wnt1 down vs E15.5 * Sox10
10408850	Nedd9	NM_001111324	0.000102199	-1.32327	E15.5 * Wnt1 down vs E15.5 * Sox10
10545576	Mrpl19	NM_026490	0.000735664	-1.32343	E15.5 * Wnt1 down vs E15.5 * Sox10
10471201	Abl1	NM_001112703	0.000787104	-1.3238	E15.5 * Wnt1 down vs E15.5 * Sox10
10353733	Prim2	NM_008922	0.00164024	-1.32409	E15.5 * Wnt1 down vs E15.5 * Sox10
10575120	Sntb2	NM_009229	0.000108201	-1.3248	E15.5 * Wnt1 down vs E15.5 * Sox10
10588403	Mrpl3	NM_053159	0.000157085	-1.32511	E15.5 * Wnt1 down vs E15.5 * Sox10
10587554	Tpbg	NM_011627	0.00382754	-1.32584	E15.5 * Wnt1 down vs E15.5 * Sox10
10564978	Blm	NM_007550	0.000259049	-1.32585	E15.5 * Wnt1 down vs E15.5 * Sox10
10466410	Psat1	NM_177420	0.000632062	-1.32617	E15.5 * Wnt1 down vs E15.5 * Sox10
10492102	Spg20	NM_144895	0.00376179	-1.32648	E15.5 * Wnt1 down vs E15.5 * Sox10
10555108	Clns1a	NM_023671	1.29402e-006	-1.3268	E15.5 * Wnt1 down vs E15.5 * Sox10
10518453	Chchd2	NM_024166	0.00295889	-1.32752	E15.5 * Wnt1 down vs E15.5 * Sox10
10349081	Phlpp	BC059254	0.000301974	-1.32772	E15.5 * Wnt1 down vs E15.5 * Sox10
10478572	Ube2c	NM_026785	0.00113498	-1.3279	E15.5 * Wnt1 down vs E15.5 * Sox10
10435305	Itgb5	NM_001145884	4.51331e-005	-1.32831	E15.5 * Wnt1 down vs E15.5 * Sox10
10568217	Dctpp1	NM_023203	0.00271986	-1.32836	E15.5 * Wnt1 down vs E15.5 * Sox10
10467637	Arhgap19	NM_027667	0.000150637	-1.32865	E15.5 * Wnt1 down vs E15.5 * Sox10
10453604	Bambi	NM_026505	0.000224079	-1.32869	E15.5 * Wnt1 down vs E15.5 * Sox10
10359161	Soat1	NM_009230	8.1817e-005	-1.32876	E15.5 * Wnt1 down vs E15.5 * Sox10
10358654	Hmcn1	NM_001024720	0.00123238	-1.32878	E15.5 * Wnt1 down vs E15.5 * Sox10
10511498	Plekhf2	NM_175175	1.11421e-005	-1.32891	E15.5 * Wnt1 down vs E15.5 * Sox10
10496417	Rg9mtd2	NM_175389	0.00144452	-1.32908	E15.5 * Wnt1 down vs E15.5 * Sox10
10358607	Hmcn1	NM_001024720	0.00263247	-1.32955	E15.5 * Wnt1 down vs E15.5 * Sox10
10517706	Mrto4	NM_023536	4.00803e-005	-1.32963	E15.5 * Wnt1 down vs E15.5 * Sox10
10596812	6230427J02Rik	NM_026597	0.000640309	-1.33013	E15.5 * Wnt1 down vs E15.5 * Sox10
10571344	D8Ertd82e	NM_172911	0.00165112	-1.33022	E15.5 * Wnt1 down vs E15.5 * Sox10
10365450	Pwp1	NM_133993	0.000266947	-1.33035	E15.5 * Wnt1 down vs E15.5 * Sox10
10416510	Nufip1	NM_013745	0.000772234	-1.3308	E15.5 * Wnt1 down vs E15.5 * Sox10
10529875	Ldb2	NM_001077398	0.00393724	-1.33162	E15.5 * Wnt1 down vs E15.5 * Sox10
10412385	Mrps30	NM_021556	0.00153226	-1.33162	E15.5 * Wnt1 down vs E15.5 * Sox10
10543004	Pon2	NM_183308	0.000707456	-1.33163	E15.5 * Wnt1 down vs E15.5 * Sox10
10558325	Bccip	NM_025392	6.90974e-006	-1.33183	E15.5 * Wnt1 down vs E15.5 * Sox10
10428103	Stk3	NM_019635	6.49388e-005	-1.33237	E15.5 * Wnt1 down vs E15.5 * Sox10
10400805	Nin	NM_008697	4.09519e-006	-1.33252	E15.5 * Wnt1 down vs E15.5 * Sox10
10388160	Slc13a5	NM_001004148	0.000457266	-1.333	E15.5 * Wnt1 down vs E15.5 * Sox10
10355893	Epha4	NM_007936	0.0011359	-1.33303	E15.5 * Wnt1 down vs E15.5 * Sox10
10503202	Chd7	NM_001081417	0.00159392	-1.33311	E15.5 * Wnt1 down vs E15.5 * Sox10
10366026	Wdr51b	NM_027740	0.00274974	-1.33418	E15.5 * Wnt1 down vs E15.5 * Sox10
10480714	Uap1l1	NM_001033293	0.000673252	-1.33436	E15.5 * Wnt1 down vs E15.5 * Sox10
10358529	Hmcn1	NM_001024720	0.00358274	-1.33437	E15.5 * Wnt1 down vs E15.5 * Sox10
10377550	Trp53	NM_001127233	1.30501e-005	-1.33456	E15.5 * Wnt1 down vs E15.5 * Sox10
10554445	Prc1	NM_145150	0.000447298	-1.33469	E15.5 * Wnt1 down vs E15.5 * Sox10
10427772	Tars	NM_033074	0.000841573	-1.33538	E15.5 * Wnt1 down vs E15.5 * Sox10
10389395	Brip1	NM_178309	7.86099e-005	-1.33674	E15.5 * Wnt1 down vs E15.5 * Sox10
10424221	Wdr67	NM_001081396	0.000634395	-1.33732	E15.5 * Wnt1 down vs E15.5 * Sox10
10467013	Prkg1	NM_001013833	0.000365049	-1.33733	E15.5 * Wnt1 down vs E15.5 * Sox10
10399575		---	0.00450301	-1.33757	E15.5 * Wnt1 down vs E15.5 * Sox10
10545672	Mthfd2	NM_008638	0.00231101	-1.33794	E15.5 * Wnt1 down vs E15.5 * Sox10
10473547	Srp9	NM_012058	1.48564e-006	-1.33803	E15.5 * Wnt1 down vs E15.5 * Sox10
10427744	Rai14	NM_030690	0.000274054	-1.33953	E15.5 * Wnt1 down vs E15.5 * Sox10
10461723	4632417K18Rik	NM_026640	0.00375134	-1.33981	E15.5 * Wnt1 down vs E15.5 * Sox10

10459755	2810433K01Rik	NM_025581	6.40899e-006	-1.34043	E15.5 * Wnt1 down vs E15.5 * Sox10
10357436	Mcm6	NM_008567	1.044e-007	-1.34098	E15.5 * Wnt1 down vs E15.5 * Sox10
10389269	Aatf	NM_019816	1.72745e-005	-1.34116	E15.5 * Wnt1 down vs E15.5 * Sox10
10488237	Snx5	NM_024225	1.63517e-006	-1.34136	E15.5 * Wnt1 down vs E15.5 * Sox10
10554463	Hddc3	NM_026812	0.000105789	-1.34139	E15.5 * Wnt1 down vs E15.5 * Sox10
10385325	Pttg1	NM_013917	0.000950069	-1.34191	E15.5 * Wnt1 down vs E15.5 * Sox10
10412394	Nnt	NR_003544	0.000129227	-1.34212	E15.5 * Wnt1 down vs E15.5 * Sox10
10416269	Ppp3cc	ENSMUST00000(	6.46927e-005	-1.34212	E15.5 * Wnt1 down vs E15.5 * Sox10
10579347	Ifi30	NM_023065	0.00139336	-1.34214	E15.5 * Wnt1 down vs E15.5 * Sox10
10554281	Fanci	NM_145946	0.000552228	-1.34238	E15.5 * Wnt1 down vs E15.5 * Sox10
10493692	Rab13	NM_026677	0.00096604	-1.34242	E15.5 * Wnt1 down vs E15.5 * Sox10
10467258	Myof	NM_001099634	0.000249743	-1.34277	E15.5 * Wnt1 down vs E15.5 * Sox10
10408656	Peci	NM_011868	0.00111683	-1.343	E15.5 * Wnt1 down vs E15.5 * Sox10
10568480	Nsmce4a	ENSMUST00000(	0.00102066	-1.34347	E15.5 * Wnt1 down vs E15.5 * Sox10
10503206	Chd7	NM_001081417	0.00373775	-1.3435	E15.5 * Wnt1 down vs E15.5 * Sox10
10461921	2410127L17Rik	NM_026120	0.000980952	-1.34368	E15.5 * Wnt1 down vs E15.5 * Sox10
10587508	Ttk	NM_009445	5.0771e-006	-1.34402	E15.5 * Wnt1 down vs E15.5 * Sox10
10476362	Mcm8	NM_025676	8.26659e-005	-1.34431	E15.5 * Wnt1 down vs E15.5 * Sox10
10500837	Dclre1b	NM_133865	0.0016412	-1.34484	E15.5 * Wnt1 down vs E15.5 * Sox10
10347232	Xrcc5	NM_009533	2.05884e-005	-1.34594	E15.5 * Wnt1 down vs E15.5 * Sox10
10375497		---	0.00109298	-1.34633	E15.5 * Wnt1 down vs E15.5 * Sox10
10558454	Glrx3	NM_023140	0.00133427	-1.34639	E15.5 * Wnt1 down vs E15.5 * Sox10
10413059	Vcl	NM_009502	0.00410377	-1.34687	E15.5 * Wnt1 down vs E15.5 * Sox10
10434441	Ece2	NM_025462	0.00154198	-1.34697	E15.5 * Wnt1 down vs E15.5 * Sox10
10440427	D16Ertd472e	NM_025967	0.00250548	-1.347	E15.5 * Wnt1 down vs E15.5 * Sox10
10498620	Trim59	NM_025863	0.000727526	-1.34706	E15.5 * Wnt1 down vs E15.5 * Sox10
10507328	Prdx1	NM_011034	0.000357657	-1.34709	E15.5 * Wnt1 down vs E15.5 * Sox10
10403727	Gli3	NM_008130	0.000722661	-1.34719	E15.5 * Wnt1 down vs E15.5 * Sox10
10379957	Rnft1	NM_029788	0.000273482	-1.3475	E15.5 * Wnt1 down vs E15.5 * Sox10
10520650	Cad	NM_023525	0.00127628	-1.34751	E15.5 * Wnt1 down vs E15.5 * Sox10
10463296	Cutc	NM_001113562	0.00106288	-1.34798	E15.5 * Wnt1 down vs E15.5 * Sox10
10431812	Nell2	NM_016743	0.000972499	-1.34814	E15.5 * Wnt1 down vs E15.5 * Sox10
10375313	Ccnjl	NM_001045530	0.00152606	-1.34828	E15.5 * Wnt1 down vs E15.5 * Sox10
10597461	Cmtm7	NM_133978	0.00192974	-1.34835	E15.5 * Wnt1 down vs E15.5 * Sox10
10514732	Slc35d1	NM_177732	0.0010218	-1.34884	E15.5 * Wnt1 down vs E15.5 * Sox10
10525419	P2rx7	NM_011027	1.88903e-005	-1.3491	E15.5 * Wnt1 down vs E15.5 * Sox10
10413710	Nt5dc2	NM_027289	0.00191239	-1.3492	E15.5 * Wnt1 down vs E15.5 * Sox10
10409259		---	0.00456854	-1.34922	E15.5 * Wnt1 down vs E15.5 * Sox10
10365288	Taf10	NM_020024	0.00165092	-1.34925	E15.5 * Wnt1 down vs E15.5 * Sox10
10538590	Herc5	ENSMUST00000(	0.000554414	-1.34948	E15.5 * Wnt1 down vs E15.5 * Sox10
10413086	Adk	NM_134079	5.29918e-005	-1.34957	E15.5 * Wnt1 down vs E15.5 * Sox10
10358609	Hmcn1	NM_001024720	0.00467067	-1.3498	E15.5 * Wnt1 down vs E15.5 * Sox10
10537712	Gstk1	NM_029555	0.00147124	-1.34989	E15.5 * Wnt1 down vs E15.5 * Sox10
10519324	Cdk6	NM_009873	0.000194711	-1.34994	E15.5 * Wnt1 down vs E15.5 * Sox10
10353438	Tram2	NM_133252	0.00357277	-1.35005	E15.5 * Wnt1 down vs E15.5 * Sox10
10513583	Cdc26	NM_139291	3.41236e-005	-1.35171	E15.5 * Wnt1 down vs E15.5 * Sox10
10357870	Prelp	NM_054077	2.71162e-005	-1.352	E15.5 * Wnt1 down vs E15.5 * Sox10
10390505	Rpl23	AF357384	0.00428195	-1.35213	E15.5 * Wnt1 down vs E15.5 * Sox10
10404531	Psmg4	NM_001101430	0.00376874	-1.35235	E15.5 * Wnt1 down vs E15.5 * Sox10
10509777	Iffo2	NM_183148	0.000680223	-1.35239	E15.5 * Wnt1 down vs E15.5 * Sox10
10404827	Nol7	NM_023554	0.000168014	-1.35288	E15.5 * Wnt1 down vs E15.5 * Sox10
10554960	Fam181b	NM_021427	0.00164704	-1.35299	E15.5 * Wnt1 down vs E15.5 * Sox10
10411728	Cenph	NM_021886	3.98494e-005	-1.35385	E15.5 * Wnt1 down vs E15.5 * Sox10
10496605	Ccbl2	NM_173763	0.00359968	-1.35403	E15.5 * Wnt1 down vs E15.5 * Sox10
10512949	Abca1	NM_013454	3.96239e-005	-1.35488	E15.5 * Wnt1 down vs E15.5 * Sox10
10407053	2810008M24Rik	BC004049	0.000132329	-1.35526	E15.5 * Wnt1 down vs E15.5 * Sox10
10435271	Heg1	NM_175256	2.718e-005	-1.35556	E15.5 * Wnt1 down vs E15.5 * Sox10
10374364	Akt2	NM_001110208	0.000806666	-1.35582	E15.5 * Wnt1 down vs E15.5 * Sox10
10444595	Lsm2	NM_030597	0.000887501	-1.35627	E15.5 * Wnt1 down vs E15.5 * Sox10
10405727	2410127L17Rik	NM_026120	0.00147483	-1.35639	E15.5 * Wnt1 down vs E15.5 * Sox10
10412481	2410127L17Rik	NM_026120	0.00147483	-1.35639	E15.5 * Wnt1 down vs E15.5 * Sox10
10542791	Ppfibp1	NM_026221	0.000198363	-1.35661	E15.5 * Wnt1 down vs E15.5 * Sox10
10560624	Apoe	NM_009696	0.00022038	-1.35672	E15.5 * Wnt1 down vs E15.5 * Sox10
10399391	Gen1	NM_177331	0.00179197	-1.35687	E15.5 * Wnt1 down vs E15.5 * Sox10
10359851	Uck2	NM_030724	0.000318728	-1.35688	E15.5 * Wnt1 down vs E15.5 * Sox10
10573583	Man2b1	NM_010764	0.000286129	-1.35705	E15.5 * Wnt1 down vs E15.5 * Sox10

10595205	2410127L17Rik	NM_026120	0.000437374	-1.35716	E15.5 * Wnt1 down vs E15.5 * Sox10
10591816	Dpy19l1	NM_172920	0.00149711	-1.35723	E15.5 * Wnt1 down vs E15.5 * Sox10
10358637	Hmcn1	NM_001024720	0.00356678	-1.35736	E15.5 * Wnt1 down vs E15.5 * Sox10
10574471	Cmtm3	NM_024217	0.000188827	-1.35796	E15.5 * Wnt1 down vs E15.5 * Sox10
10485645	Rcn1	NM_009037	9.926e-006	-1.35819	E15.5 * Wnt1 down vs E15.5 * Sox10
10378088	Mybbp1a	NM_016776	0.00374465	-1.35821	E15.5 * Wnt1 down vs E15.5 * Sox10
10509127	Fuca1	NM_024243	0.000317446	-1.35853	E15.5 * Wnt1 down vs E15.5 * Sox10
10375031	Snrnp25	NM_030093	0.000318594	-1.35873	E15.5 * Wnt1 down vs E15.5 * Sox10
10369815	Cdc2a	NM_007659	0.000829222	-1.35877	E15.5 * Wnt1 down vs E15.5 * Sox10
10352267	Lin9	NM_001103182	0.000244519	-1.35923	E15.5 * Wnt1 down vs E15.5 * Sox10
10492428	Tiparp	NM_178892	0.000297313	-1.3596	E15.5 * Wnt1 down vs E15.5 * Sox10
10472782	Hat1	NM_026115	7.7171e-005	-1.36015	E15.5 * Wnt1 down vs E15.5 * Sox10
10394819	LOC677565	XR_034928	0.000152358	-1.36069	E15.5 * Wnt1 down vs E15.5 * Sox10
10357579	Mapkapk2	NM_008551	0.00222931	-1.36085	E15.5 * Wnt1 down vs E15.5 * Sox10
10421877	Diap3	NM_019670	1.40307e-005	-1.36103	E15.5 * Wnt1 down vs E15.5 * Sox10
10441706	1700010I14Rik	NM_025851	0.00176535	-1.36108	E15.5 * Wnt1 down vs E15.5 * Sox10
10503190	Chd7	NM_001081417	0.00178298	-1.36167	E15.5 * Wnt1 down vs E15.5 * Sox10
10491977	2810046L04Rik	NM_173382	0.000392288	-1.36168	E15.5 * Wnt1 down vs E15.5 * Sox10
10465861	Incenp	NM_016692	0.000168015	-1.36184	E15.5 * Wnt1 down vs E15.5 * Sox10
10390050	Eme1	NM_177752	0.00233725	-1.36212	E15.5 * Wnt1 down vs E15.5 * Sox10
10382912	09-Sep	NM_001113486	6.47488e-005	-1.36238	E15.5 * Wnt1 down vs E15.5 * Sox10
10451472	Rpl7l1	NM_025433	0.000205566	-1.36244	E15.5 * Wnt1 down vs E15.5 * Sox10
10472916	Cdca7	NM_025866	0.00383608	-1.36293	E15.5 * Wnt1 down vs E15.5 * Sox10
10492021	Postn	NM_015784	0.000376238	-1.36295	E15.5 * Wnt1 down vs E15.5 * Sox10
10475981	Chchd5	NM_025395	0.00139888	-1.36351	E15.5 * Wnt1 down vs E15.5 * Sox10
10510391	Srm	NM_009272	0.000394501	-1.36402	E15.5 * Wnt1 down vs E15.5 * Sox10
10554325	5730590G19Rik	NM_029835	0.00240277	-1.36433	E15.5 * Wnt1 down vs E15.5 * Sox10
10518408	Plod1	NM_011122	0.00352809	-1.36435	E15.5 * Wnt1 down vs E15.5 * Sox10
10393955	Dus1l	NM_026824	0.000353087	-1.36474	E15.5 * Wnt1 down vs E15.5 * Sox10
10494551	Acp6	NM_019800	0.00201153	-1.36487	E15.5 * Wnt1 down vs E15.5 * Sox10
10527801	Brca2	NM_001081001	0.000770024	-1.36505	E15.5 * Wnt1 down vs E15.5 * Sox10
10565862	Pold3	NM_133692	0.000849088	-1.36534	E15.5 * Wnt1 down vs E15.5 * Sox10
10464754	Rhod	NM_007485	0.00371836	-1.36536	E15.5 * Wnt1 down vs E15.5 * Sox10
10560408	Psg17	NM_007677	0.00482624	-1.36561	E15.5 * Wnt1 down vs E15.5 * Sox10
10479765	Suv39h2	NM_022724	3.10743e-006	-1.36614	E15.5 * Wnt1 down vs E15.5 * Sox10
10584422	Olfr908	NM_146872	0.000184642	-1.36635	E15.5 * Wnt1 down vs E15.5 * Sox10
10463535	Nolc1	NM_053086	1.55167e-005	-1.36773	E15.5 * Wnt1 down vs E15.5 * Sox10
10436456	Pros1	NM_011173	0.000794149	-1.36777	E15.5 * Wnt1 down vs E15.5 * Sox10
10506870	Txndc12	NM_025334	0.000526367	-1.36778	E15.5 * Wnt1 down vs E15.5 * Sox10
10557992	Bag3	NM_013863	0.000114913	-1.36829	E15.5 * Wnt1 down vs E15.5 * Sox10
10344679	St18	NM_173868	0.00157085	-1.36916	E15.5 * Wnt1 down vs E15.5 * Sox10
10408321	Gmnn	NM_020567	0.00128967	-1.36925	E15.5 * Wnt1 down vs E15.5 * Sox10
10566767	St5	NM_001001326	0.000659486	-1.3693	E15.5 * Wnt1 down vs E15.5 * Sox10
10495094	6530418L21Rik	BC052371	0.000346019	-1.37027	E15.5 * Wnt1 down vs E15.5 * Sox10
10415045	Mrpl52	NM_026851	0.00020024	-1.37046	E15.5 * Wnt1 down vs E15.5 * Sox10
10454077	Taf4b	NM_001100449	0.000176162	-1.3705	E15.5 * Wnt1 down vs E15.5 * Sox10
10563780	E2f8	NM_001013368	3.58604e-005	-1.37074	E15.5 * Wnt1 down vs E15.5 * Sox10
10573615	Orc6l	NM_019716	2.38852e-005	-1.37101	E15.5 * Wnt1 down vs E15.5 * Sox10
10367641	Mthfd1l	NM_172308	1.14075e-006	-1.37138	E15.5 * Wnt1 down vs E15.5 * Sox10
10587503	Sh3bgrl2	NM_172507	0.00144694	-1.37149	E15.5 * Wnt1 down vs E15.5 * Sox10
10358549	Hmcn1	NM_001024720	0.0038264	-1.37173	E15.5 * Wnt1 down vs E15.5 * Sox10
10415875	2610028A01Rik	NM_028228	0.00262184	-1.37309	E15.5 * Wnt1 down vs E15.5 * Sox10
10474769	Bub1b	NM_009773	0.000100769	-1.3733	E15.5 * Wnt1 down vs E15.5 * Sox10
10416215	Loxl2	NM_033325	0.00280641	-1.37452	E15.5 * Wnt1 down vs E15.5 * Sox10
10547015	1500001M20Rik	NM_026894	3.1326e-005	-1.37503	E15.5 * Wnt1 down vs E15.5 * Sox10
10521555	Lyar	NM_025281	1.30772e-006	-1.3751	E15.5 * Wnt1 down vs E15.5 * Sox10
10521690	Ppih	NM_028677	4.53757e-005	-1.37565	E15.5 * Wnt1 down vs E15.5 * Sox10
10445565	Mrpl2	NM_025302	0.000408413	-1.37572	E15.5 * Wnt1 down vs E15.5 * Sox10
10358733	Rgl1	NM_016846	0.000587764	-1.376	E15.5 * Wnt1 down vs E15.5 * Sox10
10462702	Hectd2	NM_172637	0.00323272	-1.37606	E15.5 * Wnt1 down vs E15.5 * Sox10
10483401	Spc25	NM_025565	8.99541e-005	-1.37685	E15.5 * Wnt1 down vs E15.5 * Sox10
10474619	Fmn1	NM_010230	0.0019247	-1.37687	E15.5 * Wnt1 down vs E15.5 * Sox10
10447589	Fbl	NM_007991	3.87757e-006	-1.37711	E15.5 * Wnt1 down vs E15.5 * Sox10
10525591	Kntc1	NM_001042421	2.13669e-007	-1.37716	E15.5 * Wnt1 down vs E15.5 * Sox10
10501629	Cdc14a	NM_001080818	6.85683e-006	-1.37804	E15.5 * Wnt1 down vs E15.5 * Sox10
10568668	Adam12	NM_007400	0.00119535	-1.37869	E15.5 * Wnt1 down vs E15.5 * Sox10

10380381	Tob1	NM_009427	0.000655	-1.37935	E15.5 * Wnt1 down vs E15.5 * Sox10
10530421	Gabra4	NM_010251	0.0004997	-1.38014	E15.5 * Wnt1 down vs E15.5 * Sox10
10436428	Mina	NM_025910	0.000715571	-1.38017	E15.5 * Wnt1 down vs E15.5 * Sox10
10474984	Nusap1	NM_133851	0.000843153	-1.38025	E15.5 * Wnt1 down vs E15.5 * Sox10
10429452	Sf3b4	NM_153053	0.000358319	-1.38095	E15.5 * Wnt1 down vs E15.5 * Sox10
10409434	Lman2	NM_025828	0.000613819	-1.38158	E15.5 * Wnt1 down vs E15.5 * Sox10
10351347	Creg1	NM_011804	0.000479706	-1.38184	E15.5 * Wnt1 down vs E15.5 * Sox10
10433885	Cebpd	NM_007679	0.000694284	-1.38261	E15.5 * Wnt1 down vs E15.5 * Sox10
10594404	Smad3	NM_016769	0.000121425	-1.38285	E15.5 * Wnt1 down vs E15.5 * Sox10
10360934	Rrp15	NM_026041	6.90529e-006	-1.38365	E15.5 * Wnt1 down vs E15.5 * Sox10
10360985	Cenpf	NM_001081363	1.13709e-005	-1.38403	E15.5 * Wnt1 down vs E15.5 * Sox10
10542745	Fgfr1op2	NM_026218	0.000227824	-1.38417	E15.5 * Wnt1 down vs E15.5 * Sox10
10453216	Thumpd2	BC065413	0.000322821	-1.38418	E15.5 * Wnt1 down vs E15.5 * Sox10
10571142	Gpr124	NM_054044	0.000405057	-1.38466	E15.5 * Wnt1 down vs E15.5 * Sox10
10487894	Rassf2	NM_175445	0.00049229	-1.38478	E15.5 * Wnt1 down vs E15.5 * Sox10
10418171	Zcchc24	NM_001101433	0.000108455	-1.38496	E15.5 * Wnt1 down vs E15.5 * Sox10
10394978	Rrm2	NM_009104	1.37166e-007	-1.38584	E15.5 * Wnt1 down vs E15.5 * Sox10
10389005	1110002N22Rik	NM_183275	0.00172965	-1.38601	E15.5 * Wnt1 down vs E15.5 * Sox10
10359890	Nuf2	NM_023284	0.00140767	-1.38602	E15.5 * Wnt1 down vs E15.5 * Sox10
10373814	Pes1	NM_022889	0.000796853	-1.38616	E15.5 * Wnt1 down vs E15.5 * Sox10
10578123	Rbpms	NM_019733	8.15284e-005	-1.38624	E15.5 * Wnt1 down vs E15.5 * Sox10
10355403	Fn1	NM_010233	0.0021681	-1.38639	E15.5 * Wnt1 down vs E15.5 * Sox10
10447702	Ppih	NM_028677	8.99354e-005	-1.38642	E15.5 * Wnt1 down vs E15.5 * Sox10
10584142	Ets1	NM_011808	8.58437e-005	-1.38722	E15.5 * Wnt1 down vs E15.5 * Sox10
10553993	Snrpa1	NM_021336	7.602e-007	-1.38738	E15.5 * Wnt1 down vs E15.5 * Sox10
10552075	Lgi4	NM_144556	0.00103565	-1.38749	E15.5 * Wnt1 down vs E15.5 * Sox10
10446074	Uhrf1	NM_010931	0.000313164	-1.3875	E15.5 * Wnt1 down vs E15.5 * Sox10
10497520	Ect2	NM_007900	0.00365915	-1.38761	E15.5 * Wnt1 down vs E15.5 * Sox10
10469070	Nudt5	NM_016918	0.000158834	-1.38776	E15.5 * Wnt1 down vs E15.5 * Sox10
10466976	Gldc	NM_138595	0.000472952	-1.38779	E15.5 * Wnt1 down vs E15.5 * Sox10
10358523	Hmcn1	NM_001024720	0.000900773	-1.38881	E15.5 * Wnt1 down vs E15.5 * Sox10
10573261	Asf1b	NM_024184	0.000850573	-1.38923	E15.5 * Wnt1 down vs E15.5 * Sox10
10539135	Capg	NM_007599	9.8169e-005	-1.38993	E15.5 * Wnt1 down vs E15.5 * Sox10
10505894	Mtap	NM_024433	2.1372e-005	-1.39047	E15.5 * Wnt1 down vs E15.5 * Sox10
10358611	Hmcn1	NM_001024720	0.0011736	-1.39065	E15.5 * Wnt1 down vs E15.5 * Sox10
10479811	Mcm10	NM_027290	1.30437e-005	-1.39072	E15.5 * Wnt1 down vs E15.5 * Sox10
10477187	Tpx2	NM_001141977	4.93041e-005	-1.39077	E15.5 * Wnt1 down vs E15.5 * Sox10
10568150	Kif22	NM_145588	0.00131091	-1.39103	E15.5 * Wnt1 down vs E15.5 * Sox10
10396476	Rhoj	NM_023275	0.00100367	-1.39106	E15.5 * Wnt1 down vs E15.5 * Sox10
10543031	Slc25a13	NM_015829	6.98009e-007	-1.39128	E15.5 * Wnt1 down vs E15.5 * Sox10
10464425	Grk5	NM_018869	0.000399618	-1.39289	E15.5 * Wnt1 down vs E15.5 * Sox10
10515431	Kif2c	NM_134471	0.00318804	-1.39308	E15.5 * Wnt1 down vs E15.5 * Sox10
10607143	Capn6	NM_007603	1.69457e-005	-1.3932	E15.5 * Wnt1 down vs E15.5 * Sox10
10434925	Hes1	NM_008235	9.68771e-006	-1.39343	E15.5 * Wnt1 down vs E15.5 * Sox10
10520521	Cenpa	NM_007681	2.32817e-005	-1.39355	E15.5 * Wnt1 down vs E15.5 * Sox10
10553897	Mtmr10	NM_172742	0.0047107	-1.39436	E15.5 * Wnt1 down vs E15.5 * Sox10
10595702	1190002N15Rik	NM_001033145	0.000369425	-1.39492	E15.5 * Wnt1 down vs E15.5 * Sox10
10441456	Synj2	NM_011523	0.00011729	-1.39497	E15.5 * Wnt1 down vs E15.5 * Sox10
10556463	Arntl	NM_007489	7.28446e-005	-1.395	E15.5 * Wnt1 down vs E15.5 * Sox10
10441436	Snx9	NM_025664	9.97326e-006	-1.39504	E15.5 * Wnt1 down vs E15.5 * Sox10
10399973	Hdac9	NM_024124	3.1505e-005	-1.39511	E15.5 * Wnt1 down vs E15.5 * Sox10
10407709	Mtr	NM_001081128	0.00026566	-1.39518	E15.5 * Wnt1 down vs E15.5 * Sox10
10363575	Dna2	NM_177372	7.87341e-007	-1.39541	E15.5 * Wnt1 down vs E15.5 * Sox10
10487480	Bub1	NM_001113179	1.88219e-005	-1.39552	E15.5 * Wnt1 down vs E15.5 * Sox10
10452556	Rab12	NM_024448	0.00298167	-1.39558	E15.5 * Wnt1 down vs E15.5 * Sox10
10493103	Isg20l2	NM_177663	0.000931625	-1.39579	E15.5 * Wnt1 down vs E15.5 * Sox10
10584710	H2afx	NM_010436	0.000155787	-1.39618	E15.5 * Wnt1 down vs E15.5 * Sox10
10474902	Rad51	NM_011234	0.000180194	-1.3964	E15.5 * Wnt1 down vs E15.5 * Sox10
10433721	Nde1	NM_023317	9.52067e-006	-1.39644	E15.5 * Wnt1 down vs E15.5 * Sox10
10482059	Ggta1	NM_010283	0.00157498	-1.39647	E15.5 * Wnt1 down vs E15.5 * Sox10
10425945	Fbln1	NM_010180	0.000781984	-1.39671	E15.5 * Wnt1 down vs E15.5 * Sox10
10476989	Gins1	BC027537	0.000428473	-1.39679	E15.5 * Wnt1 down vs E15.5 * Sox10
10544906	Ggct	NM_026637	0.000720513	-1.39702	E15.5 * Wnt1 down vs E15.5 * Sox10
10438738	Bcl6	NM_009744	0.000149849	-1.39821	E15.5 * Wnt1 down vs E15.5 * Sox10
10500445	Chd1l	NM_026539	0.000324498	-1.39855	E15.5 * Wnt1 down vs E15.5 * Sox10
10497831	Ccna2	NM_009828	8.64143e-007	-1.39867	E15.5 * Wnt1 down vs E15.5 * Sox10

10397148	Acot1	NM_012006	0.00195346	-1.39882	E15.5 * Wnt1 down vs E15.5 * Sox10
10367076	Prim1	NM_008921	4.65619e-005	-1.39891	E15.5 * Wnt1 down vs E15.5 * Sox10
10443836	Rrp1b	NM_028244	5.49017e-005	-1.39893	E15.5 * Wnt1 down vs E15.5 * Sox10
10498319	Serp1	NM_030685	3.89791e-006	-1.39923	E15.5 * Wnt1 down vs E15.5 * Sox10
10529344	Poln	NM_181857	1.32074e-005	-1.39948	E15.5 * Wnt1 down vs E15.5 * Sox10
10424188	Mtbp	NM_134092	0.000172321	-1.40011	E15.5 * Wnt1 down vs E15.5 * Sox10
10451679	Daam2	NM_001008231	0.000348714	-1.40025	E15.5 * Wnt1 down vs E15.5 * Sox10
10449236	Nme4	NM_019731	0.00181389	-1.40042	E15.5 * Wnt1 down vs E15.5 * Sox10
10514561	E130114P18Rik	ENSMUST00000(	0.00182192	-1.40093	E15.5 * Wnt1 down vs E15.5 * Sox10
10379779	Ddx52	NM_030096	0.000572397	-1.40106	E15.5 * Wnt1 down vs E15.5 * Sox10
10539882	Ruvbl1	NM_019685	4.36849e-006	-1.40119	E15.5 * Wnt1 down vs E15.5 * Sox10
10466779	Pip5k1b	NM_008846	0.000851092	-1.40188	E15.5 * Wnt1 down vs E15.5 * Sox10
10588294	Topbp1	NM_176979	1.36024e-007	-1.40254	E15.5 * Wnt1 down vs E15.5 * Sox10
10391444	1700113i22rik	NM_026865	0.00357423	-1.40262	E15.5 * Wnt1 down vs E15.5 * Sox10
10607877	Prps2	NM_026662	0.000120355	-1.40268	E15.5 * Wnt1 down vs E15.5 * Sox10
10550316	Tmem160	NM_026938	3.47178e-005	-1.4029	E15.5 * Wnt1 down vs E15.5 * Sox10
10403584	Nid1	NM_010917	8.54796e-005	-1.40369	E15.5 * Wnt1 down vs E15.5 * Sox10
10549200	Sox5	NM_011444	0.000770868	-1.40382	E15.5 * Wnt1 down vs E15.5 * Sox10
10465912	Fen1	NM_007999	0.000163732	-1.40442	E15.5 * Wnt1 down vs E15.5 * Sox10
10450519	Tcf19	NM_025674	7.93899e-006	-1.40506	E15.5 * Wnt1 down vs E15.5 * Sox10
10404152	Fam65b	NM_029679	0.00104477	-1.4052	E15.5 * Wnt1 down vs E15.5 * Sox10
10491399	Mrpl47	NM_029017	0.000279545	-1.40638	E15.5 * Wnt1 down vs E15.5 * Sox10
10361651	Nup43	NM_145706	1.71239e-005	-1.40653	E15.5 * Wnt1 down vs E15.5 * Sox10
10506170	Efcab7	NM_145549	0.00308145	-1.40676	E15.5 * Wnt1 down vs E15.5 * Sox10
10454709	Kif20a	NM_009004	5.21545e-008	-1.40689	E15.5 * Wnt1 down vs E15.5 * Sox10
10539433	Mobkl1b	ENSMUST00000(	0.00021108	-1.4069	E15.5 * Wnt1 down vs E15.5 * Sox10
10604616	Plac1	NM_019538	0.000197913	-1.40694	E15.5 * Wnt1 down vs E15.5 * Sox10
10513061	Ctnnal1	NM_018761	1.80938e-005	-1.40736	E15.5 * Wnt1 down vs E15.5 * Sox10
10358531	Hmcn1	NM_001024720	0.00327069	-1.40824	E15.5 * Wnt1 down vs E15.5 * Sox10
10358648	Hmcn1	NM_001024720	0.00281283	-1.40854	E15.5 * Wnt1 down vs E15.5 * Sox10
10538811	Prdm5	NM_027547	5.36624e-005	-1.40865	E15.5 * Wnt1 down vs E15.5 * Sox10
10590860	9030420J04Rik	BC137891	0.000445007	-1.40882	E15.5 * Wnt1 down vs E15.5 * Sox10
10523516	4930524J08Rik	ENSMUST00000(	0.0017677	-1.40888	E15.5 * Wnt1 down vs E15.5 * Sox10
10574378	Gins3	NM_030198	0.000305889	-1.40906	E15.5 * Wnt1 down vs E15.5 * Sox10
10440419	Btg3	NM_009770	0.000971957	-1.40941	E15.5 * Wnt1 down vs E15.5 * Sox10
10547758	Emg1	NM_013536	5.48666e-006	-1.40941	E15.5 * Wnt1 down vs E15.5 * Sox10
10386909	Cenpv	NM_028448	0.000551058	-1.41004	E15.5 * Wnt1 down vs E15.5 * Sox10
10434782	Lpp	NM_178665	0.000307467	-1.41052	E15.5 * Wnt1 down vs E15.5 * Sox10
10475890	Mertk	NM_008587	0.000702469	-1.41052	E15.5 * Wnt1 down vs E15.5 * Sox10
10371676	Utp20	NM_175158	8.20973e-007	-1.41123	E15.5 * Wnt1 down vs E15.5 * Sox10
10586907	Mns1	NM_008613	5.31516e-006	-1.41124	E15.5 * Wnt1 down vs E15.5 * Sox10
10495935	4930422G04Rik	BC085187	1.44617e-005	-1.41148	E15.5 * Wnt1 down vs E15.5 * Sox10
10557519	Hirip3	NM_172746	8.75335e-005	-1.4116	E15.5 * Wnt1 down vs E15.5 * Sox10
10373467	Erbb3	NM_010153	0.00388994	-1.41166	E15.5 * Wnt1 down vs E15.5 * Sox10
10361323	Cnksr3	NM_172546	0.000192041	-1.41223	E15.5 * Wnt1 down vs E15.5 * Sox10
10542275	Etv6	NM_007961	0.000180844	-1.41228	E15.5 * Wnt1 down vs E15.5 * Sox10
10428698	Sntb1	NM_016667	0.000228605	-1.41249	E15.5 * Wnt1 down vs E15.5 * Sox10
10502830	Nexn	NM_199465	0.0046908	-1.41355	E15.5 * Wnt1 down vs E15.5 * Sox10
10454950	Slc35a4	NM_026404	5.33228e-005	-1.41568	E15.5 * Wnt1 down vs E15.5 * Sox10
10600476	EG245472	XM_141845	0.000940689	-1.41578	E15.5 * Wnt1 down vs E15.5 * Sox10
10415021	Abhd4	NM_134076	2.97633e-005	-1.41611	E15.5 * Wnt1 down vs E15.5 * Sox10
10358613	Hmcn1	NM_001024720	0.00376113	-1.41618	E15.5 * Wnt1 down vs E15.5 * Sox10
10349637	2700049P18Rik	NM_175382	0.00115188	-1.41626	E15.5 * Wnt1 down vs E15.5 * Sox10
10403076		---	5.35586e-005	-1.41666	E15.5 * Wnt1 down vs E15.5 * Sox10
10481272	1700007K13Rik	BC099566	0.001248	-1.41667	E15.5 * Wnt1 down vs E15.5 * Sox10
10574166	Cpne2	NM_153507	1.37926e-005	-1.41669	E15.5 * Wnt1 down vs E15.5 * Sox10
10552311	OTTMUSG00000	XR_030737	4.36797e-005	-1.41671	E15.5 * Wnt1 down vs E15.5 * Sox10
10533026	Prkab1	NM_031869	0.000296155	-1.41748	E15.5 * Wnt1 down vs E15.5 * Sox10
10416945	Mirhg1	AK053349	0.00165177	-1.41938	E15.5 * Wnt1 down vs E15.5 * Sox10
10471424	Fam102a	NM_153560	0.00123516	-1.42017	E15.5 * Wnt1 down vs E15.5 * Sox10
10548105	Ccnd2	NM_009829	4.27706e-005	-1.42062	E15.5 * Wnt1 down vs E15.5 * Sox10
10445875	Btg3	NM_009770	0.00150543	-1.42063	E15.5 * Wnt1 down vs E15.5 * Sox10
10504470	Melk	NM_010790	2.02116e-006	-1.42163	E15.5 * Wnt1 down vs E15.5 * Sox10
10430201	Myh9	NM_022410	6.08345e-005	-1.42177	E15.5 * Wnt1 down vs E15.5 * Sox10
10432540	Lima1	NM_001113545	2.23792e-007	-1.42205	E15.5 * Wnt1 down vs E15.5 * Sox10
10582008	2310061C15Rik	NM_026844	2.83167e-005	-1.42312	E15.5 * Wnt1 down vs E15.5 * Sox10

10573924	Mmp2	NM_008610	0.000772259	-1.42332	E15.5 * Wnt1 down vs E15.5 * Sox10
10388234	Gsg2	NM_010353	1.63552e-005	-1.42335	E15.5 * Wnt1 down vs E15.5 * Sox10
10361799	Adat2	NM_025748	0.000154876	-1.42345	E15.5 * Wnt1 down vs E15.5 * Sox10
10346000	Gulp1	NM_028450	0.00127916	-1.42411	E15.5 * Wnt1 down vs E15.5 * Sox10
10389797	Stxbp4	NM_011505	0.000252451	-1.42513	E15.5 * Wnt1 down vs E15.5 * Sox10
10476759	Rin2	NM_028724	0.000189807	-1.42631	E15.5 * Wnt1 down vs E15.5 * Sox10
10394798	EG668539	XR_034692	0.00362946	-1.42748	E15.5 * Wnt1 down vs E15.5 * Sox10
10575733	Cenpn	NM_028131	1.93179e-005	-1.42762	E15.5 * Wnt1 down vs E15.5 * Sox10
10530806	Ppat	BC023841	1.89962e-007	-1.42762	E15.5 * Wnt1 down vs E15.5 * Sox10
10480432	Mastl	NM_025979	0.00106293	-1.42808	E15.5 * Wnt1 down vs E15.5 * Sox10
10366043	Dusp6	NM_026268	0.00469524	-1.42884	E15.5 * Wnt1 down vs E15.5 * Sox10
10548176	5930416I19Rik	NR_027360	4.19582e-005	-1.42922	E15.5 * Wnt1 down vs E15.5 * Sox10
10395058	Adi1	NM_134052	0.00281192	-1.4299	E15.5 * Wnt1 down vs E15.5 * Sox10
10385248	Hmmr	NM_013552	0.00120318	-1.43028	E15.5 * Wnt1 down vs E15.5 * Sox10
10498168	Exosc8	NM_027148	4.44581e-007	-1.43047	E15.5 * Wnt1 down vs E15.5 * Sox10
10518947	Ajap1	NM_001099299	0.000319971	-1.43154	E15.5 * Wnt1 down vs E15.5 * Sox10
10362538	Lama4	NM_010681	3.50854e-007	-1.43154	E15.5 * Wnt1 down vs E15.5 * Sox10
10456357	Pmaip1	NM_021451	0.0018373	-1.43231	E15.5 * Wnt1 down vs E15.5 * Sox10
10473367	Slc43a1	NM_001081349	0.000464752	-1.43256	E15.5 * Wnt1 down vs E15.5 * Sox10
10367982	Gpr126	NM_001002268	0.000279601	-1.43421	E15.5 * Wnt1 down vs E15.5 * Sox10
10358535	Hmcn1	NM_001024720	0.00153308	-1.43429	E15.5 * Wnt1 down vs E15.5 * Sox10
10506031	Nfia	NM_010905	0.0039882	-1.43507	E15.5 * Wnt1 down vs E15.5 * Sox10
10400357	Baz1a	NM_013815	0.00030038	-1.43582	E15.5 * Wnt1 down vs E15.5 * Sox10
10508052	Grik3	NM_001081097	0.0036516	-1.43613	E15.5 * Wnt1 down vs E15.5 * Sox10
10555695	Rrm1	NM_009103	4.8728e-008	-1.43656	E15.5 * Wnt1 down vs E15.5 * Sox10
10381526	Ppih	NM_001110130	6.78158e-005	-1.43666	E15.5 * Wnt1 down vs E15.5 * Sox10
10607475	Prdx4	NM_016764	7.12453e-005	-1.43672	E15.5 * Wnt1 down vs E15.5 * Sox10
10545130	Gadd45a	NM_007836	0.000314453	-1.43812	E15.5 * Wnt1 down vs E15.5 * Sox10
10406581	Dhfr	NM_010049	0.000251243	-1.43833	E15.5 * Wnt1 down vs E15.5 * Sox10
10563883	Depdc1a	NM_029523	0.00308846	-1.43842	E15.5 * Wnt1 down vs E15.5 * Sox10
10513608	Alad	NM_008525	5.99843e-005	-1.4385	E15.5 * Wnt1 down vs E15.5 * Sox10
10573451	Syce2	NM_027954	1.61137e-007	-1.43879	E15.5 * Wnt1 down vs E15.5 * Sox10
10412207	Gpx8	NM_027127	1.10369e-005	-1.43892	E15.5 * Wnt1 down vs E15.5 * Sox10
10400405	Nfkbia	NM_010907	0.000282993	-1.43905	E15.5 * Wnt1 down vs E15.5 * Sox10
10358555	Hmcn1	NM_001024720	0.00169251	-1.44093	E15.5 * Wnt1 down vs E15.5 * Sox10
10503508	Ggh	NM_010281	0.000129951	-1.44129	E15.5 * Wnt1 down vs E15.5 * Sox10
10503523	Ggh	NM_010281	0.000129951	-1.44129	E15.5 * Wnt1 down vs E15.5 * Sox10
10516246	Cdca8	NM_026560	1.45042e-006	-1.44162	E15.5 * Wnt1 down vs E15.5 * Sox10
10394749	Nol10	NM_001008421	4.29056e-005	-1.4418	E15.5 * Wnt1 down vs E15.5 * Sox10
10532504	Hscb	NM_153571	0.000708116	-1.44209	E15.5 * Wnt1 down vs E15.5 * Sox10
10451805	Sgol1	NM_028232	5.02578e-005	-1.44228	E15.5 * Wnt1 down vs E15.5 * Sox10
10416655	EG432879	NM_001034882	0.00342286	-1.4423	E15.5 * Wnt1 down vs E15.5 * Sox10
10583008	Casp12	NM_009808	0.00207399	-1.44291	E15.5 * Wnt1 down vs E15.5 * Sox10
10506680	Tmem48	NM_028355	5.05473e-006	-1.44293	E15.5 * Wnt1 down vs E15.5 * Sox10
10584674	Mcam	NM_023061	3.10721e-005	-1.44305	E15.5 * Wnt1 down vs E15.5 * Sox10
10503952	Ifnk	NM_199157	0.00201239	-1.44356	E15.5 * Wnt1 down vs E15.5 * Sox10
10420426	F630043A04Rik	NM_198605	5.67428e-005	-1.44522	E15.5 * Wnt1 down vs E15.5 * Sox10
10460738	Cdca5	NM_026410	0.00455686	-1.44534	E15.5 * Wnt1 down vs E15.5 * Sox10
10494595	Notch2	NM_010928	0.000127592	-1.44548	E15.5 * Wnt1 down vs E15.5 * Sox10
10495675	F3	NM_010171	0.000153179	-1.44581	E15.5 * Wnt1 down vs E15.5 * Sox10
10458195	Cdc25c	NM_009860	0.000205667	-1.44674	E15.5 * Wnt1 down vs E15.5 * Sox10
10350146	Phlda3	NM_013750	0.000311608	-1.44693	E15.5 * Wnt1 down vs E15.5 * Sox10
10594774	Ccnb2	NM_007630	2.74238e-005	-1.44701	E15.5 * Wnt1 down vs E15.5 * Sox10
10547073	Snora7a	AF357398	0.00230243	-1.44707	E15.5 * Wnt1 down vs E15.5 * Sox10
10461093	Pla2g16	NM_139269	0.00202294	-1.44814	E15.5 * Wnt1 down vs E15.5 * Sox10
10524417	Iscu	NM_025526	0.00144041	-1.44887	E15.5 * Wnt1 down vs E15.5 * Sox10
10500666	Ptgfrn	NM_011197	0.0030953	-1.44893	E15.5 * Wnt1 down vs E15.5 * Sox10
10384322	Hus1	NM_008316	0.00032002	-1.45004	E15.5 * Wnt1 down vs E15.5 * Sox10
10557156	Plk1	NM_011121	0.000137531	-1.45019	E15.5 * Wnt1 down vs E15.5 * Sox10
10355325	Bard1	NM_007525	0.00155405	-1.45022	E15.5 * Wnt1 down vs E15.5 * Sox10
10462866	Cep55	NM_028760	0.000110042	-1.45045	E15.5 * Wnt1 down vs E15.5 * Sox10
10569646	Ccnd1	NM_007631	2.17053e-005	-1.45047	E15.5 * Wnt1 down vs E15.5 * Sox10
10416371	P2ry5	NM_175116	3.13305e-005	-1.4506	E15.5 * Wnt1 down vs E15.5 * Sox10
10466938	5033414D02Rik	NM_026362	0.00444785	-1.45158	E15.5 * Wnt1 down vs E15.5 * Sox10
10453553		---	0.000324152	-1.45159	E15.5 * Wnt1 down vs E15.5 * Sox10
10586781	Myo1e	NM_181072	0.000493796	-1.45196	E15.5 * Wnt1 down vs E15.5 * Sox10

10474825	D2Ertd750e	NM_026412	0.000428857	-1.45206	E15.5 * Wnt1 down vs E15.5 * Sox10
10580183	Ier2	NM_010499	0.00384483	-1.45325	E15.5 * Wnt1 down vs E15.5 * Sox10
10374777	Efemp1	NM_146015	0.00354673	-1.45337	E15.5 * Wnt1 down vs E15.5 * Sox10
10542824	Mrps35	NM_145573	0.000134751	-1.45459	E15.5 * Wnt1 down vs E15.5 * Sox10
10591781	Anln	NM_028390	8.46603e-006	-1.45463	E15.5 * Wnt1 down vs E15.5 * Sox10
10495596	Frrs1	NM_001113478	0.000673307	-1.45466	E15.5 * Wnt1 down vs E15.5 * Sox10
10462613	Ifit2	NM_008332	7.60834e-006	-1.45554	E15.5 * Wnt1 down vs E15.5 * Sox10
10546163	Mcm2	NM_008564	0.000234913	-1.45598	E15.5 * Wnt1 down vs E15.5 * Sox10
10373530	Cdk2	NM_183417	0.00118138	-1.45602	E15.5 * Wnt1 down vs E15.5 * Sox10
10524169	Pole	NM_011132	1.51543e-006	-1.45614	E15.5 * Wnt1 down vs E15.5 * Sox10
10439130	Umps	NM_009471	5.95998e-006	-1.45627	E15.5 * Wnt1 down vs E15.5 * Sox10
10542079	Foxm1	NM_008021	9.33036e-006	-1.45628	E15.5 * Wnt1 down vs E15.5 * Sox10
10506880	Kti12	NM_029571	0.00296824	-1.45668	E15.5 * Wnt1 down vs E15.5 * Sox10
10592201	Chek1	NM_007691	0.00014722	-1.45681	E15.5 * Wnt1 down vs E15.5 * Sox10
10449644	Glo1	NM_025374	1.32714e-005	-1.45709	E15.5 * Wnt1 down vs E15.5 * Sox10
10364518	Ptbp1	NM_001077363	9.746e-005	-1.45796	E15.5 * Wnt1 down vs E15.5 * Sox10
10487139	Shc4	NM_199022	8.53181e-006	-1.45825	E15.5 * Wnt1 down vs E15.5 * Sox10
10585395	Siva1	NM_013929	0.00281323	-1.45911	E15.5 * Wnt1 down vs E15.5 * Sox10
10349239	Mki67ip	NM_026472	9.36134e-006	-1.45929	E15.5 * Wnt1 down vs E15.5 * Sox10
10476301	Smox	NM_145533	0.00106875	-1.46049	E15.5 * Wnt1 down vs E15.5 * Sox10
10522712	Rest	NM_011263	5.99935e-005	-1.46097	E15.5 * Wnt1 down vs E15.5 * Sox10
10512065	OTTMUSG00000	XR_033416	0.00147569	-1.46131	E15.5 * Wnt1 down vs E15.5 * Sox10
10432986	Aaas	NM_153416	3.76825e-005	-1.46144	E15.5 * Wnt1 down vs E15.5 * Sox10
10466521	Gcnt1	NM_173442	0.00036776	-1.46146	E15.5 * Wnt1 down vs E15.5 * Sox10
10508151	Clspn	NM_175554	6.25205e-005	-1.46213	E15.5 * Wnt1 down vs E15.5 * Sox10
10470318		---	0.00195148	-1.4637	E15.5 * Wnt1 down vs E15.5 * Sox10
10436106	C330027C09Rik	NM_172616	0.000110756	-1.46388	E15.5 * Wnt1 down vs E15.5 * Sox10
10534974	Mcm7	NM_008568	0.000223255	-1.4647	E15.5 * Wnt1 down vs E15.5 * Sox10
10599498	Utp14a	NM_028276	1.18309e-007	-1.46471	E15.5 * Wnt1 down vs E15.5 * Sox10
10478160	Fam83d	BC068129	1.44069e-005	-1.46574	E15.5 * Wnt1 down vs E15.5 * Sox10
10501402	Gpsm2	NM_029522	7.57542e-005	-1.46622	E15.5 * Wnt1 down vs E15.5 * Sox10
10571815	Gpm6a	NM_153581	0.00494926	-1.46716	E15.5 * Wnt1 down vs E15.5 * Sox10
10415132	Cmtm5	NM_026066	9.23478e-005	-1.46767	E15.5 * Wnt1 down vs E15.5 * Sox10
10415640	Snora65	NR_002898	1.78313e-005	-1.46799	E15.5 * Wnt1 down vs E15.5 * Sox10
10467420	Pdlim1	NM_016861	0.00058499	-1.46809	E15.5 * Wnt1 down vs E15.5 * Sox10
10352048	Exo1	NM_012012	5.75922e-006	-1.46868	E15.5 * Wnt1 down vs E15.5 * Sox10
10426098	Creld2	NM_029720	9.57113e-008	-1.4688	E15.5 * Wnt1 down vs E15.5 * Sox10
10497122	Depdc1a	NM_029523	0.00308489	-1.46905	E15.5 * Wnt1 down vs E15.5 * Sox10
10587534	Bckdhb	NM_199195	0.000142751	-1.46953	E15.5 * Wnt1 down vs E15.5 * Sox10
10497703	Mrpl47	NM_029017	5.41959e-005	-1.46985	E15.5 * Wnt1 down vs E15.5 * Sox10
10447097	Gemin6	NM_026053	0.000563376	-1.47005	E15.5 * Wnt1 down vs E15.5 * Sox10
10547943	Ncapd2	NM_146171	3.586e-005	-1.47053	E15.5 * Wnt1 down vs E15.5 * Sox10
10466935	Rln1	NM_011272	0.00113695	-1.47159	E15.5 * Wnt1 down vs E15.5 * Sox10
10603316	2010204K13Rik	AF038507	5.57365e-005	-1.47288	E15.5 * Wnt1 down vs E15.5 * Sox10
10409190	Cenpp	NM_025495	0.00131437	-1.47346	E15.5 * Wnt1 down vs E15.5 * Sox10
10521709	Lap3	NM_024434	6.04979e-006	-1.47408	E15.5 * Wnt1 down vs E15.5 * Sox10
10604713	Arhgef6	NM_152801	0.00275323	-1.47452	E15.5 * Wnt1 down vs E15.5 * Sox10
10530692	Kdr	NM_010612	0.00012026	-1.47503	E15.5 * Wnt1 down vs E15.5 * Sox10
10499168	Kirrel	NM_130867	0.00143115	-1.47504	E15.5 * Wnt1 down vs E15.5 * Sox10
10372796	Hmga2	NM_010441	3.07575e-005	-1.47548	E15.5 * Wnt1 down vs E15.5 * Sox10
10490903	Car13	NM_024495	0.000782429	-1.47581	E15.5 * Wnt1 down vs E15.5 * Sox10
10384474	Pno1	NM_025443	7.01595e-005	-1.47652	E15.5 * Wnt1 down vs E15.5 * Sox10
10452633	Tgif1	NM_009372	4.19629e-005	-1.47766	E15.5 * Wnt1 down vs E15.5 * Sox10
10476889	Nxt1	NM_001110159	0.000122338	-1.47963	E15.5 * Wnt1 down vs E15.5 * Sox10
10576883	Shcbp1	NM_011369	8.79895e-007	-1.47991	E15.5 * Wnt1 down vs E15.5 * Sox10
10345777	Il1rl2	NM_133193	0.00479232	-1.47996	E15.5 * Wnt1 down vs E15.5 * Sox10
10485963	Arhgap11a	NM_181416	9.31484e-006	-1.48042	E15.5 * Wnt1 down vs E15.5 * Sox10
10438378	Cdc45l	NM_009862	1.09595e-005	-1.4825	E15.5 * Wnt1 down vs E15.5 * Sox10
10358650	Hmcn1	NM_001024720	0.000164081	-1.48428	E15.5 * Wnt1 down vs E15.5 * Sox10
10502778	Lphn2	NM_001081298	0.001246	-1.48447	E15.5 * Wnt1 down vs E15.5 * Sox10
10487340	Ncaph	NM_144818	2.0196e-007	-1.48525	E15.5 * Wnt1 down vs E15.5 * Sox10
10515836	Ccnb1	NM_172301	1.52559e-005	-1.48614	E15.5 * Wnt1 down vs E15.5 * Sox10
10354741	Rftn2	NM_028713	1.92606e-006	-1.48692	E15.5 * Wnt1 down vs E15.5 * Sox10
10514510	Cyp2j6	NM_010008	0.000167252	-1.48721	E15.5 * Wnt1 down vs E15.5 * Sox10
10546538	Suclg2	NM_011507	7.55725e-008	-1.48862	E15.5 * Wnt1 down vs E15.5 * Sox10
10407390	Ptbp1	NM_001077363	0.000722083	-1.48876	E15.5 * Wnt1 down vs E15.5 * Sox10

10423293	Myo10	NM_019472	6.76472e-005	-1.49	E15.5 * Wnt1 down vs E15.5 * Sox10
10358557	Hmcn1	NM_001024720	0.00103286	-1.4911	E15.5 * Wnt1 down vs E15.5 * Sox10
10471675	Glo1	NM_025374	0.000167419	-1.49111	E15.5 * Wnt1 down vs E15.5 * Sox10
10537062	Mest	NM_008590	4.88966e-008	-1.49171	E15.5 * Wnt1 down vs E15.5 * Sox10
10565852	Rnf169	AK173319	4.58647e-006	-1.49206	E15.5 * Wnt1 down vs E15.5 * Sox10
10438626	Etv5	NM_023794	0.000669892	-1.49219	E15.5 * Wnt1 down vs E15.5 * Sox10
10572989	Slc10a7	NM_029736	1.57691e-006	-1.49293	E15.5 * Wnt1 down vs E15.5 * Sox10
10364683	Stk11	NM_011492	0.000790768	-1.4935	E15.5 * Wnt1 down vs E15.5 * Sox10
10360920	Tgfb2	NM_009367	4.99914e-011	-1.49429	E15.5 * Wnt1 down vs E15.5 * Sox10
10430458	Sox10	NM_011437	0.00192583	-1.49445	E15.5 * Wnt1 down vs E15.5 * Sox10
10371959	Elk3	NM_013508	2.05716e-007	-1.49478	E15.5 * Wnt1 down vs E15.5 * Sox10
10456400	Tubb6	NM_026473	1.89471e-007	-1.49561	E15.5 * Wnt1 down vs E15.5 * Sox10
10410560	Trip13	NM_027182	1.12269e-005	-1.49606	E15.5 * Wnt1 down vs E15.5 * Sox10
10408928	Hspb1	NM_013560	0.00406109	-1.49641	E15.5 * Wnt1 down vs E15.5 * Sox10
10426016	Gtse1	NM_013882	2.18051e-006	-1.49642	E15.5 * Wnt1 down vs E15.5 * Sox10
10462035	Ldhb	NM_008492	9.42136e-007	-1.49694	E15.5 * Wnt1 down vs E15.5 * Sox10
10595404	Fam46a	ENSMUST00000	0.000954965	-1.49765	E15.5 * Wnt1 down vs E15.5 * Sox10
10408902	Ccdc90a	NM_001081059	0.000236417	-1.49831	E15.5 * Wnt1 down vs E15.5 * Sox10
10492335	Rap2b	NM_028712	7.45884e-006	-1.49837	E15.5 * Wnt1 down vs E15.5 * Sox10
10517513	C1qc	NM_007574	0.00130743	-1.49896	E15.5 * Wnt1 down vs E15.5 * Sox10
10375234	Nudcd2	NM_026023	9.37018e-009	-1.49939	E15.5 * Wnt1 down vs E15.5 * Sox10
10450374	D17H6S56E-5	L78788	1.3091e-005	-1.49942	E15.5 * Wnt1 down vs E15.5 * Sox10
10506150	Foxd3	NM_010425	0.0037256	-1.49963	E15.5 * Wnt1 down vs E15.5 * Sox10
10597413	Crtap	NM_019922	8.14629e-005	-1.50105	E15.5 * Wnt1 down vs E15.5 * Sox10
10607497	Suclg2	NM_011507	1.19271e-006	-1.50118	E15.5 * Wnt1 down vs E15.5 * Sox10
10591556	Spc24	NM_026282	2.03838e-005	-1.50153	E15.5 * Wnt1 down vs E15.5 * Sox10
10577633	Golga7	NM_020585	0.000892674	-1.50488	E15.5 * Wnt1 down vs E15.5 * Sox10
10361375	Fbxo5	NM_025995	5.97045e-006	-1.50606	E15.5 * Wnt1 down vs E15.5 * Sox10
10586604	Rps27l	NM_026467	6.38702e-006	-1.50653	E15.5 * Wnt1 down vs E15.5 * Sox10
10562223	Fxyd3	NM_008557	2.16789e-005	-1.50658	E15.5 * Wnt1 down vs E15.5 * Sox10
10505132	Akap2	NM_001035533	3.03051e-007	-1.50733	E15.5 * Wnt1 down vs E15.5 * Sox10
10506118	Usp1	NM_146144	9.08323e-005	-1.50752	E15.5 * Wnt1 down vs E15.5 * Sox10
10358978	Ier5	NM_010500	6.15715e-007	-1.50798	E15.5 * Wnt1 down vs E15.5 * Sox10
10446928	Ltbp1	NM_019919	1.27313e-006	-1.50832	E15.5 * Wnt1 down vs E15.5 * Sox10
10537157	2310005E10Rik	NM_172398	5.93498e-006	-1.50842	E15.5 * Wnt1 down vs E15.5 * Sox10
10512022	Mobkl2b	ENSMUST00000	0.00128451	-1.5088	E15.5 * Wnt1 down vs E15.5 * Sox10
10408838	Elovl2	NM_019423	0.000399589	-1.50996	E15.5 * Wnt1 down vs E15.5 * Sox10
10385153	Ccdc99	NM_027411	0.000340635	-1.51099	E15.5 * Wnt1 down vs E15.5 * Sox10
10516064	Mfsd2	NM_029662	0.000905252	-1.51132	E15.5 * Wnt1 down vs E15.5 * Sox10
10438690	Rfc4	NM_145480	0.000347123	-1.51267	E15.5 * Wnt1 down vs E15.5 * Sox10
10397002	Sipa1l1	NM_172579	6.76653e-006	-1.51291	E15.5 * Wnt1 down vs E15.5 * Sox10
10506433	Dab1	NM_177259	4.4247e-005	-1.5132	E15.5 * Wnt1 down vs E15.5 * Sox10
10416037	Pbk	NM_023209	0.000238613	-1.51335	E15.5 * Wnt1 down vs E15.5 * Sox10
10400649	Pole2	NM_011133	1.60979e-005	-1.51446	E15.5 * Wnt1 down vs E15.5 * Sox10
10363146	Slc35f1	NM_178675	0.00107337	-1.52053	E15.5 * Wnt1 down vs E15.5 * Sox10
10368556	Hey2	NM_013904	0.000339517	-1.52076	E15.5 * Wnt1 down vs E15.5 * Sox10
10419323	Dlgap5	NM_144553	1.14571e-006	-1.52143	E15.5 * Wnt1 down vs E15.5 * Sox10
10486029	Atpbd4	NM_025675	0.000181318	-1.52302	E15.5 * Wnt1 down vs E15.5 * Sox10
10475866	Bcl2l11	NM_207680	6.05106e-006	-1.52428	E15.5 * Wnt1 down vs E15.5 * Sox10
10482517	Nmi	NM_001141949	0.00258757	-1.52569	E15.5 * Wnt1 down vs E15.5 * Sox10
10475643	Fgf7	NM_008008	0.00499673	-1.526	E15.5 * Wnt1 down vs E15.5 * Sox10
10384032	Pold2	NM_008894	2.63056e-005	-1.52625	E15.5 * Wnt1 down vs E15.5 * Sox10
10498018	Pcdh18	NM_130448	4.00217e-006	-1.52735	E15.5 * Wnt1 down vs E15.5 * Sox10
10436369	Filip1l	NM_030163	4.51711e-008	-1.53015	E15.5 * Wnt1 down vs E15.5 * Sox10
10384223	Igfbp3	NM_008343	0.000128313	-1.53025	E15.5 * Wnt1 down vs E15.5 * Sox10
10554808	Fzd4	NM_008055	0.00073718	-1.53185	E15.5 * Wnt1 down vs E15.5 * Sox10
10577508	Ckap2	NM_001004140	3.96203e-005	-1.53315	E15.5 * Wnt1 down vs E15.5 * Sox10
10577190	Rasa3	NM_009025	2.99087e-005	-1.5333	E15.5 * Wnt1 down vs E15.5 * Sox10
10383632	Sfi1	NM_030207	0.000325127	-1.53363	E15.5 * Wnt1 down vs E15.5 * Sox10
10493114	Nes	NM_016701	0.00390705	-1.53535	E15.5 * Wnt1 down vs E15.5 * Sox10
10361110	Dtl	NM_029766	2.28129e-006	-1.53897	E15.5 * Wnt1 down vs E15.5 * Sox10
10545458	Tcf3	NM_001079822	5.28403e-005	-1.54021	E15.5 * Wnt1 down vs E15.5 * Sox10
10353420	Mcm3	NM_008563	3.83952e-008	-1.54064	E15.5 * Wnt1 down vs E15.5 * Sox10
10577315	Angpt2	NM_007426	4.1599e-006	-1.54207	E15.5 * Wnt1 down vs E15.5 * Sox10
10346564	Casp8	NM_009812	0.000406035	-1.5423	E15.5 * Wnt1 down vs E15.5 * Sox10
10545974	Antxr1	NM_054041	0.00198892	-1.54231	E15.5 * Wnt1 down vs E15.5 * Sox10

10419790	Jub	NM_010590	0.000287262	-1.54285	E15.5 * Wnt1 down vs E15.5 * Sox10
10455967	2610318N02Rik	BC039993	0.000341171	-1.54324	E15.5 * Wnt1 down vs E15.5 * Sox10
10601011	Kif4	NM_008446	2.68524e-007	-1.54371	E15.5 * Wnt1 down vs E15.5 * Sox10
10483809	Nfe2l2	NM_010902	0.000107338	-1.54894	E15.5 * Wnt1 down vs E15.5 * Sox10
10366796	Mettl1	NM_010792	0.000163215	-1.55082	E15.5 * Wnt1 down vs E15.5 * Sox10
10362394	Hddc2	NM_027168	0.000140519	-1.55114	E15.5 * Wnt1 down vs E15.5 * Sox10
10562637	Ccnb1	NM_172301	3.84077e-006	-1.55123	E15.5 * Wnt1 down vs E15.5 * Sox10
10437945	Mcm4	NM_008565	6.592e-007	-1.55124	E15.5 * Wnt1 down vs E15.5 * Sox10
10398874	Siva1	NM_013929	1.52745e-006	-1.55743	E15.5 * Wnt1 down vs E15.5 * Sox10
10411739	Ccnb1	NM_172301	1.66146e-006	-1.55837	E15.5 * Wnt1 down vs E15.5 * Sox10
10400304	Egln3	NM_028133	0.00318473	-1.55838	E15.5 * Wnt1 down vs E15.5 * Sox10
10502510	Lmo4	NM_010723	2.8869e-005	-1.55975	E15.5 * Wnt1 down vs E15.5 * Sox10
10469358	Mrc1	NM_008625	0.00128321	-1.56038	E15.5 * Wnt1 down vs E15.5 * Sox10
10440186	Crybg3	NM_174848	0.000228455	-1.56128	E15.5 * Wnt1 down vs E15.5 * Sox10
10401238	Zfp36l1	NM_007564	0.000340347	-1.56425	E15.5 * Wnt1 down vs E15.5 * Sox10
10394770	Odc1	NM_013614	7.24726e-008	-1.57036	E15.5 * Wnt1 down vs E15.5 * Sox10
10422598	Sepp1	NM_009155	0.00035891	-1.57108	E15.5 * Wnt1 down vs E15.5 * Sox10
10388430	Serpinf1	NM_011340	2.24023e-007	-1.57445	E15.5 * Wnt1 down vs E15.5 * Sox10
10394809	EG668645	XR_033945	0.000204602	-1.57463	E15.5 * Wnt1 down vs E15.5 * Sox10
10394816	EG668645	XR_033945	0.000204602	-1.57463	E15.5 * Wnt1 down vs E15.5 * Sox10
10596637	Mapkapk3	NM_178907	4.8662e-005	-1.57525	E15.5 * Wnt1 down vs E15.5 * Sox10
10474860	Chst14	NM_028117	7.98764e-007	-1.5843	E15.5 * Wnt1 down vs E15.5 * Sox10
10571530	Fat1	NM_001081286	1.92291e-007	-1.58467	E15.5 * Wnt1 down vs E15.5 * Sox10
10558345	Dock1	NM_001033420	5.51205e-008	-1.58595	E15.5 * Wnt1 down vs E15.5 * Sox10
10489051	5730471H19Rik	AK133873	7.05446e-005	-1.58635	E15.5 * Wnt1 down vs E15.5 * Sox10
10399087	Ncapg2	NM_133762	0.000107629	-1.59242	E15.5 * Wnt1 down vs E15.5 * Sox10
10572906	Mcm5	NM_008566	1.19019e-005	-1.59443	E15.5 * Wnt1 down vs E15.5 * Sox10
10473384	Slc43a3	NM_021398	6.47774e-006	-1.59507	E15.5 * Wnt1 down vs E15.5 * Sox10
10434845	Il1rap	NM_008364	1.27588e-006	-1.59734	E15.5 * Wnt1 down vs E15.5 * Sox10
10541091	Gemin6	NM_026053	9.3895e-007	-1.59769	E15.5 * Wnt1 down vs E15.5 * Sox10
10458906	Ppic	NM_008908	2.0445e-009	-1.59822	E15.5 * Wnt1 down vs E15.5 * Sox10
10364593	Cnn2	NM_007725	0.00105366	-1.60448	E15.5 * Wnt1 down vs E15.5 * Sox10
10513320	Ptgr1	NM_025968	4.15006e-008	-1.60594	E15.5 * Wnt1 down vs E15.5 * Sox10
10350733	Rgs16	NM_011267	0.00267535	-1.60693	E15.5 * Wnt1 down vs E15.5 * Sox10
10497265	Fabp4	NM_024406	0.00302511	-1.60809	E15.5 * Wnt1 down vs E15.5 * Sox10
10431915	Slc38a4	NM_027052	9.09492e-006	-1.61256	E15.5 * Wnt1 down vs E15.5 * Sox10
10567355	Gprc5b	NM_022420	1.50442e-006	-1.6183	E15.5 * Wnt1 down vs E15.5 * Sox10
10492341	4631416L12Rik	NM_001081295	8.83091e-005	-1.62002	E15.5 * Wnt1 down vs E15.5 * Sox10
10462683	Pcgf5	NM_029508	0.000224025	-1.62128	E15.5 * Wnt1 down vs E15.5 * Sox10
10485624	Prrg4	NM_178695	4.30209e-005	-1.62236	E15.5 * Wnt1 down vs E15.5 * Sox10
10586591	Car12	NM_178396	3.04027e-005	-1.63563	E15.5 * Wnt1 down vs E15.5 * Sox10
10351259	Slc19a2	NM_054087	0.000897294	-1.64008	E15.5 * Wnt1 down vs E15.5 * Sox10
10434806	Lpp	NM_178665	8.08399e-005	-1.6401	E15.5 * Wnt1 down vs E15.5 * Sox10
10352661	Ptpn14	NM_008976	3.59493e-006	-1.64691	E15.5 * Wnt1 down vs E15.5 * Sox10
10353010	Mybl1	NM_008651	0.000147596	-1.65498	E15.5 * Wnt1 down vs E15.5 * Sox10
10428672	Dscc1	NM_183089	2.59572e-006	-1.65788	E15.5 * Wnt1 down vs E15.5 * Sox10
10362201	Ctgf	NM_010217	1.52857e-005	-1.65867	E15.5 * Wnt1 down vs E15.5 * Sox10
10467139	Lipa	NM_021460	3.48e-007	-1.66029	E15.5 * Wnt1 down vs E15.5 * Sox10
10347792		---	5.58568e-005	-1.67247	E15.5 * Wnt1 down vs E15.5 * Sox10
10466104	Ccdc86	NM_023731	2.59198e-005	-1.67295	E15.5 * Wnt1 down vs E15.5 * Sox10
10373702	Pisd-ps1	NR_003517	0.000168435	-1.67538	E15.5 * Wnt1 down vs E15.5 * Sox10
10606071	Ercc6l	NM_146235	3.72955e-006	-1.67989	E15.5 * Wnt1 down vs E15.5 * Sox10
10357345	E030049G20Rik	NM_172484	9.36224e-005	-1.68009	E15.5 * Wnt1 down vs E15.5 * Sox10
10514520	Cyp2j9	NM_028979	3.92713e-005	-1.68348	E15.5 * Wnt1 down vs E15.5 * Sox10
10389795	Stxbp4	NM_011505	0.00195765	-1.69199	E15.5 * Wnt1 down vs E15.5 * Sox10
10483249	Galnt3	NM_015736	0.000365315	-1.70789	E15.5 * Wnt1 down vs E15.5 * Sox10
10385375	Thg1l	NM_001080969	2.43514e-005	-1.71305	E15.5 * Wnt1 down vs E15.5 * Sox10
10559649	Cox6b2	NM_183405	0.00149601	-1.73266	E15.5 * Wnt1 down vs E15.5 * Sox10
10555233		---	2.30345e-005	-1.73485	E15.5 * Wnt1 down vs E15.5 * Sox10
10577641	1810011O10Rik	NM_026931	0.00217709	-1.76177	E15.5 * Wnt1 down vs E15.5 * Sox10
10606333	Fndc3c1	NM_001007580	3.7731e-005	-1.76691	E15.5 * Wnt1 down vs E15.5 * Sox10
10360684	Ephx1	NM_010145	0.00305913	-1.77455	E15.5 * Wnt1 down vs E15.5 * Sox10
10530100	Arap2	NM_178407	8.87522e-006	-1.79838	E15.5 * Wnt1 down vs E15.5 * Sox10
10450957	Cenpq	NM_031863	6.14464e-006	-1.79874	E15.5 * Wnt1 down vs E15.5 * Sox10
10547657	C3ar1	NM_009779	0.000356828	-1.81197	E15.5 * Wnt1 down vs E15.5 * Sox10
10364149	S100b	NM_009115	5.29981e-005	-1.83433	E15.5 * Wnt1 down vs E15.5 * Sox10

10495316	Psrc1	NM_019976	1.70742e-006	-1.85122	E15.5 * Wnt1 down vs E15.5 * Sox10
10424404	Pvt1	NR_003368	4.60863e-005	-1.85856	E15.5 * Wnt1 down vs E15.5 * Sox10
10496373	Ddit4l	NM_030143	0.000639171	-1.85947	E15.5 * Wnt1 down vs E15.5 * Sox10
10511631	Slc26a7	NM_145947	2.96818e-006	-1.87629	E15.5 * Wnt1 down vs E15.5 * Sox10
10520950	Pdlim1	NM_016861	2.68189e-006	-1.88036	E15.5 * Wnt1 down vs E15.5 * Sox10
10502655	Cyr61	NM_010516	0.0013656	-1.88245	E15.5 * Wnt1 down vs E15.5 * Sox10
10474467	Muc15	NM_172979	0.00184081	-1.89595	E15.5 * Wnt1 down vs E15.5 * Sox10
10425265	ENSMUSG0000C	ENSMUST00000	5.05626e-005	-1.90684	E15.5 * Wnt1 down vs E15.5 * Sox10
10503259	Trp53inp1	NM_021897	2.94406e-008	-1.91818	E15.5 * Wnt1 down vs E15.5 * Sox10
10474524	Olfr1318	NM_001011802	7.35042e-006	-1.92967	E15.5 * Wnt1 down vs E15.5 * Sox10
10577623	Gins4	NM_024240	2.39305e-007	-1.94913	E15.5 * Wnt1 down vs E15.5 * Sox10
10472893	B230120H23Rik	NM_023057	4.22584e-007	-1.95102	E15.5 * Wnt1 down vs E15.5 * Sox10
10485745	Ano3	NM_001128103	0.000592284	-1.97009	E15.5 * Wnt1 down vs E15.5 * Sox10
10416230	Tnfrsf10b	NM_020275	4.54587e-005	-1.97523	E15.5 * Wnt1 down vs E15.5 * Sox10
10450367	Hspa1a	NM_010479	0.000965668	-1.97539	E15.5 * Wnt1 down vs E15.5 * Sox10
10371506	Stab2	NM_138673	0.00234194	-1.99097	E15.5 * Wnt1 down vs E15.5 * Sox10
10356084	Irs1	NM_010570	2.86668e-006	-2.00538	E15.5 * Wnt1 down vs E15.5 * Sox10
10430447	1700088E04Rik	NM_138581	3.0106e-006	-2.05816	E15.5 * Wnt1 down vs E15.5 * Sox10
10436978	Cbr3	NM_173047	9.53445e-005	-2.16451	E15.5 * Wnt1 down vs E15.5 * Sox10
10565570	4632434I11Rik	NM_001080995	2.48583e-007	-2.17055	E15.5 * Wnt1 down vs E15.5 * Sox10
10604743	Snord61	NR_002903	0.0038026	-2.23165	E15.5 * Wnt1 down vs E15.5 * Sox10
10384145	H2afv	BC028539	3.56725e-008	-2.24307	E15.5 * Wnt1 down vs E15.5 * Sox10
10425257	Polr2f	NM_027231	4.90177e-006	-2.2551	E15.5 * Wnt1 down vs E15.5 * Sox10
10582580		---	3.64809e-005	-2.35588	E15.5 * Wnt1 down vs E15.5 * Sox10
10425240	Micall1	NM_177461	3.94689e-009	-2.53071	E15.5 * Wnt1 down vs E15.5 * Sox10
10385271	Ccng1	NM_009831	3.9261e-011	-2.98432	E15.5 * Wnt1 down vs E15.5 * Sox10
10605874	Eda2r	NM_175540	1.83176e-006	-3.26152	E15.5 * Wnt1 down vs E15.5 * Sox10
10443463	Cdkn1a	NM_007669	2.36993e-009	-3.68721	E15.5 * Wnt1 down vs E15.5 * Sox10
10485718	Ano3	NM_001128103	4.56038e-008	-3.9725	E15.5 * Wnt1 down vs E15.5 * Sox10

W15 vs C15

Probeset ID	Gene Symbol	RefSeq	p-value	Fold-change	Comparison
10593756	Chrna3	NM_145129	3.68322e-018	57.2612	E15.5 * Wnt1 up vs E15.5 * Ctrl
10515095	Elavl4	NM_010488	9.36054e-017	42.0578	E15.5 * Wnt1 up vs E15.5 * Ctrl
10463737	Ina	AK144917	1.20069e-013	35.8826	E15.5 * Wnt1 up vs E15.5 * Ctrl
10492640	Fstl5	NM_178673	2.55841e-015	34.0967	E15.5 * Wnt1 up vs E15.5 * Ctrl
10372324	Syt1	NM_009306	2.78761e-014	29.2443	E15.5 * Wnt1 up vs E15.5 * Ctrl
10576332	Tubb3	NM_023279	1.14223e-012	28.1712	E15.5 * Wnt1 up vs E15.5 * Ctrl
10536667	Ptprz1	NM_001081306	3.48106e-016	27.124	E15.5 * Wnt1 up vs E15.5 * Ctrl
10463732	Ina	NM_146100	1.11843e-017	26.0165	E15.5 * Wnt1 up vs E15.5 * Ctrl
10595033	Scg3	NM_009130	1.08222e-016	25.9031	E15.5 * Wnt1 up vs E15.5 * Ctrl
10540298	Chl1	NM_007697	3.57063e-016	25.3792	E15.5 * Wnt1 up vs E15.5 * Ctrl
10531869	Mapk10	NM_009158	1.65179e-015	24.4878	E15.5 * Wnt1 up vs E15.5 * Ctrl
10473244	Zfp804a	NM_175513	2.14087e-015	22.2964	E15.5 * Wnt1 up vs E15.5 * Ctrl
10585484	Chrna5	NM_176844	2.54901e-014	21.675	E15.5 * Wnt1 up vs E15.5 * Ctrl
10357103	Cdh19	NM_001081386	4.40966e-012	20.5054	E15.5 * Wnt1 up vs E15.5 * Ctrl
10492169	Mab21l1	NM_010750	2.41877e-015	19.9722	E15.5 * Wnt1 up vs E15.5 * Ctrl
10536363	Tac1	NM_009311	1.70472e-010	18.9662	E15.5 * Wnt1 up vs E15.5 * Ctrl
10400926	Rtn1	NM_153457	2.07753e-014	18.7184	E15.5 * Wnt1 up vs E15.5 * Ctrl
10458685	Jakmip2	ENSMUST00000(	1.51661e-012	18.6365	E15.5 * Wnt1 up vs E15.5 * Ctrl
10417628	Cadps	NM_012061	6.11704e-014	18.4928	E15.5 * Wnt1 up vs E15.5 * Ctrl
10593767	Chrnb4	NM_148944	3.13736e-013	17.961	E15.5 * Wnt1 up vs E15.5 * Ctrl
10402020	Eml5	NM_001081191	1.00639e-016	17.9415	E15.5 * Wnt1 up vs E15.5 * Ctrl
10436636	Ncam2	NM_001113208	1.27834e-012	17.6167	E15.5 * Wnt1 up vs E15.5 * Ctrl
10498885	Gria2	NM_013540	1.74683e-013	17.4958	E15.5 * Wnt1 up vs E15.5 * Ctrl
10492628	Serpini1	NM_009250	7.45155e-014	17.4605	E15.5 * Wnt1 up vs E15.5 * Ctrl
10522208	Uchl1	NM_011670	8.03718e-013	17.2938	E15.5 * Wnt1 up vs E15.5 * Ctrl
10564163	Snord116	NR_002895	6.9669e-010	17.1658	E15.5 * Wnt1 up vs E15.5 * Ctrl
10564167	Snord116	NR_002895	6.9669e-010	17.1658	E15.5 * Wnt1 up vs E15.5 * Ctrl
10564171	Snord116	NR_002895	6.9669e-010	17.1658	E15.5 * Wnt1 up vs E15.5 * Ctrl
10564173	Snord116	NR_002895	6.9669e-010	17.1658	E15.5 * Wnt1 up vs E15.5 * Ctrl
10564175	Snord116	NR_002895	6.9669e-010	17.1658	E15.5 * Wnt1 up vs E15.5 * Ctrl
10564179	Snord116	NR_002895	6.9669e-010	17.1658	E15.5 * Wnt1 up vs E15.5 * Ctrl
10564181	Snord116	NR_002895	6.9669e-010	17.1658	E15.5 * Wnt1 up vs E15.5 * Ctrl

10564185	Snord116	NR_002895	6.9669e-010	17.1658	E15.5 * Wnt1 up vs E15.5 * Ctrl
10564187	Snord116	NR_002895	6.9669e-010	17.1658	E15.5 * Wnt1 up vs E15.5 * Ctrl
10564189	Snord116	NR_002895	6.9669e-010	17.1658	E15.5 * Wnt1 up vs E15.5 * Ctrl
10564191	Snord116	NR_002895	6.9669e-010	17.1658	E15.5 * Wnt1 up vs E15.5 * Ctrl
10564193	Snord116	NR_002895	6.9669e-010	17.1658	E15.5 * Wnt1 up vs E15.5 * Ctrl
10564195	Snord116	NR_002895	6.9669e-010	17.1658	E15.5 * Wnt1 up vs E15.5 * Ctrl
10564197	Snord116	NR_002895	6.9669e-010	17.1658	E15.5 * Wnt1 up vs E15.5 * Ctrl
10564199	Snord116	NR_002895	6.9669e-010	17.1658	E15.5 * Wnt1 up vs E15.5 * Ctrl
10564201	Snord116	AF241256	6.9669e-010	17.1658	E15.5 * Wnt1 up vs E15.5 * Ctrl
10564205	Snord116	AF241256	6.9669e-010	17.1658	E15.5 * Wnt1 up vs E15.5 * Ctrl
10564207	Snord116	AF241256	6.9669e-010	17.1658	E15.5 * Wnt1 up vs E15.5 * Ctrl
10483803	6720416L17Rik	ENSMUST00000	1.872e-014	16.9027	E15.5 * Wnt1 up vs E15.5 * Ctrl
10490818	Stmn2	NM_025285	1.42654e-014	16.8214	E15.5 * Wnt1 up vs E15.5 * Ctrl
10476512	Snap25	NM_011428	2.82087e-015	16.7296	E15.5 * Wnt1 up vs E15.5 * Ctrl
10564161	Snord116	NR_002895	5.61082e-010	16.7078	E15.5 * Wnt1 up vs E15.5 * Ctrl
10554900	Dlg2	NM_011807	1.07277e-015	16.443	E15.5 * Wnt1 up vs E15.5 * Ctrl
10605113	L1cam	NM_008478	3.47632e-015	16.1787	E15.5 * Wnt1 up vs E15.5 * Ctrl
10522388	Slc10a4	NM_173403	7.29087e-014	15.8163	E15.5 * Wnt1 up vs E15.5 * Ctrl
10346150	Tmeff2	NM_019790	7.67259e-017	15.785	E15.5 * Wnt1 up vs E15.5 * Ctrl
10418835	Slc18a3	NM_021712	9.81468e-012	15.7644	E15.5 * Wnt1 up vs E15.5 * Ctrl
10572024	Spock3	NM_023689	1.12014e-013	15.5978	E15.5 * Wnt1 up vs E15.5 * Ctrl
10564159	Snord116	AF241256	6.14517e-010	15.3133	E15.5 * Wnt1 up vs E15.5 * Ctrl
10465820	Gng3	NM_010316	3.59033e-013	15.2535	E15.5 * Wnt1 up vs E15.5 * Ctrl
10564177	Snord116	AF241256	9.43875e-010	15.0566	E15.5 * Wnt1 up vs E15.5 * Ctrl
10564183	Snord116	AF241256	5.89483e-010	14.9687	E15.5 * Wnt1 up vs E15.5 * Ctrl
10499431	Syt11	NM_018804	5.18846e-015	14.7763	E15.5 * Wnt1 up vs E15.5 * Ctrl
10547227	Ret	NM_001080780	9.25503e-015	14.574	E15.5 * Wnt1 up vs E15.5 * Ctrl
10366163	Slc6a15	NM_175328	1.09737e-012	14.3611	E15.5 * Wnt1 up vs E15.5 * Ctrl
10564165	Snord116	AF241256	1.17594e-009	13.8116	E15.5 * Wnt1 up vs E15.5 * Ctrl
10584549	Scn3b	NM_178227	6.16077e-014	13.5471	E15.5 * Wnt1 up vs E15.5 * Ctrl
10439695	Tagln3	NM_019754	1.02613e-014	13.2152	E15.5 * Wnt1 up vs E15.5 * Ctrl
10439514	Gap43	NM_008083	6.95935e-016	12.8654	E15.5 * Wnt1 up vs E15.5 * Ctrl
10506360	Sgip1	NM_144906	3.96134e-017	12.8303	E15.5 * Wnt1 up vs E15.5 * Ctrl
10367582	Vip	NM_011702	1.79122e-012	12.5651	E15.5 * Wnt1 up vs E15.5 * Ctrl
10483228	Scn3a	NM_018732	1.63011e-013	12.4168	E15.5 * Wnt1 up vs E15.5 * Ctrl
10575693	Vat1l	NM_173016	9.76018e-014	12.3239	E15.5 * Wnt1 up vs E15.5 * Ctrl
10564169	Snord116	AF241256	1.53666e-009	12.0078	E15.5 * Wnt1 up vs E15.5 * Ctrl
10408359	Nrsn1	NM_009513	9.05878e-013	11.9868	E15.5 * Wnt1 up vs E15.5 * Ctrl
10464471	Gal	NM_010253	1.25239e-010	11.805	E15.5 * Wnt1 up vs E15.5 * Ctrl
10344973	Gdap1	NM_010267	1.85703e-011	11.7746	E15.5 * Wnt1 up vs E15.5 * Ctrl
10431659	Kif21a	NM_001109040	1.40153e-015	11.6784	E15.5 * Wnt1 up vs E15.5 * Ctrl
10428509	Csmd3	NM_001081391	4.60129e-007	11.5046	E15.5 * Wnt1 up vs E15.5 * Ctrl
10472374	Scn9a	BC172147	1.94279e-010	11.4478	E15.5 * Wnt1 up vs E15.5 * Ctrl
10471154	Ass1	NM_007494	4.66792e-012	11.4436	E15.5 * Wnt1 up vs E15.5 * Ctrl
10363541	Ass1	NM_007494	2.56318e-012	11.411	E15.5 * Wnt1 up vs E15.5 * Ctrl
10362372	9330159F19Rik	BC138282	2.08601e-012	11.1956	E15.5 * Wnt1 up vs E15.5 * Ctrl
10607156	Dcx	NM_001110222	4.0625e-013	11.1571	E15.5 * Wnt1 up vs E15.5 * Ctrl
10474096	Lrrc4c	NM_178725	7.12842e-015	11.0223	E15.5 * Wnt1 up vs E15.5 * Ctrl
10471994	Kif5c	NM_008449	8.47086e-014	10.9313	E15.5 * Wnt1 up vs E15.5 * Ctrl
10416090	Stmn4	NM_019675	4.71557e-012	10.9135	E15.5 * Wnt1 up vs E15.5 * Ctrl
10458663	Dpysl3	NM_009468	4.03794e-015	10.8271	E15.5 * Wnt1 up vs E15.5 * Ctrl
10442069	Lix1	NM_025681	3.89666e-011	10.7699	E15.5 * Wnt1 up vs E15.5 * Ctrl
10411527	Cartpt	NM_013732	1.71707e-009	10.6847	E15.5 * Wnt1 up vs E15.5 * Ctrl
10567289	Syt17	NM_138649	3.93747e-014	10.6517	E15.5 * Wnt1 up vs E15.5 * Ctrl
10514352	Elavl2	NM_207685	1.6817e-013	10.6487	E15.5 * Wnt1 up vs E15.5 * Ctrl
10492136	Dclk1	NM_019978	1.7649e-015	10.4873	E15.5 * Wnt1 up vs E15.5 * Ctrl
10485979	Gjd2	NM_010290	1.77481e-009	9.92177	E15.5 * Wnt1 up vs E15.5 * Ctrl
10455942	A730017C20Rik	NM_173759	1.6478e-012	9.70107	E15.5 * Wnt1 up vs E15.5 * Ctrl
10476628	Otor	NM_020595	1.38378e-012	9.67486	E15.5 * Wnt1 up vs E15.5 * Ctrl
10402061	Eml5	BC049855	1.61347e-011	9.61354	E15.5 * Wnt1 up vs E15.5 * Ctrl
10521498	Crmp1	NM_007765	1.47908e-013	9.61023	E15.5 * Wnt1 up vs E15.5 * Ctrl
10472400	Scn2a1	NM_001099298	5.16521e-007	9.45366	E15.5 * Wnt1 up vs E15.5 * Ctrl
10485955	Scg5	NM_009162	1.47602e-014	9.43953	E15.5 * Wnt1 up vs E15.5 * Ctrl
10355836	Resp18	NM_009049	1.57932e-012	9.32966	E15.5 * Wnt1 up vs E15.5 * Ctrl
10453518	Nrxn1	NM_020252	7.75798e-012	9.27864	E15.5 * Wnt1 up vs E15.5 * Ctrl
10347036	Mtap2	NM_001039934	1.1573e-016	9.09719	E15.5 * Wnt1 up vs E15.5 * Ctrl

10534960	Gje1	NM_080450	2.23297e-010	9.0516	E15.5 * Wnt1 up vs E15.5 * Ctrl
10464370	Slc18a2	NM_172523	1.66625e-013	9.03062	E15.5 * Wnt1 up vs E15.5 * Ctrl
10411274	Sv2c	NM_029210	9.02971e-014	8.902	E15.5 * Wnt1 up vs E15.5 * Ctrl
10418921	Sncg	NM_011430	3.18098e-012	8.868	E15.5 * Wnt1 up vs E15.5 * Ctrl
10483326	Scn9a	NM_018852	8.67147e-012	8.72389	E15.5 * Wnt1 up vs E15.5 * Ctrl
10604751	Fgf13	NM_010200	6.60785e-011	8.71634	E15.5 * Wnt1 up vs E15.5 * Ctrl
10431812	Nell2	NM_016743	1.05537e-013	8.6345	E15.5 * Wnt1 up vs E15.5 * Ctrl
10421100	Nefm	NM_008691	1.98813e-016	8.5597	E15.5 * Wnt1 up vs E15.5 * Ctrl
10426656	Prph	NM_013639	1.82696e-013	8.47914	E15.5 * Wnt1 up vs E15.5 * Ctrl
10494069	Tnrc4	NM_172434	1.86924e-009	8.42772	E15.5 * Wnt1 up vs E15.5 * Ctrl
10350951	Tnr	NM_022312	8.08306e-013	8.34672	E15.5 * Wnt1 up vs E15.5 * Ctrl
10413461	Erc2	NM_177814	1.75274e-012	8.29396	E15.5 * Wnt1 up vs E15.5 * Ctrl
10588283	Rab6b	NM_173781	9.47185e-011	8.26147	E15.5 * Wnt1 up vs E15.5 * Ctrl
10368999	Grik2	NM_010349	4.60352e-013	8.25664	E15.5 * Wnt1 up vs E15.5 * Ctrl
10503416	Calb1	NM_009788	2.53027e-013	8.24463	E15.5 * Wnt1 up vs E15.5 * Ctrl
10433431		---	8.675e-006	8.07237	E15.5 * Wnt1 up vs E15.5 * Ctrl
10359113	Fam163a	NM_177838	7.10162e-011	7.98286	E15.5 * Wnt1 up vs E15.5 * Ctrl
10479698	Myt1	NM_008665	4.87351e-012	7.92744	E15.5 * Wnt1 up vs E15.5 * Ctrl
10469577	ENSMUSG0000C	ENSMUST00000	3.44767e-011	7.91795	E15.5 * Wnt1 up vs E15.5 * Ctrl
10530306	Phox2b	NM_008888	1.94523e-012	7.87775	E15.5 * Wnt1 up vs E15.5 * Ctrl
10412066	Rab3c	NM_023852	3.73715e-012	7.86976	E15.5 * Wnt1 up vs E15.5 * Ctrl
10545538	Ctnna2	NM_009819	2.62017e-012	7.85746	E15.5 * Wnt1 up vs E15.5 * Ctrl
10353192	Eya1	NM_010164	4.24921e-012	7.81819	E15.5 * Wnt1 up vs E15.5 * Ctrl
10406461	C130071C03Rik	ENSMUST00000(	3.66937e-011	7.75892	E15.5 * Wnt1 up vs E15.5 * Ctrl
10606174	Nap1l2	NM_008671	1.58757e-010	7.72966	E15.5 * Wnt1 up vs E15.5 * Ctrl
10451838	Slc5a7	NM_022025	2.36336e-011	7.71995	E15.5 * Wnt1 up vs E15.5 * Ctrl
10490665	Stmn3	NM_009133	1.02818e-012	7.6703	E15.5 * Wnt1 up vs E15.5 * Ctrl
10497663	Slc7a14	NM_172861	9.51067e-013	7.64687	E15.5 * Wnt1 up vs E15.5 * Ctrl
10355960	Scg2	NM_009129	2.70794e-010	7.59689	E15.5 * Wnt1 up vs E15.5 * Ctrl
10395553	Nrcam	NM_176930	4.00217e-013	7.43253	E15.5 * Wnt1 up vs E15.5 * Ctrl
10575019	ENSMUSG0000C	ENSMUST00000(	2.68033e-007	7.42899	E15.5 * Wnt1 up vs E15.5 * Ctrl
10428515	Csmd3	NM_001081391	4.51613e-009	7.33348	E15.5 * Wnt1 up vs E15.5 * Ctrl
10595013	Tmod2	NM_016711	4.47312e-015	7.27407	E15.5 * Wnt1 up vs E15.5 * Ctrl
10599841	C230004F18Rik	ENSMUST00000(	1.63656e-010	7.23922	E15.5 * Wnt1 up vs E15.5 * Ctrl
10599187	Zcchc12	NM_028325	3.52971e-012	7.22389	E15.5 * Wnt1 up vs E15.5 * Ctrl
10483324	Scn9a	NM_018852	1.24776e-010	7.19247	E15.5 * Wnt1 up vs E15.5 * Ctrl
10436623	Chodl	NM_139134	3.87171e-009	7.17633	E15.5 * Wnt1 up vs E15.5 * Ctrl
10456237	St8sia3	NM_009182	1.10491e-011	7.1469	E15.5 * Wnt1 up vs E15.5 * Ctrl
10458052	Epb4.1l4a	NM_013512	5.1048e-014	7.13987	E15.5 * Wnt1 up vs E15.5 * Ctrl
10594048	Islr2	NM_177193	4.57148e-012	7.08939	E15.5 * Wnt1 up vs E15.5 * Ctrl
10495878	Ndst4	NM_022565	5.14098e-009	7.08135	E15.5 * Wnt1 up vs E15.5 * Ctrl
10385283	Gabrg2	NM_008073	2.98743e-011	7.06013	E15.5 * Wnt1 up vs E15.5 * Ctrl
10363146	Slc35f1	NM_178675	4.92571e-011	7.03191	E15.5 * Wnt1 up vs E15.5 * Ctrl
10563858	Gabrg3	NM_008074	3.03513e-011	7.02148	E15.5 * Wnt1 up vs E15.5 * Ctrl
10595496	Snap91	NM_013669	2.42797e-011	6.9366	E15.5 * Wnt1 up vs E15.5 * Ctrl
10530499	EG545758	NM_001024147	2.58015e-012	6.93433	E15.5 * Wnt1 up vs E15.5 * Ctrl
10397912	9030205A07Rik	AB257853	5.64839e-014	6.9251	E15.5 * Wnt1 up vs E15.5 * Ctrl
10355259	Myl1	NM_021285	5.10869e-010	6.91029	E15.5 * Wnt1 up vs E15.5 * Ctrl
10396125	Atl1	NM_178628	2.61149e-011	6.86321	E15.5 * Wnt1 up vs E15.5 * Ctrl
10369835	Phyhipl	NM_178621	2.03267e-012	6.81726	E15.5 * Wnt1 up vs E15.5 * Ctrl
10456353	Grp	NM_175012	1.6205e-010	6.7869	E15.5 * Wnt1 up vs E15.5 * Ctrl
10501468	Ntng1	NM_030699	1.41228e-011	6.77294	E15.5 * Wnt1 up vs E15.5 * Ctrl
10495623	ENSMUSG0000C	ENSMUST00000(	5.25618e-009	6.75089	E15.5 * Wnt1 up vs E15.5 * Ctrl
10494043	Tdrkh	NM_028307	5.25957e-013	6.66719	E15.5 * Wnt1 up vs E15.5 * Ctrl
10351400	Fam78b	NM_175461	1.2323e-012	6.66256	E15.5 * Wnt1 up vs E15.5 * Ctrl
10423855	Rims2	NM_053271	2.15207e-012	6.63462	E15.5 * Wnt1 up vs E15.5 * Ctrl
10508052	Grik3	NM_001081097	9.64108e-011	6.60492	E15.5 * Wnt1 up vs E15.5 * Ctrl
10585048	Cadm1	NM_207675	1.52241e-015	6.59627	E15.5 * Wnt1 up vs E15.5 * Ctrl
10362363	6330407J23Rik	NM_026138	8.24202e-013	6.58516	E15.5 * Wnt1 up vs E15.5 * Ctrl
10472372	Scn2a1	NM_001099298	6.24485e-011	6.55082	E15.5 * Wnt1 up vs E15.5 * Ctrl
10584259	Fez1	NM_183171	4.92397e-015	6.52743	E15.5 * Wnt1 up vs E15.5 * Ctrl
10428522	Csmd3	NM_001081391	1.36606e-009	6.46778	E15.5 * Wnt1 up vs E15.5 * Ctrl
10363921	Pcdh15	NM_023115	1.47828e-011	6.45579	E15.5 * Wnt1 up vs E15.5 * Ctrl
10533055	1500001A10Rik	NM_026886	1.85637e-014	6.45186	E15.5 * Wnt1 up vs E15.5 * Ctrl
10485309	E530001K10Rik	ENSMUST00000(	1.35795e-008	6.44758	E15.5 * Wnt1 up vs E15.5 * Ctrl
10483215	Scn3a	NM_018732	2.33941e-011	6.43239	E15.5 * Wnt1 up vs E15.5 * Ctrl

10500114	Mllt11	NM_019914	4.35608e-015	6.40057	E15.5 * Wnt1 up vs E15.5 * Ctrl
10421934	Klhl1	NM_053105	1.52132e-011	6.35435	E15.5 * Wnt1 up vs E15.5 * Ctrl
10419049	Nrg3	NM_008734	1.13081e-010	6.24329	E15.5 * Wnt1 up vs E15.5 * Ctrl
10422537	Nalcn	NM_177393	6.83327e-013	6.23771	E15.5 * Wnt1 up vs E15.5 * Ctrl
10436658	7120432I05Rik	AK148667	4.10823e-007	6.19028	E15.5 * Wnt1 up vs E15.5 * Ctrl
10529937	Kcnip4	NM_030265	5.76006e-012	6.14248	E15.5 * Wnt1 up vs E15.5 * Ctrl
10472398	Scn2a1	NM_001099298	3.38957e-007	6.13541	E15.5 * Wnt1 up vs E15.5 * Ctrl
10564211	Snrpn	NM_013670	1.59159e-016	6.06582	E15.5 * Wnt1 up vs E15.5 * Ctrl
10455374	Stk32a	NM_178749	1.38713e-012	5.99779	E15.5 * Wnt1 up vs E15.5 * Ctrl
10445879	Kcnh8	NM_001031811	5.68014e-010	5.99652	E15.5 * Wnt1 up vs E15.5 * Ctrl
10407841	Hecw1	NM_001081348	1.63789e-009	5.9823	E15.5 * Wnt1 up vs E15.5 * Ctrl
10522976	Rufy3	NM_027530	5.32712e-016	5.97573	E15.5 * Wnt1 up vs E15.5 * Ctrl
10349208	Cntnap5a	NM_001077425	7.11423e-012	5.92471	E15.5 * Wnt1 up vs E15.5 * Ctrl
10555197	Mtap6	NM_010837	1.23197e-013	5.8956	E15.5 * Wnt1 up vs E15.5 * Ctrl
10427862	Cdh6	NM_007666	1.43402e-013	5.8847	E15.5 * Wnt1 up vs E15.5 * Ctrl
10363224	Fabp7	NM_021272	8.40975e-012	5.83305	E15.5 * Wnt1 up vs E15.5 * Ctrl
10525016	Tbx3	NM_011535	1.73461e-015	5.79947	E15.5 * Wnt1 up vs E15.5 * Ctrl
10503180	Chd7	NM_001081417	3.94553e-012	5.74286	E15.5 * Wnt1 up vs E15.5 * Ctrl
10553773	Gabrb3	NM_008071	2.77177e-013	5.72372	E15.5 * Wnt1 up vs E15.5 * Ctrl
10519770	Pclo	NM_011995	1.02136e-011	5.718	E15.5 * Wnt1 up vs E15.5 * Ctrl
10350506	Fam5c	NM_153539	6.68362e-012	5.69339	E15.5 * Wnt1 up vs E15.5 * Ctrl
10472809	Dlx1	NM_010053	8.53452e-013	5.69287	E15.5 * Wnt1 up vs E15.5 * Ctrl
10435765		---	4.48966e-007	5.66954	E15.5 * Wnt1 up vs E15.5 * Ctrl
10468452	Sorcs1	NM_021377	1.15744e-013	5.64207	E15.5 * Wnt1 up vs E15.5 * Ctrl
10474814	Disp2	NM_170593	1.10428e-010	5.63539	E15.5 * Wnt1 up vs E15.5 * Ctrl
10373467	Erbb3	NM_010153	2.13015e-010	5.53011	E15.5 * Wnt1 up vs E15.5 * Ctrl
10419060		---	0.00076464	5.46978	E15.5 * Wnt1 up vs E15.5 * Ctrl
10503234	Rlbp1l1	NM_028940	6.0136e-014	5.46801	E15.5 * Wnt1 up vs E15.5 * Ctrl
10439362	Stxbp5l	NM_172440	3.51081e-011	5.44768	E15.5 * Wnt1 up vs E15.5 * Ctrl
10457644	Cdh2	NM_007664	2.04791e-016	5.42684	E15.5 * Wnt1 up vs E15.5 * Ctrl
10389590	Gdpd1	NM_025638	1.96958e-012	5.42512	E15.5 * Wnt1 up vs E15.5 * Ctrl
10373113	Kif5a	NM_008447	1.57087e-010	5.38792	E15.5 * Wnt1 up vs E15.5 * Ctrl
10501555	Amy1	NM_007446	1.1666e-007	5.37799	E15.5 * Wnt1 up vs E15.5 * Ctrl
10423243	Cdh10	NM_009865	6.28914e-010	5.35963	E15.5 * Wnt1 up vs E15.5 * Ctrl
10491477	Sox2	NM_011443	9.45792e-009	5.35901	E15.5 * Wnt1 up vs E15.5 * Ctrl
10411519	Mtap1b	NM_008634	6.88336e-013	5.35776	E15.5 * Wnt1 up vs E15.5 * Ctrl
10347781	9430031J16Rik	BC082310	4.36568e-011	5.35488	E15.5 * Wnt1 up vs E15.5 * Ctrl
10520527	Dpysl5	NM_023047	9.83675e-012	5.34481	E15.5 * Wnt1 up vs E15.5 * Ctrl
10360418	Rgs7	NM_011880	7.16662e-010	5.31104	E15.5 * Wnt1 up vs E15.5 * Ctrl
10432675	I730030J21Rik	ENSMUST00000	4.15133e-007	5.30647	E15.5 * Wnt1 up vs E15.5 * Ctrl
10344679	St18	NM_173868	1.62296e-011	5.29488	E15.5 * Wnt1 up vs E15.5 * Ctrl
10499988	2310007A19Rik	NM_025506	7.32337e-007	5.29074	E15.5 * Wnt1 up vs E15.5 * Ctrl
10355225	ENSMUSG00000	ENSMUST00000	1.95632e-010	5.25788	E15.5 * Wnt1 up vs E15.5 * Ctrl
10408613	Tubb2b	NM_023716	8.21935e-010	5.25564	E15.5 * Wnt1 up vs E15.5 * Ctrl
10354229	2610017I09Rik	BC058417	6.62947e-010	5.23644	E15.5 * Wnt1 up vs E15.5 * Ctrl
10462912	Lgi1	NM_020278	1.02542e-006	5.19929	E15.5 * Wnt1 up vs E15.5 * Ctrl
10603266	Nudt10	NM_001031664	7.03139e-006	5.15915	E15.5 * Wnt1 up vs E15.5 * Ctrl
10530319	Atp8a1	NM_001038999	6.32009e-016	5.13152	E15.5 * Wnt1 up vs E15.5 * Ctrl
10418092	A830039N20Rik	BC038501	2.24867e-008	5.11775	E15.5 * Wnt1 up vs E15.5 * Ctrl
10350438	Kcnt2	NM_001081027	5.50717e-009	5.11	E15.5 * Wnt1 up vs E15.5 * Ctrl
10593245	Htr3b	NM_020274	2.73539e-010	5.09653	E15.5 * Wnt1 up vs E15.5 * Ctrl
10599884		---	1.80808e-005	5.08524	E15.5 * Wnt1 up vs E15.5 * Ctrl
10488387	Napb	NM_019632	1.62044e-012	5.08314	E15.5 * Wnt1 up vs E15.5 * Ctrl
10503198	Chd7	NM_001081417	2.20189e-010	5.06011	E15.5 * Wnt1 up vs E15.5 * Ctrl
10359480	Dnm3	NM_001038619	1.18174e-011	5.01312	E15.5 * Wnt1 up vs E15.5 * Ctrl
10474373	Kcna4	NM_021275	4.63186e-009	4.99659	E15.5 * Wnt1 up vs E15.5 * Ctrl
10490856	Ralyl	NM_178631	3.1913e-011	4.97253	E15.5 * Wnt1 up vs E15.5 * Ctrl
10411395	Rgnef	NM_012026	2.64607e-012	4.95885	E15.5 * Wnt1 up vs E15.5 * Ctrl
10472386	Scn2a1	NM_001099298	3.75776e-009	4.92827	E15.5 * Wnt1 up vs E15.5 * Ctrl
10503194	Chd7	NM_001081417	3.64934e-009	4.91459	E15.5 * Wnt1 up vs E15.5 * Ctrl
10395702	Akap6	NM_198111	1.20255e-013	4.9123	E15.5 * Wnt1 up vs E15.5 * Ctrl
10503176	Chd7	NM_001081417	4.47027e-012	4.89956	E15.5 * Wnt1 up vs E15.5 * Ctrl
10406782	Fam169a	NM_001100458	7.58544e-009	4.89109	E15.5 * Wnt1 up vs E15.5 * Ctrl
10457820	Nol4	NM_199024	1.41455e-010	4.88587	E15.5 * Wnt1 up vs E15.5 * Ctrl
10568109	Asphd1	NM_001039645	4.73304e-011	4.87862	E15.5 * Wnt1 up vs E15.5 * Ctrl
10440465		---	9.17229e-006	4.82576	E15.5 * Wnt1 up vs E15.5 * Ctrl

10423358	March11	NM_177597	2.395e-006	4.79004	E15.5 * Wnt1 up vs E15.5 * Ctrl
10543058	Dlx5	NM_010056	4.01573e-008	4.78075	E15.5 * Wnt1 up vs E15.5 * Ctrl
10356712	Kif1a	NM_008440	2.13744e-013	4.76771	E15.5 * Wnt1 up vs E15.5 * Ctrl
10472402	Scn2a1	NM_001099298	8.63298e-010	4.748	E15.5 * Wnt1 up vs E15.5 * Ctrl
10519612	9330182L06Rik	NM_172706	6.40699e-011	4.72431	E15.5 * Wnt1 up vs E15.5 * Ctrl
10356154	Sphkap	NM_172430	3.18838e-009	4.72116	E15.5 * Wnt1 up vs E15.5 * Ctrl
10362005	Ahi1	NM_026203	1.97292e-011	4.70311	E15.5 * Wnt1 up vs E15.5 * Ctrl
10571599		---	1.10047e-007	4.69011	E15.5 * Wnt1 up vs E15.5 * Ctrl
10370242	Pcbp3	NM_021568	6.59678e-013	4.68762	E15.5 * Wnt1 up vs E15.5 * Ctrl
10503200	Chd7	NM_001081417	2.33023e-010	4.68346	E15.5 * Wnt1 up vs E15.5 * Ctrl
10503186	Chd7	NM_001081417	9.80816e-013	4.6676	E15.5 * Wnt1 up vs E15.5 * Ctrl
10392484	Abca8b	NM_013851	1.15982e-008	4.66697	E15.5 * Wnt1 up vs E15.5 * Ctrl
10426244	Mapk8ip2	NM_021921	1.29631e-009	4.65838	E15.5 * Wnt1 up vs E15.5 * Ctrl
10503172	Chd7	NM_001081417	6.0156e-007	4.64598	E15.5 * Wnt1 up vs E15.5 * Ctrl
10571399	Zdhhc2	NM_178395	3.99467e-010	4.63503	E15.5 * Wnt1 up vs E15.5 * Ctrl
10374356	Vstm2a	NM_145967	3.04665e-009	4.63453	E15.5 * Wnt1 up vs E15.5 * Ctrl
10366457	Ptprr	NM_011217	4.42471e-011	4.60871	E15.5 * Wnt1 up vs E15.5 * Ctrl
10548030	Cd9	NM_007657	3.44132e-010	4.59716	E15.5 * Wnt1 up vs E15.5 * Ctrl
10568107		---	1.86528e-006	4.5926	E15.5 * Wnt1 up vs E15.5 * Ctrl
10591706	Elavl3	NM_010487	6.05492e-010	4.58724	E15.5 * Wnt1 up vs E15.5 * Ctrl
10454369	Fhod3	NM_175276	5.9079e-011	4.57965	E15.5 * Wnt1 up vs E15.5 * Ctrl
10602805	Mtap7d2	NM_001081124	1.79747e-011	4.57794	E15.5 * Wnt1 up vs E15.5 * Ctrl
10487139	Shc4	NM_199022	3.65814e-013	4.56536	E15.5 * Wnt1 up vs E15.5 * Ctrl
10428517	Csmd3	NM_001081391	2.40734e-011	4.55248	E15.5 * Wnt1 up vs E15.5 * Ctrl
10592420	AW551984	NM_178737	1.68762e-012	4.54868	E15.5 * Wnt1 up vs E15.5 * Ctrl
10503184	Chd7	NM_001081417	1.18876e-013	4.53108	E15.5 * Wnt1 up vs E15.5 * Ctrl
10606835	Bex2	NM_009749	4.14891e-011	4.51498	E15.5 * Wnt1 up vs E15.5 * Ctrl
10503196	Chd7	NM_001081417	5.87725e-011	4.5141	E15.5 * Wnt1 up vs E15.5 * Ctrl
10529903	Fam184b	NM_021416	8.12828e-010	4.5123	E15.5 * Wnt1 up vs E15.5 * Ctrl
10503182	Chd7	NM_001081417	8.44264e-014	4.50013	E15.5 * Wnt1 up vs E15.5 * Ctrl
10472396	Scn2a1	NM_001099298	1.28594e-008	4.4895	E15.5 * Wnt1 up vs E15.5 * Ctrl
10368101	D10Bwg1379e	NM_001033258	8.71368e-012	4.48521	E15.5 * Wnt1 up vs E15.5 * Ctrl
10564011	Snord115	AF357427	7.18154e-010	4.47631	E15.5 * Wnt1 up vs E15.5 * Ctrl
10563913		---	8.95698e-010	4.47505	E15.5 * Wnt1 up vs E15.5 * Ctrl
10563917		---	8.95698e-010	4.47505	E15.5 * Wnt1 up vs E15.5 * Ctrl
10563923	100040985	XM_001475615	8.95698e-010	4.47505	E15.5 * Wnt1 up vs E15.5 * Ctrl
10400609	Mdga2	NM_207010	6.36412e-011	4.44599	E15.5 * Wnt1 up vs E15.5 * Ctrl
10543802	Plxna4	NM_175750	8.60272e-012	4.44598	E15.5 * Wnt1 up vs E15.5 * Ctrl
10526559	Ache	NM_009599	1.01138e-008	4.41596	E15.5 * Wnt1 up vs E15.5 * Ctrl
10480492	Cacna1b	NM_001042528	3.68552e-014	4.3955	E15.5 * Wnt1 up vs E15.5 * Ctrl
10601888	Plp1	NM_011123	2.73043e-009	4.38939	E15.5 * Wnt1 up vs E15.5 * Ctrl
10586591	Car12	NM_178396	9.97956e-011	4.37721	E15.5 * Wnt1 up vs E15.5 * Ctrl
10503902	Cnr1	NM_007726	1.4754e-010	4.3577	E15.5 * Wnt1 up vs E15.5 * Ctrl
10598236	Nudt11	NM_021431	2.97562e-009	4.3535	E15.5 * Wnt1 up vs E15.5 * Ctrl
10375019	Nsg2	NM_008741	6.71764e-011	4.34577	E15.5 * Wnt1 up vs E15.5 * Ctrl
10478374	Gdap1l1	NM_144891	2.30602e-011	4.34116	E15.5 * Wnt1 up vs E15.5 * Ctrl
10380641	Hoxb5	NM_008268	1.73139e-014	4.30643	E15.5 * Wnt1 up vs E15.5 * Ctrl
10395074	Myt1l	NM_001093775	5.01655e-014	4.30091	E15.5 * Wnt1 up vs E15.5 * Ctrl
10417787	Gng2	NM_010315	1.17099e-013	4.27991	E15.5 * Wnt1 up vs E15.5 * Ctrl
10518585	Kif1b	NM_207682	3.66709e-014	4.26483	E15.5 * Wnt1 up vs E15.5 * Ctrl
10354506	Mfsd6	NM_133829	5.05769e-009	4.26256	E15.5 * Wnt1 up vs E15.5 * Ctrl
10457942	Syt4	NM_009308	7.14247e-010	4.25743	E15.5 * Wnt1 up vs E15.5 * Ctrl
10458767	Trim36	NM_178872	1.16027e-010	4.25637	E15.5 * Wnt1 up vs E15.5 * Ctrl
10537300	1700111E14Rik	ENSMUST00000	1.93981e-007	4.25336	E15.5 * Wnt1 up vs E15.5 * Ctrl
10368997	C130030K03Rik	AK048022	7.13157e-006	4.24639	E15.5 * Wnt1 up vs E15.5 * Ctrl
10407481	Pfkp	NM_019703	4.60561e-011	4.23916	E15.5 * Wnt1 up vs E15.5 * Ctrl
10593671	Dmxl2	NM_172771	7.56352e-013	4.23113	E15.5 * Wnt1 up vs E15.5 * Ctrl
10548043	Kcna5	NM_145983	3.24676e-008	4.22604	E15.5 * Wnt1 up vs E15.5 * Ctrl
10503222	Chd7	NM_001081417	3.15811e-011	4.21941	E15.5 * Wnt1 up vs E15.5 * Ctrl
10529953	ENSMUSG0000C	AK036806	5.62548e-006	4.2098	E15.5 * Wnt1 up vs E15.5 * Ctrl
10400126	Lrrn3	NM_010733	2.24275e-013	4.20074	E15.5 * Wnt1 up vs E15.5 * Ctrl
10345241	Dst	NM_134448	3.79717e-015	4.17122	E15.5 * Wnt1 up vs E15.5 * Ctrl
10361642	Lrp11	NM_172784	3.47597e-009	4.1685	E15.5 * Wnt1 up vs E15.5 * Ctrl
10560919	Atp1a3	NM_144921	2.60357e-008	4.15567	E15.5 * Wnt1 up vs E15.5 * Ctrl
10400607	Mdga2	AK163637	1.29235e-008	4.15418	E15.5 * Wnt1 up vs E15.5 * Ctrl
10573979	Gnao1	NM_010308	1.51071e-012	4.1529	E15.5 * Wnt1 up vs E15.5 * Ctrl

10435733	Igsf11	NM_170599	8.44344e-011	4.14948	E15.5 * Wnt1 up vs E15.5 * Ctrl
10590663	Gria4	NM_019691	4.23395e-012	4.14535	E15.5 * Wnt1 up vs E15.5 * Ctrl
10563937	Snord115	AF357427	1.51043e-008	4.14348	E15.5 * Wnt1 up vs E15.5 * Ctrl
10563947	Snord115	AF357427	1.51043e-008	4.14348	E15.5 * Wnt1 up vs E15.5 * Ctrl
10563951	Snord115	AF357427	1.51043e-008	4.14348	E15.5 * Wnt1 up vs E15.5 * Ctrl
10563953	Snord115	AF357427	1.51043e-008	4.14348	E15.5 * Wnt1 up vs E15.5 * Ctrl
10563957	Snord115	AF357427	1.51043e-008	4.14348	E15.5 * Wnt1 up vs E15.5 * Ctrl
10563995	Snord115	AF357427	1.51043e-008	4.14348	E15.5 * Wnt1 up vs E15.5 * Ctrl
10563997	Snord115	AF357427	1.51043e-008	4.14348	E15.5 * Wnt1 up vs E15.5 * Ctrl
10563999	Snord115	AF357427	1.51043e-008	4.14348	E15.5 * Wnt1 up vs E15.5 * Ctrl
10564001	Snord115	AF357427	1.51043e-008	4.14348	E15.5 * Wnt1 up vs E15.5 * Ctrl
10564003	Snord115	AF357427	1.51043e-008	4.14348	E15.5 * Wnt1 up vs E15.5 * Ctrl
10564007	Snord115	AF357427	1.51043e-008	4.14348	E15.5 * Wnt1 up vs E15.5 * Ctrl
10564029	Snord115	AF357427	1.51043e-008	4.14348	E15.5 * Wnt1 up vs E15.5 * Ctrl
10564031	Snord115	AF357427	1.51043e-008	4.14348	E15.5 * Wnt1 up vs E15.5 * Ctrl
10564035		---	1.51043e-008	4.14348	E15.5 * Wnt1 up vs E15.5 * Ctrl
10564039		---	1.51043e-008	4.14348	E15.5 * Wnt1 up vs E15.5 * Ctrl
10564051		---	1.51043e-008	4.14348	E15.5 * Wnt1 up vs E15.5 * Ctrl
10564059		---	1.51043e-008	4.14348	E15.5 * Wnt1 up vs E15.5 * Ctrl
10564061		---	1.51043e-008	4.14348	E15.5 * Wnt1 up vs E15.5 * Ctrl
10564063		---	1.51043e-008	4.14348	E15.5 * Wnt1 up vs E15.5 * Ctrl
10564065	100040985	XM_001475615	1.51043e-008	4.14348	E15.5 * Wnt1 up vs E15.5 * Ctrl
10564067		---	1.51043e-008	4.14348	E15.5 * Wnt1 up vs E15.5 * Ctrl
10564071		---	1.51043e-008	4.14348	E15.5 * Wnt1 up vs E15.5 * Ctrl
10564075		---	1.51043e-008	4.14348	E15.5 * Wnt1 up vs E15.5 * Ctrl
10564081		---	1.51043e-008	4.14348	E15.5 * Wnt1 up vs E15.5 * Ctrl
10564083		---	1.51043e-008	4.14348	E15.5 * Wnt1 up vs E15.5 * Ctrl
10564131		---	1.51043e-008	4.14348	E15.5 * Wnt1 up vs E15.5 * Ctrl
10564133		---	1.51043e-008	4.14348	E15.5 * Wnt1 up vs E15.5 * Ctrl
10564141		---	1.51043e-008	4.14348	E15.5 * Wnt1 up vs E15.5 * Ctrl
10564145		---	1.51043e-008	4.14348	E15.5 * Wnt1 up vs E15.5 * Ctrl
10564149		---	1.51043e-008	4.14348	E15.5 * Wnt1 up vs E15.5 * Ctrl
10564151		---	1.51043e-008	4.14348	E15.5 * Wnt1 up vs E15.5 * Ctrl
10564153		---	1.51043e-008	4.14348	E15.5 * Wnt1 up vs E15.5 * Ctrl
10564155		---	1.51043e-008	4.14348	E15.5 * Wnt1 up vs E15.5 * Ctrl
10367982	Gpr126	NM_001002268	5.53718e-011	4.1413	E15.5 * Wnt1 up vs E15.5 * Ctrl
10503202	Chd7	NM_001081417	4.34473e-011	4.13928	E15.5 * Wnt1 up vs E15.5 * Ctrl
10428407	Tmem74	NM_175502	2.07433e-010	4.10226	E15.5 * Wnt1 up vs E15.5 * Ctrl
10441195	Dscam	NM_031174	2.3627e-010	4.09826	E15.5 * Wnt1 up vs E15.5 * Ctrl
10433428		---	3.86365e-006	4.09235	E15.5 * Wnt1 up vs E15.5 * Ctrl
10564041		---	7.29296e-009	4.08502	E15.5 * Wnt1 up vs E15.5 * Ctrl
10571567	Sorbs2	NM_172752	1.93686e-013	4.08193	E15.5 * Wnt1 up vs E15.5 * Ctrl
10360920	Tgfb2	NM_009367	4.05072e-018	4.07046	E15.5 * Wnt1 up vs E15.5 * Ctrl
10381776	Mapt	NM_001038609	1.70065e-010	4.06901	E15.5 * Wnt1 up vs E15.5 * Ctrl
10556067	Syt9	NM_021889	1.91178e-011	4.06341	E15.5 * Wnt1 up vs E15.5 * Ctrl
10599880	Slitrk2	NM_198863	1.76939e-010	4.05889	E15.5 * Wnt1 up vs E15.5 * Ctrl
10503166	Chd7	NM_001081417	2.87404e-008	4.04695	E15.5 * Wnt1 up vs E15.5 * Ctrl
10598996	EG331392	NM_198633	2.66867e-010	4.04493	E15.5 * Wnt1 up vs E15.5 * Ctrl
10518812	Camta1	NM_001081557	5.11344e-011	4.03519	E15.5 * Wnt1 up vs E15.5 * Ctrl
10366144	Mgat4c	NM_026243	1.23248e-009	4.03286	E15.5 * Wnt1 up vs E15.5 * Ctrl
10539310	Pcgf1	NM_197992	3.68285e-009	4.02485	E15.5 * Wnt1 up vs E15.5 * Ctrl
10503214	Chd7	NM_001081417	4.17707e-010	4.02368	E15.5 * Wnt1 up vs E15.5 * Ctrl
10399892	Gpr22	NM_175191	4.8141e-011	4.02277	E15.5 * Wnt1 up vs E15.5 * Ctrl
10503218	Chd7	NM_001081417	6.04692e-007	4.02067	E15.5 * Wnt1 up vs E15.5 * Ctrl
10521602	Cpeb2	NM_175937	5.48077e-009	4.02011	E15.5 * Wnt1 up vs E15.5 * Ctrl
10435752	Lsamp	NM_175548	1.49842e-012	4.01126	E15.5 * Wnt1 up vs E15.5 * Ctrl
10514561	E130114P18Rik	ENSMUST00000	6.57417e-010	3.98604	E15.5 * Wnt1 up vs E15.5 * Ctrl
10506274	Dnajc6	NM_198412	1.22449e-012	3.97205	E15.5 * Wnt1 up vs E15.5 * Ctrl
10472384	Scn2a1	NM_001099298	1.44259e-007	3.971	E15.5 * Wnt1 up vs E15.5 * Ctrl
10596583	Dock3	NM_153413	2.18273e-011	3.97082	E15.5 * Wnt1 up vs E15.5 * Ctrl
10452295	Tubb4	NM_009451	3.73336e-009	3.95454	E15.5 * Wnt1 up vs E15.5 * Ctrl
10353632	Bai3	NM_175642	1.92891e-011	3.95248	E15.5 * Wnt1 up vs E15.5 * Ctrl
10415784	Trim13	NM_023233	1.39359e-007	3.94829	E15.5 * Wnt1 up vs E15.5 * Ctrl
10606554	Nap1l3	NM_138742	3.07718e-010	3.91663	E15.5 * Wnt1 up vs E15.5 * Ctrl
10605431	Rab39b	NM_175122	1.67832e-009	3.90083	E15.5 * Wnt1 up vs E15.5 * Ctrl
10399419	Tubb2b	NM_023716	1.4546e-010	3.88953	E15.5 * Wnt1 up vs E15.5 * Ctrl

10350896	Astn1	NM_007495	7.20233e-011	3.86931	E15.5 * Wnt1 up vs E15.5 * Ctrl
10405343	Tspan17	NM_028841	6.3931e-011	3.85873	E15.5 * Wnt1 up vs E15.5 * Ctrl
10601903	Zcchc18	NM_001035510	4.39125e-009	3.84899	E15.5 * Wnt1 up vs E15.5 * Ctrl
10425726	03-Sep	NM_011889	2.52201e-013	3.83878	E15.5 * Wnt1 up vs E15.5 * Ctrl
10513061	Ctnnal1	NM_018761	1.30821e-012	3.83389	E15.5 * Wnt1 up vs E15.5 * Ctrl
10593668	Dmxl2	NM_172771	1.59331e-009	3.82735	E15.5 * Wnt1 up vs E15.5 * Ctrl
10589800	Clasp2	NM_001114347	1.80575e-015	3.82085	E15.5 * Wnt1 up vs E15.5 * Ctrl
10503174	Chd7	NM_001081417	4.51658e-011	3.81452	E15.5 * Wnt1 up vs E15.5 * Ctrl
10587241	Hmgcll1	NM_173731	4.82303e-009	3.8059	E15.5 * Wnt1 up vs E15.5 * Ctrl
10573172	Clgn	NM_009904	2.71134e-010	3.78857	E15.5 * Wnt1 up vs E15.5 * Ctrl
10602692	Rragb	NM_001004154	2.56588e-009	3.78692	E15.5 * Wnt1 up vs E15.5 * Ctrl
10472380	Scn2a1	NM_001099298	1.21472e-006	3.78321	E15.5 * Wnt1 up vs E15.5 * Ctrl
10345212	Khdrbs2	NM_133235	2.4032e-009	3.78248	E15.5 * Wnt1 up vs E15.5 * Ctrl
10366196	Ppfia2	NM_177373	2.1734e-011	3.78088	E15.5 * Wnt1 up vs E15.5 * Ctrl
10495625	Dpyd	NM_170778	6.65595e-010	3.78045	E15.5 * Wnt1 up vs E15.5 * Ctrl
10544875	Scrn1	NM_027268	2.88413e-008	3.7722	E15.5 * Wnt1 up vs E15.5 * Ctrl
10443108	Syngap1	XM_985548	3.60064e-005	3.76427	E15.5 * Wnt1 up vs E15.5 * Ctrl
10388465	Doc2b	NM_007873	6.55713e-011	3.76245	E15.5 * Wnt1 up vs E15.5 * Ctrl
10501971	Ank2	NM_178655	2.75809e-012	3.73371	E15.5 * Wnt1 up vs E15.5 * Ctrl
10541260	Cecr2	NM_001128151	2.10663e-009	3.73014	E15.5 * Wnt1 up vs E15.5 * Ctrl
10472378	Scn2a1	NM_001099298	3.07978e-005	3.72878	E15.5 * Wnt1 up vs E15.5 * Ctrl
10481772	Garnl3	NM_178888	1.96149e-011	3.72614	E15.5 * Wnt1 up vs E15.5 * Ctrl
10542896	Bicd1	NM_009753	1.22209e-010	3.72612	E15.5 * Wnt1 up vs E15.5 * Ctrl
10542414	Ptpro	NM_011216	6.55283e-010	3.71909	E15.5 * Wnt1 up vs E15.5 * Ctrl
10540401	Lrrn1	NM_008516	3.19004e-013	3.71317	E15.5 * Wnt1 up vs E15.5 * Ctrl
10526564	Ufsp1	NM_027356	2.98263e-009	3.71308	E15.5 * Wnt1 up vs E15.5 * Ctrl
10602198	Pak3	NM_008778	4.75211e-010	3.69564	E15.5 * Wnt1 up vs E15.5 * Ctrl
10371578	Ascl1	NM_008553	1.51851e-006	3.69196	E15.5 * Wnt1 up vs E15.5 * Ctrl
10557177	Prkcb	NM_008855	1.97736e-009	3.68924	E15.5 * Wnt1 up vs E15.5 * Ctrl
10457606	Kctd1	NM_134112	6.81182e-010	3.68346	E15.5 * Wnt1 up vs E15.5 * Ctrl
10503188	Chd7	NM_001081417	4.74302e-009	3.68313	E15.5 * Wnt1 up vs E15.5 * Ctrl
10568982	BC066028	NM_001001180	1.64258e-009	3.67997	E15.5 * Wnt1 up vs E15.5 * Ctrl
10579550	Unc13a	BC058348	2.57825e-010	3.66491	E15.5 * Wnt1 up vs E15.5 * Ctrl
10397482	Tmem63c	NM_172583	6.89855e-011	3.66266	E15.5 * Wnt1 up vs E15.5 * Ctrl
10503212	Chd7	NM_001081417	4.09093e-008	3.66206	E15.5 * Wnt1 up vs E15.5 * Ctrl
10365682	Anks1b	NM_001128086	2.5971e-009	3.65642	E15.5 * Wnt1 up vs E15.5 * Ctrl
10503208	Chd7	NM_001081417	1.99162e-009	3.65133	E15.5 * Wnt1 up vs E15.5 * Ctrl
10502881	St6galnac5	NM_012028	2.70737e-011	3.63701	E15.5 * Wnt1 up vs E15.5 * Ctrl
10492720	Mtap9	NM_001081230	2.63716e-008	3.63008	E15.5 * Wnt1 up vs E15.5 * Ctrl
10440216	Epha6	NM_007938	1.6107e-009	3.61169	E15.5 * Wnt1 up vs E15.5 * Ctrl
10528090	Rundc3b	NM_198620	1.50516e-009	3.58822	E15.5 * Wnt1 up vs E15.5 * Ctrl
10497051	Negr1	NM_001039094	7.36728e-009	3.57032	E15.5 * Wnt1 up vs E15.5 * Ctrl
10422164	Ednrb	NM_007904	1.38082e-012	3.56395	E15.5 * Wnt1 up vs E15.5 * Ctrl
10503206	Chd7	NM_001081417	1.35595e-009	3.54672	E15.5 * Wnt1 up vs E15.5 * Ctrl
10549594	Ttyh1	NM_021324	5.07947e-012	3.5416	E15.5 * Wnt1 up vs E15.5 * Ctrl
10357736	Nfasc	NM_182716	6.43806e-009	3.54116	E15.5 * Wnt1 up vs E15.5 * Ctrl
10458645	Ppp2r2b	NM_028392	2.45754e-012	3.52898	E15.5 * Wnt1 up vs E15.5 * Ctrl
10472984	Hoxd3	NM_010468	1.88054e-010	3.52798	E15.5 * Wnt1 up vs E15.5 * Ctrl
10546853	Srgap3	NM_080448	8.85101e-012	3.52049	E15.5 * Wnt1 up vs E15.5 * Ctrl
10503178	Chd7	NM_001081417	1.35723e-011	3.51422	E15.5 * Wnt1 up vs E15.5 * Ctrl
10563993		---	1.32721e-009	3.51327	E15.5 * Wnt1 up vs E15.5 * Ctrl
10564021		---	1.32721e-009	3.51327	E15.5 * Wnt1 up vs E15.5 * Ctrl
10564025		---	1.32721e-009	3.51327	E15.5 * Wnt1 up vs E15.5 * Ctrl
10403796	Amph	NM_175007	3.03086e-009	3.50872	E15.5 * Wnt1 up vs E15.5 * Ctrl
10428698	Sntb1	NM_016667	1.2626e-010	3.4963	E15.5 * Wnt1 up vs E15.5 * Ctrl
10406823		---	7.87327e-008	3.47952	E15.5 * Wnt1 up vs E15.5 * Ctrl
10529656	Nsg1	NM_010942	5.32997e-012	3.47637	E15.5 * Wnt1 up vs E15.5 * Ctrl
10472136	Galnt13	NM_173030	1.88165e-009	3.45516	E15.5 * Wnt1 up vs E15.5 * Ctrl
10423951		---	1.48066e-007	3.45497	E15.5 * Wnt1 up vs E15.5 * Ctrl
10503170	Chd7	NM_001081417	1.15809e-012	3.4528	E15.5 * Wnt1 up vs E15.5 * Ctrl
10372338	EG368203	NM_203660	2.2982e-006	3.4467	E15.5 * Wnt1 up vs E15.5 * Ctrl
10435767		---	0.000365451	3.43159	E15.5 * Wnt1 up vs E15.5 * Ctrl
10580247	Mast1	NM_019945	2.9395e-010	3.42986	E15.5 * Wnt1 up vs E15.5 * Ctrl
10598359	Syp	NM_009305	1.04429e-010	3.42281	E15.5 * Wnt1 up vs E15.5 * Ctrl
10439651	Cd200	NM_010818	2.51988e-009	3.41987	E15.5 * Wnt1 up vs E15.5 * Ctrl
10456363	Gnal	NM_010307	2.07723e-007	3.41251	E15.5 * Wnt1 up vs E15.5 * Ctrl

10360349	Cadm3	NM_053199	3.63167e-010	3.4054	E15.5 * Wnt1 up vs E15.5 * Ctrl
10483719	Chn1	NM_001113246	3.88902e-012	3.39433	E15.5 * Wnt1 up vs E15.5 * Ctrl
10435745	ENSMUSG0000C	AY512926	0.000168856	3.38181	E15.5 * Wnt1 up vs E15.5 * Ctrl
10564013	Snord115	AF357427	1.53352e-009	3.38141	E15.5 * Wnt1 up vs E15.5 * Ctrl
10564017	Snord115	AF357427	1.53352e-009	3.38141	E15.5 * Wnt1 up vs E15.5 * Ctrl
10553917	Apba2	NM_007461	1.98316e-008	3.37804	E15.5 * Wnt1 up vs E15.5 * Ctrl
10431017	Ttll1	NM_178869	6.63102e-012	3.36944	E15.5 * Wnt1 up vs E15.5 * Ctrl
10545041	Nap1l5	NM_021432	3.60487e-009	3.35303	E15.5 * Wnt1 up vs E15.5 * Ctrl
10490794	Pkia	NM_008862	2.29754e-009	3.35057	E15.5 * Wnt1 up vs E15.5 * Ctrl
10564089		---	6.12469e-008	3.33673	E15.5 * Wnt1 up vs E15.5 * Ctrl
10354777	Satb2	NM_139146	3.08869e-008	3.33347	E15.5 * Wnt1 up vs E15.5 * Ctrl
10407072	Elovl7	NM_029001	4.43134e-007	3.33207	E15.5 * Wnt1 up vs E15.5 * Ctrl
10564143		---	1.55159e-010	3.33156	E15.5 * Wnt1 up vs E15.5 * Ctrl
10564147		---	1.55159e-010	3.33156	E15.5 * Wnt1 up vs E15.5 * Ctrl
10346882	Adam23	NM_011780	1.71763e-009	3.32863	E15.5 * Wnt1 up vs E15.5 * Ctrl
10538482	Adcyap1r1	NM_007407	4.72517e-009	3.32272	E15.5 * Wnt1 up vs E15.5 * Ctrl
10535577	Tmem130	NM_177735	2.14329e-010	3.32246	E15.5 * Wnt1 up vs E15.5 * Ctrl
10351056	Ankrd45	BC049713	8.58572e-008	3.31999	E15.5 * Wnt1 up vs E15.5 * Ctrl
10514520	Cyp2j9	NM_028979	4.53553e-009	3.31913	E15.5 * Wnt1 up vs E15.5 * Ctrl
10546855	Srgap3	NM_080448	2.20591e-013	3.3191	E15.5 * Wnt1 up vs E15.5 * Ctrl
10420846	Fzd3	NM_021458	3.31046e-012	3.3168	E15.5 * Wnt1 up vs E15.5 * Ctrl
10492536		---	2.45932e-007	3.31319	E15.5 * Wnt1 up vs E15.5 * Ctrl
10470529	Olfm1	NM_019498	2.00999e-012	3.3082	E15.5 * Wnt1 up vs E15.5 * Ctrl
10564135		---	1.03752e-010	3.30592	E15.5 * Wnt1 up vs E15.5 * Ctrl
10499138	Dclk2	NM_027539	1.54398e-011	3.30066	E15.5 * Wnt1 up vs E15.5 * Ctrl
10564023		---	4.26188e-007	3.29648	E15.5 * Wnt1 up vs E15.5 * Ctrl
10462113	Apba1	NM_177034	1.03948e-009	3.29339	E15.5 * Wnt1 up vs E15.5 * Ctrl
10563935		---	1.20063e-008	3.28814	E15.5 * Wnt1 up vs E15.5 * Ctrl
10564045		---	1.20063e-008	3.28814	E15.5 * Wnt1 up vs E15.5 * Ctrl
10564047		---	1.20063e-008	3.28814	E15.5 * Wnt1 up vs E15.5 * Ctrl
10381574	Rundc3a	NM_016759	2.56058e-010	3.28412	E15.5 * Wnt1 up vs E15.5 * Ctrl
10503168	Chd7	NM_001081417	1.81662e-010	3.27593	E15.5 * Wnt1 up vs E15.5 * Ctrl
10514510	Cyp2j6	NM_010008	1.04028e-009	3.27524	E15.5 * Wnt1 up vs E15.5 * Ctrl
10564033		---	3.47507e-007	3.26615	E15.5 * Wnt1 up vs E15.5 * Ctrl
10451039	Slc25a27	NM_028711	6.46862e-011	3.2625	E15.5 * Wnt1 up vs E15.5 * Ctrl
10564027		---	1.34338e-007	3.25281	E15.5 * Wnt1 up vs E15.5 * Ctrl
10564077		---	1.34338e-007	3.25281	E15.5 * Wnt1 up vs E15.5 * Ctrl
10564079		---	1.34338e-007	3.25281	E15.5 * Wnt1 up vs E15.5 * Ctrl
10564085		---	1.34338e-007	3.25281	E15.5 * Wnt1 up vs E15.5 * Ctrl
10564087		---	1.34338e-007	3.25281	E15.5 * Wnt1 up vs E15.5 * Ctrl
10564091		---	1.34338e-007	3.25281	E15.5 * Wnt1 up vs E15.5 * Ctrl
10564093		---	1.34338e-007	3.25281	E15.5 * Wnt1 up vs E15.5 * Ctrl
10564095		---	1.34338e-007	3.25281	E15.5 * Wnt1 up vs E15.5 * Ctrl
10564097		---	1.34338e-007	3.25281	E15.5 * Wnt1 up vs E15.5 * Ctrl
10564099		---	1.34338e-007	3.25281	E15.5 * Wnt1 up vs E15.5 * Ctrl
10564101		---	1.34338e-007	3.25281	E15.5 * Wnt1 up vs E15.5 * Ctrl
10564103		---	1.34338e-007	3.25281	E15.5 * Wnt1 up vs E15.5 * Ctrl
10564105		---	1.34338e-007	3.25281	E15.5 * Wnt1 up vs E15.5 * Ctrl
10564107		---	1.34338e-007	3.25281	E15.5 * Wnt1 up vs E15.5 * Ctrl
10564113		---	1.34338e-007	3.25281	E15.5 * Wnt1 up vs E15.5 * Ctrl
10564115		---	1.34338e-007	3.25281	E15.5 * Wnt1 up vs E15.5 * Ctrl
10564119		---	1.34338e-007	3.25281	E15.5 * Wnt1 up vs E15.5 * Ctrl
10564121		---	1.34338e-007	3.25281	E15.5 * Wnt1 up vs E15.5 * Ctrl
10564123		---	1.34338e-007	3.25281	E15.5 * Wnt1 up vs E15.5 * Ctrl
10564125		---	1.34338e-007	3.25281	E15.5 * Wnt1 up vs E15.5 * Ctrl
10564127		---	1.34338e-007	3.25281	E15.5 * Wnt1 up vs E15.5 * Ctrl
10564129		---	1.34338e-007	3.25281	E15.5 * Wnt1 up vs E15.5 * Ctrl
10564139		---	1.34338e-007	3.25281	E15.5 * Wnt1 up vs E15.5 * Ctrl
10354141	Lonrf2	NM_001029878	1.18272e-007	3.24383	E15.5 * Wnt1 up vs E15.5 * Ctrl
10524909	Nos1	NM_008712	1.80969e-010	3.21656	E15.5 * Wnt1 up vs E15.5 * Ctrl
10523758	Lrrc8b	NM_001033550	1.25155e-009	3.21386	E15.5 * Wnt1 up vs E15.5 * Ctrl
10455259	Arhgap26	NM_175164	1.17816e-010	3.20255	E15.5 * Wnt1 up vs E15.5 * Ctrl
10431625	Syt10	NM_018803	2.08171e-008	3.20059	E15.5 * Wnt1 up vs E15.5 * Ctrl
10468722	Gfra1	NM_010279	3.95257e-010	3.19713	E15.5 * Wnt1 up vs E15.5 * Ctrl
10532630	Adrbk2	NM_177078	1.08277e-007	3.19703	E15.5 * Wnt1 up vs E15.5 * Ctrl
10543967	Dgki	NM_001081206	5.53752e-007	3.19132	E15.5 * Wnt1 up vs E15.5 * Ctrl

10368175	Pde7b	NM_013875	8.32382e-009	3.18855	E15.5 * Wnt1 up vs E15.5 * Ctrl
10428513	Csmd3	NM_001081391	5.80005e-007	3.17004	E15.5 * Wnt1 up vs E15.5 * Ctrl
10508614	Fabp3	NM_010174	1.99738e-009	3.16242	E15.5 * Wnt1 up vs E15.5 * Ctrl
10438639	Dgkg	NM_138650	3.00187e-010	3.15518	E15.5 * Wnt1 up vs E15.5 * Ctrl
10593196	ENSMUSG0000C	ENSMUST00000	8.37964e-011	3.15061	E15.5 * Wnt1 up vs E15.5 * Ctrl
10428453	Csmd3	NM_001081391	5.3979e-012	3.1477	E15.5 * Wnt1 up vs E15.5 * Ctrl
10495343	Wdr47	NM_181400	2.2728e-008	3.14701	E15.5 * Wnt1 up vs E15.5 * Ctrl
10553537	Luzp2	NM_178705	3.87832e-007	3.13961	E15.5 * Wnt1 up vs E15.5 * Ctrl
10533633	Diablo	NM_023232	1.07416e-012	3.13407	E15.5 * Wnt1 up vs E15.5 * Ctrl
10557535	Sez6l2	NM_144926	1.07152e-009	3.13317	E15.5 * Wnt1 up vs E15.5 * Ctrl
10457929	Rit2	NM_009065	6.92159e-009	3.13094	E15.5 * Wnt1 up vs E15.5 * Ctrl
10469573		---	1.26864e-005	3.12381	E15.5 * Wnt1 up vs E15.5 * Ctrl
10600180	Atp2b3	NM_177236	1.43848e-009	3.12059	E15.5 * Wnt1 up vs E15.5 * Ctrl
10473312	Fam171b	NM_175514	8.87352e-009	3.11448	E15.5 * Wnt1 up vs E15.5 * Ctrl
10593233	Htr3a	NM_013561	1.77851e-007	3.11238	E15.5 * Wnt1 up vs E15.5 * Ctrl
10452766		---	0.00251697	3.10761	E15.5 * Wnt1 up vs E15.5 * Ctrl
10600114	Pnma3	NM_153169	4.66469e-009	3.1056	E15.5 * Wnt1 up vs E15.5 * Ctrl
10408838	Elovl2	NM_019423	1.07414e-008	3.1043	E15.5 * Wnt1 up vs E15.5 * Ctrl
10503210	Chd7	NM_001081417	8.13092e-009	3.09257	E15.5 * Wnt1 up vs E15.5 * Ctrl
10504692	Tmod1	NM_021883	4.5769e-009	3.07836	E15.5 * Wnt1 up vs E15.5 * Ctrl
10472364	Scn2a1	NM_001099298	1.34816e-005	3.07516	E15.5 * Wnt1 up vs E15.5 * Ctrl
10537458	EG434008	NM_001004182	1.99081e-007	3.07372	E15.5 * Wnt1 up vs E15.5 * Ctrl
10408268	Scgn	NM_145399	3.89726e-005	3.07265	E15.5 * Wnt1 up vs E15.5 * Ctrl
10589130	Celsr3	NM_080437	7.47639e-010	3.07052	E15.5 * Wnt1 up vs E15.5 * Ctrl
10349826	ENSMUSG0000C	ENSMUST00000	3.01837e-005	3.06937	E15.5 * Wnt1 up vs E15.5 * Ctrl
10555303	Pgm2l1	NM_027629	4.33925e-008	3.06357	E15.5 * Wnt1 up vs E15.5 * Ctrl
10391084	Hap1	NM_010404	1.36845e-009	3.06275	E15.5 * Wnt1 up vs E15.5 * Ctrl
10350697	Nmnat2	NM_175460	1.17871e-008	3.0618	E15.5 * Wnt1 up vs E15.5 * Ctrl
10457963	Gpr17	NM_001025381	8.02582e-008	3.05607	E15.5 * Wnt1 up vs E15.5 * Ctrl
10504234	Unc13b	NM_001081413	1.4161e-011	3.05278	E15.5 * Wnt1 up vs E15.5 * Ctrl
10498965	Npy2r	NM_008731	1.08097e-005	3.04575	E15.5 * Wnt1 up vs E15.5 * Ctrl
10563965		---	6.30972e-010	3.04422	E15.5 * Wnt1 up vs E15.5 * Ctrl
10563967		---	6.30972e-010	3.04422	E15.5 * Wnt1 up vs E15.5 * Ctrl
10563971		---	6.30972e-010	3.04422	E15.5 * Wnt1 up vs E15.5 * Ctrl
10564043		---	6.30972e-010	3.04422	E15.5 * Wnt1 up vs E15.5 * Ctrl
10481711	Stxbp1	NM_001113569	1.18369e-009	3.03899	E15.5 * Wnt1 up vs E15.5 * Ctrl
10406229	Pcsk1	NM_013628	5.14777e-009	3.03404	E15.5 * Wnt1 up vs E15.5 * Ctrl
10376998	A530088H08Rik	EU447173	1.33934e-008	3.03086	E15.5 * Wnt1 up vs E15.5 * Ctrl
10564019		---	2.31321e-008	3.02755	E15.5 * Wnt1 up vs E15.5 * Ctrl
10406423	Mblac2	NM_028372	1.27925e-007	3.02019	E15.5 * Wnt1 up vs E15.5 * Ctrl
10433709	2900011O08Rik	NM_144518	9.13285e-010	3.01659	E15.5 * Wnt1 up vs E15.5 * Ctrl
10551421	Zfp60	ENSMUST00000	1.69901e-006	3.01391	E15.5 * Wnt1 up vs E15.5 * Ctrl
10526614	Actl6b	NM_031404	2.15649e-009	3.013	E15.5 * Wnt1 up vs E15.5 * Ctrl
10520304	Actr3b	NM_001004365	5.48972e-009	3.00873	E15.5 * Wnt1 up vs E15.5 * Ctrl
10496727	Ddah1	NM_026993	2.68257e-011	3.00496	E15.5 * Wnt1 up vs E15.5 * Ctrl
10547641	Slc2a3	NM_011401	1.299e-009	2.99977	E15.5 * Wnt1 up vs E15.5 * Ctrl
10358999	Xpr1	NM_011273	9.46418e-013	2.99894	E15.5 * Wnt1 up vs E15.5 * Ctrl
10564069		---	8.80812e-011	2.99355	E15.5 * Wnt1 up vs E15.5 * Ctrl
10564073		---	8.80812e-011	2.99355	E15.5 * Wnt1 up vs E15.5 * Ctrl
10454254	Dtna	NM_207650	2.85572e-011	2.98913	E15.5 * Wnt1 up vs E15.5 * Ctrl
10498707	Slitrk3	NM_198864	8.45849e-010	2.98653	E15.5 * Wnt1 up vs E15.5 * Ctrl
10371321	Slc41a2	NM_177388	3.03659e-008	2.98566	E15.5 * Wnt1 up vs E15.5 * Ctrl
10351998	ENSMUSG0000C	AK142791	0.00412116	2.98484	E15.5 * Wnt1 up vs E15.5 * Ctrl
10564005		---	4.42595e-006	2.97304	E15.5 * Wnt1 up vs E15.5 * Ctrl
10558481	Dpysl4	NM_011993	3.04674e-009	2.96807	E15.5 * Wnt1 up vs E15.5 * Ctrl
10356177	Dner	NM_152915	2.31914e-008	2.96181	E15.5 * Wnt1 up vs E15.5 * Ctrl
10371502	Fabp3	NM_010174	1.34288e-010	2.96154	E15.5 * Wnt1 up vs E15.5 * Ctrl
10358928	Cacna1e	NM_009782	7.59893e-010	2.95799	E15.5 * Wnt1 up vs E15.5 * Ctrl
10595392	Elovl4	NM_148941	5.57311e-007	2.95199	E15.5 * Wnt1 up vs E15.5 * Ctrl
10426397	Cntn1	NM_007727	4.76461e-009	2.94789	E15.5 * Wnt1 up vs E15.5 * Ctrl
10465943	Dagla	NM_198114	9.24761e-007	2.93444	E15.5 * Wnt1 up vs E15.5 * Ctrl
10454441	Syt4	NM_009308	1.93476e-007	2.93243	E15.5 * Wnt1 up vs E15.5 * Ctrl
10576844	BC068157	NM_207203	1.01781e-009	2.93112	E15.5 * Wnt1 up vs E15.5 * Ctrl
10385391	Cyfip2	NM_133769	2.56511e-010	2.92325	E15.5 * Wnt1 up vs E15.5 * Ctrl
10505030	Fsd1l	NM_176966	1.77665e-008	2.92203	E15.5 * Wnt1 up vs E15.5 * Ctrl
10359908	Rgs4	NM_009062	1.33877e-007	2.91818	E15.5 * Wnt1 up vs E15.5 * Ctrl

10421526	Rb1	NM_009029	1.26775e-009	2.91449	E15.5 * Wnt1 up vs E15.5 * Ctrl
10600604	Dmd	NM_007868	9.38589e-009	2.90476	E15.5 * Wnt1 up vs E15.5 * Ctrl
10348493	Lrrfip1	NM_008515	2.09289e-012	2.90021	E15.5 * Wnt1 up vs E15.5 * Ctrl
10443383	Tmhs	NM_026571	1.5179e-007	2.89822	E15.5 * Wnt1 up vs E15.5 * Ctrl
10578810	Clcn3	NM_173874	1.3513e-011	2.89796	E15.5 * Wnt1 up vs E15.5 * Ctrl
10561335	Prkcz	NM_008860	5.70253e-007	2.89729	E15.5 * Wnt1 up vs E15.5 * Ctrl
10413419	Arhgef3	NM_027871	2.03434e-009	2.88328	E15.5 * Wnt1 up vs E15.5 * Ctrl
10403604	Lyst	NM_010748	5.67099e-011	2.8827	E15.5 * Wnt1 up vs E15.5 * Ctrl
10490569	Kcnq2	NM_010611	5.83163e-011	2.87744	E15.5 * Wnt1 up vs E15.5 * Ctrl
10432362	Rhebl1	NM_026967	1.52086e-007	2.87716	E15.5 * Wnt1 up vs E15.5 * Ctrl
10486875	Frmd5	NM_172673	1.10917e-009	2.86184	E15.5 * Wnt1 up vs E15.5 * Ctrl
10416340	Gfra2	NM_008115	1.97838e-009	2.86114	E15.5 * Wnt1 up vs E15.5 * Ctrl
10442887	Fbxl16	BC132383	8.17344e-008	2.85995	E15.5 * Wnt1 up vs E15.5 * Ctrl
10531304	Cdkl2	NM_016912	5.8612e-010	2.85931	E15.5 * Wnt1 up vs E15.5 * Ctrl
10433403	A2bp1	NM_021477	1.68889e-009	2.85841	E15.5 * Wnt1 up vs E15.5 * Ctrl
10357300	Dpp10	NM_199021	2.35835e-013	2.85744	E15.5 * Wnt1 up vs E15.5 * Ctrl
10379153	Aldoc	NM_009657	6.348e-009	2.85578	E15.5 * Wnt1 up vs E15.5 * Ctrl
10599530	Rab33a	NM_011228	7.67855e-008	2.85339	E15.5 * Wnt1 up vs E15.5 * Ctrl
10563955	100040985	XM_001475615	2.53073e-006	2.84081	E15.5 * Wnt1 up vs E15.5 * Ctrl
10384233	Tns3	NM_001083587	9.93242e-010	2.84027	E15.5 * Wnt1 up vs E15.5 * Ctrl
10479230	Phactr3	NM_028806	2.91071e-008	2.83758	E15.5 * Wnt1 up vs E15.5 * Ctrl
10367775	Stxbp5	NM_001081344	6.7611e-010	2.83221	E15.5 * Wnt1 up vs E15.5 * Ctrl
10483679	Gpr155	NM_001080707	2.32806e-009	2.82935	E15.5 * Wnt1 up vs E15.5 * Ctrl
10353117	Slco5a1	NM_172841	1.00629e-009	2.82875	E15.5 * Wnt1 up vs E15.5 * Ctrl
10393379	Mxra7	NM_026280	1.20621e-009	2.80625	E15.5 * Wnt1 up vs E15.5 * Ctrl
10539669	Add2	NM_013458	2.99556e-012	2.80497	E15.5 * Wnt1 up vs E15.5 * Ctrl
10570180	EG434280	ENSMUST00000(	1.04848e-009	2.8038	E15.5 * Wnt1 up vs E15.5 * Ctrl
10491780	Hspa4l	NM_011020	4.73075e-008	2.80299	E15.5 * Wnt1 up vs E15.5 * Ctrl
10366391	Kcnc2	NM_001025581	1.33084e-008	2.80116	E15.5 * Wnt1 up vs E15.5 * Ctrl
10580382	Neto2	NM_001081324	2.5122e-006	2.79876	E15.5 * Wnt1 up vs E15.5 * Ctrl
10353731	4930444G20Rik	NM_053264	5.39829e-006	2.7985	E15.5 * Wnt1 up vs E15.5 * Ctrl
10488709	8430427H17Rik	NM_001134300	9.44364e-010	2.7931	E15.5 * Wnt1 up vs E15.5 * Ctrl
10603843	Syn1	NM_013680	5.21046e-010	2.78487	E15.5 * Wnt1 up vs E15.5 * Ctrl
10587299	Ick	NM_019987	2.16386e-011	2.78425	E15.5 * Wnt1 up vs E15.5 * Ctrl
10587315	Gsta4	NM_010357	4.62411e-010	2.78327	E15.5 * Wnt1 up vs E15.5 * Ctrl
10503161	Chd7	NM_001081417	3.02746e-011	2.7808	E15.5 * Wnt1 up vs E15.5 * Ctrl
10467730	Morn4	NM_198108	3.16389e-006	2.77956	E15.5 * Wnt1 up vs E15.5 * Ctrl
10505705	Sh3gl2	NM_019535	4.08243e-010	2.77867	E15.5 * Wnt1 up vs E15.5 * Ctrl
10476633	Pcsk2	NM_008792	6.80301e-009	2.77659	E15.5 * Wnt1 up vs E15.5 * Ctrl
10576934	Fam155a	NM_173446	2.03561e-009	2.7728	E15.5 * Wnt1 up vs E15.5 * Ctrl
10561927	Aplp1	NM_007467	3.88703e-007	2.76534	E15.5 * Wnt1 up vs E15.5 * Ctrl
10527732	Fry	ENSMUST00000(	5.21633e-011	2.76385	E15.5 * Wnt1 up vs E15.5 * Ctrl
10436487	Vgll3	AK007165	1.56284e-007	2.76253	E15.5 * Wnt1 up vs E15.5 * Ctrl
10498720	Zbbx	NM_172515	1.56985e-010	2.76148	E15.5 * Wnt1 up vs E15.5 * Ctrl
10347734	Sgpp2	NM_001004173	2.20993e-010	2.75035	E15.5 * Wnt1 up vs E15.5 * Ctrl
10440621	Grik1	NM_146072	1.11544e-008	2.74621	E15.5 * Wnt1 up vs E15.5 * Ctrl
10399308	Fkbp1b	NM_016863	7.12473e-006	2.73458	E15.5 * Wnt1 up vs E15.5 * Ctrl
10351971	Fmn2	NM_019445	1.76127e-009	2.73246	E15.5 * Wnt1 up vs E15.5 * Ctrl
10576942		---	0.00133324	2.72629	E15.5 * Wnt1 up vs E15.5 * Ctrl
10353729	4930444G20Rik	NM_053264	1.47914e-005	2.72506	E15.5 * Wnt1 up vs E15.5 * Ctrl
10345350		---	5.4972e-010	2.72205	E15.5 * Wnt1 up vs E15.5 * Ctrl
10433953	Ypel1	NM_023249	3.11553e-010	2.72148	E15.5 * Wnt1 up vs E15.5 * Ctrl
10347188	A830006F12Rik	NM_177164	1.08137e-007	2.7186	E15.5 * Wnt1 up vs E15.5 * Ctrl
10555118	Pak1	NM_011035	9.1141e-011	2.71727	E15.5 * Wnt1 up vs E15.5 * Ctrl
10519951	Pion	NM_175437	4.02792e-008	2.71283	E15.5 * Wnt1 up vs E15.5 * Ctrl
10395457	Etv1	NM_007960	7.64208e-009	2.70644	E15.5 * Wnt1 up vs E15.5 * Ctrl
10505568	Frmd3	NM_172869	3.57177e-008	2.7031	E15.5 * Wnt1 up vs E15.5 * Ctrl
10393594	D11Bwg0517e	NM_001039167	2.82929e-008	2.6947	E15.5 * Wnt1 up vs E15.5 * Ctrl
10375322	4933415A04Rik	ENSMUST00000(	2.27938e-009	2.69215	E15.5 * Wnt1 up vs E15.5 * Ctrl
10445325	Rcan2	NM_030598	6.43936e-007	2.6879	E15.5 * Wnt1 up vs E15.5 * Ctrl
10416887	Slain1	NM_198014	1.46496e-007	2.67741	E15.5 * Wnt1 up vs E15.5 * Ctrl
10371092	Atcay	NM_178662	2.76295e-007	2.66844	E15.5 * Wnt1 up vs E15.5 * Ctrl
10519855	Cacna2d1	NM_001110843	1.7666e-007	2.66478	E15.5 * Wnt1 up vs E15.5 * Ctrl
10352554		---	5.20318e-008	2.66175	E15.5 * Wnt1 up vs E15.5 * Ctrl
10598493	Pcsk1n	NM_013892	4.58823e-010	2.66016	E15.5 * Wnt1 up vs E15.5 * Ctrl
10362003	F730021E23Rik	AK155606	8.23186e-005	2.65865	E15.5 * Wnt1 up vs E15.5 * Ctrl

10366229	Lin7a	NM_001039354	3.63908e-008	2.65164	E15.5 * Wnt1 up vs E15.5 * Ctrl
10415132	Cmtm5	NM_026066	3.88332e-009	2.64949	E15.5 * Wnt1 up vs E15.5 * Ctrl
10361191	Syt14	NM_181546	5.24757e-010	2.64429	E15.5 * Wnt1 up vs E15.5 * Ctrl
10564137		---	1.09882e-006	2.63886	E15.5 * Wnt1 up vs E15.5 * Ctrl
10352092	Zfp238	NM_001012330	8.10568e-010	2.63503	E15.5 * Wnt1 up vs E15.5 * Ctrl
10507379	Zswim5	NM_001029912	1.67781e-009	2.63416	E15.5 * Wnt1 up vs E15.5 * Ctrl
10363786	Ank3	NM_146005	2.99457e-009	2.63407	E15.5 * Wnt1 up vs E15.5 * Ctrl
10400057	Arl4a	NM_001039515	5.79876e-011	2.63262	E15.5 * Wnt1 up vs E15.5 * Ctrl
10499093		---	1.3329e-007	2.62962	E15.5 * Wnt1 up vs E15.5 * Ctrl
10510286	Mad2l2	NM_027985	7.81812e-010	2.62559	E15.5 * Wnt1 up vs E15.5 * Ctrl
10513869	Megf9	NM_172694	1.1383e-012	2.6253	E15.5 * Wnt1 up vs E15.5 * Ctrl
10503216	Chd7	NM_001081417	2.249e-009	2.62362	E15.5 * Wnt1 up vs E15.5 * Ctrl
10570178	A230072I06Rik	ENSMUST00000(	3.8589e-006	2.62323	E15.5 * Wnt1 up vs E15.5 * Ctrl
10401359	Dpf3	NM_058212	6.19634e-009	2.62138	E15.5 * Wnt1 up vs E15.5 * Ctrl
10520318	Dpp6	NM_010075	6.70115e-012	2.61713	E15.5 * Wnt1 up vs E15.5 * Ctrl
10347073	Unc80	FJ210934	5.50083e-010	2.61322	E15.5 * Wnt1 up vs E15.5 * Ctrl
10420532	Atp8a2	NM_015803	1.02645e-010	2.61289	E15.5 * Wnt1 up vs E15.5 * Ctrl
10563931		---	1.45693e-006	2.60486	E15.5 * Wnt1 up vs E15.5 * Ctrl
10606069	4930444G20Rik	NM_053264	2.26026e-005	2.59776	E15.5 * Wnt1 up vs E15.5 * Ctrl
10452734	Alk	NM_007439	1.75745e-009	2.59203	E15.5 * Wnt1 up vs E15.5 * Ctrl
10554588	Sh3gl3	NM_017400	3.4778e-007	2.59174	E15.5 * Wnt1 up vs E15.5 * Ctrl
10408798	Tcfap2a	NM_011547	2.95221e-008	2.58929	E15.5 * Wnt1 up vs E15.5 * Ctrl
10392910	C630004H02Rik	BC024617	9.63819e-006	2.58514	E15.5 * Wnt1 up vs E15.5 * Ctrl
10429083	Kcnq3	NM_152923	1.48909e-011	2.58123	E15.5 * Wnt1 up vs E15.5 * Ctrl
10459835		---	1.56182e-007	2.58087	E15.5 * Wnt1 up vs E15.5 * Ctrl
10549536	Amn1	NM_001113424	2.88449e-009	2.57251	E15.5 * Wnt1 up vs E15.5 * Ctrl
10368981	Lin28b	NM_001031772	6.98215e-009	2.57163	E15.5 * Wnt1 up vs E15.5 * Ctrl
10496854	Ttll7	NM_027594	3.43955e-008	2.5705	E15.5 * Wnt1 up vs E15.5 * Ctrl
10355024	Ica1l	NM_027407	3.09437e-009	2.56771	E15.5 * Wnt1 up vs E15.5 * Ctrl
10510725	Chd5	NM_001081376	1.8583e-010	2.56074	E15.5 * Wnt1 up vs E15.5 * Ctrl
10569646	Ccnd1	NM_007631	6.36632e-010	2.55987	E15.5 * Wnt1 up vs E15.5 * Ctrl
10519815	Cacna2d1	NM_001110843	1.68208e-008	2.55721	E15.5 * Wnt1 up vs E15.5 * Ctrl
10527799	ENSMUSG0000C	AK041221	8.63194e-005	2.55659	E15.5 * Wnt1 up vs E15.5 * Ctrl
10594301	Coro2b	NM_175484	6.19134e-008	2.55469	E15.5 * Wnt1 up vs E15.5 * Ctrl
10408850	Nedd9	NM_001111324	1.16381e-010	2.55432	E15.5 * Wnt1 up vs E15.5 * Ctrl
10540880	Syn2	NM_001111015	2.49745e-009	2.55241	E15.5 * Wnt1 up vs E15.5 * Ctrl
10492165		---	3.41144e-005	2.55213	E15.5 * Wnt1 up vs E15.5 * Ctrl
10392056	Cyb561	NM_007805	2.35116e-007	2.55172	E15.5 * Wnt1 up vs E15.5 * Ctrl
10376747	Kcnj12	NM_010603	2.79943e-008	2.55126	E15.5 * Wnt1 up vs E15.5 * Ctrl
10375313	Ccnjl	NM_001045530	1.60577e-008	2.55077	E15.5 * Wnt1 up vs E15.5 * Ctrl
10575616	Gabarapl2	AK134873	7.53149e-005	2.54223	E15.5 * Wnt1 up vs E15.5 * Ctrl
10569278	5530400B01Rik	ENSMUST00000(	3.61245e-006	2.53886	E15.5 * Wnt1 up vs E15.5 * Ctrl
10495613	4833424O15Rik	NM_029425	2.04123e-007	2.53675	E15.5 * Wnt1 up vs E15.5 * Ctrl
10392369	Cacng4	NM_019431	2.85918e-009	2.5361	E15.5 * Wnt1 up vs E15.5 * Ctrl
10570513	2900016B01Rik	ENSMUST00000(	6.82607e-008	2.53592	E15.5 * Wnt1 up vs E15.5 * Ctrl
10462922	Plce1	NM_019588	4.77911e-012	2.53573	E15.5 * Wnt1 up vs E15.5 * Ctrl
10406934	Etv1	NM_007960	1.42245e-009	2.53102	E15.5 * Wnt1 up vs E15.5 * Ctrl
10496837	Ttll7	NM_027594	1.21052e-006	2.53084	E15.5 * Wnt1 up vs E15.5 * Ctrl
10444135	AA388235	NM_001013793	9.4989e-005	2.52846	E15.5 * Wnt1 up vs E15.5 * Ctrl
10409737	Agtpbp1	NM_023328	8.6117e-011	2.52752	E15.5 * Wnt1 up vs E15.5 * Ctrl
10559146	Brsk2	NM_029426	5.26002e-009	2.52667	E15.5 * Wnt1 up vs E15.5 * Ctrl
10420988	Dpysl2	NM_009955	4.82962e-007	2.52154	E15.5 * Wnt1 up vs E15.5 * Ctrl
10446553	Epb4.1l3	NM_013813	6.90288e-012	2.52147	E15.5 * Wnt1 up vs E15.5 * Ctrl
10476594	Macrod2	NM_001013802	0.000806975	2.51961	E15.5 * Wnt1 up vs E15.5 * Ctrl
10355214	Idh1	NM_010497	1.99261e-011	2.51798	E15.5 * Wnt1 up vs E15.5 * Ctrl
10600814	Gspt2	NM_008179	7.23471e-005	2.51598	E15.5 * Wnt1 up vs E15.5 * Ctrl
10550638	Rtn2	NM_013648	2.56481e-011	2.51351	E15.5 * Wnt1 up vs E15.5 * Ctrl
10562532	Zfp536	NM_172385	3.18803e-008	2.5072	E15.5 * Wnt1 up vs E15.5 * Ctrl
10515111		---	0.00653382	2.50596	E15.5 * Wnt1 up vs E15.5 * Ctrl
10472366	Scn2a1	NM_001099298	1.4321e-005	2.50548	E15.5 * Wnt1 up vs E15.5 * Ctrl
10568298	Stx1b	NM_024414	2.72252e-008	2.50005	E15.5 * Wnt1 up vs E15.5 * Ctrl
10425852	Parvb	NM_133167	1.06869e-006	2.49894	E15.5 * Wnt1 up vs E15.5 * Ctrl
10392943	Hn1	NM_008258	6.26783e-009	2.49581	E15.5 * Wnt1 up vs E15.5 * Ctrl
10573348	Cacna1a	NM_007578	7.10042e-010	2.49439	E15.5 * Wnt1 up vs E15.5 * Ctrl
10522655	C530008M17Rik	ENSMUST00000	0.000117844	2.49387	E15.5 * Wnt1 up vs E15.5 * Ctrl
10503190	Chd7	NM_001081417	4.27601e-008	2.49348	E15.5 * Wnt1 up vs E15.5 * Ctrl

10439016	4930444G20Rik	NM_053264	4.19418e-005	2.49283	E15.5 * Wnt1 up vs E15.5 * Ctrl
10355984	Serpine2	NM_009255	3.26834e-012	2.4896	E15.5 * Wnt1 up vs E15.5 * Ctrl
10350742	Rnasel	NM_011882	7.48878e-006	2.48234	E15.5 * Wnt1 up vs E15.5 * Ctrl
10527233	Cyth3	NM_011182	4.82006e-011	2.47168	E15.5 * Wnt1 up vs E15.5 * Ctrl
10491846		---	0.000470788	2.47161	E15.5 * Wnt1 up vs E15.5 * Ctrl
10460123	9330132A10Rik	BC098197	3.03737e-007	2.47086	E15.5 * Wnt1 up vs E15.5 * Ctrl
10481845	Fam125b	BC059907	6.671e-009	2.4696	E15.5 * Wnt1 up vs E15.5 * Ctrl
10348999	Cdh20	NM_011800	3.2342e-007	2.46398	E15.5 * Wnt1 up vs E15.5 * Ctrl
10559580	Syt5	NM_016908	1.76891e-008	2.46067	E15.5 * Wnt1 up vs E15.5 * Ctrl
10490602	Eef1a2	NM_007906	7.34768e-010	2.45728	E15.5 * Wnt1 up vs E15.5 * Ctrl
10381154	Cnp	NM_009923	1.43997e-008	2.4546	E15.5 * Wnt1 up vs E15.5 * Ctrl
10524889	Ksr2	NM_001114545	3.46156e-009	2.44661	E15.5 * Wnt1 up vs E15.5 * Ctrl
10499643	Chrnb2	NM_009602	2.75612e-008	2.44595	E15.5 * Wnt1 up vs E15.5 * Ctrl
10428511	Csmd3	NM_001081391	1.22155e-006	2.44226	E15.5 * Wnt1 up vs E15.5 * Ctrl
10607562	Cnksr2	NM_177751	1.34139e-008	2.44003	E15.5 * Wnt1 up vs E15.5 * Ctrl
10601152	Nlgn3	NM_172932	1.65643e-009	2.4393	E15.5 * Wnt1 up vs E15.5 * Ctrl
10451213	Rsph9	NM_029338	2.21044e-007	2.43666	E15.5 * Wnt1 up vs E15.5 * Ctrl
10600390	Gdi1	NM_010273	1.32119e-009	2.43636	E15.5 * Wnt1 up vs E15.5 * Ctrl
10424781	Grina	NM_023168	1.86607e-008	2.42573	E15.5 * Wnt1 up vs E15.5 * Ctrl
10585905	Parp6	NM_029922	3.35644e-010	2.42552	E15.5 * Wnt1 up vs E15.5 * Ctrl
10445944	St6gal2	NM_172829	2.72775e-006	2.42224	E15.5 * Wnt1 up vs E15.5 * Ctrl
10526191	Gatsl2	BC026208	8.5957e-008	2.41589	E15.5 * Wnt1 up vs E15.5 * Ctrl
10555570	Phox2a	NM_008887	3.29655e-007	2.41533	E15.5 * Wnt1 up vs E15.5 * Ctrl
10556178	Tub	NM_021885	6.54489e-009	2.41257	E15.5 * Wnt1 up vs E15.5 * Ctrl
10435748	D930030D11Rik	ENSMUST00000(	9.97657e-005	2.41126	E15.5 * Wnt1 up vs E15.5 * Ctrl
10372342	Nav3	NM_001081035	1.57565e-009	2.40988	E15.5 * Wnt1 up vs E15.5 * Ctrl
10404792	Phactr1	NM_198419	5.75388e-009	2.40956	E15.5 * Wnt1 up vs E15.5 * Ctrl
10466374	Tle4	NM_011600	1.07034e-011	2.40487	E15.5 * Wnt1 up vs E15.5 * Ctrl
10544768	Hoxa5	NM_010453	1.41515e-005	2.40459	E15.5 * Wnt1 up vs E15.5 * Ctrl
10468762	4930506M07Rik	NM_001114312	1.04835e-007	2.40452	E15.5 * Wnt1 up vs E15.5 * Ctrl
10464283	ENSMUSG0000C	ENSMUST00000(	1.07541e-008	2.40327	E15.5 * Wnt1 up vs E15.5 * Ctrl
10395005	Kidins220	NM_001081378	6.57934e-012	2.40009	E15.5 * Wnt1 up vs E15.5 * Ctrl
10470412	Dbh	NM_138942	9.9693e-008	2.39873	E15.5 * Wnt1 up vs E15.5 * Ctrl
10530013	Gm447	BC025881	2.11151e-005	2.39381	E15.5 * Wnt1 up vs E15.5 * Ctrl
10496975	Slc44a5	NM_001081263	6.55462e-006	2.39347	E15.5 * Wnt1 up vs E15.5 * Ctrl
10449920	Zfp811	NM_183177	1.0532e-006	2.39158	E15.5 * Wnt1 up vs E15.5 * Ctrl
10444756	Atp6v1g2	NM_023179	3.35248e-007	2.39057	E15.5 * Wnt1 up vs E15.5 * Ctrl
10565910	Plekhb1	NM_013746	4.18563e-007	2.39042	E15.5 * Wnt1 up vs E15.5 * Ctrl
10356305	Htr2b	NM_008311	1.86708e-007	2.38919	E15.5 * Wnt1 up vs E15.5 * Ctrl
10579554	Unc13a	NM_001029873	7.51316e-009	2.38842	E15.5 * Wnt1 up vs E15.5 * Ctrl
10569163	Cend1	NM_021316	1.78655e-008	2.38684	E15.5 * Wnt1 up vs E15.5 * Ctrl
10472408	Csrnp3	NM_153409	1.01355e-007	2.376	E15.5 * Wnt1 up vs E15.5 * Ctrl
10355813	Ptprn	NM_008985	4.35982e-009	2.37518	E15.5 * Wnt1 up vs E15.5 * Ctrl
10503220	Chd7	NM_001081417	1.44999e-006	2.37375	E15.5 * Wnt1 up vs E15.5 * Ctrl
10360479	Cep170	NM_001099637	1.0616e-010	2.37144	E15.5 * Wnt1 up vs E15.5 * Ctrl
10607302	Gnl3l	NM_198110	1.47209e-011	2.36831	E15.5 * Wnt1 up vs E15.5 * Ctrl
10430458	Sox10	NM_011437	2.37138e-006	2.36489	E15.5 * Wnt1 up vs E15.5 * Ctrl
10566457	Apbb1	NM_009685	2.37246e-008	2.3643	E15.5 * Wnt1 up vs E15.5 * Ctrl
10498210	Nbea	NM_030595	4.08071e-012	2.36414	E15.5 * Wnt1 up vs E15.5 * Ctrl
10451763	Satb1	NM_009122	2.68247e-012	2.36409	E15.5 * Wnt1 up vs E15.5 * Ctrl
10429325		---	0.00013198	2.3564	E15.5 * Wnt1 up vs E15.5 * Ctrl
10461057	Rcor2	NM_054048	1.84764e-006	2.35567	E15.5 * Wnt1 up vs E15.5 * Ctrl
10497329		---	4.29011e-005	2.35154	E15.5 * Wnt1 up vs E15.5 * Ctrl
10494372	Sv2a	NM_022030	2.50215e-005	2.34912	E15.5 * Wnt1 up vs E15.5 * Ctrl
10551104	Tmem145	NM_183311	2.27343e-008	2.34911	E15.5 * Wnt1 up vs E15.5 * Ctrl
10498309	Pfn2	NM_019410	3.73529e-008	2.34874	E15.5 * Wnt1 up vs E15.5 * Ctrl
10600765	Pcyt1b	NM_211138	4.07252e-010	2.34358	E15.5 * Wnt1 up vs E15.5 * Ctrl
10448081	Rgmb	NM_178615	2.5456e-012	2.34195	E15.5 * Wnt1 up vs E15.5 * Ctrl
10531866	Mapk10	NM_009158	4.31735e-008	2.34109	E15.5 * Wnt1 up vs E15.5 * Ctrl
10435769	Zbtb20	NM_019778	1.91113e-009	2.34078	E15.5 * Wnt1 up vs E15.5 * Ctrl
10350113	Arl8a	NM_026823	9.02222e-008	2.33756	E15.5 * Wnt1 up vs E15.5 * Ctrl
10423230	Cdh9	NM_009869	3.2154e-008	2.33663	E15.5 * Wnt1 up vs E15.5 * Ctrl
10540333	Cntn6	NM_017383	9.80919e-007	2.33489	E15.5 * Wnt1 up vs E15.5 * Ctrl
10587107	Myo5a	NM_010864	2.23643e-012	2.33366	E15.5 * Wnt1 up vs E15.5 * Ctrl
10494007	Them4	NM_029431	4.29598e-007	2.33142	E15.5 * Wnt1 up vs E15.5 * Ctrl
10491960		---	0.00734362	2.3314	E15.5 * Wnt1 up vs E15.5 * Ctrl

10434758	St6gal1	NM_145933	2.75214e-010	2.32989	E15.5 * Wnt1 up vs E15.5 * Ctrl
10458534	Pcdh1	NM_029357	4.75491e-007	2.32827	E15.5 * Wnt1 up vs E15.5 * Ctrl
10554667	Tmc3	NM_177695	1.10485e-010	2.324	E15.5 * Wnt1 up vs E15.5 * Ctrl
10363455	Pcbd1	NM_025273	3.16004e-007	2.32316	E15.5 * Wnt1 up vs E15.5 * Ctrl
10447502	Adcyap1	NM_009625	1.81178e-006	2.32271	E15.5 * Wnt1 up vs E15.5 * Ctrl
10395414	Tmem195	NM_178767	2.13136e-005	2.32154	E15.5 * Wnt1 up vs E15.5 * Ctrl
10539169	Lrrtm1	NM_028880	1.91102e-007	2.31991	E15.5 * Wnt1 up vs E15.5 * Ctrl
10552075	Lgi4	NM_144556	1.05305e-007	2.31887	E15.5 * Wnt1 up vs E15.5 * Ctrl
10358457	Bex4	NM_212457	1.03857e-008	2.31831	E15.5 * Wnt1 up vs E15.5 * Ctrl
10606868	Bex1	NM_009052	4.13111e-008	2.31794	E15.5 * Wnt1 up vs E15.5 * Ctrl
10601569	Pcdh11x	NM_001081385	0.00102581	2.31772	E15.5 * Wnt1 up vs E15.5 * Ctrl
10362513	Hs3st5	NM_001081208	9.87488e-008	2.31748	E15.5 * Wnt1 up vs E15.5 * Ctrl
10568735	Ebf3	NM_001113415	6.63865e-009	2.31578	E15.5 * Wnt1 up vs E15.5 * Ctrl
10492174	Tm4sf4	NM_145539	7.74061e-005	2.31556	E15.5 * Wnt1 up vs E15.5 * Ctrl
10370072	Prmt2	NM_133182	4.61868e-007	2.30986	E15.5 * Wnt1 up vs E15.5 * Ctrl
10401987	Kcnk10	NM_029911	4.80942e-007	2.30949	E15.5 * Wnt1 up vs E15.5 * Ctrl
10585823	LOC665268	XM_001479935	3.60529e-005	2.30797	E15.5 * Wnt1 up vs E15.5 * Ctrl
10589368	Plxnb1	NM_172775	2.26179e-006	2.30379	E15.5 * Wnt1 up vs E15.5 * Ctrl
10375002	Cpeb4	NM_026252	4.96933e-012	2.30098	E15.5 * Wnt1 up vs E15.5 * Ctrl
10592593	Tecta	NM_009347	7.7405e-008	2.30059	E15.5 * Wnt1 up vs E15.5 * Ctrl
10420023	Fam158a	AB054001	7.34702e-006	2.30015	E15.5 * Wnt1 up vs E15.5 * Ctrl
10550402	Pnmal1	NM_001007569	3.40993e-006	2.29562	E15.5 * Wnt1 up vs E15.5 * Ctrl
10345172	ENSMUSG0000C	ENSMUST00000(	1.42025e-005	2.29169	E15.5 * Wnt1 up vs E15.5 * Ctrl
10472382	Scn2a1	NM_001099298	2.92147e-006	2.2879	E15.5 * Wnt1 up vs E15.5 * Ctrl
10564203		---	0.00013838	2.28775	E15.5 * Wnt1 up vs E15.5 * Ctrl
10533401	Cux2	ENSMUST00000	2.7957e-007	2.28721	E15.5 * Wnt1 up vs E15.5 * Ctrl
10400302		---	0.00075114	2.28576	E15.5 * Wnt1 up vs E15.5 * Ctrl
10419966	Zfhx2	NM_001039198	2.84535e-007	2.28297	E15.5 * Wnt1 up vs E15.5 * Ctrl
10469020	Bend7	NM_178663	1.72063e-010	2.28063	E15.5 * Wnt1 up vs E15.5 * Ctrl
10422240	Slitrk1	NM_199065	4.34235e-006	2.27891	E15.5 * Wnt1 up vs E15.5 * Ctrl
10402604		---	3.78357e-006	2.27816	E15.5 * Wnt1 up vs E15.5 * Ctrl
10355474	March4	NM_001045533	7.51155e-008	2.27386	E15.5 * Wnt1 up vs E15.5 * Ctrl
10507032	Bend5	NM_026279	4.4108e-009	2.27325	E15.5 * Wnt1 up vs E15.5 * Ctrl
10507273	Pik3r3	NM_181585	5.54242e-012	2.27172	E15.5 * Wnt1 up vs E15.5 * Ctrl
10602428	Wnk3	ENSMUST00000(	4.35337e-007	2.27111	E15.5 * Wnt1 up vs E15.5 * Ctrl
10433075	5730585A16Rik	ENSMUST00000(	0.000124042	2.26931	E15.5 * Wnt1 up vs E15.5 * Ctrl
10501199	Gstm7	NM_026672	6.45893e-007	2.26887	E15.5 * Wnt1 up vs E15.5 * Ctrl
10416541	Enox1	NM_172813	2.81416e-010	2.26635	E15.5 * Wnt1 up vs E15.5 * Ctrl
10597323	Arpp21	NM_033264	3.62389e-010	2.26235	E15.5 * Wnt1 up vs E15.5 * Ctrl
10384691	0610010F05Rik	NM_027860	2.28415e-009	2.26129	E15.5 * Wnt1 up vs E15.5 * Ctrl
10538247	Npy	NM_023456	2.33879e-006	2.26104	E15.5 * Wnt1 up vs E15.5 * Ctrl
10446596	Dlgap1	NM_177639	5.56527e-008	2.2578	E15.5 * Wnt1 up vs E15.5 * Ctrl
10581571	4922502B01Rik	ENSMUST00000(	2.40115e-007	2.25573	E15.5 * Wnt1 up vs E15.5 * Ctrl
10455084	Pcdhb10	NM_053135	3.54512e-005	2.25321	E15.5 * Wnt1 up vs E15.5 * Ctrl
10596051	Tmem22	NM_001101483	2.48294e-007	2.24975	E15.5 * Wnt1 up vs E15.5 * Ctrl
10536611	Kcnd2	NM_019697	4.05917e-007	2.24957	E15.5 * Wnt1 up vs E15.5 * Ctrl
10603814	Slc9a7	NM_177353	2.53922e-007	2.24842	E15.5 * Wnt1 up vs E15.5 * Ctrl
10400866	Trim9	NM_053167	1.15666e-007	2.24833	E15.5 * Wnt1 up vs E15.5 * Ctrl
10581266	Tppp3	NM_026481	3.80298e-008	2.24291	E15.5 * Wnt1 up vs E15.5 * Ctrl
10415980	Fbxo16	NM_015795	9.31281e-008	2.24196	E15.5 * Wnt1 up vs E15.5 * Ctrl
10605820	Zc4h2	NM_001003916	3.83048e-009	2.2395	E15.5 * Wnt1 up vs E15.5 * Ctrl
10454286	Mapre2	NM_153058	3.33475e-011	2.23764	E15.5 * Wnt1 up vs E15.5 * Ctrl
10581479	Smpd3	NM_021491	8.1665e-007	2.23448	E15.5 * Wnt1 up vs E15.5 * Ctrl
10547613	Rimklb	NM_027664	7.7487e-008	2.23153	E15.5 * Wnt1 up vs E15.5 * Ctrl
10497012	BC007180	BC138236	1.53994e-009	2.22718	E15.5 * Wnt1 up vs E15.5 * Ctrl
10566714	Ric3	NM_001038624	5.07769e-008	2.22666	E15.5 * Wnt1 up vs E15.5 * Ctrl
10564631	Slco3a1	NM_023908	5.44488e-008	2.22303	E15.5 * Wnt1 up vs E15.5 * Ctrl
10356020	Dock10	NM_175291	4.89807e-007	2.22189	E15.5 * Wnt1 up vs E15.5 * Ctrl
10576495	Trim67	BC094596	4.5926e-009	2.21935	E15.5 * Wnt1 up vs E15.5 * Ctrl
10539211	Lrrtm4	NR_027323	1.42356e-008	2.21364	E15.5 * Wnt1 up vs E15.5 * Ctrl
10482177	Strbp	NM_009261	7.18882e-007	2.21216	E15.5 * Wnt1 up vs E15.5 * Ctrl
10483131	Kcnh7	NM_133207	7.00514e-008	2.21117	E15.5 * Wnt1 up vs E15.5 * Ctrl
10593927	Scamp5	NM_020270	4.08352e-007	2.20641	E15.5 * Wnt1 up vs E15.5 * Ctrl
10559825	2810409K11Rik	BC117497	0.000137751	2.20602	E15.5 * Wnt1 up vs E15.5 * Ctrl
10392388	Prkca	NM_011101	4.95867e-009	2.20295	E15.5 * Wnt1 up vs E15.5 * Ctrl
10423333	Fam134b	NM_001034851	1.67807e-008	2.19974	E15.5 * Wnt1 up vs E15.5 * Ctrl

10592140	Ddx25	NM_013932	4.02725e-007	2.19914	E15.5 * Wnt1 up vs E15.5 * Ctrl
10555568	Rpl31	BC092139	3.19176e-006	2.19515	E15.5 * Wnt1 up vs E15.5 * Ctrl
10602688	Usp51	NM_001137547	0.001217	2.19449	E15.5 * Wnt1 up vs E15.5 * Ctrl
10527148	Slc29a4	NM_146257	1.02631e-009	2.19174	E15.5 * Wnt1 up vs E15.5 * Ctrl
10364149	S100b	NM_009115	7.10507e-006	2.19147	E15.5 * Wnt1 up vs E15.5 * Ctrl
10465395	Ppp2r5b	NM_198168	5.24404e-005	2.1914	E15.5 * Wnt1 up vs E15.5 * Ctrl
10404702	Gcnt2	NM_023887	9.86475e-009	2.18979	E15.5 * Wnt1 up vs E15.5 * Ctrl
10574350	Mmp15	NM_008609	4.57691e-007	2.18687	E15.5 * Wnt1 up vs E15.5 * Ctrl
10503204	Chd7	NM_001081417	1.99624e-007	2.18497	E15.5 * Wnt1 up vs E15.5 * Ctrl
10425158	Pdxp	NM_020271	5.63777e-007	2.18448	E15.5 * Wnt1 up vs E15.5 * Ctrl
10368556	Hey2	NM_013904	1.05412e-006	2.18074	E15.5 * Wnt1 up vs E15.5 * Ctrl
10435789	Zbtb20	ENSMUST00000(	8.49754e-007	2.17878	E15.5 * Wnt1 up vs E15.5 * Ctrl
10601850	Bex4	NM_212457	6.59854e-009	2.17428	E15.5 * Wnt1 up vs E15.5 * Ctrl
10352867	Plxna2	NM_008882	1.39719e-008	2.17319	E15.5 * Wnt1 up vs E15.5 * Ctrl
10518147	Pdpn	NM_010329	7.72993e-006	2.17187	E15.5 * Wnt1 up vs E15.5 * Ctrl
10472368	Scn2a1	NM_001099298	3.6595e-005	2.17138	E15.5 * Wnt1 up vs E15.5 * Ctrl
10390542	Cacnb1	NM_031173	7.97663e-007	2.17036	E15.5 * Wnt1 up vs E15.5 * Ctrl
10394674	Socs2	NM_007706	3.6574e-005	2.17036	E15.5 * Wnt1 up vs E15.5 * Ctrl
10518428	Clcn6	NM_011929	3.20517e-006	2.1692	E15.5 * Wnt1 up vs E15.5 * Ctrl
10551065	D930028M14Rik	ENSMUST00000(	8.83254e-008	2.16444	E15.5 * Wnt1 up vs E15.5 * Ctrl
10571353	6430573F11Rik	BC052184	2.03954e-007	2.16431	E15.5 * Wnt1 up vs E15.5 * Ctrl
10475247	Tmem62	NM_175285	1.43662e-005	2.1637	E15.5 * Wnt1 up vs E15.5 * Ctrl
10567589	Usp31	NM_001033173	2.45469e-005	2.16205	E15.5 * Wnt1 up vs E15.5 * Ctrl
10368484	ENSMUSG0000C	ENSMUST00000(	1.01217e-008	2.16161	E15.5 * Wnt1 up vs E15.5 * Ctrl
10590865	Cntn5	NM_001033359	2.38069e-008	2.16035	E15.5 * Wnt1 up vs E15.5 * Ctrl
10538290	Snx10	NM_028035	2.94163e-008	2.15923	E15.5 * Wnt1 up vs E15.5 * Ctrl
10375432	C030019I05Rik	NM_177075	9.47152e-005	2.15598	E15.5 * Wnt1 up vs E15.5 * Ctrl
10563939		---	0.000242286	2.15571	E15.5 * Wnt1 up vs E15.5 * Ctrl
10440849	Synj1	ENSMUST00000(	1.18374e-009	2.1544	E15.5 * Wnt1 up vs E15.5 * Ctrl
10513776	Astn2	NM_207109	1.81123e-007	2.15362	E15.5 * Wnt1 up vs E15.5 * Ctrl
10580469	Cbln1	NM_019626	2.69742e-007	2.15311	E15.5 * Wnt1 up vs E15.5 * Ctrl
10546977	Atp2b2	NM_009723	3.05683e-009	2.14981	E15.5 * Wnt1 up vs E15.5 * Ctrl
10423836	Cthrc1	NM_026778	4.57453e-009	2.14698	E15.5 * Wnt1 up vs E15.5 * Ctrl
10549879	Usp29	NM_021323	2.65719e-007	2.14641	E15.5 * Wnt1 up vs E15.5 * Ctrl
10563915		---	1.14192e-007	2.14486	E15.5 * Wnt1 up vs E15.5 * Ctrl
10563929		---	1.14192e-007	2.14486	E15.5 * Wnt1 up vs E15.5 * Ctrl
10419170	Txndc16	NM_172597	1.03464e-006	2.14436	E15.5 * Wnt1 up vs E15.5 * Ctrl
10448967	Sox8	NM_011447	4.43586e-007	2.14291	E15.5 * Wnt1 up vs E15.5 * Ctrl
10430282	Cacng2	NM_007583	7.02155e-007	2.14147	E15.5 * Wnt1 up vs E15.5 * Ctrl
10501963	Ugt8a	NM_011674	6.71504e-005	2.14027	E15.5 * Wnt1 up vs E15.5 * Ctrl
10506004	Hook1	NM_030014	1.67583e-007	2.13941	E15.5 * Wnt1 up vs E15.5 * Ctrl
10545099		---	5.51028e-005	2.13738	E15.5 * Wnt1 up vs E15.5 * Ctrl
10417048	Hs6st3	NM_015820	3.52374e-008	2.13631	E15.5 * Wnt1 up vs E15.5 * Ctrl
10551410	Zfp60	NM_009560	9.75167e-008	2.13547	E15.5 * Wnt1 up vs E15.5 * Ctrl
10347226	Tmem169	NM_175564	1.02141e-006	2.13373	E15.5 * Wnt1 up vs E15.5 * Ctrl
10503192	Chd7	NM_001081417	3.82738e-007	2.13231	E15.5 * Wnt1 up vs E15.5 * Ctrl
10588755	Camkv	NM_145621	4.61763e-006	2.13017	E15.5 * Wnt1 up vs E15.5 * Ctrl
10353460	Kcnq5	NM_023872	6.41596e-008	2.12978	E15.5 * Wnt1 up vs E15.5 * Ctrl
10437580		---	4.703e-006	2.12955	E15.5 * Wnt1 up vs E15.5 * Ctrl
10485580	Cstf3	NM_145529	1.9563e-005	2.12825	E15.5 * Wnt1 up vs E15.5 * Ctrl
10474064	Trp53i11	NM_001025246	2.80627e-005	2.12686	E15.5 * Wnt1 up vs E15.5 * Ctrl
10532784	Svop	NM_026805	4.22999e-007	2.12632	E15.5 * Wnt1 up vs E15.5 * Ctrl
10524621	Oasl2	NM_011854	7.2176e-009	2.1184	E15.5 * Wnt1 up vs E15.5 * Ctrl
10540233	Fam19a1	NM_182808	9.13737e-007	2.11525	E15.5 * Wnt1 up vs E15.5 * Ctrl
10476401	Plcb1	NM_019677	4.43327e-007	2.11251	E15.5 * Wnt1 up vs E15.5 * Ctrl
10572070	Npy1r	NM_010934	1.25795e-006	2.11107	E15.5 * Wnt1 up vs E15.5 * Ctrl
10418817	Chat	NM_009891	1.60276e-008	2.10966	E15.5 * Wnt1 up vs E15.5 * Ctrl
10499981	Oaz3	NM_016901	5.01934e-007	2.10731	E15.5 * Wnt1 up vs E15.5 * Ctrl
10529923	Lcorl	NM_172153	5.38799e-007	2.10705	E15.5 * Wnt1 up vs E15.5 * Ctrl
10434629	Map3k13	NM_172821	5.74661e-007	2.10551	E15.5 * Wnt1 up vs E15.5 * Ctrl
10379223	1810012P15Rik	NM_001076681	9.23254e-007	2.10109	E15.5 * Wnt1 up vs E15.5 * Ctrl
10492826	Fbxw7	NM_080428	6.35038e-011	2.10027	E15.5 * Wnt1 up vs E15.5 * Ctrl
10458417	ENSMUSG0000C	ENSMUST00000(	0.00013551	2.10023	E15.5 * Wnt1 up vs E15.5 * Ctrl
10528143	Ppp1r14b	NM_008889	4.36576e-011	2.09988	E15.5 * Wnt1 up vs E15.5 * Ctrl
10448557	Abca3	NM_013855	7.21232e-006	2.09881	E15.5 * Wnt1 up vs E15.5 * Ctrl
10590306	Entpd3	NM_178676	1.97515e-007	2.09613	E15.5 * Wnt1 up vs E15.5 * Ctrl

10601701	Tmem35	NM_026239	2.84169e-007	2.09324	E15.5 * Wnt1 up vs E15.5 * Ctrl
10469767	Nxph2	NM_008752	5.8785e-006	2.09281	E15.5 * Wnt1 up vs E15.5 * Ctrl
10450622	2610110G12Rik	NM_001142744	7.39723e-006	2.09093	E15.5 * Wnt1 up vs E15.5 * Ctrl
10544462	Fam115a	NM_029930	3.43536e-008	2.08933	E15.5 * Wnt1 up vs E15.5 * Ctrl
10528038	Adam22	NM_001007220	1.57234e-008	2.0893	E15.5 * Wnt1 up vs E15.5 * Ctrl
10384838	C230094A16Rik	NM_146016	3.84676e-007	2.08901	E15.5 * Wnt1 up vs E15.5 * Ctrl
10457834		---	6.40865e-006	2.08868	E15.5 * Wnt1 up vs E15.5 * Ctrl
10597309	Stac	NM_016853	3.98715e-007	2.08756	E15.5 * Wnt1 up vs E15.5 * Ctrl
10368577	Rnf217	NM_001146349	2.97042e-007	2.08752	E15.5 * Wnt1 up vs E15.5 * Ctrl
10449258	Arhgdig	NM_008113	7.36061e-007	2.08468	E15.5 * Wnt1 up vs E15.5 * Ctrl
10589685	Lrrc2	NM_028838	2.15099e-006	2.08406	E15.5 * Wnt1 up vs E15.5 * Ctrl
10564262	Mkrn3	NM_011746	2.20375e-005	2.081	E15.5 * Wnt1 up vs E15.5 * Ctrl
10366712	Ppm1h	NM_001110218	1.98922e-008	2.07891	E15.5 * Wnt1 up vs E15.5 * Ctrl
10419578	Ndrg2	NM_013864	2.23137e-007	2.0766	E15.5 * Wnt1 up vs E15.5 * Ctrl
10423258	Cdh12	NM_001008420	0.000112344	2.07658	E15.5 * Wnt1 up vs E15.5 * Ctrl
10577586	Ap3m2	NM_001122820	1.65853e-008	2.07638	E15.5 * Wnt1 up vs E15.5 * Ctrl
10431872	Slc38a1	ENSMUST00000	4.44075e-009	2.07488	E15.5 * Wnt1 up vs E15.5 * Ctrl
10438069	Ypel1	NM_023249	3.32045e-008	2.07463	E15.5 * Wnt1 up vs E15.5 * Ctrl
10376096	Acsl6	NM_001033598	3.98922e-010	2.07295	E15.5 * Wnt1 up vs E15.5 * Ctrl
10428443	Kcnv1	NM_026200	3.65702e-006	2.0683	E15.5 * Wnt1 up vs E15.5 * Ctrl
10495285	Sort1	NM_019972	4.06561e-010	2.06585	E15.5 * Wnt1 up vs E15.5 * Ctrl
10567725	Zkscan2	NM_001081329	1.26628e-007	2.06448	E15.5 * Wnt1 up vs E15.5 * Ctrl
10543120	Ica1	NM_010492	4.01236e-007	2.06264	E15.5 * Wnt1 up vs E15.5 * Ctrl
10373542	Dgka	NM_016811	8.30111e-009	2.06245	E15.5 * Wnt1 up vs E15.5 * Ctrl
10438017	Fgd4	NM_139232	2.62781e-007	2.06176	E15.5 * Wnt1 up vs E15.5 * Ctrl
10565102	Ap3b2	NM_021492	1.62484e-007	2.06066	E15.5 * Wnt1 up vs E15.5 * Ctrl
10395466	Dock4	NM_172803	4.89174e-009	2.06014	E15.5 * Wnt1 up vs E15.5 * Ctrl
10362490	Tspyl4	NM_030203	7.60677e-008	2.05792	E15.5 * Wnt1 up vs E15.5 * Ctrl
10430519	Csnk1e	NM_013767	1.3053e-007	2.05547	E15.5 * Wnt1 up vs E15.5 * Ctrl
10485582	Tcp11l1	NM_177190	3.75969e-007	2.05417	E15.5 * Wnt1 up vs E15.5 * Ctrl
10363415	Spock2	NM_052994	5.15903e-007	2.05317	E15.5 * Wnt1 up vs E15.5 * Ctrl
10512315	Ccl27a	NM_001048179	3.40883e-007	2.0529	E15.5 * Wnt1 up vs E15.5 * Ctrl
10556509	Spon1	NM_145584	5.12083e-009	2.05094	E15.5 * Wnt1 up vs E15.5 * Ctrl
10476592	Macrod2	NM_001013802	6.59079e-005	2.05052	E15.5 * Wnt1 up vs E15.5 * Ctrl
10394158	Kif3c	NM_008445	1.5318e-007	2.04984	E15.5 * Wnt1 up vs E15.5 * Ctrl
10553635	Nipa1	NM_153578	1.27135e-007	2.04886	E15.5 * Wnt1 up vs E15.5 * Ctrl
10402318	Prima1	NM_133364	1.11604e-005	2.0481	E15.5 * Wnt1 up vs E15.5 * Ctrl
10564272	Chrna7	NM_007390	1.25835e-007	2.0477	E15.5 * Wnt1 up vs E15.5 * Ctrl
10503593	6230409E13Rik	NM_175234	1.50852e-008	2.04759	E15.5 * Wnt1 up vs E15.5 * Ctrl
10423471	Ctnnd2	NM_008729	1.52668e-007	2.04699	E15.5 * Wnt1 up vs E15.5 * Ctrl
10482181	Strbp	NM_009261	1.28803e-011	2.04692	E15.5 * Wnt1 up vs E15.5 * Ctrl
10395971		---	0.000225074	2.04649	E15.5 * Wnt1 up vs E15.5 * Ctrl
10371466	Syn3	NM_013722	3.38494e-006	2.0464	E15.5 * Wnt1 up vs E15.5 * Ctrl
10593497	Zc3h12c	ENSMUST00000	1.44229e-006	2.04011	E15.5 * Wnt1 up vs E15.5 * Ctrl
10358191	Camsap1l1	NM_001081360	1.03928e-011	2.03854	E15.5 * Wnt1 up vs E15.5 * Ctrl
10480381	Arhgap21	NM_001128084	1.78073e-012	2.03717	E15.5 * Wnt1 up vs E15.5 * Ctrl
10373950	Nipsnap1	NM_008698	4.3676e-009	2.03321	E15.5 * Wnt1 up vs E15.5 * Ctrl
10406941	Sgtb	NM_144838	2.00785e-005	2.03252	E15.5 * Wnt1 up vs E15.5 * Ctrl
10488033	Pak7	NM_172858	1.72832e-009	2.03061	E15.5 * Wnt1 up vs E15.5 * Ctrl
10571860	Hand2	NM_010402	3.29079e-008	2.02914	E15.5 * Wnt1 up vs E15.5 * Ctrl
10548745	Gpr19	NM_008157	2.599e-007	2.02809	E15.5 * Wnt1 up vs E15.5 * Ctrl
10419414		---	0.00132809	2.02754	E15.5 * Wnt1 up vs E15.5 * Ctrl
10548051	Kcna6	NM_013568	1.38051e-006	2.02731	E15.5 * Wnt1 up vs E15.5 * Ctrl
10411782	Pik3r1	NM_001077495	9.52161e-009	2.02672	E15.5 * Wnt1 up vs E15.5 * Ctrl
10546685	Eif4e3	NM_025829	3.10139e-009	2.02491	E15.5 * Wnt1 up vs E15.5 * Ctrl
10418053	Kcnma1	NM_010610	1.31331e-007	2.0236	E15.5 * Wnt1 up vs E15.5 * Ctrl
10555027	Gab2	NM_010248	3.86823e-009	2.02104	E15.5 * Wnt1 up vs E15.5 * Ctrl
10399470	Trib2	NM_144551	1.45844e-008	2.01868	E15.5 * Wnt1 up vs E15.5 * Ctrl
10504164	Ccl27a	NM_011336	2.99228e-006	2.01778	E15.5 * Wnt1 up vs E15.5 * Ctrl
10504194	Ccl27a	NM_011336	2.99228e-006	2.01778	E15.5 * Wnt1 up vs E15.5 * Ctrl
10583992	Igsf9b	NM_001129787	3.47072e-009	2.01675	E15.5 * Wnt1 up vs E15.5 * Ctrl
10445338	Enpp5	NM_032003	1.02839e-006	2.01659	E15.5 * Wnt1 up vs E15.5 * Ctrl
10413416	Il17rd	NM_134437	2.21309e-007	2.01592	E15.5 * Wnt1 up vs E15.5 * Ctrl
10398996	Crip2	NM_024223	2.00441e-005	2.01556	E15.5 * Wnt1 up vs E15.5 * Ctrl
10447294	Prkce	NM_011104	2.2036e-008	2.01338	E15.5 * Wnt1 up vs E15.5 * Ctrl
10607888	Frmpd4	NM_001033330	3.82787e-007	2.01251	E15.5 * Wnt1 up vs E15.5 * Ctrl

10489985	Atp9a	NM_015731	3.8889e-006	2.00841	E15.5 * Wnt1 up vs E15.5 * Ctrl
10499045	Trim2	NM_030706	2.08617e-009	2.00748	E15.5 * Wnt1 up vs E15.5 * Ctrl
10380648	Hoxb4	NM_010459	2.94347e-008	2.00679	E15.5 * Wnt1 up vs E15.5 * Ctrl
10357472	Cxcr4	NM_009911	1.26871e-005	2.00607	E15.5 * Wnt1 up vs E15.5 * Ctrl
10512847	Alg2	NM_019998	7.21992e-008	2.00493	E15.5 * Wnt1 up vs E15.5 * Ctrl
10603598	Rpgr	NM_011285	4.34856e-006	2.00467	E15.5 * Wnt1 up vs E15.5 * Ctrl
10507798	Mycl1	NM_008506	5.41235e-007	2.00239	E15.5 * Wnt1 up vs E15.5 * Ctrl
10455019	Pcdha10	NM_009961	1.63669e-009	2.00191	E15.5 * Wnt1 up vs E15.5 * Ctrl
10416175	Nefl	NM_010910	2.06367e-013	10.3348	E15.5 * Wnt1 up vs E15.5 * Ctrl
10488617	Nrsn2	NM_001009948	4.72542e-006	1.99882	E15.5 * Wnt1 up vs E15.5 * Ctrl
10492522	Schip1	NM_001113421	3.4736e-008	1.99721	E15.5 * Wnt1 up vs E15.5 * Ctrl
10470834	Spna2	NM_001076554	1.14854e-008	1.99713	E15.5 * Wnt1 up vs E15.5 * Ctrl
10506714	Lrp8	NM_053073	1.89975e-007	1.99329	E15.5 * Wnt1 up vs E15.5 * Ctrl
10556598	Xylt1	NM_175645	9.046e-006	1.99316	E15.5 * Wnt1 up vs E15.5 * Ctrl
10526853	Fam20c	NM_030565	7.19615e-006	1.99208	E15.5 * Wnt1 up vs E15.5 * Ctrl
10396314	Lrrc9	NM_001142728	1.01246e-006	1.99047	E15.5 * Wnt1 up vs E15.5 * Ctrl
10360972	Kcnk2	NM_010607	2.74448e-007	1.98778	E15.5 * Wnt1 up vs E15.5 * Ctrl
10477986	Nnat	NM_010923	3.88782e-006	1.98745	E15.5 * Wnt1 up vs E15.5 * Ctrl
10364769	Apc2	NM_011789	3.53688e-006	1.98592	E15.5 * Wnt1 up vs E15.5 * Ctrl
10571384	Efha2	ENSMUST00000(	6.00312e-007	1.98533	E15.5 * Wnt1 up vs E15.5 * Ctrl
10461022	Ppp1r14b	NM_008889	1.45755e-011	1.98284	E15.5 * Wnt1 up vs E15.5 * Ctrl
10484941	Madd	NM_145527	2.57324e-010	1.98244	E15.5 * Wnt1 up vs E15.5 * Ctrl
10513544	Zfp37	NM_009554	1.03158e-007	1.98185	E15.5 * Wnt1 up vs E15.5 * Ctrl
10532680	Sgsm1	NM_172718	1.22333e-005	1.9811	E15.5 * Wnt1 up vs E15.5 * Ctrl
10352178	Sccpdh	NM_178653	1.69253e-008	1.98097	E15.5 * Wnt1 up vs E15.5 * Ctrl
10381934	Tanc2	NM_181071	6.89251e-006	1.98056	E15.5 * Wnt1 up vs E15.5 * Ctrl
10365471	Fbxo7	NM_153195	2.79687e-009	1.97755	E15.5 * Wnt1 up vs E15.5 * Ctrl
10374406	Cnrip1	NM_029861	6.97719e-008	1.97636	E15.5 * Wnt1 up vs E15.5 * Ctrl
10371332	Aldh1l2	NM_153543	3.04401e-007	1.97621	E15.5 * Wnt1 up vs E15.5 * Ctrl
10381187	Atp6v0a1	NM_016920	6.08034e-009	1.97298	E15.5 * Wnt1 up vs E15.5 * Ctrl
10561369	BC089491	NM_175033	3.81185e-007	1.97155	E15.5 * Wnt1 up vs E15.5 * Ctrl
10469613	Thnsl1	NM_177588	1.74718e-007	1.97086	E15.5 * Wnt1 up vs E15.5 * Ctrl
10456161	Htr4	NM_008313	3.55864e-008	1.97065	E15.5 * Wnt1 up vs E15.5 * Ctrl
10490559	Chrna4	NM_015730	1.76728e-005	1.96944	E15.5 * Wnt1 up vs E15.5 * Ctrl
10473058	Osbpl6	NM_145525	1.59728e-007	1.96869	E15.5 * Wnt1 up vs E15.5 * Ctrl
10441497	Tulp4	NM_054040	1.76091e-005	1.96747	E15.5 * Wnt1 up vs E15.5 * Ctrl
10579165	Ncan	NM_007789	4.54367e-007	1.96654	E15.5 * Wnt1 up vs E15.5 * Ctrl
10454655	Apc	NM_007462	3.35622e-009	1.96409	E15.5 * Wnt1 up vs E15.5 * Ctrl
10417713	Rarb	NM_011243	1.2103e-007	1.96344	E15.5 * Wnt1 up vs E15.5 * Ctrl
10451167	Tmem63b	NM_198167	7.64491e-007	1.96201	E15.5 * Wnt1 up vs E15.5 * Ctrl
10550915	Cadm4	NM_153112	2.30987e-005	1.95975	E15.5 * Wnt1 up vs E15.5 * Ctrl
10496192	Tacr3	NM_021382	2.32596e-005	1.95926	E15.5 * Wnt1 up vs E15.5 * Ctrl
10470027	Npdc1	NM_008721	1.07194e-006	1.95894	E15.5 * Wnt1 up vs E15.5 * Ctrl
10434165	Arvcf	NM_033474	2.37861e-007	1.95808	E15.5 * Wnt1 up vs E15.5 * Ctrl
10367803		---	0.000417977	1.95728	E15.5 * Wnt1 up vs E15.5 * Ctrl
10476314	Prnp	NM_011170	1.18819e-008	1.95719	E15.5 * Wnt1 up vs E15.5 * Ctrl
10379482	Cdk5r1	NM_009871	1.94635e-008	1.957	E15.5 * Wnt1 up vs E15.5 * Ctrl
10446777	Ehd3	NM_020578	2.23521e-006	1.95395	E15.5 * Wnt1 up vs E15.5 * Ctrl
10375265	Atp10b	NM_176999	7.24012e-007	1.95318	E15.5 * Wnt1 up vs E15.5 * Ctrl
10456254	Nedd4l	NM_001114386	9.41493e-008	1.9496	E15.5 * Wnt1 up vs E15.5 * Ctrl
10458913	Cep120	NM_178686	1.53608e-008	1.94896	E15.5 * Wnt1 up vs E15.5 * Ctrl
10388160	Slc13a5	NM_001004148	1.10497e-007	1.94804	E15.5 * Wnt1 up vs E15.5 * Ctrl
10392347	Pitpnc1	NM_145823	8.76864e-010	1.94787	E15.5 * Wnt1 up vs E15.5 * Ctrl
10499091	Dear1	NM_001040461	4.32584e-006	1.94743	E15.5 * Wnt1 up vs E15.5 * Ctrl
10578300	Mtmr7	NM_001040699	1.94871e-007	1.94713	E15.5 * Wnt1 up vs E15.5 * Ctrl
10415187	Lrrc16b	NM_001024645	9.84557e-007	1.94587	E15.5 * Wnt1 up vs E15.5 * Ctrl
10457895	Brunol4	NM_001146292	6.28729e-007	1.94317	E15.5 * Wnt1 up vs E15.5 * Ctrl
10564111		---	3.80209e-008	1.94046	E15.5 * Wnt1 up vs E15.5 * Ctrl
10593421	1110032A03Rik	NM_023483	0.000413034	1.93861	E15.5 * Wnt1 up vs E15.5 * Ctrl
10534168	Auts2	NM_177047	1.5969e-009	1.93753	E15.5 * Wnt1 up vs E15.5 * Ctrl
10601834	Gprasp1	NM_026081	1.18362e-005	1.93726	E15.5 * Wnt1 up vs E15.5 * Ctrl
10409222	Shc3	NM_009167	2.76543e-007	1.93583	E15.5 * Wnt1 up vs E15.5 * Ctrl
10477944		---	0.000457258	1.93382	E15.5 * Wnt1 up vs E15.5 * Ctrl
10351269	4930455F23Rik	NM_029115	1.608e-007	1.93359	E15.5 * Wnt1 up vs E15.5 * Ctrl
10572727	OTTMUSG00000	BC080687	4.25572e-005	1.93336	E15.5 * Wnt1 up vs E15.5 * Ctrl
10538082	Atp6v0e2	NM_133764	6.39177e-007	1.93249	E15.5 * Wnt1 up vs E15.5 * Ctrl

10576062	Jph3	NM_020605	3.61597e-007	1.9305	E15.5 * Wnt1 up vs E15.5 * Ctrl
10491885	Pcdh10	NM_001098171	7.1591e-006	1.92969	E15.5 * Wnt1 up vs E15.5 * Ctrl
10490802	Fam164a	NM_173181	0.000129185	1.92916	E15.5 * Wnt1 up vs E15.5 * Ctrl
10549162	St8sia1	NM_011374	1.0902e-006	1.92866	E15.5 * Wnt1 up vs E15.5 * Ctrl
10431697	Abcd2	NM_011994	0.000100537	1.92731	E15.5 * Wnt1 up vs E15.5 * Ctrl
10397882	Chga	NM_007693	1.55866e-006	1.92529	E15.5 * Wnt1 up vs E15.5 * Ctrl
10483624	Dlx1as	NR_002854	9.66002e-006	1.92082	E15.5 * Wnt1 up vs E15.5 * Ctrl
10588037	Rbp1	NM_011254	7.731e-009	1.92075	E15.5 * Wnt1 up vs E15.5 * Ctrl
10386636	Usp22	NM_001004143	1.07645e-009	1.92047	E15.5 * Wnt1 up vs E15.5 * Ctrl
10422628	Plcxd3	NM_177355	1.68245e-007	1.91799	E15.5 * Wnt1 up vs E15.5 * Ctrl
10536353	Dlx6	NM_010057	1.24717e-005	1.91698	E15.5 * Wnt1 up vs E15.5 * Ctrl
10400023	Tspan13	NM_025359	5.32747e-008	1.91636	E15.5 * Wnt1 up vs E15.5 * Ctrl
10564209		---	4.07536e-005	1.91514	E15.5 * Wnt1 up vs E15.5 * Ctrl
10434302	Klhl24	NM_029436	1.23252e-008	1.91431	E15.5 * Wnt1 up vs E15.5 * Ctrl
10409616	Spock1	NM_009262	8.85717e-008	1.91365	E15.5 * Wnt1 up vs E15.5 * Ctrl
10593384	Dixdc1	NM_178118	1.94085e-009	1.91265	E15.5 * Wnt1 up vs E15.5 * Ctrl
10452935	Heatr5b	BC019508	4.0431e-005	1.91212	E15.5 * Wnt1 up vs E15.5 * Ctrl
10538753	Atoh1	BC010820	2.34636e-005	1.91101	E15.5 * Wnt1 up vs E15.5 * Ctrl
10495781	Bcar3	NM_013867	5.30418e-005	1.91033	E15.5 * Wnt1 up vs E15.5 * Ctrl
10468275	Pcgf6	NM_027654	2.24939e-009	1.91023	E15.5 * Wnt1 up vs E15.5 * Ctrl
10541968	Ano2	NM_153589	7.11497e-008	1.90913	E15.5 * Wnt1 up vs E15.5 * Ctrl
10428857	Mtss1	NM_001146180	4.81758e-008	1.90873	E15.5 * Wnt1 up vs E15.5 * Ctrl
10525195	AU042671	ENSMUST00000(	5.23025e-006	1.90551	E15.5 * Wnt1 up vs E15.5 * Ctrl
10549899	Zfp418	NM_146179	0.000506679	1.9052	E15.5 * Wnt1 up vs E15.5 * Ctrl
10473349	Ypel4	NM_001005342	3.46321e-006	1.90491	E15.5 * Wnt1 up vs E15.5 * Ctrl
10355141	Klf7	NM_033563	1.98074e-009	1.90476	E15.5 * Wnt1 up vs E15.5 * Ctrl
10552929	Lin7b	NM_011698	2.87013e-005	1.90331	E15.5 * Wnt1 up vs E15.5 * Ctrl
10431113	Sult4a1	NM_013873	1.46855e-006	1.90018	E15.5 * Wnt1 up vs E15.5 * Ctrl
10532538	Asphd2	NM_028386	1.02143e-006	1.9001	E15.5 * Wnt1 up vs E15.5 * Ctrl
10476443	Plcb4	NM_013829	7.08012e-008	1.89873	E15.5 * Wnt1 up vs E15.5 * Ctrl
10600419	Plxna3	NM_008883	9.9973e-007	1.89736	E15.5 * Wnt1 up vs E15.5 * Ctrl
10490129	Bmp7	NM_007557	1.77381e-006	1.8948	E15.5 * Wnt1 up vs E15.5 * Ctrl
10455118	Pcdhb18	NM_053143	4.57934e-006	1.89381	E15.5 * Wnt1 up vs E15.5 * Ctrl
10518484	Fbxo44	NM_173401	7.35571e-007	1.89353	E15.5 * Wnt1 up vs E15.5 * Ctrl
10446594		---	1.64809e-006	1.89237	E15.5 * Wnt1 up vs E15.5 * Ctrl
10436978	Cbr3	NM_173047	0.000956448	1.89204	E15.5 * Wnt1 up vs E15.5 * Ctrl
10532052	Hfm1	ENSMUST00000	2.27544e-005	1.89198	E15.5 * Wnt1 up vs E15.5 * Ctrl
10394593	Fam49a	NM_029758	8.12539e-006	1.89175	E15.5 * Wnt1 up vs E15.5 * Ctrl
10571865	Scrg1	NM_009136	8.3158e-005	1.88931	E15.5 * Wnt1 up vs E15.5 * Ctrl
10578957		---	6.87913e-005	1.88883	E15.5 * Wnt1 up vs E15.5 * Ctrl
10377431	Vamp2	NM_009497	1.32707e-007	1.88783	E15.5 * Wnt1 up vs E15.5 * Ctrl
10542522	Plekha5	NM_144920	3.03032e-008	1.88587	E15.5 * Wnt1 up vs E15.5 * Ctrl
10551401	Ttc9b	NM_028417	8.32319e-007	1.88531	E15.5 * Wnt1 up vs E15.5 * Ctrl
10409330	4833439L19Rik	BC033445	8.01933e-005	1.88101	E15.5 * Wnt1 up vs E15.5 * Ctrl
10413726	Tnnc1	NM_009393	0.000636177	1.88099	E15.5 * Wnt1 up vs E15.5 * Ctrl
10584500	Olfr963	NM_001011827	0.000184947	1.8805	E15.5 * Wnt1 up vs E15.5 * Ctrl
10550400	Pnmal2	NM_001099636	1.76424e-006	1.87922	E15.5 * Wnt1 up vs E15.5 * Ctrl
10389617	Ppm1e	NM_177167	7.47978e-007	1.87911	E15.5 * Wnt1 up vs E15.5 * Ctrl
10460118	Socs6	NM_018821	2.46225e-006	1.87909	E15.5 * Wnt1 up vs E15.5 * Ctrl
10435676	Gsk3b	NM_019827	5.62056e-010	1.87881	E15.5 * Wnt1 up vs E15.5 * Ctrl
10504891	Tmeff1	NM_021436	2.53121e-009	1.87581	E15.5 * Wnt1 up vs E15.5 * Ctrl
10399121	Ptprn2	NM_011215	2.6388e-008	1.875	E15.5 * Wnt1 up vs E15.5 * Ctrl
10470564	Ralgds	NM_001145835	1.16557e-005	1.8746	E15.5 * Wnt1 up vs E15.5 * Ctrl
10520544	Mapre3	NM_133350	2.38559e-005	1.87242	E15.5 * Wnt1 up vs E15.5 * Ctrl
10357363	E030049G20Rik	NM_001081756	0.0002472	1.87181	E15.5 * Wnt1 up vs E15.5 * Ctrl
10578623	Wwc2	NM_133791	1.92107e-009	1.87009	E15.5 * Wnt1 up vs E15.5 * Ctrl
10588380	Cpne4	NM_028719	3.77045e-007	1.86971	E15.5 * Wnt1 up vs E15.5 * Ctrl
10574384	Ndrg4	NM_145602	3.21542e-007	1.86961	E15.5 * Wnt1 up vs E15.5 * Ctrl
10596931	Wdr6	NM_031392	2.49401e-007	1.8694	E15.5 * Wnt1 up vs E15.5 * Ctrl
10563905		---	3.68401e-010	1.86758	E15.5 * Wnt1 up vs E15.5 * Ctrl
10563907		---	3.68401e-010	1.86758	E15.5 * Wnt1 up vs E15.5 * Ctrl
10457888	5730494M16Rik	NM_001004361	2.69392e-011	1.86739	E15.5 * Wnt1 up vs E15.5 * Ctrl
10588592	Cacna2d2	NM_020263	3.71325e-008	1.86584	E15.5 * Wnt1 up vs E15.5 * Ctrl
10504068	1700066J24Rik	ENSMUST00000(	0.000101428	1.86571	E15.5 * Wnt1 up vs E15.5 * Ctrl
10422585	Fgf14	NM_207667	2.72746e-005	1.86539	E15.5 * Wnt1 up vs E15.5 * Ctrl
10443690	Glp1r	NM_021332	6.23606e-005	1.86453	E15.5 * Wnt1 up vs E15.5 * Ctrl

10469951	Rnf208	NM_176834	1.21744e-007	1.86346	E15.5 * Wnt1 up vs E15.5 * Ctrl
10476326	Cds2	NM_138651	7.90863e-007	1.86279	E15.5 * Wnt1 up vs E15.5 * Ctrl
10592535	Sorl1	NM_011436	6.47443e-009	1.85982	E15.5 * Wnt1 up vs E15.5 * Ctrl
10371396	Mterfd3	NM_028832	0.000131265	1.85874	E15.5 * Wnt1 up vs E15.5 * Ctrl
10458940	Zfp608	NM_175751	8.83205e-010	1.85836	E15.5 * Wnt1 up vs E15.5 * Ctrl
10362794	Ppil6	NM_028430	0.000368136	1.85768	E15.5 * Wnt1 up vs E15.5 * Ctrl
10404356	OTTMUSG00000	XM_909282	0.00337601	1.85602	E15.5 * Wnt1 up vs E15.5 * Ctrl
10431874	Slc38a1	NM_134086	1.26176e-008	1.85337	E15.5 * Wnt1 up vs E15.5 * Ctrl
10563685	Ptpn5	NM_013643	6.14588e-006	1.85254	E15.5 * Wnt1 up vs E15.5 * Ctrl
10563945		---	1.01468e-006	1.85177	E15.5 * Wnt1 up vs E15.5 * Ctrl
10505998	Fggy	NM_001113412	0.00182033	1.85141	E15.5 * Wnt1 up vs E15.5 * Ctrl
10368585	Nkain2	NM_001013411	1.09197e-005	1.85135	E15.5 * Wnt1 up vs E15.5 * Ctrl
10513004	ENSMUSG00000	ENSMUST00000	0.00499165	1.85047	E15.5 * Wnt1 up vs E15.5 * Ctrl
10501483	Ntng1	ENSMUST00000	3.3456e-007	1.85026	E15.5 * Wnt1 up vs E15.5 * Ctrl
10528546	Gabarapl2	NM_026693	1.67541e-007	1.85013	E15.5 * Wnt1 up vs E15.5 * Ctrl
10492590	Ppm1l	NM_178726	3.14817e-006	1.84942	E15.5 * Wnt1 up vs E15.5 * Ctrl
10598164	0610010K06Rik	NM_027861	0.000408135	1.84881	E15.5 * Wnt1 up vs E15.5 * Ctrl
10416956		---	0.00102519	1.84837	E15.5 * Wnt1 up vs E15.5 * Ctrl
10572485	Rab3a	NM_009001	8.7336e-008	1.84833	E15.5 * Wnt1 up vs E15.5 * Ctrl
10444332	BC051142	NM_001001177	0.000208687	1.84526	E15.5 * Wnt1 up vs E15.5 * Ctrl
10480676	Grin1	NM_008169	1.28395e-009	1.8434	E15.5 * Wnt1 up vs E15.5 * Ctrl
10395142	Sh3yl1	NM_013709	8.36957e-008	1.84267	E15.5 * Wnt1 up vs E15.5 * Ctrl
10576946	Lig4	NM_176953	0.000206967	1.84107	E15.5 * Wnt1 up vs E15.5 * Ctrl
10430574	4933432B09Rik	BC118956	2.14599e-007	1.84105	E15.5 * Wnt1 up vs E15.5 * Ctrl
10462039	Trpm3	NM_001035244	1.144e-009	1.84034	E15.5 * Wnt1 up vs E15.5 * Ctrl
10588091	Cep70	NM_023873	0.000170833	1.83956	E15.5 * Wnt1 up vs E15.5 * Ctrl
10571922	Nek1	NM_175089	3.25702e-006	1.83928	E15.5 * Wnt1 up vs E15.5 * Ctrl
10417798	Kcnk5	NM_021542	4.90857e-005	1.83818	E15.5 * Wnt1 up vs E15.5 * Ctrl
10511665	Necab1	NM_178617	3.84037e-005	1.8378	E15.5 * Wnt1 up vs E15.5 * Ctrl
10549171	5730419I09Rik	NM_029081	1.48563e-007	1.83748	E15.5 * Wnt1 up vs E15.5 * Ctrl
10594513		---	0.000421318	1.83725	E15.5 * Wnt1 up vs E15.5 * Ctrl
10571312	Dusp4	NM_176933	3.96282e-005	1.83687	E15.5 * Wnt1 up vs E15.5 * Ctrl
10457733	B4galt6	NM_019737	1.78431e-007	1.83623	E15.5 * Wnt1 up vs E15.5 * Ctrl
10532180	Cplx1	NM_007756	2.088e-006	1.83607	E15.5 * Wnt1 up vs E15.5 * Ctrl
10576854	Ctxn1	NM_183315	9.62128e-007	1.83577	E15.5 * Wnt1 up vs E15.5 * Ctrl
10521471	Ppp2r2c	NM_172994	0.000222542	1.83567	E15.5 * Wnt1 up vs E15.5 * Ctrl
10414278	Gpr137c	NM_027518	9.06043e-007	1.83553	E15.5 * Wnt1 up vs E15.5 * Ctrl
10578709	Wdr17	NM_028220	2.75333e-006	1.83388	E15.5 * Wnt1 up vs E15.5 * Ctrl
10536931	Ahcyl2	NM_021414	6.70296e-006	1.83333	E15.5 * Wnt1 up vs E15.5 * Ctrl
10544829	Jazf1	NM_173406	5.85114e-008	1.83262	E15.5 * Wnt1 up vs E15.5 * Ctrl
10583195	ENSMUSG00000	ENSMUST00000	5.98299e-005	1.83223	E15.5 * Wnt1 up vs E15.5 * Ctrl
10499108	Glt28d2	NM_177130	6.93533e-006	1.82848	E15.5 * Wnt1 up vs E15.5 * Ctrl
10491962	Foxo1	NM_019739	4.27796e-008	1.82816	E15.5 * Wnt1 up vs E15.5 * Ctrl
10455238	Ndfip1	NM_022996	7.94916e-010	1.82619	E15.5 * Wnt1 up vs E15.5 * Ctrl
10605067	Pnck	NM_012040	2.36225e-006	1.82611	E15.5 * Wnt1 up vs E15.5 * Ctrl
10448247	Zfp40	NM_009555	0.000136078	1.8261	E15.5 * Wnt1 up vs E15.5 * Ctrl
10580870	Zfp319	ENSMUST00000	0.000113956	1.82591	E15.5 * Wnt1 up vs E15.5 * Ctrl
10459262	Ablim3	NM_198649	1.49314e-006	1.8256	E15.5 * Wnt1 up vs E15.5 * Ctrl
10601874	Tceal3	NM_001029978	6.41793e-007	1.82398	E15.5 * Wnt1 up vs E15.5 * Ctrl
10604057	06-Sep	NM_019942	2.63098e-006	1.82209	E15.5 * Wnt1 up vs E15.5 * Ctrl
10400141	Zbed4	NM_181412	3.67189e-005	1.82188	E15.5 * Wnt1 up vs E15.5 * Ctrl
10510422	Casz1	NM_001159344	2.11855e-008	1.82072	E15.5 * Wnt1 up vs E15.5 * Ctrl
10483150	Fign	NM_021716	1.45913e-005	1.82036	E15.5 * Wnt1 up vs E15.5 * Ctrl
10426581	AI836003	NM_177716	1.12136e-005	1.81972	E15.5 * Wnt1 up vs E15.5 * Ctrl
10347060	Unc80	FJ210934	1.19531e-006	1.81886	E15.5 * Wnt1 up vs E15.5 * Ctrl
10465686	Rtn3	NM_001003934	4.83762e-010	1.81817	E15.5 * Wnt1 up vs E15.5 * Ctrl
10445078	Gabbr1	NM_019439	4.02504e-007	1.81668	E15.5 * Wnt1 up vs E15.5 * Ctrl
10477644	Trp53inp2	NM_178111	5.99064e-007	1.81609	E15.5 * Wnt1 up vs E15.5 * Ctrl
10467749	Crtac1	NM_145123	1.30977e-008	1.81585	E15.5 * Wnt1 up vs E15.5 * Ctrl
10426798	Smarcd1	NM_031842	3.03392e-006	1.8153	E15.5 * Wnt1 up vs E15.5 * Ctrl
10494388	Hist2h2be	NM_178214	0.000614061	1.81517	E15.5 * Wnt1 up vs E15.5 * Ctrl
10363868	Fam13c	NM_024244	3.48794e-008	1.81434	E15.5 * Wnt1 up vs E15.5 * Ctrl
10374998	Gpr75	NM_175490	5.90433e-005	1.81392	E15.5 * Wnt1 up vs E15.5 * Ctrl
10400155	Nova1	ENSMUST00000	7.0746e-005	1.81336	E15.5 * Wnt1 up vs E15.5 * Ctrl
10547404	Erc1	NM_053204	6.99486e-005	1.81329	E15.5 * Wnt1 up vs E15.5 * Ctrl
10374197	Ramp3	NM_019511	6.09542e-006	1.81314	E15.5 * Wnt1 up vs E15.5 * Ctrl

10495518	Olfm3	NM_153157	2.66285e-006	1.81113	E15.5 * Wnt1 up vs E15.5 * Ctrl
10502419	Rap1gds1	NM_001040690	1.02049e-007	1.81091	E15.5 * Wnt1 up vs E15.5 * Ctrl
10436594		---	0.00200006	1.80938	E15.5 * Wnt1 up vs E15.5 * Ctrl
10501778	Ptbp2	NM_019550	3.01276e-007	1.80886	E15.5 * Wnt1 up vs E15.5 * Ctrl
10379936	Tbx2	NM_009324	4.65131e-005	1.8073	E15.5 * Wnt1 up vs E15.5 * Ctrl
10379795	Ap1gbp1	NM_001115009	1.40569e-007	1.80718	E15.5 * Wnt1 up vs E15.5 * Ctrl
10490838	Fabp5	NM_010634	2.853e-008	1.80671	E15.5 * Wnt1 up vs E15.5 * Ctrl
10415866	Xkr6	NM_173393	3.05261e-005	1.80627	E15.5 * Wnt1 up vs E15.5 * Ctrl
10577544	Polb	NM_011130	4.82137e-007	1.80557	E15.5 * Wnt1 up vs E15.5 * Ctrl
10399725	Sox11	NM_009234	1.94121e-012	1.80466	E15.5 * Wnt1 up vs E15.5 * Ctrl
10536068	Zfp788	NM_023363	6.71453e-005	1.80415	E15.5 * Wnt1 up vs E15.5 * Ctrl
10552320	Zfp788	NM_023363	6.71453e-005	1.80415	E15.5 * Wnt1 up vs E15.5 * Ctrl
10376444	Hist3h2ba	NM_030082	0.000137225	1.80284	E15.5 * Wnt1 up vs E15.5 * Ctrl
10450904	Scoc	NM_001039137	0.000423184	1.80264	E15.5 * Wnt1 up vs E15.5 * Ctrl
10524105	Chfr	NM_172717	2.93524e-008	1.8022	E15.5 * Wnt1 up vs E15.5 * Ctrl
10559681	D430041B17Rik	NM_172737	2.50432e-008	1.80078	E15.5 * Wnt1 up vs E15.5 * Ctrl
10551852	Clip3	NM_001081114	3.35027e-005	1.79967	E15.5 * Wnt1 up vs E15.5 * Ctrl
10426093	Zbed4	NM_181412	2.55227e-008	1.7995	E15.5 * Wnt1 up vs E15.5 * Ctrl
10598138	Spry3	NM_001030293	0.000126473	1.79871	E15.5 * Wnt1 up vs E15.5 * Ctrl
10575291	Zfp821	NM_029468	7.74176e-006	1.79871	E15.5 * Wnt1 up vs E15.5 * Ctrl
10421810	1190002H23Rik	NM_025427	6.08607e-006	1.79824	E15.5 * Wnt1 up vs E15.5 * Ctrl
10388884	Nlk	NM_008702	9.83674e-009	1.79741	E15.5 * Wnt1 up vs E15.5 * Ctrl
10513884	Tle1	NM_011599	2.96888e-010	1.79645	E15.5 * Wnt1 up vs E15.5 * Ctrl
10381939	Tanc2	NM_181071	2.98154e-007	1.79626	E15.5 * Wnt1 up vs E15.5 * Ctrl
10385052	Ranbp17	NM_023146	4.16688e-008	1.79616	E15.5 * Wnt1 up vs E15.5 * Ctrl
10524098	A830023I12Rik	BC007165	0.000266072	1.79357	E15.5 * Wnt1 up vs E15.5 * Ctrl
10349249	Clasp1	NM_001081276	6.43251e-008	1.79348	E15.5 * Wnt1 up vs E15.5 * Ctrl
10526391	2900083I11Rik	NM_021403	4.80163e-007	1.79329	E15.5 * Wnt1 up vs E15.5 * Ctrl
10462918	Tmem20	NM_175507	2.97768e-008	1.79289	E15.5 * Wnt1 up vs E15.5 * Ctrl
10360270	Atp1a2	NM_178405	3.48128e-009	1.79245	E15.5 * Wnt1 up vs E15.5 * Ctrl
10594183	Senp8	NM_027838	0.00183851	1.79241	E15.5 * Wnt1 up vs E15.5 * Ctrl
10363195	Hsf2	NM_008297	4.68036e-007	1.7919	E15.5 * Wnt1 up vs E15.5 * Ctrl
10369877	Ube2d1	NM_145420	2.31128e-008	1.79188	E15.5 * Wnt1 up vs E15.5 * Ctrl
10547719	Clstn3	NM_153508	6.66179e-007	1.79161	E15.5 * Wnt1 up vs E15.5 * Ctrl
10538275	Nfe2l3	NM_010903	1.32428e-007	1.79104	E15.5 * Wnt1 up vs E15.5 * Ctrl
10564053	100040985	XM_001475615	8.54582e-010	1.78989	E15.5 * Wnt1 up vs E15.5 * Ctrl
10564055		---	8.54582e-010	1.78989	E15.5 * Wnt1 up vs E15.5 * Ctrl
10534531	Ywhag	NM_018871	1.4364e-005	1.78916	E15.5 * Wnt1 up vs E15.5 * Ctrl
10426924	Slc4a8	NM_021530	1.06587e-006	1.78787	E15.5 * Wnt1 up vs E15.5 * Ctrl
10454771	Reep2	NM_144865	5.08441e-007	1.78781	E15.5 * Wnt1 up vs E15.5 * Ctrl
10547575	Iqsec3	NM_001033354	1.30253e-006	1.78751	E15.5 * Wnt1 up vs E15.5 * Ctrl
10476814	Insm1	NM_016889	6.98235e-005	1.78706	E15.5 * Wnt1 up vs E15.5 * Ctrl
10563220	Ppfia3	NM_029741	1.47678e-005	1.78577	E15.5 * Wnt1 up vs E15.5 * Ctrl
10602704	Klf8	NM_173780	1.43478e-008	1.78444	E15.5 * Wnt1 up vs E15.5 * Ctrl
10571907	Mfap3l	NM_027756	2.28423e-006	1.78338	E15.5 * Wnt1 up vs E15.5 * Ctrl
10466972	Ranbp6	NM_177721	9.60271e-005	1.78254	E15.5 * Wnt1 up vs E15.5 * Ctrl
10360664	ENSMUSG0000C	ENSMUST00000(	1.60721e-005	1.78253	E15.5 * Wnt1 up vs E15.5 * Ctrl
10562204	Fxyd7	NM_022007	6.52058e-008	1.78163	E15.5 * Wnt1 up vs E15.5 * Ctrl
10505200	Hsdl2	NM_024255	1.73981e-007	1.78134	E15.5 * Wnt1 up vs E15.5 * Ctrl
10394990	Mboat2	NM_026037	1.43108e-006	1.78079	E15.5 * Wnt1 up vs E15.5 * Ctrl
10400157	Nova1	ENSMUST00000(	1.01046e-006	1.78017	E15.5 * Wnt1 up vs E15.5 * Ctrl
10416112	Pnma2	NM_175498	8.52611e-006	1.77966	E15.5 * Wnt1 up vs E15.5 * Ctrl
10357164	Epb4.1l5	NM_145506	1.07017e-009	1.77849	E15.5 * Wnt1 up vs E15.5 * Ctrl
10360463	Pld5	NM_176916	0.000217881	1.77796	E15.5 * Wnt1 up vs E15.5 * Ctrl
10344637	Atp6v1h	NM_133826	8.51275e-009	1.77619	E15.5 * Wnt1 up vs E15.5 * Ctrl
10528227	Gnai1	NM_010305	6.23206e-007	1.7734	E15.5 * Wnt1 up vs E15.5 * Ctrl
10543067	Asns	NM_012055	5.50373e-007	1.77326	E15.5 * Wnt1 up vs E15.5 * Ctrl
10558001	Inpp5f	NM_178641	1.14947e-006	1.77321	E15.5 * Wnt1 up vs E15.5 * Ctrl
10522653	A730089K16Rik	ENSMUST00000(	4.33162e-005	1.7726	E15.5 * Wnt1 up vs E15.5 * Ctrl
10519913	Magi2	NM_015823	6.84682e-005	1.77005	E15.5 * Wnt1 up vs E15.5 * Ctrl
10581914	Chst5	NM_019950	2.39184e-006	1.76785	E15.5 * Wnt1 up vs E15.5 * Ctrl
10363917	EG544705	XR_032177	0.00330128	1.76542	E15.5 * Wnt1 up vs E15.5 * Ctrl
10418766	Ankrd28	NM_001024604	1.51676e-006	1.7648	E15.5 * Wnt1 up vs E15.5 * Ctrl
10352980	Sntg1	NM_027671	1.98778e-006	1.76453	E15.5 * Wnt1 up vs E15.5 * Ctrl
10396712	Fut8	NM_016893	3.85056e-006	1.76407	E15.5 * Wnt1 up vs E15.5 * Ctrl
10564237	ENSMUSG0000C	ENSMUST00000(	7.64445e-005	1.7629	E15.5 * Wnt1 up vs E15.5 * Ctrl

10456289		---	2.24148e-005	1.76203	E15.5 * Wnt1 up vs E15.5 * Ctrl
10365833	Usp44	NM_183199	3.4925e-005	1.76193	E15.5 * Wnt1 up vs E15.5 * Ctrl
10511363	Penk	NM_001002927	0.000356291	1.76192	E15.5 * Wnt1 up vs E15.5 * Ctrl
10532892	1500011B03Rik	ENSMUST00000	2.75416e-009	1.76086	E15.5 * Wnt1 up vs E15.5 * Ctrl
10573128	Tbc1d9	NM_001111304	1.14096e-010	1.76053	E15.5 * Wnt1 up vs E15.5 * Ctrl
10369885	Cisd1	NM_134007	4.39462e-005	1.76022	E15.5 * Wnt1 up vs E15.5 * Ctrl
10597656	Scn5a	NM_021544	9.95263e-006	1.7599	E15.5 * Wnt1 up vs E15.5 * Ctrl
10585699	Fabp5	NM_010634	4.92785e-008	1.75863	E15.5 * Wnt1 up vs E15.5 * Ctrl
10594747	M5C1000I18Rik	AK147236	0.00114989	1.75813	E15.5 * Wnt1 up vs E15.5 * Ctrl
10427908	ENSMUSG0000C	ENSMUST00000(	0.000570314	1.75613	E15.5 * Wnt1 up vs E15.5 * Ctrl
10465089	Snx32	NM_001024560	5.90322e-005	1.75498	E15.5 * Wnt1 up vs E15.5 * Ctrl
10400395	Ppp2r3c	NM_021529	0.000129328	1.75487	E15.5 * Wnt1 up vs E15.5 * Ctrl
10539017	Reep1	NM_178608	5.66458e-006	1.7544	E15.5 * Wnt1 up vs E15.5 * Ctrl
10547521	Atp6v1e1	NM_007510	9.65199e-006	1.75263	E15.5 * Wnt1 up vs E15.5 * Ctrl
10412909	Fdft1	NM_010191	2.17704e-005	1.75223	E15.5 * Wnt1 up vs E15.5 * Ctrl
10484888	Ptprj	NM_008982	2.73727e-005	1.75199	E15.5 * Wnt1 up vs E15.5 * Ctrl
10526261	Stx1a	NM_016801	4.12239e-007	1.7512	E15.5 * Wnt1 up vs E15.5 * Ctrl
10601343	Magee1	NM_053201	0.000375451	1.75099	E15.5 * Wnt1 up vs E15.5 * Ctrl
10460127	Dok6	NM_001039173	7.60475e-007	1.75085	E15.5 * Wnt1 up vs E15.5 * Ctrl
10380660	Hoxb2	NM_134032	1.57971e-006	1.75076	E15.5 * Wnt1 up vs E15.5 * Ctrl
10572146	Atp6v1b2	NM_007509	2.18601e-006	1.75055	E15.5 * Wnt1 up vs E15.5 * Ctrl
10560043	Zfp329	NM_026046	1.996e-006	1.75043	E15.5 * Wnt1 up vs E15.5 * Ctrl
10357345	E030049G20Rik	NM_172484	8.57348e-005	1.75014	E15.5 * Wnt1 up vs E15.5 * Ctrl
10411156	Scamp1	NM_029153	1.06808e-008	1.75008	E15.5 * Wnt1 up vs E15.5 * Ctrl
10532072	Hfm1	BC172061	0.000947373	1.7498	E15.5 * Wnt1 up vs E15.5 * Ctrl
10590844	9030420J04Rik	BC137891	1.93305e-007	1.74829	E15.5 * Wnt1 up vs E15.5 * Ctrl
10360053	Pcp4l1	NM_025557	0.000336187	1.74799	E15.5 * Wnt1 up vs E15.5 * Ctrl
10605465	Prkx	NM_016979	0.000109702	1.74785	E15.5 * Wnt1 up vs E15.5 * Ctrl
10592515	Ubash3b	NM_176860	4.21116e-006	1.74688	E15.5 * Wnt1 up vs E15.5 * Ctrl
10570437	Fbxo25	NM_025785	1.80122e-006	1.74673	E15.5 * Wnt1 up vs E15.5 * Ctrl
10412038	Zswim6	NM_145456	2.19757e-009	1.74664	E15.5 * Wnt1 up vs E15.5 * Ctrl
10465424	Nrxn2	NM_020253	6.9094e-006	1.74602	E15.5 * Wnt1 up vs E15.5 * Ctrl
10418879	Mapk8	NM_016700	8.53653e-008	1.74566	E15.5 * Wnt1 up vs E15.5 * Ctrl
10445241	Tnfrsf21	NM_178589	1.38189e-006	1.74543	E15.5 * Wnt1 up vs E15.5 * Ctrl
10512291	Dctn3	NM_016890	6.44746e-008	1.74441	E15.5 * Wnt1 up vs E15.5 * Ctrl
10522895	Csn3	NM_007786	0.000336825	1.74436	E15.5 * Wnt1 up vs E15.5 * Ctrl
10471880		---	0.00499242	1.74345	E15.5 * Wnt1 up vs E15.5 * Ctrl
10360344	Darc	NM_010045	2.97363e-006	1.74234	E15.5 * Wnt1 up vs E15.5 * Ctrl
10525553		---	0.00279183	1.74188	E15.5 * Wnt1 up vs E15.5 * Ctrl
10399208	Tmem196	ENSMUST00000(	0.0044184	1.74165	E15.5 * Wnt1 up vs E15.5 * Ctrl
10523772	Lrrc8d	NM_178701	8.70451e-007	1.74117	E15.5 * Wnt1 up vs E15.5 * Ctrl
10519886	Sema3c	NM_013657	3.87229e-008	1.74115	E15.5 * Wnt1 up vs E15.5 * Ctrl
10589913	Dync1li1	NM_146229	4.5689e-007	1.74082	E15.5 * Wnt1 up vs E15.5 * Ctrl
10457886	ENSMUSG0000C	ENSMUST00000(	0.000129481	1.74062	E15.5 * Wnt1 up vs E15.5 * Ctrl
10403193	Sp4	NM_009239	1.119e-005	1.74032	E15.5 * Wnt1 up vs E15.5 * Ctrl
10486403	Pla2g4e	NM_177845	2.62497e-005	1.74021	E15.5 * Wnt1 up vs E15.5 * Ctrl
10604906	Ids	NM_010498	1.28755e-006	1.73953	E15.5 * Wnt1 up vs E15.5 * Ctrl
10463164	Frat1	NM_008043	4.58591e-006	1.73937	E15.5 * Wnt1 up vs E15.5 * Ctrl
10350733	Rgs16	NM_011267	0.00139256	1.73873	E15.5 * Wnt1 up vs E15.5 * Ctrl
10594800	B230380D07Rik	NM_172772	0.00144037	1.73826	E15.5 * Wnt1 up vs E15.5 * Ctrl
10362208		---	0.00892822	1.73743	E15.5 * Wnt1 up vs E15.5 * Ctrl
10531544	Paqr3	NM_198422	0.000205518	1.73705	E15.5 * Wnt1 up vs E15.5 * Ctrl
10350975	ENSMUSG0000C	ENSMUST00000(	0.000336262	1.73612	E15.5 * Wnt1 up vs E15.5 * Ctrl
10417599		---	0.0147763	1.73565	E15.5 * Wnt1 up vs E15.5 * Ctrl
10421524		---	0.0147763	1.73565	E15.5 * Wnt1 up vs E15.5 * Ctrl
10436826		---	0.0147763	1.73565	E15.5 * Wnt1 up vs E15.5 * Ctrl
10476658		---	0.0147763	1.73565	E15.5 * Wnt1 up vs E15.5 * Ctrl
10579823		---	0.0147763	1.73565	E15.5 * Wnt1 up vs E15.5 * Ctrl
10582845		---	0.0147763	1.73565	E15.5 * Wnt1 up vs E15.5 * Ctrl
10361358	Rgs17	NM_019958	8.35082e-007	1.73292	E15.5 * Wnt1 up vs E15.5 * Ctrl
10394611	Nbas	ENSMUST00000	2.52385e-007	1.73268	E15.5 * Wnt1 up vs E15.5 * Ctrl
10360764	Enah	NM_010135	5.27936e-009	1.73218	E15.5 * Wnt1 up vs E15.5 * Ctrl
10527158	Fscn1	NM_007984	0.00487262	1.732	E15.5 * Wnt1 up vs E15.5 * Ctrl
10416931	Slitrk5	NM_198865	4.88787e-006	1.73137	E15.5 * Wnt1 up vs E15.5 * Ctrl
10466865	Rfx3	NM_011265	2.9031e-006	1.731	E15.5 * Wnt1 up vs E15.5 * Ctrl
10472418	Scn9a	ENSMUST00000	2.58241e-006	1.73009	E15.5 * Wnt1 up vs E15.5 * Ctrl

10362896	Cd24a	NM_009846	1.3138e-009	1.72928	E15.5 * Wnt1 up vs E15.5 * Ctrl
10399407	Vsnl1	NM_012038	0.000554605	1.72883	E15.5 * Wnt1 up vs E15.5 * Ctrl
10460423	Spnb3	NM_021287	2.61301e-007	1.72799	E15.5 * Wnt1 up vs E15.5 * Ctrl
10457250	Arhgap12	NM_001039692	6.1069e-008	1.72741	E15.5 * Wnt1 up vs E15.5 * Ctrl
10397450	Vash1	NM_177354	3.79331e-006	1.72729	E15.5 * Wnt1 up vs E15.5 * Ctrl
10441091	6530402D11Rik	AK078339	4.41259e-006	1.72726	E15.5 * Wnt1 up vs E15.5 * Ctrl
10460057	Tshz1	NM_001081300	2.36371e-006	1.72679	E15.5 * Wnt1 up vs E15.5 * Ctrl
10396454	Syt16	NM_172804	4.87437e-008	1.72661	E15.5 * Wnt1 up vs E15.5 * Ctrl
10549200	Sox5	NM_011444	1.55593e-005	1.72648	E15.5 * Wnt1 up vs E15.5 * Ctrl
10527516	Wasf3	NM_145155	1.20213e-005	1.726	E15.5 * Wnt1 up vs E15.5 * Ctrl
10606473	Hdx	ENSMUST00000	0.000784988	1.72542	E15.5 * Wnt1 up vs E15.5 * Ctrl
10409660	Gkap1	NM_019832	0.000130326	1.72449	E15.5 * Wnt1 up vs E15.5 * Ctrl
10556463	Arntl	NM_007489	6.91035e-007	1.72411	E15.5 * Wnt1 up vs E15.5 * Ctrl
10578679	ENSMUSG0000C	ENSMUST00000	7.48493e-007	1.72369	E15.5 * Wnt1 up vs E15.5 * Ctrl
10365290	Chst11	NM_021439	2.23328e-005	1.72365	E15.5 * Wnt1 up vs E15.5 * Ctrl
10472764	Dync1i2	NM_010064	5.88582e-009	1.72292	E15.5 * Wnt1 up vs E15.5 * Ctrl
10511819	Klhl32	NM_001033531	3.00393e-006	1.7227	E15.5 * Wnt1 up vs E15.5 * Ctrl
10459552	Spire1	NM_194355	1.58842e-008	1.72146	E15.5 * Wnt1 up vs E15.5 * Ctrl
10594144	Bbs4	NM_175325	4.08345e-005	1.72123	E15.5 * Wnt1 up vs E15.5 * Ctrl
10567022	Btbd10	NM_133700	2.02238e-005	1.7201	E15.5 * Wnt1 up vs E15.5 * Ctrl
10458794	Ccdc112	ENSMUST00000	6.09573e-008	1.71966	E15.5 * Wnt1 up vs E15.5 * Ctrl
10457020		---	0.0076316	1.71908	E15.5 * Wnt1 up vs E15.5 * Ctrl
10417310	666555	ENSMUST00000	2.01856e-005	1.71888	E15.5 * Wnt1 up vs E15.5 * Ctrl
10541049	March8	NM_027920	1.61695e-006	1.71854	E15.5 * Wnt1 up vs E15.5 * Ctrl
10570516	Kbtbd11	NM_029116	5.3378e-008	1.71773	E15.5 * Wnt1 up vs E15.5 * Ctrl
10359419		---	0.000677645	1.71634	E15.5 * Wnt1 up vs E15.5 * Ctrl
10345058	Tmem14a	NM_029398	4.91272e-006	1.71622	E15.5 * Wnt1 up vs E15.5 * Ctrl
10606864	Tceal5	NM_177919	1.87821e-006	1.71621	E15.5 * Wnt1 up vs E15.5 * Ctrl
10372528	Kcnmb4	NM_021452	0.000310021	1.71453	E15.5 * Wnt1 up vs E15.5 * Ctrl
10468992	Frmd4a	NM_172475	4.59631e-007	1.71434	E15.5 * Wnt1 up vs E15.5 * Ctrl
10525487	4932422M17Rik	ENSMUST00000	6.67389e-005	1.71321	E15.5 * Wnt1 up vs E15.5 * Ctrl
10380341	Spag9	NM_027569	8.89252e-009	1.71143	E15.5 * Wnt1 up vs E15.5 * Ctrl
10575630	Cntnap4	NM_130457	1.80257e-005	1.71137	E15.5 * Wnt1 up vs E15.5 * Ctrl
10398727	Klc1	NM_001025360	7.52799e-006	1.71104	E15.5 * Wnt1 up vs E15.5 * Ctrl
10362596	Fyn	NM_001122893	8.10066e-013	1.7109	E15.5 * Wnt1 up vs E15.5 * Ctrl
10356379	Ecel1	NM_021306	2.1467e-006	1.71072	E15.5 * Wnt1 up vs E15.5 * Ctrl
10427303	Hoxc4	NM_013553	1.77095e-005	1.71062	E15.5 * Wnt1 up vs E15.5 * Ctrl
10533869	Ccdc92	NM_144819	1.90284e-005	1.71004	E15.5 * Wnt1 up vs E15.5 * Ctrl
10564624	St8sia2	NM_009181	9.36071e-006	1.70972	E15.5 * Wnt1 up vs E15.5 * Ctrl
10481804	Ralgps1	NM_175211	1.86608e-006	1.70872	E15.5 * Wnt1 up vs E15.5 * Ctrl
10426081	Fam19a5	NM_134096	7.54281e-005	1.70811	E15.5 * Wnt1 up vs E15.5 * Ctrl
10564220	Gkap1	NM_019832	2.94767e-005	1.70809	E15.5 * Wnt1 up vs E15.5 * Ctrl
10515335	RP23-233B9.8	NM_001037916	3.63718e-006	1.70657	E15.5 * Wnt1 up vs E15.5 * Ctrl
10471878		---	0.00136431	1.70552	E15.5 * Wnt1 up vs E15.5 * Ctrl
10558450	ENSMUSG0000C	ENSMUST00000	0.000399799	1.70547	E15.5 * Wnt1 up vs E15.5 * Ctrl
10512915	2810432L12Rik	NM_025944	5.72803e-005	1.70473	E15.5 * Wnt1 up vs E15.5 * Ctrl
10372069	Socs2	NM_007706	1.53781e-005	1.70455	E15.5 * Wnt1 up vs E15.5 * Ctrl
10369210	Serinc1	NM_019760	2.45132e-008	1.7045	E15.5 * Wnt1 up vs E15.5 * Ctrl
10504137	4933409K07Rik	BC072647	8.52654e-005	1.7045	E15.5 * Wnt1 up vs E15.5 * Ctrl
10504201	4933409K07Rik	BC072647	8.52654e-005	1.7045	E15.5 * Wnt1 up vs E15.5 * Ctrl
10512350	4933409K07Rik	BC072647	8.52654e-005	1.7045	E15.5 * Wnt1 up vs E15.5 * Ctrl
10512352	4933409K07Rik	BC072647	8.52654e-005	1.7045	E15.5 * Wnt1 up vs E15.5 * Ctrl
10544751	Hoxa2	NM_010451	2.66226e-005	1.70387	E15.5 * Wnt1 up vs E15.5 * Ctrl
10494945	Syt6	NM_018800	1.27399e-006	1.70378	E15.5 * Wnt1 up vs E15.5 * Ctrl
10469622	Gpr158	NM_001004761	3.11976e-006	1.7023	E15.5 * Wnt1 up vs E15.5 * Ctrl
10592336	Spa17	NM_011449	0.000314452	1.70011	E15.5 * Wnt1 up vs E15.5 * Ctrl
10558248	Bub3	NM_009774	3.50833e-009	1.69987	E15.5 * Wnt1 up vs E15.5 * Ctrl
10370587	Shc2	NM_001024539	0.000431061	1.69951	E15.5 * Wnt1 up vs E15.5 * Ctrl
10464409	Nanos1	NM_178421	2.23166e-008	1.69895	E15.5 * Wnt1 up vs E15.5 * Ctrl
10463704	As3mt	NM_020577	9.35362e-008	1.69751	E15.5 * Wnt1 up vs E15.5 * Ctrl
10590445	Snrk	NM_133741	1.19732e-006	1.69732	E15.5 * Wnt1 up vs E15.5 * Ctrl
10413398	Il17rd	NM_134437	7.58495e-006	1.69637	E15.5 * Wnt1 up vs E15.5 * Ctrl
10429295	Kcnk9	NM_001033876	4.61722e-006	1.69615	E15.5 * Wnt1 up vs E15.5 * Ctrl
10594802	B230380D07Rik	NM_172772	2.48686e-005	1.69555	E15.5 * Wnt1 up vs E15.5 * Ctrl
10495869	Tram1l1	NM_146140	4.80121e-006	1.69548	E15.5 * Wnt1 up vs E15.5 * Ctrl
10563975		---	2.48399e-008	1.69515	E15.5 * Wnt1 up vs E15.5 * Ctrl

10563981		---	2.48399e-008	1.69515	E15.5 * Wnt1 up vs E15.5 * Ctrl
10563987		---	2.48399e-008	1.69515	E15.5 * Wnt1 up vs E15.5 * Ctrl
10564009		---	2.48399e-008	1.69515	E15.5 * Wnt1 up vs E15.5 * Ctrl
10379654	Ap2b1	NM_001035854	3.12793e-009	1.69438	E15.5 * Wnt1 up vs E15.5 * Ctrl
10588509	Pcbp4	NM_021567	0.00203967	1.69398	E15.5 * Wnt1 up vs E15.5 * Ctrl
10439881	5330426P16Rik	ENSMUST00000(	7.7838e-006	1.69364	E15.5 * Wnt1 up vs E15.5 * Ctrl
10583847	Bbs9	NM_178415	6.18363e-007	1.69309	E15.5 * Wnt1 up vs E15.5 * Ctrl
10462613	Ifit2	NM_008332	3.32416e-007	1.69229	E15.5 * Wnt1 up vs E15.5 * Ctrl
10364502	Palm	NM_023128	4.28213e-005	1.69071	E15.5 * Wnt1 up vs E15.5 * Ctrl
10455227	Rnf14	NM_020012	1.37206e-009	1.69026	E15.5 * Wnt1 up vs E15.5 * Ctrl
10496262	Nhedc2	NM_178877	6.6238e-005	1.68999	E15.5 * Wnt1 up vs E15.5 * Ctrl
10514779	Prkaa2	NM_178143	1.29203e-005	1.68931	E15.5 * Wnt1 up vs E15.5 * Ctrl
10362717	Wasf1	NM_031877	8.05079e-009	1.68864	E15.5 * Wnt1 up vs E15.5 * Ctrl
10459671	Dcc	NM_007831	3.45649e-007	1.6886	E15.5 * Wnt1 up vs E15.5 * Ctrl
10592850	Trappc4	NM_021789	2.17793e-005	1.68836	E15.5 * Wnt1 up vs E15.5 * Ctrl
10483626	Dlx2	NM_010054	3.606e-006	1.68767	E15.5 * Wnt1 up vs E15.5 * Ctrl
10543219	Gpr85	NM_145066	1.18936e-006	1.68718	E15.5 * Wnt1 up vs E15.5 * Ctrl
10465342	Tm7sf2	NM_028454	4.0771e-006	1.68676	E15.5 * Wnt1 up vs E15.5 * Ctrl
10570450	Dlgap2	NM_172910	6.80558e-006	1.68596	E15.5 * Wnt1 up vs E15.5 * Ctrl
10469720	Acbd5	NM_001102437	4.01204e-006	1.68558	E15.5 * Wnt1 up vs E15.5 * Ctrl
10404763	Tmem170b	XM_886379	3.16877e-006	1.68296	E15.5 * Wnt1 up vs E15.5 * Ctrl
10559964	Zik1	NM_009577	5.1952e-005	1.68261	E15.5 * Wnt1 up vs E15.5 * Ctrl
10550925	Zfp428	NM_146183	5.34123e-009	1.68214	E15.5 * Wnt1 up vs E15.5 * Ctrl
10580534		---	2.33712e-007	1.68189	E15.5 * Wnt1 up vs E15.5 * Ctrl
10485633	ENSMUSG00000(	ENSMUST00000(	0.00814703	1.68146	E15.5 * Wnt1 up vs E15.5 * Ctrl
10404848	Jarid2	NM_021878	1.16099e-009	1.68084	E15.5 * Wnt1 up vs E15.5 * Ctrl
10576010	Gse1	NM_198671	2.68238e-005	1.6797	E15.5 * Wnt1 up vs E15.5 * Ctrl
10544406	Fam131b	NM_029528	4.58044e-006	1.67906	E15.5 * Wnt1 up vs E15.5 * Ctrl
10606893	Rab9b	NM_176971	3.25309e-005	1.67903	E15.5 * Wnt1 up vs E15.5 * Ctrl
10533323	Adam1a	NM_172126	0.000116006	1.67897	E15.5 * Wnt1 up vs E15.5 * Ctrl
10417498	666555	ENSMUST00000	0.000567914	1.67891	E15.5 * Wnt1 up vs E15.5 * Ctrl
10376033	Kif3a	NM_008443	3.858e-009	1.67836	E15.5 * Wnt1 up vs E15.5 * Ctrl
10420785	Mtmr9	ENSMUST00000(	0.00062054	1.6783	E15.5 * Wnt1 up vs E15.5 * Ctrl
10604053	Nkrf	NM_029891	3.92487e-006	1.67794	E15.5 * Wnt1 up vs E15.5 * Ctrl
10589784	Dclk3	NM_172928	2.3655e-006	1.67782	E15.5 * Wnt1 up vs E15.5 * Ctrl
10424411	Tsg101	NM_021884	5.91919e-008	1.67766	E15.5 * Wnt1 up vs E15.5 * Ctrl
10590487	Zfp660	XR_031805	0.00411177	1.67744	E15.5 * Wnt1 up vs E15.5 * Ctrl
10373740	Pik3ip1	NM_178149	6.25112e-006	1.67733	E15.5 * Wnt1 up vs E15.5 * Ctrl
10535866	Ubl3	NM_011908	1.12911e-008	1.67699	E15.5 * Wnt1 up vs E15.5 * Ctrl
10595924	Pik3cb	NM_029094	0.000138231	1.67688	E15.5 * Wnt1 up vs E15.5 * Ctrl
10396952	Ttc9	NM_001033149	5.15747e-005	1.67611	E15.5 * Wnt1 up vs E15.5 * Ctrl
10575777	4933407C03Rik	BC158118	9.08755e-006	1.67594	E15.5 * Wnt1 up vs E15.5 * Ctrl
10603768	Efhc2	NM_028916	1.3195e-005	1.67573	E15.5 * Wnt1 up vs E15.5 * Ctrl
10439239	Dirc2	NM_153550	5.67653e-006	1.6756	E15.5 * Wnt1 up vs E15.5 * Ctrl
10607089	Acsl4	NM_207625	5.59448e-006	1.6756	E15.5 * Wnt1 up vs E15.5 * Ctrl
10571745	Cldn22	NM_029383	8.26028e-007	1.67555	E15.5 * Wnt1 up vs E15.5 * Ctrl
10518763	Slc45a1	NM_173774	8.26832e-006	1.67537	E15.5 * Wnt1 up vs E15.5 * Ctrl
10516544	Hpca	NM_010471	3.06129e-005	1.67414	E15.5 * Wnt1 up vs E15.5 * Ctrl
10592471	Gramd1b	NM_172768	1.74811e-005	1.67359	E15.5 * Wnt1 up vs E15.5 * Ctrl
10436442	Fam60a	NM_019643	8.13501e-009	1.67351	E15.5 * Wnt1 up vs E15.5 * Ctrl
10596940	P4htm	NM_028944	1.16739e-006	1.67286	E15.5 * Wnt1 up vs E15.5 * Ctrl
10472376	Scn2a1	NM_001099298	4.19426e-005	1.67152	E15.5 * Wnt1 up vs E15.5 * Ctrl
10448865	Gnptg	NM_172529	2.08551e-008	1.67103	E15.5 * Wnt1 up vs E15.5 * Ctrl
10409602	Trpc7	NM_012035	1.52354e-005	1.6707	E15.5 * Wnt1 up vs E15.5 * Ctrl
10549497	3010003L21Rik	BC106181	2.67527e-009	1.67061	E15.5 * Wnt1 up vs E15.5 * Ctrl
10354207	Creg2	NM_170597	8.1517e-005	1.67049	E15.5 * Wnt1 up vs E15.5 * Ctrl
10595979	Mras	NM_008624	0.00212338	1.6704	E15.5 * Wnt1 up vs E15.5 * Ctrl
10363599	Rufy2	NM_027425	6.71019e-007	1.67029	E15.5 * Wnt1 up vs E15.5 * Ctrl
10551417	Zfp59	NM_011762	1.17143e-010	1.66933	E15.5 * Wnt1 up vs E15.5 * Ctrl
10477103	Nrsn2	NM_001009948	0.000676804	1.66847	E15.5 * Wnt1 up vs E15.5 * Ctrl
10605566		---	0.000356747	1.66748	E15.5 * Wnt1 up vs E15.5 * Ctrl
10524684	Msi1	NM_008629	0.000550516	1.66706	E15.5 * Wnt1 up vs E15.5 * Ctrl
10475324	Ckmt1	NM_009897	0.00162339	1.66677	E15.5 * Wnt1 up vs E15.5 * Ctrl
10508376	Trim62	NM_178110	1.45344e-008	1.66675	E15.5 * Wnt1 up vs E15.5 * Ctrl
10395805	1700047I17Rik1	BC049669	1.65088e-006	1.66651	E15.5 * Wnt1 up vs E15.5 * Ctrl
10516241	Maneal	NM_001007573	1.16113e-005	1.66602	E15.5 * Wnt1 up vs E15.5 * Ctrl

10554166	Akap13	NM_029332	2.37029e-008	1.66576	E15.5 * Wnt1 up vs E15.5 * Ctrl
10569830	2310057J16Rik	NM_027171	6.50542e-006	1.66517	E15.5 * Wnt1 up vs E15.5 * Ctrl
10469609	OTTMUSG00000	ENSMUST00000	0.0113043	1.66424	E15.5 * Wnt1 up vs E15.5 * Ctrl
10434446	Ece2	NM_139293	2.62597e-006	1.66421	E15.5 * Wnt1 up vs E15.5 * Ctrl
10416110	EG432870	NM_001034881	0.000563953	1.66399	E15.5 * Wnt1 up vs E15.5 * Ctrl
10557106	Hs3st2	NM_001081327	2.90456e-005	1.66394	E15.5 * Wnt1 up vs E15.5 * Ctrl
10590983	Panx1	NM_019482	2.84739e-006	1.66334	E15.5 * Wnt1 up vs E15.5 * Ctrl
10498076	Maml3	NM_001004176	2.51933e-006	1.66089	E15.5 * Wnt1 up vs E15.5 * Ctrl
10543118	Glcci1	NM_133236	1.72794e-006	1.66087	E15.5 * Wnt1 up vs E15.5 * Ctrl
10595094	2310046A06Rik	BC089626	0.00071933	1.65967	E15.5 * Wnt1 up vs E15.5 * Ctrl
10407124	AI452195	ENSMUST00000	0.00567861	1.65856	E15.5 * Wnt1 up vs E15.5 * Ctrl
10563909		---	2.61284e-008	1.65822	E15.5 * Wnt1 up vs E15.5 * Ctrl
10577996	Unc5d	NM_153135	0.000109094	1.65805	E15.5 * Wnt1 up vs E15.5 * Ctrl
10442396	Abca3	NM_013855	2.46936e-007	1.65779	E15.5 * Wnt1 up vs E15.5 * Ctrl
10433445	Abat	NM_172961	0.000416653	1.65656	E15.5 * Wnt1 up vs E15.5 * Ctrl
10479726	Pcmtd2	NM_153594	3.94755e-007	1.65654	E15.5 * Wnt1 up vs E15.5 * Ctrl
10575775	4933407C03Rik	BC158118	4.22824e-006	1.65622	E15.5 * Wnt1 up vs E15.5 * Ctrl
10452793	Galnt14	NM_027864	1.8191e-005	1.65614	E15.5 * Wnt1 up vs E15.5 * Ctrl
10366725	Fam19a2	NM_182807	1.58203e-007	1.65609	E15.5 * Wnt1 up vs E15.5 * Ctrl
10483604	Slc25a12	NM_172436	7.03434e-006	1.65593	E15.5 * Wnt1 up vs E15.5 * Ctrl
10447480	Nrxn1	NM_020252	3.62997e-005	1.65544	E15.5 * Wnt1 up vs E15.5 * Ctrl
10503259	Trp53inp1	NM_021897	1.51207e-006	1.65477	E15.5 * Wnt1 up vs E15.5 * Ctrl
10605797	Arhgef9	NM_001033329	1.66958e-007	1.65451	E15.5 * Wnt1 up vs E15.5 * Ctrl
10395961	Lrfn5	NM_178714	2.93866e-005	1.65442	E15.5 * Wnt1 up vs E15.5 * Ctrl
10535021	Gpc2	NM_172412	1.95779e-005	1.65415	E15.5 * Wnt1 up vs E15.5 * Ctrl
10490061	Bcas1	NM_029815	5.75001e-007	1.65371	E15.5 * Wnt1 up vs E15.5 * Ctrl
10585347	4930550C14Rik	NM_029247	0.00030841	1.65356	E15.5 * Wnt1 up vs E15.5 * Ctrl
10452404	Nudt12	NM_026497	1.77191e-005	1.65322	E15.5 * Wnt1 up vs E15.5 * Ctrl
10558773	B4galnt4	NM_177897	3.00118e-005	1.65236	E15.5 * Wnt1 up vs E15.5 * Ctrl
10565900	D630004N19Rik	BC096037	0.00644802	1.65227	E15.5 * Wnt1 up vs E15.5 * Ctrl
10352926		---	0.00313385	1.65129	E15.5 * Wnt1 up vs E15.5 * Ctrl
10383564	Fn3k	NM_001038699	2.43306e-006	1.65035	E15.5 * Wnt1 up vs E15.5 * Ctrl
10505073	Zfp462	NM_172867	6.52144e-008	1.65001	E15.5 * Wnt1 up vs E15.5 * Ctrl
10409345	Cltb	NM_028870	5.60943e-005	1.64988	E15.5 * Wnt1 up vs E15.5 * Ctrl
10403716	AW209491	NM_134067	2.5798e-006	1.64936	E15.5 * Wnt1 up vs E15.5 * Ctrl
10594501	Ptplad1	NM_021345	1.66218e-005	1.64909	E15.5 * Wnt1 up vs E15.5 * Ctrl
10550274	Meis3	NM_008627	3.81431e-007	1.64889	E15.5 * Wnt1 up vs E15.5 * Ctrl
10367973	Aig1	NM_025446	0.00022862	1.64774	E15.5 * Wnt1 up vs E15.5 * Ctrl
10519420	Akap9	NM_194462	2.37225e-008	1.64697	E15.5 * Wnt1 up vs E15.5 * Ctrl
10474411	Lin7c	NM_011699	4.65653e-011	1.64645	E15.5 * Wnt1 up vs E15.5 * Ctrl
10585146	Tmprss5	NM_030709	9.03603e-005	1.64537	E15.5 * Wnt1 up vs E15.5 * Ctrl
10534021	Rimbp2	NM_001081388	4.17491e-009	1.64535	E15.5 * Wnt1 up vs E15.5 * Ctrl
10389738	Dgke	NM_019505	2.68148e-006	1.64395	E15.5 * Wnt1 up vs E15.5 * Ctrl
10399234	Efr3b	NM_001082483	1.46519e-006	1.64369	E15.5 * Wnt1 up vs E15.5 * Ctrl
10446066	A230051N06Rik	ENSMUST00000	3.37025e-005	1.64326	E15.5 * Wnt1 up vs E15.5 * Ctrl
10457022	Mbp	NM_010777	1.79464e-006	1.643	E15.5 * Wnt1 up vs E15.5 * Ctrl
10603492	Porcn	NM_016913	1.75226e-007	1.64207	E15.5 * Wnt1 up vs E15.5 * Ctrl
10360506	Akt3	NM_011785	2.87958e-008	1.64166	E15.5 * Wnt1 up vs E15.5 * Ctrl
10426955	Scn8a	NM_001077499	5.15652e-006	1.641	E15.5 * Wnt1 up vs E15.5 * Ctrl
10440314	Cadm2	NM_178721	4.94858e-006	1.64085	E15.5 * Wnt1 up vs E15.5 * Ctrl
10345401	Fam123c	BC070435	6.91613e-005	1.64029	E15.5 * Wnt1 up vs E15.5 * Ctrl
10460987	Nrxn2	NM_020253	7.52308e-006	1.63984	E15.5 * Wnt1 up vs E15.5 * Ctrl
10395770	1700047I17Rik1	BC048158	7.32462e-007	1.63953	E15.5 * Wnt1 up vs E15.5 * Ctrl
10395780	1700047I17Rik1	BC048158	7.32462e-007	1.63953	E15.5 * Wnt1 up vs E15.5 * Ctrl
10419399	Slc35f4	NM_029238	9.24421e-005	1.63919	E15.5 * Wnt1 up vs E15.5 * Ctrl
10451054	Enpp4	NM_199016	4.68238e-005	1.63887	E15.5 * Wnt1 up vs E15.5 * Ctrl
10432404	Tuba1a	NM_011653	1.40038e-007	1.63841	E15.5 * Wnt1 up vs E15.5 * Ctrl
10491212	6130401L20Rik	BC117810	0.00034584	1.63794	E15.5 * Wnt1 up vs E15.5 * Ctrl
10531653	BC062109	BC055321	0.000535238	1.63722	E15.5 * Wnt1 up vs E15.5 * Ctrl
10497309	Snx16	NM_029068	0.00213408	1.63641	E15.5 * Wnt1 up vs E15.5 * Ctrl
10359235	Rasal2	NM_177644	1.23404e-008	1.63619	E15.5 * Wnt1 up vs E15.5 * Ctrl
10564839	Ap3s2	NM_009682	5.20442e-005	1.63581	E15.5 * Wnt1 up vs E15.5 * Ctrl
10520392	Rnf32	NM_021470	0.000406262	1.63569	E15.5 * Wnt1 up vs E15.5 * Ctrl
10420730	Fdft1	NM_010191	1.29433e-006	1.63542	E15.5 * Wnt1 up vs E15.5 * Ctrl
10538408	2410066E13Rik	BC042507	2.69996e-006	1.63488	E15.5 * Wnt1 up vs E15.5 * Ctrl
10588876	Nicn1	NM_025449	0.000318774	1.63463	E15.5 * Wnt1 up vs E15.5 * Ctrl

10389339	Usp32	NM_001029934	3.63513e-005	1.63448	E15.5 * Wnt1 up vs E15.5 * Ctrl
10581573		---	3.57397e-005	1.63441	E15.5 * Wnt1 up vs E15.5 * Ctrl
10467216	Cpeb3	NM_198300	2.03339e-006	1.63361	E15.5 * Wnt1 up vs E15.5 * Ctrl
10431410	Mapk11	NM_011161	2.5387e-005	1.6336	E15.5 * Wnt1 up vs E15.5 * Ctrl
10594631	Aph1b	NM_177583	1.97042e-006	1.63215	E15.5 * Wnt1 up vs E15.5 * Ctrl
10522661	C530008M17Rik	AK122469	1.49261e-005	1.63061	E15.5 * Wnt1 up vs E15.5 * Ctrl
10412036	Apoo-ps	NR_004438	7.1196e-005	1.63056	E15.5 * Wnt1 up vs E15.5 * Ctrl
10587604	Rwdd2a	NM_027100	3.7445e-005	1.62991	E15.5 * Wnt1 up vs E15.5 * Ctrl
10414802	Trav16d	ENSMUST00000	0.00316715	1.62936	E15.5 * Wnt1 up vs E15.5 * Ctrl
10532542	Sez6l	NM_019982	5.99515e-006	1.62917	E15.5 * Wnt1 up vs E15.5 * Ctrl
10488020	Tmx4	NM_029148	0.000849736	1.62847	E15.5 * Wnt1 up vs E15.5 * Ctrl
10606315	Taf9b	NM_001001176	0.000708513	1.62713	E15.5 * Wnt1 up vs E15.5 * Ctrl
10380654	Hoxb3	NM_001079869	0.000241308	1.62709	E15.5 * Wnt1 up vs E15.5 * Ctrl
10364601	Abca7	NM_013850	4.17597e-007	1.62646	E15.5 * Wnt1 up vs E15.5 * Ctrl
10395889	Sstr1	NM_009216	0.000170242	1.62613	E15.5 * Wnt1 up vs E15.5 * Ctrl
10546884	Lhfpl4	NM_177763	7.79709e-006	1.62599	E15.5 * Wnt1 up vs E15.5 * Ctrl
10600524	Vbp1	NM_011692	0.000127184	1.62536	E15.5 * Wnt1 up vs E15.5 * Ctrl
10431147	Ldoc1l	NM_177630	3.90278e-005	1.62492	E15.5 * Wnt1 up vs E15.5 * Ctrl
10517312	Tmem57	NM_025382	5.02929e-007	1.62466	E15.5 * Wnt1 up vs E15.5 * Ctrl
10477649	Acss2	NM_019811	3.71304e-006	1.62465	E15.5 * Wnt1 up vs E15.5 * Ctrl
10352954	Hmgb3	NM_008253	1.19732e-009	1.62462	E15.5 * Wnt1 up vs E15.5 * Ctrl
10456206	Wdr7	NM_001014981	9.20671e-008	1.62381	E15.5 * Wnt1 up vs E15.5 * Ctrl
10486201	OTTMUSG00000	ENSMUST00000	0.00132214	1.62358	E15.5 * Wnt1 up vs E15.5 * Ctrl
10520109		---	0.00331041	1.62314	E15.5 * Wnt1 up vs E15.5 * Ctrl
10492516	Iqcj	NM_177585	2.9561e-006	1.6231	E15.5 * Wnt1 up vs E15.5 * Ctrl
10438813		---	0.00325332	1.62274	E15.5 * Wnt1 up vs E15.5 * Ctrl
10565152	9330120H11Rik	ENSMUST00000	0.000155116	1.62247	E15.5 * Wnt1 up vs E15.5 * Ctrl
10385203	Odz2	NM_011856	2.07661e-005	1.62219	E15.5 * Wnt1 up vs E15.5 * Ctrl
10453373	Prepl	NM_145984	1.20074e-005	1.62212	E15.5 * Wnt1 up vs E15.5 * Ctrl
10504902	Murc	NM_026509	1.75784e-005	1.62133	E15.5 * Wnt1 up vs E15.5 * Ctrl
10388492	Vps53	NM_026664	1.11432e-008	1.62102	E15.5 * Wnt1 up vs E15.5 * Ctrl
10442270	1300003B13Rik	BC025651	0.000118838	1.62029	E15.5 * Wnt1 up vs E15.5 * Ctrl
10549222	Bcat1	NM_001024468	0.00011359	1.61912	E15.5 * Wnt1 up vs E15.5 * Ctrl
10376216	Slc36a1	NM_153139	0.000361495	1.61909	E15.5 * Wnt1 up vs E15.5 * Ctrl
10438815	1600021P15Rik	NM_177718	1.97197e-006	1.61869	E15.5 * Wnt1 up vs E15.5 * Ctrl
10490724	Samd10	NM_172676	2.98806e-006	1.61833	E15.5 * Wnt1 up vs E15.5 * Ctrl
10363905	Zwint	NM_025635	1.56563e-008	1.61709	E15.5 * Wnt1 up vs E15.5 * Ctrl
10418991	Gcap14	NM_027045	2.32364e-007	1.61678	E15.5 * Wnt1 up vs E15.5 * Ctrl
10392642	Abca5	NM_147219	7.25181e-005	1.61675	E15.5 * Wnt1 up vs E15.5 * Ctrl
10562125	Ffar3	NM_001033316	2.46574e-005	1.61559	E15.5 * Wnt1 up vs E15.5 * Ctrl
10379998	Trim37	NM_197987	6.2416e-008	1.61553	E15.5 * Wnt1 up vs E15.5 * Ctrl
10442231		---	0.000758667	1.61481	E15.5 * Wnt1 up vs E15.5 * Ctrl
10586971	Prtg	NM_175485	0.000110834	1.61477	E15.5 * Wnt1 up vs E15.5 * Ctrl
10546476	Magi1	NM_001029850	4.48562e-007	1.61469	E15.5 * Wnt1 up vs E15.5 * Ctrl
10388648	Ankrd13b	NM_172945	2.57802e-005	1.61373	E15.5 * Wnt1 up vs E15.5 * Ctrl
10495193	Kcna2	NM_008417	1.9427e-005	1.61351	E15.5 * Wnt1 up vs E15.5 * Ctrl
10386909	Cenpv	NM_028448	4.67834e-005	1.61315	E15.5 * Wnt1 up vs E15.5 * Ctrl
10467599	Slit1	NM_015748	7.83522e-005	1.61289	E15.5 * Wnt1 up vs E15.5 * Ctrl
10456836	St8sia5	NM_153124	1.43102e-005	1.61286	E15.5 * Wnt1 up vs E15.5 * Ctrl
10362171	Stx7	NM_016797	8.06919e-007	1.61122	E15.5 * Wnt1 up vs E15.5 * Ctrl
10471967	Mbd5	NM_029924	1.61698e-005	1.61108	E15.5 * Wnt1 up vs E15.5 * Ctrl
10417403	666555	ENSMUST00000	2.64294e-005	1.60978	E15.5 * Wnt1 up vs E15.5 * Ctrl
10599654	Cxx1c	NM_028375	0.000160066	1.60966	E15.5 * Wnt1 up vs E15.5 * Ctrl
10497505	Nlgn1	NM_138666	0.000164585	1.60958	E15.5 * Wnt1 up vs E15.5 * Ctrl
10372583	Rab3ip	NM_001003950	5.96056e-008	1.60954	E15.5 * Wnt1 up vs E15.5 * Ctrl
10525236	AU042671	BC051044	6.74365e-006	1.60752	E15.5 * Wnt1 up vs E15.5 * Ctrl
10549842	Zfp667	NM_001024928	5.83327e-005	1.60719	E15.5 * Wnt1 up vs E15.5 * Ctrl
10472370	Scn2a1	NM_001099298	0.000360211	1.60716	E15.5 * Wnt1 up vs E15.5 * Ctrl
10594221	Lrrc49	NM_145616	1.64082e-006	1.60685	E15.5 * Wnt1 up vs E15.5 * Ctrl
10563899		---	4.51244e-010	1.60628	E15.5 * Wnt1 up vs E15.5 * Ctrl
10608288		---	2.95067e-006	1.60616	E15.5 * Wnt1 up vs E15.5 * Ctrl
10608300		---	2.95067e-006	1.60616	E15.5 * Wnt1 up vs E15.5 * Ctrl
10407337	Hcn1	NM_010408	0.0130929	1.60601	E15.5 * Wnt1 up vs E15.5 * Ctrl
10461237	Bscl2	NM_001136064	1.34052e-006	1.6058	E15.5 * Wnt1 up vs E15.5 * Ctrl
10568948	Sprn	NM_183147	2.38407e-005	1.60522	E15.5 * Wnt1 up vs E15.5 * Ctrl
10561777	BC027344	BC027344	0.000652625	1.60507	E15.5 * Wnt1 up vs E15.5 * Ctrl

10373388	Obfc2b	NM_027257	0.000101974	1.60478	E15.5 * Wnt1 up vs E15.5 * Ctrl
10377215	Gas7	NM_008088	2.72054e-007	1.60465	E15.5 * Wnt1 up vs E15.5 * Ctrl
10557481	Ypel3	NM_026875	6.14477e-007	1.6042	E15.5 * Wnt1 up vs E15.5 * Ctrl
10535053	Prkar1b	NM_008923	7.03318e-006	1.6034	E15.5 * Wnt1 up vs E15.5 * Ctrl
10451303	Ttbk1	ENSMUST00000(	0.000148267	1.60304	E15.5 * Wnt1 up vs E15.5 * Ctrl
10421709	EG629678	NM_001037935	2.17562e-005	1.60304	E15.5 * Wnt1 up vs E15.5 * Ctrl
10570388	Fam70b	NM_001143671	2.51951e-006	1.60278	E15.5 * Wnt1 up vs E15.5 * Ctrl
10565089	Cpeb1	NM_007755	4.80168e-006	1.6018	E15.5 * Wnt1 up vs E15.5 * Ctrl
10565530		---	0.00119462	1.60134	E15.5 * Wnt1 up vs E15.5 * Ctrl
10475027	Tyro3	NM_019392	3.62785e-008	1.60101	E15.5 * Wnt1 up vs E15.5 * Ctrl
10436692	ENSMUSG0000C	ENSMUST00000(	0.00100923	1.6001	E15.5 * Wnt1 up vs E15.5 * Ctrl
10593293	Ncam1	NM_001081445	1.9031e-005	1.59971	E15.5 * Wnt1 up vs E15.5 * Ctrl
10408762	Eef1e1	NM_025380	0.00168316	1.59881	E15.5 * Wnt1 up vs E15.5 * Ctrl
10357220	Tmem177	NM_175106	0.00081737	1.59856	E15.5 * Wnt1 up vs E15.5 * Ctrl
10437590	Carhsp1	NM_025821	9.88668e-008	1.59812	E15.5 * Wnt1 up vs E15.5 * Ctrl
10529957	Gpr125	ENSMUST00000(	1.20088e-006	1.59762	E15.5 * Wnt1 up vs E15.5 * Ctrl
10425267	Pick1	NM_008837	2.19221e-005	1.59674	E15.5 * Wnt1 up vs E15.5 * Ctrl
10545780	Exoc6b	NM_177077	8.69708e-007	1.59649	E15.5 * Wnt1 up vs E15.5 * Ctrl
10478943	Pfdn4	NM_001013369	0.0015255	1.59578	E15.5 * Wnt1 up vs E15.5 * Ctrl
10385656	Zfp2	NM_001044697	2.95971e-005	1.59512	E15.5 * Wnt1 up vs E15.5 * Ctrl
10523297	Ccng2	NM_007635	1.40963e-005	1.59476	E15.5 * Wnt1 up vs E15.5 * Ctrl
10408610	Tubb2a	NM_009450	8.73849e-007	1.59405	E15.5 * Wnt1 up vs E15.5 * Ctrl
10446739	Clip4	NM_030179	4.71924e-005	1.5936	E15.5 * Wnt1 up vs E15.5 * Ctrl
10447490	Pja2	NM_001025309	1.70077e-008	1.59318	E15.5 * Wnt1 up vs E15.5 * Ctrl
10400803		---	0.00867209	1.59302	E15.5 * Wnt1 up vs E15.5 * Ctrl
10447354	Txndc14	NM_025868	1.83993e-005	1.593	E15.5 * Wnt1 up vs E15.5 * Ctrl
10345715	Map4k4	NM_008696	4.52219e-007	1.5927	E15.5 * Wnt1 up vs E15.5 * Ctrl
10455054	Pcdhb3	NM_053128	0.00122786	1.59254	E15.5 * Wnt1 up vs E15.5 * Ctrl
10392415	Rgs9	NM_011268	0.000140317	1.59158	E15.5 * Wnt1 up vs E15.5 * Ctrl
10538732	Grid2	NM_008167	0.000248128	1.59157	E15.5 * Wnt1 up vs E15.5 * Ctrl
10553450	Nell1	NM_001037906	0.00055	1.59098	E15.5 * Wnt1 up vs E15.5 * Ctrl
10386388	Snap47	NM_144521	1.45336e-005	1.59085	E15.5 * Wnt1 up vs E15.5 * Ctrl
10368918	Sobp	NM_175407	2.05796e-006	1.59075	E15.5 * Wnt1 up vs E15.5 * Ctrl
10594315	Fem1b	NM_010193	2.36724e-010	1.58943	E15.5 * Wnt1 up vs E15.5 * Ctrl
10563897		---	0.000303465	1.58926	E15.5 * Wnt1 up vs E15.5 * Ctrl
10493377		---	4.86867e-005	1.58842	E15.5 * Wnt1 up vs E15.5 * Ctrl
10441565	Rps6ka2	NM_011299	1.51513e-005	1.58727	E15.5 * Wnt1 up vs E15.5 * Ctrl
10363962	Gnaz	NM_010311	6.49535e-005	1.58719	E15.5 * Wnt1 up vs E15.5 * Ctrl
10425012	Zfp7	NM_145916	0.000122065	1.58647	E15.5 * Wnt1 up vs E15.5 * Ctrl
10438358	05-Sep	NM_213614	8.78387e-008	1.58639	E15.5 * Wnt1 up vs E15.5 * Ctrl
10503835	Rragd	NM_027491	2.25994e-005	1.58624	E15.5 * Wnt1 up vs E15.5 * Ctrl
10429203	Fam135b	NM_177819	0.000315202	1.5858	E15.5 * Wnt1 up vs E15.5 * Ctrl
10429220	Fam135b	NM_177819	0.00305403	1.58579	E15.5 * Wnt1 up vs E15.5 * Ctrl
10569280	Dusp8	NM_008748	0.00011093	1.58553	E15.5 * Wnt1 up vs E15.5 * Ctrl
10505922		---	3.18299e-005	1.58489	E15.5 * Wnt1 up vs E15.5 * Ctrl
10565292	Arnt2	NM_007488	5.09056e-005	1.58429	E15.5 * Wnt1 up vs E15.5 * Ctrl
10421648	Slc25a30	NM_026232	2.70279e-006	1.58317	E15.5 * Wnt1 up vs E15.5 * Ctrl
10399505	Greb1	NM_015764	0.000163613	1.58299	E15.5 * Wnt1 up vs E15.5 * Ctrl
10399973	Hdac9	NM_024124	1.93373e-006	1.58286	E15.5 * Wnt1 up vs E15.5 * Ctrl
10534966	Zfp113	NM_019747	4.04374e-006	1.58245	E15.5 * Wnt1 up vs E15.5 * Ctrl
10555414	Rab6	NM_024287	2.00464e-006	1.58212	E15.5 * Wnt1 up vs E15.5 * Ctrl
10372005	Vezt	NM_172538	3.6408e-005	1.58207	E15.5 * Wnt1 up vs E15.5 * Ctrl
10472162	Gpd2	NM_010274	1.45027e-005	1.58204	E15.5 * Wnt1 up vs E15.5 * Ctrl
10408024	Pgbd1	BC089360	6.95765e-005	1.58178	E15.5 * Wnt1 up vs E15.5 * Ctrl
10599537	Slc25a14	NM_011398	0.000590158	1.58101	E15.5 * Wnt1 up vs E15.5 * Ctrl
10504534	Frmpd1	NM_001081172	0.000313834	1.58059	E15.5 * Wnt1 up vs E15.5 * Ctrl
10503643	Ndufaf4	NM_026742	3.33399e-007	1.58006	E15.5 * Wnt1 up vs E15.5 * Ctrl
10484894	Ptprj	NM_008982	0.000120949	1.57986	E15.5 * Wnt1 up vs E15.5 * Ctrl
10388310	Garnl4	NM_001015046	0.000121984	1.57962	E15.5 * Wnt1 up vs E15.5 * Ctrl
10578572	Stox2	NM_001114311	3.53645e-005	1.57938	E15.5 * Wnt1 up vs E15.5 * Ctrl
10433077	Smug1	NM_027885	0.000131189	1.57832	E15.5 * Wnt1 up vs E15.5 * Ctrl
10460157	Cpt1a	NM_013495	4.95905e-006	1.57703	E15.5 * Wnt1 up vs E15.5 * Ctrl
10506296		---	0.000691538	1.57654	E15.5 * Wnt1 up vs E15.5 * Ctrl
10431140	1810041L15Rik	BC062953	9.33486e-005	1.57608	E15.5 * Wnt1 up vs E15.5 * Ctrl
10603166	Trappc2	NM_025432	0.00318581	1.57565	E15.5 * Wnt1 up vs E15.5 * Ctrl
10374202	Adcy1	NM_009622	3.53614e-006	1.57539	E15.5 * Wnt1 up vs E15.5 * Ctrl

10518226	Vps13d	BC038488	0.00127028	1.57478	E15.5 * Wnt1 up vs E15.5 * Ctrl
10457536	Ankrd29	ENSMUST00000	5.65159e-005	1.57451	E15.5 * Wnt1 up vs E15.5 * Ctrl
10464030	Adra2a	NM_007417	4.35753e-005	1.57392	E15.5 * Wnt1 up vs E15.5 * Ctrl
10484431	Txndc14	NM_025868	1.72373e-005	1.57377	E15.5 * Wnt1 up vs E15.5 * Ctrl
10536827	Ccdc136	BC006583	2.81687e-007	1.57372	E15.5 * Wnt1 up vs E15.5 * Ctrl
10462683	Pcgf5	NM_029508	0.000703326	1.57364	E15.5 * Wnt1 up vs E15.5 * Ctrl
10554712	2610206C17Rik	AK011897	2.24514e-005	1.57358	E15.5 * Wnt1 up vs E15.5 * Ctrl
10433163	Ppp1r1a	NM_021391	8.33086e-006	1.57355	E15.5 * Wnt1 up vs E15.5 * Ctrl
10442914	0610011F06Rik	NM_026686	3.48182e-006	1.57248	E15.5 * Wnt1 up vs E15.5 * Ctrl
10410778	Gpr98	NM_054053	5.53947e-010	1.5721	E15.5 * Wnt1 up vs E15.5 * Ctrl
10399854	Slc26a4	NM_011867	2.40406e-005	1.57139	E15.5 * Wnt1 up vs E15.5 * Ctrl
10448925	Cacna1h	NM_021415	0.00019356	1.57134	E15.5 * Wnt1 up vs E15.5 * Ctrl
10518947	Ajap1	NM_001099299	6.81154e-005	1.57124	E15.5 * Wnt1 up vs E15.5 * Ctrl
10498204	ENSMUSG0000C	ENSMUST00000(	0.000486993	1.57088	E15.5 * Wnt1 up vs E15.5 * Ctrl
10563387	EG545963	NM_001033792	0.00654564	1.5707	E15.5 * Wnt1 up vs E15.5 * Ctrl
10563643	Tsg101	NM_021884	0.000907953	1.57012	E15.5 * Wnt1 up vs E15.5 * Ctrl
10369525	2010107G23Rik	NM_027251	4.28279e-005	1.56989	E15.5 * Wnt1 up vs E15.5 * Ctrl
10528484	Srpk2	NM_009274	5.79668e-007	1.56942	E15.5 * Wnt1 up vs E15.5 * Ctrl
10384064	Camk2b	NM_007595	4.87285e-008	1.56932	E15.5 * Wnt1 up vs E15.5 * Ctrl
10581188	4931428F04Rik	BC043046	1.74844e-005	1.5685	E15.5 * Wnt1 up vs E15.5 * Ctrl
10413255	Duxbl	NM_183389	0.000180638	1.56849	E15.5 * Wnt1 up vs E15.5 * Ctrl
10413265	Duxbl	NM_183389	0.000180638	1.56849	E15.5 * Wnt1 up vs E15.5 * Ctrl
10505911	Dmrta1	NM_175647	0.000455935	1.56711	E15.5 * Wnt1 up vs E15.5 * Ctrl
10436788	Hunk	NM_015755	9.19356e-006	1.56638	E15.5 * Wnt1 up vs E15.5 * Ctrl
10525542	Bcl7a	NM_029850	4.21121e-005	1.56589	E15.5 * Wnt1 up vs E15.5 * Ctrl
10405757	2010111I01Rik	NM_028079	1.00419e-005	1.56546	E15.5 * Wnt1 up vs E15.5 * Ctrl
10391454	Vat1	NM_012037	0.00029829	1.56524	E15.5 * Wnt1 up vs E15.5 * Ctrl
10419999	Jph4	NM_177049	8.05343e-006	1.5648	E15.5 * Wnt1 up vs E15.5 * Ctrl
10383575	Tbcd	NM_029878	5.03177e-008	1.56424	E15.5 * Wnt1 up vs E15.5 * Ctrl
10457780	Fam59a	NM_001033445	7.33161e-006	1.56362	E15.5 * Wnt1 up vs E15.5 * Ctrl
10591200	EG624341	XM_888877	0.0129635	1.5635	E15.5 * Wnt1 up vs E15.5 * Ctrl
10391987		---	4.17441e-007	1.5629	E15.5 * Wnt1 up vs E15.5 * Ctrl
10520351	B930011P16Rik	NM_207282	0.00103224	1.56277	E15.5 * Wnt1 up vs E15.5 * Ctrl
10500327	Hist2h3c2	NM_054045	0.0124518	1.56264	E15.5 * Wnt1 up vs E15.5 * Ctrl
10389581	Ypel2	NM_001005341	3.72531e-006	1.56226	E15.5 * Wnt1 up vs E15.5 * Ctrl
10430389	Mfng	NM_008595	1.96055e-005	1.56136	E15.5 * Wnt1 up vs E15.5 * Ctrl
10406456	AY512938	AY512938	0.00827236	1.56122	E15.5 * Wnt1 up vs E15.5 * Ctrl
10372383	Zdhhc17	BC051527	5.68209e-005	1.56113	E15.5 * Wnt1 up vs E15.5 * Ctrl
10471882	Olfml2a	NM_172854	2.4898e-006	1.56059	E15.5 * Wnt1 up vs E15.5 * Ctrl
10456522	Tcf4	NM_013685	1.36251e-008	1.56046	E15.5 * Wnt1 up vs E15.5 * Ctrl
10522658	C530008M17Rik	ENSMUST00000	0.000286588	1.56039	E15.5 * Wnt1 up vs E15.5 * Ctrl
10500876	Lrig2	NM_001025067	0.000138593	1.56022	E15.5 * Wnt1 up vs E15.5 * Ctrl
10603151	Gpm6b	NM_023122	1.54195e-006	1.55853	E15.5 * Wnt1 up vs E15.5 * Ctrl
10397651	Spata7	NM_178914	0.00111174	1.55835	E15.5 * Wnt1 up vs E15.5 * Ctrl
10565873	Ppme1	NM_028292	0.000533429	1.55784	E15.5 * Wnt1 up vs E15.5 * Ctrl
10367546	Oprm1	NM_001039652	7.95402e-006	1.55774	E15.5 * Wnt1 up vs E15.5 * Ctrl
10600797	Apoo-ps	NR_004438	4.78541e-005	1.55764	E15.5 * Wnt1 up vs E15.5 * Ctrl
10422244	Slitrk6	NM_175499	0.000205509	1.55685	E15.5 * Wnt1 up vs E15.5 * Ctrl
10408450	Sox4	NM_009238	0.000462463	1.55671	E15.5 * Wnt1 up vs E15.5 * Ctrl
10455080	Pcdhb9	NM_053134	0.0122508	1.5564	E15.5 * Wnt1 up vs E15.5 * Ctrl
10511901	Ankrd6	NM_001012450	6.68649e-007	1.55622	E15.5 * Wnt1 up vs E15.5 * Ctrl
10499514	Trim46	NM_183037	9.77322e-006	1.55551	E15.5 * Wnt1 up vs E15.5 * Ctrl
10604038	Sfrs17b	NM_001081956	0.000107584	1.55528	E15.5 * Wnt1 up vs E15.5 * Ctrl
10409240	Sema4d	NM_013660	3.08245e-007	1.55521	E15.5 * Wnt1 up vs E15.5 * Ctrl
10520096	Klhl7	NM_026448	2.62964e-005	1.55488	E15.5 * Wnt1 up vs E15.5 * Ctrl
10445373	B230354K17Rik	ENSMUST00000(	0.00063974	1.55424	E15.5 * Wnt1 up vs E15.5 * Ctrl
10372385	Zdhhc17	NM_172554	4.06232e-006	1.55417	E15.5 * Wnt1 up vs E15.5 * Ctrl
10361828	Cited2	NM_010828	4.66733e-006	1.55408	E15.5 * Wnt1 up vs E15.5 * Ctrl
10375973	Taf13	NM_025444	1.56421e-005	1.55407	E15.5 * Wnt1 up vs E15.5 * Ctrl
10587012	Ccpg1	NM_001114328	4.16554e-006	1.55403	E15.5 * Wnt1 up vs E15.5 * Ctrl
10497673	Zmat3	NM_009517	0.000174694	1.5537	E15.5 * Wnt1 up vs E15.5 * Ctrl
10514892	2210012G02Rik	NM_025617	0.0014565	1.55346	E15.5 * Wnt1 up vs E15.5 * Ctrl
10455292	2900055J20Rik	ENSMUST00000(	0.000204456	1.55332	E15.5 * Wnt1 up vs E15.5 * Ctrl
10601303	Chic1	NM_009767	0.000597946	1.55289	E15.5 * Wnt1 up vs E15.5 * Ctrl
10600249	Plxnb3	NM_019587	7.82158e-006	1.5516	E15.5 * Wnt1 up vs E15.5 * Ctrl
10423791	4930447A16Rik	ENSMUST00000(	0.00286573	1.55142	E15.5 * Wnt1 up vs E15.5 * Ctrl

10503876	RP23-12I24.6	NM_177774	3.62912e-005	1.5512	E15.5 * Wnt1 up vs E15.5 * Ctrl
10501860	Fnbp1l	NM_001114665	2.961e-009	1.5511	E15.5 * Wnt1 up vs E15.5 * Ctrl
10554323		---	0.000342291	1.55088	E15.5 * Wnt1 up vs E15.5 * Ctrl
10475957	Ttl	NM_027192	4.68627e-005	1.55084	E15.5 * Wnt1 up vs E15.5 * Ctrl
10409118	Wnk2	NM_029361	4.4421e-009	1.55055	E15.5 * Wnt1 up vs E15.5 * Ctrl
10555205	Gdpd5	NM_201352	0.00019785	1.55046	E15.5 * Wnt1 up vs E15.5 * Ctrl
10472820	Itga6	NM_008397	5.08639e-005	1.55029	E15.5 * Wnt1 up vs E15.5 * Ctrl
10535065	Adap1	NM_172723	3.93399e-005	1.54886	E15.5 * Wnt1 up vs E15.5 * Ctrl
10480878	Camsap1	NM_001115076	1.73956e-006	1.54863	E15.5 * Wnt1 up vs E15.5 * Ctrl
10532709		---	0.00457109	1.54859	E15.5 * Wnt1 up vs E15.5 * Ctrl
10355162	Plekhm3	NM_001039493	0.000398134	1.5471	E15.5 * Wnt1 up vs E15.5 * Ctrl
10552030	C630016N16Rik	AK049951	9.65289e-005	1.54703	E15.5 * Wnt1 up vs E15.5 * Ctrl
10360460	Chml	NM_021350	0.000368164	1.54677	E15.5 * Wnt1 up vs E15.5 * Ctrl
10597531	Rbms3	NM_178660	7.78489e-008	1.54675	E15.5 * Wnt1 up vs E15.5 * Ctrl
10438478	Abcc5	NM_013790	2.11556e-007	1.54618	E15.5 * Wnt1 up vs E15.5 * Ctrl
10415766	Sacs	NM_172809	2.12618e-006	1.54497	E15.5 * Wnt1 up vs E15.5 * Ctrl
10472694	4933404M02Rik	NM_025744	2.79822e-005	1.54485	E15.5 * Wnt1 up vs E15.5 * Ctrl
10458247	Lrrtm2	NM_178005	0.000211435	1.54458	E15.5 * Wnt1 up vs E15.5 * Ctrl
10501374	5330417C22Rik	NM_001033304	2.41278e-006	1.54456	E15.5 * Wnt1 up vs E15.5 * Ctrl
10397575	Nrxn3	NM_172544	3.39583e-005	1.544	E15.5 * Wnt1 up vs E15.5 * Ctrl
10384183	AB182283	NM_001081652	2.80633e-007	1.54374	E15.5 * Wnt1 up vs E15.5 * Ctrl
10526181	Gatsl2	NM_030719	1.83797e-005	1.54336	E15.5 * Wnt1 up vs E15.5 * Ctrl
10555063	Ints4	NM_027256	4.07192e-007	1.54295	E15.5 * Wnt1 up vs E15.5 * Ctrl
10543249	EG232599	ENSMUST00000(	0.00330379	1.54255	E15.5 * Wnt1 up vs E15.5 * Ctrl
10574676	Nol3	NM_030152	4.48238e-005	1.54231	E15.5 * Wnt1 up vs E15.5 * Ctrl
10593799	Scaper	NM_001081341	1.47037e-006	1.54214	E15.5 * Wnt1 up vs E15.5 * Ctrl
10376163	Rapgef6	NM_175258	2.85711e-008	1.542	E15.5 * Wnt1 up vs E15.5 * Ctrl
10571207	Dusp26	NM_025869	0.000223656	1.5418	E15.5 * Wnt1 up vs E15.5 * Ctrl
10557705	Phkg2	NM_026888	5.12972e-006	1.54047	E15.5 * Wnt1 up vs E15.5 * Ctrl
10588083	Faim	NM_001122851	0.000101536	1.5404	E15.5 * Wnt1 up vs E15.5 * Ctrl
10447732	Pacrg	NM_027032	0.00135011	1.54038	E15.5 * Wnt1 up vs E15.5 * Ctrl
10579799	Tmem184c	NM_145599	9.26586e-006	1.54003	E15.5 * Wnt1 up vs E15.5 * Ctrl
10415576	Zmym2	NM_029498	4.55997e-007	1.53964	E15.5 * Wnt1 up vs E15.5 * Ctrl
10441706	1700010I14Rik	NM_025851	0.000180066	1.53957	E15.5 * Wnt1 up vs E15.5 * Ctrl
10544114	Hipk2	NM_010433	8.26419e-007	1.53921	E15.5 * Wnt1 up vs E15.5 * Ctrl
10432129	Olfr288	NM_001011733	0.000730545	1.53859	E15.5 * Wnt1 up vs E15.5 * Ctrl
10487748	4930402H24Rik	BC052447	7.60584e-007	1.53759	E15.5 * Wnt1 up vs E15.5 * Ctrl
10463140	Lcor	NM_172154	9.65872e-008	1.53702	E15.5 * Wnt1 up vs E15.5 * Ctrl
10540227	Kbtbd8	NM_001008785	0.00425193	1.53602	E15.5 * Wnt1 up vs E15.5 * Ctrl
10590298	Eif1b	NM_026892	7.6168e-006	1.53567	E15.5 * Wnt1 up vs E15.5 * Ctrl
10373218	Nxph4	NM_183297	0.000201023	1.53543	E15.5 * Wnt1 up vs E15.5 * Ctrl
10370651	BC005764	NM_181681	2.64424e-005	1.53539	E15.5 * Wnt1 up vs E15.5 * Ctrl
10413220	ENSMUSG0000C	ENSMUST00000	0.000138249	1.53529	E15.5 * Wnt1 up vs E15.5 * Ctrl
10505512	Trim32	NM_053084	2.08187e-005	1.53505	E15.5 * Wnt1 up vs E15.5 * Ctrl
10516393	Eif2c4	NM_153177	7.94103e-005	1.53465	E15.5 * Wnt1 up vs E15.5 * Ctrl
10571747		---	0.0137283	1.53453	E15.5 * Wnt1 up vs E15.5 * Ctrl
10394627	Nbas	BC057020	8.02887e-008	1.53451	E15.5 * Wnt1 up vs E15.5 * Ctrl
10583179	Pgr	NM_008829	3.95118e-005	1.53357	E15.5 * Wnt1 up vs E15.5 * Ctrl
10517373	Rcan3	NM_022980	3.78311e-008	1.53355	E15.5 * Wnt1 up vs E15.5 * Ctrl
10522265	Slc30a9	NM_178651	1.24915e-005	1.53331	E15.5 * Wnt1 up vs E15.5 * Ctrl
10376425	OTTMUSG00000	ENSMUST00000(	0.00288691	1.53306	E15.5 * Wnt1 up vs E15.5 * Ctrl
10566067	Rnf121	NM_029211	0.00034524	1.53278	E15.5 * Wnt1 up vs E15.5 * Ctrl
10601844	Bhlhb9	NM_198161	0.000395209	1.5327	E15.5 * Wnt1 up vs E15.5 * Ctrl
10542555	MGC7817	BC006653	0.00173551	1.53263	E15.5 * Wnt1 up vs E15.5 * Ctrl
10563921		---	2.93602e-008	1.53221	E15.5 * Wnt1 up vs E15.5 * Ctrl
10563925		---	2.93602e-008	1.53221	E15.5 * Wnt1 up vs E15.5 * Ctrl
10540544	Thumpd3	NM_008188	1.55989e-006	1.53203	E15.5 * Wnt1 up vs E15.5 * Ctrl
10553501	Slc17a6	NM_080853	0.000596306	1.53174	E15.5 * Wnt1 up vs E15.5 * Ctrl
10605616	Il1rapl1	BC119580	0.000559364	1.53098	E15.5 * Wnt1 up vs E15.5 * Ctrl
10363161	6330442E10Rik	BC079613	0.00537721	1.53092	E15.5 * Wnt1 up vs E15.5 * Ctrl
10573483	Prdx2	NM_011563	7.38999e-008	1.53088	E15.5 * Wnt1 up vs E15.5 * Ctrl
10438702	St6gal1	NM_145933	0.00100197	1.53046	E15.5 * Wnt1 up vs E15.5 * Ctrl
10437655	Fam18a	BC068110	0.00223001	1.53037	E15.5 * Wnt1 up vs E15.5 * Ctrl
10582743	Pcnxl2	NM_175561	1.64305e-005	1.52958	E15.5 * Wnt1 up vs E15.5 * Ctrl
10601848	6530401D17Rik	NR_003641	0.00707568	1.52922	E15.5 * Wnt1 up vs E15.5 * Ctrl
10442236	3110052M02Rik	ENSMUST00000(	0.0133351	1.5289	E15.5 * Wnt1 up vs E15.5 * Ctrl

10423805	Atp6v1c1	NM_025494	1.9927e-005	1.5285	E15.5 * Wnt1 up vs E15.5 * Ctrl
10417124	B930095G15Rik	BC096543	3.75543e-007	1.52831	E15.5 * Wnt1 up vs E15.5 * Ctrl
10498710	Bche	NM_009738	0.000663974	1.52821	E15.5 * Wnt1 up vs E15.5 * Ctrl
10381416	Rnd2	NM_009708	0.000311169	1.52709	E15.5 * Wnt1 up vs E15.5 * Ctrl
10412100	Map3k1	NM_011945	0.000136376	1.52687	E15.5 * Wnt1 up vs E15.5 * Ctrl
10604505	6720401G13Rik	NR_015505	1.08418e-005	1.52662	E15.5 * Wnt1 up vs E15.5 * Ctrl
10593492	Zc3h12c	AK220416	3.3749e-007	1.52633	E15.5 * Wnt1 up vs E15.5 * Ctrl
10460010	Galr1	NM_008082	0.00177518	1.52629	E15.5 * Wnt1 up vs E15.5 * Ctrl
10461844	Gnaq	NM_008139	9.17707e-006	1.52626	E15.5 * Wnt1 up vs E15.5 * Ctrl
10415952		---	0.0110517	1.52624	E15.5 * Wnt1 up vs E15.5 * Ctrl
10576288	Spire2	NM_172287	1.53275e-005	1.52613	E15.5 * Wnt1 up vs E15.5 * Ctrl
10352920	Xkr4	NM_001011874	3.54586e-005	1.52539	E15.5 * Wnt1 up vs E15.5 * Ctrl
10452516	Ankrd12	NM_001025572	0.000699137	1.52484	E15.5 * Wnt1 up vs E15.5 * Ctrl
10581493	Pdxp	NM_020271	0.00027502	1.52484	E15.5 * Wnt1 up vs E15.5 * Ctrl
10369752	Lrrtm3	NM_178678	0.000846468	1.5248	E15.5 * Wnt1 up vs E15.5 * Ctrl
10543369	Cadps2	NM_153163	1.78305e-006	1.52471	E15.5 * Wnt1 up vs E15.5 * Ctrl
10428238	Ubr5	NM_001081359	2.38673e-007	1.52456	E15.5 * Wnt1 up vs E15.5 * Ctrl
10551828	EG330503	NM_001033540	2.79011e-005	1.52455	E15.5 * Wnt1 up vs E15.5 * Ctrl
10356626		---	0.000318869	1.52409	E15.5 * Wnt1 up vs E15.5 * Ctrl
10568668	Adam12	NM_007400	0.00020619	1.52389	E15.5 * Wnt1 up vs E15.5 * Ctrl
10596200	Tmem108	NM_178638	0.0120743	1.52388	E15.5 * Wnt1 up vs E15.5 * Ctrl
10396306	1200003C05Rik	NM_024205	1.01841e-005	1.52319	E15.5 * Wnt1 up vs E15.5 * Ctrl
10585180	Ncam1	BC011310	0.00192873	1.52254	E15.5 * Wnt1 up vs E15.5 * Ctrl
10455108	Pcdhb16	NM_053141	0.0131389	1.52175	E15.5 * Wnt1 up vs E15.5 * Ctrl
10517616	Vwa5b1	NM_029401	3.58346e-005	1.52171	E15.5 * Wnt1 up vs E15.5 * Ctrl
10533007	Ccdc64	NM_001080808	7.72773e-007	1.52155	E15.5 * Wnt1 up vs E15.5 * Ctrl
10401160	6330442E10Rik	BC066067	0.00294792	1.5211	E15.5 * Wnt1 up vs E15.5 * Ctrl
10501567	Rnpc3	NM_001038696	7.40856e-006	1.52101	E15.5 * Wnt1 up vs E15.5 * Ctrl
10479685	Oprl1	NM_011012	7.57649e-005	1.52088	E15.5 * Wnt1 up vs E15.5 * Ctrl
10422238	EG668761	ENSMUST00000(	0.000288583	1.51977	E15.5 * Wnt1 up vs E15.5 * Ctrl
10516046		---	0.000612438	1.51943	E15.5 * Wnt1 up vs E15.5 * Ctrl
10504218	Dnajb5	NM_019874	0.000151301	1.51922	E15.5 * Wnt1 up vs E15.5 * Ctrl
10365817	Ntn4	NM_021320	0.000194287	1.51905	E15.5 * Wnt1 up vs E15.5 * Ctrl
10412466	Hmgcs1	NM_145942	1.58445e-005	1.51897	E15.5 * Wnt1 up vs E15.5 * Ctrl
10409365	Gprin1	NM_012014	7.97055e-006	1.51882	E15.5 * Wnt1 up vs E15.5 * Ctrl
10558468	Jakmip3	BC119557	0.000198913	1.51879	E15.5 * Wnt1 up vs E15.5 * Ctrl
10353258	4930444P10Rik	ENSMUST00000(	0.000150106	1.51875	E15.5 * Wnt1 up vs E15.5 * Ctrl
10422075	Mycbp2	NM_207215	8.01981e-008	1.51822	E15.5 * Wnt1 up vs E15.5 * Ctrl
10395227	Cog5	ENSMUST00000(	5.7389e-005	1.51821	E15.5 * Wnt1 up vs E15.5 * Ctrl
10472097	Fmnl2	NM_172409	5.02631e-007	1.51799	E15.5 * Wnt1 up vs E15.5 * Ctrl
10449041	Metrn	NM_133719	0.00068534	1.51781	E15.5 * Wnt1 up vs E15.5 * Ctrl
10504008	Chmp5	NM_029814	1.88634e-006	1.51758	E15.5 * Wnt1 up vs E15.5 * Ctrl
10457357	Mpp7	ENSMUST00000(	0.000433805	1.51725	E15.5 * Wnt1 up vs E15.5 * Ctrl
10345580	Inpp4a	NM_030266	2.38663e-005	1.51644	E15.5 * Wnt1 up vs E15.5 * Ctrl
10396740	Gphn	NM_172952	2.94439e-005	1.51631	E15.5 * Wnt1 up vs E15.5 * Ctrl
10584236	Pate2	NM_001033421	0.000261412	1.51572	E15.5 * Wnt1 up vs E15.5 * Ctrl
10517465	Kdm1	NM_133872	2.15397e-009	1.51558	E15.5 * Wnt1 up vs E15.5 * Ctrl
10600017	Hmgb3	NM_008253	1.86151e-009	1.51498	E15.5 * Wnt1 up vs E15.5 * Ctrl
10381260	Tubg2	NM_134028	1.28716e-005	1.51464	E15.5 * Wnt1 up vs E15.5 * Ctrl
10387194	Odf4	NM_145746	3.71368e-005	1.51444	E15.5 * Wnt1 up vs E15.5 * Ctrl
10607356	Gpr173	NM_027543	3.74723e-005	1.51441	E15.5 * Wnt1 up vs E15.5 * Ctrl
10477297	Kif3b	NM_008444	6.01288e-005	1.51383	E15.5 * Wnt1 up vs E15.5 * Ctrl
10547553	Mical3	NM_153396	1.65276e-005	1.51305	E15.5 * Wnt1 up vs E15.5 * Ctrl
10431463	Fam116b	NM_027081	2.26476e-005	1.5124	E15.5 * Wnt1 up vs E15.5 * Ctrl
10346668	Fam117b	NM_001037725	4.39791e-006	1.51233	E15.5 * Wnt1 up vs E15.5 * Ctrl
10579287	Tmem59l	NM_182991	0.000209236	1.51201	E15.5 * Wnt1 up vs E15.5 * Ctrl
10463112	Ccnj	NM_172839	0.00425299	1.51193	E15.5 * Wnt1 up vs E15.5 * Ctrl
10391744	Gpatch8	AK135410	0.00460062	1.5119	E15.5 * Wnt1 up vs E15.5 * Ctrl
10456248	Onecut2	NM_194268	0.000117742	1.51178	E15.5 * Wnt1 up vs E15.5 * Ctrl
10493798	S100a16	NM_026416	0.000719042	1.51172	E15.5 * Wnt1 up vs E15.5 * Ctrl
10599853	Ldoc1	NM_001018087	0.00108094	1.51123	E15.5 * Wnt1 up vs E15.5 * Ctrl
10425265	ENSMUSG0000C	ENSMUST00000	0.00399497	1.51117	E15.5 * Wnt1 up vs E15.5 * Ctrl
10483353	Scn7a	NM_009135	0.00143481	1.5111	E15.5 * Wnt1 up vs E15.5 * Ctrl
10518167	Trappc2	NM_025432	0.004763	1.51062	E15.5 * Wnt1 up vs E15.5 * Ctrl
10456723	BC031181	BC016084	6.07195e-005	1.50975	E15.5 * Wnt1 up vs E15.5 * Ctrl
10391513	Dusp3	NM_028207	0.00278861	1.50915	E15.5 * Wnt1 up vs E15.5 * Ctrl

10354741	Rftn2	NM_028713	2.7236e-006	1.50903	E15.5 * Wnt1 up vs E15.5 * Ctrl
10411491	Tnpo1	NM_178716	2.40092e-008	1.50887	E15.5 * Wnt1 up vs E15.5 * Ctrl
10560311		---	0.00382354	1.50726	E15.5 * Wnt1 up vs E15.5 * Ctrl
10505630	Snapc3	NM_029949	1.13304e-006	1.50712	E15.5 * Wnt1 up vs E15.5 * Ctrl
10584883	Fxyd6	NM_022004	9.78894e-006	1.50678	E15.5 * Wnt1 up vs E15.5 * Ctrl
10482249	Nr6a1	NM_010264	4.13098e-005	1.50668	E15.5 * Wnt1 up vs E15.5 * Ctrl
10604633	Cxx1b	NM_001018063	1.66383e-007	1.50656	E15.5 * Wnt1 up vs E15.5 * Ctrl
10551426	Zfp59	NM_011762	8.86608e-007	1.50643	E15.5 * Wnt1 up vs E15.5 * Ctrl
10499095	Fam160a1	NM_172682	1.608e-005	1.50618	E15.5 * Wnt1 up vs E15.5 * Ctrl
10545644	Wbp1	NM_016757	0.00468251	1.50591	E15.5 * Wnt1 up vs E15.5 * Ctrl
10545608	Sema4f	NM_011350	8.37645e-007	1.50553	E15.5 * Wnt1 up vs E15.5 * Ctrl
10561673	Spred3	NM_182927	1.20263e-005	1.50513	E15.5 * Wnt1 up vs E15.5 * Ctrl
10575363	Zfp612	NM_175480	0.000219276	1.50481	E15.5 * Wnt1 up vs E15.5 * Ctrl
10541131	Dcp1b	NM_001033379	0.000331406	1.50476	E15.5 * Wnt1 up vs E15.5 * Ctrl
10399457	Akr1b3	NM_009658	1.10944e-005	1.50469	E15.5 * Wnt1 up vs E15.5 * Ctrl
10452257	Slc25a23	NM_025877	0.000336935	1.50441	E15.5 * Wnt1 up vs E15.5 * Ctrl
10406877	Serf1	NM_011353	0.000102358	1.50419	E15.5 * Wnt1 up vs E15.5 * Ctrl
10512655	Rnf38	NM_001038993	7.1787e-008	1.50383	E15.5 * Wnt1 up vs E15.5 * Ctrl
10457359	Mpp7	NM_001081287	0.000242423	1.50377	E15.5 * Wnt1 up vs E15.5 * Ctrl
10496182	Cxxc4	NM_001004367	2.19111e-005	1.5037	E15.5 * Wnt1 up vs E15.5 * Ctrl
10479311	Ss18l1	NM_178750	1.37012e-005	1.50278	E15.5 * Wnt1 up vs E15.5 * Ctrl
10501319	Celsr2	NM_017392	0.000199072	1.50241	E15.5 * Wnt1 up vs E15.5 * Ctrl
10557628	Zfp689	NM_175163	2.48655e-005	1.50223	E15.5 * Wnt1 up vs E15.5 * Ctrl
10362394	Hddc2	NM_027168	0.000510421	1.50219	E15.5 * Wnt1 up vs E15.5 * Ctrl
10538658	Herc3	NM_028705	1.59211e-006	1.50195	E15.5 * Wnt1 up vs E15.5 * Ctrl
10466976	Gldc	NM_138595	0.000121185	1.50195	E15.5 * Wnt1 up vs E15.5 * Ctrl
10368092	Hebp2	NM_019487	0.00012933	1.50167	E15.5 * Wnt1 up vs E15.5 * Ctrl
10497214	Tpd52	NM_001025261	0.00246188	1.50137	E15.5 * Wnt1 up vs E15.5 * Ctrl
10413243	Duxbl	NM_183389	0.00046419	1.50128	E15.5 * Wnt1 up vs E15.5 * Ctrl
10526726	Zkscan1	NM_133906	7.7829e-007	1.50088	E15.5 * Wnt1 up vs E15.5 * Ctrl
10516064	Mfsd2	NM_029662	0.00176073	1.50039	E15.5 * Wnt1 up vs E15.5 * Ctrl
10428222	Ncald	NM_134094	1.07402e-005	1.5003	E15.5 * Wnt1 up vs E15.5 * Ctrl
10465619	Flrt1	NM_201411	0.000812638	1.49932	E15.5 * Wnt1 up vs E15.5 * Ctrl
10356936	Hisppd1	NM_173760	7.94448e-006	1.49928	E15.5 * Wnt1 up vs E15.5 * Ctrl
10412911	Fdft1	BC138301	9.56805e-007	1.49897	E15.5 * Wnt1 up vs E15.5 * Ctrl
10426611	Cacnb3	NM_007581	4.29118e-005	1.49841	E15.5 * Wnt1 up vs E15.5 * Ctrl
10478066	Snhg11	NM_175692	0.000299333	1.49775	E15.5 * Wnt1 up vs E15.5 * Ctrl
10379321	Rab11fip4	NM_175543	4.47855e-005	1.49764	E15.5 * Wnt1 up vs E15.5 * Ctrl
10348432	Agap1	NM_178119	0.000365245	1.49761	E15.5 * Wnt1 up vs E15.5 * Ctrl
10344935	Kcnb2	NM_001098528	0.00169112	1.49747	E15.5 * Wnt1 up vs E15.5 * Ctrl
10601312	ENSMUSG00000	ENSMUST00000	0.0044752	1.49745	E15.5 * Wnt1 up vs E15.5 * Ctrl
10395659	Coch	NM_007728	0.000856352	1.49716	E15.5 * Wnt1 up vs E15.5 * Ctrl
10522335	Atp10d	NR_003966	1.43644e-005	1.49671	E15.5 * Wnt1 up vs E15.5 * Ctrl
10426180	Saps2	NM_026813	5.2223e-006	1.49649	E15.5 * Wnt1 up vs E15.5 * Ctrl
10560530	Bloc1s3	NM_177692	0.00914888	1.49631	E15.5 * Wnt1 up vs E15.5 * Ctrl
10547022	Timp4	NM_080639	0.000214951	1.49608	E15.5 * Wnt1 up vs E15.5 * Ctrl
10482167	Zbtb6	NM_146253	0.00413426	1.49465	E15.5 * Wnt1 up vs E15.5 * Ctrl
10452721	Trmt61b	AK032413	2.14645e-005	1.49418	E15.5 * Wnt1 up vs E15.5 * Ctrl
10363669	Dnajc12	NM_013888	9.80948e-005	1.49396	E15.5 * Wnt1 up vs E15.5 * Ctrl
10553935	Tarsl2	NM_172310	0.000128802	1.49334	E15.5 * Wnt1 up vs E15.5 * Ctrl
10530870	Epha5	NM_007937	0.000264668	1.49321	E15.5 * Wnt1 up vs E15.5 * Ctrl
10495120	Ovgp1	NM_007696	0.00186633	1.49297	E15.5 * Wnt1 up vs E15.5 * Ctrl
10401343	Map3k9	NM_177395	1.79092e-005	1.49282	E15.5 * Wnt1 up vs E15.5 * Ctrl
10366825	Agap2	NM_001033263	9.74213e-005	1.49251	E15.5 * Wnt1 up vs E15.5 * Ctrl
10434436	Vwa5b2	NM_001144953	0.000340799	1.49226	E15.5 * Wnt1 up vs E15.5 * Ctrl
10398459	Ppp2r5c	NM_001135001	0.0106362	1.49182	E15.5 * Wnt1 up vs E15.5 * Ctrl
10384566		---	0.0036078	1.49129	E15.5 * Wnt1 up vs E15.5 * Ctrl
10509568	Camk2n1	NM_025451	1.52761e-005	1.49099	E15.5 * Wnt1 up vs E15.5 * Ctrl
10572733	BC049349	BC049349	0.00141276	1.49031	E15.5 * Wnt1 up vs E15.5 * Ctrl
10361882	Nhsl1	NM_173390	6.19013e-006	1.49013	E15.5 * Wnt1 up vs E15.5 * Ctrl
10456171	Spink10	NM_177829	0.00894666	1.49005	E15.5 * Wnt1 up vs E15.5 * Ctrl
10581289	Atp6v0d1	NM_013477	0.000512674	1.48898	E15.5 * Wnt1 up vs E15.5 * Ctrl
10571371	Tusc3	NM_030254	1.00507e-007	1.48744	E15.5 * Wnt1 up vs E15.5 * Ctrl
10430280		---	0.000344613	1.48717	E15.5 * Wnt1 up vs E15.5 * Ctrl
10511580	Ppm2c	NM_001098230	6.59105e-006	1.48712	E15.5 * Wnt1 up vs E15.5 * Ctrl
10390258	Snx11	NM_028965	0.000130554	1.48699	E15.5 * Wnt1 up vs E15.5 * Ctrl

10390430	RP23-157O10.7	NM_018873	3.23506e-007	1.4869	E15.5 * Wnt1 up vs E15.5 * Ctrl
10525210	AU042671	ENSMUST00000	0.000137339	1.48669	E15.5 * Wnt1 up vs E15.5 * Ctrl
10488862	Ggt7	NM_144786	1.43225e-006	1.48631	E15.5 * Wnt1 up vs E15.5 * Ctrl
10484488	P2rx3	NM_145526	6.87645e-005	1.48623	E15.5 * Wnt1 up vs E15.5 * Ctrl
10528238	Phtf2	NM_172992	6.70781e-005	1.48621	E15.5 * Wnt1 up vs E15.5 * Ctrl
10607225	Lrch2	NM_001081173	0.000797055	1.4862	E15.5 * Wnt1 up vs E15.5 * Ctrl
10505747	Rraga	NM_178376	8.98056e-005	1.48592	E15.5 * Wnt1 up vs E15.5 * Ctrl
10433971	Rimbp3	NM_001033338	0.00056037	1.48561	E15.5 * Wnt1 up vs E15.5 * Ctrl
10358379	Trove2	NM_013835	0.00013356	1.48549	E15.5 * Wnt1 up vs E15.5 * Ctrl
10535331	Mmd2	NM_175217	4.51444e-006	1.48536	E15.5 * Wnt1 up vs E15.5 * Ctrl
10380761	Socs7	NM_138657	2.9418e-005	1.48516	E15.5 * Wnt1 up vs E15.5 * Ctrl
10433735	Abcc1	NM_008576	6.37197e-005	1.48484	E15.5 * Wnt1 up vs E15.5 * Ctrl
10591853	Tbx20	NM_194263	0.00144769	1.48475	E15.5 * Wnt1 up vs E15.5 * Ctrl
10491848	Larp2	BC100361	0.000279071	1.48431	E15.5 * Wnt1 up vs E15.5 * Ctrl
10442968	Rgs11	NM_001081069	1.78449e-005	1.4842	E15.5 * Wnt1 up vs E15.5 * Ctrl
10486595	Ttbk2	NM_001024856	5.22906e-006	1.48419	E15.5 * Wnt1 up vs E15.5 * Ctrl
10508986	Stmn1	NM_019641	4.15085e-011	1.48358	E15.5 * Wnt1 up vs E15.5 * Ctrl
10519117	Prkcz	NM_008860	1.57576e-005	1.48346	E15.5 * Wnt1 up vs E15.5 * Ctrl
10390175	Ngfr	NM_033217	8.27452e-005	1.4826	E15.5 * Wnt1 up vs E15.5 * Ctrl
10384555	Aftph	NM_181411	0.000101236	1.4825	E15.5 * Wnt1 up vs E15.5 * Ctrl
10599612	Phf6	NM_027642	6.76729e-006	1.48241	E15.5 * Wnt1 up vs E15.5 * Ctrl
10370754	Dos	NM_015761	4.86711e-006	1.48223	E15.5 * Wnt1 up vs E15.5 * Ctrl
10407955	Epdr1	NM_134065	0.000346582	1.48206	E15.5 * Wnt1 up vs E15.5 * Ctrl
10436945	Slc5a3	NM_017391	1.55632e-005	1.48195	E15.5 * Wnt1 up vs E15.5 * Ctrl
10505996	Fggy	NM_001113412	0.00318212	1.48161	E15.5 * Wnt1 up vs E15.5 * Ctrl
10560408	Psg17	NM_007677	0.00146689	1.48145	E15.5 * Wnt1 up vs E15.5 * Ctrl
10556553	Insc	NM_173767	0.000496825	1.48096	E15.5 * Wnt1 up vs E15.5 * Ctrl
10575619	Terf2ip	NM_020584	4.12417e-005	1.48095	E15.5 * Wnt1 up vs E15.5 * Ctrl
10546775	Trnt1	NM_027296	3.03986e-005	1.48085	E15.5 * Wnt1 up vs E15.5 * Ctrl
10406434	Mef2c	NM_025282	2.29169e-006	1.48055	E15.5 * Wnt1 up vs E15.5 * Ctrl
10563390	Cyth2	NM_011181	0.000965084	1.47928	E15.5 * Wnt1 up vs E15.5 * Ctrl
10574899	Nutf2	NM_026532	0.00642029	1.47921	E15.5 * Wnt1 up vs E15.5 * Ctrl
10517731	Igsf21	NM_198610	0.000514908	1.47912	E15.5 * Wnt1 up vs E15.5 * Ctrl
10430113	D15Wsu169e	BC060637	0.000556483	1.47869	E15.5 * Wnt1 up vs E15.5 * Ctrl
10364712	Cirbp	NM_007705	0.00256914	1.47864	E15.5 * Wnt1 up vs E15.5 * Ctrl
10486469	Vps39	NM_147153	2.17534e-005	1.47814	E15.5 * Wnt1 up vs E15.5 * Ctrl
10600886	Gpr165	NM_029536	0.0036566	1.47776	E15.5 * Wnt1 up vs E15.5 * Ctrl
10553833	Ndn	NM_010882	1.37551e-005	1.4774	E15.5 * Wnt1 up vs E15.5 * Ctrl
10396831	Arg2	NM_009705	0.000581246	1.47733	E15.5 * Wnt1 up vs E15.5 * Ctrl
10483299	Scn1a	NM_018733	2.47352e-007	1.47593	E15.5 * Wnt1 up vs E15.5 * Ctrl
10438103	Ccdc116	NM_029779	2.36034e-006	1.4757	E15.5 * Wnt1 up vs E15.5 * Ctrl
10368683	1700025K23Rik	NM_183254	0.00665673	1.47568	E15.5 * Wnt1 up vs E15.5 * Ctrl
10579335	Pgpep1	NM_023217	1.14122e-005	1.47537	E15.5 * Wnt1 up vs E15.5 * Ctrl
10353272	Stau2	NM_025303	3.19023e-006	1.47535	E15.5 * Wnt1 up vs E15.5 * Ctrl
10438262	Slc25a1	NM_153150	0.000358533	1.47482	E15.5 * Wnt1 up vs E15.5 * Ctrl
10431051	Scube1	NM_022723	0.00021863	1.47455	E15.5 * Wnt1 up vs E15.5 * Ctrl
10599719	Slc9a6	NM_172780	3.29261e-005	1.47429	E15.5 * Wnt1 up vs E15.5 * Ctrl
10463430	Sema4g	NM_011976	0.00100609	1.47401	E15.5 * Wnt1 up vs E15.5 * Ctrl
10513818	Stmn1	NM_019641	1.20902e-010	1.47396	E15.5 * Wnt1 up vs E15.5 * Ctrl
10468217	Actr1a	NM_016860	0.000236848	1.4739	E15.5 * Wnt1 up vs E15.5 * Ctrl
10434556	676915	XR_034101	0.00128597	1.4736	E15.5 * Wnt1 up vs E15.5 * Ctrl
10446334	Glcci1	NM_133236	0.000323952	1.47322	E15.5 * Wnt1 up vs E15.5 * Ctrl
10526193		---	4.76726e-005	1.47315	E15.5 * Wnt1 up vs E15.5 * Ctrl
10430020	Vps28	NM_025842	0.000416283	1.47264	E15.5 * Wnt1 up vs E15.5 * Ctrl
10378833	Ssh2	NM_177710	2.36008e-005	1.47241	E15.5 * Wnt1 up vs E15.5 * Ctrl
10354389	Slc39a10	NM_172653	5.62026e-006	1.47196	E15.5 * Wnt1 up vs E15.5 * Ctrl
10593270	Ttc12	NM_172770	7.65356e-006	1.47194	E15.5 * Wnt1 up vs E15.5 * Ctrl
10555510	Pde2a	NM_001143848	8.72473e-006	1.47167	E15.5 * Wnt1 up vs E15.5 * Ctrl
10558948	Cd151	NM_009842	0.00128113	1.47143	E15.5 * Wnt1 up vs E15.5 * Ctrl
10378649	Slc43a2	NM_173388	0.000126915	1.47132	E15.5 * Wnt1 up vs E15.5 * Ctrl
10542310	Cdkn1b	NM_009875	4.25247e-006	1.47088	E15.5 * Wnt1 up vs E15.5 * Ctrl
10383684	Limk2	NM_010718	4.38803e-007	1.47061	E15.5 * Wnt1 up vs E15.5 * Ctrl
10465844	Asrgl1	NM_025610	1.49604e-006	1.47043	E15.5 * Wnt1 up vs E15.5 * Ctrl
10403229	Itgb8	NM_177290	5.41414e-005	1.46974	E15.5 * Wnt1 up vs E15.5 * Ctrl
10521467	Ccdc96	NM_025725	0.000428042	1.46964	E15.5 * Wnt1 up vs E15.5 * Ctrl
10545658	Wdr54	NM_023790	1.58088e-007	1.46905	E15.5 * Wnt1 up vs E15.5 * Ctrl

10391828	C1ql1	NM_011795	0.00214523	1.46834	E15.5 * Wnt1 up vs E15.5 * Ctrl
10447349	Cript	NM_019936	0.000166326	1.46714	E15.5 * Wnt1 up vs E15.5 * Ctrl
10415787	Kcnrg	NM_206974	0.0103993	1.46702	E15.5 * Wnt1 up vs E15.5 * Ctrl
10413771	Capn7	NM_009796	3.06259e-005	1.46695	E15.5 * Wnt1 up vs E15.5 * Ctrl
10388847	Sarm1	NM_172795	1.58595e-005	1.46685	E15.5 * Wnt1 up vs E15.5 * Ctrl
10423355	March11	NM_177597	0.00289159	1.4656	E15.5 * Wnt1 up vs E15.5 * Ctrl
10446001	Fsd1	NM_183178	0.000739405	1.465	E15.5 * Wnt1 up vs E15.5 * Ctrl
10440491	App	NM_007471	0.000126505	1.46494	E15.5 * Wnt1 up vs E15.5 * Ctrl
10594447	Map2k1	NM_008927	2.89484e-005	1.46465	E15.5 * Wnt1 up vs E15.5 * Ctrl
10474141	Slc1a2	NM_001077514	0.000675853	1.46464	E15.5 * Wnt1 up vs E15.5 * Ctrl
10479925	5430407P10Rik	NM_001159657	9.49343e-007	1.46458	E15.5 * Wnt1 up vs E15.5 * Ctrl
10390635	1810046J19Rik	NM_025559	0.00022133	1.46419	E15.5 * Wnt1 up vs E15.5 * Ctrl
10382341	Sstr2	NM_009217	0.0117872	1.4639	E15.5 * Wnt1 up vs E15.5 * Ctrl
10440206	Arl6	NM_019665	0.0040331	1.46366	E15.5 * Wnt1 up vs E15.5 * Ctrl
10549504	Dennd5b	NM_177192	0.00511055	1.4631	E15.5 * Wnt1 up vs E15.5 * Ctrl
10359255	Fam5b	NM_207583	0.000315548	1.46287	E15.5 * Wnt1 up vs E15.5 * Ctrl
10471080	Usp20	NM_028846	0.0025366	1.46275	E15.5 * Wnt1 up vs E15.5 * Ctrl
10535739	Usp12	NM_011669	0.000195712	1.46243	E15.5 * Wnt1 up vs E15.5 * Ctrl
10587627	Cyb5r4	NM_024195	1.91114e-005	1.46232	E15.5 * Wnt1 up vs E15.5 * Ctrl
10351140	Kifap3	NM_010629	9.01482e-007	1.46196	E15.5 * Wnt1 up vs E15.5 * Ctrl
10481857	Pbx3	NM_016768	5.19453e-006	1.46196	E15.5 * Wnt1 up vs E15.5 * Ctrl
10476207	Atrn	NM_009730	4.06979e-006	1.46127	E15.5 * Wnt1 up vs E15.5 * Ctrl
10553261	Kcnc1	NM_008421	0.000587371	1.46117	E15.5 * Wnt1 up vs E15.5 * Ctrl
10477725	Mmp24	NM_010808	2.06497e-005	1.461	E15.5 * Wnt1 up vs E15.5 * Ctrl
10390860	Krt23	NM_033373	0.00528727	1.46099	E15.5 * Wnt1 up vs E15.5 * Ctrl
10345571	Cnga3	NM_009918	9.29071e-005	1.46075	E15.5 * Wnt1 up vs E15.5 * Ctrl
10431154	Phf21b	NM_001081166	0.000110938	1.45943	E15.5 * Wnt1 up vs E15.5 * Ctrl
10606475	Hdx	NM_001080549	3.17215e-006	1.45911	E15.5 * Wnt1 up vs E15.5 * Ctrl
10404195	D130043K22Rik	NM_001081051	1.59592e-006	1.45889	E15.5 * Wnt1 up vs E15.5 * Ctrl
10501121	Fam40a	NM_153563	0.000100734	1.45865	E15.5 * Wnt1 up vs E15.5 * Ctrl
10459827	Rnf165	ENSMUST00000(	7.88798e-006	1.45855	E15.5 * Wnt1 up vs E15.5 * Ctrl
10596347	Atp2c1	NM_175025	3.23418e-007	1.45842	E15.5 * Wnt1 up vs E15.5 * Ctrl
10457872	Slc39a6	NM_139143	5.16746e-007	1.45842	E15.5 * Wnt1 up vs E15.5 * Ctrl
10515003	Fam159a	NM_001099303	0.00111102	1.45698	E15.5 * Wnt1 up vs E15.5 * Ctrl
10562408	Chst8	NM_175140	6.16388e-005	1.45689	E15.5 * Wnt1 up vs E15.5 * Ctrl
10604637	Cxx1b	NM_001018063	4.19801e-007	1.4565	E15.5 * Wnt1 up vs E15.5 * Ctrl
10459747	Mapk4	NM_172632	0.00075291	1.45649	E15.5 * Wnt1 up vs E15.5 * Ctrl
10482144	Rc3h2	NM_001100591	4.76461e-006	1.45615	E15.5 * Wnt1 up vs E15.5 * Ctrl
10432492	Faim2	NM_028224	0.000460585	1.45586	E15.5 * Wnt1 up vs E15.5 * Ctrl
10407833	Ggps1	NM_010282	0.000121704	1.45584	E15.5 * Wnt1 up vs E15.5 * Ctrl
10456756	Zbtb7c	NM_145356	5.30237e-005	1.45485	E15.5 * Wnt1 up vs E15.5 * Ctrl
10418901	Gprin2	ENSMUST00000(	0.00385475	1.4548	E15.5 * Wnt1 up vs E15.5 * Ctrl
10597883	Ano10	NM_133979	0.00630768	1.4547	E15.5 * Wnt1 up vs E15.5 * Ctrl
10374560	Zrsr1	NM_011663	0.000331706	1.45446	E15.5 * Wnt1 up vs E15.5 * Ctrl
10354768	Akr1b3	NM_009658	7.57641e-007	1.45424	E15.5 * Wnt1 up vs E15.5 * Ctrl
10594679	Tln2	NM_001081242	2.70391e-005	1.45418	E15.5 * Wnt1 up vs E15.5 * Ctrl
10412667	Ptprg	NM_008981	4.65735e-005	1.45382	E15.5 * Wnt1 up vs E15.5 * Ctrl
10499652	4632404H12Rik	ENSMUST00000(	0.000355033	1.45372	E15.5 * Wnt1 up vs E15.5 * Ctrl
10557285	Lcmt1	NM_025304	0.000306811	1.45361	E15.5 * Wnt1 up vs E15.5 * Ctrl
10439092	1700021K19Rik	BC060601	2.11845e-005	1.45344	E15.5 * Wnt1 up vs E15.5 * Ctrl
10494857	Nras	NM_010937	1.92654e-007	1.45263	E15.5 * Wnt1 up vs E15.5 * Ctrl
10468795	Rab11fip2	NM_001033172	0.00212033	1.45181	E15.5 * Wnt1 up vs E15.5 * Ctrl
10357858	Optc	NM_054076	0.00341994	1.45145	E15.5 * Wnt1 up vs E15.5 * Ctrl
10352358	Cnih3	NM_028408	0.00234331	1.45139	E15.5 * Wnt1 up vs E15.5 * Ctrl
10397030	Rgs6	NM_015812	0.000124553	1.45124	E15.5 * Wnt1 up vs E15.5 * Ctrl
10539710	Tia1	NM_011585	2.14888e-006	1.4506	E15.5 * Wnt1 up vs E15.5 * Ctrl
10380672	Skap1	NM_001033186	0.000325893	1.45048	E15.5 * Wnt1 up vs E15.5 * Ctrl
10590860	9030420J04Rik	BC137891	0.000391269	1.45042	E15.5 * Wnt1 up vs E15.5 * Ctrl
10505008	Slc44a1	NM_133891	8.88572e-007	1.45038	E15.5 * Wnt1 up vs E15.5 * Ctrl
10537851	Cntnap2	NM_001004357	2.39414e-005	1.4503	E15.5 * Wnt1 up vs E15.5 * Ctrl
10592126	Fam118b	NM_194257	8.90862e-006	1.45024	E15.5 * Wnt1 up vs E15.5 * Ctrl
10583929	B3gat1	NM_029792	7.71457e-005	1.45013	E15.5 * Wnt1 up vs E15.5 * Ctrl
10449034	Fam173a	BC096050	0.00014668	1.44963	E15.5 * Wnt1 up vs E15.5 * Ctrl
10584524	1700001J11Rik	XR_001606	0.00647323	1.44954	E15.5 * Wnt1 up vs E15.5 * Ctrl
10567316	Tmc7	NM_172476	0.000263861	1.44954	E15.5 * Wnt1 up vs E15.5 * Ctrl
10569291	Krtap5-2	NM_027844	0.00537363	1.44951	E15.5 * Wnt1 up vs E15.5 * Ctrl

10574204	Arl2bp	NM_024191	8.57392e-007	1.44927	E15.5 * Wnt1 up vs E15.5 * Ctrl
10499160	Cd1d1	NM_007639	0.00811135	1.44916	E15.5 * Wnt1 up vs E15.5 * Ctrl
10544746	Hoxa1	NM_010449	0.00357354	1.449	E15.5 * Wnt1 up vs E15.5 * Ctrl
10523161	Mthfd2l	NM_026788	0.0058952	1.44851	E15.5 * Wnt1 up vs E15.5 * Ctrl
10445107	EG224763	ENSMUST00000(	1.1664e-005	1.44839	E15.5 * Wnt1 up vs E15.5 * Ctrl
10421932	Pcdh9	NM_001081377	0.00559175	1.44789	E15.5 * Wnt1 up vs E15.5 * Ctrl
10413250	Cphx	NM_175342	0.00076842	1.44758	E15.5 * Wnt1 up vs E15.5 * Ctrl
10598933	Usp11	NM_145628	5.34122e-005	1.44749	E15.5 * Wnt1 up vs E15.5 * Ctrl
10349184	Cdh7	NM_172853	2.34318e-006	1.44739	E15.5 * Wnt1 up vs E15.5 * Ctrl
10415842		---	0.00460859	1.44737	E15.5 * Wnt1 up vs E15.5 * Ctrl
10404928	C78339	NM_001033192	1.28671e-006	1.44731	E15.5 * Wnt1 up vs E15.5 * Ctrl
10543859	Akr1b3	NM_009658	1.2324e-006	1.44666	E15.5 * Wnt1 up vs E15.5 * Ctrl
10479297	Lsm14b	NM_177727	0.000107717	1.44636	E15.5 * Wnt1 up vs E15.5 * Ctrl
10448235	A630033E08Rik	NM_001110254	0.000357955	1.44629	E15.5 * Wnt1 up vs E15.5 * Ctrl
10513103	6430704M03Rik	NM_001142965	0.000670931	1.44624	E15.5 * Wnt1 up vs E15.5 * Ctrl
10511014		---	0.00129631	1.44596	E15.5 * Wnt1 up vs E15.5 * Ctrl
10390252	OTTMUSG00000	ENSMUST00000	0.000304187	1.44542	E15.5 * Wnt1 up vs E15.5 * Ctrl
10586505	Herc1	BC004027	7.57682e-007	1.44525	E15.5 * Wnt1 up vs E15.5 * Ctrl
10458890		---	0.00321439	1.44518	E15.5 * Wnt1 up vs E15.5 * Ctrl
10494170	Lysmd1	NM_153121	8.5133e-005	1.4449	E15.5 * Wnt1 up vs E15.5 * Ctrl
10445235	ENSMUSG0000C	ENSMUST00000(	0.00294539	1.44436	E15.5 * Wnt1 up vs E15.5 * Ctrl
10386775	Prpsap2	NM_144806	0.0027177	1.44405	E15.5 * Wnt1 up vs E15.5 * Ctrl
10445941	100043485	XR_033353	0.000108302	1.44396	E15.5 * Wnt1 up vs E15.5 * Ctrl
10450605	Tubb5	NM_011655	2.57034e-005	1.44375	E15.5 * Wnt1 up vs E15.5 * Ctrl
10565689	Capn5	NM_007602	0.000196169	1.44373	E15.5 * Wnt1 up vs E15.5 * Ctrl
10393272	Rnf157	BC053070	0.00032686	1.44215	E15.5 * Wnt1 up vs E15.5 * Ctrl
10547597	Phc1	NM_007905	1.84103e-005	1.44197	E15.5 * Wnt1 up vs E15.5 * Ctrl
10544062	D630045J12Rik	ENSMUST00000	2.68297e-006	1.44195	E15.5 * Wnt1 up vs E15.5 * Ctrl
10487937	Prokr2	NM_144944	0.000326313	1.4414	E15.5 * Wnt1 up vs E15.5 * Ctrl
10564157		---	0.00689203	1.44129	E15.5 * Wnt1 up vs E15.5 * Ctrl
10549760	Zfp580	BC066001	0.00815151	1.44105	E15.5 * Wnt1 up vs E15.5 * Ctrl
10530215	1110003E01Rik	NM_133697	1.34489e-005	1.4409	E15.5 * Wnt1 up vs E15.5 * Ctrl
10441073	Kcnj6	NM_010606	4.75293e-005	1.44073	E15.5 * Wnt1 up vs E15.5 * Ctrl
10471129	Freq	NM_019681	0.00316328	1.44062	E15.5 * Wnt1 up vs E15.5 * Ctrl
10392207	Tex2	NM_198292	0.000714621	1.44051	E15.5 * Wnt1 up vs E15.5 * Ctrl
10344658	Rb1cc1	NM_009826	0.000117942	1.43986	E15.5 * Wnt1 up vs E15.5 * Ctrl
10403964	Zfp184	NM_183014	0.0092978	1.43971	E15.5 * Wnt1 up vs E15.5 * Ctrl
10580537	Aktip	NM_010241	0.000524657	1.43958	E15.5 * Wnt1 up vs E15.5 * Ctrl
10394625		---	0.00416543	1.43952	E15.5 * Wnt1 up vs E15.5 * Ctrl
10438791	Uts2d	NM_198166	0.000740316	1.43895	E15.5 * Wnt1 up vs E15.5 * Ctrl
10456378	Chmp1b	NM_024190	0.00301378	1.4389	E15.5 * Wnt1 up vs E15.5 * Ctrl
10501183	4933431E20Rik	BC060136	0.00104815	1.43873	E15.5 * Wnt1 up vs E15.5 * Ctrl
10582551	Dbndd1	NM_028146	4.71846e-009	1.43851	E15.5 * Wnt1 up vs E15.5 * Ctrl
10421418	Epb4.9	NM_013514	7.80348e-006	1.43845	E15.5 * Wnt1 up vs E15.5 * Ctrl
10444895	Flot1	NM_008027	0.000136655	1.43829	E15.5 * Wnt1 up vs E15.5 * Ctrl
10473118	Ube2e3	NM_009454	7.72631e-007	1.43801	E15.5 * Wnt1 up vs E15.5 * Ctrl
10447594	Dynlt1	NM_009342	2.96383e-010	1.43789	E15.5 * Wnt1 up vs E15.5 * Ctrl
10413238	Cphx	NM_175342	0.00105077	1.43786	E15.5 * Wnt1 up vs E15.5 * Ctrl
10413260	Cphx	NM_175342	0.00105077	1.43786	E15.5 * Wnt1 up vs E15.5 * Ctrl
10510687	Acot7	NM_133348	0.000172656	1.43777	E15.5 * Wnt1 up vs E15.5 * Ctrl
10530783	A730089K16Rik	ENSMUST00000(	0.000426092	1.43693	E15.5 * Wnt1 up vs E15.5 * Ctrl
10546736	Cntn3	NM_008779	0.00353103	1.43679	E15.5 * Wnt1 up vs E15.5 * Ctrl
10545940	Gmcl1	NM_011818	5.54695e-009	1.43658	E15.5 * Wnt1 up vs E15.5 * Ctrl
10504402	4930500O05Rik	NM_001085508	6.89856e-005	1.43644	E15.5 * Wnt1 up vs E15.5 * Ctrl
10593713	Cib2	NM_019686	9.11858e-005	1.4364	E15.5 * Wnt1 up vs E15.5 * Ctrl
10468517	Mxi1	NM_010847	0.000757787	1.43624	E15.5 * Wnt1 up vs E15.5 * Ctrl
10607113	Rgs3	NM_134257	0.000792896	1.43578	E15.5 * Wnt1 up vs E15.5 * Ctrl
10597918		---	0.000363801	1.43569	E15.5 * Wnt1 up vs E15.5 * Ctrl
10585494	Ube2q2	NM_180600	4.09815e-005	1.43532	E15.5 * Wnt1 up vs E15.5 * Ctrl
10350473	B3galt2	NM_020025	0.00119564	1.4349	E15.5 * Wnt1 up vs E15.5 * Ctrl
10441511			2.97785e-005	1.43482	E15.5 * Wnt1 up vs E15.5 * Ctrl
10594001	Arid3b	NM_019689	2.5679e-005	1.43359	E15.5 * Wnt1 up vs E15.5 * Ctrl
10431170	5031439G07Rik	NM_001033273	0.000309856	1.43353	E15.5 * Wnt1 up vs E15.5 * Ctrl
10400538	Trappc6b	NM_030057	2.24272e-005	1.43277	E15.5 * Wnt1 up vs E15.5 * Ctrl
10482846	Ccdc148	BC062650	0.00286926	1.43211	E15.5 * Wnt1 up vs E15.5 * Ctrl
10541098	Zfp239	NM_001001792	7.08064e-007	1.43172	E15.5 * Wnt1 up vs E15.5 * Ctrl

10501358	Sars	NM_011319	4.99159e-006	1.43145	E15.5 * Wnt1 up vs E15.5 * Ctrl
10352503	Bpnt1	NM_011794	0.00121952	1.43123	E15.5 * Wnt1 up vs E15.5 * Ctrl
10377018	Myh3	NM_001099635	7.32414e-006	1.4312	E15.5 * Wnt1 up vs E15.5 * Ctrl
10492231	Med12l	ENSMUST00000	0.000330983	1.43119	E15.5 * Wnt1 up vs E15.5 * Ctrl
10573566	Dhps	NM_001039514	5.95324e-006	1.4298	E15.5 * Wnt1 up vs E15.5 * Ctrl
10524955	Tesc	NM_021344	0.000930456	1.42979	E15.5 * Wnt1 up vs E15.5 * Ctrl
10416505	Kctd4	NM_026214	0.00316526	1.42966	E15.5 * Wnt1 up vs E15.5 * Ctrl
10549849	Zfp78	NM_001025163	0.000139953	1.42895	E15.5 * Wnt1 up vs E15.5 * Ctrl
10373728	Patz1	NM_019574	0.00233941	1.42894	E15.5 * Wnt1 up vs E15.5 * Ctrl
10483163	Grb14	NM_016719	0.0111135	1.42885	E15.5 * Wnt1 up vs E15.5 * Ctrl
10527306	Lmtk2	NM_001081109	2.44918e-005	1.42855	E15.5 * Wnt1 up vs E15.5 * Ctrl
10574404	Setd6	NM_001035123	0.000506731	1.4284	E15.5 * Wnt1 up vs E15.5 * Ctrl
10475567	Slc24a5	NM_175034	9.87144e-005	1.42837	E15.5 * Wnt1 up vs E15.5 * Ctrl
10485048	D2Ertd391e	BC023673	0.000171459	1.42819	E15.5 * Wnt1 up vs E15.5 * Ctrl
10605247	Mecp2	NM_010788	6.46133e-005	1.42793	E15.5 * Wnt1 up vs E15.5 * Ctrl
10358057	Shisa4	NM_175259	5.68897e-007	1.42771	E15.5 * Wnt1 up vs E15.5 * Ctrl
10527886	Pex1	NM_027777	0.0122199	1.42744	E15.5 * Wnt1 up vs E15.5 * Ctrl
10476596	Macrod2	NM_001013802	0.00110436	1.42689	E15.5 * Wnt1 up vs E15.5 * Ctrl
10398483	Dync1h1	NM_030238	9.90009e-006	1.42654	E15.5 * Wnt1 up vs E15.5 * Ctrl
10412773	Slc4a7	NM_001033270	0.000220286	1.42605	E15.5 * Wnt1 up vs E15.5 * Ctrl
10373407	Mbc2	NM_011843	0.00315848	1.42597	E15.5 * Wnt1 up vs E15.5 * Ctrl
10538755	Smarcad1	NM_007958	4.24033e-007	1.42594	E15.5 * Wnt1 up vs E15.5 * Ctrl
10523595	Ptpn13	NM_011204	8.50119e-007	1.42582	E15.5 * Wnt1 up vs E15.5 * Ctrl
10388154	Med31	NM_026068	0.00508791	1.42565	E15.5 * Wnt1 up vs E15.5 * Ctrl
10532586	Myo18b	BC145218	0.00490015	1.42562	E15.5 * Wnt1 up vs E15.5 * Ctrl
10462035	Ldhb	NM_008492	8.73027e-006	1.42555	E15.5 * Wnt1 up vs E15.5 * Ctrl
10574259	Gpr56	NM_018882	4.42851e-005	1.42532	E15.5 * Wnt1 up vs E15.5 * Ctrl
10519482	EG667705	ENSMUST00000	0.000425292	1.42498	E15.5 * Wnt1 up vs E15.5 * Ctrl
10501909	Mettl14	NM_201638	6.73489e-007	1.42483	E15.5 * Wnt1 up vs E15.5 * Ctrl
10597377	Fbxl2	NM_178624	1.40812e-006	1.4248	E15.5 * Wnt1 up vs E15.5 * Ctrl
10400004		---	0.0124836	1.42435	E15.5 * Wnt1 up vs E15.5 * Ctrl
10541877	Vamp1	NM_001080557	0.000494605	1.42384	E15.5 * Wnt1 up vs E15.5 * Ctrl
10441864	Mllt4	NM_010806	2.17197e-006	1.42382	E15.5 * Wnt1 up vs E15.5 * Ctrl
10577858	Bag4	NM_026121	1.0795e-005	1.42368	E15.5 * Wnt1 up vs E15.5 * Ctrl
10442616	Hagh	NM_024284	0.000192387	1.42337	E15.5 * Wnt1 up vs E15.5 * Ctrl
10377725	Dlg4	NM_007864	0.00035577	1.42319	E15.5 * Wnt1 up vs E15.5 * Ctrl
10563852	Nipa1	NM_153578	0.00341808	1.42294	E15.5 * Wnt1 up vs E15.5 * Ctrl
10593628	Rab39	NM_175562	0.000327958	1.42289	E15.5 * Wnt1 up vs E15.5 * Ctrl
10501629	Cdc14a	NM_001080818	5.01972e-006	1.42272	E15.5 * Wnt1 up vs E15.5 * Ctrl
10362630		---	0.00215991	1.42224	E15.5 * Wnt1 up vs E15.5 * Ctrl
10516427	Ncdn	NM_011986	0.000956721	1.42206	E15.5 * Wnt1 up vs E15.5 * Ctrl
10439138	Kalrn	BC157950	0.00084021	1.42202	E15.5 * Wnt1 up vs E15.5 * Ctrl
10383681	Patz1	NM_019574	1.24046e-007	1.42194	E15.5 * Wnt1 up vs E15.5 * Ctrl
10387483	Efnb3	NM_007911	0.0012536	1.42191	E15.5 * Wnt1 up vs E15.5 * Ctrl
10391732	Gpatch8	NM_001159492	4.6306e-005	1.42158	E15.5 * Wnt1 up vs E15.5 * Ctrl
10591241	Zfp426	NM_146221	0.000500397	1.42112	E15.5 * Wnt1 up vs E15.5 * Ctrl
10550514	Nova2	NM_001029877	7.82483e-005	1.42106	E15.5 * Wnt1 up vs E15.5 * Ctrl
10604799	Atp11c	NM_001037863	0.000738806	1.42029	E15.5 * Wnt1 up vs E15.5 * Ctrl
10529708	Zfp518b	NM_001081144	0.000210372	1.42011	E15.5 * Wnt1 up vs E15.5 * Ctrl
10561854	Tbcb	NM_025548	7.68467e-005	1.42006	E15.5 * Wnt1 up vs E15.5 * Ctrl
10407907	Rala	NM_019491	0.00137933	1.41972	E15.5 * Wnt1 up vs E15.5 * Ctrl
10494208	Fam63a	NM_133858	2.10357e-005	1.4191	E15.5 * Wnt1 up vs E15.5 * Ctrl
10394794	EG245297	BC030401	0.00720208	1.41904	E15.5 * Wnt1 up vs E15.5 * Ctrl
10485546	D430041D05Rik	NM_001033347	0.000598949	1.41888	E15.5 * Wnt1 up vs E15.5 * Ctrl
10590433	1700048O20Rik	BC048726	0.000343798	1.4188	E15.5 * Wnt1 up vs E15.5 * Ctrl
10511725	Cyb5r4	NM_024195	6.90477e-005	1.41807	E15.5 * Wnt1 up vs E15.5 * Ctrl
10461459	Syt7	NM_173068	1.25253e-005	1.41797	E15.5 * Wnt1 up vs E15.5 * Ctrl
10571045	Bag4	NM_026121	3.52705e-005	1.41789	E15.5 * Wnt1 up vs E15.5 * Ctrl
10434418	Vwa5b2	NM_182636	8.11397e-006	1.41721	E15.5 * Wnt1 up vs E15.5 * Ctrl
10521995	3110047P20Rik	NM_177006	0.00591928	1.41711	E15.5 * Wnt1 up vs E15.5 * Ctrl
10475990	Slc20a1	NM_015747	3.27685e-005	1.41649	E15.5 * Wnt1 up vs E15.5 * Ctrl
10604687	Mmgt1	NM_146234	6.96842e-005	1.41631	E15.5 * Wnt1 up vs E15.5 * Ctrl
10380219	Vezf1	NM_016686	2.90974e-007	1.41604	E15.5 * Wnt1 up vs E15.5 * Ctrl
10446785	Spast	NM_016962	1.37412e-009	1.41578	E15.5 * Wnt1 up vs E15.5 * Ctrl
10411171	Pde8b	NM_172263	2.17514e-005	1.41552	E15.5 * Wnt1 up vs E15.5 * Ctrl
10457844	Zfp191	NM_021559	6.72941e-007	1.41521	E15.5 * Wnt1 up vs E15.5 * Ctrl

10369479	Lrrc20	ENSMUST00000(	0.000126134	1.4149	E15.5 * Wnt1 up vs E15.5 * Ctrl
10357339	Lypd1	NM_145100	0.000582325	1.41462	E15.5 * Wnt1 up vs E15.5 * Ctrl
10429218	Fam135b	NM_177819	6.99454e-005	1.41437	E15.5 * Wnt1 up vs E15.5 * Ctrl
10549854	Zfp28	NM_175247	0.000154831	1.4143	E15.5 * Wnt1 up vs E15.5 * Ctrl
10601915	BC031748	BC031748	2.20316e-005	1.41426	E15.5 * Wnt1 up vs E15.5 * Ctrl
10560945	Grik5	NM_008168	0.00155057	1.41424	E15.5 * Wnt1 up vs E15.5 * Ctrl
10454851	Cxxc5	NM_133687	0.00023225	1.41388	E15.5 * Wnt1 up vs E15.5 * Ctrl
10473045	Rbm45	NM_153405	1.55528e-005	1.41386	E15.5 * Wnt1 up vs E15.5 * Ctrl
10377984	Zfp3	NM_177565	0.000109039	1.41369	E15.5 * Wnt1 up vs E15.5 * Ctrl
10514658	Itgb3bp	ENSMUST00000(	1.06319e-005	1.41354	E15.5 * Wnt1 up vs E15.5 * Ctrl
10396278	Daam1	NM_026102	2.229e-005	1.41341	E15.5 * Wnt1 up vs E15.5 * Ctrl
10412844	Top2b	NM_009409	7.3299e-007	1.41312	E15.5 * Wnt1 up vs E15.5 * Ctrl
10476301	Smox	NM_145533	0.00349107	1.41306	E15.5 * Wnt1 up vs E15.5 * Ctrl
10454632	Camk4	NM_009793	0.00337415	1.41293	E15.5 * Wnt1 up vs E15.5 * Ctrl
10503534	Ccnc	NM_016746	0.00559951	1.4127	E15.5 * Wnt1 up vs E15.5 * Ctrl
10578574	Stox2	NM_175162	0.00459901	1.41261	E15.5 * Wnt1 up vs E15.5 * Ctrl
10559766	Zfp583	NM_001033249	0.00353408	1.41259	E15.5 * Wnt1 up vs E15.5 * Ctrl
10508651	Sdc3	NM_011520	0.00196865	1.41219	E15.5 * Wnt1 up vs E15.5 * Ctrl
10558742	Nlrp6	NM_001081389	3.03333e-005	1.41199	E15.5 * Wnt1 up vs E15.5 * Ctrl
10532588	Myo18b	BC145218	0.000865566	1.41192	E15.5 * Wnt1 up vs E15.5 * Ctrl
10471953	Acvr2a	NM_007396	4.924e-007	1.4119	E15.5 * Wnt1 up vs E15.5 * Ctrl
10427253	Map3k12	NM_009582	0.00112421	1.41183	E15.5 * Wnt1 up vs E15.5 * Ctrl
10447100	Morn2	NM_194269	0.000457489	1.41127	E15.5 * Wnt1 up vs E15.5 * Ctrl
10375175	Slit3	NM_011412	9.10064e-005	1.41124	E15.5 * Wnt1 up vs E15.5 * Ctrl
10456405	Slmo1	NM_144867	0.000493863	1.41118	E15.5 * Wnt1 up vs E15.5 * Ctrl
10532578	Myo18b	BC145218	0.000126109	1.41083	E15.5 * Wnt1 up vs E15.5 * Ctrl
10519270	Agrn	NM_021604	0.00923214	1.41075	E15.5 * Wnt1 up vs E15.5 * Ctrl
10445071	Zfp57	NM_001013745	1.34908e-005	1.41067	E15.5 * Wnt1 up vs E15.5 * Ctrl
10548785	Dynlt1	NM_009342	8.52211e-010	1.41046	E15.5 * Wnt1 up vs E15.5 * Ctrl
10420787	Mtmr9	NM_177594	0.00021293	1.41041	E15.5 * Wnt1 up vs E15.5 * Ctrl
10384797	Ccdc85a	NM_181577	0.000127757	1.41038	E15.5 * Wnt1 up vs E15.5 * Ctrl
10578763	Sap30	NM_021788	6.49835e-007	1.41024	E15.5 * Wnt1 up vs E15.5 * Ctrl
10368197	ENSMUSG0000C	ENSMUST00000	0.00553984	1.40949	E15.5 * Wnt1 up vs E15.5 * Ctrl
10568252	Zfp689	NM_175163	0.000783612	1.40927	E15.5 * Wnt1 up vs E15.5 * Ctrl
10572613	Mrpl34	NM_053162	0.00678401	1.40903	E15.5 * Wnt1 up vs E15.5 * Ctrl
10601492	Zfp711	NM_177747	0.00262487	1.40899	E15.5 * Wnt1 up vs E15.5 * Ctrl
10483025	Rbms1	NM_001141932	5.96311e-007	1.40888	E15.5 * Wnt1 up vs E15.5 * Ctrl
10389674	Dynll2	NM_026556	2.69452e-007	1.40885	E15.5 * Wnt1 up vs E15.5 * Ctrl
10358658	Hmcn1	NM_001024720	0.000418032	1.40873	E15.5 * Wnt1 up vs E15.5 * Ctrl
10570236	Mcf2l	NM_178076	2.55799e-005	1.40859	E15.5 * Wnt1 up vs E15.5 * Ctrl
10395538	Pnpla8	NM_026164	1.33677e-005	1.40823	E15.5 * Wnt1 up vs E15.5 * Ctrl
10579996	Gpsn2	NM_134118	0.000101781	1.40806	E15.5 * Wnt1 up vs E15.5 * Ctrl
10367697	Ppp1r14c	NM_133485	2.55515e-005	1.40798	E15.5 * Wnt1 up vs E15.5 * Ctrl
10391146	Acly	NM_134037	0.00144237	1.40796	E15.5 * Wnt1 up vs E15.5 * Ctrl
10471247	Aif1l	NM_145144	0.00211504	1.40789	E15.5 * Wnt1 up vs E15.5 * Ctrl
10544941	Pde1c	NM_011054	0.000259757	1.40775	E15.5 * Wnt1 up vs E15.5 * Ctrl
10384672	Ahsa2	NM_172391	3.20074e-006	1.40768	E15.5 * Wnt1 up vs E15.5 * Ctrl
10426284	Acr	NM_013455	7.7755e-005	1.40759	E15.5 * Wnt1 up vs E15.5 * Ctrl
10428020	March6	NM_172606	1.59756e-005	1.40751	E15.5 * Wnt1 up vs E15.5 * Ctrl
10428232	Rrm2b	NM_199476	5.51293e-005	1.40698	E15.5 * Wnt1 up vs E15.5 * Ctrl
10476355	Chgb	NM_007694	0.00988184	1.40678	E15.5 * Wnt1 up vs E15.5 * Ctrl
10467091	Atad1	NM_026487	8.46755e-005	1.40653	E15.5 * Wnt1 up vs E15.5 * Ctrl
10545014	AW146242	BC024822	0.00058588	1.40648	E15.5 * Wnt1 up vs E15.5 * Ctrl
10455826	Megf10	NM_001001979	0.00451793	1.40584	E15.5 * Wnt1 up vs E15.5 * Ctrl
10601846	2900062L11Rik	NR_003642	0.00262764	1.40562	E15.5 * Wnt1 up vs E15.5 * Ctrl
10439063	Fbxo45	NM_173439	3.01831e-005	1.40534	E15.5 * Wnt1 up vs E15.5 * Ctrl
10415074	Rem2	NM_080726	0.00114017	1.40525	E15.5 * Wnt1 up vs E15.5 * Ctrl
10372831	Tbc1d30	BC138285	0.00113346	1.40446	E15.5 * Wnt1 up vs E15.5 * Ctrl
10384715	LOC100134990	NR_004857	0.00310695	1.40437	E15.5 * Wnt1 up vs E15.5 * Ctrl
10362147	Taar1	NM_053205	0.00075588	1.40436	E15.5 * Wnt1 up vs E15.5 * Ctrl
10506883	Rab3b	NM_023537	0.000615082	1.40413	E15.5 * Wnt1 up vs E15.5 * Ctrl
10395463	Ifrd1	NM_013562	0.000960474	1.40398	E15.5 * Wnt1 up vs E15.5 * Ctrl
10368227	Ube2q2	NM_180600	0.000184572	1.40373	E15.5 * Wnt1 up vs E15.5 * Ctrl
10399224	Kif3c	NM_008445	0.00527522	1.40371	E15.5 * Wnt1 up vs E15.5 * Ctrl
10353871	Lman2l	NM_001013374	0.00454115	1.40364	E15.5 * Wnt1 up vs E15.5 * Ctrl
10401422	Pnma1	NM_027438	0.000199022	1.40326	E15.5 * Wnt1 up vs E15.5 * Ctrl

10521163	Gm1673	BC147444	0.000192675	1.40298	E15.5 * Wnt1 up vs E15.5 * Ctrl
10577240	Csmd1	NM_053171	2.3805e-005	1.40283	E15.5 * Wnt1 up vs E15.5 * Ctrl
10372177	Tmtc2	NM_177368	6.51451e-005	1.40277	E15.5 * Wnt1 up vs E15.5 * Ctrl
10404376	Agtr1a	NM_177322	4.08268e-005	1.40269	E15.5 * Wnt1 up vs E15.5 * Ctrl
10456587	Mro	NM_027741	0.000470057	1.40253	E15.5 * Wnt1 up vs E15.5 * Ctrl
10555946	Smpd1	NM_011421	0.00386131	1.40249	E15.5 * Wnt1 up vs E15.5 * Ctrl
10529797		---	0.0003874	1.40248	E15.5 * Wnt1 up vs E15.5 * Ctrl
10437080	Ttc3	NM_009441	2.07521e-008	1.40187	E15.5 * Wnt1 up vs E15.5 * Ctrl
10525877	Zfp664	NM_001081750	2.51787e-005	1.40186	E15.5 * Wnt1 up vs E15.5 * Ctrl
10556640	6330503K22Rik	NM_182995	8.6206e-005	1.40171	E15.5 * Wnt1 up vs E15.5 * Ctrl
10576692	Insr	NM_010568	4.22642e-005	1.40164	E15.5 * Wnt1 up vs E15.5 * Ctrl
10399841	Cbll1	NM_134048	9.93304e-006	1.40133	E15.5 * Wnt1 up vs E15.5 * Ctrl
10374908	Rtn4	NM_194054	3.6661e-007	1.40127	E15.5 * Wnt1 up vs E15.5 * Ctrl
10392374	Cacng5	NM_080644	0.00374137	1.40062	E15.5 * Wnt1 up vs E15.5 * Ctrl
10412913	EG667344	XR_031629	0.000574331	1.40051	E15.5 * Wnt1 up vs E15.5 * Ctrl
10505213	E130308A19Rik	NM_001015681	1.17788e-005	1.4004	E15.5 * Wnt1 up vs E15.5 * Ctrl
10418895	Zfp488	NM_001013777	0.000319696	1.4003	E15.5 * Wnt1 up vs E15.5 * Ctrl
10423505	Cmbl	NM_181588	0.00469599	1.39985	E15.5 * Wnt1 up vs E15.5 * Ctrl
10389047	Accn1	NM_001034013	0.00783042	1.39938	E15.5 * Wnt1 up vs E15.5 * Ctrl
10552672	Aspdh	NM_026690	0.0012033	1.39926	E15.5 * Wnt1 up vs E15.5 * Ctrl
10445237	LOC627626	BC151166	0.00269432	1.39916	E15.5 * Wnt1 up vs E15.5 * Ctrl
10475264	Ccndbp1	NM_010761	0.000682167	1.39899	E15.5 * Wnt1 up vs E15.5 * Ctrl
10517328	Tmem50a	NM_027935	6.83816e-007	1.39897	E15.5 * Wnt1 up vs E15.5 * Ctrl
10593499	AI593442	NM_178906	0.000487086	1.39897	E15.5 * Wnt1 up vs E15.5 * Ctrl
10462957	Tbc1d12	NM_145952	0.0051667	1.39857	E15.5 * Wnt1 up vs E15.5 * Ctrl
10515924	Rimkla	NM_177572	0.000357243	1.3983	E15.5 * Wnt1 up vs E15.5 * Ctrl
10494536	Pdzk1	NM_021517	2.08724e-006	1.39807	E15.5 * Wnt1 up vs E15.5 * Ctrl
10392601	Abca6	NM_147218	0.00313099	1.39805	E15.5 * Wnt1 up vs E15.5 * Ctrl
10482139	Pdcl	NM_026176	0.00618272	1.39779	E15.5 * Wnt1 up vs E15.5 * Ctrl
10594988	Mapk6	NM_015806	1.96993e-007	1.39769	E15.5 * Wnt1 up vs E15.5 * Ctrl
10583163	Trpc6	NM_013838	0.000244996	1.39755	E15.5 * Wnt1 up vs E15.5 * Ctrl
10377534	A030009H04Rik	AB041807	7.49438e-005	1.39749	E15.5 * Wnt1 up vs E15.5 * Ctrl
10381960	ENSMUSG0000(	ENSMUST00000(	0.000344335	1.39725	E15.5 * Wnt1 up vs E15.5 * Ctrl
10371811	Scyl2	NM_198021	8.2083e-006	1.39717	E15.5 * Wnt1 up vs E15.5 * Ctrl
10514590	Dock7	NM_026082	5.86928e-008	1.3965	E15.5 * Wnt1 up vs E15.5 * Ctrl
10382866	Mgat5b	NM_172948	0.00092528	1.39642	E15.5 * Wnt1 up vs E15.5 * Ctrl
10503019	4930566N20Rik	ENSMUST00000(	9.03612e-006	1.39613	E15.5 * Wnt1 up vs E15.5 * Ctrl
10433672	Rrn3	NM_001039521	1.28984e-005	1.39601	E15.5 * Wnt1 up vs E15.5 * Ctrl
10561376	Dll3	NM_007866	0.000480572	1.39589	E15.5 * Wnt1 up vs E15.5 * Ctrl
10531437	Scarb2	NM_007644	0.000775489	1.39579	E15.5 * Wnt1 up vs E15.5 * Ctrl
10453887	Cables1	NM_001146287	7.4007e-005	1.39493	E15.5 * Wnt1 up vs E15.5 * Ctrl
10587194	Gnb5	NM_138719	0.00208066	1.39491	E15.5 * Wnt1 up vs E15.5 * Ctrl
10356628	Hdac4	NM_207225	0.000257711	1.39481	E15.5 * Wnt1 up vs E15.5 * Ctrl
10425335	Syngr1	NM_207708	6.88384e-005	1.39431	E15.5 * Wnt1 up vs E15.5 * Ctrl
10503995	Dnaja1	NM_008298	4.62117e-006	1.39409	E15.5 * Wnt1 up vs E15.5 * Ctrl
10512030	3110043O21Rik	BC076612	0.00215863	1.39387	E15.5 * Wnt1 up vs E15.5 * Ctrl
10578287	Cnot7	NM_011135	1.16848e-006	1.39377	E15.5 * Wnt1 up vs E15.5 * Ctrl
10439701	Abhd10	NM_172511	0.000171965	1.39353	E15.5 * Wnt1 up vs E15.5 * Ctrl
10542006	D6Wsu163e	NM_138594	1.18207e-005	1.39324	E15.5 * Wnt1 up vs E15.5 * Ctrl
10345879	Pou3f3	NM_008900	0.0037197	1.39289	E15.5 * Wnt1 up vs E15.5 * Ctrl
10578922	Klhl2	NM_178633	0.000580615	1.39276	E15.5 * Wnt1 up vs E15.5 * Ctrl
10544542	Zfp786	NM_177882	0.00132931	1.39266	E15.5 * Wnt1 up vs E15.5 * Ctrl
10449971	Zfp763	NM_028543	0.0119455	1.3924	E15.5 * Wnt1 up vs E15.5 * Ctrl
10512044	Lingo2	NM_175516	0.000424842	1.39237	E15.5 * Wnt1 up vs E15.5 * Ctrl
10410654		---	0.002161	1.39232	E15.5 * Wnt1 up vs E15.5 * Ctrl
10395672	Ap4s1	NM_021710	1.35384e-005	1.39169	E15.5 * Wnt1 up vs E15.5 * Ctrl
10533659	Clip1	NM_019765	1.70472e-007	1.39138	E15.5 * Wnt1 up vs E15.5 * Ctrl
10354267	A530098C11Rik	NM_001013799	0.01016	1.39135	E15.5 * Wnt1 up vs E15.5 * Ctrl
10591164		---	0.00705729	1.39134	E15.5 * Wnt1 up vs E15.5 * Ctrl
10584231	Pus3	NM_023292	0.00469914	1.39114	E15.5 * Wnt1 up vs E15.5 * Ctrl
10367122	Baz2a	NM_054078	4.81501e-007	1.39112	E15.5 * Wnt1 up vs E15.5 * Ctrl
10535883	Katnal1	NM_153572	0.00165076	1.39074	E15.5 * Wnt1 up vs E15.5 * Ctrl
10419460	Ttc5	NM_177625	0.000544746	1.39071	E15.5 * Wnt1 up vs E15.5 * Ctrl
10398267	Evl	NM_007965	3.30751e-007	1.39034	E15.5 * Wnt1 up vs E15.5 * Ctrl
10533320	Adam1b	NM_172125	0.00017996	1.39032	E15.5 * Wnt1 up vs E15.5 * Ctrl
10533403	Cux2	NM_007804	1.28365e-005	1.39017	E15.5 * Wnt1 up vs E15.5 * Ctrl

10597900	Zfp445	NM_173364	1.05081e-005	1.39007	E15.5 * Wnt1 up vs E15.5 * Ctrl
10447688	4930506C21Rik	AK043779	0.0108415	1.38971	E15.5 * Wnt1 up vs E15.5 * Ctrl
10401138	Atp6v1d	NM_023721	8.27497e-005	1.38961	E15.5 * Wnt1 up vs E15.5 * Ctrl
10592772	Abcg4	NM_138955	1.70593e-005	1.38958	E15.5 * Wnt1 up vs E15.5 * Ctrl
10388132	4933427D14Rik	BC043106	6.96393e-006	1.38952	E15.5 * Wnt1 up vs E15.5 * Ctrl
10406825	Ankra2	NM_023472	0.0032178	1.3893	E15.5 * Wnt1 up vs E15.5 * Ctrl
10578746	BC088983	BC088983	0.000753867	1.38928	E15.5 * Wnt1 up vs E15.5 * Ctrl
10386230	Rnf187	AB030190	0.000515366	1.38909	E15.5 * Wnt1 up vs E15.5 * Ctrl
10426242	Chkb	NM_007692	0.00508515	1.38908	E15.5 * Wnt1 up vs E15.5 * Ctrl
10412155	Ddx4	NM_001145885	0.00195023	1.389	E15.5 * Wnt1 up vs E15.5 * Ctrl
10419691	Mettl3	NM_019721	2.99155e-005	1.38851	E15.5 * Wnt1 up vs E15.5 * Ctrl
10582708	Exoc8	NM_198103	0.000889254	1.3884	E15.5 * Wnt1 up vs E15.5 * Ctrl
10401708	Ngb	NM_022414	5.24636e-005	1.38838	E15.5 * Wnt1 up vs E15.5 * Ctrl
10558454	Glrx3	NM_023140	0.00104162	1.38824	E15.5 * Wnt1 up vs E15.5 * Ctrl
10569898	Evi5l	NM_001039578	2.7517e-006	1.38792	E15.5 * Wnt1 up vs E15.5 * Ctrl
10401939	Lysmd1	NM_153121	0.000295257	1.38787	E15.5 * Wnt1 up vs E15.5 * Ctrl
10547686	Vmn2r22	NM_001104637	0.0109265	1.38773	E15.5 * Wnt1 up vs E15.5 * Ctrl
10407209	4833412L08Rik	ENSMUST00000(	0.00102774	1.38765	E15.5 * Wnt1 up vs E15.5 * Ctrl
10531794	Wdfy3	AB093277	0.000210479	1.38747	E15.5 * Wnt1 up vs E15.5 * Ctrl
10446013	Mpnd	NM_026530	0.0064868	1.38739	E15.5 * Wnt1 up vs E15.5 * Ctrl
10426425	Pdzrn4	BC151034	0.00946009	1.38704	E15.5 * Wnt1 up vs E15.5 * Ctrl
10590489	Zfp105	NM_009544	0.0014633	1.38691	E15.5 * Wnt1 up vs E15.5 * Ctrl
10407370	4833420G17Rik	NM_001113550	0.000863879	1.38691	E15.5 * Wnt1 up vs E15.5 * Ctrl
10409464	Dbn1	NM_019813	0.00150715	1.38665	E15.5 * Wnt1 up vs E15.5 * Ctrl
10564037		---	3.93568e-005	1.38655	E15.5 * Wnt1 up vs E15.5 * Ctrl
10595953	Fam62c	NM_177775	5.4728e-005	1.38648	E15.5 * Wnt1 up vs E15.5 * Ctrl
10400135		---	0.00263556	1.38639	E15.5 * Wnt1 up vs E15.5 * Ctrl
10455071	Pcdhb7	NM_053132	0.00873756	1.3863	E15.5 * Wnt1 up vs E15.5 * Ctrl
10590909	Endod1	NM_028013	0.000172496	1.38596	E15.5 * Wnt1 up vs E15.5 * Ctrl
10607524	Sms	NM_009214	2.70215e-006	1.38595	E15.5 * Wnt1 up vs E15.5 * Ctrl
10495001	Rsbn1	NM_172684	9.05451e-007	1.38579	E15.5 * Wnt1 up vs E15.5 * Ctrl
10582925	Alkbh8	NM_026303	0.00041505	1.38556	E15.5 * Wnt1 up vs E15.5 * Ctrl
10501754	D3Bwg0562e	NM_177664	0.00124347	1.38549	E15.5 * Wnt1 up vs E15.5 * Ctrl
10507731	Rims3	NM_182929	0.000551483	1.38476	E15.5 * Wnt1 up vs E15.5 * Ctrl
10471474	Ak1	NM_021515	0.00541616	1.38476	E15.5 * Wnt1 up vs E15.5 * Ctrl
10396346	1810048J11Rik	NM_026327	5.14205e-005	1.38475	E15.5 * Wnt1 up vs E15.5 * Ctrl
10593316		---	0.00837286	1.38448	E15.5 * Wnt1 up vs E15.5 * Ctrl
10351525	Mpz	NM_008623	0.000475107	1.38444	E15.5 * Wnt1 up vs E15.5 * Ctrl
10406856	Gm807	ENSMUST00000(	0.0040914	1.38388	E15.5 * Wnt1 up vs E15.5 * Ctrl
10511414		---	0.00562711	1.38352	E15.5 * Wnt1 up vs E15.5 * Ctrl
10368883	Tdg	NM_011561	1.0681e-005	1.38347	E15.5 * Wnt1 up vs E15.5 * Ctrl
10504551	Rg9mtd3	NM_027266	9.82548e-005	1.3831	E15.5 * Wnt1 up vs E15.5 * Ctrl
10425966	Atxn10	NM_016843	4.1252e-006	1.38298	E15.5 * Wnt1 up vs E15.5 * Ctrl
10485151	Mapk8ip1	NM_011162	0.00132075	1.3829	E15.5 * Wnt1 up vs E15.5 * Ctrl
10462228	Dmrt3	NM_177360	0.000314175	1.38262	E15.5 * Wnt1 up vs E15.5 * Ctrl
10575745	Atmin	NM_177700	0.000909262	1.3822	E15.5 * Wnt1 up vs E15.5 * Ctrl
10385236	Akr1b3	NM_009658	1.20165e-005	1.38198	E15.5 * Wnt1 up vs E15.5 * Ctrl
10512254	1110017D15Rik	NM_001048005	0.000189111	1.38144	E15.5 * Wnt1 up vs E15.5 * Ctrl
10528527	Fam126a	NM_053090	0.000240137	1.38133	E15.5 * Wnt1 up vs E15.5 * Ctrl
10491520	B230207O21Rik	AK140083	0.00137707	1.38132	E15.5 * Wnt1 up vs E15.5 * Ctrl
10491551	Exosc9	NM_019393	0.00050837	1.38125	E15.5 * Wnt1 up vs E15.5 * Ctrl
10605559		---	0.00128323	1.38115	E15.5 * Wnt1 up vs E15.5 * Ctrl
10560282	Grlf1	NM_172739	4.10871e-006	1.38089	E15.5 * Wnt1 up vs E15.5 * Ctrl
10379013	Flot2	NM_008028	0.000551105	1.38079	E15.5 * Wnt1 up vs E15.5 * Ctrl
10472436	B3galt1	NM_020283	0.00168656	1.3805	E15.5 * Wnt1 up vs E15.5 * Ctrl
10521566	Tmem128	NM_025480	0.00176765	1.38045	E15.5 * Wnt1 up vs E15.5 * Ctrl
10569848	Stxbp2	NM_011503	0.00138849	1.38043	E15.5 * Wnt1 up vs E15.5 * Ctrl
10494832	5730470L24Rik	NM_025679	4.75598e-006	1.3802	E15.5 * Wnt1 up vs E15.5 * Ctrl
10401068	Spnb1	NM_013675	0.000226311	1.38008	E15.5 * Wnt1 up vs E15.5 * Ctrl
10390780	Krt222	NM_172946	6.67551e-005	1.37942	E15.5 * Wnt1 up vs E15.5 * Ctrl
10534041	Stx2	NM_007941	0.00052874	1.37928	E15.5 * Wnt1 up vs E15.5 * Ctrl
10354472	Gls	NM_001081081	0.0108872	1.37918	E15.5 * Wnt1 up vs E15.5 * Ctrl
10585803	Stra6	NM_009291	0.00251875	1.37912	E15.5 * Wnt1 up vs E15.5 * Ctrl
10492341	4631416L12Rik	NM_001081295	0.00445189	1.37889	E15.5 * Wnt1 up vs E15.5 * Ctrl
10362861	Scml4	NM_172938	6.47089e-006	1.37827	E15.5 * Wnt1 up vs E15.5 * Ctrl
10452047	Ptprs	NM_011218	0.00611889	1.37813	E15.5 * Wnt1 up vs E15.5 * Ctrl

10414137	Grid1	NM_008166	0.000361435	1.37783	E15.5 * Wnt1 up vs E15.5 * Ctrl
10412060	EG668123	XR_032565	0.00936946	1.37771	E15.5 * Wnt1 up vs E15.5 * Ctrl
10424796	ENSMUSG0000C	AK005822	0.000286754	1.37755	E15.5 * Wnt1 up vs E15.5 * Ctrl
10471424	Fam102a	NM_153560	0.00396204	1.37734	E15.5 * Wnt1 up vs E15.5 * Ctrl
10410460	3110006E14Rik	NM_001145162	0.00243411	1.37718	E15.5 * Wnt1 up vs E15.5 * Ctrl
10411882	Nln	NM_029447	0.000229917	1.37716	E15.5 * Wnt1 up vs E15.5 * Ctrl
10544732	Skap2	NM_018773	3.7295e-005	1.37708	E15.5 * Wnt1 up vs E15.5 * Ctrl
10397752	Calm1	NM_009790	4.19601e-009	1.37683	E15.5 * Wnt1 up vs E15.5 * Ctrl
10462630	Pank1	NM_001114339	0.00619534	1.37681	E15.5 * Wnt1 up vs E15.5 * Ctrl
10391518	Mpp3	NM_007863	0.000111884	1.37669	E15.5 * Wnt1 up vs E15.5 * Ctrl
10463875	Sorcs3	NM_025696	0.00145044	1.37669	E15.5 * Wnt1 up vs E15.5 * Ctrl
10538109	AI854703	NR_027236	0.000220783	1.37655	E15.5 * Wnt1 up vs E15.5 * Ctrl
10441680	Pde10a	NM_011866	6.78159e-006	1.37637	E15.5 * Wnt1 up vs E15.5 * Ctrl
10595990	Armc8	NM_028768	6.69943e-005	1.37614	E15.5 * Wnt1 up vs E15.5 * Ctrl
10427015	Acvr1b	NM_007395	0.00290573	1.37614	E15.5 * Wnt1 up vs E15.5 * Ctrl
10518473	Fbxo6	NM_015797	0.000223724	1.37596	E15.5 * Wnt1 up vs E15.5 * Ctrl
10550840	Zfp112	NM_021307	0.00565711	1.37586	E15.5 * Wnt1 up vs E15.5 * Ctrl
10589494	Cspg5	NM_013884	0.00120928	1.37515	E15.5 * Wnt1 up vs E15.5 * Ctrl
10585889	Brunol6	NM_175235	0.000108996	1.3748	E15.5 * Wnt1 up vs E15.5 * Ctrl
10501282	Cyb561d1	NM_001081320	0.000323038	1.37479	E15.5 * Wnt1 up vs E15.5 * Ctrl
10536297	Ppp1r9a	NM_181595	1.12645e-005	1.37464	E15.5 * Wnt1 up vs E15.5 * Ctrl
10553115	Lmtk3	NM_001005511	1.25687e-006	1.37458	E15.5 * Wnt1 up vs E15.5 * Ctrl
10447923	Tcte2	NM_022311	1.72653e-005	1.37438	E15.5 * Wnt1 up vs E15.5 * Ctrl
10448455	Atp6v0c	NM_009729	0.00382884	1.37419	E15.5 * Wnt1 up vs E15.5 * Ctrl
10580771	Ciapin1	NM_134141	1.96522e-006	1.37418	E15.5 * Wnt1 up vs E15.5 * Ctrl
10552252	1600014C10Rik	NM_001085385	1.56599e-006	1.37398	E15.5 * Wnt1 up vs E15.5 * Ctrl
10485013	1110051M20Rik	NM_175123	1.63394e-006	1.37374	E15.5 * Wnt1 up vs E15.5 * Ctrl
10538420	Gars	NM_180678	8.91734e-007	1.37295	E15.5 * Wnt1 up vs E15.5 * Ctrl
10506335	Pde4b	NM_019840	0.00192609	1.37277	E15.5 * Wnt1 up vs E15.5 * Ctrl
10503709	D130062J21Rik	AK051661	0.00926499	1.37223	E15.5 * Wnt1 up vs E15.5 * Ctrl
10478364	Tox2	NM_001098799	0.000477039	1.3721	E15.5 * Wnt1 up vs E15.5 * Ctrl
10453573	Olfr63	NM_146937	0.00231139	1.37194	E15.5 * Wnt1 up vs E15.5 * Ctrl
10524882	Wsb2	NM_021539	0.000957356	1.37193	E15.5 * Wnt1 up vs E15.5 * Ctrl
10515519	Atp6v0b	NM_033617	0.00115296	1.37172	E15.5 * Wnt1 up vs E15.5 * Ctrl
10390574	Fbxl20	NM_028149	0.000219036	1.37148	E15.5 * Wnt1 up vs E15.5 * Ctrl
10579872	Tpd52	NM_001025263	0.00254535	1.3705	E15.5 * Wnt1 up vs E15.5 * Ctrl
10504206	N28178	BC057092	0.00127722	1.37033	E15.5 * Wnt1 up vs E15.5 * Ctrl
10574166	Cpne2	NM_153507	7.75776e-005	1.37009	E15.5 * Wnt1 up vs E15.5 * Ctrl
10402761	Tmem179	NM_178915	0.000335005	1.3699	E15.5 * Wnt1 up vs E15.5 * Ctrl
10363486	Lrrc20	NM_153542	0.000471563	1.36969	E15.5 * Wnt1 up vs E15.5 * Ctrl
10430660	Pdgfb	NM_011057	0.000617395	1.36948	E15.5 * Wnt1 up vs E15.5 * Ctrl
10442643	Nme3	NM_019730	3.70411e-005	1.3693	E15.5 * Wnt1 up vs E15.5 * Ctrl
10532576	Myo18b	BC145218	0.00622315	1.36918	E15.5 * Wnt1 up vs E15.5 * Ctrl
10591161	Zfp558	ENSMUST00000	0.00600184	1.36912	E15.5 * Wnt1 up vs E15.5 * Ctrl
10599120	Dock11	NM_001009947	0.000560602	1.36848	E15.5 * Wnt1 up vs E15.5 * Ctrl
10528268	Ptpn12	NM_011203	3.11582e-006	1.36832	E15.5 * Wnt1 up vs E15.5 * Ctrl
10442649	Mapk8ip3	NM_013931	0.00344571	1.3683	E15.5 * Wnt1 up vs E15.5 * Ctrl
10448694	Syngr3	NM_011522	0.000663935	1.36813	E15.5 * Wnt1 up vs E15.5 * Ctrl
10512612	4930412F15Rik	NM_175517	0.00102724	1.36773	E15.5 * Wnt1 up vs E15.5 * Ctrl
10533474	1500011H22Rik	BC019498	0.000599306	1.36768	E15.5 * Wnt1 up vs E15.5 * Ctrl
10401365	Zfyve1	NM_183154	0.000844434	1.36759	E15.5 * Wnt1 up vs E15.5 * Ctrl
10450996	Gpr111	NM_001033493	0.0010993	1.36757	E15.5 * Wnt1 up vs E15.5 * Ctrl
10526735	Zscan21	NM_011757	0.00324849	1.36754	E15.5 * Wnt1 up vs E15.5 * Ctrl
10542200	Gabarapl1	NM_020590	0.000168616	1.36748	E15.5 * Wnt1 up vs E15.5 * Ctrl
10385716	0610009B22Rik	NM_025319	0.000565332	1.36714	E15.5 * Wnt1 up vs E15.5 * Ctrl
10414374	Ktn1	NM_008477	3.96196e-006	1.36709	E15.5 * Wnt1 up vs E15.5 * Ctrl
10522217	Limch1	NM_001001980	0.000653499	1.36704	E15.5 * Wnt1 up vs E15.5 * Ctrl
10527538	Rasl11a	NM_026864	0.0029942	1.36625	E15.5 * Wnt1 up vs E15.5 * Ctrl
10388042	6330403K07Rik	NM_134022	1.79536e-005	1.36613	E15.5 * Wnt1 up vs E15.5 * Ctrl
10430871	Tdg	NM_011561	9.42562e-006	1.36582	E15.5 * Wnt1 up vs E15.5 * Ctrl
10502845	Fam73a	BC098205	0.000420889	1.36561	E15.5 * Wnt1 up vs E15.5 * Ctrl
10464688	Ankrd13d	NM_026720	0.00064408	1.36505	E15.5 * Wnt1 up vs E15.5 * Ctrl
10415911	Kif13b	NM_001081177	0.00295231	1.36498	E15.5 * Wnt1 up vs E15.5 * Ctrl
10582180	Fam92b	NM_001033980	0.00427325	1.36479	E15.5 * Wnt1 up vs E15.5 * Ctrl
10396094	Klhdc2	NM_027117	3.41909e-007	1.36468	E15.5 * Wnt1 up vs E15.5 * Ctrl
10540540	0610010K06Rik	NM_027861	9.85683e-005	1.36463	E15.5 * Wnt1 up vs E15.5 * Ctrl

10408677	Lyrm4	NM_201358	0.000419365	1.36453	E15.5 * Wnt1 up vs E15.5 * Ctrl
10452538	1110012J17Rik	NM_001114098	4.19109e-006	1.36428	E15.5 * Wnt1 up vs E15.5 * Ctrl
10578324	Mtus1	NM_001005863	4.2587e-005	1.36403	E15.5 * Wnt1 up vs E15.5 * Ctrl
10412267	Itga2	NM_008396	0.000639533	1.36398	E15.5 * Wnt1 up vs E15.5 * Ctrl
10367349	Smarcc2	NM_001114097	0.000269155	1.36397	E15.5 * Wnt1 up vs E15.5 * Ctrl
10373964	Thoc5	NM_172438	0.000671503	1.36379	E15.5 * Wnt1 up vs E15.5 * Ctrl
10394862	Ddef2	NM_001135192	9.58609e-006	1.36355	E15.5 * Wnt1 up vs E15.5 * Ctrl
10514158	Psip1	NM_133948	8.8739e-006	1.36351	E15.5 * Wnt1 up vs E15.5 * Ctrl
10394286	2410017P09Rik	BC067010	0.0121371	1.36337	E15.5 * Wnt1 up vs E15.5 * Ctrl
10357280	Insig2	NM_133748	0.00030056	1.36324	E15.5 * Wnt1 up vs E15.5 * Ctrl
10368011	Vta1	NM_025418	4.86652e-005	1.36321	E15.5 * Wnt1 up vs E15.5 * Ctrl
10598562	B630019K06Rik	NM_175327	0.000530587	1.36268	E15.5 * Wnt1 up vs E15.5 * Ctrl
10481621	1110008P14Rik	NM_198001	0.000256502	1.3624	E15.5 * Wnt1 up vs E15.5 * Ctrl
10448232		---	0.000340592	1.36189	E15.5 * Wnt1 up vs E15.5 * Ctrl
10593937	Mpi	NM_025837	0.000216308	1.36179	E15.5 * Wnt1 up vs E15.5 * Ctrl
10397000		---	0.00280299	1.36162	E15.5 * Wnt1 up vs E15.5 * Ctrl
10489053	4930518I15Rik	ENSMUST00000(	0.00465687	1.36131	E15.5 * Wnt1 up vs E15.5 * Ctrl
10499470	Rusc1	NM_028188	0.000221856	1.36099	E15.5 * Wnt1 up vs E15.5 * Ctrl
10487441	Mal	NM_010762	0.00676504	1.36086	E15.5 * Wnt1 up vs E15.5 * Ctrl
10468231	Arl3	NM_019718	2.52195e-005	1.3607	E15.5 * Wnt1 up vs E15.5 * Ctrl
10515168	Cyp4x1	NM_001003947	0.000114312	1.36056	E15.5 * Wnt1 up vs E15.5 * Ctrl
10551483	Eid2	NM_198425	0.000346691	1.36019	E15.5 * Wnt1 up vs E15.5 * Ctrl
10596925	Ndufaf3	NM_023247	0.00321133	1.35991	E15.5 * Wnt1 up vs E15.5 * Ctrl
10547469	Hsn2	NM_001037155	0.0128564	1.35987	E15.5 * Wnt1 up vs E15.5 * Ctrl
10553646	Herc2	NM_010418	1.61633e-007	1.35983	E15.5 * Wnt1 up vs E15.5 * Ctrl
10447477	ENSMUSG00000(	ENSMUST00000(	0.0122736	1.35976	E15.5 * Wnt1 up vs E15.5 * Ctrl
10503828	Lyrm2	NM_175364	0.00263614	1.35973	E15.5 * Wnt1 up vs E15.5 * Ctrl
10569542	Mrgpre	NM_175534	0.00451926	1.359	E15.5 * Wnt1 up vs E15.5 * Ctrl
10493770	Ilf2	NM_026374	2.3912e-005	1.35884	E15.5 * Wnt1 up vs E15.5 * Ctrl
10544452	Fam115c	BC011487	0.00291983	1.35876	E15.5 * Wnt1 up vs E15.5 * Ctrl
10422980	Lmbrd2	NM_177178	6.99815e-005	1.35828	E15.5 * Wnt1 up vs E15.5 * Ctrl
10554094	Igf1r	NM_010513	0.00248226	1.35828	E15.5 * Wnt1 up vs E15.5 * Ctrl
10516371	Eif2c1	NM_153403	0.00142008	1.35827	E15.5 * Wnt1 up vs E15.5 * Ctrl
10406280	Spata9	NM_029343	0.000875271	1.35775	E15.5 * Wnt1 up vs E15.5 * Ctrl
10409767	Golm1	NM_027307	0.001816	1.35739	E15.5 * Wnt1 up vs E15.5 * Ctrl
10434845	Il1rap	NM_008364	0.000230343	1.35713	E15.5 * Wnt1 up vs E15.5 * Ctrl
10512739	Xpa	NM_011728	0.00562194	1.35709	E15.5 * Wnt1 up vs E15.5 * Ctrl
10544763	Hoxa4	NM_008265	0.0002511	1.35704	E15.5 * Wnt1 up vs E15.5 * Ctrl
10405280	Arl10	NM_019968	0.0036867	1.35697	E15.5 * Wnt1 up vs E15.5 * Ctrl
10557498	Fam57b	NM_026884	0.00174251	1.35667	E15.5 * Wnt1 up vs E15.5 * Ctrl
10456764	Smad2	NM_010754	6.22562e-006	1.35667	E15.5 * Wnt1 up vs E15.5 * Ctrl
10565193	Hdgfrp3	NM_013886	6.8412e-005	1.35632	E15.5 * Wnt1 up vs E15.5 * Ctrl
10538617	Lancl2	NM_133737	3.93579e-006	1.35605	E15.5 * Wnt1 up vs E15.5 * Ctrl
10442468	Caskin1	NM_027937	5.41165e-005	1.35598	E15.5 * Wnt1 up vs E15.5 * Ctrl
10428439		---	0.0040914	1.35563	E15.5 * Wnt1 up vs E15.5 * Ctrl
10531631		---	0.0147091	1.35559	E15.5 * Wnt1 up vs E15.5 * Ctrl
10376420	OTTMUSG00000(	ENSMUST00000(	0.0133913	1.35515	E15.5 * Wnt1 up vs E15.5 * Ctrl
10486262	Ltk	NM_203345	6.79515e-006	1.3551	E15.5 * Wnt1 up vs E15.5 * Ctrl
10583773	BC018242	NM_144935	2.67962e-006	1.35499	E15.5 * Wnt1 up vs E15.5 * Ctrl
10546557	Fam19a4	NM_177233	0.000763003	1.35484	E15.5 * Wnt1 up vs E15.5 * Ctrl
10402648	Brp44l	NM_018819	0.00181792	1.35479	E15.5 * Wnt1 up vs E15.5 * Ctrl
10524234	Galnt9	NM_198306	0.000777636	1.3547	E15.5 * Wnt1 up vs E15.5 * Ctrl
10446071	Fem1a	NM_010192	0.00176379	1.35459	E15.5 * Wnt1 up vs E15.5 * Ctrl
10357946	Ppp1r12b	NM_001081307	0.00134914	1.35443	E15.5 * Wnt1 up vs E15.5 * Ctrl
10410995	Rasgrf2	NM_009027	0.000181737	1.35416	E15.5 * Wnt1 up vs E15.5 * Ctrl
10500534	Hsd3b4	NM_001111336	0.00032999	1.35407	E15.5 * Wnt1 up vs E15.5 * Ctrl
10500539	Hsd3b4	NM_001111336	0.00032999	1.35407	E15.5 * Wnt1 up vs E15.5 * Ctrl
10425559	L3mbtl2	NM_145993	3.95995e-005	1.35403	E15.5 * Wnt1 up vs E15.5 * Ctrl
10542040	Parp11	NM_181402	0.00664761	1.35403	E15.5 * Wnt1 up vs E15.5 * Ctrl
10575249	Txnl4b	NM_175646	0.000581922	1.35376	E15.5 * Wnt1 up vs E15.5 * Ctrl
10601235	Ogt	NM_139144	8.63837e-007	1.35371	E15.5 * Wnt1 up vs E15.5 * Ctrl
10388749	Traf4	NM_009423	0.00102353	1.35371	E15.5 * Wnt1 up vs E15.5 * Ctrl
10475245	AV039307	AK006022	0.00112767	1.3534	E15.5 * Wnt1 up vs E15.5 * Ctrl
10591517	Cdkn2d	NM_009878	0.000102161	1.35332	E15.5 * Wnt1 up vs E15.5 * Ctrl
10401891	Ston2	NM_175367	0.000794239	1.35289	E15.5 * Wnt1 up vs E15.5 * Ctrl
10592023	Aplp2	NM_001102455	0.000190556	1.35287	E15.5 * Wnt1 up vs E15.5 * Ctrl

10358593	Hmcn1	NM_001024720	0.00597852	1.35286	E15.5 * Wnt1 up vs E15.5 * Ctrl
10586920	Rfx7	NM_001033536	7.80366e-006	1.35279	E15.5 * Wnt1 up vs E15.5 * Ctrl
10379901	Bcas3	NM_138681	0.000152262	1.35245	E15.5 * Wnt1 up vs E15.5 * Ctrl
10586616	Vps13c	NM_177184	0.000211265	1.35237	E15.5 * Wnt1 up vs E15.5 * Ctrl
10510552	Rere	NM_001085492	0.000117372	1.35234	E15.5 * Wnt1 up vs E15.5 * Ctrl
10522250	Tmem33	NM_028975	0.000154991	1.35211	E15.5 * Wnt1 up vs E15.5 * Ctrl
10538519	Gsbs	NM_011153	0.00939622	1.35193	E15.5 * Wnt1 up vs E15.5 * Ctrl
10413171		---	0.000428209	1.3519	E15.5 * Wnt1 up vs E15.5 * Ctrl
10399421	Mycn	NM_008709	0.00121226	1.35158	E15.5 * Wnt1 up vs E15.5 * Ctrl
10568792	Stk32c	NM_021302	0.00358649	1.35148	E15.5 * Wnt1 up vs E15.5 * Ctrl
10432593	Csrnp2	NM_153407	3.91263e-008	1.35077	E15.5 * Wnt1 up vs E15.5 * Ctrl
10441361	Tiam2	NM_001122998	0.00116727	1.3507	E15.5 * Wnt1 up vs E15.5 * Ctrl
10579910	Zc3h15	NM_026934	0.00025335	1.35065	E15.5 * Wnt1 up vs E15.5 * Ctrl
10448765	Mapk8ip3	NM_013931	2.19843e-005	1.3505	E15.5 * Wnt1 up vs E15.5 * Ctrl
10538299	Hoxa3	NM_010452	0.000327313	1.3505	E15.5 * Wnt1 up vs E15.5 * Ctrl
10357191	Ptpn4	NM_019933	0.00073387	1.35034	E15.5 * Wnt1 up vs E15.5 * Ctrl
10517053	Trnp1	NM_001081156	0.00280367	1.35026	E15.5 * Wnt1 up vs E15.5 * Ctrl
10375980	Aff4	NM_033565	5.09353e-006	1.35019	E15.5 * Wnt1 up vs E15.5 * Ctrl
10592493	4931429I11Rik	NM_001081121	7.07611e-005	1.35015	E15.5 * Wnt1 up vs E15.5 * Ctrl
10602716	Ubqln2	NM_018798	9.7316e-005	1.3501	E15.5 * Wnt1 up vs E15.5 * Ctrl
10461051	5730409K12Rik	AK134852	0.0106081	1.34996	E15.5 * Wnt1 up vs E15.5 * Ctrl
10406590	Ankrd34b	NM_175455	0.00113433	1.34987	E15.5 * Wnt1 up vs E15.5 * Ctrl
10387111	2310004I24Rik	NM_025510	0.00127224	1.34985	E15.5 * Wnt1 up vs E15.5 * Ctrl
10356932	D1Ertd622e	BC023951	0.000996507	1.34975	E15.5 * Wnt1 up vs E15.5 * Ctrl
10519138	ENSMUSG0000C	ENSMUST00000(	0.00227699	1.34966	E15.5 * Wnt1 up vs E15.5 * Ctrl
10606445	Rps6ka6	NM_025949	0.000164524	1.34942	E15.5 * Wnt1 up vs E15.5 * Ctrl
10455967	2610318N02Rik	BC039993	0.0088996	1.3493	E15.5 * Wnt1 up vs E15.5 * Ctrl
10448369	Flywch1	NM_153791	4.19317e-005	1.34921	E15.5 * Wnt1 up vs E15.5 * Ctrl
10572050	March1	NM_175188	0.000263756	1.3492	E15.5 * Wnt1 up vs E15.5 * Ctrl
10389680	Msi2	NM_054043	5.05768e-005	1.34919	E15.5 * Wnt1 up vs E15.5 * Ctrl
10415163	Dhrs2	NM_027790	0.000851116	1.34918	E15.5 * Wnt1 up vs E15.5 * Ctrl
10464917	Cnih2	NM_009920	7.34449e-005	1.34903	E15.5 * Wnt1 up vs E15.5 * Ctrl
10456501	Rnmt	NM_026440	0.00032497	1.34898	E15.5 * Wnt1 up vs E15.5 * Ctrl
10383920	Nefh	NM_010904	0.00106948	1.34893	E15.5 * Wnt1 up vs E15.5 * Ctrl
10436967	Cbr1	NM_007620	0.00258688	1.34837	E15.5 * Wnt1 up vs E15.5 * Ctrl
10372524	4933416C03Rik	NR_003651	0.00116664	1.34822	E15.5 * Wnt1 up vs E15.5 * Ctrl
10589438	Mtap4	NM_008633	4.04853e-005	1.34821	E15.5 * Wnt1 up vs E15.5 * Ctrl
10358155	2310006M14Rik	AK009188	0.00132237	1.34815	E15.5 * Wnt1 up vs E15.5 * Ctrl
10388861	Tmem199	NM_199199	0.0113286	1.34801	E15.5 * Wnt1 up vs E15.5 * Ctrl
10577999	Unc5d	NM_153135	0.00466824	1.34788	E15.5 * Wnt1 up vs E15.5 * Ctrl
10454298	Zfp397	NM_027007	2.01879e-006	1.34775	E15.5 * Wnt1 up vs E15.5 * Ctrl
10371188	Brunol5	NM_176954	7.04399e-005	1.34766	E15.5 * Wnt1 up vs E15.5 * Ctrl
10581737	Ddx19b	NM_172284	0.00102374	1.34762	E15.5 * Wnt1 up vs E15.5 * Ctrl
10497996	Ikzf5	NM_175115	0.000631133	1.34754	E15.5 * Wnt1 up vs E15.5 * Ctrl
10389865	Nme1	NM_008704	3.15761e-007	1.34706	E15.5 * Wnt1 up vs E15.5 * Ctrl
10493245	Mex3a	NM_001029890	8.69675e-005	1.34692	E15.5 * Wnt1 up vs E15.5 * Ctrl
10380637	Hoxb6	NM_008269	0.000800556	1.34652	E15.5 * Wnt1 up vs E15.5 * Ctrl
10578962	ENSMUSG0000C	ENSMUST00000(	0.010595	1.34644	E15.5 * Wnt1 up vs E15.5 * Ctrl
10499483	Fdps	NM_134469	0.000139467	1.34641	E15.5 * Wnt1 up vs E15.5 * Ctrl
10347232	Xrcc5	NM_009533	4.07035e-005	1.34637	E15.5 * Wnt1 up vs E15.5 * Ctrl
10405693	Dapk1	NM_029653	9.63854e-009	1.34632	E15.5 * Wnt1 up vs E15.5 * Ctrl
10407513	Wdr37	NM_172445	0.000243232	1.34596	E15.5 * Wnt1 up vs E15.5 * Ctrl
10576118	9330133O14Rik	BC094355	0.000106466	1.34583	E15.5 * Wnt1 up vs E15.5 * Ctrl
10604844	Sms	NM_009214	1.93424e-005	1.34581	E15.5 * Wnt1 up vs E15.5 * Ctrl
10389010	5730455P16Rik	NM_027472	0.0042011	1.34571	E15.5 * Wnt1 up vs E15.5 * Ctrl
10459804	Katnal2	NM_027721	0.00473004	1.34542	E15.5 * Wnt1 up vs E15.5 * Ctrl
10550931	Irgq	NM_153134	0.000809068	1.34528	E15.5 * Wnt1 up vs E15.5 * Ctrl
10532753	Coro1c	NM_011779	0.00225225	1.34519	E15.5 * Wnt1 up vs E15.5 * Ctrl
10411958	Rnf180	NM_027934	0.000160159	1.34511	E15.5 * Wnt1 up vs E15.5 * Ctrl
10473272	Zc3h15	NM_026934	5.95728e-006	1.34497	E15.5 * Wnt1 up vs E15.5 * Ctrl
10488266	6330439K17Rik	NM_172859	0.000677217	1.34489	E15.5 * Wnt1 up vs E15.5 * Ctrl
10477994	Ctnnbl1	NM_025680	0.000134331	1.34483	E15.5 * Wnt1 up vs E15.5 * Ctrl
10536401	Nxph1	NM_008751	0.000547885	1.34478	E15.5 * Wnt1 up vs E15.5 * Ctrl
10569767	Zfp358	NM_080461	0.00394471	1.34464	E15.5 * Wnt1 up vs E15.5 * Ctrl
10391540	Mpp2	NM_016695	0.0014117	1.34457	E15.5 * Wnt1 up vs E15.5 * Ctrl
10351825	Tagln2	NM_178598	0.00255322	1.34443	E15.5 * Wnt1 up vs E15.5 * Ctrl

10450796	Armcx5	NM_001009575	0.0116008	1.3443	E15.5 * Wnt1 up vs E15.5 * Ctrl
10585444	Ireb2	NM_022655	0.000241916	1.34429	E15.5 * Wnt1 up vs E15.5 * Ctrl
10405355	Unc5a	NM_153131	0.000907233	1.34422	E15.5 * Wnt1 up vs E15.5 * Ctrl
10357590	Dyrk3	NM_145508	0.0117094	1.34421	E15.5 * Wnt1 up vs E15.5 * Ctrl
10436416	Gabrr3	NM_001081190	0.00282751	1.34417	E15.5 * Wnt1 up vs E15.5 * Ctrl
10573823	Chd9	NM_177224	3.15312e-005	1.34407	E15.5 * Wnt1 up vs E15.5 * Ctrl
10378914	Myo18a	NM_011586	8.55061e-006	1.34406	E15.5 * Wnt1 up vs E15.5 * Ctrl
10468200	Cuedc2	NM_024192	9.08463e-005	1.34393	E15.5 * Wnt1 up vs E15.5 * Ctrl
10512327	4933409K07Rik	BC072647	0.00074197	1.34388	E15.5 * Wnt1 up vs E15.5 * Ctrl
10495243	Gstm5	NM_010360	2.53185e-005	1.3437	E15.5 * Wnt1 up vs E15.5 * Ctrl
10564663	Tdg	NM_172552	0.000151114	1.34318	E15.5 * Wnt1 up vs E15.5 * Ctrl
10533729	Vps37b	NM_177876	0.0021102	1.34318	E15.5 * Wnt1 up vs E15.5 * Ctrl
10381140	Ttc25	NM_028918	0.00125767	1.34316	E15.5 * Wnt1 up vs E15.5 * Ctrl
10550519	Nanos2	NM_194064	0.000497271	1.34304	E15.5 * Wnt1 up vs E15.5 * Ctrl
10532596	Myo18b	BC145218	0.00476214	1.34302	E15.5 * Wnt1 up vs E15.5 * Ctrl
10436941	Mrps6	NM_080456	0.00430031	1.34281	E15.5 * Wnt1 up vs E15.5 * Ctrl
10591643	Rab3d	NM_031874	8.3277e-005	1.34269	E15.5 * Wnt1 up vs E15.5 * Ctrl
10401753	AI413782	NM_001142580	0.00108914	1.34263	E15.5 * Wnt1 up vs E15.5 * Ctrl
10545638	Tlx2	NM_009392	0.000816847	1.34232	E15.5 * Wnt1 up vs E15.5 * Ctrl
10607585	OTTMUSG00000	NM_001112661	0.00380892	1.34198	E15.5 * Wnt1 up vs E15.5 * Ctrl
10455253	EG545253	NM_001033789	0.00621091	1.34182	E15.5 * Wnt1 up vs E15.5 * Ctrl
10477004	LOC100044416	XR_030619	1.31294e-006	1.34175	E15.5 * Wnt1 up vs E15.5 * Ctrl
10593103	Rnf214	NM_178709	6.01555e-005	1.34167	E15.5 * Wnt1 up vs E15.5 * Ctrl
10454235	Asxl3	ENSMUST00000	0.000259646	1.34122	E15.5 * Wnt1 up vs E15.5 * Ctrl
10529567	D5Ertd579e	NM_001081232	9.10388e-006	1.34118	E15.5 * Wnt1 up vs E15.5 * Ctrl
10600144	F8a	NM_007978	0.00390271	1.34096	E15.5 * Wnt1 up vs E15.5 * Ctrl
10421924	Pcdh9	NM_001081377	0.00348892	1.34093	E15.5 * Wnt1 up vs E15.5 * Ctrl
10423745	Spag1	NM_012031	0.00764174	1.34084	E15.5 * Wnt1 up vs E15.5 * Ctrl
10429222	Fam135b	NM_177819	0.00110073	1.34077	E15.5 * Wnt1 up vs E15.5 * Ctrl
10395039	Cmpk2	NM_020557	0.000707087	1.3405	E15.5 * Wnt1 up vs E15.5 * Ctrl
10534120	Asl	NM_133768	0.000769211	1.34035	E15.5 * Wnt1 up vs E15.5 * Ctrl
10380303	Car10	NM_028296	0.000648114	1.34006	E15.5 * Wnt1 up vs E15.5 * Ctrl
10396694	Churc1	NM_206534	0.00124766	1.34002	E15.5 * Wnt1 up vs E15.5 * Ctrl
10557399	Sbk1	NM_145587	0.000199924	1.34	E15.5 * Wnt1 up vs E15.5 * Ctrl
10400572	Klhl28	NM_025707	0.00835913	1.3399	E15.5 * Wnt1 up vs E15.5 * Ctrl
10592701	Tmem136	NM_001034863	0.000109213	1.33983	E15.5 * Wnt1 up vs E15.5 * Ctrl
10583384		---	0.0119976	1.33977	E15.5 * Wnt1 up vs E15.5 * Ctrl
10349016	2310035C23Rik	NM_173187	0.00528165	1.33962	E15.5 * Wnt1 up vs E15.5 * Ctrl
10507983	Epha10	NM_177671	0.00254863	1.33961	E15.5 * Wnt1 up vs E15.5 * Ctrl
10390768	Smarce1	NM_020618	5.98651e-006	1.33956	E15.5 * Wnt1 up vs E15.5 * Ctrl
10400718	Sos2	NM_001135559	0.00306555	1.33954	E15.5 * Wnt1 up vs E15.5 * Ctrl
10497689	Gnb4	NM_013531	0.000212671	1.33934	E15.5 * Wnt1 up vs E15.5 * Ctrl
10366514	Kcnmb4	NM_021452	0.00287553	1.33902	E15.5 * Wnt1 up vs E15.5 * Ctrl
10477485	2310021H06Rik	NM_025990	0.00074523	1.33898	E15.5 * Wnt1 up vs E15.5 * Ctrl
10532368	2410025L10Rik	NM_001142642	1.30773e-005	1.33818	E15.5 * Wnt1 up vs E15.5 * Ctrl
10430132	Zfp647	NM_172817	0.000718913	1.33796	E15.5 * Wnt1 up vs E15.5 * Ctrl
10523766	Lrrc8c	NM_133897	0.0100092	1.33757	E15.5 * Wnt1 up vs E15.5 * Ctrl
10516305	Mtap7d1	NM_144941	8.01806e-006	1.33749	E15.5 * Wnt1 up vs E15.5 * Ctrl
10444079	Rgl2	NM_009059	0.00289984	1.33729	E15.5 * Wnt1 up vs E15.5 * Ctrl
10474977	1500003O03Rik	NM_019769	8.54383e-005	1.33726	E15.5 * Wnt1 up vs E15.5 * Ctrl
10405662	Mak10	NM_030153	1.19834e-006	1.33703	E15.5 * Wnt1 up vs E15.5 * Ctrl
10554076	Lysmd4	NM_175215	0.00354963	1.33694	E15.5 * Wnt1 up vs E15.5 * Ctrl
10402708	Ckb	NM_021273	0.00760587	1.33688	E15.5 * Wnt1 up vs E15.5 * Ctrl
10579987	Scoc	NM_001039137	0.00488664	1.33686	E15.5 * Wnt1 up vs E15.5 * Ctrl
10504703	AU014645	NM_001033201	4.96228e-005	1.3368	E15.5 * Wnt1 up vs E15.5 * Ctrl
10549679	Brsk1	NM_001003920	0.00660518	1.33671	E15.5 * Wnt1 up vs E15.5 * Ctrl
10365518	Nt5dc3	NM_175331	0.0079029	1.33665	E15.5 * Wnt1 up vs E15.5 * Ctrl
10424700	Zfp41	NM_011759	0.000191693	1.33653	E15.5 * Wnt1 up vs E15.5 * Ctrl
10526842	Zfp157	NM_028130	7.13334e-005	1.33643	E15.5 * Wnt1 up vs E15.5 * Ctrl
10420659	6330409N04Rik	NM_025697	3.39781e-005	1.33592	E15.5 * Wnt1 up vs E15.5 * Ctrl
10365230	Tdg	NM_172552	3.04171e-005	1.33578	E15.5 * Wnt1 up vs E15.5 * Ctrl
10573115	Rnf150	NM_177378	0.000824648	1.33574	E15.5 * Wnt1 up vs E15.5 * Ctrl
10356593	Hes6	NM_019479	0.00252885	1.33573	E15.5 * Wnt1 up vs E15.5 * Ctrl
10388954	Omg	NM_019409	0.005084	1.33564	E15.5 * Wnt1 up vs E15.5 * Ctrl
10354157	Chst10	NM_145142	0.000111059	1.33539	E15.5 * Wnt1 up vs E15.5 * Ctrl
10426835	Dip2b	NM_001159361	0.000109204	1.33539	E15.5 * Wnt1 up vs E15.5 * Ctrl

10585601	Snupn	NM_178374	0.00143672	1.33531	E15.5 * Wnt1 up vs E15.5 * Ctrl
10448376	Flywch2	NM_029798	0.000231612	1.33494	E15.5 * Wnt1 up vs E15.5 * Ctrl
10385234		---	0.00113026	1.33492	E15.5 * Wnt1 up vs E15.5 * Ctrl
10396161	Tmx1	NM_028339	0.00117203	1.3349	E15.5 * Wnt1 up vs E15.5 * Ctrl
10595831	Zbtb38	NM_175537	0.00229362	1.33487	E15.5 * Wnt1 up vs E15.5 * Ctrl
10523190	9130213B05Rik	BC006604	0.00258496	1.33461	E15.5 * Wnt1 up vs E15.5 * Ctrl
10366310	Osbpl8	NM_175489	0.00249274	1.33453	E15.5 * Wnt1 up vs E15.5 * Ctrl
10536541	St7	NM_022332	0.000844321	1.3344	E15.5 * Wnt1 up vs E15.5 * Ctrl
10419240	Ddhd1	NM_001042719	0.00184612	1.33396	E15.5 * Wnt1 up vs E15.5 * Ctrl
10599461	Calm2	NM_007589	3.63956e-006	1.3339	E15.5 * Wnt1 up vs E15.5 * Ctrl
10368534	Ncoa7	NM_172495	0.00513207	1.33367	E15.5 * Wnt1 up vs E15.5 * Ctrl
10448034	Dll1	NM_007865	0.00209367	1.33365	E15.5 * Wnt1 up vs E15.5 * Ctrl
10547140	Tmcc1	ENSMUST00000(	1.77688e-005	1.33334	E15.5 * Wnt1 up vs E15.5 * Ctrl
10504169	4933409K07Rik	BC072647	0.00072401	1.33223	E15.5 * Wnt1 up vs E15.5 * Ctrl
10377987	Rabep1	NM_019400	6.32102e-007	1.33197	E15.5 * Wnt1 up vs E15.5 * Ctrl
10512098	Aptx	NM_025545	0.000505011	1.33196	E15.5 * Wnt1 up vs E15.5 * Ctrl
10507394	Hectd3	NM_175244	0.0035807	1.33192	E15.5 * Wnt1 up vs E15.5 * Ctrl
10385926	Cdc42se2	NM_178626	0.00129508	1.33188	E15.5 * Wnt1 up vs E15.5 * Ctrl
10425822	Pnpla3	NM_054088	0.00106527	1.33185	E15.5 * Wnt1 up vs E15.5 * Ctrl
10430968	A4galt	NM_001004150	0.0065796	1.33184	E15.5 * Wnt1 up vs E15.5 * Ctrl
10593887	Neil1	NM_028347	0.0138542	1.3315	E15.5 * Wnt1 up vs E15.5 * Ctrl
10468916	Fam171a1	NM_001081161	0.000364077	1.33127	E15.5 * Wnt1 up vs E15.5 * Ctrl
10358672	Hmcn1	NM_001024720	0.00362136	1.33092	E15.5 * Wnt1 up vs E15.5 * Ctrl
10600377	Atp6ap1	NM_018794	0.0077488	1.33091	E15.5 * Wnt1 up vs E15.5 * Ctrl
10495340	Taf13	NM_025444	0.000283429	1.33069	E15.5 * Wnt1 up vs E15.5 * Ctrl
10500685	Atp1a1	NM_144900	0.000154792	1.33067	E15.5 * Wnt1 up vs E15.5 * Ctrl
10487000	Shf	NM_001013829	0.00145359	1.33065	E15.5 * Wnt1 up vs E15.5 * Ctrl
10402808	Jag2	NM_010588	0.00145207	1.33056	E15.5 * Wnt1 up vs E15.5 * Ctrl
10532267	Vmn2r9	NM_001104621	0.00589055	1.33	E15.5 * Wnt1 up vs E15.5 * Ctrl
10508272	Csmd2	ENSMUST00000(	0.000161274	1.32986	E15.5 * Wnt1 up vs E15.5 * Ctrl
10501098	Kcnc4	NM_145922	0.000169487	1.32979	E15.5 * Wnt1 up vs E15.5 * Ctrl
10474073	C230071H18Rik	BC147668	0.0017422	1.32964	E15.5 * Wnt1 up vs E15.5 * Ctrl
10411332	Hmgcr	NM_008255	0.00469573	1.32947	E15.5 * Wnt1 up vs E15.5 * Ctrl
10543785	AB041803	AB041803	0.00730748	1.32944	E15.5 * Wnt1 up vs E15.5 * Ctrl
10364194	Lss	NM_146006	0.00124241	1.32899	E15.5 * Wnt1 up vs E15.5 * Ctrl
10483770	Lnp	NM_001110209	0.00156536	1.32896	E15.5 * Wnt1 up vs E15.5 * Ctrl
10455769	Csnk1g3	NM_152809	0.000155321	1.32881	E15.5 * Wnt1 up vs E15.5 * Ctrl
10439732	Plcxd2	NM_001134480	0.00411321	1.32877	E15.5 * Wnt1 up vs E15.5 * Ctrl
10601547		---	0.00186294	1.32855	E15.5 * Wnt1 up vs E15.5 * Ctrl
10571761		---	0.0011635	1.32853	E15.5 * Wnt1 up vs E15.5 * Ctrl
10507677	Hivep3	NM_010657	0.000739579	1.32815	E15.5 * Wnt1 up vs E15.5 * Ctrl
10603362	Ccdc120	NM_207202	0.00761641	1.328	E15.5 * Wnt1 up vs E15.5 * Ctrl
10587871	Paqr9	NM_198414	0.00884424	1.32788	E15.5 * Wnt1 up vs E15.5 * Ctrl
10552118	OTTMUSG00000	XM_001481141	0.0138303	1.32753	E15.5 * Wnt1 up vs E15.5 * Ctrl
10426292	Alg10b	NM_001033441	0.000207109	1.32733	E15.5 * Wnt1 up vs E15.5 * Ctrl
10555009		---	0.000325149	1.32725	E15.5 * Wnt1 up vs E15.5 * Ctrl
10511069	Gnb1	NM_008142	2.07651e-005	1.32717	E15.5 * Wnt1 up vs E15.5 * Ctrl
10444991	EG547347	NM_001034909	0.0012667	1.32691	E15.5 * Wnt1 up vs E15.5 * Ctrl
10362904	Rtn4ip1	NM_130892	0.00207967	1.32689	E15.5 * Wnt1 up vs E15.5 * Ctrl
10502451	Bmpr1b	NM_007560	0.0145676	1.32677	E15.5 * Wnt1 up vs E15.5 * Ctrl
10594638	Aph1c	NM_026674	0.0037119	1.32665	E15.5 * Wnt1 up vs E15.5 * Ctrl
10590888	EG665154	XR_031035	0.000459375	1.32664	E15.5 * Wnt1 up vs E15.5 * Ctrl
10513397	1110054O05Rik	NM_001013577	1.94107e-005	1.32656	E15.5 * Wnt1 up vs E15.5 * Ctrl
10561958	Snx26	NM_178252	0.000552002	1.32648	E15.5 * Wnt1 up vs E15.5 * Ctrl
10540991		---	0.0011109	1.32628	E15.5 * Wnt1 up vs E15.5 * Ctrl
10488655	Bcl2l1	NM_009743	0.00223909	1.32626	E15.5 * Wnt1 up vs E15.5 * Ctrl
10431564	Cpt1b	NM_009948	0.000214092	1.32614	E15.5 * Wnt1 up vs E15.5 * Ctrl
10547410	Erc1	NM_053204	6.11575e-005	1.32582	E15.5 * Wnt1 up vs E15.5 * Ctrl
10348194	Efhd1	NM_028889	0.0014008	1.32557	E15.5 * Wnt1 up vs E15.5 * Ctrl
10454015	Ttc39c	NM_028341	0.000882168	1.3252	E15.5 * Wnt1 up vs E15.5 * Ctrl
10360914	Lyplal1	NM_146106	0.00249996	1.32508	E15.5 * Wnt1 up vs E15.5 * Ctrl
10451322	Cul9	NM_001081335	0.000197274	1.32494	E15.5 * Wnt1 up vs E15.5 * Ctrl
10577534	Vdac3	NM_011696	2.1007e-005	1.32468	E15.5 * Wnt1 up vs E15.5 * Ctrl
10524941	Fbxo21	NM_145564	0.000801407	1.32467	E15.5 * Wnt1 up vs E15.5 * Ctrl
10548116	Ccnd2	BC049086	0.000287521	1.32425	E15.5 * Wnt1 up vs E15.5 * Ctrl
10386789	Ulk2	NM_013881	2.80164e-006	1.32408	E15.5 * Wnt1 up vs E15.5 * Ctrl

10439955	Fam55c	NM_001134457	0.00879789	1.32402	E15.5 * Wnt1 up vs E15.5 * Ctrl
10353775	Bend6	NM_177235	0.00481644	1.32396	E15.5 * Wnt1 up vs E15.5 * Ctrl
10591668	Rgl3	NM_023622	0.000285555	1.32389	E15.5 * Wnt1 up vs E15.5 * Ctrl
10500133	Prune	NM_173347	0.0031959	1.32388	E15.5 * Wnt1 up vs E15.5 * Ctrl
10440738	Tiam1	NM_009384	0.00106656	1.32366	E15.5 * Wnt1 up vs E15.5 * Ctrl
10518464	2610109H07Rik	NM_027426	0.00293091	1.32343	E15.5 * Wnt1 up vs E15.5 * Ctrl
10415574	Ccni	NM_017367	2.01079e-005	1.32338	E15.5 * Wnt1 up vs E15.5 * Ctrl
10514072	Zdhhc21	NM_026647	0.002665	1.32314	E15.5 * Wnt1 up vs E15.5 * Ctrl
10502510	Lmo4	NM_010723	0.00305736	1.32279	E15.5 * Wnt1 up vs E15.5 * Ctrl
10479514	Col20a1	BC016112	0.00432028	1.32261	E15.5 * Wnt1 up vs E15.5 * Ctrl
10358091	Nav1	NM_173437	0.0019299	1.32257	E15.5 * Wnt1 up vs E15.5 * Ctrl
10491300	Skil	NM_011386	0.000476546	1.32207	E15.5 * Wnt1 up vs E15.5 * Ctrl
10597103	Dhx30	NM_133347	1.42676e-005	1.32199	E15.5 * Wnt1 up vs E15.5 * Ctrl
10522606	Exoc1	NM_027270	0.000157908	1.32191	E15.5 * Wnt1 up vs E15.5 * Ctrl
10487033	Myef2	NM_010852	0.000324754	1.32191	E15.5 * Wnt1 up vs E15.5 * Ctrl
10490946	Hsp90aa1	NM_010480	1.20104e-005	1.32132	E15.5 * Wnt1 up vs E15.5 * Ctrl
10580391	Itfg1	NM_028007	3.61471e-006	1.32105	E15.5 * Wnt1 up vs E15.5 * Ctrl
10564109		---	0.000969732	1.3207	E15.5 * Wnt1 up vs E15.5 * Ctrl
10564117		---	0.000969732	1.3207	E15.5 * Wnt1 up vs E15.5 * Ctrl
10366597	Grip1	NM_028736	0.000409332	1.32064	E15.5 * Wnt1 up vs E15.5 * Ctrl
10558707	Ric8	NM_053194	0.00193136	1.32056	E15.5 * Wnt1 up vs E15.5 * Ctrl
10401702	Zdhhc22	NM_001080943	0.0018364	1.32048	E15.5 * Wnt1 up vs E15.5 * Ctrl
10477897	Dlgap4	NM_146128	3.41318e-008	1.32041	E15.5 * Wnt1 up vs E15.5 * Ctrl
10536369	C1galt1	NM_052993	0.00664631	1.32022	E15.5 * Wnt1 up vs E15.5 * Ctrl
10467921	Chuk	NM_007700	0.00225793	1.32014	E15.5 * Wnt1 up vs E15.5 * Ctrl
10507990	Epha10	ENSMUST00000	0.000861585	1.32008	E15.5 * Wnt1 up vs E15.5 * Ctrl
10520048	Mll5	ENSMUST00000(	2.57765e-005	1.31998	E15.5 * Wnt1 up vs E15.5 * Ctrl
10523865	1700028K03Rik	NM_182745	0.00278978	1.31997	E15.5 * Wnt1 up vs E15.5 * Ctrl
10582719	Sipa1l2	NM_001081337	0.00299446	1.31988	E15.5 * Wnt1 up vs E15.5 * Ctrl
10488926	Mmp24	NM_010808	0.00033365	1.31982	E15.5 * Wnt1 up vs E15.5 * Ctrl
10394929	EG668662	AK132630	0.0121543	1.31883	E15.5 * Wnt1 up vs E15.5 * Ctrl
10407921	Vdac3	NM_011696	1.20203e-005	1.31881	E15.5 * Wnt1 up vs E15.5 * Ctrl
10407926	Vdac3	NM_011696	1.20203e-005	1.31881	E15.5 * Wnt1 up vs E15.5 * Ctrl
10423363	Ank	NM_020332	0.00352126	1.31853	E15.5 * Wnt1 up vs E15.5 * Ctrl
10403420	Idi2	NM_177197	0.00414919	1.3185	E15.5 * Wnt1 up vs E15.5 * Ctrl
10347650	Accn4	NM_183022	0.00119777	1.31832	E15.5 * Wnt1 up vs E15.5 * Ctrl
10351117	ENSMUSG00000	ENSMUST00000(	0.000396138	1.31801	E15.5 * Wnt1 up vs E15.5 * Ctrl
10369541	Hk1	NM_010438	0.000102991	1.31752	E15.5 * Wnt1 up vs E15.5 * Ctrl
10462822	Exoc6	NM_175353	0.00432881	1.31744	E15.5 * Wnt1 up vs E15.5 * Ctrl
10438189	Slc7a4	NM_144852	0.00243604	1.31738	E15.5 * Wnt1 up vs E15.5 * Ctrl
10361846	Reps1	NM_009048	0.000374214	1.31738	E15.5 * Wnt1 up vs E15.5 * Ctrl
10607539	Mbtps2	NM_172307	0.000373656	1.31732	E15.5 * Wnt1 up vs E15.5 * Ctrl
10421685	Serp2	BC100483	2.12384e-005	1.31669	E15.5 * Wnt1 up vs E15.5 * Ctrl
10374485	Peli1	NM_023324	0.00141125	1.31666	E15.5 * Wnt1 up vs E15.5 * Ctrl
10421214	Rhobtb2	NM_153514	0.0022502	1.31653	E15.5 * Wnt1 up vs E15.5 * Ctrl
10504172	4933409K07Rik	BC059060	0.000720117	1.31631	E15.5 * Wnt1 up vs E15.5 * Ctrl
10417212	Itgbl1	NM_145467	8.49883e-005	1.31594	E15.5 * Wnt1 up vs E15.5 * Ctrl
10551487	LOC434156	ENSMUST00000(	0.000815217	1.31585	E15.5 * Wnt1 up vs E15.5 * Ctrl
10400413	Garnl1	NM_001112714	0.00631033	1.31583	E15.5 * Wnt1 up vs E15.5 * Ctrl
10516982	Stx12	NM_133887	4.23636e-005	1.31582	E15.5 * Wnt1 up vs E15.5 * Ctrl
10396177	Actr10	NM_019785	0.00584873	1.31548	E15.5 * Wnt1 up vs E15.5 * Ctrl
10398286		---	0.00130045	1.31526	E15.5 * Wnt1 up vs E15.5 * Ctrl
10359024	EG433367	ENSMUST00000(	0.00135657	1.31505	E15.5 * Wnt1 up vs E15.5 * Ctrl
10385699	Rmnd5b	NM_025346	0.00915615	1.31503	E15.5 * Wnt1 up vs E15.5 * Ctrl
10472155	Kcnj3	NM_008426	6.43534e-005	1.31492	E15.5 * Wnt1 up vs E15.5 * Ctrl
10500469	Pde4dip	NM_001039376	5.61082e-005	1.31486	E15.5 * Wnt1 up vs E15.5 * Ctrl
10519392	Krit1	NM_030675	4.2383e-005	1.3148	E15.5 * Wnt1 up vs E15.5 * Ctrl
10419416	3632451O06Rik	NM_026142	0.0144584	1.31479	E15.5 * Wnt1 up vs E15.5 * Ctrl
10536895	Atp6v1f	NM_025381	0.0018566	1.31422	E15.5 * Wnt1 up vs E15.5 * Ctrl
10417002	A830021K08Rik	AK043702	0.00712856	1.31415	E15.5 * Wnt1 up vs E15.5 * Ctrl
10585201	Timm8b	NM_013897	4.77414e-005	1.314	E15.5 * Wnt1 up vs E15.5 * Ctrl
10497197	5330432E05Rik	AK030563	0.00541969	1.31396	E15.5 * Wnt1 up vs E15.5 * Ctrl
10427895	Basp1	NM_027395	1.58091e-007	1.31384	E15.5 * Wnt1 up vs E15.5 * Ctrl
10593471		---	0.00124357	1.31365	E15.5 * Wnt1 up vs E15.5 * Ctrl
10578391	Smarce1	NM_020618	4.65786e-006	1.31328	E15.5 * Wnt1 up vs E15.5 * Ctrl
10387816	Rnasek	NM_173742	0.00287644	1.31323	E15.5 * Wnt1 up vs E15.5 * Ctrl

10407192	Slc38a9	NM_178746	0.00640311	1.3129	E15.5 * Wnt1 up vs E15.5 * Ctrl
10448168	Fpr-rs3	ENSMUST00000(	0.00156307	1.31269	E15.5 * Wnt1 up vs E15.5 * Ctrl
10539111	Tmem150	NM_144916	0.0138073	1.31256	E15.5 * Wnt1 up vs E15.5 * Ctrl
10361065	Mfsd7b	NM_001081259	0.00102457	1.31234	E15.5 * Wnt1 up vs E15.5 * Ctrl
10498998	D930015E06Rik	NM_172681	0.000189022	1.31233	E15.5 * Wnt1 up vs E15.5 * Ctrl
10603387	Hdac6	NM_010413	0.000143938	1.31232	E15.5 * Wnt1 up vs E15.5 * Ctrl
10372891	Srgap1	NM_001081037	0.000634687	1.31219	E15.5 * Wnt1 up vs E15.5 * Ctrl
10357698	Tmcc2	NM_178874	0.00147624	1.31213	E15.5 * Wnt1 up vs E15.5 * Ctrl
10387723	2810408A11Rik	BC069874	0.0105313	1.31193	E15.5 * Wnt1 up vs E15.5 * Ctrl
10578515	Ankrd37	NM_001039562	0.00058347	1.31188	E15.5 * Wnt1 up vs E15.5 * Ctrl
10407814	Tbce	NM_178337	0.00790348	1.31188	E15.5 * Wnt1 up vs E15.5 * Ctrl
10499493	Hcn3	NM_008227	0.0025955	1.31178	E15.5 * Wnt1 up vs E15.5 * Ctrl
10599200	Pgrmc1	NM_016783	0.000702232	1.31174	E15.5 * Wnt1 up vs E15.5 * Ctrl
10395064	Tssc1	NM_201357	0.0012195	1.31163	E15.5 * Wnt1 up vs E15.5 * Ctrl
10474112	Traf6	NM_009424	0.000880408	1.31149	E15.5 * Wnt1 up vs E15.5 * Ctrl
10399430	Ddx1	NM_134040	3.28598e-006	1.31134	E15.5 * Wnt1 up vs E15.5 * Ctrl
10400350	Cfl2	NM_007688	0.000601135	1.31134	E15.5 * Wnt1 up vs E15.5 * Ctrl
10508253	Dlgap3	NM_198618	0.000175744	1.31128	E15.5 * Wnt1 up vs E15.5 * Ctrl
10468929	Nmt2	NM_008708	0.000450965	1.31096	E15.5 * Wnt1 up vs E15.5 * Ctrl
10384885	Spnb2	NM_175836	1.62785e-005	1.31094	E15.5 * Wnt1 up vs E15.5 * Ctrl
10503882	Rngtt	NM_011884	0.000587078	1.31065	E15.5 * Wnt1 up vs E15.5 * Ctrl
10579609	Fcho1	NM_028715	0.000800605	1.31053	E15.5 * Wnt1 up vs E15.5 * Ctrl
10452571	Ptprm	NM_008984	0.00950698	1.3104	E15.5 * Wnt1 up vs E15.5 * Ctrl
10554160	ENSMUSG0000C	ENSMUST00000(	3.91277e-005	1.31038	E15.5 * Wnt1 up vs E15.5 * Ctrl
10562578	Pop4	NM_025390	0.00235339	1.31031	E15.5 * Wnt1 up vs E15.5 * Ctrl
10562687	Iglon5	ENSMUST00000(	0.0124191	1.3103	E15.5 * Wnt1 up vs E15.5 * Ctrl
10391963	Nsf	NM_008740	0.00315204	1.3103	E15.5 * Wnt1 up vs E15.5 * Ctrl
10508819	Cd164l2	NM_027152	0.00180939	1.31016	E15.5 * Wnt1 up vs E15.5 * Ctrl
10478897	Ptpn1	NM_011201	0.000160532	1.31002	E15.5 * Wnt1 up vs E15.5 * Ctrl
10478014	Vstm2l	NM_198627	0.00145429	1.30945	E15.5 * Wnt1 up vs E15.5 * Ctrl
10396800	Plekhh1	ENSMUST00000(	2.2748e-005	1.3091	E15.5 * Wnt1 up vs E15.5 * Ctrl
10596433	Glyctk	NM_174846	6.93727e-005	1.3091	E15.5 * Wnt1 up vs E15.5 * Ctrl
10502329	Cisd2	NM_025902	0.002434	1.30909	E15.5 * Wnt1 up vs E15.5 * Ctrl
10569972	Lass4	NM_026058	0.0122839	1.30885	E15.5 * Wnt1 up vs E15.5 * Ctrl
10419370	Exoc5	NM_207214	0.0112653	1.30869	E15.5 * Wnt1 up vs E15.5 * Ctrl
10590383	Deb1	NM_026794	0.000265783	1.30848	E15.5 * Wnt1 up vs E15.5 * Ctrl
10521927	Tbc1d19	NM_144517	0.000483527	1.30835	E15.5 * Wnt1 up vs E15.5 * Ctrl
10559498		---	0.0117529	1.30825	E15.5 * Wnt1 up vs E15.5 * Ctrl
10493548	Pmvk	NM_026784	0.00221299	1.30822	E15.5 * Wnt1 up vs E15.5 * Ctrl
10491958	ENSMUSG0000C	ENSMUST00000(	0.00437471	1.30817	E15.5 * Wnt1 up vs E15.5 * Ctrl
10445992	Shd	NM_001159523	0.00368161	1.30797	E15.5 * Wnt1 up vs E15.5 * Ctrl
10593318		---	0.00491901	1.30787	E15.5 * Wnt1 up vs E15.5 * Ctrl
10373400	Myl6b	NM_172259	0.0072629	1.30782	E15.5 * Wnt1 up vs E15.5 * Ctrl
10578582	D030016E14Rik	NM_177240	9.47966e-005	1.30754	E15.5 * Wnt1 up vs E15.5 * Ctrl
10486107	Srp14	NM_009273	0.000558006	1.30752	E15.5 * Wnt1 up vs E15.5 * Ctrl
10411019	Msh3	NM_010829	0.00292139	1.3075	E15.5 * Wnt1 up vs E15.5 * Ctrl
10358611	Hmcn1	NM_001024720	0.00805362	1.30743	E15.5 * Wnt1 up vs E15.5 * Ctrl
10521481	Jakmip1	NM_178394	0.00217993	1.30697	E15.5 * Wnt1 up vs E15.5 * Ctrl
10591270	Olfm2	NM_173777	7.34282e-005	1.30692	E15.5 * Wnt1 up vs E15.5 * Ctrl
10474545	Slc12a6	NM_133649	0.000199842	1.30654	E15.5 * Wnt1 up vs E15.5 * Ctrl
10441657	Prr18	NM_178774	0.000208986	1.30649	E15.5 * Wnt1 up vs E15.5 * Ctrl
10425354	Mgat3	NM_010795	5.08162e-005	1.30616	E15.5 * Wnt1 up vs E15.5 * Ctrl
10498284	Wwtr1	NM_133784	0.000291852	1.30605	E15.5 * Wnt1 up vs E15.5 * Ctrl
10498415	Dhx36	NM_028136	0.00012492	1.30576	E15.5 * Wnt1 up vs E15.5 * Ctrl
10460782	Gpha2	NM_130453	0.0068903	1.30564	E15.5 * Wnt1 up vs E15.5 * Ctrl
10599232	Nkap	NM_025937	0.014363	1.30555	E15.5 * Wnt1 up vs E15.5 * Ctrl
10382010	Wdr68	NM_027946	3.69315e-005	1.30549	E15.5 * Wnt1 up vs E15.5 * Ctrl
10583697	Smarca4	NM_011417	8.52927e-005	1.30543	E15.5 * Wnt1 up vs E15.5 * Ctrl
10384811	Ccdc104	NM_025740	0.00150782	1.30526	E15.5 * Wnt1 up vs E15.5 * Ctrl
10577838	Ddhd2	BC046229	0.000471497	1.30482	E15.5 * Wnt1 up vs E15.5 * Ctrl
10471978	Epc2	NM_172663	0.00174514	1.3048	E15.5 * Wnt1 up vs E15.5 * Ctrl
10552433	BC043301	NM_001008549	0.00158048	1.30474	E15.5 * Wnt1 up vs E15.5 * Ctrl
10515808	LOC674677	ENSMUST00000(	0.000345254	1.30467	E15.5 * Wnt1 up vs E15.5 * Ctrl
10487879	Rnf24	NM_178607	0.00214768	1.30435	E15.5 * Wnt1 up vs E15.5 * Ctrl
10358637	Hmcn1	NM_001024720	0.0130284	1.3043	E15.5 * Wnt1 up vs E15.5 * Ctrl
10506603	Ssbp3	NM_023672	0.00437226	1.30428	E15.5 * Wnt1 up vs E15.5 * Ctrl

10383061	Ccdc40	NM_175430	0.000979858	1.30399	E15.5 * Wnt1 up vs E15.5 * Ctrl
10488322	A230067G21Rik	NM_001033348	0.00482868	1.3037	E15.5 * Wnt1 up vs E15.5 * Ctrl
10559788	Olfr1349	NM_207136	0.00806853	1.30369	E15.5 * Wnt1 up vs E15.5 * Ctrl
10468089	Mgea5	NM_023799	1.45497e-006	1.30362	E15.5 * Wnt1 up vs E15.5 * Ctrl
10428983	Fam49b	NM_144846	0.000238636	1.30362	E15.5 * Wnt1 up vs E15.5 * Ctrl
10586331	Igdcc3	NM_008988	0.00263926	1.3032	E15.5 * Wnt1 up vs E15.5 * Ctrl
10391746	Gpatch8	NM_001159492	0.0131735	1.30316	E15.5 * Wnt1 up vs E15.5 * Ctrl
10416899	Ndfip2	NM_029561	0.000859169	1.30299	E15.5 * Wnt1 up vs E15.5 * Ctrl
10434869	Ccdc50	NM_026202	1.52406e-005	1.30297	E15.5 * Wnt1 up vs E15.5 * Ctrl
10515051	Osbpl9	NM_133885	1.59657e-005	1.30289	E15.5 * Wnt1 up vs E15.5 * Ctrl
10519007	Tprgl	NM_026388	0.000901522	1.30288	E15.5 * Wnt1 up vs E15.5 * Ctrl
10477353	Mapre1	NM_007896	8.0495e-006	1.30285	E15.5 * Wnt1 up vs E15.5 * Ctrl
10575376	Ftsjd1	NM_146215	0.000614823	1.30259	E15.5 * Wnt1 up vs E15.5 * Ctrl
10511803	2610029I01Rik	ENSMUST00000	0.00672391	1.30239	E15.5 * Wnt1 up vs E15.5 * Ctrl
10460221	Chka	NM_013490	1.64538e-005	1.30214	E15.5 * Wnt1 up vs E15.5 * Ctrl
10581299	Agrp	NM_007427	0.000378243	1.30206	E15.5 * Wnt1 up vs E15.5 * Ctrl
10434754	BC106179	BC106179	0.00604778	1.30203	E15.5 * Wnt1 up vs E15.5 * Ctrl
10598678	Usp9x	NM_009481	8.20562e-006	1.30201	E15.5 * Wnt1 up vs E15.5 * Ctrl
10512682	Zbtb5	NM_173399	0.0132465	1.30198	E15.5 * Wnt1 up vs E15.5 * Ctrl
10436983	Dopey2	NM_027293	0.00196689	1.30192	E15.5 * Wnt1 up vs E15.5 * Ctrl
10398117	Bdkrb2	NM_009747	0.00900337	1.3017	E15.5 * Wnt1 up vs E15.5 * Ctrl
10463200	Npl2	NM_026152	0.00915734	1.3017	E15.5 * Wnt1 up vs E15.5 * Ctrl
10347427	Stk36	NM_175031	0.00287586	1.30134	E15.5 * Wnt1 up vs E15.5 * Ctrl
10375572	Olfr1382	NM_001011790	0.00489631	1.30132	E15.5 * Wnt1 up vs E15.5 * Ctrl
10597627	Oxsr1	NM_133985	0.000305307	1.30116	E15.5 * Wnt1 up vs E15.5 * Ctrl
10540509	Grm7	NM_177328	0.00409219	1.30101	E15.5 * Wnt1 up vs E15.5 * Ctrl
10413951	Arhgap22	NM_153800	2.62125e-006	1.30097	E15.5 * Wnt1 up vs E15.5 * Ctrl
10569071	Hras1	NM_001130444	3.71825e-005	1.30091	E15.5 * Wnt1 up vs E15.5 * Ctrl
10597833	Sec22c	NM_178677	0.0108823	1.30069	E15.5 * Wnt1 up vs E15.5 * Ctrl
10433536	Clec16a	NM_177562	0.00013578	1.30064	E15.5 * Wnt1 up vs E15.5 * Ctrl
10592926	Tmem25	NM_027865	0.000238893	1.30062	E15.5 * Wnt1 up vs E15.5 * Ctrl
10563558	Sergef	NM_013789	2.94966e-006	1.30056	E15.5 * Wnt1 up vs E15.5 * Ctrl
10588192	Msl2	AK173208	0.00144049	1.30017	E15.5 * Wnt1 up vs E15.5 * Ctrl
10482880	Baz2b	NM_001001182	1.22614e-005	1.29991	E15.5 * Wnt1 up vs E15.5 * Ctrl
10606609	Tspan6	NM_019656	0.000315569	1.29983	E15.5 * Wnt1 up vs E15.5 * Ctrl
10531899	Klhl8	NM_178741	0.00172815	1.2997	E15.5 * Wnt1 up vs E15.5 * Ctrl
10404380	Dusp22	NM_001037955	0.000411592	1.29939	E15.5 * Wnt1 up vs E15.5 * Ctrl
10568311	Zfp668	NM_146259	0.00149171	1.29906	E15.5 * Wnt1 up vs E15.5 * Ctrl
10438907	Gp5	NM_008148	0.00593028	1.29884	E15.5 * Wnt1 up vs E15.5 * Ctrl
10560842	Zfp94	NM_009568	0.000117522	1.2988	E15.5 * Wnt1 up vs E15.5 * Ctrl
10527528	Smarce1	NM_020618	4.33299e-006	1.29876	E15.5 * Wnt1 up vs E15.5 * Ctrl
10549097	Ldhb	NM_008492	1.42936e-005	1.29843	E15.5 * Wnt1 up vs E15.5 * Ctrl
10415332	Rec8	NM_020002	0.000702618	1.29835	E15.5 * Wnt1 up vs E15.5 * Ctrl
10523750	D930016D06Rik	BC082597	0.00528971	1.29827	E15.5 * Wnt1 up vs E15.5 * Ctrl
10404975	Id4	NM_031166	0.00458147	1.29826	E15.5 * Wnt1 up vs E15.5 * Ctrl
10577222	2410022L05Rik	NM_025556	0.000379928	1.29823	E15.5 * Wnt1 up vs E15.5 * Ctrl
10484318	Nckap1	NM_016965	5.0294e-005	1.29817	E15.5 * Wnt1 up vs E15.5 * Ctrl
10347862	Mff	NM_029409	0.000190569	1.29802	E15.5 * Wnt1 up vs E15.5 * Ctrl
10441815	Sod2	NM_013671	0.000944416	1.29783	E15.5 * Wnt1 up vs E15.5 * Ctrl
10562360	Gpi1	NM_008155	0.00239514	1.29782	E15.5 * Wnt1 up vs E15.5 * Ctrl
10535720	1700001J03Rik	BC048528	0.00383168	1.29781	E15.5 * Wnt1 up vs E15.5 * Ctrl
10375713	Mgat4b	NM_145926	0.00263075	1.29773	E15.5 * Wnt1 up vs E15.5 * Ctrl
10398936	Pacs2	NM_001081170	0.00725629	1.2976	E15.5 * Wnt1 up vs E15.5 * Ctrl
10578045	Nrg1	NM_178591	0.000216865	1.29745	E15.5 * Wnt1 up vs E15.5 * Ctrl
10441952	2210404J11Rik	NM_001039552	0.00954464	1.29734	E15.5 * Wnt1 up vs E15.5 * Ctrl
10562989	Cpt1c	NM_153679	0.00163394	1.2973	E15.5 * Wnt1 up vs E15.5 * Ctrl
10574033	Nup93	NM_172410	1.81965e-007	1.29726	E15.5 * Wnt1 up vs E15.5 * Ctrl
10580872	Zfp319	NM_024467	0.000766958	1.29718	E15.5 * Wnt1 up vs E15.5 * Ctrl
10578619	Cdkn2aip	NM_172407	0.000480273	1.2971	E15.5 * Wnt1 up vs E15.5 * Ctrl
10480324	2810030E01Rik	NM_028317	0.0141311	1.29699	E15.5 * Wnt1 up vs E15.5 * Ctrl
10607363		---	0.00710775	1.29676	E15.5 * Wnt1 up vs E15.5 * Ctrl
10520782	Zfp512	NM_172993	0.00764237	1.29659	E15.5 * Wnt1 up vs E15.5 * Ctrl
10349828	Lrrn2	NM_010732	0.0062533	1.29641	E15.5 * Wnt1 up vs E15.5 * Ctrl
10505187	Ugcg	NM_011673	0.0139402	1.29622	E15.5 * Wnt1 up vs E15.5 * Ctrl
10582978	8430410K20Rik	BC014729	0.000857902	1.29617	E15.5 * Wnt1 up vs E15.5 * Ctrl
10383532	Narf	NM_026272	0.0024534	1.29613	E15.5 * Wnt1 up vs E15.5 * Ctrl

10563949		---	0.0102055	1.29541	E15.5 * Wnt1 up vs E15.5 * Ctrl
10517566		---	0.00158292	1.29525	E15.5 * Wnt1 up vs E15.5 * Ctrl
10604932	Cd99l2	NM_138309	0.0145856	1.29511	E15.5 * Wnt1 up vs E15.5 * Ctrl
10553644		---	0.0142503	1.29494	E15.5 * Wnt1 up vs E15.5 * Ctrl
10494527	Rnf115	NM_026406	0.00245806	1.29487	E15.5 * Wnt1 up vs E15.5 * Ctrl
10567464	Dnahc3	XM_355934	0.00189048	1.29474	E15.5 * Wnt1 up vs E15.5 * Ctrl
10573234	Lphn1	NM_181039	0.00607136	1.29462	E15.5 * Wnt1 up vs E15.5 * Ctrl
10552303	EG434172	NM_001033782	0.00990427	1.2945	E15.5 * Wnt1 up vs E15.5 * Ctrl
10433008	Map3k12	NM_009582	0.00020496	1.29449	E15.5 * Wnt1 up vs E15.5 * Ctrl
10372965	Usp15	NM_027604	8.74276e-006	1.29416	E15.5 * Wnt1 up vs E15.5 * Ctrl
10411287	Btf3l4	NM_027453	1.45944e-005	1.29405	E15.5 * Wnt1 up vs E15.5 * Ctrl
10367830	Grm1	NM_001114333	0.000465269	1.294	E15.5 * Wnt1 up vs E15.5 * Ctrl
10487613	Pdyn	NM_018863	0.00140519	1.29397	E15.5 * Wnt1 up vs E15.5 * Ctrl
10567394	Pdilt	ENSMUST00000(	0.000575637	1.29389	E15.5 * Wnt1 up vs E15.5 * Ctrl
10498323	4932431H17Rik	NM_001081262	0.00880742	1.29386	E15.5 * Wnt1 up vs E15.5 * Ctrl
10344990	Crispld1	NM_031402	0.000333022	1.29384	E15.5 * Wnt1 up vs E15.5 * Ctrl
10461930	D030056L22Rik	NM_177640	0.000855641	1.2938	E15.5 * Wnt1 up vs E15.5 * Ctrl
10580532	EG626231	ENSMUST00000(	0.0114325	1.29373	E15.5 * Wnt1 up vs E15.5 * Ctrl
10557585	Zfp553	NM_146201	0.00146306	1.29365	E15.5 * Wnt1 up vs E15.5 * Ctrl
10363901	Etv5	NM_023794	0.00215126	1.29341	E15.5 * Wnt1 up vs E15.5 * Ctrl
10380415	Cdc34	BC094502	0.0124835	1.29328	E15.5 * Wnt1 up vs E15.5 * Ctrl
10600082	Nsdhl	NM_010941	0.00876974	1.29327	E15.5 * Wnt1 up vs E15.5 * Ctrl
10471784	Rabgap1	NM_146121	1.22356e-005	1.29318	E15.5 * Wnt1 up vs E15.5 * Ctrl
10514177	Bnc2	NM_172870	0.0112726	1.29291	E15.5 * Wnt1 up vs E15.5 * Ctrl
10435791		---	2.73947e-005	1.29258	E15.5 * Wnt1 up vs E15.5 * Ctrl
10422055	ENSMUSG0000C	ENSMUST00000(	0.00181704	1.29255	E15.5 * Wnt1 up vs E15.5 * Ctrl
10477167	Mcts2	NM_025543	0.00100248	1.29242	E15.5 * Wnt1 up vs E15.5 * Ctrl
10601062	Dlg3	NM_016747	0.000204087	1.2923	E15.5 * Wnt1 up vs E15.5 * Ctrl
10477630	Dynlrb1	NM_025947	2.16114e-010	1.29199	E15.5 * Wnt1 up vs E15.5 * Ctrl
10568780	Mapk1ip1	NM_001045483	0.0048937	1.2919	E15.5 * Wnt1 up vs E15.5 * Ctrl
10539857	EG434064	NR_026990	0.000141882	1.29185	E15.5 * Wnt1 up vs E15.5 * Ctrl
10439798	Dzip3	NM_001110017	0.00260218	1.29163	E15.5 * Wnt1 up vs E15.5 * Ctrl
10365870	Nr2c1	NM_011629	0.00597264	1.29141	E15.5 * Wnt1 up vs E15.5 * Ctrl
10347669	Inha	NM_010564	0.0142251	1.29128	E15.5 * Wnt1 up vs E15.5 * Ctrl
10571741		---	0.00103253	1.29125	E15.5 * Wnt1 up vs E15.5 * Ctrl
10547807	Eno2	NM_013509	0.00705291	1.29113	E15.5 * Wnt1 up vs E15.5 * Ctrl
10462096		---	0.00650608	1.2906	E15.5 * Wnt1 up vs E15.5 * Ctrl
10536390	Glcci1	NM_133236	5.62178e-005	1.29025	E15.5 * Wnt1 up vs E15.5 * Ctrl
10463486	Btrc	NM_001037758	0.00189866	1.29015	E15.5 * Wnt1 up vs E15.5 * Ctrl
10558239	Hmx3	NM_008257	0.0104567	1.28956	E15.5 * Wnt1 up vs E15.5 * Ctrl
10424349	Sqle	NM_009270	0.001028	1.28954	E15.5 * Wnt1 up vs E15.5 * Ctrl
10486616	Ubr1	NM_009461	0.000279525	1.28928	E15.5 * Wnt1 up vs E15.5 * Ctrl
10594551	Zfp609	NM_172536	0.00092612	1.28926	E15.5 * Wnt1 up vs E15.5 * Ctrl
10483381	Stk39	NM_016866	5.98802e-005	1.28923	E15.5 * Wnt1 up vs E15.5 * Ctrl
10567591	Usp31	NM_001033173	9.97072e-005	1.28896	E15.5 * Wnt1 up vs E15.5 * Ctrl
10517383	Nipal3	NM_028995	0.0011411	1.28894	E15.5 * Wnt1 up vs E15.5 * Ctrl
10529549	Tbc1d14	NM_001113362	0.000689898	1.2886	E15.5 * Wnt1 up vs E15.5 * Ctrl
10574607	EG13909	NM_144511	0.00736173	1.28858	E15.5 * Wnt1 up vs E15.5 * Ctrl
10358654	Hmcn1	NM_001024720	0.00462508	1.28855	E15.5 * Wnt1 up vs E15.5 * Ctrl
10523905	Mtf2	NM_013827	8.55021e-007	1.28832	E15.5 * Wnt1 up vs E15.5 * Ctrl
10504139	4933409K07Rik	BC059060	0.00215017	1.28796	E15.5 * Wnt1 up vs E15.5 * Ctrl
10453634	ENSMUSG0000C	ENSMUST00000(	0.00450045	1.28774	E15.5 * Wnt1 up vs E15.5 * Ctrl
10547408	Erc1	NM_053204	0.0146925	1.28719	E15.5 * Wnt1 up vs E15.5 * Ctrl
10511226	Acap3	NM_207223	0.00151699	1.28719	E15.5 * Wnt1 up vs E15.5 * Ctrl
10597134	Scap	NM_001001144	0.00141984	1.2871	E15.5 * Wnt1 up vs E15.5 * Ctrl
10596900	Tcta	NM_133986	0.00912748	1.28708	E15.5 * Wnt1 up vs E15.5 * Ctrl
10462005	Tmem2	NM_031997	0.000441352	1.28681	E15.5 * Wnt1 up vs E15.5 * Ctrl
10579915	Smarca5	NM_053124	0.00196686	1.2868	E15.5 * Wnt1 up vs E15.5 * Ctrl
10413185	Zmiz1	NM_183208	0.00206175	1.28631	E15.5 * Wnt1 up vs E15.5 * Ctrl
10593193		---	0.00792541	1.28616	E15.5 * Wnt1 up vs E15.5 * Ctrl
10589099	Ip6k2	NM_029634	0.00474648	1.28589	E15.5 * Wnt1 up vs E15.5 * Ctrl
10600372	B230340J04Rik	ENSMUST00000(	0.00139832	1.28579	E15.5 * Wnt1 up vs E15.5 * Ctrl
10526356	Rhbdd2	NM_146002	0.000977609	1.28576	E15.5 * Wnt1 up vs E15.5 * Ctrl
10483737	Atf2	NM_001025093	0.000326845	1.28572	E15.5 * Wnt1 up vs E15.5 * Ctrl
10569265	Tollip	NM_023764	0.000743767	1.28562	E15.5 * Wnt1 up vs E15.5 * Ctrl
10533734	Abcb9	NM_019875	0.00439682	1.28538	E15.5 * Wnt1 up vs E15.5 * Ctrl

10508412	Fndc5	NM_027402	0.0052278	1.285	E15.5 * Wnt1 up vs E15.5 * Ctrl
10421717	Gm1587	NM_001033440	0.00024915	1.28492	E15.5 * Wnt1 up vs E15.5 * Ctrl
10576696	Insr	NM_010568	4.94785e-005	1.28491	E15.5 * Wnt1 up vs E15.5 * Ctrl
10489065	Ndrg3	NM_013865	0.000516402	1.28467	E15.5 * Wnt1 up vs E15.5 * Ctrl
10351013	Rc3h1	NM_001024952	0.00105048	1.28452	E15.5 * Wnt1 up vs E15.5 * Ctrl
10533085	Pebp1	NM_018858	0.00480108	1.28443	E15.5 * Wnt1 up vs E15.5 * Ctrl
10382492	Otop3	NM_027132	0.000701237	1.28441	E15.5 * Wnt1 up vs E15.5 * Ctrl
10453141	Sos1	NM_009231	0.00167057	1.2842	E15.5 * Wnt1 up vs E15.5 * Ctrl
10449523	Pxt1	NM_153390	0.00116884	1.28418	E15.5 * Wnt1 up vs E15.5 * Ctrl
10427997	Ankrd33b	NM_027496	0.00353589	1.28413	E15.5 * Wnt1 up vs E15.5 * Ctrl
10439675	Tmprss7	NM_172455	1.82361e-005	1.28397	E15.5 * Wnt1 up vs E15.5 * Ctrl
10493891	Ywhaz	NM_011740	2.1484e-008	1.28391	E15.5 * Wnt1 up vs E15.5 * Ctrl
10545528	Pigp	NM_001159616	0.0070967	1.28371	E15.5 * Wnt1 up vs E15.5 * Ctrl
10373810	Dusp18	NM_173745	0.00705289	1.28363	E15.5 * Wnt1 up vs E15.5 * Ctrl
10479154	Tubb1	NM_001080971	0.00469976	1.28357	E15.5 * Wnt1 up vs E15.5 * Ctrl
10598882	Uba1	NM_009457	1.84341e-006	1.28325	E15.5 * Wnt1 up vs E15.5 * Ctrl
10534489	Ccl26	NM_001013412	0.000148719	1.28316	E15.5 * Wnt1 up vs E15.5 * Ctrl
10457302	Epc1	NM_007935	6.6826e-006	1.28311	E15.5 * Wnt1 up vs E15.5 * Ctrl
10510254	Fv1	NM_010244	0.0136888	1.28275	E15.5 * Wnt1 up vs E15.5 * Ctrl
10349401	Gpr39	NM_027677	0.000275562	1.28249	E15.5 * Wnt1 up vs E15.5 * Ctrl
10464015	Shoc2	NM_019658	3.88102e-005	1.28246	E15.5 * Wnt1 up vs E15.5 * Ctrl
10401684	Angel1	NM_144524	0.00362682	1.28245	E15.5 * Wnt1 up vs E15.5 * Ctrl
10441642	Brp44l	NM_018819	0.0113653	1.28232	E15.5 * Wnt1 up vs E15.5 * Ctrl
10479268	Wtap	NM_001113533	0.000667569	1.28215	E15.5 * Wnt1 up vs E15.5 * Ctrl
10563820	Svip	ENSMUST00000(	0.00310454	1.2817	E15.5 * Wnt1 up vs E15.5 * Ctrl
10596664	Cyb561d2	NM_019720	0.000305075	1.28162	E15.5 * Wnt1 up vs E15.5 * Ctrl
10536805	Fam71f1	NM_207258	0.010376	1.28152	E15.5 * Wnt1 up vs E15.5 * Ctrl
10526781	Mblac1	NM_177878	0.00543381	1.28143	E15.5 * Wnt1 up vs E15.5 * Ctrl
10399632		---	0.00395134	1.28134	E15.5 * Wnt1 up vs E15.5 * Ctrl
10450579	Ddr1	NM_007584	0.0138794	1.28105	E15.5 * Wnt1 up vs E15.5 * Ctrl
10452874	Atp6v1f	NM_025381	0.00694355	1.28065	E15.5 * Wnt1 up vs E15.5 * Ctrl
10450845	Mog	NM_010814	0.00235619	1.28054	E15.5 * Wnt1 up vs E15.5 * Ctrl
10487154	Secisbp2l	NM_177608	0.000127319	1.2805	E15.5 * Wnt1 up vs E15.5 * Ctrl
10372151	Lrriq1	BC051400	0.0100309	1.28045	E15.5 * Wnt1 up vs E15.5 * Ctrl
10429428	Gm628	AK132992	0.00358386	1.28044	E15.5 * Wnt1 up vs E15.5 * Ctrl
10455104	Pcdhb15	NM_053140	0.0041415	1.28029	E15.5 * Wnt1 up vs E15.5 * Ctrl
10413874	Ogdhl	NM_001081130	0.0084437	1.28026	E15.5 * Wnt1 up vs E15.5 * Ctrl
10603373	Pqbp1	NM_019478	0.000433786	1.28026	E15.5 * Wnt1 up vs E15.5 * Ctrl
10358668	Hmcn1	NM_001024720	0.00160765	1.28021	E15.5 * Wnt1 up vs E15.5 * Ctrl
10362520	Hdac2	NM_008229	1.84059e-005	1.28009	E15.5 * Wnt1 up vs E15.5 * Ctrl
10545891	Cml1	NM_023160	0.00454603	1.28006	E15.5 * Wnt1 up vs E15.5 * Ctrl
10518492	Ptchd2	NM_001083342	0.00237518	1.27971	E15.5 * Wnt1 up vs E15.5 * Ctrl
10439915		---	0.000805197	1.27968	E15.5 * Wnt1 up vs E15.5 * Ctrl
10493565	Adar	NM_001038587	2.02427e-005	1.27956	E15.5 * Wnt1 up vs E15.5 * Ctrl
10383767	Osbp2	NM_152818	0.0120108	1.27952	E15.5 * Wnt1 up vs E15.5 * Ctrl
10378681	Inpp5k	NM_008916	0.0085503	1.27947	E15.5 * Wnt1 up vs E15.5 * Ctrl
10371420	Prdm4	NM_181650	0.00806562	1.27934	E15.5 * Wnt1 up vs E15.5 * Ctrl
10483246		---	0.00788147	1.27919	E15.5 * Wnt1 up vs E15.5 * Ctrl
10607244		---	0.003115	1.27917	E15.5 * Wnt1 up vs E15.5 * Ctrl
10429341	Ptk2	NM_007982	0.000974898	1.27884	E15.5 * Wnt1 up vs E15.5 * Ctrl
10563941		---	0.00056513	1.27882	E15.5 * Wnt1 up vs E15.5 * Ctrl
10371032	Diras1	NM_145217	0.000939699	1.27881	E15.5 * Wnt1 up vs E15.5 * Ctrl
10403658	Gng4	NM_010317	0.000613748	1.27877	E15.5 * Wnt1 up vs E15.5 * Ctrl
10563963		---	0.000421021	1.27875	E15.5 * Wnt1 up vs E15.5 * Ctrl
10563969		---	0.000421021	1.27875	E15.5 * Wnt1 up vs E15.5 * Ctrl
10563977		---	0.000421021	1.27875	E15.5 * Wnt1 up vs E15.5 * Ctrl
10563979		---	0.000421021	1.27875	E15.5 * Wnt1 up vs E15.5 * Ctrl
10563983	100040985	XM_001475615	0.000421021	1.27875	E15.5 * Wnt1 up vs E15.5 * Ctrl
10563985		---	0.000421021	1.27875	E15.5 * Wnt1 up vs E15.5 * Ctrl
10416793	Uchl3	NM_016723	0.00749954	1.27816	E15.5 * Wnt1 up vs E15.5 * Ctrl
10486322		---	8.17258e-005	1.27738	E15.5 * Wnt1 up vs E15.5 * Ctrl
10491363	Mfn1	NM_024200	3.16708e-005	1.27731	E15.5 * Wnt1 up vs E15.5 * Ctrl
10348653	Gpc1	NM_016696	0.00690391	1.27725	E15.5 * Wnt1 up vs E15.5 * Ctrl
10374934	Psme4	NM_134013	0.000483121	1.27698	E15.5 * Wnt1 up vs E15.5 * Ctrl
10384737	Papolg	NM_172555	0.00299166	1.27683	E15.5 * Wnt1 up vs E15.5 * Ctrl
10599576	2700038C09Rik	NM_025598	0.000284312	1.27673	E15.5 * Wnt1 up vs E15.5 * Ctrl

10544243	Mrps33	NM_010270	0.000671022	1.27654	E15.5 * Wnt1 up vs E15.5 * Ctrl
10486552	Lrrc57	NM_001159612	0.000301362	1.27634	E15.5 * Wnt1 up vs E15.5 * Ctrl
10406171	Tppp	NM_182839	0.00546419	1.27631	E15.5 * Wnt1 up vs E15.5 * Ctrl
10535449	E130309D02Rik	NM_172726	0.0100322	1.27599	E15.5 * Wnt1 up vs E15.5 * Ctrl
10574985	Slc7a6	NM_178798	0.00275231	1.27588	E15.5 * Wnt1 up vs E15.5 * Ctrl
10401564	1110018G07Rik	NM_178065	0.000153813	1.27579	E15.5 * Wnt1 up vs E15.5 * Ctrl
10451104	Tmem151b	NM_001013749	0.00667646	1.27543	E15.5 * Wnt1 up vs E15.5 * Ctrl
10407097	Pde4d	NM_011056	4.88934e-006	1.27529	E15.5 * Wnt1 up vs E15.5 * Ctrl
10422892	2410089E03Rik	ENSMUST00000(	0.00867096	1.27524	E15.5 * Wnt1 up vs E15.5 * Ctrl
10557508	Doc2a	NM_010069	0.000873355	1.27514	E15.5 * Wnt1 up vs E15.5 * Ctrl
10432566		---	0.00151725	1.275	E15.5 * Wnt1 up vs E15.5 * Ctrl
10359770	Pou2f1	NM_011137	0.000237991	1.27494	E15.5 * Wnt1 up vs E15.5 * Ctrl
10466423	Cep78	NM_198019	0.000589887	1.27478	E15.5 * Wnt1 up vs E15.5 * Ctrl
10551469	Dyrk1b	NM_001037957	0.00058292	1.27475	E15.5 * Wnt1 up vs E15.5 * Ctrl
10399265	Ncoa1	NM_010881	0.000176431	1.27466	E15.5 * Wnt1 up vs E15.5 * Ctrl
10555550	Clpb	NM_009191	0.00020559	1.27459	E15.5 * Wnt1 up vs E15.5 * Ctrl
10571849	Fbxo8	NM_015791	0.0130133	1.27453	E15.5 * Wnt1 up vs E15.5 * Ctrl
10390653	Med24	NM_011869	0.000104901	1.27394	E15.5 * Wnt1 up vs E15.5 * Ctrl
10442294	Vmn2r115	NM_001104579	0.012078	1.27365	E15.5 * Wnt1 up vs E15.5 * Ctrl
10429216	Fam135b	NM_177819	0.00255992	1.27356	E15.5 * Wnt1 up vs E15.5 * Ctrl
10484884	Olfr1272	NM_146980	0.00211752	1.27344	E15.5 * Wnt1 up vs E15.5 * Ctrl
10508436	Sync	NM_023485	0.00458984	1.27328	E15.5 * Wnt1 up vs E15.5 * Ctrl
10579043	Lzts1	NM_199364	0.00256269	1.27326	E15.5 * Wnt1 up vs E15.5 * Ctrl
10542515	Capza3	NM_007605	0.00940948	1.27319	E15.5 * Wnt1 up vs E15.5 * Ctrl
10418300	Cacna2d3	NM_009785	0.00895911	1.27309	E15.5 * Wnt1 up vs E15.5 * Ctrl
10582837		---	0.0110739	1.27292	E15.5 * Wnt1 up vs E15.5 * Ctrl
10391043	Krt9	NM_201255	0.00329823	1.2729	E15.5 * Wnt1 up vs E15.5 * Ctrl
10436892	Itsn1	NM_010587	0.0010124	1.27285	E15.5 * Wnt1 up vs E15.5 * Ctrl
10581625	2400003C14Rik	BC039052	0.00389609	1.27262	E15.5 * Wnt1 up vs E15.5 * Ctrl
10454831	Paip2	NM_026420	0.000369008	1.2725	E15.5 * Wnt1 up vs E15.5 * Ctrl
10403466	Dip2c	NM_001081426	0.00280988	1.27235	E15.5 * Wnt1 up vs E15.5 * Ctrl
10422718	Ttc33	NM_026213	9.44748e-005	1.27208	E15.5 * Wnt1 up vs E15.5 * Ctrl
10472970	Hoxd4	NM_010469	0.00538301	1.27194	E15.5 * Wnt1 up vs E15.5 * Ctrl
10450744	677333	XR_031734	0.000108553	1.2719	E15.5 * Wnt1 up vs E15.5 * Ctrl
10453256	Kcng3	NM_153512	0.000114205	1.27188	E15.5 * Wnt1 up vs E15.5 * Ctrl
10536483	Tes	NM_207176	1.68048e-007	1.27163	E15.5 * Wnt1 up vs E15.5 * Ctrl
10435162	Lrch3	NM_001081255	0.00036204	1.2713	E15.5 * Wnt1 up vs E15.5 * Ctrl
10534839	Pop7	NM_028753	0.00720191	1.27123	E15.5 * Wnt1 up vs E15.5 * Ctrl
10515994	Smap2	NM_133716	0.00173702	1.27114	E15.5 * Wnt1 up vs E15.5 * Ctrl
10574866	Pard6a	NM_001047436	0.00701171	1.27104	E15.5 * Wnt1 up vs E15.5 * Ctrl
10404815	Sirt5	NM_178848	0.00242551	1.27093	E15.5 * Wnt1 up vs E15.5 * Ctrl
10358023	Gpr37l1	NM_134438	0.00949322	1.27074	E15.5 * Wnt1 up vs E15.5 * Ctrl
10394812		---	0.000561328	1.2707	E15.5 * Wnt1 up vs E15.5 * Ctrl
10439960	Cep97	NM_028815	0.00383546	1.27041	E15.5 * Wnt1 up vs E15.5 * Ctrl
10405287	Higd2a	NM_025933	0.00857503	1.27041	E15.5 * Wnt1 up vs E15.5 * Ctrl
10521415	Ablim2	NM_177678	0.000732303	1.2702	E15.5 * Wnt1 up vs E15.5 * Ctrl
10451481	BC032203	NM_001100452	0.013253	1.27018	E15.5 * Wnt1 up vs E15.5 * Ctrl
10346764	Abi2	NM_198127	5.74915e-005	1.26997	E15.5 * Wnt1 up vs E15.5 * Ctrl
10563991		---	0.000641104	1.26991	E15.5 * Wnt1 up vs E15.5 * Ctrl
10521136	Whsc1	NM_001081102	6.68501e-005	1.26982	E15.5 * Wnt1 up vs E15.5 * Ctrl
10515894	1700041C02Rik	NM_029286	0.00440615	1.26973	E15.5 * Wnt1 up vs E15.5 * Ctrl
10419062	Sh2d4b	NM_177816	0.000492974	1.26949	E15.5 * Wnt1 up vs E15.5 * Ctrl
10505587	Kdm4c	NM_144787	0.000797763	1.26945	E15.5 * Wnt1 up vs E15.5 * Ctrl
10386758	Slc5a10	NM_001033227	0.00319272	1.26944	E15.5 * Wnt1 up vs E15.5 * Ctrl
10380501	Dlx3	NM_010055	0.000348236	1.26926	E15.5 * Wnt1 up vs E15.5 * Ctrl
10599927	Aff2	NM_008032	0.0147959	1.26897	E15.5 * Wnt1 up vs E15.5 * Ctrl
10510399	Masp2	NM_001003893	0.0041497	1.26848	E15.5 * Wnt1 up vs E15.5 * Ctrl
10358656	Hmcn1	NM_001024720	0.00268401	1.26844	E15.5 * Wnt1 up vs E15.5 * Ctrl
10480139	C1ql3	NM_153155	0.0146763	1.26832	E15.5 * Wnt1 up vs E15.5 * Ctrl
10570144	Arhgef7	NM_001113518	0.000150069	1.26831	E15.5 * Wnt1 up vs E15.5 * Ctrl
10345492	Cnnm3	NM_001039551	0.000396404	1.26811	E15.5 * Wnt1 up vs E15.5 * Ctrl
10545417	Mat2a	NM_145569	1.15378e-005	1.2681	E15.5 * Wnt1 up vs E15.5 * Ctrl
10418898	Ppyr1	NM_008919	0.0143374	1.26792	E15.5 * Wnt1 up vs E15.5 * Ctrl
10439566	Atp6v1a	NM_007508	0.000201063	1.26786	E15.5 * Wnt1 up vs E15.5 * Ctrl
10405619	5133401N09Rik	NM_198004	0.000927599	1.2674	E15.5 * Wnt1 up vs E15.5 * Ctrl
10365601	Gnptab	NM_001004164	0.00138534	1.26734	E15.5 * Wnt1 up vs E15.5 * Ctrl

10576056	Map1lc3b	NM_026160	0.000613757	1.26726	E15.5 * Wnt1 up vs E15.5 * Ctrl
10420155	Dhrs1	NM_026819	0.00892119	1.26689	E15.5 * Wnt1 up vs E15.5 * Ctrl
10387108	OTTMUSG00000	ENSMUST00000	0.00378375	1.2668	E15.5 * Wnt1 up vs E15.5 * Ctrl
10443764	Slc37a1	NM_153062	2.05248e-005	1.26675	E15.5 * Wnt1 up vs E15.5 * Ctrl
10512059	Lingo2	ENSMUST00000	0.0108657	1.26641	E15.5 * Wnt1 up vs E15.5 * Ctrl
10472994	Mtx2	NM_016804	5.92884e-005	1.2664	E15.5 * Wnt1 up vs E15.5 * Ctrl
10531323	G3bp2	NM_011816	0.000121366	1.26626	E15.5 * Wnt1 up vs E15.5 * Ctrl
10513143	Ptpn3	NM_011207	0.0010166	1.26616	E15.5 * Wnt1 up vs E15.5 * Ctrl
10437992	Dnm1l	NM_152816	0.000142495	1.26594	E15.5 * Wnt1 up vs E15.5 * Ctrl
10562419	Lrp3	NM_001024707	0.00683225	1.2658	E15.5 * Wnt1 up vs E15.5 * Ctrl
10514366	Glrx3	NM_023140	0.0138787	1.26573	E15.5 * Wnt1 up vs E15.5 * Ctrl
10430384	Elfn2	NM_183141	0.00380344	1.26542	E15.5 * Wnt1 up vs E15.5 * Ctrl
10584855	Scn2b	NM_001014761	0.00205004	1.26497	E15.5 * Wnt1 up vs E15.5 * Ctrl
10607246	Tmem29	ENSMUST00000	0.00298472	1.26489	E15.5 * Wnt1 up vs E15.5 * Ctrl
10524004	Pcgf3	NM_172716	7.74119e-005	1.26487	E15.5 * Wnt1 up vs E15.5 * Ctrl
10455656	Hsd17b4	NM_008292	3.2358e-005	1.26486	E15.5 * Wnt1 up vs E15.5 * Ctrl
10591253	5730601F06Rik	NM_001082485	0.0012836	1.26479	E15.5 * Wnt1 up vs E15.5 * Ctrl
10564482	Synm	NM_201639	0.00169616	1.26479	E15.5 * Wnt1 up vs E15.5 * Ctrl
10529385	Zfyve28	NM_001015039	0.00332659	1.26474	E15.5 * Wnt1 up vs E15.5 * Ctrl
10564290	Klf13	NM_021366	0.0068382	1.26452	E15.5 * Wnt1 up vs E15.5 * Ctrl
10562416	Cebpg	NM_009884	0.00108366	1.26449	E15.5 * Wnt1 up vs E15.5 * Ctrl
10470543	Gm347	BC070440	0.0018944	1.26426	E15.5 * Wnt1 up vs E15.5 * Ctrl
10544252	E330009J07Rik	NM_175528	0.000180261	1.26405	E15.5 * Wnt1 up vs E15.5 * Ctrl
10585610	Ptpn9	NM_019651	0.000145094	1.26396	E15.5 * Wnt1 up vs E15.5 * Ctrl
10388337	Pafah1b1	NM_013625	2.72113e-005	1.26395	E15.5 * Wnt1 up vs E15.5 * Ctrl
10594480	Rab11a	NM_017382	3.93427e-005	1.26365	E15.5 * Wnt1 up vs E15.5 * Ctrl
10577432		---	0.00136046	1.26359	E15.5 * Wnt1 up vs E15.5 * Ctrl
10468180	Psd	NM_028627	0.00505187	1.26352	E15.5 * Wnt1 up vs E15.5 * Ctrl
10385155	Fbll1	NM_001004147	0.00509214	1.26352	E15.5 * Wnt1 up vs E15.5 * Ctrl
10501649	Rtcd1	NM_025517	7.19268e-006	1.26343	E15.5 * Wnt1 up vs E15.5 * Ctrl
10552668	Lrrc4b	NM_198250	0.00388639	1.26323	E15.5 * Wnt1 up vs E15.5 * Ctrl
10520003	Armc10	NM_026034	0.0132626	1.26312	E15.5 * Wnt1 up vs E15.5 * Ctrl
10521587	Dnaja1	NM_008298	2.30269e-006	1.26294	E15.5 * Wnt1 up vs E15.5 * Ctrl
10547858	Gnb3	NM_013530	0.00369095	1.26292	E15.5 * Wnt1 up vs E15.5 * Ctrl
10346310	Mobkl3	NM_025283	0.000651655	1.26283	E15.5 * Wnt1 up vs E15.5 * Ctrl
10583459	5730577I03Rik	NM_175110	0.000674403	1.26271	E15.5 * Wnt1 up vs E15.5 * Ctrl
10552982	Plekha4	NM_148927	0.00105178	1.26244	E15.5 * Wnt1 up vs E15.5 * Ctrl
10454229	Klhl14	NM_001081403	0.00477983	1.2621	E15.5 * Wnt1 up vs E15.5 * Ctrl
10578904	Cpe	NM_013494	0.00029946	1.26206	E15.5 * Wnt1 up vs E15.5 * Ctrl
10448748	Nubp2	NM_011956	0.000601247	1.26206	E15.5 * Wnt1 up vs E15.5 * Ctrl
10356514	Iqca	ENSMUST00000	0.00655248	1.26205	E15.5 * Wnt1 up vs E15.5 * Ctrl
10368992	EG668252	XR_033560	0.0101672	1.26175	E15.5 * Wnt1 up vs E15.5 * Ctrl
10384685	1700093K21Rik	NM_026105	0.00509565	1.26173	E15.5 * Wnt1 up vs E15.5 * Ctrl
10533644	Vps33a	NM_029929	0.000242653	1.26166	E15.5 * Wnt1 up vs E15.5 * Ctrl
10551462		---	0.00334205	1.26166	E15.5 * Wnt1 up vs E15.5 * Ctrl
10421012	Ppp2r2a	NM_028032	5.39887e-005	1.26159	E15.5 * Wnt1 up vs E15.5 * Ctrl
10519354	Pex1	NM_027777	0.00050455	1.26154	E15.5 * Wnt1 up vs E15.5 * Ctrl
10467328	5730455O13Rik	NM_001081075	0.00270667	1.26151	E15.5 * Wnt1 up vs E15.5 * Ctrl
10463789	Neurl1a	NM_021360	0.00055819	1.26119	E15.5 * Wnt1 up vs E15.5 * Ctrl
10455199	Rell2	NM_153793	0.00813324	1.26108	E15.5 * Wnt1 up vs E15.5 * Ctrl
10387655	Spem1	NM_028855	0.00845317	1.26108	E15.5 * Wnt1 up vs E15.5 * Ctrl
10576944	ENSMUSG00000	ENSMUST00000	0.00214799	1.26107	E15.5 * Wnt1 up vs E15.5 * Ctrl
10589057		---	0.00424862	1.26102	E15.5 * Wnt1 up vs E15.5 * Ctrl
10424945	Kifc2	NM_010630	0.00391254	1.26091	E15.5 * Wnt1 up vs E15.5 * Ctrl
10375926	Ppp2ca	NM_019411	4.22292e-006	1.26068	E15.5 * Wnt1 up vs E15.5 * Ctrl
10484704	Olfr1157	NM_146849	0.000947915	1.26067	E15.5 * Wnt1 up vs E15.5 * Ctrl
10582229	1110003O08Rik	ENSMUST00000	0.0114464	1.26047	E15.5 * Wnt1 up vs E15.5 * Ctrl
10521168	Nat8l	NM_001001985	0.000558401	1.26038	E15.5 * Wnt1 up vs E15.5 * Ctrl
10580056	Lphn1	NM_181039	0.0040474	1.2602	E15.5 * Wnt1 up vs E15.5 * Ctrl
10413897	Ercc6	NM_001081221	0.00184236	1.26009	E15.5 * Wnt1 up vs E15.5 * Ctrl
10588874	Bsn	NM_007567	0.0035018	1.25998	E15.5 * Wnt1 up vs E15.5 * Ctrl
10578157	Tnks	NM_175091	0.000680713	1.2599	E15.5 * Wnt1 up vs E15.5 * Ctrl
10585533	Dnajb6	NM_011847	0.000926	1.25984	E15.5 * Wnt1 up vs E15.5 * Ctrl
10376245	Gria1	NM_001113325	0.00108489	1.2596	E15.5 * Wnt1 up vs E15.5 * Ctrl
10456194	Napg	NM_028017	0.000243382	1.25959	E15.5 * Wnt1 up vs E15.5 * Ctrl
10415159	Thtpa	NM_153083	0.00448161	1.25937	E15.5 * Wnt1 up vs E15.5 * Ctrl

10456140	Sh3tc2	NM_172628	0.0133484	1.25937	E15.5 * Wnt1 up vs E15.5 * Ctrl
10568099	Tmem219	NM_026827	0.00347908	1.25922	E15.5 * Wnt1 up vs E15.5 * Ctrl
10422393		---	0.00442871	1.25919	E15.5 * Wnt1 up vs E15.5 * Ctrl
10449471	Srpk1	NM_016795	0.000295863	1.25911	E15.5 * Wnt1 up vs E15.5 * Ctrl
10399478	Lpin1	NM_015763	0.00956342	1.25906	E15.5 * Wnt1 up vs E15.5 * Ctrl
10400131	ENSMUSG0000(	ENSMUST00000(	0.00172781	1.25899	E15.5 * Wnt1 up vs E15.5 * Ctrl
10413670	Pbrm1	NM_001081251	0.0001543	1.25889	E15.5 * Wnt1 up vs E15.5 * Ctrl
10535900		---	0.0109228	1.25889	E15.5 * Wnt1 up vs E15.5 * Ctrl
10398885	AW555464	NM_001024602	0.00300717	1.25883	E15.5 * Wnt1 up vs E15.5 * Ctrl
10479615	C430010C01	AK082896	0.00644613	1.25883	E15.5 * Wnt1 up vs E15.5 * Ctrl
10385523		---	0.00652998	1.2587	E15.5 * Wnt1 up vs E15.5 * Ctrl
10420348	Zmym5	NM_144842	0.00172305	1.25857	E15.5 * Wnt1 up vs E15.5 * Ctrl
10345423	Plekhb2	NM_145516	0.00402542	1.25842	E15.5 * Wnt1 up vs E15.5 * Ctrl
10378964	Sez6	NM_021286	0.00663675	1.25835	E15.5 * Wnt1 up vs E15.5 * Ctrl
10456317	Zfp532	NM_207255	2.1528e-005	1.25833	E15.5 * Wnt1 up vs E15.5 * Ctrl
10484563	Olfr1043	NM_146577	0.00731862	1.25831	E15.5 * Wnt1 up vs E15.5 * Ctrl
10406953	Trim23	NM_030731	0.0130211	1.25827	E15.5 * Wnt1 up vs E15.5 * Ctrl
10525751	Ddx55	NM_026409	0.00444481	1.25824	E15.5 * Wnt1 up vs E15.5 * Ctrl
10391178	Dnajc7	NM_019795	1.64101e-005	1.25818	E15.5 * Wnt1 up vs E15.5 * Ctrl
10555235	Arrb1	NM_177231	0.000348237	1.25817	E15.5 * Wnt1 up vs E15.5 * Ctrl
10494735	Gdap2	NM_010269	0.0145051	1.25815	E15.5 * Wnt1 up vs E15.5 * Ctrl
10499299	Hapln2	NM_022031	0.000125949	1.25793	E15.5 * Wnt1 up vs E15.5 * Ctrl
10381419	Nbr1	NM_008676	0.00642835	1.25765	E15.5 * Wnt1 up vs E15.5 * Ctrl
10563829	Mrps33	NM_010270	0.000780152	1.25706	E15.5 * Wnt1 up vs E15.5 * Ctrl
10360187	Vangl2	NM_033509	0.00595779	1.25701	E15.5 * Wnt1 up vs E15.5 * Ctrl
10408487	Uqcrfs1	NM_025710	0.00138964	1.25696	E15.5 * Wnt1 up vs E15.5 * Ctrl
10514971	Zyg11a	ENSMUST00000	0.0071205	1.25682	E15.5 * Wnt1 up vs E15.5 * Ctrl
10461898	Rfk	NM_019437	0.00102029	1.25681	E15.5 * Wnt1 up vs E15.5 * Ctrl
10431726	Yaf2	NM_024189	0.00031356	1.25671	E15.5 * Wnt1 up vs E15.5 * Ctrl
10583402	Zfp317	NM_172918	0.00421053	1.25651	E15.5 * Wnt1 up vs E15.5 * Ctrl
10603321		---	0.0147819	1.25643	E15.5 * Wnt1 up vs E15.5 * Ctrl
10346255	Ormdl1	NM_145517	0.00503886	1.25633	E15.5 * Wnt1 up vs E15.5 * Ctrl
10468533	Gpam	NM_008149	0.00153325	1.25611	E15.5 * Wnt1 up vs E15.5 * Ctrl
10349771	EG638532	XM_914588	0.0106337	1.25605	E15.5 * Wnt1 up vs E15.5 * Ctrl
10379262	Nf1	NM_010897	0.000283691	1.25597	E15.5 * Wnt1 up vs E15.5 * Ctrl
10375343	Rnf145	NM_028862	0.0034406	1.25594	E15.5 * Wnt1 up vs E15.5 * Ctrl
10481868	Dnajb6	NM_011847	0.000978071	1.25568	E15.5 * Wnt1 up vs E15.5 * Ctrl
10412011	Kif2a	NM_008442	3.53206e-008	1.25557	E15.5 * Wnt1 up vs E15.5 * Ctrl
10386705	Rnf112	NM_009548	0.003891	1.25536	E15.5 * Wnt1 up vs E15.5 * Ctrl
10568921	Caly	NM_026769	0.000851022	1.25534	E15.5 * Wnt1 up vs E15.5 * Ctrl
10596680	Sema3b	NM_001042779	0.0112162	1.25532	E15.5 * Wnt1 up vs E15.5 * Ctrl
10559500	Ttyh1	AK011493	0.00499702	1.25521	E15.5 * Wnt1 up vs E15.5 * Ctrl
10417183	Pcca	NM_144844	0.00378443	1.25511	E15.5 * Wnt1 up vs E15.5 * Ctrl
10516435	Zmym4	NM_001114399	0.00165079	1.255	E15.5 * Wnt1 up vs E15.5 * Ctrl
10443808	Ndufv3	NM_030087	0.00326298	1.25496	E15.5 * Wnt1 up vs E15.5 * Ctrl
10440918	Tmem50b	NM_030018	0.00406641	1.25474	E15.5 * Wnt1 up vs E15.5 * Ctrl
10388545	Abr	NM_198895	0.00664212	1.25448	E15.5 * Wnt1 up vs E15.5 * Ctrl
10559343	Shank2	NM_001113373	0.00521474	1.25427	E15.5 * Wnt1 up vs E15.5 * Ctrl
10467425	Sorbs1	NM_178362	0.00136038	1.2542	E15.5 * Wnt1 up vs E15.5 * Ctrl
10478077	B230339M05Rik	NM_177658	0.000198863	1.25416	E15.5 * Wnt1 up vs E15.5 * Ctrl
10409265	Auh	NM_016709	0.0100499	1.25409	E15.5 * Wnt1 up vs E15.5 * Ctrl
10541446	Cpamd8	NM_008646	0.00336986	1.25408	E15.5 * Wnt1 up vs E15.5 * Ctrl
10534281	Clip2	NM_009990	0.0139875	1.25405	E15.5 * Wnt1 up vs E15.5 * Ctrl
10431711	Slc2a13	NM_001033633	0.0117335	1.2537	E15.5 * Wnt1 up vs E15.5 * Ctrl
10372600	Cct2	NM_007636	7.87016e-007	1.25365	E15.5 * Wnt1 up vs E15.5 * Ctrl
10436552	Rbm11	NM_198302	0.0129253	1.25343	E15.5 * Wnt1 up vs E15.5 * Ctrl
10461214	Tmem223	NM_025791	0.00290664	1.25336	E15.5 * Wnt1 up vs E15.5 * Ctrl
10515220	Faah	NM_010173	0.00464366	1.25323	E15.5 * Wnt1 up vs E15.5 * Ctrl
10537375	Ubn2	NM_177185	0.000760482	1.25323	E15.5 * Wnt1 up vs E15.5 * Ctrl
10565330	Zfand6	NM_022985	0.00839847	1.25322	E15.5 * Wnt1 up vs E15.5 * Ctrl
10559853	Clcn4-2	NM_011334	0.000102516	1.25315	E15.5 * Wnt1 up vs E15.5 * Ctrl
10395976	Dnajb6	NM_011847	0.0011053	1.25314	E15.5 * Wnt1 up vs E15.5 * Ctrl
10375216	Pank3	NM_145962	0.00444062	1.25313	E15.5 * Wnt1 up vs E15.5 * Ctrl
10593605	Cul5	NM_027807	0.00167737	1.2531	E15.5 * Wnt1 up vs E15.5 * Ctrl
10435661	Gpr156	NM_153394	0.00321146	1.253	E15.5 * Wnt1 up vs E15.5 * Ctrl
10382393	Dnaic2	NM_001034878	0.000132332	1.25299	E15.5 * Wnt1 up vs E15.5 * Ctrl

10546936	Prrt3	NM_172487	0.0100095	1.25282	E15.5 * Wnt1 up vs E15.5 * Ctrl
10481435	Ccbl1	NM_172404	0.00399255	1.25272	E15.5 * Wnt1 up vs E15.5 * Ctrl
10402096	Ttc7b	NM_001033213	0.00113924	1.25253	E15.5 * Wnt1 up vs E15.5 * Ctrl
10583392		---	0.00321918	1.25253	E15.5 * Wnt1 up vs E15.5 * Ctrl
10482336	Lrp1b	NM_053011	0.000292291	1.25251	E15.5 * Wnt1 up vs E15.5 * Ctrl
10493009	Arhgef11	NM_001003912	0.0124349	1.25234	E15.5 * Wnt1 up vs E15.5 * Ctrl
10452248	Slc25a41	NM_175333	0.00966361	1.25222	E15.5 * Wnt1 up vs E15.5 * Ctrl
10486754	Hisppd2a	NM_178795	0.00714652	1.25217	E15.5 * Wnt1 up vs E15.5 * Ctrl
10431612	Rabl2a	NM_026817	0.00502415	1.2521	E15.5 * Wnt1 up vs E15.5 * Ctrl
10407307	Mocs2	NM_013826	0.00798313	1.25188	E15.5 * Wnt1 up vs E15.5 * Ctrl
10568956	Olfr60	NM_146955	0.00118529	1.25188	E15.5 * Wnt1 up vs E15.5 * Ctrl
10454414	Pik3c3	NM_181414	0.000276732	1.2517	E15.5 * Wnt1 up vs E15.5 * Ctrl
10437677	Prm3	NM_013638	0.00364193	1.25151	E15.5 * Wnt1 up vs E15.5 * Ctrl
10526229	ENSMUSG0000C	ENSMUST00000	0.00466983	1.2513	E15.5 * Wnt1 up vs E15.5 * Ctrl
10595805	Rasa2	NM_053268	0.00410076	1.25128	E15.5 * Wnt1 up vs E15.5 * Ctrl
10356145	Slc19a3	NM_030556	0.0118012	1.25119	E15.5 * Wnt1 up vs E15.5 * Ctrl
10380830	B230217C12Rik	NM_001080935	0.00579874	1.25114	E15.5 * Wnt1 up vs E15.5 * Ctrl
10502058	BC002199	NM_145964	3.51264e-005	1.25109	E15.5 * Wnt1 up vs E15.5 * Ctrl
10358177	5730559C18Rik	BC053100	0.0142071	1.25088	E15.5 * Wnt1 up vs E15.5 * Ctrl
10404264	Prl	NM_011164	0.0052976	1.2506	E15.5 * Wnt1 up vs E15.5 * Ctrl
10415262	Wdr23	NM_133734	0.000503046	1.25058	E15.5 * Wnt1 up vs E15.5 * Ctrl
10404904	Rbm24	NM_001081425	0.00425874	1.25057	E15.5 * Wnt1 up vs E15.5 * Ctrl
10532716	1700069L16Rik	ENSMUST00000(	0.000819485	1.25048	E15.5 * Wnt1 up vs E15.5 * Ctrl
10477058	Scrt2	ENSMUST00000(	8.52694e-005	1.25047	E15.5 * Wnt1 up vs E15.5 * Ctrl
10454809	Matr3	NM_010771	0.00114736	1.25042	E15.5 * Wnt1 up vs E15.5 * Ctrl
10575993	6430548M08Rik	BC027126	0.00682179	1.25041	E15.5 * Wnt1 up vs E15.5 * Ctrl
10413919	Prrxl1	NM_001001796	0.00824947	1.25016	E15.5 * Wnt1 up vs E15.5 * Ctrl
10353533	Smap1	NM_028534	0.000909887	1.25015	E15.5 * Wnt1 up vs E15.5 * Ctrl
10428157	Rnf19a	NM_013923	0.00222088	1.24996	E15.5 * Wnt1 up vs E15.5 * Ctrl
10379677	Rasl10b	NM_001013386	0.0144024	1.24996	E15.5 * Wnt1 up vs E15.5 * Ctrl
10558548	Kndc1	NM_177261	0.0111508	1.24989	E15.5 * Wnt1 up vs E15.5 * Ctrl
10456346	Sec11c	NM_025468	0.00912558	1.24982	E15.5 * Wnt1 up vs E15.5 * Ctrl
10457838	Zfp397os	NM_001080810	0.0140367	1.24968	E15.5 * Wnt1 up vs E15.5 * Ctrl
10590279	Myrip	NM_144557	0.00125223	1.2496	E15.5 * Wnt1 up vs E15.5 * Ctrl
10453114	Dhx57	NM_198942	0.000470839	1.24953	E15.5 * Wnt1 up vs E15.5 * Ctrl
10428176	Snx31	NM_025712	0.00449416	1.2495	E15.5 * Wnt1 up vs E15.5 * Ctrl
10538526	Avl9	NM_030235	0.00183616	1.24944	E15.5 * Wnt1 up vs E15.5 * Ctrl
10594289	Glce	NM_033320	0.00154045	1.24942	E15.5 * Wnt1 up vs E15.5 * Ctrl
10552708	Kcnc3	NM_008422	1.00012e-006	1.24931	E15.5 * Wnt1 up vs E15.5 * Ctrl
10504294	Rusc2	NM_001037709	0.00460948	1.24928	E15.5 * Wnt1 up vs E15.5 * Ctrl
10488575	Psmf1	NM_212446	0.00443894	1.24915	E15.5 * Wnt1 up vs E15.5 * Ctrl
10568997	Sirt3	NM_022433	0.00372301	1.24913	E15.5 * Wnt1 up vs E15.5 * Ctrl
10429944	Scrt1	NM_130893	0.000256543	1.24908	E15.5 * Wnt1 up vs E15.5 * Ctrl
10544756	Hoxa3	NM_010452	7.58403e-005	1.24906	E15.5 * Wnt1 up vs E15.5 * Ctrl
10373577	Ormdl2	NM_024180	0.0106683	1.24892	E15.5 * Wnt1 up vs E15.5 * Ctrl
10467230	Ide	NM_031156	0.00600274	1.24892	E15.5 * Wnt1 up vs E15.5 * Ctrl
10498827	Fnip2	ENSMUST00000(	0.00199398	1.24878	E15.5 * Wnt1 up vs E15.5 * Ctrl
10400668	Sdccag1	NM_025441	0.00125808	1.24873	E15.5 * Wnt1 up vs E15.5 * Ctrl
10438530	Clcn2	NM_009900	7.28919e-006	1.24848	E15.5 * Wnt1 up vs E15.5 * Ctrl
10399214	Rab10	NM_016676	0.000101013	1.24839	E15.5 * Wnt1 up vs E15.5 * Ctrl
10512384	BC049635	NM_177785	0.00167254	1.24828	E15.5 * Wnt1 up vs E15.5 * Ctrl
10434880	Hrasls	NM_013751	0.0138604	1.24825	E15.5 * Wnt1 up vs E15.5 * Ctrl
10451918	Ubxn6	NM_024432	0.000189245	1.24815	E15.5 * Wnt1 up vs E15.5 * Ctrl
10393866	Sirt7	NM_153056	0.00956484	1.24804	E15.5 * Wnt1 up vs E15.5 * Ctrl
10432378	Lmbr1l	NM_029098	0.00645067	1.24767	E15.5 * Wnt1 up vs E15.5 * Ctrl
10404941	Aof1	NM_172262	0.00845633	1.24762	E15.5 * Wnt1 up vs E15.5 * Ctrl
10424853	Brp16	NM_021555	0.00650022	1.24751	E15.5 * Wnt1 up vs E15.5 * Ctrl
10558740	OTTMUSG00000	XM_001480612	0.0105733	1.24746	E15.5 * Wnt1 up vs E15.5 * Ctrl
10348963	Cntnap5b	NM_172851	0.00639689	1.24716	E15.5 * Wnt1 up vs E15.5 * Ctrl
10435031	Ubxn7	NM_177633	0.000605554	1.24713	E15.5 * Wnt1 up vs E15.5 * Ctrl
10600688	Map3k7ip3	NM_025729	0.00594999	1.24703	E15.5 * Wnt1 up vs E15.5 * Ctrl
10453900	Riok3	NM_024182	0.00379327	1.24678	E15.5 * Wnt1 up vs E15.5 * Ctrl
10495385	4921515J06Rik	NM_025723	0.0052839	1.24671	E15.5 * Wnt1 up vs E15.5 * Ctrl
10446756	Ypel5	NM_027166	0.00395586	1.24652	E15.5 * Wnt1 up vs E15.5 * Ctrl
10346330	Plcl1	NM_001114663	0.00817169	1.24642	E15.5 * Wnt1 up vs E15.5 * Ctrl
10596545	Rad54l2	NM_030730	0.000227463	1.24624	E15.5 * Wnt1 up vs E15.5 * Ctrl

10443876	677156	NM_001100187	0.00809673	1.24612	E15.5 * Wnt1 up vs E15.5 * Ctrl
10410892	Rasa1	NM_145452	0.00112122	1.24578	E15.5 * Wnt1 up vs E15.5 * Ctrl
10382788	Galr2	NM_010254	0.000578162	1.24575	E15.5 * Wnt1 up vs E15.5 * Ctrl
10565620	Rsf1	NM_001081267	0.000415639	1.24553	E15.5 * Wnt1 up vs E15.5 * Ctrl
10459210	4933429F08Rik	NM_177828	0.0126608	1.2455	E15.5 * Wnt1 up vs E15.5 * Ctrl
10393944	Rfng	NM_009053	0.00136395	1.2455	E15.5 * Wnt1 up vs E15.5 * Ctrl
10348618	Asb1	NM_001039126	0.00165026	1.24545	E15.5 * Wnt1 up vs E15.5 * Ctrl
10535312	Radil	NM_178702	0.00191629	1.2454	E15.5 * Wnt1 up vs E15.5 * Ctrl
10465625	Otub1	NM_134150	0.00868323	1.24521	E15.5 * Wnt1 up vs E15.5 * Ctrl
10433940	Mapk1	NM_011949	0.000168425	1.24515	E15.5 * Wnt1 up vs E15.5 * Ctrl
10381032	Casc3	NM_138660	0.00478737	1.24515	E15.5 * Wnt1 up vs E15.5 * Ctrl
10441053		---	0.00285338	1.24512	E15.5 * Wnt1 up vs E15.5 * Ctrl
10368136	EG237300	NM_177706	0.00172965	1.24476	E15.5 * Wnt1 up vs E15.5 * Ctrl
10566723	Lmo1	NM_057173	0.00247611	1.24445	E15.5 * Wnt1 up vs E15.5 * Ctrl
10345457		---	0.00125279	1.24442	E15.5 * Wnt1 up vs E15.5 * Ctrl
10427369	Pde1b	NM_008800	0.0021035	1.24434	E15.5 * Wnt1 up vs E15.5 * Ctrl
10534912	6430598A04Rik	NM_175521	0.00330088	1.24408	E15.5 * Wnt1 up vs E15.5 * Ctrl
10378848	Hsp90aa1	NM_010480	1.04526e-005	1.24407	E15.5 * Wnt1 up vs E15.5 * Ctrl
10509014	D4Wsu53e	BC043057	0.00131201	1.24387	E15.5 * Wnt1 up vs E15.5 * Ctrl
10388716	ENSMUSG0000C	ENSMUST00000(	0.00628288	1.24385	E15.5 * Wnt1 up vs E15.5 * Ctrl
10477854	Epb4.1l1	NM_013510	0.00121819	1.24377	E15.5 * Wnt1 up vs E15.5 * Ctrl
10528723	Mll3	NM_001081383	0.000272822	1.24371	E15.5 * Wnt1 up vs E15.5 * Ctrl
10574096	Ap3s1	NM_009681	0.00676982	1.2437	E15.5 * Wnt1 up vs E15.5 * Ctrl
10374842	Ccdc88a	NM_176841	0.00188483	1.24353	E15.5 * Wnt1 up vs E15.5 * Ctrl
10560304	Calm3	NM_007590	0.00471294	1.24352	E15.5 * Wnt1 up vs E15.5 * Ctrl
10569222	ENSMUSG0000C	AK016549	0.00963159	1.24347	E15.5 * Wnt1 up vs E15.5 * Ctrl
10408379	Prl8a6	NM_011167	0.000271515	1.24347	E15.5 * Wnt1 up vs E15.5 * Ctrl
10398695	Mark3	NM_021516	9.41334e-005	1.24346	E15.5 * Wnt1 up vs E15.5 * Ctrl
10516658	Ccdc28b	NM_025455	0.000346393	1.24345	E15.5 * Wnt1 up vs E15.5 * Ctrl
10466248	Stx3	NM_001025307	0.00573784	1.24342	E15.5 * Wnt1 up vs E15.5 * Ctrl
10398111	Tcl1b4	NM_013774	0.007639	1.24337	E15.5 * Wnt1 up vs E15.5 * Ctrl
10404827	Nol7	NM_023554	0.00411857	1.24329	E15.5 * Wnt1 up vs E15.5 * Ctrl
10498647	B3galnt1	NM_020026	0.000120912	1.24312	E15.5 * Wnt1 up vs E15.5 * Ctrl
10544248		---	0.00884087	1.24306	E15.5 * Wnt1 up vs E15.5 * Ctrl
10433433	BC024814	BC024814	0.000207884	1.24292	E15.5 * Wnt1 up vs E15.5 * Ctrl
10347018	Pth2r	NM_139270	0.0131824	1.24272	E15.5 * Wnt1 up vs E15.5 * Ctrl
10345913	Nck2	NM_010879	0.00235789	1.2427	E15.5 * Wnt1 up vs E15.5 * Ctrl
10406843	Btf3	NM_145455	0.0020914	1.24253	E15.5 * Wnt1 up vs E15.5 * Ctrl
10460621	1810058N15Rik	ENSMUST00000(	0.00963346	1.24252	E15.5 * Wnt1 up vs E15.5 * Ctrl
10558872	Tmem80	NM_027797	0.00584966	1.24251	E15.5 * Wnt1 up vs E15.5 * Ctrl
10392936	Nt5c	NM_015807	0.000854959	1.2425	E15.5 * Wnt1 up vs E15.5 * Ctrl
10591763	Zfp810	NM_145612	0.00784989	1.24246	E15.5 * Wnt1 up vs E15.5 * Ctrl
10566846	Dennd5a	NM_021494	0.000106434	1.24237	E15.5 * Wnt1 up vs E15.5 * Ctrl
10369531	Tspan15	NM_197996	0.0139084	1.24226	E15.5 * Wnt1 up vs E15.5 * Ctrl
10399387	Msgn1	NM_019544	0.000805573	1.24193	E15.5 * Wnt1 up vs E15.5 * Ctrl
10566488	Trim3	NM_018880	0.00548883	1.24192	E15.5 * Wnt1 up vs E15.5 * Ctrl
10426650	Tuba1c	NM_009448	2.8078e-005	1.24191	E15.5 * Wnt1 up vs E15.5 * Ctrl
10375893	Sar1b	NM_025535	0.00027166	1.24172	E15.5 * Wnt1 up vs E15.5 * Ctrl
10601412	Lpar4	NM_175271	0.000771424	1.24167	E15.5 * Wnt1 up vs E15.5 * Ctrl
10413795	Eaf1	NM_028932	3.6456e-005	1.24157	E15.5 * Wnt1 up vs E15.5 * Ctrl
10392251	Ddx5	NM_007840	6.83043e-006	1.24155	E15.5 * Wnt1 up vs E15.5 * Ctrl
10400894	Gpr135	NM_181752	0.000759324	1.24151	E15.5 * Wnt1 up vs E15.5 * Ctrl
10529636	Stk32b	NM_022416	0.0122753	1.24146	E15.5 * Wnt1 up vs E15.5 * Ctrl
10493335	5830417I10Rik	BC059914	0.0133157	1.24145	E15.5 * Wnt1 up vs E15.5 * Ctrl
10390381	Npepps	NM_008942	4.62191e-005	1.24135	E15.5 * Wnt1 up vs E15.5 * Ctrl
10428310	Azin1	NM_001102458	0.00304418	1.2412	E15.5 * Wnt1 up vs E15.5 * Ctrl
10417895	Ppp3cb	NM_008914	0.00022242	1.24119	E15.5 * Wnt1 up vs E15.5 * Ctrl
10581111	Nae1	NM_144931	0.000609719	1.24117	E15.5 * Wnt1 up vs E15.5 * Ctrl
10508392	Rnf19b	ENSMUST00000(	0.00926201	1.24109	E15.5 * Wnt1 up vs E15.5 * Ctrl
10569306	Krtap5-1	NM_015808	0.00244398	1.24087	E15.5 * Wnt1 up vs E15.5 * Ctrl
10597258	Tmie	NM_146260	0.0049815	1.24052	E15.5 * Wnt1 up vs E15.5 * Ctrl
10349793	Dstyk	NM_172516	0.00353481	1.24051	E15.5 * Wnt1 up vs E15.5 * Ctrl
10389882	3300001P08Rik	NM_026313	1.00677e-006	1.24032	E15.5 * Wnt1 up vs E15.5 * Ctrl
10569168	Slc25a22	NM_026646	0.00350885	1.24011	E15.5 * Wnt1 up vs E15.5 * Ctrl
10402615	Hsp90aa1	NM_010480	8.13883e-006	1.23978	E15.5 * Wnt1 up vs E15.5 * Ctrl
10584506	Olfr967	NM_001011826	0.00835891	1.23972	E15.5 * Wnt1 up vs E15.5 * Ctrl

10450212	Egfl8	NM_152922	0.00078576	1.23962	E15.5 * Wnt1 up vs E15.5 * Ctrl
10597427	Trim71	NM_001042503	0.0118	1.23936	E15.5 * Wnt1 up vs E15.5 * Ctrl
10424833	Maf1	NM_026859	0.0121499	1.23909	E15.5 * Wnt1 up vs E15.5 * Ctrl
10455542	Ap3s1	NM_009681	0.00749426	1.23908	E15.5 * Wnt1 up vs E15.5 * Ctrl
10365830		---	0.000988634	1.239	E15.5 * Wnt1 up vs E15.5 * Ctrl
10490016	Sall4	NM_175303	0.00591797	1.23897	E15.5 * Wnt1 up vs E15.5 * Ctrl
10577385	EG654453	NM_001039119	0.000303943	1.2389	E15.5 * Wnt1 up vs E15.5 * Ctrl
10358849	Dhx9	NM_007842	3.14453e-005	1.23864	E15.5 * Wnt1 up vs E15.5 * Ctrl
10388625	Efcab5	NM_176965	0.00362989	1.23863	E15.5 * Wnt1 up vs E15.5 * Ctrl
10495854	Prss12	NM_008939	0.00507634	1.23856	E15.5 * Wnt1 up vs E15.5 * Ctrl
10555089	Rsf1	NM_001081267	0.000939997	1.23851	E15.5 * Wnt1 up vs E15.5 * Ctrl
10399376	9930038B18Rik	ENSMUST00000(	0.00334787	1.2385	E15.5 * Wnt1 up vs E15.5 * Ctrl
10350039	Rabif	NM_145510	0.00314179	1.23806	E15.5 * Wnt1 up vs E15.5 * Ctrl
10472313	Tbr1	NM_009322	2.11009e-005	1.23777	E15.5 * Wnt1 up vs E15.5 * Ctrl
10523526	ENSMUSG00000	ENSMUST00000(	0.00343138	1.23764	E15.5 * Wnt1 up vs E15.5 * Ctrl
10407222	Dhx29	NM_172594	0.00523316	1.23746	E15.5 * Wnt1 up vs E15.5 * Ctrl
10505461	Atp6v1g1	NM_024173	0.000192824	1.23736	E15.5 * Wnt1 up vs E15.5 * Ctrl
10457038	ENSMUSG00000	ENSMUST00000(	0.00053941	1.23731	E15.5 * Wnt1 up vs E15.5 * Ctrl
10474477	OTTMUSG00000	XR_034254	0.0107462	1.23723	E15.5 * Wnt1 up vs E15.5 * Ctrl
10389326	Pigw	NM_027388	0.0102954	1.23719	E15.5 * Wnt1 up vs E15.5 * Ctrl
10480121	2310047O13Rik	NM_024185	0.000750485	1.23713	E15.5 * Wnt1 up vs E15.5 * Ctrl
10576657		---	0.0118337	1.23708	E15.5 * Wnt1 up vs E15.5 * Ctrl
10578149	Leprotl1	NM_026609	8.48612e-005	1.23673	E15.5 * Wnt1 up vs E15.5 * Ctrl
10498302	Gm410	NM_001033349	0.00598517	1.23667	E15.5 * Wnt1 up vs E15.5 * Ctrl
10546829	Oxtr	NM_001081147	0.0138394	1.23665	E15.5 * Wnt1 up vs E15.5 * Ctrl
10534075	Psph	NM_133900	0.00359774	1.2365	E15.5 * Wnt1 up vs E15.5 * Ctrl
10467650	Frat2	NM_177603	0.00764897	1.2365	E15.5 * Wnt1 up vs E15.5 * Ctrl
10504916		---	0.00672016	1.23643	E15.5 * Wnt1 up vs E15.5 * Ctrl
10580309	1500041N16Rik	NM_026399	0.00175516	1.23635	E15.5 * Wnt1 up vs E15.5 * Ctrl
10388352	Sgsm2	NM_197943	0.000789956	1.23633	E15.5 * Wnt1 up vs E15.5 * Ctrl
10432488	Bcdin3d	NM_029236	0.00544662	1.23619	E15.5 * Wnt1 up vs E15.5 * Ctrl
10544338	Prss3	NM_011645	0.00138795	1.23611	E15.5 * Wnt1 up vs E15.5 * Ctrl
10497086	1810013D15Rik	ENSMUST00000(	0.00989291	1.23602	E15.5 * Wnt1 up vs E15.5 * Ctrl
10494655	Zfp697	NM_172863	0.00163176	1.23575	E15.5 * Wnt1 up vs E15.5 * Ctrl
10439917	Zpld1	NM_178720	0.00286653	1.23568	E15.5 * Wnt1 up vs E15.5 * Ctrl
10497399	Pde7a	NM_001122759	0.000908492	1.23565	E15.5 * Wnt1 up vs E15.5 * Ctrl
10362149	Taar2	NM_001007266	3.30193e-005	1.23558	E15.5 * Wnt1 up vs E15.5 * Ctrl
10460202	Suv420h1	NM_144871	2.01786e-005	1.23543	E15.5 * Wnt1 up vs E15.5 * Ctrl
10545208	Gm189	ENSMUST00000	0.00226005	1.23536	E15.5 * Wnt1 up vs E15.5 * Ctrl
10524588	Ankrd13a	NM_026718	0.0134542	1.2353	E15.5 * Wnt1 up vs E15.5 * Ctrl
10589870		---	0.013383	1.23526	E15.5 * Wnt1 up vs E15.5 * Ctrl
10502135	Rrh	NM_009102	0.00216027	1.23525	E15.5 * Wnt1 up vs E15.5 * Ctrl
10540191	Nr2c2	NM_011630	8.30179e-005	1.23518	E15.5 * Wnt1 up vs E15.5 * Ctrl
10599962	Mamld1	NM_001081354	0.00463541	1.23513	E15.5 * Wnt1 up vs E15.5 * Ctrl
10485828	Slc12a6	NM_133648	0.00564056	1.23465	E15.5 * Wnt1 up vs E15.5 * Ctrl
10348682	Capn10	NM_011796	0.00373029	1.23457	E15.5 * Wnt1 up vs E15.5 * Ctrl
10378065	Med31	NM_026068	0.0139703	1.23448	E15.5 * Wnt1 up vs E15.5 * Ctrl
10572332	Sfrs14	NM_172755	0.000102004	1.23447	E15.5 * Wnt1 up vs E15.5 * Ctrl
10473217		---	0.00113947	1.2344	E15.5 * Wnt1 up vs E15.5 * Ctrl
10503695	Bach2	NM_007521	0.00733287	1.23437	E15.5 * Wnt1 up vs E15.5 * Ctrl
10486722	Trp53bp1	NM_013735	0.00093044	1.23408	E15.5 * Wnt1 up vs E15.5 * Ctrl
10460468	Ctsf	NM_019861	0.014336	1.23363	E15.5 * Wnt1 up vs E15.5 * Ctrl
10496338	Ppp3ca	NM_008913	0.000690972	1.23356	E15.5 * Wnt1 up vs E15.5 * Ctrl
10604230	Ap3s1	NM_009681	0.00919538	1.23351	E15.5 * Wnt1 up vs E15.5 * Ctrl
10477673	Myh7b	NM_001085378	0.00239148	1.23329	E15.5 * Wnt1 up vs E15.5 * Ctrl
10367708	Pcmt1	NM_008786	0.000411625	1.2331	E15.5 * Wnt1 up vs E15.5 * Ctrl
10424746	Zfp623	NM_030199	0.0075536	1.23301	E15.5 * Wnt1 up vs E15.5 * Ctrl
10422912	2410089E03Rik	BC058107	1.44632e-006	1.2328	E15.5 * Wnt1 up vs E15.5 * Ctrl
10407543	Gtpbp4	NM_027000	0.000215582	1.23277	E15.5 * Wnt1 up vs E15.5 * Ctrl
10433717		---	0.00281544	1.23232	E15.5 * Wnt1 up vs E15.5 * Ctrl
10424660	4933427E11Rik	ENSMUST00000(	0.00655611	1.23229	E15.5 * Wnt1 up vs E15.5 * Ctrl
10471294	Ppapdc3	NM_145521	0.011029	1.23196	E15.5 * Wnt1 up vs E15.5 * Ctrl
10354404	Dnajb6	NM_001037940	0.00216271	1.23168	E15.5 * Wnt1 up vs E15.5 * Ctrl
10361748	Fbxo30	NM_027968	0.0062189	1.23165	E15.5 * Wnt1 up vs E15.5 * Ctrl
10403756	1600012F09Rik	NM_025904	0.00291128	1.23161	E15.5 * Wnt1 up vs E15.5 * Ctrl
10407916	1600012F09Rik	NM_025904	0.00291128	1.23161	E15.5 * Wnt1 up vs E15.5 * Ctrl

10586458	Csnk1g1	NM_173185	0.00020748	1.23138	E15.5 * Wnt1 up vs E15.5 * Ctrl
10498515	Vmn2r-ps11	NR_003962	0.013454	1.23134	E15.5 * Wnt1 up vs E15.5 * Ctrl
10452854	Srd5a2	NM_053188	0.00530051	1.2313	E15.5 * Wnt1 up vs E15.5 * Ctrl
10482109	Rbm18	NR_027515	0.00234619	1.23085	E15.5 * Wnt1 up vs E15.5 * Ctrl
10453575	Cul2	NM_029402	0.00681296	1.2308	E15.5 * Wnt1 up vs E15.5 * Ctrl
10469239		---	0.0106197	1.23074	E15.5 * Wnt1 up vs E15.5 * Ctrl
10586357	Cilp	NM_173385	0.00620019	1.23053	E15.5 * Wnt1 up vs E15.5 * Ctrl
10551770	Zfp30	NM_013705	0.00503396	1.2305	E15.5 * Wnt1 up vs E15.5 * Ctrl
10568260	Zfp629	NM_177226	0.00174575	1.23048	E15.5 * Wnt1 up vs E15.5 * Ctrl
10442458	Gbl	NM_019988	0.00134841	1.2304	E15.5 * Wnt1 up vs E15.5 * Ctrl
10466735	Gm967	NM_001114174	0.00897508	1.23034	E15.5 * Wnt1 up vs E15.5 * Ctrl
10485681	Fshb	NM_008045	0.00309572	1.23016	E15.5 * Wnt1 up vs E15.5 * Ctrl
10537742	Clcn1	NM_013491	0.00459498	1.23004	E15.5 * Wnt1 up vs E15.5 * Ctrl
10424102	EG665097	ENSMUST00000	0.0146578	1.23002	E15.5 * Wnt1 up vs E15.5 * Ctrl
10488678	Dusp15	NM_001159376	0.00557591	1.23002	E15.5 * Wnt1 up vs E15.5 * Ctrl
10428068	Tspyl5	NM_001085421	0.0103202	1.22989	E15.5 * Wnt1 up vs E15.5 * Ctrl
10541214	B4galnt3	NM_198884	0.0128576	1.22982	E15.5 * Wnt1 up vs E15.5 * Ctrl
10491331	Pik3ca	NM_008839	0.00127928	1.22975	E15.5 * Wnt1 up vs E15.5 * Ctrl
10579144	9130404D08Rik	NM_028993	0.00248022	1.22953	E15.5 * Wnt1 up vs E15.5 * Ctrl
10596880	Bsn	NM_007567	8.67274e-005	1.22951	E15.5 * Wnt1 up vs E15.5 * Ctrl
10550022	Psip1	NM_133948	0.000148154	1.22937	E15.5 * Wnt1 up vs E15.5 * Ctrl
10545881	Cml2	NM_053096	0.00732694	1.22918	E15.5 * Wnt1 up vs E15.5 * Ctrl
10589511	Scap	NM_001001144	0.00608484	1.22897	E15.5 * Wnt1 up vs E15.5 * Ctrl
10562000	Psenen	NM_025498	0.00106916	1.22871	E15.5 * Wnt1 up vs E15.5 * Ctrl
10468037		---	0.000596086	1.22867	E15.5 * Wnt1 up vs E15.5 * Ctrl
10589407	Spink8	NM_183136	0.00838542	1.22865	E15.5 * Wnt1 up vs E15.5 * Ctrl
10577866	Ash2l	NM_011791	3.00635e-007	1.22853	E15.5 * Wnt1 up vs E15.5 * Ctrl
10504148	4933409K07Rik	BC059060	0.00879946	1.22853	E15.5 * Wnt1 up vs E15.5 * Ctrl
10430920	Tcf20	NM_001114140	0.000682862	1.22852	E15.5 * Wnt1 up vs E15.5 * Ctrl
10561831	Zfp566	NM_152814	0.00445782	1.22843	E15.5 * Wnt1 up vs E15.5 * Ctrl
10369783	Zfp365	NM_178679	0.00674694	1.22829	E15.5 * Wnt1 up vs E15.5 * Ctrl
10512125	Psenen	NM_025498	0.00112143	1.22806	E15.5 * Wnt1 up vs E15.5 * Ctrl
10463632	Tmem180	NM_029186	0.0019882	1.22804	E15.5 * Wnt1 up vs E15.5 * Ctrl
10455294	Kctd16	ENSMUST00000	0.0147484	1.22801	E15.5 * Wnt1 up vs E15.5 * Ctrl
10539606	Cct7	NM_007638	0.000676889	1.22798	E15.5 * Wnt1 up vs E15.5 * Ctrl
10440731	Krtap11-1	NM_001113406	0.00409776	1.2278	E15.5 * Wnt1 up vs E15.5 * Ctrl
10559590	Ptprh	NM_207270	0.00805066	1.22767	E15.5 * Wnt1 up vs E15.5 * Ctrl
10545379	Usp39	NM_138592	0.00100964	1.22761	E15.5 * Wnt1 up vs E15.5 * Ctrl
10380087	Mtmr4	NM_133215	6.10332e-005	1.2272	E15.5 * Wnt1 up vs E15.5 * Ctrl
10497090	Ankrd13c	NM_001013806	0.00147726	1.22719	E15.5 * Wnt1 up vs E15.5 * Ctrl
10401172	Vti1b	NM_016800	0.000163311	1.22715	E15.5 * Wnt1 up vs E15.5 * Ctrl
10385343	Ttc1	NM_133795	0.000587826	1.22714	E15.5 * Wnt1 up vs E15.5 * Ctrl
10431266	Cerk	NM_145475	0.00212781	1.22711	E15.5 * Wnt1 up vs E15.5 * Ctrl
10375553	Olfr1389	NM_147066	0.00277397	1.22693	E15.5 * Wnt1 up vs E15.5 * Ctrl
10578539	Slc25a4	NM_007450	1.39903e-005	1.22663	E15.5 * Wnt1 up vs E15.5 * Ctrl
10570278		---	0.0101887	1.22658	E15.5 * Wnt1 up vs E15.5 * Ctrl
10578262		---	4.15948e-005	1.22657	E15.5 * Wnt1 up vs E15.5 * Ctrl
10403455	Dip2c	NM_001081426	0.00720486	1.22624	E15.5 * Wnt1 up vs E15.5 * Ctrl
10350594	Ivns1abp	NM_054102	3.79839e-005	1.22614	E15.5 * Wnt1 up vs E15.5 * Ctrl
10522873	EG654494	NR_003652	0.0108682	1.22605	E15.5 * Wnt1 up vs E15.5 * Ctrl
10449873	Wiz	NM_212438	0.000499097	1.22604	E15.5 * Wnt1 up vs E15.5 * Ctrl
10497167		---	0.0117029	1.22603	E15.5 * Wnt1 up vs E15.5 * Ctrl
10531796	Wdfy3	NM_172882	0.00564932	1.22599	E15.5 * Wnt1 up vs E15.5 * Ctrl
10445407	Tcte1	NM_013688	0.00349182	1.22594	E15.5 * Wnt1 up vs E15.5 * Ctrl
10571198	Gm1698	ENSMUST00000	0.00517459	1.22593	E15.5 * Wnt1 up vs E15.5 * Ctrl
10364222	Ftcd	NM_080845	0.00964925	1.22584	E15.5 * Wnt1 up vs E15.5 * Ctrl
10388684	Taok1	NM_144825	0.00476572	1.2258	E15.5 * Wnt1 up vs E15.5 * Ctrl
10543173	Vwde	NM_001013757	0.00448734	1.22572	E15.5 * Wnt1 up vs E15.5 * Ctrl
10552689	Fam71e1	NM_028169	0.00559025	1.2257	E15.5 * Wnt1 up vs E15.5 * Ctrl
10409645	Ubqln1	NM_152234	6.35667e-005	1.22553	E15.5 * Wnt1 up vs E15.5 * Ctrl
10550605	Eml2	BC055476	0.00205837	1.22543	E15.5 * Wnt1 up vs E15.5 * Ctrl
10449631	Btbd9	NM_027060	0.00662328	1.22539	E15.5 * Wnt1 up vs E15.5 * Ctrl
10489368	Fitm2	NM_173397	0.00473197	1.22538	E15.5 * Wnt1 up vs E15.5 * Ctrl
10478698	Eya2	NM_010165	3.28766e-005	1.22536	E15.5 * Wnt1 up vs E15.5 * Ctrl
10578973	Npy5r	NM_016708	0.00478244	1.22536	E15.5 * Wnt1 up vs E15.5 * Ctrl
10472047	Tas2r134	NM_199158	0.014715	1.22533	E15.5 * Wnt1 up vs E15.5 * Ctrl

10470322	Surf2	NM_013678	0.00132035	1.22532	E15.5 * Wnt1 up vs E15.5 * Ctrl
10419354	Map1lc3b	NM_026160	0.00773572	1.22486	E15.5 * Wnt1 up vs E15.5 * Ctrl
10458709	Gm94	NM_001033280	0.0111681	1.22472	E15.5 * Wnt1 up vs E15.5 * Ctrl
10441740	Agpat4	NM_026644	0.000929535	1.22467	E15.5 * Wnt1 up vs E15.5 * Ctrl
10518494	Ptchd2	NM_001083342	0.00175207	1.22441	E15.5 * Wnt1 up vs E15.5 * Ctrl
10363915	LOC432471	AK138135	0.00938854	1.22436	E15.5 * Wnt1 up vs E15.5 * Ctrl
10355278	Erbb4	NM_010154	0.0131526	1.22425	E15.5 * Wnt1 up vs E15.5 * Ctrl
10394926		---	0.003245	1.22415	E15.5 * Wnt1 up vs E15.5 * Ctrl
10492890	Lrba	NM_030695	0.00330694	1.22414	E15.5 * Wnt1 up vs E15.5 * Ctrl
10555925	Cnga4	NM_001033317	0.0128082	1.22398	E15.5 * Wnt1 up vs E15.5 * Ctrl
10487382	Fahd2a	NM_029629	0.0008215	1.22387	E15.5 * Wnt1 up vs E15.5 * Ctrl
10345141	Lmbrd1	NM_026719	0.00186165	1.22381	E15.5 * Wnt1 up vs E15.5 * Ctrl
10405842	4932441B19Rik	NM_001083890	0.00554499	1.22342	E15.5 * Wnt1 up vs E15.5 * Ctrl
10381115	Eif1	NM_011508	0.000305739	1.22326	E15.5 * Wnt1 up vs E15.5 * Ctrl
10504178	4933409K07Rik	BC059060	0.00549612	1.22323	E15.5 * Wnt1 up vs E15.5 * Ctrl
10409053	Zfp169	NM_026450	0.00629089	1.22323	E15.5 * Wnt1 up vs E15.5 * Ctrl
10515983	Dem1	NM_028457	0.00040598	1.22304	E15.5 * Wnt1 up vs E15.5 * Ctrl
10586347	Parp16	NM_177460	0.0139495	1.22291	E15.5 * Wnt1 up vs E15.5 * Ctrl
10494005	ENSMUSG0000C	NM_001146107	0.00944327	1.22277	E15.5 * Wnt1 up vs E15.5 * Ctrl
10499969	ENSMUSG0000C	NM_001146107	0.00944327	1.22277	E15.5 * Wnt1 up vs E15.5 * Ctrl
10499979	ENSMUSG0000C	NM_001146107	0.00944327	1.22277	E15.5 * Wnt1 up vs E15.5 * Ctrl
10418275	Ccdc66	NM_177111	0.0112549	1.22274	E15.5 * Wnt1 up vs E15.5 * Ctrl
10362886	Bend3	NM_199028	0.0108894	1.22273	E15.5 * Wnt1 up vs E15.5 * Ctrl
10393404		---	0.0126286	1.22257	E15.5 * Wnt1 up vs E15.5 * Ctrl
10578399		---	0.00948211	1.22256	E15.5 * Wnt1 up vs E15.5 * Ctrl
10472942	ENSMUSG0000C	ENSMUST00000	0.0024065	1.22247	E15.5 * Wnt1 up vs E15.5 * Ctrl
10569719	A430078G23Rik	NM_001033378	0.00718006	1.2224	E15.5 * Wnt1 up vs E15.5 * Ctrl
10463515	Hps6	NM_176785	0.00621119	1.22223	E15.5 * Wnt1 up vs E15.5 * Ctrl
10370025	Smarcb1	NM_011418	0.0019564	1.2221	E15.5 * Wnt1 up vs E15.5 * Ctrl
10600947	Tmem28	NM_001081283	0.00138441	1.222	E15.5 * Wnt1 up vs E15.5 * Ctrl
10352396	Trp53bp2	NM_173378	0.000669971	1.22191	E15.5 * Wnt1 up vs E15.5 * Ctrl
10587780	Tuba1b	NM_011654	0.000139373	1.22187	E15.5 * Wnt1 up vs E15.5 * Ctrl
10539653	Tprkb	NM_176842	0.0147317	1.22185	E15.5 * Wnt1 up vs E15.5 * Ctrl
10457382	Gjd4	NM_153086	0.00754277	1.22161	E15.5 * Wnt1 up vs E15.5 * Ctrl
10498002	1700018B24Rik	NR_003617	0.00541123	1.22157	E15.5 * Wnt1 up vs E15.5 * Ctrl
10415714	Fam123a	NM_028113	0.0098967	1.22135	E15.5 * Wnt1 up vs E15.5 * Ctrl
10397460	EG627607	ENSMUST00000	0.00136585	1.22127	E15.5 * Wnt1 up vs E15.5 * Ctrl
10361869	Nhsl1	NM_173390	0.00102144	1.2212	E15.5 * Wnt1 up vs E15.5 * Ctrl
10474698		---	0.00091704	1.22095	E15.5 * Wnt1 up vs E15.5 * Ctrl
10566591	Olfr695	NM_146598	0.0130449	1.22085	E15.5 * Wnt1 up vs E15.5 * Ctrl
10490826	Zbtb10	ENSMUST00000	0.00769769	1.22084	E15.5 * Wnt1 up vs E15.5 * Ctrl
10510305	Frap1	NM_020009	0.00605573	1.22072	E15.5 * Wnt1 up vs E15.5 * Ctrl
10348032	Armc9	NM_030184	0.0107015	1.22067	E15.5 * Wnt1 up vs E15.5 * Ctrl
10593867	Lingo1	NM_181074	0.00862328	1.22065	E15.5 * Wnt1 up vs E15.5 * Ctrl
10413502	Actr8	NM_027493	0.00677983	1.22061	E15.5 * Wnt1 up vs E15.5 * Ctrl
10591228	Zfp26	NM_011753	3.74415e-005	1.22053	E15.5 * Wnt1 up vs E15.5 * Ctrl
10578872		---	0.00627732	1.22047	E15.5 * Wnt1 up vs E15.5 * Ctrl
10428827	Tmem65	NM_175212	0.00605242	1.22044	E15.5 * Wnt1 up vs E15.5 * Ctrl
10578757	Fbxo8	NM_015791	0.00275048	1.22038	E15.5 * Wnt1 up vs E15.5 * Ctrl
10420320	Cenpj	NM_001014996	0.0146162	1.22018	E15.5 * Wnt1 up vs E15.5 * Ctrl
10572514	A230052G05Rik	ENSMUST00000	0.0100678	1.21998	E15.5 * Wnt1 up vs E15.5 * Ctrl
10554463	Hddc3	NM_026812	0.00451698	1.21998	E15.5 * Wnt1 up vs E15.5 * Ctrl
10493612	4933434E20Rik	NM_025762	0.0104906	1.21972	E15.5 * Wnt1 up vs E15.5 * Ctrl
10579089	D330038O06Rik	NM_177899	0.00482428	1.21968	E15.5 * Wnt1 up vs E15.5 * Ctrl
10598422	Gripap1	NM_207670	0.00484395	1.21965	E15.5 * Wnt1 up vs E15.5 * Ctrl
10555260	Olfr521	NM_146356	0.00988254	1.21958	E15.5 * Wnt1 up vs E15.5 * Ctrl
10436493		---	0.00835114	1.21957	E15.5 * Wnt1 up vs E15.5 * Ctrl
10573549	Fbxw9	NM_026791	0.00569537	1.21927	E15.5 * Wnt1 up vs E15.5 * Ctrl
10400326	Eapp	NM_025456	0.00572812	1.21925	E15.5 * Wnt1 up vs E15.5 * Ctrl
10358537	Hmcn1	NM_001024720	0.0086511	1.21923	E15.5 * Wnt1 up vs E15.5 * Ctrl
10473517		---	0.00790856	1.2192	E15.5 * Wnt1 up vs E15.5 * Ctrl
10545425	Sh2d6	BC100525	0.00577408	1.21885	E15.5 * Wnt1 up vs E15.5 * Ctrl
10516466	Zmym1	NM_026670	0.000301566	1.21867	E15.5 * Wnt1 up vs E15.5 * Ctrl
10537494	Ssbp1	NM_028358	0.0101302	1.2186	E15.5 * Wnt1 up vs E15.5 * Ctrl
10569539	Mrgprg	NM_203492	0.00150915	1.21853	E15.5 * Wnt1 up vs E15.5 * Ctrl
10558134	Plekha1	NM_133942	0.00536131	1.21851	E15.5 * Wnt1 up vs E15.5 * Ctrl

10393628	Tbc1d16	NM_172443	0.00790124	1.21847	E15.5 * Wnt1 up vs E15.5 * Ctrl
10348666	Dusp28	NM_175118	0.00553377	1.21845	E15.5 * Wnt1 up vs E15.5 * Ctrl
10384936	Acyp2	NM_029344	0.00543197	1.21837	E15.5 * Wnt1 up vs E15.5 * Ctrl
10389283	Lhx1	NM_008498	0.00754491	1.2183	E15.5 * Wnt1 up vs E15.5 * Ctrl
10496789	Lpar3	NM_022983	0.00584688	1.21818	E15.5 * Wnt1 up vs E15.5 * Ctrl
10494662	Ywhah	NM_011738	0.000250694	1.21781	E15.5 * Wnt1 up vs E15.5 * Ctrl
10435149	Fyttd1	NM_027226	6.27357e-006	1.21761	E15.5 * Wnt1 up vs E15.5 * Ctrl
10600502	2810453I06Rik	NM_133703	0.000336545	1.21739	E15.5 * Wnt1 up vs E15.5 * Ctrl
10560791	EG381936	NM_001037248	0.0102409	1.21733	E15.5 * Wnt1 up vs E15.5 * Ctrl
10432431	Fam186b	NM_001081254	0.00693919	1.21721	E15.5 * Wnt1 up vs E15.5 * Ctrl
10409526	BC021381	NM_145382	0.000838293	1.21689	E15.5 * Wnt1 up vs E15.5 * Ctrl
10451597	Prickle4	ENSMUST00000	0.0137598	1.21687	E15.5 * Wnt1 up vs E15.5 * Ctrl
10459241	Afap1l1	NM_178928	0.0123133	1.21682	E15.5 * Wnt1 up vs E15.5 * Ctrl
10347970	Cab39	NM_133781	0.000633836	1.21669	E15.5 * Wnt1 up vs E15.5 * Ctrl
10560851	1700008P20Rik	NR_003638	0.00930672	1.21659	E15.5 * Wnt1 up vs E15.5 * Ctrl
10606186	Slc16a2	NM_009197	0.0038545	1.21639	E15.5 * Wnt1 up vs E15.5 * Ctrl
10560491	Klc3	NM_146182	0.0139627	1.21616	E15.5 * Wnt1 up vs E15.5 * Ctrl
10476353		---	0.0142616	1.21603	E15.5 * Wnt1 up vs E15.5 * Ctrl
10424894	Heatr7a	ENSMUST00000	0.000703474	1.21578	E15.5 * Wnt1 up vs E15.5 * Ctrl
10575840		---	0.00771301	1.21557	E15.5 * Wnt1 up vs E15.5 * Ctrl
10444521	Zbtb12	NM_198886	0.0114018	1.21551	E15.5 * Wnt1 up vs E15.5 * Ctrl
10512947		---	0.00659612	1.21545	E15.5 * Wnt1 up vs E15.5 * Ctrl
10408444	Prl7c1	NM_026206	0.0130719	1.21544	E15.5 * Wnt1 up vs E15.5 * Ctrl
10410380		---	0.0115023	1.21538	E15.5 * Wnt1 up vs E15.5 * Ctrl
10606064	Rgag4	NM_183318	0.0062041	1.21525	E15.5 * Wnt1 up vs E15.5 * Ctrl
10566172	Olfr78	NM_130866	0.000351265	1.21497	E15.5 * Wnt1 up vs E15.5 * Ctrl
10378739	Ywhae	NM_009536	1.64259e-007	1.21496	E15.5 * Wnt1 up vs E15.5 * Ctrl
10346533	Nif3l1	NM_022988	0.00641874	1.21471	E15.5 * Wnt1 up vs E15.5 * Ctrl
10442155	Ppp2r1a	NM_016891	0.00598484	1.21468	E15.5 * Wnt1 up vs E15.5 * Ctrl
10447860	Pnldc1	NM_001034866	0.0145457	1.21453	E15.5 * Wnt1 up vs E15.5 * Ctrl
10387251	2310047M10Rik	NM_028005	0.0133951	1.21447	E15.5 * Wnt1 up vs E15.5 * Ctrl
10573048	Anapc10	NM_026904	0.00600628	1.21432	E15.5 * Wnt1 up vs E15.5 * Ctrl
10488421	OTTMUSG00000	XM_001481087	0.00461205	1.21432	E15.5 * Wnt1 up vs E15.5 * Ctrl
10474851	Bahd1	NM_001045523	0.0122884	1.21431	E15.5 * Wnt1 up vs E15.5 * Ctrl
10520459	Fam59b	BC113782	0.00567767	1.21422	E15.5 * Wnt1 up vs E15.5 * Ctrl
10385484	Ddx5	NM_007840	6.00993e-006	1.21401	E15.5 * Wnt1 up vs E15.5 * Ctrl
10470050	Abca2	NM_007379	0.00345372	1.21393	E15.5 * Wnt1 up vs E15.5 * Ctrl
10608573	Srsy	EU052291	0.00172364	1.21387	E15.5 * Wnt1 up vs E15.5 * Ctrl
10608576		---	0.00172364	1.21387	E15.5 * Wnt1 up vs E15.5 * Ctrl
10437149		---	0.014416	1.21385	E15.5 * Wnt1 up vs E15.5 * Ctrl
10416082	Trim35	NM_029979	0.00158875	1.21381	E15.5 * Wnt1 up vs E15.5 * Ctrl
10434252	Hira	NM_010435	0.0066564	1.21377	E15.5 * Wnt1 up vs E15.5 * Ctrl
10376568	Rai1	NM_009021	0.0143062	1.21374	E15.5 * Wnt1 up vs E15.5 * Ctrl
10607259	Tro	NM_001002272	2.3206e-005	1.21357	E15.5 * Wnt1 up vs E15.5 * Ctrl
10592455	Olfr975	NM_146828	0.00100444	1.21338	E15.5 * Wnt1 up vs E15.5 * Ctrl
10533812	Sbno1	NM_001081203	2.12758e-005	1.21333	E15.5 * Wnt1 up vs E15.5 * Ctrl
10573152	Ucp1	NM_009463	0.00331711	1.21319	E15.5 * Wnt1 up vs E15.5 * Ctrl
10556149	Olfr503	NM_001011527	0.00985026	1.21315	E15.5 * Wnt1 up vs E15.5 * Ctrl
10348866	Atg4b	NM_174874	0.00450785	1.21311	E15.5 * Wnt1 up vs E15.5 * Ctrl
10489723	Zmynd8	NM_027230	0.00704797	1.21305	E15.5 * Wnt1 up vs E15.5 * Ctrl
10424842	EG626280	XM_910693	0.00215856	1.21301	E15.5 * Wnt1 up vs E15.5 * Ctrl
10416325	Nudt18	NM_153136	0.00296533	1.21298	E15.5 * Wnt1 up vs E15.5 * Ctrl
10600512	Brcc3	NM_145956	0.000595837	1.21293	E15.5 * Wnt1 up vs E15.5 * Ctrl
10476312	4930425F17Rik	ENSMUST00000	0.000436197	1.21283	E15.5 * Wnt1 up vs E15.5 * Ctrl
10386157		---	0.00924235	1.21269	E15.5 * Wnt1 up vs E15.5 * Ctrl
10508351	Phc2	NM_018774	0.00419565	1.21267	E15.5 * Wnt1 up vs E15.5 * Ctrl
10542857	Far2	NM_178797	0.00454427	1.21242	E15.5 * Wnt1 up vs E15.5 * Ctrl
10579508	Ano8	AK173222	0.0109405	1.2124	E15.5 * Wnt1 up vs E15.5 * Ctrl
10442870	Ccdc78	ENSMUST00000	0.00509565	1.21215	E15.5 * Wnt1 up vs E15.5 * Ctrl
10356999	Prdx2	NM_011563	0.00523012	1.2121	E15.5 * Wnt1 up vs E15.5 * Ctrl
10456158	ENSMUSG00000	ENSMUST00000	0.0143504	1.21204	E15.5 * Wnt1 up vs E15.5 * Ctrl
10503401	Tmem55a	NM_028264	0.000414617	1.21184	E15.5 * Wnt1 up vs E15.5 * Ctrl
10349932	Etnk2	NM_175443	0.00208457	1.21171	E15.5 * Wnt1 up vs E15.5 * Ctrl
10588582	6430571L13Rik	NM_175486	0.00756647	1.21169	E15.5 * Wnt1 up vs E15.5 * Ctrl
10534148	A330070K13Rik	ENSMUST00000	0.00918187	1.21166	E15.5 * Wnt1 up vs E15.5 * Ctrl
10462861	LOC240657	XR_034565	0.00137917	1.21163	E15.5 * Wnt1 up vs E15.5 * Ctrl

10471701	ENSMUSG00000	ENSMUST00000	0.00248398	1.21153	E15.5 * Wnt1 up vs E15.5 * Ctrl
10586880	Suhw4	NM_146224	0.0129055	1.2113	E15.5 * Wnt1 up vs E15.5 * Ctrl
10519495	4921511H03Rik	ENSMUST00000	0.0132113	1.21123	E15.5 * Wnt1 up vs E15.5 * Ctrl
10536949	Fam40b	NM_177204	0.00987486	1.21114	E15.5 * Wnt1 up vs E15.5 * Ctrl
10552288	Vstm2b	NM_021387	0.0121509	1.21107	E15.5 * Wnt1 up vs E15.5 * Ctrl
10554142	2310037I24Rik	BC083325	0.00138801	1.21106	E15.5 * Wnt1 up vs E15.5 * Ctrl
10482229	Psmb7	NM_011187	0.000267236	1.2108	E15.5 * Wnt1 up vs E15.5 * Ctrl
10582669	Ttc13	NM_145607	0.00211889	1.21079	E15.5 * Wnt1 up vs E15.5 * Ctrl
10443730	Abcg1	NM_009593	0.00591572	1.21066	E15.5 * Wnt1 up vs E15.5 * Ctrl
10496036	Col25a1	NM_029838	0.00971327	1.21056	E15.5 * Wnt1 up vs E15.5 * Ctrl
10448055	Pdcd2	NM_008799	0.0121107	1.21053	E15.5 * Wnt1 up vs E15.5 * Ctrl
10464034	Tectb	NM_009348	0.0018979	1.21042	E15.5 * Wnt1 up vs E15.5 * Ctrl
10457508	Npc1	NM_008720	0.000657937	1.21041	E15.5 * Wnt1 up vs E15.5 * Ctrl
10603738	4930403L05Rik	NM_026128	0.00489363	1.21038	E15.5 * Wnt1 up vs E15.5 * Ctrl
10381744	Arf2	NM_007477	0.0124125	1.21032	E15.5 * Wnt1 up vs E15.5 * Ctrl
10474243	Cstf3	NM_145529	0.00574509	1.21016	E15.5 * Wnt1 up vs E15.5 * Ctrl
10416666	Sugt1	NM_026474	0.00577148	1.21015	E15.5 * Wnt1 up vs E15.5 * Ctrl
10441933	2210404J11Rik	NM_001039552	0.00454199	1.21001	E15.5 * Wnt1 up vs E15.5 * Ctrl
10403929	OTTMUSG00000	NM_001039115	0.00601452	1.21001	E15.5 * Wnt1 up vs E15.5 * Ctrl
10365408	Ric8b	NM_183172	0.00196773	1.20993	E15.5 * Wnt1 up vs E15.5 * Ctrl
10413695	Pbrm1	NM_001081251	0.000453422	1.2099	E15.5 * Wnt1 up vs E15.5 * Ctrl
10378114	Ube2g1	NM_025985	0.00059753	1.20977	E15.5 * Wnt1 up vs E15.5 * Ctrl
10468691	Ablim1	NM_178688	0.00842116	1.20973	E15.5 * Wnt1 up vs E15.5 * Ctrl
10379445	Zfp207	NM_001130169	2.27723e-006	1.20967	E15.5 * Wnt1 up vs E15.5 * Ctrl
10584433	Olfr914	NM_146786	0.00329818	1.20941	E15.5 * Wnt1 up vs E15.5 * Ctrl
10380524	Slc35b1	NM_016752	0.000392854	1.20933	E15.5 * Wnt1 up vs E15.5 * Ctrl
10602454	Fam120c	NM_198105	0.00163927	1.20926	E15.5 * Wnt1 up vs E15.5 * Ctrl
10593473	1810046K07Rik	BC120857	0.00680822	1.20924	E15.5 * Wnt1 up vs E15.5 * Ctrl
10563911		---	0.00271783	1.20895	E15.5 * Wnt1 up vs E15.5 * Ctrl
10372656	Cpsf6	NM_001013391	0.00386877	1.20847	E15.5 * Wnt1 up vs E15.5 * Ctrl
10563314	Dhdh	NM_027903	0.0141281	1.2083	E15.5 * Wnt1 up vs E15.5 * Ctrl
10521693		---	0.00328554	1.20829	E15.5 * Wnt1 up vs E15.5 * Ctrl
10561861	Ovol3	ENSMUST00000	0.0106899	1.20819	E15.5 * Wnt1 up vs E15.5 * Ctrl
10601673	Cstf2	NM_133196	0.0062625	1.20805	E15.5 * Wnt1 up vs E15.5 * Ctrl
10608222	LOC621831	XR_031432	0.0109486	1.20797	E15.5 * Wnt1 up vs E15.5 * Ctrl
10608321		---	0.0109486	1.20797	E15.5 * Wnt1 up vs E15.5 * Ctrl
10608448	LOC621831	XR_031432	0.0109486	1.20797	E15.5 * Wnt1 up vs E15.5 * Ctrl
10402757	A730018C14Rik	BC172018	0.00756805	1.20766	E15.5 * Wnt1 up vs E15.5 * Ctrl
10403396	Adarb2	NM_052977	0.00622894	1.20756	E15.5 * Wnt1 up vs E15.5 * Ctrl
10570610	Defb4	NM_019728	0.00898215	1.2075	E15.5 * Wnt1 up vs E15.5 * Ctrl
10424686	BC025446	BC025446	0.0137681	1.20734	E15.5 * Wnt1 up vs E15.5 * Ctrl
10506893	Nrd1	NM_146150	7.86884e-007	1.20717	E15.5 * Wnt1 up vs E15.5 * Ctrl
10449547		---	0.00786154	1.20715	E15.5 * Wnt1 up vs E15.5 * Ctrl
10497587	1600012F09Rik	NM_025904	0.00291941	1.20681	E15.5 * Wnt1 up vs E15.5 * Ctrl
10532989	Gatc	NM_029645	0.00940895	1.20677	E15.5 * Wnt1 up vs E15.5 * Ctrl
10411593	Serf1	NM_011353	0.00458702	1.20675	E15.5 * Wnt1 up vs E15.5 * Ctrl
10402294	Moap1	NM_022323	0.00181655	1.20658	E15.5 * Wnt1 up vs E15.5 * Ctrl
10527332	Nptx2	NM_016789	0.0131709	1.2065	E15.5 * Wnt1 up vs E15.5 * Ctrl
10409065	Ptpdc1	NM_207232	0.00561053	1.20645	E15.5 * Wnt1 up vs E15.5 * Ctrl
10370259	Col18a1	NM_009929	0.00556597	1.20641	E15.5 * Wnt1 up vs E15.5 * Ctrl
10515698	BC059842	ENSMUST00000	0.0107419	1.20629	E15.5 * Wnt1 up vs E15.5 * Ctrl
10512391	Vcp	NM_009503	0.000318363	1.20627	E15.5 * Wnt1 up vs E15.5 * Ctrl
10441956	Fam120b	NM_024203	0.00190198	1.20599	E15.5 * Wnt1 up vs E15.5 * Ctrl
10445010	H2-M11	NM_177635	0.00335078	1.20594	E15.5 * Wnt1 up vs E15.5 * Ctrl
10405125	Fbxw17	NM_175401	0.00316584	1.20556	E15.5 * Wnt1 up vs E15.5 * Ctrl
10553813	Ube3a	NM_011668	0.00400514	1.20536	E15.5 * Wnt1 up vs E15.5 * Ctrl
10439934		---	0.00471898	1.20531	E15.5 * Wnt1 up vs E15.5 * Ctrl
10493585	Ube2q1	NM_027315	0.00177704	1.205	E15.5 * Wnt1 up vs E15.5 * Ctrl
10362036	Hbs1l	NM_019702	2.31452e-005	1.20496	E15.5 * Wnt1 up vs E15.5 * Ctrl
10429391	Slc45a4	NM_001033219	0.00892045	1.20496	E15.5 * Wnt1 up vs E15.5 * Ctrl
10442752	0610007P22Rik	NM_026676	0.000307164	1.20492	E15.5 * Wnt1 up vs E15.5 * Ctrl
10405504	Caml	NM_007596	0.0065639	1.20483	E15.5 * Wnt1 up vs E15.5 * Ctrl
10583444	Olfr39	NM_146825	0.00994945	1.20462	E15.5 * Wnt1 up vs E15.5 * Ctrl
10589640	1700112C13Rik	NM_183103	0.009026	1.20458	E15.5 * Wnt1 up vs E15.5 * Ctrl
10463068	Ptp4a1	NM_011200	0.0129543	1.20451	E15.5 * Wnt1 up vs E15.5 * Ctrl
10566387	Dub1	NM_007887	0.00111955	1.20417	E15.5 * Wnt1 up vs E15.5 * Ctrl

10568318	BC039632	NM_001081268	0.0133764	1.204	E15.5 * Wnt1 up vs E15.5 * Ctrl
10500412	Gpr89	NM_026229	0.00268846	1.20391	E15.5 * Wnt1 up vs E15.5 * Ctrl
10453609	Lyzl1	NM_026092	0.000103221	1.20385	E15.5 * Wnt1 up vs E15.5 * Ctrl
10434281	LOC635992	ENSMUST00000(	0.00705842	1.20384	E15.5 * Wnt1 up vs E15.5 * Ctrl
10490777	Zfhx4	NM_030708	0.00326992	1.20376	E15.5 * Wnt1 up vs E15.5 * Ctrl
10425914	Upk3a	NM_023478	0.0138826	1.20366	E15.5 * Wnt1 up vs E15.5 * Ctrl
10447729	Qk	U44941	0.00141162	1.20348	E15.5 * Wnt1 up vs E15.5 * Ctrl
10364435	Vmn2r81	ENSMUST00000(	0.0117468	1.20343	E15.5 * Wnt1 up vs E15.5 * Ctrl
10509868	Atp13a2	NM_029097	0.00393001	1.20342	E15.5 * Wnt1 up vs E15.5 * Ctrl
10491136	Tnik	BC137799	0.000862491	1.20333	E15.5 * Wnt1 up vs E15.5 * Ctrl
10382069	Psmc5	NM_008950	2.32343e-005	1.20324	E15.5 * Wnt1 up vs E15.5 * Ctrl
10546349	Xpc	NM_009531	0.000126206	1.20322	E15.5 * Wnt1 up vs E15.5 * Ctrl
10400581	Fkbp3	NM_013902	0.00022737	1.20315	E15.5 * Wnt1 up vs E15.5 * Ctrl
10417734	Nr1d2	NM_011584	0.00563696	1.20287	E15.5 * Wnt1 up vs E15.5 * Ctrl
10595327	Phip	NM_001081216	0.00146607	1.20267	E15.5 * Wnt1 up vs E15.5 * Ctrl
10367413	Dnajc14	NM_028873	0.0110266	1.20255	E15.5 * Wnt1 up vs E15.5 * Ctrl
10552284	Pin4	NM_027181	0.000591124	1.20247	E15.5 * Wnt1 up vs E15.5 * Ctrl
10532594	Myo18b	BC145218	0.00603484	1.20242	E15.5 * Wnt1 up vs E15.5 * Ctrl
10607398		---	0.00820708	1.20239	E15.5 * Wnt1 up vs E15.5 * Ctrl
10545308	Kdm3a	NM_001038695	0.0138537	1.20214	E15.5 * Wnt1 up vs E15.5 * Ctrl
10494924	Trim33	NM_053170	0.000741979	1.20209	E15.5 * Wnt1 up vs E15.5 * Ctrl
10478424	Ywhab	NM_018753	5.83815e-005	1.202	E15.5 * Wnt1 up vs E15.5 * Ctrl
10391742	Gpatch8	NM_001159492	0.0118089	1.2018	E15.5 * Wnt1 up vs E15.5 * Ctrl
10393898	Myadml2	NM_026751	0.0138272	1.20178	E15.5 * Wnt1 up vs E15.5 * Ctrl
10350478	Glrx2	NM_001038592	0.000271462	1.20163	E15.5 * Wnt1 up vs E15.5 * Ctrl
10572241	Pbx4	NM_001024954	0.00355489	1.20162	E15.5 * Wnt1 up vs E15.5 * Ctrl
10605788	Zxda	NR_003292	0.0047639	1.20156	E15.5 * Wnt1 up vs E15.5 * Ctrl
10347873	Agfg1	NM_010472	0.00176612	1.20145	E15.5 * Wnt1 up vs E15.5 * Ctrl
10352166	9630058J23Rik	BC054802	0.00694828	1.20136	E15.5 * Wnt1 up vs E15.5 * Ctrl
10548791	Grin2b	NM_008171	0.00807034	1.20127	E15.5 * Wnt1 up vs E15.5 * Ctrl
10365170	BC025920	ENSMUST00000	0.009804	1.201	E15.5 * Wnt1 up vs E15.5 * Ctrl
10497773	Mccc1	NM_023644	0.0102502	1.201	E15.5 * Wnt1 up vs E15.5 * Ctrl
10518855	Klhl21	NM_001033352	0.00102827	1.20094	E15.5 * Wnt1 up vs E15.5 * Ctrl
10422518	Tmtc4	NM_028651	0.0056854	1.20088	E15.5 * Wnt1 up vs E15.5 * Ctrl
10541803	Chd4	NM_145979	0.000610822	1.20086	E15.5 * Wnt1 up vs E15.5 * Ctrl
10588722	Mon1a	NM_028369	0.000671329	1.20085	E15.5 * Wnt1 up vs E15.5 * Ctrl
10586074		---	0.0127344	1.20075	E15.5 * Wnt1 up vs E15.5 * Ctrl
10476582	Macrod2	NM_028387	0.000835011	1.20051	E15.5 * Wnt1 up vs E15.5 * Ctrl
10465500	Kcnk4	NM_008431	0.0098584	1.2005	E15.5 * Wnt1 up vs E15.5 * Ctrl
10350377	Zbtb41	NM_172643	0.00189827	1.20048	E15.5 * Wnt1 up vs E15.5 * Ctrl
10454103		---	0.00294454	1.20029	E15.5 * Wnt1 up vs E15.5 * Ctrl
10455801	Phax	NM_019996	0.000682462	1.20022	E15.5 * Wnt1 up vs E15.5 * Ctrl
10431393	Mapk12	NM_013871	0.000567043	1.19967	E15.5 * Wnt1 up vs E15.5 * Ctrl
10420736	Neil2	NM_201610	0.00988423	1.19962	E15.5 * Wnt1 up vs E15.5 * Ctrl
10503917	Akirin2	NM_001007589	3.78087e-005	1.19954	E15.5 * Wnt1 up vs E15.5 * Ctrl
10419810	Psmb5	NM_011186	0.0043892	1.19913	E15.5 * Wnt1 up vs E15.5 * Ctrl
10482330	Atp6v1g1	NM_024173	0.00269886	1.19904	E15.5 * Wnt1 up vs E15.5 * Ctrl
10439471	Ktelc1	NM_172380	0.0141261	1.19852	E15.5 * Wnt1 up vs E15.5 * Ctrl
10432162	2310037I24Rik	BC003301	2.36276e-005	1.19827	E15.5 * Wnt1 up vs E15.5 * Ctrl
10602501	Huwe1	NM_021523	7.97118e-006	1.19817	E15.5 * Wnt1 up vs E15.5 * Ctrl
10536407	Phf14	NM_029404	0.0007979	1.19816	E15.5 * Wnt1 up vs E15.5 * Ctrl
10421730	AU021034	NM_177629	0.0105076	1.1981	E15.5 * Wnt1 up vs E15.5 * Ctrl
10556546	Calcb	NM_054084	0.00698339	1.19802	E15.5 * Wnt1 up vs E15.5 * Ctrl
10345967	Bivm	NM_144558	0.00405928	1.19801	E15.5 * Wnt1 up vs E15.5 * Ctrl
10555741	Olfr561	NM_147092	0.00728439	1.19795	E15.5 * Wnt1 up vs E15.5 * Ctrl
10441923	2210404J11Rik	NM_001039552	0.00914947	1.19776	E15.5 * Wnt1 up vs E15.5 * Ctrl
10366335	Bbs10	ENSMUST00000(	0.00465415	1.19773	E15.5 * Wnt1 up vs E15.5 * Ctrl
10529313	Letm1	NM_019694	0.00510594	1.19714	E15.5 * Wnt1 up vs E15.5 * Ctrl
10463158	AI606181	ENSMUST00000(	0.00678833	1.19702	E15.5 * Wnt1 up vs E15.5 * Ctrl
10563895		---	0.0112511	1.19691	E15.5 * Wnt1 up vs E15.5 * Ctrl
10388747	BC017647	ENSMUST00000	0.0111497	1.19677	E15.5 * Wnt1 up vs E15.5 * Ctrl
10607143	Capn6	NM_007603	0.00603241	1.19649	E15.5 * Wnt1 up vs E15.5 * Ctrl
10603125	Asb9	NM_027027	0.0108818	1.19607	E15.5 * Wnt1 up vs E15.5 * Ctrl
10552088	ENSMUSG0000C	ENSMUST00000(	0.00108698	1.19583	E15.5 * Wnt1 up vs E15.5 * Ctrl
10572109	Ints10	NM_027590	0.00189322	1.19582	E15.5 * Wnt1 up vs E15.5 * Ctrl
10524969	Hrk	NM_007545	0.00812524	1.19563	E15.5 * Wnt1 up vs E15.5 * Ctrl

10381018	Msl1	NM_028722	0.00400324	1.19549	E15.5 * Wnt1 up vs E15.5 * Ctrl
10462333	Cdc37l1	NM_025950	0.000352732	1.19542	E15.5 * Wnt1 up vs E15.5 * Ctrl
10557233	Tnrc6a	NM_144925	0.00184444	1.19542	E15.5 * Wnt1 up vs E15.5 * Ctrl
10397249	Lin52	NM_173756	0.00367931	1.19528	E15.5 * Wnt1 up vs E15.5 * Ctrl
10461143	Chrm1	NM_001112697	0.013908	1.19522	E15.5 * Wnt1 up vs E15.5 * Ctrl
10448615	Rab26	BC094931	0.00927683	1.19521	E15.5 * Wnt1 up vs E15.5 * Ctrl
10474950	Vps18	NM_172269	0.0104495	1.19506	E15.5 * Wnt1 up vs E15.5 * Ctrl
10473919	Ckap5	NM_029437	5.86034e-005	1.195	E15.5 * Wnt1 up vs E15.5 * Ctrl
10576940	Fam155a	AK149049	0.0126402	1.195	E15.5 * Wnt1 up vs E15.5 * Ctrl
10562181	Lsr	NM_017405	0.00809241	1.19483	E15.5 * Wnt1 up vs E15.5 * Ctrl
10518726	Slc25a33	NM_027460	0.00665922	1.19476	E15.5 * Wnt1 up vs E15.5 * Ctrl
10522160	N4bp2	NM_001024917	0.0113253	1.19465	E15.5 * Wnt1 up vs E15.5 * Ctrl
10563253	Lin7b	NM_011698	0.00977624	1.19448	E15.5 * Wnt1 up vs E15.5 * Ctrl
10408346	ENSMUSG00000	ENSMUST00000	0.0120944	1.19447	E15.5 * Wnt1 up vs E15.5 * Ctrl
10517948	Spen	NM_019763	0.00851607	1.19418	E15.5 * Wnt1 up vs E15.5 * Ctrl
10474919	OTTMUSG00000	NM_001039223	0.00830121	1.19415	E15.5 * Wnt1 up vs E15.5 * Ctrl
10418616	Dnahc1	BC023155	0.000493018	1.19414	E15.5 * Wnt1 up vs E15.5 * Ctrl
10391610		---	0.0113148	1.19407	E15.5 * Wnt1 up vs E15.5 * Ctrl
10419005	Rgr	NM_021340	0.00197043	1.19405	E15.5 * Wnt1 up vs E15.5 * Ctrl
10385761	Ube2b	NM_009458	0.00754209	1.19375	E15.5 * Wnt1 up vs E15.5 * Ctrl
10534575	Prkrip1	NM_025774	0.00688327	1.19368	E15.5 * Wnt1 up vs E15.5 * Ctrl
10528610	Tmub1	NM_022418	0.00305263	1.19355	E15.5 * Wnt1 up vs E15.5 * Ctrl
10471735	Olfr335	NM_001081410	0.00321526	1.19291	E15.5 * Wnt1 up vs E15.5 * Ctrl
10598229		---	0.00487043	1.19282	E15.5 * Wnt1 up vs E15.5 * Ctrl
10509930	Fbxo42	NM_172518	0.0137717	1.19266	E15.5 * Wnt1 up vs E15.5 * Ctrl
10492755	Fga	NM_001111048	0.012959	1.19254	E15.5 * Wnt1 up vs E15.5 * Ctrl
10434577	Vps8	NM_001081366	5.13268e-005	1.1924	E15.5 * Wnt1 up vs E15.5 * Ctrl
10417492	EG432825	NM_001024706	0.0114067	1.19233	E15.5 * Wnt1 up vs E15.5 * Ctrl
10480579	Mrpl41	NM_001031808	0.00161266	1.19216	E15.5 * Wnt1 up vs E15.5 * Ctrl
10447645	5830477G23Rik	ENSMUST00000	0.00890766	1.19202	E15.5 * Wnt1 up vs E15.5 * Ctrl
10375103	Fbxw11	NM_134015	0.00400984	1.19201	E15.5 * Wnt1 up vs E15.5 * Ctrl
10467001		---	0.0140063	1.19192	E15.5 * Wnt1 up vs E15.5 * Ctrl
10561140	Mrpl41	NM_001031808	0.00112643	1.19191	E15.5 * Wnt1 up vs E15.5 * Ctrl
10512669	Pax5	NM_008782	0.010126	1.19191	E15.5 * Wnt1 up vs E15.5 * Ctrl
10465838	Mta2	NM_011842	0.00069237	1.19182	E15.5 * Wnt1 up vs E15.5 * Ctrl
10587621	Ripply2	NM_001037907	0.00136944	1.19178	E15.5 * Wnt1 up vs E15.5 * Ctrl
10596796	Rbm6	NM_029169	0.000911099	1.19162	E15.5 * Wnt1 up vs E15.5 * Ctrl
10431080	Efcab6	NM_029946	0.00417549	1.19142	E15.5 * Wnt1 up vs E15.5 * Ctrl
10385495	Cdk2ap1	NM_013812	0.00245455	1.19111	E15.5 * Wnt1 up vs E15.5 * Ctrl
10472630	Ubr3	NM_001081548	0.00307635	1.19096	E15.5 * Wnt1 up vs E15.5 * Ctrl
10488844	Ncoa6	NM_019825	0.00110397	1.19087	E15.5 * Wnt1 up vs E15.5 * Ctrl
10363498	Ppa1	NM_026438	0.00121719	1.19081	E15.5 * Wnt1 up vs E15.5 * Ctrl
10417749	Ube2e1	NM_009455	1.28918e-005	1.19077	E15.5 * Wnt1 up vs E15.5 * Ctrl
10533703	Rsrc2	NM_025438	0.00029827	1.19069	E15.5 * Wnt1 up vs E15.5 * Ctrl
10594515		---	0.012597	1.19035	E15.5 * Wnt1 up vs E15.5 * Ctrl
10564057		---	0.0093114	1.19024	E15.5 * Wnt1 up vs E15.5 * Ctrl
10560964	Pou2f2	NM_011138	0.0101374	1.19013	E15.5 * Wnt1 up vs E15.5 * Ctrl
10399897	Hbp1	NM_153198	0.000206891	1.19009	E15.5 * Wnt1 up vs E15.5 * Ctrl
10415119	Pabpn1	NM_019402	0.000132608	1.18989	E15.5 * Wnt1 up vs E15.5 * Ctrl
10593159	Pafah1b2	NM_008775	0.00193806	1.18979	E15.5 * Wnt1 up vs E15.5 * Ctrl
10374181	OTTMUSG00000	XM_001001271	0.000618474	1.18979	E15.5 * Wnt1 up vs E15.5 * Ctrl
10555921	EG545989	ENSMUST00000	0.00760265	1.18973	E15.5 * Wnt1 up vs E15.5 * Ctrl
10349913	Kiss1	NM_178260	0.00681641	1.1895	E15.5 * Wnt1 up vs E15.5 * Ctrl
10601272	Pin4	NM_027181	0.00122794	1.18936	E15.5 * Wnt1 up vs E15.5 * Ctrl
10466439	Pebp1	NM_018858	0.000161776	1.18925	E15.5 * Wnt1 up vs E15.5 * Ctrl
10381697	Hexim1	NM_138753	0.0110535	1.1891	E15.5 * Wnt1 up vs E15.5 * Ctrl
10515144	Skint10	NM_177668	0.00702906	1.18893	E15.5 * Wnt1 up vs E15.5 * Ctrl
10464999	Cst6	NM_028623	0.0052481	1.18883	E15.5 * Wnt1 up vs E15.5 * Ctrl
10453248		---	0.00774876	1.18857	E15.5 * Wnt1 up vs E15.5 * Ctrl
10532472	Ulk1	NM_009469	0.0132352	1.18828	E15.5 * Wnt1 up vs E15.5 * Ctrl
10489266	Chd6	NM_173368	0.00208655	1.18813	E15.5 * Wnt1 up vs E15.5 * Ctrl
10369920	AI646023	NM_198860	0.00985416	1.18797	E15.5 * Wnt1 up vs E15.5 * Ctrl
10542223	5430401F13Rik	ENSMUST00000	0.0134824	1.18791	E15.5 * Wnt1 up vs E15.5 * Ctrl
10408130	V1rh15	ENSMUST00000	0.00829635	1.18759	E15.5 * Wnt1 up vs E15.5 * Ctrl
10551760	Zfp84	NM_023750	0.0113132	1.18753	E15.5 * Wnt1 up vs E15.5 * Ctrl
10417053	Mbnl2	NM_175341	0.0030359	1.18732	E15.5 * Wnt1 up vs E15.5 * Ctrl

10486858	Mfap1a	NM_026220	0.00675917	1.18731	E15.5 * Wnt1 up vs E15.5 * Ctrl
10350992	Rc3h1	NM_001024952	0.0100508	1.18714	E15.5 * Wnt1 up vs E15.5 * Ctrl
10463930	Mxi1	NM_010847	0.0112945	1.18696	E15.5 * Wnt1 up vs E15.5 * Ctrl
10567508	Dnahc3	XM_355934	0.00988282	1.18664	E15.5 * Wnt1 up vs E15.5 * Ctrl
10363894	Ipmk	NM_027184	0.00433318	1.18662	E15.5 * Wnt1 up vs E15.5 * Ctrl
10576373	Gas8	NM_018855	0.00126403	1.18646	E15.5 * Wnt1 up vs E15.5 * Ctrl
10519414	4932412H11Rik	NM_172879	0.00736872	1.18644	E15.5 * Wnt1 up vs E15.5 * Ctrl
10421615	Spert	NM_026457	0.00652207	1.18643	E15.5 * Wnt1 up vs E15.5 * Ctrl
10555746	Olfr568	NM_147091	0.00165329	1.18634	E15.5 * Wnt1 up vs E15.5 * Ctrl
10572064		---	0.00285825	1.18629	E15.5 * Wnt1 up vs E15.5 * Ctrl
10525296	Brap	NM_028227	0.00266936	1.186	E15.5 * Wnt1 up vs E15.5 * Ctrl
10466347	Olfr1457	NM_146575	0.0114508	1.18583	E15.5 * Wnt1 up vs E15.5 * Ctrl
10348424	Sh3bp4	NM_133816	0.00832234	1.18561	E15.5 * Wnt1 up vs E15.5 * Ctrl
10396919	4933426M11Rik	BC040401	0.00355105	1.1856	E15.5 * Wnt1 up vs E15.5 * Ctrl
10362392	Psmd2	NR_027485	0.0089093	1.18546	E15.5 * Wnt1 up vs E15.5 * Ctrl
10405924		---	0.010172	1.18534	E15.5 * Wnt1 up vs E15.5 * Ctrl
10589756	Epm2aip1	NM_175266	0.00331679	1.18526	E15.5 * Wnt1 up vs E15.5 * Ctrl
10417689	Psmd6	NM_025550	0.000342558	1.18516	E15.5 * Wnt1 up vs E15.5 * Ctrl
10453451	Calm2	NM_007589	1.0301e-005	1.18514	E15.5 * Wnt1 up vs E15.5 * Ctrl
10593652	Cyp19a1	NM_007810	0.0143551	1.18502	E15.5 * Wnt1 up vs E15.5 * Ctrl
10424559	Khdrbs3	NM_010158	0.00353132	1.18488	E15.5 * Wnt1 up vs E15.5 * Ctrl
10559743	V1rd10	NM_207543	0.00519043	1.18459	E15.5 * Wnt1 up vs E15.5 * Ctrl
10512709	Mcart1	NM_001009949	0.000874824	1.18444	E15.5 * Wnt1 up vs E15.5 * Ctrl
10479607	Lime1	NM_023684	0.00333527	1.18438	E15.5 * Wnt1 up vs E15.5 * Ctrl
10446376	Man2a1	NM_008549	0.0100834	1.18432	E15.5 * Wnt1 up vs E15.5 * Ctrl
10383883	OTTMUSG00000	ENSMUST00000	0.0032339	1.18401	E15.5 * Wnt1 up vs E15.5 * Ctrl
10508465	Marcksl1	NM_010807	0.00302023	1.18372	E15.5 * Wnt1 up vs E15.5 * Ctrl
10606926	1700025D03Rik	NM_026469	0.000160655	1.18348	E15.5 * Wnt1 up vs E15.5 * Ctrl
10575662	Mon1b	NM_173015	0.0106777	1.18342	E15.5 * Wnt1 up vs E15.5 * Ctrl
10416680	Pcdh8	NM_021543	0.0122108	1.18336	E15.5 * Wnt1 up vs E15.5 * Ctrl
10541065	Olfr213	NM_001011801	0.00792767	1.18315	E15.5 * Wnt1 up vs E15.5 * Ctrl
10496892	Fubp1	NM_057172	3.54435e-005	1.18312	E15.5 * Wnt1 up vs E15.5 * Ctrl
10545096	2410003J06Rik	NM_028025	0.0118601	1.1831	E15.5 * Wnt1 up vs E15.5 * Ctrl
10488734	Spag4l	NM_029599	0.00621862	1.18308	E15.5 * Wnt1 up vs E15.5 * Ctrl
10550762	EG545929	NM_001034904	0.00729315	1.18307	E15.5 * Wnt1 up vs E15.5 * Ctrl
10550775			0.00729315	1.18307	E15.5 * Wnt1 up vs E15.5 * Ctrl
10558458	Ppp2r2d	NM_026391	0.00887708	1.18285	E15.5 * Wnt1 up vs E15.5 * Ctrl
10512011	Zfp292	NM_013889	0.0019031	1.18254	E15.5 * Wnt1 up vs E15.5 * Ctrl
10362359	Pebp1	NM_018858	0.000129461	1.18247	E15.5 * Wnt1 up vs E15.5 * Ctrl
10605598	CN716893	NM_001033492	0.0140511	1.18206	E15.5 * Wnt1 up vs E15.5 * Ctrl
10432398	Tuba1b	NM_011654	6.2609e-005	1.18201	E15.5 * Wnt1 up vs E15.5 * Ctrl
10489629	Cdh22	NM_174988	0.00889288	1.18174	E15.5 * Wnt1 up vs E15.5 * Ctrl
10479639	Dnajc5	NM_016775	0.00555593	1.18168	E15.5 * Wnt1 up vs E15.5 * Ctrl
10496919	Usp33	NM_133247	0.00131884	1.18147	E15.5 * Wnt1 up vs E15.5 * Ctrl
10423733	Vps13b	ENSMUST00000	0.0138631	1.18129	E15.5 * Wnt1 up vs E15.5 * Ctrl
10530563	Fryl	NM_028194	0.000573254	1.18098	E15.5 * Wnt1 up vs E15.5 * Ctrl
10372926		---	0.00136319	1.18094	E15.5 * Wnt1 up vs E15.5 * Ctrl
10579500	Abhd8	NM_022419	0.00639998	1.18086	E15.5 * Wnt1 up vs E15.5 * Ctrl
10525983	Ran	NM_009391	0.00145168	1.18071	E15.5 * Wnt1 up vs E15.5 * Ctrl
10521705	Clrn2	BC147371	0.00174482	1.18071	E15.5 * Wnt1 up vs E15.5 * Ctrl
10348983		---	0.00490535	1.18053	E15.5 * Wnt1 up vs E15.5 * Ctrl
10564849	2610034B18Rik	NM_027420	0.00502236	1.1805	E15.5 * Wnt1 up vs E15.5 * Ctrl
10401002	Gphb5	NM_175644	0.0122009	1.1805	E15.5 * Wnt1 up vs E15.5 * Ctrl
10515080	4930522H14Rik	NM_026291	0.00813449	1.17995	E15.5 * Wnt1 up vs E15.5 * Ctrl
10374151	Zmiz2	NM_028601	0.0120853	1.17995	E15.5 * Wnt1 up vs E15.5 * Ctrl
10599346	Polr2k	NM_001039368	0.010803	1.17992	E15.5 * Wnt1 up vs E15.5 * Ctrl
10440717	Krtap6-1	ENSMUST00000	0.00426004	1.17992	E15.5 * Wnt1 up vs E15.5 * Ctrl
10390084	A430060F13Rik	ENSMUST00000	0.0118273	1.17992	E15.5 * Wnt1 up vs E15.5 * Ctrl
10455094	Pcdhb13	NM_053138	0.010573	1.1798	E15.5 * Wnt1 up vs E15.5 * Ctrl
10419596	Zfp219	NM_027248	0.0112601	1.17951	E15.5 * Wnt1 up vs E15.5 * Ctrl
10584602	ENSMUSG00000	ENSMUST00000	0.00727943	1.1795	E15.5 * Wnt1 up vs E15.5 * Ctrl
10391410	Becn1	NM_019584	0.00359222	1.17937	E15.5 * Wnt1 up vs E15.5 * Ctrl
10434670	EG666421	XR_035704	0.00196007	1.17932	E15.5 * Wnt1 up vs E15.5 * Ctrl
10569771	Mcoln1	NM_053177	0.009315	1.17922	E15.5 * Wnt1 up vs E15.5 * Ctrl
10432139	Zfp641	NM_173769	0.00633384	1.17908	E15.5 * Wnt1 up vs E15.5 * Ctrl
10381617		---	0.00663237	1.17902	E15.5 * Wnt1 up vs E15.5 * Ctrl

10369132	Fam184a	NM_001081428	0.0125376	1.17893	E15.5 * Wnt1 up vs E15.5 * Ctrl
10497181	1700008P02Rik	ENSMUST00000	0.00932265	1.17893	E15.5 * Wnt1 up vs E15.5 * Ctrl
10406795	Gfm2	NM_177266	0.00222743	1.17891	E15.5 * Wnt1 up vs E15.5 * Ctrl
10410690		---	0.00389101	1.17887	E15.5 * Wnt1 up vs E15.5 * Ctrl
10361200	AA408296	NM_145415	0.0013864	1.17868	E15.5 * Wnt1 up vs E15.5 * Ctrl
10422500	EG328479	NM_177817	0.00644642	1.17868	E15.5 * Wnt1 up vs E15.5 * Ctrl
10414493	Olfr748	NM_001011837	0.0093615	1.17836	E15.5 * Wnt1 up vs E15.5 * Ctrl
10467342	ENSMUSG00000	ENSMUST00000	0.0110977	1.17828	E15.5 * Wnt1 up vs E15.5 * Ctrl
10581640	Atxn1l	ENSMUST00000	0.000880283	1.17809	E15.5 * Wnt1 up vs E15.5 * Ctrl
10450669	Prr3	NM_145487	0.000149669	1.17806	E15.5 * Wnt1 up vs E15.5 * Ctrl
10475293	Tubgcp4	NM_153387	0.00947254	1.17793	E15.5 * Wnt1 up vs E15.5 * Ctrl
10525336	Fam109a	NM_175474	0.00278102	1.17791	E15.5 * Wnt1 up vs E15.5 * Ctrl
10507719	Slfnl1	NM_177570	0.0117724	1.17779	E15.5 * Wnt1 up vs E15.5 * Ctrl
10534988		---	0.00526224	1.17771	E15.5 * Wnt1 up vs E15.5 * Ctrl
10387625	Chrnb1	NM_009601	0.00233957	1.17731	E15.5 * Wnt1 up vs E15.5 * Ctrl
10463361	Scd4	NM_183216	0.00607406	1.17711	E15.5 * Wnt1 up vs E15.5 * Ctrl
10386495	Tom1l2	NM_153080	0.00397935	1.1771	E15.5 * Wnt1 up vs E15.5 * Ctrl
10453461	Fbxo11	NM_001081034	0.000908521	1.17701	E15.5 * Wnt1 up vs E15.5 * Ctrl
10541349		---	0.00388241	1.17696	E15.5 * Wnt1 up vs E15.5 * Ctrl
10449654	ENSMUSG00000	ENSMUST00000	0.00149489	1.17655	E15.5 * Wnt1 up vs E15.5 * Ctrl
10380384	Ankrd40	NM_027799	0.00130552	1.17635	E15.5 * Wnt1 up vs E15.5 * Ctrl
10462791	March5	NM_027314	0.00703131	1.17632	E15.5 * Wnt1 up vs E15.5 * Ctrl
10462330	Ppapdc2	NM_028922	0.00988092	1.17632	E15.5 * Wnt1 up vs E15.5 * Ctrl
10388938	Wsb1	NM_019653	0.00473977	1.17628	E15.5 * Wnt1 up vs E15.5 * Ctrl
10530666	Lnx1	NM_010727	0.000334993	1.17626	E15.5 * Wnt1 up vs E15.5 * Ctrl
10437846	Ifitm7	NM_028968	0.00219671	1.17625	E15.5 * Wnt1 up vs E15.5 * Ctrl
10377001	Tmem220	NM_177392	0.0133248	1.17617	E15.5 * Wnt1 up vs E15.5 * Ctrl
10367515		---	0.000400717	1.17613	E15.5 * Wnt1 up vs E15.5 * Ctrl
10537169	Akr1b7	NM_009731	0.0146003	1.1756	E15.5 * Wnt1 up vs E15.5 * Ctrl
10473477		---	0.000979798	1.17553	E15.5 * Wnt1 up vs E15.5 * Ctrl
10502982	Tnni3k	NM_177066	0.00211331	1.17539	E15.5 * Wnt1 up vs E15.5 * Ctrl
10477361	Efcab8	BC144855	0.00685439	1.17518	E15.5 * Wnt1 up vs E15.5 * Ctrl
10608298		---	0.00529633	1.17514	E15.5 * Wnt1 up vs E15.5 * Ctrl
10608311		---	0.00529633	1.17514	E15.5 * Wnt1 up vs E15.5 * Ctrl
10608533		---	0.00529633	1.17514	E15.5 * Wnt1 up vs E15.5 * Ctrl
10353707	Ptp4a1	NM_011200	0.00571775	1.17496	E15.5 * Wnt1 up vs E15.5 * Ctrl
10495197	ENSMUSG00000	ENSMUST00000	0.0143555	1.17479	E15.5 * Wnt1 up vs E15.5 * Ctrl
10584071	Prdm10	NM_001080817	0.00460598	1.17473	E15.5 * Wnt1 up vs E15.5 * Ctrl
10384529	Cep68	NM_172260	0.00291093	1.17471	E15.5 * Wnt1 up vs E15.5 * Ctrl
10366528	Best3	NM_001007583	0.00943142	1.17442	E15.5 * Wnt1 up vs E15.5 * Ctrl
10511282	Tnfrsf4	NM_011659	0.00237071	1.17437	E15.5 * Wnt1 up vs E15.5 * Ctrl
10550003	Zfp606	NM_026112	0.00380887	1.17411	E15.5 * Wnt1 up vs E15.5 * Ctrl
10607679	4932441K18Rik	NM_178935	0.0134841	1.17393	E15.5 * Wnt1 up vs E15.5 * Ctrl
10352957	Rgs20	NM_021374	0.0057088	1.17369	E15.5 * Wnt1 up vs E15.5 * Ctrl
10402444	Dicer1	NM_148948	0.000216164	1.17366	E15.5 * Wnt1 up vs E15.5 * Ctrl
10544501	Ezh2	NM_007971	0.000942956	1.17335	E15.5 * Wnt1 up vs E15.5 * Ctrl
10559513	Cab39	NM_133781	0.0065139	1.17335	E15.5 * Wnt1 up vs E15.5 * Ctrl
10489458	Svs4	NM_009300	0.00212087	1.17333	E15.5 * Wnt1 up vs E15.5 * Ctrl
10512807	Gabbr2	NM_001081141	0.00296063	1.17317	E15.5 * Wnt1 up vs E15.5 * Ctrl
10577560	Ikbkb	NM_010546	0.00402475	1.17313	E15.5 * Wnt1 up vs E15.5 * Ctrl
10511898	Gja10	NM_010289	0.0120007	1.17307	E15.5 * Wnt1 up vs E15.5 * Ctrl
10356475	Arl4c	NM_177305	0.00809654	1.17306	E15.5 * Wnt1 up vs E15.5 * Ctrl
10426150	Panx2	NM_001002005	0.00567976	1.17294	E15.5 * Wnt1 up vs E15.5 * Ctrl
10590962	1700012B09Rik	NM_029306	0.000603047	1.17279	E15.5 * Wnt1 up vs E15.5 * Ctrl
10601091	Foxo4	NM_018789	0.000718544	1.17259	E15.5 * Wnt1 up vs E15.5 * Ctrl
10494299	Ensa	NM_019561	0.00271706	1.17244	E15.5 * Wnt1 up vs E15.5 * Ctrl
10363983	Bcr	NM_001081412	0.00390372	1.1724	E15.5 * Wnt1 up vs E15.5 * Ctrl
10499996	Snx27	NM_029721	0.000764642	1.17237	E15.5 * Wnt1 up vs E15.5 * Ctrl
10513867	Cdk5rap2	ENSMUST00000	0.000254647	1.17205	E15.5 * Wnt1 up vs E15.5 * Ctrl
10374564	Cct4	NM_009837	0.000703594	1.17183	E15.5 * Wnt1 up vs E15.5 * Ctrl
10379891	Ppm1d	NM_016910	0.00862847	1.1717	E15.5 * Wnt1 up vs E15.5 * Ctrl
10418989		---	0.00686672	1.17147	E15.5 * Wnt1 up vs E15.5 * Ctrl
10452419	Efna5	NM_207654	0.000771211	1.17143	E15.5 * Wnt1 up vs E15.5 * Ctrl
10529068	Slc30a3	NM_011773	0.00544847	1.17108	E15.5 * Wnt1 up vs E15.5 * Ctrl
10533807	Cdk2ap1	NM_013812	0.00459918	1.17083	E15.5 * Wnt1 up vs E15.5 * Ctrl
10345037	Paqr8	NM_028829	0.000201921	1.17063	E15.5 * Wnt1 up vs E15.5 * Ctrl

10359299		---	0.0121094	1.17027	E15.5 * Wnt1 up vs E15.5 * Ctrl
10437330	Crebbp	ENSMUST00000	6.50465e-005	1.17006	E15.5 * Wnt1 up vs E15.5 * Ctrl
10378154	Zzef1	NM_001045536	0.00556089	1.16959	E15.5 * Wnt1 up vs E15.5 * Ctrl
10557631	Prr14	NM_145589	0.000682023	1.16954	E15.5 * Wnt1 up vs E15.5 * Ctrl
10587255	Klhl31	NM_172925	0.0147855	1.16953	E15.5 * Wnt1 up vs E15.5 * Ctrl
10396358	Ppm1a	NM_008910	0.00291322	1.16948	E15.5 * Wnt1 up vs E15.5 * Ctrl
10401882	Gtf2a1	NM_031391	0.000140262	1.1694	E15.5 * Wnt1 up vs E15.5 * Ctrl
10432734	4732456N10Rik	NM_177717	0.00625275	1.16935	E15.5 * Wnt1 up vs E15.5 * Ctrl
10414987	OTTMUSG00000	ENSMUST00000	0.0075717	1.16932	E15.5 * Wnt1 up vs E15.5 * Ctrl
10538100	Repin1	NM_001079901	0.0023178	1.169	E15.5 * Wnt1 up vs E15.5 * Ctrl
10469575	OTTMUSG00000	NR_002688	0.00577711	1.16882	E15.5 * Wnt1 up vs E15.5 * Ctrl
10581049	Cmtm2a	NM_027022	0.00417101	1.16858	E15.5 * Wnt1 up vs E15.5 * Ctrl
10370541		---	0.0123484	1.16837	E15.5 * Wnt1 up vs E15.5 * Ctrl
10433963	Ydjc	NM_026940	0.0105855	1.168	E15.5 * Wnt1 up vs E15.5 * Ctrl
10392998	Caskin2	NM_080643	0.00526211	1.16794	E15.5 * Wnt1 up vs E15.5 * Ctrl
10407300	4930544M13Rik	ENSMUST00000	0.0131666	1.16709	E15.5 * Wnt1 up vs E15.5 * Ctrl
10365145	Tle2	NM_019725	0.012464	1.16673	E15.5 * Wnt1 up vs E15.5 * Ctrl
10592942	Mll1	NM_001081049	0.000145662	1.16663	E15.5 * Wnt1 up vs E15.5 * Ctrl
10467315	I830134H01Rik	ENSMUST00000	0.00617446	1.16651	E15.5 * Wnt1 up vs E15.5 * Ctrl
10598187		---	0.0021681	1.16644	E15.5 * Wnt1 up vs E15.5 * Ctrl
10418578	Nisch	NM_022656	0.00597718	1.16631	E15.5 * Wnt1 up vs E15.5 * Ctrl
10575861	Hsbp1	NM_024219	0.00148312	1.16612	E15.5 * Wnt1 up vs E15.5 * Ctrl
10516943	Atpif1	NM_007512	0.000236305	1.16594	E15.5 * Wnt1 up vs E15.5 * Ctrl
10579422	Kcnn1	NM_032397	0.0015347	1.16593	E15.5 * Wnt1 up vs E15.5 * Ctrl
10545032	V1rc17	NM_134172	0.00350225	1.16585	E15.5 * Wnt1 up vs E15.5 * Ctrl
10397741	Psmc1	NM_008947	0.00814414	1.16531	E15.5 * Wnt1 up vs E15.5 * Ctrl
10354168	Tbc1d8	NM_018775	0.00721108	1.16508	E15.5 * Wnt1 up vs E15.5 * Ctrl
10539321	Ccdc142	NM_001081266	0.00957762	1.16502	E15.5 * Wnt1 up vs E15.5 * Ctrl
10454525	Iws1	NM_173441	0.000535747	1.16486	E15.5 * Wnt1 up vs E15.5 * Ctrl
10414947	EG667677	ENSMUST00000	0.010842	1.16481	E15.5 * Wnt1 up vs E15.5 * Ctrl
10375704	Sqstm1	NM_011018	0.000239623	1.16475	E15.5 * Wnt1 up vs E15.5 * Ctrl
10531133	Grsf1	NM_178700	0.000104302	1.16474	E15.5 * Wnt1 up vs E15.5 * Ctrl
10505438	Orm1	NM_008768	0.00616166	1.1647	E15.5 * Wnt1 up vs E15.5 * Ctrl
10566403	Olfr668	NM_147059	0.00182015	1.16451	E15.5 * Wnt1 up vs E15.5 * Ctrl
10575129	Vps4a	NM_126165	0.00370612	1.16434	E15.5 * Wnt1 up vs E15.5 * Ctrl
10537826	Olfr435	NM_146653	0.00611116	1.16411	E15.5 * Wnt1 up vs E15.5 * Ctrl
10447708	Qk	NM_021881	0.00109745	1.16389	E15.5 * Wnt1 up vs E15.5 * Ctrl
10459637		---	0.00486302	1.16383	E15.5 * Wnt1 up vs E15.5 * Ctrl
10412378	H3f3a	NM_008210	1.69722e-005	1.1638	E15.5 * Wnt1 up vs E15.5 * Ctrl
10578743	Adam29	NM_175939	0.0044393	1.16354	E15.5 * Wnt1 up vs E15.5 * Ctrl
10438112	Pi4ka	NM_001001983	0.00121308	1.16348	E15.5 * Wnt1 up vs E15.5 * Ctrl
10426507	Arid2	NM_175251	0.0020469	1.16346	E15.5 * Wnt1 up vs E15.5 * Ctrl
10397277	Fcf1	NM_028632	0.0111404	1.1634	E15.5 * Wnt1 up vs E15.5 * Ctrl
10390901	Krtap1-4	NM_001039502	0.0114897	1.16318	E15.5 * Wnt1 up vs E15.5 * Ctrl
10597173	Nradd	NM_026012	0.0030462	1.16312	E15.5 * Wnt1 up vs E15.5 * Ctrl
10388896		---	0.0144138	1.16311	E15.5 * Wnt1 up vs E15.5 * Ctrl
10461038	Macrod1	NM_134147	0.00451391	1.16288	E15.5 * Wnt1 up vs E15.5 * Ctrl
10391348	Fam134c	NM_028933	0.00972046	1.16281	E15.5 * Wnt1 up vs E15.5 * Ctrl
10601484	2010106E10Rik	NM_026333	0.00157675	1.1628	E15.5 * Wnt1 up vs E15.5 * Ctrl
10527888	Gatad1	NM_026033	0.00108712	1.16268	E15.5 * Wnt1 up vs E15.5 * Ctrl
10547789	Grcc10	NM_013535	0.0125792	1.16205	E15.5 * Wnt1 up vs E15.5 * Ctrl
10467489	Ptp4a1	NM_011200	0.00874385	1.16202	E15.5 * Wnt1 up vs E15.5 * Ctrl
10389170	Gas2l2	NM_001013759	0.00214638	1.16183	E15.5 * Wnt1 up vs E15.5 * Ctrl
10560035	Zscan18	BC094341	0.00341841	1.16174	E15.5 * Wnt1 up vs E15.5 * Ctrl
10485395	Trim44	NM_020267	0.00236031	1.16164	E15.5 * Wnt1 up vs E15.5 * Ctrl
10532317	Gtpbp6	NM_145147	0.00977362	1.16138	E15.5 * Wnt1 up vs E15.5 * Ctrl
10473343	Med19	NM_025885	0.0121547	1.16132	E15.5 * Wnt1 up vs E15.5 * Ctrl
10447695		---	0.00385464	1.16118	E15.5 * Wnt1 up vs E15.5 * Ctrl
10571474	Pcm1	NM_023662	0.00760961	1.16115	E15.5 * Wnt1 up vs E15.5 * Ctrl
10546631	Frmd4b	NM_145148	0.0121771	1.16109	E15.5 * Wnt1 up vs E15.5 * Ctrl
10372443	Tph2	NM_173391	0.0100141	1.16077	E15.5 * Wnt1 up vs E15.5 * Ctrl
10515280		---	0.00745219	1.16071	E15.5 * Wnt1 up vs E15.5 * Ctrl
10420131	Rabggta	NM_019519	0.013408	1.16028	E15.5 * Wnt1 up vs E15.5 * Ctrl
10486867	Mfap1a	NM_026220	0.00492077	1.16014	E15.5 * Wnt1 up vs E15.5 * Ctrl
10414522	Apex1	NM_009687	0.00285729	1.16013	E15.5 * Wnt1 up vs E15.5 * Ctrl
10606263	Atrx	NM_009530	0.00972349	1.15992	E15.5 * Wnt1 up vs E15.5 * Ctrl

10443550	Rnf8	NM_021419	0.00251256	1.15984	E15.5 * Wnt1 up vs E15.5 * Ctrl
10386965	AU040829	NM_175003	0.00893397	1.15978	E15.5 * Wnt1 up vs E15.5 * Ctrl
10532984	Dynll1	NM_019682	0.00196408	1.15966	E15.5 * Wnt1 up vs E15.5 * Ctrl
10478463	Svs6	NM_013679	0.000344465	1.15954	E15.5 * Wnt1 up vs E15.5 * Ctrl
10430096	Lrrc24	NM_198119	0.00118084	1.15945	E15.5 * Wnt1 up vs E15.5 * Ctrl
10520417	Gm1040	ENSMUST00000(	0.0119156	1.15942	E15.5 * Wnt1 up vs E15.5 * Ctrl
10557206	Rbbp6	NM_175023	9.52853e-005	1.15889	E15.5 * Wnt1 up vs E15.5 * Ctrl
10544263	1700016G05Rik	NM_026317	0.00785554	1.15879	E15.5 * Wnt1 up vs E15.5 * Ctrl
10383545	Foxk2	NM_001080932	0.00621417	1.1586	E15.5 * Wnt1 up vs E15.5 * Ctrl
10594969	Unc13c	NM_001081153	0.0138537	1.15837	E15.5 * Wnt1 up vs E15.5 * Ctrl
10429462	1700016M24Rik	BC111897	0.0105648	1.15829	E15.5 * Wnt1 up vs E15.5 * Ctrl
10566877	Sbf2	BC067204	0.00190124	1.15807	E15.5 * Wnt1 up vs E15.5 * Ctrl
10528236		---	0.013002	1.15759	E15.5 * Wnt1 up vs E15.5 * Ctrl
10527559	Polr1d	NM_009087	0.0145643	1.15748	E15.5 * Wnt1 up vs E15.5 * Ctrl
10562767	1700008O03Rik	ENSMUST00000	0.00503503	1.15726	E15.5 * Wnt1 up vs E15.5 * Ctrl
10352194	Cdc42bpa	BC158017	0.00388887	1.15701	E15.5 * Wnt1 up vs E15.5 * Ctrl
10471761	Olfr352	NM_146940	0.013186	1.15641	E15.5 * Wnt1 up vs E15.5 * Ctrl
10425527	Ep300	NM_177821	0.00279671	1.15621	E15.5 * Wnt1 up vs E15.5 * Ctrl
10537318	Trim24	NM_145076	0.0063459	1.15617	E15.5 * Wnt1 up vs E15.5 * Ctrl
10521031	Ywhah	NM_011738	0.0133289	1.15617	E15.5 * Wnt1 up vs E15.5 * Ctrl
10464342	6430537H07Rik	NM_178689	0.0043126	1.15595	E15.5 * Wnt1 up vs E15.5 * Ctrl
10371987	Metap2	NM_019648	0.000321721	1.15554	E15.5 * Wnt1 up vs E15.5 * Ctrl
10426240	LOC672946	XM_001474273	0.00894853	1.15538	E15.5 * Wnt1 up vs E15.5 * Ctrl
10465263	Dpf2	NM_011262	0.00864235	1.15531	E15.5 * Wnt1 up vs E15.5 * Ctrl
10455974	Dctn4	NM_026302	0.0084548	1.1551	E15.5 * Wnt1 up vs E15.5 * Ctrl
10360794	Fbxo28	NM_175127	0.000284725	1.15499	E15.5 * Wnt1 up vs E15.5 * Ctrl
10499839	Snapin	NM_133854	0.000790562	1.15417	E15.5 * Wnt1 up vs E15.5 * Ctrl
10579925	Gab1	NM_021356	0.0107271	1.15383	E15.5 * Wnt1 up vs E15.5 * Ctrl
10597933		---	0.00214855	1.15371	E15.5 * Wnt1 up vs E15.5 * Ctrl
10386743	Epn2	NM_010148	0.000371436	1.15369	E15.5 * Wnt1 up vs E15.5 * Ctrl
10349480	R3hdm1	NM_181750	0.00969913	1.15361	E15.5 * Wnt1 up vs E15.5 * Ctrl
10537957	Krba1	NM_133922	0.00750193	1.1534	E15.5 * Wnt1 up vs E15.5 * Ctrl
10532901	OTTMUSG00000	XR_033041	0.00344324	1.15329	E15.5 * Wnt1 up vs E15.5 * Ctrl
10563961		---	0.00142706	1.15297	E15.5 * Wnt1 up vs E15.5 * Ctrl
10426042	Gramd4	NM_172611	0.014424	1.15287	E15.5 * Wnt1 up vs E15.5 * Ctrl
10470392	Adamtsl2	NM_029981	0.000559039	1.15276	E15.5 * Wnt1 up vs E15.5 * Ctrl
10498296	Commd2	NM_175095	0.00274274	1.15204	E15.5 * Wnt1 up vs E15.5 * Ctrl
10540507	H3f3a	NM_008210	8.47805e-005	1.15189	E15.5 * Wnt1 up vs E15.5 * Ctrl
10363481	Nodal	NM_013611	0.00953017	1.1518	E15.5 * Wnt1 up vs E15.5 * Ctrl
10514405	Plaa	NM_172695	0.00705328	1.15159	E15.5 * Wnt1 up vs E15.5 * Ctrl
10544608		---	0.00711631	1.15152	E15.5 * Wnt1 up vs E15.5 * Ctrl
10387014	Map2k4	NM_009157	0.00856913	1.15085	E15.5 * Wnt1 up vs E15.5 * Ctrl
10358717	1700025G04Rik	BC034723	0.00539851	1.15084	E15.5 * Wnt1 up vs E15.5 * Ctrl
10357917	Tmem183a	NM_020588	0.0121951	1.15082	E15.5 * Wnt1 up vs E15.5 * Ctrl
10560760	EG545929	NM_001034904	0.0130355	1.15041	E15.5 * Wnt1 up vs E15.5 * Ctrl
10560773	EG545929	NM_001034904	0.0130355	1.15041	E15.5 * Wnt1 up vs E15.5 * Ctrl
10560803	EG545929	NM_001034904	0.0130355	1.15041	E15.5 * Wnt1 up vs E15.5 * Ctrl
10397281	Ylpm1	NM_178363	0.000775252	1.15021	E15.5 * Wnt1 up vs E15.5 * Ctrl
10527430	Arpc1a	NM_019767	0.00109571	1.15019	E15.5 * Wnt1 up vs E15.5 * Ctrl
10347254	Smarcal1	NM_018817	0.00464292	1.1488	E15.5 * Wnt1 up vs E15.5 * Ctrl
10460018	Zfp236	BC138187	0.00390656	1.14853	E15.5 * Wnt1 up vs E15.5 * Ctrl
10593367	Dlat	NM_145614	0.00297958	1.14836	E15.5 * Wnt1 up vs E15.5 * Ctrl
10561748	1700067C01Rik	NM_029714	0.00482782	1.14795	E15.5 * Wnt1 up vs E15.5 * Ctrl
10569513	Osbpl5	NM_024289	0.00719284	1.14771	E15.5 * Wnt1 up vs E15.5 * Ctrl
10376474	Mrpl55	NM_026035	0.012053	1.14734	E15.5 * Wnt1 up vs E15.5 * Ctrl
10569992	LOC674846	XR_031532	0.00657029	1.1473	E15.5 * Wnt1 up vs E15.5 * Ctrl
10364112	EG333669	NM_198635	0.0087498	1.14694	E15.5 * Wnt1 up vs E15.5 * Ctrl
10498332	Siah2	NM_009174	0.0145645	1.14682	E15.5 * Wnt1 up vs E15.5 * Ctrl
10372478	Rab21	NM_024454	0.00696974	1.14589	E15.5 * Wnt1 up vs E15.5 * Ctrl
10595798	BC043934	BC035950	0.00724597	1.14561	E15.5 * Wnt1 up vs E15.5 * Ctrl
10454966	Ik	NM_011879	0.000228339	1.14559	E15.5 * Wnt1 up vs E15.5 * Ctrl
10577419		---	0.00497566	1.14548	E15.5 * Wnt1 up vs E15.5 * Ctrl
10518361	Smarca5	NM_053124	0.0117628	1.1451	E15.5 * Wnt1 up vs E15.5 * Ctrl
10427807	Sub1	NM_011294	2.46433e-005	1.14494	E15.5 * Wnt1 up vs E15.5 * Ctrl
10600836	Msn	NM_010833	0.00680532	1.14492	E15.5 * Wnt1 up vs E15.5 * Ctrl
10568638	Uros	NM_009479	0.0125432	1.14445	E15.5 * Wnt1 up vs E15.5 * Ctrl

10410007	Fbp1	NM_019395	0.0123634	1.14438	E15.5 * Wnt1 up vs E15.5 * Ctrl
10592938	Ttc36	NM_138951	0.0112332	1.14433	E15.5 * Wnt1 up vs E15.5 * Ctrl
10383754		---	0.0125166	1.14414	E15.5 * Wnt1 up vs E15.5 * Ctrl
10441610	Rshl2a	NM_025789	0.00547632	1.14384	E15.5 * Wnt1 up vs E15.5 * Ctrl
10450435	Csnk2b	NM_009975	0.00976362	1.14342	E15.5 * Wnt1 up vs E15.5 * Ctrl
10505107	Actl7a	NM_009611	0.0137147	1.14341	E15.5 * Wnt1 up vs E15.5 * Ctrl
10461991	Zfand5	NM_009551	0.00334685	1.14308	E15.5 * Wnt1 up vs E15.5 * Ctrl
10437594	Usp7	NM_001003918	0.00318519	1.14301	E15.5 * Wnt1 up vs E15.5 * Ctrl
10355844	Dnpep	NM_001110831	0.0143886	1.14284	E15.5 * Wnt1 up vs E15.5 * Ctrl
10547471	B4galnt3	NM_198884	0.0102697	1.14248	E15.5 * Wnt1 up vs E15.5 * Ctrl
10531488	Ccni	NM_017367	0.00979231	1.142	E15.5 * Wnt1 up vs E15.5 * Ctrl
10560091	V1rk1	NM_134227	0.00376802	1.14195	E15.5 * Wnt1 up vs E15.5 * Ctrl
10385686	Hnrnpab	NM_001048061	0.000207344	1.14116	E15.5 * Wnt1 up vs E15.5 * Ctrl
10391750	Gpatch8	NM_001159492	0.00229742	1.14055	E15.5 * Wnt1 up vs E15.5 * Ctrl
10516706	Khdrbs1	NM_011317	0.00454875	1.14045	E15.5 * Wnt1 up vs E15.5 * Ctrl
10360248	Atp1a4	NM_013734	0.0138386	1.14026	E15.5 * Wnt1 up vs E15.5 * Ctrl
10440911		---	0.00598872	1.1401	E15.5 * Wnt1 up vs E15.5 * Ctrl
10485514	Caprin1	NM_016739	6.53165e-005	1.14001	E15.5 * Wnt1 up vs E15.5 * Ctrl
10456120	Csnk1a1	NM_146087	0.000769848	1.14001	E15.5 * Wnt1 up vs E15.5 * Ctrl
10479765	Suv39h2	NM_022724	0.011224	1.13958	E15.5 * Wnt1 up vs E15.5 * Ctrl
10576924	Arglu1	NM_176849	0.000645099	1.13958	E15.5 * Wnt1 up vs E15.5 * Ctrl
10477460		---	0.0133306	1.13919	E15.5 * Wnt1 up vs E15.5 * Ctrl
10517005	Gpr3	NM_008154	0.00695275	1.13909	E15.5 * Wnt1 up vs E15.5 * Ctrl
10492442		---	0.00566648	1.13895	E15.5 * Wnt1 up vs E15.5 * Ctrl
10604473	Igsf1	NM_177591	0.00605003	1.13852	E15.5 * Wnt1 up vs E15.5 * Ctrl
10424573	Khdrbs3	AF079763	0.0125124	1.13804	E15.5 * Wnt1 up vs E15.5 * Ctrl
10509463	Eif4g3	NM_172703	0.00835279	1.13773	E15.5 * Wnt1 up vs E15.5 * Ctrl
10514668	Jak1	NM_146145	0.00161921	1.13744	E15.5 * Wnt1 up vs E15.5 * Ctrl
10526792	0910001L09Rik	NM_001081108	0.0146639	1.13728	E15.5 * Wnt1 up vs E15.5 * Ctrl
10458046	D0H4S114	NM_053078	0.00509196	1.137	E15.5 * Wnt1 up vs E15.5 * Ctrl
10429199		---	0.0147936	1.13668	E15.5 * Wnt1 up vs E15.5 * Ctrl
10350046	Kdm5b	NM_152895	0.00280733	1.13662	E15.5 * Wnt1 up vs E15.5 * Ctrl
10475879	Gm355	ENSMUST00000(	0.00399937	1.13659	E15.5 * Wnt1 up vs E15.5 * Ctrl
10528340	Dnajc2	NM_009584	0.00908819	1.13652	E15.5 * Wnt1 up vs E15.5 * Ctrl
10452867	Dpy30	NM_001146222	0.00220201	1.13651	E15.5 * Wnt1 up vs E15.5 * Ctrl
10551741	Dpf1	NM_013874	0.00953055	1.1361	E15.5 * Wnt1 up vs E15.5 * Ctrl
10408490	Exoc2	NM_025588	0.00394942	1.13607	E15.5 * Wnt1 up vs E15.5 * Ctrl
10564231	Ube2nl	XM_905142	0.00933811	1.13584	E15.5 * Wnt1 up vs E15.5 * Ctrl
10548606	Tas2r107	NM_199154	0.00633349	1.13571	E15.5 * Wnt1 up vs E15.5 * Ctrl
10347491	Wnt6	NM_009526	0.00839205	1.13551	E15.5 * Wnt1 up vs E15.5 * Ctrl
10359201	Ralgps2	NM_023884	0.00269146	1.13546	E15.5 * Wnt1 up vs E15.5 * Ctrl
10425757	1500032L24Rik	BC092006	0.013883	1.13526	E15.5 * Wnt1 up vs E15.5 * Ctrl
10379028		---	0.00651798	1.13511	E15.5 * Wnt1 up vs E15.5 * Ctrl
10485402	Fjx1	NM_010218	0.00719771	1.13479	E15.5 * Wnt1 up vs E15.5 * Ctrl
10520124	Sumo2	NM_133354	0.00610482	1.13447	E15.5 * Wnt1 up vs E15.5 * Ctrl
10374621	Ahsa2	NM_172391	0.00640682	1.13433	E15.5 * Wnt1 up vs E15.5 * Ctrl
10405163	Spin1	NM_146043	0.00135192	1.13426	E15.5 * Wnt1 up vs E15.5 * Ctrl
10537078	Mkln1	NM_013791	0.0112813	1.13416	E15.5 * Wnt1 up vs E15.5 * Ctrl
10361712	Shprh	NM_172937	0.0136792	1.13407	E15.5 * Wnt1 up vs E15.5 * Ctrl
10364718	1600002K03Rik	NM_027207	0.0117339	1.13402	E15.5 * Wnt1 up vs E15.5 * Ctrl
10581311	Acd	NM_001012638	0.0131974	1.13373	E15.5 * Wnt1 up vs E15.5 * Ctrl
10490370	Psma7	NM_011969	3.48531e-005	1.13372	E15.5 * Wnt1 up vs E15.5 * Ctrl
10575021	Zfp90	NM_011764	0.000676264	1.13349	E15.5 * Wnt1 up vs E15.5 * Ctrl
10545212	ENSMUSG0000C	ENSMUST00000	0.00873747	1.13346	E15.5 * Wnt1 up vs E15.5 * Ctrl
10405948	BC048507	NM_001001185	0.0102676	1.13345	E15.5 * Wnt1 up vs E15.5 * Ctrl
10496475	Adh5	NM_007410	0.000211734	1.13339	E15.5 * Wnt1 up vs E15.5 * Ctrl
10394358	BC068281	NM_173416	0.013045	1.13318	E15.5 * Wnt1 up vs E15.5 * Ctrl
10438454	Sumo2	NM_133354	0.000168981	1.13307	E15.5 * Wnt1 up vs E15.5 * Ctrl
10382795	OTTMUSG00000	XR_034731	0.0139803	1.13295	E15.5 * Wnt1 up vs E15.5 * Ctrl
10542445	Strap	NM_011499	0.00189623	1.13213	E15.5 * Wnt1 up vs E15.5 * Ctrl
10353219	Sumo2	NM_133354	0.000601315	1.13157	E15.5 * Wnt1 up vs E15.5 * Ctrl
10525313	Atxn2	NM_009125	0.00144332	1.13133	E15.5 * Wnt1 up vs E15.5 * Ctrl
10532133	Evi5	NM_007964	0.0105021	1.13132	E15.5 * Wnt1 up vs E15.5 * Ctrl
10555425	Fam168a	BC079886	0.00561424	1.13128	E15.5 * Wnt1 up vs E15.5 * Ctrl
10494839	Csde1	NM_144901	0.00328098	1.13127	E15.5 * Wnt1 up vs E15.5 * Ctrl
10426110	Pim3	NM_145478	0.00702715	1.13114	E15.5 * Wnt1 up vs E15.5 * Ctrl

10413270	4933413J09Rik	ENSMUST00000(	0.0147564	1.13071	E15.5 * Wnt1 up vs E15.5 * Ctrl
10589848	Ubp1	NM_001083319	0.00664903	1.13065	E15.5 * Wnt1 up vs E15.5 * Ctrl
10548612	Tas2r105	NM_020501	0.00809945	1.13065	E15.5 * Wnt1 up vs E15.5 * Ctrl
10558257	Gpr26	NM_173410	0.00757999	1.13025	E15.5 * Wnt1 up vs E15.5 * Ctrl
10544976	Kbtbd2	NM_145958	0.0017555	1.13013	E15.5 * Wnt1 up vs E15.5 * Ctrl
10475625	Eid1	NM_025613	0.0058049	1.12975	E15.5 * Wnt1 up vs E15.5 * Ctrl
10493758	Gatad2b	NM_139304	0.00124505	1.12906	E15.5 * Wnt1 up vs E15.5 * Ctrl
10484351	Sumo2	NM_133354	0.000186125	1.12856	E15.5 * Wnt1 up vs E15.5 * Ctrl
10369661	Ccar1	NM_026201	0.0128546	1.12845	E15.5 * Wnt1 up vs E15.5 * Ctrl
10602795	Eif1ay	NM_025437	0.00671847	1.12779	E15.5 * Wnt1 up vs E15.5 * Ctrl
10520025	Psmc2	NM_011188	0.001646	1.12754	E15.5 * Wnt1 up vs E15.5 * Ctrl
10526069	Cct6a	NM_009838	0.00918492	1.12739	E15.5 * Wnt1 up vs E15.5 * Ctrl
10417676	Thoc7	NM_025435	0.0128565	1.12709	E15.5 * Wnt1 up vs E15.5 * Ctrl
10350286	Ddx59	NM_026500	0.0107052	1.12708	E15.5 * Wnt1 up vs E15.5 * Ctrl
10360679	H3f3a	NM_008210	0.000381445	1.12701	E15.5 * Wnt1 up vs E15.5 * Ctrl
10403112	Dnahc11	NM_010060	0.00688142	1.12636	E15.5 * Wnt1 up vs E15.5 * Ctrl
10549873		---	0.0130311	1.12633	E15.5 * Wnt1 up vs E15.5 * Ctrl
10572743	Olfr373	NM_146539	0.00886099	1.12533	E15.5 * Wnt1 up vs E15.5 * Ctrl
10563770	Csrp3	NM_013808	0.00953486	1.12515	E15.5 * Wnt1 up vs E15.5 * Ctrl
10588903	Usp4	NM_011678	0.00938265	1.12487	E15.5 * Wnt1 up vs E15.5 * Ctrl
10478196	Top1	NM_009408	0.00200038	1.12465	E15.5 * Wnt1 up vs E15.5 * Ctrl
10548931	Slc15a5	NM_177787	0.00977852	1.12454	E15.5 * Wnt1 up vs E15.5 * Ctrl
10367477	Olfr774	NM_207620	0.00159517	1.12451	E15.5 * Wnt1 up vs E15.5 * Ctrl
10442134	Vmn2r107	NM_001104569	0.00488891	1.12321	E15.5 * Wnt1 up vs E15.5 * Ctrl
10385557	Cnot6	NM_212484	0.0144735	1.12301	E15.5 * Wnt1 up vs E15.5 * Ctrl
10376263	Mfap3	NM_145426	0.0105759	1.12255	E15.5 * Wnt1 up vs E15.5 * Ctrl
10431371	Hdac10	NM_199198	0.0121315	1.12238	E15.5 * Wnt1 up vs E15.5 * Ctrl
10405539	Txndc15	NM_175150	0.0119926	1.12233	E15.5 * Wnt1 up vs E15.5 * Ctrl
10447431	Foxn2	NM_180974	0.0134366	1.12201	E15.5 * Wnt1 up vs E15.5 * Ctrl
10348004	Psmd1	NM_027357	0.000935208	1.12089	E15.5 * Wnt1 up vs E15.5 * Ctrl
10605919	Pja1	NM_001083110	0.00545207	1.12067	E15.5 * Wnt1 up vs E15.5 * Ctrl
10404464	Serpinb9f	NM_183197	0.00579274	1.12064	E15.5 * Wnt1 up vs E15.5 * Ctrl
10566966	Eif4g2	NM_013507	0.00163152	1.12034	E15.5 * Wnt1 up vs E15.5 * Ctrl
10502537	Sh3glb1	NM_019464	0.00418839	1.11993	E15.5 * Wnt1 up vs E15.5 * Ctrl
10519224	Pusl1	ENSMUST00000(	0.00490014	1.11921	E15.5 * Wnt1 up vs E15.5 * Ctrl
10391990	1700081L11Rik	NM_001081045	0.00762164	1.11894	E15.5 * Wnt1 up vs E15.5 * Ctrl
10411776	H3f3a	NM_008210	1.46466e-005	1.11782	E15.5 * Wnt1 up vs E15.5 * Ctrl
10504056	Ubap1	NM_023305	0.012679	1.1178	E15.5 * Wnt1 up vs E15.5 * Ctrl
10578981		---	0.00950968	1.11758	E15.5 * Wnt1 up vs E15.5 * Ctrl
10551009	Tmsb10	NM_025284	0.000738116	1.11731	E15.5 * Wnt1 up vs E15.5 * Ctrl
10384603	Mdh1	NM_008618	0.010762	1.11707	E15.5 * Wnt1 up vs E15.5 * Ctrl
10395910	Pnn	NM_008891	0.00488861	1.11657	E15.5 * Wnt1 up vs E15.5 * Ctrl
10537805	Olfr447	NM_146988	0.0125284	1.11656	E15.5 * Wnt1 up vs E15.5 * Ctrl
10427885	Hnrnpa1	NM_010447	0.00871294	1.11494	E15.5 * Wnt1 up vs E15.5 * Ctrl
10538318	Tax1bp1	NM_025816	0.0132556	1.11422	E15.5 * Wnt1 up vs E15.5 * Ctrl
10377851	Psmb6	NM_008946	0.00857242	1.11311	E15.5 * Wnt1 up vs E15.5 * Ctrl
10398461	Ppp2r5c	NM_012023	0.00743728	1.11262	E15.5 * Wnt1 up vs E15.5 * Ctrl
10440600	Cct8	NM_009840	0.00456947	1.11225	E15.5 * Wnt1 up vs E15.5 * Ctrl
10473503	Olfr1052	NM_147010	0.0126338	1.11191	E15.5 * Wnt1 up vs E15.5 * Ctrl
10518642	Ube4b	NM_022022	0.0119344	1.11168	E15.5 * Wnt1 up vs E15.5 * Ctrl
10414313	Ubb	NM_011664	3.18724e-006	1.11132	E15.5 * Wnt1 up vs E15.5 * Ctrl
10376864	Ubb	NM_011664	5.1966e-006	1.11111	E15.5 * Wnt1 up vs E15.5 * Ctrl
10392947	Sumo2	NM_133354	0.000584734	1.11104	E15.5 * Wnt1 up vs E15.5 * Ctrl
10380321	Mbtd1	NM_134012	0.013271	1.10965	E15.5 * Wnt1 up vs E15.5 * Ctrl
10461191	Nxf1	NM_016813	0.00746136	1.10884	E15.5 * Wnt1 up vs E15.5 * Ctrl
10369704	Hnrnph3	NM_001079824	0.00830587	1.10879	E15.5 * Wnt1 up vs E15.5 * Ctrl
10586816	Sltm	NM_025690	0.0107044	1.10862	E15.5 * Wnt1 up vs E15.5 * Ctrl
10596769	Rbm5	NM_148930	0.00197061	1.10704	E15.5 * Wnt1 up vs E15.5 * Ctrl
10477073	Csnk2a1	NM_007788	0.0126315	1.10703	E15.5 * Wnt1 up vs E15.5 * Ctrl
10368739	Gtf3c6	NM_026113	0.00219793	1.10632	E15.5 * Wnt1 up vs E15.5 * Ctrl
10470665	Setx	NM_198033	0.00409462	1.10522	E15.5 * Wnt1 up vs E15.5 * Ctrl
10437392	Magmas	NM_025571	0.00285119	1.10512	E15.5 * Wnt1 up vs E15.5 * Ctrl
10451110	Hsp90ab1	NM_008302	0.00125647	1.10453	E15.5 * Wnt1 up vs E15.5 * Ctrl
10577352	Defb12	NM_152802	0.00765294	1.10446	E15.5 * Wnt1 up vs E15.5 * Ctrl
10580370	Dnaja2	NM_019794	0.00207959	1.10415	E15.5 * Wnt1 up vs E15.5 * Ctrl
10597020	Fbxw14	NM_015793	0.00671246	1.10411	E15.5 * Wnt1 up vs E15.5 * Ctrl

10408947	ENSMUSG0000C	ENSMUST00000	0.00745757	1.10406	E15.5 * Wnt1 up vs E15.5 * Ctrl
10601857	Ngfrap1	NM_009750	0.000579284	1.10357	E15.5 * Wnt1 up vs E15.5 * Ctrl
10399379	Pgk1	NM_008828	0.00294038	1.10279	E15.5 * Wnt1 up vs E15.5 * Ctrl
10498313	Pgk1	NM_008828	0.00294038	1.10279	E15.5 * Wnt1 up vs E15.5 * Ctrl
10516859	Ythdf2	NM_145393	0.00528258	1.10231	E15.5 * Wnt1 up vs E15.5 * Ctrl
10365384	Rfx4	NM_001024918	0.0126694	1.10109	E15.5 * Wnt1 up vs E15.5 * Ctrl
10545479	Tmsb10	NM_025284	0.000371765	1.10009	E15.5 * Wnt1 up vs E15.5 * Ctrl
10588547	Vprbp	NM_001015507	0.00911085	1.09903	E15.5 * Wnt1 up vs E15.5 * Ctrl
10508217	Sfpq	NM_023603	0.000271429	1.09894	E15.5 * Wnt1 up vs E15.5 * Ctrl
10601390	Pgk1	NM_008828	0.00501627	1.09725	E15.5 * Wnt1 up vs E15.5 * Ctrl
10407809	Gpr137b	ENSMUST00000	0.00623385	1.09524	E15.5 * Wnt1 up vs E15.5 * Ctrl
10528021	Hspa8	NM_031165	0.00856769	1.09516	E15.5 * Wnt1 up vs E15.5 * Ctrl
10522716	Polr2b	NM_153798	0.00797204	1.09017	E15.5 * Wnt1 up vs E15.5 * Ctrl
10393395	Sfrs2	NM_011358	0.00698708	1.08923	E15.5 * Wnt1 up vs E15.5 * Ctrl
10507011	Dmrta2	NM_172296	0.00840205	1.08769	E15.5 * Wnt1 up vs E15.5 * Ctrl
10432527	Lass5	NM_028015	0.000165169	1.08459	E15.5 * Wnt1 up vs E15.5 * Ctrl
10386850	Ncor1	NM_011308	0.0132276	1.0817	E15.5 * Wnt1 up vs E15.5 * Ctrl
10452415	Gapdh	M32599	0.0148027	1.08036	E15.5 * Wnt1 up vs E15.5 * Ctrl
10465649	Mark2	NM_007928	0.00455004	1.0743	E15.5 * Wnt1 up vs E15.5 * Ctrl
10555055	Ndufc2	NM_024220	0.00720302	1.07396	E15.5 * Wnt1 up vs E15.5 * Ctrl
10375735	Hnrnph1	NM_021510	0.00394302	1.07334	E15.5 * Wnt1 up vs E15.5 * Ctrl
10447699	6530411M01Rik	ENSMUST00000	0.00758495	1.07237	E15.5 * Wnt1 up vs E15.5 * Ctrl
10422161	Gapdh	M32599	0.00282665	1.07171	E15.5 * Wnt1 up vs E15.5 * Ctrl
10466139	1700017D01Rik	NM_027058	0.00887871	1.06754	E15.5 * Wnt1 up vs E15.5 * Ctrl
10496379	H2afz	NM_016750	0.0137902	1.0659	E15.5 * Wnt1 up vs E15.5 * Ctrl
10380210	Sfrs1	NM_173374	0.00828544	1.06435	E15.5 * Wnt1 up vs E15.5 * Ctrl
10503150	Rab2a	NM_021518	0.0116521	1.0607	E15.5 * Wnt1 up vs E15.5 * Ctrl
10579769	Gapdh	M32599	0.00821726	1.06038	E15.5 * Wnt1 up vs E15.5 * Ctrl
10548143	Gapdh	BC092267	0.0067573	1.05834	E15.5 * Wnt1 up vs E15.5 * Ctrl
10528167	Gapdh	NM_008084	0.007999	1.05692	E15.5 * Wnt1 up vs E15.5 * Ctrl
10427241	Pcbp2	NM_001103165	0.00889457	1.05419	E15.5 * Wnt1 up vs E15.5 * Ctrl
10489049	Rpl9	NM_011292	0.00116514	-1.05024	E15.5 * Wnt1 down vs E15.5 * Ctrl
10565582	Rps8	NM_009098	0.0139018	-1.05224	E15.5 * Wnt1 down vs E15.5 * Ctrl
10561138	Rpl37a	NM_009084	0.0134441	-1.05242	E15.5 * Wnt1 down vs E15.5 * Ctrl
10513195	Txn1	NM_011660	0.0135834	-1.05985	E15.5 * Wnt1 down vs E15.5 * Ctrl
10472686	Rpl9	NM_011292	0.00476168	-1.06242	E15.5 * Wnt1 down vs E15.5 * Ctrl
10423228	5730427N09Rik	NM_021552	0.00666565	-1.06325	E15.5 * Wnt1 down vs E15.5 * Ctrl
10355173	Rpl10a	NM_011287	0.00241499	-1.06399	E15.5 * Wnt1 down vs E15.5 * Ctrl
10443360	Rpl10a	NM_011287	0.00481423	-1.06559	E15.5 * Wnt1 down vs E15.5 * Ctrl
10389162	Rpl12	NM_009076	0.00583442	-1.06622	E15.5 * Wnt1 down vs E15.5 * Ctrl
10449549	Rpl35a	BC090255	0.00543737	-1.06647	E15.5 * Wnt1 down vs E15.5 * Ctrl
10485685	Rpl35a	BC090255	0.00543737	-1.06647	E15.5 * Wnt1 down vs E15.5 * Ctrl
10533626	Rpl35a	BC090255	0.00543737	-1.06647	E15.5 * Wnt1 down vs E15.5 * Ctrl
10538080	Rpl35a	BC090255	0.00543737	-1.06647	E15.5 * Wnt1 down vs E15.5 * Ctrl
10437627	Rpl35a	BC090255	0.00299887	-1.067	E15.5 * Wnt1 down vs E15.5 * Ctrl
10521036	Rpl35a	BC090255	0.0110219	-1.06774	E15.5 * Wnt1 down vs E15.5 * Ctrl
10410506	Rpl9	NM_011292	0.000906852	-1.06779	E15.5 * Wnt1 down vs E15.5 * Ctrl
10515154	Rpl3	NM_013762	0.00701339	-1.06885	E15.5 * Wnt1 down vs E15.5 * Ctrl
10479362	Rps21	NM_025587	0.00745092	-1.06936	E15.5 * Wnt1 down vs E15.5 * Ctrl
10538629	Rps15	NM_009091	0.0145587	-1.07042	E15.5 * Wnt1 down vs E15.5 * Ctrl
10530194	Rpl9	NM_011292	0.000935457	-1.07159	E15.5 * Wnt1 down vs E15.5 * Ctrl
10576246	Rps12	NM_011295	0.00657696	-1.0723	E15.5 * Wnt1 down vs E15.5 * Ctrl
10416925		---	0.00187151	-1.07431	E15.5 * Wnt1 down vs E15.5 * Ctrl
10439402	Rpl9	NM_011292	0.00225221	-1.07467	E15.5 * Wnt1 down vs E15.5 * Ctrl
10409968	Rps18	NM_011296	0.000967167	-1.07549	E15.5 * Wnt1 down vs E15.5 * Ctrl
10594320	ENSMUSG0000C	NR_003965	0.00681532	-1.07675	E15.5 * Wnt1 down vs E15.5 * Ctrl
10435187	Rpl35a	NM_021338	0.00924367	-1.07693	E15.5 * Wnt1 down vs E15.5 * Ctrl
10360737	Rpl35a	NM_021338	0.00854087	-1.07844	E15.5 * Wnt1 down vs E15.5 * Ctrl
10405890	Rpl9	NM_011292	0.000754154	-1.07937	E15.5 * Wnt1 down vs E15.5 * Ctrl
10404354	Rps18	NM_011296	0.000151635	-1.07968	E15.5 * Wnt1 down vs E15.5 * Ctrl
10581535	Rps18	NM_011296	4.27727e-005	-1.08314	E15.5 * Wnt1 down vs E15.5 * Ctrl
10368504	Rpl12	NM_009076	0.00291135	-1.08519	E15.5 * Wnt1 down vs E15.5 * Ctrl
10380297	Cox11	NM_199008	0.0142372	-1.08537	E15.5 * Wnt1 down vs E15.5 * Ctrl
10556200	Rpl27a	NM_011975	0.0130363	-1.08667	E15.5 * Wnt1 down vs E15.5 * Ctrl
10419136	Cdv3	NM_175565	0.00388084	-1.08691	E15.5 * Wnt1 down vs E15.5 * Ctrl
10488231	EG620248	ENSMUST00000	0.0128511	-1.08727	E15.5 * Wnt1 down vs E15.5 * Ctrl

10464388	Rps12	NM_011295	0.00318655	-1.08731	E15.5 * Wnt1 down vs E15.5 * Ctrl
10561956	Rps12	NM_011295	0.00328465	-1.08754	E15.5 * Wnt1 down vs E15.5 * Ctrl
10352514	Eprs	NM_029735	0.00690211	-1.08795	E15.5 * Wnt1 down vs E15.5 * Ctrl
10496032	Rpl12	NM_009076	0.00107297	-1.08853	E15.5 * Wnt1 down vs E15.5 * Ctrl
10545471	Kcmf1	NM_019715	0.000730075	-1.08898	E15.5 * Wnt1 down vs E15.5 * Ctrl
10411393	Rps18	NM_011296	9.54736e-005	-1.0897	E15.5 * Wnt1 down vs E15.5 * Ctrl
10489186	Rpl35a	NM_021338	0.0111599	-1.09072	E15.5 * Wnt1 down vs E15.5 * Ctrl
10450063	Rps18	NM_011296	2.29828e-005	-1.09102	E15.5 * Wnt1 down vs E15.5 * Ctrl
10368270	Rps12	NM_011295	0.00638303	-1.09208	E15.5 * Wnt1 down vs E15.5 * Ctrl
10550601	Snrpd2	NM_026943	0.0124672	-1.09215	E15.5 * Wnt1 down vs E15.5 * Ctrl
10438376	EG620248	ENSMUST00000(	0.0101446	-1.09216	E15.5 * Wnt1 down vs E15.5 * Ctrl
10409424	Mxd3	NM_016662	0.00573834	-1.09353	E15.5 * Wnt1 down vs E15.5 * Ctrl
10516209	Rps2	NM_008503	0.0136648	-1.09354	E15.5 * Wnt1 down vs E15.5 * Ctrl
10380732	Mrpl10	NM_026154	0.00685518	-1.09385	E15.5 * Wnt1 down vs E15.5 * Ctrl
10600324	Rpl3	NM_013762	0.000867283	-1.09444	E15.5 * Wnt1 down vs E15.5 * Ctrl
10515383	Rpl36	NM_018730	0.00433154	-1.09539	E15.5 * Wnt1 down vs E15.5 * Ctrl
10513878	Rps18	NM_011296	0.0010755	-1.09596	E15.5 * Wnt1 down vs E15.5 * Ctrl
10591773	Hmgn2	NM_016957	0.00305633	-1.09654	E15.5 * Wnt1 down vs E15.5 * Ctrl
10556764	Ppp1cc	NM_013636	0.00464427	-1.09665	E15.5 * Wnt1 down vs E15.5 * Ctrl
10390352	Kpnb1	NM_008379	0.00989299	-1.09693	E15.5 * Wnt1 down vs E15.5 * Ctrl
10503723	Mdn1	NM_001081392	0.0107853	-1.09829	E15.5 * Wnt1 down vs E15.5 * Ctrl
10525352	Ppp1cc	NM_013636	0.00295343	-1.09863	E15.5 * Wnt1 down vs E15.5 * Ctrl
10568417	1110007A13Rik	BC038342	0.0111104	-1.09889	E15.5 * Wnt1 down vs E15.5 * Ctrl
10372750	Cand1	NM_027994	0.00712536	-1.09921	E15.5 * Wnt1 down vs E15.5 * Ctrl
10467256	Rpl10	NM_052835	0.0013979	-1.10312	E15.5 * Wnt1 down vs E15.5 * Ctrl
10411363	5730427N09Rik	NM_021552	0.000113757	-1.10418	E15.5 * Wnt1 down vs E15.5 * Ctrl
10593320	Rpl10	NM_052835	0.000533632	-1.1049	E15.5 * Wnt1 down vs E15.5 * Ctrl
10466342	Olfr1453	NM_146700	0.0106474	-1.10504	E15.5 * Wnt1 down vs E15.5 * Ctrl
10526319	Baz1b	NM_011714	0.00146062	-1.10813	E15.5 * Wnt1 down vs E15.5 * Ctrl
10368670	Amd1	NM_009665	0.00868468	-1.10882	E15.5 * Wnt1 down vs E15.5 * Ctrl
10352756	Lpgat1	NM_001134829	0.00894357	-1.1094	E15.5 * Wnt1 down vs E15.5 * Ctrl
10579659	Hmgn2	NM_016957	0.00160634	-1.11013	E15.5 * Wnt1 down vs E15.5 * Ctrl
10517141	Hmgn2	NM_016957	0.000987177	-1.11021	E15.5 * Wnt1 down vs E15.5 * Ctrl
10551489	Rps16	NM_013647	0.0107973	-1.11077	E15.5 * Wnt1 down vs E15.5 * Ctrl
10471358	Rpl36	NM_018730	0.00117628	-1.11079	E15.5 * Wnt1 down vs E15.5 * Ctrl
10437222	Hnrnpa3	NM_053263	0.00501459	-1.11102	E15.5 * Wnt1 down vs E15.5 * Ctrl
10543895	2010107G12Rik	BC075651	0.00875208	-1.11111	E15.5 * Wnt1 down vs E15.5 * Ctrl
10552740	Nup62	NM_053074	0.00777144	-1.11171	E15.5 * Wnt1 down vs E15.5 * Ctrl
10528200	Hnrnpa3	ENSMUST00000	0.00380456	-1.11177	E15.5 * Wnt1 down vs E15.5 * Ctrl
10594946	Rpl36	NM_018730	0.00076325	-1.11258	E15.5 * Wnt1 down vs E15.5 * Ctrl
10349478	Rpl28	NM_009081	0.00314087	-1.11266	E15.5 * Wnt1 down vs E15.5 * Ctrl
10541581	Clec4b1	NM_027218	0.00543473	-1.11317	E15.5 * Wnt1 down vs E15.5 * Ctrl
10367073	Snrpd2	NM_026943	0.0107699	-1.11333	E15.5 * Wnt1 down vs E15.5 * Ctrl
10515493	Rhoa	NM_016802	0.011348	-1.11346	E15.5 * Wnt1 down vs E15.5 * Ctrl
10347297	Arpc2	NM_029711	0.00430571	-1.11386	E15.5 * Wnt1 down vs E15.5 * Ctrl
10600349	Rpl10	NM_052835	0.000636918	-1.11391	E15.5 * Wnt1 down vs E15.5 * Ctrl
10537246	Nup205	NM_027513	0.00704879	-1.11412	E15.5 * Wnt1 down vs E15.5 * Ctrl
10416940	Tpm3	ENSMUST00000(	0.000853088	-1.11431	E15.5 * Wnt1 down vs E15.5 * Ctrl
10378783	Rpl36	NM_018730	0.000715974	-1.11447	E15.5 * Wnt1 down vs E15.5 * Ctrl
10499716	Ubap2l	NM_028475	0.00423482	-1.11482	E15.5 * Wnt1 down vs E15.5 * Ctrl
10538963	Rpl34	NM_026724	0.000986604	-1.11508	E15.5 * Wnt1 down vs E15.5 * Ctrl
10458823		---	0.00223642	-1.11569	E15.5 * Wnt1 down vs E15.5 * Ctrl
10403108	Hmgn2	NM_016957	0.0106204	-1.11585	E15.5 * Wnt1 down vs E15.5 * Ctrl
10430105	C030006K11Rik	NM_145472	0.00798999	-1.1159	E15.5 * Wnt1 down vs E15.5 * Ctrl
10485654	Rpl10	NM_052835	0.000859463	-1.11617	E15.5 * Wnt1 down vs E15.5 * Ctrl
10413333	Dnahc12	Z83811	0.0137876	-1.11654	E15.5 * Wnt1 down vs E15.5 * Ctrl
10546801	Rpl36	NM_018730	0.00022257	-1.11704	E15.5 * Wnt1 down vs E15.5 * Ctrl
10549714	Rpl28	NM_009081	0.00189304	-1.11745	E15.5 * Wnt1 down vs E15.5 * Ctrl
10449370	Rps10	NM_025963	0.00925919	-1.11765	E15.5 * Wnt1 down vs E15.5 * Ctrl
10439766	Pvrl3	NM_021496	0.00752185	-1.11805	E15.5 * Wnt1 down vs E15.5 * Ctrl
10514173	Rpl34	NM_026724	0.000565016	-1.11845	E15.5 * Wnt1 down vs E15.5 * Ctrl
10527832	Pds5b	NM_175310	0.00510625	-1.11863	E15.5 * Wnt1 down vs E15.5 * Ctrl
10535927	Nme2	NM_008705	0.00100146	-1.11865	E15.5 * Wnt1 down vs E15.5 * Ctrl
10494296	Rps10	NM_025963	0.00945613	-1.11919	E15.5 * Wnt1 down vs E15.5 * Ctrl
10584276	Sec61g	NM_011343	0.00935109	-1.11936	E15.5 * Wnt1 down vs E15.5 * Ctrl
10484402	Ctnnd1	NM_007615	0.0023991	-1.12005	E15.5 * Wnt1 down vs E15.5 * Ctrl

10518329	Rpl28	NM_009081	0.00733473	-1.1204	E15.5 * Wnt1 down vs E15.5 * Ctrl
10565072	Sec11a	NM_019951	0.00985371	-1.12166	E15.5 * Wnt1 down vs E15.5 * Ctrl
10590648	Top2a	NM_011623	0.0146766	-1.12247	E15.5 * Wnt1 down vs E15.5 * Ctrl
10499655	Il6ra	NM_010559	0.0139276	-1.12248	E15.5 * Wnt1 down vs E15.5 * Ctrl
10446131	Rpl36	NM_018730	0.000432556	-1.12333	E15.5 * Wnt1 down vs E15.5 * Ctrl
10556350	Usp47	NM_133758	0.0073783	-1.12369	E15.5 * Wnt1 down vs E15.5 * Ctrl
10566662	Olfr469	NM_146426	0.00452987	-1.12373	E15.5 * Wnt1 down vs E15.5 * Ctrl
10462081	5730427N09Rik	NM_021552	0.000811824	-1.12386	E15.5 * Wnt1 down vs E15.5 * Ctrl
10428912		---	0.00215059	-1.1242	E15.5 * Wnt1 down vs E15.5 * Ctrl
10574694	Elmo3	NM_172760	0.000989688	-1.12485	E15.5 * Wnt1 down vs E15.5 * Ctrl
10426648	Rpl36	NM_018730	0.000194442	-1.12513	E15.5 * Wnt1 down vs E15.5 * Ctrl
10457223	Cdh11	NM_009866	0.00417716	-1.1254	E15.5 * Wnt1 down vs E15.5 * Ctrl
10472587	Rpl13	NM_016738	0.00298624	-1.12661	E15.5 * Wnt1 down vs E15.5 * Ctrl
10384448	Sec61g	NM_011343	0.00147718	-1.12669	E15.5 * Wnt1 down vs E15.5 * Ctrl
10405971	Nsun2	NM_145354	0.00531277	-1.12692	E15.5 * Wnt1 down vs E15.5 * Ctrl
10565846	Spcs2	NM_025668	0.0133444	-1.12692	E15.5 * Wnt1 down vs E15.5 * Ctrl
10477604	Itch	NM_008395	0.0131513	-1.12725	E15.5 * Wnt1 down vs E15.5 * Ctrl
10528177	ENSMUSG0000C	NM_198666	0.0119009	-1.12735	E15.5 * Wnt1 down vs E15.5 * Ctrl
10563527	Ush1c	NM_153677	0.00914524	-1.12776	E15.5 * Wnt1 down vs E15.5 * Ctrl
10526179	Rpl28	NM_009081	0.00187485	-1.12776	E15.5 * Wnt1 down vs E15.5 * Ctrl
10592138	Rpl36	NM_018730	0.00117103	-1.12816	E15.5 * Wnt1 down vs E15.5 * Ctrl
10542129	Clec2g	NM_027562	0.00833086	-1.12873	E15.5 * Wnt1 down vs E15.5 * Ctrl
10394609	Rpl36	NM_018730	0.00054596	-1.12918	E15.5 * Wnt1 down vs E15.5 * Ctrl
10371482	Hsp90b1	NM_011631	0.00958919	-1.12924	E15.5 * Wnt1 down vs E15.5 * Ctrl
10513381	Rod1	NM_144904	0.00240077	-1.12994	E15.5 * Wnt1 down vs E15.5 * Ctrl
10606858	Tceal8	NM_025703	0.0136614	-1.13003	E15.5 * Wnt1 down vs E15.5 * Ctrl
10375234	Nudcd2	NM_026023	0.00428198	-1.1307	E15.5 * Wnt1 down vs E15.5 * Ctrl
10606195	Rlim	NM_011276	0.00805897	-1.13087	E15.5 * Wnt1 down vs E15.5 * Ctrl
10486203	Ino80	NM_026574	0.00423446	-1.13116	E15.5 * Wnt1 down vs E15.5 * Ctrl
10497964	Pgrmc2	NM_027558	0.002429	-1.13161	E15.5 * Wnt1 down vs E15.5 * Ctrl
10397666	Zc3h14	NM_029334	0.00728488	-1.1319	E15.5 * Wnt1 down vs E15.5 * Ctrl
10602840	Sh3kbp1	NM_021389	0.00152368	-1.13217	E15.5 * Wnt1 down vs E15.5 * Ctrl
10428192	Pabpc1	NM_008774	0.000977309	-1.13226	E15.5 * Wnt1 down vs E15.5 * Ctrl
10421456	Xpo7	NM_023045	0.00109433	-1.13253	E15.5 * Wnt1 down vs E15.5 * Ctrl
10583485	A230050P20Rik	NM_175687	0.00895958	-1.1326	E15.5 * Wnt1 down vs E15.5 * Ctrl
10524018	Rpl34	NM_001005859	0.0011857	-1.13287	E15.5 * Wnt1 down vs E15.5 * Ctrl
10597960	Slc6a20a	NM_139142	0.00745439	-1.133	E15.5 * Wnt1 down vs E15.5 * Ctrl
10597920	Zdhhc3	NM_026917	0.00785575	-1.13375	E15.5 * Wnt1 down vs E15.5 * Ctrl
10405094	Iars	NM_172015	0.00637549	-1.13384	E15.5 * Wnt1 down vs E15.5 * Ctrl
10487629	Idh3b	NM_130884	0.00401589	-1.13424	E15.5 * Wnt1 down vs E15.5 * Ctrl
10490203	Gnas	NM_010309	0.00607947	-1.13488	E15.5 * Wnt1 down vs E15.5 * Ctrl
10596265		---	0.00834307	-1.13549	E15.5 * Wnt1 down vs E15.5 * Ctrl
10444747		---	0.0145382	-1.13549	E15.5 * Wnt1 down vs E15.5 * Ctrl
10565591	OTTMUSG00000	ENSMUST00000(	0.0130474	-1.13679	E15.5 * Wnt1 down vs E15.5 * Ctrl
10523547	Agpat9	NM_172715	0.00408264	-1.13729	E15.5 * Wnt1 down vs E15.5 * Ctrl
10477325	Dnmt3b	NM_001003961	0.0117432	-1.13762	E15.5 * Wnt1 down vs E15.5 * Ctrl
10576210	Rpl13	NM_016738	0.00424789	-1.13817	E15.5 * Wnt1 down vs E15.5 * Ctrl
10439237	Rps21	NM_025587	0.0143967	-1.13819	E15.5 * Wnt1 down vs E15.5 * Ctrl
10474399	Bdnf	NM_001048139	0.00706641	-1.13845	E15.5 * Wnt1 down vs E15.5 * Ctrl
10518967	1190007F08Rik	ENSMUST00000(	0.0135998	-1.13892	E15.5 * Wnt1 down vs E15.5 * Ctrl
10530434	Commd8	NM_178599	0.0119887	-1.13938	E15.5 * Wnt1 down vs E15.5 * Ctrl
10403951	Hist1h2bp	NM_178202	0.00883712	-1.13965	E15.5 * Wnt1 down vs E15.5 * Ctrl
10577412	6820431F20Rik	BC025151	0.0064404	-1.14002	E15.5 * Wnt1 down vs E15.5 * Ctrl
10429972	Cpsf1	NM_053193	0.00631608	-1.14005	E15.5 * Wnt1 down vs E15.5 * Ctrl
10520952	Ppp1cb	NM_172707	0.000165156	-1.14017	E15.5 * Wnt1 down vs E15.5 * Ctrl
10606746	Nxf2	NM_031259	0.0096454	-1.14084	E15.5 * Wnt1 down vs E15.5 * Ctrl
10577070	Tubgcp3	NM_198031	0.0105157	-1.14098	E15.5 * Wnt1 down vs E15.5 * Ctrl
10434523	Polr2h	NM_145632	0.00975296	-1.14199	E15.5 * Wnt1 down vs E15.5 * Ctrl
10419471	Tep1	NM_009351	0.0059496	-1.14218	E15.5 * Wnt1 down vs E15.5 * Ctrl
10506736	Magoh	NM_010760	0.0147141	-1.14232	E15.5 * Wnt1 down vs E15.5 * Ctrl
10344741	Hnrnpa3	NM_053263	0.00126726	-1.14263	E15.5 * Wnt1 down vs E15.5 * Ctrl
10515113	Hnrnpa3	NM_053263	0.00126726	-1.14263	E15.5 * Wnt1 down vs E15.5 * Ctrl
10405234	Snrpd2	NM_026943	0.00404294	-1.14308	E15.5 * Wnt1 down vs E15.5 * Ctrl
10479112	Gnas	NM_022000	0.00372795	-1.14362	E15.5 * Wnt1 down vs E15.5 * Ctrl
10562368	4931406P16Rik	NM_172741	0.0132224	-1.14377	E15.5 * Wnt1 down vs E15.5 * Ctrl
10435832	Gm608	NM_001029889	0.00734757	-1.14388	E15.5 * Wnt1 down vs E15.5 * Ctrl

10605729	Zfx	NM_001044386	0.000612388	-1.14433	E15.5 * Wnt1 down vs E15.5 * Ctrl
10503249	Rpl13	NM_016738	0.00407072	-1.14447	E15.5 * Wnt1 down vs E15.5 * Ctrl
10581505	Rpl10	NM_052835	0.00101963	-1.14448	E15.5 * Wnt1 down vs E15.5 * Ctrl
10563362	Sphk2	NM_020011	0.00633756	-1.14455	E15.5 * Wnt1 down vs E15.5 * Ctrl
10600150	Zfp275	NM_031494	0.00914554	-1.14463	E15.5 * Wnt1 down vs E15.5 * Ctrl
10432540	Lima1	NM_001113545	0.00478819	-1.14487	E15.5 * Wnt1 down vs E15.5 * Ctrl
10384192	Tbrg4	NM_134011	0.00737332	-1.14694	E15.5 * Wnt1 down vs E15.5 * Ctrl
10547820	Lrrc23	NM_013588	0.0141324	-1.14821	E15.5 * Wnt1 down vs E15.5 * Ctrl
10424250	Snrpd2	NM_026943	0.00288456	-1.1483	E15.5 * Wnt1 down vs E15.5 * Ctrl
10550501	Mill2	NM_153761	0.0102403	-1.1484	E15.5 * Wnt1 down vs E15.5 * Ctrl
10439790	Trat1	NM_198297	0.00729991	-1.14879	E15.5 * Wnt1 down vs E15.5 * Ctrl
10596379	Rpl10	NM_052835	0.000202307	-1.14919	E15.5 * Wnt1 down vs E15.5 * Ctrl
10548859	Wbp11	NM_021714	0.0119948	-1.1493	E15.5 * Wnt1 down vs E15.5 * Ctrl
10461402	Fth1	NM_010239	1.58537e-005	-1.14934	E15.5 * Wnt1 down vs E15.5 * Ctrl
10567229	Smg1	NM_001031814	0.00519371	-1.14955	E15.5 * Wnt1 down vs E15.5 * Ctrl
10363265	Lims1	NM_026148	0.0046334	-1.14966	E15.5 * Wnt1 down vs E15.5 * Ctrl
10518019	Ddi2	NM_001017966	7.33819e-005	-1.1499	E15.5 * Wnt1 down vs E15.5 * Ctrl
10528840	Paxip1	NM_018878	0.009251	-1.15019	E15.5 * Wnt1 down vs E15.5 * Ctrl
10414113	Wapal	NM_001004436	0.00418654	-1.15019	E15.5 * Wnt1 down vs E15.5 * Ctrl
10516640	Eif3i	NM_018799	0.0120786	-1.15031	E15.5 * Wnt1 down vs E15.5 * Ctrl
10403664	B3galnt2	NM_178640	0.00877235	-1.15077	E15.5 * Wnt1 down vs E15.5 * Ctrl
10558914	Rplp2	NM_026020	0.0138372	-1.15082	E15.5 * Wnt1 down vs E15.5 * Ctrl
10608263	Sly	BC049626	0.0118005	-1.15109	E15.5 * Wnt1 down vs E15.5 * Ctrl
10603708	Cask	NM_009806	0.00254858	-1.15169	E15.5 * Wnt1 down vs E15.5 * Ctrl
10496946	Zzz3	NM_198416	0.00742782	-1.15229	E15.5 * Wnt1 down vs E15.5 * Ctrl
10446693	Wdr43	AK129031	0.00213994	-1.15234	E15.5 * Wnt1 down vs E15.5 * Ctrl
10557587	Zfp771	NM_177362	0.00468428	-1.15263	E15.5 * Wnt1 down vs E15.5 * Ctrl
10371888	Tmpo	NM_001080129	0.0134863	-1.15347	E15.5 * Wnt1 down vs E15.5 * Ctrl
10382565	Mrps7	NM_025305	0.00412573	-1.15358	E15.5 * Wnt1 down vs E15.5 * Ctrl
10493052	Insrr	NM_011832	0.0144639	-1.15365	E15.5 * Wnt1 down vs E15.5 * Ctrl
10521174	Rnf4	NM_011278	0.00814125	-1.1539	E15.5 * Wnt1 down vs E15.5 * Ctrl
10542050	Efcab4b	NM_001033464	0.012538	-1.15448	E15.5 * Wnt1 down vs E15.5 * Ctrl
10495794	Pde5a	NM_153422	0.0026069	-1.15511	E15.5 * Wnt1 down vs E15.5 * Ctrl
10371002	Lsm7	NM_025349	0.0131125	-1.15522	E15.5 * Wnt1 down vs E15.5 * Ctrl
10594835	Grinl1a	NM_178602	0.00213884	-1.15537	E15.5 * Wnt1 down vs E15.5 * Ctrl
10430811	Nhp2l1	NM_011482	0.00913824	-1.15573	E15.5 * Wnt1 down vs E15.5 * Ctrl
10441919	1600012H06Rik	NM_001083881	0.0128436	-1.15625	E15.5 * Wnt1 down vs E15.5 * Ctrl
10561552	Eif3k	NM_028659	0.00929847	-1.15654	E15.5 * Wnt1 down vs E15.5 * Ctrl
10390956	Krtap4-16	NM_001013823	0.0114281	-1.15685	E15.5 * Wnt1 down vs E15.5 * Ctrl
10421981	Dis3	NM_028315	0.00838334	-1.15704	E15.5 * Wnt1 down vs E15.5 * Ctrl
10585091	Nhp2l1	NM_011482	0.0109708	-1.1573	E15.5 * Wnt1 down vs E15.5 * Ctrl
10401637	Nek9	NM_145138	0.000231048	-1.15738	E15.5 * Wnt1 down vs E15.5 * Ctrl
10376230	G3bp1	NM_013716	0.00582377	-1.15746	E15.5 * Wnt1 down vs E15.5 * Ctrl
10461108	Iscu	NM_025526	0.00361596	-1.15882	E15.5 * Wnt1 down vs E15.5 * Ctrl
10444800	Nhp2l1	NM_011482	0.0106582	-1.16021	E15.5 * Wnt1 down vs E15.5 * Ctrl
10419198	Ero1l	NM_015774	0.00627169	-1.16057	E15.5 * Wnt1 down vs E15.5 * Ctrl
10564377	Lrrk1	NM_146191	0.0147516	-1.16068	E15.5 * Wnt1 down vs E15.5 * Ctrl
10597575	Plcd1	NM_019676	0.0133987	-1.16069	E15.5 * Wnt1 down vs E15.5 * Ctrl
10505734	Adamtsl1	NM_029967	0.0104274	-1.16082	E15.5 * Wnt1 down vs E15.5 * Ctrl
10604340		---	0.0132164	-1.16111	E15.5 * Wnt1 down vs E15.5 * Ctrl
10529330	Whsc2	NM_011914	0.0145476	-1.16117	E15.5 * Wnt1 down vs E15.5 * Ctrl
10360985	Cenpf	NM_001081363	0.0126095	-1.16173	E15.5 * Wnt1 down vs E15.5 * Ctrl
10480064	Fbxo18	NM_015792	0.000806914	-1.16203	E15.5 * Wnt1 down vs E15.5 * Ctrl
10353878	Ankrd23	NM_153502	0.00410173	-1.16291	E15.5 * Wnt1 down vs E15.5 * Ctrl
10361007	Smyd2	NM_026796	0.00477562	-1.16359	E15.5 * Wnt1 down vs E15.5 * Ctrl
10394392	Nfyc	NM_008692	0.0074999	-1.16373	E15.5 * Wnt1 down vs E15.5 * Ctrl
10415660	Rpl13	NM_016738	0.00391163	-1.16391	E15.5 * Wnt1 down vs E15.5 * Ctrl
10489961	Nfatc2	NM_010899	0.000561133	-1.16425	E15.5 * Wnt1 down vs E15.5 * Ctrl
10585301	Rdx	NM_009041	0.0119811	-1.16456	E15.5 * Wnt1 down vs E15.5 * Ctrl
10434675	Dnajb11	NM_026400	0.00948006	-1.16466	E15.5 * Wnt1 down vs E15.5 * Ctrl
10596263		---	0.00975188	-1.16498	E15.5 * Wnt1 down vs E15.5 * Ctrl
10493816	S100a5	NM_011312	0.0112749	-1.16511	E15.5 * Wnt1 down vs E15.5 * Ctrl
10481900	Rabepk	NM_145522	0.00266085	-1.16545	E15.5 * Wnt1 down vs E15.5 * Ctrl
10588007	Tfdp2	NM_178667	0.000290284	-1.16555	E15.5 * Wnt1 down vs E15.5 * Ctrl
10522445	Spata18	NM_178387	0.00102425	-1.16556	E15.5 * Wnt1 down vs E15.5 * Ctrl
10420596	Tnfrsf19	NM_013869	0.00735764	-1.16585	E15.5 * Wnt1 down vs E15.5 * Ctrl

10473414	Ssrp1	NM_182990	0.00443582	-1.16592	E15.5 * Wnt1 down vs E15.5 * Ctrl
10404578	Cdyl	NM_009881	0.0120796	-1.16663	E15.5 * Wnt1 down vs E15.5 * Ctrl
10496629	15-Sep	NM_053102	0.00141813	-1.16669	E15.5 * Wnt1 down vs E15.5 * Ctrl
10410124	Ctsl	NM_009984	0.002375	-1.16684	E15.5 * Wnt1 down vs E15.5 * Ctrl
10541482	Nhp2l1	NM_011482	0.0121739	-1.1669	E15.5 * Wnt1 down vs E15.5 * Ctrl
10444068	Tapbp	NM_001025313	0.012178	-1.16712	E15.5 * Wnt1 down vs E15.5 * Ctrl
10430006	Slc39a4	NM_028064	0.0067048	-1.16724	E15.5 * Wnt1 down vs E15.5 * Ctrl
10598369	Prickle3	NM_175097	0.00300698	-1.16725	E15.5 * Wnt1 down vs E15.5 * Ctrl
10398173	Vrk1	NM_011705	0.00236891	-1.16768	E15.5 * Wnt1 down vs E15.5 * Ctrl
10585842	Nptn	NM_009145	0.00820841	-1.16776	E15.5 * Wnt1 down vs E15.5 * Ctrl
10501179	Rpl13	NM_016738	0.00210973	-1.16802	E15.5 * Wnt1 down vs E15.5 * Ctrl
10426891	Mettl7a1	NM_027334	0.00572784	-1.16809	E15.5 * Wnt1 down vs E15.5 * Ctrl
10539104	0610030E20Rik	NM_026696	0.00612038	-1.1688	E15.5 * Wnt1 down vs E15.5 * Ctrl
10596231	Dnajc13	AB093258	0.00717089	-1.16931	E15.5 * Wnt1 down vs E15.5 * Ctrl
10463751	Pdcd11	NM_011053	0.014405	-1.16943	E15.5 * Wnt1 down vs E15.5 * Ctrl
10420694	Ints6	NM_008715	0.00072262	-1.16952	E15.5 * Wnt1 down vs E15.5 * Ctrl
10472598	Klhl23	NM_177784	0.00444451	-1.16955	E15.5 * Wnt1 down vs E15.5 * Ctrl
10607724	Zrsr2	NM_178794	0.00596379	-1.1697	E15.5 * Wnt1 down vs E15.5 * Ctrl
10436048	Prdx1	NM_011034	0.00900766	-1.16983	E15.5 * Wnt1 down vs E15.5 * Ctrl
10385034	Rpsa	NM_011029	0.00113317	-1.17013	E15.5 * Wnt1 down vs E15.5 * Ctrl
10552966	Tulp2	NM_008807	0.0111862	-1.17024	E15.5 * Wnt1 down vs E15.5 * Ctrl
10428998	Asap1	NM_010026	0.00312882	-1.17039	E15.5 * Wnt1 down vs E15.5 * Ctrl
10434094	Klhl22	NM_145479	0.00128571	-1.17042	E15.5 * Wnt1 down vs E15.5 * Ctrl
10546805	Ddx18	NM_025860	0.00910573	-1.17077	E15.5 * Wnt1 down vs E15.5 * Ctrl
10363575	Dna2	NM_177372	0.00224051	-1.17166	E15.5 * Wnt1 down vs E15.5 * Ctrl
10500434	Bcl9	NM_029933	0.0128842	-1.17175	E15.5 * Wnt1 down vs E15.5 * Ctrl
10512088	Topors	NM_134097	0.00159852	-1.17202	E15.5 * Wnt1 down vs E15.5 * Ctrl
10608557	Sly	BC049626	0.00815033	-1.17268	E15.5 * Wnt1 down vs E15.5 * Ctrl
10376832	Adora2b	NM_007413	0.0147054	-1.17273	E15.5 * Wnt1 down vs E15.5 * Ctrl
10545697	Dguok	NM_013764	0.0088739	-1.17332	E15.5 * Wnt1 down vs E15.5 * Ctrl
10444978	Gnal1	NM_008136	0.00916534	-1.17368	E15.5 * Wnt1 down vs E15.5 * Ctrl
10436678	Gabpa	NM_008065	0.00317766	-1.17399	E15.5 * Wnt1 down vs E15.5 * Ctrl
10474057	4631405J19Rik	BC100298	0.0121323	-1.17511	E15.5 * Wnt1 down vs E15.5 * Ctrl
10415636	Il17d	NM_145837	6.74056e-005	-1.1752	E15.5 * Wnt1 down vs E15.5 * Ctrl
10414360	Lgals3	NM_001145953	0.00432385	-1.17565	E15.5 * Wnt1 down vs E15.5 * Ctrl
10528332	Napepld	NM_178728	0.00382313	-1.1763	E15.5 * Wnt1 down vs E15.5 * Ctrl
10540812	Tatdn2	NM_001033463	0.00566319	-1.17635	E15.5 * Wnt1 down vs E15.5 * Ctrl
10477264	Tm9sf4	NM_133847	0.00500516	-1.17649	E15.5 * Wnt1 down vs E15.5 * Ctrl
10445867	Plcl2	NM_013880	0.00656982	-1.17666	E15.5 * Wnt1 down vs E15.5 * Ctrl
10479411	Ogfr	NM_031373	0.0040624	-1.17666	E15.5 * Wnt1 down vs E15.5 * Ctrl
10415282	Psme1	NM_011189	0.00488635	-1.17685	E15.5 * Wnt1 down vs E15.5 * Ctrl
10598198	Mia3	NM_177389	0.00136792	-1.17692	E15.5 * Wnt1 down vs E15.5 * Ctrl
10351691	Slamf6	NM_030710	0.0037525	-1.17713	E15.5 * Wnt1 down vs E15.5 * Ctrl
10389484	Rps6kb1	NM_001114334	0.00319195	-1.17763	E15.5 * Wnt1 down vs E15.5 * Ctrl
10594110	Neo1	NM_008684	0.000552983	-1.17777	E15.5 * Wnt1 down vs E15.5 * Ctrl
10608330	Sly	NM_201530	0.0121643	-1.17812	E15.5 * Wnt1 down vs E15.5 * Ctrl
10533316	Erp29	NM_026129	0.000662529	-1.1786	E15.5 * Wnt1 down vs E15.5 * Ctrl
10454039	Impact	NM_008378	0.00421826	-1.17976	E15.5 * Wnt1 down vs E15.5 * Ctrl
10440406	Nrip1	NM_173440	0.00370349	-1.17977	E15.5 * Wnt1 down vs E15.5 * Ctrl
10516605	Hdac1	NM_008228	0.00109131	-1.17992	E15.5 * Wnt1 down vs E15.5 * Ctrl
10473624	Olfr1257	NM_146982	0.00876204	-1.17998	E15.5 * Wnt1 down vs E15.5 * Ctrl
10357332	Actr3	NM_023735	0.00891439	-1.18002	E15.5 * Wnt1 down vs E15.5 * Ctrl
10521966	Papd4	NM_133905	0.00393732	-1.18016	E15.5 * Wnt1 down vs E15.5 * Ctrl
10529410	Mfsd10	NM_026660	0.0140716	-1.18016	E15.5 * Wnt1 down vs E15.5 * Ctrl
10375485		---	0.0101003	-1.18022	E15.5 * Wnt1 down vs E15.5 * Ctrl
10555438	Fchsd2	NM_199012	0.00147916	-1.18029	E15.5 * Wnt1 down vs E15.5 * Ctrl
10440661	Rpl13	NM_016738	0.00262428	-1.1806	E15.5 * Wnt1 down vs E15.5 * Ctrl
10544084	Zc3hav1l	NM_172467	0.00947984	-1.18087	E15.5 * Wnt1 down vs E15.5 * Ctrl
10398601	Rcor1	NM_198023	0.00618608	-1.18104	E15.5 * Wnt1 down vs E15.5 * Ctrl
10607646	Nhs	NM_001081052	0.00047519	-1.18125	E15.5 * Wnt1 down vs E15.5 * Ctrl
10541089	2900097C17Rik	NR_024329	0.0073837	-1.18127	E15.5 * Wnt1 down vs E15.5 * Ctrl
10492480	Gfm1	NM_138591	0.00697716	-1.18137	E15.5 * Wnt1 down vs E15.5 * Ctrl
10535381	Actb	NM_007393	0.00262511	-1.18143	E15.5 * Wnt1 down vs E15.5 * Ctrl
10435489	Ccdc58	NM_198645	0.00173464	-1.18149	E15.5 * Wnt1 down vs E15.5 * Ctrl
10436372	Dcbld2	NM_028523	0.00477641	-1.18212	E15.5 * Wnt1 down vs E15.5 * Ctrl
10408915	Dtnbp1	NM_025772	0.00100376	-1.18217	E15.5 * Wnt1 down vs E15.5 * Ctrl

10503283	1110037F02Rik	NM_001081183	0.0133755	-1.1823	E15.5 * Wnt1 down vs E15.5 * Ctrl
10569437	Phlda2	NM_009434	0.00562862	-1.18289	E15.5 * Wnt1 down vs E15.5 * Ctrl
10393309	Prpsap1	BC029621	0.012103	-1.183	E15.5 * Wnt1 down vs E15.5 * Ctrl
10465226	Scyl1	NM_023912	0.00572792	-1.18341	E15.5 * Wnt1 down vs E15.5 * Ctrl
10586907	Mns1	NM_008613	0.00578627	-1.18367	E15.5 * Wnt1 down vs E15.5 * Ctrl
10437023	Morc3	NM_001045529	0.0137202	-1.18383	E15.5 * Wnt1 down vs E15.5 * Ctrl
10577948	Brf2	NM_025686	0.00857336	-1.18415	E15.5 * Wnt1 down vs E15.5 * Ctrl
10518841	Thap3	NM_175152	0.00284542	-1.18458	E15.5 * Wnt1 down vs E15.5 * Ctrl
10569368	EG640264	XM_976302	0.00222808	-1.18534	E15.5 * Wnt1 down vs E15.5 * Ctrl
10523275	LOC674636	XR_004671	0.00244379	-1.18539	E15.5 * Wnt1 down vs E15.5 * Ctrl
10503109	Chchd7	NM_181391	0.000709237	-1.18554	E15.5 * Wnt1 down vs E15.5 * Ctrl
10396778	Mpp5	NM_019579	0.00586799	-1.18612	E15.5 * Wnt1 down vs E15.5 * Ctrl
10604501	Hnrnpf	NM_133834	0.000124973	-1.18621	E15.5 * Wnt1 down vs E15.5 * Ctrl
10457967	Myo7b	NM_032394	0.0118426	-1.18621	E15.5 * Wnt1 down vs E15.5 * Ctrl
10429081	Snrpc	NM_011432	0.00969338	-1.18622	E15.5 * Wnt1 down vs E15.5 * Ctrl
10394846	LOC100043371	ENSMUST00000	0.0132183	-1.18638	E15.5 * Wnt1 down vs E15.5 * Ctrl
10388613	Ccdc55	NM_001012309	0.00292695	-1.18658	E15.5 * Wnt1 down vs E15.5 * Ctrl
10412719	Synpr	NM_028052	0.00989707	-1.18687	E15.5 * Wnt1 down vs E15.5 * Ctrl
10395129	Tmem18	NM_172049	0.00531258	-1.18707	E15.5 * Wnt1 down vs E15.5 * Ctrl
10453272	Zfp36l2	NM_001001806	0.00588418	-1.18711	E15.5 * Wnt1 down vs E15.5 * Ctrl
10450103	H2-Ke6	NM_013543	0.00211106	-1.1875	E15.5 * Wnt1 down vs E15.5 * Ctrl
10412701	3830406C13Rik	NM_146051	0.00534803	-1.18771	E15.5 * Wnt1 down vs E15.5 * Ctrl
10436951	1190017O12Rik	NM_138743	0.00976024	-1.18786	E15.5 * Wnt1 down vs E15.5 * Ctrl
10564938	Fes	NM_010194	0.000519243	-1.18794	E15.5 * Wnt1 down vs E15.5 * Ctrl
10367282	Cnpy2	NM_019953	0.00330191	-1.18863	E15.5 * Wnt1 down vs E15.5 * Ctrl
10391378	Ezh1	NM_007970	0.000234192	-1.18873	E15.5 * Wnt1 down vs E15.5 * Ctrl
10498064	Setd7	NM_080793	0.0104836	-1.18945	E15.5 * Wnt1 down vs E15.5 * Ctrl
10567964	Cln3	NM_001146311	0.00434403	-1.18949	E15.5 * Wnt1 down vs E15.5 * Ctrl
10579142	EG665649	XR_032879	0.00342057	-1.18949	E15.5 * Wnt1 down vs E15.5 * Ctrl
10473650	Nup160	NM_021512	0.00212742	-1.18961	E15.5 * Wnt1 down vs E15.5 * Ctrl
10598474	Slc35a2	NM_078484	0.0120729	-1.18987	E15.5 * Wnt1 down vs E15.5 * Ctrl
10390685	Ormdl3	NM_025661	0.0132821	-1.18992	E15.5 * Wnt1 down vs E15.5 * Ctrl
10524124	Golga3	NM_008146	0.000806452	-1.18999	E15.5 * Wnt1 down vs E15.5 * Ctrl
10467637	Arhgap19	NM_027667	0.0106876	-1.19006	E15.5 * Wnt1 down vs E15.5 * Ctrl
10442786	Tpsb2	NM_010781	0.00276641	-1.19031	E15.5 * Wnt1 down vs E15.5 * Ctrl
10542764	Arntl2	NM_172309	0.0085988	-1.19046	E15.5 * Wnt1 down vs E15.5 * Ctrl
10577395	6820431F20Rik	BC058969	0.00679662	-1.19053	E15.5 * Wnt1 down vs E15.5 * Ctrl
10522676	Srp72	NM_025691	0.00149087	-1.19073	E15.5 * Wnt1 down vs E15.5 * Ctrl
10523001	Mobkl1a	NM_026735	0.00516108	-1.19074	E15.5 * Wnt1 down vs E15.5 * Ctrl
10462480	Cstf2t	NM_031249	0.00435783	-1.19077	E15.5 * Wnt1 down vs E15.5 * Ctrl
10360589	Ahctf1	NM_026375	8.67474e-005	-1.19111	E15.5 * Wnt1 down vs E15.5 * Ctrl
10537292	1810058I24Rik	ENSMUST00000	0.00778219	-1.19123	E15.5 * Wnt1 down vs E15.5 * Ctrl
10464240	Trub1	NM_028115	0.00992476	-1.19133	E15.5 * Wnt1 down vs E15.5 * Ctrl
10415065	Lrp10	NM_022993	0.000815563	-1.19141	E15.5 * Wnt1 down vs E15.5 * Ctrl
10599463	Xpnpep2	NM_133213	0.00344092	-1.19154	E15.5 * Wnt1 down vs E15.5 * Ctrl
10596137	Srprb	NM_009275	0.0140131	-1.19159	E15.5 * Wnt1 down vs E15.5 * Ctrl
10448202	Tpm4	NM_001001491	8.72877e-005	-1.19207	E15.5 * Wnt1 down vs E15.5 * Ctrl
10553042	Rasip1	NM_028544	0.0134618	-1.19228	E15.5 * Wnt1 down vs E15.5 * Ctrl
10415248	Pck2	NM_028994	0.00375187	-1.19256	E15.5 * Wnt1 down vs E15.5 * Ctrl
10563411	Grwd1	NM_153419	0.0136327	-1.19295	E15.5 * Wnt1 down vs E15.5 * Ctrl
10570612	Defb5	NM_030734	0.0132264	-1.19328	E15.5 * Wnt1 down vs E15.5 * Ctrl
10353032	Rpa3	NM_026632	0.0127522	-1.19329	E15.5 * Wnt1 down vs E15.5 * Ctrl
10467470	Aldh18a1	NM_019698	0.00732082	-1.19334	E15.5 * Wnt1 down vs E15.5 * Ctrl
10588429	Pik3r4	NM_001081309	0.00679874	-1.19335	E15.5 * Wnt1 down vs E15.5 * Ctrl
10530278		---	0.00107567	-1.19395	E15.5 * Wnt1 down vs E15.5 * Ctrl
10464218	Fam160b1	NM_145505	0.00131766	-1.19445	E15.5 * Wnt1 down vs E15.5 * Ctrl
10593219	Nnmt	NM_010924	0.00716862	-1.19492	E15.5 * Wnt1 down vs E15.5 * Ctrl
10541644	Cd163	NM_053094	0.0124334	-1.19523	E15.5 * Wnt1 down vs E15.5 * Ctrl
10558029	Sec23ip	NM_001029982	0.0105505	-1.19537	E15.5 * Wnt1 down vs E15.5 * Ctrl
10573713	Heatr3	NM_172757	0.00948923	-1.19548	E15.5 * Wnt1 down vs E15.5 * Ctrl
10345626	Eif5b	NM_198303	0.00632307	-1.19553	E15.5 * Wnt1 down vs E15.5 * Ctrl
10573217	Ddx39	NM_197982	0.00247341	-1.19562	E15.5 * Wnt1 down vs E15.5 * Ctrl
10477929	1110008F13Rik	NM_026124	0.00389087	-1.19583	E15.5 * Wnt1 down vs E15.5 * Ctrl
10386548	Pabpc4	NM_130881	0.00488984	-1.19598	E15.5 * Wnt1 down vs E15.5 * Ctrl
10582699	2810004N23Rik	NM_025615	0.00620943	-1.19622	E15.5 * Wnt1 down vs E15.5 * Ctrl
10521555	Lyar	NM_025281	0.000839001	-1.19646	E15.5 * Wnt1 down vs E15.5 * Ctrl

10429641	Naprt1	NM_172607	0.0131262	-1.19693	E15.5 * Wnt1 down vs E15.5 * Ctrl
10360884	Iars2	NM_198653	0.00462468	-1.19708	E15.5 * Wnt1 down vs E15.5 * Ctrl
10449644	Glo1	NM_025374	0.0111588	-1.19716	E15.5 * Wnt1 down vs E15.5 * Ctrl
10425211	Gcat	NM_013847	0.00744713	-1.19721	E15.5 * Wnt1 down vs E15.5 * Ctrl
10477572	Chmp4b	NM_029362	0.00138038	-1.19735	E15.5 * Wnt1 down vs E15.5 * Ctrl
10477286	Pofut1	NM_080463	0.00345778	-1.19753	E15.5 * Wnt1 down vs E15.5 * Ctrl
10415013		---	0.0118739	-1.19763	E15.5 * Wnt1 down vs E15.5 * Ctrl
10459534	Afg3l2	NM_027130	0.00278753	-1.19764	E15.5 * Wnt1 down vs E15.5 * Ctrl
10583586	Slc44a2	NM_152808	0.00373068	-1.19779	E15.5 * Wnt1 down vs E15.5 * Ctrl
10443836	Rrp1b	NM_028244	0.0122985	-1.19825	E15.5 * Wnt1 down vs E15.5 * Ctrl
10500976	Rap1a	NM_145541	0.00168943	-1.19838	E15.5 * Wnt1 down vs E15.5 * Ctrl
10502146	Gar1	NM_026578	0.0133369	-1.199	E15.5 * Wnt1 down vs E15.5 * Ctrl
10500406	Cd160	NM_018767	0.00353028	-1.19922	E15.5 * Wnt1 down vs E15.5 * Ctrl
10352281	Acbd3	NM_133225	0.00290488	-1.1994	E15.5 * Wnt1 down vs E15.5 * Ctrl
10407766	Lgals8	NM_018886	0.000389809	-1.19956	E15.5 * Wnt1 down vs E15.5 * Ctrl
10535247	Iqce	NM_028833	0.00191756	-1.19969	E15.5 * Wnt1 down vs E15.5 * Ctrl
10468329	Obfc1	NM_175360	0.00706441	-1.19989	E15.5 * Wnt1 down vs E15.5 * Ctrl
10426225	Ncaph2	NM_001115132	0.010341	-1.2011	E15.5 * Wnt1 down vs E15.5 * Ctrl
10570432	Rpl27a	AF357390	0.0118212	-1.20123	E15.5 * Wnt1 down vs E15.5 * Ctrl
10554061	Adamts17	NM_001033877	0.00347328	-1.20127	E15.5 * Wnt1 down vs E15.5 * Ctrl
10553993	Snrpa1	NM_021336	0.00058941	-1.2016	E15.5 * Wnt1 down vs E15.5 * Ctrl
10532040	Zfp644	NM_026856	0.000909375	-1.20165	E15.5 * Wnt1 down vs E15.5 * Ctrl
10414497	Parp2	NM_009632	0.00121355	-1.20166	E15.5 * Wnt1 down vs E15.5 * Ctrl
10413304	Arf4	NM_007479	0.000143083	-1.20173	E15.5 * Wnt1 down vs E15.5 * Ctrl
10401713	Pomt2	NM_153415	0.00332327	-1.2018	E15.5 * Wnt1 down vs E15.5 * Ctrl
10566201	Olfr589	NM_147052	0.0123895	-1.20186	E15.5 * Wnt1 down vs E15.5 * Ctrl
10569458	Cars	NM_013742	0.000382239	-1.20199	E15.5 * Wnt1 down vs E15.5 * Ctrl
10379127	Spag5	NM_017407	0.00628616	-1.20216	E15.5 * Wnt1 down vs E15.5 * Ctrl
10393509	Usp36	NM_001033528	0.00632071	-1.20223	E15.5 * Wnt1 down vs E15.5 * Ctrl
10468593	Nrap	NM_008733	0.0018371	-1.20232	E15.5 * Wnt1 down vs E15.5 * Ctrl
10426477	Snrpc	NM_011432	0.00348234	-1.20238	E15.5 * Wnt1 down vs E15.5 * Ctrl
10437928	0610037P05Rik	BC011300	0.0020713	-1.20265	E15.5 * Wnt1 down vs E15.5 * Ctrl
10443021	Atp6v0e	NM_025272	0.00329148	-1.20272	E15.5 * Wnt1 down vs E15.5 * Ctrl
10378508	Tsr1	NM_177325	0.00203293	-1.20306	E15.5 * Wnt1 down vs E15.5 * Ctrl
10528385	Reln	NM_011261	0.0117134	-1.20313	E15.5 * Wnt1 down vs E15.5 * Ctrl
10462346	Rcl1	NM_021525	0.00141234	-1.20357	E15.5 * Wnt1 down vs E15.5 * Ctrl
10520234	2010209O12Rik	NM_133913	0.000409971	-1.20361	E15.5 * Wnt1 down vs E15.5 * Ctrl
10539042	Polr1a	NM_009088	0.0126421	-1.20373	E15.5 * Wnt1 down vs E15.5 * Ctrl
10408684	Tmed10	NM_026775	1.33754e-007	-1.20391	E15.5 * Wnt1 down vs E15.5 * Ctrl
10445053	Trim31	NM_146077	0.000333682	-1.20392	E15.5 * Wnt1 down vs E15.5 * Ctrl
10547153	Alox5	NM_009662	0.0126997	-1.20411	E15.5 * Wnt1 down vs E15.5 * Ctrl
10492078	Alg5	NM_025442	0.00501212	-1.20419	E15.5 * Wnt1 down vs E15.5 * Ctrl
10595793	Atp1b3	NM_007502	0.000896526	-1.2047	E15.5 * Wnt1 down vs E15.5 * Ctrl
10546066	Isy1	NM_133934	0.00333936	-1.20505	E15.5 * Wnt1 down vs E15.5 * Ctrl
10372687	Nup107	NM_134010	0.000731801	-1.20508	E15.5 * Wnt1 down vs E15.5 * Ctrl
10442445	Dci	NM_010023	0.00476517	-1.20515	E15.5 * Wnt1 down vs E15.5 * Ctrl
10379721	Ccl4	NM_013652	0.00161716	-1.20518	E15.5 * Wnt1 down vs E15.5 * Ctrl
10382228	Axin2	NM_015732	0.00152621	-1.20519	E15.5 * Wnt1 down vs E15.5 * Ctrl
10375951	9530068E07Rik	NM_153117	0.000181193	-1.20575	E15.5 * Wnt1 down vs E15.5 * Ctrl
10438442	A930003A15Rik	ENSMUST00000	0.0145122	-1.20586	E15.5 * Wnt1 down vs E15.5 * Ctrl
10608492	LOC380994	BC099537	0.0113329	-1.20619	E15.5 * Wnt1 down vs E15.5 * Ctrl
10447004	Hdac1	NM_008228	0.000761186	-1.20636	E15.5 * Wnt1 down vs E15.5 * Ctrl
10474048	Syt13	NM_030725	0.000451486	-1.20644	E15.5 * Wnt1 down vs E15.5 * Ctrl
10421021	EG628416	NM_001037931	0.00371721	-1.20668	E15.5 * Wnt1 down vs E15.5 * Ctrl
10556206	Rpl27a	AF357390	0.00922841	-1.20677	E15.5 * Wnt1 down vs E15.5 * Ctrl
10508074	Csf3r	NM_007782	0.00541353	-1.20706	E15.5 * Wnt1 down vs E15.5 * Ctrl
10460833	Ehd1	NM_010119	0.000558386	-1.20724	E15.5 * Wnt1 down vs E15.5 * Ctrl
10446656	Lpin2	NM_022882	0.00879377	-1.20739	E15.5 * Wnt1 down vs E15.5 * Ctrl
10542321	Ddx47	NM_026360	0.0113523	-1.20742	E15.5 * Wnt1 down vs E15.5 * Ctrl
10579479	Nr2f6	NM_010150	0.00968685	-1.20769	E15.5 * Wnt1 down vs E15.5 * Ctrl
10386543	Pabpc4	NM_130881	0.00202918	-1.20788	E15.5 * Wnt1 down vs E15.5 * Ctrl
10369388	Unc5b	NM_029770	0.0113109	-1.20801	E15.5 * Wnt1 down vs E15.5 * Ctrl
10384044	Myl7	NM_022879	0.00240914	-1.20805	E15.5 * Wnt1 down vs E15.5 * Ctrl
10458498	Arap3	NM_139206	0.00942081	-1.20824	E15.5 * Wnt1 down vs E15.5 * Ctrl
10518847	Phf13	NM_172705	0.0002158	-1.20847	E15.5 * Wnt1 down vs E15.5 * Ctrl
10469581	Etl4	NM_001081006	0.00159467	-1.2085	E15.5 * Wnt1 down vs E15.5 * Ctrl

10396862	Strm	AF031663	0.00733604	-1.20859	E15.5 * Wnt1 down vs E15.5 * Ctrl
10571288	Gtf2e2	NM_026584	0.000802935	-1.2088	E15.5 * Wnt1 down vs E15.5 * Ctrl
10551021	Rps19	NM_023133	0.00783671	-1.20883	E15.5 * Wnt1 down vs E15.5 * Ctrl
10370303	Adarb1	NR_004429	0.000990918	-1.20909	E15.5 * Wnt1 down vs E15.5 * Ctrl
10481023	Sec16a	NM_153125	0.000108864	-1.20927	E15.5 * Wnt1 down vs E15.5 * Ctrl
10487711	Ddrgk1	NM_029832	0.0144741	-1.20942	E15.5 * Wnt1 down vs E15.5 * Ctrl
10577315	Angpt2	NM_007426	0.00989061	-1.20951	E15.5 * Wnt1 down vs E15.5 * Ctrl
10368356	Akap7	NM_018747	0.0135287	-1.20952	E15.5 * Wnt1 down vs E15.5 * Ctrl
10500736	Vangl1	NM_177545	0.000305645	-1.2096	E15.5 * Wnt1 down vs E15.5 * Ctrl
10513190	D630039A03Rik	BC095953	0.00274553	-1.2098	E15.5 * Wnt1 down vs E15.5 * Ctrl
10502284	Tet2	NM_001040400	0.00943512	-1.20984	E15.5 * Wnt1 down vs E15.5 * Ctrl
10344725	Adhfe1	NM_175236	0.00324111	-1.21063	E15.5 * Wnt1 down vs E15.5 * Ctrl
10519096	Rer1	NM_026395	0.0100304	-1.21074	E15.5 * Wnt1 down vs E15.5 * Ctrl
10512919	Grin3a	NM_001033351	0.0131422	-1.21078	E15.5 * Wnt1 down vs E15.5 * Ctrl
10453276	Thada	EF222207	0.00276338	-1.21094	E15.5 * Wnt1 down vs E15.5 * Ctrl
10544596	Tmem176b	NM_023056	0.00973443	-1.21106	E15.5 * Wnt1 down vs E15.5 * Ctrl
10443598	Dnahc8	NM_013811	0.000687054	-1.21124	E15.5 * Wnt1 down vs E15.5 * Ctrl
10402195	Tc2n	NM_028924	0.00340929	-1.21133	E15.5 * Wnt1 down vs E15.5 * Ctrl
10554150	Rgma	NM_177740	0.00676703	-1.21138	E15.5 * Wnt1 down vs E15.5 * Ctrl
10500847	Magi3	NM_133853	0.00767499	-1.21145	E15.5 * Wnt1 down vs E15.5 * Ctrl
10447417	Msh6	NM_010830	0.000834416	-1.21166	E15.5 * Wnt1 down vs E15.5 * Ctrl
10583817	Pigyl	NM_001082532	0.0074589	-1.21169	E15.5 * Wnt1 down vs E15.5 * Ctrl
10525555	Mlxip	NM_133917	0.000675938	-1.21219	E15.5 * Wnt1 down vs E15.5 * Ctrl
10405605	Smad5	NM_008541	0.000788547	-1.21221	E15.5 * Wnt1 down vs E15.5 * Ctrl
10515836	Ccnb1	NM_172301	0.0108575	-1.21261	E15.5 * Wnt1 down vs E15.5 * Ctrl
10487513	Anapc1	NM_008569	4.00487e-006	-1.21262	E15.5 * Wnt1 down vs E15.5 * Ctrl
10602592	Hsd17b10	NM_016763	0.00327271	-1.21276	E15.5 * Wnt1 down vs E15.5 * Ctrl
10422854	Nup155	NM_133227	0.000356178	-1.21291	E15.5 * Wnt1 down vs E15.5 * Ctrl
10435693	Cox17	NM_001017429	0.000202162	-1.21297	E15.5 * Wnt1 down vs E15.5 * Ctrl
10607587	Pdha1	NM_008810	0.00957648	-1.2136	E15.5 * Wnt1 down vs E15.5 * Ctrl
10386125	Gemin5	NM_172558	0.0108682	-1.21371	E15.5 * Wnt1 down vs E15.5 * Ctrl
10444505	Rdbp	NM_001045864	0.00116802	-1.21392	E15.5 * Wnt1 down vs E15.5 * Ctrl
10409666	Kif27	NM_175214	0.000495132	-1.21412	E15.5 * Wnt1 down vs E15.5 * Ctrl
10503659	Epha7	NM_010141	0.000643828	-1.21425	E15.5 * Wnt1 down vs E15.5 * Ctrl
10565935	Arhgef17	NM_001081116	0.0104922	-1.21428	E15.5 * Wnt1 down vs E15.5 * Ctrl
10385428	Itk	NM_010583	0.0117009	-1.21433	E15.5 * Wnt1 down vs E15.5 * Ctrl
10471191	Exosc2	NM_144886	0.0111675	-1.21437	E15.5 * Wnt1 down vs E15.5 * Ctrl
10475335	Pdia3	NM_007952	0.00238085	-1.21473	E15.5 * Wnt1 down vs E15.5 * Ctrl
10521391	4931431C16Rik	ENSMUST00000(	0.00110681	-1.21493	E15.5 * Wnt1 down vs E15.5 * Ctrl
10528310	Fbxl13	NM_177076	0.00389564	-1.21497	E15.5 * Wnt1 down vs E15.5 * Ctrl
10542557	Aebp2	NM_001005605	0.00354142	-1.21515	E15.5 * Wnt1 down vs E15.5 * Ctrl
10582429	Cbfa2t3	NM_009824	0.000397219	-1.21522	E15.5 * Wnt1 down vs E15.5 * Ctrl
10414245	4930503E14Rik	NM_029131	0.00945508	-1.21554	E15.5 * Wnt1 down vs E15.5 * Ctrl
10484927	Slc39a13	NM_026721	0.00944542	-1.21583	E15.5 * Wnt1 down vs E15.5 * Ctrl
10491753	Intu	NM_175515	0.0106202	-1.21615	E15.5 * Wnt1 down vs E15.5 * Ctrl
10563026	Bcl2l12	NM_029410	0.00651351	-1.21624	E15.5 * Wnt1 down vs E15.5 * Ctrl
10443470	Rab44	NM_001002786	0.00149903	-1.2163	E15.5 * Wnt1 down vs E15.5 * Ctrl
10514926	B230314M03Rik	ENSMUST00000(	0.0134821	-1.21656	E15.5 * Wnt1 down vs E15.5 * Ctrl
10538438	C330043M08Rik	NM_001142781	0.00136938	-1.21681	E15.5 * Wnt1 down vs E15.5 * Ctrl
10487340	Ncaph	NM_144818	0.000649733	-1.21706	E15.5 * Wnt1 down vs E15.5 * Ctrl
10347593	Speg	NM_007463	0.00336815	-1.21709	E15.5 * Wnt1 down vs E15.5 * Ctrl
10383010	Socs3	U72673	0.00749927	-1.21725	E15.5 * Wnt1 down vs E15.5 * Ctrl
10465853	Stxbp3b	NM_001030317	0.00804185	-1.2174	E15.5 * Wnt1 down vs E15.5 * Ctrl
10576034	Irf8	NM_008320	0.00116566	-1.21759	E15.5 * Wnt1 down vs E15.5 * Ctrl
10554240	Isg20	NM_020583	0.000812066	-1.21764	E15.5 * Wnt1 down vs E15.5 * Ctrl
10433153	BC048502	BC048502	0.00590811	-1.21771	E15.5 * Wnt1 down vs E15.5 * Ctrl
10568758	9430038I01Rik	ENSMUST00000(	0.0120907	-1.21777	E15.5 * Wnt1 down vs E15.5 * Ctrl
10571162	Eif4ebp1	NM_007918	0.00849689	-1.21777	E15.5 * Wnt1 down vs E15.5 * Ctrl
10479775	Hspa14	NM_015765	0.00153329	-1.21837	E15.5 * Wnt1 down vs E15.5 * Ctrl
10435237	Zfp148	NM_011749	0.000749826	-1.21841	E15.5 * Wnt1 down vs E15.5 * Ctrl
10581772	Glg1	NM_009149	0.000520546	-1.21854	E15.5 * Wnt1 down vs E15.5 * Ctrl
10381408	Ifi35	NM_027320	0.00716657	-1.21881	E15.5 * Wnt1 down vs E15.5 * Ctrl
10437664	Dexi	NM_021428	0.000873816	-1.21887	E15.5 * Wnt1 down vs E15.5 * Ctrl
10368380	L3mbtl3	NM_172787	0.000356799	-1.219	E15.5 * Wnt1 down vs E15.5 * Ctrl
10391870	Map3k14	NM_016896	0.0003776	-1.21909	E15.5 * Wnt1 down vs E15.5 * Ctrl
10578448	Cyp4v3	NM_133969	7.51925e-005	-1.21917	E15.5 * Wnt1 down vs E15.5 * Ctrl

10517060	Nudc	NM_010948	0.00929403	-1.21919	E15.5 * Wnt1 down vs E15.5 * Ctrl
10557758	Setd1a	NM_178029	0.0139595	-1.21939	E15.5 * Wnt1 down vs E15.5 * Ctrl
10512704	Exosc3	NM_025513	0.00547442	-1.21942	E15.5 * Wnt1 down vs E15.5 * Ctrl
10352735	Ints7	NM_178632	0.00304803	-1.21961	E15.5 * Wnt1 down vs E15.5 * Ctrl
10416736	6720463M24Rik	NM_175265	0.00711314	-1.21972	E15.5 * Wnt1 down vs E15.5 * Ctrl
10483000	Itgb6	NM_001159564	0.00320142	-1.21978	E15.5 * Wnt1 down vs E15.5 * Ctrl
10461391	Pcna	NM_011045	1.5294e-006	-1.22008	E15.5 * Wnt1 down vs E15.5 * Ctrl
10577973	Adrb3	NM_013462	0.00122607	-1.22016	E15.5 * Wnt1 down vs E15.5 * Ctrl
10487447	Mall	NM_145532	0.00969523	-1.22026	E15.5 * Wnt1 down vs E15.5 * Ctrl
10350753	Glul	NM_008131	0.0082161	-1.2203	E15.5 * Wnt1 down vs E15.5 * Ctrl
10569038	Ano9	NM_178381	0.0146658	-1.22056	E15.5 * Wnt1 down vs E15.5 * Ctrl
10487930	Pcna	NM_011045	2.55507e-006	-1.22068	E15.5 * Wnt1 down vs E15.5 * Ctrl
10411595	Naip2	NM_010872	0.00236946	-1.22093	E15.5 * Wnt1 down vs E15.5 * Ctrl
10517892	D4Ertd22e	NM_001025608	0.00228551	-1.22097	E15.5 * Wnt1 down vs E15.5 * Ctrl
10565775	Dgat2	NM_026384	0.00373656	-1.22098	E15.5 * Wnt1 down vs E15.5 * Ctrl
10457007	Pqlc1	NM_025861	0.00226755	-1.22127	E15.5 * Wnt1 down vs E15.5 * Ctrl
10372716	Rap1b	NM_024457	0.000176096	-1.22151	E15.5 * Wnt1 down vs E15.5 * Ctrl
10391949	Gosr2	NM_019650	0.0083734	-1.22251	E15.5 * Wnt1 down vs E15.5 * Ctrl
10404033	Hist1h1d	NM_145713	0.00258979	-1.22286	E15.5 * Wnt1 down vs E15.5 * Ctrl
10465521	Plcb3	NM_008874	0.00547467	-1.22286	E15.5 * Wnt1 down vs E15.5 * Ctrl
10392261	Smurf2	NM_025481	0.0010104	-1.2235	E15.5 * Wnt1 down vs E15.5 * Ctrl
10427461	Ptger4	NM_001136079	0.0088253	-1.22358	E15.5 * Wnt1 down vs E15.5 * Ctrl
10362538	Lama4	NM_010681	0.000326988	-1.22363	E15.5 * Wnt1 down vs E15.5 * Ctrl
10396645	Zbtb1	NM_178744	0.0132951	-1.22397	E15.5 * Wnt1 down vs E15.5 * Ctrl
10393222	Srp68	NM_146032	0.00407619	-1.22427	E15.5 * Wnt1 down vs E15.5 * Ctrl
10565486	Eed	NM_021876	0.000748036	-1.22428	E15.5 * Wnt1 down vs E15.5 * Ctrl
10566529	Tpp1	NM_009906	0.00835281	-1.22453	E15.5 * Wnt1 down vs E15.5 * Ctrl
10397835	Slc24a4	NM_172152	0.0133808	-1.22459	E15.5 * Wnt1 down vs E15.5 * Ctrl
10514666		---	0.00947834	-1.22465	E15.5 * Wnt1 down vs E15.5 * Ctrl
10391130	Nt5c3l	NM_026561	0.00501099	-1.22486	E15.5 * Wnt1 down vs E15.5 * Ctrl
10520734	Nrbp1	NM_147201	0.00594508	-1.22494	E15.5 * Wnt1 down vs E15.5 * Ctrl
10516910	Phactr4	NM_175306	0.0081366	-1.22533	E15.5 * Wnt1 down vs E15.5 * Ctrl
10557139	Ubfd1	NM_138589	0.00778098	-1.22557	E15.5 * Wnt1 down vs E15.5 * Ctrl
10423971	Pkhd1l1	NM_138674	4.83496e-005	-1.2257	E15.5 * Wnt1 down vs E15.5 * Ctrl
10425410	Grap2	NM_010815	0.0142501	-1.22673	E15.5 * Wnt1 down vs E15.5 * Ctrl
10453436	Mcfd2	NM_176808	0.0043698	-1.22683	E15.5 * Wnt1 down vs E15.5 * Ctrl
10473406	Prg3	NM_016914	0.0114156	-1.22703	E15.5 * Wnt1 down vs E15.5 * Ctrl
10561920	Hcst	NM_011827	0.00162071	-1.22714	E15.5 * Wnt1 down vs E15.5 * Ctrl
10433797	Prkdc	NM_011159	0.00254278	-1.22723	E15.5 * Wnt1 down vs E15.5 * Ctrl
10524844	Taok3	NM_001081308	0.00308942	-1.22731	E15.5 * Wnt1 down vs E15.5 * Ctrl
10436239	2310061J03Rik	BC017620	0.00550129	-1.22781	E15.5 * Wnt1 down vs E15.5 * Ctrl
10476648	Dstn	NM_019771	0.000910574	-1.22781	E15.5 * Wnt1 down vs E15.5 * Ctrl
10414417	Peli2	NM_033602	0.00759912	-1.22805	E15.5 * Wnt1 down vs E15.5 * Ctrl
10380398	Chad	NM_007689	0.00103461	-1.22836	E15.5 * Wnt1 down vs E15.5 * Ctrl
10382425	Gprc5c	NM_001110337	0.0022905	-1.22836	E15.5 * Wnt1 down vs E15.5 * Ctrl
10508479	Ptp4a2	NM_008974	0.00316928	-1.22864	E15.5 * Wnt1 down vs E15.5 * Ctrl
10476362	Mcm8	NM_025676	0.00313221	-1.22869	E15.5 * Wnt1 down vs E15.5 * Ctrl
10478048	Lbp	NM_008489	0.00938899	-1.2287	E15.5 * Wnt1 down vs E15.5 * Ctrl
10478407	0610039K10Rik	ENSMUST00000(	0.00807341	-1.22897	E15.5 * Wnt1 down vs E15.5 * Ctrl
10548146	Tead4	NM_011567	0.00203435	-1.22932	E15.5 * Wnt1 down vs E15.5 * Ctrl
10599736	Fhl1	NM_001077361	0.000630495	-1.22942	E15.5 * Wnt1 down vs E15.5 * Ctrl
10591618	Dock6	NM_177030	0.00659197	-1.22957	E15.5 * Wnt1 down vs E15.5 * Ctrl
10591628	Dock6	NM_177030	0.0101933	-1.22963	E15.5 * Wnt1 down vs E15.5 * Ctrl
10425632	Xrcc6	NM_010247	0.00304919	-1.22978	E15.5 * Wnt1 down vs E15.5 * Ctrl
10450325	Cfb	NM_008198	0.000524512	-1.22983	E15.5 * Wnt1 down vs E15.5 * Ctrl
10602625	Iqsec2	NM_001005475	0.00210398	-1.22984	E15.5 * Wnt1 down vs E15.5 * Ctrl
10544891	Nod1	NM_172729	0.00723491	-1.23	E15.5 * Wnt1 down vs E15.5 * Ctrl
10430974	Arfgap3	NM_025445	0.000852577	-1.23006	E15.5 * Wnt1 down vs E15.5 * Ctrl
10473356	Ube2l6	NM_019949	0.0047999	-1.23025	E15.5 * Wnt1 down vs E15.5 * Ctrl
10444605	Vars	NM_011690	0.012207	-1.23061	E15.5 * Wnt1 down vs E15.5 * Ctrl
10588466	Twf2	NM_011876	0.00429873	-1.23067	E15.5 * Wnt1 down vs E15.5 * Ctrl
10530477	Nfxl1	NM_133921	0.00994788	-1.23082	E15.5 * Wnt1 down vs E15.5 * Ctrl
10451061	Runx2	NM_001146038	0.00180673	-1.23086	E15.5 * Wnt1 down vs E15.5 * Ctrl
10515848	Ermap	NM_013848	0.0137562	-1.23092	E15.5 * Wnt1 down vs E15.5 * Ctrl
10469083	Upf2	NM_001081132	0.000517232	-1.23112	E15.5 * Wnt1 down vs E15.5 * Ctrl
10451710	Rftn1	NM_181397	0.0050485	-1.23117	E15.5 * Wnt1 down vs E15.5 * Ctrl

10560247	Ccdc9	NM_172297	0.00203083	-1.2312	E15.5 * Wnt1 down vs E15.5 * Ctrl
10403934	Isca1	NM_026921	0.00330827	-1.23127	E15.5 * Wnt1 down vs E15.5 * Ctrl
10471201	Abl1	NM_001112703	0.0102477	-1.23183	E15.5 * Wnt1 down vs E15.5 * Ctrl
10411454	Sec61b	NM_024171	0.000149658	-1.232	E15.5 * Wnt1 down vs E15.5 * Ctrl
10478389	Hnf4a	NM_008261	0.0054325	-1.23216	E15.5 * Wnt1 down vs E15.5 * Ctrl
10556456	2610024B07Rik	BC049153	0.00203248	-1.23235	E15.5 * Wnt1 down vs E15.5 * Ctrl
10475932	Fbln7	NM_024237	0.000484309	-1.23235	E15.5 * Wnt1 down vs E15.5 * Ctrl
10427910	Fam105b	NM_001013792	0.00585866	-1.23243	E15.5 * Wnt1 down vs E15.5 * Ctrl
10388758	Nek8	NM_080849	0.00297587	-1.23247	E15.5 * Wnt1 down vs E15.5 * Ctrl
10580010	Pkn1	NM_177262	0.00337179	-1.2325	E15.5 * Wnt1 down vs E15.5 * Ctrl
10467560	Tm9sf3	NM_133352	0.000371847	-1.2326	E15.5 * Wnt1 down vs E15.5 * Ctrl
10516590	Zbtb8a	NM_028603	0.0100979	-1.23262	E15.5 * Wnt1 down vs E15.5 * Ctrl
10570683	Defa-ps1	NR_003146	0.0038696	-1.23267	E15.5 * Wnt1 down vs E15.5 * Ctrl
10529156	Ift172	NM_026298	0.00132284	-1.23269	E15.5 * Wnt1 down vs E15.5 * Ctrl
10396108	Arf6	NM_007481	1.14178e-005	-1.23274	E15.5 * Wnt1 down vs E15.5 * Ctrl
10427772	Tars	NM_033074	0.0125475	-1.23274	E15.5 * Wnt1 down vs E15.5 * Ctrl
10367179	Timeless	NM_001136082	0.0146051	-1.23298	E15.5 * Wnt1 down vs E15.5 * Ctrl
10584700	Dpagt1	NM_007875	0.00979088	-1.23314	E15.5 * Wnt1 down vs E15.5 * Ctrl
10376929	Fam18b	NM_026210	0.0073646	-1.23318	E15.5 * Wnt1 down vs E15.5 * Ctrl
10607868	Tlr8	NM_133212	0.00577113	-1.23332	E15.5 * Wnt1 down vs E15.5 * Ctrl
10389245	Tada2l	NM_172562	0.001638	-1.23341	E15.5 * Wnt1 down vs E15.5 * Ctrl
10555389	Ucp2	NM_011671	0.01377	-1.23355	E15.5 * Wnt1 down vs E15.5 * Ctrl
10480775	Mamdc4	NM_001081199	0.00221685	-1.23356	E15.5 * Wnt1 down vs E15.5 * Ctrl
10493474	Muc1	NM_013605	0.0098082	-1.23357	E15.5 * Wnt1 down vs E15.5 * Ctrl
10480849	Fcna	NM_007995	0.0127943	-1.23384	E15.5 * Wnt1 down vs E15.5 * Ctrl
10539337	Mogs	NM_020619	0.00199535	-1.23406	E15.5 * Wnt1 down vs E15.5 * Ctrl
10514383	Larp7	NM_138593	0.00597619	-1.23425	E15.5 * Wnt1 down vs E15.5 * Ctrl
10385941	Tnip1	NM_021327	0.00265824	-1.23431	E15.5 * Wnt1 down vs E15.5 * Ctrl
10570397	Cdc16	NM_027276	0.00211801	-1.23441	E15.5 * Wnt1 down vs E15.5 * Ctrl
10525887	Bri3bp	NM_029752	0.000636458	-1.23447	E15.5 * Wnt1 down vs E15.5 * Ctrl
10488090	Tasp1	NM_175225	0.0038361	-1.23466	E15.5 * Wnt1 down vs E15.5 * Ctrl
10381601	Grn	M86736	0.0111395	-1.23473	E15.5 * Wnt1 down vs E15.5 * Ctrl
10509620	Capzb	NM_001037761	0.00300029	-1.23484	E15.5 * Wnt1 down vs E15.5 * Ctrl
10372293	EG628870	XM_893664	0.000592127	-1.23495	E15.5 * Wnt1 down vs E15.5 * Ctrl
10445674	Mrfap1	NM_026242	0.0135945	-1.23513	E15.5 * Wnt1 down vs E15.5 * Ctrl
10596257		---	0.0141488	-1.23518	E15.5 * Wnt1 down vs E15.5 * Ctrl
10421817	Narg1l	NM_025832	0.00430998	-1.23549	E15.5 * Wnt1 down vs E15.5 * Ctrl
10356859	Dtymk	NM_001105667	2.69364e-005	-1.23557	E15.5 * Wnt1 down vs E15.5 * Ctrl
10504838	Nr4a3	NM_015743	1.84249e-005	-1.23567	E15.5 * Wnt1 down vs E15.5 * Ctrl
10455550	4833403I15Rik	BC113158	0.00169608	-1.23585	E15.5 * Wnt1 down vs E15.5 * Ctrl
10467139	Lipa	NM_021460	0.00345856	-1.23604	E15.5 * Wnt1 down vs E15.5 * Ctrl
10583044	Mmp13	NM_008607	0.000447806	-1.23604	E15.5 * Wnt1 down vs E15.5 * Ctrl
10547251	Bms1	NM_194339	0.00129049	-1.23665	E15.5 * Wnt1 down vs E15.5 * Ctrl
10483633	Sp3	NM_001018042	0.00707817	-1.23697	E15.5 * Wnt1 down vs E15.5 * Ctrl
10390196	B4galnt2	NM_008081	0.00927607	-1.23704	E15.5 * Wnt1 down vs E15.5 * Ctrl
10349453	Rab3gap1	NM_178690	0.00672525	-1.23725	E15.5 * Wnt1 down vs E15.5 * Ctrl
10393166	St6galnac2	NM_009180	0.00987798	-1.23735	E15.5 * Wnt1 down vs E15.5 * Ctrl
10451736	Tbc1d5	NM_028162	2.71121e-005	-1.23735	E15.5 * Wnt1 down vs E15.5 * Ctrl
10447629	Tagap1	NM_147155	0.00368167	-1.2376	E15.5 * Wnt1 down vs E15.5 * Ctrl
10488697	Plagl2	NM_018807	0.00230285	-1.23763	E15.5 * Wnt1 down vs E15.5 * Ctrl
10349174	Serpinb8	NM_011459	0.00239332	-1.23782	E15.5 * Wnt1 down vs E15.5 * Ctrl
10482528	Neb	NM_010889	0.00111821	-1.23785	E15.5 * Wnt1 down vs E15.5 * Ctrl
10590253	Rpsa	NM_011029	0.000843366	-1.23788	E15.5 * Wnt1 down vs E15.5 * Ctrl
10411853	Erbb2ip	NM_001005868	0.000314626	-1.23804	E15.5 * Wnt1 down vs E15.5 * Ctrl
10468898	Lax1	NM_001159649	0.00237943	-1.23818	E15.5 * Wnt1 down vs E15.5 * Ctrl
10453732	EG225134	XR_031272	0.0133307	-1.23825	E15.5 * Wnt1 down vs E15.5 * Ctrl
10476668	Csrp2bp	NM_181417	0.000973775	-1.23834	E15.5 * Wnt1 down vs E15.5 * Ctrl
10420631	Ebpl	NM_026598	0.00821238	-1.23877	E15.5 * Wnt1 down vs E15.5 * Ctrl
10360338	Fcer1a	NM_010184	0.00508004	-1.23879	E15.5 * Wnt1 down vs E15.5 * Ctrl
10362073	Sgk1	NM_011361	0.00621546	-1.23894	E15.5 * Wnt1 down vs E15.5 * Ctrl
10522819	Ugt2b35	NM_172881	0.00487896	-1.23963	E15.5 * Wnt1 down vs E15.5 * Ctrl
10463599	Nfkb2	NM_019408	3.23123e-005	-1.23973	E15.5 * Wnt1 down vs E15.5 * Ctrl
10459912	Adnp2	NM_175028	0.00929059	-1.23998	E15.5 * Wnt1 down vs E15.5 * Ctrl
10363137	Nus1	NM_030250	0.00202722	-1.2401	E15.5 * Wnt1 down vs E15.5 * Ctrl
10549265	Ifltd1	NM_028742	0.00022027	-1.24015	E15.5 * Wnt1 down vs E15.5 * Ctrl
10429657	Eef1d	NM_029663	6.43346e-006	-1.24017	E15.5 * Wnt1 down vs E15.5 * Ctrl

10427606	Skp2	NM_145468	0.00275653	-1.24017	E15.5 * Wnt1 down vs E15.5 * Ctrl
10484440	Zdhhc5	NM_144887	0.0043976	-1.2404	E15.5 * Wnt1 down vs E15.5 * Ctrl
10602925	Phka2	NM_172783	0.00597202	-1.24071	E15.5 * Wnt1 down vs E15.5 * Ctrl
10505481	8030463A06Rik	ENSMUST00000(	0.00740364	-1.24082	E15.5 * Wnt1 down vs E15.5 * Ctrl
10358991	BC034090	AK129406	0.00289309	-1.24137	E15.5 * Wnt1 down vs E15.5 * Ctrl
10529613	Evc	NM_021292	3.31432e-005	-1.24138	E15.5 * Wnt1 down vs E15.5 * Ctrl
10435266	Heg1	NM_175256	0.00914099	-1.24142	E15.5 * Wnt1 down vs E15.5 * Ctrl
10365127	2210404O07Rik	BC117023	0.00409911	-1.24149	E15.5 * Wnt1 down vs E15.5 * Ctrl
10500295	Plekho1	NM_023320	0.00220796	-1.24153	E15.5 * Wnt1 down vs E15.5 * Ctrl
10454709	Kif20a	NM_009004	2.26063e-005	-1.2416	E15.5 * Wnt1 down vs E15.5 * Ctrl
10438980	Acap2	NM_030138	0.00432037	-1.24173	E15.5 * Wnt1 down vs E15.5 * Ctrl
10435415	Sema5b	NM_013661	0.0015574	-1.24173	E15.5 * Wnt1 down vs E15.5 * Ctrl
10430770	Tob2	NM_020507	0.00410086	-1.24188	E15.5 * Wnt1 down vs E15.5 * Ctrl
10593591	Acat1	NM_144784	4.98149e-005	-1.24193	E15.5 * Wnt1 down vs E15.5 * Ctrl
10401519	Npc2	NM_023409	7.07598e-005	-1.2421	E15.5 * Wnt1 down vs E15.5 * Ctrl
10357535	Pfkfb2	NM_008825	0.0113228	-1.24227	E15.5 * Wnt1 down vs E15.5 * Ctrl
10521626	Cc2d2a	NM_172274	0.00408	-1.24241	E15.5 * Wnt1 down vs E15.5 * Ctrl
10427049	6030408B16Rik	ENSMUST00000(	0.00723372	-1.24244	E15.5 * Wnt1 down vs E15.5 * Ctrl
10358399	Rgs13	NM_153171	0.000660957	-1.2426	E15.5 * Wnt1 down vs E15.5 * Ctrl
10481155	Rexo4	NM_207234	0.000997889	-1.24267	E15.5 * Wnt1 down vs E15.5 * Ctrl
10494335	Otud7b	NM_001025613	0.00878262	-1.24294	E15.5 * Wnt1 down vs E15.5 * Ctrl
10376796	Grap	NM_027817	0.00603524	-1.24311	E15.5 * Wnt1 down vs E15.5 * Ctrl
10526493	Myl10	NM_021611	0.0012879	-1.24327	E15.5 * Wnt1 down vs E15.5 * Ctrl
10389269	Aatf	NM_019816	0.000578534	-1.24328	E15.5 * Wnt1 down vs E15.5 * Ctrl
10359734	Iqwd1	ENSMUST00000(	0.00236844	-1.24344	E15.5 * Wnt1 down vs E15.5 * Ctrl
10455954	EG240327	NM_001033767	0.000634078	-1.24345	E15.5 * Wnt1 down vs E15.5 * Ctrl
10435920	Cd200r4	NM_207244	0.00823034	-1.24383	E15.5 * Wnt1 down vs E15.5 * Ctrl
10504450	Glipr2	NM_027450	0.000695301	-1.24421	E15.5 * Wnt1 down vs E15.5 * Ctrl
10553092	Dbp	NM_016974	0.000448207	-1.24498	E15.5 * Wnt1 down vs E15.5 * Ctrl
10474312	Pax6	NM_013627	0.0134011	-1.24499	E15.5 * Wnt1 down vs E15.5 * Ctrl
10449295	Cuta	NM_026307	0.000406678	-1.24524	E15.5 * Wnt1 down vs E15.5 * Ctrl
10369379	Slc29a3	NM_023596	0.000912722	-1.24531	E15.5 * Wnt1 down vs E15.5 * Ctrl
10596912	Klhdc8b	NM_030075	0.00812213	-1.24535	E15.5 * Wnt1 down vs E15.5 * Ctrl
10455472	Dcp2	NM_027490	0.00300648	-1.2454	E15.5 * Wnt1 down vs E15.5 * Ctrl
10488465	Zfp345	NM_001034900	0.00235599	-1.24551	E15.5 * Wnt1 down vs E15.5 * Ctrl
10375956	Fstl4	NM_177059	0.000883993	-1.24557	E15.5 * Wnt1 down vs E15.5 * Ctrl
10476033	Stk35	NM_183262	0.000802639	-1.24575	E15.5 * Wnt1 down vs E15.5 * Ctrl
10604523	Rap2c	NM_172413	0.000116577	-1.24625	E15.5 * Wnt1 down vs E15.5 * Ctrl
10582664	2310022B05Rik	BC058626	0.00112939	-1.24629	E15.5 * Wnt1 down vs E15.5 * Ctrl
10425880	Prr5	NM_146061	0.00121599	-1.24651	E15.5 * Wnt1 down vs E15.5 * Ctrl
10463645	Sufu	NM_015752	0.00349919	-1.24697	E15.5 * Wnt1 down vs E15.5 * Ctrl
10461452	Fen1	NM_007999	0.00852983	-1.24702	E15.5 * Wnt1 down vs E15.5 * Ctrl
10356780	Pask	NM_080850	0.00261092	-1.24711	E15.5 * Wnt1 down vs E15.5 * Ctrl
10380566	Phospho1	NM_153104	0.000231204	-1.24724	E15.5 * Wnt1 down vs E15.5 * Ctrl
10388591	Cpd	NM_007754	1.81904e-005	-1.24736	E15.5 * Wnt1 down vs E15.5 * Ctrl
10595466	Pgm3	NM_028352	0.00280178	-1.24759	E15.5 * Wnt1 down vs E15.5 * Ctrl
10577792	Plekha2	NM_031257	0.0124561	-1.24761	E15.5 * Wnt1 down vs E15.5 * Ctrl
10561004	Erf	NM_010155	0.00592548	-1.24764	E15.5 * Wnt1 down vs E15.5 * Ctrl
10516666	Txlna	NM_001005506	0.0041703	-1.24765	E15.5 * Wnt1 down vs E15.5 * Ctrl
10596072	3222402P14Rik	BC151170	0.00328814	-1.24786	E15.5 * Wnt1 down vs E15.5 * Ctrl
10413100	Myst4	NM_017479	0.000189254	-1.24798	E15.5 * Wnt1 down vs E15.5 * Ctrl
10467739	Avpi1	NM_027106	0.00115568	-1.24807	E15.5 * Wnt1 down vs E15.5 * Ctrl
10426889	Sec61b	NM_024171	0.000199864	-1.24817	E15.5 * Wnt1 down vs E15.5 * Ctrl
10403081	Wdr60	NM_146039	0.00425722	-1.24818	E15.5 * Wnt1 down vs E15.5 * Ctrl
10554475	Zfp592	NM_178707	0.00667865	-1.24826	E15.5 * Wnt1 down vs E15.5 * Ctrl
10548000	Ltbr	NM_010736	0.000312833	-1.24861	E15.5 * Wnt1 down vs E15.5 * Ctrl
10456439	Cep192	BC064462	0.00129174	-1.24862	E15.5 * Wnt1 down vs E15.5 * Ctrl
10483871	Ttn	NM_011652	4.03936e-005	-1.24881	E15.5 * Wnt1 down vs E15.5 * Ctrl
10433034	Atf7	NM_146065	0.00237003	-1.24891	E15.5 * Wnt1 down vs E15.5 * Ctrl
10570639	6820431F20Rik	BC025151	0.00929332	-1.24896	E15.5 * Wnt1 down vs E15.5 * Ctrl
10586039	Tle3	NM_001083927	0.00330663	-1.24912	E15.5 * Wnt1 down vs E15.5 * Ctrl
10583320	BC017612	NM_133214	0.00946106	-1.24917	E15.5 * Wnt1 down vs E15.5 * Ctrl
10461671	Patl1	NM_172635	0.00411238	-1.24918	E15.5 * Wnt1 down vs E15.5 * Ctrl
10546967	Sec13	NM_024206	0.00290604	-1.2497	E15.5 * Wnt1 down vs E15.5 * Ctrl
10382890	Sec14l1	NM_028777	0.0023854	-1.24973	E15.5 * Wnt1 down vs E15.5 * Ctrl
10490736	Rgs19	NM_026446	0.00967519	-1.25006	E15.5 * Wnt1 down vs E15.5 * Ctrl

10562637	Ccnb1	NM_172301	0.00369313	-1.25016	E15.5 * Wnt1 down vs E15.5 * Ctrl
10419568	Ear11	NM_053113	0.00920763	-1.25016	E15.5 * Wnt1 down vs E15.5 * Ctrl
10509122	Cnr2	NM_009924	0.00183061	-1.25027	E15.5 * Wnt1 down vs E15.5 * Ctrl
10397359	Batf	NM_016767	0.000245956	-1.25045	E15.5 * Wnt1 down vs E15.5 * Ctrl
10584309	BC024479	BC066002	0.00121986	-1.25048	E15.5 * Wnt1 down vs E15.5 * Ctrl
10545534	Rnf26	NM_153762	0.000372148	-1.25052	E15.5 * Wnt1 down vs E15.5 * Ctrl
10390103	Pdk2	NM_133667	0.00747791	-1.25076	E15.5 * Wnt1 down vs E15.5 * Ctrl
10572757	Rab8a	NM_023126	0.00683046	-1.25098	E15.5 * Wnt1 down vs E15.5 * Ctrl
10504831	Sec61b	NM_024171	1.6652e-005	-1.25101	E15.5 * Wnt1 down vs E15.5 * Ctrl
10491279	Prkci	NM_008857	0.00555506	-1.25113	E15.5 * Wnt1 down vs E15.5 * Ctrl
10529741	Rab28	NM_027295	0.000483544	-1.25122	E15.5 * Wnt1 down vs E15.5 * Ctrl
10463557	Gbf1	NM_178930	4.13433e-005	-1.25141	E15.5 * Wnt1 down vs E15.5 * Ctrl
10408656	Peci	NM_011868	0.0115157	-1.25145	E15.5 * Wnt1 down vs E15.5 * Ctrl
10469816	Il1rn	NM_031167	0.000177517	-1.25152	E15.5 * Wnt1 down vs E15.5 * Ctrl
10607694	Syap1	NM_025932	0.00321127	-1.25166	E15.5 * Wnt1 down vs E15.5 * Ctrl
10525043	Rbm19	NM_028762	0.00306097	-1.25185	E15.5 * Wnt1 down vs E15.5 * Ctrl
10389207	Ccl5	NM_013653	0.000612276	-1.25186	E15.5 * Wnt1 down vs E15.5 * Ctrl
10427744	Rai14	NM_030690	0.00377649	-1.25225	E15.5 * Wnt1 down vs E15.5 * Ctrl
10356172	5033414K04Rik	BC080290	0.00817301	-1.25232	E15.5 * Wnt1 down vs E15.5 * Ctrl
10377662	Ybx2	NM_016875	0.00445617	-1.25307	E15.5 * Wnt1 down vs E15.5 * Ctrl
10388488	Fam101b	ENSMUST00000(	0.0137297	-1.25312	E15.5 * Wnt1 down vs E15.5 * Ctrl
10497622	Lrrc34	NM_027941	0.000305051	-1.25338	E15.5 * Wnt1 down vs E15.5 * Ctrl
10530467	Nfxl1	NM_133921	0.0106272	-1.25361	E15.5 * Wnt1 down vs E15.5 * Ctrl
10360028	Fcgr2b	NM_001077189	0.00575427	-1.2537	E15.5 * Wnt1 down vs E15.5 * Ctrl
10593361	AU019823	NM_001134902	0.012984	-1.25437	E15.5 * Wnt1 down vs E15.5 * Ctrl
10528929	Hadha	NM_178878	0.000189906	-1.25453	E15.5 * Wnt1 down vs E15.5 * Ctrl
10418480	Gnl3	NM_153547	0.000949744	-1.25463	E15.5 * Wnt1 down vs E15.5 * Ctrl
10431894	Slc38a2	NM_175121	0.000601497	-1.25482	E15.5 * Wnt1 down vs E15.5 * Ctrl
10411739	Ccnb1	NM_172301	0.00208267	-1.25491	E15.5 * Wnt1 down vs E15.5 * Ctrl
10416709	Tdrd3	NM_172605	0.000298745	-1.25493	E15.5 * Wnt1 down vs E15.5 * Ctrl
10368041	3110003A17Rik	NM_028440	0.00178837	-1.25494	E15.5 * Wnt1 down vs E15.5 * Ctrl
10408212	Hist1h1e	NM_015787	0.00152009	-1.25501	E15.5 * Wnt1 down vs E15.5 * Ctrl
10494306	Mcl1	NM_008562	7.53104e-005	-1.25507	E15.5 * Wnt1 down vs E15.5 * Ctrl
10376324	OTTMUSG00000	NM_001135115	0.00771024	-1.25524	E15.5 * Wnt1 down vs E15.5 * Ctrl
10550365	Prkd2	NM_178900	0.00136823	-1.25569	E15.5 * Wnt1 down vs E15.5 * Ctrl
10576561	2310079N02Rik	NM_025636	0.0015686	-1.25583	E15.5 * Wnt1 down vs E15.5 * Ctrl
10458251	Sil1	NM_030749	0.00682158	-1.25653	E15.5 * Wnt1 down vs E15.5 * Ctrl
10595000	Tmod3	NM_016963	0.00499187	-1.25656	E15.5 * Wnt1 down vs E15.5 * Ctrl
10352294	BC031781	BC031781	0.0025345	-1.2566	E15.5 * Wnt1 down vs E15.5 * Ctrl
10431749	Adamts20	NM_177431	0.00911233	-1.2568	E15.5 * Wnt1 down vs E15.5 * Ctrl
10480155	Cubn	NM_001081084	0.0019073	-1.25687	E15.5 * Wnt1 down vs E15.5 * Ctrl
10538253	Mpp6	NM_019939	0.00153757	-1.25715	E15.5 * Wnt1 down vs E15.5 * Ctrl
10597432	Cnot10	NM_153585	0.000561239	-1.25745	E15.5 * Wnt1 down vs E15.5 * Ctrl
10376534	Mprip	NM_012027	0.000772503	-1.25765	E15.5 * Wnt1 down vs E15.5 * Ctrl
10554839	Picalm	NM_146194	4.27986e-006	-1.25769	E15.5 * Wnt1 down vs E15.5 * Ctrl
10377148	Myh8	NM_177369	0.00102777	-1.25839	E15.5 * Wnt1 down vs E15.5 * Ctrl
10436169	Ift57	NM_028680	0.00806092	-1.2584	E15.5 * Wnt1 down vs E15.5 * Ctrl
10552210	Ankrd27	NM_145633	0.0020859	-1.25844	E15.5 * Wnt1 down vs E15.5 * Ctrl
10567173	Pik3c2a	NM_011083	0.00590112	-1.25855	E15.5 * Wnt1 down vs E15.5 * Ctrl
10513583	Cdc26	NM_139291	0.000786446	-1.25862	E15.5 * Wnt1 down vs E15.5 * Ctrl
10513666	Akna	NM_001045514	7.54877e-005	-1.25876	E15.5 * Wnt1 down vs E15.5 * Ctrl
10396610	Mthfd1	NM_138745	0.00788468	-1.2588	E15.5 * Wnt1 down vs E15.5 * Ctrl
10399202	Macc1	ENSMUST00000(	0.00562442	-1.25898	E15.5 * Wnt1 down vs E15.5 * Ctrl
10360858	C130074G19Rik	NM_178692	0.00524056	-1.25912	E15.5 * Wnt1 down vs E15.5 * Ctrl
10380927	Grb7	NM_010346	0.00110192	-1.25924	E15.5 * Wnt1 down vs E15.5 * Ctrl
10566516	Rrp8	NM_025897	0.0103723	-1.25925	E15.5 * Wnt1 down vs E15.5 * Ctrl
10547830	Tpi1	NM_009415	7.31131e-005	-1.25949	E15.5 * Wnt1 down vs E15.5 * Ctrl
10507784	Ppt1	NM_008917	0.00462326	-1.25969	E15.5 * Wnt1 down vs E15.5 * Ctrl
10416785	4930517O19Rik	ENSMUST00000(	0.0118938	-1.2597	E15.5 * Wnt1 down vs E15.5 * Ctrl
10552311	OTTMUSG00000	XR_030737	0.00280954	-1.2598	E15.5 * Wnt1 down vs E15.5 * Ctrl
10607499	Phex	NM_011077	0.0131282	-1.2598	E15.5 * Wnt1 down vs E15.5 * Ctrl
10367728	Map3k7ip2	NM_138667	0.000947158	-1.26004	E15.5 * Wnt1 down vs E15.5 * Ctrl
10478572	Ube2c	NM_026785	0.00769005	-1.26023	E15.5 * Wnt1 down vs E15.5 * Ctrl
10579532	Bst2	NM_198095	0.00142809	-1.26102	E15.5 * Wnt1 down vs E15.5 * Ctrl
10603896	Klhl13	NM_026167	0.0010566	-1.26112	E15.5 * Wnt1 down vs E15.5 * Ctrl
10535084	3110082I17Rik	NM_028469	0.000329276	-1.26115	E15.5 * Wnt1 down vs E15.5 * Ctrl

10600341	Emd	NM_007927	0.00261483	-1.26134	E15.5 * Wnt1 down vs E15.5 * Ctrl
10394699	Rock2	NM_009072	0.00296155	-1.26163	E15.5 * Wnt1 down vs E15.5 * Ctrl
10578504	1700029J07Rik	NM_001033148	0.00650686	-1.26172	E15.5 * Wnt1 down vs E15.5 * Ctrl
10493711	Crtc2	NM_028881	0.00217266	-1.26182	E15.5 * Wnt1 down vs E15.5 * Ctrl
10398599	Rps19	NM_023133	0.000186057	-1.26185	E15.5 * Wnt1 down vs E15.5 * Ctrl
10445977	Ebi3	NM_015766	0.00262795	-1.26188	E15.5 * Wnt1 down vs E15.5 * Ctrl
10437934	Rpsa	NM_011029	0.00214027	-1.262	E15.5 * Wnt1 down vs E15.5 * Ctrl
10529028	2310016E02Rik	NM_001134692	0.00238024	-1.26204	E15.5 * Wnt1 down vs E15.5 * Ctrl
10435937	Cd200r3	NM_029018	0.00138079	-1.26238	E15.5 * Wnt1 down vs E15.5 * Ctrl
10582295	Odc1	NM_013614	0.000866071	-1.26277	E15.5 * Wnt1 down vs E15.5 * Ctrl
10377859	Pld2	NM_008876	0.000938551	-1.26289	E15.5 * Wnt1 down vs E15.5 * Ctrl
10448004	Phf10	NM_024250	5.60502e-006	-1.26317	E15.5 * Wnt1 down vs E15.5 * Ctrl
10414433	6720456H20Rik	NM_172600	0.000801087	-1.26356	E15.5 * Wnt1 down vs E15.5 * Ctrl
10414963	OTTMUSG00000	ENSMUST00000	0.00946348	-1.2636	E15.5 * Wnt1 down vs E15.5 * Ctrl
10372917	Tmem5	NM_153059	0.00903058	-1.26376	E15.5 * Wnt1 down vs E15.5 * Ctrl
10544798	Hoxa10	NM_008263	0.00228759	-1.26391	E15.5 * Wnt1 down vs E15.5 * Ctrl
10466888	Glis3	NM_175459	0.00602673	-1.26402	E15.5 * Wnt1 down vs E15.5 * Ctrl
10467766	Loxl4	ENSMUST00000	0.00923432	-1.26412	E15.5 * Wnt1 down vs E15.5 * Ctrl
10594103	2410076I21Rik	ENSMUST00000	0.00239278	-1.26426	E15.5 * Wnt1 down vs E15.5 * Ctrl
10456184	Apcdd1	NM_133237	0.0054918	-1.26433	E15.5 * Wnt1 down vs E15.5 * Ctrl
10414767	Rps19	NM_023133	5.62597e-006	-1.2645	E15.5 * Wnt1 down vs E15.5 * Ctrl
10414874	Rps19	NM_023133	5.62597e-006	-1.2645	E15.5 * Wnt1 down vs E15.5 * Ctrl
10518108	Tmem51	NM_145402	3.05377e-005	-1.26461	E15.5 * Wnt1 down vs E15.5 * Ctrl
10451434	Ppp2r5d	NM_009358	0.0010137	-1.26472	E15.5 * Wnt1 down vs E15.5 * Ctrl
10410477	Adamts16	NM_172053	0.00253488	-1.26483	E15.5 * Wnt1 down vs E15.5 * Ctrl
10375307	C1qtnf2	NM_026979	0.00165753	-1.26492	E15.5 * Wnt1 down vs E15.5 * Ctrl
10432957	Itgb7	NM_013566	0.000752734	-1.26502	E15.5 * Wnt1 down vs E15.5 * Ctrl
10560644	Pvrl2	NM_008990	0.00553861	-1.26504	E15.5 * Wnt1 down vs E15.5 * Ctrl
10395287	Atxn7l1	NM_028139	0.000174424	-1.26518	E15.5 * Wnt1 down vs E15.5 * Ctrl
10585358	Npat	NM_001081152	0.0062335	-1.26523	E15.5 * Wnt1 down vs E15.5 * Ctrl
10382542	Nup85	NM_001002929	0.000165174	-1.26524	E15.5 * Wnt1 down vs E15.5 * Ctrl
10455813	Lmnb1	NM_010721	0.000153819	-1.26564	E15.5 * Wnt1 down vs E15.5 * Ctrl
10586724	Narg2	NM_145618	0.00855744	-1.26566	E15.5 * Wnt1 down vs E15.5 * Ctrl
10596465	Acy1	NM_025371	0.000190638	-1.26614	E15.5 * Wnt1 down vs E15.5 * Ctrl
10570700	Defa25	NM_007849	0.00116428	-1.26628	E15.5 * Wnt1 down vs E15.5 * Ctrl
10375667	Rnf130	NM_021540	0.00273875	-1.2663	E15.5 * Wnt1 down vs E15.5 * Ctrl
10587262	Klhl31	NM_172925	0.00220502	-1.26673	E15.5 * Wnt1 down vs E15.5 * Ctrl
10495206	Slc16a4	NM_146136	0.00056965	-1.26674	E15.5 * Wnt1 down vs E15.5 * Ctrl
10598994	Atp1b3	NM_007502	0.000836445	-1.26694	E15.5 * Wnt1 down vs E15.5 * Ctrl
10413710	Nt5dc2	NM_027289	0.0137545	-1.26694	E15.5 * Wnt1 down vs E15.5 * Ctrl
10530089	Cckar	NM_009827	0.0146785	-1.26695	E15.5 * Wnt1 down vs E15.5 * Ctrl
10529581	Mrfap1	NM_026242	0.00122741	-1.26712	E15.5 * Wnt1 down vs E15.5 * Ctrl
10511755	Wwp1	NM_177327	0.00427267	-1.26712	E15.5 * Wnt1 down vs E15.5 * Ctrl
10431974	Rapgef3	NM_144850	0.00383219	-1.26752	E15.5 * Wnt1 down vs E15.5 * Ctrl
10399391	Gen1	NM_177331	0.0142465	-1.26808	E15.5 * Wnt1 down vs E15.5 * Ctrl
10501420	Stxbp3a	NM_011504	0.000304082	-1.26847	E15.5 * Wnt1 down vs E15.5 * Ctrl
10509127	Fuca1	NM_024243	0.00398273	-1.26847	E15.5 * Wnt1 down vs E15.5 * Ctrl
10378334	Tax1bp3	NM_029564	0.000212215	-1.26872	E15.5 * Wnt1 down vs E15.5 * Ctrl
10531370	Naaa	NM_025972	0.00361984	-1.26875	E15.5 * Wnt1 down vs E15.5 * Ctrl
10465366	Zfpl1	NM_024231	0.0120606	-1.2688	E15.5 * Wnt1 down vs E15.5 * Ctrl
10552874	Tead2	NM_011565	0.013302	-1.26911	E15.5 * Wnt1 down vs E15.5 * Ctrl
10484999	Ddb2	NM_028119	0.000429662	-1.26922	E15.5 * Wnt1 down vs E15.5 * Ctrl
10391762	Gjc1	NM_001159382	6.94666e-007	-1.26929	E15.5 * Wnt1 down vs E15.5 * Ctrl
10427125	Igfbp6	NM_008344	0.00511367	-1.26933	E15.5 * Wnt1 down vs E15.5 * Ctrl
10358330	BC026782	NM_001025575	0.000368939	-1.26936	E15.5 * Wnt1 down vs E15.5 * Ctrl
10446441	Ddx11	NM_001003919	0.00155483	-1.26959	E15.5 * Wnt1 down vs E15.5 * Ctrl
10430904		---	0.0139638	-1.2696	E15.5 * Wnt1 down vs E15.5 * Ctrl
10559509	Cdc42ep5	NM_021454	0.0110937	-1.26975	E15.5 * Wnt1 down vs E15.5 * Ctrl
10437698	Txndc11	NM_029582	0.00137092	-1.26983	E15.5 * Wnt1 down vs E15.5 * Ctrl
10375497		---	0.00817772	-1.26996	E15.5 * Wnt1 down vs E15.5 * Ctrl
10394477	Laptm4a	NM_008640	3.41035e-006	-1.27011	E15.5 * Wnt1 down vs E15.5 * Ctrl
10472724	Gorasp2	NM_027352	0.00105514	-1.27017	E15.5 * Wnt1 down vs E15.5 * Ctrl
10456727	Dym	NM_027727	0.00109496	-1.27017	E15.5 * Wnt1 down vs E15.5 * Ctrl
10481949	Traf1	NM_009421	0.00316846	-1.27071	E15.5 * Wnt1 down vs E15.5 * Ctrl
10391301	Stat3	NM_213659	0.0034784	-1.27073	E15.5 * Wnt1 down vs E15.5 * Ctrl
10411751	Slc30a5	NM_022885	0.00448685	-1.27086	E15.5 * Wnt1 down vs E15.5 * Ctrl

10511843	1810074P20Rik	NM_026194	0.000976244	-1.27096	E15.5 * Wnt1 down vs E15.5 * Ctrl
10538970	Krcc1	NM_145568	0.00788202	-1.2711	E15.5 * Wnt1 down vs E15.5 * Ctrl
10497209	Mrps28	NM_025434	0.00728025	-1.2711	E15.5 * Wnt1 down vs E15.5 * Ctrl
10481931	Phf19	NM_028716	0.00172195	-1.2712	E15.5 * Wnt1 down vs E15.5 * Ctrl
10352048	Exo1	NM_012012	0.00104401	-1.27122	E15.5 * Wnt1 down vs E15.5 * Ctrl
10507328	Prdx1	NM_011034	0.00338048	-1.27124	E15.5 * Wnt1 down vs E15.5 * Ctrl
10539766	Aak1	NM_001040106	4.02672e-005	-1.27137	E15.5 * Wnt1 down vs E15.5 * Ctrl
10522627	Cep135	NM_199032	0.00102916	-1.27142	E15.5 * Wnt1 down vs E15.5 * Ctrl
10365749	Lta4h	NM_008517	0.000197189	-1.27161	E15.5 * Wnt1 down vs E15.5 * Ctrl
10518805	Vamp3	NM_009498	0.00219331	-1.27178	E15.5 * Wnt1 down vs E15.5 * Ctrl
10586484	Fam96a	NM_026635	0.00198207	-1.27218	E15.5 * Wnt1 down vs E15.5 * Ctrl
10583145	Tmem123	NM_133739	0.000346003	-1.27233	E15.5 * Wnt1 down vs E15.5 * Ctrl
10436779	100040496	XR_031827	0.0108345	-1.27262	E15.5 * Wnt1 down vs E15.5 * Ctrl
10535780	Flt3	NM_010229	0.00332167	-1.27272	E15.5 * Wnt1 down vs E15.5 * Ctrl
10582390	Aprt	NM_009698	0.000181591	-1.27279	E15.5 * Wnt1 down vs E15.5 * Ctrl
10497321	Pgam1	NM_023418	0.00106032	-1.27346	E15.5 * Wnt1 down vs E15.5 * Ctrl
10518561	Apitd1	NM_027263	0.0141563	-1.27352	E15.5 * Wnt1 down vs E15.5 * Ctrl
10528598	Fastk	NM_023229	0.00231128	-1.27357	E15.5 * Wnt1 down vs E15.5 * Ctrl
10538901	BC005685	BC005685	0.000140664	-1.27363	E15.5 * Wnt1 down vs E15.5 * Ctrl
10463404	Fam178a	NM_001081225	0.000125312	-1.27378	E15.5 * Wnt1 down vs E15.5 * Ctrl
10452648	Emilin2	NM_145158	0.0128104	-1.27399	E15.5 * Wnt1 down vs E15.5 * Ctrl
10358299	EG214403	NM_001029977	0.000791872	-1.27417	E15.5 * Wnt1 down vs E15.5 * Ctrl
10459183	Slc26a2	NM_007885	0.00180091	-1.27435	E15.5 * Wnt1 down vs E15.5 * Ctrl
10491805	Plk4	NM_011495	0.000320927	-1.27463	E15.5 * Wnt1 down vs E15.5 * Ctrl
10410695	Rhobtb3	NM_028493	6.92611e-005	-1.2749	E15.5 * Wnt1 down vs E15.5 * Ctrl
10447880	Mrpl18	NM_026310	0.00176549	-1.27527	E15.5 * Wnt1 down vs E15.5 * Ctrl
10596492	Parp3	NM_145619	0.0010066	-1.27535	E15.5 * Wnt1 down vs E15.5 * Ctrl
10498477	E130311K13Rik	NM_177856	0.000875949	-1.27537	E15.5 * Wnt1 down vs E15.5 * Ctrl
10500445	Chd1l	NM_026539	0.00633505	-1.27544	E15.5 * Wnt1 down vs E15.5 * Ctrl
10475665	Usp8	NM_019729	6.66789e-006	-1.27549	E15.5 * Wnt1 down vs E15.5 * Ctrl
10479971	ENSMUSG0000C	ENSMUST00000	0.00720192	-1.27556	E15.5 * Wnt1 down vs E15.5 * Ctrl
10540738	Fancd2	NM_001033244	0.00181518	-1.27575	E15.5 * Wnt1 down vs E15.5 * Ctrl
10354432	Myo1b	NM_010863	1.12705e-005	-1.2759	E15.5 * Wnt1 down vs E15.5 * Ctrl
10584057	Zbtb44	NM_001115130	0.00451175	-1.27594	E15.5 * Wnt1 down vs E15.5 * Ctrl
10453939	Lama3	NM_010680	0.00102969	-1.27622	E15.5 * Wnt1 down vs E15.5 * Ctrl
10411949	Rgs7bp	NM_029879	0.00239403	-1.27624	E15.5 * Wnt1 down vs E15.5 * Ctrl
10607183	Lhfpl1	NM_178358	0.00817574	-1.27639	E15.5 * Wnt1 down vs E15.5 * Ctrl
10471139	Hmcn2	ENSMUST00000(	0.000180588	-1.27643	E15.5 * Wnt1 down vs E15.5 * Ctrl
10515257	Rad54l	NM_009015	0.00122205	-1.27646	E15.5 * Wnt1 down vs E15.5 * Ctrl
10475394	Ctdspl2	NM_212450	0.0043883	-1.2766	E15.5 * Wnt1 down vs E15.5 * Ctrl
10384985	Rhbdf1	NM_010117	0.00836781	-1.2766	E15.5 * Wnt1 down vs E15.5 * Ctrl
10476817	Atpaf1	NM_181040	0.0111425	-1.27665	E15.5 * Wnt1 down vs E15.5 * Ctrl
10437311	Trap1	NM_026508	0.000219278	-1.27677	E15.5 * Wnt1 down vs E15.5 * Ctrl
10360227	Pea15a	NM_011063	0.00171438	-1.27683	E15.5 * Wnt1 down vs E15.5 * Ctrl
10360105	Usp21	NM_013919	0.00211865	-1.27693	E15.5 * Wnt1 down vs E15.5 * Ctrl
10354542	Pms1	NM_153556	0.000994744	-1.27698	E15.5 * Wnt1 down vs E15.5 * Ctrl
10387807	0610010K14Rik	NM_026757	0.00595589	-1.27708	E15.5 * Wnt1 down vs E15.5 * Ctrl
10539882	Ruvbl1	NM_019685	0.000232796	-1.27714	E15.5 * Wnt1 down vs E15.5 * Ctrl
10500362	Polr3gl	NM_027241	0.0136756	-1.27717	E15.5 * Wnt1 down vs E15.5 * Ctrl
10502787	Rpsa	NM_011029	0.0013579	-1.27735	E15.5 * Wnt1 down vs E15.5 * Ctrl
10570344	Lamp1	NM_010684	0.000175144	-1.27751	E15.5 * Wnt1 down vs E15.5 * Ctrl
10490289		---	0.0086899	-1.2776	E15.5 * Wnt1 down vs E15.5 * Ctrl
10462406	C030046E11Rik	NM_001081319	0.00391339	-1.27775	E15.5 * Wnt1 down vs E15.5 * Ctrl
10571621	Ufsp2	NM_138668	0.00370009	-1.27787	E15.5 * Wnt1 down vs E15.5 * Ctrl
10601416	P2ry10	NM_172435	0.00999781	-1.27794	E15.5 * Wnt1 down vs E15.5 * Ctrl
10568131	2900092E17Rik	NM_030240	0.00132977	-1.27804	E15.5 * Wnt1 down vs E15.5 * Ctrl
10578203	Cldn23	NM_027998	0.00412197	-1.27812	E15.5 * Wnt1 down vs E15.5 * Ctrl
10505894	Mtap	NM_024433	0.000657165	-1.27861	E15.5 * Wnt1 down vs E15.5 * Ctrl
10471677	Dab2ip	NM_001114124	7.95766e-005	-1.27863	E15.5 * Wnt1 down vs E15.5 * Ctrl
10504757	BC005685	BC005685	0.00357351	-1.27882	E15.5 * Wnt1 down vs E15.5 * Ctrl
10482814	Acvr1c	NM_001111030	0.00922394	-1.27886	E15.5 * Wnt1 down vs E15.5 * Ctrl
10440647	Cldn8	NM_018778	0.00583868	-1.27908	E15.5 * Wnt1 down vs E15.5 * Ctrl
10445347	Clic5	NM_172621	0.0121417	-1.2791	E15.5 * Wnt1 down vs E15.5 * Ctrl
10540122	Slc6a6	NM_009320	0.000800401	-1.27916	E15.5 * Wnt1 down vs E15.5 * Ctrl
10444244	Tap1	NM_013683	0.00350195	-1.2793	E15.5 * Wnt1 down vs E15.5 * Ctrl
10462853	Cyp26a1	NM_007811	0.00314402	-1.27937	E15.5 * Wnt1 down vs E15.5 * Ctrl

10513362	OTTMUSG00000	ENSMUST00000	0.00516811	-1.27948	E15.5 * Wnt1 down vs E15.5 * Ctrl
10534935	Pilrb1	NM_133209	0.00294348	-1.27954	E15.5 * Wnt1 down vs E15.5 * Ctrl
10560459	Vasp	NM_009499	5.85198e-005	-1.27957	E15.5 * Wnt1 down vs E15.5 * Ctrl
10564343	Tjp1	NM_009386	1.34167e-006	-1.27963	E15.5 * Wnt1 down vs E15.5 * Ctrl
10554281	Fanci	NM_145946	0.00364829	-1.27967	E15.5 * Wnt1 down vs E15.5 * Ctrl
10470950	Endog	NM_007931	0.00950425	-1.27974	E15.5 * Wnt1 down vs E15.5 * Ctrl
10495549	Dbt	NM_010022	0.00299783	-1.2799	E15.5 * Wnt1 down vs E15.5 * Ctrl
10391625	Ubtf	NM_011551	0.000771246	-1.27991	E15.5 * Wnt1 down vs E15.5 * Ctrl
10514255	Mllt3	NM_027326	2.85898e-005	-1.28003	E15.5 * Wnt1 down vs E15.5 * Ctrl
10407049	3830408C21Rik	ENSMUST00000	0.010103	-1.28016	E15.5 * Wnt1 down vs E15.5 * Ctrl
10501051	Cept1	NM_133869	0.0018017	-1.28017	E15.5 * Wnt1 down vs E15.5 * Ctrl
10472440	Tax1bp3	NM_029564	0.000105786	-1.28032	E15.5 * Wnt1 down vs E15.5 * Ctrl
10536996	Klhdc10	NM_029742	0.00891185	-1.28054	E15.5 * Wnt1 down vs E15.5 * Ctrl
10350838	2810417H13Rik	NM_026515	4.39413e-005	-1.28058	E15.5 * Wnt1 down vs E15.5 * Ctrl
10593050	Il10ra	NM_008348	0.0094271	-1.28067	E15.5 * Wnt1 down vs E15.5 * Ctrl
10388033	Derl2	NM_033562	0.0114848	-1.2807	E15.5 * Wnt1 down vs E15.5 * Ctrl
10466712	Mamdc2	NM_174857	0.00517805	-1.28073	E15.5 * Wnt1 down vs E15.5 * Ctrl
10404731	Tmem14c	NM_025387	0.000562833	-1.28073	E15.5 * Wnt1 down vs E15.5 * Ctrl
10423030	Prlr	NM_011169	5.0897e-005	-1.28121	E15.5 * Wnt1 down vs E15.5 * Ctrl
10528664	Smarcd3	NM_025891	0.00468323	-1.28123	E15.5 * Wnt1 down vs E15.5 * Ctrl
10485562	Hipk3	NM_010434	0.00037775	-1.28132	E15.5 * Wnt1 down vs E15.5 * Ctrl
10464836	Actn3	NM_013456	0.000288397	-1.28148	E15.5 * Wnt1 down vs E15.5 * Ctrl
10421309	Slc39a14	NM_001135151	0.000999739	-1.28175	E15.5 * Wnt1 down vs E15.5 * Ctrl
10451123	Slc29a1	NM_022880	0.000746865	-1.28177	E15.5 * Wnt1 down vs E15.5 * Ctrl
10594825	Aqp9	NM_022026	6.32172e-005	-1.28177	E15.5 * Wnt1 down vs E15.5 * Ctrl
10389143	Slfn8	NM_181545	0.0028922	-1.28204	E15.5 * Wnt1 down vs E15.5 * Ctrl
10471108	Gpr107	NM_178760	0.0044804	-1.2823	E15.5 * Wnt1 down vs E15.5 * Ctrl
10360003	Dusp12	NM_023173	0.000226038	-1.28243	E15.5 * Wnt1 down vs E15.5 * Ctrl
10588154	Stag1	NM_009282	0.000851413	-1.28249	E15.5 * Wnt1 down vs E15.5 * Ctrl
10433782	Efcab1	NM_025769	0.00774232	-1.28287	E15.5 * Wnt1 down vs E15.5 * Ctrl
10457400		---	0.00122308	-1.28293	E15.5 * Wnt1 down vs E15.5 * Ctrl
10384223	Igfbp3	NM_008343	0.0124307	-1.28336	E15.5 * Wnt1 down vs E15.5 * Ctrl
10527694	B3galtl	NM_001081204	0.000227627	-1.28351	E15.5 * Wnt1 down vs E15.5 * Ctrl
10360443	Fh1	NM_010209	0.00237011	-1.28406	E15.5 * Wnt1 down vs E15.5 * Ctrl
10519105	Ski	NM_011385	3.91496e-005	-1.28425	E15.5 * Wnt1 down vs E15.5 * Ctrl
10395155	Fam110c	BC085288	0.00158666	-1.28429	E15.5 * Wnt1 down vs E15.5 * Ctrl
10593483	Fdx1	NM_007996	0.00523226	-1.28442	E15.5 * Wnt1 down vs E15.5 * Ctrl
10569113	Mupcdh	NM_001114322	0.0041276	-1.28445	E15.5 * Wnt1 down vs E15.5 * Ctrl
10497831	Ccna2	NM_009828	4.52085e-005	-1.28463	E15.5 * Wnt1 down vs E15.5 * Ctrl
10445119	H2-M3	NM_013819	0.00781143	-1.28478	E15.5 * Wnt1 down vs E15.5 * Ctrl
10582868	Sp110	NM_175397	0.00451769	-1.28491	E15.5 * Wnt1 down vs E15.5 * Ctrl
10474221	ENSMUSG00000	ENSMUST00000	0.0086475	-1.28504	E15.5 * Wnt1 down vs E15.5 * Ctrl
10363007	Ascc3	NM_001146089	2.03728e-006	-1.28507	E15.5 * Wnt1 down vs E15.5 * Ctrl
10566822	Scube2	NM_020052	0.00024498	-1.28507	E15.5 * Wnt1 down vs E15.5 * Ctrl
10606362		---	0.00204677	-1.28508	E15.5 * Wnt1 down vs E15.5 * Ctrl
10359648	Scyl3	NM_028776	1.3807e-005	-1.28538	E15.5 * Wnt1 down vs E15.5 * Ctrl
10451551	Guca1a	NM_008189	0.00276973	-1.28542	E15.5 * Wnt1 down vs E15.5 * Ctrl
10392241	Polg2	NM_015810	0.00998535	-1.28544	E15.5 * Wnt1 down vs E15.5 * Ctrl
10366293	Csrp2	NM_007792	0.00361711	-1.28554	E15.5 * Wnt1 down vs E15.5 * Ctrl
10441107	Psmg1	NM_019537	0.00148745	-1.28578	E15.5 * Wnt1 down vs E15.5 * Ctrl
10414250	4930503E14Rik	NM_029131	0.0128085	-1.28587	E15.5 * Wnt1 down vs E15.5 * Ctrl
10408321	Gmnn	NM_020567	0.00949581	-1.28604	E15.5 * Wnt1 down vs E15.5 * Ctrl
10590494	Kif15	NM_010620	0.00198265	-1.28619	E15.5 * Wnt1 down vs E15.5 * Ctrl
10552469	Klk13	NM_001039042	0.000130576	-1.28634	E15.5 * Wnt1 down vs E15.5 * Ctrl
10355327	Bard1	NM_007525	0.01013	-1.28638	E15.5 * Wnt1 down vs E15.5 * Ctrl
10585318	Rdx	NM_001104617	0.00024358	-1.28658	E15.5 * Wnt1 down vs E15.5 * Ctrl
10577030	Cars2	NM_024248	0.000141124	-1.28672	E15.5 * Wnt1 down vs E15.5 * Ctrl
10407145	Mier3	NM_172593	0.000481483	-1.28677	E15.5 * Wnt1 down vs E15.5 * Ctrl
10429754	Nrbp2	NM_144847	0.00103636	-1.28688	E15.5 * Wnt1 down vs E15.5 * Ctrl
10553403	Htatip2	NM_001146049	0.00133271	-1.28714	E15.5 * Wnt1 down vs E15.5 * Ctrl
10391461	Brca1	NM_009764	0.000923098	-1.28734	E15.5 * Wnt1 down vs E15.5 * Ctrl
10534667	Serpine1	NM_008871	0.00446417	-1.28735	E15.5 * Wnt1 down vs E15.5 * Ctrl
10383423	Anapc11	NM_025389	0.000284508	-1.28739	E15.5 * Wnt1 down vs E15.5 * Ctrl
10455533	Eif1a	NM_010120	0.00103977	-1.28748	E15.5 * Wnt1 down vs E15.5 * Ctrl
10591563	Kank2	NM_145611	0.0139665	-1.28824	E15.5 * Wnt1 down vs E15.5 * Ctrl
10352717	Batf3	NM_030060	0.00521113	-1.28828	E15.5 * Wnt1 down vs E15.5 * Ctrl

10466190	Ms4a14	ENSMUST00000(	0.00685946	-1.28844	E15.5 * Wnt1 down vs E15.5 * Ctrl
10528804	ENSMUSG0000C	NM_001134299	0.00310432	-1.28884	E15.5 * Wnt1 down vs E15.5 * Ctrl
10450957	Cenpq	NM_031863	0.0132135	-1.28902	E15.5 * Wnt1 down vs E15.5 * Ctrl
10452356	Gpr108	NM_030084	0.002324	-1.28923	E15.5 * Wnt1 down vs E15.5 * Ctrl
10412830	Ngly1	NM_021504	0.00162472	-1.28931	E15.5 * Wnt1 down vs E15.5 * Ctrl
10587023	Rab27a	NM_023635	0.000156064	-1.28955	E15.5 * Wnt1 down vs E15.5 * Ctrl
10450819	Znrd1	NM_023162	0.00600541	-1.28955	E15.5 * Wnt1 down vs E15.5 * Ctrl
10451786	Efhb	NM_172497	0.00232847	-1.28964	E15.5 * Wnt1 down vs E15.5 * Ctrl
10412981	Sec24c	NM_172596	0.00108804	-1.28981	E15.5 * Wnt1 down vs E15.5 * Ctrl
10408455	Cdkal1	NM_144536	0.00109898	-1.29011	E15.5 * Wnt1 down vs E15.5 * Ctrl
10539933	Txnrd3	NM_153162	0.0128091	-1.29064	E15.5 * Wnt1 down vs E15.5 * Ctrl
10344960	Tmem70	NM_027415	0.00567182	-1.29068	E15.5 * Wnt1 down vs E15.5 * Ctrl
10593430	Sik2	NM_178710	0.000501364	-1.29079	E15.5 * Wnt1 down vs E15.5 * Ctrl
10381809	Itgb3	NM_016780	0.00818589	-1.29095	E15.5 * Wnt1 down vs E15.5 * Ctrl
10351404	Tmco1	NM_001039483	0.00139215	-1.29122	E15.5 * Wnt1 down vs E15.5 * Ctrl
10534974	Mcm7	NM_008568	0.00804461	-1.2913	E15.5 * Wnt1 down vs E15.5 * Ctrl
10565218	Il16	NM_010551	0.00108451	-1.29142	E15.5 * Wnt1 down vs E15.5 * Ctrl
10448803	Hn1l	NM_198937	0.000202615	-1.29156	E15.5 * Wnt1 down vs E15.5 * Ctrl
10557992	Bag3	NM_013863	0.00121601	-1.29199	E15.5 * Wnt1 down vs E15.5 * Ctrl
10469867	Pnpla7	NM_146251	0.000623544	-1.29228	E15.5 * Wnt1 down vs E15.5 * Ctrl
10444780	H2-D1	NM_010380	0.00386949	-1.2923	E15.5 * Wnt1 down vs E15.5 * Ctrl
10391052	Krt14	NM_016958	0.0106269	-1.29246	E15.5 * Wnt1 down vs E15.5 * Ctrl
10384940	4933407N01Rik	NM_025745	0.000419534	-1.29249	E15.5 * Wnt1 down vs E15.5 * Ctrl
10451679	Daam2	NM_001008231	0.00483228	-1.29253	E15.5 * Wnt1 down vs E15.5 * Ctrl
10520187	Slc4a2	NM_009207	0.000107334	-1.29269	E15.5 * Wnt1 down vs E15.5 * Ctrl
10417070	Ipo5	NM_023579	1.80157e-008	-1.29275	E15.5 * Wnt1 down vs E15.5 * Ctrl
10455970	BC023105	BC023105	0.00143699	-1.29282	E15.5 * Wnt1 down vs E15.5 * Ctrl
10549382	EG545893	NM_001034903	0.002931	-1.2929	E15.5 * Wnt1 down vs E15.5 * Ctrl
10430032	Nfkbil2	NM_183091	0.00317932	-1.2932	E15.5 * Wnt1 down vs E15.5 * Ctrl
10409833	Gas1	NM_008086	0.000205118	-1.2932	E15.5 * Wnt1 down vs E15.5 * Ctrl
10524790	Cit	NM_007708	0.00127286	-1.29336	E15.5 * Wnt1 down vs E15.5 * Ctrl
10491721	Spry1	NM_011896	0.0015939	-1.29339	E15.5 * Wnt1 down vs E15.5 * Ctrl
10364361	Icosl	NM_015790	0.000189071	-1.29342	E15.5 * Wnt1 down vs E15.5 * Ctrl
10597493	Stt3b	NM_024222	2.10223e-006	-1.29376	E15.5 * Wnt1 down vs E15.5 * Ctrl
10503551	Usp45	NM_152825	0.00287722	-1.29387	E15.5 * Wnt1 down vs E15.5 * Ctrl
10572747	Tpm4	NM_001001491	1.24115e-006	-1.2943	E15.5 * Wnt1 down vs E15.5 * Ctrl
10501699	Agl	NM_001081326	0.000271509	-1.29464	E15.5 * Wnt1 down vs E15.5 * Ctrl
10567299	Itpripl2	NM_001033380	0.0060095	-1.2948	E15.5 * Wnt1 down vs E15.5 * Ctrl
10514732	Slc35d1	NM_177732	0.00489327	-1.29496	E15.5 * Wnt1 down vs E15.5 * Ctrl
10498576	Lxn	NM_016753	0.00466894	-1.2951	E15.5 * Wnt1 down vs E15.5 * Ctrl
10596207	Uba5	NM_025692	0.000292924	-1.29522	E15.5 * Wnt1 down vs E15.5 * Ctrl
10482500	Rnd3	NM_028810	0.000516792	-1.29526	E15.5 * Wnt1 down vs E15.5 * Ctrl
10529445	Lrpap1	NM_013587	0.000133195	-1.2954	E15.5 * Wnt1 down vs E15.5 * Ctrl
10357391	Zranb3	NM_027678	0.000201437	-1.29542	E15.5 * Wnt1 down vs E15.5 * Ctrl
10458090	Reep5	NM_007874	0.00286148	-1.29553	E15.5 * Wnt1 down vs E15.5 * Ctrl
10520521	Cenpa	NM_007681	0.000476851	-1.29565	E15.5 * Wnt1 down vs E15.5 * Ctrl
10432636	BC004728	NM_174992	0.0103757	-1.29572	E15.5 * Wnt1 down vs E15.5 * Ctrl
10473880	Lrp4	NM_172668	0.00212748	-1.29591	E15.5 * Wnt1 down vs E15.5 * Ctrl
10600547	Tbl1x	NM_020601	1.05209e-005	-1.29605	E15.5 * Wnt1 down vs E15.5 * Ctrl
10405785	0610007P08Rik	NM_023507	0.00483332	-1.29627	E15.5 * Wnt1 down vs E15.5 * Ctrl
10379215	Ift20	NM_018854	0.000378603	-1.29632	E15.5 * Wnt1 down vs E15.5 * Ctrl
10393364	Cygb	NM_030206	0.00195401	-1.29643	E15.5 * Wnt1 down vs E15.5 * Ctrl
10537157	2310005E10Rik	NM_172398	0.000971575	-1.29648	E15.5 * Wnt1 down vs E15.5 * Ctrl
10359181	Tor3a	NM_023141	0.0010212	-1.2966	E15.5 * Wnt1 down vs E15.5 * Ctrl
10503711	Casp8ap2	NM_011997	0.000140344	-1.29671	E15.5 * Wnt1 down vs E15.5 * Ctrl
10462195	Kank1	NM_181404	0.00240124	-1.29697	E15.5 * Wnt1 down vs E15.5 * Ctrl
10534395	Cldn4	NM_009903	0.00989087	-1.29734	E15.5 * Wnt1 down vs E15.5 * Ctrl
10377605	1810027O10Rik	BC117077	0.00727468	-1.29738	E15.5 * Wnt1 down vs E15.5 * Ctrl
10574456	Cklf	NM_001037841	0.00842116	-1.29739	E15.5 * Wnt1 down vs E15.5 * Ctrl
10492180	Rnf13	NM_001113413	0.0127908	-1.29756	E15.5 * Wnt1 down vs E15.5 * Ctrl
10357604	Ikbke	NM_019777	0.00237586	-1.29796	E15.5 * Wnt1 down vs E15.5 * Ctrl
10525665	Ccdc62	NM_001134767	0.00995281	-1.29796	E15.5 * Wnt1 down vs E15.5 * Ctrl
10459772	Lipg	NM_010720	0.000924841	-1.298	E15.5 * Wnt1 down vs E15.5 * Ctrl
10476889	Nxt1	NM_001110159	0.00555981	-1.29817	E15.5 * Wnt1 down vs E15.5 * Ctrl
10360806	Capn2	NM_009794	0.00243903	-1.29821	E15.5 * Wnt1 down vs E15.5 * Ctrl
10416533	Ccdc122	NM_175369	0.00146103	-1.29826	E15.5 * Wnt1 down vs E15.5 * Ctrl

10476237	Hspa12b	NM_028306	0.0039107	-1.29876	E15.5 * Wnt1 down vs E15.5 * Ctrl
10471608	Cep110	NM_012018	0.00017932	-1.29877	E15.5 * Wnt1 down vs E15.5 * Ctrl
10460666	Ltbp3	NM_008520	0.00710566	-1.29889	E15.5 * Wnt1 down vs E15.5 * Ctrl
10516277	1700029G01Rik	BC053421	0.00345195	-1.29908	E15.5 * Wnt1 down vs E15.5 * Ctrl
10445826	Mocs1	NM_020042	0.0053883	-1.29909	E15.5 * Wnt1 down vs E15.5 * Ctrl
10443949	Adamts10	NM_172619	0.0102135	-1.2991	E15.5 * Wnt1 down vs E15.5 * Ctrl
10445839	Kif6	NM_177052	0.00364362	-1.29968	E15.5 * Wnt1 down vs E15.5 * Ctrl
10559200	Tnni2	NM_009405	0.000641975	-1.29973	E15.5 * Wnt1 down vs E15.5 * Ctrl
10439744	Cd96	NM_032465	0.000891598	-1.29973	E15.5 * Wnt1 down vs E15.5 * Ctrl
10605315	Lage3	NM_025410	0.0127954	-1.30021	E15.5 * Wnt1 down vs E15.5 * Ctrl
10369647	Ddx50	NM_053183	5.17227e-005	-1.30027	E15.5 * Wnt1 down vs E15.5 * Ctrl
10365899	Ccdc41	NM_029852	0.00123017	-1.30036	E15.5 * Wnt1 down vs E15.5 * Ctrl
10420241	Mcpt9	NM_010782	0.00195026	-1.30042	E15.5 * Wnt1 down vs E15.5 * Ctrl
10570837	Slc20a2	NM_011394	0.0116745	-1.30062	E15.5 * Wnt1 down vs E15.5 * Ctrl
10444858	Cchcr1	NM_146248	0.000387708	-1.30064	E15.5 * Wnt1 down vs E15.5 * Ctrl
10459944	Nfatc1	NM_198429	0.000644855	-1.30066	E15.5 * Wnt1 down vs E15.5 * Ctrl
10434105	Scarf2	NM_153790	0.00534881	-1.30068	E15.5 * Wnt1 down vs E15.5 * Ctrl
10596454	Alas1	NM_020559	0.0055448	-1.30074	E15.5 * Wnt1 down vs E15.5 * Ctrl
10446771	Lclat1	NM_001081071	0.000136371	-1.30085	E15.5 * Wnt1 down vs E15.5 * Ctrl
10390707	Top2a	NM_011623	9.76572e-006	-1.30114	E15.5 * Wnt1 down vs E15.5 * Ctrl
10456566	Mbd2	NM_010773	0.00113971	-1.30173	E15.5 * Wnt1 down vs E15.5 * Ctrl
10409799	Isca1	NM_026921	8.61908e-006	-1.30176	E15.5 * Wnt1 down vs E15.5 * Ctrl
10348354	Ugt1a9	NM_201644	0.000144191	-1.30181	E15.5 * Wnt1 down vs E15.5 * Ctrl
10539008	Vps24	NM_025783	0.00258016	-1.30181	E15.5 * Wnt1 down vs E15.5 * Ctrl
10398368		---	0.00696332	-1.30206	E15.5 * Wnt1 down vs E15.5 * Ctrl
10477090	Tbc1d20	NM_024196	2.05516e-006	-1.30208	E15.5 * Wnt1 down vs E15.5 * Ctrl
10451458	Cnpy3	NM_028065	0.00571301	-1.30255	E15.5 * Wnt1 down vs E15.5 * Ctrl
10492330	P2ry1	NM_008772	0.00333796	-1.30273	E15.5 * Wnt1 down vs E15.5 * Ctrl
10495107	Adora3	NM_009631	0.00423605	-1.30288	E15.5 * Wnt1 down vs E15.5 * Ctrl
10600476	EG245472	XM_141845	0.0101369	-1.30292	E15.5 * Wnt1 down vs E15.5 * Ctrl
10371379	Nuak1	NM_001004363	0.00653875	-1.30307	E15.5 * Wnt1 down vs E15.5 * Ctrl
10494238	Arnt	NM_001037737	8.09886e-006	-1.30356	E15.5 * Wnt1 down vs E15.5 * Ctrl
10399148	Rapgef5	NM_175930	0.0103503	-1.30377	E15.5 * Wnt1 down vs E15.5 * Ctrl
10434229	Cldn5	NM_013805	0.00259744	-1.30428	E15.5 * Wnt1 down vs E15.5 * Ctrl
10522301	Grxcr1	AY616753	0.00150611	-1.30437	E15.5 * Wnt1 down vs E15.5 * Ctrl
10403076		---	0.0011307	-1.30438	E15.5 * Wnt1 down vs E15.5 * Ctrl
10516735	Tinagl1	NM_023476	0.00109859	-1.30469	E15.5 * Wnt1 down vs E15.5 * Ctrl
10559248	Tspan32	NM_020286	0.000116861	-1.30474	E15.5 * Wnt1 down vs E15.5 * Ctrl
10605421	Mtcp1	NM_001039373	3.68816e-005	-1.30502	E15.5 * Wnt1 down vs E15.5 * Ctrl
10583732	Ldlr	NM_010700	0.00257912	-1.30509	E15.5 * Wnt1 down vs E15.5 * Ctrl
10376021	08-Sep	NM_033144	0.00135143	-1.30559	E15.5 * Wnt1 down vs E15.5 * Ctrl
10473250	Mrpl18	NM_026310	0.00263673	-1.30561	E15.5 * Wnt1 down vs E15.5 * Ctrl
10508382	Ak2	NM_016895	0.000159419	-1.30565	E15.5 * Wnt1 down vs E15.5 * Ctrl
10375838	Col23a1	NM_153393	0.00524909	-1.30589	E15.5 * Wnt1 down vs E15.5 * Ctrl
10531383	Sdad1	NM_172713	0.000246832	-1.30593	E15.5 * Wnt1 down vs E15.5 * Ctrl
10425501		---	0.00696029	-1.30595	E15.5 * Wnt1 down vs E15.5 * Ctrl
10481627	Lcn2	NM_008491	0.000127293	-1.30619	E15.5 * Wnt1 down vs E15.5 * Ctrl
10426875	Atf1	NM_007497	6.26903e-005	-1.30623	E15.5 * Wnt1 down vs E15.5 * Ctrl
10411373	Hexb	NM_010422	0.00996704	-1.3063	E15.5 * Wnt1 down vs E15.5 * Ctrl
10488291	Rbbp9	NM_015754	0.00452819	-1.3064	E15.5 * Wnt1 down vs E15.5 * Ctrl
10471464	St6galnac6	NM_016973	0.0003349	-1.30644	E15.5 * Wnt1 down vs E15.5 * Ctrl
10352936	Sox17	NM_011441	0.0031034	-1.3066	E15.5 * Wnt1 down vs E15.5 * Ctrl
10564417	Aldh1a3	NM_053080	0.0119785	-1.30665	E15.5 * Wnt1 down vs E15.5 * Ctrl
10551966	Hspb6	NM_001012401	0.000431004	-1.30677	E15.5 * Wnt1 down vs E15.5 * Ctrl
10420390	Xpo4	NM_020506	0.000142495	-1.30691	E15.5 * Wnt1 down vs E15.5 * Ctrl
10505749	Dennd4c	NM_001081014	1.91462e-005	-1.30692	E15.5 * Wnt1 down vs E15.5 * Ctrl
10481186	Sardh	NM_138665	0.000927434	-1.30694	E15.5 * Wnt1 down vs E15.5 * Ctrl
10493484	Krtcap2	NM_025327	0.000496743	-1.30703	E15.5 * Wnt1 down vs E15.5 * Ctrl
10367626	1700052N19Rik	NM_024261	0.0144464	-1.30735	E15.5 * Wnt1 down vs E15.5 * Ctrl
10569057	Rnh1	NM_145135	1.29794e-005	-1.30735	E15.5 * Wnt1 down vs E15.5 * Ctrl
10541614	Clec4d	NM_010819	0.000427663	-1.30741	E15.5 * Wnt1 down vs E15.5 * Ctrl
10407811	Hnrnpf	NM_133834	2.08519e-008	-1.30747	E15.5 * Wnt1 down vs E15.5 * Ctrl
10542885	2810474O19Rik	NM_026054	0.00626907	-1.30753	E15.5 * Wnt1 down vs E15.5 * Ctrl
10448676	Slc9a3r2	NM_023055	0.00174243	-1.30755	E15.5 * Wnt1 down vs E15.5 * Ctrl
10499309	Apoa1bp	NM_144897	0.00355832	-1.30764	E15.5 * Wnt1 down vs E15.5 * Ctrl
10495449	Col11a1	NM_007729	1.28487e-005	-1.30773	E15.5 * Wnt1 down vs E15.5 * Ctrl

10579852	Mmaa	NM_133823	0.000393608	-1.30786	E15.5 * Wnt1 down vs E15.5 * Ctrl
10558754	Athl1	NM_145387	0.00468264	-1.30796	E15.5 * Wnt1 down vs E15.5 * Ctrl
10513622	Pole3	NM_021498	0.00186997	-1.30824	E15.5 * Wnt1 down vs E15.5 * Ctrl
10521090	Tacc3	NM_001040435	5.92681e-005	-1.30829	E15.5 * Wnt1 down vs E15.5 * Ctrl
10466886		---	0.00324028	-1.30839	E15.5 * Wnt1 down vs E15.5 * Ctrl
10546184	Plxna1	NM_008881	0.00447681	-1.30844	E15.5 * Wnt1 down vs E15.5 * Ctrl
10355113		---	0.00186499	-1.30848	E15.5 * Wnt1 down vs E15.5 * Ctrl
10351644	Cd244	NM_018729	0.00169341	-1.30857	E15.5 * Wnt1 down vs E15.5 * Ctrl
10551431	Zfp607	NM_001024726	0.00923836	-1.30861	E15.5 * Wnt1 down vs E15.5 * Ctrl
10379363	Atad5	NM_001029856	0.00154694	-1.30885	E15.5 * Wnt1 down vs E15.5 * Ctrl
10554140	Zic4	BC116191	0.00201696	-1.30891	E15.5 * Wnt1 down vs E15.5 * Ctrl
10554156	Fam174b	ENSMUST00000	0.0016382	-1.30916	E15.5 * Wnt1 down vs E15.5 * Ctrl
10483401	Spc25	NM_025565	0.000773291	-1.30919	E15.5 * Wnt1 down vs E15.5 * Ctrl
10380551	Phb	NM_008831	0.00998064	-1.30919	E15.5 * Wnt1 down vs E15.5 * Ctrl
10525516	Wdr66	BC138176	0.000595282	-1.30922	E15.5 * Wnt1 down vs E15.5 * Ctrl
10496555	Gbp1	NM_010259	0.00102163	-1.30941	E15.5 * Wnt1 down vs E15.5 * Ctrl
10586477	Ppib	NM_011149	3.77326e-005	-1.30948	E15.5 * Wnt1 down vs E15.5 * Ctrl
10467136	Ch25h	NM_009890	0.00126077	-1.30957	E15.5 * Wnt1 down vs E15.5 * Ctrl
10387568	Tnfsf12-tnfsf13	NM_001034097	1.98764e-005	-1.3097	E15.5 * Wnt1 down vs E15.5 * Ctrl
10564507	Arrdc4	NM_001042592	0.00601268	-1.30974	E15.5 * Wnt1 down vs E15.5 * Ctrl
10487508		---	7.65305e-005	-1.30976	E15.5 * Wnt1 down vs E15.5 * Ctrl
10497475	Agtr1b	NM_175086	0.00603404	-1.30981	E15.5 * Wnt1 down vs E15.5 * Ctrl
10524515	Myo1h	BC144867	3.33678e-006	-1.31027	E15.5 * Wnt1 down vs E15.5 * Ctrl
10447084	Galm	NM_176963	0.00180862	-1.31031	E15.5 * Wnt1 down vs E15.5 * Ctrl
10503303	1110037F02Rik	NM_001081183	6.58675e-005	-1.31044	E15.5 * Wnt1 down vs E15.5 * Ctrl
10544273	Clec5a	NM_001038604	0.00182845	-1.31046	E15.5 * Wnt1 down vs E15.5 * Ctrl
10566564	Mrpl17	NM_025301	0.00060598	-1.31063	E15.5 * Wnt1 down vs E15.5 * Ctrl
10492558	Smc4	NM_133786	1.47282e-005	-1.31085	E15.5 * Wnt1 down vs E15.5 * Ctrl
10597973	Lztfl1	NM_033322	0.00130614	-1.31096	E15.5 * Wnt1 down vs E15.5 * Ctrl
10510700	Gpr153	NM_178406	0.000101203	-1.31112	E15.5 * Wnt1 down vs E15.5 * Ctrl
10415408	BC030046	BC151043	0.00585843	-1.31118	E15.5 * Wnt1 down vs E15.5 * Ctrl
10535369	B630006N21Rik	AK046749	0.0119818	-1.3112	E15.5 * Wnt1 down vs E15.5 * Ctrl
10574246	Gpr114	NM_001145972	0.000243184	-1.3113	E15.5 * Wnt1 down vs E15.5 * Ctrl
10510241	OTTMUSG00000	NM_001014397	0.000315589	-1.31166	E15.5 * Wnt1 down vs E15.5 * Ctrl
10602068	Mid2	NM_011845	0.000241229	-1.31182	E15.5 * Wnt1 down vs E15.5 * Ctrl
10565002	Crtc3	NM_173863	0.000558266	-1.31184	E15.5 * Wnt1 down vs E15.5 * Ctrl
10377405	Aurkb	NM_011496	0.00211573	-1.31204	E15.5 * Wnt1 down vs E15.5 * Ctrl
10382912	09-Sep	NM_001113486	0.00040182	-1.31267	E15.5 * Wnt1 down vs E15.5 * Ctrl
10437040	Chaf1b	NM_028083	0.00160438	-1.31268	E15.5 * Wnt1 down vs E15.5 * Ctrl
10503508	Ggh	NM_010281	0.0027096	-1.3127	E15.5 * Wnt1 down vs E15.5 * Ctrl
10503523	Ggh	NM_010281	0.0027096	-1.3127	E15.5 * Wnt1 down vs E15.5 * Ctrl
10485963	Arhgap11a	NM_181416	0.000622616	-1.31318	E15.5 * Wnt1 down vs E15.5 * Ctrl
10601854	Wbp5	NM_011712	1.58223e-005	-1.31321	E15.5 * Wnt1 down vs E15.5 * Ctrl
10425834	Samm50	NM_178614	0.0117559	-1.31337	E15.5 * Wnt1 down vs E15.5 * Ctrl
10571233	Fut10	NM_134161	0.00795775	-1.31367	E15.5 * Wnt1 down vs E15.5 * Ctrl
10592919	Arcn1	NM_145985	0.000999068	-1.31445	E15.5 * Wnt1 down vs E15.5 * Ctrl
10592719	Oaf	NM_178644	3.11349e-005	-1.31467	E15.5 * Wnt1 down vs E15.5 * Ctrl
10522596	Tmem165	NM_011626	9.39182e-007	-1.31496	E15.5 * Wnt1 down vs E15.5 * Ctrl
10565862	Pold3	NM_133692	0.00371646	-1.31499	E15.5 * Wnt1 down vs E15.5 * Ctrl
10361169	Hhat	NM_144881	0.010041	-1.31505	E15.5 * Wnt1 down vs E15.5 * Ctrl
10375537	Mgat1	NM_001110148	0.0002232	-1.31516	E15.5 * Wnt1 down vs E15.5 * Ctrl
10464415	1810055E12Rik	BC013514	0.00277313	-1.31543	E15.5 * Wnt1 down vs E15.5 * Ctrl
10409567	Tifab	NM_145976	0.00586828	-1.31556	E15.5 * Wnt1 down vs E15.5 * Ctrl
10362581	Tube1	NM_028006	0.000395743	-1.31566	E15.5 * Wnt1 down vs E15.5 * Ctrl
10563641	Rap1a	NM_145541	3.5794e-005	-1.31576	E15.5 * Wnt1 down vs E15.5 * Ctrl
10424779	Cks2	NM_025415	0.000196623	-1.31599	E15.5 * Wnt1 down vs E15.5 * Ctrl
10472501	Lass6	NM_172856	1.70447e-005	-1.31621	E15.5 * Wnt1 down vs E15.5 * Ctrl
10349868	Ppp1r15b	NM_133819	0.000367901	-1.31641	E15.5 * Wnt1 down vs E15.5 * Ctrl
10533256	Oas1a	NM_145211	0.00638968	-1.31646	E15.5 * Wnt1 down vs E15.5 * Ctrl
10409551	Pitx1	NM_011097	0.00142781	-1.31683	E15.5 * Wnt1 down vs E15.5 * Ctrl
10508112	Lsm10	NM_138721	0.00145347	-1.31693	E15.5 * Wnt1 down vs E15.5 * Ctrl
10599686	Zfp449	NM_030139	0.00644775	-1.31699	E15.5 * Wnt1 down vs E15.5 * Ctrl
10578361	Asah1	NM_019734	0.00366326	-1.31736	E15.5 * Wnt1 down vs E15.5 * Ctrl
10515771	Tie1	NM_011587	0.000861644	-1.31745	E15.5 * Wnt1 down vs E15.5 * Ctrl
10481420	D2Wsu81e	NM_172660	0.00131821	-1.31753	E15.5 * Wnt1 down vs E15.5 * Ctrl
10362811	Sesn1	NM_001013370	0.000769178	-1.31803	E15.5 * Wnt1 down vs E15.5 * Ctrl

10365578	Nup37	NM_028334	0.000723345	-1.31816	E15.5 * Wnt1 down vs E15.5 * Ctrl
10422776	Phb	NM_008831	0.0144949	-1.31818	E15.5 * Wnt1 down vs E15.5 * Ctrl
10553598	Cyfip1	NM_011370	3.78752e-005	-1.31819	E15.5 * Wnt1 down vs E15.5 * Ctrl
10387180	Ndel1	NM_023668	0.000601275	-1.31825	E15.5 * Wnt1 down vs E15.5 * Ctrl
10548086	Rad51ap1	NM_009013	0.00166606	-1.31827	E15.5 * Wnt1 down vs E15.5 * Ctrl
10408335	Aldh5a1	NM_172532	0.0003484	-1.31842	E15.5 * Wnt1 down vs E15.5 * Ctrl
10368638	Fam26e	NM_178908	0.000309482	-1.3185	E15.5 * Wnt1 down vs E15.5 * Ctrl
10502484	Pkn2	NM_178654	0.00032493	-1.31866	E15.5 * Wnt1 down vs E15.5 * Ctrl
10557754	Orai3	NM_198424	0.00302682	-1.31894	E15.5 * Wnt1 down vs E15.5 * Ctrl
10408280	Lrrc16a	NM_026825	0.000368543	-1.3192	E15.5 * Wnt1 down vs E15.5 * Ctrl
10475708	Blvra	NM_026678	0.000683918	-1.31922	E15.5 * Wnt1 down vs E15.5 * Ctrl
10471072	Tor1b	NM_133673	2.66138e-005	-1.31949	E15.5 * Wnt1 down vs E15.5 * Ctrl
10436802		---	0.0134259	-1.31985	E15.5 * Wnt1 down vs E15.5 * Ctrl
10602255	Zcchc16	NM_001033795	0.00823025	-1.31996	E15.5 * Wnt1 down vs E15.5 * Ctrl
10380067	04-Sep	NM_011129	0.00275745	-1.32005	E15.5 * Wnt1 down vs E15.5 * Ctrl
10404874	Mylip	NM_153789	0.00556927	-1.32006	E15.5 * Wnt1 down vs E15.5 * Ctrl
10558325	Bccip	NM_025392	1.99073e-005	-1.32017	E15.5 * Wnt1 down vs E15.5 * Ctrl
10592816	Hmbs	NM_013551	0.000532128	-1.32096	E15.5 * Wnt1 down vs E15.5 * Ctrl
10445046	Trim10	NM_011280	0.00121091	-1.32113	E15.5 * Wnt1 down vs E15.5 * Ctrl
10399254	Cenpo	NM_134046	0.00143151	-1.3212	E15.5 * Wnt1 down vs E15.5 * Ctrl
10518455	Agtrap	NM_009642	0.00717669	-1.32136	E15.5 * Wnt1 down vs E15.5 * Ctrl
10513604	Hdhd3	NM_024257	0.00719553	-1.32148	E15.5 * Wnt1 down vs E15.5 * Ctrl
10350247	Kif21b	NM_001039472	0.000550421	-1.32165	E15.5 * Wnt1 down vs E15.5 * Ctrl
10486664	Epb4.2	NM_013513	0.0109474	-1.32174	E15.5 * Wnt1 down vs E15.5 * Ctrl
10424188	Mtbp	NM_134092	0.00151759	-1.32175	E15.5 * Wnt1 down vs E15.5 * Ctrl
10384373	Fignl1	NM_021891	0.000621116	-1.32193	E15.5 * Wnt1 down vs E15.5 * Ctrl
10495945	4930422G04Rik	BC030185	6.08457e-005	-1.32213	E15.5 * Wnt1 down vs E15.5 * Ctrl
10384824	Ncrna00117	NM_026465	0.00792231	-1.32241	E15.5 * Wnt1 down vs E15.5 * Ctrl
10548359	Clec12b	NM_027709	0.000215378	-1.32276	E15.5 * Wnt1 down vs E15.5 * Ctrl
10490053	Zfp217	NM_001033299	0.000171805	-1.32293	E15.5 * Wnt1 down vs E15.5 * Ctrl
10519527	Abcb1a	NM_011076	0.000256204	-1.32324	E15.5 * Wnt1 down vs E15.5 * Ctrl
10596671	Rassf1	NM_019713	0.00253715	-1.3233	E15.5 * Wnt1 down vs E15.5 * Ctrl
10521972	Pcdh7	NM_018764	0.00095813	-1.32361	E15.5 * Wnt1 down vs E15.5 * Ctrl
10566097	Nup98	NM_022979	3.72928e-006	-1.32392	E15.5 * Wnt1 down vs E15.5 * Ctrl
10382438	Cd300a	NM_170758	0.00298263	-1.32394	E15.5 * Wnt1 down vs E15.5 * Ctrl
10499612	Zbtb7b	NM_009565	0.0018766	-1.32452	E15.5 * Wnt1 down vs E15.5 * Ctrl
10406581	Dhfr	NM_010049	0.00341489	-1.32456	E15.5 * Wnt1 down vs E15.5 * Ctrl
10483824	Ttc30a1	NM_030188	0.0129209	-1.32486	E15.5 * Wnt1 down vs E15.5 * Ctrl
10479811	Mcm10	NM_027290	0.000129469	-1.32489	E15.5 * Wnt1 down vs E15.5 * Ctrl
10471567	C130021I20Rik	AK147796	0.00355198	-1.32493	E15.5 * Wnt1 down vs E15.5 * Ctrl
10540105	Tmem43	NM_028766	0.00567186	-1.32515	E15.5 * Wnt1 down vs E15.5 * Ctrl
10494509	Pias3	NM_146135	0.00555593	-1.32533	E15.5 * Wnt1 down vs E15.5 * Ctrl
10521911	Rbpj	AY512934	0.0137608	-1.32568	E15.5 * Wnt1 down vs E15.5 * Ctrl
10569203	Chid1	NM_001142681	0.000819311	-1.32577	E15.5 * Wnt1 down vs E15.5 * Ctrl
10425866	Parvg	NM_022321	0.00550307	-1.32615	E15.5 * Wnt1 down vs E15.5 * Ctrl
10403361	Pitrm1	NM_145131	4.23551e-005	-1.32638	E15.5 * Wnt1 down vs E15.5 * Ctrl
10453715	Rab18	BC056351	0.00513062	-1.32648	E15.5 * Wnt1 down vs E15.5 * Ctrl
10444595	Lsm2	NM_030597	0.00264989	-1.32661	E15.5 * Wnt1 down vs E15.5 * Ctrl
10546056	Rab43	NM_001039394	0.00240391	-1.32668	E15.5 * Wnt1 down vs E15.5 * Ctrl
10520833	Mrpl33	NM_025796	0.000488771	-1.32677	E15.5 * Wnt1 down vs E15.5 * Ctrl
10412345	Parp8	NM_001081009	0.00448601	-1.3268	E15.5 * Wnt1 down vs E15.5 * Ctrl
10541104	Hnrnpf	NM_133834	1.20592e-009	-1.32686	E15.5 * Wnt1 down vs E15.5 * Ctrl
10602105	Col4a5	NM_007736	0.000921724	-1.32758	E15.5 * Wnt1 down vs E15.5 * Ctrl
10586491	Dapk2	NM_010019	0.000433608	-1.32766	E15.5 * Wnt1 down vs E15.5 * Ctrl
10556583	Nucb2	NM_001130479	0.000162973	-1.32815	E15.5 * Wnt1 down vs E15.5 * Ctrl
10385239	Mat2b	NM_134017	1.07053e-005	-1.32837	E15.5 * Wnt1 down vs E15.5 * Ctrl
10520080	Rint1	NM_177323	0.000126821	-1.32875	E15.5 * Wnt1 down vs E15.5 * Ctrl
10518346	OTTMUSG00000	NM_001085522	0.00213851	-1.32881	E15.5 * Wnt1 down vs E15.5 * Ctrl
10565437	Tmem135	NM_028343	0.00783452	-1.32895	E15.5 * Wnt1 down vs E15.5 * Ctrl
10544417	Epha1	NM_023580	0.0034627	-1.32895	E15.5 * Wnt1 down vs E15.5 * Ctrl
10507218	Mknk1	NM_021461	0.00110741	-1.32895	E15.5 * Wnt1 down vs E15.5 * Ctrl
10439854	Bbx	NM_027444	0.000645363	-1.32909	E15.5 * Wnt1 down vs E15.5 * Ctrl
10470446	Rxra	NM_011305	0.00195207	-1.32917	E15.5 * Wnt1 down vs E15.5 * Ctrl
10352767	Nek2	NM_010892	0.00203817	-1.32925	E15.5 * Wnt1 down vs E15.5 * Ctrl
10401900	Sel1l	NM_001039089	0.000192065	-1.32943	E15.5 * Wnt1 down vs E15.5 * Ctrl
10576857	Timm44	NM_011592	0.00453506	-1.32943	E15.5 * Wnt1 down vs E15.5 * Ctrl

10533844	Rilpl2	NM_030259	0.00321679	-1.32962	E15.5 * Wnt1 down vs E15.5 * Ctrl
10577954	Rab11fip1	NM_001080813	0.00548229	-1.32998	E15.5 * Wnt1 down vs E15.5 * Ctrl
10548437	Klra17	NM_133203	2.17997e-006	-1.33001	E15.5 * Wnt1 down vs E15.5 * Ctrl
10456488		---	0.0131941	-1.33045	E15.5 * Wnt1 down vs E15.5 * Ctrl
10604019	1810037I17Rik	NM_024461	0.000137792	-1.33101	E15.5 * Wnt1 down vs E15.5 * Ctrl
10444911	Mdc1	NM_001010833	0.00505791	-1.3312	E15.5 * Wnt1 down vs E15.5 * Ctrl
10402409	Serpina1e	NM_009247	0.00719918	-1.33142	E15.5 * Wnt1 down vs E15.5 * Ctrl
10514939	Podn	NM_172874	0.0014488	-1.33154	E15.5 * Wnt1 down vs E15.5 * Ctrl
10406681	Ap3b1	NM_009680	2.8158e-005	-1.33158	E15.5 * Wnt1 down vs E15.5 * Ctrl
10458960	Aldh7a1	NM_001127338	0.000351829	-1.33161	E15.5 * Wnt1 down vs E15.5 * Ctrl
10492195	Tsc22d2	NM_001081229	4.97161e-005	-1.33199	E15.5 * Wnt1 down vs E15.5 * Ctrl
10586017	Uaca	NM_028283	0.000374222	-1.33211	E15.5 * Wnt1 down vs E15.5 * Ctrl
10377550	Trp53	NM_001127233	2.8122e-005	-1.33233	E15.5 * Wnt1 down vs E15.5 * Ctrl
10350800	Tor1aip2	NM_172843	5.59475e-007	-1.33283	E15.5 * Wnt1 down vs E15.5 * Ctrl
10551511	Gmfg	NM_022024	0.000687862	-1.33285	E15.5 * Wnt1 down vs E15.5 * Ctrl
10420935	Ephx2	NM_007940	0.00100921	-1.33288	E15.5 * Wnt1 down vs E15.5 * Ctrl
10459918	1110032A13Rik	BC064066	0.00013793	-1.33289	E15.5 * Wnt1 down vs E15.5 * Ctrl
10409866	Ctla2b	NM_007797	0.0128218	-1.33297	E15.5 * Wnt1 down vs E15.5 * Ctrl
10474984	Nusap1	NM_133851	0.00334239	-1.33307	E15.5 * Wnt1 down vs E15.5 * Ctrl
10509030	Runx3	NM_019732	0.000350151	-1.33317	E15.5 * Wnt1 down vs E15.5 * Ctrl
10547943	Ncapd2	NM_146171	0.0009881	-1.33323	E15.5 * Wnt1 down vs E15.5 * Ctrl
10459590	Ptpn2	NM_001127177	0.00201906	-1.33324	E15.5 * Wnt1 down vs E15.5 * Ctrl
10585588	Cspg4	NM_139001	0.00616879	-1.33327	E15.5 * Wnt1 down vs E15.5 * Ctrl
10402225	Trip11	NM_028446	0.000419748	-1.3333	E15.5 * Wnt1 down vs E15.5 * Ctrl
10518743	H6pd	NM_173371	0.00233682	-1.33333	E15.5 * Wnt1 down vs E15.5 * Ctrl
10571274	Gsr	NM_010344	0.00310376	-1.33353	E15.5 * Wnt1 down vs E15.5 * Ctrl
10463476	Kazald1	NM_178929	0.00684796	-1.33362	E15.5 * Wnt1 down vs E15.5 * Ctrl
10405185	Cks2	NM_025415	0.000306029	-1.33372	E15.5 * Wnt1 down vs E15.5 * Ctrl
10388086	Nlrp1c	NM_001039234	7.25307e-005	-1.33391	E15.5 * Wnt1 down vs E15.5 * Ctrl
10487109	Cep152	NM_001081091	0.000177008	-1.33402	E15.5 * Wnt1 down vs E15.5 * Ctrl
10354286	Kdelc1	NM_023645	0.00074552	-1.33424	E15.5 * Wnt1 down vs E15.5 * Ctrl
10364280	Pttg1ip	NM_145925	0.00877921	-1.33425	E15.5 * Wnt1 down vs E15.5 * Ctrl
10502224	Sgms2	NM_028943	0.00558066	-1.3343	E15.5 * Wnt1 down vs E15.5 * Ctrl
10565712	Acer3	NM_025408	0.0061812	-1.33448	E15.5 * Wnt1 down vs E15.5 * Ctrl
10390059	Xylt2	NM_145828	0.000138581	-1.33508	E15.5 * Wnt1 down vs E15.5 * Ctrl
10521709	Lap3	NM_024434	0.000238313	-1.33509	E15.5 * Wnt1 down vs E15.5 * Ctrl
10440463	LOC436427	XR_034439	5.31294e-005	-1.33509	E15.5 * Wnt1 down vs E15.5 * Ctrl
10366645	1700006J14Rik	ENSMUST00000(	0.0105848	-1.33517	E15.5 * Wnt1 down vs E15.5 * Ctrl
10535759	Lnx2	NM_080795	0.00354099	-1.33553	E15.5 * Wnt1 down vs E15.5 * Ctrl
10502863	Ak5	NM_001081277	0.00234292	-1.33564	E15.5 * Wnt1 down vs E15.5 * Ctrl
10531588	Prkg2	NM_008926	0.00347998	-1.33583	E15.5 * Wnt1 down vs E15.5 * Ctrl
10592847	EG667952	ENSMUST00000(	2.68935e-007	-1.3359	E15.5 * Wnt1 down vs E15.5 * Ctrl
10450699	H2-Q2	AB359227	0.0037537	-1.33644	E15.5 * Wnt1 down vs E15.5 * Ctrl
10588049	Copb2	NM_015827	8.6414e-008	-1.33645	E15.5 * Wnt1 down vs E15.5 * Ctrl
10477187	Tpx2	NM_001141977	0.000316213	-1.33665	E15.5 * Wnt1 down vs E15.5 * Ctrl
10492699	Ctso	NM_177662	0.000437749	-1.33666	E15.5 * Wnt1 down vs E15.5 * Ctrl
10361338	Ipcef1	NM_001033391	0.000128089	-1.33669	E15.5 * Wnt1 down vs E15.5 * Ctrl
10493086	Hdgf	NM_008231	0.00117886	-1.3368	E15.5 * Wnt1 down vs E15.5 * Ctrl
10596747	Sema3f	NM_011349	6.99319e-005	-1.33696	E15.5 * Wnt1 down vs E15.5 * Ctrl
10598159			0.0117257	-1.33751	E15.5 * Wnt1 down vs E15.5 * Ctrl
10383025	C1qtnf1	NM_019959	0.00580696	-1.33753	E15.5 * Wnt1 down vs E15.5 * Ctrl
10374035	Xbp1	NM_013842	0.00070586	-1.33781	E15.5 * Wnt1 down vs E15.5 * Ctrl
10588495	Dusp7	NM_153459	0.000546376	-1.33799	E15.5 * Wnt1 down vs E15.5 * Ctrl
10411711	Cdk7	NM_009874	0.00633439	-1.33807	E15.5 * Wnt1 down vs E15.5 * Ctrl
10538206	Ccdc126	NM_175098	0.00329216	-1.33815	E15.5 * Wnt1 down vs E15.5 * Ctrl
10605081	Bcap31	NM_012060	5.70255e-005	-1.33839	E15.5 * Wnt1 down vs E15.5 * Ctrl
10453233	Slc8a1	NM_011406	0.000521641	-1.3384	E15.5 * Wnt1 down vs E15.5 * Ctrl
10546834	Rad18	NM_021385	1.33179e-005	-1.33847	E15.5 * Wnt1 down vs E15.5 * Ctrl
10350864	Sec16b	NM_033354	0.000876169	-1.33922	E15.5 * Wnt1 down vs E15.5 * Ctrl
10398388		---	0.0022018	-1.33923	E15.5 * Wnt1 down vs E15.5 * Ctrl
10376144	Fnip1	NM_173753	0.000446926	-1.33926	E15.5 * Wnt1 down vs E15.5 * Ctrl
10583264	Mre11a	NM_018736	0.000898002	-1.33941	E15.5 * Wnt1 down vs E15.5 * Ctrl
10432511	Racgap1	NM_012025	0.0117028	-1.33943	E15.5 * Wnt1 down vs E15.5 * Ctrl
10427454	Card6	ENSMUST00000	0.000528356	-1.33948	E15.5 * Wnt1 down vs E15.5 * Ctrl
10543082	Col28a1	NM_001037865	0.0050665	-1.33959	E15.5 * Wnt1 down vs E15.5 * Ctrl
10387368	Tmem88	NM_025915	0.000528398	-1.33976	E15.5 * Wnt1 down vs E15.5 * Ctrl

10555671	Frag1	NM_145583	0.000180422	-1.33977	E15.5 * Wnt1 down vs E15.5 * Ctrl
10463836	Gsto1	NM_010362	0.000613406	-1.33979	E15.5 * Wnt1 down vs E15.5 * Ctrl
10353004	Cks2	NM_025415	0.000231676	-1.33981	E15.5 * Wnt1 down vs E15.5 * Ctrl
10514933	Cpt2	NM_009949	0.000149804	-1.33987	E15.5 * Wnt1 down vs E15.5 * Ctrl
10538832	Mad2l1	NM_019499	0.00181975	-1.34003	E15.5 * Wnt1 down vs E15.5 * Ctrl
10457587	Zfp521	NM_145492	6.00113e-005	-1.34049	E15.5 * Wnt1 down vs E15.5 * Ctrl
10432939	Csad	NM_144942	0.000648634	-1.34081	E15.5 * Wnt1 down vs E15.5 * Ctrl
10491699	Fgf2	NM_008006	0.00100909	-1.34108	E15.5 * Wnt1 down vs E15.5 * Ctrl
10348929	Hnrnpf	NM_133834	1.24387e-009	-1.34108	E15.5 * Wnt1 down vs E15.5 * Ctrl
10347639	Gmppa	NM_133708	0.0102355	-1.34133	E15.5 * Wnt1 down vs E15.5 * Ctrl
10382328	Sox9	NM_011448	8.82287e-005	-1.34137	E15.5 * Wnt1 down vs E15.5 * Ctrl
10564960	Furin	NM_011046	3.95958e-005	-1.34137	E15.5 * Wnt1 down vs E15.5 * Ctrl
10499639	Cks1b	NM_016904	2.99735e-006	-1.3415	E15.5 * Wnt1 down vs E15.5 * Ctrl
10501690	Slc35a3	NM_144902	0.00393715	-1.34159	E15.5 * Wnt1 down vs E15.5 * Ctrl
10396656	Gm70	ENSMUST00000(	0.0119477	-1.3417	E15.5 * Wnt1 down vs E15.5 * Ctrl
10548375	Clec7a	NM_020008	0.000499882	-1.34191	E15.5 * Wnt1 down vs E15.5 * Ctrl
10570982	Fgfr1	NM_010206	0.00611929	-1.34194	E15.5 * Wnt1 down vs E15.5 * Ctrl
10517463	Luzp1	BC137786	1.175e-005	-1.34211	E15.5 * Wnt1 down vs E15.5 * Ctrl
10374185	Ccm2	NM_146014	0.00933624	-1.34211	E15.5 * Wnt1 down vs E15.5 * Ctrl
10395293	Atxn7l1	NM_001033436	0.000486979	-1.34219	E15.5 * Wnt1 down vs E15.5 * Ctrl
10435543	Golgb1	NM_030035	3.2562e-005	-1.34224	E15.5 * Wnt1 down vs E15.5 * Ctrl
10384474	Pno1	NM_025443	0.00151124	-1.34229	E15.5 * Wnt1 down vs E15.5 * Ctrl
10528915	Tyms	NM_021288	0.000432358	-1.3425	E15.5 * Wnt1 down vs E15.5 * Ctrl
10570201	Atp11a	NM_015804	0.00020378	-1.34251	E15.5 * Wnt1 down vs E15.5 * Ctrl
10521515	Evc2	NM_145920	7.61027e-005	-1.34265	E15.5 * Wnt1 down vs E15.5 * Ctrl
10481092	Agpat2	NM_026212	0.00153387	-1.34273	E15.5 * Wnt1 down vs E15.5 * Ctrl
10494565	Fmo5	NM_010232	0.000523323	-1.34311	E15.5 * Wnt1 down vs E15.5 * Ctrl
10386070	Atox1	NM_009720	1.92934e-005	-1.3434	E15.5 * Wnt1 down vs E15.5 * Ctrl
10423770	Grhl2	NM_026496	0.00440936	-1.34352	E15.5 * Wnt1 down vs E15.5 * Ctrl
10573261	Asf1b	NM_024184	0.00320853	-1.34352	E15.5 * Wnt1 down vs E15.5 * Ctrl
10413609	Mustn1	NM_181390	0.00141944	-1.34353	E15.5 * Wnt1 down vs E15.5 * Ctrl
10401935	BC005685	BC005685	0.000993317	-1.34358	E15.5 * Wnt1 down vs E15.5 * Ctrl
10541729	Cdca3	NM_013538	0.000743234	-1.34365	E15.5 * Wnt1 down vs E15.5 * Ctrl
10496494	Tspan5	NM_019571	0.00519921	-1.34367	E15.5 * Wnt1 down vs E15.5 * Ctrl
10473547	Srp9	NM_012058	2.70182e-006	-1.34385	E15.5 * Wnt1 down vs E15.5 * Ctrl
10373101	Pip4k2c	NM_054097	0.000701291	-1.3439	E15.5 * Wnt1 down vs E15.5 * Ctrl
10461487	Cybasc3	NM_201351	8.04221e-005	-1.34406	E15.5 * Wnt1 down vs E15.5 * Ctrl
10567049	Copb1	NM_033370	1.37122e-005	-1.3441	E15.5 * Wnt1 down vs E15.5 * Ctrl
10448117	Has1	NM_008215	0.00785299	-1.34415	E15.5 * Wnt1 down vs E15.5 * Ctrl
10450038	Angptl4	NM_020581	0.00818135	-1.34456	E15.5 * Wnt1 down vs E15.5 * Ctrl
10477069	Tcf15	NM_009328	0.00203094	-1.34469	E15.5 * Wnt1 down vs E15.5 * Ctrl
10485445	Ehf	NM_007914	0.00036525	-1.34475	E15.5 * Wnt1 down vs E15.5 * Ctrl
10424584	Dennd3	NM_001081066	0.00100452	-1.34503	E15.5 * Wnt1 down vs E15.5 * Ctrl
10415640	Snora65	NR_002898	0.000418801	-1.3452	E15.5 * Wnt1 down vs E15.5 * Ctrl
10411229	F2r	NM_010169	1.6358e-005	-1.34522	E15.5 * Wnt1 down vs E15.5 * Ctrl
10373313	Nab2	NM_008668	0.00341052	-1.34547	E15.5 * Wnt1 down vs E15.5 * Ctrl
10516246	Cdca8	NM_026560	3.01283e-005	-1.34572	E15.5 * Wnt1 down vs E15.5 * Ctrl
10380174	Mpo	NM_010824	0.00132142	-1.34591	E15.5 * Wnt1 down vs E15.5 * Ctrl
10607366	Shroom2	NM_172441	0.00022295	-1.34607	E15.5 * Wnt1 down vs E15.5 * Ctrl
10441254	Tmprss2	NM_015775	0.000444767	-1.34618	E15.5 * Wnt1 down vs E15.5 * Ctrl
10495316	Psrc1	NM_019976	0.00322898	-1.34634	E15.5 * Wnt1 down vs E15.5 * Ctrl
10550593	Six5	NM_011383	0.00538406	-1.34639	E15.5 * Wnt1 down vs E15.5 * Ctrl
10412078	Gapt	NM_177713	0.000295101	-1.34655	E15.5 * Wnt1 down vs E15.5 * Ctrl
10451613	Foxp4	NM_001110824	0.00555943	-1.34663	E15.5 * Wnt1 down vs E15.5 * Ctrl
10465861	Incenp	NM_016692	0.000429531	-1.34666	E15.5 * Wnt1 down vs E15.5 * Ctrl
10416269	Ppp3cc	ENSMUST00000(	0.000111118	-1.3467	E15.5 * Wnt1 down vs E15.5 * Ctrl
10543004	Pon2	NM_183308	0.000890912	-1.34682	E15.5 * Wnt1 down vs E15.5 * Ctrl
10429114	Tmem71	NM_172514	0.00582568	-1.34729	E15.5 * Wnt1 down vs E15.5 * Ctrl
10406968	Cenpk	NM_021790	0.00240293	-1.34762	E15.5 * Wnt1 down vs E15.5 * Ctrl
10525989	Gpr133	NM_001081342	0.000131322	-1.34769	E15.5 * Wnt1 down vs E15.5 * Ctrl
10496656	Col24a1	NM_027770	0.000472915	-1.34771	E15.5 * Wnt1 down vs E15.5 * Ctrl
10394471	Sdc1	NM_011519	0.000359338	-1.3478	E15.5 * Wnt1 down vs E15.5 * Ctrl
10357814	Zc3h11a	NM_144530	4.865e-007	-1.34788	E15.5 * Wnt1 down vs E15.5 * Ctrl
10360173	Slamf7	NM_144539	0.00663358	-1.34834	E15.5 * Wnt1 down vs E15.5 * Ctrl
10379646	Slfn3	NM_011409	0.0097387	-1.34848	E15.5 * Wnt1 down vs E15.5 * Ctrl
10502375	Mttp	NM_008642	0.00980173	-1.34891	E15.5 * Wnt1 down vs E15.5 * Ctrl

10408902	Ccdc90a	NM_001081059	0.00439738	-1.34901	E15.5 * Wnt1 down vs E15.5 * Ctrl
10478692	Slc2a10	NM_130451	0.000408504	-1.34941	E15.5 * Wnt1 down vs E15.5 * Ctrl
10395376	Ankmy2	NM_146033	0.000213729	-1.34959	E15.5 * Wnt1 down vs E15.5 * Ctrl
10351703	Copa	NM_009938	3.26336e-007	-1.34978	E15.5 * Wnt1 down vs E15.5 * Ctrl
10584591		---	0.000154397	-1.35012	E15.5 * Wnt1 down vs E15.5 * Ctrl
10607012	Col4a6	NM_053185	0.00109036	-1.35036	E15.5 * Wnt1 down vs E15.5 * Ctrl
10409202		---	0.000208845	-1.3504	E15.5 * Wnt1 down vs E15.5 * Ctrl
10359034	Qsox1	NM_001024945	0.00211463	-1.35057	E15.5 * Wnt1 down vs E15.5 * Ctrl
10465783	Tmem179b	NM_026325	3.05767e-005	-1.35065	E15.5 * Wnt1 down vs E15.5 * Ctrl
10516229	Utp11l	NM_026031	0.00297175	-1.35066	E15.5 * Wnt1 down vs E15.5 * Ctrl
10550509	Pglyrp1	NM_009402	0.00119046	-1.35069	E15.5 * Wnt1 down vs E15.5 * Ctrl
10503305	1110037F02Rik	NM_001081183	0.00071132	-1.35083	E15.5 * Wnt1 down vs E15.5 * Ctrl
10388517	Gemin4	NM_177367	0.00452662	-1.35145	E15.5 * Wnt1 down vs E15.5 * Ctrl
10481518	Ptges	NM_022415	0.00265219	-1.35166	E15.5 * Wnt1 down vs E15.5 * Ctrl
10531776	Fam175a	NM_172405	0.00711151	-1.35193	E15.5 * Wnt1 down vs E15.5 * Ctrl
10588137	Dzip1l	NM_028258	8.09032e-005	-1.35239	E15.5 * Wnt1 down vs E15.5 * Ctrl
10462796	Kif11	NM_010615	5.49601e-005	-1.35257	E15.5 * Wnt1 down vs E15.5 * Ctrl
10372730	Iltifb	NM_054079	0.00431962	-1.35261	E15.5 * Wnt1 down vs E15.5 * Ctrl
10581495	Rplp1	NM_018853	0.0010322	-1.35285	E15.5 * Wnt1 down vs E15.5 * Ctrl
10435930	Cd200r2	NM_206535	8.329e-005	-1.35303	E15.5 * Wnt1 down vs E15.5 * Ctrl
10478022	Rprd1b	NM_027434	0.00175059	-1.35311	E15.5 * Wnt1 down vs E15.5 * Ctrl
10418905	3110001K24Rik	NM_029389	0.00179812	-1.35314	E15.5 * Wnt1 down vs E15.5 * Ctrl
10375083	Stk10	NM_009288	0.00013476	-1.35359	E15.5 * Wnt1 down vs E15.5 * Ctrl
10422396	Stk24	NM_145465	0.000797329	-1.35368	E15.5 * Wnt1 down vs E15.5 * Ctrl
10533026	Prkab1	NM_031869	0.00167333	-1.35376	E15.5 * Wnt1 down vs E15.5 * Ctrl
10421517	Cysltr2	NM_133720	3.57913e-005	-1.35382	E15.5 * Wnt1 down vs E15.5 * Ctrl
10347481	Cyp27a1	NM_024264	0.000144036	-1.35399	E15.5 * Wnt1 down vs E15.5 * Ctrl
10462866	Cep55	NM_028760	0.00119515	-1.35408	E15.5 * Wnt1 down vs E15.5 * Ctrl
10578193	Eri1	NM_026067	0.00138672	-1.35417	E15.5 * Wnt1 down vs E15.5 * Ctrl
10443391	Mapk14	NM_011951	8.37645e-005	-1.35457	E15.5 * Wnt1 down vs E15.5 * Ctrl
10397351	Jdp2	NM_030887	6.26378e-005	-1.3546	E15.5 * Wnt1 down vs E15.5 * Ctrl
10552140	Cebpa	NM_007678	0.000223844	-1.35463	E15.5 * Wnt1 down vs E15.5 * Ctrl
10385518	Tgtp	NM_011579	0.000470311	-1.35508	E15.5 * Wnt1 down vs E15.5 * Ctrl
10501593	Slc30a7	NM_023214	7.7799e-005	-1.35508	E15.5 * Wnt1 down vs E15.5 * Ctrl
10526972	Nudt1	NM_008637	0.00425619	-1.35511	E15.5 * Wnt1 down vs E15.5 * Ctrl
10442027	ENSMUSG0000C	ENSMUST00000(	0.00167479	-1.35518	E15.5 * Wnt1 down vs E15.5 * Ctrl
10379489	Tmem98	NM_029537	0.0107419	-1.35535	E15.5 * Wnt1 down vs E15.5 * Ctrl
10535979	Rfc3	NM_027009	0.00535383	-1.35572	E15.5 * Wnt1 down vs E15.5 * Ctrl
10593913	1700017B05Rik	NM_028820	0.000415831	-1.35591	E15.5 * Wnt1 down vs E15.5 * Ctrl
10367843	Utrn	NM_011682	6.87101e-005	-1.35593	E15.5 * Wnt1 down vs E15.5 * Ctrl
10499536	Efna1	NM_010107	0.00017921	-1.35597	E15.5 * Wnt1 down vs E15.5 * Ctrl
10568150	Kif22	NM_145588	0.00385416	-1.35628	E15.5 * Wnt1 down vs E15.5 * Ctrl
10464153	Nhlrc2	NM_025811	0.000383654	-1.35639	E15.5 * Wnt1 down vs E15.5 * Ctrl
10383887	1110020P15Rik	NM_197979	0.00299306	-1.35644	E15.5 * Wnt1 down vs E15.5 * Ctrl
10406407	Arrdc3	NM_001042591	0.006671	-1.35651	E15.5 * Wnt1 down vs E15.5 * Ctrl
10419296	Wdhd1	NM_172598	1.05157e-005	-1.35676	E15.5 * Wnt1 down vs E15.5 * Ctrl
10416451	Cpb2	NM_019775	0.012687	-1.35715	E15.5 * Wnt1 down vs E15.5 * Ctrl
10467368	Ctdspl2	NM_212450	0.00321536	-1.35775	E15.5 * Wnt1 down vs E15.5 * Ctrl
10555695	Rrm1	NM_009103	8.6009e-007	-1.35792	E15.5 * Wnt1 down vs E15.5 * Ctrl
10605319	Ubl4	NM_145405	0.00112234	-1.35804	E15.5 * Wnt1 down vs E15.5 * Ctrl
10564520	Rpl23	AF357384	0.00835804	-1.35805	E15.5 * Wnt1 down vs E15.5 * Ctrl
10359377	Zbtb37	NM_173424	2.31195e-005	-1.35837	E15.5 * Wnt1 down vs E15.5 * Ctrl
10355554	Aamp	NM_146110	0.0139417	-1.3584	E15.5 * Wnt1 down vs E15.5 * Ctrl
10491272	Gpr160	NM_001134385	0.00450428	-1.35845	E15.5 * Wnt1 down vs E15.5 * Ctrl
10539435	Bola3	NM_175277	0.0072054	-1.35863	E15.5 * Wnt1 down vs E15.5 * Ctrl
10574087	Herpud1	NM_022331	0.00306552	-1.35869	E15.5 * Wnt1 down vs E15.5 * Ctrl
10551359	Sertad1	NM_018820	0.00038968	-1.3587	E15.5 * Wnt1 down vs E15.5 * Ctrl
10508936	Ubxn11	NM_026257	0.00740973	-1.3588	E15.5 * Wnt1 down vs E15.5 * Ctrl
10406777	Gm73	AK157788	0.000964775	-1.35891	E15.5 * Wnt1 down vs E15.5 * Ctrl
10498775	Golim4	NM_175193	7.82246e-005	-1.35902	E15.5 * Wnt1 down vs E15.5 * Ctrl
10386110	Hand1	NM_008213	0.000461895	-1.35924	E15.5 * Wnt1 down vs E15.5 * Ctrl
10435821	Nat13	NM_028108	1.13957e-007	-1.3594	E15.5 * Wnt1 down vs E15.5 * Ctrl
10454067	Psma8	BC116990	0.00532333	-1.35956	E15.5 * Wnt1 down vs E15.5 * Ctrl
10446425		---	0.00388796	-1.35958	E15.5 * Wnt1 down vs E15.5 * Ctrl
10569656	Tpcn2	NM_146206	0.000722526	-1.3596	E15.5 * Wnt1 down vs E15.5 * Ctrl
10491313	Cldn11	NM_008770	0.000471412	-1.35982	E15.5 * Wnt1 down vs E15.5 * Ctrl

10578771	Galnt7	NM_144731	0.000109127	-1.35985	E15.5 * Wnt1 down vs E15.5 * Ctrl
10496737	Bcl10	NM_009740	3.66231e-006	-1.35999	E15.5 * Wnt1 down vs E15.5 * Ctrl
10498168	Exosc8	NM_027148	5.58849e-006	-1.36009	E15.5 * Wnt1 down vs E15.5 * Ctrl
10361234	Hsd11b1	NM_008288	0.000329995	-1.36028	E15.5 * Wnt1 down vs E15.5 * Ctrl
10497894	Cetn4	NM_145825	0.00257966	-1.3607	E15.5 * Wnt1 down vs E15.5 * Ctrl
10430247	Txn2	NM_019913	0.00242892	-1.36074	E15.5 * Wnt1 down vs E15.5 * Ctrl
10552418	Etfb	NM_026695	0.000582457	-1.36074	E15.5 * Wnt1 down vs E15.5 * Ctrl
10598750	Gpr34	NM_011823	0.0101943	-1.36086	E15.5 * Wnt1 down vs E15.5 * Ctrl
10584841	Amica1	NM_001005421	0.00114829	-1.36096	E15.5 * Wnt1 down vs E15.5 * Ctrl
10503520	Ttpa	NM_015767	0.00855179	-1.36112	E15.5 * Wnt1 down vs E15.5 * Ctrl
10575844	Cdh13	NM_019707	0.00383035	-1.36117	E15.5 * Wnt1 down vs E15.5 * Ctrl
10356423	Usp40	NM_001033291	0.000166841	-1.36133	E15.5 * Wnt1 down vs E15.5 * Ctrl
10413542	Tkt	NM_009388	0.000827347	-1.36151	E15.5 * Wnt1 down vs E15.5 * Ctrl
10594762	Fam81a	NM_029784	0.0101925	-1.36164	E15.5 * Wnt1 down vs E15.5 * Ctrl
10394258	Adcy3	NM_001159537	5.46132e-006	-1.36227	E15.5 * Wnt1 down vs E15.5 * Ctrl
10510178	OTTMUSG00000	NM_001037926	0.000256236	-1.36234	E15.5 * Wnt1 down vs E15.5 * Ctrl
10365242	Hcfc2	NM_001081218	0.000136237	-1.36247	E15.5 * Wnt1 down vs E15.5 * Ctrl
10559478	Pira3	NM_011090	0.00615819	-1.36249	E15.5 * Wnt1 down vs E15.5 * Ctrl
10463906	Ins1	NM_008386	0.000198602	-1.36276	E15.5 * Wnt1 down vs E15.5 * Ctrl
10525804	Atp6v0a2	NM_011596	0.00127987	-1.36278	E15.5 * Wnt1 down vs E15.5 * Ctrl
10565840	Neu3	NM_016720	8.38627e-005	-1.36306	E15.5 * Wnt1 down vs E15.5 * Ctrl
10346747	Cyp20a1	NM_030013	0.000133445	-1.36306	E15.5 * Wnt1 down vs E15.5 * Ctrl
10348879	Ing5	NM_025454	0.000577123	-1.36396	E15.5 * Wnt1 down vs E15.5 * Ctrl
10554926	Ccdc90b	NM_025515	0.00188949	-1.36421	E15.5 * Wnt1 down vs E15.5 * Ctrl
10459755	2810433K01Rik	NM_025581	7.01276e-006	-1.36435	E15.5 * Wnt1 down vs E15.5 * Ctrl
10550102	Lig1	NM_001083188	5.42464e-005	-1.36462	E15.5 * Wnt1 down vs E15.5 * Ctrl
10463826	6330577E15Rik	NM_026377	5.95969e-008	-1.36466	E15.5 * Wnt1 down vs E15.5 * Ctrl
10491319	Kcnmb2	NM_028231	0.0143897	-1.36493	E15.5 * Wnt1 down vs E15.5 * Ctrl
10405753	Me1	NM_008615	0.00921395	-1.36495	E15.5 * Wnt1 down vs E15.5 * Ctrl
10461629	Ms4a4d	NM_025658	0.000786941	-1.36499	E15.5 * Wnt1 down vs E15.5 * Ctrl
10476910	Cst12	AF440737	0.000314696	-1.36523	E15.5 * Wnt1 down vs E15.5 * Ctrl
10605303	Dnase1l1	NM_027109	8.30673e-005	-1.36538	E15.5 * Wnt1 down vs E15.5 * Ctrl
10408937	Atxn1	NM_009124	0.00694758	-1.36566	E15.5 * Wnt1 down vs E15.5 * Ctrl
10535342			0.00109696	-1.36601	E15.5 * Wnt1 down vs E15.5 * Ctrl
10508759	Rpa2	NM_011284	0.00123507	-1.36614	E15.5 * Wnt1 down vs E15.5 * Ctrl
10420413	Lats2	NM_015771	0.00316221	-1.3662	E15.5 * Wnt1 down vs E15.5 * Ctrl
10460947	Pygm	NM_011224	0.0053863	-1.36637	E15.5 * Wnt1 down vs E15.5 * Ctrl
10519988	AI847670	NM_177869	0.00208294	-1.36667	E15.5 * Wnt1 down vs E15.5 * Ctrl
10582094	Mbtps1	NM_019709	0.00324096	-1.36673	E15.5 * Wnt1 down vs E15.5 * Ctrl
10489357	Jph2	NM_021566	0.000116935	-1.3671	E15.5 * Wnt1 down vs E15.5 * Ctrl
10555681	Stim1	NM_009287	0.00490241	-1.36715	E15.5 * Wnt1 down vs E15.5 * Ctrl
10350102	Ptpn7	NM_177081	0.000287532	-1.36723	E15.5 * Wnt1 down vs E15.5 * Ctrl
10478907	Pard6b	NM_021409	0.000779388	-1.36778	E15.5 * Wnt1 down vs E15.5 * Ctrl
10366277	E2f7	NM_178609	7.4361e-005	-1.36802	E15.5 * Wnt1 down vs E15.5 * Ctrl
10487480	Bub1	NM_001113179	6.93239e-005	-1.36853	E15.5 * Wnt1 down vs E15.5 * Ctrl
10586184	Tipin	NM_025372	6.5082e-006	-1.36879	E15.5 * Wnt1 down vs E15.5 * Ctrl
10404152	Fam65b	NM_029679	0.0031633	-1.36893	E15.5 * Wnt1 down vs E15.5 * Ctrl
10425923	Fam118a	NM_133750	0.000854531	-1.36915	E15.5 * Wnt1 down vs E15.5 * Ctrl
10428736	Derl1	NM_024207	0.00364417	-1.36924	E15.5 * Wnt1 down vs E15.5 * Ctrl
10416989	Gpr180	NM_021434	0.000382073	-1.36931	E15.5 * Wnt1 down vs E15.5 * Ctrl
10385719	Sec24a	NM_175255	3.06615e-005	-1.36952	E15.5 * Wnt1 down vs E15.5 * Ctrl
10441539	Rnaset2a	NM_001083938	1.15978e-008	-1.36954	E15.5 * Wnt1 down vs E15.5 * Ctrl
10529299	Slbp	NM_009193	0.000935995	-1.36971	E15.5 * Wnt1 down vs E15.5 * Ctrl
10529041	Preb	NM_016703	0.00608809	-1.37031	E15.5 * Wnt1 down vs E15.5 * Ctrl
10495935	4930422G04Rik	BC085187	7.24646e-005	-1.37039	E15.5 * Wnt1 down vs E15.5 * Ctrl
10455653	ENSMUSG00000	ENSMUST00000	0.00469483	-1.37049	E15.5 * Wnt1 down vs E15.5 * Ctrl
10354372	EG667952	ENSMUST00000	4.43789e-007	-1.37066	E15.5 * Wnt1 down vs E15.5 * Ctrl
10600301	Ssr4	NM_009279	0.00153597	-1.37092	E15.5 * Wnt1 down vs E15.5 * Ctrl
10455901	Slc27a6	NM_001081072	0.00591305	-1.37116	E15.5 * Wnt1 down vs E15.5 * Ctrl
10481592	Dnm1	NM_010065	0.00118481	-1.37124	E15.5 * Wnt1 down vs E15.5 * Ctrl
10494889	Dennd2c	NM_177857	0.000295999	-1.37156	E15.5 * Wnt1 down vs E15.5 * Ctrl
10538684	Tigd2	NM_001081145	0.00156035	-1.37173	E15.5 * Wnt1 down vs E15.5 * Ctrl
10532150	Fam69a	NM_026062	0.000844967	-1.37177	E15.5 * Wnt1 down vs E15.5 * Ctrl
10571325	Mfhas1	NM_001081279	9.46554e-006	-1.37195	E15.5 * Wnt1 down vs E15.5 * Ctrl
10564451	Mef2a	NM_001033713	0.000129976	-1.37207	E15.5 * Wnt1 down vs E15.5 * Ctrl
10453797	AK220484	NM_001083628	0.00209448	-1.37217	E15.5 * Wnt1 down vs E15.5 * Ctrl

10533034	Ccdc60	NM_177759	0.00185971	-1.37236	E15.5 * Wnt1 down vs E15.5 * Ctrl
10540935	Cand2	NM_025958	0.00679562	-1.37253	E15.5 * Wnt1 down vs E15.5 * Ctrl
10539517	Dysf	NM_021469	1.17583e-005	-1.3726	E15.5 * Wnt1 down vs E15.5 * Ctrl
10366667	Gns	NM_029364	7.34901e-007	-1.37271	E15.5 * Wnt1 down vs E15.5 * Ctrl
10574378	Gins3	NM_030198	0.00104299	-1.3735	E15.5 * Wnt1 down vs E15.5 * Ctrl
10380823	Lasp1	NM_010688	0.00975309	-1.37354	E15.5 * Wnt1 down vs E15.5 * Ctrl
10365482	Timp3	NM_011595	0.0106226	-1.37361	E15.5 * Wnt1 down vs E15.5 * Ctrl
10463277	Nkx2-3	NM_008699	0.000469498	-1.37373	E15.5 * Wnt1 down vs E15.5 * Ctrl
10353844	Neurl3	NM_153408	0.000181354	-1.37387	E15.5 * Wnt1 down vs E15.5 * Ctrl
10496539	Gbp5	NM_153564	0.000904195	-1.37396	E15.5 * Wnt1 down vs E15.5 * Ctrl
10465772	Slc3a2	NM_008577	0.00122051	-1.37407	E15.5 * Wnt1 down vs E15.5 * Ctrl
10465282	Pola2	NM_008893	0.0026773	-1.37453	E15.5 * Wnt1 down vs E15.5 * Ctrl
10506767	Echdc2	NM_026728	0.000202624	-1.37474	E15.5 * Wnt1 down vs E15.5 * Ctrl
10604961	Gabra3	NM_008067	0.00115065	-1.37487	E15.5 * Wnt1 down vs E15.5 * Ctrl
10505064	Tmem38b	NM_028053	0.000106309	-1.37491	E15.5 * Wnt1 down vs E15.5 * Ctrl
10385466	Sgcd	NM_011891	0.00138833	-1.37506	E15.5 * Wnt1 down vs E15.5 * Ctrl
10440037	Nit2	NM_023175	0.000970456	-1.37509	E15.5 * Wnt1 down vs E15.5 * Ctrl
10512939	2610030H06Rik	NM_001081356	0.0122051	-1.3754	E15.5 * Wnt1 down vs E15.5 * Ctrl
10606056	2610030H06Rik	NM_001081356	0.0122051	-1.3754	E15.5 * Wnt1 down vs E15.5 * Ctrl
10514713	Wdr78	NM_146254	0.00263302	-1.37546	E15.5 * Wnt1 down vs E15.5 * Ctrl
10495826	1810037I17Rik	NM_024461	0.00515133	-1.3756	E15.5 * Wnt1 down vs E15.5 * Ctrl
10415425	Cma2	NM_001024714	0.000424038	-1.37571	E15.5 * Wnt1 down vs E15.5 * Ctrl
10546163	Mcm2	NM_008564	0.00162718	-1.37587	E15.5 * Wnt1 down vs E15.5 * Ctrl
10366983	Tmem194	NM_001113211	0.0011037	-1.3761	E15.5 * Wnt1 down vs E15.5 * Ctrl
10353733	Prim2	NM_008922	0.00100399	-1.37636	E15.5 * Wnt1 down vs E15.5 * Ctrl
10466779	Pip5k1b	NM_008846	0.00220823	-1.37654	E15.5 * Wnt1 down vs E15.5 * Ctrl
10518300	Tnfrsf1b	NM_011610	0.00164913	-1.3766	E15.5 * Wnt1 down vs E15.5 * Ctrl
10532819	Kctd10	NM_026145	0.00400246	-1.37674	E15.5 * Wnt1 down vs E15.5 * Ctrl
10392815	AF251705	NM_134158	0.00736425	-1.37681	E15.5 * Wnt1 down vs E15.5 * Ctrl
10522051	Klf3	NM_008453	2.97479e-005	-1.37691	E15.5 * Wnt1 down vs E15.5 * Ctrl
10438572	2510009E07Rik	NM_001001881	6.45175e-006	-1.37729	E15.5 * Wnt1 down vs E15.5 * Ctrl
10488626	Zcchc3	NM_175126	8.50823e-005	-1.37731	E15.5 * Wnt1 down vs E15.5 * Ctrl
10409099	Fam120a	NM_001033268	2.98454e-005	-1.37736	E15.5 * Wnt1 down vs E15.5 * Ctrl
10549473	Caprin2	NM_181541	2.05254e-005	-1.37758	E15.5 * Wnt1 down vs E15.5 * Ctrl
10521832	Pi4k2b	NM_028744	0.00499456	-1.37765	E15.5 * Wnt1 down vs E15.5 * Ctrl
10557816	Bckdk	NM_009739	0.0037595	-1.37783	E15.5 * Wnt1 down vs E15.5 * Ctrl
10462632	Kif20b	NM_183046	8.37215e-005	-1.37784	E15.5 * Wnt1 down vs E15.5 * Ctrl
10506870	Txndc12	NM_025334	0.000755869	-1.37817	E15.5 * Wnt1 down vs E15.5 * Ctrl
10577326	Xkr5	NM_176951	0.00364639	-1.37825	E15.5 * Wnt1 down vs E15.5 * Ctrl
10451225	Polh	NM_030715	0.00110903	-1.37833	E15.5 * Wnt1 down vs E15.5 * Ctrl
10547088	Mbd4	NM_010774	0.00347175	-1.37904	E15.5 * Wnt1 down vs E15.5 * Ctrl
10504957	Smc2	NM_008017	1.17689e-005	-1.37918	E15.5 * Wnt1 down vs E15.5 * Ctrl
10490913	Car3	NM_007606	0.00902132	-1.37926	E15.5 * Wnt1 down vs E15.5 * Ctrl
10474223	Cd59b	NM_181858	0.00373942	-1.37927	E15.5 * Wnt1 down vs E15.5 * Ctrl
10361375	Fbxo5	NM_025995	0.000144036	-1.37928	E15.5 * Wnt1 down vs E15.5 * Ctrl
10602164	Rbbp7	NM_009031	3.58742e-007	-1.37934	E15.5 * Wnt1 down vs E15.5 * Ctrl
10525744	Tmed2	NM_019770	4.00168e-005	-1.37944	E15.5 * Wnt1 down vs E15.5 * Ctrl
10349752	Elk4	NM_007923	0.000262192	-1.37973	E15.5 * Wnt1 down vs E15.5 * Ctrl
10531529	Cnot6l	NM_178854	3.11273e-005	-1.37992	E15.5 * Wnt1 down vs E15.5 * Ctrl
10591537	Tmed1	NM_010744	0.00519354	-1.38004	E15.5 * Wnt1 down vs E15.5 * Ctrl
10421029	Cdca2	NM_175384	6.5054e-006	-1.38008	E15.5 * Wnt1 down vs E15.5 * Ctrl
10406364	2210408I21Rik	NM_001145676	0.0106192	-1.38024	E15.5 * Wnt1 down vs E15.5 * Ctrl
10491704	Spata5	NM_021343	0.00011842	-1.38109	E15.5 * Wnt1 down vs E15.5 * Ctrl
10400006	Ahr	NM_013464	0.000141907	-1.38124	E15.5 * Wnt1 down vs E15.5 * Ctrl
10540650	Arpc4	NM_026552	0.0034638	-1.38137	E15.5 * Wnt1 down vs E15.5 * Ctrl
10364072	Ggt5	NM_011820	9.47246e-006	-1.38139	E15.5 * Wnt1 down vs E15.5 * Ctrl
10566993	Galntl4	NM_173739	0.00262315	-1.38151	E15.5 * Wnt1 down vs E15.5 * Ctrl
10473190	Dnajc10	NM_024181	1.63539e-005	-1.38215	E15.5 * Wnt1 down vs E15.5 * Ctrl
10346562	Cflar	NM_207653	0.000795725	-1.38231	E15.5 * Wnt1 down vs E15.5 * Ctrl
10466104	Ccdc86	NM_023731	0.00284452	-1.38231	E15.5 * Wnt1 down vs E15.5 * Ctrl
10520405	Gm1040	NM_001033457	0.000544071	-1.38234	E15.5 * Wnt1 down vs E15.5 * Ctrl
10409190	Cenpp	NM_025495	0.00743743	-1.38236	E15.5 * Wnt1 down vs E15.5 * Ctrl
10591522	Ap1m2	NM_001110300	0.00137352	-1.38304	E15.5 * Wnt1 down vs E15.5 * Ctrl
10485225	Ext2	NM_010163	9.90554e-005	-1.38317	E15.5 * Wnt1 down vs E15.5 * Ctrl
10537026	Cpa4	NM_027926	0.000386352	-1.38327	E15.5 * Wnt1 down vs E15.5 * Ctrl
10363773	Rhobtb1	NM_001081347	2.2941e-005	-1.38336	E15.5 * Wnt1 down vs E15.5 * Ctrl

10578071	Wrn	NM_011721	0.000214793	-1.38392	E15.5 * Wnt1 down vs E15.5 * Ctrl
10415991	Zfp395	NM_199029	0.001573	-1.38402	E15.5 * Wnt1 down vs E15.5 * Ctrl
10429564	Ly6a	NM_010738	0.00842375	-1.38456	E15.5 * Wnt1 down vs E15.5 * Ctrl
10372208	Acss3	NM_001142804	0.0011334	-1.38479	E15.5 * Wnt1 down vs E15.5 * Ctrl
10480249	Ptpla	NM_013935	0.00182177	-1.38483	E15.5 * Wnt1 down vs E15.5 * Ctrl
10526302	Tbl2	NM_013763	4.3254e-007	-1.38519	E15.5 * Wnt1 down vs E15.5 * Ctrl
10554081	Ttc23	NM_025905	0.00167751	-1.38521	E15.5 * Wnt1 down vs E15.5 * Ctrl
10602865	Pdha1	NM_008810	7.10411e-005	-1.38525	E15.5 * Wnt1 down vs E15.5 * Ctrl
10575160	Nfat5	NM_133957	4.30503e-006	-1.3853	E15.5 * Wnt1 down vs E15.5 * Ctrl
10379060	Proca1	NM_001045516	9.48337e-005	-1.3856	E15.5 * Wnt1 down vs E15.5 * Ctrl
10520604	Emilin1	NM_133918	0.00383798	-1.3859	E15.5 * Wnt1 down vs E15.5 * Ctrl
10373569	Rpsa	NM_011029	0.00444621	-1.38608	E15.5 * Wnt1 down vs E15.5 * Ctrl
10465059	Ctsw	NM_009985	0.000576141	-1.38616	E15.5 * Wnt1 down vs E15.5 * Ctrl
10505779	Acer2	NM_139306	0.00178533	-1.38621	E15.5 * Wnt1 down vs E15.5 * Ctrl
10463803	Slk	NM_009289	1.05632e-005	-1.38648	E15.5 * Wnt1 down vs E15.5 * Ctrl
10479063	Rab22a	NM_024436	0.000720237	-1.38664	E15.5 * Wnt1 down vs E15.5 * Ctrl
10356880	St8sia4	NM_009183	0.000832931	-1.38675	E15.5 * Wnt1 down vs E15.5 * Ctrl
10590620	Ccr9	NM_009913	0.00117142	-1.38705	E15.5 * Wnt1 down vs E15.5 * Ctrl
10451004	Cd2ap	NM_009847	4.41966e-006	-1.38739	E15.5 * Wnt1 down vs E15.5 * Ctrl
10473160	Ssfa2	NM_080558	0.00095203	-1.3874	E15.5 * Wnt1 down vs E15.5 * Ctrl
10584067	Zbtb44	NM_001115130	2.51313e-005	-1.38743	E15.5 * Wnt1 down vs E15.5 * Ctrl
10572800	Klf2	NM_008452	0.00690785	-1.38778	E15.5 * Wnt1 down vs E15.5 * Ctrl
10473491	Olfr1033	NM_146578	0.000386823	-1.38784	E15.5 * Wnt1 down vs E15.5 * Ctrl
10573867	Rbl2	NM_011250	0.00191977	-1.38806	E15.5 * Wnt1 down vs E15.5 * Ctrl
10411915	Ppwd1	NM_172807	2.15885e-005	-1.38807	E15.5 * Wnt1 down vs E15.5 * Ctrl
10568705	B830028B13Rik	NM_001143802	0.00571961	-1.38861	E15.5 * Wnt1 down vs E15.5 * Ctrl
10404036	Hist1h2bg	NM_178196	0.0129712	-1.38888	E15.5 * Wnt1 down vs E15.5 * Ctrl
10503251	2610301B20Rik	NM_026005	0.00304711	-1.38897	E15.5 * Wnt1 down vs E15.5 * Ctrl
10564869	Cib1	NM_011870	0.000592327	-1.38918	E15.5 * Wnt1 down vs E15.5 * Ctrl
10597354	Pdcd6ip	NM_011052	2.10853e-005	-1.38922	E15.5 * Wnt1 down vs E15.5 * Ctrl
10471586	Hspa5	NM_022310	8.49178e-006	-1.38945	E15.5 * Wnt1 down vs E15.5 * Ctrl
10420488	D14Ertd668e	BC145673	9.5576e-005	-1.38967	E15.5 * Wnt1 down vs E15.5 * Ctrl
10570068	Col4a2	NM_009932	1.42285e-005	-1.39001	E15.5 * Wnt1 down vs E15.5 * Ctrl
10535771	Cdx2	NM_007673	0.00194887	-1.39008	E15.5 * Wnt1 down vs E15.5 * Ctrl
10355967	Ap1s3	NM_183027	0.000943411	-1.39022	E15.5 * Wnt1 down vs E15.5 * Ctrl
10600597	Tmem47	NM_175771	3.75212e-005	-1.39025	E15.5 * Wnt1 down vs E15.5 * Ctrl
10407709	Mtr	NM_001081128	0.000525779	-1.39028	E15.5 * Wnt1 down vs E15.5 * Ctrl
10456490		---	0.00430062	-1.39035	E15.5 * Wnt1 down vs E15.5 * Ctrl
10480901	Nacc2	NM_001037098	4.35182e-006	-1.39037	E15.5 * Wnt1 down vs E15.5 * Ctrl
10584317	Esam	NM_027102	0.000980485	-1.39039	E15.5 * Wnt1 down vs E15.5 * Ctrl
10347115		---	0.00439934	-1.39051	E15.5 * Wnt1 down vs E15.5 * Ctrl
10456545	Ccdc68	NM_201362	0.00420805	-1.3908	E15.5 * Wnt1 down vs E15.5 * Ctrl
10428070	9430069I07Rik	AK020480	0.00320735	-1.39086	E15.5 * Wnt1 down vs E15.5 * Ctrl
10390505	Rpl23	AF357384	0.00366447	-1.39088	E15.5 * Wnt1 down vs E15.5 * Ctrl
10567010	Dkk3	NM_015814	0.000189685	-1.39108	E15.5 * Wnt1 down vs E15.5 * Ctrl
10510144	OTTMUSG00000	NM_001014397	0.0103001	-1.39118	E15.5 * Wnt1 down vs E15.5 * Ctrl
10404840	Cd83	NM_009856	0.000153183	-1.39131	E15.5 * Wnt1 down vs E15.5 * Ctrl
10462499	A1cf	AY566862	0.000340196	-1.39134	E15.5 * Wnt1 down vs E15.5 * Ctrl
10436106	C330027C09Rik	NM_172616	0.000729895	-1.39138	E15.5 * Wnt1 down vs E15.5 * Ctrl
10556790	2610020H08Rik	NM_028129	0.00565651	-1.3914	E15.5 * Wnt1 down vs E15.5 * Ctrl
10453600	Cul2	ENSMUST00000	7.24037e-006	-1.39148	E15.5 * Wnt1 down vs E15.5 * Ctrl
10378453	1300001I01Rik	BC072573	0.00462824	-1.39154	E15.5 * Wnt1 down vs E15.5 * Ctrl
10498599	Ift80	NM_026641	0.00100084	-1.39158	E15.5 * Wnt1 down vs E15.5 * Ctrl
10584422	Olfr908	NM_146872	0.000200548	-1.392	E15.5 * Wnt1 down vs E15.5 * Ctrl
10387797	Bcl6b	NM_007528	0.00191493	-1.39219	E15.5 * Wnt1 down vs E15.5 * Ctrl
10344939	Terf1	NM_009352	2.79749e-006	-1.39221	E15.5 * Wnt1 down vs E15.5 * Ctrl
10425781	Serhl	NM_023475	2.30571e-005	-1.39229	E15.5 * Wnt1 down vs E15.5 * Ctrl
10503399	EG667952	ENSMUST00000	1.22122e-005	-1.39237	E15.5 * Wnt1 down vs E15.5 * Ctrl
10387743	Slc2a4	NM_009204	0.000168784	-1.39245	E15.5 * Wnt1 down vs E15.5 * Ctrl
10595570	Slc25a40	NM_178766	0.0132792	-1.39257	E15.5 * Wnt1 down vs E15.5 * Ctrl
10421269	Sorbs3	NM_011366	0.000473742	-1.39307	E15.5 * Wnt1 down vs E15.5 * Ctrl
10587639	Nt5e	NM_011851	0.002625	-1.39312	E15.5 * Wnt1 down vs E15.5 * Ctrl
10545255	Rpia	NM_009075	0.000220219	-1.39349	E15.5 * Wnt1 down vs E15.5 * Ctrl
10420030	Psme2	NM_011190	8.57026e-005	-1.39355	E15.5 * Wnt1 down vs E15.5 * Ctrl
10557156	Plk1	NM_011121	0.000686036	-1.39373	E15.5 * Wnt1 down vs E15.5 * Ctrl
10536917	Smo	NM_176996	0.00245846	-1.39382	E15.5 * Wnt1 down vs E15.5 * Ctrl

10445688	Ccnd3	NM_001081636	7.60403e-005	-1.39384	E15.5 * Wnt1 down vs E15.5 * Ctrl
10589889	Glb1	NM_009752	0.00717324	-1.39429	E15.5 * Wnt1 down vs E15.5 * Ctrl
10452879	Nlrc4	NM_001033367	4.20605e-005	-1.39436	E15.5 * Wnt1 down vs E15.5 * Ctrl
10519504	Sri	NM_025618	4.7398e-005	-1.39437	E15.5 * Wnt1 down vs E15.5 * Ctrl
10420483	Phf11	NM_172603	0.00259309	-1.39439	E15.5 * Wnt1 down vs E15.5 * Ctrl
10494595	Notch2	NM_010928	0.000588114	-1.39448	E15.5 * Wnt1 down vs E15.5 * Ctrl
10406287	Ttc37	NM_001081352	0.00010154	-1.39459	E15.5 * Wnt1 down vs E15.5 * Ctrl
10461176	Stx5a	NM_019829	0.000418615	-1.39461	E15.5 * Wnt1 down vs E15.5 * Ctrl
10409990	6720489N17Rik	BC053725	0.014354	-1.39465	E15.5 * Wnt1 down vs E15.5 * Ctrl
10499113	Gm1019	NM_001001650	0.000136037	-1.39535	E15.5 * Wnt1 down vs E15.5 * Ctrl
10495933	4930422G04Rik	BC079891	0.000550445	-1.39543	E15.5 * Wnt1 down vs E15.5 * Ctrl
10384154	Myo1g	NM_178440	0.00015766	-1.39573	E15.5 * Wnt1 down vs E15.5 * Ctrl
10547322	Cacna1c	NM_001159533	5.44109e-006	-1.39584	E15.5 * Wnt1 down vs E15.5 * Ctrl
10534909	Sp110	NM_175397	0.00198078	-1.39607	E15.5 * Wnt1 down vs E15.5 * Ctrl
10598839	Rp2h	NM_133669	0.00599685	-1.39609	E15.5 * Wnt1 down vs E15.5 * Ctrl
10350977	4930523C07Rik	ENSMUST00000(	0.000120362	-1.39609	E15.5 * Wnt1 down vs E15.5 * Ctrl
10406530	Tmem167	NM_025335	0.00395004	-1.39639	E15.5 * Wnt1 down vs E15.5 * Ctrl
10350146	Phlda3	NM_013750	0.00127507	-1.39664	E15.5 * Wnt1 down vs E15.5 * Ctrl
10579525	Plvap	NM_032398	0.00104837	-1.39677	E15.5 * Wnt1 down vs E15.5 * Ctrl
10581222	Fhod1	NM_177699	0.000637296	-1.39699	E15.5 * Wnt1 down vs E15.5 * Ctrl
10379633	Slfn1	NM_011407	0.00023023	-1.39706	E15.5 * Wnt1 down vs E15.5 * Ctrl
10556302	Ampd3	NM_009667	0.00467941	-1.39716	E15.5 * Wnt1 down vs E15.5 * Ctrl
10547894	Cd4	NM_013488	6.1791e-005	-1.39725	E15.5 * Wnt1 down vs E15.5 * Ctrl
10504424	Reck	NM_016678	7.2444e-007	-1.39744	E15.5 * Wnt1 down vs E15.5 * Ctrl
10376285	Sap30l	NM_001081168	0.00311093	-1.39757	E15.5 * Wnt1 down vs E15.5 * Ctrl
10531183	Adamts3	NM_001081401	0.00203901	-1.39774	E15.5 * Wnt1 down vs E15.5 * Ctrl
10392845	Cd300lf	NM_145634	0.00163612	-1.39779	E15.5 * Wnt1 down vs E15.5 * Ctrl
10508860	Slc9a1	NM_016981	0.00012247	-1.39805	E15.5 * Wnt1 down vs E15.5 * Ctrl
10600825	Zc3h12b	NM_001034907	0.000525842	-1.39819	E15.5 * Wnt1 down vs E15.5 * Ctrl
10585703	Rpp25	NM_133982	0.000850211	-1.39834	E15.5 * Wnt1 down vs E15.5 * Ctrl
10509246	Luzp1	NM_024452	1.46364e-006	-1.39846	E15.5 * Wnt1 down vs E15.5 * Ctrl
10507190	4732418C07Rik	BC059213	0.000106254	-1.39876	E15.5 * Wnt1 down vs E15.5 * Ctrl
10502052	Alpk1	ENSMUST00000(	0.00405239	-1.3988	E15.5 * Wnt1 down vs E15.5 * Ctrl
10430931	Nfam1	NM_028728	0.000532512	-1.39881	E15.5 * Wnt1 down vs E15.5 * Ctrl
10600698	5430427O19Rik	BC115576	0.00753697	-1.39891	E15.5 * Wnt1 down vs E15.5 * Ctrl
10571036	Ppapdc1b	NM_028000	2.40643e-005	-1.39902	E15.5 * Wnt1 down vs E15.5 * Ctrl
10403959	RP23-38E20.1	NM_001097979	0.00941918	-1.39928	E15.5 * Wnt1 down vs E15.5 * Ctrl
10408087	RP23-38E20.1	NM_001097979	0.00941918	-1.39928	E15.5 * Wnt1 down vs E15.5 * Ctrl
10532744	Selplg	NM_009151	0.00242036	-1.39939	E15.5 * Wnt1 down vs E15.5 * Ctrl
10602223	Alg13	NM_026247	1.81271e-005	-1.39947	E15.5 * Wnt1 down vs E15.5 * Ctrl
10606654	Xkrx	NM_183319	0.00626263	-1.39966	E15.5 * Wnt1 down vs E15.5 * Ctrl
10502522	Hs2st1	NM_011828	9.51758e-009	-1.39999	E15.5 * Wnt1 down vs E15.5 * Ctrl
10367641	Mthfd1l	NM_172308	1.18584e-006	-1.40004	E15.5 * Wnt1 down vs E15.5 * Ctrl
10517443	Tceb3	NM_013736	0.000192071	-1.40029	E15.5 * Wnt1 down vs E15.5 * Ctrl
10372781	Irak3	NM_028679	0.00217465	-1.40038	E15.5 * Wnt1 down vs E15.5 * Ctrl
10384622	Ehbp1	NM_153078	0.000242249	-1.4006	E15.5 * Wnt1 down vs E15.5 * Ctrl
10479221	OTTMUSG00000	ENSMUST00000	0.00662496	-1.40067	E15.5 * Wnt1 down vs E15.5 * Ctrl
10526754	Ap4m1	NM_021392	0.0111077	-1.40105	E15.5 * Wnt1 down vs E15.5 * Ctrl
10347980	Itm2c	NM_022417	0.00264808	-1.40135	E15.5 * Wnt1 down vs E15.5 * Ctrl
10547926	Cops7a	NM_012003	0.00056947	-1.40167	E15.5 * Wnt1 down vs E15.5 * Ctrl
10410173	Hiatl1	NM_133680	0.0065814	-1.40198	E15.5 * Wnt1 down vs E15.5 * Ctrl
10598180	BC085271	BC085271	0.00193523	-1.40203	E15.5 * Wnt1 down vs E15.5 * Ctrl
10349051	Tnfrsf11a	NM_009399	0.000456425	-1.40212	E15.5 * Wnt1 down vs E15.5 * Ctrl
10396671	Plekhg3	NM_153804	1.46383e-005	-1.40227	E15.5 * Wnt1 down vs E15.5 * Ctrl
10379190	Vtn	NM_011707	0.00293462	-1.40261	E15.5 * Wnt1 down vs E15.5 * Ctrl
10487577	Ckap2l	NM_181589	0.000170339	-1.40277	E15.5 * Wnt1 down vs E15.5 * Ctrl
10465132	Sipa1	NM_011379	0.000207749	-1.40288	E15.5 * Wnt1 down vs E15.5 * Ctrl
10584024	Opcml	NM_177906	0.00187289	-1.40299	E15.5 * Wnt1 down vs E15.5 * Ctrl
10375229		---	0.00805711	-1.40304	E15.5 * Wnt1 down vs E15.5 * Ctrl
10484307	Frzb	NM_011356	0.00638801	-1.40319	E15.5 * Wnt1 down vs E15.5 * Ctrl
10417147	Tm9sf2	NM_080556	8.85687e-005	-1.40341	E15.5 * Wnt1 down vs E15.5 * Ctrl
10580282	Junb	NM_008416	0.000175349	-1.40346	E15.5 * Wnt1 down vs E15.5 * Ctrl
10598507	Slc38a5	NM_172479	0.00172194	-1.40357	E15.5 * Wnt1 down vs E15.5 * Ctrl
10490104	Aurka	NM_011497	0.000156143	-1.40407	E15.5 * Wnt1 down vs E15.5 * Ctrl
10385153	Ccdc99	NM_027411	0.0028063	-1.40418	E15.5 * Wnt1 down vs E15.5 * Ctrl
10385533	Tgtp	NM_011579	0.00240604	-1.40475	E15.5 * Wnt1 down vs E15.5 * Ctrl

10400515	Sec23a	NM_009147	0.000851042	-1.40476	E15.5 * Wnt1 down vs E15.5 * Ctrl
10506880	Kti12	NM_029571	0.00894871	-1.40482	E15.5 * Wnt1 down vs E15.5 * Ctrl
10402399	Serpina1a	NM_009243	0.00251924	-1.40484	E15.5 * Wnt1 down vs E15.5 * Ctrl
10531166	Adamts3	NM_001081401	0.0006099	-1.40513	E15.5 * Wnt1 down vs E15.5 * Ctrl
10508115	Stk40	NM_001145827	0.00405194	-1.40539	E15.5 * Wnt1 down vs E15.5 * Ctrl
10502776	Lphn2	NM_001081298	4.83234e-007	-1.40556	E15.5 * Wnt1 down vs E15.5 * Ctrl
10384961	Stc2	NM_011491	0.00392842	-1.40561	E15.5 * Wnt1 down vs E15.5 * Ctrl
10598612	Otc	NM_008769	0.0035055	-1.40581	E15.5 * Wnt1 down vs E15.5 * Ctrl
10440307	Chmp2b	NM_026879	1.56546e-005	-1.40635	E15.5 * Wnt1 down vs E15.5 * Ctrl
10382998	Birc5	NM_001012273	0.000388537	-1.40642	E15.5 * Wnt1 down vs E15.5 * Ctrl
10401028	Sgpp1	NM_030750	0.00350649	-1.40651	E15.5 * Wnt1 down vs E15.5 * Ctrl
10416355	Rcbtb2	NM_134083	0.000107275	-1.40658	E15.5 * Wnt1 down vs E15.5 * Ctrl
10454786	Ctnna1	NM_009818	2.10691e-005	-1.40665	E15.5 * Wnt1 down vs E15.5 * Ctrl
10360934	Rrp15	NM_026041	8.34578e-006	-1.40666	E15.5 * Wnt1 down vs E15.5 * Ctrl
10516765	Serinc2	NM_172702	9.18548e-005	-1.4068	E15.5 * Wnt1 down vs E15.5 * Ctrl
10452709	Ndc80	NM_023294	0.000143774	-1.40686	E15.5 * Wnt1 down vs E15.5 * Ctrl
10352709	Nsl1	NM_198654	7.03598e-005	-1.40715	E15.5 * Wnt1 down vs E15.5 * Ctrl
10524983	Med13l	NM_172424	0.000607331	-1.40721	E15.5 * Wnt1 down vs E15.5 * Ctrl
10407281	Esm1	NM_023612	0.000381772	-1.40741	E15.5 * Wnt1 down vs E15.5 * Ctrl
10439249	Parp14	NM_001039530	0.000565725	-1.40741	E15.5 * Wnt1 down vs E15.5 * Ctrl
10486061	Rasgrp1	NM_011246	0.0019964	-1.40743	E15.5 * Wnt1 down vs E15.5 * Ctrl
10510215	OTTMUSG00000	NM_001083918	0.00660643	-1.40797	E15.5 * Wnt1 down vs E15.5 * Ctrl
10557009	Eef2k	NM_007908	4.82011e-006	-1.40801	E15.5 * Wnt1 down vs E15.5 * Ctrl
10563178	Cd37	NM_007645	0.000888824	-1.40807	E15.5 * Wnt1 down vs E15.5 * Ctrl
10366886	Arhgap9	NM_146011	0.000243895	-1.40818	E15.5 * Wnt1 down vs E15.5 * Ctrl
10463687	D19Wsu162e	BC058949	0.000321418	-1.40821	E15.5 * Wnt1 down vs E15.5 * Ctrl
10552500	Klk8	NM_008940	0.000297358	-1.40862	E15.5 * Wnt1 down vs E15.5 * Ctrl
10417095	Farp1	NM_134082	0.00179402	-1.40887	E15.5 * Wnt1 down vs E15.5 * Ctrl
10574962	Nfatc3	NM_010901	5.93667e-007	-1.40921	E15.5 * Wnt1 down vs E15.5 * Ctrl
10416850	Scel	NM_022886	2.94509e-005	-1.40929	E15.5 * Wnt1 down vs E15.5 * Ctrl
10500103	Gabpb2	NM_029885	3.18352e-007	-1.40933	E15.5 * Wnt1 down vs E15.5 * Ctrl
10519555	Abcb1b	NM_011075	0.00456296	-1.40937	E15.5 * Wnt1 down vs E15.5 * Ctrl
10372766	Helb	NM_080446	0.00340096	-1.40967	E15.5 * Wnt1 down vs E15.5 * Ctrl
10419216	Gnpnat1	NM_019425	0.000203995	-1.40978	E15.5 * Wnt1 down vs E15.5 * Ctrl
10411668	Ocln	NM_008756	0.000282719	-1.40985	E15.5 * Wnt1 down vs E15.5 * Ctrl
10554325	5730590G19Rik	NM_029835	0.0018645	-1.41007	E15.5 * Wnt1 down vs E15.5 * Ctrl
10585721	Scamp2	NM_022813	0.000181958	-1.41025	E15.5 * Wnt1 down vs E15.5 * Ctrl
10577757	Adam9	NM_007404	1.30116e-005	-1.41083	E15.5 * Wnt1 down vs E15.5 * Ctrl
10417759	Ube2e2	NM_144839	0.000697325	-1.41086	E15.5 * Wnt1 down vs E15.5 * Ctrl
10385870	Irf1	NM_008390	4.70299e-006	-1.41109	E15.5 * Wnt1 down vs E15.5 * Ctrl
10383152		---	3.74731e-006	-1.41156	E15.5 * Wnt1 down vs E15.5 * Ctrl
10438749		---	0.00131725	-1.41156	E15.5 * Wnt1 down vs E15.5 * Ctrl
10571984	Ddx60	NM_001081215	6.21777e-005	-1.41183	E15.5 * Wnt1 down vs E15.5 * Ctrl
10605437	Pls3	NM_145629	0.000157146	-1.41183	E15.5 * Wnt1 down vs E15.5 * Ctrl
10531790	Nkx6-1	NM_144955	4.50211e-005	-1.41217	E15.5 * Wnt1 down vs E15.5 * Ctrl
10461408	Rab3il1	NM_144538	6.22717e-005	-1.41217	E15.5 * Wnt1 down vs E15.5 * Ctrl
10359097	Tor1aip1	NM_144791	1.18863e-005	-1.41263	E15.5 * Wnt1 down vs E15.5 * Ctrl
10452508	Twsg1	NM_023053	4.4591e-007	-1.41263	E15.5 * Wnt1 down vs E15.5 * Ctrl
10430344	Il2rb	NM_008368	0.00208787	-1.41264	E15.5 * Wnt1 down vs E15.5 * Ctrl
10371387	Ckap4	NM_175451	3.71481e-006	-1.41278	E15.5 * Wnt1 down vs E15.5 * Ctrl
10387890	Cxcl16	NM_023158	6.64656e-005	-1.41279	E15.5 * Wnt1 down vs E15.5 * Ctrl
10582260		---	0.00102097	-1.41292	E15.5 * Wnt1 down vs E15.5 * Ctrl
10542745	Fgfr1op2	NM_026218	0.000239401	-1.41294	E15.5 * Wnt1 down vs E15.5 * Ctrl
10416843	Cln5	NM_001033242	0.000390246	-1.41304	E15.5 * Wnt1 down vs E15.5 * Ctrl
10455948	Chsy3	NM_001081328	0.000355877	-1.4134	E15.5 * Wnt1 down vs E15.5 * Ctrl
10511679	Decr1	NM_026172	3.95109e-005	-1.41344	E15.5 * Wnt1 down vs E15.5 * Ctrl
10375614	Gfpt2	NM_013529	0.000503033	-1.41371	E15.5 * Wnt1 down vs E15.5 * Ctrl
10528821	ENSMUSG00000	NM_001134299	9.6416e-005	-1.41376	E15.5 * Wnt1 down vs E15.5 * Ctrl
10438738	Bcl6	NM_009744	0.000205649	-1.41396	E15.5 * Wnt1 down vs E15.5 * Ctrl
10464425	Grk5	NM_018869	0.000484596	-1.41411	E15.5 * Wnt1 down vs E15.5 * Ctrl
10511498	Plekhf2	NM_175175	2.59505e-006	-1.41411	E15.5 * Wnt1 down vs E15.5 * Ctrl
10542965	Sgce	NM_001130190	1.62417e-005	-1.41439	E15.5 * Wnt1 down vs E15.5 * Ctrl
10376733	Map2k3	NM_008928	0.000167991	-1.41443	E15.5 * Wnt1 down vs E15.5 * Ctrl
10388409	Rpa1	NM_026653	4.85001e-005	-1.41468	E15.5 * Wnt1 down vs E15.5 * Ctrl
10596442	Ppm1m	NM_198931	0.000483827	-1.41512	E15.5 * Wnt1 down vs E15.5 * Ctrl
10490854	OTTMUSG00000	NM_001037926	0.00147672	-1.41518	E15.5 * Wnt1 down vs E15.5 * Ctrl

10603011	Rbbp7	NM_009031	2.57482e-010	-1.41536	E15.5 * Wnt1 down vs E15.5 * Ctrl
10478678	Cd40	NM_170701	1.98403e-005	-1.41549	E15.5 * Wnt1 down vs E15.5 * Ctrl
10560139	B430211C08Rik	NM_001039146	1.01971e-005	-1.41596	E15.5 * Wnt1 down vs E15.5 * Ctrl
10541091	Gemin6	NM_026053	5.13958e-005	-1.41598	E15.5 * Wnt1 down vs E15.5 * Ctrl
10346810	Pard3b	NM_001081050	0.00021108	-1.41606	E15.5 * Wnt1 down vs E15.5 * Ctrl
10478890	Cebpb	NM_009883	0.00189146	-1.4162	E15.5 * Wnt1 down vs E15.5 * Ctrl
10487021	Slc30a4	NM_011774	0.00232656	-1.41624	E15.5 * Wnt1 down vs E15.5 * Ctrl
10569504	Tnfrsf23	NM_024290	0.000204535	-1.41631	E15.5 * Wnt1 down vs E15.5 * Ctrl
10474825	D2Ertd750e	NM_026412	0.00131228	-1.4168	E15.5 * Wnt1 down vs E15.5 * Ctrl
10490989	Cp	NM_001042611	0.000121496	-1.41683	E15.5 * Wnt1 down vs E15.5 * Ctrl
10477495	U46068	NM_001012392	0.00215778	-1.41692	E15.5 * Wnt1 down vs E15.5 * Ctrl
10543319	Fam3c	NM_138587	8.38939e-005	-1.41733	E15.5 * Wnt1 down vs E15.5 * Ctrl
10495035	Slc16a1	NM_009196	0.00540933	-1.4175	E15.5 * Wnt1 down vs E15.5 * Ctrl
10590060	Ctdspl	NM_133710	6.9655e-006	-1.41763	E15.5 * Wnt1 down vs E15.5 * Ctrl
10544660	Osbpl3	NM_027881	1.57652e-005	-1.41776	E15.5 * Wnt1 down vs E15.5 * Ctrl
10360406	Ifi205	NM_172648	6.51333e-005	-1.41791	E15.5 * Wnt1 down vs E15.5 * Ctrl
10474875	Casc5	NM_029617	0.000108573	-1.41817	E15.5 * Wnt1 down vs E15.5 * Ctrl
10522467	Rasl11b	NM_026878	0.00271259	-1.41822	E15.5 * Wnt1 down vs E15.5 * Ctrl
10501811	Slc44a3	NM_145394	6.5358e-005	-1.41836	E15.5 * Wnt1 down vs E15.5 * Ctrl
10395733	Npas3	NM_013780	0.00313221	-1.41851	E15.5 * Wnt1 down vs E15.5 * Ctrl
10566144	Trim21	NM_009277	0.000340249	-1.41861	E15.5 * Wnt1 down vs E15.5 * Ctrl
10594840	Gcom1	NM_001033208	0.00117689	-1.41872	E15.5 * Wnt1 down vs E15.5 * Ctrl
10544610	Igf2bp3	NM_023670	5.45101e-006	-1.41877	E15.5 * Wnt1 down vs E15.5 * Ctrl
10492402	Kcnab1	NM_010597	0.000115978	-1.41878	E15.5 * Wnt1 down vs E15.5 * Ctrl
10487321	2010106G01Rik	NM_023220	2.18334e-005	-1.41897	E15.5 * Wnt1 down vs E15.5 * Ctrl
10584162	Tmed2	NM_019770	2.18422e-005	-1.41901	E15.5 * Wnt1 down vs E15.5 * Ctrl
10519196	Vwa1	NM_147776	0.00389587	-1.41968	E15.5 * Wnt1 down vs E15.5 * Ctrl
10490384	Lama5	NM_001081171	1.45331e-005	-1.41978	E15.5 * Wnt1 down vs E15.5 * Ctrl
10587508	Ttk	NM_009445	1.56446e-006	-1.4198	E15.5 * Wnt1 down vs E15.5 * Ctrl
10420372	Cryl1	NM_030004	0.000104767	-1.41987	E15.5 * Wnt1 down vs E15.5 * Ctrl
10528815	5031410I06Rik	NM_207657	0.000104137	-1.42006	E15.5 * Wnt1 down vs E15.5 * Ctrl
10559261	Cd81	NM_133655	3.74331e-006	-1.4201	E15.5 * Wnt1 down vs E15.5 * Ctrl
10503617	F730047E07Rik	NM_199467	0.00013804	-1.42046	E15.5 * Wnt1 down vs E15.5 * Ctrl
10602756	Smpx	NM_025357	0.000493825	-1.42089	E15.5 * Wnt1 down vs E15.5 * Ctrl
10420426	F630043A04Rik	NM_198605	0.000170146	-1.42123	E15.5 * Wnt1 down vs E15.5 * Ctrl
10368654	Nt5dc1	NM_176968	0.000436838	-1.42123	E15.5 * Wnt1 down vs E15.5 * Ctrl
10492102	Spg20	NM_144895	0.00122357	-1.42125	E15.5 * Wnt1 down vs E15.5 * Ctrl
10580219	Calr	NM_007591	2.47833e-005	-1.42132	E15.5 * Wnt1 down vs E15.5 * Ctrl
10350123	Lmod1	NM_053106	0.000173918	-1.42137	E15.5 * Wnt1 down vs E15.5 * Ctrl
10571114	Erlin2	NM_153592	8.41178e-005	-1.42164	E15.5 * Wnt1 down vs E15.5 * Ctrl
10347792		---	0.00266964	-1.42174	E15.5 * Wnt1 down vs E15.5 * Ctrl
10549377	1700034J05Rik	BC052362	0.00551625	-1.42191	E15.5 * Wnt1 down vs E15.5 * Ctrl
10386370	Arf1	NM_007476	0.00294841	-1.42192	E15.5 * Wnt1 down vs E15.5 * Ctrl
10562761	Clec11a	NM_009131	0.00232575	-1.42205	E15.5 * Wnt1 down vs E15.5 * Ctrl
10421774	Dgkh	NM_001081336	0.00186512	-1.42207	E15.5 * Wnt1 down vs E15.5 * Ctrl
10461636		---	7.54106e-005	-1.42211	E15.5 * Wnt1 down vs E15.5 * Ctrl
10528015	Steap1	NM_027399	0.00150518	-1.42218	E15.5 * Wnt1 down vs E15.5 * Ctrl
10447675	Rnaset2a	NM_001083938	3.07024e-008	-1.42221	E15.5 * Wnt1 down vs E15.5 * Ctrl
10394978	Rrm2	NM_009104	1.21556e-007	-1.4223	E15.5 * Wnt1 down vs E15.5 * Ctrl
10356084	Irs1	NM_010570	0.00317692	-1.4224	E15.5 * Wnt1 down vs E15.5 * Ctrl
10508734	Ptafr	NM_001081211	0.000104891	-1.42285	E15.5 * Wnt1 down vs E15.5 * Ctrl
10456653	Myo5b	NM_201600	5.46473e-005	-1.42289	E15.5 * Wnt1 down vs E15.5 * Ctrl
10596383	E330026B02Rik	NM_001102607	0.00032676	-1.4232	E15.5 * Wnt1 down vs E15.5 * Ctrl
10459227		---	0.0117531	-1.42334	E15.5 * Wnt1 down vs E15.5 * Ctrl
10562500	Dpy19l3	NM_178704	9.38022e-008	-1.42357	E15.5 * Wnt1 down vs E15.5 * Ctrl
10592251	Pknox2	NM_001029838	0.000665712	-1.42374	E15.5 * Wnt1 down vs E15.5 * Ctrl
10393431	Tk1	NM_009387	0.000511333	-1.42431	E15.5 * Wnt1 down vs E15.5 * Ctrl
10563338	Myd116	NM_008654	0.00107793	-1.42462	E15.5 * Wnt1 down vs E15.5 * Ctrl
10451472	Rpl7l1	NM_025433	0.000107798	-1.4248	E15.5 * Wnt1 down vs E15.5 * Ctrl
10529485	Htra3	NM_030127	2.81895e-006	-1.42484	E15.5 * Wnt1 down vs E15.5 * Ctrl
10607712	Grpr	NM_008177	1.03698e-006	-1.42486	E15.5 * Wnt1 down vs E15.5 * Ctrl
10503241	Gdf6	NM_013526	0.00873919	-1.42517	E15.5 * Wnt1 down vs E15.5 * Ctrl
10474381	Kif18a	NM_139303	0.000260041	-1.42534	E15.5 * Wnt1 down vs E15.5 * Ctrl
10519497	Steap4	NM_054098	0.00034456	-1.42537	E15.5 * Wnt1 down vs E15.5 * Ctrl
10439483	Cdgap	NM_020260	0.000224082	-1.42541	E15.5 * Wnt1 down vs E15.5 * Ctrl
10569429	Cdkn1c	NM_009876	1.43491e-007	-1.4256	E15.5 * Wnt1 down vs E15.5 * Ctrl

10355329	Bard1	NM_007525	5.82721e-005	-1.42577	E15.5 * Wnt1 down vs E15.5 * Ctrl
10572865	Isx	NM_027837	0.00133539	-1.42615	E15.5 * Wnt1 down vs E15.5 * Ctrl
10430358	C1qtnf6	NM_028331	0.00151268	-1.42624	E15.5 * Wnt1 down vs E15.5 * Ctrl
10399605	Adam17	NM_009615	5.65357e-005	-1.42657	E15.5 * Wnt1 down vs E15.5 * Ctrl
10518335	OTTMUSG00000	NM_001083918	7.53014e-005	-1.42677	E15.5 * Wnt1 down vs E15.5 * Ctrl
10436428	Mina	NM_025910	0.00054526	-1.42722	E15.5 * Wnt1 down vs E15.5 * Ctrl
10552938	Ntf5	NM_198190	0.00043629	-1.42787	E15.5 * Wnt1 down vs E15.5 * Ctrl
10601778	Armcx3	NM_027870	7.44855e-007	-1.42798	E15.5 * Wnt1 down vs E15.5 * Ctrl
10373396	EG667952	ENSMUST00000(	2.42231e-006	-1.42827	E15.5 * Wnt1 down vs E15.5 * Ctrl
10475437	Sord	NM_146126	0.00626879	-1.42837	E15.5 * Wnt1 down vs E15.5 * Ctrl
10533929	Scarb1	NM_016741	0.0019899	-1.42856	E15.5 * Wnt1 down vs E15.5 * Ctrl
10369116	Mcm9	NM_027830	0.00149294	-1.42868	E15.5 * Wnt1 down vs E15.5 * Ctrl
10473432	Tnks1bp1	NM_001081260	0.000134877	-1.42877	E15.5 * Wnt1 down vs E15.5 * Ctrl
10490731	Sox18	NM_009236	0.000645671	-1.4288	E15.5 * Wnt1 down vs E15.5 * Ctrl
10586448	2810417H13Rik	NM_026515	0.00115737	-1.42895	E15.5 * Wnt1 down vs E15.5 * Ctrl
10465474	Prdx5	NM_012021	2.86415e-006	-1.42958	E15.5 * Wnt1 down vs E15.5 * Ctrl
10485191	Prdm11	ENSMUST00000	0.000813526	-1.42975	E15.5 * Wnt1 down vs E15.5 * Ctrl
10423593	Laptm4b	NM_033521	6.18024e-005	-1.43037	E15.5 * Wnt1 down vs E15.5 * Ctrl
10472782	Hat1	NM_026115	3.2819e-005	-1.43039	E15.5 * Wnt1 down vs E15.5 * Ctrl
10356461	6430706D22Rik	NM_198652	0.000306614	-1.43048	E15.5 * Wnt1 down vs E15.5 * Ctrl
10384398	Grb10	NM_010345	4.50628e-005	-1.43098	E15.5 * Wnt1 down vs E15.5 * Ctrl
10512236	Kif24	NM_024241	0.00110384	-1.43106	E15.5 * Wnt1 down vs E15.5 * Ctrl
10598152	LOC625360	NM_001037925	0.000689647	-1.43116	E15.5 * Wnt1 down vs E15.5 * Ctrl
10593789	Etfa	NM_145615	1.3388e-008	-1.43144	E15.5 * Wnt1 down vs E15.5 * Ctrl
10531189	Adamts3	NM_001081401	0.0071017	-1.43146	E15.5 * Wnt1 down vs E15.5 * Ctrl
10356333	Snord82	NR_002851	0.00339517	-1.43176	E15.5 * Wnt1 down vs E15.5 * Ctrl
10571321	Ppp1r3b	NM_177741	3.57329e-005	-1.4321	E15.5 * Wnt1 down vs E15.5 * Ctrl
10382435	Gprc5c	NM_001110337	0.000167966	-1.43221	E15.5 * Wnt1 down vs E15.5 * Ctrl
10516966	BC013712	NM_001033308	0.00613309	-1.43229	E15.5 * Wnt1 down vs E15.5 * Ctrl
10429573	Ly6c2	NM_001099217	0.00291124	-1.43254	E15.5 * Wnt1 down vs E15.5 * Ctrl
10447141	Eml4	NM_001114361	2.80973e-007	-1.43258	E15.5 * Wnt1 down vs E15.5 * Ctrl
10545583	Pole4	NM_025882	8.75702e-005	-1.43268	E15.5 * Wnt1 down vs E15.5 * Ctrl
10601360	Atp7a	NM_001109757	2.25485e-005	-1.43273	E15.5 * Wnt1 down vs E15.5 * Ctrl
10448506	Ccnf	NM_007634	0.00830679	-1.43286	E15.5 * Wnt1 down vs E15.5 * Ctrl
10416566	Epsti1	NM_029495	1.58855e-005	-1.4331	E15.5 * Wnt1 down vs E15.5 * Ctrl
10559420	Tmc4	NM_181820	4.44149e-006	-1.43325	E15.5 * Wnt1 down vs E15.5 * Ctrl
10409282	Ror2	NM_013846	0.000247007	-1.43359	E15.5 * Wnt1 down vs E15.5 * Ctrl
10591988	Adamts15	NM_001024139	0.00025613	-1.43378	E15.5 * Wnt1 down vs E15.5 * Ctrl
10392177	Icam2	NM_010494	7.63907e-005	-1.43391	E15.5 * Wnt1 down vs E15.5 * Ctrl
10477946	Rpn2	NM_019642	3.47962e-007	-1.43401	E15.5 * Wnt1 down vs E15.5 * Ctrl
10417065	Rap2a	NM_029519	5.90927e-006	-1.43403	E15.5 * Wnt1 down vs E15.5 * Ctrl
10380116	Rnf43	NM_172448	0.000249923	-1.43443	E15.5 * Wnt1 down vs E15.5 * Ctrl
10420877	Esco2	NM_028039	0.000374735	-1.43468	E15.5 * Wnt1 down vs E15.5 * Ctrl
10520650	Cad	NM_023525	0.000455395	-1.43494	E15.5 * Wnt1 down vs E15.5 * Ctrl
10439276	Fam162a	NM_027342	3.64468e-005	-1.43495	E15.5 * Wnt1 down vs E15.5 * Ctrl
10495931		---	0.00228023	-1.43543	E15.5 * Wnt1 down vs E15.5 * Ctrl
10355464	Pecr	NM_023523	0.0106447	-1.43565	E15.5 * Wnt1 down vs E15.5 * Ctrl
10346551	Cflar	NM_207653	0.00245112	-1.43581	E15.5 * Wnt1 down vs E15.5 * Ctrl
10365590	Ccdc53	NM_026070	0.00139354	-1.43618	E15.5 * Wnt1 down vs E15.5 * Ctrl
10444927	Nrm	NM_134122	0.000383373	-1.43625	E15.5 * Wnt1 down vs E15.5 * Ctrl
10572679	Glt25d1	NM_146211	3.78547e-005	-1.43636	E15.5 * Wnt1 down vs E15.5 * Ctrl
10468309	2310014D11Rik	AK009333	9.84807e-005	-1.4365	E15.5 * Wnt1 down vs E15.5 * Ctrl
10498146	Nhlrc3	NM_172501	0.00272377	-1.43665	E15.5 * Wnt1 down vs E15.5 * Ctrl
10470462	Col5a1	NM_015734	9.22587e-006	-1.43675	E15.5 * Wnt1 down vs E15.5 * Ctrl
10473107		---	8.77198e-005	-1.43732	E15.5 * Wnt1 down vs E15.5 * Ctrl
10581395	Slc12a4	NM_009195	0.00178529	-1.43733	E15.5 * Wnt1 down vs E15.5 * Ctrl
10524327	Mn1	NM_001081235	0.000726036	-1.43735	E15.5 * Wnt1 down vs E15.5 * Ctrl
10551393	Akt2	NM_007434	0.00422206	-1.43748	E15.5 * Wnt1 down vs E15.5 * Ctrl
10548585	Csda	NM_139117	0.000120113	-1.43759	E15.5 * Wnt1 down vs E15.5 * Ctrl
10507347	Tesk2	NM_146151	0.000372819	-1.43791	E15.5 * Wnt1 down vs E15.5 * Ctrl
10531179	Adamts3	NM_001081401	0.00343971	-1.43797	E15.5 * Wnt1 down vs E15.5 * Ctrl
10551169	B3gnt8	NM_146184	0.00647106	-1.43803	E15.5 * Wnt1 down vs E15.5 * Ctrl
10389894	Abcc3	NM_029600	0.000831451	-1.43804	E15.5 * Wnt1 down vs E15.5 * Ctrl
10542740	Sspn	NM_010656	0.000937627	-1.43824	E15.5 * Wnt1 down vs E15.5 * Ctrl
10439762	Ahcy	NM_016661	0.00248563	-1.43882	E15.5 * Wnt1 down vs E15.5 * Ctrl
10425905	Nup50	NM_016714	8.2073e-007	-1.43896	E15.5 * Wnt1 down vs E15.5 * Ctrl

10456357	Pmaip1	NM_021451	0.00272784	-1.43916	E15.5 * Wnt1 down vs E15.5 * Ctrl
10452633	Tgif1	NM_009372	0.000159814	-1.43931	E15.5 * Wnt1 down vs E15.5 * Ctrl
10485378	2600010E01Rik	NM_001083810	0.000817972	-1.43952	E15.5 * Wnt1 down vs E15.5 * Ctrl
10498160	Ufm1	NM_026435	0.0127953	-1.4396	E15.5 * Wnt1 down vs E15.5 * Ctrl
10449236	Nme4	NM_019731	0.00163531	-1.44045	E15.5 * Wnt1 down vs E15.5 * Ctrl
10394731	Rock2	ENSMUST00000	0.00434881	-1.44049	E15.5 * Wnt1 down vs E15.5 * Ctrl
10434672	Gng5	NM_010318	0.00102874	-1.44085	E15.5 * Wnt1 down vs E15.5 * Ctrl
10416290	Reep4	NM_180588	0.000314163	-1.44097	E15.5 * Wnt1 down vs E15.5 * Ctrl
10571680	Mlf1ip	NM_027973	0.00252096	-1.44156	E15.5 * Wnt1 down vs E15.5 * Ctrl
10510574	Errfi1	NM_133753	0.0010423	-1.44158	E15.5 * Wnt1 down vs E15.5 * Ctrl
10488860		---	0.00145334	-1.44163	E15.5 * Wnt1 down vs E15.5 * Ctrl
10453178	Map4k3	NM_001081357	3.68584e-006	-1.44165	E15.5 * Wnt1 down vs E15.5 * Ctrl
10523717	Spp1	NM_009263	1.94246e-005	-1.4419	E15.5 * Wnt1 down vs E15.5 * Ctrl
10581813	Mlkl	NM_029005	0.000983096	-1.4428	E15.5 * Wnt1 down vs E15.5 * Ctrl
10502191	Ostc	NM_025509	3.0245e-005	-1.4429	E15.5 * Wnt1 down vs E15.5 * Ctrl
10485948	Grem1	NM_011824	0.00202342	-1.443	E15.5 * Wnt1 down vs E15.5 * Ctrl
10584634	Usp2	NM_198092	2.11436e-005	-1.44338	E15.5 * Wnt1 down vs E15.5 * Ctrl
10453216	Thumpd2	BC065413	0.000196513	-1.44346	E15.5 * Wnt1 down vs E15.5 * Ctrl
10570668	Defa3	NM_007850	0.000578545	-1.44384	E15.5 * Wnt1 down vs E15.5 * Ctrl
10387699	Centb1	NM_153788	3.19568e-005	-1.44401	E15.5 * Wnt1 down vs E15.5 * Ctrl
10351509	Fcgr4	NM_144559	0.000509545	-1.44412	E15.5 * Wnt1 down vs E15.5 * Ctrl
10463410	Fam178a	NM_001081225	0.000901791	-1.44427	E15.5 * Wnt1 down vs E15.5 * Ctrl
10406419	Lysmd3	NM_030257	0.0027363	-1.4446	E15.5 * Wnt1 down vs E15.5 * Ctrl
10538890	LOC641050	M11024	0.00580485	-1.44485	E15.5 * Wnt1 down vs E15.5 * Ctrl
10587534	Bckdhb	NM_199195	0.000395658	-1.44491	E15.5 * Wnt1 down vs E15.5 * Ctrl
10446473	Lama1	NM_008480	8.09404e-006	-1.44521	E15.5 * Wnt1 down vs E15.5 * Ctrl
10404069	Hist1h1a	NM_030609	0.00350688	-1.44535	E15.5 * Wnt1 down vs E15.5 * Ctrl
10451016	Mep1a	NM_008585	5.78598e-005	-1.44552	E15.5 * Wnt1 down vs E15.5 * Ctrl
10530641	Usp46	NM_177561	0.00584181	-1.44556	E15.5 * Wnt1 down vs E15.5 * Ctrl
10479490	Arfgap1	NM_145760	6.11354e-006	-1.44558	E15.5 * Wnt1 down vs E15.5 * Ctrl
10537770	Zyx	NM_011777	0.000312062	-1.44596	E15.5 * Wnt1 down vs E15.5 * Ctrl
10362511	Gstm3	NM_010359	0.00314464	-1.44608	E15.5 * Wnt1 down vs E15.5 * Ctrl
10366707	Avpr1a	NM_016847	0.000561783	-1.44622	E15.5 * Wnt1 down vs E15.5 * Ctrl
10555041	Alg8	NM_199035	0.000114279	-1.44623	E15.5 * Wnt1 down vs E15.5 * Ctrl
10480035	Pfkfb3	NM_133232	8.24665e-006	-1.44659	E15.5 * Wnt1 down vs E15.5 * Ctrl
10380859	Crkrs	BC057057	0.00816785	-1.44678	E15.5 * Wnt1 down vs E15.5 * Ctrl
10600355	Snora70	NR_002899	3.31699e-005	-1.44688	E15.5 * Wnt1 down vs E15.5 * Ctrl
10493490	Dpm3	NM_026767	0.0003021	-1.44706	E15.5 * Wnt1 down vs E15.5 * Ctrl
10504470	Melk	NM_010790	2.4711e-006	-1.4471	E15.5 * Wnt1 down vs E15.5 * Ctrl
10512701	Tomm5	NM_001099675	0.00015252	-1.4474	E15.5 * Wnt1 down vs E15.5 * Ctrl
10539135	Capg	NM_007599	6.03301e-005	-1.44749	E15.5 * Wnt1 down vs E15.5 * Ctrl
10375382	Clint1	NM_001045520	1.82252e-006	-1.44765	E15.5 * Wnt1 down vs E15.5 * Ctrl
10572989	Slc10a7	NM_029736	8.2399e-006	-1.44778	E15.5 * Wnt1 down vs E15.5 * Ctrl
10420011	EG667952	ENSMUST00000	4.28348e-006	-1.44782	E15.5 * Wnt1 down vs E15.5 * Ctrl
10371662	Spic	NM_011461	0.00377588	-1.44798	E15.5 * Wnt1 down vs E15.5 * Ctrl
10385479	Ibtk	NM_001081282	4.91133e-005	-1.44824	E15.5 * Wnt1 down vs E15.5 * Ctrl
10447848	Mas1	NM_008552	0.00131172	-1.44824	E15.5 * Wnt1 down vs E15.5 * Ctrl
10446235	Trip10	NM_134125	4.82325e-005	-1.44827	E15.5 * Wnt1 down vs E15.5 * Ctrl
10371676	Utp20	NM_175158	7.62948e-007	-1.44852	E15.5 * Wnt1 down vs E15.5 * Ctrl
10419323	Dlgap5	NM_144553	9.94961e-006	-1.44853	E15.5 * Wnt1 down vs E15.5 * Ctrl
10488816	Ahcy	NM_016661	0.00107878	-1.44871	E15.5 * Wnt1 down vs E15.5 * Ctrl
10461311	Eml3	NM_144872	7.36209e-005	-1.44877	E15.5 * Wnt1 down vs E15.5 * Ctrl
10565570	4632434I11Rik	NM_001080995	0.00101749	-1.44878	E15.5 * Wnt1 down vs E15.5 * Ctrl
10429140	Ndrg1	NM_008681	0.000612739	-1.44903	E15.5 * Wnt1 down vs E15.5 * Ctrl
10501218	Gstm3	NM_010359	0.00969992	-1.4496	E15.5 * Wnt1 down vs E15.5 * Ctrl
10351551	Adamts4	NM_172845	0.000869467	-1.44967	E15.5 * Wnt1 down vs E15.5 * Ctrl
10436596	2810055G20Rik	ENSMUST00000	0.00223814	-1.44978	E15.5 * Wnt1 down vs E15.5 * Ctrl
10506058	Inadl	NM_172696	1.27366e-005	-1.44983	E15.5 * Wnt1 down vs E15.5 * Ctrl
10462484	A1cf	NM_001081074	0.000356169	-1.44984	E15.5 * Wnt1 down vs E15.5 * Ctrl
10507484	Eri3	NM_080469	0.000838385	-1.4499	E15.5 * Wnt1 down vs E15.5 * Ctrl
10542181	Clec9a	NM_172732	0.00124856	-1.45024	E15.5 * Wnt1 down vs E15.5 * Ctrl
10578952	BC030870	ENSMUST00000	0.00980248	-1.45047	E15.5 * Wnt1 down vs E15.5 * Ctrl
10425053	Ncf4	NM_008677	0.000524167	-1.45076	E15.5 * Wnt1 down vs E15.5 * Ctrl
10542691	Lrmp	NM_008511	0.00010147	-1.45146	E15.5 * Wnt1 down vs E15.5 * Ctrl
10383233	Rnf213	AK173199	0.0129112	-1.45199	E15.5 * Wnt1 down vs E15.5 * Ctrl
10558961	Tspan4	NM_053082	4.498e-006	-1.45248	E15.5 * Wnt1 down vs E15.5 * Ctrl

10469167	Sfmbt2	NM_177386	9.95113e-009	-1.45256	E15.5 * Wnt1 down vs E15.5 * Ctrl
10441620	Fgfr1op	NM_201230	0.00233327	-1.45271	E15.5 * Wnt1 down vs E15.5 * Ctrl
10427280	Hoxc10	NM_010462	0.000503449	-1.45293	E15.5 * Wnt1 down vs E15.5 * Ctrl
10517287	Man1c1	NM_207237	0.00171041	-1.45325	E15.5 * Wnt1 down vs E15.5 * Ctrl
10591556	Spc24	NM_026282	9.30922e-005	-1.45376	E15.5 * Wnt1 down vs E15.5 * Ctrl
10347193	Atic	NM_026195	1.7867e-005	-1.45383	E15.5 * Wnt1 down vs E15.5 * Ctrl
10365991	Epyc	NM_007884	0.000275401	-1.45384	E15.5 * Wnt1 down vs E15.5 * Ctrl
10530163	Rfc1	NM_011258	3.78398e-008	-1.454	E15.5 * Wnt1 down vs E15.5 * Ctrl
10517169	Sh3bgrl3	NM_080559	0.000164378	-1.45424	E15.5 * Wnt1 down vs E15.5 * Ctrl
10569877	1810033B17Rik	NM_026985	0.000250609	-1.45439	E15.5 * Wnt1 down vs E15.5 * Ctrl
10451993	D17Wsu104e	NM_080837	0.00739579	-1.45487	E15.5 * Wnt1 down vs E15.5 * Ctrl
10424221	Wdr67	NM_001081396	0.000116052	-1.45512	E15.5 * Wnt1 down vs E15.5 * Ctrl
10568714	Mki67	NM_001081117	1.91185e-006	-1.45527	E15.5 * Wnt1 down vs E15.5 * Ctrl
10441003	Runx1	NM_001111023	0.00180262	-1.45531	E15.5 * Wnt1 down vs E15.5 * Ctrl
10435704	Cd80	NM_009855	0.00152613	-1.45536	E15.5 * Wnt1 down vs E15.5 * Ctrl
10512999	AI427809	NM_001033454	3.3431e-006	-1.45564	E15.5 * Wnt1 down vs E15.5 * Ctrl
10379727	OTTMUSG00000	NM_001081957	0.000111622	-1.45569	E15.5 * Wnt1 down vs E15.5 * Ctrl
10422022	ENSMUSG00000	ENSMUST00000	0.0104477	-1.4558	E15.5 * Wnt1 down vs E15.5 * Ctrl
10587503	Sh3bgrl2	NM_172507	0.00061031	-1.4559	E15.5 * Wnt1 down vs E15.5 * Ctrl
10529689	Wdr1	NM_011715	0.000126928	-1.45625	E15.5 * Wnt1 down vs E15.5 * Ctrl
10416057	Clu	NM_013492	0.000317134	-1.45632	E15.5 * Wnt1 down vs E15.5 * Ctrl
10403948	Hist1h2be	NM_178194	0.012457	-1.45684	E15.5 * Wnt1 down vs E15.5 * Ctrl
10510047	ENSMUSG00000	NR_027137	0.00225822	-1.45692	E15.5 * Wnt1 down vs E15.5 * Ctrl
10590918	Amotl1	NM_001081395	0.00266983	-1.45737	E15.5 * Wnt1 down vs E15.5 * Ctrl
10396383	Slc38a6	BC157917	1.09131e-005	-1.45758	E15.5 * Wnt1 down vs E15.5 * Ctrl
10556005	Ilk	NM_010562	3.02285e-006	-1.45775	E15.5 * Wnt1 down vs E15.5 * Ctrl
10412559	Slbp	NM_009193	3.1295e-005	-1.45784	E15.5 * Wnt1 down vs E15.5 * Ctrl
10589703	Ltf	NM_008522	0.000124052	-1.45785	E15.5 * Wnt1 down vs E15.5 * Ctrl
10450116	Slc39a7	NM_008202	2.70336e-005	-1.4579	E15.5 * Wnt1 down vs E15.5 * Ctrl
10389775	Pctp	NM_008796	0.00133598	-1.45801	E15.5 * Wnt1 down vs E15.5 * Ctrl
10361186	Sertad4	NM_198247	1.15345e-005	-1.45805	E15.5 * Wnt1 down vs E15.5 * Ctrl
10602180	Tmem164	NM_177592	0.000294297	-1.45861	E15.5 * Wnt1 down vs E15.5 * Ctrl
10409978	EG630579	NM_001039239	0.0014379	-1.45897	E15.5 * Wnt1 down vs E15.5 * Ctrl
10507671	Guca2a	NM_008190	0.00145692	-1.45921	E15.5 * Wnt1 down vs E15.5 * Ctrl
10355806	Tuba4a	NM_009447	0.000246786	-1.45951	E15.5 * Wnt1 down vs E15.5 * Ctrl
10576661	Itgb1	NM_010578	1.23536e-007	-1.45952	E15.5 * Wnt1 down vs E15.5 * Ctrl
10407053	2810008M24Rik	BC004049	2.92777e-005	-1.45961	E15.5 * Wnt1 down vs E15.5 * Ctrl
10371907	Nedd1	NM_008682	0.000230231	-1.45989	E15.5 * Wnt1 down vs E15.5 * Ctrl
10354111	Aff3	NM_010678	4.65519e-005	-1.46011	E15.5 * Wnt1 down vs E15.5 * Ctrl
10427286	Hoxc9	NM_008272	0.000152766	-1.46028	E15.5 * Wnt1 down vs E15.5 * Ctrl
10398372		---	0.00544446	-1.46049	E15.5 * Wnt1 down vs E15.5 * Ctrl
10398376		---	0.00544446	-1.46049	E15.5 * Wnt1 down vs E15.5 * Ctrl
10374727	Bcl11a	NM_016707	0.000187848	-1.46051	E15.5 * Wnt1 down vs E15.5 * Ctrl
10379953	4632419I22Rik	BC067002	3.85166e-006	-1.4608	E15.5 * Wnt1 down vs E15.5 * Ctrl
10575976	Crispld2	NM_030209	0.000386161	-1.4609	E15.5 * Wnt1 down vs E15.5 * Ctrl
10451805	Sgol1	NM_028232	6.84893e-005	-1.46167	E15.5 * Wnt1 down vs E15.5 * Ctrl
10384423	Cobl	NM_172496	9.82276e-005	-1.46169	E15.5 * Wnt1 down vs E15.5 * Ctrl
10434441	Ece2	NM_025462	0.000358498	-1.46188	E15.5 * Wnt1 down vs E15.5 * Ctrl
10440469	EG547311	XR_031351	0.000197901	-1.46207	E15.5 * Wnt1 down vs E15.5 * Ctrl
10435907	Cd200r1	NM_021325	2.95288e-006	-1.46256	E15.5 * Wnt1 down vs E15.5 * Ctrl
10449467	Clps	NM_025469	0.0143862	-1.46285	E15.5 * Wnt1 down vs E15.5 * Ctrl
10504329	RP23-195K8.6	NM_001037913	3.31556e-006	-1.46338	E15.5 * Wnt1 down vs E15.5 * Ctrl
10494023	Rorc	NM_011281	0.000418898	-1.46339	E15.5 * Wnt1 down vs E15.5 * Ctrl
10479294		---	0.00240579	-1.46365	E15.5 * Wnt1 down vs E15.5 * Ctrl
10447461	Ston1	NM_029858	0.000377247	-1.46395	E15.5 * Wnt1 down vs E15.5 * Ctrl
10605811	Fam123b	NM_175179	7.13879e-006	-1.46401	E15.5 * Wnt1 down vs E15.5 * Ctrl
10592001	St14	NM_011176	5.8059e-005	-1.46416	E15.5 * Wnt1 down vs E15.5 * Ctrl
10447130	Pkdcc	NM_134117	4.73115e-005	-1.46442	E15.5 * Wnt1 down vs E15.5 * Ctrl
10474769	Bub1b	NM_009773	3.06124e-005	-1.46511	E15.5 * Wnt1 down vs E15.5 * Ctrl
10561702	Kcnk6	NM_001033525	9.51073e-006	-1.46514	E15.5 * Wnt1 down vs E15.5 * Ctrl
10380403	Lrrc59	NM_133807	0.00216161	-1.46519	E15.5 * Wnt1 down vs E15.5 * Ctrl
10607169	Trpc5	NM_009428	0.00032261	-1.46539	E15.5 * Wnt1 down vs E15.5 * Ctrl
10509204	Tcea3	NM_011542	8.71179e-006	-1.46594	E15.5 * Wnt1 down vs E15.5 * Ctrl
10563883	Depdc1a	NM_029523	0.00344366	-1.4662	E15.5 * Wnt1 down vs E15.5 * Ctrl
10514221	Adfp	NM_007408	1.92767e-005	-1.46662	E15.5 * Wnt1 down vs E15.5 * Ctrl
10510201	Rex2	NM_009051	2.13425e-005	-1.46674	E15.5 * Wnt1 down vs E15.5 * Ctrl

10546417	Trh	NM_009426	1.09143e-005	-1.46679	E15.5 * Wnt1 down vs E15.5 * Ctrl
10506397	Mier1	NM_027696	7.65477e-005	-1.46719	E15.5 * Wnt1 down vs E15.5 * Ctrl
10606235	Zdhhc15	NM_175358	0.000267061	-1.46722	E15.5 * Wnt1 down vs E15.5 * Ctrl
10444229	H2-DMa	NM_010386	0.00243199	-1.46722	E15.5 * Wnt1 down vs E15.5 * Ctrl
10408709	Ssr1	NM_025965	1.7781e-007	-1.46736	E15.5 * Wnt1 down vs E15.5 * Ctrl
10587988	Gk5	NM_177352	0.0047582	-1.46792	E15.5 * Wnt1 down vs E15.5 * Ctrl
10460738	Cdca5	NM_026410	0.0053826	-1.46815	E15.5 * Wnt1 down vs E15.5 * Ctrl
10405733	EG630579	NM_001039239	0.00203244	-1.46862	E15.5 * Wnt1 down vs E15.5 * Ctrl
10497122	Depdc1a	NM_029523	0.00489845	-1.46869	E15.5 * Wnt1 down vs E15.5 * Ctrl
10397895	Ubr7	NM_025666	0.0018353	-1.46899	E15.5 * Wnt1 down vs E15.5 * Ctrl
10346365	Sgol2	NM_199007	0.000650394	-1.46948	E15.5 * Wnt1 down vs E15.5 * Ctrl
10393449	Socs3	NM_007707	0.000583497	-1.46993	E15.5 * Wnt1 down vs E15.5 * Ctrl
10588786	Ube1l	NM_023738	7.23184e-005	-1.4703	E15.5 * Wnt1 down vs E15.5 * Ctrl
10480432	Mastl	NM_025979	0.000962193	-1.4706	E15.5 * Wnt1 down vs E15.5 * Ctrl
10372877	Xpot	NM_001081056	1.13108e-005	-1.47075	E15.5 * Wnt1 down vs E15.5 * Ctrl
10473809	Sfpi1	NM_011355	4.14921e-006	-1.47088	E15.5 * Wnt1 down vs E15.5 * Ctrl
10525591	Kntc1	NM_001042421	4.81669e-008	-1.47097	E15.5 * Wnt1 down vs E15.5 * Ctrl
10390850	Krt20	NM_023256	6.2901e-005	-1.47109	E15.5 * Wnt1 down vs E15.5 * Ctrl
10569358	Ins2	NM_008387	9.70272e-005	-1.47116	E15.5 * Wnt1 down vs E15.5 * Ctrl
10510167	OTTMUSG00000	NM_001037926	0.000288266	-1.4712	E15.5 * Wnt1 down vs E15.5 * Ctrl
10584325	Vsig2	NM_020518	5.23378e-005	-1.4714	E15.5 * Wnt1 down vs E15.5 * Ctrl
10369276	Ccdc109a	NM_001033259	0.00284282	-1.4716	E15.5 * Wnt1 down vs E15.5 * Ctrl
10489391	Ada	NM_007398	0.00196731	-1.47176	E15.5 * Wnt1 down vs E15.5 * Ctrl
10383200		---	0.00112241	-1.47177	E15.5 * Wnt1 down vs E15.5 * Ctrl
10568363	Armcx3	NM_027870	7.23604e-007	-1.47177	E15.5 * Wnt1 down vs E15.5 * Ctrl
10506188	Pgm2	NM_028132	0.000295492	-1.47247	E15.5 * Wnt1 down vs E15.5 * Ctrl
10429387	Ptplb	NM_023587	4.74436e-005	-1.47253	E15.5 * Wnt1 down vs E15.5 * Ctrl
10430993	1700001L05Rik	ENSMUST00000	0.00221706	-1.47256	E15.5 * Wnt1 down vs E15.5 * Ctrl
10361246	G0s2	NM_008059	2.46245e-005	-1.4726	E15.5 * Wnt1 down vs E15.5 * Ctrl
10554059	ENSMUSG00000	ENSMUST00000	0.00255542	-1.47274	E15.5 * Wnt1 down vs E15.5 * Ctrl
10593878	Snx33	NM_175483	0.00170765	-1.47401	E15.5 * Wnt1 down vs E15.5 * Ctrl
10406270	Glrx	NM_053108	0.00013372	-1.47417	E15.5 * Wnt1 down vs E15.5 * Ctrl
10589350	Shisa5	NM_025858	2.5512e-007	-1.47447	E15.5 * Wnt1 down vs E15.5 * Ctrl
10592342	Tbrg1	NM_025289	3.67666e-006	-1.47451	E15.5 * Wnt1 down vs E15.5 * Ctrl
10435897	BC027231	BC027231	4.73181e-005	-1.47451	E15.5 * Wnt1 down vs E15.5 * Ctrl
10474936	Spint1	NM_016907	0.000386344	-1.47452	E15.5 * Wnt1 down vs E15.5 * Ctrl
10595205	2410127L17Rik	NM_026120	8.77646e-005	-1.47462	E15.5 * Wnt1 down vs E15.5 * Ctrl
10595480	Me1	NM_008615	0.000424129	-1.4747	E15.5 * Wnt1 down vs E15.5 * Ctrl
10598757	Gpr82	NM_175669	0.00724218	-1.47485	E15.5 * Wnt1 down vs E15.5 * Ctrl
10527801	Brca2	NM_001081001	0.000196837	-1.47492	E15.5 * Wnt1 down vs E15.5 * Ctrl
10598603			3.20717e-005	-1.47513	E15.5 * Wnt1 down vs E15.5 * Ctrl
10481259	OTTMUSG00000	AK158259	0.00134134	-1.47536	E15.5 * Wnt1 down vs E15.5 * Ctrl
10398751	Zfyve21	NM_026752	4.20279e-006	-1.47547	E15.5 * Wnt1 down vs E15.5 * Ctrl
10383088	Gaa	NM_008064	4.80761e-005	-1.4757	E15.5 * Wnt1 down vs E15.5 * Ctrl
10484276	Neurod1	NM_010894	0.00130208	-1.4761	E15.5 * Wnt1 down vs E15.5 * Ctrl
10420957	Ptk2b	NM_172498	0.000776722	-1.47748	E15.5 * Wnt1 down vs E15.5 * Ctrl
10499366	Pmf1	NM_025928	0.00170793	-1.4776	E15.5 * Wnt1 down vs E15.5 * Ctrl
10489377	Serinc3	NM_012032	6.26012e-009	-1.47829	E15.5 * Wnt1 down vs E15.5 * Ctrl
10539861	Rpn1	NM_133933	5.56713e-007	-1.47846	E15.5 * Wnt1 down vs E15.5 * Ctrl
10403943	Hist1h2bm	NM_178200	0.00857323	-1.47863	E15.5 * Wnt1 down vs E15.5 * Ctrl
10375515	Ifi47	NM_008330	2.67788e-008	-1.47897	E15.5 * Wnt1 down vs E15.5 * Ctrl
10396652	Hspa2	NM_001002012	0.000531341	-1.47898	E15.5 * Wnt1 down vs E15.5 * Ctrl
10344713	Ahcy	NM_016661	0.00670427	-1.47903	E15.5 * Wnt1 down vs E15.5 * Ctrl
10435841	Ccdc52	NM_144550	0.000196076	-1.47931	E15.5 * Wnt1 down vs E15.5 * Ctrl
10570690	Defa3	NM_007850	0.000402682	-1.47954	E15.5 * Wnt1 down vs E15.5 * Ctrl
10570729	Defa3	NM_007850	0.000402682	-1.47954	E15.5 * Wnt1 down vs E15.5 * Ctrl
10503315	E130016E03Rik	NM_001039556	4.30248e-005	-1.47971	E15.5 * Wnt1 down vs E15.5 * Ctrl
10503448	Mmp16	NM_019724	1.05997e-006	-1.47987	E15.5 * Wnt1 down vs E15.5 * Ctrl
10530156	Tmem156	ENSMUST00000	5.22975e-005	-1.48012	E15.5 * Wnt1 down vs E15.5 * Ctrl
10451851	Armcx3	NM_027870	1.76426e-005	-1.48018	E15.5 * Wnt1 down vs E15.5 * Ctrl
10548829	Gucy2c	NM_001127318	2.03616e-005	-1.48028	E15.5 * Wnt1 down vs E15.5 * Ctrl
10496864	EG381483	ENSMUST00000	0.000622217	-1.48037	E15.5 * Wnt1 down vs E15.5 * Ctrl
10405727	2410127L17Rik	NM_026120	0.000313044	-1.48039	E15.5 * Wnt1 down vs E15.5 * Ctrl
10412481	2410127L17Rik	NM_026120	0.000313044	-1.48039	E15.5 * Wnt1 down vs E15.5 * Ctrl
10529824	Prom1	NM_008935	3.98946e-006	-1.48061	E15.5 * Wnt1 down vs E15.5 * Ctrl
10530151	Tlr6	NM_011604	1.94652e-006	-1.48067	E15.5 * Wnt1 down vs E15.5 * Ctrl

10591131	Fat3	NM_001080814	0.00501192	-1.48075	E15.5 * Wnt1 down vs E15.5 * Ctrl
10545130	Gadd45a	NM_007836	0.000285499	-1.48086	E15.5 * Wnt1 down vs E15.5 * Ctrl
10601456	EG622976	NM_001037917	0.00620669	-1.48099	E15.5 * Wnt1 down vs E15.5 * Ctrl
10415742	Mipep	NM_027436	4.04196e-005	-1.48115	E15.5 * Wnt1 down vs E15.5 * Ctrl
10503264	Ccne2	NM_009830	0.0025552	-1.48121	E15.5 * Wnt1 down vs E15.5 * Ctrl
10436392	Cpox	NM_007757	0.000646061	-1.48134	E15.5 * Wnt1 down vs E15.5 * Ctrl
10353750	Bag2	NM_145392	0.00282413	-1.48162	E15.5 * Wnt1 down vs E15.5 * Ctrl
10368527	Hint3	NM_025798	0.00130468	-1.48205	E15.5 * Wnt1 down vs E15.5 * Ctrl
10591576	Dock6	NM_177030	0.000142806	-1.48211	E15.5 * Wnt1 down vs E15.5 * Ctrl
10547217	Csgalnact2	NM_030165	0.000313821	-1.48214	E15.5 * Wnt1 down vs E15.5 * Ctrl
10523923	Ccdc18	NM_028481	0.00141642	-1.48232	E15.5 * Wnt1 down vs E15.5 * Ctrl
10509063	Il22ra1	NM_178257	0.000660638	-1.48241	E15.5 * Wnt1 down vs E15.5 * Ctrl
10375472	Timd4	NM_178759	4.79799e-006	-1.4831	E15.5 * Wnt1 down vs E15.5 * Ctrl
10477250	Hck	NM_010407	4.37734e-006	-1.48318	E15.5 * Wnt1 down vs E15.5 * Ctrl
10548128	Tspan9	NM_175414	5.50304e-005	-1.48332	E15.5 * Wnt1 down vs E15.5 * Ctrl
10506680	Tmem48	NM_028355	4.71182e-006	-1.48359	E15.5 * Wnt1 down vs E15.5 * Ctrl
10588243	Ryk	NM_013649	1.70399e-006	-1.48365	E15.5 * Wnt1 down vs E15.5 * Ctrl
10470959	Phyhd1	NM_172267	0.000622465	-1.48388	E15.5 * Wnt1 down vs E15.5 * Ctrl
10419736	Dad1	NM_010015	0.00197207	-1.48403	E15.5 * Wnt1 down vs E15.5 * Ctrl
10367734	Ust	NM_177387	0.000220789	-1.4841	E15.5 * Wnt1 down vs E15.5 * Ctrl
10588294	Topbp1	NM_176979	4.5453e-008	-1.48438	E15.5 * Wnt1 down vs E15.5 * Ctrl
10461921	2410127L17Rik	NM_026120	0.000136143	-1.48468	E15.5 * Wnt1 down vs E15.5 * Ctrl
10405488	Tmed9	NM_026211	0.00385437	-1.48479	E15.5 * Wnt1 down vs E15.5 * Ctrl
10605357	Gab3	NM_181584	0.000417466	-1.48483	E15.5 * Wnt1 down vs E15.5 * Ctrl
10399465	Fam84a	NM_029007	1.96242e-005	-1.48512	E15.5 * Wnt1 down vs E15.5 * Ctrl
10500529	Phgdh	NM_016966	0.00289268	-1.48516	E15.5 * Wnt1 down vs E15.5 * Ctrl
10490047	AY702102	AY702102	0.00319444	-1.4855	E15.5 * Wnt1 down vs E15.5 * Ctrl
10606436	Nsbp1	NM_016710	0.000169498	-1.48575	E15.5 * Wnt1 down vs E15.5 * Ctrl
10499062	Fhdc1	NM_001033301	0.00679386	-1.48594	E15.5 * Wnt1 down vs E15.5 * Ctrl
10421186	ENSMUSG0000C	ENSMUST00000(	0.010457	-1.48598	E15.5 * Wnt1 down vs E15.5 * Ctrl
10544788	Hoxa9	NM_010456	0.000684861	-1.486	E15.5 * Wnt1 down vs E15.5 * Ctrl
10493203	0610031J06Rik	NM_020003	1.79186e-005	-1.48649	E15.5 * Wnt1 down vs E15.5 * Ctrl
10580795	Ccdc102a	NM_001033533	7.93758e-005	-1.48665	E15.5 * Wnt1 down vs E15.5 * Ctrl
10367436	Cd63	NM_001042580	1.32846e-005	-1.48676	E15.5 * Wnt1 down vs E15.5 * Ctrl
10515007	Gpx7	NM_024198	0.000124923	-1.48688	E15.5 * Wnt1 down vs E15.5 * Ctrl
10532517	Cryba4	NM_021351	0.00125075	-1.48745	E15.5 * Wnt1 down vs E15.5 * Ctrl
10568988	Bet1l	NM_018742	0.000885833	-1.48754	E15.5 * Wnt1 down vs E15.5 * Ctrl
10531185	Adamts3	NM_001081401	8.46065e-005	-1.48758	E15.5 * Wnt1 down vs E15.5 * Ctrl
10349724	Rab7l1	NM_144875	7.86264e-005	-1.48758	E15.5 * Wnt1 down vs E15.5 * Ctrl
10515986	Zfp69	NM_001005788	0.0139489	-1.48772	E15.5 * Wnt1 down vs E15.5 * Ctrl
10597758	Csrnp1	NM_153287	1.66642e-006	-1.48829	E15.5 * Wnt1 down vs E15.5 * Ctrl
10430302	Csf2rb2	NM_007781	0.00166505	-1.48842	E15.5 * Wnt1 down vs E15.5 * Ctrl
10462973	Hells	NM_008234	7.3873e-005	-1.48859	E15.5 * Wnt1 down vs E15.5 * Ctrl
10602772	Rps6ka3	NM_148945	3.72267e-005	-1.48863	E15.5 * Wnt1 down vs E15.5 * Ctrl
10584600		---	0.00595076	-1.4891	E15.5 * Wnt1 down vs E15.5 * Ctrl
10558159	Dmbt1	NM_007769	0.00626259	-1.48969	E15.5 * Wnt1 down vs E15.5 * Ctrl
10410164	Cntnap3	NM_001081129	1.80027e-005	-1.48993	E15.5 * Wnt1 down vs E15.5 * Ctrl
10348739	Sned1	NM_172463	0.000175587	-1.49003	E15.5 * Wnt1 down vs E15.5 * Ctrl
10554521	Pde8a	NM_008803	0.00044633	-1.49015	E15.5 * Wnt1 down vs E15.5 * Ctrl
10568536	Cpxm2	NM_018867	0.000490672	-1.49087	E15.5 * Wnt1 down vs E15.5 * Ctrl
10366994	Myo1a	NM_001081219	3.28662e-005	-1.49098	E15.5 * Wnt1 down vs E15.5 * Ctrl
10513008	Klf4	NM_010637	0.000797334	-1.49121	E15.5 * Wnt1 down vs E15.5 * Ctrl
10515396	9530048O09Rik	NR_024078	0.00192712	-1.49135	E15.5 * Wnt1 down vs E15.5 * Ctrl
10522589	Srd5a3	NM_020611	0.00103752	-1.49137	E15.5 * Wnt1 down vs E15.5 * Ctrl
10586306	Igdcc4	NM_020043	2.85032e-005	-1.49144	E15.5 * Wnt1 down vs E15.5 * Ctrl
10463296	Cutc	NM_001113562	0.000148375	-1.49157	E15.5 * Wnt1 down vs E15.5 * Ctrl
10508405	Tmem54	NM_025452	0.00487653	-1.49188	E15.5 * Wnt1 down vs E15.5 * Ctrl
10584710	H2afx	NM_010436	4.98908e-005	-1.49323	E15.5 * Wnt1 down vs E15.5 * Ctrl
10496580	Gbp3	NM_018734	8.65717e-006	-1.49514	E15.5 * Wnt1 down vs E15.5 * Ctrl
10534202	Ncf1	NM_010876	2.42398e-005	-1.49597	E15.5 * Wnt1 down vs E15.5 * Ctrl
10442098	Fpr3	NM_008042	0.000127581	-1.49615	E15.5 * Wnt1 down vs E15.5 * Ctrl
10460108	Gnpnat1	NM_019425	7.50659e-005	-1.49645	E15.5 * Wnt1 down vs E15.5 * Ctrl
10603573	Sytl5	NM_177704	0.000200442	-1.4965	E15.5 * Wnt1 down vs E15.5 * Ctrl
10591739	Acp5	NM_001102404	0.000676323	-1.49654	E15.5 * Wnt1 down vs E15.5 * Ctrl
10507885	Mycbp	NM_019660	0.00375848	-1.49743	E15.5 * Wnt1 down vs E15.5 * Ctrl
10359890	Nuf2	NM_023284	0.000421291	-1.49752	E15.5 * Wnt1 down vs E15.5 * Ctrl

10480347	Pip4k2a	NM_008845	4.57449e-005	-1.49782	E15.5 * Wnt1 down vs E15.5 * Ctrl
10527963		---	0.000350419	-1.49787	E15.5 * Wnt1 down vs E15.5 * Ctrl
10499529	Rag1ap1	NM_009057	5.53762e-005	-1.49803	E15.5 * Wnt1 down vs E15.5 * Ctrl
10556442	Tead1	NM_009346	7.3768e-005	-1.49903	E15.5 * Wnt1 down vs E15.5 * Ctrl
10433032	Atf7	ENSMUST00000(	0.00898544	-1.49904	E15.5 * Wnt1 down vs E15.5 * Ctrl
10597978	Fyco1	NM_148925	5.60583e-007	-1.49934	E15.5 * Wnt1 down vs E15.5 * Ctrl
10523802	Cdc7	NM_009863	0.000960077	-1.49943	E15.5 * Wnt1 down vs E15.5 * Ctrl
10415411	BC030046	BC151043	0.0132824	-1.49951	E15.5 * Wnt1 down vs E15.5 * Ctrl
10546113	Sec61a1	NM_016906	1.15173e-007	-1.4998	E15.5 * Wnt1 down vs E15.5 * Ctrl
10416155	Kctd9	NM_001111028	0.00186024	-1.5005	E15.5 * Wnt1 down vs E15.5 * Ctrl
10571657	Acsl1	NM_007981	2.50179e-005	-1.50067	E15.5 * Wnt1 down vs E15.5 * Ctrl
10383214	Rnf213	AK173199	9.21521e-006	-1.50069	E15.5 * Wnt1 down vs E15.5 * Ctrl
10504753	LOC641050	M11024	0.00274498	-1.50071	E15.5 * Wnt1 down vs E15.5 * Ctrl
10557591	Itgal	NM_008400	0.000223644	-1.50121	E15.5 * Wnt1 down vs E15.5 * Ctrl
10496771	Mcoln2	NM_026656	0.00330925	-1.50162	E15.5 * Wnt1 down vs E15.5 * Ctrl
10426098	Creld2	NM_029720	1.0918e-007	-1.50176	E15.5 * Wnt1 down vs E15.5 * Ctrl
10394770	Odc1	NM_013614	5.81103e-007	-1.50187	E15.5 * Wnt1 down vs E15.5 * Ctrl
10525439	P2rx4	NM_011026	0.00221347	-1.50194	E15.5 * Wnt1 down vs E15.5 * Ctrl
10386211	3100002J23Rik	ENSMUST00000(	0.0140996	-1.50205	E15.5 * Wnt1 down vs E15.5 * Ctrl
10504775	Col15a1	NM_009928	1.08336e-005	-1.50238	E15.5 * Wnt1 down vs E15.5 * Ctrl
10539263	Loxl3	NM_013586	1.99094e-005	-1.50276	E15.5 * Wnt1 down vs E15.5 * Ctrl
10576639	Nrp1	NM_008737	3.45812e-005	-1.50304	E15.5 * Wnt1 down vs E15.5 * Ctrl
10361787		---	0.00165474	-1.50336	E15.5 * Wnt1 down vs E15.5 * Ctrl
10489051	5730471H19Rik	AK133873	0.00041732	-1.50339	E15.5 * Wnt1 down vs E15.5 * Ctrl
10417950	Ndst2	NM_010811	1.13516e-005	-1.50398	E15.5 * Wnt1 down vs E15.5 * Ctrl
10436369	Filip1l	NM_030163	1.71771e-007	-1.50424	E15.5 * Wnt1 down vs E15.5 * Ctrl
10409994	EG435366	ENSMUST00000(	0.00331439	-1.50447	E15.5 * Wnt1 down vs E15.5 * Ctrl
10425066	Csf2rb	NM_007780	0.00015008	-1.50485	E15.5 * Wnt1 down vs E15.5 * Ctrl
10474902	Rad51	NM_011234	4.81317e-005	-1.50511	E15.5 * Wnt1 down vs E15.5 * Ctrl
10556381	Mical2	NM_177282	7.76868e-006	-1.50511	E15.5 * Wnt1 down vs E15.5 * Ctrl
10413152	1700112E06Rik	NM_028275	0.000923741	-1.50545	E15.5 * Wnt1 down vs E15.5 * Ctrl
10551614	Ech1	NM_016772	1.42509e-005	-1.50563	E15.5 * Wnt1 down vs E15.5 * Ctrl
10581175	B3gnt9	NM_178879	5.05742e-005	-1.5058	E15.5 * Wnt1 down vs E15.5 * Ctrl
10444830	H2-Q7	NM_010394	8.50299e-006	-1.50613	E15.5 * Wnt1 down vs E15.5 * Ctrl
10461093	Pla2g16	NM_139269	0.00155896	-1.50633	E15.5 * Wnt1 down vs E15.5 * Ctrl
10396877	Galntl1	NM_001081421	5.54162e-006	-1.50657	E15.5 * Wnt1 down vs E15.5 * Ctrl
10501734	Palmd	NM_023245	3.99562e-005	-1.50698	E15.5 * Wnt1 down vs E15.5 * Ctrl
10443009	Ergic1	NM_026170	6.73553e-005	-1.50699	E15.5 * Wnt1 down vs E15.5 * Ctrl
10510391	Srm	NM_009272	5.30396e-005	-1.50761	E15.5 * Wnt1 down vs E15.5 * Ctrl
10494548	Gja5	NM_008121	0.0012507	-1.50772	E15.5 * Wnt1 down vs E15.5 * Ctrl
10559818		---	0.00295206	-1.50779	E15.5 * Wnt1 down vs E15.5 * Ctrl
10526120	Tpst1	NM_001130476	6.92485e-008	-1.508	E15.5 * Wnt1 down vs E15.5 * Ctrl
10458875	Dtwd2	NM_026854	0.00775019	-1.50816	E15.5 * Wnt1 down vs E15.5 * Ctrl
10527934		---	0.000496931	-1.50848	E15.5 * Wnt1 down vs E15.5 * Ctrl
10584047	Adamts8	NM_013906	0.00127829	-1.50849	E15.5 * Wnt1 down vs E15.5 * Ctrl
10558441	Mgmt	NM_008598	2.32979e-005	-1.50896	E15.5 * Wnt1 down vs E15.5 * Ctrl
10346000	Gulp1	NM_028450	0.000639947	-1.50907	E15.5 * Wnt1 down vs E15.5 * Ctrl
10351197	Sell	NM_011346	2.99621e-006	-1.50936	E15.5 * Wnt1 down vs E15.5 * Ctrl
10495359	Clcc1	NM_145543	1.02062e-005	-1.50961	E15.5 * Wnt1 down vs E15.5 * Ctrl
10607924	Amelx	NM_001081978	0.000107423	-1.51098	E15.5 * Wnt1 down vs E15.5 * Ctrl
10551347	Blvrb	NM_144923	1.04283e-005	-1.51121	E15.5 * Wnt1 down vs E15.5 * Ctrl
10372796	Hmga2	NM_010441	3.30831e-005	-1.51122	E15.5 * Wnt1 down vs E15.5 * Ctrl
10366004	Atp2b1	NM_026482	8.38312e-011	-1.51124	E15.5 * Wnt1 down vs E15.5 * Ctrl
10507112	Stil	NM_009185	0.000379244	-1.51133	E15.5 * Wnt1 down vs E15.5 * Ctrl
10550574	Dmpk	NM_032418	1.35493e-005	-1.51137	E15.5 * Wnt1 down vs E15.5 * Ctrl
10554013	Chsy1	NM_001081163	1.7398e-005	-1.51156	E15.5 * Wnt1 down vs E15.5 * Ctrl
10601705	Cenpi	NM_145924	1.71657e-005	-1.51158	E15.5 * Wnt1 down vs E15.5 * Ctrl
10361282	Cr1l	NM_013499	0.0011496	-1.51179	E15.5 * Wnt1 down vs E15.5 * Ctrl
10592217	Stt3a	NM_008408	3.49828e-006	-1.51189	E15.5 * Wnt1 down vs E15.5 * Ctrl
10347928	Sp110	NM_175397	0.000128291	-1.51257	E15.5 * Wnt1 down vs E15.5 * Ctrl
10582874	Sp110	NM_175397	0.000128291	-1.51257	E15.5 * Wnt1 down vs E15.5 * Ctrl
10425114	Tmed2	NM_019770	7.0021e-005	-1.51287	E15.5 * Wnt1 down vs E15.5 * Ctrl
10448186	Zfp820	NM_029281	0.000620402	-1.51331	E15.5 * Wnt1 down vs E15.5 * Ctrl
10355514	Tns1	NM_027884	5.3987e-005	-1.51343	E15.5 * Wnt1 down vs E15.5 * Ctrl
10360848	Mosc2	NM_133684	6.87265e-008	-1.51403	E15.5 * Wnt1 down vs E15.5 * Ctrl
10531415	Cxcl10	NM_021274	0.000978261	-1.5142	E15.5 * Wnt1 down vs E15.5 * Ctrl

10396068	Ppil5	NM_001081406	0.00292805	-1.5143	E15.5 * Wnt1 down vs E15.5 * Ctrl
10537014	Cpa2	NM_001024698	0.0036842	-1.51465	E15.5 * Wnt1 down vs E15.5 * Ctrl
10530516	Txk	NM_001122754	0.00389951	-1.51502	E15.5 * Wnt1 down vs E15.5 * Ctrl
10375578	Flt4	NM_008029	6.15874e-005	-1.51506	E15.5 * Wnt1 down vs E15.5 * Ctrl
10403980	RP23-38E20.1	NM_001097979	0.0102718	-1.51508	E15.5 * Wnt1 down vs E15.5 * Ctrl
10437970	2310008H04Rik	NM_146068	9.40545e-008	-1.51518	E15.5 * Wnt1 down vs E15.5 * Ctrl
10451677		---	0.000864874	-1.51538	E15.5 * Wnt1 down vs E15.5 * Ctrl
10523756		---	0.000864874	-1.51538	E15.5 * Wnt1 down vs E15.5 * Ctrl
10537504		---	0.000864874	-1.51538	E15.5 * Wnt1 down vs E15.5 * Ctrl
10356876		---	0.00570455	-1.51551	E15.5 * Wnt1 down vs E15.5 * Ctrl
10559454	Pira2	NM_011089	0.000177664	-1.51553	E15.5 * Wnt1 down vs E15.5 * Ctrl
10479981	Gata3	NM_008091	0.0115764	-1.51556	E15.5 * Wnt1 down vs E15.5 * Ctrl
10354677	Ankrd44	NM_001081433	0.000266288	-1.51585	E15.5 * Wnt1 down vs E15.5 * Ctrl
10591622	Dock6	NM_177030	0.000709717	-1.5164	E15.5 * Wnt1 down vs E15.5 * Ctrl
10419082	5730469M10Rik	BC056635	9.33328e-005	-1.51648	E15.5 * Wnt1 down vs E15.5 * Ctrl
10562563	Ccne1	NM_007633	0.000420825	-1.51681	E15.5 * Wnt1 down vs E15.5 * Ctrl
10361381	Syne1	NM_001079686	2.23085e-007	-1.51743	E15.5 * Wnt1 down vs E15.5 * Ctrl
10372121	Tmtc3	NM_001033332	7.42245e-006	-1.51783	E15.5 * Wnt1 down vs E15.5 * Ctrl
10401852	4930534B04Rik	NM_181815	0.000442165	-1.5181	E15.5 * Wnt1 down vs E15.5 * Ctrl
10464045	Acsl5	NM_027976	3.73285e-005	-1.51821	E15.5 * Wnt1 down vs E15.5 * Ctrl
10428534	Trps1	NM_032000	8.70061e-005	-1.51828	E15.5 * Wnt1 down vs E15.5 * Ctrl
10432243	Fkbp11	NM_024169	3.70055e-005	-1.51835	E15.5 * Wnt1 down vs E15.5 * Ctrl
10512757	Hemgn	NM_053149	0.000730263	-1.51838	E15.5 * Wnt1 down vs E15.5 * Ctrl
10559221	Tnnt3	NM_011620	0.000484844	-1.51867	E15.5 * Wnt1 down vs E15.5 * Ctrl
10388880	Tmem97	NM_133706	0.00411822	-1.51873	E15.5 * Wnt1 down vs E15.5 * Ctrl
10429515	Lynx1	NM_011838	7.78077e-005	-1.5195	E15.5 * Wnt1 down vs E15.5 * Ctrl
10412227	Snx18	NM_130796	2.57258e-006	-1.51958	E15.5 * Wnt1 down vs E15.5 * Ctrl
10492815	Tmem154	NM_177260	0.000179456	-1.51977	E15.5 * Wnt1 down vs E15.5 * Ctrl
10596812	6230427J02Rik	NM_026597	3.2452e-005	-1.52015	E15.5 * Wnt1 down vs E15.5 * Ctrl
10488147	Flrt3	NM_178382	1.68701e-005	-1.52019	E15.5 * Wnt1 down vs E15.5 * Ctrl
10559486	Lair1	NM_001113474	0.00170793	-1.52055	E15.5 * Wnt1 down vs E15.5 * Ctrl
10469828	Psd4	NM_177611	8.40138e-005	-1.52062	E15.5 * Wnt1 down vs E15.5 * Ctrl
10444236	H2-DMb2	NM_010388	0.0018371	-1.52062	E15.5 * Wnt1 down vs E15.5 * Ctrl
10367076	Prim1	NM_008921	9.014e-006	-1.52105	E15.5 * Wnt1 down vs E15.5 * Ctrl
10543226	2610001J05Rik	NR_024619	5.78758e-006	-1.52113	E15.5 * Wnt1 down vs E15.5 * Ctrl
10492310	Mbnl1	NM_020007	1.94144e-007	-1.52204	E15.5 * Wnt1 down vs E15.5 * Ctrl
10448124	Fpr1	NM_013521	0.000138467	-1.52263	E15.5 * Wnt1 down vs E15.5 * Ctrl
10355147	Fam119a	NM_025964	0.0115414	-1.52296	E15.5 * Wnt1 down vs E15.5 * Ctrl
10487994	Fermt1	NM_198029	0.000472498	-1.52347	E15.5 * Wnt1 down vs E15.5 * Ctrl
10500614	Man1a2	NM_010763	8.85065e-008	-1.52353	E15.5 * Wnt1 down vs E15.5 * Ctrl
10554445	Prc1	NM_145150	2.23733e-005	-1.52381	E15.5 * Wnt1 down vs E15.5 * Ctrl
10484205	2610301F02Rik	ENSMUST00000	0.000204928	-1.52433	E15.5 * Wnt1 down vs E15.5 * Ctrl
10423024	Capsl	NM_029341	0.00100364	-1.52444	E15.5 * Wnt1 down vs E15.5 * Ctrl
10357856		---	0.000548647	-1.52554	E15.5 * Wnt1 down vs E15.5 * Ctrl
10387932	Pfn1	NM_011072	0.000939319	-1.52557	E15.5 * Wnt1 down vs E15.5 * Ctrl
10410209	Rybp	NM_019743	0.00026508	-1.52562	E15.5 * Wnt1 down vs E15.5 * Ctrl
10497817	Anxa5	NM_009673	1.58469e-005	-1.52564	E15.5 * Wnt1 down vs E15.5 * Ctrl
10355534	Tns1	NM_027884	6.12185e-005	-1.52594	E15.5 * Wnt1 down vs E15.5 * Ctrl
10361767	Sf3b5	NM_175102	1.66335e-006	-1.52603	E15.5 * Wnt1 down vs E15.5 * Ctrl
10552156	Rhpn2	NM_027897	9.54041e-005	-1.52613	E15.5 * Wnt1 down vs E15.5 * Ctrl
10505172	Dnajc25	NM_001033165	0.000103363	-1.52614	E15.5 * Wnt1 down vs E15.5 * Ctrl
10348451	Cxcr7	NM_007722	0.000463016	-1.52659	E15.5 * Wnt1 down vs E15.5 * Ctrl
10538138	Gimap1	NM_008376	0.000244887	-1.52682	E15.5 * Wnt1 down vs E15.5 * Ctrl
10508151	Clspn	NM_175554	4.12092e-005	-1.52695	E15.5 * Wnt1 down vs E15.5 * Ctrl
10592888	Cxcr5	NM_007551	4.45557e-006	-1.52719	E15.5 * Wnt1 down vs E15.5 * Ctrl
10444298	H2-Eb1	NM_010382	0.000208061	-1.52728	E15.5 * Wnt1 down vs E15.5 * Ctrl
10351224	F5	NM_007976	2.25634e-005	-1.52765	E15.5 * Wnt1 down vs E15.5 * Ctrl
10590623	Cxcr6	NM_030712	0.00566409	-1.52774	E15.5 * Wnt1 down vs E15.5 * Ctrl
10410078	Slc35d2	NM_001001321	0.000217495	-1.52784	E15.5 * Wnt1 down vs E15.5 * Ctrl
10554045	Adamts17	NM_001033877	4.73073e-008	-1.52808	E15.5 * Wnt1 down vs E15.5 * Ctrl
10398338		---	0.00634879	-1.52911	E15.5 * Wnt1 down vs E15.5 * Ctrl
10444752	Ltb	NM_008518	2.84314e-005	-1.52924	E15.5 * Wnt1 down vs E15.5 * Ctrl
10504743	Nans	NM_053179	7.58071e-005	-1.52963	E15.5 * Wnt1 down vs E15.5 * Ctrl
10534718	Trip6	NM_011639	0.000108083	-1.52963	E15.5 * Wnt1 down vs E15.5 * Ctrl
10517116	Rps6ka1	NM_009097	7.15679e-005	-1.53021	E15.5 * Wnt1 down vs E15.5 * Ctrl
10463799	EG329070	ENSMUST00000	1.11278e-006	-1.53068	E15.5 * Wnt1 down vs E15.5 * Ctrl

10385271	Ccng1	NM_009831	1.14733e-005	-1.53103	E15.5 * Wnt1 down vs E15.5 * Ctrl
10449303	Bak1	NM_007523	3.85432e-005	-1.53112	E15.5 * Wnt1 down vs E15.5 * Ctrl
10595768	Pls1	NM_001033210	2.09049e-005	-1.53135	E15.5 * Wnt1 down vs E15.5 * Ctrl
10531610	Rasgef1b	NM_145839	0.00133886	-1.53167	E15.5 * Wnt1 down vs E15.5 * Ctrl
10381798	Myl4	NM_010858	0.000174546	-1.53246	E15.5 * Wnt1 down vs E15.5 * Ctrl
10527441	Arpc1b	NM_023142	8.84945e-005	-1.53246	E15.5 * Wnt1 down vs E15.5 * Ctrl
10578615	Ing2	NM_023503	0.00286756	-1.53367	E15.5 * Wnt1 down vs E15.5 * Ctrl
10581378	Psmb10	NM_013640	2.61946e-005	-1.53452	E15.5 * Wnt1 down vs E15.5 * Ctrl
10454950	Slc35a4	NM_026404	1.21484e-005	-1.53454	E15.5 * Wnt1 down vs E15.5 * Ctrl
10485840	Ryr3	NM_177652	1.34672e-007	-1.53462	E15.5 * Wnt1 down vs E15.5 * Ctrl
10513101		---	0.0143552	-1.53471	E15.5 * Wnt1 down vs E15.5 * Ctrl
10548701	Lrp6	NM_008514	4.69071e-006	-1.53486	E15.5 * Wnt1 down vs E15.5 * Ctrl
10412328	Pelo	NM_134058	5.36401e-007	-1.53511	E15.5 * Wnt1 down vs E15.5 * Ctrl
10371846	Apaf1	NM_001042558	1.28718e-006	-1.53523	E15.5 * Wnt1 down vs E15.5 * Ctrl
10362823	Snx3	NM_017472	1.43381e-006	-1.53538	E15.5 * Wnt1 down vs E15.5 * Ctrl
10493555	Kcnn3	NM_080466	1.87193e-005	-1.53557	E15.5 * Wnt1 down vs E15.5 * Ctrl
10433885	Cebpd	NM_007679	0.000100503	-1.53562	E15.5 * Wnt1 down vs E15.5 * Ctrl
10542214	Klrd1	NM_010654	0.000189422	-1.53632	E15.5 * Wnt1 down vs E15.5 * Ctrl
10506822	Orc1l	NM_011015	0.002504	-1.53635	E15.5 * Wnt1 down vs E15.5 * Ctrl
10348817	02-Sep	NM_001159719	7.21308e-007	-1.53703	E15.5 * Wnt1 down vs E15.5 * Ctrl
10504761	LOC641050	M11024	0.00253327	-1.53734	E15.5 * Wnt1 down vs E15.5 * Ctrl
10591612	Dock6	NM_177030	0.00229599	-1.53759	E15.5 * Wnt1 down vs E15.5 * Ctrl
10408227	Hfe	NM_010424	0.000967303	-1.53774	E15.5 * Wnt1 down vs E15.5 * Ctrl
10581308	EG667952	ENSMUST00000(	2.06229e-006	-1.53823	E15.5 * Wnt1 down vs E15.5 * Ctrl
10588669	Rassf1	NM_019713	8.23233e-005	-1.5385	E15.5 * Wnt1 down vs E15.5 * Ctrl
10346191	Stat1	NM_009283	1.87598e-005	-1.53868	E15.5 * Wnt1 down vs E15.5 * Ctrl
10411728	Cenph	NM_021886	1.73033e-006	-1.53978	E15.5 * Wnt1 down vs E15.5 * Ctrl
10528810	Speer4a	NM_029376	3.39603e-006	-1.53999	E15.5 * Wnt1 down vs E15.5 * Ctrl
10422844	Gdnf	NM_010275	5.46049e-005	-1.54004	E15.5 * Wnt1 down vs E15.5 * Ctrl
10354003	Mgat4a	NM_173870	0.00973941	-1.54084	E15.5 * Wnt1 down vs E15.5 * Ctrl
10435383	Ptplb	NM_023587	4.40906e-005	-1.54125	E15.5 * Wnt1 down vs E15.5 * Ctrl
10452907	Fez2	NM_199448	0.000321835	-1.54193	E15.5 * Wnt1 down vs E15.5 * Ctrl
10514070	2310067E19Rik	BC066147	0.00172226	-1.54216	E15.5 * Wnt1 down vs E15.5 * Ctrl
10357436	Mcm6	NM_008567	1.69558e-009	-1.54226	E15.5 * Wnt1 down vs E15.5 * Ctrl
10542959	Bet1	NM_009748	0.00110794	-1.54258	E15.5 * Wnt1 down vs E15.5 * Ctrl
10553131	Kdelr1	NM_133950	1.75248e-006	-1.54262	E15.5 * Wnt1 down vs E15.5 * Ctrl
10366767	Ctdsp2	NM_146012	2.10497e-005	-1.54317	E15.5 * Wnt1 down vs E15.5 * Ctrl
10476560	Ism1	NM_001126490	0.000624054	-1.54343	E15.5 * Wnt1 down vs E15.5 * Ctrl
10598175	Ear10	NM_053112	0.000105664	-1.54383	E15.5 * Wnt1 down vs E15.5 * Ctrl
10491970	Lhfp	NM_175386	2.31977e-005	-1.54403	E15.5 * Wnt1 down vs E15.5 * Ctrl
10601011	Kif4	NM_008446	5.93178e-007	-1.54426	E15.5 * Wnt1 down vs E15.5 * Ctrl
10400805	Nin	NM_008697	6.45171e-008	-1.5448	E15.5 * Wnt1 down vs E15.5 * Ctrl
10541034	Anubl1	NM_001081317	0.000737185	-1.54501	E15.5 * Wnt1 down vs E15.5 * Ctrl
10434925	Hes1	NM_008235	9.54002e-007	-1.5451	E15.5 * Wnt1 down vs E15.5 * Ctrl
10426751	Tmbim6	NM_026669	1.91828e-008	-1.5466	E15.5 * Wnt1 down vs E15.5 * Ctrl
10541484	M6pr	NM_010749	2.41385e-006	-1.54667	E15.5 * Wnt1 down vs E15.5 * Ctrl
10405058	Omd	NM_012050	0.0016408	-1.54791	E15.5 * Wnt1 down vs E15.5 * Ctrl
10427214	Sp1	NM_013672	2.60316e-005	-1.54847	E15.5 * Wnt1 down vs E15.5 * Ctrl
10501229	Gstm1	NM_010358	0.00121689	-1.54898	E15.5 * Wnt1 down vs E15.5 * Ctrl
10584142	Ets1	NM_011808	8.35507e-006	-1.54939	E15.5 * Wnt1 down vs E15.5 * Ctrl
10346067		---	0.000579221	-1.54985	E15.5 * Wnt1 down vs E15.5 * Ctrl
10570483	Arhgef10	NM_172751	3.90628e-005	-1.55001	E15.5 * Wnt1 down vs E15.5 * Ctrl
10605501		---	0.00094121	-1.5505	E15.5 * Wnt1 down vs E15.5 * Ctrl
10494583	Sec22b	NM_011342	6.71685e-009	-1.5509	E15.5 * Wnt1 down vs E15.5 * Ctrl
10607658	Reps2	NM_178256	0.000224144	-1.55097	E15.5 * Wnt1 down vs E15.5 * Ctrl
10560655	Bcam	NM_020486	1.6759e-005	-1.55163	E15.5 * Wnt1 down vs E15.5 * Ctrl
10506118	Usp1	NM_146144	8.9516e-005	-1.55188	E15.5 * Wnt1 down vs E15.5 * Ctrl
10582378		---	0.000126746	-1.552	E15.5 * Wnt1 down vs E15.5 * Ctrl
10388234	Gsg2	NM_010353	3.03833e-006	-1.55222	E15.5 * Wnt1 down vs E15.5 * Ctrl
10602166	Nxt2	NM_172782	0.000147216	-1.55229	E15.5 * Wnt1 down vs E15.5 * Ctrl
10357630	Srgap2	NM_001081011	2.60285e-005	-1.55279	E15.5 * Wnt1 down vs E15.5 * Ctrl
10422962	1110020G09Rik	NM_001085410	3.94143e-007	-1.55302	E15.5 * Wnt1 down vs E15.5 * Ctrl
10533345	Aldh2	NM_009656	6.90049e-005	-1.55306	E15.5 * Wnt1 down vs E15.5 * Ctrl
10524169	Pole	NM_011132	5.157e-007	-1.55307	E15.5 * Wnt1 down vs E15.5 * Ctrl
10432640	Bin2	ENSMUST00000	3.61549e-005	-1.55333	E15.5 * Wnt1 down vs E15.5 * Ctrl
10379034	Tlcd1	NM_026708	0.000143781	-1.55359	E15.5 * Wnt1 down vs E15.5 * Ctrl

10405545	Pcbd2	NM_028281	0.000119379	-1.55372	E15.5 * Wnt1 down vs E15.5 * Ctrl
10578690	Neil3	NM_146208	4.62594e-006	-1.55451	E15.5 * Wnt1 down vs E15.5 * Ctrl
10440522	Adamts1	NM_009621	1.38422e-005	-1.55466	E15.5 * Wnt1 down vs E15.5 * Ctrl
10390186	Abi3	NM_025659	1.61103e-006	-1.55479	E15.5 * Wnt1 down vs E15.5 * Ctrl
10569319	Ctsd	NM_009983	0.000395038	-1.55495	E15.5 * Wnt1 down vs E15.5 * Ctrl
10377418	Tmem107	NM_028336	4.05854e-005	-1.55514	E15.5 * Wnt1 down vs E15.5 * Ctrl
10606714	Gla	NM_013463	0.00135878	-1.5561	E15.5 * Wnt1 down vs E15.5 * Ctrl
10583920	Eepd1	NM_026189	0.000498797	-1.55641	E15.5 * Wnt1 down vs E15.5 * Ctrl
10592201	Chek1	NM_007691	5.81738e-005	-1.55658	E15.5 * Wnt1 down vs E15.5 * Ctrl
10455721	Sncaip	NM_026408	0.00131542	-1.55683	E15.5 * Wnt1 down vs E15.5 * Ctrl
10346799	Icos	NM_017480	3.50098e-006	-1.55695	E15.5 * Wnt1 down vs E15.5 * Ctrl
10570855	Plat	NM_008872	0.000779463	-1.55711	E15.5 * Wnt1 down vs E15.5 * Ctrl
10540275	Glt8d4	NM_198612	4.21747e-006	-1.55727	E15.5 * Wnt1 down vs E15.5 * Ctrl
10475226	Stard9	ENSMUST00000	0.00126315	-1.55752	E15.5 * Wnt1 down vs E15.5 * Ctrl
10416689	Olfm4	NM_001030294	0.000641219	-1.55761	E15.5 * Wnt1 down vs E15.5 * Ctrl
10438378	Cdc45l	NM_009862	6.20868e-006	-1.55787	E15.5 * Wnt1 down vs E15.5 * Ctrl
10497349	Sirpb1	NM_001002898	5.84797e-005	-1.55805	E15.5 * Wnt1 down vs E15.5 * Ctrl
10410295	Zfp595	NM_177622	0.00501684	-1.5581	E15.5 * Wnt1 down vs E15.5 * Ctrl
10504849	Stx17	NM_026343	0.000109025	-1.55819	E15.5 * Wnt1 down vs E15.5 * Ctrl
10449741	Sik1	NM_010831	5.18271e-005	-1.5589	E15.5 * Wnt1 down vs E15.5 * Ctrl
10604763	Arpc1b	NM_023142	4.26806e-006	-1.55966	E15.5 * Wnt1 down vs E15.5 * Ctrl
10503431	Nbn	NM_013752	3.79854e-005	-1.55991	E15.5 * Wnt1 down vs E15.5 * Ctrl
10408074	Hist1h4b	NM_178193	0.0105446	-1.56007	E15.5 * Wnt1 down vs E15.5 * Ctrl
10384032	Pold2	NM_008894	3.10448e-005	-1.56026	E15.5 * Wnt1 down vs E15.5 * Ctrl
10605674	Pola1	NM_008892	5.77274e-006	-1.5608	E15.5 * Wnt1 down vs E15.5 * Ctrl
10424695	Gpihbp1	NM_026730	7.45594e-006	-1.56178	E15.5 * Wnt1 down vs E15.5 * Ctrl
10480714	Uap1l1	NM_001033293	2.00943e-005	-1.56209	E15.5 * Wnt1 down vs E15.5 * Ctrl
10369815	Cdc2a	NM_007659	4.87596e-005	-1.56226	E15.5 * Wnt1 down vs E15.5 * Ctrl
10459496	Ccbe1	NM_178793	2.85555e-008	-1.56228	E15.5 * Wnt1 down vs E15.5 * Ctrl
10536762	Snd1	NM_019776	2.38605e-005	-1.56228	E15.5 * Wnt1 down vs E15.5 * Ctrl
10555470	Arap1	NM_001040111	3.95958e-006	-1.56238	E15.5 * Wnt1 down vs E15.5 * Ctrl
10419790	Jub	NM_010590	0.000402157	-1.56261	E15.5 * Wnt1 down vs E15.5 * Ctrl
10600936	Efnb1	NM_010110	0.000119935	-1.56349	E15.5 * Wnt1 down vs E15.5 * Ctrl
10421581	Lrch1	NM_001033439	2.539e-007	-1.5639	E15.5 * Wnt1 down vs E15.5 * Ctrl
10588942	Lamb2	NM_008483	1.65704e-005	-1.56411	E15.5 * Wnt1 down vs E15.5 * Ctrl
10507101	OTTMUSG00000	NM_001085549	2.06456e-005	-1.56413	E15.5 * Wnt1 down vs E15.5 * Ctrl
10498519	Ssr3	NM_026155	1.37138e-008	-1.56443	E15.5 * Wnt1 down vs E15.5 * Ctrl
10587778		---	0.00884853	-1.56454	E15.5 * Wnt1 down vs E15.5 * Ctrl
10539143	Retsat	NM_026159	0.000979597	-1.56475	E15.5 * Wnt1 down vs E15.5 * Ctrl
10404686	Bmp6	NM_007556	1.69764e-005	-1.56479	E15.5 * Wnt1 down vs E15.5 * Ctrl
10503281	Glt8d3	NM_001033275	0.000836451	-1.56535	E15.5 * Wnt1 down vs E15.5 * Ctrl
10437817	Pdxdc1	NM_053181	3.90288e-007	-1.56547	E15.5 * Wnt1 down vs E15.5 * Ctrl
10590957	Fut4	NM_010242	0.000527742	-1.56574	E15.5 * Wnt1 down vs E15.5 * Ctrl
10409579	Cxcl14	NM_019568	0.000263198	-1.5658	E15.5 * Wnt1 down vs E15.5 * Ctrl
10594251	Kif23	NM_024245	1.14893e-006	-1.56585	E15.5 * Wnt1 down vs E15.5 * Ctrl
10499080	Arfip1	NM_001081093	0.000516048	-1.56594	E15.5 * Wnt1 down vs E15.5 * Ctrl
10594774	Ccnb2	NM_007630	6.97594e-006	-1.56642	E15.5 * Wnt1 down vs E15.5 * Ctrl
10420225	Cma1	NM_010780	2.73719e-005	-1.56653	E15.5 * Wnt1 down vs E15.5 * Ctrl
10596409	EG665033	AM748257	0.00044852	-1.56653	E15.5 * Wnt1 down vs E15.5 * Ctrl
10532157	Tmed5	NM_028876	5.2706e-006	-1.56654	E15.5 * Wnt1 down vs E15.5 * Ctrl
10425138	Sh3bp1	NM_009164	6.70299e-008	-1.56664	E15.5 * Wnt1 down vs E15.5 * Ctrl
10461558	Slc15a3	NM_023044	0.000231359	-1.56713	E15.5 * Wnt1 down vs E15.5 * Ctrl
10542917	Hepacam2	NM_178899	0.00145054	-1.5672	E15.5 * Wnt1 down vs E15.5 * Ctrl
10347351	Ctdsp1	NM_153088	1.78395e-005	-1.5684	E15.5 * Wnt1 down vs E15.5 * Ctrl
10418171	Zcchc24	NM_001101433	7.61006e-006	-1.56849	E15.5 * Wnt1 down vs E15.5 * Ctrl
10374364	Akt2	NM_001110208	3.99589e-005	-1.56867	E15.5 * Wnt1 down vs E15.5 * Ctrl
10493709	Slc39a1	NM_013901	3.12315e-005	-1.56882	E15.5 * Wnt1 down vs E15.5 * Ctrl
10540493	Edem1	NM_138677	6.29591e-005	-1.56907	E15.5 * Wnt1 down vs E15.5 * Ctrl
10552406	Nkg7	NM_024253	0.000530486	-1.56933	E15.5 * Wnt1 down vs E15.5 * Ctrl
10367673	Plekhg1	NM_001033253	0.0002074	-1.56988	E15.5 * Wnt1 down vs E15.5 * Ctrl
10382980	Syngr2	NM_009304	1.70774e-005	-1.57005	E15.5 * Wnt1 down vs E15.5 * Ctrl
10536472	Mdfic	NM_175088	0.000379376	-1.57065	E15.5 * Wnt1 down vs E15.5 * Ctrl
10360745	Lbr	NM_133815	6.24068e-006	-1.57145	E15.5 * Wnt1 down vs E15.5 * Ctrl
10476969	Pygb	NM_153781	4.06854e-006	-1.5723	E15.5 * Wnt1 down vs E15.5 * Ctrl
10497237	Pag1	NM_053182	6.00689e-005	-1.57241	E15.5 * Wnt1 down vs E15.5 * Ctrl
10582997	Casp4	NM_007609	0.000174058	-1.57256	E15.5 * Wnt1 down vs E15.5 * Ctrl

10542650	Golt1b	NM_025872	8.62719e-007	-1.57262	E15.5 * Wnt1 down vs E15.5 * Ctrl
10541695	Lpcat3	NM_145130	0.000340726	-1.57302	E15.5 * Wnt1 down vs E15.5 * Ctrl
10607189	Amot	NM_153319	0.000178017	-1.5732	E15.5 * Wnt1 down vs E15.5 * Ctrl
10429968		---	0.00737351	-1.5733	E15.5 * Wnt1 down vs E15.5 * Ctrl
10578222	Dlc1	NM_015802	2.44868e-005	-1.57348	E15.5 * Wnt1 down vs E15.5 * Ctrl
10408081	Hist1h1b	NM_020034	0.000361646	-1.57355	E15.5 * Wnt1 down vs E15.5 * Ctrl
10393047	Galk1	NM_016905	7.58425e-006	-1.57445	E15.5 * Wnt1 down vs E15.5 * Ctrl
10456745	Smad7	NM_001042660	2.40508e-005	-1.57446	E15.5 * Wnt1 down vs E15.5 * Ctrl
10372028	Plxnc1	NM_018797	3.97573e-006	-1.57546	E15.5 * Wnt1 down vs E15.5 * Ctrl
10492888		---	0.000754814	-1.57552	E15.5 * Wnt1 down vs E15.5 * Ctrl
10453604	Bambi	NM_026505	3.52016e-006	-1.57561	E15.5 * Wnt1 down vs E15.5 * Ctrl
10542079	Foxm1	NM_008021	2.31036e-006	-1.57652	E15.5 * Wnt1 down vs E15.5 * Ctrl
10381072	Cdc6	NM_011799	0.000215575	-1.5771	E15.5 * Wnt1 down vs E15.5 * Ctrl
10366238	Ppp1r12a	NM_027892	5.13591e-010	-1.57711	E15.5 * Wnt1 down vs E15.5 * Ctrl
10370510	Syde1	NM_027875	0.000610739	-1.57776	E15.5 * Wnt1 down vs E15.5 * Ctrl
10453026	Prkd3	NM_029239	2.43976e-005	-1.57799	E15.5 * Wnt1 down vs E15.5 * Ctrl
10478594	Ctsa	NM_008906	7.04512e-008	-1.57831	E15.5 * Wnt1 down vs E15.5 * Ctrl
10358978	Ier5	NM_010500	3.88372e-007	-1.57835	E15.5 * Wnt1 down vs E15.5 * Ctrl
10568361	Yipf5	NM_023311	3.43312e-005	-1.57899	E15.5 * Wnt1 down vs E15.5 * Ctrl
10559558	Tnni3	NM_009406	6.17835e-006	-1.57902	E15.5 * Wnt1 down vs E15.5 * Ctrl
10383206		---	0.00666213	-1.57962	E15.5 * Wnt1 down vs E15.5 * Ctrl
10546272	Iqsec1	NM_001134384	7.88081e-007	-1.57971	E15.5 * Wnt1 down vs E15.5 * Ctrl
10528794	5031410I06Rik	NM_207657	1.2213e-006	-1.57988	E15.5 * Wnt1 down vs E15.5 * Ctrl
10494351	Mtmr11	NM_181409	4.05782e-005	-1.58024	E15.5 * Wnt1 down vs E15.5 * Ctrl
10416126	Ebf2	NM_010095	0.000243462	-1.58037	E15.5 * Wnt1 down vs E15.5 * Ctrl
10414527	Pnp2	NM_001123371	0.000190615	-1.58038	E15.5 * Wnt1 down vs E15.5 * Ctrl
10402606	Rtl1	NM_184109	1.68058e-005	-1.58055	E15.5 * Wnt1 down vs E15.5 * Ctrl
10455852	Prrc1	NM_028447	3.39958e-007	-1.58065	E15.5 * Wnt1 down vs E15.5 * Ctrl
10439218	Pdia5	NM_028295	1.97091e-007	-1.5808	E15.5 * Wnt1 down vs E15.5 * Ctrl
10582190	Gins2	NM_178856	0.00211218	-1.58105	E15.5 * Wnt1 down vs E15.5 * Ctrl
10408243	Hist1h4b	NM_178193	0.00934627	-1.58106	E15.5 * Wnt1 down vs E15.5 * Ctrl
10385248	Hmmr	NM_013552	0.000259872	-1.5811	E15.5 * Wnt1 down vs E15.5 * Ctrl
10496001	Cfi	NM_007686	0.00123293	-1.58144	E15.5 * Wnt1 down vs E15.5 * Ctrl
10474596	Aven	NM_028844	0.00156468	-1.58207	E15.5 * Wnt1 down vs E15.5 * Ctrl
10583825	Zfp809	NM_172763	0.000246555	-1.58225	E15.5 * Wnt1 down vs E15.5 * Ctrl
10385842	Rad50	NM_009012	2.25519e-005	-1.58294	E15.5 * Wnt1 down vs E15.5 * Ctrl
10493692	Rab13	NM_026677	2.89938e-005	-1.58304	E15.5 * Wnt1 down vs E15.5 * Ctrl
10350297	Kif14	NM_001081258	4.934e-006	-1.58314	E15.5 * Wnt1 down vs E15.5 * Ctrl
10552945	Gys1	NM_030678	0.000378583	-1.58371	E15.5 * Wnt1 down vs E15.5 * Ctrl
10372410	Glipr1	NM_028608	0.000359762	-1.58376	E15.5 * Wnt1 down vs E15.5 * Ctrl
10502772	Lphn2	NM_001081298	1.70262e-005	-1.58391	E15.5 * Wnt1 down vs E15.5 * Ctrl
10385511	Psme2	NM_011190	0.00010483	-1.58403	E15.5 * Wnt1 down vs E15.5 * Ctrl
10503118	Fam110b	NM_173426	0.000388347	-1.58432	E15.5 * Wnt1 down vs E15.5 * Ctrl
10384656	B3gnt2	NM_016888	0.000339272	-1.58466	E15.5 * Wnt1 down vs E15.5 * Ctrl
10497931	Cflar	NM_009805	9.26028e-005	-1.58583	E15.5 * Wnt1 down vs E15.5 * Ctrl
10537410	Tbxas1	NM_011539	1.94242e-006	-1.58631	E15.5 * Wnt1 down vs E15.5 * Ctrl
10530560	Slain2	NM_153567	0.000239707	-1.58677	E15.5 * Wnt1 down vs E15.5 * Ctrl
10441456	Synj2	NM_011523	7.98949e-006	-1.58689	E15.5 * Wnt1 down vs E15.5 * Ctrl
10601616	Diap2	NM_017398	0.000886813	-1.58713	E15.5 * Wnt1 down vs E15.5 * Ctrl
10568709	Clrn3	NM_178669	0.000197989	-1.58733	E15.5 * Wnt1 down vs E15.5 * Ctrl
10488237	Snx5	NM_024225	1.44665e-008	-1.58752	E15.5 * Wnt1 down vs E15.5 * Ctrl
10466087	Tmem109	NM_134142	4.35016e-007	-1.58858	E15.5 * Wnt1 down vs E15.5 * Ctrl
10466127	AW112010	EF660528	0.00348537	-1.58905	E15.5 * Wnt1 down vs E15.5 * Ctrl
10410560	Trip13	NM_027182	5.0211e-006	-1.58936	E15.5 * Wnt1 down vs E15.5 * Ctrl
10347921	A530040E14Rik	ENSMUST00000(	0.00411935	-1.59092	E15.5 * Wnt1 down vs E15.5 * Ctrl
10550906	Plaur	NM_011113	5.53351e-005	-1.59119	E15.5 * Wnt1 down vs E15.5 * Ctrl
10409486	Pdlim7	NM_001114088	1.18756e-005	-1.59121	E15.5 * Wnt1 down vs E15.5 * Ctrl
10404061	Hist1h2bb	NM_175664	0.000781697	-1.59123	E15.5 * Wnt1 down vs E15.5 * Ctrl
10535559	Baiap2l1	NM_025833	7.14866e-005	-1.59222	E15.5 * Wnt1 down vs E15.5 * Ctrl
10519324	Cdk6	NM_009873	4.22478e-006	-1.59228	E15.5 * Wnt1 down vs E15.5 * Ctrl
10578322	Pdgfrl	NM_026840	0.000330665	-1.59249	E15.5 * Wnt1 down vs E15.5 * Ctrl
10376060	Irf1	NM_008390	0.000107982	-1.59306	E15.5 * Wnt1 down vs E15.5 * Ctrl
10504398	Serf2	NM_011354	0.000263779	-1.59317	E15.5 * Wnt1 down vs E15.5 * Ctrl
10416657	Elf1	NM_007920	3.70159e-005	-1.59351	E15.5 * Wnt1 down vs E15.5 * Ctrl
10349661	5430435G22Rik	NM_145509	8.14951e-006	-1.59353	E15.5 * Wnt1 down vs E15.5 * Ctrl
10368144	Tnfaip3	NM_009397	2.08531e-005	-1.59395	E15.5 * Wnt1 down vs E15.5 * Ctrl

10350136	Csrp1	NM_007791	2.77113e-005	-1.59447	E15.5 * Wnt1 down vs E15.5 * Ctrl
10594426	Zwilch	NM_026507	0.00016525	-1.59522	E15.5 * Wnt1 down vs E15.5 * Ctrl
10560618	Apoc1	NM_007469	0.000106107	-1.59543	E15.5 * Wnt1 down vs E15.5 * Ctrl
10458583	Yipf5	NM_023311	0.000438555	-1.59558	E15.5 * Wnt1 down vs E15.5 * Ctrl
10455961	Iigp1	NM_001146275	5.92797e-006	-1.59558	E15.5 * Wnt1 down vs E15.5 * Ctrl
10607116	Ammecr1	NM_019496	1.41914e-005	-1.59577	E15.5 * Wnt1 down vs E15.5 * Ctrl
10346874		---	0.0148189	-1.59644	E15.5 * Wnt1 down vs E15.5 * Ctrl
10576883	Shcbp1	NM_011369	2.3468e-007	-1.59668	E15.5 * Wnt1 down vs E15.5 * Ctrl
10367931	Ltv1	NM_181470	4.15867e-006	-1.59698	E15.5 * Wnt1 down vs E15.5 * Ctrl
10407390	Ptbp1	NM_001077363	0.000314965	-1.59728	E15.5 * Wnt1 down vs E15.5 * Ctrl
10584593		---	0.00501605	-1.59728	E15.5 * Wnt1 down vs E15.5 * Ctrl
10504763	Galnt12	NM_172693	1.47958e-005	-1.59793	E15.5 * Wnt1 down vs E15.5 * Ctrl
10416419	ENSMUSG0000(	ENSMUST00000	0.00301322	-1.59801	E15.5 * Wnt1 down vs E15.5 * Ctrl
10350630	Fam129a	NM_022018	8.40372e-005	-1.59853	E15.5 * Wnt1 down vs E15.5 * Ctrl
10493449	Thbs3	NM_013691	1.14874e-007	-1.59861	E15.5 * Wnt1 down vs E15.5 * Ctrl
10435271	Heg1	NM_175256	4.32176e-007	-1.5997	E15.5 * Wnt1 down vs E15.5 * Ctrl
10404067	Hist1h4b	NM_178193	0.00921112	-1.59974	E15.5 * Wnt1 down vs E15.5 * Ctrl
10473965	Arhgap1	NR_027373	4.73114e-005	-1.59997	E15.5 * Wnt1 down vs E15.5 * Ctrl
10352320	Tmem63a	NM_144794	9.52972e-007	-1.6002	E15.5 * Wnt1 down vs E15.5 * Ctrl
10395058	Adi1	NM_134052	0.000553391	-1.60026	E15.5 * Wnt1 down vs E15.5 * Ctrl
10419223	Fermt2	NM_146054	6.31656e-008	-1.6007	E15.5 * Wnt1 down vs E15.5 * Ctrl
10430245		---	0.00241642	-1.60151	E15.5 * Wnt1 down vs E15.5 * Ctrl
10502748	Lphn2	NM_001081298	5.25579e-007	-1.60226	E15.5 * Wnt1 down vs E15.5 * Ctrl
10408600	Serpinb6a	NM_009254	3.84516e-005	-1.60263	E15.5 * Wnt1 down vs E15.5 * Ctrl
10357158	Ralb	NM_022327	0.000292915	-1.6027	E15.5 * Wnt1 down vs E15.5 * Ctrl
10357008	Pign	NM_013784	6.74379e-008	-1.60277	E15.5 * Wnt1 down vs E15.5 * Ctrl
10386951	Hs3st3b1	NM_018805	8.67517e-005	-1.60306	E15.5 * Wnt1 down vs E15.5 * Ctrl
10420071	Tm9sf1	NM_028780	2.52681e-007	-1.60324	E15.5 * Wnt1 down vs E15.5 * Ctrl
10420198	Ripk3	NM_019955	1.45974e-005	-1.60344	E15.5 * Wnt1 down vs E15.5 * Ctrl
10522248	EG624662	XR_033663	9.37803e-006	-1.60349	E15.5 * Wnt1 down vs E15.5 * Ctrl
10428672	Dscc1	NM_183089	1.15349e-005	-1.60421	E15.5 * Wnt1 down vs E15.5 * Ctrl
10403584	Nid1	NM_010917	5.27915e-006	-1.60429	E15.5 * Wnt1 down vs E15.5 * Ctrl
10530421	Gabra4	NM_010251	2.41345e-005	-1.60432	E15.5 * Wnt1 down vs E15.5 * Ctrl
10500204	Ecm1	NM_007899	1.64824e-005	-1.60439	E15.5 * Wnt1 down vs E15.5 * Ctrl
10583669	AB124611	AB124611	0.000139094	-1.60488	E15.5 * Wnt1 down vs E15.5 * Ctrl
10382200	Ccdc46	NM_029606	9.79053e-007	-1.60505	E15.5 * Wnt1 down vs E15.5 * Ctrl
10401667	0610007P14Rik	BC004591	1.64212e-006	-1.60568	E15.5 * Wnt1 down vs E15.5 * Ctrl
10519747	Sema3e	NM_011348	6.51308e-005	-1.60576	E15.5 * Wnt1 down vs E15.5 * Ctrl
10592084	St3gal4	NM_009178	1.17663e-005	-1.60653	E15.5 * Wnt1 down vs E15.5 * Ctrl
10509542	Ddost	NM_007838	5.73253e-006	-1.60672	E15.5 * Wnt1 down vs E15.5 * Ctrl
10558227	Acadsb	NM_025826	0.00562019	-1.6073	E15.5 * Wnt1 down vs E15.5 * Ctrl
10584194	Srpr	NM_026130	0.00148488	-1.60759	E15.5 * Wnt1 down vs E15.5 * Ctrl
10497358	Sirpb1	NM_001002898	4.4041e-005	-1.60796	E15.5 * Wnt1 down vs E15.5 * Ctrl
10356248	C130026I21Rik	NM_175219	2.47249e-006	-1.60825	E15.5 * Wnt1 down vs E15.5 * Ctrl
10447602	Ezr	NM_009510	2.32792e-005	-1.60984	E15.5 * Wnt1 down vs E15.5 * Ctrl
10549276	Bhlhe41	NM_024469	3.95199e-007	-1.61116	E15.5 * Wnt1 down vs E15.5 * Ctrl
10537545	EG638904	XM_976192	0.000494196	-1.61124	E15.5 * Wnt1 down vs E15.5 * Ctrl
10482968	Pla2r1	NM_008867	2.29822e-005	-1.61179	E15.5 * Wnt1 down vs E15.5 * Ctrl
10581992	Maf	NM_001025577	0.000135498	-1.61189	E15.5 * Wnt1 down vs E15.5 * Ctrl
10507500	Slc6a9	NM_008135	2.87703e-006	-1.61219	E15.5 * Wnt1 down vs E15.5 * Ctrl
10601581	9230105E10Rik	NM_001146007	0.00124568	-1.61279	E15.5 * Wnt1 down vs E15.5 * Ctrl
10538142	Gimap5	NM_175035	1.25376e-005	-1.61304	E15.5 * Wnt1 down vs E15.5 * Ctrl
10455752	Snx24	NM_029394	0.00355991	-1.61313	E15.5 * Wnt1 down vs E15.5 * Ctrl
10369932	Susd2	NM_027890	0.00055195	-1.61349	E15.5 * Wnt1 down vs E15.5 * Ctrl
10389719	Scpep1	NM_029023	4.32515e-007	-1.61382	E15.5 * Wnt1 down vs E15.5 * Ctrl
10446074	Uhrf1	NM_010931	1.42974e-005	-1.61395	E15.5 * Wnt1 down vs E15.5 * Ctrl
10523516	4930524J08Rik	ENSMUST00000(	0.000184083	-1.61409	E15.5 * Wnt1 down vs E15.5 * Ctrl
10499666	Atp8b2	NM_001081182	5.26538e-007	-1.61429	E15.5 * Wnt1 down vs E15.5 * Ctrl
10606658	Trmt2b	NM_172540	0.00060195	-1.61449	E15.5 * Wnt1 down vs E15.5 * Ctrl
10555862	Trim34	NM_030684	7.32955e-005	-1.61451	E15.5 * Wnt1 down vs E15.5 * Ctrl
10499394	Lmna	NM_001002011	1.41517e-006	-1.61461	E15.5 * Wnt1 down vs E15.5 * Ctrl
10471503	Taf1d	BC110660	7.41985e-005	-1.61513	E15.5 * Wnt1 down vs E15.5 * Ctrl
10509992	Hspb7	NM_013868	0.000576506	-1.61547	E15.5 * Wnt1 down vs E15.5 * Ctrl
10573779	Nkd1	NM_027280	9.49284e-006	-1.61571	E15.5 * Wnt1 down vs E15.5 * Ctrl
10501313	OTTMUSG00000	XR_034306	4.21287e-005	-1.61613	E15.5 * Wnt1 down vs E15.5 * Ctrl
10353947	Tmem131	NM_018872	4.23034e-010	-1.61707	E15.5 * Wnt1 down vs E15.5 * Ctrl

10463308	Abcc2	NM_013806	2.04616e-005	-1.61747	E15.5 * Wnt1 down vs E15.5 * Ctrl
10360158	Ly9	NM_008534	9.89852e-006	-1.6175	E15.5 * Wnt1 down vs E15.5 * Ctrl
10479165	Edn3	NM_007903	1.23417e-007	-1.61792	E15.5 * Wnt1 down vs E15.5 * Ctrl
10493259	Ssr2	NM_025448	9.72182e-005	-1.61909	E15.5 * Wnt1 down vs E15.5 * Ctrl
10497490	Naaladl2	ENSMUST00000(	0.000101604	-1.61915	E15.5 * Wnt1 down vs E15.5 * Ctrl
10358879	Npl	NM_028749	0.000512992	-1.6196	E15.5 * Wnt1 down vs E15.5 * Ctrl
10379630	Slfn2	NM_011408	0.000829375	-1.61997	E15.5 * Wnt1 down vs E15.5 * Ctrl
10565018	Iqgap1	NM_016721	3.23209e-007	-1.62029	E15.5 * Wnt1 down vs E15.5 * Ctrl
10575733	Cenpn	NM_028131	1.3712e-006	-1.62042	E15.5 * Wnt1 down vs E15.5 * Ctrl
10391444	1700113i22rik	NM_026865	0.000364947	-1.62061	E15.5 * Wnt1 down vs E15.5 * Ctrl
10365420	AI597468	NM_001013028	5.43642e-008	-1.62101	E15.5 * Wnt1 down vs E15.5 * Ctrl
10477600		---	1.81384e-006	-1.62121	E15.5 * Wnt1 down vs E15.5 * Ctrl
10589929	Cmtm6	NM_026036	4.36301e-005	-1.62185	E15.5 * Wnt1 down vs E15.5 * Ctrl
10514000	Mpdz	NM_010820	7.92307e-008	-1.62211	E15.5 * Wnt1 down vs E15.5 * Ctrl
10548940	Lmo3	NM_207222	0.00152799	-1.62245	E15.5 * Wnt1 down vs E15.5 * Ctrl
10453867	Rbbp8	NM_001081223	0.000161605	-1.62251	E15.5 * Wnt1 down vs E15.5 * Ctrl
10443463	Cdkn1a	NM_007669	0.000389378	-1.62269	E15.5 * Wnt1 down vs E15.5 * Ctrl
10562720	Siglece	NM_031181	1.00378e-005	-1.62283	E15.5 * Wnt1 down vs E15.5 * Ctrl
10442087	4930546H06Rik	BC171984	0.000232745	-1.62306	E15.5 * Wnt1 down vs E15.5 * Ctrl
10582811	Irf2bp2	BC048951	7.28328e-006	-1.62321	E15.5 * Wnt1 down vs E15.5 * Ctrl
10449284	Dusp1	NM_013642	0.00730866	-1.62347	E15.5 * Wnt1 down vs E15.5 * Ctrl
10598976	Timp1	NM_001044384	0.000122967	-1.62401	E15.5 * Wnt1 down vs E15.5 * Ctrl
10495405	Slc25a24	NM_172685	1.38656e-007	-1.62431	E15.5 * Wnt1 down vs E15.5 * Ctrl
10439936	Nfkbiz	NM_030612	6.9128e-007	-1.62466	E15.5 * Wnt1 down vs E15.5 * Ctrl
10601659	Srpx2	NM_026838	0.000184896	-1.62599	E15.5 * Wnt1 down vs E15.5 * Ctrl
10435504	EG408196	NM_001082542	0.000277791	-1.62667	E15.5 * Wnt1 down vs E15.5 * Ctrl
10558410	Ptpre	NM_011212	7.78283e-005	-1.62714	E15.5 * Wnt1 down vs E15.5 * Ctrl
10531919	Hsd17b11	NM_053262	5.10139e-007	-1.62775	E15.5 * Wnt1 down vs E15.5 * Ctrl
10425945	Fbln1	NM_010180	4.44085e-005	-1.62782	E15.5 * Wnt1 down vs E15.5 * Ctrl
10463517	Pprc1	NM_001081214	1.43328e-005	-1.62936	E15.5 * Wnt1 down vs E15.5 * Ctrl
10365574	Pmch	NM_029971	0.0024454	-1.62949	E15.5 * Wnt1 down vs E15.5 * Ctrl
10415413	BC030046	BC151043	4.5104e-005	-1.63081	E15.5 * Wnt1 down vs E15.5 * Ctrl
10400405	Nfkbia	NM_010907	3.07254e-005	-1.631	E15.5 * Wnt1 down vs E15.5 * Ctrl
10377953	Kif1c	NM_153103	7.80357e-008	-1.63279	E15.5 * Wnt1 down vs E15.5 * Ctrl
10546661	Foxp1	NM_053202	2.41727e-006	-1.63285	E15.5 * Wnt1 down vs E15.5 * Ctrl
10490903	Car13	NM_024495	0.000184568	-1.63289	E15.5 * Wnt1 down vs E15.5 * Ctrl
10425283	Maff	NM_010755	0.000584458	-1.63316	E15.5 * Wnt1 down vs E15.5 * Ctrl
10557793	Stx4a	NM_009294	4.4705e-007	-1.63364	E15.5 * Wnt1 down vs E15.5 * Ctrl
10529457	Cpz	NM_153107	0.000189602	-1.63381	E15.5 * Wnt1 down vs E15.5 * Ctrl
10524878	BC023744	NM_001033311	2.96298e-005	-1.63489	E15.5 * Wnt1 down vs E15.5 * Ctrl
10455873	Slc12a2	NM_009194	1.21832e-006	-1.63521	E15.5 * Wnt1 down vs E15.5 * Ctrl
10436561	Usp25	NM_013918	2.86315e-006	-1.63552	E15.5 * Wnt1 down vs E15.5 * Ctrl
10371784	Nr1h4	NM_009108	0.000593963	-1.63558	E15.5 * Wnt1 down vs E15.5 * Ctrl
10590808	Yap1	NM_009534	1.89507e-007	-1.6356	E15.5 * Wnt1 down vs E15.5 * Ctrl
10440427	D16Ertd472e	NM_025967	5.69291e-005	-1.63607	E15.5 * Wnt1 down vs E15.5 * Ctrl
10591614	Dock6	NM_177030	0.00304145	-1.6361	E15.5 * Wnt1 down vs E15.5 * Ctrl
10546452	Adamts9	NM_175314	1.16166e-007	-1.63631	E15.5 * Wnt1 down vs E15.5 * Ctrl
10351603	Arhgap30	NM_001005508	4.55005e-006	-1.63656	E15.5 * Wnt1 down vs E15.5 * Ctrl
10533050	Hspb8	NM_030704	1.1603e-005	-1.6368	E15.5 * Wnt1 down vs E15.5 * Ctrl
10423654	Osr2	NM_054049	0.00432104	-1.63741	E15.5 * Wnt1 down vs E15.5 * Ctrl
10422496	Gpr183	NM_183031	9.17329e-006	-1.63787	E15.5 * Wnt1 down vs E15.5 * Ctrl
10594404	Smad3	NM_016769	2.92645e-006	-1.6384	E15.5 * Wnt1 down vs E15.5 * Ctrl
10565794	Serpinh1	NM_009825	2.64154e-007	-1.63935	E15.5 * Wnt1 down vs E15.5 * Ctrl
10584827	Mpzl2	NM_007962	6.98646e-005	-1.63945	E15.5 * Wnt1 down vs E15.5 * Ctrl
10401607	Pgf	NM_008827	0.000334478	-1.64062	E15.5 * Wnt1 down vs E15.5 * Ctrl
10554599	Adamtsl3	ENSMUST00000(	0.000108018	-1.64072	E15.5 * Wnt1 down vs E15.5 * Ctrl
10544525	Pdia4	NM_009787	5.17011e-005	-1.64097	E15.5 * Wnt1 down vs E15.5 * Ctrl
10509777	Iffo2	NM_183148	1.05623e-005	-1.64261	E15.5 * Wnt1 down vs E15.5 * Ctrl
10454310	Galnt1	NM_013814	1.84521e-007	-1.64294	E15.5 * Wnt1 down vs E15.5 * Ctrl
10520362	Insig1	NM_153526	2.7172e-005	-1.64386	E15.5 * Wnt1 down vs E15.5 * Ctrl
10579548	Glt25d1	NM_146211	0.000250753	-1.64408	E15.5 * Wnt1 down vs E15.5 * Ctrl
10500610	Fam46c	NM_001142952	0.000186129	-1.64451	E15.5 * Wnt1 down vs E15.5 * Ctrl
10398356		---	0.00036073	-1.64454	E15.5 * Wnt1 down vs E15.5 * Ctrl
10503833	Rplp1	BC091747	0.00497132	-1.64501	E15.5 * Wnt1 down vs E15.5 * Ctrl
10408689	Nrn1	NM_153529	3.66079e-007	-1.64559	E15.5 * Wnt1 down vs E15.5 * Ctrl
10493789	S100a13	NM_009113	8.68638e-008	-1.64572	E15.5 * Wnt1 down vs E15.5 * Ctrl

10361323	Cnksr3	NM_172546	9.12699e-006	-1.64611	E15.5 * Wnt1 down vs E15.5 * Ctrl
10413839	Ncoa4	NM_001033988	1.37045e-008	-1.6466	E15.5 * Wnt1 down vs E15.5 * Ctrl
10578703	Spcs3	NM_029701	2.231e-009	-1.64708	E15.5 * Wnt1 down vs E15.5 * Ctrl
10501164	Csf1	NM_007778	0.000741472	-1.64712	E15.5 * Wnt1 down vs E15.5 * Ctrl
10406519	Hapln1	NM_013500	0.00082517	-1.64721	E15.5 * Wnt1 down vs E15.5 * Ctrl
10563323	Nucb1	NM_008749	2.05458e-005	-1.64807	E15.5 * Wnt1 down vs E15.5 * Ctrl
10450367	Hspa1a	NM_010479	0.0127264	-1.64813	E15.5 * Wnt1 down vs E15.5 * Ctrl
10603746	Maob	NM_172778	0.00125705	-1.64821	E15.5 * Wnt1 down vs E15.5 * Ctrl
10414514	Pnp1	NM_013632	0.000259146	-1.64823	E15.5 * Wnt1 down vs E15.5 * Ctrl
10364262	Itgb2	NM_008404	6.97825e-006	-1.64884	E15.5 * Wnt1 down vs E15.5 * Ctrl
10575799	Plcg2	NM_172285	2.00321e-005	-1.64929	E15.5 * Wnt1 down vs E15.5 * Ctrl
10364251	Pofut2	NM_030262	6.93286e-007	-1.64984	E15.5 * Wnt1 down vs E15.5 * Ctrl
10428763	Atad2	NM_027435	5.34186e-008	-1.65022	E15.5 * Wnt1 down vs E15.5 * Ctrl
10403957	Hist1h4m	NM_175657	0.00757153	-1.65025	E15.5 * Wnt1 down vs E15.5 * Ctrl
10408092	Hist1h4m	NM_175657	0.00757153	-1.65025	E15.5 * Wnt1 down vs E15.5 * Ctrl
10420171	Adcy4	NM_080435	0.000265684	-1.65059	E15.5 * Wnt1 down vs E15.5 * Ctrl
10388389	Hic1	NM_010430	0.000273408	-1.65067	E15.5 * Wnt1 down vs E15.5 * Ctrl
10465185	Ehbp1l1	NM_001114597	2.59389e-007	-1.65119	E15.5 * Wnt1 down vs E15.5 * Ctrl
10369234		---	0.00523698	-1.65207	E15.5 * Wnt1 down vs E15.5 * Ctrl
10503410	Tmem64	NM_181401	4.56356e-008	-1.65212	E15.5 * Wnt1 down vs E15.5 * Ctrl
10600024	Gpr50	NM_010340	0.0109201	-1.65269	E15.5 * Wnt1 down vs E15.5 * Ctrl
10596394	E330026B02Rik	NM_172927	1.67189e-008	-1.65328	E15.5 * Wnt1 down vs E15.5 * Ctrl
10395277	Sypl	NM_013635	1.33608e-007	-1.6534	E15.5 * Wnt1 down vs E15.5 * Ctrl
10502791	Ifi44	NM_133871	0.000417395	-1.65366	E15.5 * Wnt1 down vs E15.5 * Ctrl
10456296	Malt1	NM_172833	2.58033e-005	-1.65404	E15.5 * Wnt1 down vs E15.5 * Ctrl
10364518	Ptbp1	NM_001077363	9.85451e-006	-1.65425	E15.5 * Wnt1 down vs E15.5 * Ctrl
10538706	Mmrn1	BC137623	0.00131008	-1.65542	E15.5 * Wnt1 down vs E15.5 * Ctrl
10507908	Fhl3	NM_010213	0.000139917	-1.6558	E15.5 * Wnt1 down vs E15.5 * Ctrl
10429160	St3gal1	NM_009177	3.84807e-005	-1.65589	E15.5 * Wnt1 down vs E15.5 * Ctrl
10466521	Gcnt1	NM_173442	4.63611e-005	-1.65611	E15.5 * Wnt1 down vs E15.5 * Ctrl
10512334	100038993	NM_001100596	9.39104e-008	-1.65619	E15.5 * Wnt1 down vs E15.5 * Ctrl
10512356	100038993	NM_001100596	9.39104e-008	-1.65619	E15.5 * Wnt1 down vs E15.5 * Ctrl
10554945	Prcp	NM_028243	1.14282e-005	-1.65684	E15.5 * Wnt1 down vs E15.5 * Ctrl
10530130	Rell1	NM_145923	1.18702e-005	-1.65801	E15.5 * Wnt1 down vs E15.5 * Ctrl
10516303		---	0.0124643	-1.65831	E15.5 * Wnt1 down vs E15.5 * Ctrl
10418848	Wdfy4	NM_001146022	5.15108e-006	-1.65838	E15.5 * Wnt1 down vs E15.5 * Ctrl
10523563	Cds1	NM_173370	3.91069e-005	-1.65868	E15.5 * Wnt1 down vs E15.5 * Ctrl
10591135	Fat3	NM_001080814	0.00206427	-1.65891	E15.5 * Wnt1 down vs E15.5 * Ctrl
10355325	Bard1	NM_007525	0.0001979	-1.65951	E15.5 * Wnt1 down vs E15.5 * Ctrl
10570656	Defa23	NM_001012307	8.67566e-005	-1.65954	E15.5 * Wnt1 down vs E15.5 * Ctrl
10570676	Defa23	NM_001012307	8.67566e-005	-1.65954	E15.5 * Wnt1 down vs E15.5 * Ctrl
10492426	Acsf2	NM_153807	0.000127265	-1.65998	E15.5 * Wnt1 down vs E15.5 * Ctrl
10367544	Ctdsp2	NM_001113470	0.000382112	-1.66002	E15.5 * Wnt1 down vs E15.5 * Ctrl
10470775	Cercam	NM_207298	2.31298e-006	-1.66088	E15.5 * Wnt1 down vs E15.5 * Ctrl
10506415	Oma1	NM_025909	3.32693e-005	-1.66111	E15.5 * Wnt1 down vs E15.5 * Ctrl
10446027	Chaf1a	NM_013733	3.60453e-008	-1.66133	E15.5 * Wnt1 down vs E15.5 * Ctrl
10455738	Snx2	NM_026386	1.23382e-008	-1.66156	E15.5 * Wnt1 down vs E15.5 * Ctrl
10416734		---	0.000517095	-1.66177	E15.5 * Wnt1 down vs E15.5 * Ctrl
10434668	Tmem97	NM_133706	4.27721e-006	-1.66215	E15.5 * Wnt1 down vs E15.5 * Ctrl
10377841	Tm4sf5	NM_029360	0.000152095	-1.66401	E15.5 * Wnt1 down vs E15.5 * Ctrl
10539119	Ggcx	NM_019802	1.33424e-005	-1.66408	E15.5 * Wnt1 down vs E15.5 * Ctrl
10439268	Dtx3l	NM_001013371	6.59961e-005	-1.66432	E15.5 * Wnt1 down vs E15.5 * Ctrl
10489484	Sdc4	NM_011521	7.52227e-008	-1.665	E15.5 * Wnt1 down vs E15.5 * Ctrl
10438708	Masp1	NM_008555	0.000357264	-1.66502	E15.5 * Wnt1 down vs E15.5 * Ctrl
10368343	Arg1	NM_007482	0.000291921	-1.66523	E15.5 * Wnt1 down vs E15.5 * Ctrl
10419156	Ear10	NM_053112	1.17375e-005	-1.66524	E15.5 * Wnt1 down vs E15.5 * Ctrl
10500938	Wnt2b	NM_009520	4.66014e-005	-1.66564	E15.5 * Wnt1 down vs E15.5 * Ctrl
10454192	Ttr	NM_013697	0.000808806	-1.66566	E15.5 * Wnt1 down vs E15.5 * Ctrl
10476538	Btbd3	NM_145534	1.48541e-007	-1.66683	E15.5 * Wnt1 down vs E15.5 * Ctrl
10558345	Dock1	NM_001033420	3.48918e-008	-1.6675	E15.5 * Wnt1 down vs E15.5 * Ctrl
10598178	Disp1	NM_026866	4.4895e-007	-1.66802	E15.5 * Wnt1 down vs E15.5 * Ctrl
10393573	Lgals3bp	NM_011150	1.54834e-005	-1.66824	E15.5 * Wnt1 down vs E15.5 * Ctrl
10523260	Shroom3	NM_015756	6.69835e-007	-1.6684	E15.5 * Wnt1 down vs E15.5 * Ctrl
10451372	Ptk7	NM_175168	1.24381e-008	-1.66856	E15.5 * Wnt1 down vs E15.5 * Ctrl
10446986	Crim1	NM_015800	3.04694e-006	-1.66974	E15.5 * Wnt1 down vs E15.5 * Ctrl
10572906	Mcm5	NM_008566	8.69644e-006	-1.67085	E15.5 * Wnt1 down vs E15.5 * Ctrl

10542822	Rep15	NM_025620	6.62481e-005	-1.67095	E15.5 * Wnt1 down vs E15.5 * Ctrl
10427496	Egflam	NM_178748	3.13992e-007	-1.67112	E15.5 * Wnt1 down vs E15.5 * Ctrl
10452316	C3	NM_009778	4.30619e-006	-1.67152	E15.5 * Wnt1 down vs E15.5 * Ctrl
10599696	Ddx26b	NM_172779	3.81545e-005	-1.67183	E15.5 * Wnt1 down vs E15.5 * Ctrl
10391561	Pyy	NM_145435	5.10617e-005	-1.67199	E15.5 * Wnt1 down vs E15.5 * Ctrl
10511180	Mxra8	NM_024263	3.42289e-007	-1.67224	E15.5 * Wnt1 down vs E15.5 * Ctrl
10362803	Cd164	NM_016898	5.45773e-008	-1.67279	E15.5 * Wnt1 down vs E15.5 * Ctrl
10511333	Plag1	NM_019969	0.000129978	-1.67346	E15.5 * Wnt1 down vs E15.5 * Ctrl
10411226	F2rl1	NM_007974	0.000129248	-1.67368	E15.5 * Wnt1 down vs E15.5 * Ctrl
10549877		---	0.000748414	-1.67404	E15.5 * Wnt1 down vs E15.5 * Ctrl
10497149	Gpr177	NM_026582	1.31038e-007	-1.67456	E15.5 * Wnt1 down vs E15.5 * Ctrl
10504106	Il11ra1	NM_010549	1.23864e-006	-1.67524	E15.5 * Wnt1 down vs E15.5 * Ctrl
10431424	Plxnb2	NM_001159521	0.000164095	-1.67545	E15.5 * Wnt1 down vs E15.5 * Ctrl
10474201	Lmo2	NM_008505	7.80962e-006	-1.67701	E15.5 * Wnt1 down vs E15.5 * Ctrl
10452643	2900073G15Rik	NM_026064	1.55685e-007	-1.67705	E15.5 * Wnt1 down vs E15.5 * Ctrl
10408525	1700018A04Rik	ENSMUST00000(	3.1806e-005	-1.6772	E15.5 * Wnt1 down vs E15.5 * Ctrl
10522788	Stap1	NM_019992	0.000308514	-1.67722	E15.5 * Wnt1 down vs E15.5 * Ctrl
10500666	Ptgfrn	NM_011197	0.000372672	-1.6773	E15.5 * Wnt1 down vs E15.5 * Ctrl
10381462	Rdm1	NM_025654	5.13393e-005	-1.67739	E15.5 * Wnt1 down vs E15.5 * Ctrl
10429607	Top1mt	NM_028404	2.03558e-005	-1.67748	E15.5 * Wnt1 down vs E15.5 * Ctrl
10483698	Wipf1	NM_153138	1.40807e-007	-1.67817	E15.5 * Wnt1 down vs E15.5 * Ctrl
10435697	Popdc2	NM_001081984	6.24991e-005	-1.67828	E15.5 * Wnt1 down vs E15.5 * Ctrl
10551185	Tgfb1	NM_011577	1.55919e-005	-1.67848	E15.5 * Wnt1 down vs E15.5 * Ctrl
10399198	Ncoa4	NM_019744	3.47254e-008	-1.68077	E15.5 * Wnt1 down vs E15.5 * Ctrl
10497364	Sirpb1	NM_001002898	2.03498e-005	-1.68081	E15.5 * Wnt1 down vs E15.5 * Ctrl
10406733	Zbed3	NM_028106	8.0521e-005	-1.68125	E15.5 * Wnt1 down vs E15.5 * Ctrl
10424439	Efr3a	NM_133766	6.89135e-006	-1.68196	E15.5 * Wnt1 down vs E15.5 * Ctrl
10387855	Alox15	NM_009660	9.17565e-006	-1.68206	E15.5 * Wnt1 down vs E15.5 * Ctrl
10396849	Rad51l1	NM_009014	0.00037412	-1.68293	E15.5 * Wnt1 down vs E15.5 * Ctrl
10384579	Ugp2	NM_139297	8.70674e-007	-1.68369	E15.5 * Wnt1 down vs E15.5 * Ctrl
10355871	Obsl1	NM_178884	4.62997e-006	-1.68411	E15.5 * Wnt1 down vs E15.5 * Ctrl
10407598	Ryr2	NM_023868	1.40222e-008	-1.68493	E15.5 * Wnt1 down vs E15.5 * Ctrl
10433633	Mkl2	NM_181860	3.32937e-005	-1.6852	E15.5 * Wnt1 down vs E15.5 * Ctrl
10356278	Sp110	NM_175397	6.66118e-007	-1.68538	E15.5 * Wnt1 down vs E15.5 * Ctrl
10424543	Wisp1	NM_018865	7.53618e-005	-1.68626	E15.5 * Wnt1 down vs E15.5 * Ctrl
10524266	Chek2	NM_016681	7.33727e-006	-1.68637	E15.5 * Wnt1 down vs E15.5 * Ctrl
10404506	Ripk1	NM_009068	1.35398e-006	-1.6865	E15.5 * Wnt1 down vs E15.5 * Ctrl
10528145	Grm3	NM_181850	0.000880509	-1.68736	E15.5 * Wnt1 down vs E15.5 * Ctrl
10382462	Slc9a3r1	NM_012030	0.000447546	-1.68748	E15.5 * Wnt1 down vs E15.5 * Ctrl
10428536	Trps1	NM_032000	6.99759e-006	-1.68763	E15.5 * Wnt1 down vs E15.5 * Ctrl
10578557	Ccdc111	NM_001001184	2.21977e-005	-1.68766	E15.5 * Wnt1 down vs E15.5 * Ctrl
10399965	F730043M19Rik	ENSMUST00000(	6.25177e-005	-1.68838	E15.5 * Wnt1 down vs E15.5 * Ctrl
10359303	4930562F07Rik	ENSMUST00000(	0.000133843	-1.68857	E15.5 * Wnt1 down vs E15.5 * Ctrl
10605143	Arhgap4	NM_138630	0.000196609	-1.68895	E15.5 * Wnt1 down vs E15.5 * Ctrl
10353167	Tram1	NM_028173	8.3513e-011	-1.6892	E15.5 * Wnt1 down vs E15.5 * Ctrl
10597461	Cmtm7	NM_133978	2.14065e-005	-1.68952	E15.5 * Wnt1 down vs E15.5 * Ctrl
10562576	Plekhf1	NM_024413	0.000329303	-1.68975	E15.5 * Wnt1 down vs E15.5 * Ctrl
10605792	Spin4	NM_178753	0.000485533	-1.68985	E15.5 * Wnt1 down vs E15.5 * Ctrl
10521622	4833430A08Rik	AK076499	0.000567773	-1.69004	E15.5 * Wnt1 down vs E15.5 * Ctrl
10509280	Hspg2	NM_008305	1.09377e-007	-1.69031	E15.5 * Wnt1 down vs E15.5 * Ctrl
10467578	Pik3ap1	NM_031376	2.93799e-007	-1.69041	E15.5 * Wnt1 down vs E15.5 * Ctrl
10606301	Magt1	NM_025952	3.69898e-006	-1.6909	E15.5 * Wnt1 down vs E15.5 * Ctrl
10437160	Ets2	NM_011809	1.15724e-006	-1.6914	E15.5 * Wnt1 down vs E15.5 * Ctrl
10451363	Srf	NM_020493	8.42009e-006	-1.69156	E15.5 * Wnt1 down vs E15.5 * Ctrl
10495675	F3	NM_010171	8.00082e-006	-1.69203	E15.5 * Wnt1 down vs E15.5 * Ctrl
10353989		---	0.00577144	-1.69234	E15.5 * Wnt1 down vs E15.5 * Ctrl
10459391	Fech	NM_007998	4.7198e-007	-1.69345	E15.5 * Wnt1 down vs E15.5 * Ctrl
10591620	Dock6	NM_177030	0.000606454	-1.69376	E15.5 * Wnt1 down vs E15.5 * Ctrl
10534102	Gusb	NM_010368	1.56508e-005	-1.69493	E15.5 * Wnt1 down vs E15.5 * Ctrl
10360789	Degs1	NM_007853	8.04201e-006	-1.6951	E15.5 * Wnt1 down vs E15.5 * Ctrl
10411359	Plp2	NM_019755	0.000164147	-1.69655	E15.5 * Wnt1 down vs E15.5 * Ctrl
10597239	Pth1r	NM_011199	3.12766e-007	-1.69659	E15.5 * Wnt1 down vs E15.5 * Ctrl
10345777	Il1rl2	NM_133193	0.000853116	-1.69664	E15.5 * Wnt1 down vs E15.5 * Ctrl
10358816	Lamc1	NM_010683	6.48741e-007	-1.69692	E15.5 * Wnt1 down vs E15.5 * Ctrl
10564857	Idh2	NM_173011	2.07983e-005	-1.69714	E15.5 * Wnt1 down vs E15.5 * Ctrl
10472916	Cdca7	NM_025866	7.4361e-005	-1.69735	E15.5 * Wnt1 down vs E15.5 * Ctrl

10363860	Slc16a9	NM_025807	0.00029962	-1.69741	E15.5 * Wnt1 down vs E15.5 * Ctrl
10520452	Il6	NM_031168	0.0050862	-1.69766	E15.5 * Wnt1 down vs E15.5 * Ctrl
10554938	Rab30	NM_029494	0.000179806	-1.69769	E15.5 * Wnt1 down vs E15.5 * Ctrl
10388958	Evi2a	NM_001033711	0.000497429	-1.69822	E15.5 * Wnt1 down vs E15.5 * Ctrl
10596113		---	0.000786423	-1.69824	E15.5 * Wnt1 down vs E15.5 * Ctrl
10532711	Cmklr1	NM_008153	2.84848e-006	-1.69918	E15.5 * Wnt1 down vs E15.5 * Ctrl
10379389	Adap2	NM_172133	1.96272e-006	-1.69937	E15.5 * Wnt1 down vs E15.5 * Ctrl
10394054	Cd7	NM_009854	7.07186e-006	-1.701	E15.5 * Wnt1 down vs E15.5 * Ctrl
10573451	Syce2	NM_027954	3.06805e-009	-1.70192	E15.5 * Wnt1 down vs E15.5 * Ctrl
10517967	Fblim1	NM_133754	2.9316e-005	-1.7035	E15.5 * Wnt1 down vs E15.5 * Ctrl
10381849	Efcab3	NM_001081046	1.20427e-006	-1.70366	E15.5 * Wnt1 down vs E15.5 * Ctrl
10355893	Epha4	NM_007936	5.62223e-006	-1.70387	E15.5 * Wnt1 down vs E15.5 * Ctrl
10406676	Lhfpl2	NM_172589	5.25509e-005	-1.70622	E15.5 * Wnt1 down vs E15.5 * Ctrl
10473022	Plp2	NM_019755	0.000664903	-1.70629	E15.5 * Wnt1 down vs E15.5 * Ctrl
10505791	BC057079	NM_001081184	6.51593e-008	-1.70649	E15.5 * Wnt1 down vs E15.5 * Ctrl
10404429	Serpinb9	NM_009256	3.3411e-005	-1.70699	E15.5 * Wnt1 down vs E15.5 * Ctrl
10404982	Barx1	NM_007526	0.000268588	-1.70707	E15.5 * Wnt1 down vs E15.5 * Ctrl
10572089	Nat2	NM_010874	0.00157204	-1.70709	E15.5 * Wnt1 down vs E15.5 * Ctrl
10414262	Ear2	NM_007895	4.86243e-005	-1.7076	E15.5 * Wnt1 down vs E15.5 * Ctrl
10570585		---	0.00217579	-1.70783	E15.5 * Wnt1 down vs E15.5 * Ctrl
10449775	Notch3	NM_008716	1.61807e-006	-1.70785	E15.5 * Wnt1 down vs E15.5 * Ctrl
10463224	Marveld1	BC054384	4.00999e-007	-1.70787	E15.5 * Wnt1 down vs E15.5 * Ctrl
10435457	Parp9	NM_030253	2.55774e-006	-1.70941	E15.5 * Wnt1 down vs E15.5 * Ctrl
10543762	Tsga13	NM_054073	2.51869e-005	-1.71018	E15.5 * Wnt1 down vs E15.5 * Ctrl
10581990		---	3.07818e-005	-1.71071	E15.5 * Wnt1 down vs E15.5 * Ctrl
10587818	Plscr4	NM_178711	0.000375184	-1.71112	E15.5 * Wnt1 down vs E15.5 * Ctrl
10426999	Acvrl1	NM_009612	4.99434e-006	-1.71286	E15.5 * Wnt1 down vs E15.5 * Ctrl
10520862	Fosl2	NM_008037	5.88645e-006	-1.71409	E15.5 * Wnt1 down vs E15.5 * Ctrl
10603346	Plp2	NM_019755	4.13184e-005	-1.71464	E15.5 * Wnt1 down vs E15.5 * Ctrl
10508012	Rspo1	NM_138683	6.15725e-005	-1.71464	E15.5 * Wnt1 down vs E15.5 * Ctrl
10547869	Leprel2	NM_013534	5.16301e-008	-1.71469	E15.5 * Wnt1 down vs E15.5 * Ctrl
10496605	Ccbl2	NM_173763	4.49617e-005	-1.71528	E15.5 * Wnt1 down vs E15.5 * Ctrl
10508800	ENSMUSG0000C	ENSMUST00000(	0.00520572	-1.71535	E15.5 * Wnt1 down vs E15.5 * Ctrl
10573865	Chd9	ENSMUST00000	0.00520572	-1.71535	E15.5 * Wnt1 down vs E15.5 * Ctrl
10586079	Itga11	NM_176922	4.21495e-005	-1.71593	E15.5 * Wnt1 down vs E15.5 * Ctrl
10428604	Tnfrsf11b	NM_008764	3.03971e-006	-1.71709	E15.5 * Wnt1 down vs E15.5 * Ctrl
10403743	Inhba	NM_008380	7.94372e-005	-1.71738	E15.5 * Wnt1 down vs E15.5 * Ctrl
10355567	Tmbim1	NM_027154	2.22921e-005	-1.71769	E15.5 * Wnt1 down vs E15.5 * Ctrl
10410138	Cntnap3	NM_001081129	2.90888e-006	-1.71823	E15.5 * Wnt1 down vs E15.5 * Ctrl
10531479	4932430I15Rik	ENSMUST00000	6.31963e-007	-1.71833	E15.5 * Wnt1 down vs E15.5 * Ctrl
10556266	Wee1	NM_009516	3.45826e-006	-1.71921	E15.5 * Wnt1 down vs E15.5 * Ctrl
10404649	Dsp	NM_023842	3.77381e-005	-1.71922	E15.5 * Wnt1 down vs E15.5 * Ctrl
10481540	Fnbp1	NM_001038700	5.77189e-006	-1.72006	E15.5 * Wnt1 down vs E15.5 * Ctrl
10395103	Pxdn	NM_181395	7.53166e-007	-1.72051	E15.5 * Wnt1 down vs E15.5 * Ctrl
10505674	Cntln	NM_175275	1.02538e-009	-1.72093	E15.5 * Wnt1 down vs E15.5 * Ctrl
10585395	Siva1	NM_013929	0.000252472	-1.7212	E15.5 * Wnt1 down vs E15.5 * Ctrl
10468909	Disp1	NM_026866	3.22311e-006	-1.7214	E15.5 * Wnt1 down vs E15.5 * Ctrl
10530692	Kdr	NM_010612	7.49611e-006	-1.72244	E15.5 * Wnt1 down vs E15.5 * Ctrl
10465916	Gm98	BC157942	2.02632e-005	-1.72273	E15.5 * Wnt1 down vs E15.5 * Ctrl
10556280	Swap70	NM_009302	3.89201e-007	-1.72385	E15.5 * Wnt1 down vs E15.5 * Ctrl
10448836	Tmem204	NM_001001183	1.31155e-006	-1.724	E15.5 * Wnt1 down vs E15.5 * Ctrl
10577808	Tacc1	NM_177089	2.99823e-008	-1.72576	E15.5 * Wnt1 down vs E15.5 * Ctrl
10538590	Herc5	ENSMUST00000(	2.57633e-006	-1.72613	E15.5 * Wnt1 down vs E15.5 * Ctrl
10480601	A830007P12Rik	NM_146115	5.96878e-006	-1.72641	E15.5 * Wnt1 down vs E15.5 * Ctrl
10385504	EG432555	NM_001024230	8.99893e-005	-1.72657	E15.5 * Wnt1 down vs E15.5 * Ctrl
10366796	Mettl1	NM_010792	3.61362e-005	-1.72775	E15.5 * Wnt1 down vs E15.5 * Ctrl
10528008	Steap2	NM_001103157	1.09524e-005	-1.72776	E15.5 * Wnt1 down vs E15.5 * Ctrl
10512024	Mobkl2b	NM_178061	3.91206e-005	-1.72804	E15.5 * Wnt1 down vs E15.5 * Ctrl
10411622	Birc1f	NM_010871	0.000100198	-1.72809	E15.5 * Wnt1 down vs E15.5 * Ctrl
10496324	Slc39a8	NM_001135149	2.84635e-005	-1.72842	E15.5 * Wnt1 down vs E15.5 * Ctrl
10538802	A930038C07Rik	NM_172399	9.27553e-005	-1.7285	E15.5 * Wnt1 down vs E15.5 * Ctrl
10596637	Mapkapk3	NM_178907	1.40508e-005	-1.72858	E15.5 * Wnt1 down vs E15.5 * Ctrl
10443007	EG240055	NM_001081656	1.02546e-006	-1.7287	E15.5 * Wnt1 down vs E15.5 * Ctrl
10596148	Trf	NM_133977	2.35786e-006	-1.72886	E15.5 * Wnt1 down vs E15.5 * Ctrl
10492864	Sh3d19	NM_001082414	2.00659e-007	-1.73023	E15.5 * Wnt1 down vs E15.5 * Ctrl
10450694	H2-T22	NM_010397	1.46057e-005	-1.73039	E15.5 * Wnt1 down vs E15.5 * Ctrl

10437236	B230307C23Rik	ENSMUST00000	0.00173623	-1.731	E15.5 * Wnt1 down vs E15.5 * Ctrl
10432439	Fmnl3	NM_011711	3.15335e-006	-1.73181	E15.5 * Wnt1 down vs E15.5 * Ctrl
10541246	Il17ra	NM_008359	4.27983e-005	-1.73299	E15.5 * Wnt1 down vs E15.5 * Ctrl
10363545	Neurog3	NM_009719	1.18418e-006	-1.73311	E15.5 * Wnt1 down vs E15.5 * Ctrl
10364444	Madcam1	NM_013591	0.000171269	-1.73491	E15.5 * Wnt1 down vs E15.5 * Ctrl
10606333	Fndc3c1	NM_001007580	0.000101439	-1.73505	E15.5 * Wnt1 down vs E15.5 * Ctrl
10349157	Serpinb2	NM_011111	6.593e-007	-1.73522	E15.5 * Wnt1 down vs E15.5 * Ctrl
10439895	Alcam	NM_009655	2.70996e-005	-1.73561	E15.5 * Wnt1 down vs E15.5 * Ctrl
10364375	Cstb	NM_007793	7.78592e-005	-1.73632	E15.5 * Wnt1 down vs E15.5 * Ctrl
10497608	Mds1	NM_021442	0.000112267	-1.73653	E15.5 * Wnt1 down vs E15.5 * Ctrl
10461909	BC016495	NM_145497	7.46929e-005	-1.73696	E15.5 * Wnt1 down vs E15.5 * Ctrl
10566346	9230105E10Rik	NM_001146007	0.000879356	-1.73718	E15.5 * Wnt1 down vs E15.5 * Ctrl
10458303	Ecscr	NM_001033141	2.03548e-006	-1.73809	E15.5 * Wnt1 down vs E15.5 * Ctrl
10398240	Eml1	NM_001043335	1.32064e-006	-1.7391	E15.5 * Wnt1 down vs E15.5 * Ctrl
10372652	Lyz1	NM_013590	0.00103833	-1.73939	E15.5 * Wnt1 down vs E15.5 * Ctrl
10396936	Smoc1	NM_001146217	0.000352707	-1.73984	E15.5 * Wnt1 down vs E15.5 * Ctrl
10606071	Ercc6l	NM_146235	3.77625e-006	-1.74008	E15.5 * Wnt1 down vs E15.5 * Ctrl
10362097	Raet1b	BC132022	0.000302585	-1.74027	E15.5 * Wnt1 down vs E15.5 * Ctrl
10552919	Hrc	NM_010473	0.000800256	-1.74052	E15.5 * Wnt1 down vs E15.5 * Ctrl
10453394	Six2	NM_011380	2.66603e-006	-1.74055	E15.5 * Wnt1 down vs E15.5 * Ctrl
10423049	Prlr	BC096586	0.00013773	-1.74092	E15.5 * Wnt1 down vs E15.5 * Ctrl
10543959	Ptn	NM_008973	9.7683e-005	-1.74121	E15.5 * Wnt1 down vs E15.5 * Ctrl
10450519	Tcf19	NM_025674	5.40692e-008	-1.74126	E15.5 * Wnt1 down vs E15.5 * Ctrl
10495083	Kcnd3	NM_001039347	1.31708e-005	-1.74148	E15.5 * Wnt1 down vs E15.5 * Ctrl
10546510	Lrig1	NM_008377	4.06747e-006	-1.7415	E15.5 * Wnt1 down vs E15.5 * Ctrl
10482802	Cytip	NM_139200	0.000199794	-1.74236	E15.5 * Wnt1 down vs E15.5 * Ctrl
10454782	Egr1	NM_007913	0.00134105	-1.74265	E15.5 * Wnt1 down vs E15.5 * Ctrl
10532504	Hscb	NM_153571	2.5964e-005	-1.74327	E15.5 * Wnt1 down vs E15.5 * Ctrl
10458382	Cd14	NM_009841	0.000588682	-1.74585	E15.5 * Wnt1 down vs E15.5 * Ctrl
10467871	Dnmbp	NM_028029	3.63857e-005	-1.74616	E15.5 * Wnt1 down vs E15.5 * Ctrl
10464251	Atrnl1	NM_181415	2.64077e-006	-1.74665	E15.5 * Wnt1 down vs E15.5 * Ctrl
10502535		---	0.0130134	-1.74794	E15.5 * Wnt1 down vs E15.5 * Ctrl
10582464		---	0.0125101	-1.74797	E15.5 * Wnt1 down vs E15.5 * Ctrl
10529819	Fgfbp1	NM_008009	1.4728e-006	-1.74853	E15.5 * Wnt1 down vs E15.5 * Ctrl
10607705	S100g	NM_009789	0.000450685	-1.74868	E15.5 * Wnt1 down vs E15.5 * Ctrl
10580183	Ier2	NM_010499	0.0002638	-1.74886	E15.5 * Wnt1 down vs E15.5 * Ctrl
10466624	Aldh1a7	NM_011921	0.000231539	-1.74895	E15.5 * Wnt1 down vs E15.5 * Ctrl
10398362	Rian	AF357355	0.00636848	-1.74918	E15.5 * Wnt1 down vs E15.5 * Ctrl
10410152	Cntnap3	NM_001081129	2.17175e-008	-1.74941	E15.5 * Wnt1 down vs E15.5 * Ctrl
10456492	D18Ertd653e	NM_172631	7.80896e-006	-1.74964	E15.5 * Wnt1 down vs E15.5 * Ctrl
10518069	Efhd2	NM_025994	1.26188e-005	-1.75009	E15.5 * Wnt1 down vs E15.5 * Ctrl
10371591	4930547N16Rik	NM_029249	8.37652e-006	-1.75038	E15.5 * Wnt1 down vs E15.5 * Ctrl
10518408	Plod1	NM_011122	3.84735e-005	-1.75069	E15.5 * Wnt1 down vs E15.5 * Ctrl
10438753	Leprel1	NM_173379	2.23679e-008	-1.75073	E15.5 * Wnt1 down vs E15.5 * Ctrl
10379044	Rab34	NM_033475	5.60473e-007	-1.75148	E15.5 * Wnt1 down vs E15.5 * Ctrl
10544133	Parp12	NM_172893	1.8876e-005	-1.75171	E15.5 * Wnt1 down vs E15.5 * Ctrl
10502774	Lphn2	NM_001081298	1.46516e-008	-1.75338	E15.5 * Wnt1 down vs E15.5 * Ctrl
10400254	Heatr5a	NM_177171	4.26247e-008	-1.75553	E15.5 * Wnt1 down vs E15.5 * Ctrl
10441436	Snx9	NM_025664	4.43555e-008	-1.75633	E15.5 * Wnt1 down vs E15.5 * Ctrl
10505623	D4Bwg0951e	BC030404	0.000121559	-1.75684	E15.5 * Wnt1 down vs E15.5 * Ctrl
10559467	Pira2	NM_011089	3.11192e-006	-1.75769	E15.5 * Wnt1 down vs E15.5 * Ctrl
10473367	Slc43a1	NM_001081349	1.07422e-005	-1.75813	E15.5 * Wnt1 down vs E15.5 * Ctrl
10497356	Sirpb1	NM_001002898	3.59625e-005	-1.75825	E15.5 * Wnt1 down vs E15.5 * Ctrl
10364950	Gadd45b	NM_008655	1.19196e-005	-1.75892	E15.5 * Wnt1 down vs E15.5 * Ctrl
10430510	Tmem184b	NM_172608	0.000307465	-1.76072	E15.5 * Wnt1 down vs E15.5 * Ctrl
10504127	Ccl21a	NM_011124	5.33265e-006	-1.76106	E15.5 * Wnt1 down vs E15.5 * Ctrl
10504154	Ccl21a	NM_011124	5.33265e-006	-1.76106	E15.5 * Wnt1 down vs E15.5 * Ctrl
10504183	Ccl21a	NM_011124	5.33265e-006	-1.76106	E15.5 * Wnt1 down vs E15.5 * Ctrl
10512377	Ccl21a	NM_011124	5.33265e-006	-1.76106	E15.5 * Wnt1 down vs E15.5 * Ctrl
10506154	Alg6	NM_001081264	3.25341e-005	-1.7615	E15.5 * Wnt1 down vs E15.5 * Ctrl
10378697	Myo1c	NM_008659	4.75993e-008	-1.7625	E15.5 * Wnt1 down vs E15.5 * Ctrl
10443408	Mapk13	NM_011950	2.99779e-006	-1.76267	E15.5 * Wnt1 down vs E15.5 * Ctrl
10565759	Uvrag	NM_178635	0.000155234	-1.76298	E15.5 * Wnt1 down vs E15.5 * Ctrl
10439296	Stfa2	NM_001082545	3.98072e-005	-1.76395	E15.5 * Wnt1 down vs E15.5 * Ctrl
10542632	Iapp	NM_010491	8.32351e-009	-1.76422	E15.5 * Wnt1 down vs E15.5 * Ctrl
10582008	2310061C15Rik	NM_026844	2.76114e-007	-1.76438	E15.5 * Wnt1 down vs E15.5 * Ctrl

10483800		---	0.00048804	-1.76487	E15.5 * Wnt1 down vs E15.5 * Ctrl
10538150	Tmem176a	NM_025326	7.38684e-008	-1.76512	E15.5 * Wnt1 down vs E15.5 * Ctrl
10436841	Il10rb	NM_008349	6.41041e-005	-1.76546	E15.5 * Wnt1 down vs E15.5 * Ctrl
10457091	Neto1	NM_144946	0.000544383	-1.76564	E15.5 * Wnt1 down vs E15.5 * Ctrl
10493990	S100a11	NM_016740	8.99634e-008	-1.76616	E15.5 * Wnt1 down vs E15.5 * Ctrl
10399046	Vipr2	NM_009511	5.74792e-005	-1.76775	E15.5 * Wnt1 down vs E15.5 * Ctrl
10375245	Gabrb2	NM_008070	0.00018906	-1.76797	E15.5 * Wnt1 down vs E15.5 * Ctrl
10551803	Zfp568	NM_001033355	1.82365e-008	-1.76818	E15.5 * Wnt1 down vs E15.5 * Ctrl
10366956	Stat6	NM_009284	1.82031e-006	-1.76839	E15.5 * Wnt1 down vs E15.5 * Ctrl
10469255	Prkcq	NM_008859	2.22084e-005	-1.7693	E15.5 * Wnt1 down vs E15.5 * Ctrl
10365845	Fgd6	NM_053072	9.10106e-007	-1.76934	E15.5 * Wnt1 down vs E15.5 * Ctrl
10429568	Ly6c1	NM_010741	0.000119551	-1.76942	E15.5 * Wnt1 down vs E15.5 * Ctrl
10426451	Irak4	NM_029926	0.000652114	-1.7706	E15.5 * Wnt1 down vs E15.5 * Ctrl
10419744	Slc7a7	NM_011405	2.68548e-005	-1.77082	E15.5 * Wnt1 down vs E15.5 * Ctrl
10441601	Tagap	NM_145968	2.63774e-005	-1.77142	E15.5 * Wnt1 down vs E15.5 * Ctrl
10410766	Nr2f1	NM_010151	9.1323e-009	-1.77336	E15.5 * Wnt1 down vs E15.5 * Ctrl
10474642	BC052040	NM_001145898	1.06385e-005	-1.77369	E15.5 * Wnt1 down vs E15.5 * Ctrl
10385776	Tcf7	NM_009331	9.72749e-006	-1.77455	E15.5 * Wnt1 down vs E15.5 * Ctrl
10502205	Hadh	NM_008212	1.52629e-005	-1.77458	E15.5 * Wnt1 down vs E15.5 * Ctrl
10490212	Ctsz	NM_022325	1.12629e-008	-1.77484	E15.5 * Wnt1 down vs E15.5 * Ctrl
10363512	Sar1a	NM_009120	3.85679e-008	-1.77519	E15.5 * Wnt1 down vs E15.5 * Ctrl
10457640	S100a11	NM_016740	3.1146e-007	-1.77542	E15.5 * Wnt1 down vs E15.5 * Ctrl
10414025	Gdf10	NM_145741	3.69917e-009	-1.77563	E15.5 * Wnt1 down vs E15.5 * Ctrl
10440393	Samsn1	NM_023380	6.65926e-007	-1.77576	E15.5 * Wnt1 down vs E15.5 * Ctrl
10466745	Tjp2	NM_011597	8.54829e-007	-1.77606	E15.5 * Wnt1 down vs E15.5 * Ctrl
10468081	Npm3	NM_008723	0.00139139	-1.7762	E15.5 * Wnt1 down vs E15.5 * Ctrl
10456904	Pstpip2	NM_013831	6.50618e-006	-1.77665	E15.5 * Wnt1 down vs E15.5 * Ctrl
10435345	Mylk	NM_139300	3.30089e-007	-1.77688	E15.5 * Wnt1 down vs E15.5 * Ctrl
10345046	Efhc1	EU520262	2.46564e-006	-1.77728	E15.5 * Wnt1 down vs E15.5 * Ctrl
10380896	Erbb2	NM_001003817	1.9094e-005	-1.77751	E15.5 * Wnt1 down vs E15.5 * Ctrl
10433656		---	3.94575e-005	-1.77755	E15.5 * Wnt1 down vs E15.5 * Ctrl
10500982	I830077J02Rik	NM_001033780	1.04026e-005	-1.77767	E15.5 * Wnt1 down vs E15.5 * Ctrl
10478160	Fam83d	BC068129	2.78545e-007	-1.78015	E15.5 * Wnt1 down vs E15.5 * Ctrl
10413803	Btd	NM_025295	5.07753e-005	-1.78138	E15.5 * Wnt1 down vs E15.5 * Ctrl
10513608	Alad	NM_008525	7.77139e-007	-1.78189	E15.5 * Wnt1 down vs E15.5 * Ctrl
10582809	Tk1	NM_009387	6.00819e-006	-1.78328	E15.5 * Wnt1 down vs E15.5 * Ctrl
10578649	Odz3	NM_011857	4.22092e-007	-1.78376	E15.5 * Wnt1 down vs E15.5 * Ctrl
10520612	Khk	NM_008439	1.16996e-005	-1.7839	E15.5 * Wnt1 down vs E15.5 * Ctrl
10375751	Adamts2	NM_175643	1.3351e-007	-1.78444	E15.5 * Wnt1 down vs E15.5 * Ctrl
10526797	Stag3	NM_016964	5.74866e-007	-1.78458	E15.5 * Wnt1 down vs E15.5 * Ctrl
10475866	Bcl2l11	NM_207680	3.24519e-007	-1.78563	E15.5 * Wnt1 down vs E15.5 * Ctrl
10531737	Hpse	NM_152803	2.68318e-005	-1.78577	E15.5 * Wnt1 down vs E15.5 * Ctrl
10389395	Brip1	NM_178309	6.62609e-008	-1.78645	E15.5 * Wnt1 down vs E15.5 * Ctrl
10539421	Mobkl1b	NM_145571	4.87765e-007	-1.78702	E15.5 * Wnt1 down vs E15.5 * Ctrl
10498978	Lrat	NM_023624	0.00391264	-1.78766	E15.5 * Wnt1 down vs E15.5 * Ctrl
10464128	Casp7	NM_007611	2.38509e-005	-1.78822	E15.5 * Wnt1 down vs E15.5 * Ctrl
10549420	Tmtc1	NM_198967	1.44217e-006	-1.78826	E15.5 * Wnt1 down vs E15.5 * Ctrl
10496813	Ctbs	NM_028836	6.17252e-005	-1.78848	E15.5 * Wnt1 down vs E15.5 * Ctrl
10393559	Timp2	NM_011594	3.47023e-010	-1.78914	E15.5 * Wnt1 down vs E15.5 * Ctrl
10439130	Umps	NM_009471	7.48596e-008	-1.78943	E15.5 * Wnt1 down vs E15.5 * Ctrl
[truncated: 2,105,241 more chars]
