# Supplementary material for: Transcription and Signaling Regulators in Developing Neuronal Subtypes of Mouse and Human Enteric Nervous System
Source: Gastroenterology. 2018 Feb;154(3):624–36. doi: 10.1053/j.gastro.2017.10.005 (PMC6381388; doi:10.1053/j.gastro.2017.10.005)
Supplement: Supplementary Table 3 [file mmc13.pdf]

Supplementary Table 3: Transcription Factors enriched (>1.2) in pairwise comparisons between cell populations in the developing gut

Transcription factors were identified using GO terms *transcription factor activity* (GO:0000989), and *regulation of transcription, DNA dependent* (GO:0006355). The combined lists of genes were manually screened to remove wrongly annotated genes.

**Sox11vsWnt11**		**Sox11vsSox15**	
**S11vsW11**	**Fold Change >1.2**	**S11vsS15**	**Fold Change >1.2**
Hdx	highly divergent homeobox	Adnp2	ADNP homeobox 2
		Alx1	ALX homeobox 1
		Arid3a	AT rich interactive domain 3A (B
		Csda	cold shock domain protein A
		Dbp	D site albumin promoter binding
		E2f3	E2F transcription factor 3
		E2f5	E2F transcription factor 5
		E2f6	E2F transcription factor 6
		Elk3	ELK3, member of ETS oncogene
		Ets1	E26 avian leukemia oncogene 1,
		Etv2	similar to ETS related protein 71
		Etv6	ets variant gene 6 (TEL oncogene
		Fli1	Friend leukemia integration 1
		Foxf1a	forkhead box F1a
		Foxp2	forkhead box P2
		Gata3	GATA binding protein 3
		Gatad1	GATA zinc finger domain contair
		Gatad2a	similar to Transcriptional repress
		Gli1	GLI-Kruppel family member GLI1
		Gzf1	GDNF-inducible zinc finger prote
		Hand1	heart and neural crest derivative
		Hdac1	similar to histone deacetylase HI
		Hmga1	high mobility group AT-hook I, re
		Hoxa1	homeo box A1
		Hoxc9	homeo box C9
		Hoxd10	homeo box D10
		Hoxd9	homeo box D9
		Isl1	ISL1 transcription factor, LIM/ho
		Jarid2	jumonji, AT rich interactive dom
		Lbh	limb-bud and heart
		Lef1	lymphoid enhancer binding facto
		Meis2	Meis homeobox 2
		Mrpl28	mitochondrial ribosomal protein
		Myc	myelocytomatosis oncogene
		Mycn	v-myc myelocytomatosis viral re

		NeuroD4	neurogenic differentiation 4
		Nfe2l2	nuclear factor, erythroid derivec
		Nkx6-1	NK6 homeobox 1
		Nr1h5	nuclear receptor subfamily 1, gr
		Nr2f1	nuclear receptor subfamily 2, gr
		Nr2f2	similar to COUP-TFI; nuclear rece
		Pax3	paired box gene 3
		Pbx2	pre B-cell leukemia transcription
		Pitx2	paired-like homeodomain transc
		Plagl2	pleiomorphic adenoma gene-like
		Pou4f2	POU domain, class 4, transcripti
		Prrx1	paired related homeobox 1
		Rest	RE1-silencing transcription facto
		Rex2	hypothetical protein LOC100048
		Rsl1	regulator of sex limited protein 1
		Sall4	sal-like 4 (Drosophila)
		Six1	sine oculis-related homeobox 1
		Six2	similar to Homeobox protein SIX
		Sox9	SRY-box containing gene 9
		Tbx20	T-box 20
		Tcf21	transcription factor 21
		Tcfap2a	transcription factor AP-2, alpha
		Tcfap2b	transcription factor AP-2 beta
		Tfam	transcription factor A, mitochon
		Tfdp1	predicted gene 7390; transcripti
		Tgif1	TGFB-induced factor homeobox
		Trp53	transformation related protein 5
		Zbtb16	zinc finger and BTB domain cont
		Zfp101	zinc finger protein 101
		Zfp334	zinc finger protein 334
		Zfp454	zinc finger protein 454
		Zfp54	hypothetical protein LOC100044
		Zfp568	zinc finger protein 568
		Zfp58	zinc finger protein 58
		Zfp607	zinc finger proten 607
		Zfp617	zinc finger protein 617
		Zfp708	zinc finger protein 708
		Zfp820	hypothetical protein LOC100044
		Zfp936	RIKEN cDNA I1C0022H11 gene; s
		Zik1	zinc finger protein interacting wi
		Zim1	zinc finger, imprinted 1
		Zscan12	zinc finger and SCAN domain cor
		Zscan4-ps2	
**WntE11vsSoxE11**		**WntE11vsWntE15**	
**W11vsS11**	**Fold Change >1.2**	**W11vsW15**	**Fold Change >1.2**
Foxd1	forkhead box D1	Adnp2	ADNP homeobox 2

Neurod1	neurogenic differentiation 1; neu	Alx1	ALX homeobox 1
Nr2f1	nuclear receptor subfamily 2, gro	Arid3a	AT rich interactive domain 3A (B
Prrx1	paired related homeobox 1	Atf1	predicted gene 1862; activating
Satb2	special AT-rich sequence binding	Creb5	RIKEN cDNA 9430076C15 gene;
		Csda	cold shock domain protein A
		E2f1	E2F transcription factor 1
		E2f3	E2F transcription factor 3
		E2f4	E2F transcription factor 4
		E2f5	E2F transcription factor 5
		E2f6	E2F transcription factor 6
		E2f7	E2F transcription factor 7
		E2f8	E2F transcription factor 8
		Elk3	ELK3, member of ETS oncogene
		Erf	Ets2 repressor factor
		Erg	avian erythroblastosis virus E-26
		Ets1	E26 avian leukemia oncogene 1,
		Etv4	ets variant gene 4 (E1A enhance
		Etv5	ets variant gene 5
		Etv6	ets variant gene 6 (TEL oncogene
		Fli1	Friend leukemia integration 1
		Foxc1	forkhead box C1
		Foxc2	forkhead box C2
		Foxd1	forkhead box D1
		Foxf1a	forkhead box F1a
		Foxf2	forkhead box F2
		Foxm1	forkhead box M1; RIKEN cDNA 4
		Foxp2	forkhead box P2
		Foxp4	forkhead box P4
		Gabpa	GA repeat binding protein, alpha
		Gata3	GATA binding protein 3
		Gatad2a	similar to Transcriptional repres
		Gli1	GLI-Kruppel family member GLI1
		Gli2	GLI-Kruppel family member GLI2
		Glis2	GLIS family zinc finger 2
		Gzf1	GDNF-inducible zinc finger prote
		Hes1	hairy and enhancer of split 1 (Dr
		Hesx1	homeo box gene expressed in ES
		Heyl	hairy/enhancer-of-split related w
		Hmx1	H6 homeo box 1
		Homez	homeodomain leucine zipper-en
		Hoxa1	homeo box A1
		Hsf1	heat shock factor 1
		I1C0022H1	RIKEN cDNA I1C0022H11 gene; s
		Isl1	ISL1 transcription factor, LIM/ho
		Jarid2	jumonji, AT rich interactive dom
		Lbh	limb-bud and heart

			Lef1	lymphoid enhancer binding facto
			Mef2c	myocyte enhancer factor 2C
			Meis2	Meis homeobox 2
			Mkx	mohawk homeobox
			Mta1	similar to metastasis-associated
			Mta2	metastasis-associated gene fami
			Mtf1	metal response element binding
			Myb	myeloblastosis oncogene
			Myc	myelocytomatosis oncogene
			Mycn	v-myc myelocytomatosis viral re
			Neurod1	neurogenic differentiation 1; neu
			NeuroD4	neurogenic differentiation 4
			Nfatc3	nuclear factor of activated T-cell
			Nfe2l2	nuclear factor, erythroid derived
			Nfkb1	nuclear factor of kappa light poly
			Nfya	nuclear transcription factor-Y alp
			Nfyc	nuclear transcription factor-Y ga
			Nr2f1	nuclear receptor subfamily 2, gro
			Nr2f2	similar to COUP-TFI; nuclear rece
			Nr2f6	nuclear receptor subfamily 2, gro
			Patz1	POZ (BTB) and AT hook containin
			Pax3	paired box gene 3
			Pbx2	pre B-cell leukemia transcription
			Plagl2	pleiomorphic adenoma gene-like
			Pou4f1	POU domain, class 4, transcriptio
			Preb	prolactin regulatory element bin
			Prox1	prospero-related homeobox 1
			Prrx1	paired related homeobox 1
			Rara	retinoic acid receptor, alpha
			Rest	RE1-silencing transcription facto
			Rex2	hypothetical protein LOC100048
			Rxra	retinoid X receptor alpha; simila
			Rxrg	retinoid X receptor gamma
			Sall4	sal-like 4 (Drosophila)
			Six1	sine oculis-related homeobox 1
			Six2	similar to Homeobox protein SIX
			Six4	sine oculis-related homeobox 4
			Smad3	MAD homolog 3 (Drosophila)
			Sox10	SRY-box containing gene 10
			Sox5	SRY-box containing gene 5
			Sox8	SRY-box containing gene 8
			Sox9	SRY-box containing gene 9
			Sp1	trans-acting transcription factor
			Tbx20	T-box 20
			Tcf3	transcription factor E2a
			Tcf7	transcription factor 7, T-cell spec

		Tcfap2a	transcription factor AP-2, alpha
		Tcfap2b	transcription factor AP-2 beta
		Tcfap2c	transcription factor AP-2, gamm
		Tead2	TEA domain family member 2
		Tead3	TEA domain family member 3
		Tfdp1	predicted gene 7390; transcripti
		Tfdp2	transcription factor Dp 2
		Tgif1	TGFB-induced factor homeobox
		Trp53	transformation related protein 5
		Trps1	trichorhinophalangeal syndrome
		Twist1	twist homolog 1 (Drosophila)
		Twist2	twist homolog 2 (Drosophila)
		Ybx1	predicted gene 6540; predicted
		Zbtb16	zinc finger and BTB domain cont
		Zeb2	zinc finger E-box binding homeo
		Zfp101	zinc finger protein 101
		Zfp191	zinc finger protein 191
		Zfp334	zinc finger protein 334
		Zfp386	zinc finger protein 386 (Kruppel-
		Zfp566	zinc finger protein 566
		Zfp568	zinc finger protein 568
		Zfp58	zinc finger protein 58
		Zfp617	zinc finger protein 617
		Zfp69	zinc finger protein 69
		Zfp809	zinc finger protein 809
		Zfp820	hypothetical protein LOC100044
		Zfp931	RIKEN cDNA 2810021G02 gene
		Zscan12	zinc finger and SCAN domain co
		Zscan29	zinc finger SCAN domains 29
**SoxE15vsWntE15**		**SoxE15 vs SoxE11**	
**S15vsW15**	**Fold Change >1.2**	**S15vsS11**	**Fold Change >1.2**
Arntl	aryl hydrocarbon receptor nuclea	Aff1	AF4/FMR2 family, member 1
Bcl6	B-cell leukemia/lymphoma 6	Ahr	aryl-hydrocarbon receptor
Cebpd	CCAAT/enhancer binding protein	Arntl	aryl hydrocarbon receptor nucle
Csda	cold shock domain protein A	Atf3	activating transcription factor 3
E2f3	E2F transcription factor 3	Bcl6	B-cell leukemia/lymphoma 6
E2f5	E2F transcription factor 5	Cebpa	CCAAT/enhancer binding protei
E2f7	E2F transcription factor 7	Cebpd	CCAAT/enhancer binding protei
E2f8	E2F transcription factor 8	Csrnp3	cysteine-serine-rich nuclear prot
Elk3	ELK3, member of ETS oncogene fa	Cux1	cut-like homeobox 1
Ets1	E26 avian leukemia oncogene 1, 5	Cux2	cut-like homeobox 2
Etv5	ets variant gene 5	Dach1	dachshund 1 (Drosophila)
Etv6	ets variant gene 6 (TEL oncogene)	Dlx5	distal-less homeobox 5
Foxd3	forkhead box D3	Dlx6	distal-less homeobox 6
Foxm1	forkhead box M1; RIKEN cDNA 49	Ebf1	early B-cell factor 1
Foxn2	forkhead box N2	Ebf3	early B-cell factor 3

Foxo1	forkhead box O1	Ebf4	early B-cell factor 4
Hdac1	similar to histone deacetylase HD	Egr1	early growth response 1
Hes1	hairy and enhancer of split 1 (Dro	Egr2	early growth response 2
Hey2	hairy/enhancer-of-split related w	Esrrg	estrogen-related receptor gamm
Hsf1	heat shock factor 1	Ets2	E26 avian leukemia oncogene 2,
Irf1	interferon regulatory factor 1	Etv1	ets variant gene 1
Mef2c	myocyte enhancer factor 2C	Fos	FBJ osteosarcoma oncogene
Mkl2	MKL/myocardin-like 2	Foxj2	forkhead box J2
Myb	myeloblastosis oncogene	Foxj3	forkhead box J3
Myc	myelocytomatosis oncogene	Foxo1	forkhead box O1
Neurog3	neurogenin 3	Foxo3	forkhead box O3
Nfe2l2	nuclear factor, erythroid derived	Foxo6	forkhead box O6
Nfia	nuclear factor I/A	Gatad2b	GATA zinc finger domain contair
Nfkb1	nuclear factor of kappa light poly	Hey2	hairy/enhancer-of-split related v
Nfyb	nuclear transcription factor-Y bet	Hoxa2	homeo box A2
Nfyc	nuclear transcription factor-Y gan	Hoxa3	homeo box A3
Nr2f1	nuclear receptor subfamily 2, gro	Hoxa4	homeo box A4
Nr2f2	similar to COUP-TFI; nuclear rece	Hoxa5	homeo box A5
Nr4a1	nuclear receptor subfamily 4, gro	Hoxa6	homeo box A6
Plagl2	pleiomorphic adenoma gene-like	Hoxb5	homeo box B5
Rela	v-rel reticuloendotheliosis viral or	Hoxb6	homeo box B6
Relb	avian reticuloendotheliosis viral (	Hoxb8	homeo box B8
Rest	RE1-silencing transcription factor	Hoxc4	homeo box C4
Rex2	hypothetical protein LOC100048	Hoxc5	homeo box C5
Smad3	MAD homolog 3 (Drosophila)	Hoxd3	homeo box D3
Sox10	SRY-box containing gene 10	Irf1	interferon regulatory factor 1
Sox5	SRY-box containing gene 5	Klf12	Kruppel-like factor 12
Srf	serum response factor	Klf2	Kruppel-like factor 2 (lung)
St18	suppression of tumorigenicity 18	Klf4	Kruppel-like factor 4 (gut)
Tcf3	transcription factor E2a	Klf6	Kruppel-like factor 6
Tcfap2a	transcription factor AP-2, alpha	Klf7	Kruppel-like factor 7 (ubiquitous
Tgif1	TGFB-induced factor homeobox 1	Meis1	Meis homeobox 1
Trp53	transformation related protein 5	Mxi1	Max interacting protein 1
Zfp238	zinc finger protein 238	Myt1	myelin transcription factor 1
Zfp568	zinc finger protein 568	Myt1l	myelin transcription factor 1-like
		Neurog3	neurogenin 3
		Nfatc1	nuclear factor of activated T-cell
		Nfe2l3	nuclear factor, erythroid derived
		Nfia	nuclear factor I/A
		Nfib	nuclear factor I/B
		Nfil3	similar to NFIL3/E4BP4 transcrip
		Nfix	nuclear factor I/X
		Nr1d2	nuclear receptor subfamily 1, gr
		Nr1h4	nuclear receptor subfamily 1, gr
		Nr2c2	nuclear receptor subfamily 2, gr
		Nr3c1	nuclear receptor subfamily 3, gr

Nr4a1	nuclear receptor subfamily 4, gr
Nr4a2	nuclear receptor subfamily 4, gr
Phox2a	paired-like homeobox 2a
Pknox1	Pbx/knotted 1 homeobox
Rarb	retinoic acid receptor, beta
Rbak	RB-associated KRAB repressor
Relb	avian reticuloendotheliosis viral
Rora	RAR-related orphan receptor alp
Rorc	RAR-related orphan receptor ga
Satb1	special AT-rich sequence binding
Satb2	special AT-rich sequence binding
Sox6	SRY-box containing gene 6
Spen	SPEN homolog, transcriptional r
St18	suppression of tumorigenicity 18
Stat3	similar to Stat3B; signal transdu
Stat5b	signal transducer and activator c
Tbx3	T-box 3
Tcf4	transcription factor 4
Tcf7l2	transcription factor 7-like 2, T-ce
Thra	thyroid hormone receptor alpha
Tshz1	teashirt zinc finger family memb
Zeb1	zinc finger E-box binding homeo
Zfhx4	zinc finger homeodomain 4
Zfp263	zinc finger protein 263
Zfp445	zinc finger protein 445
Zfp612	zinc finger protein 612
Zfp788	zinc finger protein 788
Zfp941	cDNA sequence BC066028
Zhx3	zinc fingers and homeoboxes 3
Zkscan14	zinc finger with KRAB and SCAN
Zkscan2	zinc finger with KRAB and SCAN

WntE15vsSoxE15

W15vsS15	Fold Change >1.2
Csrnp3	cysteine-serine-rich nuclear prote
Cux2	cut-like homeobox 2
Ebf1	early B-cell factor 1
Etv1	ets variant gene 1
Hlf	hepatic leukemia factor
Hoxb5	homeo box B5
Hoxb6	homeo boxB6
Hoxc4	homeo box C4
Jazf1	JAZF zinc finger 1
Klf7	Kruppel-like factor 7 (ubiquitous)
Meis1	Meis homeobox 1

WntE15vsWntE11

W15vsW11	Fold Change >1.2
Ahr	aryl-hydrocarbon receptor
Ar	androgen receptor
Atf2	activating transcription factor 2;
Atf6	activating transcription factor 6
Atf7	activating transcription factor 7
Carf	calcium response factor
Cebpa	CCAAT/enhancer binding protei
Clock	circadian locomoter output cycle
Crebl2	cAMP responsive element bindi
Csrnp3	cysteine-serine-rich nuclear prot
Cux2	cut-like homeobox 2

Meis3	Meis homeobox 3	Dach1	dachshund 1 (Drosophila)
Myt1l	myelin transcription factor 1-like	Dlx3	distal-less homeobox 3
Onecut2	one cut domain, family member 2	Ebf1	early B-cell factor 1
Pbx3	similar to PBX3a; pre B-cell leuker	Ebf3	early B-cell factor 3
Pgr	progesterone receptor	Ebf4	early B-cell factor 4
Tcf7l2	transcription factor 7-like 2, T-cel	Esrrg	estrogen-related receptor gamm
Thra	thyroid hormone receptor alpha;	Ets2	E26 avian leukemia oncogene 2,
Trps1	trichorhinophalan	Etv1	ets variant gene 1
Tshz3	teashirt zinc finger family membe	Foxj2	forkhead box J2
Zfp40	zinc finger protein 40	Foxn3	forkhead box N3
Zfp52	zinc finger protein 52	Foxo1	forkhead box O1
Zfp57	zinc finger protein 57	Foxo3	forkhead box O3
Zfp60	similar to Zinc finger protein 60; z	Hey2	hairy/enhancer-of-split related w
Zfp78	zinc finger protein 78	Hivep2	human immunodeficiency virus t
Zfp811	zinc finger protein 811	Hlf	hepatic leukemia factor
Zfp9	zinc finger protein 9	Hoxa2	homeo box A2
Zfp941	cDNA sequence BC066028	Hoxa3	homeo box A3
		Hoxa4	homeo box A4
		Hoxa5	homeo box A5
		Hoxa6	homeo box A6
		Hoxb5	homeo box B5
		Hoxb6	homeo box B6
		Hoxc4	homeo box C4
		Hoxc5	homeo box C5
		Hoxd3	homeo box D3
		Hoxd4	homeo box D4
		Irf2	interferon regulatory factor 2
		Jazf1	JAZF zinc finger 1
		Klf7	Kruppel-like factor 7 (ubiquitous
		Mafg	similar to mafG; v-maf musculoa
		Mef2a	similar to Myocyte enhancer fac
		Meis1	Meis homeobox 1
		Mycl1	v-myc myelocytomatosis viral or
		Myt1	myelin transcription factor 1
		Myt1l	myelin transcription factor 1-like
		Nfatc1	nuclear factor of activated T-cell
		Nfe2l3	nuclear factor, erythroid derived
		Nfia	nuclear factor I/A
		Nfib	nuclear factor I/B
		Nfil3	similar to NFIL3/E4BP4 transcrip
		Nfix	nuclear factor I/X
		Nr1d2	nuclear receptor subfamily 1, gro
		Nr2c2	nuclear receptor subfamily 2, gro
		Nr3c1	nuclear receptor subfamily 3, gro
		Nr4a1	nuclear receptor subfamily 4, gro
		Nr6a1	nuclear receptor subfamily 6, gro

		Onecut2	one cut domain, family member
		Pbx3	similar to PBX3a; pre B-cell leuke
		Pgr	progesterone receptor
		Phox2a	paired-like homeobox 2a
		Pknox1	Pbx/knotted 1 homeobox
		Rarb	retinoic acid receptor, beta
		Rora	RAR-related orphan receptor alp
		Rorc	RAR-related orphan receptor ga
		Runx1	runt related transcription factor
		Satb2	special AT-rich sequence binding
		Smad9	MAD homolog 9 (Drosophila)
		St18	suppression of tumorigenicity 18
		Stat2	signal transducer and activator c
		Stat3	similar to Stat3B; signal transduc
		Stat5b	signal transducer and activator c
		Tbx3	T-box 3
		Tcf4	transcription factor 4
		Thra	thyroid hormone receptor alpha
		Tshz1	teashirt zinc finger family memb
		Tshz3	teashirt zinc finger family memb
		Zeb1	zinc finger E-box binding homeo
		Zfhx2	zinc finger homeobox 2; similar t
		Zfhx4	zinc finger homeodomain 4
		Zfp40	zinc finger protein 40
		Zfp445	zinc finger protein 445
		Zfp551	zinc fingr protein 551
		Zfp57	zinc finger protein 57
		Zfp59	zinc finger protein 59; predicted
		Zfp78	zinc finger protein 78
		Zfp788	zinc finger protein 788
		Zfp811	zinc finger protein 811
		Zfp9	zinc finger protein 9
		Zfp941	cDNA sequence BC066028
		Zhx3	zinc fingers and homeoboxes 3
		Zkscan2	zinc finger with KRAB and SCAN

**SoxE11vsCtrlE11**		**SoxE15vsCtrlE15**	
**S11vsC11**	**Fold Change >1.2**	**S15vsC15**	**Fold Change >1.2**
Ascl1	achaete-scute complex homolog	Arnt2	aryl hydrocarbon receptor nucle
Arntl	aryl hydrocarbon receptor nuclea	Arntl	aryl hydrocarbon receptor nucle
Ahr	aryl-hydrocarbon receptor	Ascl1	achaete-scute complex homolog
Atoh1	atonal homolog 1 (Drosophila)	Atoh1	atonal homolog 1 (Drosophila)
Creb3	cAMP responsive element binding	Bach2	BTB and CNC homology 2
Crem	cAMP responsive element modula	Cebpg	CCAAT/enhancer binding protein
Ctnnb1	catenin (cadherin associated prot	Cphx	predicted gene 2135; predicted
Dlx1	distal-less homeobox 1	Crem	cAMP responsive element modu

Dlx2	distal-less homeobox 2	Csrnp2	cysteine-serine-rich nuclear prot
Dlx5	distal-less homeobox 5	Csrnp3	cysteine-serine-rich nuclear prot
Ets1	E26 avian leukemia oncogene 1, 5	Cux2	cut-like homeobox 2
Elf2	E74-like factor 2	Dlx1	distal-less homeobox 1
Ebf3	early B-cell factor 3	Dlx2	distal-less homeobox 2
Etv1	ets variant gene 1	Dlx5	distal-less homeobox 5
Etv5	ets variant gene 5	Dlx6	distal-less homeobox 6
Foxd3	forkhead box D3	Dmrt3	doublesex and mab-3 related tra
Foxn2	forkhead box N2	Dmrta1	doublesex and mab-3 related tra
Foxo1	forkhead box O1	Duxbl	predicted gene 10394; predicted
Gzf1	GDNF-inducible zinc finger protei	E2f5	E2F transcription factor 5
Glis2	GLIS family zinc finger 2	Ebf3	early B-cell factor 3
Hes1	hairy and enhancer of split 1 (Dro	Elf2	E74-like factor 2
Hey2	hairy/enhancer-of-split related w	Etv1	ets variant gene 1
Heyl	hairy/enhancer-of-split related w	Etv5	ets variant gene 5
Hand2	heart and neural crest derivatives	Foxb1	forkhead box B1
Hoxb2	homeo box B2	Foxd3	forkhead box D3
Hoxb4	homeo box B4	Foxn2	forkhead box N2
Hoxb5	homeo box B5	Foxo1	forkhead box O1
Hoxd1	homeo box D1	Foxo3	forkhead box O3
Hoxd3	homeo box D3	Gm239	predicted gene 239
Hivep2	human immunodeficiency virus ty	Hand2	heart and neural crest derivative
Irf2	interferon regulatory factor 2	Hdac2	histone deacetylase 2
Irf6	interferon regulatory factor 6	Hdx	highly divergent homeobox
Jarid2	jumonji, AT rich interactive doma	Hes6	hairy and enhancer of split 6 (Dr
Jun	Jun oncogene	Hey2	hairy/enhancer-of-split related v
Klf7	Kruppel-like factor 7 (ubiquitous)	Hmx2	H6 homeo box 2
Lbh	limb-bud and heart	Hmx3	H6 homeo box 3
Lef1	lymphoid enhancer binding factor	Hoxa1	homeo box A1
Smad1	MAD homolog 1 (Drosophila)	Hoxa2	homeo box A2
Smad2	MAD homolog 2 (Drosophila)	Hoxa3	homeo box A3
Mitf	microphthalmia-associated transc	Hoxa4	homeo box A4
Myt1	myelin transcription factor 1	Hoxa5	homeo box A5
Myt1l	myelin transcription factor 1-like	Hoxb2	homeo box B2
Myc	myelocytomatosis oncogene	Hoxb3	homeo box B3
Mef2c	myocyte enhancer factor 2C	Hoxb4	homeo box B4
Notch1	Notch gene homolog 1 (Drosophi	Hoxb5	homeo box B5
Nr4a1	nuclear receptor subfamily 4, gro	Hoxc4	homeo box C4
Nfyb	nuclear transcription factor-Y bet	Hoxd1	homeo box D1
Onecut2	one cut domain, family member 2	Hoxd3	homeo box D3
Pax3	paired box gene 3	Hsf2	heat shock factor 2
Phox2a	paired-like homeobox 2a	Ilf2	interleukin enhancer binding fac
Phox2b	paired-like homeobox 2b	Jarid2	jumonji, AT rich interactive dom
Pou4f1	POU domain, class 4, transcriptio	Jazf1	JAZF zinc finger 1
Pou4f2	POU domain, class 4, transcriptio	Klf15	Kruppel-like factor 15
Patz1	POZ (BTB) and AT hook containing	Klf7	Kruppel-like factor 7 (ubiquitous

Duxbl	predicted gene 10394; predicted	Lbh	limb-bud and heart
Cphx	predicted gene 2135; predicted g	Lcor	ligand dependent nuclear recept
Gm239	predicted gene 239	Lef1	lymphoid enhancer binding facto
Tfdp1	predicted gene 7390; transcriptio	Lhx1	LIM homeobox protein 1
Preb	prolactin regulatory element binc	Mecp2	methyl CpG binding protein 2
Pa2g4	proliferation-associated 2G4; pre	Mef2c	myocyte enhancer factor 2C
Prox1	prospero-related homeobox 1	Meis3	similar to Myeloid ecotropic vira
Nkap	reproductive homeobox 3B; UPF3	Mitf	microphthalmia-associated trans
Rcor2	REST corepressor 2	Mta1	similar to metastasis-associated
Rara	retinoic acid receptor, alpha	Mycl1	v-myc myelocytomatosis viral or
Rarb	retinoic acid receptor, beta	Mycn	v-myc myelocytomatosis viral re
Creb5	RIKEN cDNA 9430076C15 gene; c/	Myt1	myelin transcription factor 1
Etv2	similar to ETS related protein 71;	Myt1l	myelin transcription factor 1-like
Hdac1	similar to histone deacetylase HD	NeuroD4	neurogenic differentiation 4
Mta1	similar to metastasis-associated p	Nfe2l3	nuclear factor, erythroid derived
Satb1	special AT-rich sequence binding	Nfyb	nuclear transcription factor-Y be
Sox10	SRY-box containing gene 10	Nkap	reproductive homeobox 3B; UPF
Sox4	SRY-box containing gene 19; SRY-	Notch1	Notch gene homolog 1 (Drosoph
Sox2	SRY-box containing gene 2	Nr1d2	nuclear receptor subfamily 1, gr
Sox5	SRY-box containing gene 5	Nr2c1	nuclear receptor subfamily 2, gr
Sox8	SRY-box containing gene 8	Nr2c2	nuclear receptor subfamily 2, gr
St18	suppression of tumorigenicity 18	Nr6a1	nuclear receptor subfamily 6, gr
Tbx2	T-box 2	Otx2	orthodenticle homolog 2 (Droso
Tbx20	T-box 20	Pbx4	pre-B-cell leukemia homeobox 4
Tbx3	T-box 3	Phox2a	paired-like homeobox 2a
Tshz1	teashirt zinc finger family membe	Phox2b	paired-like homeobox 2b
Tcf25	transcription factor 25 (basic heli	Pou2f1	POU domain, class 2, transcripti
Tcf4	transcription factor 4	Pou3f3	POU domain, class 3, transcripti
Tcfap2b	transcription factor AP-2 beta	Pou4f1	POU domain, class 4, transcripti
Tcfap2a	transcription factor AP-2, alpha	Prox1	prospero-related homeobox 1
Mycn	v-myc myelocytomatosis viral rel	Prrxl1	paired related homeobox protei
Rela	v-rel reticuloendotheliosis viral or	Rara	retinoic acid receptor, alpha
Zbtb16	zinc finger and BTB domain conta	Rarb	retinoic acid receptor, beta
Zbtb38	zinc finger and BTB domain conta	Rcor2	REST corepressor 2
Zscan4-ps2	zinc finger and SCAN domain cont	Rere	arginine glutamic acid dipeptide
Zeb2	zinc finger E-box binding homeob	Rhox12	reproductive homeobox 12
Zfhx2	zinc finger homeobox 2; similar to	Sall4	sal-like 4 (Drosophila)
Zfp238	zinc finger protein 238	Satb1	special AT-rich sequence binding
Zfp334	zinc finger protein 334	Satb2	special AT-rich sequence binding
Zfp617	zinc finger protein 617	Smad2	MAD homolog 2 (Drosophila)
Zfp667	zinc finger protein 667	Sox10	SRY-box containing gene 10
Zfp689	zinc finger protein 689	Sox11	SRY-box containing gene 11
Zfp7	zinc finger protein 7	Sox2	SRY-box containing gene 2
Zfp811	zinc finger protein 811	Sox5	SRY-box containing gene 5
Zscan29	zinc finger SCAN domains 29	Sox8	SRY-box containing gene 8
		St18	suppression of tumorigenicity 18

		Taf4a	TAF4A RNA polymerase II, TATA
		Tbx2	T-box 2
		Tbx20	T-box 20
		Tbx3	T-box 3
		Tcf12	transcription factor 12
		Tcf4	transcription factor 4
		Tcfap2a	transcription factor AP-2, alpha
		Tcfap2b	transcription factor AP-2 beta
		Tfam	transcription factor A, mitochon
		Tle4	transducin-like enhancer of split
		Tlx2	T-cell leukemia, homeobox 2
		Tlx3	T-cell leukemia, homeobox 3
		Tshz1	teashirt zinc finger family memb
		Zbtb38	zinc finger and BTB domain cont
		Zfhx2	zinc finger homeobox 2; similar t
		Zfp112	zinc finger protein 112
		Zfp113	zinc finger protein 113
		Zfp184	zinc finger protein 184 (Kruppel-
		Zfp191	zinc finger protein 191
		Zfp238	zinc finger protein 238
		Zfp263	zinc finger protein 263
		Zfp266	RIKEN cDNA 5730601F06 gene
		Zfp28	zinc finger protein 28; predicted
		Zfp37	zinc finger protein 37
		Zfp397	zinc finger protein 397
		Zfp418	zinc finger protein 418
		Zfp426	zinc finger protein 426
		Zfp445	zinc finger protein 445
		Zfp462	zinc finger protein 462
		Zfp566	zinc finger protein 566
		Zfp583	zinc finger protein 583
		Zfp59	zinc finger protein 59; predicted
		Zfp60	similar to Zinc finger protein 60;
		Zfp612	zinc finger protein 612
		Zfp647	zinc finger protein 647
		Zfp667	zinc finger protein 667
		Zfp689	zinc finger protein 689
		Zfp7	zinc finger protein 7
		Zfp763	zinc finger protein 763
		Zfp786	zinc finger protein 786
		Zfp788	zinc finger protein 788
		Zfp825	zinc finger protein 825
		Zfp84	zinc finger protein 84
		Zfp941	cDNA sequence BC066028
		Zfp945	RIKEN cDNA A630033E08 gene
		Zfp946	RIKEN cDNA 1300003B13 gene;

		Zik1	zinc finger protein interacting wi
		Zkscan1	zinc finger with KRAB and SCAN
		Zkscan2	zinc finger with KRAB and SCAN
		Zscan21	zinc finger and SCAN domain co
		Zscan5b	zinc finger and SCAN domain co
		Zxdc	ZXD family zinc finger C
**WntE11vsCtrlE11**		**WntE15vsCtrlE15**	
**W11vsC11**	**Fold Change >1.2**	**W15vsC15**	**Fold Change >1.2**
Ahr	aryl-hydrocarbon receptor	Arnt2	aryl hydrocarbon receptor nucle
Alx1	ALX homeobox 1	Arntl	aryl hydrocarbon receptor nucle
Arid3a	AT rich interactive domain 3A (BR	Ascl1	achaete-scute complex homolog
Arntl	aryl hydrocarbon receptor nuclea	Atf2	activating transcription factor 2;
Ascl1	achaete-scute complex homolog	Atoh1	atonal homolog 1 (Drosophila)
Creb3	cAMP responsive element bindin	Bach2	BTB and CNC homology 2
Creb5	RIKEN cDNA 9430076C15 gene; c	Cebpg	CCAAT/enhancer binding protei
Crem	cAMP responsive element modul	Cphx	predicted gene 2135; predicted
Ctnnb1	catenin (cadherin associated prot	Csrnp2	cysteine-serine-rich nuclear prot
Dlx1	distal-less homeobox 1	Csrnp3	cysteine-serine-rich nuclear prot
Dlx2	distal-less homeobox 2	Cux2	cut-like homeobox 2
Dlx5	distal-less homeobox 5	Dlx1	distal-less homeobox 1
Dlx6	distal-less homeobox 6	Dlx2	distal-less homeobox 2
Dmrta1	doublesex and mab-3 related tran	Dlx3	distal-less homeobox 3
Duxbl	predicted gene 10394; predicted	Dlx5	distal-less homeobox 5
Ebf3	early B-cell factor 3	Dlx6	distal-less homeobox 6
Erf	Ets2 repressor factor	Dmrt3	doublesex and mab-3 related tra
Ets1	E26 avian leukemia oncogene 1, 5	Dmrta1	doublesex and mab-3 related tra
Etv1	ets variant gene 1	Duxbl	predicted gene 10394; predicted
Etv5	ets variant gene 5	Ebf3	early B-cell factor 3
Foxd1	forkhead box D1	Etv1	ets variant gene 1
Foxd3	forkhead box D3	Etv5	ets variant gene 5
Foxn2	forkhead box N2	Foxo1	forkhead box O1
Foxo1	forkhead box O1	Hand2	heart and neural crest derivative
Glis2	GLIS family zinc finger 2	Hdac2	histone deacetylase 2
Gm239	predicted gene 239	Hdx	highly divergent homeobox
Hand2	heart and neural crest derivatives	Hes6	hairy and enhancer of split 6 (Dr
Hes1	hairy and enhancer of split 1 (Dro	Hey2	hairy/enhancer-of-split related
Heyl	hairy/enhancer-of-split related w	Hmx3	H6 homeo box 3
Hivep2	human immunodeficiency virus ty	Hoxa1	homeo box A1
Hoxa1	homeo box A1	Hoxa2	homeo box A2
Hoxb2	homeo box B2	Hoxa3	homeo box A3
Hoxb4	homeo box B4	Hoxa3	homeo box A3
Hoxc11	homeo box C11	Hoxa4	homeo box A4

Hoxd3	homeo box D3	Hoxa5	homeo box A5
Irf6	interferon regulatory factor 6	Hoxb2	homeo box B2
Jarid2	jumonji, AT rich interactive doma	Hoxb3	homeo box B3
Jun	Jun oncogene	Hoxb4	homeo box B4
Klf7	Kruppel-like factor 7 (ubiquitous)	Hoxb5	homeo box B5
Lbh	limb-bud and heart	Hoxb6	homeo box B6
Lef1	lymphoid enhancer binding factor	Hoxc4	homeo box C4
Mef2c	myocyte enhancer factor 2C	Hoxd3	homeo box D3
Meis3	similar to Myeloid ecotropic viral	Hoxd4	homeo box D4
Mitf	microphthalmia-associated transc	Hsf2	heat shock factor 2
Mta1	similar to metastasis-associated p	Ilf2	interleukin enhancer binding fac
Mta2	metastasis-associated gene family	Jarid2	jumonji, AT rich interactive dom
Mxi1	Max interacting protein 1	Jazf1	JAZF zinc finger 1
Myc	myelocytomatosis oncogene	Klf13	Kruppel-like factor 13
Mycn	v-myc myelocytomatosis viral rela	Klf7	Kruppel-like factor 7 (ubiquitous
Myt1	myelin transcription factor 1	Lhx1	LIM homeobox protein 1
Nfe2l1	nuclear factor, erythroid derived :	Mef2c	myocyte enhancer factor 2C
Nkap	reproductive homeobox 3B; UPF3	Meis3	similar to Myeloid ecotropic vira
Notch1	Notch gene homolog 1 (Drosophi	Mxi1	Max interacting protein 1
Nr4a1	nuclear receptor subfamily 4, gro	Mycl1	v-myc myelocytomatosis viral or
Nr4a2	nuclear receptor subfamily 4, gro	Mycn	v-myc myelocytomatosis viral re
Patz1	POZ (BTB) and AT hook containing	Myt1	myelin transcription factor 1
Pax3	paired box gene 3	Myt1l	myelin transcription factor 1-like
Phox2a	paired-like homeobox 2a	NeuroD4	neurogenic differentiation 4
Phox2b	paired-like homeobox 2b	Nfe2l3	nuclear factor, erythroid derived
Pou3f2	POU domain, class 3, transcriptio	Nkap	reproductive homeobox 3B; UPF
Pou4f1	POU domain, class 4, transcriptio	Nr1d2	nuclear receptor subfamily 1, gr
Pou4f2	POU domain, class 4, transcriptio	Nr2c1	nuclear receptor subfamily 2, gr
Preb	prolactin regulatory element binc	Nr2c2	nuclear receptor subfamily 2, gr
Prox1	prospero-related homeobox 1	Nr6a1	nuclear receptor subfamily 6, gr
Prrx1	paired related homeobox 1	Onecut2	one cut domain, family member
Rara	retinoic acid receptor, alpha	Patz1	POZ (BTB) and AT hook containin
Rarb	retinoic acid receptor, beta	Pbx3	similar to PBX3a; pre B-cell leuke
Rela	v-rel reticuloendotheliosis viral or	Pbx4	pre-B-cell leukemia homeobox 4
Satb1	special AT-rich sequence binding	Pgr	progesterone receptor
Satb2	special AT-rich sequence binding	Phox2a	paired-like homeobox 2a
Shox2	short stature homeobox 2	Phox2b	paired-like homeobox 2b
Smad1	MAD homolog 1 (Drosophila)	Pou2f1	POU domain, class 2, transcripti
Smad2	MAD homolog 2 (Drosophila)	Pou3f3	POU domain, class 3, transcripti
Sox10	SRY-box containing gene 10	Prrxl1	paired related homeobox protei
Sox2	SRY-box containing gene 2	Rarb	retinoic acid receptor, beta
Sox4	SRY-box containing gene 19; SRY-	Sall4	sal-like 4 (Drosophila)
Sox5	SRY-box containing gene 5	Satb1	special AT-rich sequence binding
Sox8	SRY-box containing gene 8	Satb2	special AT-rich sequence binding
St18	suppression of tumorigenicity 18	Smad2	MAD homolog 2 (Drosophila)
Stat3	similar to Stat3B; signal transduce	Sox10	SRY-box containing gene 10

Tbx2	T-box 2	Sox11	SRY-box containing gene 11
Tbx20	T-box 20	Sox2	SRY-box containing gene 2
Tbx3	T-box 3	Sox4	SRY-box containing gene 19; SRY
Tcf25	transcription factor 25 (basic heli:	Sox5	SRY-box containing gene 5
Tcf4	transcription factor 4	Sox8	SRY-box containing gene 8
Tcfap2a	transcription factor AP-2, alpha	St18	suppression of tumorigenicity 18
Tcfap2b	transcription factor AP-2 beta	Tbr1	T-box brain gene 1
Tcfap2c	transcription factor AP-2, gamma	Tbx2	T-box 2
Tfdp1	predicted gene 7390; transcriptio	Tbx20	T-box 20
Tshz1	teashirt zinc finger family membe	Tbx3	T-box 3
Zbtb16	zinc finger and BTB domain conta	Tcf4	transcription factor 4
Zbtb38	zinc finger and BTB domain conta	Tcfap2a	transcription factor AP-2, alpha
Zeb2	zinc finger E-box binding homeob	Tlx2	T-cell leukemia, homeobox 2
Zfhx2	zinc finger homeobox 2; similar tc	Tshz1	teashirt zinc finger family memb
Zfp191	zinc finger protein 191	Zbtb38	zinc finger and BTB domain cont
Zfp238	zinc finger protein 238	Zfhx2	zinc finger homeobox 2; similar t
Zfp28	zinc finger protein 28; predicted g	Zfhx4	zinc finger homeodomain 4
Zfp334	zinc finger protein 334	Zfp112	zinc finger protein 112
Zfp617	zinc finger protein 617	Zfp113	zinc finger protein 113
Zfp667	zinc finger protein 667	Zfp169	zinc finger protein 169
Zfp689	zinc finger protein 689	Zfp191	zinc finger protein 191
Zfp7	zinc finger protein 7	Zfp238	zinc finger protein 238
Zfp763	zinc finger protein 763	Zfp26	zinc finger protein 26
Zfp811	zinc finger protein 811	Zfp28	zinc finger protein 28; predicted
Zfp84	zinc finger protein 84	Zfp30	zinc finger protein 30
Zfp948	cDNA sequence BC049807	Zfp317	zinc finger protein 317
Zkscan1	zinc finger with KRAB and SCAN d	Zfp37	zinc finger protein 37
		Zfp397	zinc finger protein 397
		Zfp397os	zinc finger protein 397 opposite
		Zfp40	zinc finger protein 40
		Zfp418	zinc finger protein 418
		Zfp426	zinc finger protein 426
		Zfp445	zinc finger protein 445
		Zfp462	zinc finger protein 462
		Zfp558	zinc finger protein 558
		Zfp566	zinc finger protein 566
		Zfp57	zinc finger protein 57
		Zfp583	zinc finger protein 583
		Zfp59	zinc finger protein 59; predicted
		Zfp60	similar to Zinc finger protein 60;
		Zfp612	zinc finger protein 612
		Zfp617	zinc finger protein 617
		Zfp647	zinc finger protein 647
		Zfp667	zinc finger protein 667
		Zfp689	zinc finger protein 689
		Zfp7	zinc finger protein 7

		Zfp763	zinc finger protein 763
		Zfp78	zinc finger protein 78
		Zfp786	zinc finger protein 786
		Zfp788	zinc finger protein 788
		Zfp84	zinc finger protein 84
		Zfp810	zinc finger protein 810
		Zfp811	zinc finger protein 811
		Zfp94	zinc finger protein 94
		Zfp945	RIKEN cDNA A630033E08 gene
		ZFP946	RIKEN cDNA 1300003B13 gene;
		Zfp961	cDNA sequence BC049349
		Zik1	zinc finger protein interacting wi
		Zkscan1	zinc finger with KRAB and SCAN
		Zkscan2	zinc finger with KRAB and SCAN
		Zmiz1	RIKEN cDNA D930049A15 gene;
		Zscan21	zinc finger and SCAN domain cor
		Zxda	zinc finger, X-linked, duplicated
**Ctrl11vsWnt11**		**Ctr15vsWnt15**	
**C11vsW11**	**Fold Change >1.3**	**C15vsW15**	**Fold Change >1.3**
5730507C01	RIKEN cDNA 5730507C01 gene; p	Aff3	AF4/FMR2 family, member 3; sir
Barx1	BarH-like homeobox 1	Ahr	aryl-hydrocarbon receptor
Bcl6b	B-cell CLL/lymphoma 6, member	Arap1	ArfGAP with RhoGAP domain, ar
Bhlhe41	basic helix-loop-helix family, mem	Arnt	aryl hydrocarbon receptor nucle
Creb3l1	cAMP responsive element binding	Atf1	predicted gene 1862; activating
Creb3l2	cAMP responsive element binding	Atf3	activating transcription factor 3
Cux2	cut-like homeobox 2	Atf7	activating transcription factor 7
Dmrt2	doublesex and mab-3 related trar	Barx1	BarH-like homeobox 1
Ebf1	early B-cell factor 1	Bcl10	B-cell leukemia/lymphoma 10; p
Epas1	endothelial PAS domain protein 1	Bcl6	B-cell leukemia/lymphoma 6
Erg	avian erythroblastosis virus E-26 (	Bcl6b	B-cell CLL/lymphoma 6, member
Esrrg	estrogen-related receptor gamma	Bhlhe40	basic helix-loop-helix family, me
Ets2	E26 avian leukemia oncogene 2, 3	Bhlhe41	basic helix-loop-helix family, me
Fli1	Friend leukemia integration 1	Cdx2	caudal type homeo box 2
Foxc2	forkhead box C2	Cebpa	CCAAT/enhancer binding protein
Foxf1a	forkhead box F1a	Cebpb	CCAAT/enhancer binding protein
Foxf2	forkhead box F2	Cebpd	CCAAT/enhancer binding protein
Foxp1	forkhead box P1	Creb3l1	cAMP responsive element bindi
Foxp2	forkhead box P2	Creb3l2	cAMP responsive element bindi
Foxp4	forkhead box P4	Csda	cold shock domain protein A
Gata1	GATA binding protein 1	Csrnp1	cysteine-serine-rich nuclear prot
Gata4	GATA binding protein 4	E2f7	E2F transcription factor 7
Gata6	GATA binding protein 6	E2f8	E2F transcription factor 8

Gli1	GLI-Kruppel family member GLI1	Ebf1	early B-cell factor 1
Gli2	GLI-Kruppel family member GLI2	Ebf2	early B-cell factor 2
Gm98	predicted gene 98	Egr1	early growth response 1
Hand1	heart and neural crest derivatives	Egr2	early growth response 2
Hhex	hematopoietically expressed hom	Ehf	ets homologous factor
Hif3a	hypoxia inducible factor 3, alpha s	Elf1	E74-like factor 1
Hlf	hepatic leukemia factor	Elk3	ELK3, member of ETS oncogene
Hlx	H2.0-like homeobox	Elk4	ELK4, member of ETS oncogene
Hoxa10	homeo box A10	Epas1	endothelial PAS domain protein
Hoxa11	homeo box A11	Erg	avian erythroblastosis virus E-26
Hoxa4	homeo box A4	Ets1	E26 avian leukemia oncogene 1,
Hoxa5	homeo box A5	Ets2	E26 avian leukemia oncogene 2,
Hoxa6	homeo box A6	Etv6	ets variant gene 6 (TEL oncogene
Hoxa9	homeo box A9	Fli1	Friend leukemia integration 1
Hoxb6	homeo box B6	Fos	FBJ osteosarcoma oncogene
Hoxb8	homeo box B8	Fosb	FBJ osteosarcoma oncogene B
Hoxc10	homeo box C10	Fosl2	similar to fos-like antigen 2; fos-
Hoxc4	homeo box C4	Foxa3	forkhead box A3
Hoxc5	homeo box C5	Foxc2	forkhead box C2
Hoxc6	homeo box C6	Foxf1a	forkhead box F1a
Hoxc8	homeo box C8	Foxf2	forkhead box F2
Hoxc9	homeo box C9	Foxm1	forkhead box M1; RIKEN cDNA 4
Hoxd10	homeo box D10	Foxp1	forkhead box P1
Hoxd11	homeo box D11	Foxp2	forkhead box P2
Hoxd4	homeo box D4	Foxp4	forkhead box P4
Hoxd8	homeo box D8	Gata3	GATA binding protein 3
Hoxd9	homeo box D9	Gata6	GATA binding protein 6
Ikzf1	IKAROS family zinc finger 1	Gli1	GLI-Kruppel family member GLI1
Isl1	ISL1 transcription factor, LIM/hon	Gli2	GLI-Kruppel family member GLI2
Klf1	Kruppel-like factor 1 (erythroid)	Gm98	predicted gene 98
Klf12	Kruppel-like factor 12	Grhl2	grainyhead-like 2 (Drosophila)
Lhx9	LIM homeobox protein 9	Hand1	heart and neural crest derivative
Meis1	Meis homeobox 1	Hes1	hairy and enhancer of split 1 (Dr
Meis2	Meis homeobox 2	Hhex	hematopoietically expressed hor
Nfatc4	nuclear factor of activated T-cells	Hif3a	hypoxia inducible factor 3, alpha
Nfe2	nuclear factor, erythroid derived	Hlx	H2.0-like homeobox
Nfe2l2	nuclear factor, erythroid derived	Hnf4g	hepatocyte nuclear factor 4, gan
Nfia	nuclear factor I/A	Hoxa11	homeo box A11
Nfib	nuclear factor I/B	Hoxa9	homeo box A9
Nkx2-3	NK2 transcription factor related, l	Hoxc10	homeo box C10
Nkx6-1	NK6 homeobox 1	Hoxc6	homeo box C6
Npas3	neuronal PAS domain protein 3	Hoxc8	homeo box C8
Nr1h5	nuclear receptor subfamily 1, gro	Hoxc9	homeo box C9
Nr2f2	similar to COUP-TFI; nuclear rece	Hoxd10	homeo box D10
Nr3c1	nuclear receptor subfamily 3, gro	Hoxd11	homeo box D11
Nr5a2	nuclear receptor subfamily 5, gro	Hoxd8	homeo box D8

Pbx1	pre B-cell leukemia transcription f	Hoxd9	homeo box D9
Pitx2	paired-like homeodomain transcr	Ikzf1	IKAROS family zinc finger 1
Plagl1	pleiomorphic adenoma gene-like	Irf1	interferon regulatory factor 1
Rest	RE1-silencing transcription factor	Isl1	ISL1 transcription factor, LIM/ho
Runx1	runt related transcription factor 1	Isx	intestine specific homeobox
Runx1t1	runt-related transcription factor 1	Jdp2	Jun dimerization protein 2
Sfpi1	SFFV proviral integration 1	Junb	Jun-B oncogene
Six2	similar to Homeobox protein SIX2	Klf12	Kruppel-like factor 12
Smad5	MAD homolog 5 (Drosophila)	Klf2	Kruppel-like factor 2 (lung)
Smad7	MAD homolog 7 (Drosophila)	Klf4	Kruppel-like factor 4 (gut)
Tbx18	T-box18	Klf5	Kruppel-like factor 5
Tbx5	T-box 5	Maf	similar to c-Maf long form; aviar
Tcf21	transcription factor 21	Maff	v-maf musculoaponeurotic fibro
Tead3	TEA domain family member 3	Mef2a	similar to Myocyte enhancer fac
Thra	thyroid hormone receptor alpha;	Meis2	Meis homeobox 2
Trps1	trichorhinophalangeal syndrome	Meox2	mesenchyme homeobox 2
Tshz2	teashirt zinc finger family membe	Mkx	mohawk homeobox
Tshz3	teashirt zinc finger family membe	Neurod1	neurogenic differentiation 1; ne
Twist2	twist homolog 2 (Drosophila)	Neurog3	neurogenin 3
Wt1	similar to Wilms tumor homolog;	Nfat5	nuclear factor of activated T-cell
Zfhx4	zinc finger homeodomain 4	Nfatc1	nuclear factor of activated T-cell
Zfp558	zinc finger protein 558	Nfatc3	nuclear factor of activated T-cell
Zim1	zinc finger, imprinted 1	Nfatc4	nuclear factor of activated T-cell
Znfng936	RIKEN cDNA I1C0022H11 gene; si	Nfe2l2	nuclear factor, erythroid derived
		Nfia	nuclear factor I/A
		Nfib	nuclear factor I/B
		Nfic	nuclear factor I/C
		Nfix	nuclear factor I/X
		Nfkb1	nuclear factor of kappa light pol
		Nkx2-3	NK2 transcription factor related,
		Nkx6-1	NK6 homeobox 1
		Npas3	neuronal PAS domain protein 3
		Nr1h4	nuclear receptor subfamily 1, gr
		Nr2f1	nuclear receptor subfamily 2, gr
		Nr2f2	similar to COUP-TFI; nuclear rece
		Nr3c1	nuclear receptor subfamily 3, gr
		Nr4a1	nuclear receptor subfamily 4, gr
		Nr5a2	nuclear receptor subfamily 5, gr
		Pbx1	pre B-cell leukemia transcription
		Per1	period homolog 1 (Drosophila)
		Pitx1	paired-like homeodomain transc
		Pitx2	paired-like homeodomain transc
		Pknox2	Pbx/knotted 1 homeobox 2
		Plagl1	pleiomorphic adenoma gene-like
		Pparg	peroxisome proliferator activate
		Preb	prolactin regulatory element bin

		Prrx1	paired related homeobox 1
		Purb	purine rich element binding prot
		Rbpj	recombination signal binding pro
		Rel	reticuloendotheliosis oncogene
		Rest	RE1-silencing transcription facto
		Rex2	hypothetical protein LOC100048
		Rora	RAR-related orphan receptor alp
		Rorc	RAR-related orphan receptor ga
		Runx1	runt related transcription factor
		Runx1t1	runt-related transcription factor
		Runx3	runt related transcription factor
		Rxra	retinoid X receptor alpha; simila
		Sfpi1	SFFV proviral integration 1
		Six2	similar to Homeobox protein SIX
		Six5	sine oculis-related homeobox 5
		Smad3	MAD homolog 3 (Drosophila)
		Smad7	MAD homolog 7 (Drosophila)
		Sox18	SRY-box containing gene 18
		Sox9	SRY-box containing gene 9
		Sp1	trans-acting transcription factor
		Spic	Spi-C transcription factor (Spi-1/
		Srf	serum response factor
		Stat1	signal transducer and activator o
		Stat4	signal transducer and activator o
		Stat6	signal transducer and activator o
		Tcf21	transcription factor 21
		Tcf3	transcription factor E2a
		Tcf7	transcription factor 7, T-cell spe
		Tead1	TEA domain family member 1
		Tead3	TEA domain family member 3
		Tgif1	TGFB-induced factor homeobox
		Trp53	transformation related protein 5
		Trps1	trichorhinophalangeal syndrome
		Tshz2	teashirt zinc finger family memb
		Twist1	twist homolog 1 (Drosophila)
		Twist2	twist homolog 2 (Drosophila)
		Wt1	similar to Wilms tumor homolog
		Xbp1	X-box binding protein 1
		Zbtb7b	similar to Zinc finger and BTB do
		Zfp449	zinc finger protein 449
		Zfp568	zinc finger protein 568
		Zfp595	zinc finger protein 595
		Zfp607	zinc finger proten 607
		Zfp69	zinc finger protein 69
		Zfp809	zinc finger protein 809
		Zfp820	hypothetical protein LOC100044

		Zim1	zinc finger, imprinted 1
**Ctrl11vsSox11**		**Ctrl15vsSox15**	
**C11vsS11**	**Fold Change >1.3**	**C15vsS15**	**Fold Change >1.3**
5730507C01	RIKEN cDNA 5730507C01 gene; p	Ahr	aryl-hydrocarbon receptor
Barx1	BarH-like homeobox 1	Arap1	ArfGAP with RhoGAP domain, ar
Bhlhe41	basic helix-loop-helix family, mem	Atf3	activating transcription factor 3
Creb3l2	cAMP responsive element bindin	Atf7	activating transcription factor 7
Cux2	cut-like homeobox 2	Barx1	BarH-like homeobox 1
Ebf1	early B-cell factor 1	Bcl6b	B-cell CLL/lymphoma 6, membe
Epas1	endothelial PAS domain protein 1	Bhlhe40	basic helix-loop-helix family, me
Erg	avian erythroblastosis virus E-26 (	Bhlhe41	basic helix-loop-helix family, me
Esrrg	estrogen-related receptor gamma	Cdx2	caudal type homeo box 2
Ets2	E26 avian leukemia oncogene 2, 3	Cebpb	CCAAT/enhancer binding protei
Fli1	Friend leukemia integration 1	Creb3l1	cAMP responsive element bindi
Foxc1	forkhead box C1	Creb3l2	cAMP responsive element bindi
Foxc2	forkhead box C2	Csrnp1	cysteine-serine-rich nuclear prot
Foxd1	forkhead box D1	E2f8	E2F transcription factor 8
Foxf1a	forkhead box F1a	Ebf1	early B-cell factor 1
Foxf2	forkhead box F2	Elf1	E74-like factor 1
Foxp1	forkhead box P1	Elk3	ELK3, member of ETS oncogene
Foxp2	forkhead box P2	Epas1	endothelial PAS domain protein
Foxp4	forkhead box P4	Erg	avian erythroblastosis virus E-26
Foxq1	forkhead box Q1	Ets2	E26 avian leukemia oncogene 2,
Gata1	GATA binding protein 1	Etv6	ets variant gene 6 (TEL oncogen
Gata4	GATA binding protein 4	Fli1	Friend leukemia integration 1
Gata5	GATA binding protein 5	Fosl2	similar to fos-like antigen 2; fos-
Gata6	GATA binding protein 6	Foxa1	forkhead box A1; similar to Hepa
Gli1	GLI-Kruppel family member GLI1	Foxa3	forkhead box A3
Gli2	GLI-Kruppel family member GLI2	Foxc2	forkhead box C2
Gm98	predicted gene 98	Foxf1a	forkhead box F1a
Hand1	heart and neural crest derivatives	Foxf2	forkhead box F2
Hhex	hematopoietically expressed hom	Foxp1	forkhead box P1
Hif3a	hypoxia inducible factor 3, alpha s	Foxp2	forkhead box P2
Hlf	hepatic leukemia factor	Foxp4	forkhead box P4
Hlx	H2.0-like homeobox	Gata3	GATA binding protein 3
Hoxa11	homeo box A11	Gata6	GATA binding protein 6
Hoxa4	homeo box A4	Gli1	GLI-Kruppel family member GLI1
Hoxa5	homeo box A5	Gli2	GLI-Kruppel family member GLI2
Hoxa6	homeo box A6	Gm98	predicted gene 98
Hoxa7	homeo box A7	Grhl2	grainyhead-like 2 (Drosophila)
Hoxa9	homeo box A9	Hand1	heart and neural crest derivative
Hoxb6	homeo box B6	Hhex	hematopoietically expressed ho
Hoxb8	homeo box B8	Hif3a	hypoxia inducible factor 3, alpha
Hoxc10	homeo box C10	Hlx	H2.0-like homeobox

Hoxc4	homeo box C4	Hnf4g	hepatocyte nuclear factor 4, gan
Hoxc5	homeo box C5	Hoxa11	homeo box A11
Hoxc6	homeo box C6	Hoxa9	homeo box A9
Hoxc8	homeo box C8	Hoxc10	homeo box C10
Hoxc9	homeo box C9	Hoxc6	homeo box C6
Hoxd10	homeo box D10	Hoxc8	homeo box C8
Hoxd11	homeo box D11	Hoxc9	homeo box C9
Hoxd8	homeo box D8	Hoxd10	homeo box D10
Hoxd9	homeo box D9	Hoxd11	homeo box D11
Ikzf1	IKAROS family zinc finger 1	Hoxd8	homeo box D8
Isl1	ISL1 transcription factor, LIM/hon	Hoxd9	homeo box D9
Klf1	Kruppel-like factor 1 (erythroid)	Ikzf1	IKAROS family zinc finger 1
Klf12	Kruppel-like factor 12	Irf1	interferon regulatory factor 1
Klf2	Kruppel-like factor 2 (lung)	Isl1	ISL1 transcription factor, LIM/ho
Lhx9	LIM homeobox protein 9	Isx	intestine specific homeobox
Maf	similar to c-Maf long form; avian	Jdp2	Jun dimerization protein 2
Mafb	v-maf musculoaponeurotic fibros	Klf12	Kruppel-like factor 12
Meis1	Meis homeobox 1	Klf5	Kruppel-like factor 5
Meis2	Meis homeobox 2	Maf	similar to c-Maf long form; avian
Meox1	mesenchyme homeobox 1	Mafb	v-maf musculoaponeurotic fibro
Meox2	mesenchyme homeobox 2	Maff	v-maf musculoaponeurotic fibro
Myb	myeloblastosis oncogene	Mef2a	similar to Myocyte enhancer fac
Nfatc4	nuclear factor of activated T-cells	Meis1	Meis homeobox 1
Nfe2	nuclear factor, erythroid derived	Meis2	Meis homeobox 2
Nfia	nuclear factor I/A	Meox2	mesenchyme homeobox 2
Nfib	nuclear factor I/B	Mkx	mohawk homeobox
Nfix	nuclear factor I/X	Neurod1	neurogenic differentiation 1; ne
Nkx6-1	NK6 homeobox 1	Neurog3	neurogenin 3
Nr1h5	nuclear receptor subfamily 1, gro	Nfat5	nuclear factor of activated T-cell
Nr2f1	nuclear receptor subfamily 2, gro	Nfatc4	nuclear factor of activated T-cell
Nr2f2	similar to COUP-TFI; nuclear rece	Nfe2l2	nuclear factor, erythroid derived
Pbx1	pre B-cell leukemia transcription	Nfia	nuclear factor I/A
Pbx3	similar to PBX3a; pre B-cell leuker	Nfib	nuclear factor I/B
Pitx2	paired-like homeodomain transcr	Nfic	nuclear factor I/C
Plagl1	pleiomorphic adenoma gene-like	Nfix	nuclear factor I/X
Prrx1	paired related homeobox 1	Nfkb1	nuclear factor of kappa light pol
Rhox6	reproductive homeobox 6	Nkx2-3	NK2 transcription factor related,
Runx1	runt related transcription factor 1	Nkx6-1	NK6 homeobox 1
Runx1t1	runt-related transcription factor 1	Nr1h4	nuclear receptor subfamily 1, gr
Sfpi1	SFFV proviral integration 1	Nr2f1	nuclear receptor subfamily 2, gr
Six2	similar to Homeobox protein SIX2	Nr2f2	similar to COUP-TFI; nuclear rece
Smad5	MAD homolog 5 (Drosophila)	Nr3c1	nuclear receptor subfamily 3, gr
Smad7	MAD homolog 7 (Drosophila)	Nr4a1	nuclear receptor subfamily 4, gr
Tbx4	T-box 4	Nr5a2	nuclear receptor subfamily 5, gr
Tbx5	T-box 5	Pbx1	pre B-cell leukemia transcription
Tcf21	transcription factor 21	Per1	period homolog 1 (Drosophila)

Tead3	TEA domain family member 3	Pitx1	paired-like homeodomain transc
Tfdp2	transcription factor Dp 2	Pitx2	paired-like homeodomain transc
Trps1	trichorhinophalangeal syndrome	Plagl1	pleiomorphic adenoma gene-like
Tshz2	teashirt zinc finger family membe	Pparg	peroxisome proliferator activate
Tshz3	teashirt zinc finger family membe	Prrx1	paired related homeobox 1
Twist1	twist homolog 1 (Drosophila)	Purb	purine rich element binding prot
Twist2	twist homolog 2 (Drosophila)	Rel	reticuloendotheliosis oncogene
Wt1	similar to Wilms tumor homolog;	Rest	RE1-silencing transcription facto
Zfhx3	zinc finger homeobox 3	Rora	RAR-related orphan receptor alp
Zfhx4	zinc finger homeodomain 4	Rorc	RAR-related orphan receptor ga
Zfp57	zinc finger protein 57	Rsl1	regulator of sex limited protein 1
Zfp936	RIKEN cDNA I1C0022H11 gene; si	Runx1	runt related transcription factor
Zim1	zinc finger, imprinted 1	Runx1t1	runt-related transcription factor
		Runx3	runt related transcription factor
		Sfpi1	SFFV proviral integration 1
		Six2	similar to Homeobox protein SIX
		Smad7	MAD homolog 7 (Drosophila)
		Sox18	SRY-box containing gene 18
		Sp1	trans-acting transcription factor
		Spic	Spi-C transcription factor (Spi-1/
		Srf	serum response factor
		Stat1	signal transducer and activator c
		Stat4	signal transducer and activator c
		Stat5b	signal transducer and activator c
		Stat6	signal transducer and activator c
		Tbx5	T-box 5
		Tcf21	transcription factor 21
		Tcf3	transcription factor E2a
		Tcf7	transcription factor 7, T-cell spec
		Tead1	TEA domain family member 1
		Tead3	TEA domain family member 3
		Thra	thyroid hormone receptor alpha
		Trps1	trichorhinophalangeal syndrome
		Tshz2	teashirt zinc finger family memb
		Twist1	twist homolog 1 (Drosophila)
		Twist2	twist homolog 2 (Drosophila)
		Wt1	similar to Wilms tumor homolog
		Xbp1	X-box binding protein 1
		Zbtb7b	similar to Zinc finger and BTB do
		Zfp449	zinc finger protein 449
		Zfp52	hypothetical protein LOC100044
		Zfp568	zinc finger protein 568
		Zfp595	zinc finger protein 595
		Zfp607	zinc finger proten 607
		Zfp809	zinc finger protein 809
		Zfp9	zinc finger protein 9

			Zim1	zinc finger, imprinted 1
