# Supplementary material for: Transcription and Signaling Regulators in Developing Neuronal Subtypes of Mouse and Human Enteric Nervous System
Source: Gastroenterology. 2018 Feb;154(3):624–36. doi: 10.1053/j.gastro.2017.10.005 (PMC6381388; doi:10.1053/j.gastro.2017.10.005)
Supplement: Supplementary Table 4 [file mmc14.pdf]

**Supplementary Table 4: Signaling ligands enriched (>1.2) in pairwise comparisons between cell populations in the developing gut.**

Signaling factors were identified from the terms *growth factor (Panther* MF00019) and *secreted* (SP_PIR_KEYWORDS). Semaphorins and Slit proteins were identified manually and added to the lists.

**SoxE11 vs WntE11**

| S11vsW11 | Fold Change >1.2 |
|---|---|
| Sema3c | semaphorin 3C |

**SoxE11 vs SoxE15**

| S11vsS15 | Fold Change >1.2 |
|---|---|
| D17Wsu104 | DNA segment, Chr 17, Wayne St |
| Efnb2 | ephrin B2 |
| Gdf10 | growth differentiation factor 10 |
| Igf1 | insulin-like growth factor 1 |
| Mdk | midkine |
| Mstn | myostatin |
| Pdgfc | platelet-derived growth factor, ( |
| Sema3d | semaphorin 3D |

**WntE11 vs SoxE11**

| W11vsS11 | Fold Change >1.2 |
|---|---|
| Gdf10 | growth differentiation factor 10 |

**WntE11 vs WntE15**

| W11vsW15 | Fold Change >1.2 |
|---|---|
| Bmp1 | bone morphogenetic protein 1 |
| Cxcl12 | chemokine (C-X-C motif) ligand |
| Dll3 | delta-like 3 (Drosophila) |
| Efna4 | ephrin A4 |
| Efnb2 | ephrin B2 |
| Gdf10 | growth differentiation factor 10 |
| Gdf11 | growth differentiation factor 11 |
| Igf1 | insulin-like growth factor 1 |
| Jag1 | jagged 1 |
| Mdk | midkine |
| Mia1 | melanoma inhibitory activity 1 |
| Mstn | myostatin |
| Pdgfc | platelet-derived growth factor, ( |
| Sema3d | semaphorin 3D |
| Wnt2b | wingless related MMTV integrat |
| Wnt5a | wingless-related MMTV integrat |

**SoxE15 vs WntE15**

| S15vsW15 | Fold Change >1.2 |
|---|---|
| Ctgf | connective tissue growth factor |
| Dll3 | delta-like 3 (Drosophila) |
| Fgf7 | fibroblast growth factor 7 |
| Jag1 | jagged 1 |
| Mdk | midkine |
| Tgfb2 | transforming growth factor, beta |

**SoxE15 vs SoxE11**

| S15vsS11 | Fold Change >1.2 |
|---|---|
| Ctgf | connective tissue growth factor |
| Efna5 | ephrin A5 |
| Fgf7 | fibroblast growth factor 7 |
| Fgf12 | fibroblast growth factor 12 |
| Fgf13 | fibroblast growth factor 13 |
| Fgf14 | fibroblast growth factor 14 |
| Fgf2 | fibroblast growth factor 2 |

| | |
|---|---|
| Hdgfrp3 | hepatoma-derived growth facto |
| Inha | inhibin alpha |
| Nrg1 | neuregulin 1 |
| Nrg3 | neuregulin 3 |
| Ntng1 | netrin G1 |
| Ogn | osteoglycin |
| Pdgfa | platelet derived growth factor, a |
| Pdgfb | similar to platelet-derived growt |
| Pdgfd | platelet-derived growth factor, |
| Pgf | placental growth factor |
| Ptn | pleiotrophin |
| Sema3c | semaphorin 3C |
| Sema3e | semaphorin 3E |
| Sema4d | semaphorin 4D |
| Sema4g | semaphorin 4G |
| Sema6a | semaphorin 6A |
| Sema6d | semaphorin 6D |
| Slit1 | slit homolog 1 |
| Slit2 | slit homolog 2 |
| Tgfb2 | transforming growth factor, bet |

**WntE15 vs SoxE15**

| W15vsS15 | Fold Change >1.2 |
|---|---|
| Efnb3 | ephrin B3 |
| Nrg1 | neuregulin 1 |
| Nrg3 | neuregulin 3 |
| Ntng1 | netrin G1 |
| Sema3d | Semaphorin 3D |
| Sema4g | Semaphorin 4G |
| Sema6a | Semaphorin 6A |
| Sema6d | Semaphorin 6D |
| Slit2 | Slit homolog 2 |

**WntE15 vs WntE11**

| W15vsW11 | Fold Change >1.2 |
|---|---|
| Ctgf | connective tissue growth factor |
| Efna5 | ephrin A5 |
| Efnb3 | ephrin B3 |
| Fgf1 | fibroblast growth factor 1 |
| Fgf11 | fibroblast growth factor 11 |
| Fgf12 | fibroblast growth factor 12 |
| Fgf13 | fibroblast growth factor 13 |
| Fgf14 | fibroblast growth factor 14 |
| Fgf9 | fibroblast growth factor 9 |
| Gdf5 | growth differentiation factor 5 |
| Hdgfrp3 | hepatoma-derived growth facto |
| Inha | inhibin alpha |
| Nrg1 | neuregulin 1 |
| Nrg3 | neuregulin 3 |
| Ntng1 | netrin G1 |
| Pdgfa | platelet derived growth factor, a |
| Ptn | pleiotrophin |
| Sema3c | semaphorin 3C |
| Sema3e | semaphorin 3E |
| Sema4d | semaphorin 4D |
| Sema4f | semaphorin 4F |
| Sema4g | semaphorin 4G |
| Sema6a | semaphorin 6A |

| | | | |
|---|---|---|---|
| | | Sema6d | semaphorin 6D |
| | | Slit1 | slit homolog 1 |
| | | Slit2 | slit homolog 2 |
| | | | |
| **SoxE11 vs CtrlE11** | | **SoxE15 vs CtrlE15** | |
| **S11vsC11** | **Fold Change >1.2** | **S15vsC15** | **Fold Change >1.2** |
| Bmp7 | bone morphogenetic protein 7 | Bmp15 | bone morphogenetic protein 15 |
| Dll1 | delta-like 1 | Bmp7 | bone morphogenetic protein 7 |
| Dll3 | delta-like 3 | Ctgf | connective tissue growth factor |
| Fgf13 | fibroblast growth factor 13 | Dhh | desert hedgehog |
| Fgf7 | fibroblast growth factor 7 | Dll1 | delta-like 1 (Drosophila) |
| Gpi1 | glucose phosphate isomerase 1 | Dll3 | delta-like 3 (Drosophila) |
| Nrg3 | neuregulin 3 | Fgf12 | fibroblast growth factor 12 |
| Rabep1 | rabaptin, RAB GTPase binding eff | Fgf13 | fibroblast growth factor 13 |
| Sema3b | semaphorin 3B | Gdf3 | growth differentiation factor 3 |
| Sema3c | semaphorin 3C | Gpi1 | glucose phosphate isomerase 1 |
| Sema3d | semaphorin 3D | Nrg3 | neuregulin 3 |
| Sema4d | semaphorin 4D | Ntn4 | netrin 4 |
| Slit3 | slit homolog 3 | Ntng1 | netrin G1 |
| Tgfb2 | transforming growth factor, beta | Pdgfb | similar to platelet-derived grow |
| | | Rabep1 | rabaptin, RAB GTPase binding ef |
| | | Sema3b | semaphorin 3B |
| | | Sema3c | semaphorin 3C |
| | | Sema4d | semaphorin 4D |
| | | Sema4f | semaphorin 4F |
| | | Slit1 | slit homolog 1 |
| | | Slit3 | slit homolog 2 |
| | | Tgfb2 | transforming growth factor, bet |
| | | | |
| **WntE11 vs CtrlE11** | | **WntE15 vs CtrlE15** | |
| **W11vsC11** | **Fold Change >1.2** | **W15vsC15** | **Fold Change >1.2** |
| Bmp7 | bone morphogenetic protein 7 | Bmp7 | bone morphogenetic protein 7 |
| Dll1 | delta-like 1 (Drosophila) | Dll1 | delta-like 1 (Drosophila) |
| Dll3 | delta-like 3 (Drosophila) | Dll3 | delta-like 3 (Drosophila) |
| Fgf13 | fibroblast growth factor 13 | Efnab3 | ephrin B3 |
| Fgf7 | fibroblast growth factor 7 | Efnb3 | ephrin B3 |
| Gdf10 | growth differentiation factor 10 | Fgf13 | fibroblast growth factor 13 |
| Gpi1 | glucose phosphate isomerase 1 | Fgf14 | fibroblast growth factor 14 |
| Mstn | myostatin | Gpi1 | glucose phosphate isomerase 1 |
| Sema3b | semaphorin 3B | Hdgfrp3 | hepatoma-derived growth facto |
| Sema3d | semaphorin 3D | Inha | inhibin alpha |
| Slit3 | slit homolog 3 | Jag1 | jagged 1 |
| Tgfb2 | transforming growth factor, beta | Jag2 | jagged 2 |
| | | Nrg1 | neuregulin 1 |
| | | Nrg3 | neuregulin 3 |
| | | Ntn4 | netrin 4 |

| | |
|---|---|
| Ntng1 | netrin G1 |
| Pdgfb | similar to platelet-derived grow |
| Rabep1 | rabaptin, RAB GTPase binding e |
| Sema3b | semaphorin 3B |
| Sema3c | semaphorin 3C |
| Sema4d | semaphorin 4D |
| Sema4f | semaphorin 4F |
| Sema4g | semaphorin 4G |
| Slit1 | slit homolog 1 |
| Slit3 | slit homolog 3 |
| Tgfb2 | transforming growth factor, bet |

**CtrlE11 vs WntE11**

| C11vsW11 | Column2 |
|---|---|
| Amelx | amelogenin X chromosome |
| Bmp4 | bone morphogenetic protein 4 |
| Bmp5 | bone morphogenetic protein 5 |
| Csf1 | colony stimulating factor 1 (macr |
| Cxcl12 | chemokine (C-X-C motif) ligand 1 |
| Edn1 | endothelin 1 |
| Edn3 | endothelin 3 |
| Efnb2 | ephrin B2 |
| Fgf10 | fibroblast growth factor 10 |
| Fgf9 | fibroblast growth factor 9 |
| Gdf6 | growth differentiation factor 6 |
| Gdnf | glial cell line derived neurotrophi |
| Gmfg | similar to Chain A, Crystal Structu |
| Hdgf | hepatoma-derived growth factor |
| Hdgfrp3 | hepatoma-derived growth factor |
| Hgf | hepatocyte growth factor |
| Igf1 | insulin-like growth factor 1 |
| Igf2 | insulin-like growth factor 2 |
| Inhba | inhibin beta-A |
| Kitl | kit ligand |
| Mdk | midkine |
| Nrg1 | neuregulin 1 |
| Ntf3 | neurotrophin 3 |
| Ntng1 | netrin G1 |
| Pdgfc | platelet-derived growth factor, C |
| Pdgfd | platelet-derived growth factor, D |
| Pgf | placental growth factor |
| Ptn | pleiotrophin |
| Sema3a | semaphorin 3A |
| Sema3f | semaphorin 3F |
| Sema5a | semaphorin 5A |
| Sema6a | semaphorin 6A |

**CtrlE15 vs WntE15**

| C15vsW15 | Column2 |
|---|---|
| Amelx | amelogenin X chromosome |
| Bmp1 | bone morphogenetic protein 1 |
| Bmp2 | bone morphogenetic protein 2 |
| Bmp4 | bone morphogenetic protein 4 |
| Bmp5 | bone morphogenetic protein 5 |
| Bmp6 | bone morphogenetic protein 6 |
| Clec11a | C-type lectin domain family 11, |
| Csf1 | colony stimulating factor 1 (mac |
| Cxcl12 | chemokine (C-X-C motif) ligand |
| Edn3 | endothelin 3 |
| Efna1 | ephrin A1 |
| Efnb1 | ephrin B1 |
| Fgf10 | fibroblast growth factor 10 |
| Fgf18 | fibroblast growth factor 18 |
| Fgf2 | fibroblast growth factor 2 |
| Fgf7 | fibroblast growth factor 7 |
| Fgf9 | fibroblast growth factor 9 |
| Figf | c-fos induced growth factor |
| Gdf10 | growth differentiation factor 10 |
| Gdf6 | growth differentiation factor 6 |
| Gdnf | glial cell line derived neurotroph |
| Gmfg | similar to Chain A, Crystal Struct |
| Hdgf | hepatoma-derived growth facto |
| Hgf | hepatocyte growth factor |
| Igf1 | insulin-like growth factor 1 |
| Igf2 | insulin-like growth factor 2 |
| Il1b | interleukin 1 beta |
| Il6 | interleukin 6 |
| Inhba | inhibin beta-A |
| Jag1 | jagged 1 |
| Kitl | kit ligand |
| Mdk | midkine |

| | | | |
|---|---|---|---|
| Sema6d | semaphorin 6D | Ngf | nerve growth factor |
| Slit2 | slit homolog 2 | Ntf3 | neurotrophin 3 |
| Tgfb1 | transforming growth factor, beta | Ntf5 | neurotrophin 5 |
| Tgfb3 | transforming growth factor, beta | Ogn | osteoglycin |
| Vegfc | vascular endothelial growth facto | Pdgfc | platelet-derived growth factor, ( |
| Wnt11 | wingless-related MMTV integrati | Pgf | placental growth factor |
| Wnt2b | wingless related MMTV integrati | Ptn | pleiotrophin |
| Wnt4 | wingless-related MMTV integrati | Sema3a | semaphorin 3A |
| Wnt5a | wingless-related MMTV integrati | Sema3e | semaphorin 3E |
| Wnt5b | wingless-related MMTV integrati | Sema3f | semaphorin 3F |
| | | Sema5a | semaphorin 5A |
| | | Sema5b | semaphorin 5B |
| | | Sema6d | semaphorin 6D |
| | | Tgfb1 | transforming growth factor, bet |
| | | Tgfb3 | transforming growth factor, bet |
| | | Vegfc | vascular endothelial growth fact |
| | | Wnt2b | wingless related MMTV integrat |
| | | Wnt4 | wingless-related MMTV integrat |
| | | Wnt5a | wingless-related MMTV integrat |
| | | Wnt5b | wingless-related MMTV integrat |
