# Supplementary material for: Transcription and Signaling Regulators in Developing Neuronal Subtypes of Mouse and Human Enteric Nervous System
Source: Gastroenterology. 2018 Feb;154(3):624–36. doi: 10.1053/j.gastro.2017.10.005 (PMC6381388; doi:10.1053/j.gastro.2017.10.005)
Supplement: Supplementary Table 5 [file mmc15.pdf]

Supplementary Table 5: Signaling Receptors enriched (>1.2) in pairwise comparisons between cell populations in the developing gut.

Receptors were identified from the annotation terms *receptor* (SP_PIR_KEYWORDS) and *receptor activity* (GO:0004872). The combined lists of genes were manually screened to remove wrongly annotated genes.

SoxE11 vs WntE11

S11vsW11	Fold Change >1.2
Eda2r	ectodysplasin A2 isoform receptor

SoxE11 vs SoxE15

S11vsS15	Fold Change >1.2
Antxr1	anthrax toxin receptor 1
Ednra	endothelin receptor type A
Epha3	Eph receptor A3
Fkbp4	FK506 binding protein 4
Fzd7	frizzled homolog 7 (Drosophila
Gabra3	gamma-aminobutyric acid (GA
Ghr	growth hormone receptor
Gpr50	G-protein-coupled receptor 50
Gprc5c	G protein-coupled receptor, fa
Grb10	growth factor receptor bound
Hmmr	hyaluronan mediated motility
Hyal2	hyaluronoglucosaminidase 2
Itga4	integrin alpha 4
Itga8	integrin alpha 8
Itga9	integrin alpha 9
Itpr1	inositol 1,4,5-triphosphate rec
Jmjd6	jumonji domain containing 6
Lbr	lamin B receptor
Ly75	lymphocyte antigen 75
Notch3	Notch gene homolog 3 (Drosop
Npr3	natriuretic peptide receptor 3
Nr1h5	nuclear receptor subfamily 1, g
Nr2f1	nuclear receptor subfamily 2, g
Nr2f2	similar to COUP-TFI; nuclear re
Nrip1	nuclear receptor interacting pr
Nrp1	neuropilin 1
Olfr543	olfactory receptor 543
Opn3	opsin 3
Pdgfra	platelet derived growth factor
Phb2	prohibitin 2
Plxna4	plexin A4
Ptch1	patched homolog 1
Ptpn1	protein tyrosine phosphatase,
Ptprk	protein tyrosine phosphatase,
Ret	ret proto-oncogene
Robo1	roundabout homolog 1 (Droso
Rtn4rl1	reticulon 4 receptor-like 1

Scarb1	scavenger receptor class B, me
Sigmar1	sigma non-opioid intracellular
Tas2r137	taste receptor, type 2, membe
Tfrc	transferrin receptor
Tomm22	predicted gene 12906; predicte
Traf4	TNF receptor associated factor
Trip13	thyroid hormone receptor inte
Unc5c	unc-5 homolog C (C. elegans)

WntE11 vs SoxE11

W11vsS11	Fold Change >1.2
Epha3	Eph receptor A3
Gabra3	gamma-aminobutyric acid (GABA) A r
Npr3	natriuretic peptide receptor 3
Nr2f1	nuclear receptor subfamily 2, group F
Slitrk2	SLIT and NTRK-like family, member 2

WntE11 vs WntE15

W11vsW15	Fold Change >1.2
Antxr1	anthrax toxin receptor 1
Bai1	brain-specific angiogenesis inh
Cd200r2	Cd200 receptor 2; CD200 recep
Celsr1	cadherin, EGF LAG seven-pass
Edaradd	EDAR (ectodysplasin-A recepto
Ednra	endothelin receptor type A
Epha3	Eph receptor A3
Ephb4	Eph receptor B4
Erbb2	v-erb-b2 erythroblastic leukem
Erbb3	v-erb-b2 erythroblastic leukem
F2r	coagulation factor II (thrombin
Fkbp4	FK506 binding protein 4
Fzd2	frizzled homolog 2 (Drosophila
Fzd7	frizzled homolog 7 (Drosophila
Gabra3	gamma-aminobutyric acid (GA
Gabra4	gamma-aminobutyric acid (GA
Gfra3	glial cell line derived neurotrop
Git1	G protein-coupled receptor kin
Gpr124	G protein-coupled receptor 12
Gpr125	G protein-coupled receptor 12
Gpr4	G protein-coupled receptor 4
Gprc5b	G protein-coupled receptor, fa
Gprc5c	G protein-coupled receptor, fa
Grb10	growth factor receptor bound
Grk6	G protein-coupled receptor kin
Hmmr	hyaluronan mediated motility
Hyal2	hyaluronoglucosaminidase 2
Ifnar2	interferon (alpha and beta) rec
Ifngr1	interferon gamma receptor 1
Igf2r	insulin-like growth factor 2 rec
Il1rap	interleukin 1 receptor accessor
Il1rl2	interleukin 1 receptor-like 2
Itga4	integrin alpha 4
Itga5	integrin alpha 5 (fibronectin re
Itga6	integrin alpha 6

			Itga9	integrin alpha 9
			Itgav	integrin alpha V
			Itgb5	integrin beta 5
			Itpr1	inositol 1,4,5-triphosphate rec
			Jmjd6	jumonji domain containing 6
			Klra5	killer cell lectin-like receptor, s
			Lbr	lamin B receptor
			Lpar3	lysophosphatidic acid receptor
			Lphn2	latrophilin 2
			Lrp4	low density lipoprotein recepto
			Lrp5	low density lipoprotein recepto
			Ly75	lymphocyte antigen 75
			M6pr	mannose-6-phosphate recepto
			Mertk	c-mer proto-oncogene tyrosine
			Mrgprb2	MAS-related GPR, member B2
			Notch2	Notch gene homolog 2 (Drosop
			Notch3	Notch gene homolog 3 (Drosop
			Npr3	natriuretic peptide receptor 3
			Nr2f1	nuclear receptor subfamily 2, g
			Nr2f2	similar to COUP-TFI; nuclear re
			Nr2f6	nuclear receptor subfamily 2, g
			Nrip1	nuclear receptor interacting pr
			Nrp1	neuropilin 1
			Ogfr	opioid growth factor receptor
			Olfr1134	olfactory receptor 1134
			Olfr121	olfactory receptor 121
			Olfr908	olfactory receptor 908
			Opn3	opsin 3
			Pdgfra	platelet derived growth factor
			Phb2	prohibitin 2
			Plaur	plasminogen activator, urokina
			Plxna1	plexin A1
			Ptch1	patched homolog 1
			Ptk7	PTK7 protein tyrosine kinase 7
			Ptpn1	protein tyrosine phosphatase,
			Ptprk	protein tyrosine phosphatase,
			Rara	retinoic acid receptor, alpha
			Ret	ret proto-oncogene
			Rhbdf1	rhomboid family 1 (Drosophila
			Rnf135	ring finger protein 135
			Robo1	roundabout homolog 1 (Droso
			Rpsa	
			Rpsa	
			Rtn4rl1	reticulon 4 receptor-like 1
			Rxra	retinoid X receptor alpha; simi
			Rxrg	retinoid X receptor gamma

		Ryk	receptor-like tyrosine kinase
		Scarb1	scavenger receptor class B, me
		Sigmar1	sigma non-opioid intracellular
		Slc20a2	hypothetical protein LOC10004
		Slitrk2	SLIT and NTRK-like family, mer
		Smo	predicted gene 4066; smoothe
		Sra1	steroid receptor RNA activator
		Srprb	signal recognition particle rece
		Strap	serine/threonine kinase recept
		Tmem123	transmembrane protein 123
		Tnfrsf19	tumor necrosis factor receptor
		Tomm22	predicted gene 12906; predicte
		Traf4	TNF receptor associated factor
		Traf6	TNF receptor-associated factor
		Trip13	thyroid hormone receptor inte
		Trip6	thyroid hormone receptor inte
**SoxE15 vs WntE15**		**SoxE15 Vs SoxE11**	
**S15vsW15**	**Fold Change >1.2**	**S15vsS11**	**Fold Change >1.2**
Antxr1	anthrax toxin receptor 1	Abcc9	ATP-binding cassette, sub-fami
Arntl	aryl hydrocarbon receptor nuclear tra	Acvr1	activin A receptor, type 1
C3ar1	complement component 3a receptor	Adam22	a disintegrin and metallopeptic
Eda2r	ectodysplasin A2 isoform receptor	Adcyap1r1	adenylate cyclase activating po
Epha2	Eph receptor A2	Agtr1a	angiotensin II receptor, type 1a
Epha4	Eph receptor A4	Ahr	aryl-hydrocarbon receptor
Epha7	Eph receptor A7	Amfr	autocrine motility factor recep
Erbb3	v-erb-b2 erythroblastic leukemia viral	Arntl	aryl hydrocarbon receptor nuc
Fzd4	frizzled homolog 4 (Drosophila)	Atrn	attractin
Fzd6	frizzled homolog 6 (Drosophila)	Avpr1a	arginine vasopressin receptor
Fzd7	frizzled homolog 7 (Drosophila)	Bai2	brain-specific angiogenesis inh
Gab1	growth factor receptor bound proteir	Bai3	brain-specific angiogenesis inh
Gabra4	gamma-aminobutyric acid (GABA) A r	Bmpr2	bone morphogenic protein rec
Gfra1	glial cell line derived neurotrophic fac	Calcrl	calcitonin receptor-like
Gpr124	G protein-coupled receptor 124	Cd36	CD36 antigen
Gpr125	G protein-coupled receptor 125	Celsr3	cadherin, EGF LAG seven-pass
Gpr126	G protein-coupled receptor 126	Chrna3	cholinergic receptor, nicotinic,
Gprc5b	G protein-coupled receptor, family C,	Chrna5	cholinergic receptor, nicotinic,
Grb10	growth factor receptor bound proteir	Chrna7	cholinergic receptor, nicotinic,
Grik3	glutamate receptor, ionotropic, kaina	Chrnb2	cholinergic receptor, nicotinic,
Hmmr	hyaluronan mediated motility recepto	Chrnb4	cholinergic receptor, nicotinic,
Igf2r	insulin-like growth factor 2 receptor	Clec2d	C-type lectin domain family 2,
Il1rap	interleukin 1 receptor accessory prote	Cxcr4	chemokine (C-X-C motif) recep
Il1rl2	interleukin 1 receptor-like 2	Dner	delta/notch-like EGF-related re
Irs1	insulin receptor substrate 1	Ednrb	endothelin receptor type B
Itga2	integrin alpha 2	Epha2	Eph receptor A2
Itga6	integrin alpha 6	Epha4	Eph receptor A4

Itgav	integrin alpha V	Epha5	Eph receptor A5
Itgb5	integrin beta 5	Epha6	Eph receptor A6
Kdr	kinase insert domain protein recepto	Epha7	Eph receptor A7
Lphn2	latrophilin 2	Ephb1	Eph receptor B1
Mertk	c-mer proto-oncogene tyrosine kinas	Esrrg	estrogen-related receptor gam
Mrc1	mannose receptor, C type 1	Fcgrt	Fc receptor, IgG, alpha chain tr
Notch2	Notch gene homolog 2 (Drosophila)	Fzd6	frizzled homolog 6 (Drosophila
Nr2f1	nuclear receptor subfamily 2, group F	Gabbr1	gamma-aminobutyric acid (GA
Nr2f2	similar to COUP-TFI; nuclear receptor	Gabra4	gamma-aminobutyric acid (GA
Nr4a1	nuclear receptor subfamily 4, group A	Gabrb3	gamma-aminobutyric acid (GA
Ogfr	opioid growth factor receptor	Gabrg2	gamma-aminobutyric acid (GA
Olfr1318	olfactory receptor 1318	Gpr126	G protein-coupled receptor 12
Olfr908	olfactory receptor 908	Gpr155	G protein-coupled receptor 15
P2rx7	purinergic receptor P2X, ligand-gated	Gpr173	G-protein coupled receptor 17
Phb2	prohibitin 2	Gpr22	G protein-coupled receptor 22
Ptgfrn	prostaglandin F2 receptor negative re	Gpr37l1	G protein-coupled receptor 37
Ptk7	PTK7 protein tyrosine kinase 7	Gpr39	G protein-coupled receptor 39
Robo1	roundabout homolog 1 (Drosophila)	Gpr56	G protein-coupled receptor 56
Ror1	receptor tyrosine kinase-like orphan r	Gpr64	G protein-coupled receptor 64
Rpsa	predicted gene 9323; predicted gene	Gpr98	G protein-coupled receptor 98
Ryk	receptor-like tyrosine kinase	Grb14	growth factor receptor bound
Slitrk2	SLIT and NTRK-like family, member 2	Gria2	glutamate receptor, ionotropic
Srprb	signal recognition particle receptor, B	Gria3	glutamate receptor, ionotropic
Stab2	similar to FEX2; stabilin 2	Gria4	glutamate receptor, ionotropic
Tmem123	transmembrane protein 123	Grik1	glutamate receptor, ionotropic
Tnfrsf10b	tumor necrosis factor receptor super	Grik2	glutamate receptor, ionotropic
Tnfrsf12a	tumor necrosis factor receptor super	Grik3	glutamate receptor, ionotropic
Tnfrsf19	tumor necrosis factor receptor super	Grin1	glutamate receptor, ionotropic
Trip13	thyroid hormone receptor interactor	Grin2b	glutamate receptor, ionotropic
		Grm7	glutamate receptor, metabotro
		Grm8	glutamate receptor, metabotro
		Htr2b	5-hydroxytryptamine (serotoni
		Htr3a	5-hydroxytryptamine (serotoni
		Htr4	5 hydroxytryptamine (serotoni
		Ifngr2	interferon gamma receptor 2
		Il13ra1	interleukin 13 receptor, alpha
		Il17rd	interleukin 17 receptor D
		Il1rapl1	interleukin 1 receptor accessor
		Il6st	interleukin 6 signal transducer
		Irs1	insulin receptor substrate 1
		Itga1	integrin alpha 1
		Itga2	integrin alpha 2
		Itgb3	integrin beta 3
		Lgr5	leucine rich repeat containing
		Lifr	leukemia inhibitory factor rece
		Lphn1	latrophilin 1

			Lphn2	latrophilin 2
			Lrp11	low density lipoprotein recepto
			Mfsd6	major facilitator superfamily do
			Mmd2	monocyte to macrophage diffe
			Notch2	Notch gene homolog 2 (Drosop
			Npr2	natriuretic peptide receptor 2
			Npy1r	neuropeptide Y receptor Y1
			Npy2r	neuropeptide Y receptor Y2
			Nr1d2	nuclear receptor subfamily 1, g
			Nr1h4	nuclear receptor subfamily 1, g
			Nr2c2	nuclear receptor subfamily 2, g
			Nr3c1	nuclear receptor subfamily 3, g
			Nr4a1	nuclear receptor subfamily 4, g
			Nr4a2	nuclear receptor subfamily 4, g
			Olfr1316	olfactory receptor 1316
			Olfr1318	olfactory receptor 1318
			Olfr1402	olfactory receptor 1402
			P2rx7	purinergic receptor P2X, ligand
			P2ry1	purinergic receptor P2Y, G-pro
			Paqr8	progestin and adipoQ receptor
			Pcdha10	
			Plxna2	plexin A2
			Plxna3	plexin A3
			Plxnb1	plexin B1
			Plxnb3	plexin B3
			Ptger4	prostaglandin E receptor 4 (sub
			Ptpn3	protein tyrosine phosphatase,
			Ptprj	protein tyrosine phosphatase,
			Ptprn2	protein tyrosine phosphatase,
			Ptprr	protein tyrosine phosphatase,
			Ptprs	protein tyrosine phosphatase,
			Ptprz1	protein tyrosine phosphatase,
			Ramp1	receptor (calcitonin) activity m
			Rarb	retinoic acid receptor, beta
			Robo2	roundabout homolog 2 (Drosop
			Rora	RAR-related orphan receptor a
			Rorc	RAR-related orphan receptor g
			Ryr2	ryanodine receptor 2, cardiac
			S1pr3	sphingosine-1-phosphate recep
			Slc22a17	solute carrier family 22 (organi
			Slitrk1	SLIT and NTRK-like family, mem
			Slitrk2	SLIT and NTRK-like family, mem
			Slitrk3	SLIT and NTRK-like family, mem
			Sorl1	similar to sortilin-related recep
			Sort1	sortilin 1
			Sv2a	synaptic vesicle glycoprotein 2

Sv2b	synaptic vesicle glycoprotein 2
Sv2c	synaptic vesicle glycoprotein 2
Tacr3	tachykinin receptor 3
Tas2r143	taste receptor, type 2, membe
Thra	thyroid hormone receptor alpl
Tnfrsf12a	tumor necrosis factor receptor
Traf5	TNF receptor-associated factor
Trpc1	transient receptor potential ca
Trpc6	transient receptor potential ca
Trpm3	transient receptor potential ca
Trpv3	transient receptor potential ca
Unc5d	unc-5 homolog D (C. elegans)
Vldlr	very low density lipoprotein re
Xpr1	xenotropic and polytropic retr

Wnt15 vs Sox15

W15vsS15	Fold Change >1.2
Adam22	a disintegrin and metallopeptidase dc
Adra2a	adrenergic receptor, alpha 2a
Alk	anaplastic lymphoma kinase
Avpr1a	arginine vasopressin receptor 1A
Bai3	brain-specific angiogenesis inhibitor 3
Bmpr1b	bone morphogenetic protein recepto
Calcrl	calcitonin receptor-like
Celsr3	cadherin, EGF LAG seven-pass G-type
Chrna3	cholinergic receptor, nicotinic, alpha
Chrna5	cholinergic receptor, nicotinic, alpha
Cnr1	cannabinoid receptor 1 (brain)
Cxadr	coxsackie virus and adenovirus recept
Cxcr4	chemokine (C-X-C motif) receptor 4
Cxcr7	chemokine (C-X-C motif) receptor 7
Darc	Duffy blood group, chemokine recept
Dcc	deleted in colorectal carcinoma
Dner	delta/notch-like EGF-related receptor
Epha5	Eph receptor A5
Epha6	Eph receptor A6
Gabbr1	gamma-aminobutyric acid (GABA) B r
Gabra3	gamma-aminobutyric acid (GABA) A r
Gabrb3	gamma-aminobutyric acid (GABA) A r
Gabrg2	gamma-aminobutyric acid (GABA) A r
Galr2	galanin receptor 2
Gfra2	glial cell line derived neurotrophic fac
Glp1r	glucagon-like peptide 1 receptor; sim
Gpr155	G protein-coupled receptor 155
Gpr158	G protein-coupled receptor 158
Gpr64	G protein-coupled receptor 64

Wnt15 vs Wnt11

W15vsW11	Fold Change >1.2
Abcc9	ATP-binding cassette, sub-fam
Acvr2a	activin receptor IIA
Adam22	a disintegrin and metallopeptic
Adcyap1r1	adenylate cyclase activating po
Adra2a	adrenergic receptor, alpha 2a
Agtr1a	angiotensin II receptor, type 1a
Ahr	aryl-hydrocarbon receptor
Amfr	autocrine motility factor recep
Ar	androgen receptor
Atp6ap2	ATPase, H+ transporting, lysos
Atrn	attractin
Avpr1a	arginine vasopressin receptor
Bai2	brain-specific angiogenesis inh
Bai3	brain-specific angiogenesis inh
Bmpr1b	bone morphogenetic protein r
Bmpr2	bone morphogenic protein rec
Calcrl	calcitonin receptor-like
Celsr3	cadherin, EGF LAG seven-pass
Chrm2	cholinergic receptor, muscarin
Chrna3	cholinergic receptor, nicotinic,
Chrna5	cholinergic receptor, nicotinic,
Chrna7	cholinergic receptor, nicotinic,
Chrnb1	cholinergic receptor, nicotinic,
Chrnb2	cholinergic receptor, nicotinic,
Chrnb4	cholinergic receptor, nicotinic,
Cnr1	cannabinoid receptor 1 (brain)
Csf2ra	colony stimulating factor 2 rec
Cxadr	coxsackie virus and adenovirus
Cxcr4	chemokine (C-X-C motif) recep

Gpr85	G protein-coupled receptor 85	Cxcr7	chemokine (C-X-C motif) recep
Gria1	glutamate receptor, ionotropic, AMP/	Darc	Duffy blood group, chemokine
Gria2	glutamate receptor, ionotropic, AMP/	Dner	delta/notch-like EGF-related re
Grik1	glutamate receptor, ionotropic, kaina	Dpp4	dipeptidylpeptidase 4
Grin1	glutamate receptor, ionotropic, NMD	Ednrb	endothelin receptor type B
Grm7	glutamate receptor, metabotropic 7	Epha4	Eph receptor A4
Htr1d	5-hydroxytryptamine (serotonin) rece	Epha5	Eph receptor A5
Htr2b	5-hydroxytryptamine (serotonin) rece	Epha6	Eph receptor A6
Htr3b	5-hydroxytryptamine (serotonin) rece	Ephb1	Eph receptor B1
Htr4	5 hydroxytryptamine (serotonin) rece	Esrrg	estrogen-related receptor gam
Il13ra1	interleukin 13 receptor, alpha 1	Ffar3	free fatty acid receptor 3
Itga3	integrin alpha 3	Gab2	growth factor receptor bound
Lcor	ligand dependent nuclear receptor cc	Gabbr1	gamma-aminobutyric acid (GA
Lpar4	lysophosphatidic acid receptor 4	Gabrb2	gamma-aminobutyric acid (GA
Lrp11	low density lipoprotein receptor-relat	Gabrb3	gamma-aminobutyric acid (GA
Lrp1b	low density lipoprotein-related prote	Gabrg2	gamma-aminobutyric acid (GA
Ltk	leukocyte tyrosine kinase	Galr1	galanin receptor 1
Npr2	natriuretic peptide receptor 2	Gfra2	glial cell line derived neurotrop
Npy1r	neuropeptide Y receptor Y1	Glp1r	glucagon-like peptide 1 recept
Npy2r	neuropeptide Y receptor Y2	Gpr126	G protein-coupled receptor 12
Nrp2	neuropilin 2	Gpr128	G protein-coupled receptor 12
Ntrk3	neurotrophic tyrosine kinase, recepto	Gpr137c	G protein-coupled receptor 13
Olfr1382	olfactory receptor 1382	Gpr155	G protein-coupled receptor 15
Olfr191	olfactory receptor 191	Gpr158	G protein-coupled receptor 15
Olfr264	olfactory receptor 264; olfactory rece	Gpr165	G protein-coupled receptor 16
Oprm1	opioid receptor, mu 1	Gpr173	G-protein coupled receptor 17
Pcdha10	similar to protocadherin; protocadhe	Gpr176	G protein-coupled receptor 17
Pgr	progesterone receptor	Gpr22	G protein-coupled receptor 22
Plxna3	plexin A3	Gpr56	G protein-coupled receptor 56
Plxna4	plexin A4	Gpr64	G protein-coupled receptor 64
Plxnc1	plexin C1; similar to plexin C1	Gpr75	G protein-coupled receptor 75
Prokr1	prokineticin receptor 1	Gpr85	G protein-coupled receptor 85
Pth2r	parathyroid hormone 2 receptor	Gpr98	G protein-coupled receptor 98
Ptprg	protein tyrosine phosphatase, recept	Grb14	growth factor receptor bound
Ptprn	protein tyrosine phosphatase, recept	Gria1	glutamate receptor, ionotropic
Ptprn2	protein tyrosine phosphatase, recept	Gria2	glutamate receptor, ionotropic
Ptpro	protein tyrosine phosphatase, non-re	Gria3	glutamate receptor, ionotropic
Ptprr	protein tyrosine phosphatase, recept	Gria4	glutamate receptor, ionotropic
Ramp1	receptor (calcitonin) activity modifyin	Grid1	glutamate receptor, ionotropic
Robo2	roundabout homolog 2 (Drosophila)	Grik1	glutamate receptor, ionotropic
Ryr2	ryanodine receptor 2, cardiac	Grik2	glutamate receptor, ionotropic
Ryr3	ryanodine receptor 3	Grik4	glutamate receptor, ionotropic
Slitrk3	SLIT and NTRK-like family, member 3	Grin1	glutamate receptor, ionotropic
Sorl1	similar to sortilin-related receptor, LD	Grin2b	glutamate receptor, ionotropic
Sv2a	synaptic vesicle glycoprotein 2 a	Grm1	similar to metabotropic glutam
Sv2c	synaptic vesicle glycoprotein 2c	Grm7	glutamate receptor, metabotro

Taar1	trace amine-associated receptor 1	Htr1d	5-hydroxytryptamine (serotoni
Thra	thyroid hormone receptor alpha; simi	Htr2b	5-hydroxytryptamine (serotoni
Tnfrsf18	tumor necrosis factor receptor superf	Htr3a	5-hydroxytryptamine (serotoni
Trpc4	transient receptor potential cation ch	Htr3b	5-hydroxytryptamine (serotoni
Trpc6	transient receptor potential cation ch	Htr4	5 hydroxytryptamine (serotoni
Tyro3	similar to Rse; TYRO3 protein tyrosine	Ifngr2	interferon gamma receptor 2
Unc5c	unc-5 homolog C (C. elegans)	Il13ra1	interleukin 13 receptor, alpha
Unc5d	unc-5 homolog D (C. elegans)	Il17rd	interleukin 17 receptor D
Vldlr	very low density lipoprotein receptor	Il1rapl1	interleukin 1 receptor accessor
Vmn2r29	vomeronasal 2, receptor, pseudogene	Il1rapl2	interleukin 1 receptor accessor
Xpr1	xenotropic and polytropic retrovirus r	Il4ra	interleukin 4 receptor, alpha
		Insr	insulin receptor
		Itga1	integrin alpha 1
		Itga2	integrin alpha 2
		Itga3	integrin alpha 3
		Itgb3	integrin beta 3
		Kit	kit oncogene
		Lcor	ligand dependent nuclear rece
		Lgr5	leucine rich repeat containing
		Lmbr1	limb region 1
		Lpar4	lysophosphatidic acid receptor
		Lphn1	latrophilin 1
		Lphn2	latrophilin 2
		Lphn3	latrophilin 3
		Lrp11	low density lipoprotein recepto
		Lrp1b	low density lipoprotein-related
		Ltk	leukocyte tyrosine kinase
		Mfsd6	major facilitator superfamily d
		Mmd2	monocyte to macrophage diffe
		Mrgprg	MAS-related GPR, member G
		Mst1r	macrophage stimulating 1 rece
		Ncor1	nuclear receptor co-repressor
		Npr2	natriuretic peptide receptor 2
		Npy1r	neuropeptide Y receptor Y1
		Npy2r	neuropeptide Y receptor Y2
		Nr1d2	nuclear receptor subfamily 1, g
		Nr2c2	nuclear receptor subfamily 2, g
		Nr3c1	nuclear receptor subfamily 3, g
		Nr4a1	nuclear receptor subfamily 4, g
		Nr6a1	nuclear receptor subfamily 6, g
		Nrp2	neuropilin 2
		Ntrk3	neurotrophic tyrosine kinase, r
		Olfr1042	olfactory receptor 1042
		Olfr1043	olfactory receptor 1043
		Olfr1138	olfactory receptor 1138
		Olfr1361	olfactory receptor 1361

			Olfr159	olfactory receptor 159
			Olfr288	olfactory receptor 288; olfacto
			Olfr78	olfactory receptor 78
			Olfr938	olfactory receptor 938
			Oprl1	opioid receptor-like 1
			Oprm1	opioid receptor, mu 1
			P2rx7	purinergic receptor P2X, ligand
			P2ry6	pyrimidinergic receptor P2Y, G
			Paqr8	progestin and adipoQ receptor
			Pcdha10	
			Pgr	progesterone receptor
			Plxna2	plexin A2
			Plxna3	plexin A3
			Plxnb3	plexin B3
			Plxnc1	plexin C1; similar to plexin C1
			Prokr1	prokineticin receptor 1
			Ptger3	prostaglandin E receptor 3 (sub
			Ptger4	prostaglandin E receptor 4 (sub
			Ptgir	prostaglandin I receptor (IP)
			Ptpn3	protein tyrosine phosphatase,
			Ptprb	protein tyrosine phosphatase,
			Ptprg	protein tyrosine phosphatase,
			Ptprj	protein tyrosine phosphatase,
			Ptprn	protein tyrosine phosphatase,
			Ptprn2	protein tyrosine phosphatase,
			Ptpro	protein tyrosine phosphatase,
			Ptprr	protein tyrosine phosphatase,
			Ptprs	protein tyrosine phosphatase,
			Ptprt	protein tyrosine phosphatase,
			Ptprz1	protein tyrosine phosphatase,
			Ramp1	receptor (calcitonin) activity m
			Rarb	retinoic acid receptor, beta
			Reep5	receptor accessory protein 5
			Robo2	roundabout homolog 2 (Droso
			Rora	RAR-related orphan receptor a
			Rorc	RAR-related orphan receptor g
			Ryr2	ryanodine receptor 2, cardiac
			Ryr3	ryanodine receptor 3
			Sctr	secretin receptor; similar to Sc
			Slitrk1	SLIT and NTRK-like family, mer
			Slitrk3	SLIT and NTRK-like family, mer
			Sorl1	similar to sortilin-related recep
			Sstr4	somatostatin receptor 4
			Sv2a	synaptic vesicle glycoprotein 2
			Sv2b	synaptic vesicle glycoprotein 2
			Sv2c	synaptic vesicle glycoprotein 2

		Taar1	trace amine-associated recepto
		Tacr3	tachykinin receptor 3
		Tas2r143	taste receptor, type 2, membe
		Thra	thyroid hormone receptor alph
		Tnfrsf21	tumor necrosis factor receptor
		Tnfsf18	tumor necrosis factor (ligand)
		Trem2	triggering receptor expressed
		Trpc1	transient receptor potential ca
		Trpc4	transient receptor potential ca
		Trpc6	transient receptor potential ca
		Trpc7	transient receptor potential ca
		Trpm3	transient receptor potential ca
		Tyro3	similar to Rse; TYRO3 protein t
		Unc5a	unc-5 homolog A (C. elegans)
		Unc5d	unc-5 homolog D (C. elegans)
		Vldlr	very low density lipoprotein re
		Vmn2r29	
		Vmn2r81	vomeronasal 2, receptor 81; vo
		Xpr1	xenotropic and polytropic retr
**SoxE11vsCtrlE11**		**SoxE15vsCtrlE15**	
**S11vsC11**	**Fold Change >1.2**	**S15vsC15**	**Fold Change >1.2**
Acvr1	activin A receptor, type 1	Acvr1b	activin A receptor, type 1B
Adam22	a disintegrin and metallopeptidase dc	Acvr2a	activin receptor IIA
Adcyap1r1	adenylate cyclase activating polypept	Adam22	a disintegrin and metallopeptic
Agtr2	angiotensin II receptor, type 2	Adcyap1r1	adenylate cyclase activating po
Ahr	aryl-hydrocarbon receptor	Agtr1a	angiotensin II receptor, type 1a
Alk	anaplastic lymphoma kinase	Alk	anaplastic lymphoma kinase
Arntl	aryl hydrocarbon receptor nuclear tra	Arntl	aryl hydrocarbon receptor nuc
Bai1	brain-specific angiogenesis inhibitor 1	Atrn	attractin
Bai3	brain-specific angiogenesis inhibitor 3	Bai3	brain-specific angiogenesis inh
Celsr2	cadherin, EGF LAG seven-pass G-type	Celsr2	cadherin, EGF LAG seven-pass
Celsr3	cadherin, EGF LAG seven-pass G-type	Celsr3	cadherin, EGF LAG seven-pass
Chrna3	cholinergic receptor, nicotinic, alpha	Chrna3	cholinergic receptor, nicotinic,
Chrna4	cholinergic receptor, nicotinic, alpha	Chrna4	cholinergic receptor, nicotinic,
Chrna5	cholinergic receptor, nicotinic, alpha	Chrna5	cholinergic receptor, nicotinic,
Chrnb2	cholinergic receptor, nicotinic, beta p	Chrna6	cholinergic receptor, nicotinic,
Chrnb4	cholinergic receptor, nicotinic, beta p	Chrna7	cholinergic receptor, nicotinic,
Cnr1	cannabinoid receptor 1 (brain)	Chrnb2	cholinergic receptor, nicotinic,
Dcc	deleted in colorectal carcinoma	Chrnb4	cholinergic receptor, nicotinic,
Dear1	dual endothelin 1/angiotensin II rece	Cnr1	cannabinoid receptor 1 (brain)
Eda2r	ectodysplasin A2 isoform receptor	Darc	Duffy blood group, chemokine
Ednrb	endothelin receptor type B	Dcc	deleted in colorectal carcinom
Epha3	Eph receptor A3	Dear1	dual endothelin 1/angiotensin
Ephb2	Eph receptor B2	Dgcr2	DiGeorge syndrome critical reg
Erbb3	v-erb-b2 erythroblastic leukemia viral	Dner	delta/notch-like EGF-related re

Fkbp4	FK506 binding protein 4	Eda2r	ectodysplasin A2 isoform recep
Fzd3	frizzled homolog 3 (Drosophila)	Ednrb	endothelin receptor type B
Gab1	growth factor receptor bound protein	Epha10	Eph receptor A10
Gab2	growth factor receptor bound protein	Epha6	Eph receptor A6
Gabrb3	gamma-aminobutyric acid (GABA) A r	Erbb3	v-erb-b2 erythroblastic leukem
Gabrg2	gamma-aminobutyric acid (GABA) A r	Ffar3	free fatty acid receptor 3
Gfra1	glial cell line derived neurotrophic fac	Fkbp4	FK506 binding protein 4
Gfra2	glial cell line derived neurotrophic fac	Fzd3	frizzled homolog 3 (Drosophila
Gfra3	glial cell line derived neurotrophic fac	Fzd6	frizzled homolog 6 (Drosophila
Git1	G protein-coupled receptor kinase-int	Gab1	growth factor receptor bound
Gpr125	G protein-coupled receptor 125	Gab2	growth factor receptor bound
Gpr126	G protein-coupled receptor 126	Gabra6	gamma-aminobutyric acid (GA
Gpr17	G protein-coupled receptor 17	Gabrb3	gamma-aminobutyric acid (GA
Gpr19	G protein-coupled receptor 19	Gabrg2	gamma-aminobutyric acid (GA
Gprc5b	G protein-coupled receptor, family C,	Gfra1	glial cell line derived neurotrop
Grb10	growth factor receptor bound protein	Gfra2	glial cell line derived neurotrop
Gria2	glutamate receptor, ionotropic, AMPA	Gfra3	glial cell line derived neurotrop
Gria4	glutamate receptor, ionotropic, AMPA	Git1	G protein-coupled receptor kin
Grik2	glutamate receptor, ionotropic, kaina	Gpr125	G protein-coupled receptor 12
Grik3	glutamate receptor, ionotropic, kaina	Gpr126	G protein-coupled receptor 12
Htr3b	5-hydroxytryptamine (serotonin) rece	Gpr137c	G protein-coupled receptor 13
Igf1r	insulin-like growth factor I receptor	Gpr155	G protein-coupled receptor 15
Igsf11	immunoglobulin superfamily, membe	Gpr158	G protein-coupled receptor 15
Il1rap	interleukin 1 receptor accessory prot	Gpr17	G protein-coupled receptor 17
Il1rl2	interleukin 1 receptor-like 2	Gpr173	G-protein coupled receptor 17
Itga4	integrin alpha 4	Gpr19	G protein-coupled receptor 19
Itga6	integrin alpha 6	Gpr22	G protein-coupled receptor 22
Jmjd6	jumonji domain containing 6	Gpr37l1	G protein-coupled receptor 37
Ldlrad3	low density lipoprotein receptor class	Gpr56	G protein-coupled receptor 56
Lpar3	lysophosphatidic acid receptor 3	Gpr75	G protein-coupled receptor 75
Lrp11	low density lipoprotein receptor-relat	Gpr85	G protein-coupled receptor 85
Lrp5	low density lipoprotein receptor-relat	Gpr98	G protein-coupled receptor 98
Mfsd6	major facilitator superfamily domain	Gprc5b	G protein-coupled receptor, fa
Ngfr	nerve growth factor receptor (TNFR s	Gria2	glutamate receptor, ionotropic
Notch1	Notch gene homolog 1 (Drosophila)	Gria4	glutamate receptor, ionotropic
Notch2	Notch gene homolog 2 (Drosophila)	Grid2	glutamate receptor, ionotropic
Nr2c2ap	nuclear receptor 2C2-associated prot	Grik2	glutamate receptor, ionotropic
Nr4a1	nuclear receptor subfamily 4, group A	Grik3	glutamate receptor, ionotropic
Nrp1	neuropilin 1	Grik5	glutamate receptor, ionotropic
Olfr1043	olfactory receptor 1043	Grin1	glutamate receptor, ionotropic
Olfr382	olfactory receptor 382	Grin2b	glutamate receptor, ionotropic
P2rx4	purinergic receptor P2X, ligand-gated	Grlf1	glucocorticoid receptor DNA bi
Phb2	prohibitin 2	Grm1	similar to metabotropic glutam
Plxna4	plexin A4	H2-Ke2	H2-K region expressed gene 2
Ptpn1	protein tyrosine phosphatase, non-re	Hrh3	histamine receptor H3
Ptpn9	protein tyrosine phosphatase, non-re	Htr2b	5-hydroxytryptamine (serotoni

Ptprg	protein tyrosine phosphatase, recept	Htr3a	5-hydroxytryptamine (serotoni
Ptprj	protein tyrosine phosphatase, recept	Htr3b	5-hydroxytryptamine (serotoni
Ptprm	protein tyrosine phosphatase, recept	Htr4	5 hydroxytryptamine (serotoni
Ptprn	protein tyrosine phosphatase, recept	Igf1r	insulin-like growth factor I rece
Ptprn2	protein tyrosine phosphatase, recept	Igsf11	immunoglobulin superfamily, r
Ptpro	protein tyrosine phosphatase, non-re	Il17rd	interleukin 17 receptor D
Ptprz1	protein tyrosine phosphatase, recept	Il1rap	interleukin 1 receptor accessor
Ramp3	receptor (calcitonin) activity modifyin	Insr	insulin receptor
Rara	retinoic acid receptor, alpha	Irs1	insulin receptor substrate 1
Rarb	retinoic acid receptor, beta	Itga2	integrin alpha 2
Ret	ret proto-oncogene	Itga6	integrin alpha 6
Rhbdf1	rhomboid family 1 (Drosophila)	Lcor	ligand dependent nuclear rece
Rpsa	predicted gene 9323; predicted gene	Lpar3	lysophosphatidic acid receptor
Rtn4rl1	reticulon 4 receptor-like 1	Lrp11	low density lipoprotein recept
Scarb1	scavenger receptor class B, member 1	Lrp12	low density lipoprotein-related
Scarb2	scavenger receptor class B, member 2	Lrp8	low density lipoprotein recept
Sigmar1	sigma non-opioid intracellular recept	Lsr	lipolysis stimulated lipoprotein
Slc7a1	solute carrier family 7 (cationic amino	Mfsd6	major facilitator superfamily d
Slitrk2	SLIT and NTRK-like family, member 2	Mmd2	monocyte to macrophage diffe
Sort1	sortilin 1	Ms4a15	membrane-spanning 4-domain
Srprb	signal recognition particle receptor, B	Mtvr2	mammary tumor virus recepto
Strap	serine/threonine kinase receptor asso	Neto2	neuropilin (NRP) and tolloid (T
Sv2c	synaptic vesicle glycoprotein 2c	Ngfr	nerve growth factor receptor (
Tlr3	toll-like receptor 3	Notch1	Notch gene homolog 1 (Drosop
Tmem123	transmembrane protein 123	Npc1	Niemann Pick type C1
Tnfrsf21	tumor necrosis factor receptor superf	Npy2r	neuropeptide Y receptor Y2
Traf4	TNF receptor associated factor 4	Nr1d2	nuclear receptor subfamily 1, g
		Nr2c1	nuclear receptor subfamily 2, g
		Nr2c2	nuclear receptor subfamily 2, g
		Nr6a1	nuclear receptor subfamily 6, g
		Olfr1015	olfactory receptor 1015
		Olfr1045	olfactory receptor 1045
		Olfr105	olfactory receptor 105
		Olfr1157	olfactory receptor 1157
		Olfr123	olfactory receptor 123
		Olfr1262	olfactory receptor 1262
		Olfr1318	olfactory receptor 1318
		Olfr1392	olfactory receptor 1392
		Olfr1402	olfactory receptor 1402
		Olfr1416	olfactory receptor 1416
		Olfr161	olfactory receptor 161
		Olfr382	olfactory receptor 382
		Olfr39	olfactory receptor 39
		Olfr420	olfactory receptor 420
		Olfr50	olfactory receptor 50
		Olfr521	olfactory receptor 521

			Olfr63	olfactory receptor 63
			Olfr66	similar to MOR 5beta1; olfacto
			Olfr695	olfactory receptor 695
			Olfr824	olfactory receptor 824
			Olfr914	olfactory receptor 914
			Olfr958	olfactory receptor 958
			Olfr963	olfactory receptor 963
			Opn1mw	opsin 1 (cone pigments), mediu
			Opn3	opsin 3
			Oprl1	opioid receptor-like 1
			Oprm1	opioid receptor, mu 1
			P2rx3	purinergic receptor P2X, ligand
			P2rx7	purinergic receptor P2X, ligand
			Paqr3	progestin and adipoQ receptor
			Pcdha10	
			Pgrmc1	progesterone receptor membr
			Phb2	prohibitin 2
			Plxna2	plexin A2
			Plxna3	plexin A3
			Plxna4	plexin A4
			Plxnb1	plexin B1
			Plxnb3	plexin B3
			Pnrc1	proline-rich nuclear receptor c
			Prokr2	prokineticin receptor 2
			Ptpn1	protein tyrosine phosphatase,
			Ptpn12	protein tyrosine phosphatase,
			Ptpn9	protein tyrosine phosphatase,
			Ptprj	protein tyrosine phosphatase,
			Ptprm	protein tyrosine phosphatase,
			Ptprn	protein tyrosine phosphatase,
			Ptprn2	protein tyrosine phosphatase,
			Ptpro	protein tyrosine phosphatase,
			Ptprr	protein tyrosine phosphatase,
			Ptprz1	protein tyrosine phosphatase,
			Ramp3	receptor (calcitonin) activity m
			Rara	retinoic acid receptor, alpha
			Rarb	retinoic acid receptor, beta
			Ret	ret proto-oncogene
			Scarb2	scavenger receptor class B, me
			Slc20a1	solute carrier family 20, memb
			Slc22a17	solute carrier family 22 (organi
			Slitrk1	SLIT and NTRK-like family, mer
			Slitrk2	SLIT and NTRK-like family, mer
			Slitrk3	SLIT and NTRK-like family, mer
			Sort1	sortilin 1
			Sra1	steroid receptor RNA activator

WntE11 vs CtrlE11

W11vsC11	Fold Change >1.2
Acvr1	activin A receptor, type 1
Adcyap1r1	adenylate cyclase activating polypept
Adrbk1	adrenergic receptor kinase, beta 1
Agtr2	angiotensin II receptor, type 2
Ahr	aryl-hydrocarbon receptor
Alk	anaplastic lymphoma kinase
Arntl	aryl hydrocarbon receptor nuclear tra
Bai1	brain-specific angiogenesis inhibitor 1
Bai3	brain-specific angiogenesis inhibitor 3
Celsr1	cadherin, EGF LAG seven-pass G-type
Celsr2	cadherin, EGF LAG seven-pass G-type
Celsr3	cadherin, EGF LAG seven-pass G-type
Chrna3	cholinergic receptor, nicotinic, alpha
Chrna4	cholinergic receptor, nicotinic, alpha
Chrna5	cholinergic receptor, nicotinic, alpha
Chrnb2	cholinergic receptor, nicotinic, beta p
Chrnb4	cholinergic receptor, nicotinic, beta p
Cnr1	cannabinoid receptor 1 (brain)
Dcc	deleted in colorectal carcinoma
Ddr1	discoidin domain receptor family, me
Dear1	dual endothelin 1/angiotensin II recep
Edaradd	EDAR (ectodysplasin-A receptor)-assc
Ednrb	endothelin receptor type B
Epha3	Eph receptor A3
Ephb2	Eph receptor B2
Erbb3	v-erb-b2 erythroblastic leukemia viral
Fzd3	frizzled homolog 3 (Drosophila)
Gab1	growth factor receptor bound protein

Sstr1	somatostatin receptor 1
Sstr2	somatostatin receptor 2
Stab2	similar to FEX2; stabilin 2
Strap	serine/threonine kinase recept
Sv2c	synaptic vesicle glycoprotein 2
Tacr3	tachykinin receptor 3
Tgfbrap1	transforming growth factor, be
Tlr3	toll-like receptor 3
Tnfrsf21	tumor necrosis factor receptor
Tnfrsf8	tumor necrosis factor receptor
Traf4	TNF receptor associated factor
Trpc7	transient receptor potential ca
Trpm3	transient receptor potential ca
Tyro3	similar to Rse; TYRO3 protein t
Xpr1	xenotropic and polytropic retr

WntE15 vs CtrlE15

W15vsC15	Fold Change >1.2
Acvr1b	activin A receptor, type 1B
Acvr2a	activin receptor IIA
Adam22	a disintegrin and metallopepti
Adcyap1r1	adenylate cyclase activating po
Adra2a	adrenergic receptor, alpha 2a
Agtr1a	angiotensin II receptor, type 1
Alk	anaplastic lymphoma kinase
Arntl	aryl hydrocarbon receptor nuc
Atrn	attractin
Bai3	brain-specific angiogenesis inh
Bdkrb2	bradykinin receptor, beta 2
Bmpr1b	bone morphogenetic protein r
Celsr2	cadherin, EGF LAG seven-pass
Celsr3	cadherin, EGF LAG seven-pass
Chrna3	cholinergic receptor, nicotinic,
Chrna4	cholinergic receptor, nicotinic,
Chrna5	cholinergic receptor, nicotinic,
Chrna7	cholinergic receptor, nicotinic,
Chrnb2	cholinergic receptor, nicotinic,
Chrnb4	cholinergic receptor, nicotinic,
Cnr1	cannabinoid receptor 1 (brain)
Cxcr4	chemokine (C-X-C motif) recep
Darc	Duffy blood group, chemokine
Dcc	deleted in colorectal carcinom
Ddr1	discoidin domain receptor fam
Dear1	dual endothelin 1/angiotensin
Dner	delta/notch-like EGF-related re
Ednrb	endothelin receptor type B

Gab2	growth factor receptor bound protein	Epha10	Eph receptor A10
Gabra3	gamma-aminobutyric acid (GABA) A r	Epha5	Eph receptor A5
Gabrb3	gamma-aminobutyric acid (GABA) A r	Epha6	Eph receptor A6
Gabrg2	gamma-aminobutyric acid (GABA) A r	Erbb3	v-erb-b2 erythroblastic leukem
Gfra1	glial cell line derived neurotrophic fac	Erbb4	v-erb-a erythroblastic leukemi
Gfra2	glial cell line derived neurotrophic fac	Ffar3	free fatty acid receptor 3
Gfra3	glial cell line derived neurotrophic fac	Fpr-rs3	formyl peptide receptor, relate
Git1	G protein-coupled receptor kinase-int	Fzd3	frizzled homolog 3 (Drosophila
Gpr125	G protein-coupled receptor 125	Gab2	growth factor receptor bound
Gpr126	G protein-coupled receptor 126	Gabbr1	gamma-aminobutyric acid (GA
Gpr17	G protein-coupled receptor 17	Gabrb3	gamma-aminobutyric acid (GA
Gpr19	G protein-coupled receptor 19	Gabrg2	gamma-aminobutyric acid (GA
Gpr37	G protein-coupled receptor 37	Gabrr3	gamma-aminobutyric acid (GA
Gpr39	G protein-coupled receptor 39	Galr1	galanin receptor 1
Gprc5b	G protein-coupled receptor, family C,	Galr2	galanin receptor 2
Grb10	growth factor receptor bound protein	Gfra1	glial cell line derived neurotrop
Gria2	glutamate receptor, ionotropic, AMPA	Gfra2	glial cell line derived neurotrop
Gria4	glutamate receptor, ionotropic, AMPA	Glp1r	glucagon-like peptide 1 recept
Grid2	glutamate receptor, ionotropic, delta	Gpr111	G protein-coupled receptor 11
Grik2	glutamate receptor, ionotropic, kaina	Gpr125	G protein-coupled receptor 12
Grik3	glutamate receptor, ionotropic, kaina	Gpr126	G protein-coupled receptor 12
Grk4	G protein-coupled receptor kinase 4	Gpr135	G protein-coupled receptor 13
Htr3b	5-hydroxytryptamine (serotonin) rece	Gpr137c	G protein-coupled receptor 13
Igf1r	insulin-like growth factor I receptor	Gpr155	G protein-coupled receptor 15
Igsf11	immunoglobulin superfamily, membe	Gpr156	G protein-coupled receptor 15
Il1rap	interleukin 1 receptor accessory prot	Gpr158	G protein-coupled receptor 15
Il1rl2	interleukin 1 receptor-like 2	Gpr165	G protein-coupled receptor 16
Insr	insulin receptor	Gpr17	G protein-coupled receptor 17
Itga4	integrin alpha 4	Gpr173	G-protein coupled receptor 17
Itga6	integrin alpha 6	Gpr19	G protein-coupled receptor 19
Itgav	integrin alpha V	Gpr22	G protein-coupled receptor 22
Jmjd6	jumonji domain containing 6	Gpr37l1	G protein-coupled receptor 37
Lpar3	lysophosphatidic acid receptor 3	Gpr39	G protein-coupled receptor 39
Lrp11	low density lipoprotein receptor-relat	Gpr56	G protein-coupled receptor 56
Lrp4	low density lipoprotein receptor-relat	Gpr75	G protein-coupled receptor 75
Lrp5	low density lipoprotein receptor-relat	Gpr85	G protein-coupled receptor 85
Lrp8	low density lipoprotein receptor-relat	Gpr98	G protein-coupled receptor 98
Mfsd6	major facilitator superfamily domain	Grb14	growth factor receptor bound
Ngfr	nerve growth factor receptor (TNFR s	Gria1	glutamate receptor, ionotropic
Nisch	nischarin	Gria2	glutamate receptor, ionotropic
Notch1	Notch gene homolog 1 (Drosophila)	Gria4	glutamate receptor, ionotropic
Notch2	Notch gene homolog 2 (Drosophila)	Grid1	glutamate receptor, ionotropic
Npr3	natriuretic peptide receptor 3	Grid2	glutamate receptor, ionotropic
Nr4a1	nuclear receptor subfamily 4, group A	Grik1	glutamate receptor, ionotropic
Nr4a2	nuclear receptor subfamily 4, group A	Grik2	glutamate receptor, ionotropic
Nrip1	nuclear receptor interacting protein 1	Grik3	glutamate receptor, ionotropic

Nrp1	neuropilin 1		Grik5	glutamate receptor, ionotropic
Ogfr	opioid growth factor receptor		Grin1	glutamate receptor, ionotropic
Olfr847	olfactory receptor 847		Grin2b	glutamate receptor, ionotropic
Olfr908	olfactory receptor 908		Grlf1	glucocorticoid receptor DNA bi
P2rx4	purinergic receptor P2X, ligand-gated		Grm1	similar to metabotropic glutam
Phb2	prohibitin 2		Grm7	glutamate receptor, metabotro
Plxna4	plexin A4		Htr2b	5-hydroxytryptamine (serotoni
Plxnb1	plexin B1		Htr3a	5-hydroxytryptamine (serotoni
Ptpn1	protein tyrosine phosphatase, non-re		Htr3b	5-hydroxytryptamine (serotoni
Ptpn9	protein tyrosine phosphatase, non-re		Htr4	5 hydroxytryptamine (serotoni
Ptprf	protein tyrosine phosphatase, recept		Igf1r	insulin-like growth factor I rece
Ptprj	protein tyrosine phosphatase, recept		Igsf11	immunoglobulin superfamily, m
Ptprm	protein tyrosine phosphatase, recept		Il17rd	interleukin 17 receptor D
Ptprn	protein tyrosine phosphatase, recept		Il17rd	interleukin 17 receptor D
Ptpro	protein tyrosine phosphatase, non-re		Il1rap	interleukin 1 receptor accessor
Ptprz1	protein tyrosine phosphatase, recept		Il1rapl1	interleukin 1 receptor accessor
Ramp3	receptor (calcitonin) activity modifyin		Insr	insulin receptor
Rara	retinoic acid receptor, alpha		Itga2	integrin alpha 2
Rarb	retinoic acid receptor, beta		Itga6	integrin alpha 6
Ret	ret proto-oncogene		Lcor	ligand dependent nuclear rece
Rhbdf1	rhomboid family 1 (Drosophila)		Lmbr1l	limb region 1 like
Rtn4rl1	reticulon 4 receptor-like 1		Lpar3	lysophosphatidic acid receptor
Rxra	retinoid X receptor alpha; similar to re		Lpar4	lysophosphatidic acid receptor
Rxrg	retinoid X receptor gamma		Lphn1	latrophilin 1
Scarb2	scavenger receptor class B, member 2		Lrp11	low density lipoprotein recepto
Sigmar1	sigma non-opioid intracellular recept		Lrp1b	low density lipoprotein-related
Slc7a1	solute carrier family 7 (cationic amino		Lrp8	low density lipoprotein recepto
Slitrk2	SLIT and NTRK-like family, member 2		Ltk	leukocyte tyrosine kinase
Sort1	sortilin 1		Mfsd6	major facilitator superfamily do
Sstr2	somatostatin receptor 2		Mmd2	monocyte to macrophage diffe
Sv2c	synaptic vesicle glycoprotein 2c		Mrgpre	MAS-related GPR, member E
Tgfbrap1	transforming growth factor, beta rece		Mrgprg	MAS-related GPR, member G
Tlr3	toll-like receptor 3		Myh7b	myosin, heavy chain 7B, cardia
Tmem123	transmembrane protein 123		Neto2	neuropilin (NRP) and tolloid (T
Tnfrsf21	tumor necrosis factor receptor superf		Ngfr	nerve growth factor receptor (
Traf4	TNF receptor associated factor 4		Npy1r	neuropeptide Y receptor Y1
			Npy2r	neuropeptide Y receptor Y2
			Npy5r	neuropeptide Y receptor Y5
			Nr1d2	nuclear receptor subfamily 1, g
			Nr2c1	nuclear receptor subfamily 2, g
			Nr2c2	nuclear receptor subfamily 2, g
			Nr6a1	nuclear receptor subfamily 6, g
			Olfr1043	olfactory receptor 1043
			Olfr1157	olfactory receptor 1157
			Olfr1272	olfactory receptor 1273; olfact
			Olfr1349	olfactory receptor 1349

			Olfr1382	olfactory receptor 1382
			Olfr1389	olfactory receptor 1389
			Olfr288	olfactory receptor 288; olfacto
			Olfr39	olfactory receptor 39
			Olfr503	olfactory receptor 503; olfacto
			Olfr521	olfactory receptor 521
			Olfr60	olfactory receptor 60
			Olfr63	olfactory receptor 63
			Olfr695	olfactory receptor 695
			Olfr78	olfactory receptor 78
			Olfr914	olfactory receptor 914
			Olfr963	olfactory receptor 963
			Olfr967	olfactory receptor 967
			Olfr975	olfactory receptor 975
			Oprl1	opioid receptor-like 1
			Oprm1	opioid receptor, mu 1
			Oxtr	oxytocin receptor
			P2rx3	purinergic receptor P2X, ligand
			Paqr3	progestin and adipoQ receptor
			Paqr9	progestin and adipoQ receptor
			Pcdha10	
			Pgr	progesterone receptor
			Pgrmc1	progesterone receptor membr
			Plxna2	plexin A2
			Plxna3	plexin A3
			Plxna4	plexin A4
			Plxnb1	plexin B1
			Plxnb3	plexin B3
			Ppyr1	pancreatic polypeptide recepto
			Prokr2	prokineticin receptor 2
			Pth2r	parathyroid hormone 2 recept
			Ptpn1	protein tyrosine phosphatase,
			Ptpn12	protein tyrosine phosphatase,
			Ptpn3	protein tyrosine phosphatase,
			Ptpn9	protein tyrosine phosphatase,
			Ptprg	protein tyrosine phosphatase,
			Ptprj	protein tyrosine phosphatase,
			Ptprm	protein tyrosine phosphatase,
			Ptprn	protein tyrosine phosphatase,
			Ptprn2	protein tyrosine phosphatase,
			Ptpro	protein tyrosine phosphatase,
			Ptprr	protein tyrosine phosphatase,
			Ptprs	protein tyrosine phosphatase,
			Ptprz1	protein tyrosine phosphatase,
			Ramp3	receptor (calcitonin) activity m
			Rarb	retinoic acid receptor, beta

			Ret	ret proto-oncogene
			Rrh	retinal pigment epithelium der
			Scarb2	scavenger receptor class B, me
			Slc20a1	solute carrier family 20, memb
			Slitrk1	SLIT and NTRK-like family, me
			Slitrk2	SLIT and NTRK-like family, me
			Slitrk3	SLIT and NTRK-like family, me
			Sorl1	similar to sortilin-related recep
			Sort1	sortilin 1
			Sstr1	somatostatin receptor 1
			Sstr2	somatostatin receptor 2
			Stra6	stimulated by retinoic acid gen
			Sv2a	synaptic vesicle glycoprotein 2
			Sv2c	synaptic vesicle glycoprotein 2
			Taar1	trace amine-associated recept
			Taar2	trace amine-associated recept
			Tacr3	tachykinin receptor 3
			Tas2r134	taste receptor, type 2, membe
			Tnfrsf21	tumor necrosis factor receptor
			Traf4	TNF receptor associated factor
			Trpc6	transient receptor potential ca
			Trpc7	transient receptor potential ca
			Trpm3	transient receptor potential ca
			Tyro3	similar to Rse; TYRO3 protein t
			Unc5a	unc-5 homolog A (C. elegans)
			Unc5d	unc-5 homolog D (C. elegans)
			Vmn2r81	vomeronasal 2, receptor 81; vo
			Xpr1	xenotropic and polytropic retr
