# Supplementary material for: Transcription and Signaling Regulators in Developing Neuronal Subtypes of Mouse and Human Enteric Nervous System
Source: Gastroenterology. 2018 Feb;154(3):624–36. doi: 10.1053/j.gastro.2017.10.005 (PMC6381388; doi:10.1053/j.gastro.2017.10.005)
Supplement: Supplementary Table 6 [file mmc16.pdf]

SUPPLEMENTARY TABLE 6.
Transcription factors in the developing ENS

Gene:	Online ISH resources:	IHC:	Current screen:	Reference/Comment:
Alx1*	N/D	**In the ENS Fig. 2A, 3A; S3, 4**	W11vsC11	
Arntl*	Few ENS cells, E14 **S2**		W11vsC11 S15vsW15 >2,4	
Ascl1 (Mash1)	ENS E11-15		W11vsC11 W15vsC15	In ENS, well-studied [1, 2]
Atoh1* (Math1)	Few ENS cells E15, **S2**		S11vsC11 S15vsC15	Comment: Function in other system[3]
Cux1*	N/D	**In the ENS Fig. 2A,C; S3, 4**	S15vsS11	
Cux2*	ENS E13-15 **S2**		S15vsC15 S15vsS11	Comment: Function in other neural crest derivative[4]
Dlx1	ENS E14		S11vsC11 S15vsC15	In the ENS [5]
Dlx2	ENS E11-15		S11vsC11 S15vsC15	In the ENS [5]
Dlx3*	ENS E14 **S2**		W15vsC15	
Dlx5	ENS E14 **S2**		S11vsC11 S15vsC15	ENS transcriptome [6]
Dlx6	ENS E14 **S2**		W11vsC11 W15vsC15	ENS transcriptome [7]
Dmrta1*	N/D		W11vsC11 S15vsC15 W15vsC15 S15vsC15 >1,5	
Ebf1*	N/D	**In the ENS Fig. 2A, 3B, C; S3, 4**	W15vsW11	
Ebf3	ENS E15	**In the ENS Fig. 2A, 3B; S3, 4**	W15vsW11 S11vsC11 S15vsC15	In the ENS [8]
Ebf4*	ENS E14 **S2**		S15vsS11 W15vsW11	
Egr1	N/D		S15vsS11	In the ENS[7]
Etv1 (Er81)	ENS E15	**In the ENS Fig. 2A,C, 3B,C; S3, 4**	W15vsW11 W15vsC15 W15vsS15	In the ENS[8]
Etv5* (Erm)	ENS E13-15 **S2**		W11vsC11 W11vsW15 S15vsW15	
Foxd1*	Many bowel cells, incl. ENS E11-14	**In the ENS Fig. 2A, 3B; S3, 4**	W11vsS11 W11vsW15 W11vsC11	Comment: Function in CNS[9]
Foxd3	ENS E11-13		S15vsW15 S11vsC11	In the ENS[7, 10]

Foxj2*	Few ENS cells, E13-15 **S2**		S15vsS11 W15vsW11	
Foxj3*	Few ENS cells, E15 **S2**		S15vsS11	
Foxn2*	Few ENS cells, E14-15 **S2**		S15vsW15 S11vsC11 S15vsC15	
Foxn3*	Few ENS cells, E15 **S2**		W15vsW11	
Foxo1	Few ENS cells E15, **S2**		S15vsW15 S15vsS11	ENS progenitors *in vitro*[11]
Foxo3	Few ENS cells E15, **S2**		S15vsS11	ENS progenitors *in vitro*[11]
Gatad2a*	ENS E14 **S2**		W11vsW15 S11vsS15	
Glis2*	Many gut cells, incl. ENS E14 **S2**		W11vsC11 S11vsC11 W11vsW15 >1,4	
Hand2	N/D		W11vsC11 W15vsC15	In the ENS, well-studied [12]
Hdx*	N/D	**In the ENS Fig. 2A, 3A; S3, 4**	S11vsW11 S15vsC15 W15vsC15	
Hlf*	Few ENS cells E15, **S2**		W15vsW11 W15vsS15	
Hmx1*	N/D	**In the ENS Fig. 2A, 3B; S3, 4**	W11vsW15	Comment: Function in other neural crest derivative[13]
Hmx2*	Few ENS cells E13-15, **S2**		S15vsC15	
Hmx3	Few ENS cells E15		S15vsC15	In the ENS[8]
Homez*	ENS E13-15 **S2**		W11vsW15	
Hoxa1*	N/D		S11vsS15 S15vsW15 W15vsC15 >1,35-1,5	
Hoxa2*	ENS E14-15 **S2**		W15vsC15	
Hoxa3*	N/D	**In the ENS Fig. 2A,E, 3A; S3, 4**	W15vsC15	
Hoxa4*	E14 **S2**		W15vsC15 W15vsW11 S15vsC15	
Hoxa5	ENS E14		W15vsC15	In the ENS[7, 8]
Hoxb2*	ENS E11-15 **Fig. 2D**		W11vsC11 W15vsC15	
Hoxb3	ENS E14-15	**In the ENS Fig. 2A,E, 3A; S3, 4**	W11vsC11 W15vsC15	Hoxb3 enhancer traced in the ENS[14]

Hoxb4*	ENS E13-15<br>**S2**		W11vsC11<br>W15vsC15	
Hoxb5	ENS, E11-15		W15vsC15<br>W15vsW11<br>W15vsS15	In the ENS[15, 16]<br>HSCR locus
Hoxb6	Few ENS cells<br>E14-15<br>**Fig. 2D**		W15vsC15<br>W15vsW11<br>W15vsS15	ENS transcriptome[8]
Hoxb8*	Few ENS cells<br>E14-15<br>**Fig. 2D**		W15vsW11	
Hoxc4*	N/D	**In the ENS<br>Fig. 2A,E;<br>S3, 4**	W15vsC15<br>W15vsS15<br>W15vsW11	
Hoxc5*	N/D	**In the ENS<br>Fig. 2A,E; S3, 4**	W15vsW11	
Hoxd1*	E15<br>**S2**		S11vsC11<br>W15vsC15<br>S15vsC15	
Hoxd3*	ENS, E14-15<br>**Fig. 2D**		W11vsC11<br>W15vsW11	
Hoxd4	ENS E11-15		W15vsW11	In the ENS[15]
Jarid2	ENS E14<br>**S2**		W11vsC11<br>W15vsC15	ENS transcriptome[8]
Jazf1*	Few ENS cells<br>E15<br>**S2**		W15vsC15<br>W15vsS15<br>S15vsC15<br>W15vsW11	
Klf6*	ENS E11-14<br>**S2**		S15vsS11<br>S11vsC11	
Klf7	ENS E11-E15	**In the ENS<br>Fig. 2A, 3A, C;<br>S3, 4**	W15vsS15<br>W15vsW11<br>W11vsC11	ENS transcriptome [8]<br>Comment: Function in other system[17]
Lbh*	ENS E11<br>**S2**		S11vsC11<br>W11vsC11<br>W11vsW15<br>>1,3-1,5	Comment: Wnt signaling pathway[18]
Lef1*	general gut<br>E11		S11vsC11<br>S11vsS15<br>W11vsW15<br>>1,7-2,5	Comment: Wnt signaling pathway
Mef2c*	ENS E14	**In the ENS<br>Fig. 2A,C;<br>S3, 4**	S15vsW15<br>S15vsC15<br>S11vsC15	Comment: Function in CNS[19]
Meis2*	General gut<br>incl.d ENS<br>E11-15	**In the ENS<br>Fig. 2A,C, 3B,C;<br>S3, 4**	W11vsW15	
Myc*	ENS E14<br>**S2**		S15vsW15<br>S11vsC11<br>S11vsS15	In the adult ENS[20]
Mycn*	ENS E14<br>**S2**		S15vsC15<br>S11vsC11<br>S11vsS15	Comment: Function in CNS[21]
Myt1	Many ENS<br>cells E11-15		S11vsC11<br>S15vsC15<br>W15vsW11	In the ENS[7]<br>Comment: Function in CNS[22]

Myt1l*	ENS E13-15<br>**S2**		S11vsC11<br>S15vsC15<br>W15vsS15	Comment: Function in CNS[22]
Nr2f1	General gut<br>E11		W11vsS11<br>S11vsS15<br>S15vsW15	In the ENS[23]
NeuroD1	N/D		W11vsW15<br>W11vsS11<br>C15vsW15	In the ENS[7]
NeuroD4*	N/D	**In the ENS<br>Fig. 2A, 3A;<br>S3, 4**	W11vsC11<br>W15vsC15<br>W11vsW15	
Onecut2*	ENS E13-15	**In the ENS<br>Fig. 2A, 3A;<br>S3, 4**	W15vsW11<br>W15vsS15<br>S11vsC11	Comment: Function in CNS[24]
Patz1*	ENS E13-15<br>**S2**		W11vsW15<br>W15vsC15	
Pax3*	N/D	**In the ENS<br>Fig. 2A; S3, 4**	S11vsC11<br>W11vsC11	$Pax3^{-/-}$ mutant display ENS defects Not previously described in the ENS. [25]
Phox2a	ENS E11-15		S15vsS11	In the ENS, well-studied[26]
Phox2b	ENS E11-15		W11vsC11<br>W15vsC15	In the ENS, well-studied[26]
Pbx2*	ENS E11-14<br>**S2**		S11vsS15<br>W11vsW15	
Pbx3	Few ENS cells<br>E13-15	**In the ENS<br>Fig. 2A, 3B;<br>S3, 4**	W15vsS15<br>W15vsW11<br>W15vsC15	In the ENS[27]
Pknox1*	ENS E15<br>**S2**		S15vsS11<br>W15vsW11	
Runx1*	ENS E13-15<br>**S2**		W15vsW11	Comment:Function in other neural crest derivative[28]
Satb1	ENS E13-15	**In the ENS<br>Fig. 2A,C, 3B,C;<br>S3, 4**	S15vsS11<br>W11vsC11<br>W15vsC15	ENS transcriptome[8]
Satb2*	ENS E15	**In the ENS<br>Fig. 2A, 3B;<br>S3, 4**	S15vsS11<br>W15vsW11<br>W11vsS11	
Sox2	ENS E11-15		S11vsC11<br>S15vsC15	In $Sox10^{+}$ cells[8, 29]
Sox4*	ENS E13-15	**In the ENS<br>Fig. 2A,D, 3A;<br>S3, 4**	W11vsC11<br>S11vsC11<br>W15vsC15	
Sox5	ENS E11-15	**In the ENS<br>Fig. 2A,D;<br>S3, 4**	S11vsC11<br>W11vsW15<br>S15vsW15	ENS transcriptome[30]
Sox6*	N/D	**In the ENS<br>Fig. 2A,D, 3B,C,<br>4**	S15vsS11	
Sox8	ENS E11-15		S11vsC11<br>S15vsC15	In $Sox10^{+}$ cells[31]
Sox9*	N/D	**In the ENS<br>Fig. 2A,D, 3A;**	W11vsW15<br>S11vsS15	

		**S3, 4**		
Sox10	ENS E11-15		S11vsC11<br>W11vsW15<br>S11vsW11	In ENS progenitors[32]
Sox11*	ENS E13	**In the ENS<br>Fig. 2A,D, 3A;<br>S3, 4**	W15vsC15<br>S15vsC15	
St18	N/D		S15vsW15<br>S11vsC11<br>S15vsC15<br>>1,3-7,2	ENS transcriptome[30]<br>Function in CNS[22]
Tbx2	ENS E11-13<br>**S2**		S11vsC11<br>S15vsC15	ENS transcriptome[8]
Tbx3	ENS E11-15	**In the ENS<br>Fig. 2A,C, 3A;<br>S3, 4**	W15vsW11<br>W15vsC15<br>W11vsC11	In the ENS[7, 8]
Tbx20*	ENS E11-15<br>**S2**		S11vsC11<br>S15vsC15<br>S11vsS15<br>>1,6-1,8	Comment: Function in CNS differentiation[33]
Tcf4	ENS E11-15<br>**S2**		W11vsC11<br>W15vsC15<br>W15vsW11	ENS transcriptome[8] HSCR locus[34]
Tcf7l2*	ENS E15<br>**S2**		W15vsS15<br>S15vsS11	
Tcf12*	ENS E14<br>**S2**		S15vsC15	
Tcf21	ENS E14<br>**S2**		S11vsS15<br>C15vsW15<br>C11vsW11	ENS transcriptome[8]
Tcf25*	ENS E11-15<br>**S2**		S11vsC11<br>W11vsC11	
Tlx2	ENS E11-15		S15vsC15<br>W15vsC15	In the ENS, well-studied[35]
Tlx3*	ENS E13-15	**In the ENS<br>Fig. 2A, 3A;<br>S3, 4**	S15vsC15	Comment: Function in other neural crest derivative[13]
Tshz1*	ENS E11-15<br>**S2**		S15vsS11<br>S11vsC11	
Tshz3*	N/D	**In the ENS<br>Fig. 2A, 3B;<br>S3, 4**	W15vsS15<br>W15vsW11	
Zeb1*	N/D	**In the ENS<br>Fig. 2A,C, 3A;<br>S3, 4**	S15vsS11<br>W15vsW11	
Zeb2	ENS E11-15		W11vsC11<br>S11vsC11<br>W11vsW15	In ENS HSCR locus[36, 37]
Zfhx2*	ENS E13-15<br>**S2**		W11vsC11<br>W15vsW11	
Zfhx4*	ENS E11-15	**In the ENS<br>Fig. 2A,C, 3A;<br>S3, 4**	W15vsW11	
Zfp7*	ENS E14<br>**S2**		W11vsC11<br>W15vsC15	
Zfp113*	ENS E14		W15vsC15	

	**S2**		S15vsC15	
Zfp184*	ENS E14<br>**S2**		W15vsC15<br>S15vsC15	
Zfp191*	ENS E13-15<br>**S2**		W11vsC11<br>W15vsC15<br>S15vsC15	
Zfp462*	ENS E14<br>**S2**		W15vsC15<br>S15vsC15	
Zfp647*	ENS E14<br>**S2**		W15vsC15<br>S15vsC15	
Zfp689	ENS E14<br>**S2**		W11vsC11<br>W15vsC15	
Zhx3*	ENS E14<br>**S2**		S15vsS11<br>W15vsW11	
Zik1*	ENS E14<br>**S2**		W15vsC15<br>S15vsC15<br>S11vsS15	
Zkscan1*	ENS E15<br>**S2**		W11vsC11<br>W15vsC15	

Supplementary Table 6. Up-to date list of transcription factors identified in the developing ENS.
*: genes previously not described in the developing mouse ENS. Expression was confirmed by IHC and/or convincing staining in online expression resources (bold black text). Enrichment in enteric populations of >2 and/or significant enrichment in at least 3 enteric populations were also considered as validation when quality was suboptimal in Online Expression Resources. Online ISH Resources = Allen Brain Atlas and GenePaint. ISH= *In situ* Hybridisation; IHC= Immunohistochemistry; S: Supplementary Figure; N/D: no (or suboptimal) data.

REFERENCES:

1. Blaugrund E, Pham TD, Tennyson VM, et al. Distinct subpopulations of enteric neuronal progenitors defined by time of development, sympathoadrenal lineage markers and Mash-1-dependence. Development 1996;122:309-20.
2. Memic F, Knoflach V, Sadler R, et al. Ascl1 Is Required for the Development of Specific Neuronal Subtypes in the Enteric Nervous System. J Neurosci 2016;36:4339-50.
3. Costa A, Powell LM, Lowell S, et al. Atoh1 in sensory hair cell development: constraints and cofactors. Semin Cell Dev Biol 2017;65:60-68.
4. Bachy I, Franck MC, Li L, et al. The transcription factor Cux2 marks development of an A-delta sublineage of TrkA sensory neurons. Dev Biol 2011;360:77-86.
5. Qiu M, Bulfone A, Martinez S, et al. Null mutation of Dlx-2 results in abnormal morphogenesis of proximal first and second branchial arch derivatives and abnormal differentiation in the forebrain. Genes Dev 1995;9:2523-38.

6. **Iwashita T, Kruger GM**, Pardal R, et al. Hirschsprung disease is linked to defects in neural crest stem cell function. Science 2003;301:972-6.
7. Vohra BP, Tsuji K, Nagashimada M, et al. Differential gene expression and functional analysis implicate novel mechanisms in enteric nervous system precursor migration and neuritogenesis. Dev Biol 2006;298:259-71.
8. Heanue TA, Pachnis V. Expression profiling the developing mammalian enteric nervous system identifies marker and candidate Hirschsprung disease genes. Proc Natl Acad Sci U S A 2006;103:6919-24.
9. Takahashi H, Sakuta H, Shintani T, et al. Functional mode of FoxD1/CBF2 for the establishment of temporal retinal specificity in the developing chick retina. Dev Biol 2009;331:300-10.
10. Mundell NA, Plank JL, LeGrone AW, et al. Enteric nervous system specific deletion of Foxd3 disrupts glial cell differentiation and activates compensatory enteric progenitors. Dev Biol 2012;363:373-87.
11. Srinivasan S, Anitha M, Mwangi S, et al. Enteric neuroblasts require the phosphatidylinositol 3-kinase/Akt/Forkhead pathway for GDNF-stimulated survival. Mol Cell Neurosci 2005;29:107-19.
12. Lei J, Howard MJ. Targeted deletion of Hand2 in enteric neural precursor cells affects its functions in neurogenesis, neurotransmitter specification and gangliogenesis, causing functional aganglionosis. Development 2011;138:4789-800.
13. Furlan A, Lubke M, Adameyko I, et al. The transcription factor Hmx1 and growth factor receptor activities control sympathetic neurons diversification. EMBO J 2013;32:1613-25.
14. **Chan KK, Chen YS**, Yau TO, et al. Hoxb3 vagal neural crest-specific enhancer element for controlling enteric nervous system development. Dev Dyn 2005;233:473-83.
15. Gaunt SJ, Krumlauf R, Duboule D. Mouse homeo-genes within a subfamily, Hox-1.4, -2.6 and -5.1, display similar anteroposterior domains of expression in the embryo, but show stage- and tissue-dependent differences in their regulation. Development 1989;107:131-41.
16. Pitera JE, Smith VV, Thorogood P, et al. Coordinated expression of 3' hox genes during murine embryonal gut development: an enteric Hox code. Gastroenterology 1999;117:1339-51.
17. Laub F, Dragomir C, Ramirez F. Mice without transcription factor KLF7 provide new insight into olfactory bulb development. Brain Res 2006;1103:108-13.
18. Rieger ME, Sims AH, Coats ER, et al. The embryonic transcription cofactor LBH is a direct target of the Wnt signaling pathway in epithelial development and in aggressive basal subtype breast cancers. Mol Cell Biol 2010;30:4267-79.
19. **Li H, Radford JC**, Ragusa MJ, et al. Transcription factor MEF2C influences neural stem/progenitor cell differentiation and maturation in vivo. Proc Natl Acad Sci U S A 2008;105:9397-402.
20. Parr EJ, Gibson AW, Sharkey KA. c-Myc antigens in the mammalian enteric nervous system. Neuroscience 1994;58:807-16.
21. Xue XY, Harris WA. Using myc genes to search for stem cells in the ciliary margin of the Xenopus retina. Dev Neurobiol 2012;72:475-90.

22. Kameyama T, Matsushita F, Kadokawa Y, et al. Myt/NZF family transcription factors regulate neuronal differentiation of P19 cells. Neurosci Lett 2011;497:74-9.
23. Bergeron KF, Nguyen CM, Cardinal T, et al. Upregulation of the Nr2f1-A830082K12Rik gene pair in murine neural crest cells results in a complex phenotype reminiscent of Waardenburg syndrome type 4. Dis Model Mech 2016;9:1283-1293.
24. Espana A, Clotman F. Onecut factors control development of the Locus Coeruleus and of the mesencephalic trigeminal nucleus. Mol Cell Neurosci 2012;50:93-102.
25. Lang D, Chen F, Milewski R, et al. Pax3 is required for enteric ganglia formation and functions with Sox10 to modulate expression of c-ret. J Clin Invest 2000;106:963-71.
26. Young HM, Ciampoli D, Hsuan J, et al. Expression of Ret-, p75(NTR)-, Phox2a-, Phox2b-, and tyrosine hydroxylase-immunoreactivity by undifferentiated neural crest-derived cells and different classes of enteric neurons in the embryonic mouse gut. Dev Dyn 1999;216:137-52.
27. Di Giacomo G, Koss M, Capellini TD, et al. Spatio-temporal expression of Pbx3 during mouse organogenesis. Gene Expr Patterns 2006;6:747-57.
28. **Hadjab S, Franck MC**, Wang Y, et al. A local source of FGF initiates development of the unmyelinated lineage of sensory neurons. J Neurosci 2013;33:17656-66.
29. Heanue TA, Pachnis V. Prospective identification and isolation of enteric nervous system progenitors using Sox2. Stem Cells 2011;29:128-40.
30. Lasrado R, Boesmans W, Kleinjung J, et al. Lineage-dependent spatial and functional organization of the mammalian enteric nervous system. Science 2017;356:722-726.
31. Maka M, Stolt CC, Wegner M. Identification of Sox8 as a modifier gene in a mouse model of Hirschsprung disease reveals underlying molecular defect. Dev Biol 2005;277:155-69.
32. Southard-Smith EM, Kos L, Pavan WJ. Sox10 mutation disrupts neural crest development in Dom Hirschsprung mouse model. Nat Genet 1998;18:60-4.
33. Takeuchi JK, Mileikovskaia M, Koshiba-Takeuchi K, et al. Tbx20 dose-dependently regulates transcription factor networks required for mouse heart and motoneuron development. Development 2005;132:2463-74.
34. Jiang Q, Ho YY, Hao L, et al. Copy number variants in candidate genes are genetic modifiers of Hirschsprung disease. PLoS One 2011;6:e21219.
35. Shirasawa S, Yunker AM, Roth KA, et al. Enx (Hox11L1)-deficient mice develop myenteric neuronal hyperplasia and megacolon. Nat Med 1997;3:646-50.
36. Van de Putte T, Maruhashi M, Francis A, et al. Mice lacking ZFHX1B, the gene that codes for Smad-interacting protein-1, reveal a role for multiple neural crest cell defects in the etiology of Hirschsprung disease-mental retardation syndrome. Am J Hum Genet 2003;72:465-70.
37. **Watanabe Y, Stanchina L**, Lecerf L, et al. Differentiation of Mouse Enteric Nervous System Progenitor Cells Is Controlled by Endothelin 3 and Requires Regulation of Ednrb by SOX10 and ZEB2. Gastroenterology 2017;152:1139-1150 e4.

Author names in bold designate shared co-first authorship.
