# Supplementary material for: Transcription and Signaling Regulators in Developing Neuronal Subtypes of Mouse and Human Enteric Nervous System
Source: Gastroenterology. 2018 Feb;154(3):624–36. doi: 10.1053/j.gastro.2017.10.005 (PMC6381388; doi:10.1053/j.gastro.2017.10.005)
Supplement: Supplementary Table 7 [file mmc17.pdf]

SUPPLEMENTARY TABLE 7.
Signaling pathways in the developing ENS

Gene:	Online ISH resources:	IHC:	Current screen:	Comment/ References:
***Activin A signaling**				Comment: Function in CNS[1]
Inhba* (homodimerize to Activin A)	mesenchyme E11-13	**In the ENS, Fig 5B, 6; S6**	C11vsW11 C15vsW15	
Inha* (heterodimer with INHBA to form Inhibin)	N/D	**In the ENS, Fig 6; S6, 7**	W15vsC15 W15vsS15 W15vsW11	
Acvr1b* (*Alk4*)	ENS E14-15 Fig. 5B		W15vsC15 S15vsC15	
Acvr2a*	ENS E13-15	**In the ENS, Fig 5B, 6; S6, 7**	W15vsC15 W15vsW11 S15vsC15	
***ALK/LTK signaling**				Comment: Function in neural crest[2]
Alk	ENS E15 **S5**		W15vsC15 W15vsS15 >2,5	In bowel wall[3]
Ltk*	ENS E13-E15 **Fig. 5C**		W15vsC15 W15vsS15	
***Aryl-hydrocarbon signaling**				Comment: Function in CNS[4]
Ahr*	General gut E14	**In the ENS, Fig. 5C, 6; S6, 7**	W11vsC11 S15vsC15 S15vsS11	
Arnt2*	ENS E13-15 **S5**		W15vsC15 S15vsC15	
**BMP signaling**				Well-studied in ENS[5]
Bmp1*	General gut E13-15, S4		C15vsW15	
Bmp2	mesenchyme E14		C15vsW15	In bowel[6]
Bmp4	Serosa, mesenchyme E14		C11vsW11 C15svW15	In bowel[6]
Bmp5*	mesenchyme E11-15, **S5**		C11vsW11 C15svW15	In adult colon[7]
Bmp6*	mucosa E15 **S5**		C15vsW15	
***Bmp7*^ENS^**	ENS and mucosa E15 **Fig. 5B**		S11vsC11 S15vsC15 >2.3	In chick mesenchyme[8] Comment: Function in CNS[9]
Gdf6* **(*Bmp13*)**	General gut, close to ENS E11-15 **S5**		C11vsW11 C15vsW15	

Gdf10* **(*Bmp3b*)**	ENS and serosa E13-15	**In the ENS Fig. 5B, 6; S6, 7**	W11vsC11 C15vsW15	Comment: Function in other system[10]
Acvr1a* **(*Alk2*)**	ENS E15	**In the ENS, Fig. 5B, 6; S6, 7**	S11vsC11 S15vsS11	
Bmpr1b **(*Alk6*)**	N/D		W15vsC15 W15vsW11 W15vsS15	In ENS and mesenchyme (RT-PCR)[6]
Acvr2a*	ENS and mucosa E13-15	**In the ENS, Fig. 5B, 6; S6, 7**	W15vsC15 S15vsC15	
Bmpr2	General gut E11-15		W15vsW11	In ENS (RT-PCR)[6]
***Chemokine signaling**				Comment: Function in CNS[11-13]
Cxcl12*	serosa and mesenchyme E11-E15	**serosa and mucosa Fig. 5D, 6; S6, 7**	C15vsW15	
Cxcr4*	ENS E11-E15	**In the ENS, Fig. 5D, 6; S6, 7**	W15vsC15 W15vsW11 W15vsS15	Cxcr4$^{-/-}$ mutant display ENS migration defect[14] Not detected in developing ENS before, but in adult ENS ganglia[15]
***CCN family matricellular modulators**				Comment: Function in other systems[16]
Ctgf **(*Ccn2)****	ENS and mucosa E13-E15	**In the ENS, Fig. 5B, 6; S6, 7**	S15vsC15 S15vsS11 S15vsW15	
Ngfr (p75)	ENS E11-E15		W11vsC11 W15vsC15	In the ENS[17]
Itga2	N/D		S15vsC15 S15vsS11 S15vsW15 >1,7-2,7	
**Endothelin signaling**				Well-studied in ENS[18]
Edn1*	mucosa E14 **S5**		C11vsW11	
Edn3	mesenchyme E14		C11vsW11 C15vsW15	In gut mesenchyme[19] HSCR locus[20]
Ednrb	ENS and mesenchyme E13-15		W11vsC11 W15vsC15	In the ENS[21] HSCR locus[20]
***Eph/ephrin signaling**				Comment: Function in other systems[22]
Efna1*	mesenchyme E11-15, **S4**		C15vsW15	
Efna4*	mesenchyme mucosa E11-15 **S4**		W11vsW15	

Efna5*	mesenchyme, ENS E13-15 **Fig. 5B**		W15vsW11	
Efnb1*	mesenchyme E11-E14, **S5**		C15vsW15	
Efnb2*	mesenchyme E11-15, **S5**		C11vsW11 C15vsS15	
Efnb3*	ENS E13-15 **Fig. 5B**		W15vsC15 W15vsW11 W15vsS15	
Epha5*	ENS E14-E15 **Fig. 5B**		W15vsC15 W15vsW11 W15vsS15	
Epha6*	N/D		W15vsC15 W15vsW11 W15vsS15 >2.2-3.6	
Ephb2*	ENS E14-15 **Fig. 5B**		S11vsC11	
***FGF signaling**				Comment: Function in other system[23, 24]
Fgf1*	General gut E11-15	**In the ENS, Fig. 5B, 6; S6, 7**	W15vsW11	
Fgf7*	N/D		S15vsW15 W11vsC11 S11vsC11 >1,5	
Fgf9	serosa and mesenchyme E11-14		C11vsW11 C15vsW15	In gut mesenchyme[25]
Fgf10	Stomach and hindgut mesenchyme E11-E15		C11vsW11 C15vsW15	In gut mesenchyme[26]
Fgf13	ENS E14		W11vsC11 W15vsC15	In the ENS[27, 28]
Ffgr1*	serosa mesenchyme E11-15	**In the ENS, Fig. 5B, 6; S6, 7**	C15vsW15	
Fgfr2	bowel wall E14 **S5**		C11vsW15 C15vsW15	
***FLRT/UNC-5D signaling**				Comment: Function in CNS[29, 30]
Flrt1	Mesentery, ENS? E14 **S5**		W15vsC15 S15vsS11 S15vsC15 >1,26	ENS transcriptome[31]
Flrt2*	mesenchyme and ENS E14 **Fig. 5B**		W11vsW15 W15vsS15 >1,4	
Flrt3*	mesenchyme E14, **S5**		C11vsW11 C15vsW15	
Lphn1* **(bind UNC-5D and FLRT)**	N/D		W15vsC15 W15vsS15 W15vsW11	

			>1,49	
Unc5d*	Few ENS cells E13-15 **Fig. 5B**		W15vsC15 W15vsS15 W15vsW11	
**GDNF/Nrtn signaling**				Well-studied in ENS[18]
Gdnf	mesenchyme E11-15		C11vsW11 C15vsW15	In gut mesenchyme HSCR locus[32]
Nrtn	mesenchyme E14		N/D	In the ENS[33, 34]
Ret	ENS E11-15		W11vsC11 W15vsC15	In the ENS HSCR locus[35]
Gfra1	ENS and mesenchyme E11-15		W11vsC11 W15vsC15	In the ENS[36]
Gfra2	ENS and mesenchyme E13-15		W11vsC11 W15vsC15	In the ENS[37]
**Hedgehog signaling**				Well-studied in ENS[38]
Ihh	mucosa E14		N/D	In mucosa[39]
Shh	mucosa E11-15		N/D	In mucosa[39]
Dhh	N/D		S15vsC15	In Schwann-cell derived ENS[40]
Ptch1	ENS and muscle E11-14		W11vsW15	In the ENS[39]
Smo	general gut E13		W11vsW15	In the ENS[41]
Gas1	serosa, muscle and ENS E15		C15vsW15	In the ENS[41]
***IGF/Insulin signaling**				Comment: Function in other systems[42]
Igf1*	mesenchyme E11-15	**Mesenchym e and ENS. Fig. 5B, 6; S6, 7**	C11vsW11 C15vsW15	In adult ENS[43]
Igf2*	mucosa and serosa E11-15 **S5**		C11vsW11 C15vsW15	
Igf1r*	mesenchyme E11-15	**In the ENS, Fig. 5B, 6; S7**	W11vsC11 W15vsC15	In adult ENS[43]
Insr*	Few ENS cells E13-E15, **S5**		W15vsC15	
***Midkine signaling**				Comment: Function in other systems[44]
Ptn	Mesenchyme E14		C11vsW11 C15vsW15	In mesenchyme[45]
Mdk*	Mesenchyme ENS? E14	**In the ENS, Fig. 5B, 6; S6, 7**	C11vsW11 C15vsW15 W11vsW15	
Ptprz1	ENS E13-15		W11vsC11 W15vsC15	ENS

	**S5**		W15vsW11 >3-30	transcriptome[31]
Itga4	ENS E11-14 **S5**		W11vsC11 W11vsW15	In human iPS-derived ENSCs[31, 46]
Itga6	ENS and mucosa E14 **Fig. 5B**		W11vsC11 W15vsC15 W11vsW15	ENS transcriptome[31]
***Cspg5* (*Ngc*)**	ENS E14?		W11vsC11 W15vsC15 W15vsW11	In the ENS[47]
**Netrin signaling**				Studied in ENS[18]
Ntn1	mesenchyme E13-15			In the ENS[48]
Ntn3	mesenchyme E13-15			In the ENS[48]
Ntn4*	Few ENS cells E15, **S5**		W15vsC15 S15vsC15	
Ntng1*	ENS E13-15 **Fig. 5C**		W15vsC15 W15vsS15 W15vsW11	
Lrrc4c*	N/D		W11vsC11 W15vsC15 W15vsW11 >4-11	Comment: Function in CNS[49]
Unc5d*	Few ENS cells E13-15 **Fig 5B**		W15vsC15 W15vsS15 W15vsW11 >2	
Dscam	N/D		W11vsC11 W15vsC15 W15vsS15	In the ENS, HSCR locus[50]
Dcc	N/D		W11vsC11 W15vsC15 W15vsS15	In the ENS[51]
**Neuregulin signaling**				Studied in ENS[18]
Nrg1ENS***	ENS and mesenchyme E13-E15, **S5**		W15vsC15 W15vsS15	In mesenchyme[52] HSCR locus[53] In adult ENS[54]
Nrg3ENS***	N/D		W15vsC11 S15vsC15 W15vsW11 >4.5-6.2	HSCR locus[55, 56]
Erbb3	ENS E13-15		W11vsC11 W15vsC15 S15vsW15	In the ENS[52]
**Neurotrophin signaling**				Well-studied in ENS[57]
Bdnf	mesenchyme E14		C11vsS11 C15vsS15	In the gut[58]
***Ntf3* (*Nt3*)**	mesenchyme E11-E15		C11vsW11 C15vsW15	In mesenchyme[59]
***Ntf5** (*Nt4*)**	General gut E14 **S5**		C15vsW15	

Ngf*	N/D		C15vsW15 >2,5	In adult ENS[60]
***Ntrk2* (TrkB)**	Few ENS cells? E14			In the ENS (E16)[61]
***Ntrk3* (TrkC)**	Mesenchyme and ENS? E14		W15vsS15 W15vsW11	In the ENS[59]
***Ngfr* (p75)**	ENS E11-15		W11vsC11 W15vsC15	In the ENS[17]
**Notch signaling**				Studied in ENS[62]
Dll1	ENS E11-E15		W11vsC11 W15vsC15	In the ENS, [62]
Dll3	N/D		W15vsC15 S15vsW15 S11vsC11	In the ENS[62, 63]
Dll4	N/D		N/D	In the ENS[62]
Jag1	mesenchyme, ENS? E14		S15vsW15 W11vsW15 W15vsC15	In the ENS[62]
Notch1	General gut incl. ENS E11-15		S11vsC11 S15vsC15	In the ENS[62]
Notch2*	General gut incl. ENS E11-E14 **S5**		S11vsC11 S15vsS11 S15vsW15	
Notch4	N/A			In the ENS[62]
**Prokineticin signaling**				Studied in ENS[64]
Prok-1	N/D		N/D	In the mesenchyme[64]
Prokr1	N/D		W15vsS15 W15vsW11	In the ENS[64]
**RA signaling**				Studied in ENS[65]
Rara	General gut incl. ENS E11-14		S11vsC11 S15vsC15	In the ENS[65]
Rarb	ENS E15		S11vsC11 W15vsC15	In the ENS[27, 65]
Rarg	General gut incl. ENS E13-15		N/D	In the ENS[65]
Rxra	General gut incl. ENS E11-15		W11vsC11 C15vsW15	In the ENS[65]
Rxrb	General gut incl. ENS E11-14		N/D	In the ENS[65]
Rxrg	General gut incl. ENS E11-14		W11vsW15	In the ENS[65]
**Semaphorin signaling**				Sema3 studied in ENS[66]
Sema3a	mesenchyme E11-15		C11vsW11 C15vsW15	In the bowel[66] HSCR locus[67]
Sema3b*	mucosa and ENS E14-E15 **S5**		W15vsC15 S11vsC11 S15vsC15 >1,25-1,45	
Sema3c	mucosa and ENS E11-15		S11vsW11 W15vsC15	In the ENS[68]
Sema3d	mesenchyme		W11vsC11	In the ENS[68]

	ENS E11-15		C15vsW15	
Sema4d*	ENS E15<br>**Fig 5B**		W15vsC15<br>W15vsW11	Comment: Function in CNS[69]
Sema4f*	ENS E14<br>**S5**		W15vsC15<br>W15vsW11<br>S15vsC15	
Sema4g*	ENS E14<br>**S5**		W15vsC15<br>W15vsS15	
Sema5a*	mesenchyme E11-15, **S5**		C11vsW11<br>C15vsW15	
Sema6a*	ENS E13-15<br>**S5**		W15vsW11<br>W15vsS15	
Sema6d*	mesenchyme E11-15, **S5**		C15vsW15<br>W15vsW11	
Plxna1	mesenchyme E15		C15vsW15	In the ENS[68]
Plxna2	ENS E15		C11vsW11<br>W15vsC15	In the ENS[68]
Plxna3	ENS E13-15		W15vsC15<br>W15vsS15	In the ENS[68]
Plxna4	N/D		W11vsC11<br>W15vsC15<br>W15vsS15<br>>4-9	In the ENS[68]
Plxnb1*	mesenchyme and ENS E13-15	**In the ENS, Fig. 5B, 6; S6**	W11vsC11<br>W15vsC15<br>W15vsW11	
Plxnb3*	N/D		W15vsC15<br>S15vsC15<br>W15vsW11<br>>1,3-1,5	
Nrp1	ENS E11-15		W11vsC11<br>W11vsW15	In the ENS[68]
Nrp2	ENS E14		W15vsW11	In the ENS[68]
***L1cam* (co-receptor**	ENS E11-15		W11vsC11<br>W15vsS15	In the ENS[66]
***Nrcam* (co-receptor)**	ENS E11-15		W11vsC11<br>W15vsC15	In the chick ENS[70]
**Slit signaling**				Studied in ENS[71]
Slit1	ENS and mesenchyme E14-15		W15vsC15<br>W15vsW11	In the bowel wall[71]
Slit2	ENS and mesenchyme E14		C11vsW11<br>W15vsW11	In the ENS[71]
Slit3	ENS and mesenchyme E14		W11vsC11<br>W15vsC15	In the bowel wall[71]
Robo2	mesenchyme, hindgut mucosa E14		C11vsW11<br>C15vsW15<br>W15vsS15	In chick ENS[72]
***Slitrk signaling**				Comment: Function in CNS[73-75]
Bdnf	mesenchyme E14		C11vsS11<br>C15vsS15	Comment: Slitrk5 enhance TrkB

	**S5**			mediated signaling triggered by BDNF[74]
Ptprd*	mesenchyme and ENS **Fig 5B**		C11vsW11 C15vsW15	
Ptpro*	ENS E14 **S5**		W11vsC11 W15vsC15 W15vsS15	
Slitrk1*	ENS E13-15 **S5**		W15vsC15	
Slitrk2*	ENS? E14	**In the ENS Fig 6; S7**	W11vsC11 W15vsC15	
Slitrk3*	ENS and mesenchyme, E14	**In the ENS Fig. 5B, 6; S6, 7**	W15vsC15	
Slitrk5*	whole gut incl. ENS E14 **S5**		W15vsC15 W15vsW11	
Slitrk6	ENS and muscle E14		W11vsC11 W15vsC15	In the bowel wall[76]
**TGFβ signaling**				Comment: Function in CNS[77]
Tgfb1*	mesenchyme E11-15, **S5**		C11vsW11 C15vsW15	
Tgfb2	ENS E14-15	**In the ENS Fig 5B, 6; S6, 7**	W11vsC11 W15vsC15 S15vsS11	In the ENS[27]
Tgfb3*	serosa and mesenchyme E14, **S5**		C11vsW11 C15vsW15	
Tgfbr1*	Gut wall and mucosa, E14	**In the ENS; Fig 6; S6, 7**		
Tgfbr2*	Gut wall E14	**In the ENS, Fig 5B, 6; S6, 7**	C15vsW15	
**Wnt signaling (canonical*)**				Non-canonical pathway is studied in ENS[78]
Wnt2b* (Canonical)	serosa and mesenchyme E11-15 **Fig. 5D**		C11vsW11 C15vsW15 W11vsW15	In the adult gut[79]
Wnt4	Hindgut mucosa E11-15		C11vsW11 C15vsW15	In mucosa[80]
Wnt5a	mesenchyme E11-15		C11vsW11 C15vsW15 W11vsW15	In mesenchyme[80]
Wnt5b	mesenchyme E13		C11vsW11 C15vsW15	In serosa[80]
Wnt6	Esophagus E14			In serosa[80]
Wnt11	Esophagus E14		C11vsW11	In mesenchyme[80]
Lrp5* (Canonical)	ENS E11-14 **Fig. 5D**		W11vsC11 S11vsC11	

			S11vsS15	
Fzd3	ENS E11-15		W11vsC11<br>W15vsC15	In the ENS[78]
Celsr2*	ENS E14-15<br>**S5**		S11vsC11<br>S15vsC15	Comment: Function in non-canonical Wnt signaling[81]
Celsr3	ENS 13-15		S11vsC11<br>S15vsC15	In the ENS[78]
**Receptors-Miscellaneous**				
Lrp8*	ENS E14, **S5**		W15vsC15	
Lrp11*	ENS<br>E15	**In the ENS**<br>**S6, 7**	W15vsC15<br>S15vsS15	
Ptprg*	N/D		W11vsC11<br>W15vsC15<br>W15vsS15<br>>1,45	
Ptprj*	N/D		S11vsC11<br>S15vsC15<br>S15vsW15<br>>1,5	
Ptprm*	Mesenchyme and ENS E14<br>**S5**		S11vsC11<br>S15vsW15	
Ptprn*	ENS E14<br>**S5**		W11vsC11<br>W15vsS15	
Ptprn2*	N/D		W15vsC15<br>W15vsW15<br>W15vsS15<br>>1,8	
Ptprr*	N/D		W11vsC11<br>W15csS15<br>W15vsW11<br>>4,5	
Ptprs*	ENS E14<br>**S5**		W15vsC15<br>W15vsW11	

Supplementary Table 7. Up-to-date list of signaling factors (ligands and receptors) identified in or around the developing ENS.
Note that ligand-receptor interactions suggested in this table are based on experiments in other tissue.
*: genes previously not described in the developing mouse ENS. Expression was confirmed by IHC and/or convincing staining in online expression resources (bold black text). Enrichment in enteric populations of >2 and/or significant enrichment in at least 3 enteric populations were also considered as validation when quality was suboptimal in Online Expression Resources. Online ISH Resources = Allen Brain Atlas and GenePaint. ISH= *In situ* Hybridisation; IHC= Immunohistochemistry; S: Supplementary Figure; N/D: no (or suboptimal) data; HSCR: Hirschsprung disease

REFERENCES:

1. Davis AA, Matzuk MM, Reh TA. Activin A promotes progenitor differentiation into photoreceptors in rodent retina. Mol Cell Neurosci 2000;15:11-21.
2. Fadeev A, Krauss J, Singh AP, et al. Zebrafish Leucocyte tyrosine kinase controls iridophore establishment, proliferation and survival. Pigment Cell Melanoma Res 2016;29:284-96.
3. Vernersson E, Khoo NK, Henriksson ML, et al. Characterization of the expression of the ALK receptor tyrosine kinase in mice. Gene Expr Patterns 2006;6:448-61.
4. Dever DP, Adham ZO, Thompson B, et al. Aryl hydrocarbon receptor deletion in cerebellar granule neuron precursors impairs neurogenesis. Dev Neurobiol 2016;76:533-50.
5. Chalazonitis A, Kessler JA. Pleiotropic effects of the bone morphogenetic proteins on development of the enteric nervous system. Dev Neurobiol 2012;72:843-56.
6. Chalazonitis A, D'Autreaux F, Guha U, et al. Bone morphogenetic protein-2 and -4 limit the number of enteric neurons but promote development of a TrkC-expressing neurotrophin-3-dependent subset. J Neurosci 2004;24:4266-82.
7. Wu M, Chen W, Mi J, et al. Expression analysis of BMP2, BMP5, BMP10 in human colon tissues from Hirschsprung disease patients. Int J Clin Exp Pathol 2014;7:529-36.
8. Goldstein AM, Brewer KC, Doyle AM, et al. BMP signaling is necessary for neural crest cell migration and ganglion formation in the enteric nervous system. Mech Dev 2005;122:821-33.
9. Ohyama K, Ellis P, Kimura S, et al. Directed differentiation of neural cells to hypothalamic dopaminergic neurons. Development 2005;132:5185-97.
10. **Li S, Nie EH**, Yin Y, et al. GDF10 is a signal for axonal sprouting and functional recovery after stroke. Nat Neurosci 2015;18:1737-45.
11. Abe P, Molnar Z, Tzeng YS, et al. Intermediate Progenitors Facilitate Intracortical Progression of Thalamocortical Axons and Interneurons through CXCL12 Chemokine Signaling. J Neurosci 2015;35:13053-63.
12. Tiveron MC, Cremer H. CXCL12/CXCR4 signalling in neuronal cell migration. Curr Opin Neurobiol 2008;18:237-44.
13. Yang S, Edman LC, Sanchez-Alcaniz JA, et al. Cxcl12/Cxcr4 signaling controls the migration and process orientation of A9-A10 dopaminergic neurons. Development 2013;140:4554-64.
14. Nishiyama C, Uesaka T, Manabe T, et al. Trans-mesenteric neural crest cells are the principal source of the colonic enteric nervous system. Nat Neurosci 2012;15:1211-8.
15. Ding X, Zhao Z, Duan W, et al. Expression patterns of CXCR4 in different colon tissue segments of patients with Hirschsprung's disease. Exp Mol Pathol 2013;95:111-6.
16. Malik AR, Liszewska E, Jaworski J. Matricellular proteins of the Cyr61/CTGF/NOV (CCN) family and the nervous system. Front Cell Neurosci 2015;9:237.

17. Young HM, Ciampoli D, Hsuan J, et al. Expression of Ret-, p75(NTR)-, Phox2a-, Phox2b-, and tyrosine hydroxylase-immunoreactivity by undifferentiated neural crest-derived cells and different classes of enteric neurons in the embryonic mouse gut. Dev Dyn 1999;216:137-52.
18. Obermayr F, Hotta R, Enomoto H, et al. Development and developmental disorders of the enteric nervous system. Nat Rev Gastroenterol Hepatol 2013;10:43-57.
19. Leibl MA, Ota T, Woodward MN, et al. Expression of endothelin 3 by mesenchymal cells of embryonic mouse caecum. Gut 1999;44:246-52.
20. Kusafuka T, Puri P. Mutations of the endothelin-B receptor and endothelin-3 genes in Hirschsprung's disease. Pediatr Surg Int 1997;12:19-23.
21. Nataf V, Lecoin L, Eichmann A, et al. Endothelin-B receptor is expressed by neural crest cells in the avian embryo. Proc Natl Acad Sci U S A 1996;93:9645-50.
22. Cramer KS, Miko IJ. Eph-ephrin signaling in nervous system development. F1000Res 2016;5.
23. **Hadjab S, Franck MC**, Wang Y, et al. A local source of FGF initiates development of the unmyelinated lineage of sensory neurons. J Neurosci 2013;33:17656-66.
24. Raju R, Palapetta SM, Sandhya VK, et al. A Network Map of FGF-1/FGFR Signaling System. J Signal Transduct 2014;2014:962962.
25. Geske MJ, Zhang X, Patel KK, et al. Fgf9 signaling regulates small intestinal elongation and mesenchymal development. Development 2008;135:2959-68.
26. Zacchetti G, Duboule D, Zakany J. Hox gene function in vertebrate gut morphogenesis: the case of the caecum. Development 2007;134:3967-73.
27. Heanue TA, Pachnis V. Expression profiling the developing mammalian enteric nervous system identifies marker and candidate Hirschsprung disease genes. Proc Natl Acad Sci U S A 2006;103:6919-24.
28. Vohra BP, Tsuji K, Nagashimada M, et al. Differential gene expression and functional analysis implicate novel mechanisms in enteric nervous system precursor migration and neuritogenesis. Dev Biol 2006;298:259-71.
29. Jackson VA, Mehmood S, Chavent M, et al. Super-complexes of adhesion GPCRs and neural guidance receptors. Nat Commun 2016;7:11184.
30. **Yamagishi S, Hampel F**, Hata K, et al. FLRT2 and FLRT3 act as repulsive guidance cues for Unc5-positive neurons. EMBO J 2011;30:2920-33.
31. Lasrado R, Boesmans W, Kleinjung J, et al. Lineage-dependent spatial and functional organization of the mammalian enteric nervous system. Science 2017;356:722-726.
32. Sanchez MP, Silos-Santiago I, Frisen J, et al. Renal agenesis and the absence of enteric neurons in mice lacking GDNF. Nature 1996;382:70-3.
33. Heuckeroth RO, Enomoto H, Grider JR, et al. Gene targeting reveals a critical role for neurturin in the development and maintenance of enteric, sensory, and parasympathetic neurons. Neuron 1999;22:253-63.
34. Rossi J, Luukko K, Poteryaev D, et al. Retarded growth and deficits in the enteric and parasympathetic nervous system in mice lacking GFR alpha2, a functional neurturin receptor. Neuron 1999;22:243-52.

35. Schuchardt A, D'Agati V, Larsson-Blomberg L, et al. Defects in the kidney and enteric nervous system of mice lacking the tyrosine kinase receptor Ret. Nature 1994;367:380-3.
36. **Cacalano G, Farinas I, Wang LC**, et al. GFRalpha1 is an essential receptor component for GDNF in the developing nervous system and kidney. Neuron 1998;21:53-62.
37. Rossi J, Herzig KH, Voikar V, et al. Alimentary tract innervation deficits and dysfunction in mice lacking GDNF family receptor alpha2. J Clin Invest 2003;112:707-16.
38. Bondurand N, Southard-Smith EM. Mouse models of Hirschsprung disease and other developmental disorders of the enteric nervous system: Old and new players. Dev Biol 2016;417:139-57.
39. Ramalho-Santos M, Melton DA, McMahon AP. Hedgehog signals regulate multiple aspects of gastrointestinal development. Development 2000;127:2763-72.
40. Uesaka T, Nagashimada M, Enomoto H. Neuronal Differentiation in Schwann Cell Lineage Underlies Postnatal Neurogenesis in the Enteric Nervous System. J Neurosci 2015;35:9879-88.
41. Jin S, Martinelli DC, Zheng X, et al. Gas1 is a receptor for sonic hedgehog to repel enteric axons. Proc Natl Acad Sci U S A 2015;112:E73-80.
42. Liu J, Speder P, Brand AH. Control of brain development and homeostasis by local and systemic insulin signalling. Diabetes Obes Metab 2014;16 Suppl 1:16-20.
43. Zhang GQ, Yang S, Li XS, et al. Expression and possible role of IGF-IR in the mouse gastric myenteric plexus and smooth muscles. Acta Histochem 2014;116:788-94.
44. Xu C, Zhu S, Wu M, et al. Functional receptors and intracellular signal pathways of midkine (MK) and pleiotrophin (PTN). Biol Pharm Bull 2014;37:511-20.
45. Vanderwinden JM, Mailleux P, Schiffmann SN, et al. Cellular distribution of the new growth factor pleiotrophin (HB-GAM) mRNA in developing and adult rat tissues. Anat Embryol (Berl) 1992;186:387-406.
46. Fattahi F, Steinbeck JA, Kriks S, et al. Deriving human ENS lineages for cell therapy and drug discovery in Hirschsprung disease. Nature 2016;531:105-9.
47. Newgreen DF, Hartley L. Extracellular matrix and adhesive molecules in the early development of the gut and its innervation in normal and spotting lethal rat embryos. Acta Anat (Basel) 1995;154:243-60.
48. Ratcliffe EM, Fan L, Mohammed TJ, et al. Enteric neurons synthesize netrins and are essential for the development of the vagal sensory innervation of the fetal gut. Dev Neurobiol 2011;71:362-73.
49. **Woo J, Kwon SK**, Choi S, et al. Trans-synaptic adhesion between NGL-3 and LAR regulates the formation of excitatory synapses. Nat Neurosci 2009;12:428-37.
50. Jannot AS, Pelet A, Henrion-Caude A, et al. Chromosome 21 scan in Down syndrome reveals DSCAM as a predisposing locus in Hirschsprung disease. PLoS One 2013;8:e62519.

51. Seaman C, Anderson R, Emery B, et al. Localization of the netrin guidance receptor, DCC, in the developing peripheral and enteric nervous systems. Mech Dev 2001;103:173-5.
52. Chalazonitis A, D'Autreaux F, Pham TD, et al. Bone morphogenetic proteins regulate enteric gliogenesis by modulating ErbB3 signaling. Dev Biol 2011;350:64-79.
53. **Garcia-Barcelo MM, Tang CS**, Ngan ES, et al. Genome-wide association study identifies NRG1 as a susceptibility locus for Hirschsprung's disease. Proc Natl Acad Sci U S A 2009;106:2694-9.
54. Barrenschee M, Lange C, Cossais F, et al. Expression and function of Neuregulin 1 and its signaling system ERBB2/3 in the enteric nervous system. Front Cell Neurosci 2015;9:360.
55. **Tang CS, Cheng G, So MT**, et al. Genome-wide copy number analysis uncovers a new HSCR gene: NRG3. PLoS Genet 2012;8:e1002687.
56. Yang J, Duan S, Zhong R, et al. Exome sequencing identified NRG3 as a novel susceptible gene of Hirschsprung's disease in a Chinese population. Mol Neurobiol 2013;47:957-66.
57. Obermayr F, Stamp LA, Anderson CR, et al. Genetic fate-mapping of tyrosine hydroxylase-expressing cells in the enteric nervous system. Neurogastroenterol Motil 2013;25:e283-91.
58. Fox EA, Murphy MC. Factors regulating vagal sensory development: potential role in obesities of developmental origin. Physiol Behav 2008;94:90-104.
59. Chalazonitis A, Rothman TP, Chen J, et al. Neurotrophin-3 induces neural crest-derived cells from fetal rat gut to develop in vitro as neurons or glia. J Neurosci 1994;14:6571-84.
60. Lucini C, Maruccio L, de Girolamo P, et al. Localisation of neurotrophin - containing cells in higher vertebrate intestine. Anat Embryol (Berl) 2002;205:135-40.
61. **Levanti MB, Esteban I**, Ciriaco E, et al. Enteric glial cells express full-length TrkB and depend on TrkB expression for normal development. Neurosci Lett 2009;454:16-21.
62. Okamura Y, Saga Y. Notch signaling is required for the maintenance of enteric neural crest progenitors. Development 2008;135:3555-65.
63. Ngan ES, Garcia-Barcelo MM, Yip BH, et al. Hedgehog/Notch-induced premature gliogenesis represents a new disease mechanism for Hirschsprung disease in mice and humans. J Clin Invest 2011;121:3467-78.
64. Ngan ES, Lee KY, Sit FY, et al. Prokineticin-1 modulates proliferation and differentiation of enteric neural crest cells. Biochim Biophys Acta 2007;1773:536-45.
65. Sato Y, Heuckeroth RO. Retinoic acid regulates murine enteric nervous system precursor proliferation, enhances neuronal precursor differentiation, and reduces neurite growth in vitro. Dev Biol 2008;320:185-98.
66. Anderson RB, Bergner AJ, Taniguchi M, et al. Effects of different regions of the developing gut on the migration of enteric neural crest-derived cells: a role for Sema3A, but not Sema3F. Dev Biol 2007;305:287-99.

67. Wang LL, Zhang Y, Fan Y, et al. SEMA3A rs7804122 polymorphism is associated with Hirschsprung disease in the Northeastern region of China. Birth Defects Res A Clin Mol Teratol 2012;94:91-5.
68. **Jiang Q, Arnold S, Heanue T**, et al. Functional loss of semaphorin 3C and/or semaphorin 3D and their epistatic interaction with ret are critical to Hirschsprung disease liability. Am J Hum Genet 2015;96:581-96.
69. **Kuzirian MS, Moore AR**, Staudenmaier EK, et al. The class 4 semaphorin Sema4D promotes the rapid assembly of GABAergic synapses in rodent hippocampus. J Neurosci 2013;33:8961-73.
70. Krushel LA, Prieto AL, Cunningham BA, et al. Expression patterns of the cell adhesion molecule Nr-CAM during histogenesis of the chick nervous system. Neuroscience 1993;53:797-812.
71. Goldberg D, Borojevic R, Anderson M, et al. Slit/Robo-mediated chemorepulsion of vagal sensory axons in the fetal gut. Dev Dyn 2013;242:9-15.
72. **Zuhdi N, Ortega B, Giovannone D**, et al. Slit molecules prevent entrance of trunk neural crest cells in developing gut. Int J Dev Neurosci 2015;41:8-16.
73. **Beaubien F, Raja R,** Kennedy TE, et al. Slitrk1 is localized to excitatory synapses and promotes their development. Sci Rep 2016;6:27343.
74. Song M, Giza J, Proenca CC, et al. Slitrk5 Mediates BDNF-Dependent TrkB Receptor Trafficking and Signaling. Dev Cell 2015;33:690-702.
75. Takahashi H, Katayama K, Sohya K, et al. Selective control of inhibitory synapse development by Slitrk3-PTPdelta trans-synaptic interaction. Nat Neurosci 2012;15:389-98, S1-2.
76. Aruga J. Slitrk6 expression profile in the mouse embryo and its relationship to that of Nlrr3. Gene Expr Patterns 2003;3:727-33.
77. Dias JM, Alekseenko Z, Applequist JM, et al. Tgfbeta signaling regulates temporal neurogenesis and potency of neural stem cells in the CNS. Neuron 2014;84:927-39.
78. Sasselli V, Boesmans W, Vanden Berghe P, et al. Planar cell polarity genes control the connectivity of enteric neurons. J Clin Invest 2013;123:1763-72.
79. **Valenta T, Degirmenci B**, Moor AE, et al. Wnt Ligands Secreted by Subepithelial Mesenchymal Cells Are Essential for the Survival of Intestinal Stem Cells and Gut Homeostasis. Cell Rep 2016;15:911-8.
80. Lickert H, Kispert A, Kutsch S, et al. Expression patterns of Wnt genes in mouse gut development. Mech Dev 2001;105:181-4.
81. Qu Y, Huang Y, Feng J, et al. Genetic evidence that Celsr3 and Celsr2, together with Fzd3, regulate forebrain wiring in a Vangl-independent manner. Proc Natl Acad Sci U S A 2014;111:E2996-3004.

Author names in bold designate shared co-first authorship.
