# Supplementary material for: Transcription and Signaling Regulators in Developing Neuronal Subtypes of Mouse and Human Enteric Nervous System
Source: Gastroenterology. 2018 Feb;154(3):624–36. doi: 10.1053/j.gastro.2017.10.005 (PMC6381388; doi:10.1053/j.gastro.2017.10.005)
Supplement: Supplementary Table 8 [file mmc18.pdf]

SUPPLEMENTARY TABLE 8:
ENS enriched genes associated with siganling pathways

Pathway Name	FGF	TGFb	Wnt	Cxcr4	Insulin/IGF	Ephrin
**Comparison, Fold Enrichment**	W15vsC15; 1.25	W15vsC15; 1,25	W11vsC11; 1,25	W15vsC15; 1,25	W15vsC15; 1,25	W15vsC15; 1,25
**Pathway ID**	Panther: 00021	Panther: 00052	Panther: P00057	Biocarta: Cxcr4 pathway	Panther: 00033	Reactome: 2132295
	Hras1<br>*Rasa2*<br>*Prkce*<br>*Fgf13*<br>*Fgf14*<br>*Mapk10*<br>*Mapk11*<br>*Mapk8*<br>*Map2k1*<br>*Map3k1*<br>*Pebp1*<br>*Pik3cb*<br>*Pik3c3*<br>*Prkca*<br>*Prkcb*<br>*Prkcz*<br>*Ppp2ca*<br>*Ppp2r2a*<br>*Ppp2r2b*<br>*Ppp2r2c*<br>*Ppp2r5b*<br>*Ppp2r5c*<br>*Nras*<br>*Sos1*<br>*Sos2*<br>*Spry3*<br>*Shc3*<br>*Syngap1*<br>*Akt3*<br>*Ywhag*<br>*Ywhaz*	*Fkbp1b*<br>*Skil*<br>*Atf2*<br>*Mapk10*<br>*Smad2*<br>*Dcp1b*<br>*Cited2*<br>*Bmp7*<br>*Hras1*<br>*Acvr1b*<br>*Acvr2a*<br>*Nras*<br>*Mapk8*<br>*Mapk11*<br>*Bmpr1b*<br>*Tgfb2*	*Gsk3b*<br>*Smarcd1*<br>*Smarcb1*<br>*Hdac3*<br>*Ctbp1*<br>*Lef1*<br>*Lrp5*<br>*Nlk*<br>*Prkcz*<br>*Celsr3*<br>*Nkd2*<br>*Cdh6*<br>*Gng3*<br>*Gng2*<br>*CCND1*<br>*Cdh2*<br>*Myc*<br>*Gna11*<br>*Dvl3*<br>*Ctnnal1*<br>*Smad1*<br>*Fzd3*<br>*Kremen1*<br>*Ppp2r5b*<br>*Prkca*<br>*Smarca4*<br>*Plcb4*<br>*Mycn*<br>*Myh3*<br>*Cdh10*<br>*Gnaq*<br>*Csnk1g2*<br>*Cdh19*	*Hras*<br>*Ptk2*<br>*Cxcr4*<br>*Gnai1*<br>*Gnb1*<br>*Gnaq*<br>*Map2k1*<br>*Pik3r1*<br>*Prkcb*	*Foxo1*<br>*Gsk3b*<br>*Insr*<br>*Igf1r*<br>*Pik3r3*<br>*Pik3cb*<br>*Pik3r1*<br>*Pik3c3*<br>*Akt3*	*Actr1a*<br>*Sh3gl2*<br>*Ap2b1*<br>*Dctn3*<br>*Dnm3*<br>*Dync1h1*<br>*Dync1i2*<br>*Dynll2*<br>*Kif2a*<br>*Kif3a*<br>*Kif3b*<br>*Kif3c*<br>*Kif5a*<br>*Klc1*<br>*Kifap3*
